# Supplementary material for: Divergent regioselective Heck-type reaction of unactivated alkenes and N-fluoro-sulfonamides
Source: Nat Commun. 2022 Oct 22;13:6297. doi: 10.1038/s41467-022-33996-1 (PMC9588056; doi:10.1038/s41467-022-33996-1)
Supplement: Supplementary file 1 — Supplementary Information [file 41467_2022_33996_MOESM1_ESM.pdf]

Supplementary Information

Divergent Regioselective Heck-type Reaction of Unactivated Alkenes and *N*-Fluoro-sulfonamides

Chunyang Zhao,[1,#] Yang Li,[1,#] Yujiao Dong,[2,#] Miao Li,[1] Dan Xia,[1] Shuangqiu Gao,[1] Qian Zhang,[1] Qun Liu,[1] Wei Guan,*[,2] and Junkai Fu*[,1,3]

[1] Jilin Province Key Laboratory of Organic Functional Molecular Design & Synthesis, Department of Chemistry, Northeast Normal University, Changchun 130024, China

[2] Institute of Functional Material Chemistry, Department of Chemistry, Northeast Normal University, Changchun 130024, China

[3] State Key Laboratory of Chemical Oncogenomics and Key Laboratory of Chemical Genomics, Peking University Shenzhen Graduate School, Shenzhen 518055, China

[#] These authors contributed equally to this work

* Correspondence: fujk109@nenu.edu.cn (J. F.); guanw580@nenu.edu.cn (W. G.)

Table of Contents

Part 1: Supplementary methods --S2

Part 2: Supplementary discussion --S3

Part 2.1: Supplementary data and crystallography data ---------------------------S3

Part 2.2: DFT calculations --S6

Part 3: Supplementary Notes ---S10

Part 3.1: Procedure and characteristic data for substrates **1w, 1ab, 2b-13** ------S10

Part 3.2: Procedure and characteristic data for products **3a-3az, 4a-4az, 5a-5k, 6a-6aa, 7a, 7b, 8** --S35

Part 3.3: Procedure and characteristic data for radical clock experiments ------S112

Part 3.4: Procedure and characteristic data for compound **19** and **20** ------------S115

Part 3.5: NMR spectra --S117

Part 4: Supplementary references ---S330

Part 1: Supplementary methods

Unless otherwise noted, all reactions were carried out under an argon atmosphere as well as anhydrous conditions. Commercial reagents were purchased from Adamas, Aldrich, TCI, Energy Chemical, Bide, Leyan and J&K chemical, and were used as received. Anhydrous 1, 4-dioxane, 1, 2-dichloroethane (DCE), benzene and 1-methyl-2-pyrrolidinone (NMP) were purchased from Energy Chemical. The isopropanol (^{i}PrOH), isobutanol (^{i}BuOH) and tert-Butanol (^{s}BuOH) were distilled from sodium. Anhydrous acetonitrile (CH_3CN) was distilled from calcium hydride. Anhydrous toluene (Tol.) was distilled from sodium.

Reactions were monitored by Thin Layer Chromatography (TLC) on plates (GF254) supplied by Yantai Chemicals (China) visualized by UV or stained with ethanolic solution of phosphomolybdic acid and basic solution of $KMnO_4$. The products were purified by column chromatography over silica gel (300 - 400 size).

NMR spectra were recorded on a Brüker Advance 600 (^{1}H: 600 MHz, ^{13}C:150 MHz, ^{19}F: 565 MHz), Brüker Advance 500 (^{1}H: 500 MHz, ^{13}C: 125 MHz, ^{19}F: 470 MHz), and TMS was used as internal standard. The following abbreviations were used to explain the multiplicities: s = singlet, d = doublet, t = triplet, q = quartet, dd = doublet of doublets, m = multiplet, br = broad.

IR spectra were recorded on an IRPrestige-21 FTIR spectrometer. High resolution mass spectrometric (HRMS) data was recorded on Brüker Apex IV RTMS by using ESI method.

Part 2. Supplementary discussion

Part 2.1: Supplementary data and crystallography data

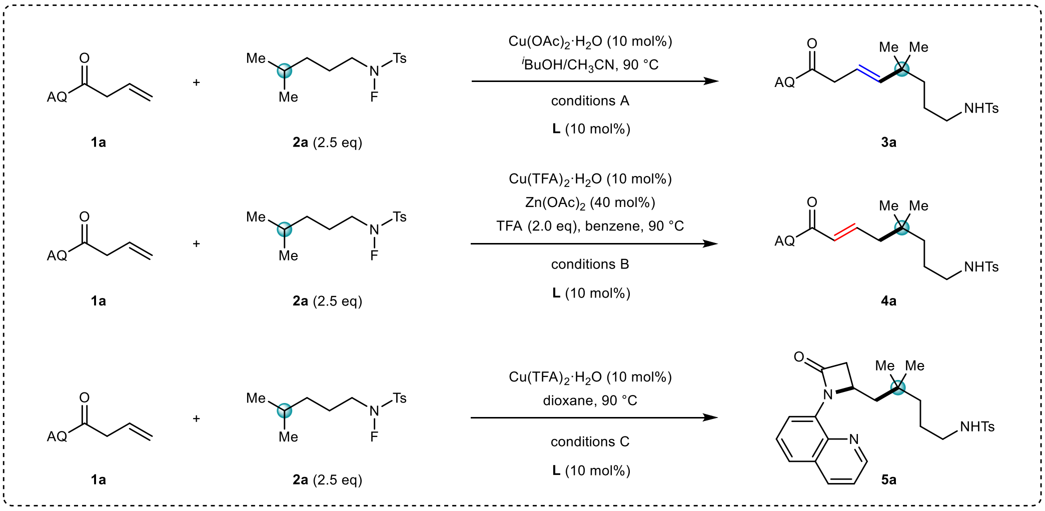

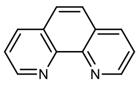

L1

conditions A: **3a** (70%, *r.r.* > 20:1, *E/Z* > 20:1)

conditions B: **4a** (50%, *r.r.* > 20:1, *E/Z* > 20:1)

conditions C: **5a** (66%)

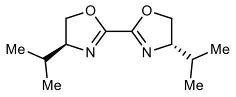

L2

conditions A : **3a** (62%, *r.r.* > 20:1, *E/Z* > 20:1)

conditions B : **4a** (48%, *r.r.* > 20:1, *E/Z* > 20:1)

conditions C: **5a** (62%, 0% *ee*)

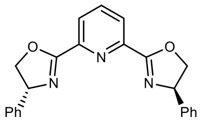

L3

conditions A : **3a** (73%, *r.r.* > 20:1, *E/Z* > 20:1)

conditions B : **4a** (44%, *r.r.* > 20:1, *E/Z* > 20:1)

conditions C: **5a** (45%, 10% *ee*)

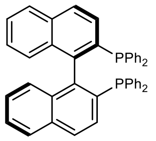

L4

conditions A : **3a** (60%, *r.r.* > 20:1, *E/Z* > 20:1)

conditions B : **4a** (37%, *r.r.* > 20:1, *E/Z* > 20:1)

conditions C: **5a** (52%, 0% *ee*)

Supplementary Fig. 1. Investigation of the formation of 3a, 4a, and 5a with external ligands. Conditions A: **1a** (0.20 mmol), **2a** (0.50 mmol), $Cu(OAc)_2{\cdot}H_2O$ (0.02 mmol), and **L** (0.02 mmol) in a mixed ^{i}BuOH/CH_3CN (2.5/0.5 mL) at 90 °C for 3 h. Conditions B: **1a** (0.20 mmol), **2a** (0.50 mmol), $Cu(TFA)_2{\cdot}H_2O$ (0.02 mmol), $Zn(OAc)_2$ (0.08 mmol), TFA (0.40 mmol), and **L** (0.02 mmol) in benzene (2.0 mL) at 90 °C for 4 h. Conditions C: **1a** (0.20 mmol), **2a** (0.50 mmol), $Cu(TFA)_2{\cdot}H_2O$ (0.02 mmol), and **L** (0.02 mmol) in dioxane (3.0 mL) at 90 °C for 3 h. TFA, trifluoroacetic acid; *ee*, enantioselectivity.

HPLC for compound *rac*-**5a** (OJ-H, *n*-hexane/*i*-PrOH = 85/15, flow rate = 0.8 mL/min, I = 254 nm, T = 40 ℃) t_R = 47.9 min, 70.3 min.

〈色谱图〉

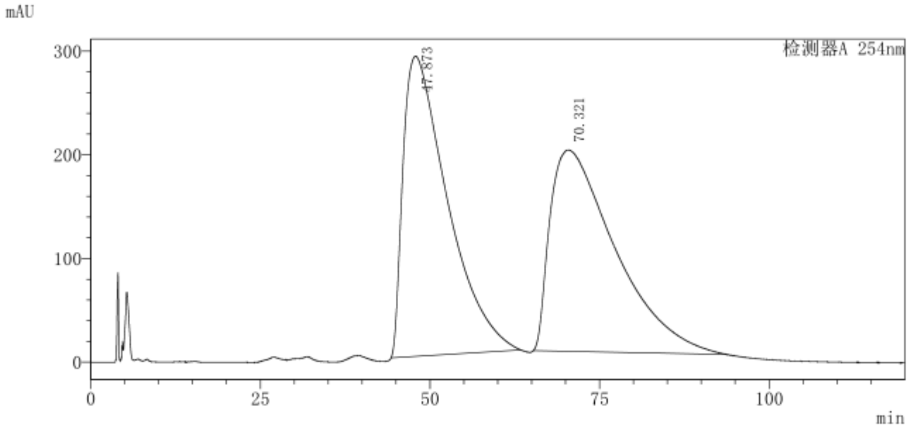

〈峰表〉
检测器A 254nm

峰号	保留时间	面积	高度	浓度	浓度单位	标记	化合物名
1	47.873	131770354	289510	50.296		M	
2	70.321	130217734	194150	49.704		M	
总计		261988088	483660				

HPLC for compound **5a** (10% *ee*) obtained with chair ligand **L3** (OJ-H, *n*-hexane/*i*-PrOH = 85/15, flow rate = 0.8 mL/min, I = 254 nm, T = 40 ℃) t_R = 51.3 min, 74.7 min.

〈色谱图〉

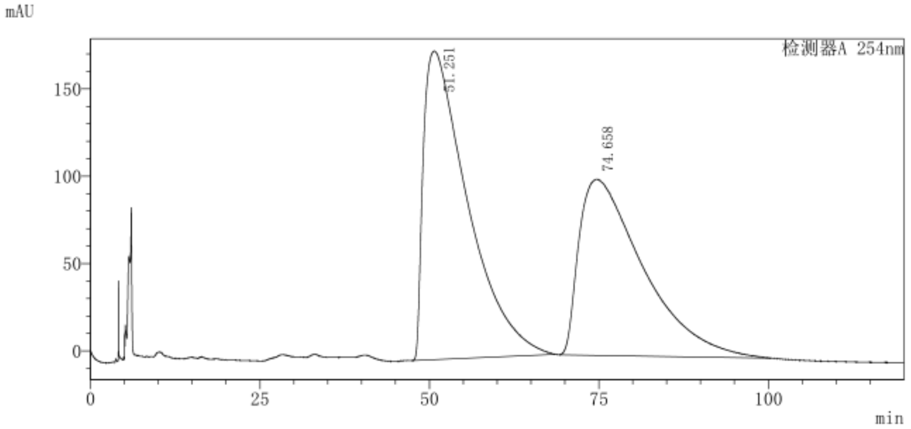

〈峰表〉
检测器A 254nm

峰号	保留时间	面积	高度	浓度	浓度单位	标记	化合物名
1	51.251	80215841	173822	54.758		M	
2	74.658	66276658	100750	45.242		M	
总计		146492499	274572				

crystallography of compound 6d (CCDC 2163753)

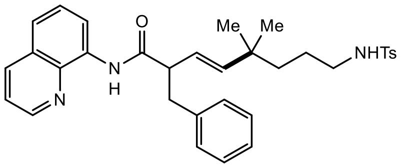

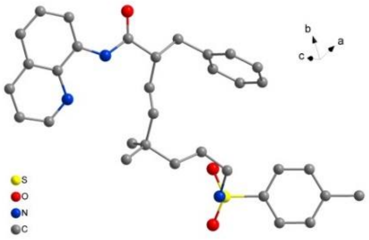

Crystal data

Empirical formula	$C_{33}H_{37}N_3O_3S$
Formula weight	552.71
Temperature	274 K
Cu *Kα* radiation(*λ*)	1.54178 Å
Unit cell dimensions	*a* = 8.9489 (3) Å, *α* = 72.897 (2)°<br>*b* = 10.9618 (4) Å, *β* = 75.797 (2)°<br>*c* = 17.5510 (6) Å, *γ* = 66.275 (2)°
Volume	1490.52 (9) Å^3
Z	2
Calculated desity	1.232 Mg m^{-3}
Crystal System	Triclinic, $P\bar{1}$
Absorption coefficient	1.26 mm^{-1}
F(000)	589
Crystal size	0.05 × 0.03 × 0.02 mm
Theta range for data collection	2.7-63.6°
Data / restraints / paramaters	4822/0/364
Goodness-of-fit on F^2	1.03
R_1^a(I>2σ(I))	0.050
wR_2^b (all data)	0.250

$^aR_1=\Sigma||F_o|-|F_c||/\Sigma|F_o|$, $^bwR_2=[\Sigma w(F_o^2-F_c^2)^2/\Sigma w(F_o^2)^2]^{1/2}$.

Part 2.2. DFT calculations

All DFT calculations were performed with the Gaussian 16 program.[S1] Geometry optimizations were carried out using (U)M06 hybrid functional with the SMD solvation model. The LanL2DZ basis set was applied for Cu atom. The standard 6-31G(d) basis set was used for the other main-group elements. The vibrational frequency analysis was calculated at the same level as the geometry optimizations to guarantee the minimum without imaginary frequency but only one imaginary frequency for the saddle point. The intrinsic reaction coordinate (IRC) [S2] was conducted to ensure the transition states actually connect with the correct reactants and products. In addition, the single-point energies of all studied systems were performed at the SMD(^{i}BuOH/benzene/dioxane)/(U)M06[6-311++G(d,p)/SDD(Cu)] level. The translational entropy was corrected with the method developed by Whitesides et.al.[S3]

Correction of translational entropy in solution

We evaluated the electronic energy (E_{sol}) with zero-point energy correction in solution. For each species, the E_{sol} is defined through equation (S1):

$$E_{sol} = E_{sol}^{pot} + E_{gas}^{v_0} \qquad \text{(S1)}$$

the E_{sol}^{pot} is the potential energy including non-electrostatic energy in solution and $E_{gas}^{v_0}$ denotes the zero-point vibrational energy in the gas phase. In a bimolecular process, such as the coordination of ^{i}BuOH to the copper, the entropy change which can decreases considerably must be taken into consideration. In such case, Gibbs energy (G_{sol}^{o}) need be computed as follows:

$$\begin{aligned} G_{sol}^{o} &= H_0 - T(S_r^o + S_v^o + S_t^o) \\ &= E^T + P\Delta V - T(S_r^o + S_v^o + S_t^o) \\ &= E_{sol} + E_{therm} - T(S_r^o + S_v^o + S_t^o) \qquad \text{(S2)} \end{aligned}$$

where ΔV is 0 in solution, E_{therm} is the thermal correction by translational, vibrational, and rotational movements, and S_r^o, S_v^o, and S_t^o are rotational, vibrational, and translational entropies, respectively. In general, the Sackur-Tetrode equation is used to

evaluate translational entropy S_t^o. In solution, however, the usual Sackur-Tetrode equation cannot be directly applied to the evaluation of S_t^o, because the translation movement is suppressed very much in solution.[S4] In this context, the translational entropy was corrected with the method developed by Whitesides et al., where the rotational entropy was evaluated in a normal manner. Thermal correction and entropy contributions of vibration movements to the Gibbs energy were evaluated with the frequencies calculated at 298.15 K and 1 atm.

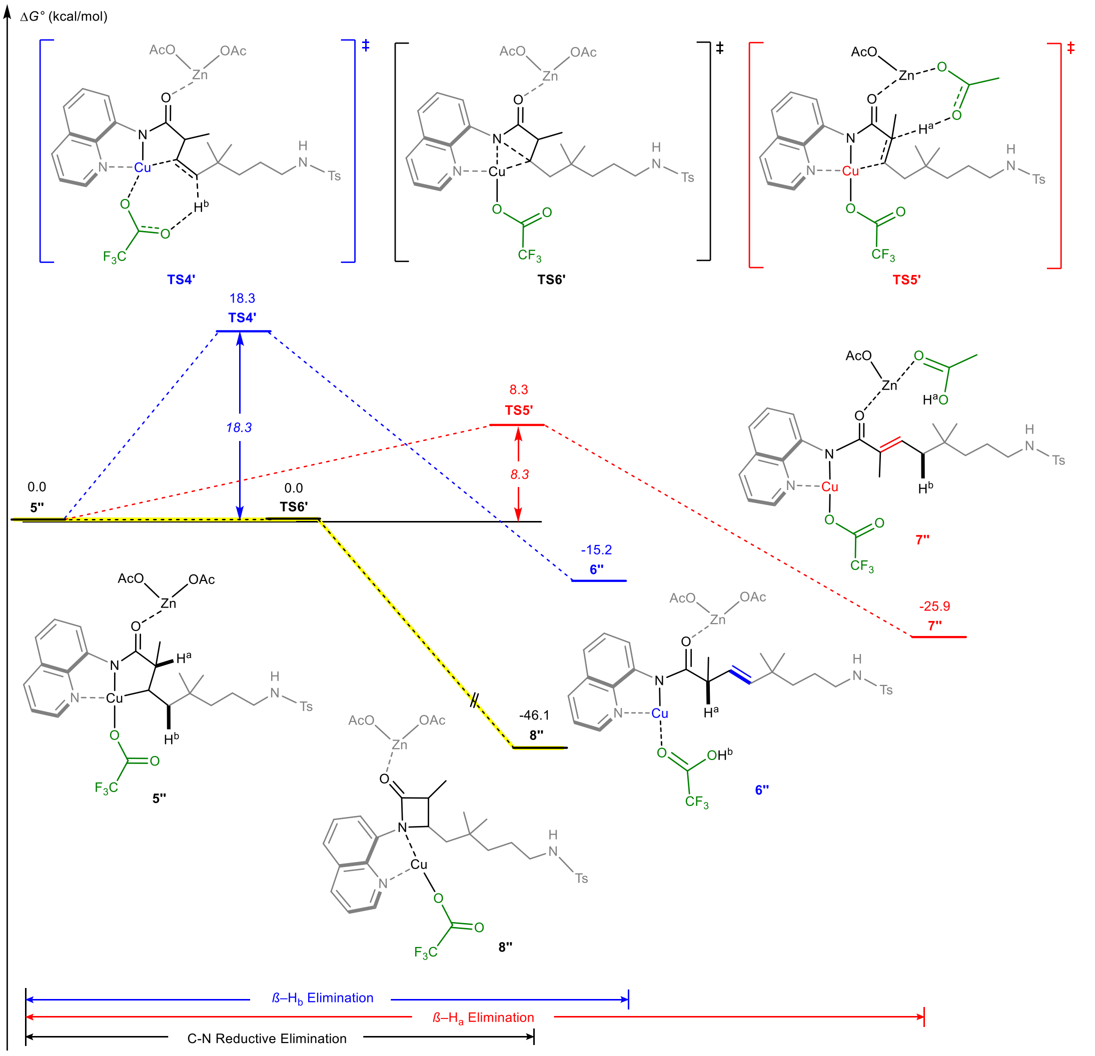

Supplementary Fig. 2. Gibbs energy profiles (Δ*G*°) of three possible reaction mechanisms using the *α*-methyl alkenyl amide to this reaction in benzene solvent with the presence of Zn(OAc)$_2$ and TFA. TFA = trifluoroacetic acid.

When one H_a is replaced by a methyl, the regioselectivity switches to the C–N reductive elimination. As shown in Supplementary Fig. 2, the $\Delta G^{\circ\ddagger}$ and ΔG° values of C–N reductive elimination are 0.0 and −46.1 kcal/mol, respectively. In contrast, the β–H_a and β–H_b eliminations require larger energy barriers of 8.3 and 18.3 kcal/mol, respectively. The regioselectivity switch can be understood from two aspects: one is that the electron-donating α-methyl substituent can promote the C–N reductive elimination; another is that the steric hindrance caused by α-methyl substitution is disadvantageous to a coplanar concerted β–H_a elimination process.

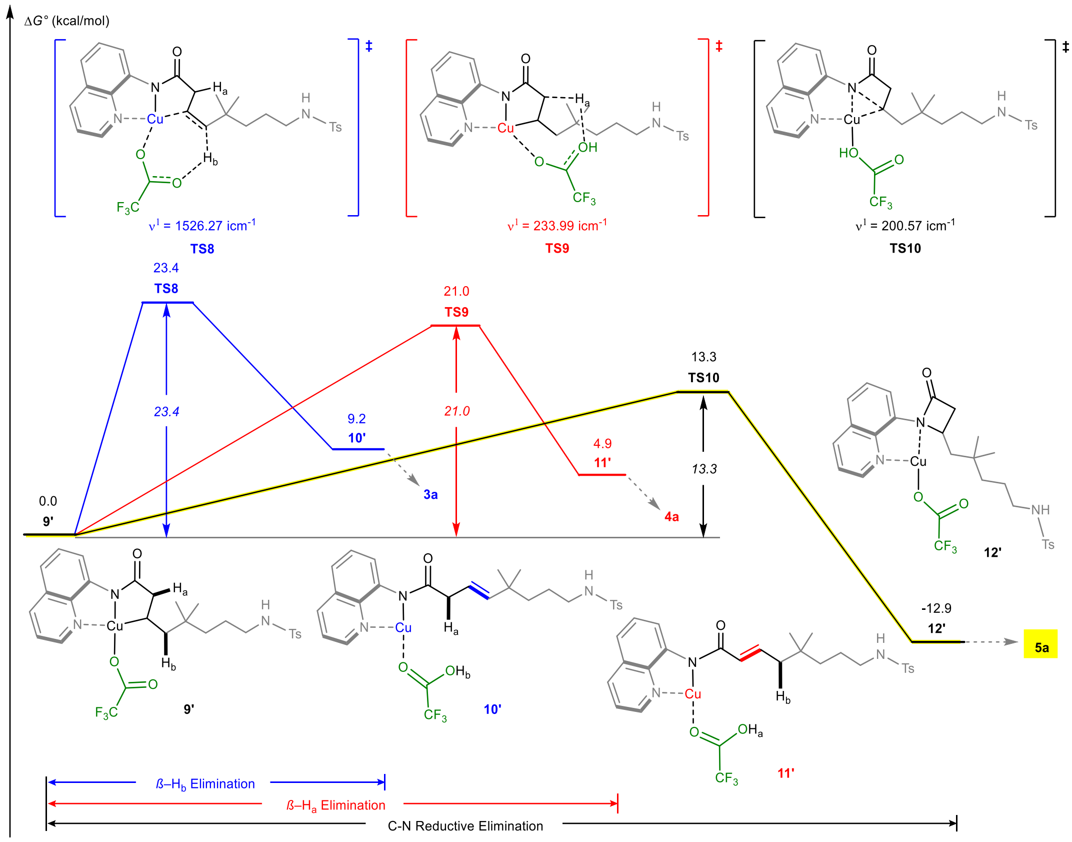

Supplementary Fig. 3. Gibbs energy profiles (Δ*G*°) for RE product 5a under conditions C without any promoting factors calculated at the SMD(dioxane)/(U)M06/[6-311++G(d,p) /SDD(Cu)]//SMD(dioxane)/(U)M06/[6-31G(d)/LanL2DZ(Cu)] level.

References

S1. M. J. Frisch, G. W. Trucks, H. B. Schlegel, G. E. Scuseria, M. A. Robb, J. R. Cheeseman, G.

Scalmani, V. Barone, G. A. Petersson, H. Nakatsuji, X. Li, M. Caricato, A. V. Marenich, J. Bloino, B. G. Janesko, R. Gomperts, B. Mennucci, H. P. Hratchian, J. V. Ortiz, A. F. Izmaylov, J. L. Sonnenberg, D. Williams-Young, F. Ding, F. Lipparini, F. Egidi, J. Goings, B. Peng, A. Petrone, T. Henderson, D. Ranasinghe, V. G. Zakrzewski, J. Gao, N. Rega, G. Zheng, W. Liang, M. Hada, M. Ehara, K. Toyota, R. Fukuda, J. Hasegawa, M. Ishida, T. Nakajima, Y. Honda, O. Kitao, H. Nakai, T. Vreven, K. Throssell, J. A. Montgomery, Jr., J. E. Peralta, F. Ogliaro, M. J. Bearpark, J. J. Heyd, E. N. Brothers, K. N. Kudin, V. N. Staroverov, T. A. Keith, R. Kobayashi, J. Normand, K. Raghavachari, A. P. Rendell, J. C. Burant, S. S. Iyengar, J. Tomasi, M. Cossi, J. M. Millam, M. Klene, C. Adamo, R. Cammi, J. W. Ochterski, R. L. Martin, K. Morokuma, O. Farkas, J. B. Foresman, and D. J. Fox, Gaussian 16, Revision C.01, Gaussian, Inc., Wallingford CT, 2019.

S2. K. Fukui, *J. Phys. Chem.* **1970,** *74*, 4161.

S3. M. Mammen, E. I. Shakhnovich, J. M. Deutch, G. M. Whitesides, *J. Org. Chem.* **1998,** *63*, 3821.

S4. (a) S. Sakaki, Y. Y. Ohnishi, H. Sato, *Chem. Rec.* **2010,** *10*, 29; (b) A. Ishikawa, Y. Nakao, H. Sato, S. Sakaki, *lnorg. Chem.* **2009,** *48*, 8154; (c) A. Ishikawa, Y. Nakao, H. Sato, S. Sakaki, *Dalton Trans.* **2010,** *39*, 3279.

Cartesian Coordinates of Optimized Structures

The Cartesian coordinates have been uploaded as a separate Supplementary Data file.

Part 3: Supplementary Notes

Part 3.1: Procedure and characteristic data for products **1w**, **1ab**, **2b-13**

The alkenes used in this paper:

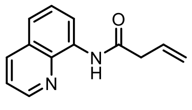
1a
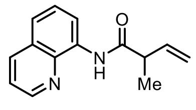
1b
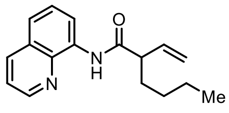
1c
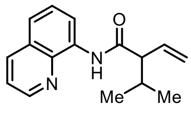
1d
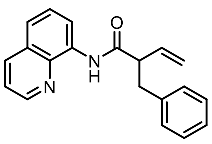
1e
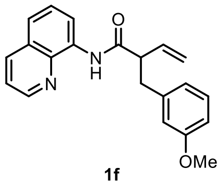
1f
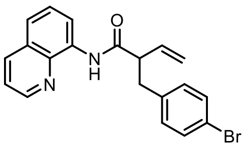
1g
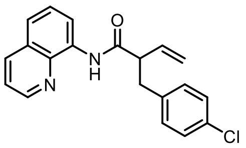
1h
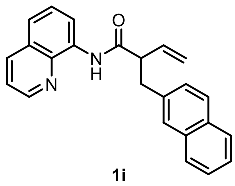
1i
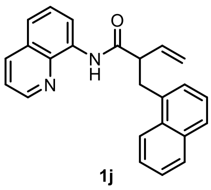
1j
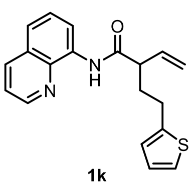
1k
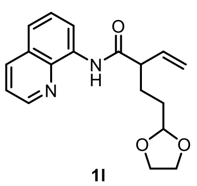
1l
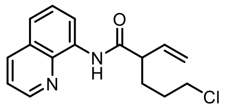
1m
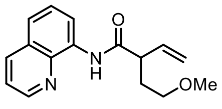
1n
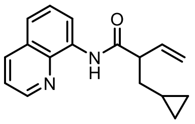
1o
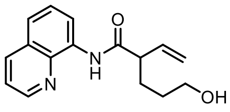
1p
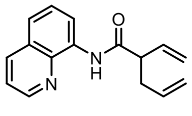
1q
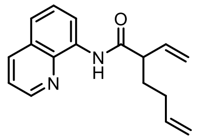
1r
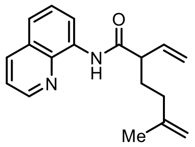
1s
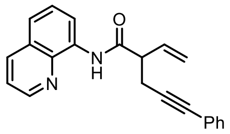
1t
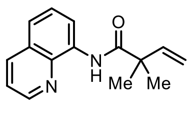
1u
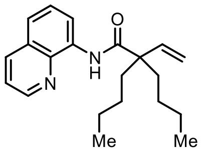
1v
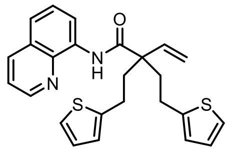
1w
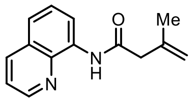
1x
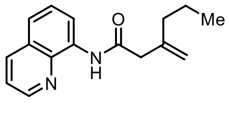
1y
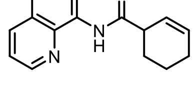
1z
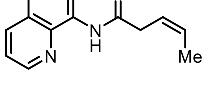
1aa
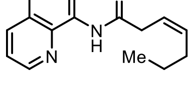
1ab
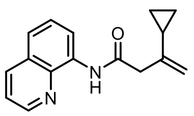
15

1a, 1c-1r, 1t, 1u, 1x-1z, 15 are known compounds according to ref. 1; **1b** is a known compound according to ref. 2; **1s** is a known compound according to ref. 3; **1v** is a known compound according to ref. 4; **1aa** is a known compound according to ref. 5.

The procedure for the synthesis of substrate **1w**:

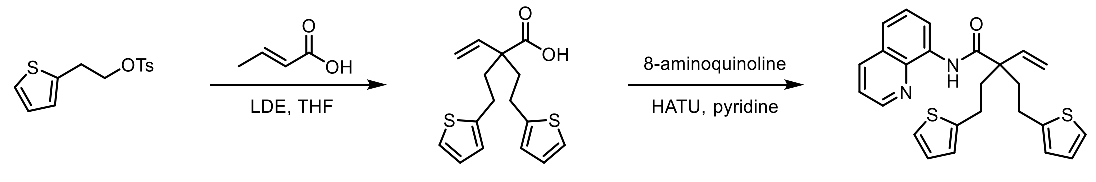

Step 1: To a 100 mL schlenk flask equipped with a magnetic stir bar was added *n*-BuLi (6.40 mL, 16.0 mmol, 2.5 M in hexanes, 3.2 equiv) under a positive pressure of N_2. The flask was evacuated, and hexanes was removed under gentle stirring to leave an opaque paste. The flask was filled with N_2 and charged with anhydrous THF (10 mL), and the resulting light yellow solution was stirred at -78 °C. Diethylamine (1.66 mL, 16.0 mmol, 3.2 equiv) was added dropwise over 5 min. The resulting solution was warmed to 0 °C for 10 min and cooled again to -78 °C. A solution of crotonic acid (430 mg, 5.0 mmol, 1.0 equiv) in THF (5.0 mL) was added slowly over 5 min, and the mixture was warmed up to 0 °C. After 45 min, the reaction was recooled to -78 °C, and a solution of 2-(thiophen-2-yl)ethyl 4-methylbenzenesulfonate (4.23 g, 15.0 mmol, 3.0 equiv) in THF (10 mL) was added dropwise over 5 min. After 30 min, the solution was warmed up to 0 °C. After being stirred for 4 h at 0 °C, the reaction was quenched by slow addition of water (20 mL), and the mixture was acidified to pH = 2-3 with hydrochloric acid (1.0 M). The milky solution was extracted with EtOAc (20 mL × 3). The combined organic extracts were washed with brine, dried over Na_2SO_4, and concentrated *in vacuo*. The crude product (about 4.0 mmol, determinate by ^1H NMR) was carried forward to the next step without purification.

Step 2: The crude acid (about 4.0 mmol, 1.1 equiv) was charged into a 100 mL RB flask containing DCM (10 mL). 8-Aminoquinoline (519 mg, 3.60 mmol, 1.0 equiv), pyridine (0.58 mL, 7.20 mmol, 2.0 equiv), and 2-(7-Azabenzotriazol-1-yl) *N,N,N',N'*-tetramethyluronium hexafluorophosphate (HATU, 1.52 g, 4.0 mmol, 1.1 equiv) were added sequentially, and the reaction was stirred at ambient temperature for 16 h. The deep brown solution was quenched by saturated $NaHCO_3$ solution (20 mL), and then the mixture was extracted with DCM (20 mL × 3). The combined organic layers were washed with brine, dried over Na_2SO_4, and filtered. The solvent was removed under reduced pressure and the residue was purified by flash chromatography using eluents (PE/EA = 15:1) to provide the desired substrate **1w** as yellow oil (933 mg, 2.16 mmol, 43%

over two steps). **^{1}H NMR** (600 MHz, $CDCl_3$) δ 10.36 (s, 1H), 8.81 - 8.79 (m, 2H), 8.15 (dd, J = 8.4, 1.8 Hz, 1H), 7.56 - 7.50 (m, 2H), 7.45 (dd, J = 7.8, 4.2 Hz, 1H), 7.10 (d, J = 4.8 Hz, 2H), 6.90 (dd, J = 4.8, 3.6 Hz, 2H), 6.82 (d, J = 3.0 Hz, 2H), 6.31 (dd, J = 18.0, 10.8 Hz, 1H), 5.63 (d, J = 10.8 Hz, 1H), 5.57 (d, J = 18.0 Hz, 1H), 2.94 - 2.91 (m, 4H), 2.34 - 2.31 (m, 4H). **^{13}C NMR** (150 MHz, $CDCl_3$) δ 172.5, 148.6, 145.0, 139.5, 139.1, 136.4, 134.5, 128.1, 127.5, 126.9, 124.3, 123.2, 121.8, 121.7, 117.9, 116.6, 53.8, 38.0, 25.1. **IR** ν_{max} (film): 3335, 3066, 2930, 1771, 1716, 1681, 1634, 1595, 1576, 1485, 1457, 1439, 1423, 1384, 1324, 1239, 1189, 1169, 1131, 1075, 1036, 1003, 929, 850, 825, 805, 791, 756, 693, 611 cm^{-1}. **HRMS** (ESI) m/z calcd for $C_{25}H_{24}N_2NaOS_2$ $[M+Na]^+$: 455.1222.; found: 455.1231.

The procedure for the synthesis of substrate **1ab**:

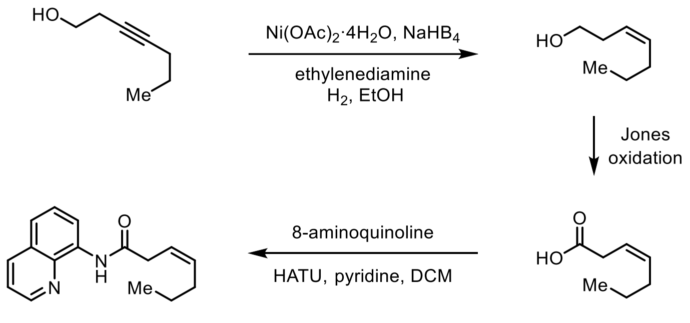

1ab

Step 1: To a stirred solution of $Ni(OAc)_2{\cdot}4H_2O$ (6.22 g, 25.0 mmol, 1.0 equiv) in ethanol (25 mL) under H_2 atmosphere, $NaBH_4$ (946 mg, 25.0 mmol, 1.0 equiv) in ethanol (25 mL) was added at room temperature. After being stirred for 0.5 h, a solution of ethylenediamine (6.65 mL, 100 mmol, 4.0 equiv) and hept-3-yn-1-ol (3.05 mL, 25.0 mmol, 1.0 equiv) in ethanol (25 mL) was added. The reaction mixture was allowed to stir for 4 h. After that, ethanol was removed under reduced pressure, and reaction mixture was diluted with 25 mL 10% ethyl acetate in *n*-hexane and filtered through a pad of celite. The filtrate was concentrated *in vacuo* and the resulting crude product (about 20.0 mmol, determinate by ^{1}H NMR) was used directly for the next step.

Step 2: The crude product (about 20.0 mmol, 1.0 equiv) was dissolved in acetone (40 mL) and the reaction mixture was cooled to 20 °C in a water bath. Jones reagent (2.78 mL, 10.0 mmol, 0.50 equiv, 3.6 M, prepared from 3.80 g CrO_3, 7.20 mL H_2O, and 3.30 mL H_2SO_4) was added dropwise. After being stirred for 2 h, the reaction mixture was diluted in Et_2O (150 mL) and extracted with saturated $NaHCO_3$ solution (75 mL × 2). The aqueous extracts were combined in a 250 mL flask and acidified with conc. H_2SO_4 to pH = 2 under ice cooling. The cloudy aqueous layer was extracted with Et_2O (75 mL × 3). The combined organic extracts were dried over $MgSO_4$ and concentrated *in*

vacuo to afford the crude product (about 11.0 mmol, determinate by ^{1}H NMR), which can be used in the next step without further purification.

Step 3: The crude acid (about 11.0 mmol, 1.1 equiv) was charged into a 100 mL flask containing DCM (20 mL). 8-Aminoquinoline (1.44 g, 10.0 mmol, 1.0 equiv), pyridine (1.62 mL, 20.0 mmol, 2.0 equiv), and HATU (4.18 g, 11.0 mmol, 1.1 equiv) were added sequentially, and the reaction was stirred at ambient temperature for 16 h. The reaction was quenched by saturated $NaHCO_3$ solution (40 mL), and then the mixture was extracted with DCM (40 mL × 3). The combined organic layers were washed with brine, dried over anhydrous Na_2SO_4, filtered, and concentrated *in vacuo*. The residue was purified by a flash column chromatography (PE/EA = 15:1) to afford the desired product **1ab** as yellow oil (1.78 g, 7.0 mmol, 28% over three steps). **^{1}H NMR** (600 MHz, $CDCl_3$) δ 10.04 (s, 1H), 8.79 - 8.75 (m, 2H), 8.09 - 8.07 (m, 1H), 7.50 - 7.43 (m, 2H), 7.38 (dd, J = 7.8, 4.2 Hz, 1H), 5.86 - 5.76 (m, 2H), 3.34 (d, J = 7.2 Hz, 2H), 2.16 (dt, J = 7.2, 7.2 Hz, 2H), 1.51 - 1.45 (m, 2H), 0.94 (t, J = 7.2 Hz, 3H). **^{13}C NMR** (150 MHz, $CDCl_3$) δ 169.6, 148.1, 138.5, 136.2, 135.3, 134.5, 127.9, 127.3, 121.50, 121.45, 121.4, 116.3, 36.9, 29.5, 22.5, 13.8. **IR** ν_{max} (film): 3326, 2959, 2913, 2846, 1683, 1599, 1576, 1521, 1485, 1472, 1461, 1437, 1424, 1383, 1325, 1259, 1161, 1091, 1059, 950, 825, 790, 756, 728, 717, 684, 501 cm^{-1}. **HRMS** (ESI) m/z calcd for $C_{16}H_{18}N_2NaO$ $[M+Na]^+$: 277.1311; found: 277.1311.

The *N*-fluoroamides used in this paper:

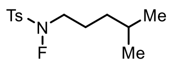

2a

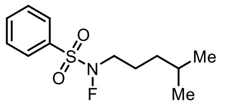

2b

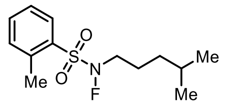

2c

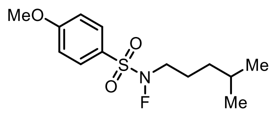

2d

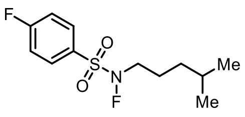

2e

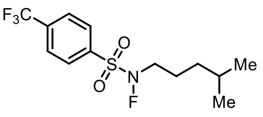

2f

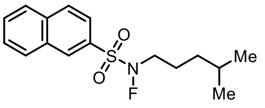

2g

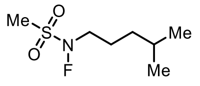

2h

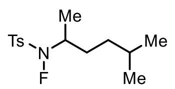

2i

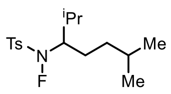

2j

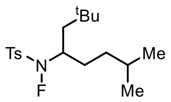

2k

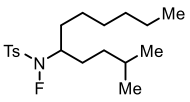

2l

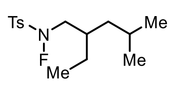

2m

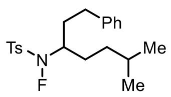

2n

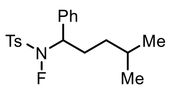

2o

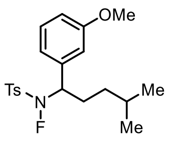

2p

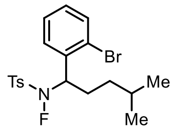
2q

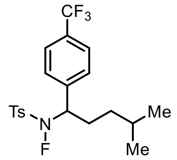
2r

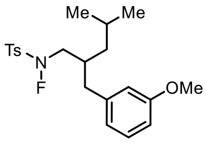
2s

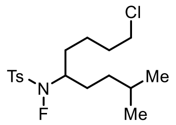
2t

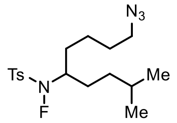
2u

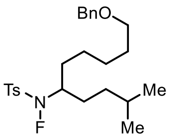
2v

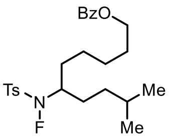
2w

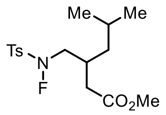
2x

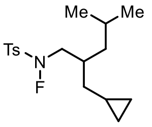
2y

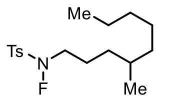
2z

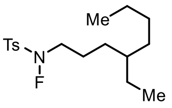
2aa

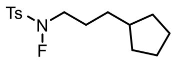
2ab

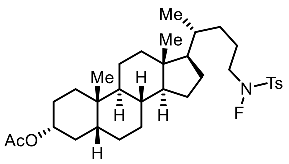
2ac

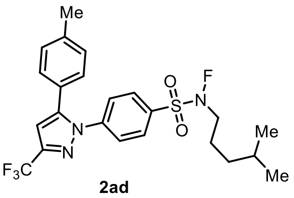
2ad

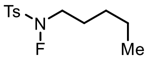
2ae

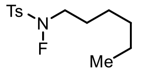
2af

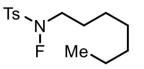
2ag

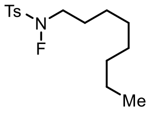
2ah

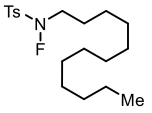
2ai

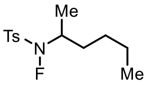
2aj

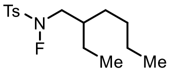
2ak

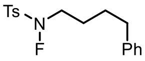
2al

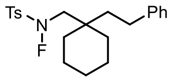
2am

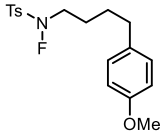
2an

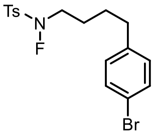
2ao

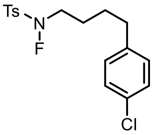
2ap

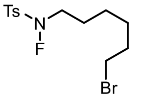
2aq

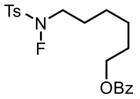
2ar

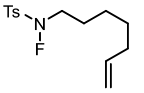
2as

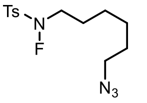
2at

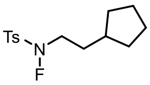
2au

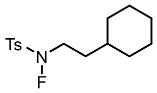
2av

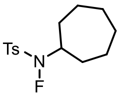
2aw

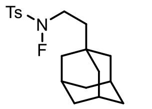
2ax

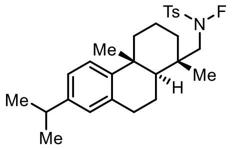
2ay

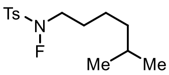
2az

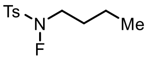
2ba

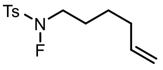
11

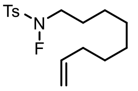
13

2ab, 2ag, 2ak, 2at, 2au are known compounds according to ref. 6; **2d** is a known compound according to ref. 7; **2x, 2ae, 2ah, 2ai, 2ar, 2ax, 2ay, 2az, 2ba, 11** are known compounds according to ref. 8; **2af, 2aj, 2al, 2av** are known compounds according to ref. 9; **2am** is a known compound according to ref. 10; **2aq** is a known compound according to ref. 11; **2aw** is a known compound according to ref. 12.

Procedure A

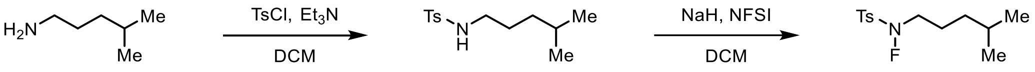

S1 **2a**

Synthesis of compound **S1**

The known 4-methylpentan-1-amine[13] (1.01 g, 10.0 mmol, 1.0 equiv) was dissolved in anhydrous DCM (20 mL). Triethylamine (2.08 mL, 15.0 mmol, 1.5 equiv) and 4-toluolsulfonyl chloride (2.10 g, 11.0 mmol, 1.1 equiv) were added at 0 °C. The solution was stirred overnight at 25 °C. Water (20 mL) was added and the aqueous layer was extracted with DCM (20 mL × 3). The combined organic layers were dried over anhydrous Na_2SO_4, filtered, and the solvent was evaporated under reduced pressure. The residue was purified by a flash column chromatography (PE/EA = 10:1) to afford the desired known product **S1**[14] as yellow solid (2.29 g, 9.0 mmol, 90%).

Synthesis of compound **2a**

A flame dried Schlenk equipped with a stirrer bar was charged with NaH (1.08 g, 27.0 mmol, 3.0 equiv, 60% in oil) and anhydrous DCM (10 mL). A solution of **S1** (2.29 g, 9.0 mmol, 1.0 equiv) in anhydrous DCM (10 mL) was slowly added at 0 °C. The mixture was allowed to stir for 30 min at room temperature under nitrogen atmosphere. Then *N*-fluorobenzenesulfonimide (NFSI, 8.51 g, 27.0 mmol, 3.0 equiv) in anhydrous DCM (20 mL) was added dropwise to the mixture and the resulting slurry was stirred for another 12 h. The reaction was quenched by saturated $NaHCO_3$ solution (20 mL) at 0 °C, and then extracted with DCM (20 mL × 3). The combined organic layers were washed sequentially with HCl (1.0 M) and brine, dried over anhydrous Na_2SO_4, filtered and concentrated *in vacuo*. The residue was purified by a flash column chromatography (PE/EA = 15:1) to afford the known product **2a**[15] as yellow oil (1.97 g, 7.2 mmol, 80%).

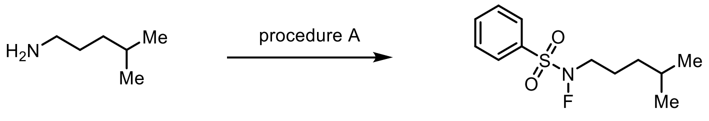

2b

Substrate **2b** was prepared in 68% overall yield as yellow oil following procedure A with known 4-methylpentan-1-amine and commercially available benzenesulfonyl chloride. **^{1}H NMR** (600 MHz, $CDCl_3$) δ 7.95 (d, J = 7.8 Hz, 2H), 7.74 (t, J = 7.2 Hz, 1H), 7.62 (t, J = 7.8 Hz, 2H), 3.21 (dt, J = 40.2, 6.6 Hz, 2H), 1.74 - 1.69 (m, 2H), 1.58 - 1.53 (m, 1H), 1.29 - 1.26 (m, 2H), 0.88 (d, J = 6.6 Hz, 6H). **^{13}C NMR** (150 MHz, $CDCl_3$) δ 135.0, 132.3, 130.1, 129.4, 54.1 (d, J = 12.2 Hz), 35.8, 27.8, 24.4, 22.6. **^{19}F NMR** (470 MHz, $CDCl_3$) δ -49.90 (t, J = 40.9 Hz). **IR** ν_{max} (film): 3067, 2956, 2871, 1770, 1739, 1585, 1468, 1449, 1375, 1312, 1296, 1242, 1214, 1185, 1089, 1049, 1024, 999, 897, 755, 743, 730, 688, 600, 580 cm^{-1}. **HRMS** (ESI) m/z calcd for $C_{12}H_{18}FNNaO_2S$ $[M+Na]^+$: 282.0934; found: 282.0932.

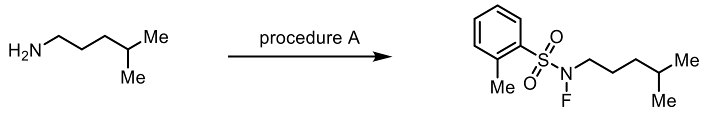

2c

Substrate **2c** was prepared in 70% overall yield as yellow oil following procedure A with known 4-methylpentan-1-amine and commercially available 2-methylbenzenesulfonyl chloride. **^{1}H NMR** (600 MHz, $CDCl_3$) δ 8.03 (d, J = 7.8 Hz, 1H), 7.58 (t, J = 7.8 Hz, 1H), 7.39 (t, J = 7.8 Hz, 2H), 3.44 (dt, J = 40.8, 7.2 Hz, 2H), 2.69 (s, 3H), 1.79 - 1.74 (m, 2H), 1.61 - 1.56 (m, 1H), 1.33 - 1.29 (m, 2H), 0.90 (d, J = 6.6 Hz, 6H). **^{13}C NMR** (150 MHz, $CDCl_3$) δ 140.7, 134.9, 133.1, 132.2, 131.8, 126.5, 51.9 (d, J = 12.6 Hz), 35.9, 27.8, 24.2, 22.6, 21.0. **^{19}F NMR** (470 MHz, $CDCl_3$) δ -49.97 (t, J = 40.8 Hz). **IR** ν_{max} (film): 3064, 2957, 2871, 1740, 1597, 1569, 1470, 1431, 1370, 1351, 1280, 1245, 1213, 1200, 1176, 1134, 1063, 1047, 994, 887, 807, 761, 728, 710, 660, 606, 590, 570, 542, 499, 462 cm^{-1}. **HRMS** (ESI) m/z calcd for $C_{13}H_{20}FNNaO_2S$ $[M+Na]^+$: 296.1091; found: 296.1080.

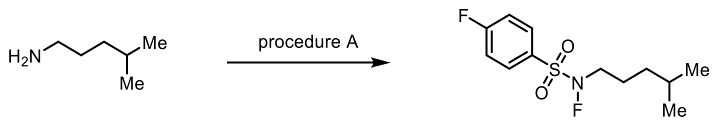

2e

Substrate **2e** was prepared in 67% overall yield as yellow oil following procedure A with known 4-methylpentan-1-amine and commercially available 4-fluorobenzenesulfonyl chloride. **^{1}H NMR** (600 MHz, $CDCl_3$) δ 7.98 (dd, J = 8.4, 5,4 Hz, 2H), 7.31 - 7.28 (m, 2H), 3.23 (dt, J = 40.8, 7.2 Hz, 2H), 1.75 - 1.70 (m, 2H), 1.59 - 1.52 (m, 1H), 1.29 - 1.26 (m, 2H), 0.88 (d, J = 6.6 Hz, 6H). **^{13}C NMR** (150 MHz, $CDCl_3$) δ 166.7 (d, J = 256.8 Hz), 133.0 (d, J = 9.8 Hz), 128.4 (d, J = 3.2 Hz), 116.9 (d, J = 22.7 Hz), 53.9 (d, J = 12.5 Hz), 35.8, 27.8, 24.3, 22.5. **^{19}F NMR** (470 MHz, $CDCl_3$) δ -49.53 (t, J = 40.4 Hz, 1F), -101.06 - (-101.11) (m, 1F). **IR** ν_{max} (film): 3107, 3079, 2957, 2872, 1683, 1653, 1592, 1558, 1540, 1493, 1469, 1429, 1407, 1381, 1295, 1242, 1183, 1158, 1090, 1054, 1014, 899, 841, 819, 728, 712, 700, 669, 653, 640, 576, 549, 490, 461, 418 cm^{-1}. **HRMS** (ESI) m/z calcd for $C_{12}H_{17}F_2NNaO_2S$ $[M+Na]^+$: 300.0840; found: 300.0832.

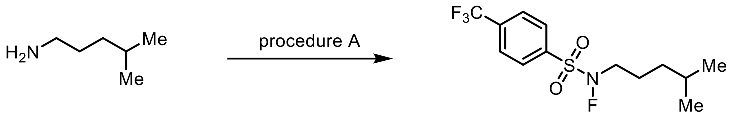

2f

Substrate **2f** was prepared in 73% overall yield as yellow oil following procedure A with known 4-methylpentan-1-amine and commercially available 4-(trifluoromethyl)benzenesulfonyl chloride. **^{1}H NMR** (600 MHz, $CDCl_3$) δ 8.10 (d, J = 7.8 Hz, 2H), 7.89 (d, J = 7.8 Hz, 2H), 3.26 (dt, J = 40.2, 7.2 Hz, 2H), 1.77 - 1.71 (m, 2H), 1.59 - 1.54 (m, 1H), 1.31 - 1.27 (m, 2H), 0.89 (d, J = 7.2 Hz, 6H). **^{13}C NMR** (150 MHz, $CDCl_3$) δ 136.5 (q, J = 33.3 Hz), 136.1, 130.6, 126.5, (q, J = 3.6 Hz), 123.1 (q, J = 271.5 Hz), 53.9 (d, J = 12.6 Hz), 35.7, 27.8, 24.3, 22.5. **^{19}F NMR** (470 MHz, $CDCl_3$) δ -49.67 (t, J = 40.0 Hz, 1F), -63.36 (s, 3F). **IR** ν_{max} (film): 2959, 2873, 1771, 1684, 1647, 1558, 1521, 1507, 1471, 1405, 1386, 1322, 1246, 1178, 1139, 1109, 1091, 1063, 1016, 844, 787, 719, 669, 617, 598, 561, 426 cm^{-1}. **HRMS** (ESI) m/z calcd for $C_{13}H_{17}F_4NNaO_2S$ $[M+Na]^+$: 350.0808; found: 350.0812.

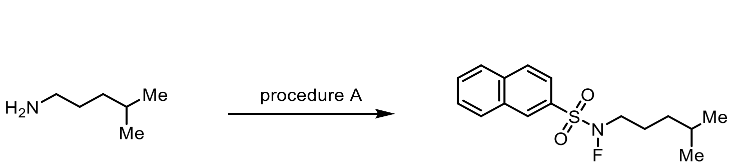

2g

Substrate **2g** was prepared in 69% overall yield as yellow oil following procedure A with known 4-methylpentan-1-amine and commercially available naphthalene-2-sulfonyl chloride. **^{1}H NMR** (600 MHz, $CDCl_3$) δ 8.54 (s, 1H), 8.04 (dd, J = 9.0, 3.6 Hz, 2H), 7.97 (d, J = 7.8 Hz, 1H), 7.90 (d, J = 9.0 Hz, 1H), 7.73 (t, J = 7.8 Hz, 1H), 7.67 (t, J = 7.8 Hz, 1H), 3.26 (dt, J = 40.8, 7.2 Hz, 2H), 1.75 - 1.69 (m, 2H), 1.57 - 1.53 (m, 1H), 1.29 - 1.26 (m, 2H), 0.87 (d, J = 6.6 Hz, 6H). **^{13}C NMR**

(150 MHz, $CDCl_3$) δ 136.0, 132.4, 132.1, 130.0, 129.7, 129.6, 129.1, 128.2, 128.1, 124.1, 54.2 (d, J = 12.5 Hz), 35.8, 27.8, 24.4, 22.6. **^{19}F NMR** (470 MHz, $CDCl_3$) δ -49.72 (t, J = 40.9 Hz). **IR** ν_{max} (film): 3058, 2956, 2870, 1770, 1758, 1739, 1625, 1589, 1505, 1467, 1429, 1378, 1269, 1242, 1175, 1133, 1074, 1051, 1019, 949, 900, 862, 817, 749, 722, 695, 644, 613, 589, 567, 549, 477 cm^{-1}. **HRMS** (ESI) m/z calcd for $C_{16}H_{20}FNNaO_2S$ $[M+Na]^+$: 332.1091; found: 332.1099.

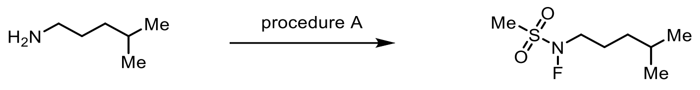

2h

Substrate **2h** was prepared in 56% overall yield as yellow oil following procedure A with known 4-methylpentan-1-amine and commercially available methanesulfonyl chloride. **^{1}H NMR** (500 MHz, $CDCl_3$) δ 3.44 (dt, J = 42.0, 7.0 Hz, 2H), 3.14 (d, J = 2.0 Hz, 3H), 1.82 - 1.76 (m, 2H), 1.62 - 1.57 (m, 1H), 1.33 - 1.29 (m, 2H), 0.91 (d, J = 7.0 Hz, 6H). **^{13}C NMR** (150 MHz, $CDCl_3$) δ 51.3 (d, J = 12.3 Hz), 36.2, 35.8, 27.8, 24.2, 22.6. **^{19}F NMR** (470 MHz, $CDCl_3$) δ -49.54 (t, J = 41.8 Hz). **IR** ν_{max} (film): 2956, 1770, 1683, 1652, 1591, 1558, 1522, 1507, 1493, 1457, 1379, 1294, 1242, 1182, 1157, 1089, 913, 840, 747, 669, 575, 548 cm^{-1}. **HRMS** (ESI) m/z calcd for $C_7H_{16}FNNaO_2S$ $[M+Na]^+$: 220.0778; found: 220.0785.

Procedure B

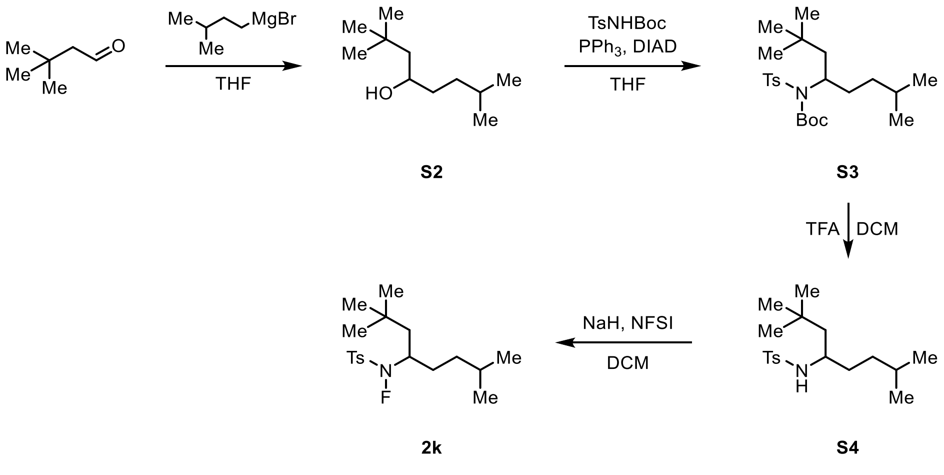

Synthesis of compound **S2**

Grignard Preparation: An I_2 crystal was added to a suspension of Mg turnings (875 mg, 36.0 mmol, 1.2 equiv) in anhydrous THF (20 mL). After the color disappeared (~ 2.0 min), 1-bromo-3-methylbutane (3.59 mL, 30.0 mmol, 1.0 equiv) was added slowly at a rate to maintain gentle reflux

(~5.0 min) and then stirred at 50 °C for 3 h. After that, the solution was cooled to 0 °C for use in the next step.

A solution of 3,3-dimethylbutyraldehyde (4.52 mL, 36.0 mmol, 1.2 equiv) in anhydrous THF (15 mL) was added dropwise to the above Grignard solution at 0 °C. After 15 min, the ice bath was removed. After being stirred for 2.5 h at room temperature, the reaction was re-cooled to 0 °C and quenched by dropwise addition of hydrochloric acid (1.0 M, 15 mL). The mixture was poured into H_2O (15 mL) and extracted with EtOAc (30 mL × 3). The combined organic extracts were washed with brine, dried over anhydrous Na_2SO_4, filtered, and concentrated *in vacuo*. The residue was purified by a flash column chromatography (PE/EA = 7:1) to afford the desired alcohol **S2** as colorless oil (3.72 g, 21.6 mmol, 72% from 1-bromo-3- methylbutane). **^{1}H NMR** (600 MHz, $CDCl_3$) δ 3.73 - 3.69 (m, 1H), 1.57 - 1.50 (m, 1H), 1.44 - 1.39 (m, 2H), 1.37 - 1.33 (m, 2H), 1.31 - 1.27 (m, 1H), 1.22 - 1.16 (m, 1H), 0.96 (s, 9H), 0.90 (d, *J* = 6.6 Hz, 3H), 0.89 (d, *J* = 6.6 Hz, 3H). **^{13}C NMR** (150 MHz, $CDCl_3$) δ 70.1, 51.5, 37.6, 34.9, 30.4, 30.3, 28.3, 22.9, 22.7. **IR** ν_{max} (film): 2952, 2928, 2868, 1733, 1716, 1683, 1558, 1540, 1506, 1465, 1457, 1363, 1243, 1188, 1175, 1024, 911, 814, 696, 669, 573, 548 cm^{-1}. **HRMS** (ESI) m/z calcd for $C_{11}H_{24}NaO$ $[M+Na]^+$: 195.1719; found: 195.1726.

Synthesis of compound **S4**

Step 1: PPh_3 (5.77 g, 22.0 mmol, 1.1 equiv), alcohol **S2** (3.44 g, 20.0 mmol, 1.0 equiv), and tert-butyl tosylcarbamate (5.96 g, 22.0 mmol, 1.1 equiv) were dissolved in anhydrous THF (40 mL). The mixture was stirred for 10 min at room temperature, and then cooled to 0 °C. Diisopropyl azodicarboxylate (DIAD, 4.33 mL, 22.0 mmol, 1.1 equiv) was added dropwise at 0 °C. The reaction was stirred at room temperature for 12 h. Once completion, the reaction was quenched with cold water (40 mL), and the mixture was extracted with EtOAc (30 mL × 3). The combined organic layers were washed with brine, dried over Na_2SO_4, and filtered. The solution was concentrated *in vacuo* and the residue was washed through a short column chromatography (PE/EA = 1:1) to afford the crude compound **S3**, which was used in the next step without further purification.

Step 2: The crude compound **S3** was dissolved in DCM (15 mL). Trifluoroacetic acid (TFA, 3.0 mL) was added at room temperature. The solution was stirred for 3 h until the complete consumption of compound **S3** (monitored by TLC). Excess acid was quenched with saturated $NaHCO_3$ solution (~40 mL). The organic phase was collected and the aqueous solution was extracted with DCM (30 mL × 3). The combined organic fractions were dried over $MgSO_4$, filtered, and concentrated *in vacuo*. Further purification by a flash column chromatography (PE/EA = 5:1)

afforded the desired product **S4** as colorless oil (3.80 g, 11.7 mmol, 58% from **S2**). **^{1}H NMR** (500 MHz, $CDCl_3$) δ 7.78 (d, J = 8.5 Hz, 2H), 7.28 (d, J = 8.5 Hz, 2H), 4.77 (d, J = 8.5 Hz, 1H), 3.36 - 3.30 (m, 1H), 2.41 (s, 3H), 1.34 - 1.27 (m, 4H), 1.22 - 1.16 (m, 1H), 1.03 - 0.97 (m, 2H), 0.85 (s, 9H), 0.72 (d, J = 7.0 Hz, 3H), 0.72 (d, J = 7.0 Hz, 3H). **^{13}C NMR** (150 MHz, $CDCl_3$) δ 143.1, 139.0, 129.6, 127.2, 51.8, 49.1, 34.8, 34.0, 30.4, 30.0, 27.9, 22.53, 22.46, 21.5. **IR** ν_{max} (film): 3284, 2954, 2868, 1670, 1598, 1526, 1466, 1422, 1384, 1325, 1287, 1158, 1119, 1094, 1026, 951, 925, 886, 814, 746, 666, 583, 550 cm^{-1}. **HRMS** (ESI) m/z calcd for $C_{18}H_{31}NNaO_2S$ [M+Na]$^+$: 348.1968; found: 348.1973.

Synthesis of compound **2k**

A flame dried Schlenk equipped with a stirrer bar was charged with NaH (1.20 g, 30.0 mmol, 3.0 equiv, 60% in oil) and anhydrous DCM (10 mL). A solution of **S4** (3.25 g, 10.0 mmol, 1.0 equiv) in anhydrous DCM (10 mL) was slowly added at 0 °C. The mixture was allowed to stir for 30 min at room temperature under nitrogen atmosphere. Then NFSI (9.46 g, 30.0 mmol, 3.0 equiv) in anhydrous DCM (20 mL) was added dropwise to the mixture and the resulting slurry was stirred for another 12 h. The reaction was quenched by saturated $NaHCO_3$ solution (20 mL) at 0 °C, and then extracted with DCM (20 mL × 3). The combined organic layers were washed sequentially with hydrochloric acid (1.0 M) and brine, dried over anhydrous Na_2SO_4, and filtered. The solvent was removed under reduced pressure and the residue was purified by a flash column chromatography (PE/EA = 15:1) to afford the desired product **2k** as yellow oil (2.16 g, 6.29 mmol, 63%). **^{1}H NMR** (600 MHz, $CDCl_3$) δ 7.83 (d, J = 7.2 Hz, 2H), 7.36 (d, J = 7.2 Hz, 2H), 4.08 - 3.97 (m, 1H), 2.46 (s, 3H), 1.85 - 1.82 (m, 1H), 1.77 - 1.71 (m, 1H), 1.53 - 1.47 (m, 2H), 1.36 - 1.33 (m, 1H), 1.24 - 1.19 (m, 2H), 0.97 (s, 9H), 0.86 (d, J = 6.6 Hz, 3H), 0.84 (d, J = 6.6 Hz, 3H). **^{13}C NMR** (150 MHz, $CDCl_3$) δ 145.6, 133.5, 129.9, 129.2, 60.8 (d, J = 13.2 Hz), 44.6, 36.0, 30.5, 30.3 (d, J = 9.2 Hz), 29.9, 28.1, 22.7, 22.5, 21.9. **^{19}F NMR** (565 MHz, $CDCl_3$) δ -82.71 (d, J = 41.8 Hz). **IR** ν_{max} (film): 2955, 2924, 2853, 1457, 1364, 1169, 706, 685, 669, 659, 577, 419 cm^{-1}. **HRMS** (ESI) m/z calcd for $C_{18}H_{30}FNNaO_2S$ [M+Na]$^+$: 366.1873; found: 366.1862.

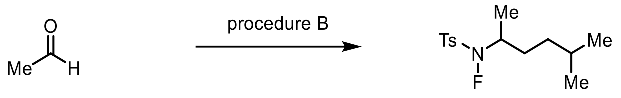

2i

Substrate **2i** was prepared in 30% overall yield as yellow oil following procedure B from commercially available acetaldehyde. **^{1}H NMR** (600 MHz, $CDCl_3$) δ 7.84 (d, J = 8.4 Hz, 2H), 7.34 (d, J = 8.4 Hz, 2H), 4.04 - 3.93 (m, 1H), 2.46 (s, 3H), 1.77 - 1.71 (m, 1H), 1.56 - 1.49 (m, 2H),

1.33 - 1.25 (m, 2H), 1.23 (d, *J* = 6.6 Hz, 3H), 0.88 (d, *J* = 6.6 Hz, 3H), 0.87 (d, *J* = 6.6 Hz, 3H). **^{13}C NMR** (150 MHz, $CDCl_3$) δ 145.8, 132.9, 130.0, 129.4, 59.6 (d, *J* = 12.9 Hz), 35.2, 32.3 (d, *J* = 2.1 Hz), 28.0, 22.65, 22.61, 21.9, 16.0 (d, *J* = 5.0 Hz). **^{19}F NMR** (470 MHz, $CDCl_3$) δ -80.22 (d, *J* = 37.6 Hz). **IR** ν_{max} (film): 2955, 2870, 1596, 1458, 1356, 1172, 1090, 890, 813, 706, 677, 573, 539 cm^{-1}. **HRMS** (ESI) m/z calcd for $C_{14}H_{22}FNNaO_2S$ [M+Na]$^+$: 310.1247; found: 310.1246.

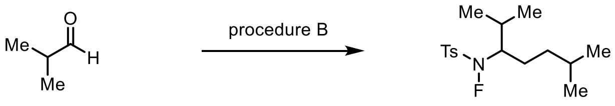

2j

Substrate **2j** was prepared in 32% overall yield as yellow oil following procedure B from commercially available isobutyraldehyde. **^{1}H NMR** (600 MHz, $CDCl_3$) δ 7.84 (d, *J* = 7.8 Hz, 2H), 7.37 (d, *J* = 7.8 Hz, 2H), 3.76 - 3.67 (m, 1H), 2.46 (s, 3H), 2.04 - 2.00 (m, 1H), 1.55 - 1.53 (m, 1H), 1.43 - 1.36 (m, 2H), 1.16 - 1.11 (m, 2H), 1.03 (d, *J* = 6.6 Hz, 3H), 0.94 (d, *J* = 6.6 Hz, 3H), 0.82 (d, *J* = 6.6 Hz, 3H), 0.77 (d, *J* = 6.6 Hz, 3H). **^{13}C NMR** (150 MHz, $CDCl_3$) δ 145.7, 133.0, 130.0, 129.3, 68.3 (d, *J* = 11.9 Hz), 36.3, 30.2, 29.9, 28.2, 24.7 (d, *J* = 12.8 Hz), 22.5 (d, *J* = 11.7 Hz), 21.8, 20.2, 17.8. **^{19}F NMR** (470 MHz, $CDCl_3$) δ -75.50 (d, *J* = 37.1 Hz). **IR** ν_{max} (film): 2960, 2926, 2872, 1748, 1558, 1540, 1507, 1457, 1369, 1187, 1170, 897, 813, 667, 590 cm^{-1}. **HRMS** (ESI) m/z calcd for $C_{16}H_{26}FNNaO_2S$ [M+Na]$^+$: 338.1560; found: 338.1563.

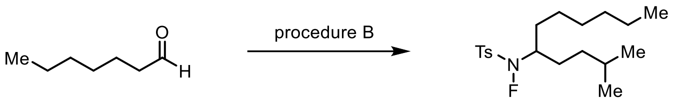

2l

Substrate **2l** was prepared in 28% overall yield as yellow oil following procedure B from commercially available heptanal. **^{1}H NMR** (600 MHz, $CDCl_3$) δ 7.83 (d, *J* = 7.8 Hz, 2H), 7.37 (d, *J* = 7.8 Hz, 2H), 3.86 - 3.76 (m, 1H), 2.46 (s, 3H), 1.64 - 1.61 (m, 2H), 1.59 - 1.55 (m, 2H), 1.51 - 1.46 (m, 1H), 1.38 - 1.35 (m, 2H), 1.29 - 1.27 (m, 3H), 1.26 - 1.22 (m, 5H), 0.89 - 0.86 (m, 6H), 0.84 (d, *J* = 6.6 Hz, 3H). **^{13}C NMR** (150 MHz, $CDCl_3$) δ 145.7, 133.2, 130.0, 129.3, 64.0 (d, *J* = 12.6 Hz), 35.5, 31.8, 31.0 (d, *J* = 5.7 Hz), 29.2, 28.8 (d, *J* = 6.2 Hz), 28.1, 26.4, 22.73, 22.66, 22.5, 21.9, 14.2. **^{19}F NMR** (470 MHz, $CDCl_3$) δ -80.03 (d, *J* = 38.1 Hz). **IR** ν_{max} (film): 2956, 2929, 2870, 1772, 1733, 1716, 1698, 1683, 1669, 1653, 1647, 1635, 1623, 1616, 1593, 1558, 1540, 1533, 1521, 1507, 1493, 1466, 1457, 1436, 1406, 1379, 1294, 1242, 1213, 1172, 1158, 1091, 1019, 898, 841, 814, 753, 686, 576, 548 cm^{-1}. **HRMS** (ESI) m/z calcd for $C_{19}H_{32}FNNaO_2S$ [M+Na]$^+$: 380.2030; found: 380.2013.

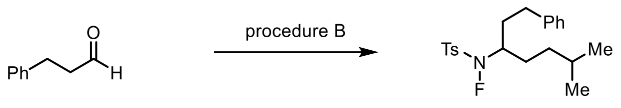

2n

Substrate **2n** was prepared in 27% overall yield as yellow oil following procedure B from commercially available 3-phenylpropanal. **^{1}H NMR** (600 MHz, $CDCl_3$) δ 7.83 (d, J = 8.4 Hz, 2H), 7.37 (d, J = 8.4 Hz, 2H), 7.28 (t, J = 7.2 Hz, 2H), 7.19 (t, J = 7.2 Hz, 1H), 7.15 (d, J = 7.2 Hz, 2H), 3.91 - 3.81 (m, 1H), 2.77 - 2.72 (m, 1H), 2.69 - 2.65 (m, 1H), 2.46 (s, 3H), 2.01 - 1.95 (m, 1H), 1.92 - 1.86 (m, 1H), 1.70 - 1.64 (m, 1H), 1.63 - 1.57 (m, 1H), 1.47 - 1.43 (m, 1H), 1.22 - 1.18 (m, 2H), 0.84 (d, J = 6.6 Hz, 3H), 0.81 (d, J = 6.6 Hz, 3H). **^{13}C NMR** (150 MHz, $CDCl_3$) δ 145.8, 141.4, 133.1, 130.0, 129.3, 128.57, 128.56, 126.2, 63.40 (d, J = 12.8 Hz), 35.5, 32.9 (d, J = 4.7 Hz), 32.6, 28.7 (d, J = 6.6 Hz), 28.0, 22.6, 21.9. **^{19}F NMR** (470 MHz, $CDCl_3$) δ -79.43 (d, J = 37.6 Hz). **IR** ν_{max} (film): 3063, 3027, 2956, 2869, 1597, 1495, 1454, 1353, 1306, 1293, 1261, 1213, 1187, 1120, 1091, 892, 814, 748, 702, 685, 579, 539 cm^{-1}. **HRMS** (ESI) m/z calcd for $C_{21}H_{28}FNNaO_2S$ $[M+Na]^+$: 400.1717; found: 400.1716.

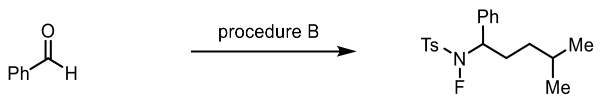

2o

Substrate **2o** was prepared in 34% overall yield as yellow oil following procedure B from commercially available benzaldehyde. **^{1}H NMR** (600 MHz, $CDCl_3$) δ 7.65 (d, J = 7.8 Hz, 2H), 7.25 - 7.20 (m, 7H), 4.72 (ddd, J = 37.2, 9.0, 6.6 Hz, 1H), 2.39 (s, 3H), 2.18 - 2.12 (m, 1H), 1.98 - 1.91 (m, 1H), 1.55 - 1.48 (m, 1H), 1.25 - 1.19 (m, 1H), 1.06 - 0.99 (m, 1H), 0.83 (d, J = 6.6 Hz, 6H). **^{13}C NMR** (150 MHz, $CDCl_3$) δ 145.5, 136.80, 136.77, 132.0, 129.6, 129.5, 128.8, 128.43, 128.35, 68.2 (d, J = 12.2, Hz), 35.2, 31.0 (d, J = 4.8 Hz), 27.9, 22.6, 22.4, 21.8. **^{19}F NMR** (470 MHz, $CDCl_3$) δ -70.97 (d, J = 38.1 Hz). **IR** ν_{max} (film): 3089, 3065, 3032, 2956, 2870, 1684, 1652, 1596, 1558, 1540, 1495, 1467, 1455, 1369, 1307, 1295, 1213, 1187, 1121, 1090, 1019, 1004, 966, 919, 813, 760, 720, 668, 608, 564, 545, 532, 419 cm^{-1}. **HRMS** (ESI) m/z calcd for $C_{19}H_{24}FNNaO_2S$ $[M+Na]^+$: 372.1404; found: 372.1397

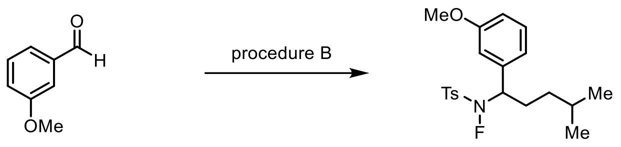

2p

Substrate **2p** was prepared in 30% overall yield as yellow oil following procedure B from commercially available 3-methoxybenzaldehyde. **^{1}H NMR** (600 MHz, $CDCl_3$) δ 7.64 (d, J = 8.4 Hz, 2H), 7.20 (d, J = 8.4 Hz, 2H), 7.14 (t, J = 7.8 Hz, 1H), 6.82 (d, J = 7.8 Hz, 1H), 6.77 (dd, J = 7.8, 2.4 Hz, 1H), 6.72, (t, J = 2.4 Hz, 1H), 4.68 (ddd, J = 37.8, 8.4, 6.0 Hz, 1H), 3.74, (s, 3H), 2.39 (s, 3H), 2.15 - 2.09 (m, 1H), 1.95 - 1.89 (m, 1H), 1.54 - 1.48 (m, 1H), 1.24 - 1.19 (m, 1H), 1.07 - 1.01 (m, 1H), 0.83 (d, J = 6.6 Hz, 6H). **^{13}C NMR** (150 MHz, $CDCl_3$) δ 159.5, 145.4, 138.2, 131.9, 129.53, 129.48, 129.37, 121.3, 114.3, 113.7, 68.3 (d, J = 12.2 Hz), 55.3, 35.2, 31.1 (d, J = 4.7 Hz), 27.9, 22.6 22.5, 21.8. **^{19}F NMR** (470 MHz, $CDCl_3$) δ -71.43 (d, J = 38.1 Hz). **IR** ν_{max} (film): 3107, 3079, 2957, 2872, 1592, 1493, 1469, 1407, 1381, 1295, 1242, 1183, 1158, 1090, 1054, 1014, 899, 841, 819, 728, 712, 700, 669, 653, 640, 576, 549, 490, 461, 418 cm^{-1}. **HRMS** (ESI) m/z calcd for $C_{20}H_{26}FNNaO_3S$ $[M+Na]^+$: 402.1510; found: 402.1507.

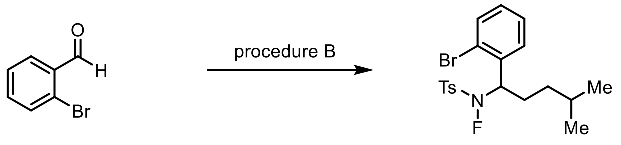

2q

Substrate **2q** was prepared in 32% overall yield as yellow oil following procedure B from commercially available 2-bromobenzaldehyde. **^{1}H NMR** (600 MHz, $CDCl_3$) δ 7.73 (d, J = 8.4 Hz, 2H), 7.50 (d, J = 7.8 Hz, 1H), 7.37 (d, J = 7.8 Hz, 1H), 7.23 (d, J = 8.4 Hz, 2H), 7.14 (t, J = 7.8 Hz, 1H), 7.07 (td, J = 7.8, 1.8 Hz, 1H), 5.43 (ddd, J = 36.0, 9.0, 6.0 Hz, 1H), 2.39 (s, 3H), 2.15 - 2.10 (m, 1H), 1.98 - 1.92 (m, 1H), 1.54 - 1.49 (m, 1H), 1.29 - 1.23 (m, 1H), 1.07 - 1.01 (m, 1H), 0.83 (d, J = 6.6 Hz, 6H). **^{13}C NMR** (150 MHz, $CDCl_3$) δ 145.7, 136.8, 133.0, 131.5, 129.75, 129.73, 129.6, 129.5, 127.7, 124.6, 65.9 (d, J = 12.0 Hz), 34.4, 31.0 (d, J = 5.9 Hz), 27.9, 22.6, 22.5, 21.8. **^{19}F NMR** (470 MHz, $CDCl_3$) δ -71.50 (d, J = 35.7 Hz). **IR** ν_{max} (film): 2956, 2870, 1771, 1471, 1374, 1241, 1188, 1173, 1090, 1025, 912, 813, 745, 617, 566, 545 cm^{-1}. **HRMS** (ESI) m/z calcd for $C_{19}H_{23}BrFNNaO_2S$ $[M+Na]^+$: 450.0509; found: 450.0507.

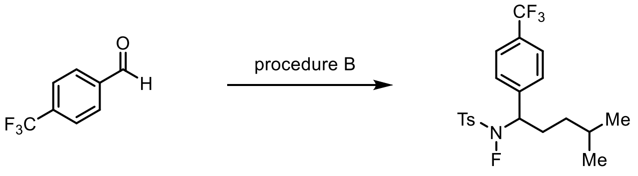

2r

Substrate **2r** was prepared in 35% overall yield as yellow oil following procedure B from commercially available 4-(trifluoromethyl)benzaldehyde acetaldehyde. **^{1}H NMR** (600 MHz,

CDCl3) δ 7.57 (d, *J* = 8.4 Hz, 2H), 7.44 (d, *J* = 8.4 Hz, 2H), 7.32 (d, *J* = 8.4 Hz, 2H), 7.17 (d, *J* = 8.4 Hz, 2H), 4.80 (ddd, *J* = 39.0, 8.4, 6.6 Hz, 1H), 2.38 (s, 3H), 2.21 - 2.14 (m, 1H), 1.95 - 1.89 (m, 1H), 1.57 - 1.50 (m, 1H), 1.29 - 1.23 (m, 1H), 1.07 - 1.00 (m, 1H), 0.84 (dd, *J* = 6.6, 3.0 Hz, 6H). **^{13}C NMR** (150 MHz, $CDCl_3$) δ 145.8, 140.5, 131.6, 130.5 (q, *J* = 32.1 Hz), 129.6, 129.4, 129.2, 125.4 (q, *J* = 3.6 Hz), 124.1 (q, *J* = 270.6 Hz), 67.7 (d, *J* = 12.2 Hz), 35.1, 31.3, (d, *J* = 4.1 Hz), 27.9, 22.6, 22.4, 21.7. **^{19}F NMR** (470 MHz, $CDCl_3$) δ -62.69 (s, 3F), -73.17 (d, *J* = 39.0 Hz, 1F). **IR** ν_{max} (film): 2958, 2872, 1621, 1596, 1420, 1373, 1326, 1326, 1172, 1128, 1069, 1019, 840, 813, 674, 580, 564 cm^{-1}. **HRMS** (ESI) m/z calcd for $C_{20}H_{23}F_4NNaO_2S$ $[M+Na]^+$: 440.1278; found: 440.1282.

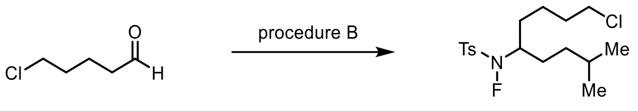

2t

Substrate **2t** was prepared in 37% overall yield as yellow oil following procedure B from commercially available 5-chloropentanal. **^{1}H NMR** (600 MHz, $CDCl_3$) δ 7.84 (d, *J* = 8.4 Hz, 2H), 7.38 (d, *J* = 8.4 Hz, 2H), 3.89 - 3.78 (m, 1H), 3.52 (t, *J* = 6.6 Hz, 2H), 2.47 (s, 3H), 1.81 - 1.73 (m, 2H), 1.72 - 1.59 (m, 4H), 1.58 - 1.56 (m, 1H), 1.54 - 1.52 (m, 1H), 1.51 - 1.47 (m, 1H), 1.25 - 1.21 (m, 2H), 0.86 (d, *J* = 6.6 Hz, 3H), 0.84 (d, *J* = 6.6 Hz, 3H). **^{13}C NMR** (150 MHz, $CDCl_3$) δ 145.9, 133.0, 130.0, 129.3, 63.7 (d, *J* = 12.6 Hz), 44.8, 35.5, 32.4, 30.4 (d, *J* = 5.4 Hz), 28.7 (d, *J* = 6.3 Hz), 28.1, 23.8, 22.6, 22.5, 21.9. **^{19}F NMR** (470 MHz, $CDCl_3$) δ -79.77 (d, *J* = 39.0 Hz). **IR** ν_{max} (film): 2956, 2870, 1596, 1458, 1374, 1306, 1171, 1091, 1019, 893, 814, 705, 685, 576, 542 cm^{-1}. **HRMS** (ESI) m/z calcd for $C_{17}H_{27}ClFNNaO_2S$ $[M+Na]^+$: 386.1327; found: 386.1327.

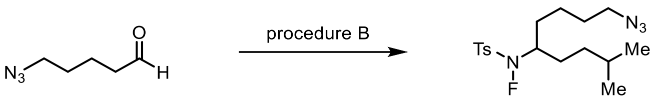

2u

Substrate **2u** was prepared in 28% overall yield as yellow oil following procedure B from known 5-azidopentanal[16]. **^{1}H NMR** (600 MHz, $CDCl_3$) δ 7.84 (d, *J* = 8.4 Hz, 2H), 7.38 (d, *J* = 8.4 Hz, 2H), 3.89 - 3.78 (m, 1H), 3.27 (t, *J* = 6.6 Hz, 2H), 2.47 (s, 3H), 1.74 - 1.69 (m, 1H), 1.67 - 1.53 (m, 6H), 1.49 - 1.46 (m, 2H), 1.25 - 1.21 (m, 2H), 0.86 (d, *J* = 6.6 Hz, 3H), 0.84 (d, *J* = 6.6 Hz, 3H). **^{13}C NMR** (150 MHz, $CDCl_3$) δ 145.9, 133.0, 130.0, 129.3, 63.7 (d, *J* = 12.6 Hz), 51.4, 35.6, 30.7 (d, *J* = 5.1 Hz), 28.8, 28.7 (d, *J* = 6.5 Hz), 28.1 23.6, 22.6, 22.5, 21.9. **^{19}F NMR** (470 MHz, $CDCl_3$) δ -79.77 (d, *J* = 38.1 Hz). **IR** ν_{max} (film): 2955, 2870, 2095, 1596, 1457, 1374, 1353, 1292, 1259, 1170, 1090, 814, 705, 685, 576, 542 cm^{-1}. **HRMS** (ESI) m/z calcd for $C_{17}H_{27}FN_4NaO_2S$ $[M+Na]^+$:

393.1731; found: 393.1735.

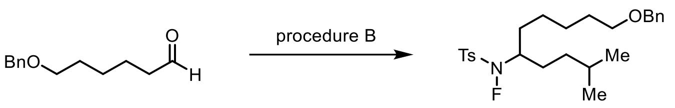

2v

Substrate **2v** was prepared in 27% overall yield as yellow oil following procedure B from known 6-(benzyloxy)hexanal[17]. **^{1}H NMR** (600 MHz, $CDCl_3$) δ 7.83 (d, J = 8.4 Hz, 2H), 7.36 - 7.33 (m, 6H), 7.29 - 7.26 (m, 1H), 4.49 (s, 2H), 3.86 - 3.76 (m, 1H), 3.45 (t, J = 6.6 Hz, 2H), 2.44 (s, 3H), 1.65 - 1.57 (m, 6H), 1.49 - 1.45 (m, 1H), 1.41 - 1.34 (m, 4H), 1.25 - 1.20 (m, 2H), 0.85 (d, J = 6.6 Hz, 3H), 0.83 (d, J = 6.6 Hz, 3H). **^{13}C NMR** (150 MHz, $CDCl_3$) δ 145.7, 138.8, 133.2, 130.0, 129.3, 128.5, 127.7, 127.6, 73.0, 70.4, 64.0 (d, J = 12.5 Hz), 35.5, 31.0 (d, J = 5.1 Hz), 29.7, 28.8 (d, J = 6.3 Hz), 28.0, 26.3, 26.2, 22.6, 22.5, 21.8. **^{19}F NMR** (470 MHz, $CDCl_3$) δ -79.88 (d, J = 38.1 Hz). **IR** ν_{max} (film): 2930, 2865, 1545, 1355, 1170, 1091, 814, 735, 685, 576, 545 cm^{-1}. **HRMS** (ESI) m/z calcd for $C_{25}H_{36}FNNaO_3S$ $[M+Na]^+$: 472.2292; found: 472.2297.

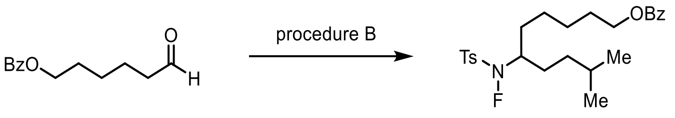

2w

Substrate **2w** was prepared in 29% overall yield as yellow oil following procedure B from known 6-oxohexyl benzoate[18]. **^{1}H NMR** (600 MHz, $CDCl_3$) δ 8.05 (d, J = 7.8 Hz, 2H), 7.83 (d, J = 7.8 Hz, 2H), 7.55 (t, J = 7.8 Hz, 1H), 7.44 (t, J = 7.8 Hz, 2H), 7.36 (d, J = 7.8 Hz, 2H), 4.31 (t, J = 6.6 Hz, 2H), 3.89 - 3.78 (m, 1H), 2.45 (s, 3H), 1.78 - 1.74 (m, 2H), 1.71 - 1.67 (m, 1H), 1.66 - 1.61 (m, 2H), 1.58 - 1.55 (m, 1H), 1.50 - 1.42 (m, 5H), 1.25 - 1.21 (m, 2H), 0.85 (d, J = 6.6 Hz, 3H), 0.83 (d, J = 6.6 Hz, 3H). **^{13}C NMR** (150 MHz, $CDCl_3$) δ 166.8, 145.8, 133.1, 133.0, 130.6, 130.0, 129.7, 129.3, 128.5, 65.1, 63.9 (d, J = 12.5 Hz), 35.6, 31.0 (d, J = 5.3 Hz), 28.8 (d, J = 7.5 Hz), 28.1, 26.2, 26.1, 22.6, 22.5 21.8. **^{19}F NMR** (470 MHz, $CDCl_3$) δ -79.92 (d, J = 38.1 Hz). **IR** ν_{max} (film): 2954, 2868, 1718, 1597, 1451, 1374, 1354, 1274, 1187, 1170, 1111, 1091, 1070, 1026, 814, 712, 685, 576, 556 cm^{-1}. **HRMS** (ESI) m/z calcd for $C_{25}H_{34}FNNaO_4S$ $[M+Na]^+$: 486.2085; found: 486.2097.

Procedure C

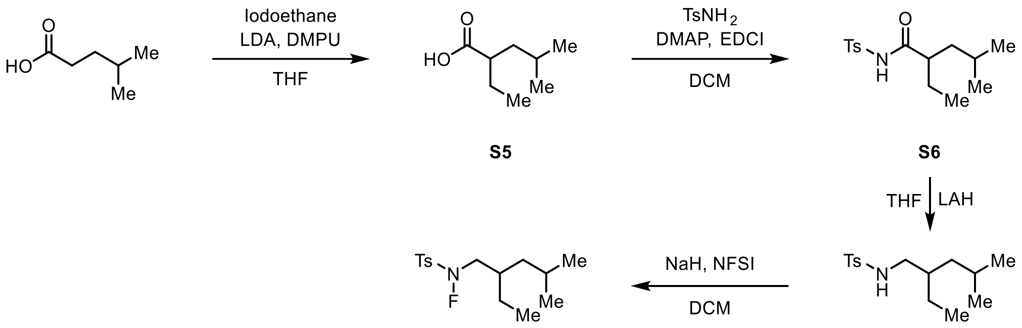

S5 **S6** **2m** **S7**

Synthesis of compound **S5**

A solution of 4-methylpentanoic acid (3.46 mL, 27.5 mmol, 1.0 equiv) in anhydrous THF (10 mL) was added to LDA (30.3 mL, 60.5 mmol, 2.2 equiv, 2.0 M in hexane) at -15 °C, and the mixture was allowed to warm up to 0 °C during a period of about 15 min. Then 1,3-dimethyl-perhydro-2-pyrimidinone (DMPU, 3.33 mL, 27.5 mmol, 1.0 equiv) was added dropwise at 0 °C, and the mixture was allowed to stir for additional 30 min followed by a dropwise addition of iodoethane (4.84 mL, 60.5 mmol, 2.2 equiv) in anhydrous THF (10 mL). Then the reaction mixture was stirred at room temperature for 2 h. THF was removed under reduced pressure and the oily residue was dispersed in petroleum ether (50 mL). Hydrochloric acid (1.0 M) solution was added to the mixture until pH 1-2. The organic phase was separated from the aqueous phase, and the aqueous phase was extracted with petroleum ether (50 mL × 3). The combined petroleum ether extracts were washed with brine, dried over anhydrous Na_2SO_4, filtered, and concentrated *in vacuo* to give crude **S5** (about 26.0 mmol, 95% yield, determinate by ^{1}H NMR), which can be used directly for the next step without further purification.

Synthesis of compound **S6**

To a solution of $TsNH_2$ (4.45 g, 26.0 mmol, 1.0 equiv), 1-ethyl-3-(3-dimethylaminopropyl) carbodiimide hydrochloride (EDCI, 5.98 g, 31.2 mmol, 1.2 equiv), and 4-dimethylaminopyridine (DMAP, 0.32 g, 2.60 mmol, 0.10 equiv) in DCM (80 mL) was added crude **S5** (about 26.0 mmol, 1.0 equiv) at room temperature. The reaction mixture was stirred at room temperature for 12 h, and then washed sequentially with H_2O and brine. The organic phase was dried over anhydrous $MgSO_4$, filtered, and concentrated *in vacuo*. The residue was washed through a short column chromatography (PE/EA = 1:1) to afford the crude compound **S6** (about 20.0 mmol, 77% yield, determinate by ^{1}H NMR), which was used in the next step without further purification.

Synthesis of compound **S7**

To a solution of crude **S6** (5.94 g, 20.0 mmol, 1.0 equiv) in anhydrous THF (40 mL) was added $LiAlH_4$ (LAH, 1.90 g, 50.0 mmol, 2.5 equiv) in 3 portions at -10 °C. The reaction mixture was stirred at room temperature until completion (monitored by TLC), and then cooled to -10 °C. H_2O (3.0 mL) was added dropwise. After 10 min, NaOH (15 wt% in H_2O, 3.0 mL) was added dropwise. The mixture was stirred for an additional 10 min, dried over anhydrous $MgSO_4$, filtered, and concentrated *in vacuo*. Further purification by a flash column chromatography (PE/EA = 10:1) afforded the desired product **S7** as yellow oil (3.28 g, 11.6 mmol, 58%). **^{1}H NMR** (600 MHz, $CDCl_3$) δ 7.75 (d, J = 7.8 Hz, 2H), 7.31 (d, J = 7.8 Hz, 2H), 4.56 - 4.35 (m, 1H), 2.88 - 2.79 (m, 2H), 2.43 (s, 3H), 1.54 - 1.48 (m, 1H), 1.45 - 1.41 (m, 1H), 1.29 - 1.24 (m, 2H), 1.06 - 1.01 (m, 2H), 0.82 - 0.76 (m, 9H). **^{13}C NMR** (150 MHz, $CDCl_3$) δ 143.4, 137.2, 129.8, 127.3, 46.0, 40.8, 36.9, 25.2, 24.2, 22.87, 22.85, 21.6, 10.6. **IR** ν_{max} (film): 3285, 2957, 2927, 2871, 1598, 1495, 1464, 1426, 1384, 1366, 1326, 1161, 1094, 845, 814, 706, 663, 572, 551 cm^{-1}. **HRMS** (ESI) m/z calcd for $C_{15}H_{25}NNaO_2S$ $[M+Na]^+$: 306.1498; found: 306.1492.

Synthesis of compound **2m**

A flame dried Schlenk equipped with a stirrer bar was charged with NaH (1.20 g, 30.0 mmol, 3.0 equiv, 60% in oil) and anhydrous DCM (10 mL). A solution of **S7** (2.83 g, 10.0 mmol, 1.0 equiv) in anhydrous DCM (10 mL) was slowly added at 0 °C. The mixture was allowed to stir for 30 min at room temperature under nitrogen atmosphere. Then NFSI (9.46 g, 30.0 mmol, 3.0 equiv) in anhydrous DCM (20 mL) was added dropwise to the mixture and the resulting slurry was stirred for another 12 h. The reaction was quenched by saturated $NaHCO_3$ solution (20 mL) at 0 °C, and then extracted with DCM (20 mL × 3). The combined organic layers were washed sequentially with hydrochloric acid (1.0 M) and brine, dried over anhydrous Na_2SO_4, and filtered. The solvent was removed under reduced pressure, and the residue was purified by a flash column chromatography (PE/EA = 15:1) to afford the desired product **2m** as yellow oil (2.20 g, 7.3 mmol, 73%). **^{1}H NMR** (600 MHz, $CDCl_3$) δ 7.82 (d, J = 8.4 Hz, 2H), 7.41 (d, J = 8.4 Hz, 2H), 3.19 - 3.00 (m, 2H), 2.49 (s, 3H), 1.82 - 1.75 (m, 1H), 1.63 - 1.59 (m, 1H), 1.48 - 1.39 (m, 2H), 1.21 - 1.19 (m, 2H), 0.86 - 0.84 (m, 9H). **^{13}C NMR** (150 MHz, $CDCl_3$) δ 146.2, 130.1, 130.0, 129.3, 57.1 (d, J = 11.9 Hz), 41.0, 34.6, 25.2, 24.5, 23.0, 22.7, 21.9, 10.3. **^{19}F NMR** (470 MHz, $CDCl_3$) δ -46.05 (t, J = 41.8 Hz). **IR** ν_{max} (film): 2960, 2931, 2873, 1596, 1458, 1424, 1376, 1175, 1090, 814, 730, 667, 653, 578, 553 cm^{-1}. **HRMS** (ESI) m/z calcd for $C_{15}H_{24}FNNaO_2S$ $[M+Na]^+$: 324.1404; found: 324.1406.

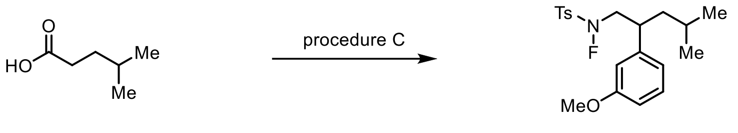

2s

Substrate **2s** was prepared in 24% overall yield as yellow oil following procedure C from commercially available 4-methylpentanoic acid. **^{1}H NMR** (600 MHz, $CDCl_3$) δ 7.77 (d, J = 7.8 Hz, 2H), 7.37 (d, J = 7.8 Hz, 2H), 7.17 (t, J = 7.8 Hz, 1H), 6.74 - 6.71 (m, 2H), 6.68 (s, 1H), 3.79 (s, 3H), 3.18 - 2.99 (m, 2H), 2.72 - 2.65 (m, 2H), 2.47 (s, 3H), 2.17 - 2.12 (m, 1H), 1.67 - 1.63 (m, 1H), 1.28 - 1.18 (m, 2H), 0.85 (d, J = 6.6 Hz, 3H), 0.82 (d, J = 6.6 Hz, 3H). **^{13}C NMR** (150 MHz, $CDCl_3$) δ 159.7, 146.3, 141.1, 130.0, 129.3, 129.1, 122.0, 115.1, 111.8, 56.9 (d, J = 11.6 Hz), 55.3, 41.2, 38.4, 35.4, 25.2, 22.8, 22.7, 21.9. **^{19}F NMR** (470 MHz, $CDCl_3$) δ -45.07 (t, J = 41.4 Hz). **IR** ν_{max} (film): 2954, 2925, 2868, 1749, 1597, 1584, 1489, 1465, 1376, 1295, 1261, 1175, 1089, 1045, 814, 783, 731, 697, 578, 552 cm^{-1}. **HRMS** (ESI) m/z calcd for $C_{21}H_{28}FNNaO_3S$ $[M+Na]^+$: 416.1666; found: 416.1658.

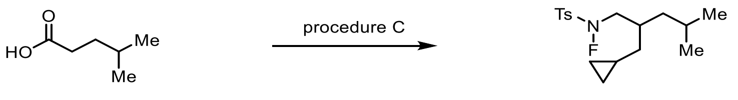

2y

Substrate **2y** was prepared in 28% overall yield as yellow oil following procedure C from commercially available 4-methylpentanoic acid. **^{1}H NMR** (600 MHz, $CDCl_3$) δ 7.82 (d, J = 8.4 Hz, 2H), 7.40 (d, J = 8.4 Hz, 2H), 3.25 (ddd, J = 42.0, 14.4, 6.6 Hz, 1H), 3.11 (ddd, J = 43.2, 14.4, 6.6 Hz, 1H), 2.48 (s, 3H), 1.98 - 1.92 (m, 1H), 1.66 - 1.59 (m, 1H), 1.37 - 1.32 (m, 1H), 1.29 - 1.24 (m, 3H), 0.86 (d, J = 6.6 Hz, 3H), 0.84 (d, J = 6.6 Hz, 3H), 0.65 - 0.59 (m, 1H), 0.44 - 0.37 (m, 2H), 0.04 - (-0.04) (m, 2H). **^{13}C NMR** (150 MHz, $CDCl_3$) δ 146.2, 130.1, 130.0, 129.2, 57.6 (d, J = 11.7 Hz), 41.5, 36.9, 34.3, 25.2, 23.1, 22.6, 21.9, 7.9, 4.9, 4.8. **^{19}F NMR** (470 MHz, $CDCl_3$) δ -46.15 (t, J = 42.3 Hz). **IR** ν_{max} (film): 3001, 2956, 2925, 2869, 1772, 1375, 1307, 1175, 1090, 1018, 814, 733, 653, 579, 553, 472, 418 cm^{-1}. **HRMS** (ESI) m/z calcd for $C_{17}H_{26}FNNaO_2S$ $[M+Na]^+$: 350.1560; found: 350.1567.

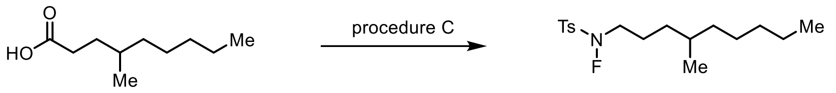

2z

Substrate **2z** was prepared in 36% overall yield as yellow oil following procedure C from

commercially available 4-methylnonanoic acid. **^{1}H NMR** (600 MHz, $CDCl_3$) δ 7.82 (d, J = 8.4 Hz, 2H), 7.41 (d, J = 8.4 Hz, 2H), 3.25 (dt, J = 40.8, 6.6 Hz, 2H), 2.48 (s, 3H), 1.78 - 1.70 (m, 1H), 1.69 - 1.63 (m, 1H), 1.41 - 1.36 (m, 2H), 1.30 - 1.19 (m, 8H), 1.12 - 1.06 (m, 1H), 0.88 (t, J = 7.2 Hz, 3H), 0.85 (d, J = 6.0 Hz, 3H). **^{13}C NMR** (150 MHz, $CDCl_3$) δ 146.3, 130.1, 130.0, 129.1, 54.2 (d, J = 12.2 Hz), 36.9, 33.9, 32.5, 32.3, 26.7, 24.1, 22.8, 21.9, 19.6, 14.2. **^{19}F NMR** (470 MHz, $CDCl_3$) δ -49.87 (t, J = 40.9 Hz). **IR** ν_{max} (film): 3957, 3064, 2871, 1596, 1569, 1470, 1369, 1351, 1280, 1213, 1176, 1134, 1063, 994, 887, 807, 761, 728, 710, 606, 590, 570, 542, 499, 462 cm^{-1}. **HRMS** (ESI) m/z calcd for $C_{17}H_{28}FNNaO_2S$ $[M+Na]^+$: 352.1717; found: 352.1711.

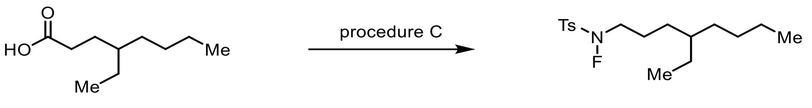

2aa

Substrate **2aa** was prepared in 38% overall yield as yellow oil following procedure C from commercially available 4-ethyloctanoic acid. **^{1}H NMR** (600 MHz, $CDCl_3$) δ 7.82 (d, J = 8.4 Hz, 2H), 7.41 (d, J = 8.4 Hz, 2H), 3.19 (dt, J = 40.8, 7.2 Hz, 2H), 2.48 (s, 3H), 1.69 - 1.66 (m, 2H), 1.34 - 1.31 (m, 2H), 1.29 - 1.21 (m, 9H), 0.88 (t, J = 7.2 Hz, 3H), 0.82 (t, J = 7.2 Hz, 3H). **^{13}C NMR** (150 MHz, $CDCl_3$) δ 146.3, 130.1, 130.0, 129.1, 54.3 (d, J = 12.5 Hz), 38.6, 32.8, 30.2, 29.0, 25.9, 23.8, 23.2, 21.9, 14.2, 10.9. **^{19}F NMR** (470 MHz, $CDCl_3$) δ -49.89 (t, J = 40.0 Hz). **IR** ν_{max} (film): 2958, 2928, 2859, 1596, 1492, 1458, 1377, 1307, 1295, 1175, 1121, 1090, 1019, 896, 814, 749, 724, 706, 696, 650, 576, 550 cm^{-1}. **HRMS** (ESI) m/z calcd for $C_{17}H_{28}FNNaO_2S$ $[M+Na]^+$: 352.1717; found: 352.1718.

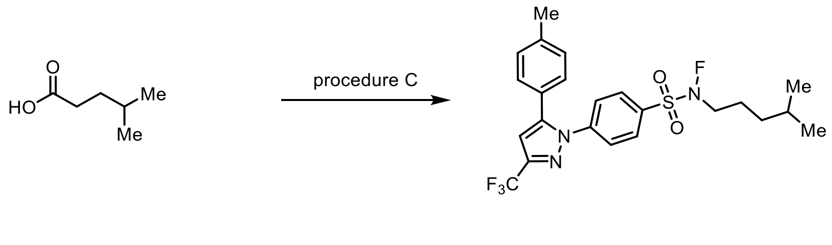

2ad

Substrate **2ad** was prepared in 22% overall yield as yellow oil following procedure C from commercially available 4-methylpentanoic acid. **^{1}H NMR** (600 MHz, $CDCl_3$) δ 7.92 (d, J = 8.4 Hz, 2H), 7.58 (d, J = 8.4 Hz, 2H), 7.20 (d, J = 7.8 Hz, 2H), 7.12 (d, J = 7.8 Hz, 2H), 6.76 (s, 1H), 3.19 (dt, J = 40.2, 6.6 Hz, 2H), 2.39 (s, 3H), 1.75 - 1.69 (m, 2H), 1.58 - 1.53 (m, 1H), 1.28 - 1.25 (m, 2H), 0.89 (d, J = 6.6 Hz, 6H). **^{13}C NMR** (150 MHz, $CDCl_3$) δ 145.6, 144.6 (q, J = 38.3 Hz), 144.4, 140.2, 131.4, 131.1, 130.0, 128.9, 125.7, 125.5, 121.1 (q, J = 267.2 Hz), 106.8, 54.1 (d, J = 12.2

Hz), 35.8, 27.7, 24.3, 22.5, 21.5. **^{19}F NMR** (470 MHz, $CDCl_3$) δ -49.50 (t, J = 40.9 Hz, 1F), -62.56 (s, 3F). **IR** ν_{max} (film): 2957, 2871, 1595, 1498, 1471, 1410, 1309, 1271, 1237, 1164, 1137, 1096, 974, 843, 808, 766, 745, 718, 683, 623, 614, 600, 576 cm^{-1}. **HRMS** (ESI) m/z calcd for $C_{23}H_{25}F_4N_3NaO_2S$ [M+Na]$^+$: 506.1496; found: 506.1487.

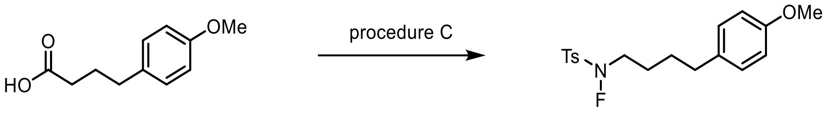

2an

Substrate **2an** was prepared in 40% overall yield as yellow oil following procedure C from commercially available 4-(4-methoxyphenyl)butanoic acid. **^{1}H NMR** (600 MHz, $CDCl_3$) δ 7.81 (d, J = 7.8 Hz, 2H), 7.39 (d, J = 7.8 Hz, 2H), 7.06 (d, J = 8.4 Hz, 2H), 6.81 (d, J = 8.4 Hz, 2H), 3.78 (s, 3H), 3.21 (dt, J = 40.8, 6.0 Hz, 2H), 2.57 (t, J = 7.2 Hz, 2H), 2.48 (s, 3H), 1.74 - 1.68 (m, 4H). **^{13}C NMR** (150 MHz, $CDCl_3$) δ 158.0, 146.3, 133.9, 130.10, 130.06, 129.4, 129.1, 113.9, 55.4, 53.7 (d, J = 12.3 Hz), 34.5, 28.7, 26.0, 21.9. **^{19}F NMR** (470 MHz, $CDCl_3$) δ -49.75 (t, J = 40.0 Hz). **IR** ν_{max} (film): 3437, 2920, 1769, 1758, 1632, 1595, 1511, 1462, 1373, 1299, 1245, 1187, 1173, 1089, 1056, 813, 720, 693, 576, 547 cm^{-1}. **HRMS** (ESI) m/z calcd for $C_{18}H_{22}FNNaO_3S$ [M+Na]$^+$: 374.1197; found: 374.1203.

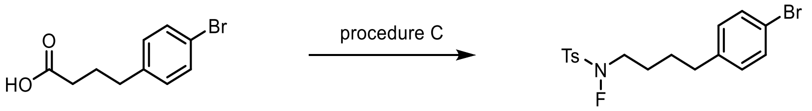

2ao

Substrate **2ao** was prepared in 45% overall yield as yellow oil following procedure C from commercially available 4-(4-bromophenyl)butanoic acid. **^{1}H NMR** (600 MHz, $CDCl_3$) δ 7.80 (d, J = 8.4 Hz, 2H), 7.40 - 7.37 (m, 4H), 7.02 (d, J = 8.4 Hz, 2H), 3.26 - 3.17 (m, 2H), 2.59 - 2.57 (m, 2H), 2.48 (s, 3H), 1.73-1.71 (m, 4H). **^{13}C NMR** (150 MHz, $CDCl_3$) δ 146.4, 140.8, 131.5, 130.3, 130.1, 129.0, 128.5, 119.8, 53.5 (d, J = 12.2 Hz), 34.8, 28.2, 25.9, 21.9. **^{19}F NMR** (470 MHz, $CDCl_3$) δ -49.71 (t, J = 40.4 Hz). **IR** ν_{max} (film): 3445, 2926, 2862, 1769, 1758, 1595, 1487, 1454, 1372, 1306, 1245, 1188, 1172, 1089, 1070, 1010, 799, 721, 694, 649, 571, 548 cm^{-1}. **HRMS** (ESI) m/z calcd for $C_{17}H_{19}BrFNNaO_2S$ [M+Na]$^+$: 422.0196; found: 422.0209.

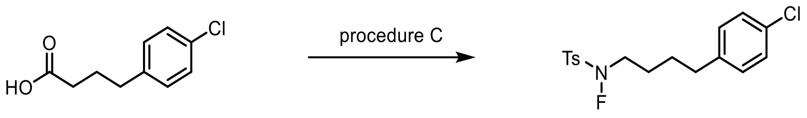

2ap

Substrate **2ap** was prepared in 46% overall yield as yellow oil following procedure C from commercially available 4-(4-chlorophenyl)butanoic acid. **^{1}H NMR** (600 MHz, $CDCl_3$) δ 7.80 (d, J = 8.4 Hz, 2H), 7.39 (d, J = 8.4 Hz, 2H), 7.22 (d, J = 8.4 Hz, 2H), 7.07 (d, J = 8.4 Hz, 2H), 3.26 - 3.17 (m, 2H), 2.61 - 2.59 (m, 2H), 2.47 (s, 3H), 1.73 - 1.71 (m, 4H). **^{13}C NMR** (150 MHz, $CDCl_3$) δ 146.4, 140.3, 131.7, 130.1, 129.8, 129.0, 128.6, 53.5 (d, J = 12.2 Hz), 34.8, 28.3, 25.9, 21.9. **^{19}F NMR** (470 MHz, $CDCl_3$) δ -49.72 (t, J = 40.4 Hz). **IR** ν_{max} (film): 1769, 1758, 1595, 1491, 1373, 1246, 1188, 1172, 1089, 1056, 1014, 813, 726, 695, 669, 649, 572, 550 cm^{-1}. **HRMS** (ESI) m/z calcd for $C_{17}H_{19}ClFNNaO_2S$ $[M+Na]^+$: 378.0701; found: 378.0706.

Procedure D

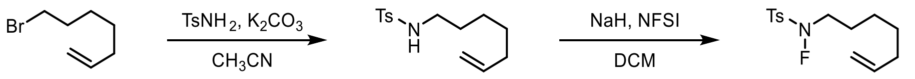

S8 2as

Step 1: To a solution of 7-bromohept-1-ene (1.52 mL, 10.0 mmol, 1.0 equiv) in MeCN (40 mL) were added K_2CO_3 (2.76 g, 20.0 mmol, 2.0 equiv) and $TsNH_2$ (3.42 g, 20.0 mmol, 2.0 equiv), and the reaction was heated to reflux. After 5 h, the reaction mixture was cooled to room temperature, and filtrated through a pad of celite. The filtrate was concentrated *in vacuo* and the residue was purified by a flash column chromatography (PE/EA = 10:1) to afford known compound **S8**[17] as a colourless oil (2.02 g, 7.6 mmol, 76%).

Step 2: A flame dried Schlenk equipped with a stirrer bar was charged with NaH (0.91 g, 22.8 mmol, 3.0 equiv, 60% in oil) and anhydrous DCM (10 mL). A solution of **S8** (2.02 g, 7.6 mmol, 1.0 equiv) in anhydrous DCM (10 mL) was slowly added at 0 °C. The mixture was allowed to stir for 30 min at room temperature under nitrogen atmosphere. Then NFSI (7.19 g, 22.8 mmol, 3.0 equiv) in anhydrous DCM (20 mL) was added dropwise to the mixture and the resulting slurry was stirred for another 12 h. The reaction was quenched by saturated $NaHCO_3$ solution (20 mL) at 0 °C, and then extracted with DCM (20 mL × 3). The combined organic layers were washed sequentially with hydrochloric acid (1.0 M) and brine, dried over anhydrous Na_2SO_4, and filtered. The solvent was removed under reduced pressure, and the residue was purified by a flash column chromatography

(PE/EA = 15:1) to afford the desired product **2as** as yellow oil (1.43 g, 5.07 mmol, 67%). **^{1}H NMR** (600 MHz, $CDCl_3$) δ 7.82 (d, *J* = 8.4 Hz, 2H), 7.41 (d, *J* = 8.4 Hz, 2H), 5.81 - 5.75 (m, 1H), 4.99 (d, *J* = 17.4 Hz, 1H), 4.94 (d, *J* = 10.2 Hz, 1H), 3.20 (dt, *J* = 40.8, 7.2 Hz, 2H), 2.48 (s, 3H), 2.06 - 2.03 (m, 2H), 1.74 - 1.69 (m, 2H), 1.43 - 1.39 (m, 4H). **^{13}C NMR** (150 MHz, $CDCl_3$) δ 146.3, 138.7, 130.1, 130.0, 129.0, 114.7, 53.8 (d, *J* = 12.2 Hz), 33.6, 28.5, 26.3, 26.1, 21.9. **^{19}F NMR** (470 MHz, $CDCl_3$) δ -49.94 (t, *J* = 40.0 Hz). **IR** ν_{max} (film): 2926, 2857, 1770, 1640, 1596, 1458, 1375, 1307, 1246, 1188, 1174, 1090, 994, 911, 814, 726, 696, 650, 574, 550 cm^{-1}. **HRMS** (ESI) m/z calcd for $C_{14}H_{20}FNNaO_2S$ [M+Na]$^+$: 308.1091; found: 308.1089

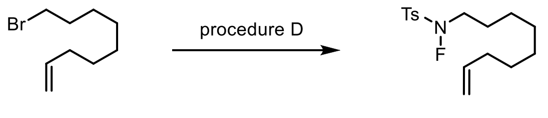

13

Substrate **13** was prepared in 55% overall yield as yellow oil following procedure D from commercially available 9-bromonon-1-ene. **^{1}H NMR** (600 MHz, $CDCl_3$) δ 7.82 (d, *J* = 8.4 Hz, 2H), 7.40 (d, *J* = 8.4 Hz, 2H), 5.83 - 5.76 (m, 1H), 5.00 - 4.97 (m, 1H), 4.94 - 4.92 (m, 1H), 3.20 (dt, *J* = 40.8, 7.2 Hz, 2H), 2.48 (s, 3H), 2.05 - 2.01 (m, 2H), 1.73 - 1.68 (m, 2H), 1.40 - 1.36 (m, 4H), 1.32 - 1.28 (m, 4H). **^{13}C NMR** (150 MHz, $CDCl_3$) δ 146.3, 139.2, 130.11, 130.05, 129.2, 114.4, 53.8 (d, *J* = 12.5 Hz), 33.9, 29.1, 29.0, 28.9, 26.7, 26.4, 21.9. **^{19}F NMR** (470 MHz, $CDCl_3$) δ -49.93 (t, *J* = 40.4 Hz). **IR** ν_{max} (film): 2928, 2856, 1770, 1639, 1596, 1457, 1376, 1307, 1246, 1175, 1089, 1045, 994, 910, 814, 726, 705, 696, 649, 575, 550 cm^{-1}. **HRMS** (ESI) m/z calcd for $C_{16}H_{24}FNNaO_2S$ [M+Na]$^+$: 336.1404; found: 336.1411

Synthesis of compound 2ac

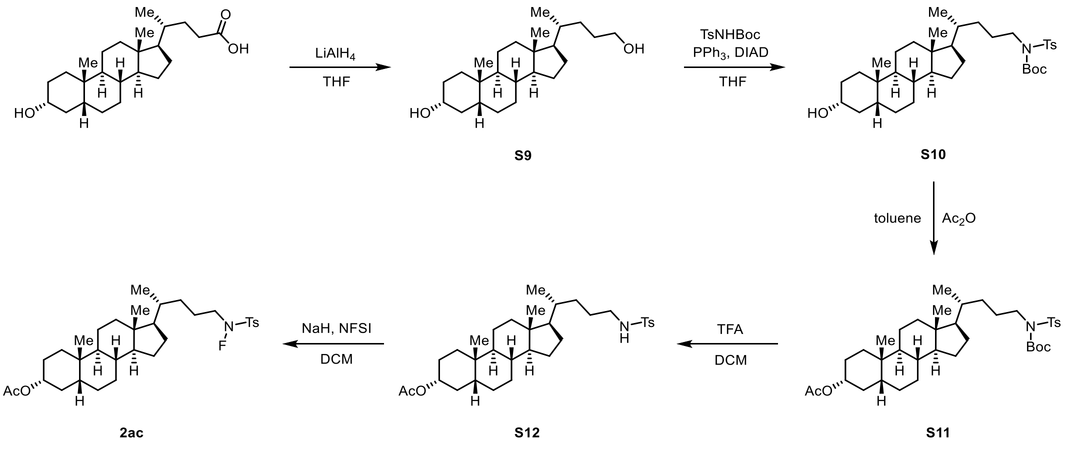

Step 1: Lithocholic acid (11.3 g, 30.0 mmol, 1.0 equiv) was dissolved in anhydrous THF (150 mL), and $LiAlH_4$ (2.85 g, 75.0 mmol, 2.5 equiv) was added portion wise under 0 °C. The mixture

was stirred at 90 °C for 12 h. After that, the reaction was cooled down, and $Na_2SO_4·10H_2O$ was slowly added to quench the reaction until the mixture turning to be clear. The reaction solution was filtered through a pad of celite and washed with Et_2O. The combined organic phases were concentrated *in vacuo* to give known compound **S9**[8] as white gum (9.06 g, 25.0 mmol, 83%).

Step 2: In an oven dried round bottom flask, PPh_3 (7.87 g, 30.0 mmol, 1.2 equiv) and TsNHBoc (8.14 g, 30.0 mmol, 1.2 equiv) were dissolved in anhydrous THF (60 mL). Then a solution of alcohol **S9** (9.06 g, 25.0 mmol, 1.0 equiv) in THF (25 mL) was added. The mixture was cooled to 0 ºC and DIAD (5.91 mL, 30.0 mmol, 1.2 equiv) was slowly added. The reaction was slowly warmed up to room temperature and stirred overnight. Upon completion, the reaction was quenched by water (30 mL). The aqueous layer was extracted with EtOAc (30 mL × 3). The combined organic layers were dried over anhydrous Na_2SO_4, filtered and the solvent was removed under reduced pressure. The residue was purified by a flash column chromatography (PE/EA/DCM = 10:1:0.5) to afford known compound **S10**[8] as white gum (9.23 g, 15.0 mmol, 60%).

Step 3: To a solution of **S10** (9.23 g, 15.0 mmol, 1.0 equiv) in anhydrous toluene (40 mL) was added Ac_2O (4.23 mL, 45.0 mmol, 3.0 equiv) at room temperature. The reaction mixture was stirred at 80 °C for 12 h. Upon completion, the reaction was cooled to room temperature, and quenched with water (10 mL) followed by the addition of saturated $NaHCO_3$ solution (~50 mL). The aqueous layer was extracted with EtOAc (50 mL × 3). The combined organic layers were dried over anhydrous Na_2SO_4, filtered and the solvent was removed under reduced pressure. Further purification by a flash column chromatography (PE/EA = 10:1) afforded the desired product **S11** as yellow oil (8.41 g, 12.8 mmol, 85%). **^{1}H NMR** (500 MHz, $CDCl_3$) δ 7.78 (d, J = 8.5 Hz, 2H), 7.30 (d, J = 8.5 Hz, 2H), 4.75 - 4.69 (m, 1H), 3.83 - 3.72 (m, 2H), 2.44 (s, 3H), 2.03 (s, 3H), 1.99 - 1.96 (m, 1H), 1.88 - 1.79 (m, 5H), 1.70 - 1.61 (m, 2H), 1.58 - 1.52 (m, 2H), 1.46 - 1.38 (m, 8H), 1.34 (s, 9H), 1.25 - 1.21 (m, 3H), 1.16 - 1.01 (m, 7H), 0.95 - 0.93 (m, 6H), 0.65 (s, 3H). **^{13}C NMR** (150 MHz, $CDCl_3$) δ 170.6, 151.0, 144.0, 137.7, 129.2, 127.8, 83.9, 74.4, 56.5, 56.3, 47.7, 42.7, 41.9, 40.5, 40.2, 35.8, 35.5, 35.1, 34.6, 32.8, 32.3, 28.3, 27.9, 27.1, 26.8, 26.7, 26.4, 24.2, 23.4, 21.6, 21.5, 20.9, 18.7, 12.1. **IR** ν_{max} (film): 2937, 2867, 1731, 1598, 1495, 1455, 1361, 1286, 1243, 1157, 1088, 1060, 1027, 979, 913, 887, 813, 732, 674, 648, 603, 581, 546 cm^{-1}. **HRMS** (ESI) m/z calcd for $C_{38}H_{59}NNaO_6S$ $[M+Na]^+$: 680.3955; found: 680.3963.

Step 4: The compound **S11** (8.41 g, 12.8 mmol, 1.0 equiv) was dissolved in DCM (20 mL). TFA (3.0 mL) was added at room temperature. The solution was stirred for 3 h until the complete consumption of compound **S11** (monitored by TLC). Then the reaction was quenched by saturated $NaHCO_3$ solution (~40 mL). The aqueous layer was extracted with DCM (40 mL × 3). The

combined organic fractions were dried over $MgSO_4$, filtered, and concentrated *in vacuo*. Further purification by a flash column chromatography (PE/EA/DCM = 5:1:0.5) afforded **S12** as yellow oil (5.79 g, 10.4 mmol, 81%). **^{1}H NMR** (600 MHz, $CDCl_3$) δ 7.76 (d, J = 7.8 Hz, 2H), 7.31 (d, J = 7.8 Hz, 2H), 4.89 (t, J = 6.0 Hz, 1H), 4.70 - 4.68 (m, 1H), 2.93 - 2.85 (m, 2H), 2.43 (s, 3H), 2.03 (s, 3H), 1.94 - 1.92 (m, 1H), 1.86 - 1.79 (m, 3H), 1.73 - 1.66 (m, 2H), 1.55 - 1.28 (m, 13H), 1.26 - 1.20 (m, 2H), 1.13 - 0.99 (m, 7H), 0.92 (s, 3H), 0.82 (d, J = 6.0 Hz, 3H), 0.59 (s, 3H). **^{13}C NMR** (150 MHz, $CDCl_3$) δ 170.7, 143.3, 137.2, 129.7, 127.2, 74.5, 56.5, 56.1, 43.8, 42.7, 42.0, 40.5, 40.2, 35.8, 35.4, 35.1, 34.6, 32.8, 32.3, 28.3, 27.1, 26.7, 26.4, 26.3, 24.2, 23.4, 21.60, 21.55, 20.9, 18.6, 12.1. **IR** ν_{max} (film): 3283, 2938, 2866, 1735, 1598, 1495, 1448, 1379, 1362, 1328, 1244, 1161, 1094, 1027, 980, 948, 912, 814, 733, 707, 662, 614, 551 cm^{-1}. **HRMS** (ESI) m/z calcd for $C_{33}H_{51}NNaO_4S$ $[M+Na]^+$: 580.3431; found: 580.3434.

Step 5: A flame dried Schlenk equipped with a stirrer bar was charged with NaH (1.20 g, 30.0 mmol, 3.0 equiv, 60% in oil) and anhydrous DCM (10 mL). A solution of **S12** (5.57 g, 10.0 mmol, 1.0 equiv) in DCM (10 mL) was slowly added at 0 °C. The mixture was allowed to stir for 30 min at room temperature under nitrogen atmosphere. Then NFSI (9.46 g, 30.0 mmol, 3.0 equiv) in anhydrous DCM (20 mL) was added dropwise to the mixture and the resulting slurry was stirred for another 12 h. The reaction was quenched by saturated $NaHCO_3$ solution (20 mL) at 0 °C, and then extracted with DCM (20 mL × 3). The combined organic layers were washed sequentially with hydrochloric acid (1.0 M) and brine, dried over anhydrous Na_2SO_4, filtered and concentrated *in vacuo*. The residue was purified by a flash column chromatography (PE/EA = 15:1) to afford the desired product **2ac** as yellow oil (3.67 g, 6.38 mmol, 64%). **^{1}H NMR** (600 MHz, $CDCl_3$) δ 7.82 (d, J = 8.4 Hz, 2H), 7.30 (d, J = 8.4 Hz, 2H), 4.74 - 4.69 (m, 1H), 3.17 (dt, J = 40.8, 7.2 Hz, 2H), 2.48 (s, 3H), 2.03 (s, 3H), 1.97 - 1.94 (m, 1H), 1.85 - 1.78 (m, 4H), 1.69 - 1.66 (m, 1H), 1.61 - 1.52 (m, 3H), 1.47 - 1.36 (m, 8H), 1.26 - 0.99 (m, 11H), 0.92 (s, 3H), 0.90 (d, J = 6.6 Hz, 3H), 0.63 (s, 3H). **^{13}C NMR** (150 MHz, $CDCl_3$) δ 170.7, 146.2, 130.04, 130.01, 129.1, 74.5, 56.6, 56.1, 54.3 (d, J = 12.3 Hz), 42.8, 42.0, 40.5, 40.2, 35.9, 35.5, 35.1, 34.7, 32.9, 32.4, 28.3, 27.1, 26.7, 26.4, 24.3, 23.4, 23.1, 21.9, 21.6, 20.9, 18.6, 12.1. **^{19}F NMR** (565 MHz, $CDCl_3$) δ -49.92 (t, J = 40.7 Hz). **IR** ν_{max} (film): 2936, 2866, 1735, 1596, 1448, 1378, 1362, 1243, 1188, 1175, 1120, 1090, 1027, 981, 949, 912, 814, 730, 706, 697, 649, 572, 548 cm^{-1}. **HRMS** (ESI) m/z calcd for $C_{33}H_{50}FNNaO_4S$ $[M+Na]^+$: 598.3337; found: 598.3346.

Part 3.2: Procedure and characteristic data for products **3a-3az**, **4a-4az**, **5a-5k**, **6a-6aa**, **7a**, **7b**, **8**.

The general procedure A (for products 3a-3ad):

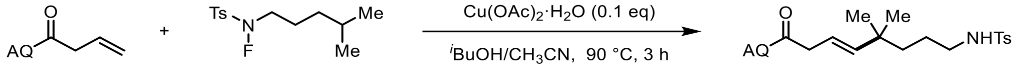

To a dry Schlenk flask were added **1a** (42.4 mg, 0.20 mmol, 1.0 equiv), **2a** (137 mg, 0.50 mmol, 2.5 equiv), $Cu(OAc)_2{\cdot}H_2O$ (4.0 mg, 0.02 mmol, 0.10 equiv), anhydrous ^{i}BuOH (2.5 mL), and anhydrous CH_3CN (0.50 mL). The mixture was degassed for three times with argon and stirred at 90 °C (oil bath) for 3 h. Once completion, the reaction was cooled to room temperature. The reaction mixture was filtered by celite, and the filtrate was concentrated *in vacuo*. Further purification by a flash column chromatography using eluents (PE/EA = 5:1) afforded the desired product **3a** as yellow oil (76.3 mg, 0.16 mmol, 82%, *r.r.* > 20:1, *E/Z* > 20:1). **^{1}H NMR** (600 MHz, $CDCl_3$) δ 10.03 (s, 1H), 8.77 - 8.75 (m, 2H), 8.16 (dd, *J* = 8.4, 1.8 Hz, 1H), 7.70 (d, *J* = 7.8 Hz, 2H), 7.55 - 7.50 (m, 2H), 7.45 (dd, *J* = 8.4, 4.2 Hz, 1H), 7.27 (d, *J* = 7.8 Hz, 2H), 5.68 (d, *J* = 15.6 Hz, 1H), 5.60 (dt, *J* = 15.6, 7.2 Hz, 1H), 4.44 (t, *J* = 6.6 Hz, 1H), 3.25 (d, *J* = 7.2 Hz, 2H), 2.91 (dt, *J* = 6.6, 6.6 Hz, 2H), 2.40 (s, 3H), 1.49 - 1.44 (m, 2H), 1.35 - 1.32 (m, 2H), 1.06 (s, 6H). **^{13}C NMR** (150 MHz, $CDCl_3$) δ 170.2, 148.3, 146.4, 143.4, 138.7, 137.2, 136.5, 134.5, 129.8, 128.1, 127.5, 127.2, 121.8, 121.7, 119.2, 116.5, 44.0, 42.3, 39.6, 36.2, 27.2, 25.1, 21.6. **IR** ν_{max} (film): 3313, 3047, 2958, 2869, 1669, 1619, 1597, 1577, 1486, 1425, 1326, 1160, 1068, 1018, 910, 792, 733, 613, 560 cm^{-1}. **HRMS** (ESI) m/z calcd for $C_{26}H_{31}N_3NaO_3S$ $[M+Na]^+$: 488.1978; found: 488.1971.

Lagre-scale synthesis of 3a:

To a dry Schlenk flask were added **1a** (1.06 g, 5.0 mmol, 1.0 equiv), **2a** (3.41 g, 12.5 mmol, 2.5 equiv), $Cu(OAc)_2{\cdot}H_2O$ (99.8 mg, 0.50 mmol, 0.10 equiv), anhydrous ^{i}BuOH (62.5 mL), and anhydrous CH_3CN (12.5 mL). The mixture was degassed for three times with argon and stirred at 90 °C (oil bath) for 3 h. Once completion, the reaction was cooled to room temperature. The reaction mixture was filtered by celite, and the filtrate was concentrated *in vacuo*. Further purification by a flash column chromatography using eluents (PE/EA = 5:1) afforded the desired product **3a** as yellow oil (1.74 g, 3.74 mmol, 75%, *r.r.* > 20:1, *E/Z* > 20:1).

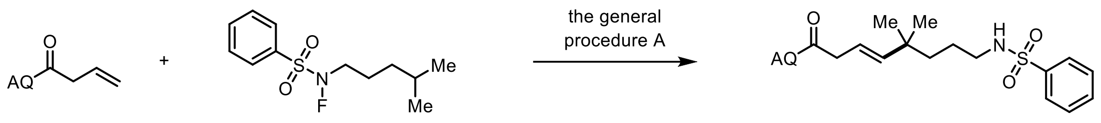

1a **2b** **3b**

Product **3b** was prepared by the general procedure A. Purification using column chromatography (PE/EA = 5:1) afforded **3b** as yellow oil (65.8 mg, 0.15 mmol, 73%, *r.r.* > 20:1, *E/Z* > 20:1). **^{1}H NMR** (600 MHz, $CDCl_3$) δ 10.04 (s, 1H), 8.76 - 8.74 (m, 2H), 8.14 (dd, *J* = 8.4, 1.8 Hz, 1H), 7.84 (d, *J* = 7.2 Hz, 2H), 7.55 - 7.50 (m, 3H), 7.48 - 7.43 (m, 3H), 5.66 (d, *J* = 15.6 Hz, 1H), 5.57 (dt, *J* = 15.6, 7.2 Hz, 1H), 4.94 (t, *J* = 6.6 Hz, 1H), 3.23 (d, *J* = 7.2 Hz, 2H), 2.93 (dt, *J* = 6.6, 6.6 Hz, 2H), 1.49 - 1.43 (m, 2H), 1.34 - 1.31 (m, 2H), 1.05 (s, 6H). **^{13}C NMR** (150 MHz, $CDCl_3$) δ 170.2, 148.2, 146.4, 140.2, 138.6, 136.4, 134.4, 132.6, 129.1, 128.0, 127.4, 127.1, 121.8, 121.7, 119.0, 116.4, 44.0, 42.2, 39.6, 36.1, 27.1, 25.0. **IR** ν_{max} (film): 3314, 2957, 1528, 1486, 1424, 1386, 1326, 1160, 1094, 979, 826, 755, 586 cm^{-1}. **HRMS** (ESI) m/z calcd for $C_{25}H_{29}N_3NaO_3S$ $[M+Na]^+$: 474.1822; found: 474.1831.

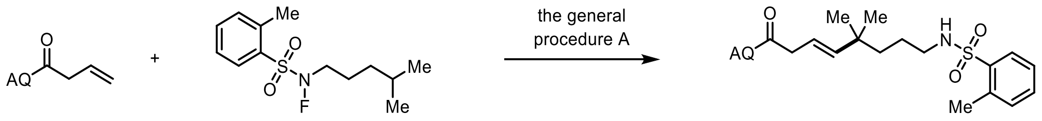

1a **2c** **3c**

Product **3c** was prepared by the general procedure A. Purification using column chromatography (PE/EA = 5:1) afforded **3c** as yellow oil (69.8 mg, 0.15 mmol, 75%, *r.r.* > 20:1, *E/Z* > 20:1). **^{1}H NMR** (600 MHz, $CDCl_3$) δ 10.03 (s, 1H), 8.77 - 8.75 (m, 2H), 8.16 - 8.14 (m, 1H), 7.94 - 7.92 (m, 1H), 7.55 - 7.49 (m, 2H), 7.46 - 7.41 (m, 2H), 7.27 (d, *J* = 8.4 Hz, 2H), 5.66 (d, *J* = 15.6 Hz, 1H), 5.61 - 5.56 (m, 1H), 4.63 - 4.58 (m, 1H), 3.24 (d, *J* = 6.6 Hz, 2H), 2.92 (dt, *J* = 6.6, 6.6 Hz, 2H), 2.59 (s, 3H), 1.46 - 1.42 (m, 2H), 1.31 - 1.29 (m, 2H), 1.05 (s, 6H). **^{13}C NMR** (150 MHz, $CDCl_3$) δ 170.1, 148.2, 146.3, 138.6, 138.2, 137.0, 136.5, 134.5, 132.8, 132.6, 129.6, 128.1, 127.5, 126.3, 121.8, 121.7, 119.1, 116.5, 43.9, 42.3, 39.6, 36.2, 27.2, 25.2, 20.4. **IR** ν_{max} (film): 2920, 2850, 2360, 2341, 1771, 1522, 1488, 1472, 1424, 1419, 669, 649, 419 cm^{-1}. **HRMS** (ESI) m/z calcd for $C_{26}H_{31}N_3NaO_3S$ $[M+Na]^+$: 488.1978; found: 488.1974.

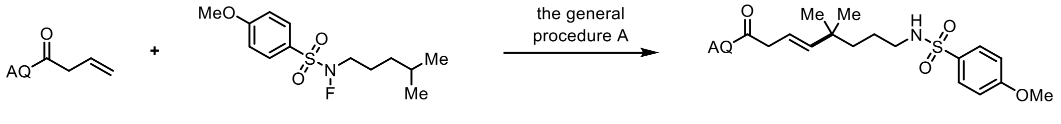

1a **2d** **3d**

Product **3d** was prepared by the general procedure A. Purification using column chromatography (PE/EA = 3:1) afforded **3d** as yellow oil (70.3 mg, 0.15 mmol, 73%, *r.r.* > 20:1, *E/Z* > 20:1). **^{1}H NMR** (600 MHz, $CDCl_3$) δ 10.05 (s, 1H), 8.76 - 8.74 (m, 2H), 8.14 (dd, *J* = 8.4, 1.8 Hz, 1H), 7.76 (d, *J* = 8.4 Hz, 2H), 7.53 - 7.48 (m, 2H), 7.44 (dd, *J* = 8.4, 4.2 Hz, 1H), 6.93 (d, *J* = 8.4 Hz, 2H), 5.67 (d, *J* = 15.6 Hz, 1H), 5.58 (dt, *J* = 15.6, 7.2 Hz, 1H), 4.81 (t, *J* = 6.6 Hz, 1H), 3.83 (s, 3H), 3.24 (d, *J* = 7.2 Hz, 2H), 2.89 (dt, *J* = 6.6, 6.6 Hz, 2H), 1.49 - 1.44 (m, 2H), 1.35 - 1.32 (m, 2H), 1.06 (s, 6H). **^{13}C NMR** (150 MHz, $CDCl_3$) δ 170.2, 162.8, 148.2, 146.5, 138.6, 136.4, 134.4, 131.7, 129.3, 128.0, 127.4, 121.8, 121.7, 119.0, 116.3, 114.2, 55.7, 43.9, 42.2, 39.6, 36.2, 27.1, 24.9. **IR** ν_{max} (film): 3735, 3629, 3315, 2958, 1682, 1596, 1578, 1486, 1424, 1386, 1326, 1258, 1155, 1096, 1026, 979, 913, 827, 793, 742, 617, 560, 418 cm^{-1}. **HRMS** (ESI) m/z calcd for $C_{26}H_{31}N_3NaO_4S$ $[M+Na]^+$: 504.1927; found: 504.1922.

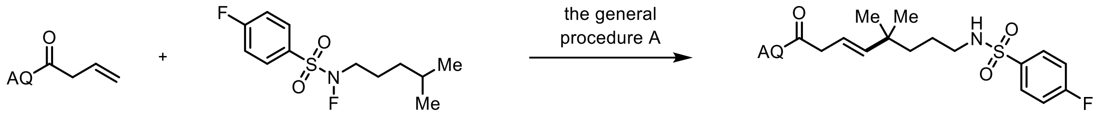

1a **2e** **3e**

Product **3e** was prepared by the general procedure A. Purification using column chromatography (PE/EA = 5:1) afforded **3e** as yellow oil (79.8 mg, 0.17 mmol, 85%, *r.r.* > 20:1, *E/Z* > 20:1). **^{1}H NMR** (600 MHz, $CDCl_3$) δ 10.02 (s, 1H), 8.76 - 8.75 (m, 2H), 8.16 (dd, *J* = 7.8, 1.2 Hz, 1H), 7.85 - 7.82 (m, 2H), 7.55 - 7.49 (m, 2H), 7.45 (dd, *J* = 7.8, 4.2 Hz, 1H), 7.17 - 7.13 (m, 2H), 5.68 (d, *J* = 16.2 Hz, 1H), 5.60 (dt, *J* = 16.2, 7.2 Hz, 1H), 4.65 (t, *J* = 6.6 Hz, 1H), 3.26 (d, *J* = 7.2 Hz, 2H), 2.92 (dt, *J* = 6.6, 6.6 Hz, 2H), 1.51 - 1.46 (m, 2H), 1.36 - 1.33 (m, 2H), 1.06 (s, 6H). **^{13}C NMR** (150 MHz, $CDCl_3$) δ 170.2, 165.1 (d, *J* = 254.3 Hz), 148.3, 146.2, 138.6, 136.5, 136.3 (d, *J* = 3.0 Hz), 134.5, 129.9 (d, *J* = 10.5 Hz), 128.1, 127.5, 121.80, 121.76, 119.2, 116.5, 116.4 (d, *J* = 22.5 Hz), 44.0, 42.2, 39.6, 36.2, 27.2, 25.0. **^{19}F NMR** (470 MHz, $CDCl_3$) δ -105.5 - (-105.6) (m). **IR** ν_{max} (film): 3309, 2994, 1770, 1758, 1527, 1374, 1325, 1241, 1155, 1093, 1058, 913, 826, 747, 549, 418 cm^{-1}. **HRMS** (ESI) m/z calcd for $C_{25}H_{28}FN_3NaO_3S$ $[M+Na]^+$: 492.1728; found: 492.1737.

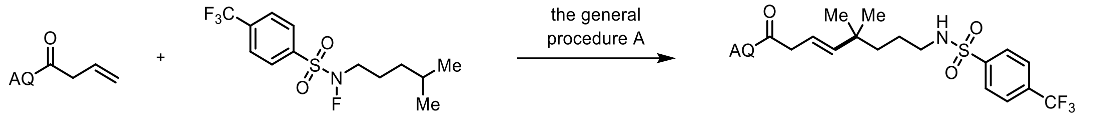

1a **2f** **3f**

Product **3f** was prepared by the general procedure A. Purification using column chromatography (PE/EA = 3:1) afforded **3f** as yellow oil (72.7 mg, 0.14 mmol, 70%, *r.r.* > 20:1, *E/Z* > 20:1). **^{1}H NMR** (600 MHz, $CDCl_3$) δ 10.04 (s, 1H), 8.76 - 8.72 (m, 2H), 8.15 (dd, J = 8.4, 1.8 Hz, 1H), 7.96 (d, J = 7.8 Hz, 2H), 7.73 (d, J = 7.8 Hz, 2H), 7.53 - 7.49 (m, 2H), 7.44 (dd, J = 8.4, 4.2 Hz, 1H), 5.67 (d, J = 15.6 Hz, 1H), 5.58 (dt, J = 15.6, 6.6 Hz, 1H), 5.19 (t, J = 6.6 Hz, 1H), 3.25 (d, J = 6.6 Hz, 2H), 2.95 (dt, J = 6.6, 6.6 Hz, 2H), 1.52 - 1.47 (m, 2H), 1.36 - 1.33 (m, 2H), 1.05 (s, 6H). **^{13}C NMR** (150 MHz, $CDCl_3$) δ 170.3, 148.3, 146.3, 143.9, 138.6, 136.5, 134.4, 134.3 (q, J = 33.3 Hz), 128.1, 127.7, 127.4, 126.3 (q, J = 3.0 Hz), 123.4 (q, J = 270.0 Hz), 121.82, 121.79, 119.1, 116.4, 44.1, 42.2, 39.4, 36.2, 27.1, 25.0. **^{19}F NMR** (470 MHz, $CDCl_3$) δ -63.1 (s). **IR** ν_{max} (film): 3315, 2959, 1670, 1596, 1529, 1486, 1425, 1404, 1323, 1261, 1166, 1133, 1062, 1016, 979, 843, 826, 792, 710, 606,560, 428 cm^{-1}. **HRMS** (ESI) m/z calcd for $C_{26}H_{28}F_3N_3NaO_3S$ $[M+Na]^+$: 542.1696; found: 542.1702.

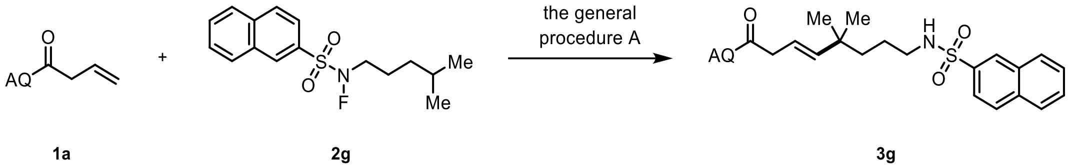

Product **3g** was prepared by the general procedure A. Purification using column chromatography (PE/EA = 4:1) afforded **3g** as yellow oil (80.2 mg, 0.16 mmol, 80%, *r.r.* > 20:1, *E/Z* > 20:1). **^{1}H NMR** (600 MHz, $CDCl_3$) δ 9.99 (s, 1H), 8.76 - 8.73 (m, 2H), 8.41 (s, 1H), 8.13 (d, J = 8.4 Hz, 1H), 7.93 - 7.88 (m, 3H), 7.80 (d, J = 9.0 Hz, 1H), 7.62 (t, J = 7.8 Hz, 1H), 7.58 (t, J = 7.8 Hz, 1H), 7.53 - 7.48 (m, 2H), 7.41 (dd, J = 8.4, 4.2 Hz, 1H) 5.60 (d, J = 15.6 Hz, 1H), 5.58 (dt, J = 15.6, 7.2 Hz, 1H), 4.77 (t, J = 6.6 Hz, 1H), 3.17 (d, J = 7.2 Hz, 2H), 2.97 (dt, J = 6.6, 6.6 Hz, 2H), 1.49 - 1.44 (m, 2H), 1.33 - 1.30 (m, 2H), 1.02 (s, 6H). **^{13}C NMR** (150 MHz, $CDCl_3$) δ 170.2, 148.2, 146.3, 138.6, 137.0, 136.4, 134.9, 134.5, 132.3, 129.5, 129.3, 128.9, 128.5, 128.1, 128.0, 127.7, 127.5, 122.5, 121.8, 121.7, 119.1, 116.5, 44.1, 42.2, 39.6, 36.2, 27.1, 25.0. **IR** ν_{max} (film): 3314, 2958, 1669, 1527, 1486, 1424, 1386, 1242, 1158, 1131, 1075, 979, 912, 743, 656, 617, 549, 477 cm^{-1}. **HRMS** (ESI) m/z calcd for $C_{29}H_{31}N_3NaO_3S$ $[M+Na]^+$: 524.1978; found: 524.1972.

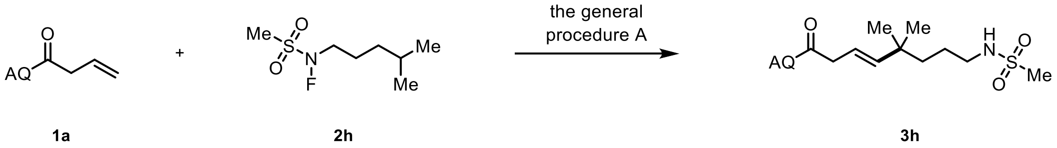

Product **3h** was prepared by the general procedure A. Purification using column chromatography (PE/EA = 5:1) afforded **3h** as yellow oil (58.4 mg, 0.15 mmol, 75%, *r.r.* > 20:1,

E/Z > 20:1). **^{1}H NMR** (600 MHz, $CDCl_3$) δ 10.06 (s, 1H), 8.78 - 8.74 (m, 2H), 8.15 (dd, *J* = 8.4, 1.8 Hz, 1H), 7.54 - 7.49 (m, 2H), 7.45 (dd, *J* = 8.4, 4.2 Hz, 1H), 5.74 (d, *J* = 15.6 Hz, 1H), 5.58 (dt, *J* = 15.6, 7.2 Hz, 1H), 4.64 (t, *J* = 6.0 Hz, 1H), 3.28 (d, *J* = 7.2 Hz, 2H), 3.11 - 3.07 (m, 2H), 2.90 (s, 3H), 1.61 - 1.55 (m, 2H), 1.44 - 1.41 (m, 2H), 1.12 (s, 6H). **^{13}C NMR** (150 MHz, $CDCl_3$) δ 170.2, 148.3, 146.3, 138.6, 136.4, 134.4, 128.0, 127.5, 121.8, 121.7, 119.2, 116.4, 44.0, 43.5, 42.2, 40.2, 39.6, 36.2, 27.2, 25.5. **IR** ν_{max} (film): 3310, 2958, 2851, 2095, 1672, 1528, 1457, 1425, 1386, 1326, 1156, 1092, 979, 827, 793, 750, 524 cm^{-1}. **HRMS** (ESI) m/z calcd for $C_{20}H_{27}N_3NaO_3S$ [M+Na]$^+$: 412.1665; found: 412.1655.

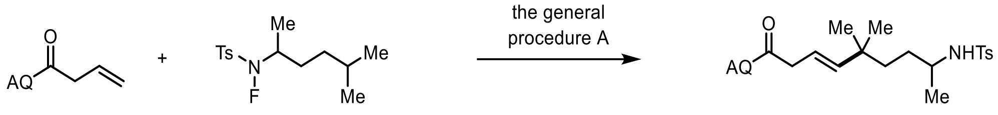

1a **2i** **3i**

Product **3i** was prepared by the general procedure A. Purification using column chromatography (PE/EA = 5:1) afforded **3i** as yellow oil (73.8 mg, 0.15 mmol, 77%, *r.r.* > 20:1, *E/Z* > 20:1). **^{1}H NMR** (600 MHz, $CDCl_3$) δ 10.05 (s, 1H), 8.78 - 8.75 (m, 2H), 8.14 (dd, *J* = 8.4, 1.8 Hz, 1H), 7.73 (d, *J* = 8.4 Hz, 2H), 7.54 - 7.48 (m, 2H), 7.44 (dd, *J* = 7.8, 4.2 Hz, 1H), 7.24 (d, *J* = 8.4 Hz, 2H), 5.64 (d, *J* = 15.6 Hz, 1H), 5.56 (dt, *J* = 15.6, 6.6 Hz, 1H), 4.68 (d, *J* = 8.4 Hz, 1H), 3.28 - 3.23 (m, 3H), 2.39 (s, 3H), 1.35 - 1.29 (m, 3H), 1.21 - 1.16 (m, 1H), 1.02 (s, 6H), 0.96 (d, *J* = 6.6 Hz, 3H). **^{13}C NMR** (150 MHz, $CDCl_3$) δ 170.2, 148.2, 146.5, 143.2, 138.60, 138.58, 136.4, 134.5, 129.6, 128.0, 127.5, 127.1, 121.7, 121.6, 118.9, 116.4, 50.8, 42.3, 38.4, 36.0, 32.6, 27.2, 27.0, 22.0, 21.6. **IR** ν_{max} (film): 3313, 2958, 1526, 1386, 1326, 1275, 1260, 1158, 1093, 980, 826, 792, 749, 580, 550, 418 cm^{-1}. **HRMS** (ESI) m/z calcd for $C_{27}H_{33}N_3NaO_3S$ [M+Na]$^+$: 502.2135; found: 502.2136.

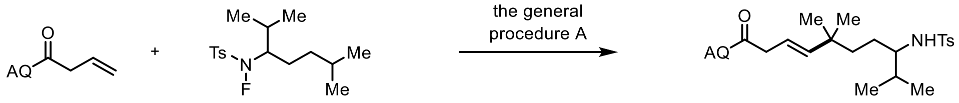

1a **2j** **3j**

Product **3j** was prepared by the general procedure A. Purification using column chromatography (PE/EA = 5:1) afforded **3j** as yellow oil (71.0 mg, 0.14 mmol, 70%, *r.r.* > 20:1, *E/Z* > 20:1). **^{1}H NMR** (600 MHz, $CDCl_3$) δ 10.03 (s, 1H), 8.79 - 8.77 (m, 2H), 8.17 (d, *J* = 8.4 Hz, 1H), 7.71 (d, *J* = 8.4 Hz, 2H), 7.56 - 7.51 (m, 2H), 7.47 (dd, *J* = 7.8, 4.2 Hz, 1H), 7.25 (d, *J* = 8.4 Hz, 2H), 5.57 (d, *J* = 15.6 Hz, 1H), 5.56 (dt, *J* = 15.6, 6.6 Hz, 1H), 4.34 (d, *J* = 9.0 Hz, 1H), 3.23 (d, *J* = 6.6 Hz, 2H), 3.05 - 3.00 (m, 1H), 2.39 (s, 3H), 1.67 - 1.64 (m, 1H), 1.35 - 1.32 (m, 1H), 1.14 - 1.09 (m, 1H), 0.97 (s, 3H), 0.96 (s, 3H), 0.89 - 0.85 (m, 2H), 0.73 (d, *J* = 6.6 Hz, 3H), 0.72

(d, J = 6.6 Hz, 3H). **^{13}C NMR** (150 MHz, $CDCl_3$) δ 170.2, 148.2, 146.3, 143.1, 139.0, 136.6, 134.5, 129.6, 128.1, 127.6, 127.2, 121.8, 121.7, 119.1, 116.5, 60.2, 42.3, 38.7, 36.1, 31.6, 27.4, 27.0, 26.8, 21.6, 18.6, 17.7. **IR** ν_{max} (film): 3733, 3648, 2955, 2925, 2854, 1716, 1652, 1522, 1457, 1325, 1158, 1094, 979, 910, 576, 552 cm^{-1}. **HRMS** (ESI) m/z calcd for $C_{29}H_{37}N_3NaO_3S$ [M+Na]$^+$: 530.2448; found: 530.2427.

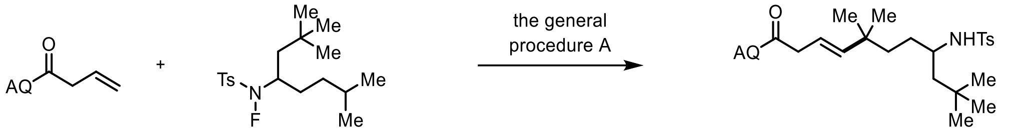

1a **2k** **3k**

Product **3k** was prepared by the general procedure A. Purification using column chromatography (PE/EA = 5:1) afforded **3k** as yellow oil (69.6 mg, 0.13 mmol, 65%, *r.r.* > 20:1, *E/Z* > 20:1). **^{1}H NMR** (600 MHz, $CDCl_3$) δ 10.03 (s, 1H), 8.78 - 8.76 (m, 2H), 8.16 (dd, J = 8.4, 1.8 Hz, 1H), 7.71 (d, J = 8.4 Hz, 2H), 7.55 - 7.49 (m, 2H), 7.45 (dd, J = 8.4, 4.2 Hz, 1H), 7.25 (d, J = 8.4 Hz, 2H), 5.59 - 5.51 (m, 2H), 4.33 (d, J = 8.4 Hz, 1H), 3.34 - 3.30 (m, 1H), 3.22 (d, J = 6.0 Hz, 2H), 2.39 (s, 3H), 1.39 - 1.36 (m, 1H), 1.24 - 1.17 (m, 5H), 0.97 (s, 6H), 0.80 (s, 9H). **^{13}C NMR** (150 MHz, $CDCl_3$) δ 170.1, 148.2, 146.6, 143.2, 139.0, 138.7, 136.5, 134.6, 129.6, 128.1, 127.6, 127.2, 121.74, 121.67, 118.9, 116.4, 52.1, 49.4, 42.3, 37.9, 36.0, 32.2, 30.5, 30.0, 27.1, 27.0, 21.6. **IR** ν_{max} (film): 3566, 3446, 2955, 1771, 1683, 1670, 1653, 1647, 1526, 1507, 1487, 1457, 1385, 1325, 1245, 1156, 1094, 1063, 748, 418 cm^{-1}. **HRMS** (ESI) m/z calcd for $C_{31}H_{41}N_3NaO_3S$ [M+Na]$^+$: 558.2761; found: 558.2764.

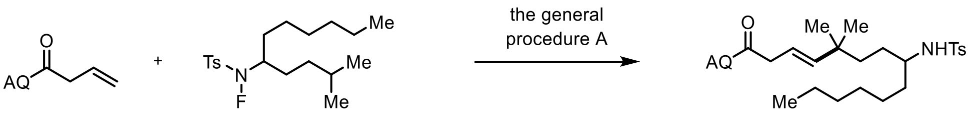

1a **2l** **3l**

Product **3l** was prepared by the general procedure A. Purification using column chromatography (PE/EA = 5:1) afforded **3l** as yellow oil (87.9 mg, 0.16 mmol, 80%, *r.r.* > 20:1, *E/Z* > 20:1). **^{1}H NMR** (600 MHz, $CDCl_3$) δ 10.05 (s, 1H), 8.78 - 8.75 (m, 2H), 8.14 (dd, J = 8.4, 1.8 Hz, 1H), 7.72 (d, J = 8.4 Hz, 2H), 7.54 - 7.48 (m, 2H), 7.44 (dd, J = 8.4, 4.2 Hz, 1H), 7.24 (d, J = 8.4 Hz, 2H), 5.62 (d, J = 15.6 Hz, 1H), 5.55 (dt, J = 15.6, 7.2 Hz, 1H), 4.64 (d, J = 8.4 Hz, 1H), 3.23 (d, J = 7.2 Hz, 2H), 3.18 - 3.13 (m, 1H), 2.38 (s, 3H), 1.42 - 1.36 (m, 1H), 1.32 - 1.13 (m, 9H), 1.06 - 1.02 (m, 4H), 1.00 (s, 6H), 0.82 (t, J = 7.2 Hz, 3H). **^{13}C NMR** (150 MHz, $CDCl_3$) δ 170.2, 148.2, 146.6, 143.1, 138.8, 138.6, 136.4, 134.5, 129.6, 128.0, 127.5, 127.1, 121.7, 121.6, 118.8, 116.4, 54.8,

42.3, 38.2, 36.0, 35.3, 31.7, 30.2, 29.1, 27.2, 26.9, 25.3, 22.6, 21.5, 14.1. **IR** ν_{max} (film): 3313, 2954, 2928, 2857, 1770, 1758, 1683, 1596, 1528, 1486, 1457, 1424, 1385, 1326, 1242, 1158, 1094, 1059, 979, 913, 826, 814, 792, 749, 580, 550, 418 cm^{-1}. **HRMS** (ESI) m/z calcd for $C_{32}H_{43}N_3NaO_3S$ $[M+Na]^+$: 572.2917; found: 572.2919.

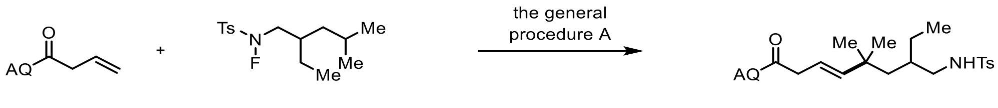

1a 2m 3m

Product **3m** was prepared by the general procedure A. Purification using column chromatography (PE/EA = 5:1) afforded **3m** as yellow oil (81.9 mg, 0.17 mmol, 83%, *r.r.* > 20:1, *E/Z* > 20:1). **^{1}H NMR** (600 MHz, $CDCl_3$) δ 9.95 (s, 1H), 8.78 - 8.76 (m, 2H), 8.17 (dd, J = 8.4, 1.8 Hz, 1H), 7.69 (d, J = 8.4 Hz, 2H), 7.56 - 7.51 (m, 2H), 7.45 (dd, J = 7.8, 4.2 Hz, 1H), 7.21 (d, J = 8.4 Hz, 2H), 5.68 (d, J = 15.6 Hz, 1H), 5.52 - 5.47 (m, 1H), 5.25 (t, J = 6.0 Hz, 1H), 3.28 - 3.20 (m, 2H), 2.38 - 2.74 (m, 2H), 2.38 (s, 3H), 1.43 - 1.40 (m, 1H), 1.39 - 1.33 (m, 2H), 1.29 - 1.24 (m, 2H), 1.06 (s, 3H), 1.05 (s, 3H), 0.79 (t, J = 7.8 Hz, 3H). **^{13}C NMR** (150 MHz, $CDCl_3$) δ 170.5, 148.3, 146.7, 143.0, 138.6, 137.4, 136.5, 134.5, 129.6, 128.1, 127.5, 127.2, 121.83, 121.79, 118.6, 116.7, 47.8, 44.3, 42.0, 36.9, 35.8, 29.1, 26.7, 25.8, 21.6, 11.0. **IR** ν_{max} (film): 3314, 2959, 2926, 2872, 1682, 1597, 1576, 1529, 1486, 1459, 1425, 1386, 1326, 1262, 1160, 1093, 979, 910, 826, 815, 792, 757, 732, 706, 551, 501 cm^{-1}. **HRMS** (ESI) m/z calcd for $C_{28}H_{35}N_3NaO_3S$ $[M+Na]^+$: 516.2291; found: 516.2284.

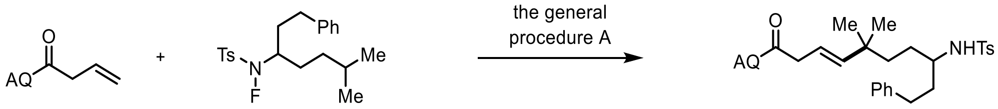

1a 2n 3n

Product **3n** was prepared by the general procedure A. Purification using column chromatography (PE/EA = 5:1) afforded **3n** as yellow oil (99.1 mg, 0.17 mmol, 87%, *r.r.* > 20:1, *E/Z* > 20:1). **^{1}H NMR** (600 MHz, $CDCl_3$) δ 10.03 (s, 1H), 8.77 (dd, J = 7.8, 1.8 Hz, 1H), 8.71 (dd, J = 4.2, 1.2 Hz, 1H), 8.11 (dd, J = 8.4, 1.2 Hz, 1H), 7.71 (d, J = 7.8 Hz, 2H), 7.51 - 7.46 (m, 2H), 7.39 (dd, J = 8.4, 4.2 Hz, 1H), 7.22 (d, J = 7.8 Hz, 2H), 7.19 (t, J = 7.2 Hz, 2H), 7.13 (t, J = 7.2 Hz, 1H), 6.95 (d, J = 7.2 Hz, 2H), 5.59 (d, J = 15.6 Hz, 1H), 5.53 (dt, J = 15.6, 6.6 Hz, 1H), 4.92 - 4.87 (m, 1H), 3.25 - 3.19 (m, 3H), 2.53 - 2.48 (m, 1H), 2.45 - 2.39 (m, 1H), 2.37 (s, 3H), 1.67 - 1.61 (m, 1H), 1.59 - 1.53 (m, 1H), 1.45 -1.39 (m, 1H), 1.32 - 1.26 (m, 1H), 1.25 -1.21 (m, 1H), 1.18 - 1.13 (m, 1H), 0.99 (s, 6H). **^{13}C NMR** (150 MHz, $CDCl_3$) δ 170.1, 148.2, 146.5, 143.2,

141.5, 138.7, 138.6, 136.4, 134.5, 129.6, 128.4, 128.3, 128.0, 127.4, 127.1, 125.9, 121.71, 121.66, 119.0, 116.4, 54.5, 42.2, 38.1, 37.0, 36.0, 31.8, 30.0, 27.2, 26.9, 21.5. **IR** ν_{max} (film): 3313, 3025, 2954, 2865, 1771, 1683, 1654, 1597, 1527, 1486, 1456, 1424, 1386, 1326, 1245, 1157, 1092, 1061, 979, 913, 826, 815, 792, 748, 666, 576, 550, 418 cm^{-1}. **HRMS** (ESI) m/z calcd for $C_{34}H_{39}N_3NaO_3S$ $[M+Na]^+$: 592.2604; found: 592.2586.

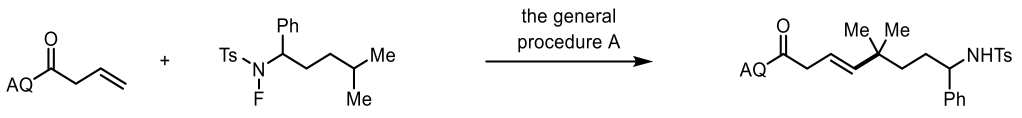

1a **2o** **3o**

Product **3o** was prepared by the general procedure A. Purification using column chromatography (PE/EA = 5:1) afforded **3o** as yellow oil (87.7 mg, 0.16 mmol, 81%, *r.r.* > 20:1, *E/Z* > 20:1). **^{1}H NMR** (600 MHz, $CDCl_3$) δ 10.03 (s, 1H), 8.77 (dd, *J* = 7.2, 1.8 Hz, 1H), 8.70 (dd, *J* = 4.2, 1.8 Hz, 1H), 8.14 (dd, *J* = 8.4, 1.2 Hz, 1H), 7.54 - 7.49 (m, 2H), 7.47 (d, *J* = 8.4 Hz, 2H), 7.42 (dd, *J* = 7.8, 4.2 Hz, 1H), 7.08 - 7.05 (m, 2H), 7.04 - 7.02 (m, 3H), 6.94 (d, *J* = 7.2 Hz, 2H), 5.62 (d, *J* = 15.6 Hz, 1H), 5.53 (dt, *J* = 15.6, 6.6 Hz, 1H), 5.31 (d, *J* = 7.2 Hz, 1H), 4.22 (dt, *J* = 7.2, 7.2 Hz, 1H), 3.26 - 3.19 (m, 2H), 2.31 (s, 3H), 1.74 - 1.69 (m, 1H), 1.68 - 1.63 (m, 1H), 1.47 - 1.42 (m, 1H), 1.21 - 1.16 (m, 1H), 1.03 (s, 3H), 0.99 (s, 3H). **^{13}C NMR** (150 MHz, $CDCl_3$) δ 170.3, 148.3, 146.4, 142.8, 141.2, 138.6, 138.1, 136.4, 134.5, 129.2, 128.4, 128.0, 127.5, 127.2, 127.1, 126.5, 121.8, 121.7, 119.1, 116.4, 59.1, 42.2, 38.7, 36.2, 32.9, 27.5, 26.8, 21.5. **IR** ν_{max} (film): 2956, 2925, 1771, 1734, 1683, 1653, 1522, 1507, 1473, 1419, 1386, 1374, 1326, 1245, 1158, 748, 549 cm^{-1}. **HRMS** (ESI) m/z calcd for $C_{32}H_{35}N_3NaO_3S$ $[M+Na]^+$: 564.2291; found: 564.2293.

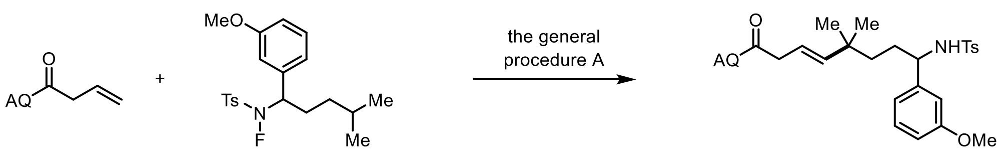

1a **2p** **3p**

Product **3p** was prepared by the general procedure A. Purification using column chromatography (PE/EA = 5:1) afforded **3p** as yellow oil (82.3 mg, 0.14 mmol, 72%, *r.r.* > 20:1, *E/Z* > 20:1). **^{1}H NMR** (600 MHz, $CDCl_3$) δ 10.04 (s, 1H), 8.76 (dd, *J* = 7.2, 1.8 Hz, 1H), 8.69 (dd, *J* = 4.2, 1.2 Hz, 1H), 8.13 (d, *J* = 7.8, 1.2 Hz, 1H), 7.53 - 7.49 (m, 2H), 7.48 (d, *J* = 7.8 Hz, 2H), 7.41 (dd, *J* = 7.8, 4.2 Hz, 1H), 7.03 (d, *J* = 7.8 Hz, 2H), 6.97 (t, *J* = 7.8 Hz, 1H), 6.66 (dd, *J* = 8.4, 3.0 Hz, 1H), 6.56 (d, *J* = 7.2 Hz, 1H), 6.45 (s, 1H), 5.63 (d, *J* = 15.6 Hz, 1H), 5.54 (dt, *J* = 15.6,

7.2 Hz, 1H), 5.39 (d, J = 7.8 Hz, 1H), 4.19 (dt, J = 7.8, 7.8 Hz, 1H), 3.60 (s, 3H), 3.27 - 3.20 (m, 2H), 2.31 (s, 3H), 1.77 - 1.72 (m, 1H), 1.69 - 1.64 (m, 1H), 1.48 - 1.44 (m, 1H), 1.24 - 1.19 (m, 1H), 1.04 (s, 3H), 1.00 (s, 3H). **^{13}C NMR** (150 MHz, $CDCl_3$) δ 170.3, 159.6, 148.3, 146.4, 142.8, 138.6, 138.1, 136.4, 134.5, 129.5, 129.2, 128.0, 127.4, 127.1, 121.8, 121.7, 119.1, 118.9, 116.4, 112.9, 112.1, 59.1, 55.1, 42.2, 38.7, 36.2, 32.9, 27.6, 26.7, 21.5. **IR** ν_{max} (film): 2956, 2925, 2854, 1683, 1670, 1653, 1526, 1472, 1457, 1424, 1386, 1326, 1259, 1159, 749, 660, 551, 418 cm^{-1}. **HRMS** (ESI) m/z calcd for $C_{33}H_{37}N_3NaO_4S$ $[M+Na]^+$: 594.2397; found: 594.2385.

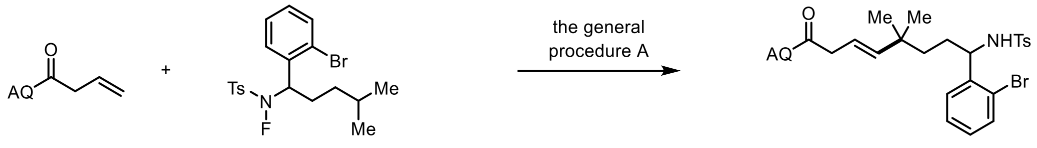

1a **2q** **3q**

Product **3q** was prepared by the general procedure A. Purification using column chromatography (PE/EA = 5:1) afforded **3q** as yellow solid (95.3 mg, 0.15 mmol, 77%, *r.r.* > 20:1, *E/Z* > 20:1). **^{1}H NMR** (600 MHz, DMSO-d_6) δ 10.07 (s, 1H), 8.72 (d, J = 3.0 Hz, 1H), 8.60 (d, J = 7.8 Hz, 1H), 8.37 (d, J = 7.8 Hz, 1H), 8.30 (d, J = 9.0 Hz, 1H), 7.64 (d, J = 8.4 Hz, 1H), 7.58 - 7.55 (m, 2H), 7.46 (d, J = 7.8 Hz, 2H), 7.32 (d, J = 7.8 Hz, 1H), 7.26 (d, J = 7.2 Hz, 1H), 7.16 (d, J = 7.8 Hz, 2H), 7.08 (t, J = 7.8 Hz, 1H), 6.98 (t, J = 7.8 Hz, 1H), 5.61 (d, J = 15.6 Hz, 1H), 5.48 (dt, J = 15.6, 6.6 Hz, 1H), 4.52 - 4.49 (m, 1H), 3.23 (d, J = 6.6 Hz, 2H), 2.25 (s, 3H), 1.50 - 1.44 (m, 2H), 1.42 - 1.37 (m, 1H), 1.02 - 0.98 (m, 1H), 0.94 (s, 6H). **^{13}C NMR** (150 MHz, DMSO-d_6) δ 169.5, 148.6, 144.9, 142.2, 141.6, 138.2, 137.8, 136.5, 134.2, 131.9, 129.1, 128.4, 127.74, 127.71, 127.6, 127.0, 126.2, 122.1, 121.72, 121.68, 119.1, 115.8, 57.0, 41.0, 38.5, 35.4, 31.8, 27.1, 26.7, 20.8. **IR** ν_{max} (film): 3441, 2251, 2125, 1653, 1558, 1540, 1521, 1507, 1457, 1008, 823, 761, 626 cm^{-1}. **HRMS** (ESI) m/z calcd for $C_{32}H_{34}BrN_3NaO_3S$ $[M+Na]^+$: 642.1396; found: 642.1384.

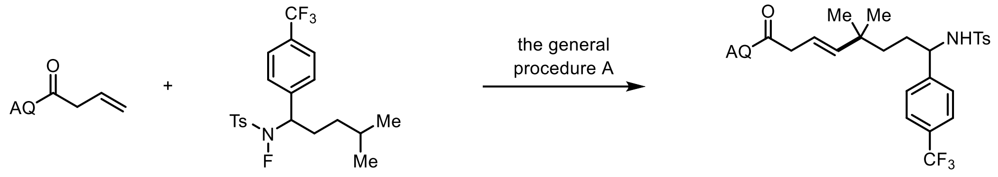

1a **2r** **3r**

Product **3r** was prepared by the general procedure A. Purification using column chromatography (PE/EA = 5:1) afforded **3r** as yellow oil (91.4 mg, 0.15 mmol, 75%, *r.r.* > 20:1, *E/Z* > 20:1). **^{1}H NMR** (600 MHz, $CDCl_3$) δ 10.03 (s, 1H), 8.75 (dd, J = 6.0, 3.6 Hz, 1H), 8.66 (dd, J = 4.2, 1.8 Hz, 1H), 8.13 (d, J = 8.4, 1.8 Hz, 1H), 7.53 - 7.49 (m, 2H), 7.41 - 7.38 (m, 3H), 7.18

(d, J = 7.8 Hz, 2H), 7.05 (d, J = 7.8 Hz, 2H), 6.95 (d, J = 7.8 Hz, 2H), 6.04 (d, J = 8.4 Hz, 1H), 5.63 (d, J = 15.6 Hz, 1H), 5.54 (dt, J = 15.6, 6.6 Hz, 1H), 4.31 - 4.27 (m, 1H), 3.27 - 3.22 (m, 2H), 2.27 (s, 3H), 1.75 - 1.68 (m, 1H), 1.66 - 1.60 (m, 1H), 1.55 - 1.50 (m, 1H), 1.26 - 1.21 (m, 1H), 1.03 (s, 3H), 0.99 (s, 3H). **^{13}C NMR** (150 MHz, $CDCl_3$) δ 170.4, 148.3, 146.2, 145.3, 143.0, 138.5, 137.9, 136.4, 134.4, 129.2 (q, J = 31.5 Hz), 129.0, 128.0, 127.3, 127.01, 126.99, 125.1 (q, J = 3.0 Hz), 124.1 (q, J = 270.3), 121.81, 121.76, 119.3, 116.4, 58.8, 42.1, 38.7, 36.2, 32.7, 27.7, 26.5, 21.3. **^{19}F NMR** (470 MHz, $CDCl_3$) δ -62.5 (s). **IR** ν_{max} (film): 3313, 2930, 1734, 1716, 1683, 1618, 1597, 1528, 1486, 1457, 1425, 1386, 1325, 1243, 1160, 1122, 1093, 1067, 1018, 814, 792, 750, 548 cm^{-1}. **HRMS** (ESI) m/z calcd for $C_{33}H_{34}F_3N_3NaO_3S$ $[M+Na]^+$: 632.2165; found: 632.2174.

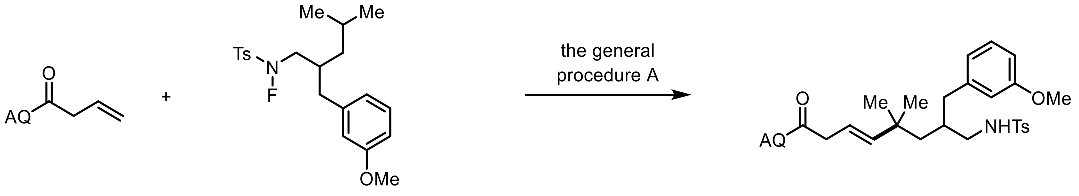

1a **2s** **3s**

Product **3s** was prepared by the general procedure A. Purification using column chromatography (PE/EA = 5:1) afforded **3s** as yellow oil (73.7 mg, 0.13 mmol, 63%, *r.r.* > 20:1, *E/Z* > 20:1). **^{1}H NMR** (600 MHz, $CDCl_3$) δ 9.93 (s, 1H), 8.77 (dd, J = 7.2, 1.8 Hz, 1H), 8.70 (dd, J = 4.2, 1.8 Hz, 1H), 8.17 (d, J = 7.8, 1.2 Hz, 1H), 7.63 (d, J = 7.8 Hz, 2H), 7.56 - 7.51 (m, 2H), 7.44 (dd, J = 7.8, 4.2 Hz, 1H), 7.19 (d, J = 7.8 Hz, 2H), 7.10 (t, J = 7.8 Hz, 1H), 6.70 (dd, J = 8.4, 3.0 Hz, 1H), 6.65 - 6.62 (m, 2H), 5.57 (d, J = 15.6 Hz, 1H), 5.51 - 5.46 (m, 1H), 5.23 (t, J = 6.6 Hz, 1H), 3.74 (s, 3H), 3.24 - 3.16 (m, 2H), 2.87 - 2.83 (m, 1H), 2.81 - 2.77 (m, 1H), 2.60 (dd, J = 13.8, 7.2 Hz, 1H), 2.51 (dd, J = 13.8, 7.2 Hz, 1H), 2.38 (s, 3H), 1.81 - 1.77 (m, 1H), 1.42 - 1.39 (m, 1H), 1.35 - 1.32 (m, 1H), 1.02 (s, 3H), 0.93 (s, 3H). **^{13}C NMR** (150 MHz, $CDCl_3$) δ 170.4, 159.7, 148.3, 146.5, 143.1, 141.7, 138.6, 137.3, 136.5, 134.4, 129.6, 129.4, 128.1, 127.5, 127.2, 121.84, 121.80, 118.8, 116.7, 115.2, 111.5, 55.3, 48.0, 44.1, 42.0, 40.8, 36.9, 36.7, 28.8, 26.1, 21.6. **IR** ν_{max} (film): 3314, 2925, 1683, 1597, 1526, 1487, 1457, 1424, 1386, 1326, 1260, 1159, 1092, 1041, 977, 912, 826, 791, 742, 617, 551 cm^{-1}. **HRMS** (ESI) m/z calcd for $C_{34}H_{39}N_3NaO_4S$ $[M+Na]^+$: 608.2553; found: 608.2543.

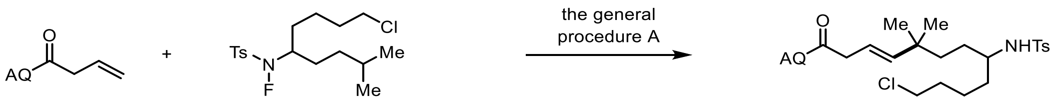

1a **2t** **3t**

Product **3t** was prepared by the general procedure A. Purification using column chromatography (PE/EA = 5:1) afforded **3t** as yellow oil (83.3 mg, 0.15 mmol, 75%, *r.r.* > 20:1, *E/Z* > 20:1). **^{1}H NMR** (600 MHz, $CDCl_3$) δ 10.03 (s, 1H), 8.77 - 8.75 (m, 2H), 8.14 (dd, *J* = 8.4, 1.8 Hz, 1H), 7.72 (d, *J* = 8.4 Hz, 2H), 7.54 - 7.48 (m, 2H), 7.44 (dd, *J* = 8.4, 4.2 Hz, 1H), 7.24 (d, *J* = 8.4 Hz, 2H), 5.60 (d, *J* = 15.6 Hz, 1H), 5.54 (dt, *J* = 15.6, 6.6 Hz, 1H), 4.87 (d, *J* = 8.4 Hz, 1H), 3.32 (t, *J* = 6.6 Hz, 2H), 3.23 (d, *J* = 6.6 Hz, 2H), 3.18 - 3.13 (m, 1H), 2.39 (s, 3H), 1.55 - 1.50 (m, 2H), 1.39 - 1.31 (m, 2H), 1.28 - 1.18 (m, 5H), 1.16 - 1.11 (m, 1H), 0.99 (s, 6H). **^{13}C NMR** (150 MHz, $CDCl_3$) δ 170.1, 148.2, 146.4, 143.2, 138.7, 138.5, 136.4, 134.4, 129.6, 128.0, 127.4, 127.1, 121.71, 121.65, 118.9, 116.3, 54.6, 44.7, 42.2, 38.2, 36.0, 34.6, 32.3, 30.1, 27.2, 26.9, 22.7, 21.5. **IR** ν_{max} (film): 3315, 2954, 2866, 1678, 1596, 1528, 1485, 1460, 1425, 1386, 1326, 1156, 1092, 978, 826, 815, 792, 757, 580, 550 cm^{-1}. **HRMS** (ESI) m/z calcd for $C_{30}H_{38}ClN_3NaO_3S$ $[M+Na]^+$: 578.2215; found: 578.2235.

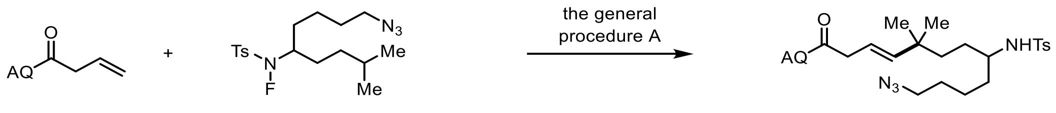

1a **2u** **3u**

Product **3u** was prepared by the general procedure A. Purification using column chromatography (PE/EA = 3:1) afforded **3u** as yellow oil (84.3 mg, 0.15 mmol, 75%, *r.r.* > 20:1, *E/Z* > 20:1). **^{1}H NMR** (600 MHz, $CDCl_3$) δ 10.03 (s, 1H), 8.77 - 8.75 (m, 2H), 8.15 (dd, *J* = 8.4, 1.8 Hz, 1H), 7.72 (d, *J* = 7.8 Hz, 2H), 7.54 - 7.49 (m, 2H), 7.45 (dd, *J* = 8.4, 4.2 Hz, 1H), 7.25 (d, *J* = 7.8 Hz, 2H), 5.59 (d, *J* = 15.6 Hz, 1H), 5.54 (dt, *J* = 15.6, 6.6 Hz, 1H), 4.79 (d, *J* = 8.4 Hz, 1H), 3.23 (d, *J* = 6.6 Hz, 2H), 3.18 - 3.12 (m, 1H), 3.06 (t, *J* = 7.2 Hz, 2H), 2.39 (s, 3H), 1.38 - 1.32 (m, 4H), 1.26 - 1.20 (m, 4H), 1.17 - 1.10 (m, 2H), 0.98 (s, 6H). **^{13}C NMR** (150 MHz, $CDCl_3$) δ 170.1, 148.2, 146.4, 143.3, 138.7, 138.6, 136.4, 134.4, 129.6, 128.0, 127.5, 127.1, 121.73, 121.68, 119.0, 116.4, 54.6, 51.2, 42.2, 38.2, 36.0, 34.9, 30.1, 28.6, 27.2, 26.9, 22.5, 21.5. **IR** ν_{max} (film): 3315, 2944, 2864, 2094, 1678, 1528, 1485, 1461, 1425, 1386, 1326, 1260, 1157, 1092, 978, 826, 815, 792, 756, 580, 550 cm^{-1}. **HRMS** (ESI) m/z calcd for $C_{30}H_{38}N_6NaO_3S$ $[M+Na]^+$: 585.2618; found: 585.2610.

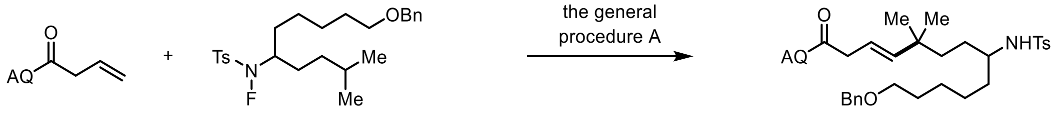

1a **2v** **3v**

Product **3v** was prepared by the general procedure A. Purification using column chromatography (PE/EA = 3:1) afforded **3v** as yellow oil (89.8 mg, 0.14 mmol, 70%, *r.r.* > 20:1, *E/Z* > 20:1). **^{1}H NMR** (600 MHz, $CDCl_3$) δ 10.04 (s, 1H), 8.78 - 8.74 (m, 2H), 8.12 (dd, *J* = 7.8, 1.2 Hz, 1H), 7.71 (d, *J* = 7.8 Hz, 2H), 7.53 - 7.41 (m, 2H) 7.42 (dd, *J* = 8.4, 4.2 Hz, 1H), 7.34 - 7.31 (m, 4H), 7.28 - 7.26 (m, 1H), 7.22 (d, *J* = 7.8 Hz, 2H), 5.60 (d, *J* = 15.6 Hz, 1H), 5.54 (dt, *J* = 15.6, 6.6 Hz, 1H), 4.65 (d, *J* = 8.4 Hz, 1H), 4.45 (s, 2H), 3.35 (t, *J* = 6.6 Hz, 2H), 3.22 (d, *J* = 6.6 Hz, 2H), 3.18 - 3.12 (m, 1H), 2.36 (s, 3H), 1.44 - 1.39 (m, 2H), 1.38 - 1.34 (m, 2H), 1.33 - 1.19 (m, 4H), 1.18 - 1.11 (m, 4H), 0.99 (s, 6H). **^{13}C NMR** (150 MHz, $CDCl_3$) δ 170.1, 148.2, 146.5, 143.1, 138.8, 138.7, 138.6, 136.4, 134.5, 129.6, 128.4, 128.0, 127.7, 127.6, 127.5, 127.1, 121.7, 121.6, 118.9, 116.3, 72.9, 70.3, 54.8, 42.2, 38.2, 36.0, 35.2, 30.2, 29.6, 27.2, 26.9, 26.0, 25.2, 21.5. **IR** ν_{max} (film): 3313, 2937, 2859, 1771, 1683, 1526, 1486, 1457, 1424, 1386, 1326, 1245, 1158, 1094, 979, 826, 748, 697, 580, 550, 418 cm^{-1}. **HRMS** (ESI) m/z calcd for $C_{38}H_{47}N_3NaO_4S$ $[M+Na]^+$: 664.3179; found: 664.3167.

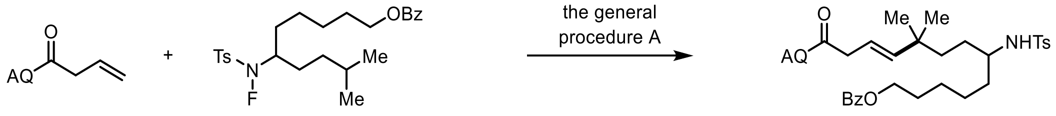

1a **2w** **3w**

Product **3w** was prepared by the general procedure A. Purification using column chromatography (PE/EA = 3:1) afforded **3w** as yellow oil (94.4 mg, 0.14 mmol, 72%, *r.r.* > 20:1, *E/Z* > 20:1). **^{1}H NMR** (600 MHz, $CDCl_3$) δ 10.04 (s, 1H), 8.78 - 8.74 (m, 2H), 8.12 (dd, *J* = 7.8, 1.2 Hz, 1H), 8.03 (d, *J* = 7.2 Hz, 2H), 7.73 (d, *J* = 7.8 Hz, 2H), 7.56 - 7.49 (m, 2H), 7.47 (dd, *J* = 8.4, 1.2 Hz, 1H), 7.44 - 7.41 (m, 3H), 7.23 (d, *J* = 7.8 Hz, 2H), 5.60 (d, *J* = 15.6 Hz, 1H), 5.54 (dt, *J* = 15.6, 7.2 Hz, 1H), 4.85 (d, *J* = 7.8 Hz, 1H), 4.19 (t, *J* = 6.6 Hz, 2H), 3.23 (d, *J* = 7.2 Hz, 2H), 3.19 - 3.14 (m, 1H), 2.36 (s, 3H), 1.58 - 1.53 (m, 2H), 1.41 - 1.31 (m, 2H), 1.29 - 1.22 (m, 4H), 1.19 - 1.10 (m, 4H), 0.99 (s, 6H). **^{13}C NMR** (150 MHz, $CDCl_3$) δ 170.1, 166.6, 148.2, 146.5, 143.1, 138.8, 138.5, 136.4, 134.4, 132.9, 130.5, 129.6, 129.5, 128.4, 128.0, 127.4, 127.0, 121.7, 121.6, 118.9, 116.3, 64.9, 54.7, 42.2, 38.2, 36.0, 35.2, 30.1, 28.6, 27.2, 26.9, 25.8, 25.0, 21.5. **IR** ν_{max} (film): 3313, 2950, 2862, 1772, 1716, 1683, 1576, 1527, 1486, 1457, 1424, 1386, 1326, 1275, 1157, 1114, 1095, 1069, 1026, 978, 913, 826, 792, 750, 713, 580, 550, 418 cm^{-1}. **HRMS** (ESI) m/z calcd for $C_{38}H_{45}N_3NaO_5S$ $[M+Na]^+$: 678.2972; found: 678.2958.

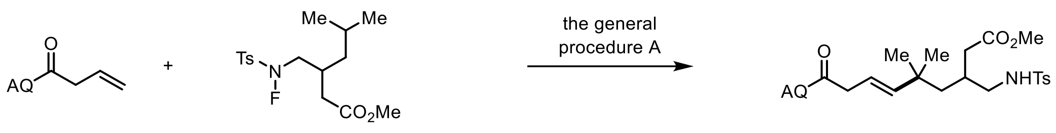

1a **2x** **3x**

Product **3x** was prepared by the general procedure A. Purification using column chromatography (PE/EA = 3:1) afforded **3x** as yellow oil (83.8 mg, 0.16 mmol, 78%, *r.r.* > 20:1, *E/Z* > 20:1). **^{1}H NMR** (600 MHz, $CDCl_3$) δ 9.94 (s, 1H), 8.79 - 8.76 (m, 2H), 8.17 (dd, *J* = 8.4, 1.8 Hz, 1H), 7.66 (d, *J* = 7.8 Hz, 2H), 7.56 - 7.52 (m, 2H), 7.46 (dd, *J* = 8.4, 4.2 Hz, 1H) 7.20 (d, *J* = 7.8 Hz, 2H), 5.67 (d, *J* = 15.6 Hz, 1H), 5.58 - 5.53 (m, 1H), 5.46 (t, *J* = 6.6 Hz, 1H), 3.59 (s, 3H), 3.27 - 3.19 (m, 2H), 2.92 - 2.88 (m, 1H), 2.85 - 2.80 (m, 1H), 2.42 (dd, *J* = 15.6, 6.6 Hz, 1H), 2.37 (s, 3H), 2.33 (dd, *J* = 15.6, 6.6 Hz, 1H), 2.03 - 2.00 (m, 1H), 1.45 - 1.43 (m, 1H), 1.32 - 1.29 (m, 1H), 1.07 (s, 3H), 1.06 (s, 3H). **^{13}C NMR** (150 MHz, $CDCl_3$) δ 173.3, 170.2, 148.4, 146.1, 143.2, 138.6, 137.4, 136.6, 134.4, 129.7, 128.1, 127.5, 127.1, 121.9, 121.8, 119.2, 116.7, 51.7, 48.0, 44.5, 41.9, 38.7, 36.8, 32.2, 28.9, 26.0, 21.6. **IR** ν_{max} (film): 3316, 2955, 1735, 1683, 1597, 1528, 1486, 1425, 1386, 1326, 1245, 1092, 980, 826, 792, 758, 661, 551 cm^{-1}. **HRMS** (ESI) m/z calcd for $C_{29}H_{35}N_3NaO_5S$ $[M+Na]^+$: 560.2190; found: 560.2188.

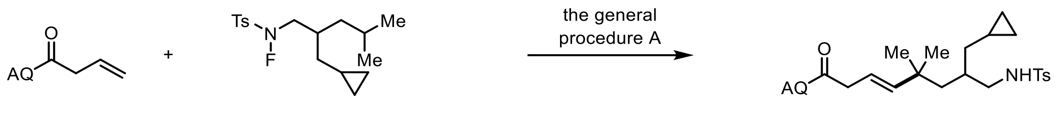

1a **2y** **3y**

Product **3y** was prepared by the general procedure A. Purification using column chromatography (PE/EA = 5:1) afforded **3y** as yellow oil (83.1 mg, 0.16 mmol, 80%, *r.r.* > 20:1, *E/Z* > 20:1). **^{1}H NMR** (600 MHz, $CDCl_3$) δ 9.96 (s, 1H), 8.78 - 8.76 (m, 2H), 8.16 (dd, *J* = 8.4, 1.8 Hz, 1H), 7.69 (d, *J* = 8.4 Hz, 2H), 7.55 - 7.50 (m, 2H), 7.45 (dd, *J* = 7.8, 4.2 Hz, 1H), 7.21 (d, *J* = 8.4 Hz, 2H), 5.69 (d, *J* = 15.6 Hz, 1H), 5.51 (dt, *J* = 15.6, 7.2 Hz, 1H), 5.32 - 5.28 (m, 1H), 3.25 - 3.23 (m, 2H), 2.87 (dd, *J* = 6.6, 6.6 Hz, 2H), 2.38 (s, 3H), 1.65 - 1.59 (m, 1H), 1.42 - 1.39 (m, 1H), 1.35 - 1.32 (m, 1H), 1.23 - 1.15 (m, 2H), 1.06 (s, 6H), 0.60 - 0.53 (m, 1H), 0.38 - 0.32 (m, 2H), -0.01 - (-0.08) (m, 2H). **^{13}C NMR** (150 MHz, $CDCl_3$) δ 170.5, 148.3, 146.7, 143.0, 138.6, 137.4, 136.5, 134.4, 129.6, 128.1, 127.5, 127.2, 121.79, 121.76, 118.6, 116.6, 48.2, 44.6, 42.0, 39.4, 37.0, 35.3, 29.0, 25.9, 21.6, 8.7, 5.00, 4.97. **IR** ν_{max} (film): 3313, 2918, 2850, 1771, 1749, 1716, 1683, 1670, 1653, 1636, 1558, 1526, 1507, 1487, 1473, 1436, 1424, 1326, 1242, 1160, 1093, 1064, 792, 749, 550 cm^{-1}. **HRMS** (ESI) m/z calcd for $C_{30}H_{37}N_3NaO_3S$ $[M+Na]^+$: 542.2448; found: 542.2436.

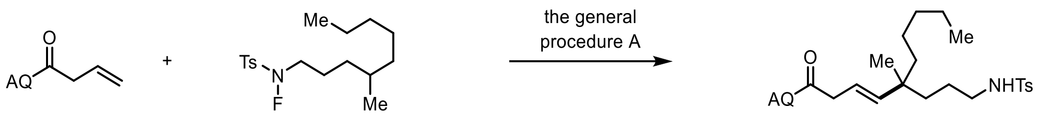

Product **3z** was prepared by the general procedure A. Purification using column chromatography (PE/EA = 5:1) afforded **3z** as yellow oil (83.4 mg, 0.16 mmol, 80%, *r.r.* > 20:1, *E/Z* > 20:1). **^{1}H NMR** (600 MHz, $CDCl_3$) δ 9.99 (s, 1H), 8.77 - 8.75 (m, 2H), 8.16 (dd, *J* = 8.4, 1.8 Hz, 1H), 7.70 (d, *J* = 7.8 Hz, 2H), 7.54 - 7.49 (m, 2H), 7.45 (dd, *J* = 8.4, 4.2 Hz, 1H), 7.25 (d, *J* = 7.8 Hz, 2H), 5.59 (d, *J* = 15.6 Hz, 1H), 5.51 (dt, *J* = 15.6, 6.0 Hz, 1H), 4.67 (t, *J* = 6.6 Hz, 1H), 3.26 (d, *J* = 6.6 Hz, 2H), 2.90 (dt, *J* = 6.6, 6.6 Hz, 2H) 2.39 (s, 3H), 1.47 - 1.42 (m, 2H), 1.33 - 1.27 (m, 4H), 1.27 - 1.19 (m, 6H), 0.99 (s, 3H), 0.82 (t, *J* = 6.6 Hz, 3H). **^{13}C NMR** (150 MHz, $CDCl_3$) δ 170.3, 148.3, 145.5, 143.4, 138.6, 137.2, 136.5, 134.5, 129.7, 128.1, 127.5, 127.2, 121.8, 121.7, 119.9, 116.5, 44.1, 42.4, 41.2, 39.0, 37.7, 32.8, 24.6, 23.8, 23.3, 22.7, 21.6, 14.2. **IR** ν_{max} (film): 3735, 3629, 2928, 1683, 1596, 1526, 1486, 1457, 1424, 1326, 1286, 1242, 1159, 1093, 913, 826, 742, 550 cm^{-1}. **HRMS** (ESI) m/z calcd for $C_{30}H_{39}N_3NaO_3S$ $[M+Na]^+$: 544.2604; found: 544.2601.

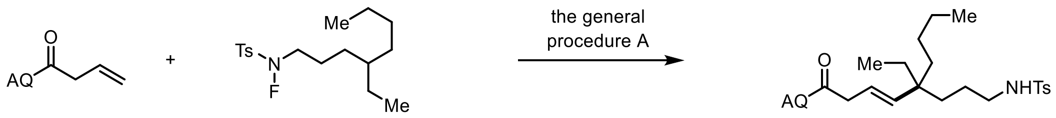

Product **3aa** was prepared by the general procedure A. Purification using column chromatography (PE/EA = 5:1) afforded **3aa** as yellow oil (85.5 mg, 0.16 mmol, 82%, *r.r.* > 20:1, *E/Z* > 20:1). **^{1}H NMR** (600 MHz, $CDCl_3$) δ 9.96 (s, 1H), 8.78 - 8.75 (m, 2H), 8.16 (dd, *J* = 8.4, 1.8 Hz, 1H), 7.69 (d, *J* = 8.4 Hz, 2H), 7.55 - 7.50 (m, 2H), 7.45 (dd, *J* = 8.4, 4.2 Hz, 1H), 7.26 (d, *J* = 8.4 Hz, 2H), 5.57 - 5.49 (m, 2H), 4.43 (t, *J* = 6.6 Hz, 1H), 3.28 (d, *J* = 6.0 Hz, 2H), 2.90 (dt, *J* = 6.6, 6.6 Hz, 2H) 2.40 (s, 3H), 1.42 - 1.35 (m, 4H), 1.32 - 1.28 (m, 4H), 1.25 - 1.22 (m, 2H), 1.16 - 1.12 (m, 2H), 0.84 (t, *J* = 7.2 Hz, 3H), 0.78 (t, *J* = 7.2 Hz, 3H). **^{13}C NMR** (150 MHz, $CDCl_3$) δ 170.3, 148.3, 145.0, 143.4, 138.6, 137.2, 136.5, 134.6, 129.8, 128.1, 127.5, 127.2, 121.8, 121.7, 120.7, 116.6, 44.2, 42.7, 41.6, 35.7, 33.6, 29.1, 25.7, 24.0, 23.6, 21.6, 14.2, 8.1. **IR** ν_{max} (film): 3442, 2923, 2854, 1666, 1640, 1597, 1527, 1485, 1425, 1377, 1328, 1242, 1159, 1093, 826, 792, 550 cm^{-1}. **HRMS** (ESI) m/z calcd for $C_{30}H_{39}N_3NaO_3S$ $[M+Na]^+$: 544.2604; found: 544.2603.

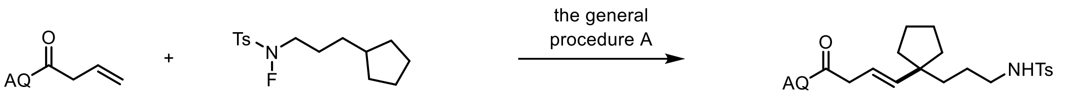

1a **2ab** **3ab**

Product **3ab** was prepared by the general procedure A. Purification using column chromatography (PE/EA = 5:1) afforded **3ab** as yellow oil (83.5 mg, 0.17 mmol, 85%, *r.r.* > 20:1, *E/Z* > 20:1). **^{1}H NMR** (600 MHz, $CDCl_3$) δ 9.97 (s, 1H), 8.78 - 8.74 (m, 2H), 8.16 (dd, *J* = 8.4, 1.8 Hz, 1H), 7.69 (d, *J* = 8.4 Hz, 2H), 7.55 - 7.50 (m, 2H), 7.45 (dd, *J* = 8.4, 4.2 Hz, 1H), 7.27 (d, *J* = 8.4 Hz, 2H), 5.67 (d, *J* = 15.6 Hz, 1H), 5.59 (dt, *J* = 15.6, 7.2 Hz, 1H), 4.37 (t, *J* = 6.6 Hz, 1H), 3.26 (d, *J* = 7.2 Hz, 2H), 2.90 (dt, *J* = 6.6, 6.6 Hz, 2H) 2.41 (s, 3H), 1.72 - 1.69 (m, 4H), 1.65 - 1.61 (m, 2H), 1.48 - 1.44 (m, 2H), 1.43 - 1.38 (m, 4H). **^{13}C NMR** (150 MHz, $CDCl_3$) δ 170.2, 148.2, 144.4, 143.4, 138.7, 137.2, 136.5, 134.6, 129.8, 128.1, 127.6, 127.2, 121.8, 121.7, 119.8, 116.6, 48.7, 44.0, 42.4, 37.5, 37.2, 25.9, 23.8, 21.7. **IR** ν_{max} (film): 3750, 2923, 1771, 1749, 1697, 1683, 1670, 1653, 1647, 1636, 1558, 1540, 1532, 1521, 1488, 1473, 1457, 1419, 1324, 1245, 1158, 1093, 748, 659, 549, 472, 457, 418 cm^{-1}. **HRMS** (ESI) m/z calcd for $C_{28}H_{33}N_3NaO_3S$ $[M+Na]^+$: 514.2135; found: 514.2143.

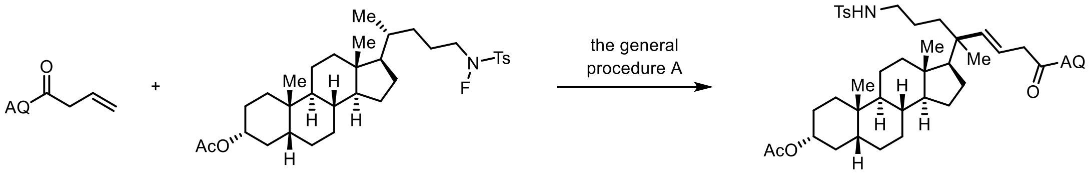

1a **2ac** **3ac**

Product **3ac** was prepared by the general procedure A. Purification using column chromatography (PE/EA = 5:1) afforded **3ac** as yellow oil (92.1 mg, 0.12 mmol, 60%, *r.r.* > 20:1, *E/Z* > 20:1, *d.r.* = 1:1). **^{1}H NMR** (600 MHz, $CDCl_3$) δ 9.91 (s, 1H), 8.78 - 8.76 (m, 2H), 8.17 - 8.15 (m, 1H), 7.70 - 7.68 (m, 2H), 7.55 - 7.50 (m, 2H), 7.47 - 7.44 (m, 1H), 7.27 -7.25 (m, 2H), 5.80 (d, *J* = 15.6 Hz, 0.5H), 5.69 (d, *J* = 15.6 Hz, 0.5H), 5.55 - 5.47 (m, 1H), 4.73 - 4.67 (m, 1H), 4.66 - 4.61 (m, 1H), 3.28 - 3.26 (m, 2H), 2.89 - 2.83 (m, 2H), 2.41 (s, 3H), 2.04 (s, 3H), 1.99 - 1.88 (m, 1H), 1.82 - 1.74 (m, 3H), 1.69 - 1.51 (m, 6H), 1.44 - 1.32 (m, 10H), 1.23 - 1.12 (m, 4H) 1.06 - 0.97 (m, 6H), 0.88 (s, 1.5H), 0.85 (s, 1.5H), 0.66 (s, 1.5 H), 0.65 (s, 1.5H). **^{13}C NMR** (150 MHz, $CDCl_3$) δ 170.8, 170.32, 170.26, 148.29, 148.25, 145.0, 144.1, 143.30, 143.29, 138.53, 138.52, 137.2, 136.5, 134.5, 129.73, 129.72, 128.1, 127.5, 127.2, 121.8, 121.7, 119.23, 119.17, 116.6, 74.5, 60.4, 60.1, 56.7, 56.6, 44.10, 44.07, 44.00, 43.96, 42.7, 42.6, 42.5, 42.3, 41.9, 41.1, 40.8, 40.5, 38.9, 38.2, 35.4, 35.3, 35.11, 35.08, 34.65, 34.63, 32.4, 27.1, 26.7, 26.3, 24.3, 24.2, 23.9, 23.7, 23.40, 23.39, 23.2, 22.7, 21.64, 21.62, 20.8, 20.69, 20.66, 15.0, 14.9. **IR** ν_{max} (film):

3326, 2931, 2867, 2359, 2341, 1732, 1683, 1597, 1526, 1486, 1425, 1383, 1362, 1326, 1244, 1160, 1094, 1027, 981, 912, 826, 792, 732, 550 cm^{-1}. **HRMS** (ESI) m/z calcd for $C_{46}H_{61}N_3NaO_5S$ $[M+Na]^+$: 790.4224; found: 790.4228.

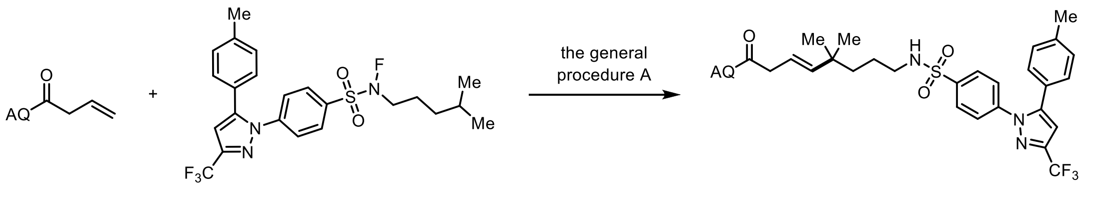

1a **2ad** **3ad**

Product **3ad** was prepared by the general procedure A. Purification using column chromatography (PE/EA = 5:1) afforded **3ad** as yellow oil (105 mg, 0.16 mmol, 78%, *r.r.* > 20:1, *E/Z* > 20:1). ^{1}**H NMR** (600 MHz, $CDCl_3$) δ 10.03 (s, 1H), 8.75 - 8.73 (m, 2H), 8.14 (dd, J = 8.4, 1.8 Hz, 1H), 7.81 (d, J = 8.4 Hz, 2H), 7.52 - 7.48 (m, 2H), 7.45 - 7.42 (m, 3H), 7.16 (d, J = 7.8 Hz, 2H), 7.09 (d, J = 7.8 Hz, 2H), 6.74 (s, 1H), 5.69 (d, J = 15.6 Hz, 1H), 5.60 (dt, J = 15.6, 7.2 Hz, 1H), 4.80 (t, J = 6.6 Hz, 1H), 3.26 (d, J = 7.2 Hz, 2H), 2.90 (dt, J = 6.6, 6.6 Hz, 2H), 2.37 (s, 3H), 1.52 - 1.47 (m, 2H), 1.38 - 1.35 (m, 2H), 1.07 (s, 6H). ^{13}**C NMR** (150 MHz, $CDCl_3$) δ 170.2, 148.3, 146.3, 145.3, 144.2 (q, J = 39.0 Hz), 142.5, 139.9, 139.6, 138.6, 136.5, 134.4, 129.9, 128.8, 128.2, 128.1, 127.5, 125.8, 125.6, 121.80, 121.76, 121.2, (q, J = 267.0 Hz), 119.2, 116.5, 106.4, 44.0, 42.2, 39.5, 36.3, 27.2, 25.0, 21.5. ^{19}**F NMR** (470 MHz, $CDCl_3$) δ -62.4 (s). **IR** ν_{max} (film): 3316, 2959, 2927, 2869, 1673, 1597, 1529, 1498, 1486, 1471, 1411, 1328, 1271, 1237, 1162, 1136, 1097, 976, 910, 842, 826, 806, 759, 733, 626, 616, 573 cm^{-1}. **HRMS** (ESI) m/z calcd for $C_{36}H_{36}F_3N_5NaO_3S$ $[M+Na]^+$: 698.2383; found: 698.2399.

The general procedure B (for products 3ae-3az):

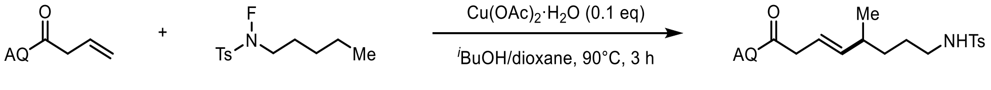

1a **2ae** **3ae**

To a dry Schlenk flask were added **1a** (42.4 mg, 0.20 mmol, 1.0 equiv), **2ae** (130 mg, 0.50 mmol, 2.5 equiv), $Cu(OAc)_2 \cdot H_2O$ (4.0 mg, 0.02 mmol, 0.10 equiv), anhydrous ^{i}BuOH (2.5 mL), and anhydrous dioxane (0.50 mL). The mixture was degassed for three times with argon and stirred at 90 °C (oil bath) for 3 h. Once completion, the reaction was cooled to room temperature. The reaction mixture was filtered by celite, and the filtrate was concentrated *in vacuo*. Further

purification by a flash column chromatography using eluents (PE/EA = 5:1) afforded the desired product **3ae** as yellow oil (72.2 mg, 0.16 mmol, 80%, *r.r.* > 20:1, *E/Z* = 5:1). **^{1}H NMR** (600 MHz, $CDCl_3$) δ 10.01 (s, 0.83H), 9.96 (s, 0.17H), 8.79 - 8.76 (m, 2H), 8.18 - 8.15 (m, 1H), 7.70 (d, *J* = 8.4 Hz, 1.66H), 7.66 (d, *J* = 8.4 Hz, 0.34H), 7.56 - 7.49 (m, 2H), 7.47 - 7.44 (m, 1H), 7.27 (d, *J* = 8.4 Hz, 1.66H), 7.24 (d, *J* = 8.4 Hz, 0.34H), 5.69 - 5.63 (m, 1H), 5.61 - 5.71 (m, 0.83H), 5.50 - 5.46 (m, 0.17H), 4.50 (t, *J* = 6.6 Hz, 0.17H), 4.41 (t, *J* = 6.6 Hz, 0.83H), 3.27 (d, *J* = 7.2 Hz, 0.34H), 3.24 (d, *J* = 7.2 Hz, 1.66H), 2.94 (dt, *J* = 6.6, 6.6 Hz, 1.66H), 2.91 - 2.88 (m, 0.34H), 2.41 (s, 2.49H), 2.39 (s, 0.51H), 2.23 - 2.16 (m, 1H), 1.57 - 1.48 (m, 2H), 1.39 - 1.34 (m, 2H), 1.06 (d, *J* = 6.6 Hz, 2.49H), 0.99 (d, *J* = 6.6 Hz, 0.51H). **^{13}C NMR** (150 MHz, $CDCl_3$) δ 170.0, 169.7, 148.4, 148.3, 143.5, 143.4, 142.3, 140.9, 138.7, 138.6, 137.20, 137.16, 136.54, 136.49, 134.6, 129.79, 129.76, 128.1, 127.6, 127.22, 127.18, 121.83, 121.80, 121.7, 121.4, 120.6, 116.7, 116.5, 43.5, 43.4, 42.2, 37.2, 36.8, 34.1, 33.7, 31.7, 27.52, 27.46, 21.7, 21.6, 21.1, 20.7. **IR** ν_{max} (film): 3310, 2995, 2923, 2852, 1675, 1596, 1525, 1485, 1457, 1424, 1379, 1325, 1245, 1158, 1092, 973, 825, 791, 659, 550 cm^{-1}. **HRMS** (ESI) m/z calcd for $C_{25}H_{29}N_3NaO_3S$ $[M+Na]^+$: 474.1822; found: 474.1812.

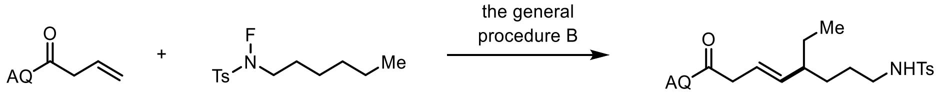

1a **2af** **3af**

Product **3af** was prepared by the general procedure B. Purification using column chromatography (PE/EA = 5:1) afforded **3af** as yellow oil (71.6 mg, 0.15 mmol, 77%, *r.r.* > 20:1, *E/Z* = 5:1). **^{1}H NMR** (600 MHz, $CDCl_3$) δ 9.97 (s, 0.83H), 9.93 (s, 0.17H), 8.79 - 8.76 (m, 2H), 8.17 - 8.14 (m, 1H), 7.70 (d, *J* = 8.4 Hz, 1.66H), 7.65 (d, *J* = 8.4 Hz, 0.34H), 7.55 - 7.49 (m, 2H), 7.46 - 7.43 (m, 1H), 7.26 (d, *J* = 8.4 Hz, 1.66H), 7.23 (d, *J* = 8.4 Hz, 0.34H), 5.80 - 5.76 (m, 0.17H), 5.65 - 5.60 (m, 0.83H), 5.47 - 5.37 (m, 1H), 4.69 - 4.62 (m, 1H), 3.29 - 3.21 (m, 2H), 2.92 (dt, *J* = 6.6, 6.6 Hz, 1.66H), 2.90 (dt, *J* = 6.6, 6.6 Hz, 0.34H), 2.39 (s, 2.49H), 2.38 (s, 0.51H), 2.29 - 2.25 (m, 0.17H), 1.95 - 1.89 (m, 0.83H), 1.58 - 1.38 (m, 4H), 1.32 - 1.22 (m, 2H), 0.90 (t, *J* = 7.2 Hz, 2.49H), 0.85 (t, *J* = 7.2 Hz, 0.51H). **^{13}C NMR** (150 MHz, $CDCl_3$) δ 170.1, 169.8, 148.4, 148.3, 143.4, 143.3, 140.8, 139.3, 138.6, 138.5, 137.19, 137.16, 136.51, 136.45, 134.53, 134.48, 129.73, 129.71, 128.08, 128.06, 127.51, 127.49, 127.2, 127.1, 122.8, 122.0, 121.81, 121.78, 121.71, 121.65, 116.6, 116.5, 44.4, 43.4, 42.3, 38.8, 37.4, 37.0, 32.2, 31.6, 28.4, 28.1, 27.4, 27.3, 24.4, 21.6, 20.6, 11.8. **IR** ν_{max} (film): 3324, 2957, 2926, 2870, 1680, 1596, 1527, 1486, 1458, 1424, 1385, 1326, 1241, 1159, 1093, 826, 814, 792, 550 cm^{-1}. **HRMS** (ESI) m/z calcd for $C_{26}H_{31}N_3NaO_3S$ $[M+Na]^+$: 488.1978; found: 488.1968.

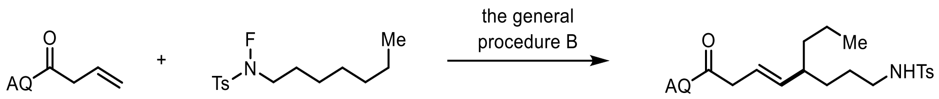

1a **2ag** **3ag**

Product **3ag** was prepared by the general procedure B. Purification using column chromatography (PE/EA = 5:1) afforded **3ag** as yellow oil (69.9 mg, 0.15 mmol, 73%, *r.r.* > 20:1, *E/Z* = 10:1). ^{1}**H NMR** (600 MHz, $CDCl_3$) δ 9.95 (s, 1H), 8.78 - 8.76 (m, 2H), 8.15 (dd, *J* = 8.4, 1.8 Hz, 1H), 7.70 (d, *J* = 8.4 Hz, 2H), 7.57 - 7.49 (m, 2H), 7.45 (dd, *J* = 8.4, 4.2 Hz, 1H), 7.26 (d, *J* = 8.4 Hz, 2H), 5.61 (dt, *J* = 15.0, 7.2 Hz, 1H), 5.45 (dd, *J* = 15.0, 9.0 Hz, 1H), 4.59 (t, *J* = 6.6 Hz, 1H), 3.25 (d, *J* = 7.2 Hz, 2H), 2.92 (dt, *J* = 6.6, 6.6 Hz, 2H), 2.39 (s, 3H), 2.05 - 1.99 (m, 1H), 1.56 - 1.45 (m, 2H), 1.43 - 1.36 (m, 2H), 1.32 - 1.26 (m, 4H), 0.86 (t, *J* = 6.6 Hz, 3H). ^{13}**C NMR** (150 MHz, $CDCl_3$) δ 170.1, 148.3, 143.4, 141.0, 138.6, 137.2, 136.5, 134.5, 129.7, 128.1, 127.5, 127.2, 122.6, 121.8, 121.7, 116.6, 43.4, 42.5, 42.3, 37.6, 32.0, 27.4, 21.6, 20.4, 14.3. **IR** ν_{max} (film): 3321, 2954, 2926, 2869, 1738, 1680, 1597, 1526, 1486, 1457, 1424, 1326, 1243, 1159, 1093, 1047, 975, 826, 815, 792, 757, 550 cm^{-1}. **HRMS** (ESI) m/z calcd for $C_{27}H_{33}N_3NaO_3S$ $[M+Na]^+$: 502.2135; found: 502.2130.

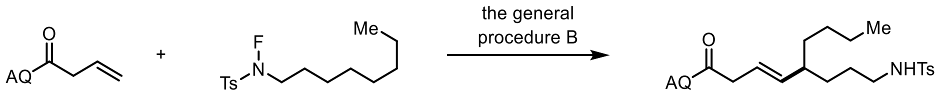

1a **2ah** **3ah**

Product **3ah** was prepared by the general procedure B. Purification using column chromatography (PE/EA = 5:1) afforded **3ah** as yellow oil (70.0 mg, 0.14 mmol, 71%, *r.r.* > 20:1, *E/Z* > 20:1). ^{1}**H NMR** (600 MHz, $CDCl_3$) δ 9.96 (s, 1H), 8.77 - 8.76 (m, 2H), 8.15 (dd, *J* = 8.4, 1.8 Hz, 1H), 7.70 (d, *J* = 8.4 Hz, 2H), 7.54 - 7.49 (m, 2H), 7.43 (dd, *J* = 7.8, 4.2 Hz, 1H), 7.25 (d, *J* = 8.4 Hz, 2H), 5.61 (dt, *J* = 15.6, 7.2 Hz, 1H), 5.45 (dd, *J* = 15.6, 9.0 Hz, 1H), 4.73 (t, *J* = 6.6 Hz, 1H), 3.25 (d, *J* = 7.2 Hz, 2H), 2.91 (dt, *J* = 6.6, 6.6 Hz, 2H), 2.39 (s, 3H), 2.02 - 1.96 (m, 1H), 1.58 - 1.52 (m, 1H), 1.50 - 1.41 (m, 2H), 1.35 - 1.32 (m, 1H), 1.29 - 1.21 (m, 6H), 0.82 (t, *J* = 7.2 Hz, 3H). ^{13}**C NMR** (150 MHz, $CDCl_3$) δ 170.1, 148.3, 143.3, 141.0, 138.5, 137.1, 136.4, 134.5, 129.7, 128.0, 127.5, 127.2, 122.5, 121.8, 121.7, 116.5, 43.4, 42.7, 42.3, 35.0, 31.9, 29.4, 27.3, 22.9, 21.6, 14.1. **IR** ν_{max} (film): 3323, 2954, 2926, 2857, 1683, 1597, 1576, 1528, 1486, 1458, 1425, 1385, 1326, 1261, 1159, 1093, 976, 912, 826, 814, 792, 740, 551 cm^{-1}. **HRMS** (ESI) m/z calcd for $C_{28}H_{35}N_3NaO_3S$ $[M+Na]^+$: 516.2291; found: 516.2280.

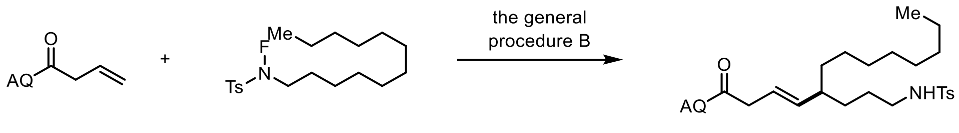

Product **3ai** was prepared by the general procedure B. Purification using column chromatography (PE/EA = 5:1) afforded **3ai** as yellow oil (69.2 mg, 0.13 mmol, 63%, *r.r.* > 20:1, *E/Z* = 5:1). **^{1}H NMR** (600 MHz, $CDCl_3$) δ 9.95 (s, 0.83H), 0.93 (s, 0.17H), 8.78 - 8.76 (m, 2H), 8.15 (dd, J = 8.4, 1.8 Hz, 1H), 7.70 (d, J = 8.4 Hz, 1.66H), 7.66 (d, J = 8.4 Hz, 0.34H), 7.54 - 7.49 (m, 2H), 7.44 (dd, J = 8.4, 4.2 Hz, 1H), 7.25 (d, J = 8.4 Hz, 1.66H), 7.22 (d, J = 8.4 Hz, 0.34H), 5.78 - 5.74 (m, 0.17H), 5.61 (dt, J = 15.0, 7.2 Hz, 0.83H), 5.45 (dd, J = 15.0, 9.0 Hz, 0.83H), 5.41 - 5.37 (m, 0.17H), 4.75 (t, J = 6.6 Hz, 0.17H), 4.71 (t, J = 6.6 Hz, 0.83H), 3.24 (d, J = 7.2 Hz, 2H), 2.91 (dt, J = 6.6, 6.6 Hz, 2H), 2.39 (s, 2.49H), 2.38 (s, 0.51H), 2.02 - 1.96 (m, 1H), 1.56 - 1.41 (m, 3H), 1.36 - 1.19 (m, 15H), 0.86 - 0.83 (m, 3H). **^{13}C NMR** (150 MHz, $CDCl_3$) δ 170.1, 169.8, 148.3, 148.2, 143.30, 143.26, 141.2, 141.0, 139.8, 138.6, 138.5, 137.20, 137.17, 136.5, 136.4, 134.51, 134.45, 129.71, 129.69, 128.1, 127.48, 127.46, 127.17, 127.13, 122.5, 122.2, 121.77, 121.75, 121.7, 121.6, 116.6, 116.5, 43.44, 43.41, 43.3, 42.9, 42.7, 42.3, 37.4, 37.2, 35.7, 35.3, 34.5, 32.5, 32.0, 31.9, 29.94, 29.90, 29.64, 29.60, 29.41, 29.36, 27.4, 27.3, 24.3, 22.74, 22.71, 21.6, 14.21, 14.18. **IR** ν_{max} (film): 3323, 3046, 2925, 2853, 1737, 1682, 1597, 1577, 1528, 1486, 1424, 1385, 1326, 1242, 1159, 1094, 1019, 975, 949, 909, 826, 792, 756, 551, 508 cm^{-1}. **HRMS** (ESI) m/z calcd for $C_{32}H_{43}N_3NaO_3S$ $[M+Na]^+$: 572.2917; found: 572.2913.

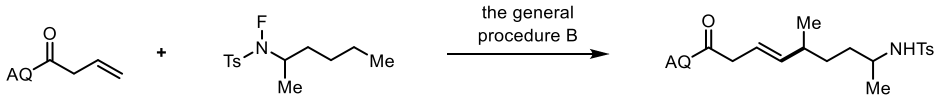

Product **3aj** was prepared by the general procedure B. Purification using column chromatography (PE/EA = 5:1) afforded **3aj** as yellow oil (68.8 mg, 0.15 mmol, 74%, *r.r.* > 20:1, *E/Z* = 10:1, *d.r.* = 1:1). **^{1}H NMR** (600 MHz, $CDCl_3$) δ 10.04 (s, 0.5H), 10.03 (s, 0.5H), 8.79 - 8.76 (m, 2H), 8.15 - 8.13 (m, 1H), 7.75 - 7.69 (m, 2H), 7.53 - 7.48 (m, 2H), 7.45 - 7.43 (m, 1H), 7.26 - 7.22 (m, 2H), 5.70 - 5.54 (m, 2H), 4.76 (d, J = 8.4 Hz, 0.5H), 4.74 (d, J = 8.4 Hz, 0.5H), 3.32 - 3.27 (m, 1H), 3.25 - 3.21 (m, 2H), 2.39 (s, 1.5H), 2.38 (s, 1.5H), 2.15 - 2.08 (m, 1H), 1.42 - 1.36 (m, 2H), 1.35 - 1.31 (m, 1H), 1.27 - 1.23 (m, 1H), 1.04 (d, J = 6.6 Hz, 1.5H), 1.01 (d, J = 6.6 Hz, 1.5H), 0.98 (d, J = 4.2 Hz, 1.5H), 0.97 (d, J = 4.2 Hz, 1.5H). **^{13}C NMR** (150 MHz, $CDCl_3$) δ 170.04, 170.01, 148.27, 148.26, 143.2, 143.1, 142.52, 142.48, 138.6, 138.52, 138.49, 136.4, 134.5, 129.6, 128.0, 127.4, 127.1, 121.7, 121.6, 121.10, 121.05, 116.38, 116.36, 50.3, 50.1, 42.2, 42.1,

36.8, 36.7, 35.3, 35.2, 32.6, 32.4, 21.9, 21.8, 21.54, 21.51, 20.6, 20.5. **IR** ν_{max} (film): 3311, 3046, 2962, 2927, 2869, 1735, 1681, 1597, 1529, 1486, 1458, 1425, 1384, 1326, 1242, 1160, 1093, 1063, 977, 904, 826, 815, 793, 758, 732, 707, 579, 552, 506 cm^{-1}. **HRMS** (ESI) m/z calcd for $C_{26}H_{31}N_3NaO_3S$ $[M+Na]^+$: 488.1978; found: 488.1973.

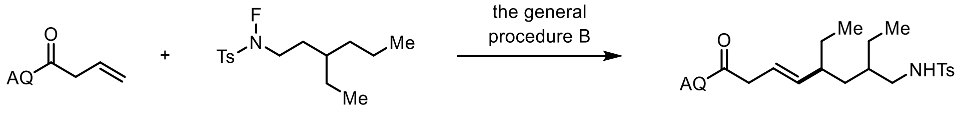

1a **2ak** **3ak**

Product **3ak** was prepared by the general procedure B. Purification using column chromatography (PE/EA = 5:1) afforded **3ak** as yellow oil (64.1 mg, 0.13 mmol, 65%, *r.r.* > 20:1, *E/Z* > 20:1, *d.r.* = 1:1). **^{1}H NMR** (600 MHz, $CDCl_3$) δ 9.93 (s, 0.5H), 9.91 (s, 0.5H), 8.79 - 8.75 (m, 2H), 8.17 - 8.15 (m, 1H), 7.72 - 7.70 (m, 2H), 7.56 - 7.50 (m, 2H), 7.46 - 7.44 (m, 1H), 7.26 - 7.25 (m, 2H), 5.60 (dt, *J* = 15.0, 7.2 Hz, 0.5H), 5.51 (dt, *J* = 15.0, 7.2 Hz, 0.5H), 5.45 - 5.42 (m, 0.5H), 5.41 - 5.38 (m, 0.5H), 4.86 (d, *J* = 6.6 Hz, 0.5 H), 4.72 (d, *J* = 6.6 Hz, 0.5 Hz), 3.29 - 3.23 (m, 2H), 2.90 - 2.81 (m, 2H), 2.40 (s, 1.5H), 2.39 (s, 1.5H), 1.99 - 1.93 (m, 1H), 1.53 - 1.48 (m, 1H), 1.42 - 1.33 (m, 2H), 1.32 - 1.29 (m, 2H), 1.27 - 1.22 (m, 2H), 0.91 - 0.87 (m, 3H), 0.79 - 0.76 (m, 3H). **^{13}C NMR** (150 MHz, $CDCl_3$) δ 170.3, 170.1, 148.3, 143.3, 143.2, 141.1, 140.8, 138.59, 138.57, 137.3, 137.1, 136.53, 136.50, 134.52, 134.51, 129.71, 129.69, 128.1, 127.6, 127.5, 127.3, 127.2, 122.8, 122.6, 121.8, 116.69, 116.67, 46.9, 45.4, 43.1, 42.18, 42.15, 42.12, 36.9, 36.7, 36.5, 28.58, 28.56, 25.2, 24.2, 21.6, 11.89, 11.85, 10.9, 10.5. **IR** ν_{max} (film): 3323, 2960, 2925, 2873, 1683, 1597, 1582, 1576, 1486, 1458, 1425, 1384, 1326, 1260, 1160, 1093, 1065, 976, 912, 826, 815, 792, 757, 732, 706, 662, 551, 419 cm^{-1}. **HRMS** (ESI) m/z calcd for $C_{28}H_{35}N_3NaO_3S$ $[M+Na]^+$: 516.2291; found: 516.2285.

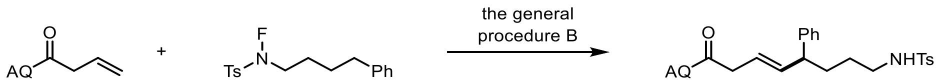

1a **2al** **3al**

Product **3al** was prepared by the general procedure B. Purification using column chromatography (PE/EA = 5:1) afforded **3al** as yellow oil (65.7 mg, 0.13 mmol, 64%, *r.r.* > 20:1, *E/Z* > 20:1). **^{1}H NMR** (600 MHz, $CDCl_3$) δ 9.90 (s, 1H), 8.68 - 8.66 (m, 2H), 8.07 (dd, *J* = 8.4, 1.8 Hz, 1H), 7.61 (d, *J* = 8.4 Hz, 2H), 7.46 - 7.41 (m, 2H), 7.37 (dd, *J* = 7.8, 4.2 Hz, 1H), 7.19 - 7.18 (m, 2H), 7.16 (d, *J* = 7.8 Hz, 2H), 7.13 - 7.10 (m, 3H), 5.80 (dd, *J* = 15.6, 7.8 Hz, 1H), 5.62 (dt, *J* = 15.6, 7.2 Hz, 1H), 4.48 (t, *J* = 6.6 Hz, 1H), 3.22 (dt, *J* = 7.2, 7.2 Hz, 1H), 3.18 (d, *J* = 7.2

Hz, 2H), 2.86 (dt, J = 6.6, 6.6 Hz, 2H), 2.31 (s, 3H), 1.78 - 1.67 (m, 2H), 1.49 - 1.42 (m, 1H), 1.39 - 1.32 (m, 1H). ^{13}**C NMR** (150 MHz, $CDCl_3$) δ 169.7, 148.4, 143.6, 143.4, 139.9, 138.6, 137.1, 136.4, 134.5, 129.8, 128.7, 128.1, 127.7, 127.5, 127.2, 126.6, 122.6, 121.80, 121.75, 116.5, 48.6, 43.3, 42.1, 32.7, 27.7, 21.6. **IR** ν_{max} (film): 3323, 2925, 1682, 1597, 1527, 1486, 1452, 1424, 1386, 1326, 1242, 1158, 1093, 975, 826, 815, 792, 757, 700, 550 cm^{-1}. **HRMS** (ESI) m/z calcd for $C_{30}H_{31}N_3NaO_3S$ [M+Na]$^+$: 536.1978; found: 536.1971.

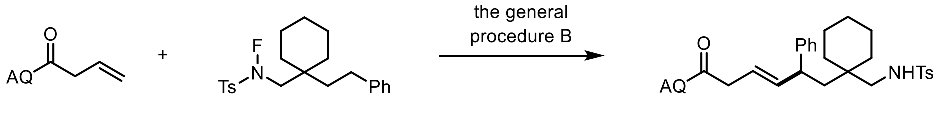

Product **3am** was prepared by the general procedure B. Purification using column chromatography (PE/EA = 5:1) afforded **3am** as yellow oil (75.6 mg, 0.13 mmol, 65%, *r.r.* > 20:1, *E/Z* > 20:1). ^{1}**H NMR** (600 MHz, $CDCl_3$) δ 9.92 (s, 1H), 8.76 - 8.73 (m, 2H), 8.15 (dd, J = 8.4, 1.8 Hz, 1H), 7.54 - 7.48 (m, 4H), 7.44 (dd, J = 7.8, 4.2 Hz, 1H), 7.28 - 7.25 (m, 4H), 7.21 - 7.17 (m, 3H), 5.94 (dd, J = 15.0, 7.2 Hz, 1H), 5.60 (dt, J = 15.0, 7.2 Hz, 1H), 4.20 (dd, J = 9.0, 6.0 Hz, 1H), 3.45 (dt, J = 7.2, 7.2 Hz, 1H), 3.21 (d, J = 7.2 Hz, 2H), 2.85 (dd, J = 13.2, 9.0 Hz, 1H), 2.49 (dd, J = 13.2, 6.0 Hz, 1H), 2.37 (s, 3H), 1.92 - 1.86 (m, 2H), 1.37 - 1.24 (m, 10H). ^{13}**C NMR** (150 MHz, $CDCl_3$) δ 169.7, 148.3, 145.3, 143.1, 141.8, 138.6, 137.0, 136.4, 134.5, 129.6, 129.1, 128.1, 127.8, 127.5, 127.1, 126.7, 121.73, 121.71, 121.6, 116.5, 48.9, 44.3, 42.2, 41.8, 37.0, 34.4, 34.2, 26.1, 21.6, 21.4, 21.3. **IR** ν_{max} (film): 3327, 3026, 2926, 2856, 1682, 1597, 1577, 1527, 1486, 1453, 1424, 1385, 1326, 1261, 1161, 1093, 1064, 977, 909, 826, 814, 757, 732, 702, 663, 567, 552 cm^{-1}. **HRMS** (ESI) m/z calcd for $C_{35}H_{39}N_3NaO_3S$ [M+Na]$^+$: 604.2604; found: 604.2603.

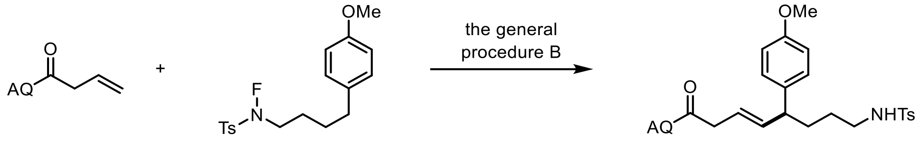

Product **3an** was prepared by the general procedure B. Purification using column chromatography (PE/EA = 5:1) afforded **3an** as yellow oil (65.2 mg, 0.12 mmol, 60%, *r.r.* > 20:1, *E/Z* = 5:1). ^{1}**H NMR** (600 MHz, $CDCl_3$) δ 9.98 (s, 0.83H), 9.89 (s, 0.17H), 8.76 - 8.71 (m, 2H), 8.16 - 8.13 (m, 1H), 7.68 (d, J = 8.4 Hz, 1.66H), 7.65 (d, J = 8.4 Hz, 0.34H), 7.54 - 7.49 (m, 2H), 7.46 - 7.42 (m, 1H), 7.24 - 7.21 (m, 2H), 7.11 (d, J = 8.4 Hz, 1.66H), 7.06 (d, J = 8.4 Hz, 0.34H), 6.81 (d, J = 8.4 Hz, 1.66H), 6.72 (d, J = 8.4 Hz, 0.34H), 5.86 - 5.81 (m, 1H), 5.79 - 5.64 (m, 1H), 4.73 (t, J = 6.6 Hz, 0.17H), 4.60 (t, J = 6.6 Hz, 0.83H), 3.77 (s, 2.49H), 3.69 (s, 0.51H), 3.26 -

3.24 (m, 3H), 2.94 - 2.88 (m, 2H), 2.39 (s, 2.49H), 2.38 (s. 0.51H), 1.83 - 1.75 (m, 1H), 1.74 - 1.67 (m, 1H), 1.55 - 1.47 (m, 1H), 1.46 - 1.38 (m, 1H). **^{13}C NMR** (150 MHz, $CDCl_3$) δ 169.8, 169.4, 158.3, 158.1, 148.4, 143.4, 143.3, 140.3, 138.7, 138.6, 137.2, 137.1, 136.5, 136.0, 135.6, 134.5, 134.4, 129.8, 129.7, 128.6, 128.2, 128.1, 128.0, 127.5, 127.2, 127.1, 122.2, 121.8, 121.7, 121.2, 116.7, 116.5, 114.12, 114.09, 55.4, 55.3, 47.6, 43.3, 43.1, 42.3, 42.1, 37.2, 33.7, 32.7, 27.7, 27.4, 21.6. **IR** ν_{max} (film): 3319, 2928, 2867, 1734, 1682, 1608, 1597, 1578, 1527, 1511, 1485, 1460, 1424, 1385, 1326, 1304, 1246, 1177, 1158, 1093, 1035, 975, 827, 792, 758, 550 cm^{-1}. **HRMS** (ESI) m/z calcd for $C_{31}H_{33}N_3NaO_4S$ $[M+Na]^+$: 566.2084; found: 566.2074.

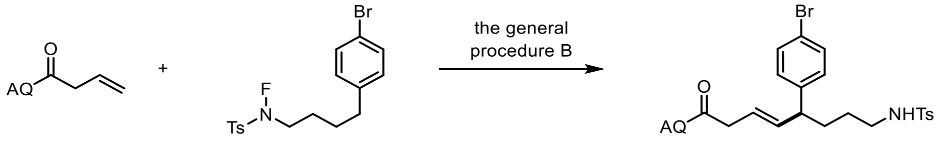

Product **3ao** was prepared by the general procedure B. Purification using column chromatography (PE/EA = 5:1) afforded **3ao** as yellow oil (82.8 mg, 0.14 mmol, 70%, *r.r.* > 20:1, *E/Z* > 20:1). **^{1}H NMR** (600 MHz, $CDCl_3$) δ 9.94 (s, 1H), 8.74 - 8.70 (m, 2H), 8.15 (dd, *J* = 8.4, 1.8 Hz, 1H), 7.68 (d, *J* = 8.4 Hz, 2H), 7.53 - 7.49 (m, 2H), 7.45 (dd, *J* = 8.4, 4.2 Hz, 1H), 7.37 (d, *J* = 8.4 Hz, 2H), 7.24 (d, *J* = 8.4 Hz, 2H), 7.06 (d, *J* = 8.4 Hz, 2H), 5.81 (dd, *J* = 15.6, 7.2 Hz, 1H), 5.71 - 5.67 (m, 1H), 4.75 (t, *J* = 6.6 Hz, 1H), 3.27 - 3.24 (m, 3H), 2.92 (dt, *J* = 6.6, 6.6 Hz, 2H), 2.39 (s, 3H), 1.81 - 1.76 (m, 1H), 1.73 - 1.69 (m, 1H), 1.54 - 1.47 (m, 1H), 1.44 - 1.37 (m, 1H). **^{13}C NMR** (150 MHz, $CDCl_3$) δ 169.6, 148.3, 143.5, 142.7, 139.2, 138.5, 137.1, 136.5, 134.4, 131.8, 129.8, 129.5, 128.1, 127.5, 127.2, 123.1, 121.84, 121.80, 120.3, 116.5, 47.9, 43.2, 42.0, 32.5, 27.6, 21.6. **IR** ν_{max} (film): 3329, 2954, 2920, 2852, 1650, 1595, 1526, 1485, 1457, 1424, 1378, 1335, 1322, 1305, 1245, 1160, 1069, 1010, 967, 903, 813, 790, 765, 730, 719, 705, 659, 571, 549, 519, 501 cm^{-1}. **HRMS** (ESI) m/z calcd for $C_{30}H_{30}BrN_3NaO_3S$ $[M+Na]^+$: 614.1083; found: 614.1078.

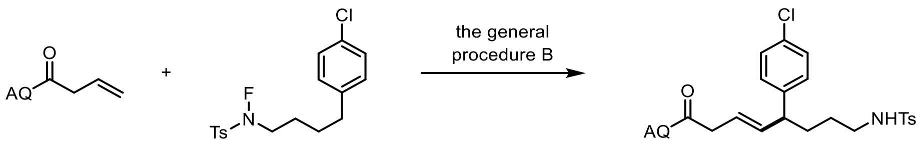

Product **3ap** was prepared by the general procedure B. Purification using column chromatography (PE/EA = 5:1) afforded **3ap** as yellow oil (79.9 mg, 0.15 mmol, 73%, *r.r.* > 20:1, *E/Z* > 20:1). **^{1}H NMR** (600 MHz, $CDCl_3$) δ 9.94 (s, 1H), 8.75 - 8.72 (m, 2H), 8.15 (dd, *J* = 8.4,

1.8 Hz, 1H), 7.68 (d, J = 7.8 Hz, 2H), 7.54 - 7.45 (m, 2H), 7.46 (dd, J = 7.8, 4.2 Hz, 1H), 7.25 -7.22 (m, 4H), 7.12 (d, J = 8.4 Hz, 2H), 5.82 (dd, J = 15.6, 7.8 Hz, 1H), 5.69 (dt, J = 15.6, 7.2 Hz, 1H), 4.62 (t, J = 6.6 Hz, 1H), 3.29 - 3.25 (m, 3H), 2.93 (dt, J = 6.6, 6.6 Hz, 2H), 2.39 (s, 3H), 1.82 - 1.77 (m, 1H), 1.75 - 1.71 (m, 1H), 1.54 - 1.49 (m, 1H), 1.44 - 1.39 (m, 1H). **^{13}C NMR** (150 MHz, $CDCl_3$) δ 169.6, 148.3, 143.5, 142.1, 139.3, 138.6, 137.1, 136.5, 134.4, 132.3, 129.8, 129.1, 128.8, 128.1, 127.5, 127.2, 123.0, 121.84, 121.81, 116.5, 47.9, 43.2, 42.0, 32.6, 27.6, 21.6. **IR** ν_{max} (film): 1679, 1527, 1486, 1424, 1386, 1326, 1245, 1158, 1092, 826, 792, 550 cm^{-1}. **HRMS** (ESI) m/z calcd for $C_{30}H_{30}ClN_3NaO_3S$ $[M+Na]^+$: 570.1589; found: 570.1585.

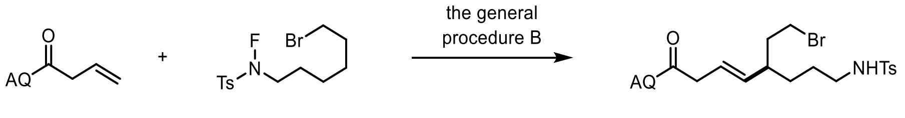

1a **2aq** **3aq**

Product **3aq** was prepared by the general procedure B. Purification using column chromatography (PE/EA = 5:1) afforded **3aq** as yellow oil (71.7 mg, 0.13 mmol, 66%, *r.r.* > 20:1, *E/Z* > 20:1). **^{1}H NMR** (600 MHz, $CDCl_3$) δ 9.92 (s, 1H), 8.81 - 8.75 (m, 2H), 8.17 - 8.15 (m, 1H), 7.72 - 7.69 (m, 2H), 7.55 - 7.49 (m, 2H), 7.47 - 7.44 (m, 1H), 7.28 - 7.26 (m, 2H), 5.76 - 5.70 (m, 1H), 5.43 - 5.38 (m, 1H), 4.71 - 4.68 (m, 1H), 3.51 - 3.47 (m, 1H), 3.43 - 3.38 (m, 1H), 3.31 - 3.23 (m, 2H), 2.96 - 2.91 (m, 2H), 2.41 (s, 3H), 2.29 - 2.23 (m, 1H), 1.93 - 1.81 (m, 2H), 1.59 - 1.41 (m, 3H), 1.37 - 1.30 (m, 1H). **^{13}C NMR** (150 MHz, $CDCl_3$) δ 169.6, 148.5, 143.5, 138.7, 138.5, 137.1, 136.5, 134.4, 129.8, 128.1, 127.5, 127.2, 124.6, 121.9, 121.8, 116.6, 43.2, 42.1, 41.4, 38.0, 31.9, 31.7, 27.2, 21.7. **IR** ν_{max} (film): 3331, 2925, 1770, 1682, 1596, 1527, 1486, 1457, 1424, 1385, 1326, 1247, 1158, 1093, 977, 826, 792, 550, cm^{-1}. **HRMS** (ESI) m/z calcd for $C_{26}H_{30}BrN_3NaO_3S$ $[M+Na]^+$: 566.1083; found: 566.1091.

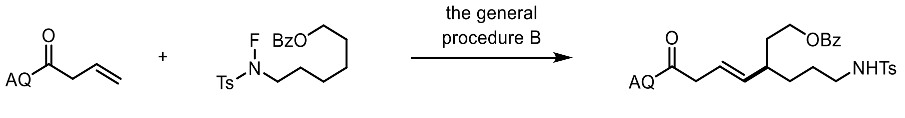

1a **2ar** **3ar**

Product **3ar** was prepared by the general procedure B. Purification using column chromatography (PE/EA = 3:1) afforded **3ar** as yellow oil (79.6 mg, 0.14 mmol, 68%, *r.r.* > 20:1, *E/Z* > 20:1). **^{1}H NMR** (600 MHz, $CDCl_3$) δ 9.94 (s, 1H), 8.77 - 8.75 (m, 2H), 8.15 (d, J = 8.4 Hz, 1H), 8.01 (d, J = 7.8 Hz, 2H), 7.69 (d, J = 7.8 Hz, 2H), 7.56 - 7.50 (m, 3H), 7.44 - 7.42 (m, 3H), 7.25 (d, J = 8.4 Hz, 2H), 5.71 (dt, J = 15.0, 7.2 Hz, 1H), 5.52 (dd, J = 15.0, 9.0 Hz, 1H), 4.51 (t, J = 6.6 Hz, 1H), 4.44 - 4.40 (m, 1H), 4.35 - 4.31 (m, 1H), 3.27 (d, J = 7.2 Hz, 2H), 2.93 (dt, J = 6.6, 6.6 Hz, 2H), 2.39 (s, 3H), 2.30 - 2.26 (m, 1H), 1.91 - 1.86 (m, 1H), 1.81 - 1.75 (m, 1H), 1.59 -

1.56 (m, 1H), 1.55 - 1.51 (m, 2H), 1.42 - 1.38 (m, 1H), **^{13}C NMR** (150 MHz, $CDCl_3$) δ 169.7, 166.7, 148.4, 143.4, 139.3, 138.5, 137.1, 136.5, 134.4, 133.1, 130.4, 129.8, 129.7, 128.5, 128.1, 127.5, 127.2, 124.0, 121.83, 121.81, 116.6, 63.2, 43.3, 42.1, 39.8, 34.2, 31.9, 27.3, 21.7. **IR** ν_{max} (film): 3445, 2924, 1769, 1758, 1713, 1679, 1525, 1484, 1451, 1424, 1379, 1324, 1246, 1156, 1093, 1063, 825, 791, 711, 549 cm^{-1}. **HRMS** (ESI) m/z calcd for $C_{33}H_{35}N_3NaO_5S$ $[M+Na]^+$: 608.2190; found: 608.2185.

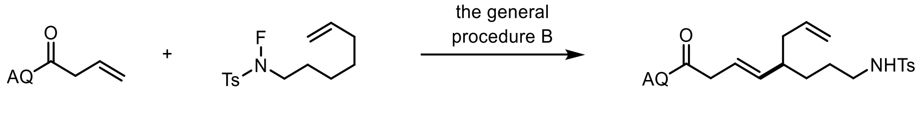

1a **2as** **3as**

Product **3as** was prepared by the general procedure B. Purification using column chromatography (PE/EA = 5:1) afforded **3as** as yellow oil (62.0 mg, 0.13 mmol, 65%, *r.r.* > 20:1, *E/Z* = 4:1). **^{1}H NMR** (600 MHz, $CDCl_3$) δ 9.97 (s, 0.8H), 9.92 (s, 0.2H), 8.78 - 8.75 (m, 2H), 8.17 - 8.14 (m, 1H), 7.75 -7.69 (m, 2H), 7.54 - 7.49 (m, 2H), 7.46 - 7.43 (m, 1H), 7.28 - 7.25 (m, 2H), 5.82 - 5.76 (m, 1H), 5.75 - 5.72 (m, 0.2H), 5.64 (dt, *J* = 15.6, 7.2 Hz, 0.8H), 5.51 (dd, *J* = 15.6, 7.8 Hz, 0.8H), 5.45 - 5.41 (m, 0.2H), 5.07 - 4.92 (m, 2H), 4.82 - 4.75 (m, 1H), 3.29 - 3.24 (m, 2H), 2.93 - 2.89 (m, 2H), 2.39 (s, 2.4H), 2.38 (s, 0.6H), 2.17 - 2.11 (m, 2H), 1.59 - 1.52(m, 1H), 1.49 - 1.44 (m, 2H), 1.37 - 1.22 (m, 2H). **^{13}C NMR** (150 MHz, $CDCl_3$) δ 169.9, 148.3, 143.4, 140.2, 138.5, 137.1, 136.52, 136.45, 134.4, 129.7, 128.0, 127.5, 127.2, 122.9, 121.8, 121.7, 116.5, 116.4, 43.4, 42.3, 42.2, 39.7, 31.0, 27.3, 21.6. **IR** ν_{max} (film): 3320, 2924, 1679, 1597, 1577, 1528, 1486, 1425, 1385, 1326, 1242, 1159, 1093, 976, 912, 826, 815, 792, 737, 661, 551 cm^{-1}. **HRMS** (ESI) m/z calcd for $C_{27}H_{31}N_3NaO_3S$ $[M+Na]^+$: 500.1978; found: 500.1975.

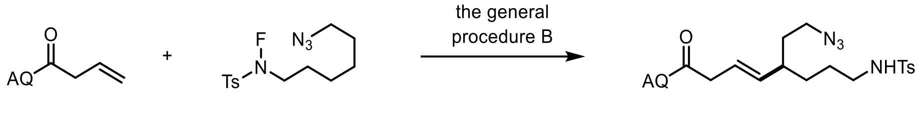

1a **2at** **3at**

Product **3at** was prepared by the general procedure B. Purification using column chromatography (PE/EA = 3:1) afforded **3at** as yellow oil (56.7 mg, 0.11 mmol, 56%, *r.r.* > 20:1, *E/Z* > 20:1). **^{1}H NMR** (600 MHz, $CDCl_3$) δ 9.90 (s, 1H), 8.79 - 8.75 (m, 2H), 8.16 (d, *J* = 8.4, 1.8 Hz, 1H), 7.70 (d, *J* = 8.4 Hz, 2H), 7.55 - 7.51 (m, 2H), 7.46 (dd, *J* = 8.4, 4.2 Hz, 1H), 7.27 (d, *J* = 8.4 Hz, 2H), 5.69 (dt, *J* = 15.6, 7.2 Hz, 1H), 5.42 (dd, *J* = 15.6, 9.0 Hz, 1H), 4.59 (t, *J* = 6.6 Hz, 1H), 3.38 - 3.34 (m, 1H), 3.32 - 3.27 (m, 3H), 2.95 - 2.91 (m, 2H), 2.41 (s, 3H), 2.19 - 2.13 (m, 1H), 1.71 - 1.67 (m, 1H), 1.59 - 1.53 (m, 2H), 1.51 - 1.42 (m, 2H), 1.36 - 1.32 (m, 1H). **^{13}C NMR** (150 MHz, $CDCl_3$) δ 169.6, 148.4, 143.5, 138.9, 138.6, 137.2, 136.6, 134.4, 129.8, 128.1, 127.5,

127.2, 124.5, 121.87, 121.86, 116.7, 49.5, 43.3, 42.1, 40.3, 34.3, 32.0, 27.3, 21.6. **IR** ν_{max} (film): 3325, 2924, 2853, 2093, 1683, 1596, 1525, 1486, 1456, 1424, 1384, 1325, 1260, 1158, 1092, 976, 826, 814, 792, 757, 730, 704, 660, 550 cm^{-1}. **HRMS** (ESI) m/z calcd for $C_{26}H_{30}N_6NaO_3S$ $[M+Na]^+$: 529.1992; found: 529.1989.

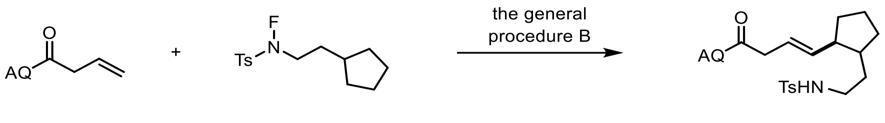

1a **2au** **3au**

Product **3au** was prepared by the general procedure B. Purification using column chromatography (PE/EA = 5:1) afforded **3au** as yellow oil (63.9 mg, 0.13 mmol, 67%, *r.r.* > 20:1, *E/Z* = 1:1, *d.r.* = 2:1). **^{1}H NMR** (600 MHz, $CDCl_3$) δ 10.01 (s, 0.34H), 9.97 (s, 0.34H), 9.96 (s, 0.16H), 9.95 (s, 0.16H), 8.79 - 8.75 (m, 2H), 8.15 - 8.13 (m, 1H), 7.71 - 7.69 (m, 0.68H), 7.67 - 7.64 (m, 1.32H), 7.54 - 7.49 (m, 2H), 7.45 - 7.42 (m, 1H), 7.23 (d, *J* = 7.8 Hz, 1.66H), 7.20 (d, *J* = 7.8 Hz, 0.34H), 5.72 - 5,63 (m, 1H), 5.62 - 5.54 (m, 1H), 5.02 - 4.97 (m, 0.5H), 4.97 - 4.77 (m, 0.5H), 3.32 - 3.19 (m, 2H), 2.99 - 2.84 (m, 2H), 2.38 (s, 2H), 2.36 (s, 1H), 2.35 - 2.03 (m, 1H), 1.93 - 1.79 (m, 2H), 1.76 - 1.59 (m, 3H), 1.54 - 1.35 (m, 2H), 1.32 - 1.09 (m, 2H). **^{13}C NMR** (150 MHz, $CDCl_3$) δ 170.1, 169.8, 169.7, 148.39, 148.36, 148.3, 143.3, 143.20, 143.18, 140.9, 139.4, 138.50, 138.46, 138.4, 137.1, 137.03, 137.01, 136.42, 136.38, 136.2, 134.4, 129.7, 129.63, 129.61, 128.0, 127.41, 127.39, 127.37, 127.11, 127.08, 127.06, 121.9, 121.77, 121.75, 121.69, 121.3, 121.1, 116.52, 116.46, 116.41, 50.2, 45.6, 45.0, 44.3, 43.2, 42.6, 42.5, 42.41, 42.38, 42.1, 42.0, 41.2, 41.0, 40.1, 37.2, 36.9, 34.3, 33.9, 33.1, 33.0, 32.5, 32.1, 31.7, 31.6, 31.2, 30.9, 30.5, 30.4, 23.8, 23.7, 23.1, 23.0, 21.6, 21.5. **IR** ν_{max} (film): 3314, 2950, 2868, 1679, 1597, 1577, 1527, 1486, 1457, 1424, 1385, 1327, 1261, 1159, 1093, 975, 912, 826, 814, 735, 551, 506 cm^{-1}. **HRMS** (ESI) m/z calcd for $C_{27}H_{31}N_3NaO_3S$ $[M+Na]^+$: 500.1978; found: 500.1967.

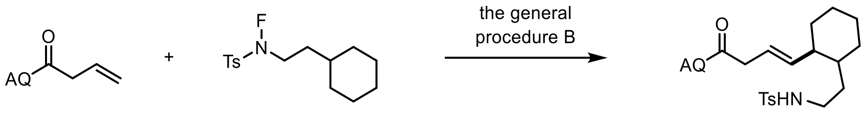

1a **2av** **3av**

Product **3av** was prepared by the general procedure B. Purification using column chromatography (PE/EA = 5:1) afforded **3av** as yellow oil (74.7 mg, 0.15 mmol, 76%, *r.r.* > 20:1, *E/Z* > 20:1, *d.r.* = 1:1). **^{1}H NMR** (600 MHz, $CDCl_3$) δ 9.96 (s, 0.5H), 9.95 (s, 0.5H), 8.78 - 8.73 (m, 2H), 8.17 - 8.15 (m, 1H), 7.68 - 7.67 (m, 2H), 7.55 - 7.49 (m, 2H), 7.46 - 7.43 (m, 1H), 7.24 - 7.23 (m, 2H), 5.89 (dd, *J* = 15.6, 3.0 Hz, 0.5H), 5.63 - 5.59 (m, 0.5H), 5.58 - 5.56 (m, 0.5H), 5.48

(dd, J = 15.6, 9.0 Hz, 0.5H), 4.82 (t, J = 6.0 Hz, 0.5H), 4.74 (t, J = 6.0 Hz, 0.5H), 3.26 - 3.24 (m, 1H), 3.23 - 3.21 (m, 1H), 2.98 - 2.93 (m, 1H), 2.92 - 2.86 (m, 1H), 2.39 (s, 3H), 1.84 - 1.68 (m, 4H), 1.62 - 1.55 (m, 2H), 1.49 - 1.44 (m, 1H), 1.41 - 1.37 (m, 1H), 1.31 - 1.25 (m, 2H), 1.22 - 1.14 (m, 2H). **^{13}C NMR** (150 MHz, $CDCl_3$) δ 170.2, 170.1, 148.30, 148.27, 143.3, 143.2, 141.7, 138.52, 138.51, 137.03, 137.00, 136.51, 136.49, 134.4, 129.68, 129.65, 128.1, 127.5, 127.2, 123.2, 122.2, 121.79, 121.77, 121.74, 116.6, 116.5, 47.4, 42.4, 42.0, 41.9, 41.3, 41.1, 39.0, 36.9, 34.2, 33.8, 31.6, 28.5, 26.2, 25.9, 21.6. **IR** ν_{max} (film): 3317, 2924, 2853, 1680, 1596, 1527, 1486, 1447, 1424, 1385, 1326, 1260, 1159, 1093, 976, 910, 826, 815, 792, 756, 731, 580, 551 cm^{-1}. **HRMS** (ESI) m/z calcd for $C_{28}H_{33}N_3NaO_3S$ $[M+Na]^+$: 514.2135; found: 514.2136.

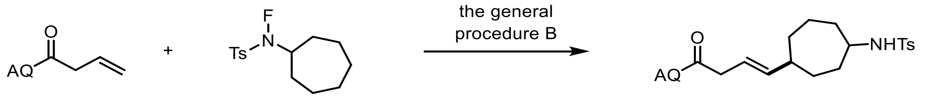

1a 2aw 3aw

Product **3aw** was prepared by the general procedure B. Purification using column chromatography (PE/EA = 5:1) afforded **3aw** as yellow oil (62.0 mg, 0.13 mmol, 65%, *r.r.* > 20:1, *E/Z* = 10:1, *d.r.* = 1:1). **^{1}H NMR** (600 MHz, $CDCl_3$) δ 10.06 (s, 1H), 8.77 - 8.70 (m, 2H), 8.16 - 8.14 (m, 1H), 7.76 (d, J = 7.8 Hz, 2H), 7.53 - 7.48 (m, 2H), 7.44 (dd, J = 7.8, 4.2 Hz, 1H), 7.28 (d, J = 7.8 Hz, 2H), 5.76 - 5.71 (m, 1H), 5.67 - 5.62 (m, 1H), 4.86 (d, J = 7.8 Hz, 0.5H), 4.73 (d, J = 7.8 Hz, 0.5H), 3.43 - 3.39 (m, 0.5H), 3.37 - 3.34 (m, 0.5H), 3.23 (dd, J = 7.2, 3.0 Hz, 2H), 2.41 (s, 3H), 2.30 - 2.24 (m, 1H), 1.95 - 1.86 (m, 2H), 1.85 - 1.75 (m, 2H), 1.74 - 1.66 (m, 2H), 1.62 - 1.55 (m, 1H), 1.51 - 1.46 (m, 1H), 1.37 - 1.35 (m, 1H), 1.31 - 1.25 (m, 1H). **^{13}C NMR** (125 MHz, $CDCl_3$) δ 170.0, 148.24, 148.19, 143.3, 142.8, 138.6, 138.31, 138.27, 136.4, 134.5, 129.8, 128.0, 127.5, 127.1, 121.8, 121.72, 121.65, 120.21, 120.17, 116.4, 55.1, 54.3, 42.9, 42.3, 42.2, 36.6, 36.0, 35.2, 34.4, 33.9, 32.7, 30.3, 28.6, 22.9, 21.6, 21.5. **IR** ν_{max} (film): 3375, 2928, 2857, 1742, 1670, 1597, 1528, 1486, 1425, 1374, 1327, 1242, 1159, 1095, 1047, 940, 913, 814, 793, 705, 569, 551 cm^{-1}. **HRMS** (ESI) m/z calcd for $C_{27}H_{31}N_3NaO_3S$ $[M+Na]^+$: 500.1978; found: 500.1974.

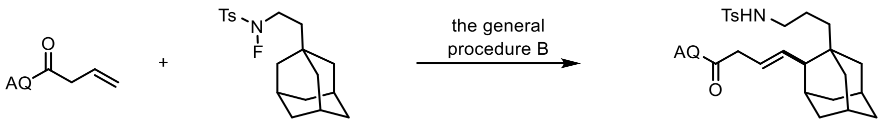

1a 2ax 3ax

Product **3ax** was prepared by the general procedure B. Purification using column chromatography (PE/EA = 5:1) afforded **3ax** as yellow oil (83.7 mg, 0.15 mmol, 77%, *r.r.* > 20:1, *E/Z* = 5:1). **^{1}H NMR** (600 MHz, $CDCl_3$) δ 9.91 (s, 0.16H), 9.88 (s, 0.84H), 8.82 - 8.76 (m, 2H),

8.19 - 8.17 (m, 1H), 7.63 (d, *J* = 7.8 Hz, 1.68H),7.59 - 7.52 (m, 2.32H), 7.49 - 7.46 (m, 1H), 7.23 - 7.18 (m, 2H), 6.07 - 6.03 (m, 0.16H), 5.96 (dd, *J* = 15.0, 10.2 Hz, 0.84H), 5.76 - 5.72 (m, 0.16H), 5.46 (dt, *J* = 15.0, 7.2 Hz, 0.84H), 4.78 - 4.76 (m, 0.84H), 4.46 - 4.40 (m, 0.16H), 3.27 - 3.19 (m, 2H), 2.99 - 2.93 (m, 1H), 2.91 - 2.87 (m, 0.84H), 2.84 - 2.79 (m, 0.16H), 2.50 - 2.48 (m, 0.16H), 2.39 (s, 2.5H), 2.36 (s, 0.5H), 2.24 - 2.22 (m, 0.84H), 1.97 - 1.88 (m, 4H), 1.80 - 1.77 (m, 1H), 1.73 - 1.69 (m, 3H), 1.64 - 1.61 (m, 1H), 1.57 - 1.53 (m, 2H), 1.49 - 1.39 (m, 2H), 1.28 - 1.22 (m, 2H). **^{13}C NMR** (150 MHz, $CDCl_3$) δ 170.1, 169.8, 148.6, 148.4, 143.3, 143.2, 138.5, 137.6, 137.1, 136.69, 136.65, 136.0, 134.4, 129.7, 129.6, 128.2, 127.7, 127.6, 127.2, 127.1, 123.7, 122.1, 121.91, 121.88, 121.85, 116.8, 116.7, 51.3, 48.5, 45.6, 42.44, 42.38, 42.2, 40.9, 40.8, 38.8, 38.6, 38.5, 38.4, 37.9, 37.8, 37.7, 37.6, 36.9, 34.9, 34.7, 34.3, 34.1, 31.9, 31.7, 28.62, 28.55, 28.5, 21.63, 21.60. **IR** ν_{max} (film): 3446, 2914, 1770, 1683, 1525, 1486, 1377, 1326, 1246, 1158, 791, 550 cm^{-1}. **HRMS** (ESI) m/z calcd for $C_{32}H_{37}N_3NaO_3S$ $[M+Na]^+$: 566.2448; found: 566.2453.

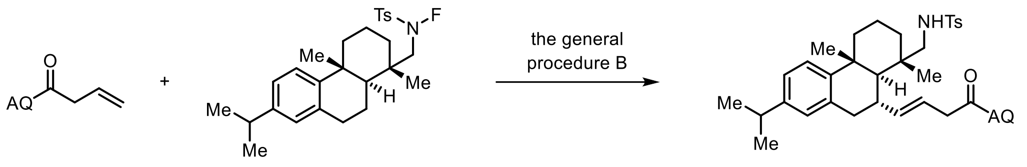

1a **2ay** **3ay**

Product **3ay** was prepared by the general procedure B. Purification using column chromatography (PE/EA = 5:1) afforded **3ay** as yellow oil (75.3 mg, 0.12 mmol, 58%, *r.r.* > 20:1, *E/Z* > 20:1, *d.r.* > 20:1). **^{1}H NMR** (600 MHz, $CDCl_3$) δ 9.90 (s, 1H), 8.84 (dd, *J* = 4.2, 1.2 Hz, 1H), 8.79 (dd, *J* = 7.2, 1.2 Hz, 1H), 8.19 (d, *J* = 8.4, 1.8 Hz, 1H), 7.67 (d, *J* = 8.4 Hz, 2H), 7.57 - 7.52 (m, 2H), 7.48 (dd, *J* = 7.8, 4.2 Hz, 1H), 7.19 (d, *J* = 8.4 Hz, 2H), 7.12 (d, *J* = 8.4 Hz, 1H), 7.01 (dd, *J* = 7.8, 1.8 Hz, 1H), 6.85 (s, 1H), 5,72 (dd, *J* = 15.6, 9.6 Hz, 1H), 5.67 - 5.62 (m, 1H), 4.64 (t, *J* = 6.6 Hz, 1H), 3.28 (dd, *J* = 16.2, 7.8 Hz, 1H), 3.11 (dd, *J* = 16.2, 7.8 Hz, 1H), 3.06 (dd, *J* = 16.2, 6.6 Hz, 1H), 2.98 (dd, *J* = 13.2, 6.6 Hz, 1H), 2.91 - 2.86 (m, 1H), 2.80 - 2.74 (m, 2H), 2.68 (dd, *J* = 13.2, 8.4 Hz, 1H), 2.33 (s, 3H), 2.23 - 2.20 (m, 1H), 1.79 - 1.74 (m, 1H), 1.70 - 1.67 (m, 1H), 1.54 - 1.46 (m, 2H), 1.43 (d, *J* = 9.0 Hz, 1H), 1.36 - 1.33 (m, 1H), 1.28 (s, 3H), 1.18 (d, *J* = 4.8 Hz, 3H), 1.17 (d, *J* = 4.8 Hz, 3H), 1.10 (s, 3H). **^{13}C NMR** (150 MHz, $CDCl_3$) δ 169.7, 148.3, 147.2, 146.1, 144.2, 143.4, 138.6, 137.4, 136.6, 134.5, 134.4, 129.7, 128.1, 127.6, 127.1, 126.4, 124.3, 122.7, 121.8, 120.9, 116.7, 54.5, 48.7, 41.4, 38.8, 38.7, 38.5, 38.1, 37.5, 37.3, 33.6, 24.2, 24.1, 24.0, 21.6, 19.4, 18.3. **IR** ν_{max} (film): 3330, 2956, 2926, 2868, 1683, 1596, 1526, 1486, 1458, 1424, 1385, 1326, 1261, 1161, 1093, 1063, 910, 825, 792, 757, 732, 662, 552 cm^{-1}. **HRMS** (ESI) m/z calcd for $C_{40}H_{47}N_3NaO_3S$ $[M+Na]^+$: 672.3230; found: 672.3221.

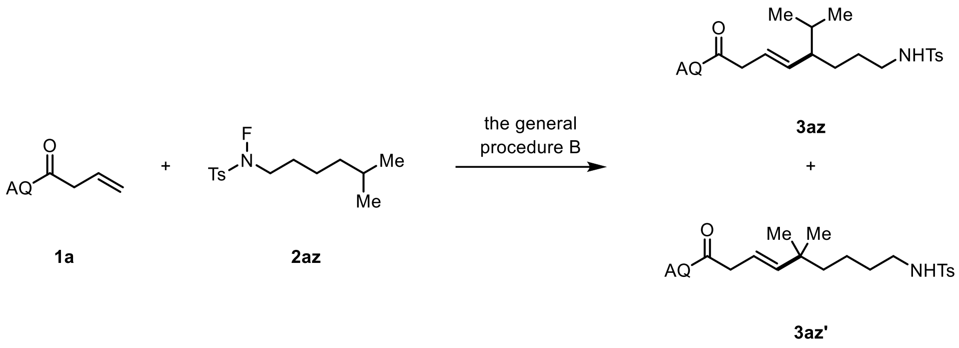

Product **3az** and **3az′** was prepared by the general procedure B. Purification using column chromatography (PE/EA = 5:1) afforded a mixture of **3az** and **3az′** as yellow oil (64.2 mg, 0.13 mmol, 67%, *r.r.* > 20:1, *E/Z* > 20:1, **3az**:**3az′** = 1:1). **^{1}H NMR** (600 MHz, $CDCl_3$) δ 10.08 (s, 0.5H), 9.95 (s, 0.5H), 8.78 - 8.73 (m, 2H), 8.16 - 8.14 (m, 1H), 7.72 - 7.69 (m, 2H), 7.54 - 7.49 (m, 2H), 7.46 - 7.43 (m, 1H), 7.27 - 7.25 (m, 2H), 5.70 (d, *J* = 15.6, 0.5H), 5.62 - 5.59 (m, 0.5H), 5.58 - 5.57 (m, 0.5H), 5.48 (dd, *J* = 15.6, 9.0 Hz, 0.5H), 4.74 - 4.69 (m, 1H), 3.30 - 3.23 (m, 2H), 2.94 - 2.87 (m, 2H), 2.39 (s, 3H), 1.84 - 1.79 (m, 0.5H), 1.62 - 1.54 (m, 1H), 1.46 - 1.40 (m, 2H), 1.29 - 1.24 (m, 2.5H), 1.06 (s, 3H), 0.90 (d, *J* = 7.2 Hz, 1.5H), 0.86 (d, *J* = 7.2 Hz, 1.5H). **^{13}C NMR** (125 MHz, $CDCl_3$) δ 170.3, 170.1, 148.3, 148.2, 146.8, 143.3, 138.63, 138.60, 138.5, 137.2, 137.1, 136.5, 136.4, 134.5, 129.8, 129.7, 128.1, 127.52, 127.47, 127.19, 127.17, 127.14, 123.7, 121.79, 121.73, 121.65, 118.7, 116.6, 116.5, 49.2, 43.4, 43.2, 42.39, 42.35, 42.32, 36.4, 32.0, 30.4, 29.0, 27.8, 27.1, 21.8, 21.6, 20.7, 19.1. **IR** ν_{max} (film): 3282, 2956, 2869, 1742, 1670, 1597, 1527, 1486, 1458, 1425, 1374, 1326, 1242, 1160, 1094, 1047, 978, 913, 827, 814, 793, 742, 607, 551 cm^{-1}. **HRMS** (ESI) m/z calcd for $C_{27}H_{33}N_3NaO_3S$ $[M+Na]^+$: 502.2135; found: 502.2137.

The general procedure C (for products 6a-6aa):

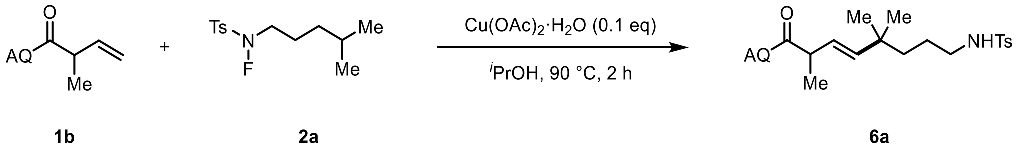

To a dry Schlenk flask were added **1b** (45.2 mg, 0.20 mmol, 1.0 equiv), **2a** (137 mg, 0.50 mmol, 2.5 equiv), $Cu(OAc)_2\cdot H_2O$ (4.0 mg, 0.02 mmol, 0.10 equiv), and anhydrous ^{i}PrOH (3.0 mL). The mixture was degassed for three times with argon and stirred at 90 °C (oil bath) for 2 h. Once completion, the reaction was cooled to room temperature. The reaction mixture was filtered by celite, and the filtrate was concentrated *in vacuo*. Further purification by a flash column

chromatography using eluents (PE/EA = 5:1) afforded the desired product **6a** as yellow oil (80.5 mg, 0.17 mmol, 84%, *r.r.* > 20:1, *E/Z* > 20:1). **^{1}H NMR** (500 MHz, $CDCl_3$) δ 10.09 (s, 1H), 8.77 - 8.74 (m, 2H), 8.14 (dd, *J* = 8.0, 1.5 Hz, 1H), 7.69 (d, *J* = 8.5 Hz, 2H), 7.54 - 7.48 (m, 2H), 7.44 (dd, *J* = 8.0, 4.0 Hz, 1H), 7.25 (d, *J* = 8.5 Hz, 2H), 5.68 (d, *J* = 16.0 Hz, 1H), 5.53 (dd, *J* = 16.0, 8.5 Hz, 1H), 4.62 (t, *J* = 6.0 Hz, 1H), 3.28 - 3.22 (m, 1H), 2.88 (dt, *J* = 6.0, 6.0 Hz, 2H), 2.39 (s, 3H), 1.46 - 1.41 (m, 2H), 1.38 (d, *J* = 7.5 Hz, 3H), 1.33 - 1.30 (m, 2H), 1.05 (s, 3H), 1.03 (s, 3H). **^{13}C NMR** (125 MHz, $CDCl_3$) δ 173.3, 148.2, 143.5, 143.4, 138.7, 137.1, 136.4, 134.7, 129.7, 128.1, 127.5, 127.2, 126.3, 121.8, 121.6, 116.3, 46.1, 44.0, 39.7, 35.9, 27.3, 27.0, 25.0, 21.6, 17.4. **IR** ν_{max} (film): 3501, 3320, 2960, 2870, 1677, 1597, 1577, 1526, 1485, 1424, 1385, 1326, 1260, 1240, 1159, 1094, 1019, 978, 940, 894, 826, 815, 792, 756, 661, 575, 550, 494 cm^{-1}. **HRMS** (ESI) m/z calcd for $C_{27}H_{33}N_3NaO_3S$ $[M+Na]^+$: 502.2135; found: 502.2125.

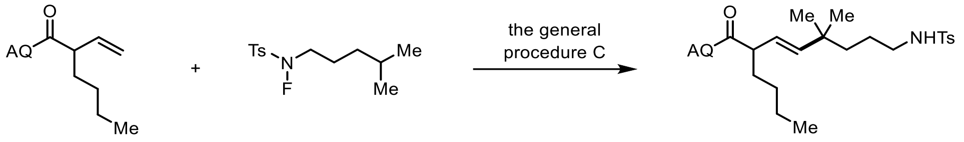

1c **2a** **6b**

Product **6b** was prepared by the general procedure C. Purification using column chromatography (PE/EA = 5:1) afforded **6b** as yellow oil (91.7 mg, 0.18 mmol, 88%, *r.r.* > 20:1, *E/Z* > 20:1). **^{1}H NMR** (600 MHz, $CDCl_3$) δ 10.05 (s, 1H), 8.78 - 8.75 (m, 2H), 8.13 - 8.11 (m, 1H), 7.70 (d, *J* = 8.4 Hz, 2H), 7.51 - 7.46 (m, 2H), 7.44 - 7.41 (m, 1H), 7.25 - 7.22 (m, 2H), 5.64 (d, *J* = 15.6 Hz, 1H), 5.46 (dd, *J* = 15.6, 9.0 Hz, 1H), 5.00 - 4.88 (m, 1H), 3.09 - 3.06 (m, 1H), 2.87 (dt, *J* = 6.6, 6.6 Hz, 2H), 2.38 (s, 3H), 2.03 - 1.97 (m, 1H), 1.64 - 1.58 (m, 1H), 1.45 - 1.40 (m, 2H), 1.36 - 1.29 (m, 6H), 1.02 (s, 3H), 1.01 (s, 3H), 0.90 (t, *J* = 5.4 Hz, 3H). **^{13}C NMR** (150 MHz, $CDCl_3$) δ 172.9, 148.2, 144.3, 143.2, 138.5, 137.1, 136.3, 134.6, 129.6, 127.9, 127.3, 127.1, 125.0, 121.7, 121.4, 116.2, 52.1, 43.9, 39.6, 35.9, 31.5, 29.5, 27.2, 26.9, 24.9, 22.5, 21.5, 14.0. **IR** νmax (film): 3500, 2956, 2870, 1770, 1682, 1577, 1485, 1424, 1325, 1260, 1094, 792, 576, 550 cm^{-1}. **HRMS** (ESI) m/z calcd for $C_{30}H_{39}N_3NaO_3S$ $[M+Na]^+$: 544.2604; found: 544.26595.

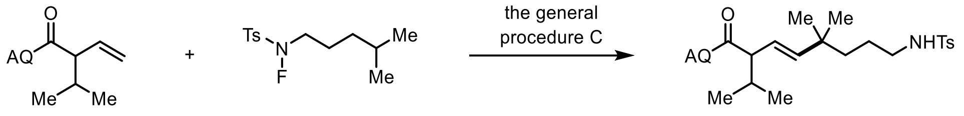

1d **2a** **6c**

Product **6c** was prepared by the general procedure C. Purification using column chromatography (PE/EA = 5:1) afforded **6c** as yellow oil (71.0 mg, 0.14 mmol, 70%, *r.r.* > 20:1,

E/Z > 20:1). **¹H NMR** (600 MHz, $CDCl_3$) δ 9.96 (s, 1H), 8.79 - 8.78 (m, 2H), 8.15 (dd, J = 8.4, 1.8 Hz, 1H), 7.68 (d, J = 8.4 Hz, 2H), 7.54 - 7.49 (m, 2H), 7.45 (dd, J = 8.4, 4.2 Hz, 1H), 7.26 (d, J = 8.4 Hz, 2H), 5.59 (d, J = 15.6 Hz, 1H), 5.52 (dd, J = 15.6, 9.0 Hz, 1H), 4.42 - 4.39 (m, 1H), 2.87 - 2.81 (m, 3H), 2.39 (s, 3H), 2.35 - 2.29 (m, 1H), 1.42 - 1.37 (m, 2H), 1.29 - 1.26 (m, 2H), 1.02 (s, 3H), 1.01 (s, 3H), 0.99 (d, J = 6.6 Hz, 3H), 0.93 (d, J = 6.6 Hz, 3H). **¹³C NMR** (150 MHz, $CDCl_3$) δ 172.8, 148.3, 145.1, 143.4, 138.7, 137.2, 136.5, 134.7, 129.8, 128.1, 127.5, 127.2, 123.2, 121.8, 121.5, 116.4, 59.7, 44.0, 39.8, 36.2, 30.1, 27.3, 27.2, 25.1, 21.6, 21.3, 19.5. **IR** ν_{max} (film): 3271, 2958, 2869, 2359, 1682, 1596, 1576, 1525, 1485, 1425, 1385, 1325, 1261, 1159, 1094, 979, 826, 814, 792, 757, 661, 550 cm^{-1}. **HRMS** (ESI) m/z calcd for $C_{29}H_{37}N_3NaO_3S$ $[M+Na]^+$: 530.2448; found: 530.2438.

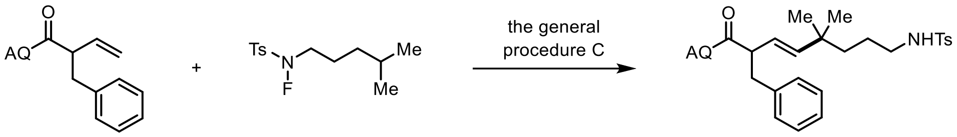

1e **2a** **6d**

Product **6d** was prepared by the general procedure C. Purification using column chromatography (PE/EA = 5:1) afforded **6d** as yellow solid (94.4 mg, 0.17 mmol, 85%, *r.r.* > 20:1, *E/Z* > 20:1). **¹H NMR** (600 MHz, $CDCl_3$) δ 10.10 (s, 1H), 8.78 (dd, J = 7.8, 1.8 Hz, 1H), 8.73 (dd, J = 4.2, 1.8 Hz, 1H), 8.14 (dd, J = 7.8, 1.2 Hz, 1H), 7.69 (d, J = 7.8 Hz, 2H), 7.55 - 7.49 (m, 2H), 7.43 (dd, J = 8.4, 4.2 Hz, 1H), 7.28 (d, J = 7.8 Hz, 2H), 7.25 - 7.22 (m, 2H), 7.19 (d, J = 7.2 Hz, 2H), 7.12 (t, J = 7.2 Hz, 1H), 5.46 - 5.40 (m, 2H), 4.18 - 4.17 (m, 1H), 3.45 (dd, J = 13.8, 5.4 Hz, 1H), 3.42 - 3.39 (m, 1H), 2.86 (dd, J = 13.8, 9.0 Hz, 1H), 2.73 (dt, J = 6.6, 6.6 Hz, 2H), 2.41 (s, 3H), 1.17 - 1.11 (m, 3H), 0.98 (s, 3H), 0.97 - 0.93 (m, 1H), 0.92 (s, 3H). **¹³C NMR** (150 MHz, $CDCl_3$) δ 166.4, 142.7, 139.9, 137.9, 134.3, 133.1, 131.6, 130.9, 129.0, 124.2, 124.0, 122.8, 122.5, 122.0, 121.6, 120.6, 118.8, 116.2, 116.1, 110.8, 48.5, 38.3, 34.1, 32.4, 30.6, 22.4, 20.6, 19.2, 16.1. **IR** ν_{max} (film): 3502, 2955, 2925, 2853, 1697, 1683, 1770, 1758, 1734, 1670, 1653, 1647, 1636, 1623, 1558, 1507, 1496, 1487, 1472, 1457, 1436, 1423, 1375, 1324, 1245, 1158, 1052, 913, 743, 668 cm^{-1}. **HRMS** (ESI) m/z calcd for $C_{33}H_{37}N_3NaO_3S$ $[M+Na]^+$: 578.2448; found: 578.2445.

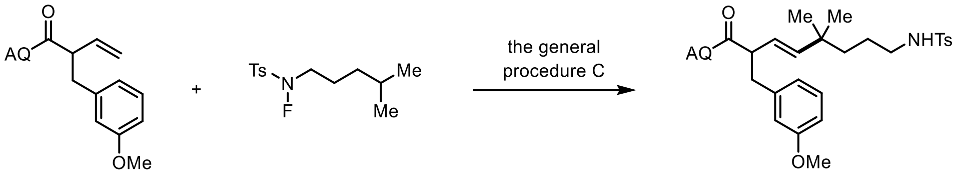

1f **2a** **6e**

Product **6e** was prepared by the general procedure C. Purification using column chromatography (PE/EA = 5:1) afforded **6e** as yellow oil (105 mg, 0.18 mmol, 90%, *r.r.* > 20:1, *E/Z* > 20:1). **^{1}H NMR** (600 MHz, $CDCl_3$) δ 10.12 (s, 1H), 8.78 (dd, *J* = 7.2, 1.2 Hz, 1H), 8.73 (dd, *J* = 4.8, 1.8 Hz, 1H), 8.14 (dd, *J* = 8.4, 1.8 Hz, 1H), 7.71 (d, *J* = 8.4 Hz, 2H), 7.55 - 7.49 (m, 2H), 7.43 (dd, *J* = 7.8, 4.2 Hz, 1H), 7.29 (d, *J* = 8.4 Hz, 2H), 7.16 (t, *J* = 7.8 Hz, 1H), 6.81 (d, *J* = 7.8 Hz, 1H), 6.74 (s, 1H), 6.69 (dd, *J* = 8.4, 2.4 Hz, 1H), 5.46 - 5.39 (m, 2H), 4.37 (t, *J* = 6.6 Hz, 1H), 3.77 (s, 3H), 3.45 (dd, *J* = 13.8, 5.4 Hz, 1H), 3.42 - 3.38 (m, 1H), 2.83 (dd, *J* = 13.8, 9.6 Hz, 1H), 2.77 - 2.69 (m, 2H), 2.42 (s, 3H), 1.17 - 1.12 (m, 3H), 0.99 (s, 3H), 0.94 (s, 3H), 0.92 - 0.88 (m, 1H). **^{13}C NMR** (150 MHz, $CDCl_3$) δ 171.9, 159.5, 148.2, 145.6, 143.4, 141.5, 138.7, 137.3, 136.4, 134.6, 129.8, 129.4, 128.1, 127.5, 127.2, 124.3, 122.1, 121.8, 121.7, 116.4, 115.5, 111.7, 55.5, 54.0, 43.9, 39.6, 38.0, 36.2, 28.3, 25.9, 24.8, 21.6. **IR** ν_{max} (film): 3316, 2956, 2867, 1770, 1682, 1597, 1583, 1525, 1486, 1425, 1385, 1326, 1259, 1159, 1094, 1050, 978, 914, 883, 826, 815, 791, 741, 696, 575, 550, 418 cm^{-1}. **HRMS** (ESI) m/z calcd for $C_{34}H_{39}N_3NaO_4S$ $[M+Na]^+$: 608.2553; found: 608.2563.

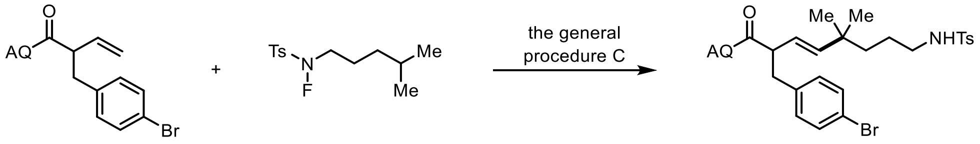

1g **2a** **6f**

Product **6f** was prepared by the general procedure C. Purification using column chromatography (PE/EA = 5:1) afforded **6f** as yellow oil (103 mg, 0.16 mmol, 81%, *r.r.* > 20:1, *E/Z* > 20:1). **^{1}H NMR** (600 MHz, $CDCl_3$) δ 10.05 (s, 1H), 8.77 - 8.73 (m, 2H), 8.15 (dd, *J* = 7.8, 1.2 Hz, 1H), 7.70 (d, *J* = 8.4 Hz, 2H), 7.55 - 7.49 (m, 2H), 7.44 (dd, *J* = 8.4, 4.2 Hz, 1H), 7.35 (d, *J* = 7.8 Hz, 2H), 7.27 (d, *J* = 7.8 Hz, 2H), 7.07 (d, *J* = 8.4 Hz, 2H), 5.48 (d, *J* = 15.6 Hz, 1H), 5.41 (dd, *J* = 15.6, 8.4 Hz, 1H), 4.29 - 4.27 (m, 1H), 3.39 - 3.33 (m, 2H), 2.86 - 2.78 (m, 3H), 2.41 (s, 3H), 1.21 - 1.18 (m, 3H), 1.05 - 1.02 (m, 1H), 0.99 (s, 3H), 0.93 (s, 3H). **^{13}C NMR** (150 MHz, $CDCl_3$) δ 171.6, 148.3, 145.8, 143.4, 138.8, 138.7, 137.2, 136.5, 134.5, 131.4, 131.3, 129.8, 128.1, 127.5, 127.2, 124.0, 121.82, 121.77, 119.9, 116.4, 53.9, 43.9, 39.7, 37.4, 36.2, 27.8, 26.4, 24.9, 21.6. **IR** ν_{max} (film): 3650, 3414, 2957, 2925, 2867, 1676, 1637, 1618, 1597, 1525, 1486, 1424, 1385, 1326, 1159, 1094, 1072, 1011, 826, 813, 792, 737, 577, 550, 480 cm^{-1}. **HRMS** (ESI) m/z calcd for $C_{33}H_{36}BrN_3NaO_3S$ $[M+Na]^+$: 656.1553; found: 656.1556.

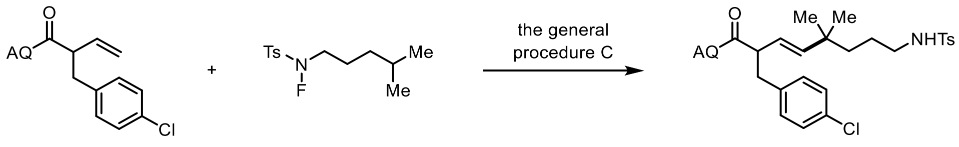

1h **2a** **6g**

Product **6g** was prepared by the general procedure C. Purification using column chromatography (PE/EA = 5:1) afforded **6g** as yellow oil (106 mg, 0.18 mmol, 90%, *r.r.* > 20:1, *E/Z* > 20:1). **^{1}H NMR** (600 MHz, $CDCl_3$) δ 10.05 (s, 1H), 8.76 (dd, *J* = 7.8, 1.8 Hz, 1H), 8.73 (dd, *J* = 4.2, 1.2 Hz, 1H), 8.14 (dd, *J* = 7.8, 1.2 Hz, 1H), 7.70 (d, *J* = 7.8 Hz, 2H), 7.54 - 7.49 (m, 2H), 7.43 (dd, *J* = 8.4, 4.2 Hz, 1H), 7.26 (d, *J* = 8.4 Hz, 2H), 7.19 (d, *J* = 8.4 Hz, 2H), 7.12 (d, *J* = 7.8 Hz, 2H), 5.47 (d, *J* = 15.6 Hz, 1H), 5.41 (dd, *J* = 15.6, 8.4 Hz, 1H), 4.37 (t, *J* = 6.6 Hz, 1H), 3.39 - 3.33 (m, 2H), 2.88 - 2.84 (m, 1H), 2.80 - 2.77 (m, 2H), 2.40 (s, 3H), 1.22 - 1.18 (m, 3H), 1.08 - 1.02 (m, 1H), 0.99 (s, 3H), 0.93 (s, 3H). **^{13}C NMR** (150 MHz, $CDCl_3$) δ 171.6, 148.3, 145.8, 143.4, 138.6, 138.3, 137.2, 136.4, 134.5, 131.9, 130.9, 129.8, 128.4, 128.1, 127.5, 127.2, 124.0, 121.80, 121.76, 116.4, 53.9, 43.9, 39.7, 37.4, 36.2, 27.7, 26.4, 24.9, 21.6. **IR** ν_{max} (film): 2956, 2925, 1770, 1760, 1749, 1716, 1683, 1652, 1647, 1635, 1596, 1525, 1487, 1456, 1424, 1385, 1325, 1264, 1246, 1159, 1093, 1015, 978, 913, 892, 826, 814, 792, 740, 705, 576, 550 cm^{-1}. **HRMS** (ESI) m/z calcd for $C_{33}H_{36}ClN_3NaO_3S$ $[M+Na]^+$: 612.2058; found: 612.2049.

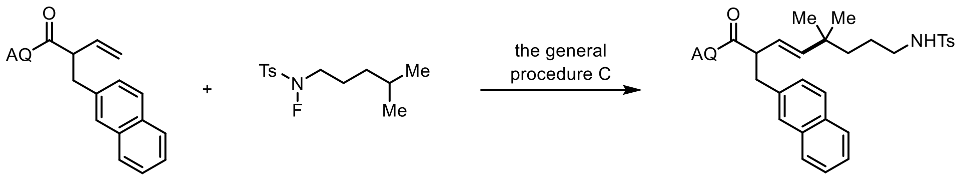

1i **2a** **6h**

Product **6h** was prepared by the general procedure C. Purification using column chromatography (PE/EA = 5:1) afforded **6h** as yellow oil (105 mg, 0.17 mmol, 87%, *r.r.* > 20:1, *E/Z* > 20:1). **^{1}H NMR** (600 MHz, $CDCl_3$) δ 10.15 (s, 1H), 8.80 (dd, *J* = 7.8, 1.2 Hz, 1H), 8.70 (dd, *J* = 4.2, 1.8 Hz, 1H), 8.14 (dd, *J* = 8.4, 1.8 Hz, 1H), 7.75 - 7.73 (m, 3H), 7.63 - 7.61 (m, 3H), 7.56 - 7.54 (m, 1H), 7.51 - 7.49 (m, 1H), 7.43 - 7.40 (m, 2H), 7.38 - 7.35 (m, 2H), 7.28 (d, *J* = 7.8 Hz, 2H), 5.47 (dd, *J* = 15.6, 9.0 Hz, 1H), 5.40 (d, *J* = 15.6 Hz, 1H), 3.64 (dd, *J* = 13.8, 4.8 Hz, 1H), 3.59 (t, *J* = 6.6 Hz, 1H), 3.53 - 3.49 (m, 1H), 3.02 (dd, *J* = 13.8, 10.2 Hz, 1H), 2.42 (s, 3H), 2.41 - 2.37 (m, 2H), 1.04 - 1.01 (m, 2H), 0.97 (s, 3H), 0.89 (s, 3H), 0.87 - 0.82 (m, 1H), 0.57 - 0.50 (m, 1H). **^{13}C NMR** (150 MHz, $CDCl_3$) δ 171.9, 148.2, 145.8, 143.3, 138.7, 137.5, 137.3, 136.4, 134.6, 133.6, 132.1, 129.7, 128.09, 128.06, 128.00, 127.95, 127.7, 127.6, 127.2, 126.2, 125.6, 124.3, 121.8, 121.7, 116.4, 54.1, 43.6, 39.7, 38.1, 36.2, 28.5, 25.7, 24.7, 21.6. **IR** ν_{max} (film): 3566, 3445,

2925, 1646, 1634, 1558, 1524, 1487, 1457, 1424, 1384, 1325, 1241, 1159, 1095, 791, 747, 550, 418 cm^{-1}. **HRMS** (ESI) m/z calcd for $C_{37}H_{39}N_3NaO_3S$ $[M+Na]^+$: 628.2604; found: 628.2594.

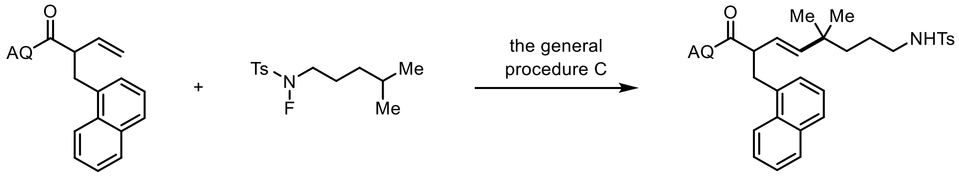

1j **2a** **6i**

Product **6i** was prepared by the general procedure C. Purification using column chromatography (PE/EA = 5:1) afforded **6i** as yellow oil (99.3 mg, 0.16 mmol, 82%, *r.r.* > 20:1, *E/Z* > 20:1). **^{1}H NMR** (600 MHz, $CDCl_3$) δ 10.15 (s, 1H), 8.83 (dd, *J* = 7.2, 1.2 Hz, 1H), 8.69 (dd, *J* = 4.2, 1.8 Hz, 1H), 8.14 (dd, *J* = 8.4, 1.8 Hz, 2H), 7.81 (d, *J* = 8.4 Hz, 1H), 7.68 - 7.66 (m, 3H), 7.57 - 7.52 (m, 2H), 7.51 - 7.46 (m, 2H), 7.42 (dd, *J* = 8.4, 4.2 Hz, 1H), 7.36 - 7.31 (m, 2H), 7.27 (d, *J* = 7.8 Hz, 2H), 5.50 (dd, *J* = 15.6, 9.6 Hz, 1H), 5.22 (d, *J* = 15.6 Hz, 1H), 4.01 - 3.97 (m, 2H), 3.59 - 3.55 (m, 1H), 3.26 (dd, *J* = 13.8, 10.2 Hz, 1H), 2.63 - 2.59 (m, 2H), 2.41 (s, 3H), 0.99 - 0.95 (m, 2H), 0.92 (s, 3H), 0.89 - 0.87 (m, 1H), 0.81 (s, 3H), 0.63 - 0.56 (m, 1H). **^{13}C NMR** (150 MHz, $CDCl_3$) δ 172.0, 148.2, 145.5, 143.4, 138.7, 137.2, 136.4, 136.1, 134.6, 134.0, 132.3, 129.8, 128.9, 128.1, 127.8, 127.5, 127.2, 127.0, 126.1, 125.7, 125.5, 124.3, 124.2, 121.8, 121.7, 116.4, 53.5, 43.8, 39.5, 36.0, 34.7, 28.1, 25.7, 24.5, 21.6. **IR** ν_{max} (film): 3317, 3047, 2957, 2926, 2686, 1771, 1749, 1716, 1683, 1596, 1524, 1485, 1456, 1424, 1385, 1326, 1241, 1159, 1094, 978, 826, 814, 792, 779, 739, 576, 550 cm^{-1}. **HRMS** (ESI) m/z calcd for $C_{37}H_{39}N_3NaO_3S$ $[M+Na]^+$: 628.2604; found: 628.2606.

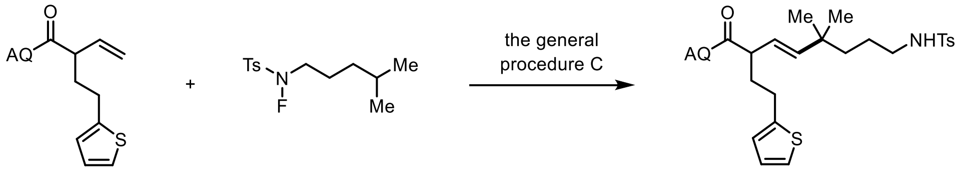

1k **2a** **6j**

Product **6j** was prepared by the general procedure C. Purification using column chromatography (PE/EA = 5:1) afforded **6j** as yellow oil (74.8 mg, 0.13 mmol, 65%, *r.r.* > 20:1, *E/Z* > 20:1). **^{1}H NMR** (600 MHz, $CDCl_3$) δ 10.04 (s, 1H), 8.77 - 8.75 (m, 2H), 8.14 (dd, *J* = 7.8, 1.2 Hz, 1H), 7.67 (d, *J* = 7.8 Hz, 2H), 7.54 - 7.49 (m, 2H), 7.44 (dd, *J* = 8.4, 4.2 Hz, 1H), 7.24 (d, *J* = 7.8 Hz, 2H), 7.12 (dd, *J* = 4.8, 1.2 Hz, 1H), 6.93 - 6.92 (m, 1H), 6.82 (d, *J* = 3.6 Hz, 1H), 5.68 (d, *J* = 15.6 Hz, 1H), 5.47 (d, *J* = 15.6, 9.0 Hz, 1H), 4.50 (t, *J* = 6.6 Hz, 1H), 3.18 - 3.14 (m, 1H), 2.96 - 2.91 (m, 1H), 2.89 - 2.84 (m, 3H), 2.46 - 2.40 (m, 1H), 2.38 (s, 3H), 2.00 - 1.94 (m, 1H), 1.45 - 1.40 (m, 2H), 1.33 - 1.29 (m, 2H), 1.04 (s, 3H), 1.03 (s, 3H). **^{13}C NMR** (150 MHz, $CDCl_3$) δ 172.3, 148.3,

145.4, 144.4, 143.4, 138.6, 137.1, 136.4, 134.6, 129.7, 128.1, 127.5, 127.2, 126.9, 124.6, 124.5, 123.2, 121.8, 121.7, 116.4, 51.1, 44.0, 39.7, 36.2, 33.4, 27.5, 27.2, 27.1, 25.1, 21.6. **IR** ν_{max} (film): 3318, 3046, 2956, 2867, 1680, 1596, 1577, 1525, 1485, 1424, 1385, 1326, 1262, 1241, 1159, 1094, 981, 887, 848, 826, 815, 792, 757, 735, 696, 575, 551 cm^{-1}. **HRMS** (ESI) m/z calcd for $C_{32}H_{37}N_3NaO_3S_2$ $[M+Na]^+$: 598.2169; found: 598.2169.

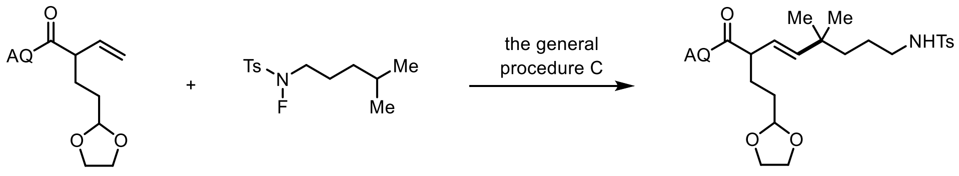

1l **2a** **6k**

Product **6k** was prepared by the general procedure C. Purification using column chromatography (PE/EA = 3:1) afforded **6k** as yellow oil (98.4 mg, 0.17 mmol, 87%, *r.r.* > 20:1, *E/Z* > 20:1). **^{1}H NMR** (600 MHz, $CDCl_3$) δ 10.05 (s, 1H), 8.77 - 8.76 (m, 2H), 8.15 (dd, *J* = 7.8, 1.2 Hz, 1H), 7.69 (d, *J* = 7.8 Hz, 2H), 7.54 - 7.48 (m, 2H), 7.44 (dd, *J* = 8.4, 4.2 Hz, 1H), 7.26 (d, *J* = 7.8 Hz, 2H), 5.66 (d, *J* = 15.6 Hz, 1H), 5.47 (d, *J* = 15.6, 9.0 Hz, 1H), 4.92 (t, *J* = 4.2 Hz, 1H), 4.61 (t, *J* = 6.6 Hz, 1H), 4.01 - 3.96 (m, 2H), 3.88 - 3.85 (m, 2H), 3.18 - 3.14 (m, 1H), 2.86 (dt, *J* = 6.6, 6.6 Hz, 2H), 2.40 (s, 3H), 2.19 - 2.14 (m, 1H), 1.76 - 1.72 (m, 3H), 1.43 - 1.37 (m, 2H), 1.29 - 1.26 (m, 2H), 1.03 (s, 3H), 1.01 (s, 3H). **^{13}C NMR** (150 MHz, $CDCl_3$) δ 172.5, 148.2, 144.8, 143.4, 138.7, 137.2, 136.4, 134.7, 129.8, 128.1, 127.5, 127.2, 125.0, 121.8, 121.6, 116.4, 104.4, 65.1, 65.0, 51.8, 44.0, 39.6, 36.2, 31.6, 27.5, 26.8, 26.2, 25.2, 21.6. **IR** ν_{max} (film): 2956, 1682, 1525, 1485, 1425, 1385, 1326, 1159, 1094, 979, 826, 814, 792, 757, 661, 576, 550 cm^{-1}. **HRMS** (ESI) m/z calcd for $C_{31}H_{39}N_3NaO_5S$ $[M+Na]^+$: 588.2503; found: 588.2487.

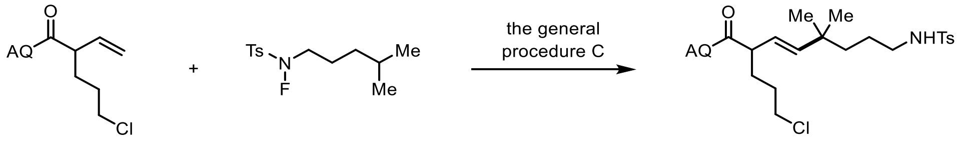

1m **2a** **6l**

Product **6l** was prepared by the general procedure C. Purification using column chromatography (PE/EA = 5:1) afforded **6l** as yellow oil (89.8 mg, 0.17 mmol, 83%, *r.r.* > 20:1, *E/Z* > 20:1). **^{1}H NMR** (600 MHz, $CDCl_3$) δ 10.06 (s, 1H), 8.76 - 8.74 (m, 2H), 8.15 (dd, *J* = 8.4, 1.2 Hz, 1H), 7.69 (d, *J* = 7.8 Hz, 2H), 7.54 - 7.49 (m, 2H), 7.45 (dd, *J* = 8.4, 4.2 Hz, 1H), 7.26 (d, *J* = 7.8 Hz, 2H), 5.70 (d, *J* = 15.6 Hz, 1H), 5.46 (d, *J* = 15.6, 9.0 Hz, 1H), 4.56 (t, *J* = 6.6 Hz, 1H), 3.61 - 3.54 (m, 2H), 3.14 - 3.10 (m, 1H), 2.87 (dt, *J* = 6.6, 6.6 Hz, 2H), 2.39 (s, 3H), 2.17 - 2.11 (m, 1H), 1.88 -

1.81 (m, 2H), 1.80 - 1.76 (m, 1H), 1.45 - 1.39 (m, 2H), 1.32 - 1.29 (m, 2H), 1.04 (s, 3H), 1.02 (s, 3H). **^{13}C NMR** (150 MHz, $CDCl_3$) δ 172.1, 148.3, 145.2, 143.4, 138.6, 137.1, 136.5, 134.5, 129.8, 128.1, 127.5, 127.2, 124.6, 121.8, 121.7, 116.4, 51.5, 44.9, 44.0, 39.7, 36.2, 30.6, 29.1, 27.3, 27.0, 25.1, 21.6. **IR** ν_{max} (film): 3317, 2957, 2868, 1682, 1596, 1576, 1525, 1485, 1457, 1424, 1385, 1326, 1241, 1159, 1094, 980, 826, 815, 792, 757, 706, 575, 550 cm^{-1}. **HRMS** (ESI) m/z calcd for $C_{29}H_{36}ClN_3NaO_3S$ $[M+Na]^+$: 564.2058; found: 564.2050.

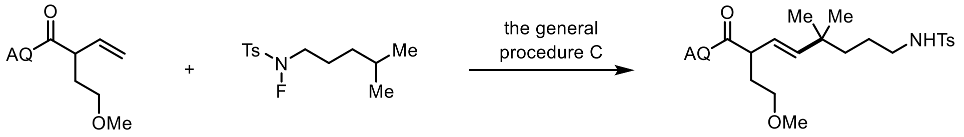

1n **2a** **6m**

Product **6m** was prepared by the general procedure C. Purification using column chromatography (PE/EA = 5:1) afforded **6m** as yellow oil (78.5 mg, 0.15 mmol, 75%, *r.r.* > 20:1, *E/Z* > 20:1). **^{1}H NMR** (600 MHz, $CDCl_3$) δ 10.07 (s, 1H), 8.77 - 8.76 (m, 2H), 8.15 (dd, *J* = 8.4, 1.8 Hz, 1H), 7.68 (d, *J* = 7.8 Hz, 2H), 7.54 - 7.49 (m, 2H), 7.45 (dd, *J* = 8.4, 4.2 Hz, 1H), 7.26 (d, *J* = 7.8 Hz, 2H), 5.68 (d, *J* = 15.6 Hz, 1H), 5.47 (d, *J* = 15.6, 9.0 Hz, 1H), 4.46 (t, *J* = 6.6 Hz, 1H), 3.49 - 3.43 (m, 2H), 3.36 - 3.33 (m, 4H), 2.85 (dt, *J* = 6.6, 6.6 Hz, 2H), 2.40 (s, 3H), 2.33 - 2.28 (m, 1H), 1.84 - 1.78 (m, 1H), 1.45 - 1.36 (m, 2H), 1.31 - 1.28 (m, 2H), 1.03 (s, 3H), 1.01 (s, 3H). **^{13}C NMR** (150 MHz, $CDCl_3$) δ 172.6, 148.3, 144.6, 143.4, 138.7, 137.2, 136.4, 134.7, 129.8, 128.1, 127.5, 127.2, 124.7, 121.8, 121.6, 116.4, 70.1, 58.7, 48.7, 44.0, 39.7, 36.1, 31.7, 27.3, 27.1, 25.1, 21.6. **IR** ν_{max} (film): 3266, 2955, 2924, 2867, 1683, 1524, 1485, 1457, 1424, 1384, 1324, 1241, 1158, 1117, 1093, 976, 826, 814, 791, 749, 660, 550 cm^{-1}. **HRMS** (ESI) m/z calcd for $C_{29}H_{37}N_3NaO_4S$ $[M+Na]^+$: 546.2397; found: 546.2404.

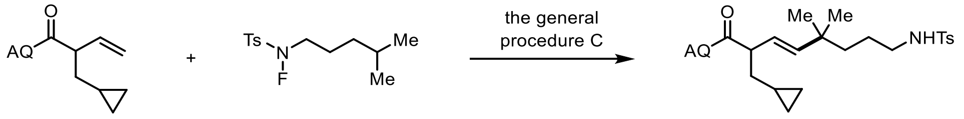

1o **2a** **6n**

Product **6n** was prepared by the general procedure C. Purification using column chromatography (PE/EA = 5:1) afforded **6n** as yellow oil (87.2 mg, 0.17 mmol, 84%, *r.r.* > 20:1, *E/Z* > 20:1). **^{1}H NMR** (600 MHz, $CDCl_3$) δ 10.06 (s, 1H), 8.78 - 8.76 (m, 2H), 8.15 (dd, *J* = 8.4, 1.8 Hz, 1H), 7.68 (d, *J* = 7.8 Hz, 2H), 7.54 - 7.48 (m, 2H), 7.44 (dd, *J* = 8.4, 4.2 Hz, 1H), 7.25 (d, *J* = 7.8 Hz, 2H), 5.67 (d, *J* = 15.6 Hz, 1H), 5.55 (d, *J* = 15.6, 8.4 Hz, 1H), 4.52 - 4.51 (m, 1H), 3.25 - 3.21 (m, 1H), 2.86 (dt, *J* = 6.6, 6.6 Hz, 2H), 2.39 (s, 3H), 1.82 - 1.78 (m, 1H), 1.67 - 1.62

(m, 1H), 1.45 - 1.39 (m, 2H), 1.32 - 1.29 (m, 2H), 1.04 (s, 3H), 1.02 (s, 3H), 0.77 - 0.71 (m, 1H), 0.47 - 0.39 (m, 2H), 0.15 - 0.08 (m, 2H). **^{13}C NMR** (150 MHz, $CDCl_3$) δ 172.9, 148.3, 144.0, 143.4, 138.7, 137.2, 136.4, 134.7, 129.7, 128.1, 127.5, 127.2, 125.3, 121.8, 121.5, 116.4, 52.8, 44.0, 39.8, 37.3, 36.0, 27.3, 27.1, 25.1, 21.6, 9.2, 5.1, 4.6. **IR** ν_{max} (film): 3318, 3073, 3000, 2958, 2868, 1684, 1596, 1523, 1485, 1425, 1385, 1326, 1263, 1184, 1159, 1094, 1018, 978, 925, 887, 826, 792, 736, 705, 575, 551 cm^{-1}. **HRMS** (ESI) m/z calcd for $C_{30}H_{37}N_3NaO_3S$ [M+Na]$^+$: 542.2448; found: 542.2443.

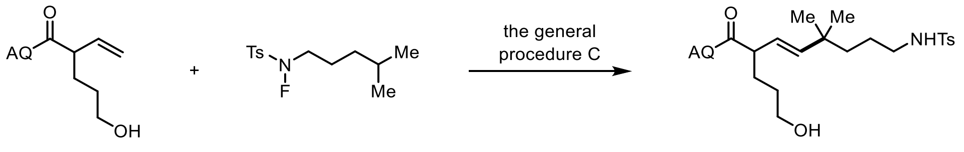

Product **6o** was prepared by the general procedure C. Purification using column chromatography (PE/EA = 2:1) afforded **6o** as yellow oil (69.1 mg, 0.13 mmol, 66%, *r.r.* > 20:1, *E/Z* > 20:1). **^{1}H NMR** (600 MHz, $CDCl_3$) δ 10.08 (s, 1H), 8.76 - 8.74 (m, 2H), 8.14 (dd, *J* = 8.4, 1.8 Hz, 1H), 7.70 (d, *J* = 7.8 Hz, 2H), 7.53 - 7.47 (m, 2H), 7.43 (dd, *J* = 8.4, 4.2 Hz, 1H), 7.25 (d, *J* = 7.8 Hz, 2H), 5.68 (d, *J* = 15.6 Hz, 1H), 5.47 (d, *J* = 15.6, 9.0 Hz, 1H), 5.08 (t, *J* = 6.0 Hz, 1H), 3.72 - 3.64 (m, 2H), 3.16 - 3.12 (m, 1H), 2.85 (dt, *J* = 6.0, 6.0 Hz, 2H), 2.39 (s, 3H), 2.10 - 2.04 (m, 1H), 1.74 - 1.66 (m, 2H), 1.63 - 1.58 (m, 1H), 1.46 - 1.39 (m, 2H), 1.32 - 1.29 (m, 2H), 1.04 (s, 3H), 1.02 (s, 3H). **^{13}C NMR** (150 MHz, $CDCl_3$) δ 172.8, 148.3, 144.8, 143.4, 138.7, 137.2, 136.5, 134.6, 129.8, 128.1, 127.5, 127.2, 124.9, 121.8, 121.7, 116.5, 62.6, 51.8, 43.9, 39.7, 36.2, 30.5, 28.0, 27.8, 26.6, 25.2, 21.6. **IR** ν_{max} (film): 2954, 2926, 2868, 1683, 1596, 1525, 1486, 1457, 1425, 1385, 1325, 1241, 1158, 1094, 978, 826, 792, 756, 551 cm^{-1}. **HRMS** (ESI) m/z calcd for $C_{29}H_{37}N_3NaO_4S$ [M+Na]$^+$: 546.2397; found: 546.2392.

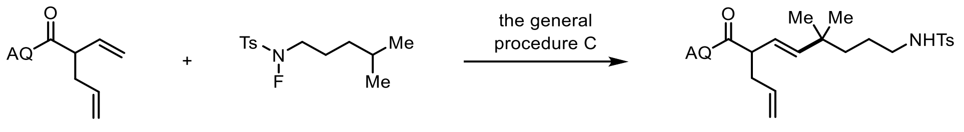

Product **6p** was prepared by the general procedure C. Purification using column chromatography (PE/EA = 5:1) afforded **6p** as yellow oil (85.9 mg, 0.17 mmol, 85%, *r.r.* > 20:1, *E/Z* > 20:1). **^{1}H NMR** (600 MHz, $CDCl_3$) δ 10.06 (s, 1H), 8.77 - 8.75 (m, 2H), 8.15 (dd, *J* = 8.4, 1.8 Hz, 1H), 7.69 (d, *J* = 7.8 Hz, 2H), 7.54 - 7.49 (m, 2H), 7.44 (dd, *J* = 8.4, 4.2 Hz, 1H), 7.26 (d, *J* = 7.8 Hz, 2H), 5.83 - 5.76 (m, 1H), 5.66 (d, *J* = 16.2 Hz, 1H), 5.47 (dd, *J* = 16.2, 9.0 Hz, 1H),

5.10 (dd, *J* = 16.8, 1.8 Hz, 1H), 5.02 (dd, *J* = 10.2, 1.8 Hz, 1H), 4.51 (t, *J* = 6.6 Hz, 1H), 3.21 - 3.17 (m, 1H), 2.87 (dt, *J* = 6.6, 6.6 Hz, 2H), 2.77 - 2.72 (m, 1H), 2.45 - 2.41 (m, 1H), 2.39 (s, 3H), 1.44 - 1.39 (m, 2H), 1.31 - 1.28 (m, 2H), 1.04 (s, 3H), 1.02 (s, 3H). **^{13}C NMR** (150 MHz, $CDCl_3$) δ 172.1, 148.3, 144.8, 143.4, 138.7, 137.2, 136.4, 135.9, 134.6, 129.8, 128.1, 127.5, 127.2, 124.6, 121.8, 121.6, 117.0, 116.4, 51.8, 44.0, 39.7, 36.3, 36.2, 27.4, 27.0, 25.0, 21.6. **IR** ν_{max} (film): 3318, 2958, 2868, 1771, 1716, 1683, 1596, 1525, 1485, 1457, 1424, 1385, 1326, 1241, 1159, 1094, 979, 914, 826, 814, 792, 742, 706, 575, 550, 418 cm^{-1}. **HRMS** (ESI) m/z calcd for $C_{29}H_{35}N_3NaO_3S$ $[M+Na]^+$: 528.2291; found: 528.2278.

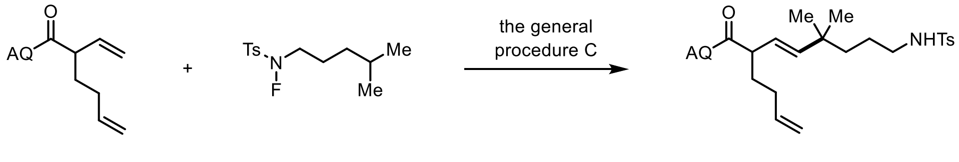

1r **2a** **6q**

Product **6q** was prepared by the general procedure C. Purification using column chromatography (PE/EA = 5:1) afforded **6q** as yellow oil (89.3 mg, 0.17 mmol, 86%, *r.r.* > 20:1, *E/Z* > 20:1). **^{1}H NMR** (600 MHz, $CDCl_3$) δ 10.03 (s, 1H), 8.78 - 8.76 (m, 2H), 8.15 (dd, *J* = 8.4, 1.8 Hz, 1H), 7.68 (d, *J* = 8.4 Hz, 2H), 7.54 - 7.49 (m, 2H), 7.45 (dd, *J* = 8.4, 4.2 Hz, 1H), 7.25 (d, *J* = 8.4 Hz, 2H), 5.86 - 5.79 (m, 1H), 5.66 (d, *J* = 15.6 Hz, 1H), 5.45 (dd, *J* = 15.6, 9.0 Hz, 1H), 5.04 (dd, *J* = 16.8, 1.8 Hz, 1H), 5.00 (d, *J* = 10.2, 1H), 4.45 - 4.43 (m, 1H), 3.15 - 3.11 (m, 1H), 2.86 (dt, *J* = 6.6, 6.6 Hz, 2H), 2.39 (s, 3H), 2.15 - 2.09 (m, 2H), 1.72 - 1.67 (m, 2H), 1.44 - 1.39 (m, 2H), 1.32 - 1.29 (m, 2H), 1.04 (s, 3H), 1.02 (s, 3H). **^{13}C NMR** (150 MHz, $CDCl_3$) δ 172.7, 148.3, 144.7, 143.4, 138.7, 138.1, 137.1, 136.5, 134.7, 129.8, 128.1, 127.5, 127.2, 124.9, 121.8, 121.6, 116.4, 115.4, 51.4, 44.0, 39.7, 36.1, 31.4, 30.9, 27.19, 27.18, 25.1, 21.6. **IR** ν_{max} (film): 2957, 1685, 1596, 1525, 1485, 1424, 1385, 1325, 1260, 1159, 1094, 980, 912, 826, 792, 756, 550 cm^{-1}. **HRMS** (ESI) m/z calcd for $C_{30}H_{37}N_3NaO_3S$ $[M+Na]^+$: 542.2448; found: 542.2424.

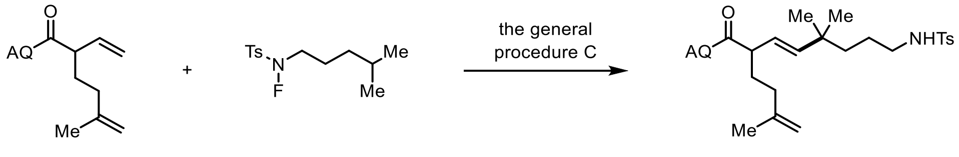

1s **2a** **6r**

Product **6r** was prepared by the general procedure C. Purification using column chromatography (PE/EA = 5:1) afforded **6r** as yellow oil (90.7 mg, 0.17 mmol, 85%, *r.r.* > 20:1, *E/Z* > 20:1). **^{1}H NMR** (600 MHz, $CDCl_3$) δ 10.03 (s, 1H), 8.78 - 8.76 (m, 2H), 8.15 (dd, *J* = 7.8, 1.2 Hz, 1H), 7.68 (d, *J* = 8.4 Hz, 2H), 7.54 - 7.48 (m, 2H), 7.44 (dd, *J* = 8.4, 4.2 Hz, 1H), 7.25 (d,

J = 8.4 Hz, 2H), 5.66 (d, J = 15.6 Hz, 1H), 5.45 (dd, J = 15.6, 9.0 Hz, 1H), 4.75 (s, 1H), 4.71 (s, 1H), 4.43 (t, J = 6.0 Hz, 1H), 3.11 - 3.08 (m, 1H), 2.89 - 2.84 (m, 2H), 2.39 (s, 3H), 2.23 - 2.17 (m, 1H), 2.12 - 2.02 (m, 2H), 1.74 (s, 3H), 1.72 - 1.68 (m, 1H), 1.45 - 1.39 (m, 2H), 1.32 - 1.29 (m, 2H), 1.04 (s, 3H), 1.02 (s, 3H). **^{13}C NMR** (150 MHz, $CDCl_3$) δ 172.7, 148.3, 145.2, 144.8, 143.4, 138.7, 137.1, 136.5, 134.7, 129.8, 128.1, 127.5, 127.2, 125.0, 121.8, 121.6, 116.4, 110.7, 51.5, 44.0, 39.7, 36.1, 35.4, 29.7, 27.22, 27.15, 25.1, 22.5, 21.6. **IR** ν_{max} (film): 3319, 3068, 2958, 2868, 1682, 1596, 1577, 1525, 1485, 1456, 1424, 1385, 1326, 1261, 1159, 1094, 979, 887, 826, 814, 792, 756, 551 cm^{-1}. **HRMS** (ESI) m/z calcd for $C_{31}H_{39}N_3NaO_3S$ [M+Na]$^+$: 556.2604; found: 556.2611.

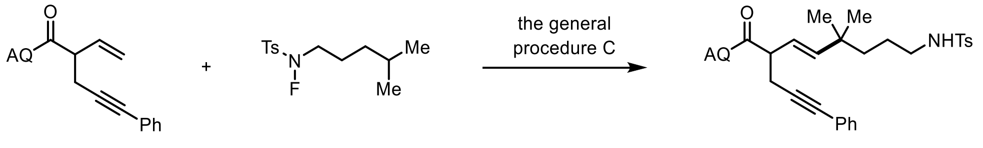

1t 2a 6s

Product **6s** was prepared by the general procedure C. Purification using column chromatography (PE/EA = 5:1) afforded **6s** as yellow oil (85.7 mg, 0.15 mmol, 74%, *r.r.* > 20:1, *E/Z* > 20:1). **^{1}H NMR** (600 MHz, $CDCl_3$) δ 10.21 (s, 1H), 8.77 (dd, J = 7.8, 1.8 Hz, 1H), 8.71 (dd, J = 4.2, 1.2 Hz, 1H), 8.14 (dd, J = 8.4, 1.8 Hz, 1H), 7.64 (d, J = 7.8 Hz, 2H), 7.55 - 7.49 (m, 2H), 7.43 (dd, J = 8.4, 4.2 Hz, 1H), 7.32 - 7.31 (m, 2H), 7.26 (d, J = 7.8 Hz, 2H), 7.22 - 7.19 (m, 3H), 5.81 (d, J = 15.6 Hz, 1H), 5.60 (dd, J = 15.6, 9.0 Hz, 1H), 4.12 (t, J = 6.6 Hz, 1H), 3.45 - 3.41 (m, 1H), 3.09 (dd, J = 16.8, 5.4 Hz, 1H), 2.81 (dd, J = 16.8, 9.0 Hz, 1H), 2.74 (dt, J = 6.6, 6.6 Hz, 2H), 2.40 (s, 3H), 1.43 - 1.38 (m, 2H), 1.32 - 1.29 (m, 2H), 1.09 (s, 3H), 1.06 (s, 3H). **^{13}C NMR** (150 MHz, $CDCl_3$) δ 171.0, 148.3, 146.1, 143.4, 138.7, 137.2, 136.4, 134.5, 131.6, 129.7, 128.3, 128.1, 127.9, 127.5, 127.2, 123.9, 123.7, 121.8, 116.5, 88.0, 82.6, 51.3, 44.0, 39.8, 36.3, 27.9, 26.5, 25.1, 22.5, 21.6. **IR** ν_{max} (film): 3442, 3321, 2957, 2925, 2854, 1770, 1758, 1683, 1653, 1525, 1487, 1457, 1424, 1385, 1326, 1241, 1159, 1094, 1063, 976, 913, 826, 814, 791, 755, 692, 574, 550, 471, 418 cm^{-1}. **HRMS** (ESI) m/z calcd for $C_{35}H_{37}N_3NaO_3S$ [M+Na]$^+$: 602.2448; found: 602.2445.

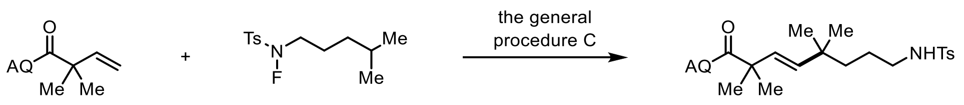

1u 2a 6t

Product **6t** was prepared by the general procedure C. Purification using column chromatography (PE/EA = 5:1) afforded **6t** as yellow oil (85.8 mg, 0.17 mmol, 87%, *r.r.* > 20:1, *E/Z* > 20:1). **^{1}H NMR** (600 MHz, $CDCl_3$) δ 10.23 (s, 1H), 8.76 (dd, J = 7.8, 1.8 Hz, 1H), 8.72 (dd, J = 4.2, 1.8 Hz, 1H), 8.14 (dd, J = 8.4, 1.8 Hz, 1H), 7.69 (d, J = 7.8 Hz, 2H), 7.53 - 7.47 (m, 2H), 7.43 (dd, J = 8.4,

4.2 Hz, 1H), 7.25 (d, *J* = 7.8 Hz, 2H), 5.72 - 5.66 (m, 2H), 4.59 - 4.54 (m, 1H), 2.90 (dt, *J* = 6.6, 6.6 Hz, 2H), 2.39 (s, 3H), 1.46 - 1.43 (m, 2H), 1.41 (s, 6H), 1.37 - 1.34 (m, 2H), 1.09 (s, 6H). **^{13}C NMR** (150 MHz, $CDCl_3$) δ 175.8, 148.2, 143.4, 140.4, 138.9, 137.1, 136.4, 134.9, 131.1, 129.8, 128.1, 127.5, 127.2, 121.7, 121.5, 116.2, 46.0, 44.1, 39.9, 35.9, 27.2, 25.6, 25.1, 21.6. **IR** ν_{max} (film): 3318, 2962, 2929, 2869, 1681, 1596, 1577, 1525, 1485, 1424, 1385, 1326, 1261, 1158, 1094, 986, 922, 826, 815, 792, 756, 707, 576, 550 cm^{-1}. **HRMS** (ESI) m/z calcd for $C_{28}H_{35}N_3NaO_3S$ [M+Na]$^+$: 516.2291; found: 516.2282.

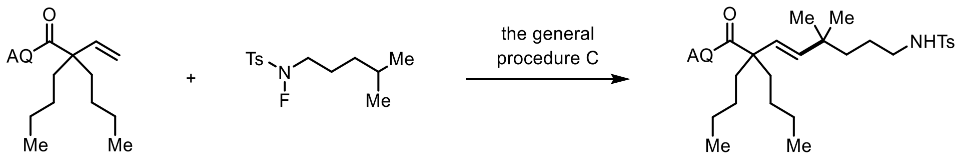

1v **2a** **6u**

Product **6u** was prepared by the general procedure C. Purification using column chromatography (PE/EA = 5:1) afforded **6u** as yellow oil (103 mg, 0.18 mmol, 89%, *r.r.* > 20:1, *E/Z* > 20:1). **^{1}H NMR** (600 MHz, $CDCl_3$) δ 10.20 (s, 1H), 8.78 (dd, *J* = 7.2, 1.2 Hz, 1H), 8.73 (dd, *J* = 4.2, 1.2 Hz, 1H), 8.14 (dd, *J* = 7.8, 1.2 Hz, 1H), 7.69 (d, *J* = 8.4 Hz, 2H), 7.53 - 7.47 (m, 2H), 7.43 (dd, *J* = 8.4, 4.2 Hz, 1H), 7.26 (d, *J* = 7.8 Hz, 2H), 5.68 (d, *J* = 16.2 Hz, 1H), 5.60 (d, *J* = 16.2 Hz, 1H), 4.40 (t, *J* = 6.6 Hz, 1H), 2.89 (dt, *J* = 6.6, 6.6 Hz, 2H), 2.39 (s, 3H), 1.84 - 1.79 (m, 2H), 1.78 - 1.73 (m, 2H), 1.49 - 1.44 (m, 2H), 1.37 - 1.30 (m, 6H), 1.27 - 1.21 (m, 4H), 1.09 (s, 6H), 0.88 (t, *J* = 6.6 Hz, 6H). **^{13}C NMR** (150 MHz, $CDCl_3$) δ 174.8, 148.2, 143.4, 141.3, 138.9, 137.1, 136.4, 134.9, 129.8, 129.2, 128.1, 127.6, 127.2, 121.7, 121.3, 116.2, 52.8, 44.1, 39.9, 36.2, 35.8, 27.3, 26.6, 25.2, 23.4, 21.6, 14.2. **IR** ν_{max} (film): 3321, 2956, 2931, 2869, 1680, 1596, 1577, 1524, 1484, 1467, 1424, 1383, 1326, 1260, 1240, 1184, 1160, 1094, 1019, 986, 916, 889, 826, 814, 792, 756, 707, 576, 551 cm^{-1}. **HRMS** (ESI) m/z calcd for $C_{34}H_{47}N_3NaO_3S$ [M+Na]$^+$: 600.3230; found: 600.3239.

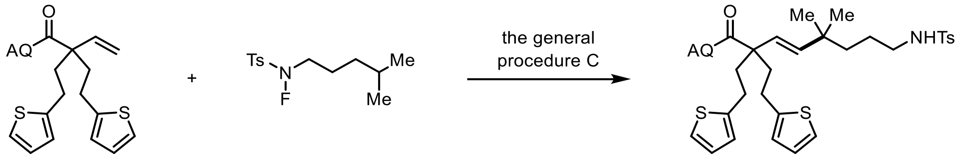

1w **2a** **6v**

Product **6v** was prepared by the general procedure C. Purification using column chromatography (PE/EA = 5:1) afforded **6v** as yellow oil (118 mg, 0.17 mmol, 86%, *r.r.* > 20:1, *E/Z* > 20:1). **^{1}H NMR** (600 MHz, $CDCl_3$) δ 10.32 (s, 1H), 8.80 (dd, *J* = 7.8, 1.8 Hz, 1H), 8.74 (dd,

J = 4.2, 1.8 Hz, 1H), 8.17 (dd, *J* = 8.4, 1.8 Hz, 1H), 7.66 (d, *J* = 8.4 Hz, 2H), 7.57 - 7.51 (m, 2H), 7.46 (dd, *J* = 8.4, 4.2 Hz, 1H), 7.23 (d, *J* = 8.4 Hz, 2H), 7.10 (dd, *J* = 5.4, 1.2 Hz, 2H), 6.90 (dd, *J* = 5.4, 3.6 Hz, 2H), 6.80 (dd, *J* = 3.6, 1.2Hz, 2H), 5.81 (d, *J* = 16.2 Hz, 1H), 5.73 (d, *J* = 16.2 Hz, 1H), 4.21 (t, *J* = 6.0 Hz, 1H), 2.91 - 2.86 (m, 6H), 2.38 (s, 3H), 2.28 - 2.25 (m, 4H), 1.50 - 1.45 (m, 2H), 1.39 - 1.37 (m, 2H), 1.26 (s, 6H). **^{13}C NMR** (150 MHz, $CDCl_3$) δ 173.3, 148.3, 145.0, 143.5, 142.8, 138.9, 137.1, 136.5, 134.6, 129.8, 128.1, 127.7, 127.6, 127.2, 127.0, 124.3, 123.2, 121.9, 121.8, 116.5, 52.9, 44.1, 39.9, 38.3, 36.5, 27.3, 25.3, 25.2, 21.6. **IR** ν_{max} (film): 2954, 2925, 2854, 1734, 1675, 1595, 1577, 1523, 1483, 1457, 1424, 1384, 1325, 1260, 1239, 1186, 1158, 1093, 986, 889, 849, 825, 792, 757, 692, 669, 550, 418 cm^{-1}. **HRMS** (ESI) m/z calcd for $C_{38}H_{43}N_3NaO_3S_3$ $[M+Na]^+$: 708.2359; found: 708.2350.

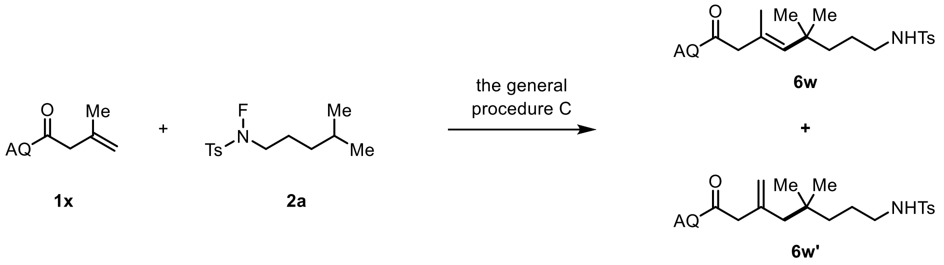

Product **6w** and **6w′** was prepared by the general procedure C. Purification using column chromatography (PE/EA = 5:1) afforded a mixture of **6w** and **6w′** as yellow oil (78.6 mg, 0.16 mmol, 82%, **6w**/**6w′** = 2:1, *E/Z* > 20:1 for **6w**). **^{1}H NMR** (600 MHz, $CDCl_3$) δ 10.11 (s, 0.33H), 10.09 (s, 0.67H), 8.80 - 8.75 (m, 2H), 8.17 - 8.14 (m, 1H), 7.74 (d, *J* = 7.8 Hz, 0.66H), 7.69 (d, *J* = 7.8 Hz, 1.34H), 7.56 - 7.49 (m, 2H), 7.46 - 7.43 (m, 1H), 7.28 (d, *J* = 7.8 Hz, 0.66H), 7.25 (d, *J* = 7.8 Hz, 1.34H), 5.42 (s, 0.67H), 5.29 (s, 0.33H), 5.09 (s, 0.33H), 4.90 - 4.86 (m, 1H), 3.39 (s, 1.34H), 3.31 (s, 0.66 H), 2.94 - 2.90 (m, 2H), 2.40 (s, 1H), 2.39 (s, 2H), 2.07 (s, 0.66H), 1.84 (s, 2H), 1.56 - 1.51 (m, 1.34H), 1.47 - 1.42 (m, 0.66H), 1.39 - 1.37 (m, 1.34H), 1.24 - 1.21 (m, 0.66H), 1.12 (s, 4H), 0.87 (s, 2H). **^{13}C NMR** (150 MHz, $CDCl_3$) δ 169.7, 169.6, 148.5, 148.4, 143.4, 143.2, 141.4, 138.9, 138.74, 138.69, 137.34, 137.31, 136.5, 136.4, 134.5, 134.4, 129.8, 129.7, 128.4, 128.08, 128.07, 127.54, 127.45, 127.22, 127.18, 121.82, 121.79, 121.7, 119.6, 116.7, 116.5, 48.5, 46.9, 44.02, 43.95, 42.3, 41.5, 39.1, 35.8, 34.0, 29.3, 27.6, 26.2, 25.2, 24.5, 21.6. **IR** ν_{max} (film): 3302, 3048, 2957, 2868, 1682, 1597, 1577, 1527, 1485, 1424, 1385, 1327, 1262, 1212, 1159, 1094, 896, 827, 815, 793, 757, 738, 706, 662, 573, 551, 507 cm^{-1}. **HRMS** (ESI) m/z calcd for $C_{27}H_{33}N_3NaO_3S$ $[M+Na]^+$: 502.2135; found: 502.2132.

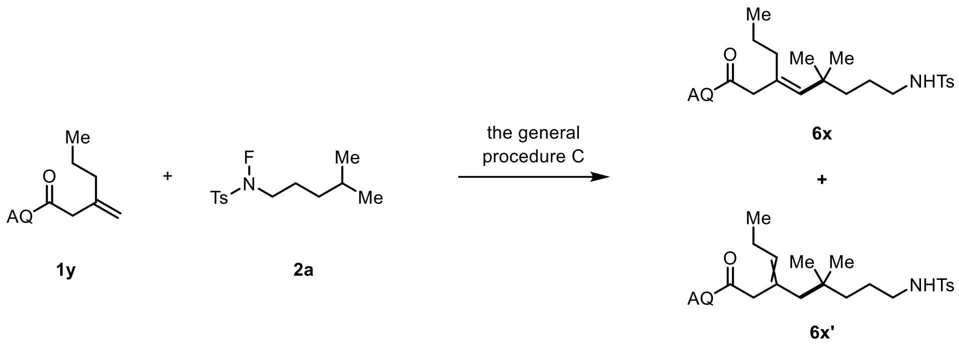

Product **6x** and **6x′** was prepared by the general procedure C. Purification using column chromatography (PE/EA = 5:1) afforded a mixture of **6x** and **6x′** as yellow oil (65.9 mg, 0.13 mmol, 65%, **6x**/**6x′** = 2:3. *E/Z* > 20:1 for **6x**; **6x′** was isolated as single stereomer, but the exact *E*- or *Z*-isomer has not been confirmed in current stage). **^{1}H NMR** (500 MHz, $CDCl_3$) δ 10.19 (s, 0.6H), 10.12 (s, 0.4H), 8.79 - 8.75 (m, 2H), 8.17 - 8.13 (m, 1H), 7.74 (d, *J* = 8.0 Hz, 1.2H), 7.69 (d, *J* = 8.0 Hz, 0.8H), 7.56 - 7.48 (m, 2H), 7.46 - 7.42 (m, 1H), 7.28 (d, *J* = 8.0 Hz, 1.2H), 7.25 (d, *J* = 8.0 Hz, 0.8H), 5.58 (t, *J* = 7.5 Hz, 0.6H), 5.43 (s, 0.4H), 4.89 (t, *J* = 6.5 Hz, 0.6H), 4.85 (t, *J* = 6.5 Hz, 0.4H), 3.39 (s, 0.8H), 3.33 (s, 1.2H), 2.93 - 2.88 (m, 2H), 2.39 (s, 3H), 2.25 - 2.19 (m, 1.2H), 2.09 - 2.06 (m, 0.8H), 2.02 (s, 1.2H), 1.55 - 1.42 (m, 2.8H), 1.39 - 1.36 (m, 0.8H), 1.21 - 1.18 (m, 1.2H), 1.13 (s, 2.4H), 1.08 (t, *J* = 7.5 Hz, 1.8H), 0.88 - 0.84 (m, 4.8H). **^{13}C NMR** (125 MHz, $CDCl_3$) δ 169.8, 169.7, 148.43, 148.40, 143.33, 143.25, 138.81, 138.78, 138.7, 137.3, 136.8, 136.4, 136.3, 134.5, 134.4, 132.2, 130.1, 129.8, 129.7, 128.1, 127.5, 127.4, 127.22, 127.16, 121.79, 121.77, 121.70, 121.69, 116.6, 116.4, 48.7, 44.1, 44.0, 42.0, 41.73, 41.65, 40.4, 39.2, 35.8, 34.3, 29.3, 27.4, 25.2, 24.5, 22.1, 21.7, 21.6, 14.3, 13.8. **IR** ν_{max} (film): 3297, 2958, 2930, 2870, 1771, 1682, 1597, 1576, 1526, 1486, 1457, 1424, 1385, 1327, 1245, 1159, 1094, 912, 827, 814, 793, 740, 574, 551, 506 cm^{-1}. **HRMS** (ESI) m/z calcd for $C_{29}H_{37}N_3NaO_3S$ $[M+Na]^+$: 530.2448; found: 530.2439.

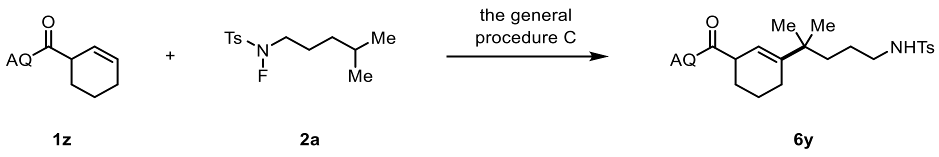

Product **6y** was prepared by the general procedure C. Purification using column chromatography (PE/EA = 5:1) afforded **6y** as yellow solid (87.9 mg, 0.17 mmol, 87%, *r.r.* > 20:1, *E/Z* > 20:1). **^{1}H NMR** (600 MHz, DMSO-d_6) δ 10.11 (s, 1H), 8.82 (dd, *J* = 4.2, 1.8 Hz, 1H), 8.65 (d, *J* = 7.8 Hz, 1H), 8.41 (dd, *J* = 8.4, 1.8 Hz, 1H), 7.66 (d, *J* = 7.8 Hz, 1H), 7.63 - 7.60 (m, 2H), 7.58 (t, *J* = 7.8 Hz, 1H), 7.45 (t, *J* = 6.0 Hz, 1H), 7.33 (d, *J* = 8.4 Hz, 2H), 5.61 (d, *J* = 3.6 Hz, 1H), 3.26 - 3.23 (m, 1H), 2.70 (dt, *J* = 6.6, 6.6 Hz, 2H), 2.34 (s, 3H), 2.01 - 1.91 (m, 2H), 1.87 - 1.77

(m, 2H), 1.63 - 1.54 (m, 2H), 1.33 - 1.30 (m, 2H), 1.27 - 1.22 (m, 2H), 1.08 (s, 3H), 1.03 (s, 3H). **^{13}C NMR** (150 MHz, DMSO-d_6) δ 173.5, 149.2, 148.6, 142.9, 138.32, 138.30, 137.1, 134.6, 130.0, 128.2, 127.5, 126.9, 122.7, 122.1, 117.3, 116.0, 44.5, 43.7, 38.7, 37.7, 27.5, 27.3, 26.6, 25.0, 24.3, 21.4, 21.3. **IR** ν_{max} (film): 3439, 2984, 1740, 1662, 1524, 1374, 1242, 1158, 1054, 1027, 1008, 823, 761, 626 , cm^{-1}. **HRMS** (ESI) m/z calcd for $C_{29}H_{35}N_3NaO_3S$ [M+Na]$^+$: 528.2291; found: 528.2285.

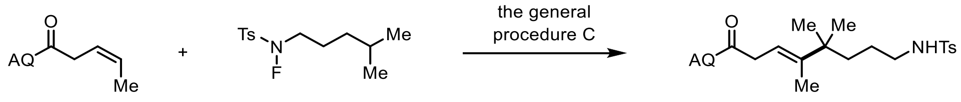

1aa **2a** **6z**

Product **6z** was prepared by the general procedure C. Purification using column chromatography (PE/EA = 5:1) afforded **6z** as yellow oil (75.7 mg, 0.16 mmol, 79%, *r.r.* > 20:1, *E/Z* > 20:1). **^{1}H NMR** (600 MHz, $CDCl_3$) δ 10.08 (s, 1H), 8.78 - 8.74 (m, 2H), 8.15 (d, J = 8.4 Hz, 1H), 7.69 (dd, J = 7.8, 1.8 Hz, 2H), 7.55 - 7.49 (m, 2H), 7.46 - 7.43 (m, 1H), 7.27 (d, J = 7.8 Hz, 2H), 5.55 (t, J = 7.8 Hz, 1H), 4.48 (t, J = 6.0 Hz, 1H), 3.28 (d, J = 7.8 Hz, 2H), 2.89 (dt, J = 6.6, 6.6 Hz, 2H), 2.41 (s, 3H), 1.66 (s, 3H), 1.41 - 1.32 (m, 4H), 1.13 (s, 6H). **^{13}C NMR** (150 MHz, $CDCl_3$) δ 170.2, 148.2, 147.8, 143.4, 138.7, 137.1, 136.5, 134.6, 129.8, 128.1, 127.5, 127.2, 121.8, 121.7, 116.5, 115.5, 44.0, 39.6, 37.9, 37.7, 27.2, 25.1, 21.6, 13.1. **IR** ν_{max} (film): 3299, 3213, 2918, 2868, 1651, 1594, 1523, 1420, 1316, 1300, 1154, 1094, 1083, 932, 868, 829, 822, 797, 766, 743, 659, 577, 551, 531, 490, 473, 439 cm^{-1}. **HRMS** (ESI) m/z calcd for $C_{27}H_{33}N_3NaO_3S$ [M+Na]$^+$: 502.2135; found: 502.2136.

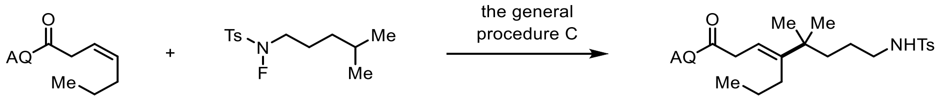

1ab **2a** **6aa**

Product **6aa** was prepared by the general procedure C. Purification using column chromatography (PE/EA = 5:1) afforded **6aa** as yellow oil (85.2 mg, 0.17 mmol, 84%, *r.r.* > 20:1, *E/Z* > 20:1). **^{1}H NMR** (600 MHz, $CDCl_3$) δ 10.07 (s, 1H), 8.77 - 8.74 (m, 2H), 8.14 (dd, J = 8.4, 1.2 Hz, 1H), 7.70 (d, J = 8.4 Hz, 2H), 7.53 - 7.48 (m, 2H), 7.43 (dd, J = 8.4, 4.2 Hz, 1H), 7.24 (d, J = 8.4 Hz, 2H), 5.52 (t, J = 7.8 Hz, 1H), 4.92 - 4.89 (m, 1H), 3.27 (d, J = 7.8 Hz, 2H), 2.90 - 2.87 (m, 2H), 2.39 (s, 3H), 2.01 - 1.98 (m, 2H), 1.41 - 1.35 (m, 6H), 1.10 (s, 6H), 0.91 (t, J = 7.2 Hz, 3H). **^{13}C NMR** (150 MHz, $CDCl_3$) δ 170.5, 151.7, 148.2, 143.3, 138.6, 137.2, 136.4, 134.5, 129.7, 128.0, 127.5, 127.2, 121.8, 121.7, 116.4, 116.2, 44.0, 39.9, 38.0, 37.9, 30.6, 27.3, 25.1, 24.0, 21.6, 15.0. **IR** ν_{max} (film): 2913, 2846, 1648, 1526, 1458, 1326, 1154, 1096, 1019, 728, 717, 669, 659,

585, 574, 551 cm^{-1}. **HRMS** (ESI) m/z calcd for $C_{29}H_{37}N_3NaO_3S$ $[M+Na]^+$: 530.2448; found: 530.2448.

The general procedure D (for products 4a-4az, 7a, 7b, 8):

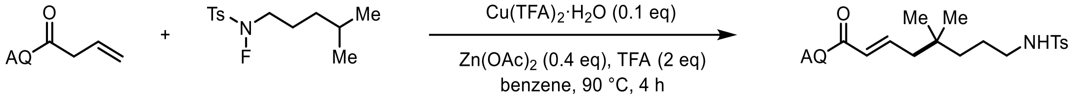

1a **2a** **4a**

To a dry Schlenk flask were added **1a** (42.4 mg, 0.20 mmol, 1.0 equiv), **2a** (137 mg, 0.50 mmol, 2.5 equiv), $Cu(TFA)_2{\cdot}H_2O$ (5.8 mg, 0.02 mmol, 0.10 equiv), $Zn(OAc)_2$ (14.7 mg, 0.08 mmol, 0.40 equiv), TFA (30 uL, 0.40 mmol, 2.0 equiv), and anhydrous benzene (2.0 mL). The mixture was degassed for three times with argon and stirred at 90 °C (oil bath) for 4 h. Once completion, the reaction was cooled to room temperature. The reaction mixture was filtered by celite, and the filtrate was concentrated *in vacuo*. Further purification by a flash column chromatography using eluents (PE/EA = 5:1) afforded the desired product **4a** as yellow oil (65.1 mg, 0.14 mmol, 70%, *r.r.* > 20:1, *E/Z* > 20:1). **^{1}H NMR** (600 MHz, $CDCl_3$) δ 9.84 (s, 1H), 8.84 (dd, *J* = 7.8, 1.8 Hz, 1H), 8.80 (dd, *J* = 4.2, 1.8 Hz, 1H), 8.16 (dd, *J* = 7.8, 1.2 Hz, 1H), 7.75 (d, *J* = 8.4 Hz, 2H), 7.55 - 7.51 (m, 2H), 7.45 (dd, *J* = 7.8, 4.2 Hz, 1H), 7.28 (d, *J* = 8.4 Hz, 2H), 7.00 (dt, *J* = 15.0, 7.8 Hz, 1H), 6.14 (d, *J* = 15.0 Hz, 1H), 4.80 (t, *J* = 6.6 Hz, 1H), 2.91 (dt, *J* = 6.6, 6.6 Hz, 2H), 2.39 (s, 3H), 2.10 (d, *J* = 7.8 Hz, 2H), 1.47 - 1.42 (m, 2H), 1.90 - 1.62 (m, 2H), 0.87 (s, 6H). **^{13}C NMR** (150 MHz, $CDCl_3$) δ 164.1, 148.3, 143.4, 143.2, 138.6, 137.2, 136.5, 134.7, 129.8, 128.1, 127.5, 127.2, 126.9, 121.8, 121.7, 116.8, 44.8, 44.1, 38.9, 33.7, 27.0, 24.6, 21.6. **IR** ν_{max} (film): 3324, 2956, 2869, 1675, 1638, 1596, 1527, 1486, 1425, 1386, 1328, 1260, 1184, 1158, 1094, 978, 827, 815, 792, 758, 610, 574, 551 cm^{-1}. **HRMS** (ESI) m/z calcd for $C_{26}H_{31}N_3NaO_3S$ $[M+Na]^+$: 488.1978; found: 488.1975.

Lagre-scale synthesis of 4a:

To a dry Schlenk flask were added **1a** (1.06 g, 5.0 mmol, 1.0 equiv), **2a** (3.41 g, 12.5 mmol, 2.5 equiv), $Cu(TFA)_2{\cdot}H_2O$ (145 mg, 0.50 mmol, 0.10 equiv), $Zn(OAc)_2$ (367 mg, 2.0 mmol, 0.40 equiv), TFA (0.74 mL, 10.0 mmol, 2.0 equiv), and anhydrous benzene (50 mL). The mixture was degassed for three times with argon and stirred at 90 °C (oil bath) for 4 h. Once completion, the reaction was cooled to room temperature. The reaction mixture was filtered by celite, and the filtrate was concentrated *in vacuo*. Further purification by a flash column chromatography using eluents (PE/EA = 5:1) afforded the desired product **4a** as yellow oil (1.42 g, 3.05 mmol, 61%, *r.r.* >

20:1, *E/Z* > 20:1).

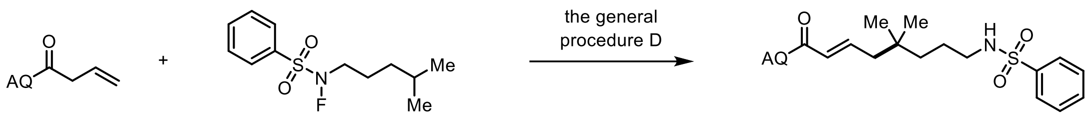

1a **2b** **4b**

Product **4b** was prepared by the general procedure D. Purification using column chromatography (PE/EA = 5:1) afforded **4b** as yellow oil (58.7 mg, 0.13 mmol, 65%, *r.r.* > 20:1, *E/Z* > 20:1). **^{1}H NMR** (600 MHz, $CDCl_3$) δ 9.76 (s, 1H), 8.75 (dd, *J* = 7.2, 1.2 Hz, 1H), 8.72 (dd, *J* = 4.2, 1.2 Hz, 1H), 8.07 (dd, *J* = 8.4, 1.8 Hz, 1H), 7.80 (d, *J* = 7.2 Hz, 2H), 7.47 - 7.44 (m, 2H), 7.43 - 7.39 (m, 3H), 7.37 (dd, *J* = 7.8, 4.2 Hz, 1H), 6.92 (dt, *J* = 15.0, 7.8 Hz, 1H), 6.05 (d, *J* = 15.0 Hz, 1H), 4.96 (t, *J* = 6.6 Hz, 1H), 2.85 (dt, *J* = 6.6, 6.6 Hz, 2H), 2.00 (d, *J* = 7.8 Hz, 2H), 1.39 - 1.34 (m, 2H), 1.10 - 1.08 (m, 2H), 0.78 (s, 6H). **^{13}C NMR** (150 MHz, $CDCl_3$) δ 164.1, 148.3, 143.3, 140.2, 138.5, 136.5, 134.7, 132.6, 129.2, 128.0, 127.5, 127.1, 126.8, 121.8, 121.7, 116.8, 44.7, 44.1, 38.8, 33.7, 27.0, 24.6. **IR** ν_{max} (film): 3349, 2956, 1771, 1670, 1636, 1526, 1487, 1424, 1387, 1327, 1247, 1159, 1094, 912, 826, 792, 720, 586 cm^{-1}. **HRMS** (ESI) m/z calcd for $C_{25}H_{29}N_3NaO_3S$ $[M+Na]^+$: 474.1822; found: 474.1827.

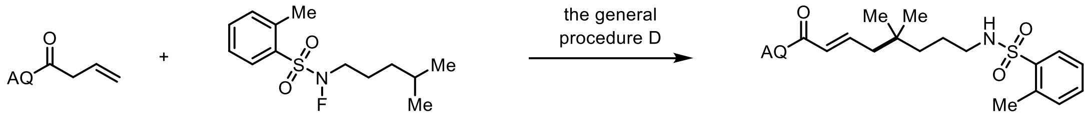

1a **2c** **4c**

Product **4c** was prepared by the general procedure D. Purification using column chromatography (PE/EA = 5:1) afforded **4c** as yellow oil (56.8 mg, 0.12 mmol, 61%, *r.r.* > 20:1, *E/Z* > 20:1). **^{1}H NMR** (600 MHz, $CDCl_3$) δ 9.84 (s, 1H), 8.85 (dd, *J* = 7.8, 1.2 Hz, 1H), 8.81 (dd, *J* = 4.2, 1.8 Hz, 1H), 8.17 (dd, *J* = 8.4, 1.8 Hz, 1H), 7.98 - 7.96 (m, 1H), 7.57 - 7.51 (m, 2H), 7.46 (dd, *J* = 8.4, 4.2 Hz, 1H), 7.45 - 7.42 (m, 1H), 7.30 (d, *J* = 7.2 Hz, 2H), 7.00 (dt, *J* = 15.0, 7.8 Hz, 1H), 6.14 (d, *J* = 15.0 Hz, 1H), 4.62 - 4.57 (m, 1H), 2.94 (dt, *J* = 6.6, 6.6 Hz, 2H), 2.65 (s, 3H), 2.10 (dd, *J* = 7.8, 1.8 Hz, 2H), 1.47 - 1.41 (m, 2H), 1.17 - 1.14 (m, 2H), 0.87 (s, 6H). **^{13}C NMR** (150 MHz, $CDCl_3$) δ 164.1, 148.3, 143.2, 138.6, 138.2, 137.1, 136.6, 134.8, 132.8, 132.7, 129.7, 128.1, 127.6, 126.9, 126.3, 121.8, 121.7, 116.9, 44.9, 43.9, 38.9, 33.8, 27.1, 24.8, 20.5. **IR** νmax (film): 3350, 2956, 1672, 1640, 1596, 1527, 1487, 1425, 1327, 1093, 913, 815, 792, 610, 586, 551 cm^{-1}. **HRMS** (ESI) m/z calcd for $C_{26}H_{31}N_3NaO_3S$ $[M+Na]^+$: 488.1978; found: 488.1985.

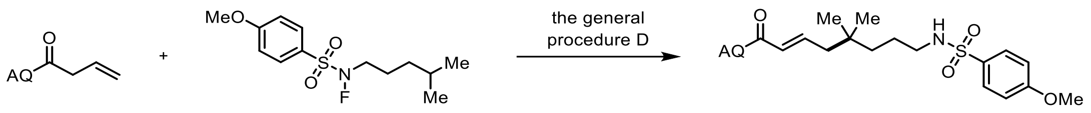

1a **2d** **4d**

Product **4d** was prepared by the general procedure D. Purification using column chromatography (PE/EA = 3:1) afforded **4d** as yellow oil (64.5 mg, 0.13 mmol, 67%, *r.r.* > 20:1, *E/Z* > 20:1). **^{1}H NMR** (600 MHz, $CDCl_3$) δ 9.84 (s, 1H), 8.83 (d, J = 7.8 Hz, 1H), 8.80 (dd, J = 4.2, 1.8 Hz, 1H), 8.16 (dd, J = 8.4 Hz, 1H), 7.80 (d, J = 8.4 Hz, 2H), 7.55 - 7.49 (m, 2H), 7.45 (dd, J = 8.4, 4.2 Hz, 1H), 7.01 (dt, J = 15.0, 7.8 Hz, 1H), 6.95 (d, J = 8.4 Hz, 2H), 6.14 (d, J = 15.0 Hz, 1H), 4.86 (t, J = 6.6 Hz, 1H), 3.82 (s, 3H), 2.90 (dt, J = 6.6, 6.6 Hz, 2H), 2.10 (d, J = 7.8 Hz, 2H), 1.48 - 1.42 (m, 2H), 1.19 - 1.16 (m, 2H), 0.87 (s, 6H). **^{13}C NMR** (150 MHz, $CDCl_3$) δ 164.1, 162.9, 148.3, 143.3, 138.5, 136.5, 134.7, 131.7, 129.3, 128.0, 127.5, 126.8, 121.8, 121.7, 116.8, 114.3, 55.7, 44.7, 44.0, 38.9, 33.7, 27.0, 24.5. **IR** ν_{max} (film): 3337, 2956, 1675, 1637, 1596, 1577, 1527, 1497, 1486, 1425, 1386, 1328, 1258, 1155, 1095, 1026, 979, 913, 828, 793, 740, 616, 561 cm^{-1}. **HRMS** (ESI) m/z calcd for $C_{26}H_{31}N_3NaO_4S$ $[M+Na]^+$: 504.1927; found: 504.1936.

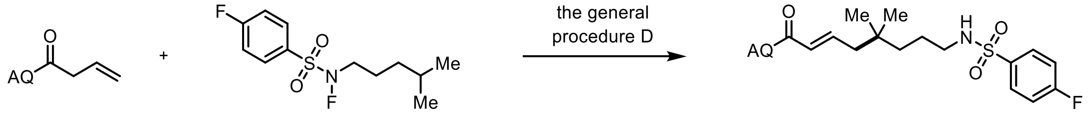

1a **2e** **4e**

Product **4e** was prepared by the general procedure D. Purification using column chromatography (PE/EA = 5:1) afforded **4e** as yellow oil (68.5 mg, 0.15 mmol, 73%, *r.r.* > 20:1, *E/Z* > 20:1). **^{1}H NMR** (600 MHz, $CDCl_3$) δ 9.84 (s, 1H), 8.83 (dd, J = 7.8, 1.2 Hz, 1H), 8.81 (dd, J = 4.2, 1.8 Hz, 1H), 8.17 (dd, J = 8.4, 1.8 Hz, 1H), 7.89 - 7.86 (m, 2H), 7.56 - 7.51 (m, 2H), 7.46 (dd, J = 8.4, 4.2 Hz, 1H), 7.19 - 7.15 (m, 2H), 7.01 (dt, J = 15.0, 7.8 Hz, 1H), 6.15 (dd, J = 15.0 Hz, 1H), 4.72 (t, J = 6.6 Hz, 1H), 2.94 (dt, J = 6.6, 6.6 Hz, 2H), 2.12 (dd, J = 7.8, 1.8 Hz, 2H), 1.50 - 1.45 (m, 2H), 1.21 - 1.18 (m, 2H), 0.89 (s, 6H). **^{13}C NMR** (150 MHz, $CDCl_3$) δ 165.2 (d, J = 253.1 Hz), 164.1, 148.3, 143.2, 138.6, 136.6, 136.3 (d, J = 3.0 Hz), 134.7, 129.9 (d, J = 9.0 Hz), 128.1, 127.6, 126.9, 121.8, 121.7, 116.9, 116.4 (d, J = 22.4 Hz), 44.8, 44.1, 38.8, 33.8, 27.1, 24.7. **^{19}F NMR** (470 MHz, $CDCl_3$) δ -105.4 - (-105.5) (m). **IR** ν_{max} (film): 3349, 2920, 2851, 1672, 1640, 1592, 1527, 1485, 1425, 1329, 1291, 1237, 1165, 1153, 1092, 976, 827, 791, 610, 549 cm^{-1}. **HRMS** (ESI) m/z calcd for $C_{25}H_{28}FN_3NaO_3S$ $[M+Na]^+$: 492.1728; found: 492.1730.

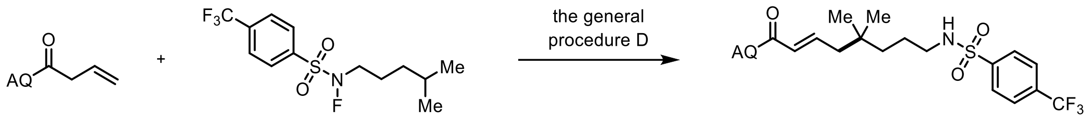

1a **2f** **4f**

Product **4f** was prepared by the general procedure D. Purification using column chromatography (PE/EA = 3:1) afforded **4f** as yellow oil (67.5 mg, 0.13 mmol, 65%, *r.r.* > 20:1, *E/Z* > 20:1). **^{1}H NMR** (600 MHz, $CDCl_3$) δ 9.77 (s, 1H), 8.73 - 8.70 (m, 2H), 8.07 (dd, *J* = 8.4, 1.8 Hz, 1H), 7.92 (d, *J* = 8.4 Hz, 2H), 7.65 (d, *J* = 7.8 Hz, 2H), 7.46 - 9.41 (m, 2H), 7.36 (dd, *J* = 8.4, 4.2 Hz, 1H), 6.93 (dt, *J* = 15.0, 7.8 Hz, 1H), 6.06 (d, *J* = 15.0 Hz, 1H), 5.32 (t, *J* = 6.6 Hz, 1H), 2.88 (dt, *J* = 6.6, 6.6 Hz, 2H), 2.01 (d, *J* = 7.8 Hz, 2H), 1.42 - 1.37 (m, 2H), 1.12 - 1.09 (m, 2H), 0.78 (s, 6H). **^{13}C NMR** (150 MHz, $CDCl_3$) δ 164.2, 148.3, 143.9, 143.3, 138.5, 136.5, 134.6, 134.3 (q, *J* = 32.9 Hz), 128.1, 127.7, 127.5, 126.8, 126.3 (q, *J* = 3.0 Hz), 123.4 (q, *J* = 271.5 Hz), 121.82, 121.78, 116.8, 44.7, 44.1, 38.7, 33.7, 27.1, 24.6. **^{19}F NMR** (470 MHz, $CDCl_3$) δ -63.1 (s). **IR** ν_{max} (film): 3341, 2958, 2254, 1672, 1637, 1608, 1596, 1528, 1487, 1425, 1404, 1323, 1261, 1166, 1134, 1107, 1096, 1062, 1016, 979, 910, 842, 826, 791, 734, 711, 650, 607, 428 cm^{-1}. **HRMS** (ESI) m/z calcd for $C_{26}H_{28}F_3N_3NaO_3S$ $[M+Na]^+$: 542.1696; found: 542.1705.

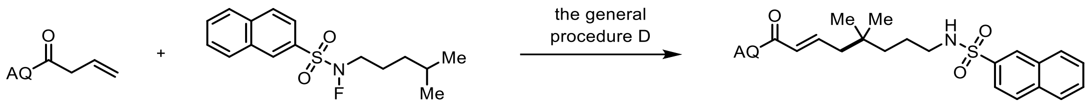

1a **2g** **4g**

Product **4g** was prepared by the general procedure D. Purification using column chromatography (PE/EA = 5:1) afforded **4g** as yellow oil (62.1 mg, 0.12 mmol, 62%, *r.r.* > 20:1, *E/Z* > 20:1). **^{1}H NMR** (600 MHz, $CDCl_3$) δ 9.82 (s, 1H), 8.84 - 8.79 (m, 2H), 8.44 (s, 1H), 8.15 (dd, *J* = 8.4, 1.8 Hz, 1H), 7.95 - 7.92 (m, 2H), 7.87 (d, *J* = 8.4 Hz, 1H), 7.85 (dd, *J* = 9.0, 1.8 Hz, 1H), 7.63 - 7.60 (m, 1H), 7.59 - 7.56 (m, 1H), 7.55 - 7.49 (m, 2H), 7.44 (dd, *J* = 8.4, 4.2 Hz, 1H), 6.98 (dt, *J* = 15.0, 7.8 Hz, 1H), 6.08 (d, *J* = 15.0 Hz, 1H), 4.87 (t, *J* = 6.6 Hz), 2.98 (dt, *J* = 6.6, 6.6 Hz, 2H), 2.04 (d, *J* = 7.8 Hz, 2H), 1.48 - 1.43 (m, 2H), 1.18 - 1.15 (m, 2H), 0.83 (s, 6H). **^{13}C NMR** (150 MHz, $CDCl_3$) δ 164.1, 148.3, 143.2, 138.6, 137.0, 136.5, 134.9, 134.7, 132.3, 129.6, 129.3, 128.8, 128.5, 128.1, 128.0, 127.61, 127.56, 126.8, 122.5, 121.8, 121.7, 116.8, 44.7, 44.1, 38.9, 33.7, 27.0, 24.7. **IR** ν_{max} (film): 2926, 1683, 1636, 1595, 1525, 1486, 1436, 1327, 1239, 1157, 1093, 913, 815, 720, 658, 617, 541, 418 cm^{-1}. **HRMS** (ESI) m/z calcd for $C_{29}H_{31}N_3NaO_3S$ $[M+Na]^+$: 524.1978; found: 524.1976.

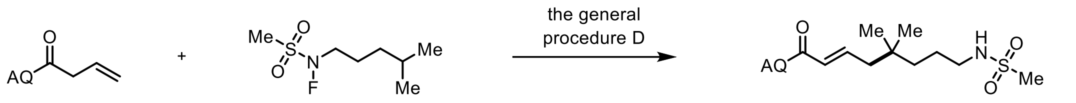

1a **2h** **4h**

Product **4h** was prepared by the general procedure D. Purification using column chromatography (PE/EA = 5:1) afforded **4h** as yellow oil (45.1 mg, 0.12 mmol, 58%, *r.r.* > 20:1, *E/Z* > 20:1). **^{1}H NMR** (600 MHz, $CDCl_3$) δ 9.85 (s, 1H), 8.84 (dd, *J* = 7.8, 1.8 Hz, 1H), 8.81 (dd, *J* = 4.2, 1.8 Hz, 1H), 8.16 (dd, *J* = 8.4, 1.8 Hz, 1H), 7.56 - 7.49 (m, 2H), 7.45 (dd, *J* = 8.4, 4.8 Hz, 1H), 7.06 (dt, *J* = 15.0, 7.8 Hz, 1H), 6.18 (d, *J* = 15.0, 1H), 4.74 (t, *J* = 6.6 Hz, 1H), 3.11 (dt, *J* = 6.6, 6.6 Hz, 2H), 2.95 (s, 3H), 2.17 (d, *J* = 7.8 Hz, 2H), 1.60 - 1.55 (m, 2H), 1.31 - 1.28 (m, 2H), 0.95 (s, 6H). **^{13}C NMR** (150 MHz, $CDCl_3$) δ 164.1, 148.3, 143.2, 138.6, 136.5, 134.7, 128.1, 127.5, 126.9, 121.8, 121.7, 116.8, 44.8, 44.1, 40.3, 38.9, 33.8, 27.2, 25.2. **IR** ν_{max} (film): 3342, 2930, 2849, 1770, 1758, 1717, 1683, 1652, 1637, 1595, 1527, 1486, 1457, 1425, 1386, 1321, 1246, 1151, 1064, 976, 912, 827, 792, 749, 610, 521, 418 cm^{-1}. **HRMS** (ESI) m/z calcd for $C_{20}H_{27}N_3NaO_3S$ $[M+Na]^+$: 412.1665; found: 412.1656.

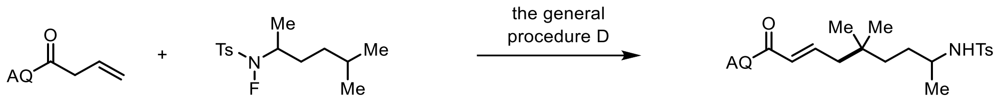

1a **2i** **4i**

Product **4i** was prepared by the general procedure D. Purification using column chromatography (PE/EA = 5:1) afforded **4i** as yellow oil (64.2 mg, 0.13 mmol, 67%, *r.r.* > 20:1, *E/Z* > 20:1). **^{1}H NMR** (600 MHz, $CDCl_3$) δ 9.85 (s, 1H), 8.86 (dd, *J* = 7.2, 1.2 Hz, 1H), 8.81 (dd, *J* = 4.2, 1.8 Hz, 1H), 8.16 (dd, *J* = 8.4, 1.8 Hz, 1H), 7.77 (d, *J* = 8.4 Hz, 2H), 7.56 - 7.49 (m, 2H), 7.45 (dd, *J* = 8.4, 4.2 Hz, 1H), 7.28 (d, *J* = 8.4 Hz, 2H), 6.98 (dt, *J* = 15.0, 7.8 Hz, 1H), 6.13 (d, *J* = 15.0 Hz, 1H), 4.57 (d, *J* = 8.4 Hz, 1H), 3.28 - 3.23 (m, 1H), 2.38 (s, 3H), 2.07 (d, *J* = 7.8 Hz, 2H), 1.39 - 1.31 (m, 2H), 1.18 - 1.13 (m, 1H), 1.09 - 1.05 (m, 1H), 1.04 (d, *J* = 6.6 Hz, 3H), 0.84 (s, 6H). **^{13}C NMR** (150 MHz, $CDCl_3$) δ 164.1, 148.3, 143.3, 143.2, 138.6, 138.4, 136.5, 134.8, 129.7, 128.1, 127.6, 127.2, 126.9, 121.8, 121.7, 116.8, 50.8, 44.6, 37.8, 33.6, 32.1, 27.03, 27.00, 21.9, 21.6. **IR** ν_{max} (film): 3350, 2956, 1667, 1641, 1631, 1526, 1485, 1461, 1425, 1382, 1327, 1158, 1093, 979, 826, 791, 580, 551 cm^{-1}. **HRMS** (ESI) m/z calcd for $C_{27}H_{33}N_3NaO_3S$ $[M+Na]^+$: 502.2135; found: 502.2132.

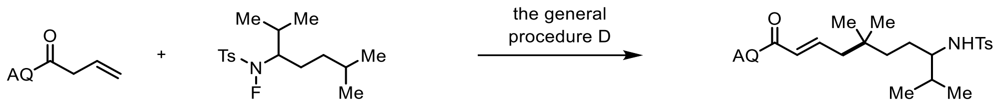

1a **2j** **4j**

Product **4j** was prepared by the general procedure D. Purification using column chromatography (PE/EA = 5:1) afforded **4j** as yellow oil (63.9 mg, 0.13 mmol, 63%, *r.r.* = 20:1, *E/Z* > 20:1). **^{1}H NMR** (600 MHz, $CDCl_3$) δ 9.85 (s, 1H), 8.86 (dd, *J* = 7.2, 1.2 Hz, 1H), 8.80 (dd, *J* = 4.2, 1.8 Hz, 1H), 8.16 (dd, *J* = 8.4, 1.8 Hz, 1H), 7.77 (d, *J* = 8.4 Hz, 2H), 7.57 - 7.49 (m, 2H), 7.45 (dd, *J* = 8.4, 4.2 Hz, 1H), 7.28 (d, *J* = 8.4 Hz, 2H), 6.94 (dt, *J* = 15.0, 7.8 Hz, 1H), 6.11 (d, *J* = 15.0 Hz, 1H), 4.58 (d, *J* = 9.0 Hz, 1H), 3.04 - 2.99 (m, 1H), 2.38 (s, 3H), 2.00 (d, *J* = 7.8 Hz, 2H), 1.78 - 1.71 (m, 1H), 1.38 - 1.34 (m, 1H), 1.22 - 1.16 (m, 1H), 0.99 - 0.93 (m, 2H), 0.83 (d, *J* = 6.6 Hz, 3H), 0.81 (d, *J* = 6.6 Hz, 3H), 0.78 (s, 3H), 0.77 (s, 3H). **^{13}C NMR** (150 MHz, $CDCl_3$) δ 164.0, 148.3, 143.18, 143.15, 138.7, 138.6, 136.5, 134.8, 129.7, 128.1, 127.6, 127.2, 126.9, 121.74, 121.65, 116.8, 60.1, 44.5, 37.9, 33.7, 31.1, 27.0, 26.9, 26.5, 21.5, 18.9, 17.4. **IR** ν_{max} (film): 3286, 2958, 1677, 1640, 1596, 1526, 1485, 1462, 1425, 1386, 1326, 1261, 1158, 1093, 1023, 976, 885, 826, 791, 757, 590, 549 cm^{-1}. **HRMS** (ESI) m/z calcd for $C_{29}H_{37}N_3NaO_3S$ $[M+Na]^+$: 530.2448; found: 530.2434.

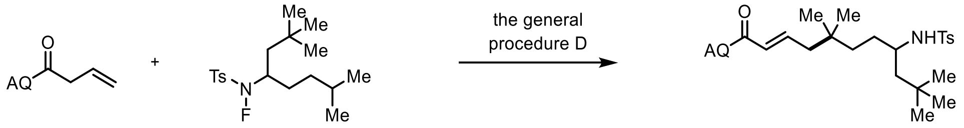

1a **2k** **4k**

Product **4k** was prepared by the general procedure D. Purification using column chromatography (PE/EA = 5:1) afforded **4k** as yellow oil (64.2 mg, 0.12 mmol, 60%, *r.r.* > 20:1, *E/Z* > 20:1). **^{1}H NMR** (600 MHz, $CDCl_3$) δ 9.86 (s, 1H), 8.86 (dd, *J* = 7.2, 1.2 Hz, 1H), 8.79 (dd, *J* = 4.2, 1.8 Hz, 1H), 8.15 (dd, *J* = 8.4, 1.8 Hz, 1H), 7.78 (d, *J* = 8.4 Hz, 2H), 7.56 - 7.49 (m, 2H), 7.44 (dd, *J* = 8.4, 4.2 Hz, 1H), 7.27 (d, *J* = 8.4 Hz, 2H), 6.97 (dt, *J* = 15.0, 7.8 Hz, 1H), 6.13 (d, *J* = 15.0 Hz, 1H), 4.66 (d, *J* = 8.4 Hz, 1H), 3.36 - 3.32 (m, 1H), 2.38 (s, 3H), 2.01 - 1.99 (m, 2H), 1.34 - 1.28 (m, 4H), 1.25 - 1.23 (m, 1H), 1.08 - 1.05 (m, 1H), 0.85 (s, 9H), 0.77 (s, 3H), 0.75 (s, 3H). **^{13}C NMR** (150 MHz, $CDCl_3$) δ 164.0, 148.2, 143.23, 143.18, 138.9, 138.5, 136.5, 134.7, 129.7, 128.0, 127.5, 127.2, 126.8, 121.7, 121.6, 116.8, 52.1, 49.1, 44.6, 37.2, 33.5, 31.5, 30.5, 30.0, 27.0, 26.9, 21.5. **IR** ν_{max} (film): 3346, 2967, 2954, 1683, 1652, 1646, 1636, 1558, 1526, 1507, 1473, 1457, 1424, 1386, 1325, 1260, 1156, 1093, 826, 792, 749, 581, 549, 418 cm^{-1}. **HRMS** (ESI) m/z calcd for $C_{31}H_{41}N_3NaO_3S$ $[M+Na]^+$: 558.2761; found: 558.2765.

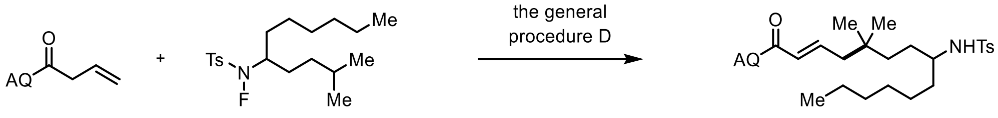

1a **2l** **4l**

Product **4l** was prepared by the general procedure D. Purification using column chromatography (PE/EA = 5:1) afforded **4l** as yellow oil (74.7 mg, 0.14 mmol, 68%, *r.r.* = 20:1, *E/Z* > 20:1). **^{1}H NMR** (600 MHz, $CDCl_3$) δ 9.85 (s, 1H), 8.86 (dd, *J* = 7.8, 1.2 Hz, 1H), 8.80 (dd, *J* = 4.8, 1.8 Hz, 1H), 8.16 (dd, *J* = 8.4, 1.8 Hz, 1H), 7.76 (d, *J* = 8.4 Hz, 2H), 7.57 - 7.49 (m, 2H), 7.45 (dd, *J* = 8.4, 4.2 Hz, 1H), 7.27 (d, *J* = 8.4 Hz, 2H), 6.98 (dt, *J* = 15.0, 7.8 Hz, 1H), 6.14 (d, *J* = 15.0 Hz, 1H), 4.51 (d, *J* = 9.0 Hz, 1H), 3.19 - 3.13 (m, 1H), 2.39 (s, 3H), 2.05 (d, *J* = 7.8 Hz, 2H), 1.42 - 1.34 (m, 3H), 1.32 - 1.26 (m, 3H), 1.19 - 1.16 (m, 3H), 1.11 - 1.02 (m, 5H), 0.84 - 0.80 (m, 9H). **^{13}C NMR** (150 MHz, $CDCl_3$) δ 164.1, 148.3, 143.3, 143.2, 138.61, 138.59, 136.5, 134.8, 129.7, 128.1, 127.6, 127.2, 126.9, 121.7, 121.6, 116.8, 54.9, 44.6, 37.4, 35.1, 33.6, 31.8, 29.7, 29.1, 27.04, 27.00, 25.4, 22.6, 21.5, 14.1. **IR** ν_{max} (film): 3483, 2953, 2928, 2857, 1758, 1677, 1640, 1597, 1527, 1486, 1461, 1425, 1385, 1327, 1246, 1158, 1093, 1049, 978, 913, 826, 814, 791, 742 cm^{-1}. **HRMS** (ESI) m/z calcd for $C_{32}H_{43}N_3NaO_3S$ $[M+Na]^+$: 572.2917; found: 572.2932.

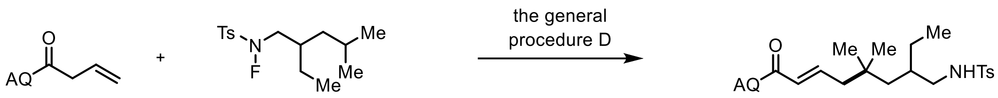

1a **2m** **4m**

Product **4m** was prepared by the general procedure D. Purification using column chromatography (PE/EA = 5:1) afforded **4m** as yellow oil (72.0 mg, 0.15 mmol, 73%, *r.r.* > 20:1, *E/Z* > 20:1). **^{1}H NMR** (600 MHz, $CDCl_3$) δ 9.84 (s, 1H), 8.85 (dd, *J* = 7.2, 1.2 Hz, 1H), 8.81 (dd, *J* = 4.2, 1.8 Hz, 1H), 8.16 (dd, *J* = 8.4, 1.8 Hz, 1H), 7.75 (d, *J* = 8.4 Hz, 2H), 7.56 - 7.49 (m, 2H), 7.45 (dd, *J* = 7.8, 4.2 Hz, 1H), 7.28 (d, *J* = 8.4 Hz, 2H), 7.03 (dt, *J* = 15.0, 7.8 Hz, 1H), 6.14 (d, *J* = 15.0 Hz, 1H), 4.76 - 4.73 (m, 1H), 2.92 - 2.88 (m, 1H), 2.79 - 2.75 (m, 1H), 2.39 (s, 3H), 2.12 (d, *J* = 7.8 Hz, 2H), 1.51 - 1.46 (m, 1H), 1.39 - 1.34 (m, 1H), 1.30 - 1.26 (m, 1H), 1.18 - 1.15 (m, 1H), 1.13 - 1.09 (m, 1H), 0.89 (s, 3H), 0.88 (s, 3H), 0.80 (t, *J* = 7.2 Hz, 3H). **^{13}C NMR** (150 MHz, $CDCl_3$) δ 164.1, 148.3, 143.4, 143.2, 138.6, 137.2, 136.5, 134.8, 129.8, 128.1, 127.6, 127.3, 127.0, 121.8, 121.7, 116.8, 47.5, 45.6, 43.7, 35.4, 34.6, 27.12, 27.10, 26.3, 21.6, 10.8. **IR** ν_{max} (film): 3344, 2959, 2927, 2872, 1735, 1675, 1638, 1596, 1527, 1486, 1460, 1425, 1386, 1327, 1260, 1184, 1159, 1093, 978, 885, 827, 814, 792, 757, 610, 551, 418 cm^{-1}. **HRMS** (ESI) m/z calcd for $C_{28}H_{35}N_3NaO_3S$ $[M+Na]^+$: 516.2291; found: 516.2301.

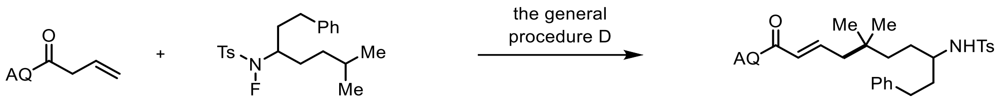

1a **2n** **4n**

Product **4n** was prepared by the general procedure D. Purification using column chromatography (PE/EA = 5:1) afforded **4n** as yellow oil (85.4 mg, 0.15 mmol, 75%, *r.r.* > 20:1, *E/Z* > 20:1). **^{1}H NMR** (600 MHz, $CDCl_3$) δ 9.85 (s, 1H), 8.86 (dd, *J* = 7.2, 1.2 Hz, 1H), 8.79 (dd, *J* = 4.2, 1.8 Hz, 1H), 8.15 (dd, *J* = 7.8, 1.2 Hz, 1H), 7.76 (d, *J* = 8.4 Hz, 2H), 7.56 - 7.49 (m, 2H), 7.44 (dd, *J* = 8.4, 4.2 Hz, 1H), 7.27 (d, *J* = 8.4 Hz, 2H), 7.22 - 7.19 (m, 2H), 7.14 - 7.12 (m, 1H), 7.03 (d, *J* = 7.8 Hz, 2H), 6.97 (dt, *J* = 15.0, 7.8 Hz, 1H), 6.12 (d, *J* = 15.0 Hz, 1H), 4.65 (d, *J* = 8.4 Hz, 1H), 3.26 - 3.20 (m, 1H), 2.62 - 2.58 (m, 1H), 2.51 - 2.46 (m, 1H), 2.39 (s, 3H), 2.02 (d, *J* = 7.8 Hz, 2H), 1.77 - 1.71 (m, 1H), 1.65 - 1.59 (m, 1H), 1.44 - 1.37 (m, 1H), 1.35 - 1.29 (m, 1H), 1.10 - 1.05 (m, 1H), 1.04 - 0.99 (m, 1H), 0.80 (s, 3H), 0.79 (s, 3H). **^{13}C NMR** (150 MHz, $CDCl_3$) δ 164.0, 148.3, 143.4, 143.1, 141.5, 138.6, 138.5, 136.5, 134.8, 129.8, 128.5, 128.4, 128.1, 127.6, 127.2, 126.9, 126.0, 121.8, 121.7, 116.8, 54.6, 44.6, 37.4, 37.0, 33.6, 31.9, 29.7, 26.98, 26.95, 21.6. **IR** ν_{max} (film): 3436, 2926, 1677, 1640, 1597, 1526, 1485, 1460, 1452, 1425, 1387, 1327, 1260, 1156, 1092, 977, 826, 791, 755, 699, 577, 550 cm^{-1}. **HRMS** (ESI) m/z calcd for $C_{34}H_{39}N_3NaO_3S$ $[M+Na]^+$: 592.2604; found: 592.2609.

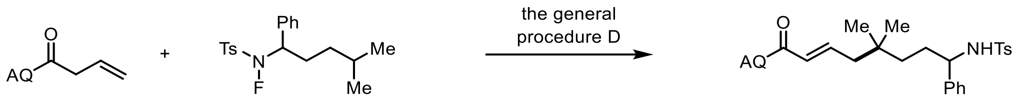

1a **2o** **4o**

Product **4o** was prepared by the general procedure D. Purification using column chromatography (PE/EA = 5:1) afforded **4o** as yellow oil (86.6 mg, 0.16 mmol, 80%, *r.r.* > 20:1, *E/Z* > 20:1). **^{1}H NMR** (600 MHz, $CDCl_3$) δ 9.82 (s, 1H), 8.85 (dd, *J* = 7.8, 1.8 Hz, 1H), 8.81 (dd, *J* = 4.2, 1.8 Hz, 1H), 8.16 (dd, *J* = 8.4, 1.8 Hz, 1H), 7.56 - 7.54 (m, 3H), 7.51 - 7.49 (m, 1H), 7.45 (dd, *J* = 8.4, 4.2 Hz, 1H), 7.15 - 7.11 (m, 3H), 7.09 (d, *J* = 8.4 Hz, 2H), 7.03 - 7.02 (m, 2H), 6.95 (dt, *J* = 15.0, 7.8 Hz, 1H), 6.08 (d, *J* = 15.0 Hz, 1H), 5.26 - 5.25 (m, 1H), 4.19 (dt, *J* = 7.2, 7.2 Hz, 1H), 2.32 (s, 3H), 2.04 (d, *J* = 7.8 Hz, 2H), 1.81 - 1.75 (m, 1H), 1.71 - 1.65 (m, 1H), 1.26 - 1.21 (m, 1H), 0.99 - 0.94 (m, 1H), 0.83 (s, 3H), 0.82 (s, 3H). **^{13}C NMR** (150 MHz, $CDCl_3$) δ 164.0, 148.3, 143.1, 143.0, 141.0, 138.6, 137.9, 136.5, 134.8, 129.4, 128.6, 128.1, 127.6, 127.5, 127.2, 126.9, 126.6, 121.8, 121.6, 116.8, 59.2, 44.5, 38.2, 33.7, 32.3, 27.1, 27.0, 21.5. **IR** ν_{max} (film): 2365, 1792, 1772, 1697, 1683, 1670, 1655, 1576, 1569, 1558, 1540, 1528, 1488, 1473, 1407, 1259, 1158, 813, 749, 551 cm^{-1}. **HRMS** (ESI) m/z calcd for $C_{32}H_{35}N_3NaO_3S$ $[M+Na]^+$: 564.2291; found: 564.2281.

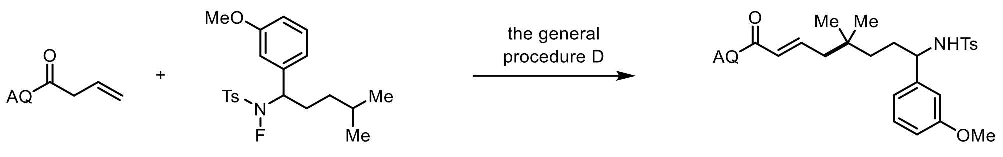

1a **2p** **4p**

Product **4p** was prepared by the general procedure D. Purification using column chromatography (PE/EA = 5:1) afforded **4p** as yellow oil (62.8 mg, 0.11 mmol, 55%, *r.r.* > 20:1, *E/Z* > 20:1). **^{1}H NMR** (600 MHz, $CDCl_3$) δ 9.83 (s, 1H), 8.85 (dd, *J* = 7.8, 1.2 Hz, 1H), 8.80 (dd, *J* = 4.2, 1.8 Hz, 1H), 8.15 (dd, *J* = 8.4, 1.8 Hz, 1H), 7.56 - 7.53 (m, 3H), 7.51 - 7.49 (m, 1H), 7.45 (dd, *J* = 8.4, 4.2 Hz, 1H), 7.09 - 7.05 (m, 3H), 6.96 (dt, *J* = 15.0, 7.8 Hz, 1H), 6.66 - 6.63 (m, 2H), 6.50 (s, 1H), 6.09 (d, *J* = 15.0 Hz, 1H), 5.24 (d, *J* = 7.2 Hz, 1H), 4.16 (dt, *J* = 7.2, 7.2 Hz, 1H), 3.65 (s, 3H), 2.32 (s, 3H), 2.09 - 2.02 (m, 2H), 1.80 - 1.74 (m, 1H), 1.71 - 1.64 (m, 1H), 1.28 - 1.23 (m, 1H), 1.02 - 0.97 (m, 1H), 0.84 (s, 3H), 0.83 (s, 3H). **^{13}C NMR** (150 MHz, $CDCl_3$) δ 164.1, 159.7, 148.3, 143.2, 143.0, 142.6, 138.6, 137.9, 136.5, 134.8, 129.7, 129.4, 128.1, 127.6, 127.2, 126.9, 121.7, 121.6, 118.9, 116.8, 113.1, 112.2, 59.2, 55.2, 44.5, 38.2, 33.7, 32.3, 27.1, 27.0, 21.5. **IR** ν_{max} (film): 3349, 2955, 1710, 1672, 1640, 1598, 1527, 1486, 1462, 1425, 1385, 1327, 1289, 1260, 1158, 1092, 1043, 977, 888, 827, 791, 758, 701, 668, 549 cm^{-1}. **HRMS** (ESI) m/z calcd for $C_{33}H_{37}N_3NaO_4S$ $[M+Na]^+$: 594.2397; found: 594.2386.

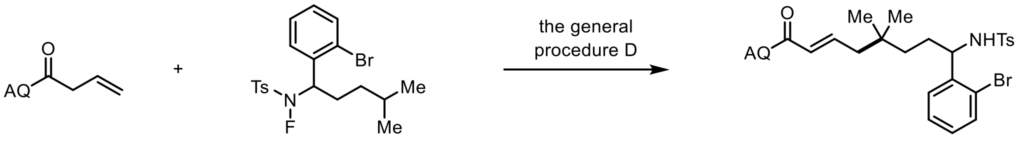

1a **2q** **4q**

Product **4q** was prepared by the general procedure D. Purification using column chromatography (PE/EA = 5:1) afforded **4q** as yellow oil (66.9 mg, 0.11 mmol, 54%, *r.r.* > 20:1, *E/Z* > 20:1). **^{1}H NMR** (600 MHz, $CDCl_3$) δ 9.82 (s, 1H), 8.85 - 8.82 (m, 2H), 8.16 (d, *J* = 8.4 Hz, 1H), 7.59 (d, *J* = 7.8 Hz, 2H), 7.56 - 7.50 (m, 2H), 7.46 (dd, *J* = 7.8, 4.2 Hz, 1H), 7.34 (d, *J* = 7.8 Hz, 1H), 7.17 (d, *J* = 7.8 Hz, 1H), 7.09 - 7.07 (m, 3H), 6.98 - 6.93 (m, 2H), 6.08 (d, *J* =15.0 Hz, 1H), 5.69 (d, *J* = 7.8 Hz, 1H), 4.67 (dt, *J* = 7.8, 7.8 Hz, 1H), 2.29 (s, 3H), 2.05 (d, *J* = 7.2 Hz, 2H), 1.74 - 1.61 (m, 2H), 1.35 - 1.30 (m, 1H), 1.04 - 0.98 (m, 1H), 0.84 (s, 3H), 0.83 (s, 3H). **^{13}C NMR** (150 MHz, $CDCl_3$) δ 164.0, 148.3, 143.2, 143.1, 138.6, 137.2, 136.5, 134.7, 132.9, 129.4, 128.6, 128.1, 127.7, 127.6, 127.2, 126.9, 122.5, 121.8, 121.7, 116.8, 58.1, 44.3, 37.9, 33.7, 31.7, 27.12, 27.11, 21.5. **IR** ν_{max} (film): 3446, 2955, 1771, 1733, 1717, 1697, 1683, 1670, 1653, 1646, 1636, 1558, 1525, 1507, 1487, 1473, 1457, 1424, 1386, 1328, 1259, 1159, 1093, 750, 562, 418 cm^{-1}. **HRMS** (ESI) m/z calcd for $C_{32}H_{34}BrN_3NaO_3S$ $[M+Na]^+$: 642.1396; found: 642.1395.

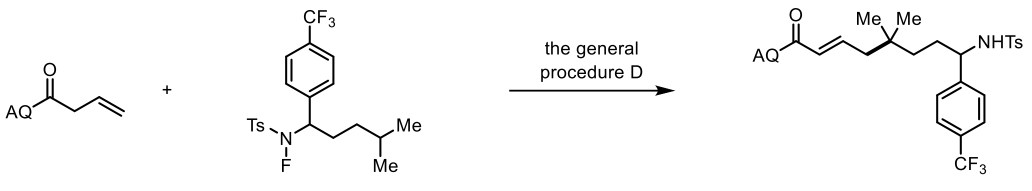

1a **2r** **4r**

Product **4r** was prepared by the general procedure D. Purification using column chromatography (PE/EA = 5:1) afforded **4r** as yellow oil (80.4 mg, 0.13 mmol, 66%, *r.r.* > 20:1, *E/Z* > 20:1). **^{1}H NMR** (600 MHz, $CDCl_3$) δ 9.84 (s, 1H), 8.84 (dd, *J* = 7.8, 1.8 Hz, 1H), 8.81 (dd, *J* = 4.2, 1.2 Hz, 1H), 8.17 (d, *J* = 8.4, 1.8 Hz, 1H), 7.57 - 7.51 (m, 2H), 7.47 - 7.45 (m, 3H), 7.33 (d, *J* = 7.8 Hz, 2H), 7.14 (d, *J* = 7.8 Hz, 2H), 7.03 (d, *J* = 7.8 Hz, 2H), 6.97 (dt, *J* = 15.0, 7.8 Hz, 1H), 6.11 (d, *J* = 15.0 Hz, 1H), 5.57 - 5.53 (m, 1H), 4.29 (dt, *J* = 7.2, 7.2 Hz, 1H), 2.29 (s, 3H), 2.05 (dd, *J* = 7.8, 4.2 Hz, 2H), 1.79 - 1.73 (m, 1H), 1.69 - 1.62 (m, 1H), 1.33 - 1.28 (m, 1H), 1.02 - 0.98 (m, 1H), 0.84 (s, 3H), 0.83 (s, 3H). **^{13}C NMR** (150 MHz, $CDCl_3$) δ 164.0, 148.3, 145.0, 143.3, 143.1, 138.6, 137.6, 136.6, 134.7, 129.6 (q, *J* = 32.4 Hz), 129.4, 128.1, 127.6, 127.1, 127.0, 125.4 (q, *J* = 3.3 Hz), 124.1 (q, *J* = 270.3 Hz), 121.8, 116.9, 58.8, 44.5, 38.2, 33.7, 32.2, 27.12, 27.06, 21.4. **^{19}F NMR** (470 MHz, $CDCl_3$) δ -62.6 (s). **IR** ν_{max} (film): 3446, 2930, 1868, 1829, 1792, 1772, 1749, 1733, 1717, 1697, 1683, 1670, 1653, 1646, 1636, 1576, 1569, 1558, 1540, 1526, 1488, 1473, 1457, 1424, 1386, 1325, 1259, 1159, 1122, 1093, 1067, 977, 913, 826, 792, 749, 616, 547, 418 cm^{-1}. **HRMS** (ESI) m/z calcd for $C_{33}H_{34}F_3N_3NaO_3S$ $[M+Na]^+$: 632.2165; found: 632.2152.

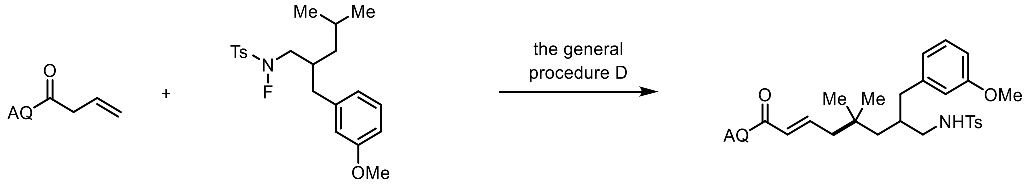

1a **2s** **4s**

Product **4s** was prepared by the general procedure D. Purification using column chromatography (PE/EA = 5:1) afforded **4s** as yellow oil (70.2 mg, 0.12 mmol, 60%, *r.r.* > 20:1, *E/Z* > 20:1). **^{1}H NMR** (600 MHz, $CDCl_3$) δ 9.83 (s, 1H), 8.84 (dd, *J* = 7.8, 1.2 Hz, 1H), 8.80 (dd, *J* = 4.2, 1.8 Hz, 1H), 8.16 (dd, *J* = 8.4, 1.8 Hz, 1H), 7.68 (d, *J* = 7.8 Hz, 2H), 7.56 - 7.49 (m, 2H), 7.45 (dd, *J* = 7.8, 4.2 Hz, 1H), 7.25 (d, *J* = 7.8 Hz, 2H), 7.15 (t, *J* = 7.8 Hz, 1H), 6.98 (dt, *J* = 15.0, 7.8 Hz, 1H), 6.73 (dd, *J* = 8.4, 3.0 Hz, 1H), 6.68 - 6.65 (m, 2H), 6.09 (d, *J* = 15.0 Hz, 1H), 4.84 (t, *J* = 6.0 Hz, 1H), 3.77 (s, 3H), 2.93 - 2.88 (m, 1H), 2.78 - 2.74 (m, 1H), 2.58 (dd, *J* = 13.8, 7.8 Hz, 1H), 2.51 (dd, *J* = 13.8, 6.6 Hz, 1H), 2.38 (s, 3H), 2.10 - 2.03 (m, 2H), 1.89 - 1.86 (m, 1H), 1.27 - 1.20 (m, 2H), 0.86 (s, 3H), 0.83 (s, 3H). **^{13}C NMR** (150 MHz, $CDCl_3$) δ 164.0, 159.8, 148.2,

143.4, 143.1, 141.5, 138.6, 137.0, 136.5, 134.7, 129.8, 129.5, 128.0, 127.5, 127.2, 127.0, 121.8, 121.69, 121.65, 116.8, 115.1, 111.7, 55.3, 47.9, 45.5, 43.6, 40.7, 36.4, 34.6, 27.1, 27.0, 21.6. **IR** ν_{max} (film): 3344, 2956, 1771, 1675, 1637, 1597, 1526, 1486, 1425, 1386, 1327, 1260, 1159, 1092, 1041, 976, 909, 826, 790, 734, 616, 551, 418 cm^{-1}. **HRMS** (ESI) m/z calcd for $C_{34}H_{39}N_3NaO_4S$ $[M+Na]^+$: 608.2553; found: 608.2561.

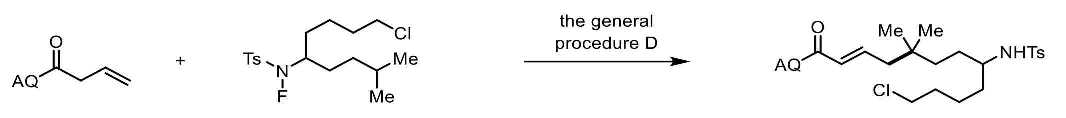

1a 2t 4t

Product **4t** was prepared by the general procedure D. Purification using column chromatography (PE/EA = 5:1) afforded **4t** as yellow oil (72.2 mg, 0.13 mmol, 65%, *r.r.* > 20:1, *E/Z* > 20:1). **^{1}H NMR** (500 MHz, $CDCl_3$) δ 9.85 (s, 1H), 8.86 (d, *J* = 7.5 Hz, 1H), 8.80 (dd, *J* = 5.5, 1.5 Hz, 1H), 8.15 (dd, *J* = 8.0, 1.5 Hz, 1H), 7.77 (d, *J* = 8.0 Hz, 2H), 7.56 - 7.49 (m, 2H), 7.45 (dd, *J* = 8.5, 4.5 Hz, 1H), 7.28 (d, *J* = 8.0 Hz, 2H), 6.97 (dt, *J* = 15.0, 8.0 Hz, 1H), 6.13 (d, *J* = 15.0 Hz, 1H), 4.85 (d, *J* = 8.5 Hz, 1H), 3.39 (t, *J* = 6.5 Hz, 2H), 3.19 - 3.14 (m, 1H), 2.39 (s, 3H), 2.03 (d, *J* = 8.5 Hz, 2H), 1.64 - 1.59 (m, 2H), 1.44 - 1.37 (m, 2H), 1.36 - 1.32 (m, 2H), 1.31 - 1.24 (m, 2H), 1.09 - 0.97 (m, 2H), 0.80 (s, 3H), 0.79 (s, 3H). **^{13}C NMR** (125 MHz, $CDCl_3$) δ 164.0, 148.3, 143.3, 143.2, 138.52, 138.48, 136.5, 134.7, 129.7, 128.0, 127.5, 127.2, 126.8, 121.74, 121.68, 116.8, 54.6, 44.8, 44.5, 37.4, 34.4, 33.6, 32.3, 29.6, 27.0, 26.9, 22.8, 21.5. **IR** ν_{max} (film): 3348, 2953, 2867, 1677, 1640, 1596, 1526, 1486, 1461, 1425, 1386, 1327, 1259, 1156, 1092, 978, 826, 815, 792, 549 cm^{-1}. **HRMS** (ESI) m/z calcd for $C_{30}H_{38}ClN_3NaO_3S$ $[M+Na]^+$: 578.2215; found: 578.2204.

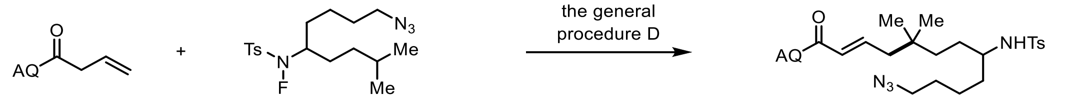

1a 2u 4u

Product **4u** was prepared by the general procedure D. Purification using column chromatography (PE/EA = 3:1) afforded **4u** as yellow oil (50.6 mg, 0.09 mmol, 45%, *r.r.* > 20:1, *E/Z* > 20:1). **^{1}H NMR** (600 MHz, $CDCl_3$) δ 9.85 (s, 1H), 8.86 (dd, *J* = 7.2, 1.2 Hz, 1H), 8.81 (dd, *J* = 4.8, 1.8 Hz, 1H), 8.17 (dd, *J* = 8.4, 1.8 Hz, 1H), 7.77 (d, *J* = 7.8 Hz, 2H), 7.57 - 7.51 (m, 2H), 7.46 (dd, *J* = 8.4, 4.2 Hz, 1H), 7.29 (d, *J* = 7.8 Hz, 2H), 6.96 (dt, *J* = 15.0, 7.8 Hz, 1H), 6.13 (d, *J* = 15.0 Hz, 1H), 4.66 (d, *J* = 8.4 Hz, 1H), 3.17 - 3.13 (m, 3H), 2.39 (s, 3H), 2.03 (d, *J* = 7.8 Hz, 2H), 1.47 - 1.42 (m, 3H), 1.36 - 1.32 (m, 3H), 1.28 - 1.25 (m, 2H), 1.05 - 0.99 (m, 2H), 0.80 (s, 3H), 0.79 (s, 3H). **^{13}C NMR** (150 MHz, $CDCl_3$) δ 164.0, 148.3, 143.4, 143.2, 138.6, 138.5, 136.5,

134.7, 129.7, 128.1, 127.6, 127.2, 126.9, 121.8, 121.7, 116.8, 54.7, 51.3, 44.5, 37.4, 34.8, 33.6, 29.7, 28.6, 27.04, 26.99, 22.7, 21.6. **IR** ν_{max} (film): 3342, 2951, 2866, 2095, 1772, 1733, 1716, 1683, 1652, 1636, 1596, 1526, 1486, 1457, 1425, 1386, 1327, 1260, 1157, 1093, 978, 912, 827, 815, 749, 610, 549, 418 cm^{-1}. **HRMS** (ESI) m/z calcd for $C_{30}H_{38}N_6NaO_3S$ $[M+Na]^+$: 585.2618; found: 585.2625.

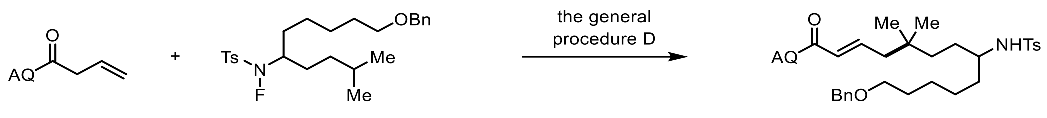

1a **2v** **4v**

Product **4v** was prepared by the general procedure D. Purification using column chromatography (PE/EA = 3:1) afforded **4v** as yellow oil (83.4 mg, 0.13 mmol, 65%, *r.r.* > 20:1, *E/Z* > 20:1). **^{1}H NMR** (600 MHz, $CDCl_3$) δ 9.85 (s, 1H), 8.86 (dd, *J* = 7.2 Hz, 1H), 8.79 (dd, *J* = 4.2, 1.8 Hz, 1H), 8.14 (dd, *J* = 8.4, 1.8 Hz, 1H), 7.76 (d, *J* = 7.8 Hz, 2H), 7.55 - 7.49 (m, 2H), 7.43 (dd, *J* = 7.8, 4.2 Hz, 1H), 7.34 - 7.30 (m, 4H), 7.27 - 7.24 (m, 3H), 6.97 (dt, *J* = 15.0, 7.8 Hz, 1H), 6.12 (d, *J* = 15.0 Hz, 1H), 4.65 (d, *J* = 8.4 Hz, 1H), 4.45 (s, 2H), 3.36 (t, *J* = 6.6 Hz, 2H), 3.18 - 3.12 (m, 1H), 2.36 (s, 3H), 2.03 (d, *J* = 7.8 Hz, 2H), 1.50 - 1.45 (m, 2H), 1.42 - 1.37 (m, 2H), 1.34 - 1.26 (m, 4H), 1.16 - 0.99 (m, 4H), 0.81 (s, 3H), 0.80 (s, 3H). **^{13}C NMR** (150 MHz, $CDCl_3$) δ 164.0, 148.2, 143.22, 143.18, 138.7, 138.6, 138.5, 136.5, 134.7, 129.6, 128.4, 128.0, 127.7, 127.6, 127.5, 127.2, 126.8, 121.7, 121.6, 116.8, 72.9, 70.3, 54.8, 44.5, 37.5, 35.0, 33.6, 29.7, 29.6, 27.0, 26.9, 26.0, 25.3, 21.5. **IR** ν_{max} (film): 3277, 2935, 2859, 1771, 1683, 1637, 1596, 1526, 1486, 1456, 1425, 1385, 1327, 1241, 1158, 1094, 979, 912, 827, 792, 748, 697, 610, 580, 550 cm^{-1}. **HRMS** (ESI) m/z calcd for $C_{38}H_{47}N_3NaO_4S$ $[M+Na]^+$: 664.3179; found: 664.3171.

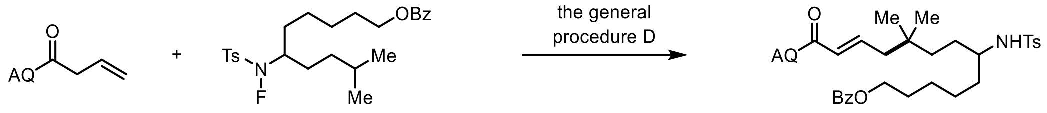

1a **2w** **4w**

Product **4w** was prepared by the general procedure D. Purification using column chromatography (PE/EA = 3:1) afforded **4w** as yellow oil (78.6 mg, 0.12 mmol, 60%, *r.r.* = 20:1, *E/Z* > 20:1). **^{1}H NMR** (600 MHz, $CDCl_3$) δ 9.85 (s, 1H), 8.86 (dd, *J* = 7.2, 1.2 Hz, 1H), 8.80 (dd, *J* = 4.2, 1.8 Hz, 1H), 8.15 (dd, *J* = 8.4, 1.8 Hz, 1H), 8.03 - 8.01 (m, 2H), 7.77 (d, *J* = 7.8 Hz, 2H), 7.55 - 7.53 (m, 2H), 7.50 (dd, *J* = 7.8, 1.2 Hz, 1H), 7.45 - 7.42 (m, 3H), 7.27 (d, *J* = 7.8 Hz, 2H), 6.97 (dt, *J* = 15.0, 7.8 Hz, 1H), 6.15 (d, *J* = 15.0 Hz, 1H), 4.66 (d, *J* = 9.0 Hz, 1H), 4.21 (t, *J* = 6.6 Hz, 2H), 3.20 - 3.15 (m, 1H), 2.37 (s, 3H), 2.03 (d, *J* = 7.8 Hz, 2H), 1.67 - 1.60 (m, 2H), 1.47 -

1.41 (m, 1H), 1.39 - 1.34 (m, 2H), 1.33 - 1.28 (m, 4H), 1.22 - 1.18 (m, 1H), 1.09 - 1.00 (m, 2H), 0.80 (s, 3H), 0.79 (s, 3H). **^{13}C NMR** (150 MHz, $CDCl_3$) δ 166.7, 164.0, 148.2, 143.3, 143.2, 138.6, 138.5, 136.5, 134.7, 132.9, 130.5, 129.7, 129.6, 128.4, 128.1, 127.5, 127.2, 126.9, 121.73, 121.66, 116.8, 64.9, 54.8, 44.5, 37.5, 35.1, 33.6, 29.8, 28.7, 27.01, 26.96, 25.9, 25.2, 21.5. **IR** ν_{max} (film): 3348, 2951, 2862, 1771, 1759, 1716, 1683, 1652, 1646, 1636, 1598, 1526, 1507, 1487, 1472, 1457, 1424, 1385, 1316, 1274, 1157, 1113, 1094, 1069, 1026, 977, 913, 826, 792, 749, 549, 418 cm^{-1}. **HRMS** (ESI) m/z calcd for $C_{38}H_{45}N_3NaO_5S$ $[M+Na]^+$: 678.2972; found: 678.2967.

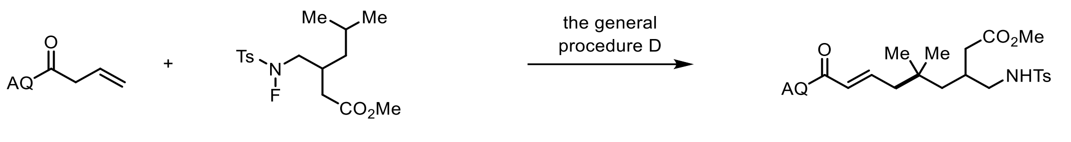

1a 2x 4x

Product **4x** was prepared by the general procedure D. Purification using column chromatography (PE/EA = 3:1) afforded **4x** as yellow oil (70.9 mg, 0.13 mmol, 66%, *r.r.* > 20:1, *E/Z* > 20:1). **^{1}H NMR** (600 MHz, $CDCl_3$) δ 9.84 (s, 1H), 8.84 (dd, *J* = 7.2, 1.2 Hz, 1H), 8.81 (dd, *J* = 4.2, 1.8 Hz, 1H), 8.16 (dd, *J* = 7.8, 1.2 Hz, 1H), 7.72 (d, *J* = 8.4. Hz, 2H), 7.56 - 7.50 (m, 2H), 7.46 (dd, *J* = 8.4, 4.2 Hz, 1H) 7.26 (d, *J* = 8.4 Hz, 2H), 7.01 (dt, *J* = 15.0, 7.8 Hz, 1H), 6.16 (d, *J* = 15.0 Hz, 1H), 5.00 (t, *J* = 6.6 Hz, 1H), 3.65 (s, 3H), 2.98 - 2.94 (m, 1H), 2.86 - 2.81 (m, 1H), 2.39 - 2.38 (m, 5H), 2.15 (d, *J* = 7.8 Hz, 2H), 2.13 - 2.09 (m, 1H), 1.26 - 1.18 (m, 2H), 0.93 (s, 3H), 0.92 (s, 3H). **^{13}C NMR** (150 MHz, $CDCl_3$) δ 173.4, 164.0, 148.3, 143.5, 142.8, 138.6, 137.2, 136.5, 134.7, 129.8, 128.1, 127.6, 127.22, 127.18, 121.8, 121.7, 116.8, 51.8, 48.3, 45.4, 44.0, 38.7, 34.7, 31.6, 27.2, 27.0, 21.6. **IR** ν_{max} (film): 3341, 2955, 1735, 1683, 1637, 1596, 1577, 1527, 1486, 1458, 1425, 1386, 1329, 1242, 1160, 1092, 1047, 978, 892, 827, 792, 758, 610, 551 cm^{-1}. **HRMS** (ESI) m/z calcd for $C_{29}H_{35}N_3NaO_5S$ $[M+Na]^+$: 560.2190; found: 560.2189.

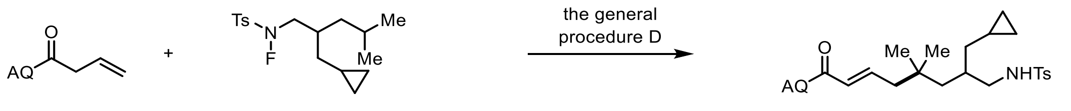

1a 2y 4y

Product **4y** was prepared by the general procedure D. Purification using column chromatography (PE/EA = 5:1) afforded **4y** as yellow oil (75.8 mg, 0.15 mmol, 73%, *r.r.* > 20:1, *E/Z* > 20:1). **^{1}H NMR** (600 MHz, $CDCl_3$) δ 9.78 (s, 1H), 8.78 (dd, *J* = 7.8, 1.2 Hz, 1H), 8.74 (dd, *J* = 4.2, 1.8 Hz, 1H), 8.10 (dd, *J* = 7.8, 1.2 Hz, 1H), 7.67 (d, *J* = 7.8 Hz, 2H), 7.49 - 7.43 (m, 2H), 7.39 (dd, *J* = 8.4, 4.2 Hz, 1H), 7.22 (d, *J* = 7.8 Hz, 2H), 6.96 (dt, *J* = 15.0, 7.8 Hz, 1H), 6.08 (d, *J* = 15.0 Hz, 1H), 4.45 - 4.44 (m, 1H), 2.93 - 2.89 (m, 1H), 2.83 - 2.79 (m, 1H), 2.33 (s, 3H), 2.06 (d,

J = 7.8 Hz, 2H), 1.64 - 1.60 (m, 1H), 1.19 - 1.07 (m, 5H), 0.84 (s, 3H), 0.83 (s, 3H), 0.55 - 0.49 (m, 1H), 0.38 - 0.31 (m, 2H), 0.05 - (-0.12) (m, 2H). **^{13}C NMR** (150 MHz, $CDCl_3$) δ 164.1, 148.3, 143.5, 143.2, 138.6, 137.1, 136.5, 134.8, 129.8, 128.1, 127.6, 127.3, 127.1, 121.8, 121.7, 116.9, 48.2, 45.7, 44.1, 39.3, 35.0, 34.8, 27.2, 27.1, 21.6, 8.7, 5.1, 5.0. **IR** ν_{max} (film): 2925, 1741, 1683, 1637, 1596, 1526, 1486, 1458, 1425, 1386, 1327, 1241, 1159, 1093, 1047, 975, 826, 792, 550 cm^{-1}. **HRMS** (ESI) m/z calcd for $C_{30}H_{37}N_3NaO_3S$ $[M+Na]^+$: 542.2448; found: 542.2451.

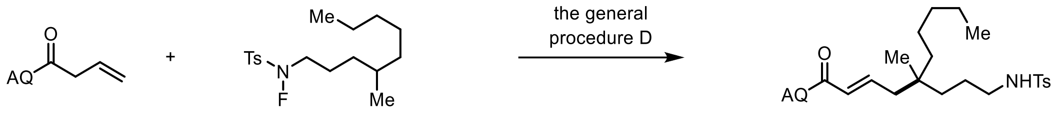

1a **2z** **4z**

Product **4z** was prepared by the general procedure D. Purification using column chromatography (PE/EA = 5:1) afforded **4z** as yellow oil (70.9 mg, 0.14 mmol, 68%, *r.r.* > 20:1, *E/Z* > 20:1). **^{1}H NMR** (600 MHz, $CDCl_3$) δ 9.84 (s, 1H), 8.84 (dd, J = 7.2, 1.2 Hz, 1H), 8.81 (dd, J = 4.8, 1.8 Hz, 1H), 8.16 (dd, J = 8.4, 1.8 Hz, 1H), 7.75 (d, J = 7.8 Hz, 2H), 7.56 - 7.50 (m, 2H), 7.46 (dd, J = 8.4, 4.8 Hz, 1H), 7.28 (d, J = 7.8 Hz, 2H), 6.99 (dt, J = 15.0, 7.8 Hz, 1H), 6.14 (d, J = 15.0 Hz, 1H), 4.70 (t, J = 6.6 Hz, 1H), 2.91(dt, J = 6.6, 6.6 Hz, 2H), 2.39 (s, 3H), 2.11 (d, J = 7.8 Hz, 2H), 1.44 - 1.39 (m, 2H), 1.32 - 1.26 (m, 3H), 1.18 - 1.15 (m, 7H), 0.88 (t, J = 7.2 Hz, 3H), 0.84 (s, 3H). **^{13}C NMR** (150 MHz, $CDCl_3$) δ 164.1, 148.3, 143.4, 143.3, 138.6, 137.2, 136.5, 134.7, 129.8, 128.1, 127.6, 127.2, 126.8, 121.8, 121.7, 116.8, 44.1, 42.4, 39.4, 36.5, 36.1, 32.7, 24.9, 24.2, 23.3, 22.8, 21.6, 14.2. **IR** ν_{max} (film): 3344, 2954, 2928, 2857, 1683, 1637, 1596, 1527, 1486, 1458, 1425, 1385, 1328, 1260, 1159, 1094, 979, 911, 826, 739, 550 cm^{-1}. **HRMS** (ESI) m/z calcd for $C_{30}H_{39}N_3NaO_3S$ $[M+Na]^+$: 544.2604; found: 544.2609.

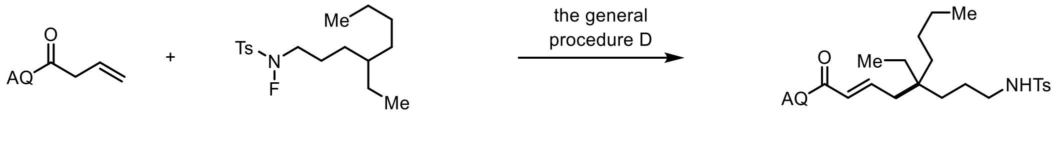

1a **2aa** **4aa**

Product **4aa** was prepared by the general procedure D. Purification using column chromatography (PE/EA = 5:1) afforded **4aa** as yellow oil (67.8 mg, 0.13 mmol, 65%, *r.r.* > 20:1, *E/Z* > 20:1). **^{1}H NMR** (500 MHz, $CDCl_3$) δ 9.83 (s, 1H), 8.83 (dd, J = 7.5, 1.5 Hz, 1H), 8.80 (dd, J = 4.5, 2.0 Hz, 1H), 8.15 (dd, J = 8.5, 2.0 Hz, 1H), 7.75 (d, J = 8.5 Hz, 2H), 7.55 - 7.49 (m, 2H), 7.45 (dd, J = 8.0, 4.0 Hz, 1H), 7.26 (d, J = 8.5 Hz, 2H), 6.97 (dt, J = 15.0, 7.5 Hz, 1H), 6.14 (d, J = 15.0 Hz, 1H), 4.85 (t, J = 7.0 Hz, 1H), 2.90 (dt, J = 7.0, 7.0 Hz, 2H), 2.38 (s, 3H), 2.09 (d, J =

7.5 Hz, 2H), 1.41 - 1.35 (m, 2H), 1.27 - 1.19 (m, 4H), 1.18 - 1.09 (m, 6H), 0.89 (t, *J* = 7.5 Hz, 3H), 0.75 (t, *J* = 7.5 Hz, 3H). **^{13}C NMR** (125 MHz, $CDCl_3$) δ 164.1, 148.3, 143.4, 143.2, 138.6, 137.1, 136.5, 134.7, 129.8, 128.0, 127.5, 127.2, 126.5, 121.74, 121.67, 116.8, 44.1, 39.0, 38.5, 35.9, 33.3, 28.8, 25.3, 23.7, 23.5, 21.6, 14.2, 7.7. **IR** ν_{max} (film): 3346, 2957, 2929, 2869, 1738, 1678, 1640, 1596, 1526, 1485, 1461, 1425, 1384, 1328, 1241, 1159, 1093, 977, 826, 792, 757, 662, 550 cm^{-1}. **HRMS** (ESI) m/z calcd for $C_{30}H_{39}N_3NaO_3S$ [M+Na]$^+$: 544.2604; found: 544.2604.

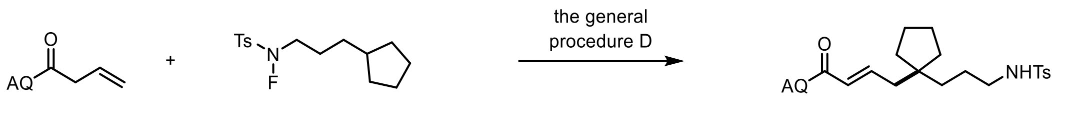

1a 2ab 4ab

Product **4ab** was prepared by the general procedure D. Purification using column chromatography (PE/EA = 5:1) afforded **4ab** as yellow oil (70.7 mg, 0.14 mmol, 72%, *r.r.* > 20:1, *E/Z* > 20:1). **^{1}H NMR** (600 MHz, $CDCl_3$) δ 9.83 (s, 1H), 8.83 (dd, *J* = 7.8, 1.8 Hz, 1H), 8.80 (dd, *J* = 4.2, 1.8 Hz, 1H), 8.15 (dd, *J* = 7.8, 1.2 Hz, 1H), 7.74 (d, *J* = 8.4 Hz, 2H), 7.55 - 7.49 (m, 2H), 7.45 (dd, *J* = 8.4, 4.2 Hz, 1H), 7.26 (d, *J* = 8.4 Hz, 2H), 6.97 (dt, *J* = 15.0, 7.8 Hz, 1H), 6.15 (d, *J* = 15.0 Hz, 1H), 4.78 (t, *J* = 6.6 Hz, 1H), 2.90 (dt, *J* = 6.6, 6.6 Hz, 2H), 2.37 (s, 3H), 2.18 (d, *J* = 7.8 Hz, 2H), 1.60 - 1.54 (m, 4H), 1.47 - 1.41 (m, 4H), 1.34 - 1.29 (m, 2H), 1.26 - 1.23 (m, 2H). **^{13}C NMR** (150 MHz, $CDCl_3$) δ 164.1, 148.3, 143.8, 143.4, 138.6, 137.2, 136.5, 134.7, 129.8, 128.1, 127.5, 127.2, 126.6, 121.8, 121.7, 116.8, 45.3, 44.1, 41.3, 37.4, 36.3, 25.3, 24.8, 21.6. **IR** ν_{max} (film): 3440, 3351, 2956, 1740, 1672, 1640, 1597, 1526, 1485, 1461, 1425, 1373, 1327, 1241, 1158, 1093, 1047, 976, 914, 827, 813, 792, 734, 700, 608, 561 cm^{-1}. **HRMS** (ESI) m/z calcd for $C_{28}H_{33}N_3NaO_3S$ [M+Na]$^+$: 514.2135; found: 514.2136.

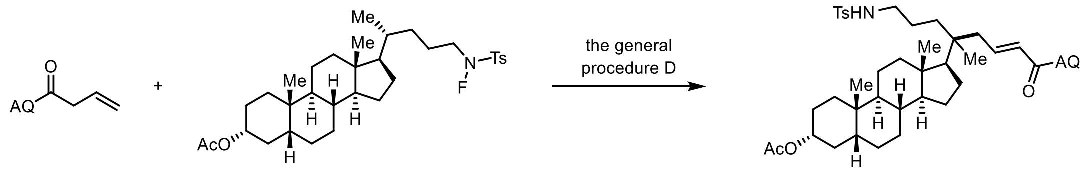

1a 2ac 4ac

Product **4ac** was prepared by the general procedure D. Purification using column chromatography (PE/EA = 5:1) afforded **4ac** as yellow oil (79.8 mg, 0.10 mmol, 52%, *r.r.* > 20:1, *E/Z* > 20:1, *d.r.* = 1:1). **^{1}H NMR** (600 MHz, $CDCl_3$) δ 9.85 (s, 0.5H), 9.84 (s, 0.5H), 8.86 - 8.81 (m, 2H), 8.17 (dd, *J* = 8.4, 1.8 Hz, 1H), 7.75 (d, *J* = 8.4 Hz, 1H), 7.73 (d, *J* = 8.4 Hz, 1H), 7.57 - 7.51 (m, 2H), 7.48 - 7.45 (m, 1H), 7.27 - 7.23 (m, 2H), 7.05 - 6.96 (m, 1H), 6.18 - 6.15 (m, 1H), 4.75 - 4.67 (m, 2H), 2.93 - 2.84 (m, 2H), 2.38 (s, 1.5H), 2.37 (s, 1.5H), 2.34 - 2.24 (m, 1H), 2.16 -

2.15 (m, 1H), 2.02 (s, 1.5H), 2.01 (s, 1.5H), 1.95 - 1.89 (m, 1H), 1.85 - 1.76 (m, 4H), 1.69 - 1.67 (m, 1H), 1.58 - 1.48 (m, 5H), 1.44 - 1.33 (m, 8H), 1.26 - 1.17 (m, 4H), 1.07 - 0.99 (m, 4H), 0.95 (s, 1.5H), 0.93 (s, 1.5H), 0.92 (s, 1.5H), 0.91 (s, 1.5H), 0.74 (s, 1.5H), 0.73 (s, 1.5H). **^{13}C NMR** (150 MHz, $CDCl_3$) δ 170.9, 164.1, 148.3, 143.8, 143.6, 143.37, 143.35, 138.6, 137.2, 137.1, 136.5, 134.7, 129.80, 129.77, 128.1, 127.6, 127.3, 127.2, 126.59, 126.57, 121.8, 121.7, 116.8, 74.4, 57.3, 57.1, 56.71, 56.65, 44.2, 44.1, 44.00, 43.97, 41.91, 41.90, 41.84, 41.81, 41.1, 41.0, 40.5, 40.3, 40.0, 36.4, 36.3, 35.34, 35.33, 35.1, 34.64, 34.61, 32.32, 32.30, 27.1, 26.7, 26.3, 26.2, 24.5, 24.2, 23.74, 23.70, 23.5, 23.4, 23.1, 23.0, 22.8, 21.61, 21.60, 20.8, 15.3, 15.1. **IR** ν_{max} (film): 3343, 2931, 2867, 1732, 1682, 1636, 1596, 1526, 1486, 1457, 1425, 1384, 1362, 1328, 1244, 1184, 1160, 1093, 1027, 980, 912, 826, 814, 792, 733, 661, 569, 550 cm^{-1}. **HRMS** (ESI) m/z calcd for $C_{46}H_{61}N_3NaO_5S$ $[M+Na]^+$: 790.4224; found: 790.4226.

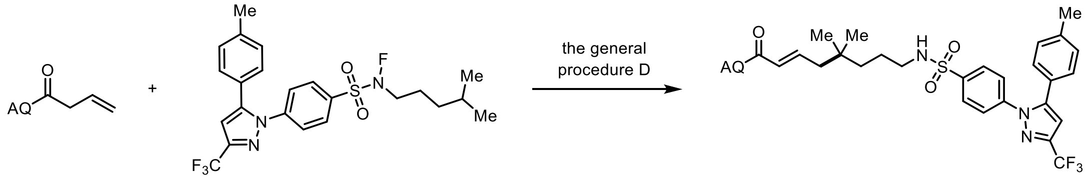

1a **2ad** **4ad**

Product **4ad** was prepared by the general procedure D. Purification using column chromatography (PE/EA = 5:1) afforded **4ad** as yellow oil (90.5 mg, 0.13 mmol, 67%, *r.r.* > 20:1, *E/Z* > 20:1). **^{1}H NMR** (600 MHz, $CDCl_3$) δ 9.84 (s, 1H), 8.82 (d, *J* = 7.2 Hz, 1H), 8.79 (dd, *J* = 4.2, 1.8 Hz, 1H), 8.15 (dd, *J* = 8.4, 1.8 Hz, 1H), 7.85 (d, *J* = 8.4 Hz, 2H), 7.55 - 7.49 (m, 2H), 7.46 - 7.44 (m, 3H), 7.15 (d, *J* = 7.8 Hz, 2H), 7.08 (d, *J* = 7.8 Hz, 2H), 7.02 (dt, *J* = 15.0, 7.8 Hz, 1H), 6.72 (s, 1H), 6.14 (d, *J* = 15.0 Hz, 1H), 4.95 (t, *J* = 6.6 Hz, 1H), 2.93 (dt, *J* = 6.6, 6.6 Hz, 2H), 2.36 (s, 3H), 2.12 (d, *J* = 7.8 Hz, 2H), 1.51 - 1.46 (m, 2H), 1.22 - 1.19 (m, 2H), 0.89 (s, 6H). **^{13}C NMR** (150 MHz, $CDCl_3$) δ 164.1, 148.3, 145.3, 144.1 (q, *J* = 38.1 Hz), 143.2, 142.5, 139.9, 139.7, 138.6, 136.5, 134.7, 129.8, 128.8, 128.2, 128.1, 127.6, 126.9, 125.8, 125.6, 121.8, 121.7, 121.2 (q, *J* = 267.0 Hz), 116.9, 106.3, 44.8, 44.1, 38.8, 33.8, 27.1, 24.7, 21.4. **^{19}F NMR** (470 MHz, $CDCl_3$) δ -62.4 (s). **IR** ν_{max} (film): 3342, 2956, 1771, 1670, 1636, 1596, 1527, 1487, 1472, 1425, 1374, 1331, 1270, 1236, 1161, 1135, 1096, 975, 911, 826, 792, 759, 649, 626, 616, 574, 418 cm^{-1}. **HRMS** (ESI) m/z calcd for $C_{36}H_{36}F_3N_5NaO_3S$ $[M+Na]^+$: 698.2383; found: 698.2376.

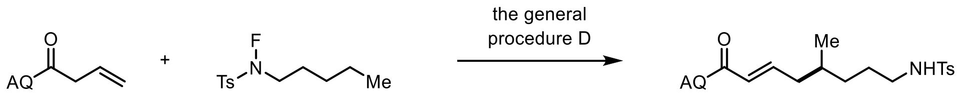

1a **2ae** **4ae**

Product **4ae** was prepared by the general procedure D. Purification using column chromatography (PE/EA = 5:1) afforded **4ae** as yellow oil (55.0 mg, 0.12 mmol, 61%, *r.r.* = 10:1, *E/Z* > 20:1). **^{1}H NMR** (600 MHz, $CDCl_3$) δ 9.83 (s, 1H), 8.84 (dd, *J* = 7.8, 1.8 Hz, 1H), 8.81 (dd, *J* = 4.2, 1.8 Hz, 1H), 8.17 (dd, *J* = 7.8, 1.8 Hz, 1H), 7.75 (d, *J* = 7.8 Hz, 2H), 7.56 - 7.50 (m, 2H), 7.46 (dd, *J* = 7.8, 4.2 Hz, 1H), 7.30 (d, *J* = 7.8 Hz, 2H), 6.97 (dt, *J* = 15.0, 7.2 Hz, 1H), 6.15 (d, *J* = 15.0 Hz, 1H), 4.46 (t, *J* = 6.6 Hz, 1H), 2.95 - 2.92 (m, 2H), 2.41 (s, 3H), 2.24 - 2.19 (m, 1H), 2.12 - 2.07 (m, 1H), 1.62 - 1.58 (m, 1H), 156 - 1.49 (m, 1H), 1.48 - 1.43 (m, 1H), 1.36 - 1.32 (m, 1H), 1.17 - 1.11 (m, 1H), 0.89 (d, *J* = 6.6 Hz, 3H). **^{13}C NMR** (150 MHz, $CDCl_3$) δ 164.2, 148.3, 144.6, 143.5, 138.6, 137.2, 136.6, 134.8, 129.9, 128.1, 127.6, 127.3, 126.1, 121.8, 121.7, 116.9, 43.6, 39.6, 33.5, 32.5, 27.4, 21.6, 19.6. **IR** ν_{max} (film): 3341, 2923, 1770, 1672, 1637, 1596, 1526, 1485, 1457, 1424, 1379, 1326, 1241, 1158, 1092, 974, 826, 792, 550, 418 cm^{-1}. **HRMS** (ESI) m/z calcd for $C_{25}H_{29}N_3NaO_3S$ $[M+Na]^+$: 474.1822; found: 474.1811.

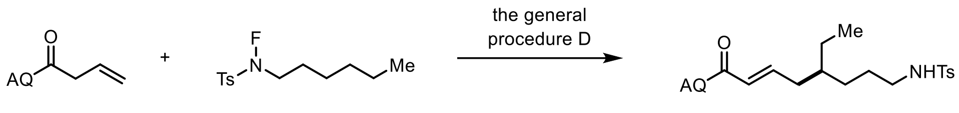

1a **2af** **4af**

Product **4af** was prepared by the general procedure D. Purification using column chromatography (PE/EA = 5:1) afforded **4af** as yellow oil (58.6 mg, 0.13 mmol, 63%, *r.r.* = 10:1, *E/Z* > 20:1). **^{1}H NMR** (600 MHz, $CDCl_3$) δ 9.83 (s, 1H), 8.84 (dd, *J* = 7.2, 1.2 Hz, 1H), 8.81 (dd, *J* = 4.2, 1.8 Hz, 1H), 8.17 (dd, *J* = 8.4, 1.8 Hz, 1H), 7.74 (d, *J* = 7.8 Hz, 2H), 7.57 - 7.50 (m, 2H), 7.46 (dd, *J* = 8.4, 4.2 Hz, 1H), 7.29 (d, *J* = 7.8 Hz, 2H), 6.96 (dt, *J* = 15.0, 7.2 Hz, 1H), 6.15 (d, *J* = 15.0 Hz, 1H), 4.43 (t, *J* = 6.6 Hz, 1H), 2.93 (dd, *J* = 6.6, 6.6 Hz, 2H), 2.40 (s, 3H), 2.25 - 2.15 (m, 2H), 1.50 - 1.41 (m, 3H), 1.33 - 1.29 (m, 2H), 1.27 - 1.24 (m, 2H), 0.86 (t, *J* = 7.2 Hz, 3H). **^{13}C NMR** (150 MHz, $CDCl_3$) δ 164.2, 148.3, 144.7, 143.5, 138.6, 137.2, 136.6, 134.8, 129.9, 128.1, 127.6, 127.3, 126.1, 121.8, 121.7, 116.9, 43.7, 38.7, 36.0, 30.2, 27.2, 26.0, 21.6, 11.0. **IR** ν_{max} (film): 3446, 2920, 1770, 1636, 1525, 1487, 1375, 1326, 1246, 1158, 550, 418 cm^{-1}. **HRMS** (ESI) m/z calcd for $C_{26}H_{31}N_3NaO_3S$ $[M+Na]^+$: 488.1978; found: 488.1979.

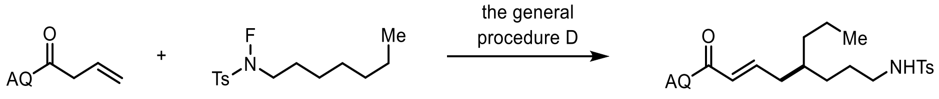

1a **2ag** **4ag**

Product **4ag** was prepared by the general procedure D. Purification using column chromatography (PE/EA = 5:1) afforded **4ag** as yellow oil (59.4 mg, 0.12 mmol, 62%, *r.r.* > 20:1, *E/Z* > 20:1). **^{1}H NMR** (600 MHz, $CDCl_3$) δ 9.83 (s, 1H), 8.84 (dd, *J* = 7.8, 1.2 Hz, 1H), 8.80 (dd,

J = 4.2, 1.8 Hz, 1H), 8.16 (dd, *J* = 7.8, 1.2 Hz, 1H), 7.75 (d, *J* = 8.4 Hz, 2H), 7.56 - 7.49 (m, 2H), 7.45 (dd, *J* = 8.4, 4.2 Hz, 1H), 7.28 (d, *J* = 8.4 Hz, 2H), 6.96 (dt, *J* = 15.0, 7.2 Hz, 1H), 6.14 (d, *J* = 15.0 Hz, 1H), 4.70 (t, *J* = 6.6 Hz, 1H), 2.92 (dt, *J* = 6.6, 6.6 Hz, 2H), 2.39 (s, 3H), 2.23 - 2.13 (m, 2H), 1.52 - 1.43 (m, 3H), 1.29 - 1.21 (m, 6H), 0.86 (t, *J* = 7.2 Hz, 3H). **^{13}C NMR** (150 MHz, $CDCl_3$) δ 164.2, 148.3, 144.8, 143.4, 138.6, 137.2, 136.5, 134.7, 129.8, 128.1, 127.6, 127.2, 126.0, 121.8, 121.7, 116.8, 43.7, 37.0, 36.4, 35.8, 30.6, 27.1, 21.6, 19.9, 14.4. **IR** ν_{max} (film): 3343, 2926, 1770, 1683, 1636, 1596, 1526, 1486, 1457, 1424, 1386, 1327, 1241, 1159, 1093, 826, 792, 550, 418 cm^{-1}. **HRMS** (ESI) m/z calcd for $C_{27}H_{33}N_3NaO_3S$ [M+Na]$^+$: 502.2135; found: 502.2124.

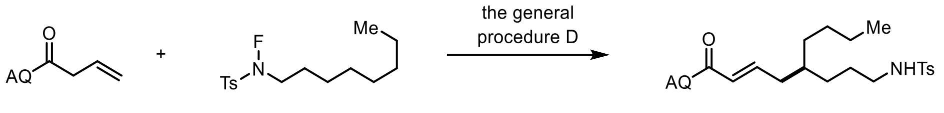

1a **2ah** **4ah**

Product **4ah** was prepared by the general procedure D. Purification using column chromatography (PE/EA = 5:1) afforded **4ah** as yellow oil (59.2 mg, 0.12 mmol, 60%, *r.r.* > 20:1, *E/Z* > 20:1). **^{1}H NMR** (600 MHz, $CDCl_3$) δ 9.83 (s, 1H), 8.84 (dd, *J* = 7.2, 1.2 Hz, 1H), 8.81 (dd, *J* = 4.2, 1.8 Hz, 1H), 8.16 (dd, *J* = 7.8, 1.2 Hz, 1H), 7.75 (d, *J* = 8.4 Hz, 2H), 7.56 - 7.49 (m, 2H), 7.46 (dd, *J* = 8.4, 4.2 Hz, 1H), 7.29 (d, *J* = 8.4 Hz, 2H), 6.96 (dt, *J* = 15.0, 7.8 Hz, 1H), 6.15 (d, *J* = 15.0 Hz, 1H), 4.58 (t, *J* = 6.6 Hz, 1H), 2.92 (dt, *J* = 6.6, 6.6 Hz, 2H), 2.39 (s, 3H), 2.24 - 2.14 (m, 2H), 1.51 - 1.45 (m, 3H), 1.27 - 1.23 (m, 8H), 0.88 (t, *J* = 7.2 Hz, 3H). **^{13}C NMR** (150 MHz, $CDCl_3$) δ 164.2, 148.3, 144.8, 143.5, 138.6, 137.2, 136.5, 134.8, 129.8, 128.1, 127.6, 127.3, 126.1, 121.8, 121.7, 116.9, 43.7, 37.2, 36.4, 33.2, 30.6, 29.0, 27.1, 23.1, 21.6, 14.2. **IR** ν_{max} (film): 3344, 2954, 2925, 2857, 1769, 1758, 1673, 1637, 1596, 1576, 1525, 1485, 1457, 1424, 1382, 1327, 1241, 1158, 1093, 1050, 975, 912, 826, 814, 792, 756, 609, 550 cm^{-1}. **HRMS** (ESI) m/z calcd for $C_{28}H_{35}N_3NaO_3S$ [M+Na]$^+$: 516.2291; found: 516.2284.

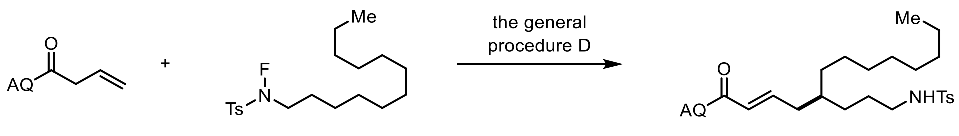

1a **2ai** **4ai**

Product **4ai** was prepared by the general procedure D. Purification using column chromatography (PE/EA = 5:1) afforded **4ai** as yellow oil (63.7 mg, 0.12 mmol, 58%, *r.r.* > 20:1, *E/Z* > 20:1). **^{1}H NMR** (600 MHz, $CDCl_3$) δ 9.83 (s, 1H), 8.84 (dd, *J* = 7.8, 1.8 Hz, 1H), 8.81 (dd, *J* = 4.2, 1.8 Hz, 1H), 8.16 (dd, *J* = 8.4, 1.8 Hz, 1H), 7.75 (d, *J* = 8.4 Hz, 2H), 7.56 - 7.49 (m, 2H), 7.45 (dd, *J* = 8.4, 4.2 Hz, 1H), 7.28 (d, *J* = 8.4 Hz, 2H), 6.96 (dt, *J* = 15.0, 7.8 Hz, 1H), 6.14 (d, *J*

= 15.0 Hz, 1H), 4.67 (t, *J* = 6.6 Hz, 1H), 2.29 (dt, *J* = 6.6, 6.6 Hz, 2H), 2.39 (s, 3H), 2.23 - 2.13 (m, 2H), 1.49 - 1.43 (m, 3H), 1.30 - 1.23 (m, 16H), 0.87 (t, *J* = 7.2 Hz, 3H). **^{13}C NMR** (150 MHz, $CDCl_3$) δ 164.2, 148.3, 144.8, 143.4, 138.6, 137.2, 136.5, 134.8, 129.8, 128.1, 127.6, 127.2, 126.1, 121.8, 121.7, 116.8, 43.7, 37.3, 36.4, 33.6, 32.0, 30.6, 30.0, 29.7, 29.5, 27.1, 26.8, 22.8, 21.6, 14.2. **IR** ν_{max} (film): 3343, 2925, 2854, 1770, 1682, 1639, 1597, 1576, 1527, 1486, 1458, 1425, 1385, 1329, 1240, 1159, 1094, 976, 884, 826, 814, 792, 756, 610, 551 cm^{-1}. **HRMS** (ESI) m/z calcd for $C_{32}H_{43}N_3NaO_3S$ [M+Na]$^+$: 572.2917; found: 572.2912.

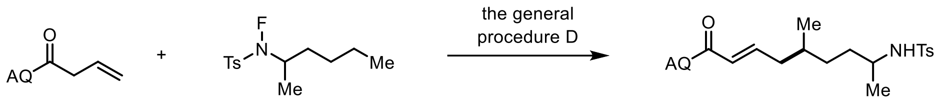

1a **2aj** **4aj**

Product **4aj** was prepared by the general procedure D. Purification using column chromatography (PE/EA = 5:1) afforded **4aj** as yellow oil (58.6 mg, 0.13 mmol, 63%, *r.r.* = 10:1, *E/Z* > 20:1, *d.r.* = 1:1). **^{1}H NMR** (600 MHz, $CDCl_3$) δ 9.84 (s, 1H), 8.85 (d, *J* = 7.8 Hz, 1H), 8.81 (dd, *J* = 4.2, 1.8 Hz, 1H), 8.16 (dd, *J* = 8.4, 1.8 Hz, 1H), 7.78 (d, *J* = 8.4 Hz, 1H), 7.76 (d, *J* = 8.4 Hz, 1H), 7.56 - 7.49 (m, 2H), 7.45 (dd, *J* = 8.4, 4.2 Hz, 1H), 7.30 - 7.28 (m, 2H), 6.98 - 6.92 (m, 1H), 6.15 - 6.11 (m, 1H), 4.59 - 4.57 (m, 1H), 3.31 - 3.26 (m, 1H), 2.41 (s, 1.5H), 2.40 (s, 1.5H), 2.21 - 2.14 (m, 1H), 2.06 - 1.99 (m, 1H), 1.55 - 1.49 (m, 1H), 1.45 - 1.19 (m, 4H), 1.04 (d, *J* = 6.6 Hz, 3H), 0.85 (d, *J* = 5.4 Hz, 1.5H), 0.84 (d, *J* = 5.4 Hz, 1.5H). **^{13}C NMR** (150 MHz, $CDCl_3$) δ 164.2, 148.3, 144.7, 143.32, 143.29, 138.6, 138.41, 138.40, 136.5, 134.8, 134.7, 129.7, 128.1, 127.6, 127.18, 127.16, 126.01, 126.00, 121.7, 121.6, 116.8, 50.31, 50.30, 39.52, 39.51, 35.04, 35.03, 32.6, 32.5, 21.9, 21.8, 21.6, 19.6, 19.5. **IR** ν_{max} (film): 3276, 2926, 1734, 1675, 1637, 1596, 1527, 1486, 1458, 1425, 1385, 1327, 1241, 1160, 1093, 1046, 977, 889, 826, 792, 757, 578, 551, 418 cm^{-1}. **HRMS** (ESI) m/z calcd for $C_{26}H_{31}N_3NaO_3S$ [M+Na]$^+$: 488.1978; found: 488.1972.

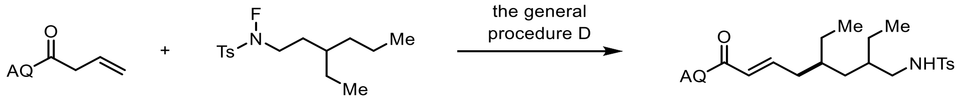

1a **2ak** **4ak**

Product **4ak** was prepared by the general procedure D. Purification using column chromatography (PE/EA = 5:1) afforded **4ak** as yellow oil (61.2 mg, 0.12 mmol, 62%, *r.r.* > 20:1, *E/Z* > 20:1, *d.r.* = 1:1). **^{1}H NMR** (600 MHz, $CDCl_3$) δ 9.84 (s, 0.5H), 9.83 (s, 0.5H), 8.85 - 8.81 (m, 2H), 8.16 (dd, *J* = 8.4, 1.8 Hz, 1H), 7.76 - 7.74 (m, 2H), 7.56 - 7.50 (m, 2H), 7.46 (dd, *J* = 8.4, 4.2 Hz, 1H), 7.30 (d, *J* = 7.8 Hz, 1H), 7.28 (d, *J* = 7.8 Hz, 1H), 6.98 - 6.93 (m, 1H), 6.17 - 6.14 (m, 1H), 4.65 - 4.53 (m, 1H), 2.89 - 2.80 (m, 2H), 2.40 (s, 1.5H), 2.39 (s, 1.5H), 2.24 - 2.14 (m, 2H),

1.51 - 1.45 (m, 2H), 1.34 - 1.27 (m, 4H), 1.23 - 1.17 (m, 1H), 1.16 - 1.10 (m, 1H), 0.85 (t, *J* = 7.2 Hz, 3H), 0.81 (t, *J* = 7.2 Hz, 1.5H), 0.80 (t, *J* = 7.2 Hz, 1.5H). **^{13}C NMR** (150 MHz, $CDCl_3$) *δ* 164.2, 164.1, 148.27, 148.26, 144.7, 144.5, 143.5, 143.4, 138.62, 138.60, 137.2, 137.1, 136.5, 134.78, 134.76, 129.82, 129.80, 128.1, 127.6, 127.252, 127.247, 126.23, 126.15, 121.8, 121.7, 121.6, 116.9, 116.8, 46.2, 46.1, 36.85, 36.76, 36.3, 36.2, 36.1, 35.5, 26.2, 26.1, 24.23, 24.18, 21.62, 21.61, 10.9, 10.8, 10.7, 10.6. **IR** ν_{max} (film): 3344, 2959, 2925, 2873, 1675, 1637, 1596, 1527, 1486, 1458, 1425, 1383, 1327, 1259, 1160, 1093, 977, 826, 814, 791, 757, 551 cm^{-1}. **HRMS** (ESI) m/z calcd for $C_{28}H_{35}N_3NaO_3S$ $[M+Na]^+$: 516.2291; found: 516.2299.

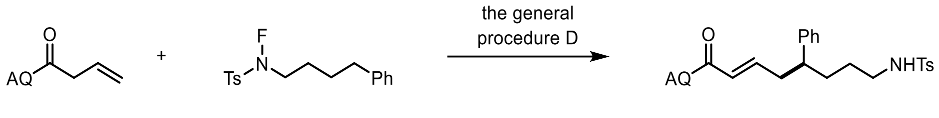

1a **2al** **4al**

Product **4al** was prepared by the general procedure D. Purification using column chromatography (PE/EA = 5:1) afforded **4al** as yellow oil (72.9 mg, 0.14 mmol, 71%, *r.r.* > 20:1, *E/Z* > 20:1). **^{1}H NMR** (600 MHz, $CDCl_3$) *δ* 9.78 (s, 1H), 8.79 - 8.78 (m, 2H), 8.14 (dd, *J* = 8.4, 1.8 Hz, 1H), 7.69 (d, *J* = 8.4 Hz, 2H), 7.53 - 7.47 (m, 2H), 7.43 (dd, *J* = 8.4, 4.2 Hz, 1H), 7.28 - 7.25 (m, 4H), 7.18 (t, *J* = 7.2 Hz, 1H), 7.08 (d, *J* = 8.4 Hz, 2H), 6.86 (dt, *J* = 15.0, 7.2 Hz, 1H), 6.07 (d, *J* = 15.0 Hz, 1H), 4.63 (t, *J* = 6.6 Hz, 1H), 2.86 (dt, *J* = 6.6, 6.6 Hz, 2H), 2.67 - 2.62 (m, 1H), 2.50 (dd, *J* = 7.2, 7.2 Hz, 2H), 2.39 (s, 3H), 1.73 - 1.67 (m, 1H), 1.59 - 1.53 (m, 1H), 1.37 - 1.32 (m, 1H), 1.29 - 1.25 (m, 1H). **^{13}C NMR** (150 MHz, $CDCl_3$) *δ* 164.0, 148.2, 143.8, 143.7, 143.4, 138.5, 137.1, 136.5, 134.7, 129.8, 128.7, 128.0, 127.6, 127.5, 127.2, 126.7, 126.2, 121.73, 121.65, 116.8, 45.1, 43.2, 39.8, 32.8, 27.6, 21.6. **IR** ν_{max} (film): 3342, 2923, 1770, 1675, 1637, 1596, 1525, 1485, 1456, 1424, 1386, 1326, 1241, 1158, 1092, 972, 791, 758, 700, 617, 549, 418 cm^{-1}. **HRMS** (ESI) m/z calcd for $C_{30}H_{31}N_3NaO_3S$ $[M+Na]^+$: 536.1978; found: 536.1986.

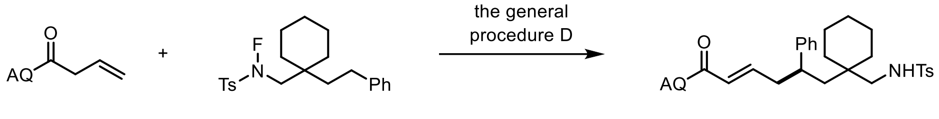

1a **2am** **4am**

Product **4am** was prepared by the general procedure D. Purification using column chromatography (PE/EA = 5:1) afforded **4am** as yellow oil (77.9 mg, 0.13 mmol, 67%, *r.r.* > 20:1, *E/Z* > 20:1). **^{1}H NMR** (600 MHz, $CDCl_3$) *δ* 9.76 (s, 1H), 8.80 - 8.77 (m, 2H), 8.14 (dd, *J* = 8.4, 1.8 Hz, 1H), 7.53 - 7.47 (m, 2H), 7.45 - 7.42 (m, 3H), 7.26 - 7.22 (m, 4H), 7.20 - 7.18 (m, 1H), 7.17 (d, *J* = 8.4 Hz, 2H), 6.78 (dt, *J* = 15.0, 7.2 Hz, 1H), 6.06 (d, *J* = 15.0 Hz, 1H), 3.77 - 3.74 (m, 1H), 2.79 - 2.73 (m, 2H), 2.47 (dd, *J* = 7.2, 7.2 Hz, 2H), 2.39 (s, 3H), 2.29 - 2.27 (m, 1H), 1.82 -

1.78 (m, 1H), 1.69 - 1.67 (m, 1H), 1.44 - 1.31 (m, 10H). **^{13}C NMR** (150 MHz, $CDCl_3$) δ 163.9, 148.2, 145.7, 143.5, 143.1, 138.5, 137.0, 136.5, 134.7, 129.5, 129.2, 128.0, 127.6, 127.5, 127.1, 127.0, 126.5, 121.7, 121.6, 116.8, 48.4, 42.6, 42.5, 40.7, 36.9, 34.5, 33.8, 26.1, 21.6, 21.5, 21.2. **IR** ν_{max} (film): 3339, 2926, 2856, 1679, 1644, 1597, 1526, 1486, 1454, 1425, 1385, 1328, 1261, 1237, 1161, 1094, 1064, 975, 910, 826, 792, 759, 732, 704, 663, 615, 566, 551 cm^{-1}. **HRMS** (ESI) m/z calcd for $C_{35}H_{39}N_3NaO_3S$ $[M+Na]^+$: 604.2604; found: 604.2597.

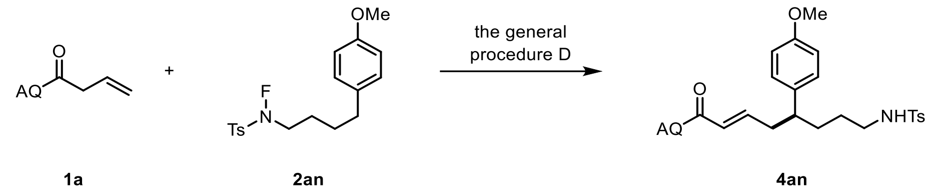

Product **4an** was prepared by the general procedure D. Purification using column chromatography (PE/EA = 5:1) afforded **4an** as yellow oil (67.4 mg, 0.12 mmol, 62%, *r.r.* > 20:1, *E/Z* > 20:1). **^{1}H NMR** (500 MHz, $CDCl_3$) δ 9.78 (s, 1H), 8.80 - 8.77 (m, 2H), 8.14 (dd, *J* = 8.5, 1.5 Hz, 1H), 7.70 (d, *J* = 8.0 Hz, 2H), 7.53 - 7.47 (m, 2H), 7.43 (dd, *J* = 8.0, 4.0 Hz, 1H), 7.26 (d, *J* = 8.5 Hz, 2H), 6.99 (d, *J* = 8.0 Hz, 2H), 6.85 (dt, *J* = 15.0, 7.5 Hz, 1H), 6.80 (d, *J* = 8.5 Hz, 2H), 6.06 (d, *J* = 15.0 Hz, 1H), 4.67 (t, *J* = 6.0 Hz, 1H), 3.76 (s, 3H), 2.85 (dt, *J* = 6.0, 6.0 Hz, 2H), 2.62 - 2.56 (m, 1H), 2.46 (dd, *J* = 7.5, 7.5 Hz, 2H), 2.39 (s, 3H), 1.71 - 1.64 (m, 1H), 1.56 - 1.48 (m, 1H), 1.33 - 1.25 (m, 2H). **^{13}C NMR** (125 MHz, $CDCl_3$) δ 164.1, 158.2, 148.2, 144.0, 143.4, 138.5, 137.0, 136.5, 135.7, 134.7, 129.8, 128.4, 128.0, 127.5, 127.2, 126.1, 121.7, 121.6, 116.8, 114.1, 55.3, 44.2, 43.2, 40.0, 33.0, 27.6, 21.6. **IR** ν_{max} (film): 2935, 1770, 1682, 1636, 1525, 1486, 1424, 1386, 1326, 1246, 1157, 1058, 827, 550 cm^{-1}. **HRMS** (ESI) m/z calcd for $C_{31}H_{33}N_3NaO_4S$ $[M+Na]^+$: 566.2084; found: 566.2087.

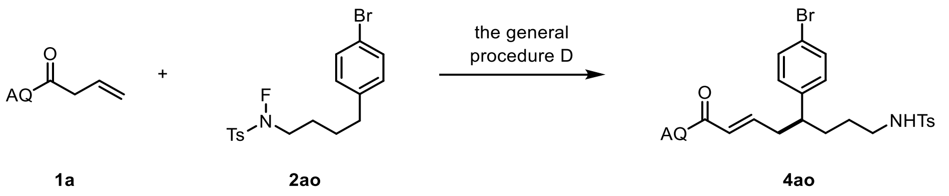

Product **4ao** was prepared by the general procedure D. Purification using column chromatography (PE/EA = 5:1) afforded **4ao** as yellow oil (74.5 mg, 0.13 mmol, 63%, *r.r.* > 20:1, *E/Z* > 20:1). **^{1}H NMR** (600 MHz, $CDCl_3$) δ 9.78 (s, 1H), 8.78 - 8.77 (m, 2H), 8.14 (dd, *J* = 7.8, 1.8 Hz, 1H), 7.69 (d, *J* = 8.4 Hz, 2H), 7.52 - 7.48 (m, 2H), 7.43 (dd, *J* = 8.4, 4.2 Hz, 1H), 7.36 (d, *J* = 8.4 Hz, 2H), 7.25 (d, *J* = 7.8 Hz, 2H), 6.94 (d, *J* = 8.4 Hz, 2H), 6.81 (dd, *J* = 15.0, 7.8 Hz, 1H), 6.05 (d, *J* = 15.0 Hz, 1H), 4.93 (t, *J* = 6.6 Hz, 1H), 2.85 (dt, *J* = 6.6, 6.6 Hz, 2H), 2.63 - 2.58 (m,

1H), 2.44 (dd, J = 7.8, 7.8 Hz, 2H), 2.39 (s, 3H), 1.71 - 1.65 (m, 1H), 1.56 - 1.49 (m, 1H), 1.34 - 1.28 (m, 1H), 1.25 - 1.21 (m, 1H). **^{13}C NMR** (150 MHz, $CDCl_3$) δ 163.9, 148.3, 143.4, 143.3, 142.8, 138.5, 137.1, 136.5, 134.6, 131.7, 129.8, 129.3, 128.0, 127.5, 127.1, 126.4, 121.74, 121.73, 120.3, 116.8, 44.6, 43.1, 39.5, 32.7, 27.5, 21.6. **IR** ν_{max} (film): 3340, 2925, 1734, 1680, 1526, 1485, 1458, 1424, 1385, 1326, 1241, 1157, 1093, 1071, 1009, 973, 825, 791, 758, 660, 606, 550 cm^{-1}. **HRMS** (ESI) m/z calcd for $C_{30}H_{30}BrN_3NaO_3S$ [M+Na]$^+$: 614.1083; found: 614.1091.

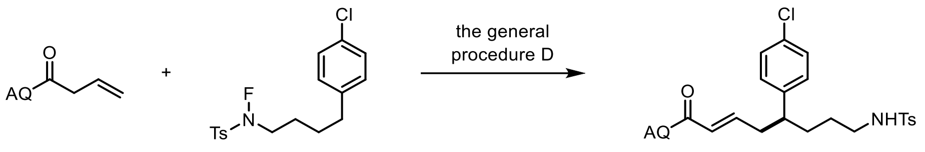

1a **2ap** **4ap**

Product **4ap** was prepared by the general procedure D. Purification using column chromatography (PE/EA = 5:1) afforded **4ap** as yellow oil (70.0 mg, 0.13 mmol, 64%, *r.r.* > 20:1, *E/Z* > 20:1). **^{1}H NMR** (600 MHz, $CDCl_3$) δ 9.78 (s, 1H), 8.79 - 8.79 (m, 2H), 8.15 (dd, J = 8.4, 1.8 Hz, 1H), 7.69 (d, J = 8.4 Hz, 2H), 7.54 - 7.49 (m, 2H), 7.45 (dd, J = 8.4, 4.2 Hz, 1H), 7.27 (d, J = 8.4 Hz, 2H), 7.24 (d, J = 8.4 Hz, 2H), 7.02 (d, J = 8.4 Hz, 2H), 6.82 (dt, J = 15.0, 7.8 Hz, 1H), 6.07 (d, J = 15.0 Hz, 1H), 4.53 (t, J = 6.6 Hz, 1H), 2.87 (dt, J = 6.6, 6.6 Hz, 2H), 2.67 - 2.62 (m, 1H), 2.48 (dd, J = 7.2, 7.2 Hz, 2H), 2.41 (s, 3H), 1.74 - 1.69 (m, 1H), 1.58 - 1.52 (m, 1H), 1.36 - 1.26 (m, 2H). **^{13}C NMR** (150 MHz, $CDCl_3$) δ 163.9, 148.3, 143.5, 143.3, 142.2, 138.6, 137.1, 136.5, 134.6, 132.3, 129.8, 128.94, 128.90, 128.1, 127.6, 127.2, 126.5, 121.78, 121.75, 116.9, 44.6, 43.2, 39.7, 32.8, 27.7, 21.6. **IR** ν_{max} (film): 2922, 1770, 1675, 1637, 1595, 1525, 1486, 1457, 1424, 1378, 1325, 1241, 1157, 1091, 1012, 971, 825, 791, 549, 418 cm^{-1}. **HRMS** (ESI) m/z calcd for $C_{30}H_{30}ClN_3NaO_3S$ [M+Na]$^+$: 570.1589; found: 570.1588.

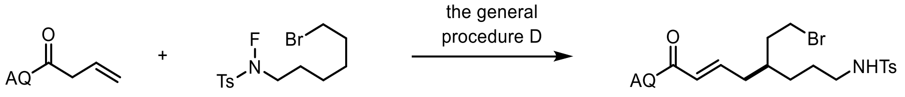

1a **2aq** **4aq**

Product **4aq** was prepared by the general procedure D. Purification using column chromatography (PE/EA = 5:1) afforded **4aq** as yellow oil (67.3 mg, 0.12 mmol, 62%, *r.r.* > 20:1, *E/Z* > 20:1). **^{1}H NMR** (600 MHz, $CDCl_3$) δ 9.86 (s, 1H), 8.84 - 8.81 (m, 2H), 8.18 - 8.16 (m, 1H), 7.75 (d, J = 8.4, 3.0 Hz, 2H), 7.57 - 7.51 (m, 2H), 7.48 - 7.45 (m, 1H), 7.31 - 7.29 (m, 2H), 6.97 - 6.92 (m, 1H), 6.21 - 6.18 (m, 1H), 4.69 (t, J = 6.6 Hz, 1H), 3.41 - 3.38 (m, 2H), 2.97 - 2.93 (m, 2H), 2.40 (s, 3H), 2.26 - 2.21 (m, 2H), 1.85 - 1.75 (m, 3H), 1.53 - 1.48 (m, 2H), 1.32 - 1.28 (m, 2H). **^{13}C NMR** (150 MHz, $CDCl_3$) δ 163.9, 148.3, 143.6, 143.3, 138.6, 137.1, 136.6, 134.6, 129.9,

128.1, 127.6, 127.2, 126.8, 121.8, 116.9, 43.5, 36.6, 35.8, 35.7, 31.4, 30.0, 26.9, 21.7. **IR** ν_{max} (film): 3344, 2956, 2928, 2869, 1739, 1682, 1638, 1596, 1576, 1526, 1486, 1463, 1425, 1385, 1328, 1241, 1184, 1159, 1093, 1046, 976, 885, 827, 815, 792, 758, 610, 572, 550 cm^{-1}. **HRMS** (ESI) m/z calcd for $C_{26}H_{30}BrN_3NaO_3S$ $[M+Na]^+$: 566.1083; found: 566.1095.

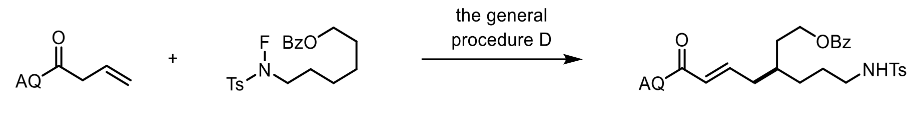

1a **2ar** **4ar**

Product **4ar** was prepared by the general procedure D. Purification using column chromatography (PE/EA = 3:1) afforded **4ar** as yellow oil (73.7 mg, 0.13 mmol, 63%, *r.r.* > 20:1, *E/Z* > 20:1). **^{1}H NMR** (600 MHz, $CDCl_3$) δ 9.85 (s, 1H), 8.84 - 8.79 (m, 2H), 8.17 (dd, *J* = 8.4, 1.8 Hz, 1H), 8.02 (d, *J* = 8.4 Hz, 2H), 7.73 (d, *J* = 8.4 Hz, 2H), 7.56 - 7.51 (m, 3H), 7.47 - 7.42 (m, 3H), 7.28 (d, *J* = 8.4 Hz, 2H), 6.98 (dt, *J* = 15.0, 7.2 Hz, 1H), 6.20 (d, *J* = 15.0 Hz, 1H), 4.67 (t, *J* = 6.6 Hz, 1H), 4.37 - 4.33 (m, 2H), 2.94 (dt, *J* = 6.6, 6.6 Hz, 2H), 2.39 (s, 3H), 2.30 (dd, *J* = 7.2, 7.2 Hz, 2H), 1.77 - 1.74 (m, 3H), 1.56 - 1.51 (m, 2H), 1.39 - 1.35 (m, 2H). **^{13}C NMR** (150 MHz, $CDCl_3$) δ 166.7, 163.9, 148.3, 143.6, 143.5, 138.6, 137.1, 136.5, 134.7, 133.1, 130.3, 129.8, 129.7, 128.6, 128.1, 127.6, 127.2, 126.7, 121.79, 121.76, 116.9, 62.9, 43.5, 36.1, 34.4, 32.4, 30.4, 27.0, 21.6. **IR** ν_{max} (film): 3341, 2924, 1715, 1679, 1640, 1597, 1526, 1485, 1451, 1425, 1386, 1327, 1273, 1158, 1094, 1070, 1025, 976, 912, 826, 791, 712, 660, 609, 550 cm^{-1}. **HRMS** (ESI) m/z calcd for $C_{33}H_{35}N_3NaO_5S$ $[M+Na]^+$: 608.2190; found: 608.2179.

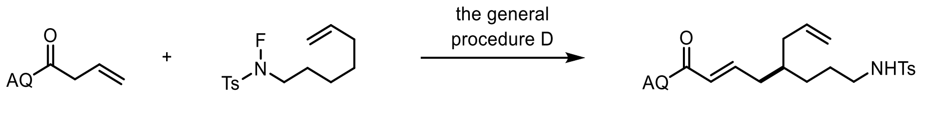

1a **2as** **4as**

Product **4as** was prepared by the general procedure D. Purification using column chromatography (PE/EA = 5:1) afforded **4as** as yellow oil (51.5 mg, 0.11 mmol, 54%, *r.r.* > 20:1, *E/Z* > 20:1). **^{1}H NMR** (600 MHz, $CDCl_3$) δ 9.84 (s, 1H), 8.84 - 8.80 (m, 2H), 8.16 (d, *J* = 8.4 Hz, 1H), 7.75 (d, *J* = 7.8 Hz, 2H), 7.56 - 7.50 (m, 2H), 7.46 (dd, *J* = 8.4, 4.2 Hz, 1H), 7.28 (d, *J* = 7.8 Hz, 2H), 6.96 (dt, *J* = 15.0, 7.2 Hz, 1H), 6.15 (d, *J* = 15.0 Hz, 1H), 5.73 - 5.67 (m, 1H), 5.04 - 5.00 (m, 2H), 4.76 (t, *J* = 6.6 Hz, 1H), 2.92 (dt, *J* = 6.6, 6.6 Hz, 2H), 2.39 (s, 3H), 2.25 - 2.20 (m, 1H), 2.19 - 2.14 (m, 1H), 2.02 (dd, *J* = 7.2, 7.2 Hz, 2H), 1.61 - 1.56 (m, 1H), 1.52 - 1.45 (m, 2H), 1.28 - 1.25 (m, 2H). **^{13}C NMR** (150 MHz, $CDCl_3$) δ 164.1, 148.3, 144.4, 143.4, 138.5, 137.1, 136.5, 136.3, 134.7, 129.8, 128.1, 127.6, 127.2, 126.3, 121.8, 121.7, 117.0, 116.9, 43.5, 37.9, 37.0, 36.1, 30.3, 27.1, 21.6. **IR** ν_{max} (film): 3343, 2923, 1675, 1638, 1596, 1527, 1486, 1425, 1385, 1328,

1240, 1159, 1093, 977, 911, 826, 792, 733, 550 cm^{-1}. **HRMS** (ESI) m/z calcd for $C_{27}H_{31}N_3NaO_3S$ $[M+Na]^+$: 500.1978; found: 500.1968.

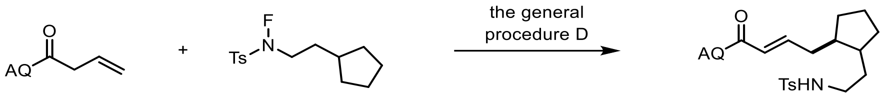

1a **2au** **4au**

Product **4au** was prepared by the general procedure D. Purification using column chromatography (PE/EA = 5:1) afforded **4au** as yellow oil (59.2 mg, 0.12 mmol, 62%, *r.r.* = 14:1, *E/Z* > 20:1, *d.r.* = 2:1). **^{1}H NMR** (600 MHz, $CDCl_3$) δ 9.83 (s, 1H), 8.85 - 8.81 (m, 2H), 8.17 (dd, J = 8.4, 1.8 Hz, 1H), 7.76 - 7.74 (m, 2H), 7.57 - 7.51 (m, 2H), 7.46 (dd, J = 7.8, 4.2 Hz, 1H), 7.31 - 7.29 (m, 2H), 7.00 - 6.94 (m, 1H), 6.17 - 6.13 (m, 1H), 4.48 (t, J = 6.0 Hz, 0.67H), 4.45 (t, J = 6.0 Hz, 0.33H), 3.05 - 2.98 (m, 1H), 2.96 - 2.90 (m, 1H), 2.41 (s, 1H), 2.40 (s, 2H), 2.36 - 2.33 (m, 0.67H), 2.23 - 2.19 (m, 0.33H), 2.12 - 2.07 (m, 0.67H), 2.01 - 1.99 (m, 0.33H), 1.88 - 1.76 (m, 2H), 1.70 - 1.66 (m, 1H), 1.56 - 1.53 (m, 2H), 1.48 - 1.39 (m, 1H), 1.35 - 1.26 (m, 3H), 1.24 - 1.22 (m, 0.33H), 1.15 - 1.09 (m, 0.67H). **^{13}C NMR** (150 MHz, $CDCl_3$) δ 164.31, 164.26, 148.27, 148.26, 145.6, 145.2, 143.5, 143.4, 138.6, 137.2, 136.5, 134.7, 129.81, 129.80, 129.7, 128.1, 127.6, 127.3, 127.2, 127.1, 125.5, 125.4, 121.8, 121.67, 121.65, 116.9, 116.8, 45.1, 42.7, 42.6, 42.5, 41.5, 39.9, 37.4, 35.0, 32.4, 32.1, 32.0, 30.2, 29.9, 23.8, 22.4, 21.6. **IR** ν_{max} (film): 2924, 1770, 1683, 1527, 1487, 1375, 1326, 1245, 1158, 1053, 814, 550, 418 cm^{-1}. **HRMS** (ESI) m/z calcd for $C_{27}H_{31}N_3NaO_3S$ $[M+Na]^+$: 500.1978; found: 500.1988.

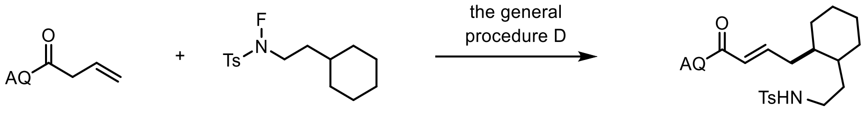

1a **2av** **4av**

Product **4av** was prepared by the general procedure D. Purification using column chromatography (PE/EA = 5:1) afforded **4av** as yellow oil (62.9 mg, 0.13 mmol, 64%, *r.r.* > 20:1, *E/Z* > 20:1, *d.r.* = 1:1). **^{1}H NMR** (600 MHz, $CDCl_3$) δ 9.83 (s, 1H), 8.84 - 8.80 (m, 2H), 8.15 (dd, J = 8.4, 1.2 Hz, 1H), 7.77 (d, J = 8.4 Hz, 1H), 7.76 (d, J = 8.4 Hz, 1H), 7.55 - 7.49 (m, 2H), 7.45 (dd, J = 7.8, 4.2 Hz, 1H), 7.29 - 7.27 (m, 2H), 6.98 - 3.91 (m, 1H), 6.15 - 6.11 (m, 1H), 4.81 - 4.78 (m, 1H), 3.05 - 2.97 (m, 1H), 2.94 - 2.86 (m, 1H), 2.39 (s, 1.5H), 2.38 (s, 1.5H), 2.36 - 2.33 (m, 0.5H), 2.16 - 2.11 (m, 0.5H), 2.09 - 2.01 (m, 1H), 1.78 - 1.63 (m, 3H), 1.50 - 1.41 (m, 2H), 1.39 - 1.25 (m, 4H), 1.23 - 1.16 (m, 1H), 1.13 - 1.09 (m, 1H), 1.03 - 0.97 (m, 0.5H), 0.92 - 0.85 (m, 0.5H). **^{13}C NMR** (150 MHz, $CDCl_3$) δ 164.21, 164.16, 148.3, 145.5, 144.8, 143.44, 143.42, 138.6,

137.2, 137.1, 136.5, 134.7, 129.80, 129.79, 128.1, 127.6, 127.3, 126.1, 125.7, 121.74, 121.67, 116.8, 41.7, 41.3, 41.0, 38.9, 38.6, 36.3, 36.2, 33.4, 32.1, 31.5, 28.4, 28.2, 25.95, 25.90, 23.4, 23.2, 21.60, 21.59. **IR** ν_{max} (film): 3343, 2924, 2854, 1770, 1675, 1637, 1596, 1526, 1486, 1458, 1425, 1385, 1328, 1241, 1159, 1093, 1047, 977, 827, 815, 792, 757, 609, 550 cm^{-1}. **HRMS** (ESI) m/z calcd for $C_{28}H_{33}N_3NaO_3S$ $[M+Na]^+$: 514.2135; found: 514.2129.

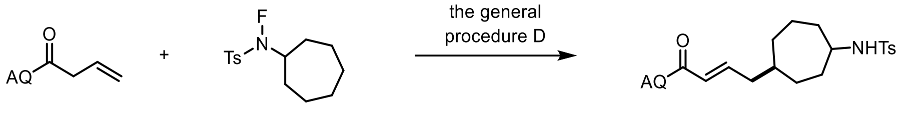

1a **2aw** **4aw**

Product **4aw** was prepared by the general procedure D. Purification using column chromatography (PE/EA = 5:1) afforded **4aw** as yellow oil (63.9 mg, 0.13 mmol, 67%, *r.r.* > 20:1, *E/Z* > 20:1, *d.r.* = 1:1). **^{1}H NMR** (600 MHz, $CDCl_3$) δ 9.82 (s, 1H), 8.84 - 8.79 (m, 2H), 8.17 - 8.15 (m, 1H), 7.77 - 7.74 (m, 2H), 7.56 - 7.49 (m, 2H), 7.47 - 7.45 (m, 1H), 7.29 - 7.27 (m, 2H), 6.99 - 6.92 (m, 1H), 6.13 (d, *J* = 15.0 Hz, 1H), 4.71 - 4.68 (m, 0.5H), 4.65 - 4.63 (m, 0.5H), 3.40 - 3.35 (m, 0.5H), 3.33 - 3.27 (m, 0.5H), 2.41 (s, 3H), 2.17 - 2.13 (m, 2H), 1.87 - 1.70 (m, 3H), 1.67 - 1.59 (m, 3H), 1.57 - 1.45 (m, 2H), 1.36 - 1.28 (m, 2H), 1.12 - 1.06 (m, 1H). **^{13}C NMR** (150 MHz, $CDCl_3$) δ 164.17 164.15, 148.2, 144.82, 144.76, 143.3, 138.6, 138.4, 138.3, 136.5, 134.7, 129.8, 128.1, 127.6, 127.09, 127.06, 126.05, 126.02, 121.8, 121.7, 116.8, 55.2, 54.3, 40.5, 40.4, 39.1, 38.5, 36.5, 35.9, 35.4, 34.7, 33.8, 32.8, 30.4, 28.4, 23.1, 21.6, 21.5. **IR** ν_{max} (film): 3344, 3276, 3046, 2925, 2856, 1676, 1640, 1596, 1526, 1485, 1460, 1425, 1385, 1327, 1260, 1184, 1157, 1094, 1048, 977, 947, 910, 884, 826, 815, 757, 707, 666, 609, 573, 550 cm^{-1}. **HRMS** (ESI) m/z calcd for $C_{27}H_{31}N_3NaO_3S$ $[M+Na]^+$: 500.1978; found: 500.1974.

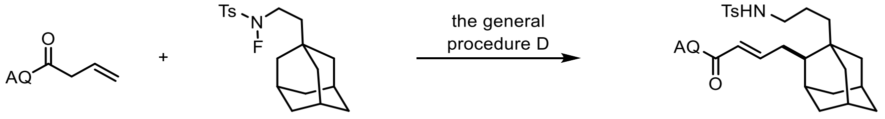

1a **2ax** **4ax**

Product **4ax** was prepared by the general procedure D. Purification using column chromatography (PE/EA = 5:1) afforded **4ax** as yellow oil (73.9 mg, 0.14 mmol, 68%, *r.r.* > 20:1, *E/Z* > 20:1). **^{1}H NMR** (600 MHz, $CDCl_3$) δ 9.82 (s, 1H), 8.85 (dd, *J* = 7.8, 1.2 Hz, 1H), 8.82 (dd, *J* = 4.2, 1.8 Hz, 1H), 8.17 (dd, *J* = 8.4, 1.8 Hz, 1H), 7.77 (d, *J* = 8.4 Hz, 2H), 7.57 - 7.50 (m, 2H), 7.46 (dd, *J* = 8.4, 4.2 Hz, 1H), 7.30 (d, *J* = 8.4 Hz, 2H), 6.90 (dt, *J* = 15.0, 8.4 Hz, 1H), 6.12 (d, *J* = 15.0 Hz, 1H), 4.66 (t, *J* = 6.0 Hz, 1H), 3.02 - 2.96 (m, 1H), 2.93 - 2.87 (m, 1H), 2.38 (s, 3H),

2.26 - 2.21 (m, 2H), 1.90 - 1.86 (m, 2H), 1.79 - 1.73 (m, 3H), 1.69 - 1.66 (m, 1H), 1.59 - 1.53 (m, 5H), 1.47 - 1.39 (m, 3H), 1.27 - 1.22 (m, 2H). **^{13}C NMR** (150 MHz, $CDCl_3$) δ 164.2, 148.3, 145.8, 143.5, 138.6, 137.1, 136.5, 134.7, 129.9, 128.1, 127.6, 127.3, 125.5, 121.8, 121.7, 116.9, 45.9, 43.0, 40.6, 38.6, 38.2, 38.0, 37.7, 34.4, 30.8, 30.6, 30.5, 28.5, 28.3, 21.6. **IR** ν_{max} (film): 2906, 1770, 1683, 1636, 1525, 1486, 1456, 1424, 1378, 1328, 1245, 1158, 1058, 826, 719, 550, 418 cm^{-1}. **HRMS** (ESI) m/z calcd for $C_{32}H_{37}N_3NaO_3S$ $[M+Na]^+$: 566.2448; found: 566.2446.

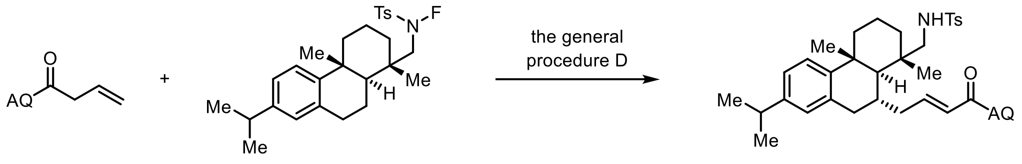

1a **2ay** **4ay**

Product **4ay** was prepared by the general procedure D. Purification using column chromatography (PE/EA = 5:1) afforded **4ay** as yellow oil (93.5 mg, 0.14 mmol, 72%, *r.r.* > 20:1, *E/Z* > 20:1, *dr* > 20:1). **^{1}H NMR** (600 MHz, $CDCl_3$) δ 9.88 (s, 1H), 8.89 (d, *J* = 7.2 Hz, 1H), 8.85 (dd, *J* = 4.2, 1.8 Hz, 1H), 8.18 (d, *J* = 8.4, 1.8 Hz, 1H), 7.75 (d, *J* = 7.8 Hz, 2H), 7.59 - 7.53 (m, 2H), 7.48 (dd, *J* = 7.8, 4.2 Hz, 1H), 7.31 (d, *J* = 7.8 Hz, 2H), 7.07 - 7.04 (m, 2H), 6.96 - 6.91 (m, 2H), 6.03 (d, *J* = 15.6 Hz, 1H), 4.60 (t, *J* = 6.6 Hz, 1H), 3.10 - 3.06 (m, 1H), 2.88 - 2.83 (m, 2H), 2.69 - 2.66 (m, 1H), 2.55 - 2.52 (m, 1H), 2.33 (s, 3H), 2.13 - 2.08 (m, 2H), 1.99 - 1.96 (m, 1H), 1.87 - 1.78 (m, 2H), 1.73 - 1.69 (m, 1H), 1.61 - 1.57 (m, 1H), 1.51 - 1.46 (m, 1H), 1.26 - 1.23 (m, 9H), 1.21 - 1.19 (m, 1H), 1.11 (s, 3H), 0.95 (d, *J* = 6.6 Hz, 1H). **^{13}C NMR** (150 MHz, $CDCl_3$) δ 164.1, 148.3, 147.3, 146.2, 144.4, 143.8, 138.6, 136.7, 136.6, 135.2, 134.8, 130.0, 128.1, 127.6, 127.4, 127.2, 125.9, 124.4, 121.8, 121.7, 121.5, 116.9, 55.4, 53.4, 40.6, 39.2, 38.6, 38.5, 37.1, 33.7, 33.2, 33.1, 24.3, 24.2, 23.0, 21.6, 18.3, 17.9. **IR** ν_{max} (film): 3346, 3282, 2927, 2868, 1683, 1637, 1596, 1525, 1486, 1458, 1424, 1385, 1328, 1160, 1093, 1065, 975, 910, 826, 792, 732, 663, 607, 552 cm^{-1}. **HRMS** (ESI) m/z calcd for $C_{40}H_{47}N_3NaO_3S$ $[M+Na]^+$: 672.3230; found: 672.3223.

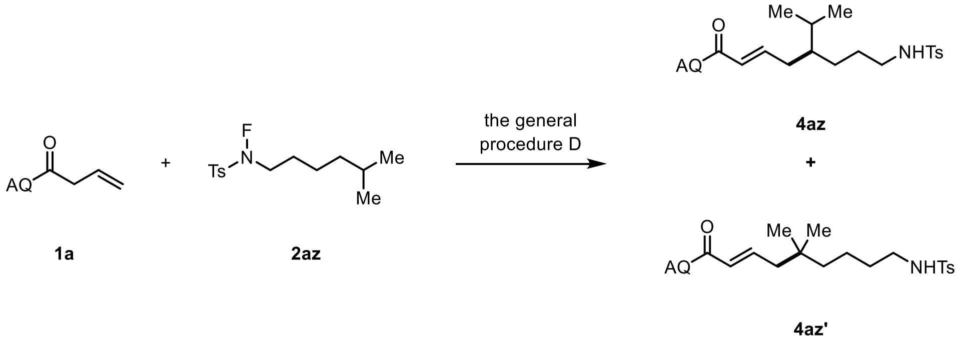

1a **2az** **4az'**

Product **4az** and **4az′** was prepared by the general procedure D. Purification using column chromatography (PE/EA = 5:1) afforded a mixture of **4az** and **4az′** as yellow oil (67.1 mg, 0.14 mmol, 70%, *r.r.* > 20:1, *E/Z* > 20:1, **4az**:**4az′** = 1.5:1). **^{1}H NMR** (600 MHz, $CDCl_3$) δ 9.83 (s, 0.4H), 9.82 (s, 0.6H), 8.85 - 8.79 (m, 2H), 8.16 (dd, *J* = 8.4, 1.8 Hz, 1H), 7.76 - 7.74 (m, 2H), 7.56 - 7.49 (m, 2H), 7.45 (dd, *J* = 8.4, 4.2 Hz, 1H), 7.29 - 7.27 (m, 2H), 7.03 (dt, *J* = 15.0, 7.8 Hz, 0.4H), 6.96 (dt, *J* = 15.0, 7.8 Hz, 0.6H), 6.16 - 6.13 (m, 1H), 4.69 - 4.67 (m, 1H), 2.97 - 2.91 (m, 2H), 2.40 (s, 1.2H), 2.39 (s, 1.8H), 2.26 - 2.21 (m, 0.6H), 2.12 - 2.10 (m, 1H), 2.09 - 2.06 (m, 0.4H), 1.71 - 1.66 (m, 0.6H), 1.49 - 1.47 (m, 0.4H), 1.44 - 1.42 (m, 1H), 1.35 - 1.26 (m, 2H), 1.24 - 1.14 (m, 2H), 0.89 (s, 2.4H), 0.84 (d, *J* = 6.6 Hz, 1.8H), 0.82 (d, *J* = 6.6 Hz, 1.8H). **^{13}C NMR** (150 MHz, $CDCl_3$) δ 164.19, 164.16, 148.3, 145.8, 143.6, 143.41, 143.39, 138.61, 138.59, 137.23, 137.16, 136.5, 134.8, 134.7, 129.8, 128.1, 127.6, 127.23, 127.21, 126.7, 125.7, 121.8, 121.7, 116.9, 116.8, 44.8, 43.7, 43.5, 43.4, 41.6, 34.0, 33.5, 30.5, 29.8, 29.5, 27.9, 27.5, 27.2, 21.61, 21.59, 21.2, 19.2, 19.1. **IR** ν_{max} (film): 3344, 2955, 2928, 2869, 1736, 1682, 1638, 1596, 1576, 1526, 1486, 1463, 1425, 1385, 1328, 1241, 1184, 1159, 1093, 1047, 976, 827, 815, 792, 758, 660, 610, 558, 572, 550 cm^{-1}. **HRMS** (ESI) m/z calcd for $C_{27}H_{33}N_3NaO_3S$ $[M+Na]^+$: 502.2135; found: 502.2123.

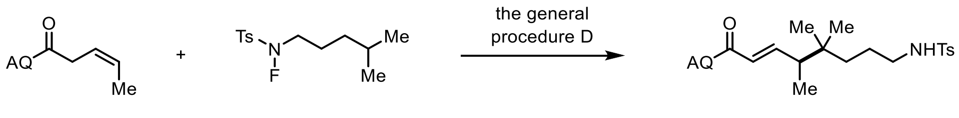

1aa **2a** **7a**

Product **7a** was prepared by the general procedure D. Purification using column chromatography (PE/EA = 5:1) afforded **7a** as yellow oil (68.0 mg, 0.14 mmol, 71%, *r.r.* > 20:1 *E/Z* > 20:1). **^{1}H NMR** (600 MHz, $CDCl_3$) δ 9.84 (s, 1H), 8.84 (dd, *J* = 7.8, 1.8 Hz, 1H), 8.81 (dd, *J* = 4.2, 1.8 Hz, 1H), 8.16 (dd, *J* = 8.4, 1.8 Hz, 1H), 7.75 (d, *J* = 8.4 Hz, 2H), 7.56 - 7.49 (m, 2H), 7.45 (dd, *J* = 8.4, 4.2 Hz, 1H), 7.27 (d, *J* = 8.4 Hz, 2H), 7.01 (dd, *J* = 15.0, 9.0 Hz, 1H), 6.11 (d, *J* = 15.0 Hz, 1H), 4.91 (t, *J* = 6.6 Hz, 1H), 2.90 (dt, *J* = 6.6, 6.6 Hz, 2H), 2.38 (s, 3H), 2.21 - 2.16 (m, 1H), 1.46 - 1.39 (m, 2H), 1.22 - 1.13 (m, 2H), 0.99 (d, *J* = 7.2 Hz, 3H), 0.82 (s, 3H), 0.81 (s, 3H). **^{13}C NMR** (150 MHz, $CDCl_3$) δ 164.3, 149.2, 148.2, 143.4, 138.5, 137.1, 136.6, 134.7, 129.8, 128.1, 127.6, 127.2, 124.5, 121.74, 121.66, 116.9, 45.2, 44.1, 37.4, 35.5, 24.44, 24.41, 24.3, 21.6, 14.5. **IR** ν_{max} (film): 2913, 2846, 1671, 1640, 1526, 1485, 1461, 1426, 1325, 1157, 1094, 1025, 827, 792, 728, 717, 659, 615, 563, 551, 533 cm^{-1}. **HRMS** (ESI) m/z calcd for $C_{27}H_{33}N_3NaO_3S$ $[M+Na]^+$: 502.2135; found: 502.2131.

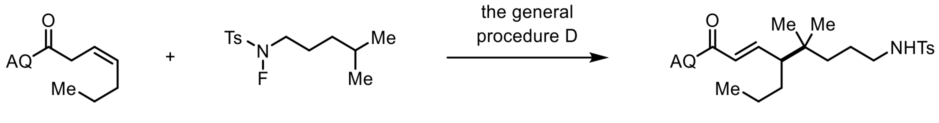

1ab **2a** **7b**

Product **7b** was prepared by the general procedure D. Purification using column chromatography (PE/EA = 5:1) afforded **7b** as yellow oil (69.0 mg, 0.14 mmol, 68%, *r.r.* > 20:1 *E/Z* > 20:1). **^{1}H NMR** (500 MHz, $CDCl_3$) δ 9.85 (s, 1H), 8.85 (dd, *J* = 7.0, 1.5 Hz, 1H), 8.81 (dd, *J* = 4.5, 2.0 Hz, 1H), 8.15 (dd, *J* = 8.0, 1.5 Hz, 1H), 7.74 (d, *J* = 8.0 Hz, 2H), 7.55 - 7.49 (m, 2H), 7.46 (dd, *J* = 8.5, 4.0 Hz, 1H), 7.25 (d, *J* = 8.0 Hz, 2H), 6.84 (dd, *J* = 15.0, 10.5 Hz, 1H), 6.08 (d, *J* = 15.0 Hz, 1H), 4.91 (t, *J* = 6.0, Hz, 1H), 2.88 (dt, *J* = 6.0, 6.0 Hz, 2H), 2.37 (s, 3H), 1.92 (td, J = 11.0, 2.5 Hz, 1H), 1.48 - 1.38 (m, 3H), 1.36 - 1.29 (m, 1H), 1.25 - 1.15 (m, 3H), 1.09 - 1.02 (m, 1H), 0.86 (t, *J* = 8.4 Hz, 3H), 0.83 (s, 3H), 0.81 (s, 3H). **^{13}C NMR** (150 MHz, $CDCl_3$) δ 164.0, 148.2, 148.1, 143.3, 138.5, 137.1, 136.5, 134.8, 129.7, 128.0, 127.6, 127.2, 126.0, 121.73, 121.65, 116.8, 51.8, 44.1, 37.6, 35.6, 30.5, 24.8, 24.3, 21.6, 21.4, 14.2. **IR** ν_{max} (film): 2954, 2928, 2870, 1671, 1636, 1596, 1523, 1485, 1424, 1323, 1305, 1154, 1092, 977, 825, 812, 790, 704, 659, 615, 563, 503, 484, 462, 439, 421 cm^{-1}. **HRMS** (ESI) m/z calcd for $C_{29}H_{37}N_3NaO_3S$ $[M+Na]^+$: 530.2448; found: 530.2448.

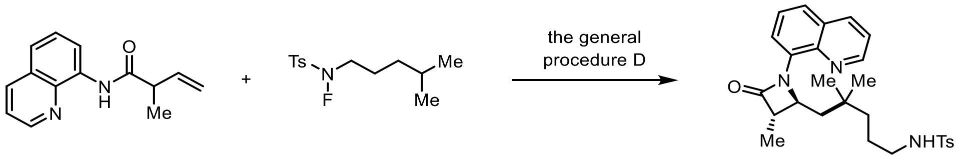

1b **2a** **8**

Product **8** was prepared by the general procedure D. Purification using column chromatography (PE/EA = 5:1) afforded **8** as yellow oil (30.7 mg, 0.06 mmol, 32%, *d.r.* > 20:1) with 30% of **1b** recovered (13.6 mg, 0.06 mmol). **^{1}H NMR** (600 MHz, $CDCl_3$) δ 8.79 (dd, *J* = 4.2, 1.8 Hz, 1H), 8.24 (dd, *J* = 7.8, 1.2 Hz, 1H), 8.11 (dd, *J* = 8.4, 1.8 Hz, 1H), 7.72 (d, *J* = 8.4 Hz, 2H), 7.57 (dd, *J* = 7.8, 1.2 Hz, 1H), 7.51 - 7.48 (m, 1H), 7.39 (dd, *J* = 8.4, 4.2 Hz, 1H), 7.26 (d, *J* = 8.4 Hz, 2H), 4.87 (dt, *J* = 11.4, 1.8 Hz, 1H), 4.74 (t, *J* = 6.0 Hz, 1H), 2.95 - 2.92 (m, 1H), 2.91 - 2.83 (m, 2H), 2.39 (s, 3H), 1.85 (dd, *J* = 13.8, 1.8 Hz, 1H), 1.48 (d, *J* = 7.2 Hz, 3H), 1.44 - 1.38 (m, 1H), 1.37 - 1.31 (m, 1H), 1.29 (dd, *J* = 13.8, 10.8 Hz, 1H), 1.19 - 1.16 (m, 2H), 0.93 (s, 3H), 0.85 (s, 3H). **^{13}C NMR** (150 MHz, $CDCl_3$) δ 170.2, 149.0, 143.5, 140.8, 137.1, 136.1, 133.0, 129.8, 129.1, 127.2, 126.8, 124.1, 122.3, 121.5, 62.7, 52.9, 44.7, 44.1, 39.8, 32.6, 27.3, 27.2, 24.4, 21.6, 13.7. **IR** ν_{max} (film): 2959, 2927, 2864, 1718, 1594, 1502, 1472, 1400, 1346, 1323, 1305, 1152, 1092, 900, 824,

812, 790, 730, 706, 659, 637, 574, 549, 507, 492 cm^{-1}. **HRMS** (ESI) m/z calcd for $C_{27}H_{33}N_3NaO_3S$ [M+Na]$^+$: 502.2135; found: 502.2140.

The general procedure E (for products 5a-5h, 5k):

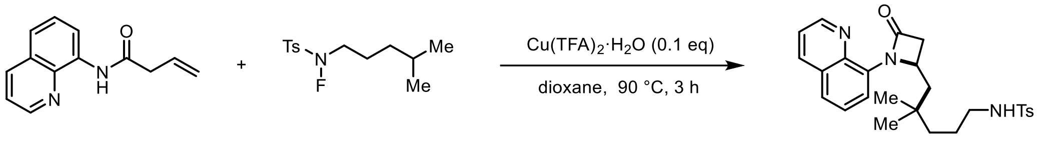

1a **2a** **5a**

To a dry Schlenk flask were added **1a** (42.4 mg, 0.20 mmol, 1.0 equiv), **2a** (137 mg, 0.50 mmol, 2.5 equiv), $Cu(TFA)_2{\cdot}H_2O$ (5.8 mg, 0.02 mmol, 0.10 equiv), and anhydrous dioxane (3.0 mL). The mixture was degassed for three times with argon and stirred at 90 °C (oil bath) for 3 h. Once completion, the reaction was cooled to room temperature. The reaction mixture was filtered by celite, and the filtrate was concentrated *in vacuo*. Further purification by a flash column chromatography using eluents (PE/EA = 5:1) afforded the desired product **5a** as yellow oil (67.9 mg, 0.15 mmol, 73%). **^{1}H NMR** (600 MHz, $CDCl_3$) δ 8.80 (dd, J = 4.2, 1.8 Hz, 1H), 8.22 (d, J = 7.2 Hz, 1H), 8.11 (dd, J = 8.4, 1.8 Hz, 1H), 7.73 (d, J = 7.8 Hz, 2H), 7.57 (dd, J = 7.8 Hz, 1H), 7.51 - 7.48 (m, 1H), 7.40 (dd, J = 8.4, 4.2 Hz, 1H), 7.25 (d, J = 7.8 Hz, 2H), 5.26 - 5.21 (m, 1H), 4.92 (t, J = 6.0 Hz, 1H), 3.32 (dd, J = 15.0, 5.4 Hz, 1H), 2.87 (dt, J = 6.0, 6.0 Hz, 2H), 2.78 (dd, J = 15.0, 2.4 Hz, 1H), 2.38 (s, 3H), 1.85 (d, J = 13.8 Hz, 1H), 1.43 - 1.35 (m, 1H), 1.34 - 1.29 (m, 1H), 1.26 (dd, J = 13.8, 10.8 Hz, 1H), 1.18 - 1.15 (m, 2H), 0.90 (s, 3H), 0.81 (s, 3H). **^{13}C NMR** (150 MHz, $CDCl_3$) δ 166.5, 149.1, 143.4, 140.7, 137.1, 136.1, 133.0, 129.7, 129.1, 127.1, 126.8, 124.2, 122.1, 121.6, 54.3, 45.6, 44.7, 44.0, 39.6, 32.8, 27.10, 27.08, 24.3, 21.6. **IR** ν_{max} (film): 3235, 3199, 2950, 2914, 2855, 1718, 1599, 1499, 1467, 1400, 1327, 1321, 1209, 1157, 1096, 1066, 1017, 900, 814, 792, 758, 706, 656, 576, 551, 540, 514 cm^{-1}. **HRMS** (ESI) m/z calcd for $C_{26}H_{31}N_3NaO_3S$ [M+Na]$^+$: 488.1978; found: 488.1967.

Lagre-scale synthesis of 5a:

To a dry Schlenk flask were added **1a** (1.06 g, 5.0 mmol, 1.0 equiv), **2a** (3.41 g, 12.5 mmol, 2.5 equiv), $Cu(TFA)_2{\cdot}H_2O$ (145 mg, 0.50 mmol, 0.10 equiv), and anhydrous dioxane (75 mL). The mixture was degassed for three times with argon and stirred at 90 °C (oil bath) for 3 h. Once completion, the reaction was cooled to room temperature. The reaction mixture was filtered by celite, and the filtrate was concentrated *in vacuo*. Further purification by a flash column

chromatography using eluents (PE/EA = 5:1) afforded the desired product **5a** as yellow oil (1.51 g, 3.24 mmol, 65%).

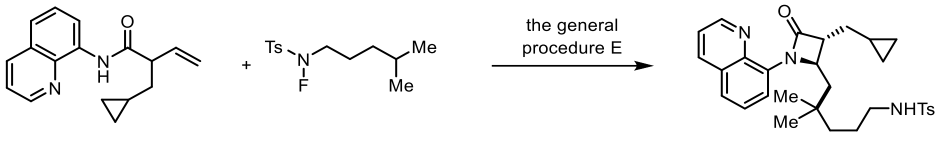

1o **2a** **5b**

Product **5b** was prepared by the general procedure E. Purification using column chromatography (PE/EA = 5:1) afforded **5b** as yellow oil (88.3 mg, 0.17 mmol, 85%, *d.r.* > 20:1). **^{1}H NMR** (600 MHz, $CDCl_3$) δ 8.79 (dd, *J* = 4.2, 1.2 Hz, 1H), 8.27 (dd, *J* = 7.8, 1.2 Hz, 1H), 8.12 (dd, *J* = 8.4, 1.8 Hz, 1H), 7.72 (d, *J* = 7.8 Hz, 2H), 7.57 (d, *J* = 8.4 Hz, 1H), 7.52 - 7.49 (m, 1H), 7.39 (dd, *J* = 8.4, 4.2 Hz, 1H), 7.27 (d, *J* = 7.8 Hz, 2H), 5.09 (dt, *J* = 10.8, 2.4 Hz, 1H), 4.50 (t, *J* = 6.6 Hz, 1H), 3.05 - 3.03 (m, 1H), 2.87 (dt, *J* = 6.6, 6.6 Hz, 2H), 2.40 (s, 3H), 1.92 - 1.84 (m, 2H), 1.74 - 1.69 (m, 1H), 1.43 - 1.38 (m, 1H), 1.35 - 1.30 (m, 2H), 1.18 - 1.15 (m, 2H), 1.05 - 0.99 (m, 1H), 0.93 (s, 3H), 0.85 (s, 3H), 0.55 - 0.49 (m, 2H), 0.18 - 0.12 (m, 2H). **^{13}C NMR** (150 MHz, $CDCl_3$) δ 169.8, 149.0, 143.5, 140.9, 137.1, 136.1, 133.1, 129.8, 129.1, 127.2, 126.9, 124.0, 122.3, 121.5, 60.0, 58.2, 44.8, 44.1, 39.8, 34.2, 32.7, 27.4, 27.3, 24.4, 21.6, 8.8, 5.3, 5.0. **IR** νmax (film): 3267, 2958, 1718, 1599, 1500, 1469, 1362, 1325, 1305, 1150, 788, 549, 514 cm^{-1}. **HRMS** (ESI) m/z calcd for $C_{30}H_{38}N_3O_3S$ $[M+H]^+$: 520.2628; found: 520.2626.

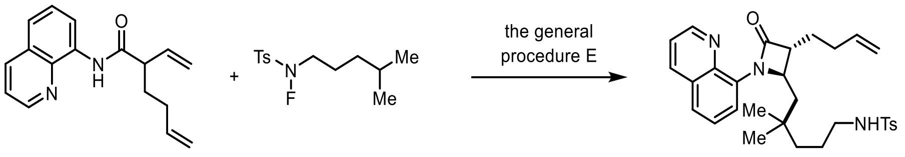

1r **2a** **5c**

Product **5c** was prepared by the general procedure E. Purification using column chromatography (PE/EA = 5:1) afforded **5c** as yellow oil (78.9 mg, 0.15 mmol, 76%, *d.r.* > 20:1). **^{1}H NMR** (600 MHz, $CDCl_3$) δ 8.79 (dd, *J* = 4.2, 1.8 Hz, 1H), 8.25 (dd, *J* = 7.8, 1.2 Hz, 1H), 8.11 (dd, *J* = 8.4, 1.8 Hz, 1H), 7.72 (d, *J* = 8.4 Hz, 2H), 7.56 (d, *J* = 8.4 Hz, 1H), 7.50 - 7.48 (m, 1H), 7.39 (dd, *J* = 8.4, 4.2 Hz, 1H), 7.26 (d, *J* = 8.4 Hz, 2H), 5.86 (ddt, *J* = 16.8, 10.2, 6.6 Hz, 1H), 5.09 (dt, *J* = 16.8, 1.8 Hz, 1H), 5.00 (d, *J* = 10.2 Hz, 1H), 4.97 (t, *J* = 10.2 Hz, 1H), 4.83 (t, *J* = 6.0 Hz, 1H), 2.99 - 2.96 (m, 1H), 2.86 (dt, *J* = 6.0, 6.0 Hz, 2H), 2.49 - 2.43 (m, 1H), 2.39 (s, 3H), 2.33 - 2.26 (m, 1H), 2.01 - 1.97 (m, 2H), 1.83 (d, *J* = 13.8 Hz, 1H), 1.43 - 1.37 (m, 1H), 1.34 - 1.25 (m, 2H), 1.18 - 1.14 (m, 2H), 0.91 (s, 3H), 0.83 (s, 3H). **^{13}C NMR** (150 MHz, $CDCl_3$) δ 169.5, 149.0,

143.4, 140.8, 138.0, 137.1, 136.1, 132.9, 129.7, 129.1, 127.1, 126.8, 124.1, 122.2, 121.5, 115.3, 60.3, 57.2, 44.7, 44.0, 39.7, 32.6, 31.0, 28.5, 27.3, 27.2, 24.3, 21.6. **IR** ν_{max} (film): 3261, 2923, 1718, 1596, 1502, 1472, 1400, 1364, 1325, 1305, 1154, 1094, 909, 825, 812, 788, 756, 730, 706, 659, 574, 549, 525 cm^{-1}. **HRMS** (ESI) m/z calcd for $C_{30}H_{38}N_3O_3S$ $[M+H]^+$: 520.2628; found: 520.2634.

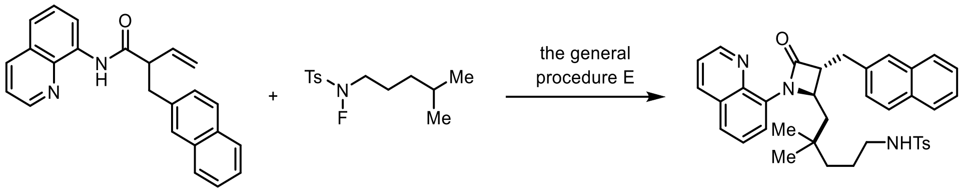

1i **2a** **5d**

Product **5d** was prepared by the general procedure E. Purification using column chromatography (PE/EA = 5:1) afforded **5d** as yellow oil (93.2 mg, 0.15 mmol, 77%, *d.r.* > 20:1). **^{1}H NMR** (600 MHz, $CDCl_3$) δ 8.70 (dd, J = 4.2, 1.8 Hz, 1H), 8.22 (d, J = 7.8 Hz, 1H), 8.04 (d, J = 7.8 Hz, 1H), 7.79 - 7.77 (m, 2H), 7.74 (d, J = 8.4 Hz, 2H), 7.70 (d, J = 8.4 Hz, 2H), 7.53 (d, J = 7.8 Hz, 1H), 7.47 - 7.45 (m, 2H), 7.42 - 7.38 (m, 2H), 7.32 (dd, J = 8.4, 4.2 Hz, 1H), 7.22 (d, J = 8.4 Hz, 2H), 5.07 (dt, J = 10.8, 2.4 Hz, 1H), 4.75 (t, J = 6.0 Hz, 1H), 3.41 (dd, J = 13.8, 7.2 Hz, 1H), 3.32 (dd, J = 13.8, 6.6 Hz, 1H), 3.27 - 3.25 (m, 1H), 2.69 (dt, J = 6.0, 6.0 Hz, 2H), 2.36 (s, 3H), 1.78 (d, J = 13.8 Hz, 1H), 1.27 (dd, J = 13.8, 10.8 Hz, 1H), 1.21 - 1.11 (m, 2H), 0.93 - 0.89 (m, 2H), 0.71 (s, 3H), 0.66 (s, 3H). **^{13}C NMR** (150 MHz, $CDCl_3$) δ 169.0, 149.0, 143.3, 140.8, 137.1, 136.4, 136.0, 133.6, 132.8, 132.3, 129.7, 129.0, 128.3, 127.82, 127.78, 127.7, 127.6, 127.1, 126.7, 126.0, 125.5, 124.3, 122.4, 121.5, 60.4, 58.9, 44.8, 43.8, 39.1, 35.7, 32.4, 27.2, 27.0, 24.2, 21.5. **IR** ν_{max} (film): 2959, 2927, 2859, 1718, 1594, 1502, 1470, 1400, 1349, 1321, 1305, 1152, 1092, 950, 900, 812, 788, 728, 706, 658, 643, 574, 548, 518, 477 cm^{-1}. **HRMS** (ESI) m/z calcd for $C_{37}H_{40}N_3O_3S$ $[M+H]^+$: 606.2785; found: 606.2792.

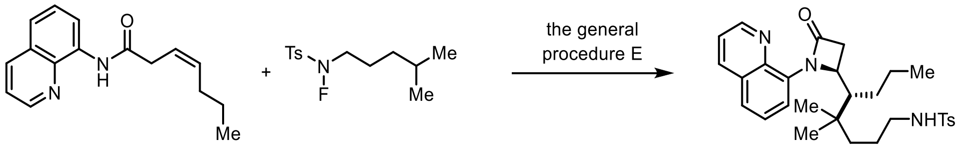

1ab **2a** **5e**

Product **5e** was prepared by the general procedure E. Purification using column chromatography (PE/EA = 5:1) afforded **5e** as yellow oil (63.9 mg, 0.13 mmol, 63%, *d.r.* > 20:1), the diastereochemistry of compound **5e** was determinated according to the comparison to that in chen's paper[20]. **^{1}H NMR** (600 MHz, $CDCl_3$) δ 8.81 (dd, J = 4.2, 1.8 Hz, 1H), 8.14 - 8.12 (m, 2H),

7.76 (d, J = 8.4 Hz, 2H), 7.59 (dd, J = 8.4, 1.2 Hz, 1H), 7.52 - 7.49 (m, 1H), 7.41 (dd, J = 8.4, 4.2 Hz, 1H), 7.26 (d, J = 8.4 Hz, 2H), 5.46 (dt, J = 5.4, 2.4 Hz, 1H), 4.93 (t, J = 6.0 Hz, 1H), 3.07 (dd, J = 15.0, 5.4 Hz, 1H), 2.98 - 2.89 (m, 2H), 2.86 (dd, J = 15.0, 3.0 Hz, 1H), 2.38 (s, 3H), 1.68 - 1.66 (m, 1H), 1.55 - 1.48 (m, 1H), 1.35 - 1.28 (m, 2H), 1.26 - 1.23 (m, 2H), 1.19 - 1.13 (m, 1H), 0.94 - 0.88 (m, 1H), 0.84 (s, 3H), 0.79 (s, 3H), 0.65 - 0.60 (m, 1H), 0.58 (t, J = 6.6 Hz, 3H). **^{13}C NMR** (150 MHz, $CDCl_3$) δ 167.0, 149.2, 143.5, 141.2, 137.1, 136.2, 133.2, 129.8, 129.0, 127.2, 126.8, 124.4, 122.6, 121.6, 57.0, 45.2, 44.2, 40.1, 37.7, 36.3, 25.7, 25.6, 24.9, 24.5, 24.1, 21.6, 14.8. **IR** ν_{max} (film): 3275, 2958, 2923, 2870, 1745, 1588, 1504, 1469, 1396, 1362, 1349, 1318, 1306, 1290, 1154, 1094, 1070, 938, 885, 818, 788, 747, 661, 635, 583, 570, 549, 494, 480 cm^{-1}. **HRMS** (ESI) m/z calcd for $C_{29}H_{38}N_3O_3S$ [M+H]$^+$: 508.2628; found: 508.2628.

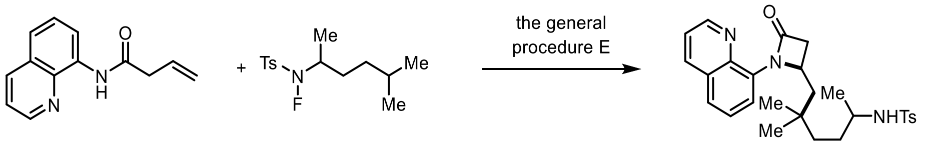

1a **2i** **5f**

Product **5f** was prepared by the general procedure E. Purification using column chromatography (PE/EA = 5:1) afforded **5f** as yellow oil (79.6 mg, 0.17 mmol, 83%, *d.r.* = 1:1). **^{1}H NMR** (600 MHz, $CDCl_3$) δ 8.84 (dd, J = 4.2, 1.8 Hz, 0.5H), 8.81 (dd, J = 4.2, 1.8 Hz, 0.5H), 8.22 (d, J = 7.8 Hz, 1H), 8.12 (dd, J = 8.4, 1.8 Hz, 1H), 7.74 (dd, J = 8.4, 4.2 Hz, 2H), 7.58 (d, J = 7.8 Hz, 1H), 7.52 - 7.49 (m, 1H), 7.42 - 7.39 (m, 1H), 7.24 - 7.20 (m, 2H), 5.24 - 5.19 (m, 1H), 4.86 - 4.84 (m, 1H), 3.34 - 3.29 (m, 1H), 3.25 - 3.19 (m, 1H), 2.79 - 2.75 (m, 1H), 2.36 (s, 1.5H), 2.35 (s, 1.5H), 1.84 - 1.79 (m, 1H), 1.29 - 1.15 (m, 4H), 1.09 - 1.02 (m, 1H), 0.97 (d, J = 2.4 Hz, 1.5H), 0.96 (d, J = 2.4 Hz, 1.5H), 0.88 (s, 1.5H), 0.87 (s, 1.5H), 0.80 (s, 1.5H), 0.79 (s, 1.5H). **^{13}C NMR** (150 MHz, $CDCl_3$) δ 166.5, 149.1, 149.0, 143.20, 143.18, 140.7, 138.5, 138.4, 136.1, 133.1, 129.6, 129.0, 127.0, 126.8, 126.7, 124.14, 124.12, 122.1, 121.51, 121.49, 54.3, 50.78, 50.77, 45.6, 44.9, 38.5, 32.7, 31.9, 31.8, 27.0, 26.90, 26.88, 21.84, 21.79, 21.50, 21.49. **IR** ν_{max} (film): 3258, 2918, 2855, 1739, 1599, 1499, 1467, 1426, 1398, 1344, 1319, 1305, 1200, 1154, 1129, 1094, 1083, 978, 900, 829, 816, 792, 764, 667, 659, 577, 555, 544, 533, 477 cm^{-1}. **HRMS** (ESI) m/z calcd for $C_{27}H_{34}N_3O_3S$ [M+H]$^+$: 480.2315; found: 480.2322.

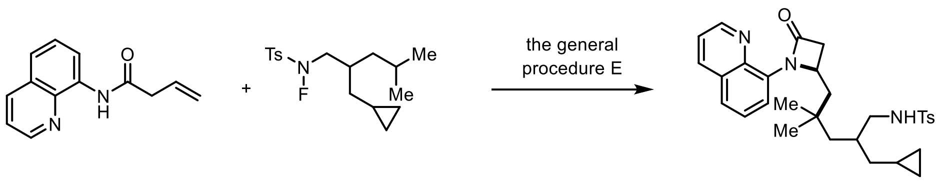

1a **2y** **5g**

Product **5g** was prepared by the general procedure E. Purification using column chromatography (PE/EA = 5:1) afforded **5g** as yellow oil (78.9 mg, 0.15 mmol, 76%, *d.r.* = 1:1). **^{1}H NMR** (600 MHz, $CDCl_3$) δ 8.80 - 8.79 (m, 1H), 8.26 - 8.23 (m, 1H), 8.13 - 8.11 (m, 1H), 7.69 (d, J = 7.2 Hz, 2H), 7.59 - 7.57 (m, 1H), 7.52 - 7.49 (m, 1H), 7.41 - 7.39 (m, 1H), 7.24 - 7.22 (m, 2H), 5.27 - 5.22 (m, 1H), 4.95 (t, J = 6.0 Hz, 0.5H), 4.89 (t, J = 6.0 Hz, 0.5H), 3.35 - 3.31 (m, 1H), 2.93 - 2.89 (m, 1H), 2.86 - 2.76 (m, 2H), 2.39 (s, 1.5H), 2.37 (s, 1.5H), 1.91 - 1.84 (m, 1H), 1.61 - 1.56 (m, 1H), 1.29 - 1.25 (m, 1H), 1.21 - 1.17 (m, 1H), 1.15 - 1.07 (m, 3H), 0.96 (s, 1.5H), 0.94 (s, 1.5H), 0.86 (s, 3H), 0.51 - 0.45 (m, 1H), 0.35 - 0.23 (m, 2H), -0.08 - (-0.19) (m, 2H). **^{13}C NMR** (150 MHz, $CDCl_3$) δ 166.50, 166.48, 149.0, 148.9, 143.4, 143.3, 140.73, 140.65, 137.2, 137.1, 136.2, 136.1, 133.1, 129.7, 129.08, 129.06, 127.2, 127.1, 126.8, 124.1, 122.03, 122.01, 121.53, 121.49, 54.23, 54.20, 48.1, 48.0, 46.1, 45.91, 45.89, 45.83, 44.7, 44.6, 39.08, 39.06, 34.6, 33.90, 33.89, 27.1, 26.83, 26.77, 26.73, 21.6, 21.5, 8.6, 8.5, 5.0, 4.9, 4.8, 4.7. **IR** ν_{max} (film): 3267, 2918, 1718, 1594, 1567, 1502, 1472, 1398, 1362, 1342, 1323, 1305, 1200, 1154, 1092, 1068, 950, 909, 825, 814, 786, 730, 706, 659, 570, 548, 514 cm^{-1}. **HRMS** (ESI) m/z calcd for $C_{30}H_{38}N_3O_3S$ $[M+H]^+$: 520.2628; found: 520.2635.

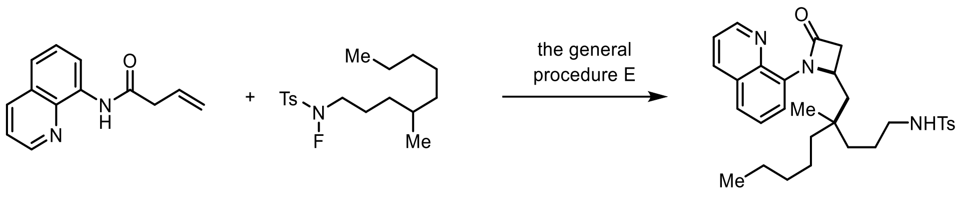

1a **2z** **5h**

Product **5h** was prepared by the general procedure E. Purification using column chromatography (PE/EA = 5:1) afforded **5h** as yellow oil (82.4 mg, 0.16 mmol, 79%, *d.r.* = 1:1). **^{1}H NMR** (600 MHz, $CDCl_3$) δ 8.81 (dd, J = 4.2, 1.8 Hz, 0.5H), 8.80 (dd, J = 4.2, 1.8 Hz, 0.5H), 8.19 - 8.17 (m, 1H), 8.13 - 8.11 (m, 1H), 7.75 (d, J = 7.8 Hz, 1H), 7.73 (d, J = 7.8 Hz, 1H), 7.59 - 7.58 (m, 1H), 7.51 - 7.48 (m, 1H), 7.42 - 7.39 (m, 1H), 7.27 - 7.24 (m, 2H), 5.22 - 5.19 (m, 1H), 5.01 (t, J = 6.0 Hz, 0.5H), 4.98 (t, J = 6.0 Hz, 0.5H), 3.32 - 3.28 (m, 1H), 2.92 - 2.83 (m, 2H), 2.81 - 2.77 (m, 1H), 2.38 (s, 1.5H), 2.37 (s, 1.5H), 1.81 - 1.79 (m, 1H), 1.38 - 1.34 (m, 1H), 1.31 - 1.16 (m, 8H), 1.14 - 1.09 (m, 2H), 1.08 - 1.05 (m, 2H), 0.89 - 0.80 (m, 6H). **^{13}C NMR** (150 MHz,

CDCl3) δ 166.6, 149.2, 149.1, 143.36, 143.35, 141.0, 140.9, 137.1, 136.18, 136.15, 132.89, 132.88, 129.73, 129.72, 129.1, 127.1, 126.73, 126.72, 124.41, 124.35, 122.6, 122.5, 121.6, 121.5, 54.20, 54.18, 45.3, 45.2, 44.1, 44.0, 42.4, 42.3, 39.8, 39.5, 36.8, 36.5, 35.2, 32.8, 32.7, 25.02, 24.95, 23.9, 23.8, 23.19, 23.17, 22.76, 22.69, 21.54, 21.51, 14.3, 14.2. **IR** ν_{max} (film): 3267, 2927, 2864, 1718, 1594, 1562, 1502, 1472, 1398, 1362, 1325, 1305, 1200, 1152, 1092, 1073, 955, 900, 825, 814, 788, 756, 728, 706, 659, 572, 548, 514 cm^{-1}. **HRMS** (ESI) m/z calcd for $C_{30}H_{40}N_3O_3S$ $[M+H]^+$: 522.2785; found: 522.2794.

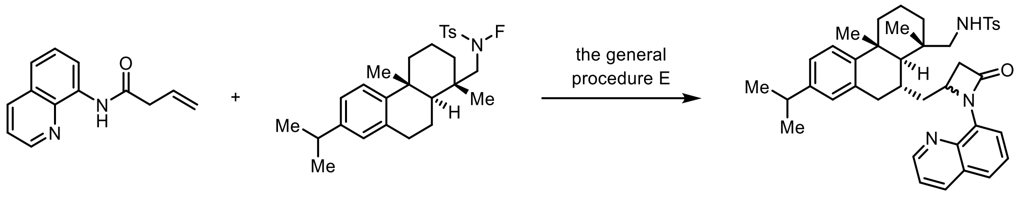

1a **2ay** **5k**

Product **5k** was prepared by the general procedure E. Purification using column chromatography (PE/EA = 5:1) afforded **5k** as yellow oil (97.4 mg, 0.15 mmol, 75%, *d.r.* = 3:1). **^{1}H NMR** (600 MHz, CDCl3) δ 8.98 (dd, *J* = 4.2, 1.8 Hz, 0.25H), 8.91 (dd, *J* = 4.2, 1.8 Hz, 0.75H), 8.13 (dd, *J* = 7.8, 1.8 Hz, 0.75H), 8.10 (d, *J* = 7.8, 1.8 Hz, 0.25H), 8.08 (dd, *J* = 7.8, 1.8 Hz, 0.25H), 7.77 (dd, *J* = 7.8, 1.8 Hz, 0.75H), 7.66 - 7.54 (m, 3H), 7.49 - 7.46 (m, 1H), 7.43 - 7.39 (m, 1H), 7.33 (d, *J* = 7.8 Hz, 0.5H), 7.27 (d, *J* = 7.8 Hz, 1.5H), 7.00 - 6.92 (m, 2H), 6.81 (s, 0.75H), 6.76 (s, 0.25H), 5.44 - 5.40 (m, 0.75H), 5.38 - 5.36 (m, 0.25H), 4.49 - 4.47 (m, 0.25H), 4.14 - 4.12 (m, 0.75H), 3.40 - 3.36 (m, 1H), 3.14 - 3.10 (m, 0.25H), 2.94 - 2.90 (m, 0.25H), 2.84 - 2.82 (m, 0.75H), 2.80 - 2.78 (m, 0.75H), 2.74 - 2.61 (m, 2H), 2.45 (s, 3H), 2.43 - 2.36 (m, 2H), 2.04 - 1.99 (m, 1H), 1.78 - 1.73 (m, 1H), 1.69 - 1.66 (m, 1H), 1.59 - 1.56 (m, 2H), 1.43 - 1.31 (m, 3H), 1.23 - 1.21 (m, 6H), 1.13 - 1.11 (m, 2H), 1.06 - 1.00 (m, 1H), 0.98 (s, 2H), 0.82 - 0.77 (m, 1H), 0.40 (s, 2H). **^{13}C NMR** (150 MHz, CDCl3) δ 166.3, 166.1, 149.4, 147.3, 147.2, 146.1, 145.7, 143.9, 143.7, 141.7, 141.3, 137.1, 136.9, 136.4, 136.3, 135.2, 135.1, 133.7, 133.1, 130.2, 130.0, 129.2, 129.1, 127.5, 127.1, 127.0, 126.9, 126.8, 126.7, 125.0, 124.5, 124.3, 124.1, 123.5, 122.6, 121.8, 121.7, 121.6, 121.5, 55.7, 55.3, 55.22, 55.16, 53.3, 52.8, 44.6, 43.4, 43.2, 41.5, 39.5, 38.8, 38.6, 38.44, 38.36, 37.03, 36.98, 33.81, 33.76, 33.7, 33.1, 31.2, 31.1, 24.4, 24.3, 24.1, 24.0, 23.1, 23.0, 21.7, 18.14, 18.10, 17.6, 17.4. **IR** ν_{max} (film): 2954, 2923, 1723, 1589, 1499, 1457, 1396, 1360, 1327, 1200, 1155, 1094, 1066, 964, 907, 824, 812, 788, 728, 706, 659, 646, 566, 551 cm^{-1}. **HRMS** (ESI) m/z calcd for $C_{40}H_{48}N_3O_3S$ $[M+H]^+$: 650.3411; found: 650.3418.

The procedure for the synthesis of product 5i - 5j:

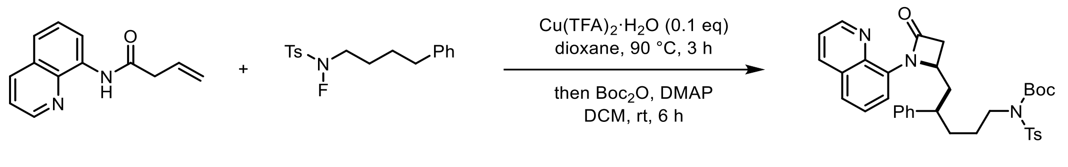

1a **2al** **5i**

To a dry Schlenk flask were added **1a** (42.4 mg, 0.20 mmol, 1.0 equiv), **2al** (161 mg, 0.50 mmol, 2.5 equiv), $Cu(TFA)_2 \cdot H_2O$ (5.8 mg, 0.02 mmol, 0.10 equiv), and anhydrous dioxane (3.0 mL). The mixture was degassed for three times with argon and stirred at 90 °C (oil bath) for 3 h. Once completion, the reaction was cooled to room temperature. The reaction mixture was filtered by celite, and the filtrate was concentrated *in vacuo*. The residue was dissolved in DCM (2.0 mL), and then Boc_2O (55 uL, 0.24 mmol, 1.2 equiv) and DMAP (2.4 mg, 0.02 mmol, 0.10 equiv) were added. After being stirred at room temperature for 6 h, the reaction mixture was filtered by celite, and the filtrate was concentrated *in vacuo*. Further purification by a flash column chromatography using eluents (PE/EA = 10:1) afforded the desired product **5i** as yellow oil (101 mg, 0.16 mmol, 82%, *d.r.* = 1.5:1). **^{1}H NMR** (600 MHz, $CDCl_3$) δ 8.89 (dd, J = 4.2, 1.8 Hz, 0.4H), 8.64 (dd, J = 4.2, 1.8 Hz, 0.6H), 8.27 (dd, J = 7.8, 1.8 Hz, 0.6H), 8.17 (d, J = 7.8, 1.8 Hz, 0.4H), 8.13 (dd, J = 8.4, 1.8 Hz, 0.4H), 8.10 (dd, J = 8.4, 1.8 Hz, 0.6H), 7.67 (d, J = 8.4 Hz, 2H), 7.57 (dd, J = 7.8, 1.2 Hz, 0.4H), 7.55 (dd, J = 7.8, 1.2 Hz, 0.6H), 7.50 - 7.46 (m, 1H), 7.43 - 7.41 (m, 0.4H), 7.39 - 7.37 (m, 1.6H), 7.29 - 7.27 (m, 1H), 7.25 - 7.18 (m, 4H), 7.11 - 7.09 (m, 1H), 5.19 - 5.16 (m, 0.4H), 4.89 - 4.86 (m, 0.6H), 3.77 - 3.69 (m, 2H), 3.23 (dd, J = 15.0, 5.4 Hz, 0.6H), 2.97 (dd, J = 15.0, 5.4 Hz, 0.4H), 2.84 (dd, J = 15.0, 2.4 Hz, 0.6H), 2.73 - 2.63 (m, 1H), 2.48 - 2.42 (m, 1H), 2.41 (s, 3H), 2.36 (dd, J = 15.0, 2.4 Hz, 0.4H), 1.75 - 1.73 (m, 1H), 1.65 - 1.51 (m, 4H), 1.26 (s, 5.4H), 1.25 (s, 3.6H). **^{13}C NMR** (150 MHz, $CDCl_3$) δ 166.6, 166.5, 151.0, 149.3, 148.8, 144.5, 144.1, 143.9, 140.8, 140.4, 137.5, 136.1, 136.0, 133.7, 133.5, 129.3, 129.1, 129.0, 128.7, 128.6, 127.89, 127.86, 127.83, 127.6, 126.81, 126.76, 126.7, 126.6, 124.1, 123.8, 121.8, 121.5, 121.4, 121.3, 84.20, 84.15, 56.1, 55.2, 47.1, 47.0, 43.9, 43.4, 43.0, 41.2, 40.6, 34.4, 33.5, 28.1, 28.0, 27.94, 27.93, 21.7. **IR** ν_{max} (film): 2916, 1724, 1594, 1567, 1499, 1472, 1396, 1342, 1286, 1254, 1152, 1103, 1086, 982, 950, 914, 846, 814, 788, 758, 723, 700, 669, 659, 646, 596, 574, 544 cm^{-1}. **HRMS** (ESI) m/z calcd for $C_{35}H_{40}N_3O_5S$ $[M+H]^+$: 614.2683; found: 614.2675.

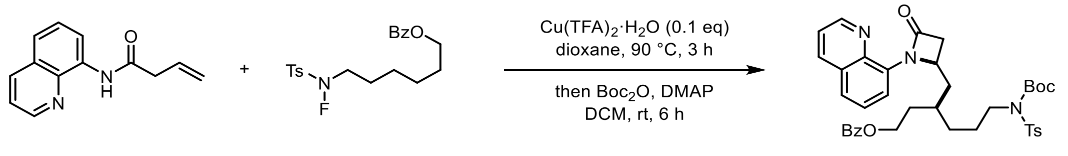

1a **2ar** **5j**

To a dry Schlenk flask were added **1a** (42.4 mg, 0.20 mmol, 1.0 equiv), **2ar** (197 mg, 0.50 mmol, 2.5 equiv), $Cu(TFA)_2 \cdot H_2O$ (5.8 mg, 0.02 mmol, 0.10 equiv), and anhydrous dioxane (3.0 mL). The mixture was degassed for three times with argon and stirred at 90 °C (oil bath) for 3 h. Once completion, the reaction was cooled to room temperature. The reaction mixture was filtered by celite, and the filtrate was concentrated *in vacuo*. The residue was dissolved in DCM (2.0 mL), and then Boc_2O (55 uL, 0.24 mmol, 1.2 equiv) and DMAP (2.4 mg, 0.02 mmol, 0.10 equiv) were added. After being stirred at room temperature for 6 h, the reaction was filtered by celite, and the filtrate was concentrated *in vacuo*. Further purification by a flash column chromatography using eluents (PE/EA = 10:1) afforded the desired product **5j** as yellow oil (86.3 mg, 0.13 mmol, 63%, *d.r.* = 2:1). **^{1}H NMR** (600 MHz, $CDCl_3$) δ 8.90 (dd, J = 4.2, 1.8 Hz, 0.33H), 8.87 (dd, J = 4.2, 1.8 Hz, 0.67H), 8.29 - 8.27 (m, 1H), 8.12 - 8.09 (m, 1H), 8.02 - 7.99 (m, 2H), 7.77 (d, J = 8.4 Hz, 0.66H), 7.74 (d, J = 8.4 Hz, 1.34H), 7.59 - 7.56 (m, 1H), 7.56 - 7.54 (m, 1H), 7.52 - 7.48 (m, 1H), 7.46 - 7.41 (m, 2H), 7.38 (dd, J = 8.4, 4.2 Hz, 1H), 7.29 - 7.27 (m, 2H), 5.33 - 5.29 (m, 1H), 4.46 - 4.36 (m, 1H), 4.35 - 4.29 (m, 1H), 3.87 - 3.83 (m, 0.66H), 3.79 - 3.77 (m, 1.34H), 3.37 (dd, J = 15.0, 5.4 Hz, 1H), 2.86 - 2.82 (m, 1H), 2.43 (s, 1H), 2.42 (s, 2H), 2.12 - 2.08 (m, 1H), 1.93 - 1.89 (m, 1H), 1.86 - 1.82 (m, 1H), 1.77 - 1.69 (m, 3H), 1.56 - 1.51 (m, 1H), 1.47 - 1.38 (m, 2H), 1.32 (s, 3H), 1.30 (s, 6H). **^{13}C NMR** (150 MHz, $CDCl_3$) δ 166.7, 166.6, 166.41, 166.38, 151.13, 151.07, 149.4, 149.3, 144.3, 144.2, 140.6, 137.7, 137.6, 136.2, 136.1, 133.6, 133.5, 133.1, 130.4, 130.3, 129.7, 129.6, 129.39, 129.38, 129.13, 129.12, 128.6, 128.5, 127.9, 127.8, 126.83, 126.81, 124.08, 124.05, 121.71, 121.66, 121.6, 84.4, 63.0, 62.9, 55.3, 55.1, 47.5, 47.3, 44.1, 43.8, 38.4, 37.9, 33.1, 32.7, 32.3, 31.5, 30.4, 29.8, 28.03, 28.01, 27.5, 27.0, 21.7. **IR** ν_{max} (film): 2918, 2855, 1735, 1713, 1599, 1503, 1471, 1399, 1342, 1271, 1152, 1107, 1086, 1070, 1025, 946, 825, 814, 788, 768, 756, 710, 669, 659, 596, 574, 544 cm^{-1}. **HRMS** (ESI) m/z calcd for $C_{38}H_{44}N_3O_7S$ $[M+H]^+$: 686.2894; found: 686.2901.

Part 3.3. Procedure and characteristic data for radical clock experiments.

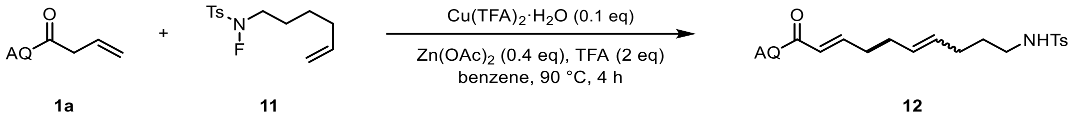

To a dry Schlenk flask were added **1a** (42.4 mg, 0.20 mmol, 1.0 equiv), **11** (136 mg, 0.50 mmol, 2.5 equiv), $Cu(TFA)_2 \cdot H_2O$ (5.8 mg, 0.02 mmol, 0.10 equiv), $Zn(OAc)_2$ (14.7 mg, 0.08 mmol, 0.40

equiv), TFA (30 uL, 0.40 mmol, 2.0 equiv), and anhydrous benzene (2.0 mL). The mixture was degassed for three times with argon and stirred at 90 °C (oil bath) for 4 h. Once completion, the reaction was cooled to room temperature. The reaction mixture was filtered by celite, and the filtrate was concentrated *in vacuo*. Further purification by a flash column chromatography using eluents (PE/EA = 5:1) afforded the desired product **12** as yellow oil (52.8 mg, 0.11 mmol, 57%, *r.r.* > 20:1, *E/Z* = 2:1). **^{1}H NMR** (500 MHz, $CDCl_3$) δ 9.85 (s, 1H), 8.86 - 8.81 (m, 2H), 8.19 (d, *J* = 8.0 Hz, 1H), 7.76 (d, *J* = 8.0 Hz, 0.66H), 7.73 (d, *J* = 8.0 Hz, 1.34H), 7.58 - 7.52 (m, 2H), 7.48 (dd, *J* = 8.0, 4.0 Hz, 1H), 7.28 (d, *J* = 8.0 Hz, 2H), 7.04 - 6.96 (m, 1H), 6.20 - 6.16 (m, 1H), 5.41 - 5.36 (m, 2H), 5.09 - 5.07 (m, 0.33H), 4.48 - 4.47 (m, 0.67H), 2.94 (dt, *J* = 7.0, 7.0 Hz, 2H), 2.41 (s, 3H), 2.33 (dt, *J* = 7.0, 7.0 Hz, 2H), 2.25 - 2.19 (m, 2H), 2.09 (dt, *J* = 7.0, 7.0 Hz, 0.66H), 2.00 (dt, *J* = 7.0, 7.0 Hz, 1.34H), 1.57 - 1.52 (m, 2H). **^{13}C NMR** (150 MHz, $CDCl_3$) δ 164.5, 164.4, 148.3, 145.9, 145.5, 143.4, 143.3, 137.3, 137.2, 136.72, 136.66, 134.71, 134.65, 130.19, 130.16, 130.06, 130.03, 130.0, 129.80, 129.75, 129.1, 128.2, 127.7, 127.6, 127.28, 127.25, 125.2, 121.81, 121.79, 121.77, 121.70, 117.1, 117.0, 43.0, 42.8, 32.1, 31.3, 29.6, 29.4, 27.4, 26.2, 25.7, 24.5, 22.8, 21.7. **IR** ν_{max} (film): 2923, 2851, 1683, 1653, 1646, 1636, 1596, 1526, 1486, 1457, 1424, 1385, 1326, 1238, 1158, 1093, 970, 826, 814, 792, 730, 660, 606, 568, 550 cm^{-1}. **HRMS** (ESI) m/z calcd for $C_{26}H_{29}N_3NaO_3S$ $[M+Na]^+$: 486.1822; found: 486.1812.

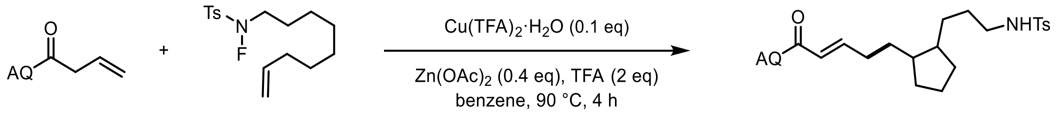

1a **13** **14**

To a dry Schlenk flask were added **1a** (42.4 mg, 0.20 mmol, 1.0 equiv), **13** (157 mg, 0.50 mmol, 2.5 equiv), $Cu(TFA)_2{\cdot}H_2O$ (5.8 mg, 0.02 mmol, 0.10 equiv), $Zn(OAc)_2$ (14.7 mg, 0.08 mmol, 0.40 equiv), TFA (30 uL, 0.40 mmol, 2.0 equiv), and anhydrous benzene (2.0 mL). The mixture was degassed for three times with argon and stirred at 90 °C (oil bath) for 4 h. Once completion, the reaction was cooled to room temperature. The reaction mixture was filtered by celite, and the filtrate was concentrated *in vacuo*. Further purification by a flash column chromatography using eluents (PE/EA = 5:1) afforded the desired product **14** as yellow oil (63.7 mg, 0.13 mmol, 63%, *r.r.* > 20:1, *E/Z* > 20:1, *d.r.* = 2:1). **^{1}H NMR** (500 MHz, $CDCl_3$) δ 9.85 (s, 1H), 8.86 - 8.80 (m, 2H), 8.17 (dd, *J* = 8.5, 2.0 Hz, 1H), 7.77 - 7.74 (m, 2H), 7.57 - 7.50 (m, 2H), 7.46 (dd, *J* = 8.5, 4.5 Hz, 1H), 7.30 (d, *J* = 8.0 Hz, 2H), 7.07 - 7.01 (m, 1H), 6.19 - 6.16 (m, 1H), 4.63 (t, *J* = 6.0 Hz, 0.66H), 4.59 (t, *J* = 6.0 Hz, 0.34H), 2.97 - 2.92 (m, 2H), 2.41 (s, 3H), 2.35 - 2.29 (m, 1H), 2.23 - 2.15 (m, 1H), 1.84 - 1.78 (m, 1H), 1.76 - 1.58 (m, 4H), 1.56 - 1.42 (m, 4H), 1.40 - 1.36 (m, 1H), 1.34 - 1.27 (m, 3H), 1.08 - 1.01 (m, 1H). **^{13}C NMR** (125 MHz, $CDCl_3$) δ 164.5, 148.2, 146.6, 146.5, 143.4, 138.6, 137.2, 136.6, 134.8, 129.8, 128.1, 127.6, 127.3, 124.69, 124.66, 121.8, 121.6, 116.9, 45.6,

45.5, 43.71, 43.69, 42.1, 33.8, 32.21, 32.19, 32.1, 31.2, 30.2, 30.1, 28.8, 28.7, 28.2, 26.4, 23.8, 22.5, 21.6. **IR** ν_{max} (film): 3343, 2929, 2862, 1683, 1637, 1596, 1526, 1486, 1457, 1425, 1385, 1328, 1158, 1093, 972, 912, 827, 792, 732, 660, 606, 550 cm^{-1}. **HRMS** (ESI) m/z calcd for $C_{29}H_{35}N_3NaO_3S$ $[M+Na]^+$: 528.2291; found: 528.2293.

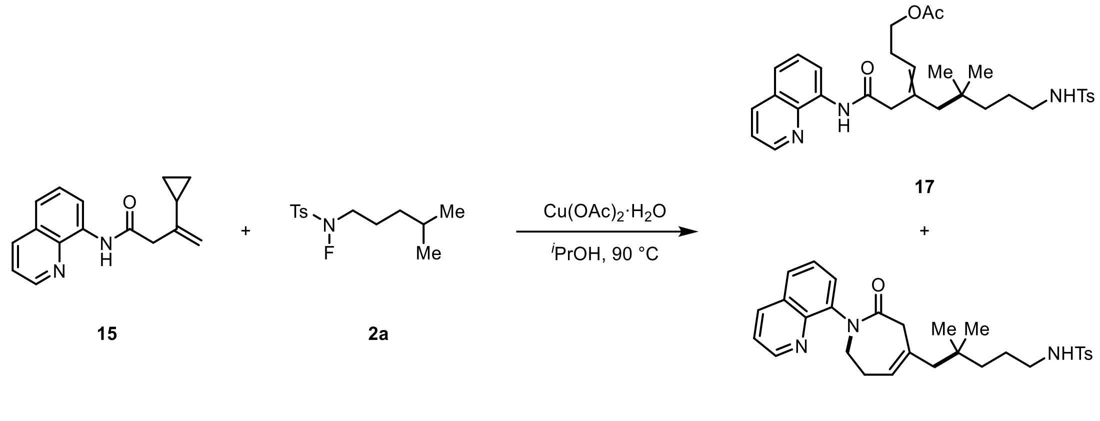

To a dry Schlenk flask were added **15** (50.4 mg, 0.20 mmol, 1.0 equiv), **2a** (137 mg, 0.50 mmol, 2.5 equiv), $Cu(OAc)_2 \cdot H_2O$ (4.0 mg, 0.02 mmol, 0.10 equiv), and anhydrous ^{i}PrOH (3.0 mL). The mixture was degassed for three times with argon and stirred at 90 °C (oil bath) for 2 h. Once completion, the reaction was cooled to room temperature. The reaction mixture was filtered by celite, and the filtrate was concentrated *in vacuo*. Further purification by a flash column chromatography using eluents (PE/EA = 5:1) afforded compound **17** (10.2 mg, 0.02 mmol, 9%, yellow oil) as single stereomer (the exact *E*- or *Z*-isomer has not been confirmed in current stage) and compound **18** (62.6 mg, 0.12 mmol, 62%, yellow oil).

compound **17:** ^{1}**H NMR** (600 MHz, $CDCl_3$) δ 10.21 (s, 1H), 8.83 (dd, *J* = 4.2, 1.8 Hz, 1H), 8.74 (dd, *J* = 7.2, 1.8 Hz, 1H), 8.15 (d, *J* = 7.8 Hz, 1H), 7.76 - 7.74 (m, 2H), 7.55 - 7.49 (m, 2H), 7.46 (dd, *J* = 8.4, 4.2 Hz, 1H), 7.29 (d, *J* = 7.2 Hz, 2H), 5.75 (t, *J* = 7.2 Hz, 1H), 4.96 (brs, 1H), 4.27 (t, *J* = 7.8 Hz, 2H), 3.33 (s, 2H), 2.93 (dt, *J* = 6.0, 6.0 Hz, 2H), 2.54 (dt, *J* = 7.2, 7.2 Hz, 2H), 2.41 (s, 3H), 2.09 (s, 2H), 2.03 (s, 3H), 1.48 - 1.43 (m, 2H), 1.29 -1.25 (m, 2H), 0.89 (s, 6H). ^{13}**C NMR** (150 MHz, $CDCl_3$) δ 171.4, 170.1, 148.6, 143.3, 137.4, 136.5, 135.4, 134.4, 129.9, 129.8, 128.1, 127.5, 127.3, 121.9, 121.8, 116.5, 63.9, 49.7, 44.0, 41.1, 39.9, 35.4, 28.9, 27.9, 24.5, 21.6, 21.1. **IR** ν_{max} (film): 2925, 2854, 2096, 1716, 1698, 1652, 1638, 1647, 1539, 1521, 1507, 1488, 1472, 1456, 1418, 1386, 1363, 1326, 1245, 1158, 1117, 1093, 1068, 1034, 826, 669, 550 cm^{-1}. **HRMS** (ESI) m/z calcd for $C_{31}H_{39}N_3NaO_5S$ $[M+Na]^+$: 588.2503; found: 588.2506.

compound **18:** ^{1}**H NMR** (600 MHz, Acetone-$CDCl_3$) δ 8.90 (dd, *J* = 4.2, 1.8 Hz, 1H), 8.36 (dd, *J* = 8.4, 1.8 Hz, 1H), 7.91 (dd, *J* = 7.8, 1.8 Hz, 1H), 7.71 (d, *J* = 8.4 Hz, 2H), 7.63 - 7.59 (m, 2H), 7.52 (dd, *J* = 8.4, 4.2 Hz, 1H), 7.35 (d, *J* = 8.4 Hz, 2H), 6.50 (t, *J* = 6.0 Hz, 1H), 5.48 (t, *J* = 3.6 Hz,

1H), 4.07 - 3.99 (m, 2H), 3.48 (s, 2H), 2.89 (dt, *J* = 6.0, 6.0 Hz, 2H), 2.51 (dt, *J* = 3.6, 3.6 Hz, 2H), 2.39 (s, 3H), 2.01 (s, 2H), 1.51 - 1.46 (m, 2H), 1.28 -1.25 (m, 2H), 0.90 (s, 6H). **^{13}C NMR** (150 MHz, Acetone-$CDCl_3$) δ 173.4, 151.2, 145.3, 143.7, 142.0, 139.6, 137.2, 130.8, 130.6, 130.5, 130.3, 128.3, 127.9, 127.6, 127.2, 122.5, 53.9, 50.3, 44.9, 42.8, 39.9, 35.1, 30.3, 27.9, 25.4, 21.5. **IR** ν_{max} (film): 3197, 2956, 1700, 1647, 1614, 1596, 1572, 1496, 1473, 1435, 1411, 1386, 1364, 1328, 1247, 1159, 1094, 1024, 829, 816, 794, 761, 707, 574, 551 cm^{-1}. **HRMS** (ESI) m/z calcd for $C_{29}H_{35}N_3NaO_3S$ $[M+Na]^+$: 528.2291; found: 528.2299.

Part 3.4. Procedur**e** and characteristic data for compound **19** and **20**.

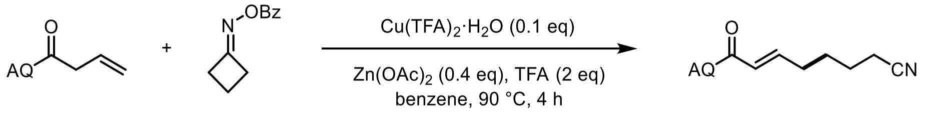

1a **19**

To a dry Schlenk flask were added **1a** (42.4 mg, 0.20 mmol, 1.0 equiv), cyclobutanone O-benzoyl oxime (94.5 mg, 0.50 mmol, 2.5 equiv), $Cu(TFA)_2 \cdot H_2O$ (5.8 mg, 0.02 mmol, 0.10 equiv), $Zn(OAc)_2$ (14.7 mg, 0.08 mmol, 0.40 equiv), TFA (30 uL, 0.40 mmol, 2.0 equiv), and anhydrous benzene (2.0 mL). The mixture was degassed for three times with argon and stirred at 90 °C (oil bath) for 4 h. Once completion, the reaction was cooled to room temperature. The reaction mixture was filtered by celite, and the filtrate was concentrated *in vacuo*. Further purification by a flash column chromatography using eluents (PE/EA = 5:1) afforded the desired compound **19** as yellow oil (40.2 mg, 0.14 mmol, 72%, *r.r.* > 20:1, *E/Z* > 20:1). **^{1}H NMR** (600 MHz, $CDCl_3$) δ 9.86 (s, 1H), 8.84 (dd, *J* = 7.2, 1.2 Hz, 1H), 8.81 (dd, *J* = 4.8, 1.8 Hz, 1H), 8.16 (dd, *J* = 7.8, 1.2 Hz, 1H), 7.57 - 7.50 (m, 2H), 7.46 (dd, *J* = 7.8, 4.2 Hz, 1H), 7.02 (dt, *J* = 15.0, 7.2 Hz, 1H), 6.21 (d, *J* = 15.0 Hz, 1H), 2.39 (t, *J* = 6.6 Hz, 2H), 2.35 (dt, *J* = 7.2, 7.2 Hz, 2H), 1.75 - 1.69 (m, 4H). **^{13}C NMR** (150 MHz, $CDCl_3$) δ 164.0, 148.3, 144.5, 138.6, 136.5, 134.6, 128.1, 127.6, 125.6, 121.8, 121.7, 119.5, 116.9, 31.3, 27.4, 25.0, 17.2. **IR** ν_{max} (film): 3332, 2923, 2243, 1670, 1640, 1598, 1523, 1483, 1459, 1424, 1383, 1321, 1167, 969, 895, 838, 797, 768, 757, 730, 704, 659, 607, 533, 417 cm^{-1}. **HRMS** (ESI) m/z calcd for $C_{17}H_{17}N_3NaO$ $[M+Na]^+$: 302.1264; found: 302.1270.

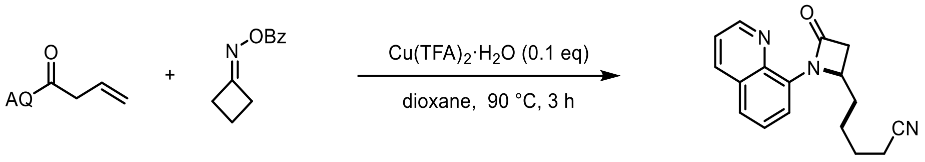

1a **20**

To a dry Schlenk flask were added **1a** (42.4 mg, 0.20 mmol, 1.0 equiv), cyclobutanone O-benzoyl oxime (94.5 mg, 0.50 mmol, 2.5 equiv), $Cu(TFA)_2{\cdot}H_2O$ (5.8 mg, 0.02 mmol, 0.10 equiv), and anhydrous dioxane (3.0 mL). The mixture was degassed for three times with argon and stirred at 90 °C (oil bath) for 3 h. Once completion, the reaction was cooled to room temperature. The reaction mixture was filtered by celite, and the filtrate was concentrated *in vacuo*. Further purification by a flash column chromatography using eluents (PE/EA = 5:1) afforded the desired compound **20** as yellow oil (37.9 mg, 0.14 mmol, 68%). **^{1}H NMR** (600 MHz, $CDCl_3$) δ 8.85 (dd, J = 4.2, 1.8 Hz, 1H), 8.27 (dd, J = 7.8, 1.2 Hz, 1H), 8.14 (dd, J = 7.8, 1.2 Hz, 1H), 7.59 (d, J = 8.4, 1.8 Hz, 1H), 7.53 - 7.51 (m, 1H), 7.42 (dd, J = 8.4, 4.2 Hz, 1H), 5.21 - 5.18 (m, 1H), 3.36 (dd, J = 15.0, 5.4 Hz, 1H), 2.82 (dd, J = 15.0, 2.4 Hz, 1H), 2.30 (t, J = 6.6 Hz, 2H), 2.08 - 2.03 (m, 1H), 1.68 - 1.64 (m, 2H), 1.59 - 1.49 (m, 3H). **^{13}C NMR** (150 MHz, $CDCl_3$) δ 166.3, 149.1, 140.8, 136.3, 133.7, 129.2, 126.9, 124.2, 121.8, 121.5, 119.4, 56.0, 43.3, 33.1, 25.2, 24.4, 17.1. **IR** ν_{max} (film): 2950, 2924, 2854, 1732, 1567, 1456, 1396, 1377, 1339, 1274, 1197, 1151, 1118, 913, 824, 787, 747, 659 cm^{-1}. **HRMS** (ESI) m/z calcd for $C_{17}H_{17}N_3NaO$ [M+Na]$^+$: 302.1264; found: 302.1262.

Part 3.5. NMR spectra.

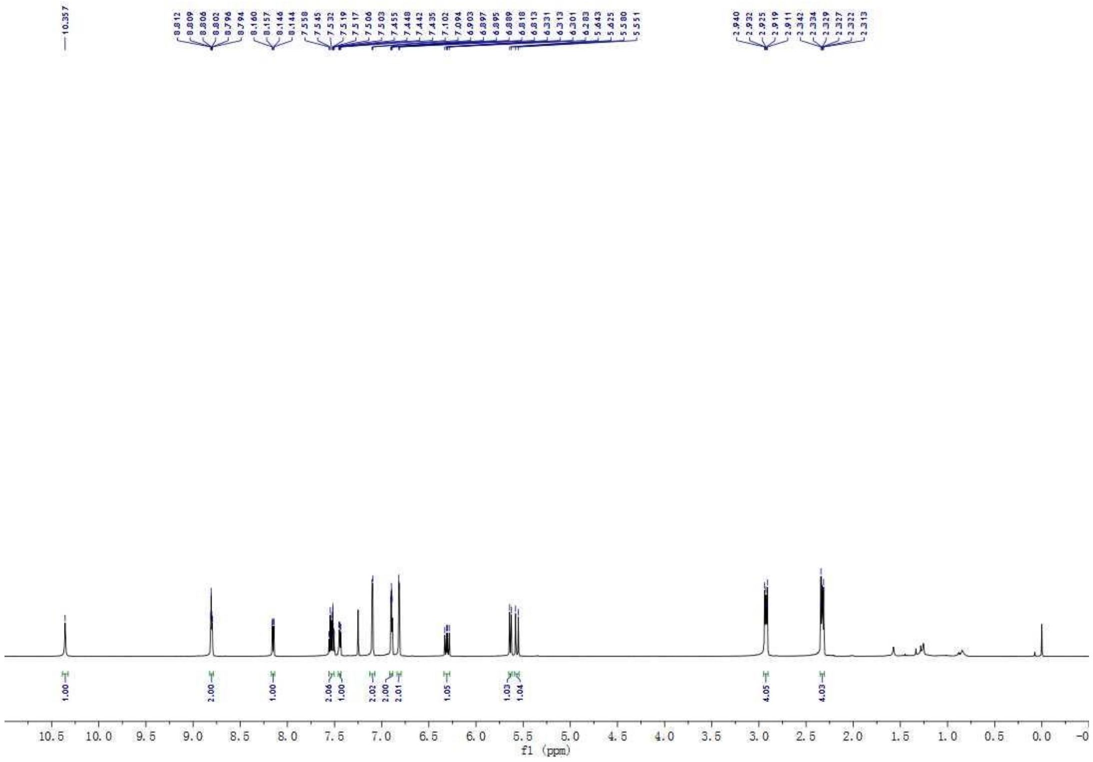

Supplementary Fig. 4. ^{1}H NMR of compound 1w. The sample has been recorded in 600 MHz, $CDCl_3$ at 25 °C.

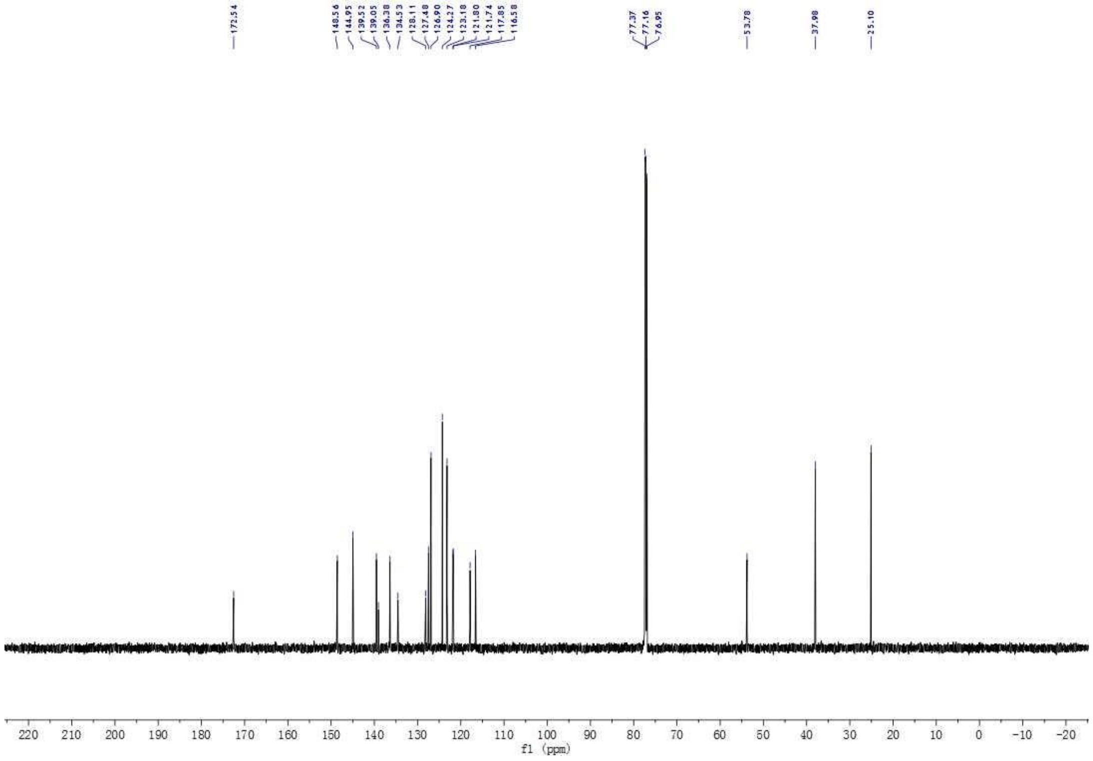

Supplementary Fig. 5. ^{13}C NMR of compound 1w. The sample has been recorded in 150 MHz, $CDCl_3$ at 25 °C.

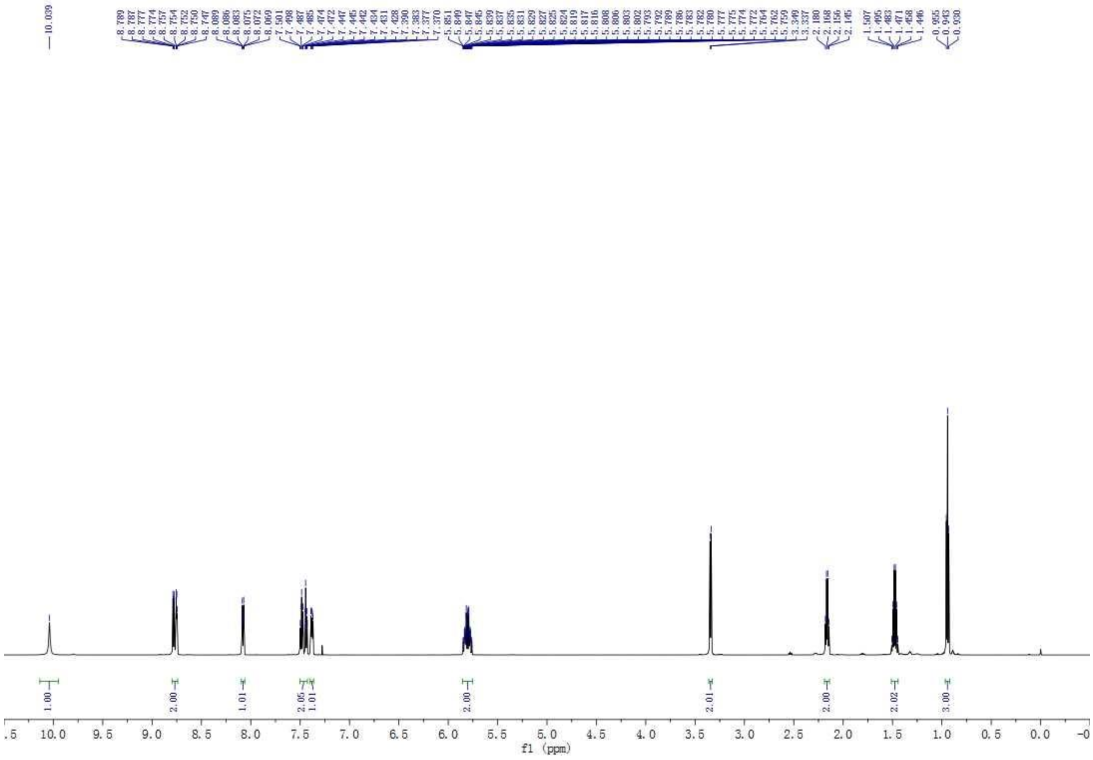

Supplementary Fig. 6. ¹H NMR of compound 1ab. The sample has been recorded in 600 MHz, $CDCl_3$ at 25 °C.

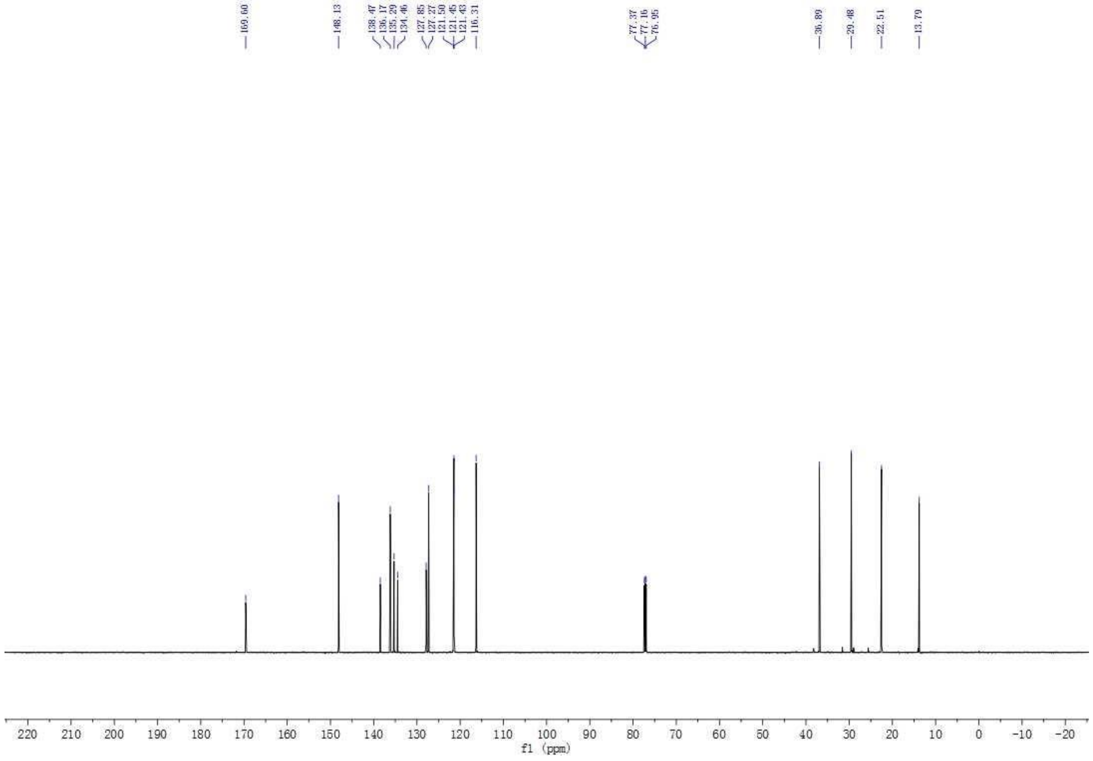

Supplementary Fig. 7. ¹³C NMR of compound 1ab. The sample has been recorded in 150 MHz, $CDCl_3$ at 25 °C.

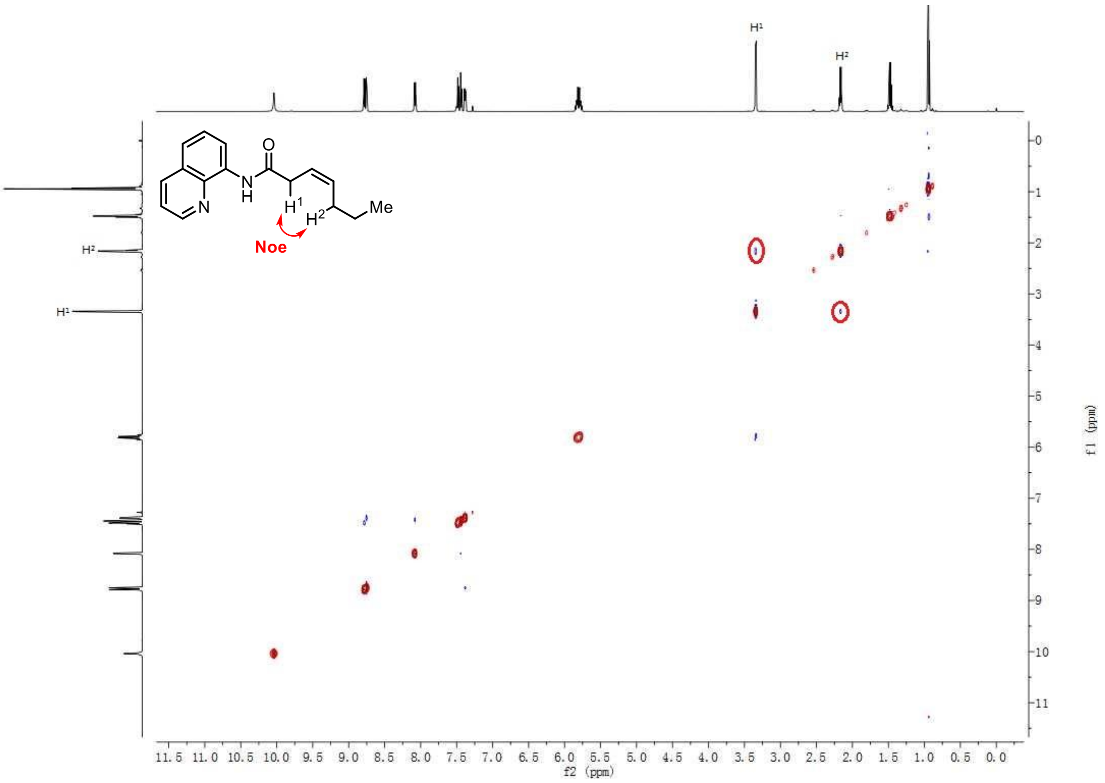

Supplementary Fig. 8. Noesy NMR of compound 1ab. The sample has been recorded in 600 MHz, $CDCl_3$ at 25 °C.

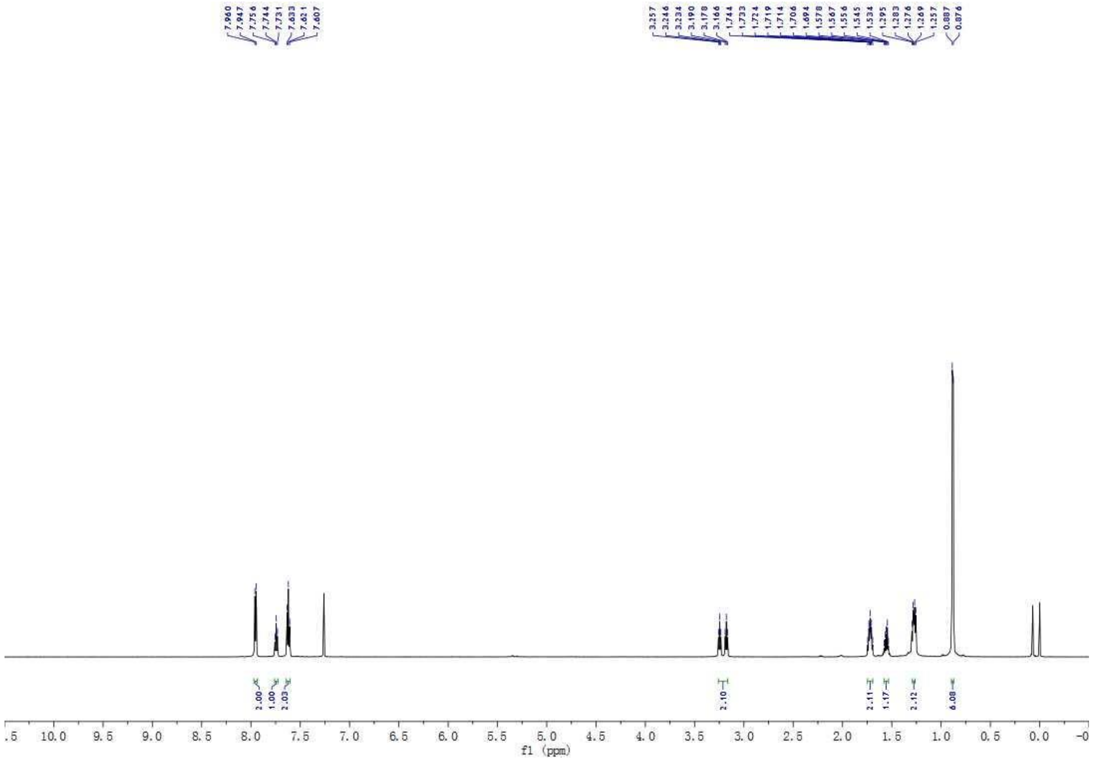

Supplementary Fig. 9. ^{1}H NMR of compound 2b. The sample has been recorded in 600 MHz, $CDCl_3$ at 25 °C.

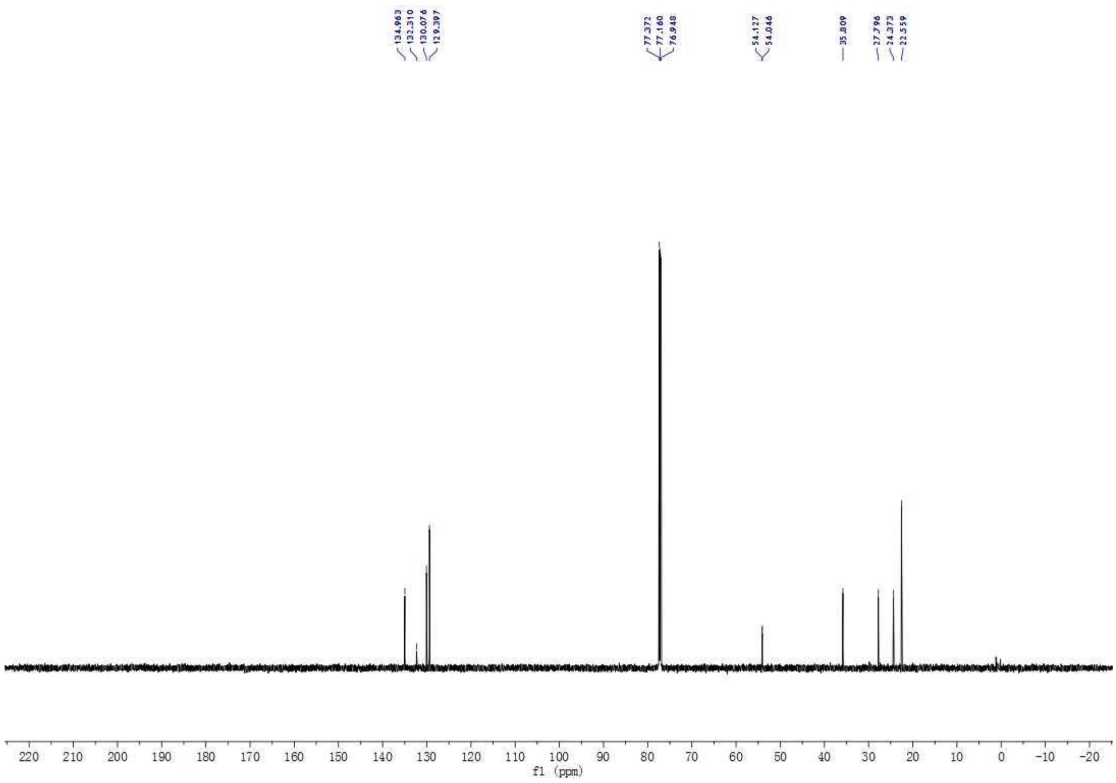

Supplementary Fig. 10. ^{13}C NMR of compound 2b. The sample has been recorded in 150 MHz, $CDCl_3$ at 25 °C.

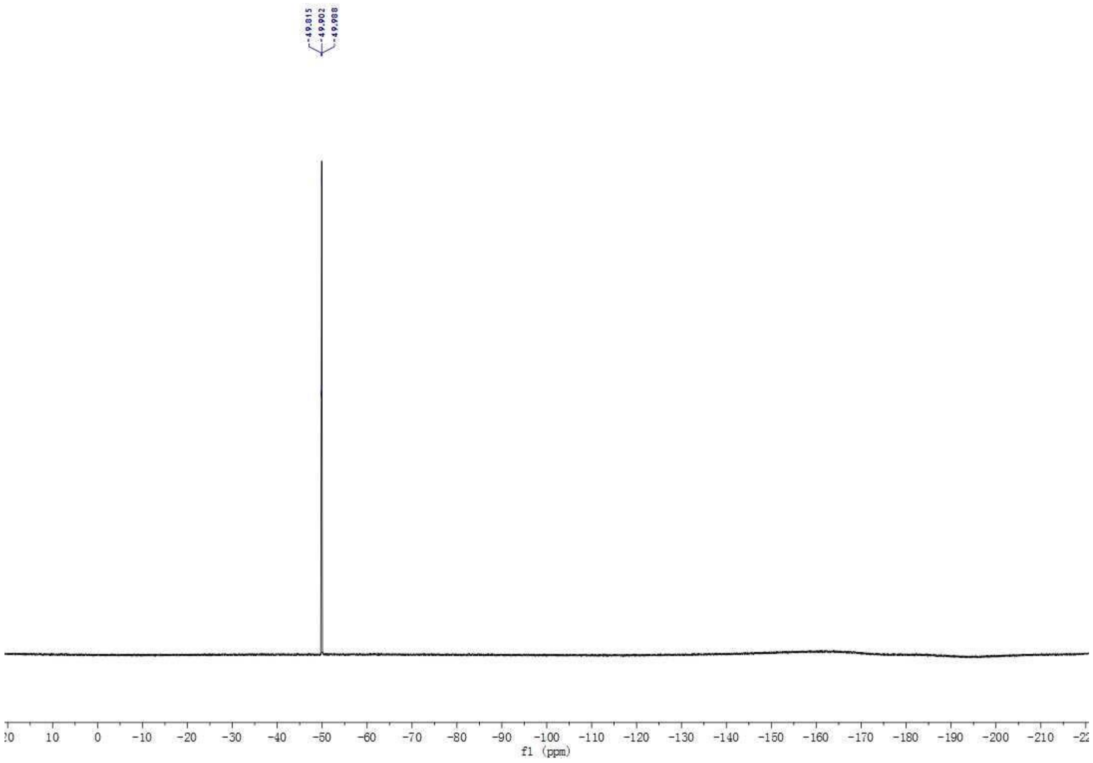

Supplementary Fig. 11. ^{19}F NMR of compound 2b. The sample has been recorded in 470 MHz, $CDCl_3$ at 25 °C.

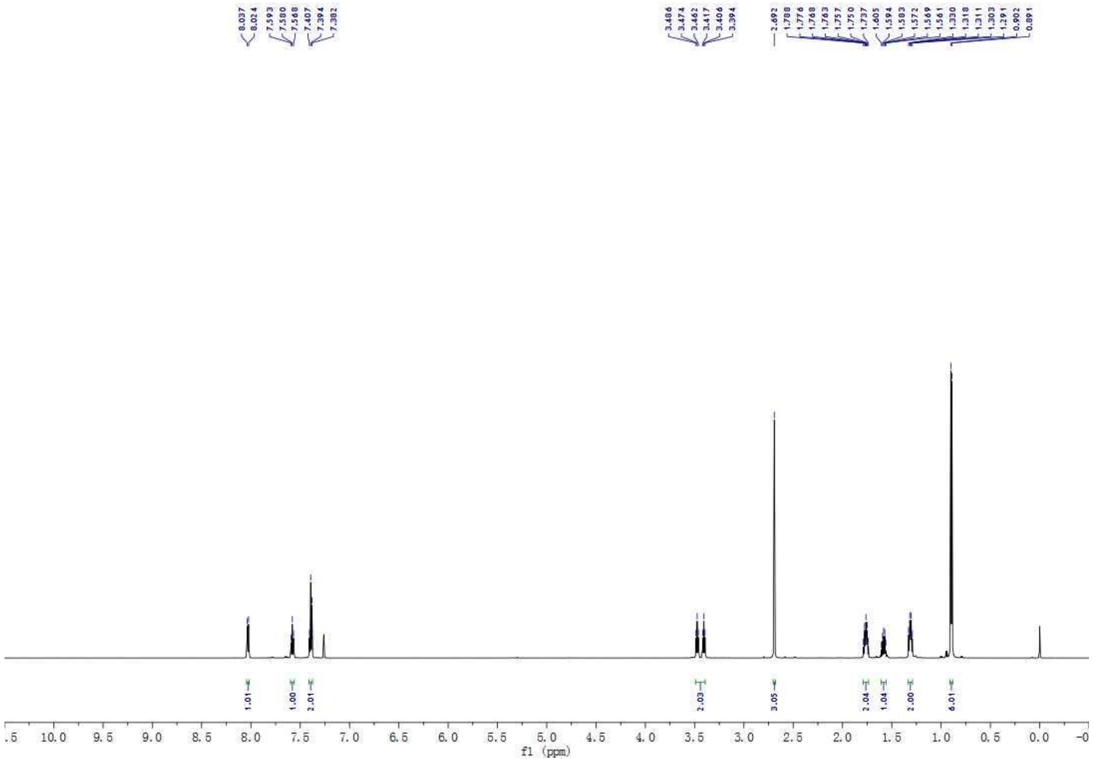

Supplementary Fig. 12. ^{1}H NMR of compound 2c. The sample has been recorded in 600 MHz, $CDCl_3$ at 25 °C.

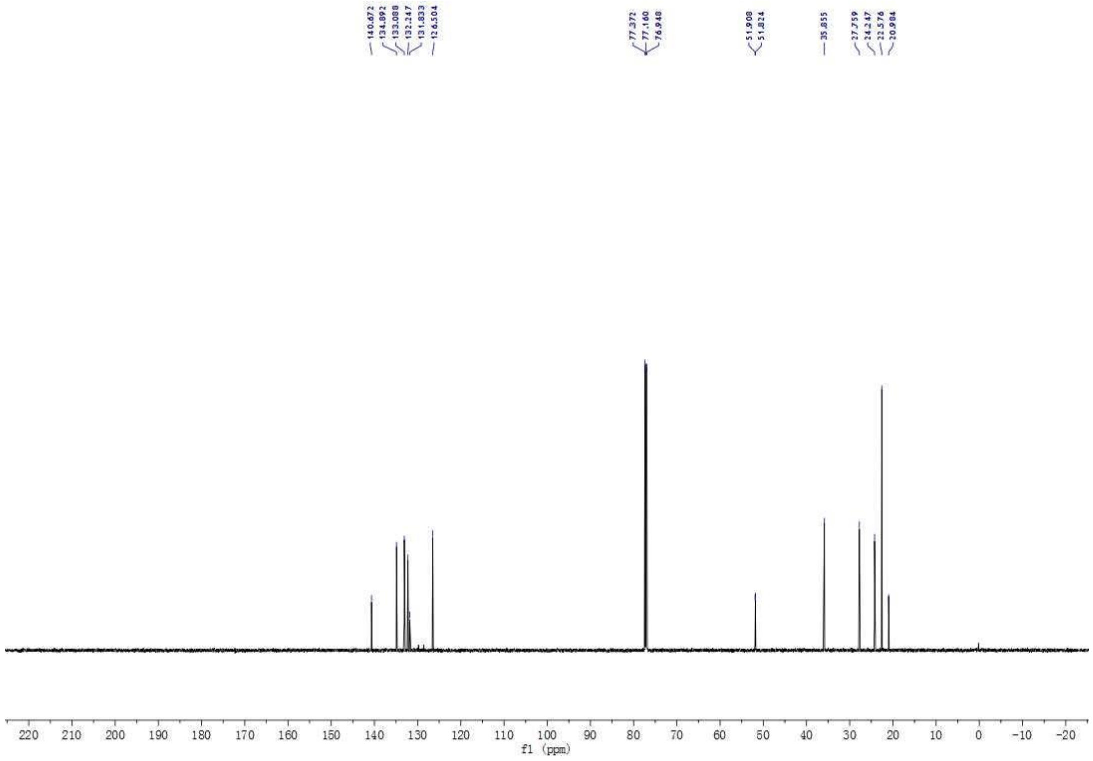

Supplementary Fig. 13. ^{13}C NMR of compound 2c. The sample has been recorded in 150 MHz, $CDCl_3$ at 25 °C.

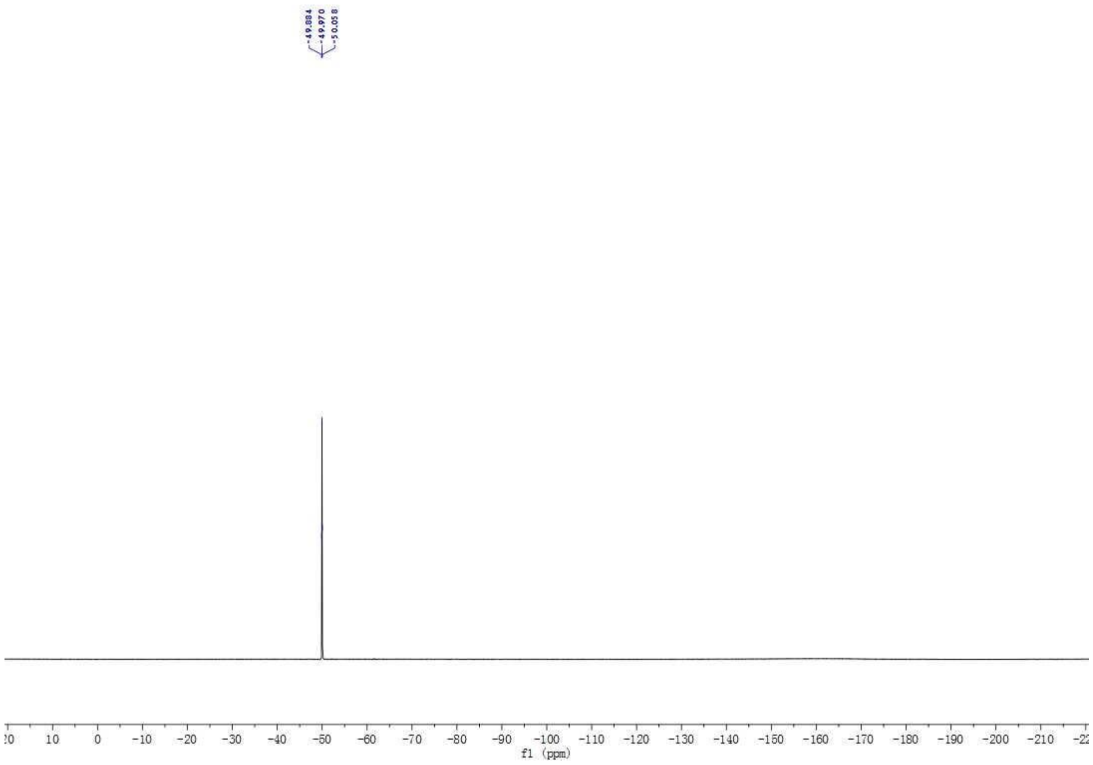

Supplementary Fig. 14. ^{19}F NMR of compound 2c. The sample has been recorded in 470 MHz, $CDCl_3$ at 25 °C.

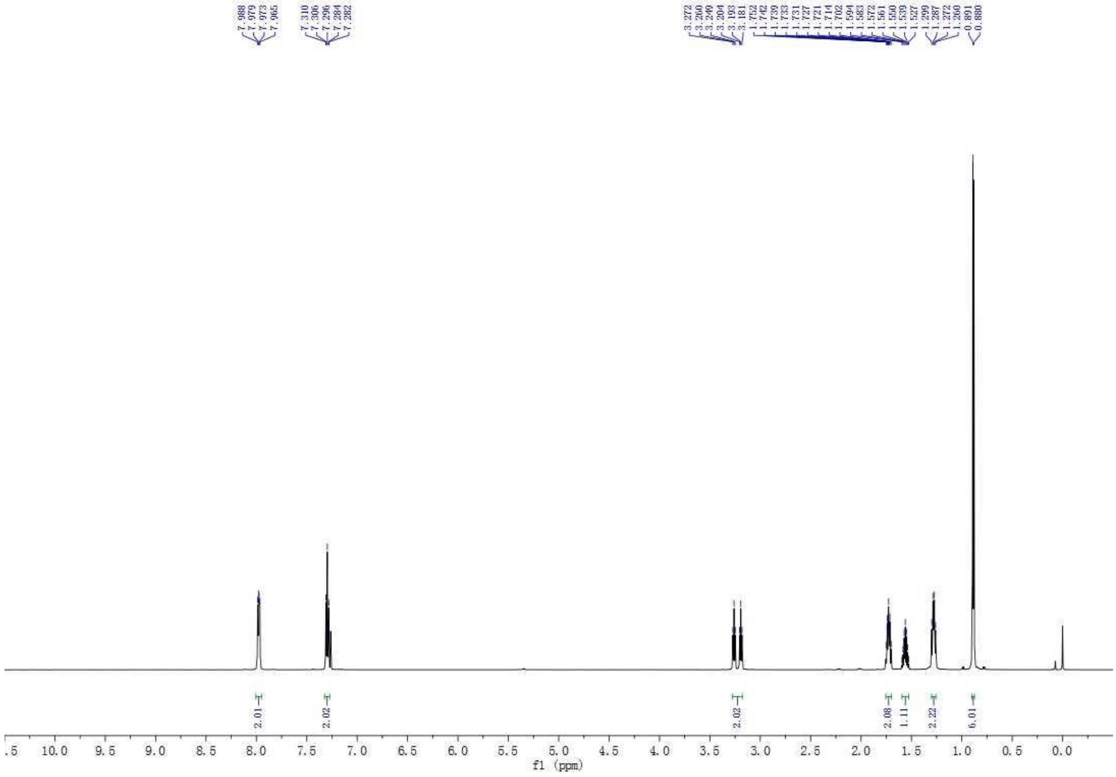

Supplementary Fig. 15. ^{1}H NMR of compound 2e. The sample has been recorded in 600 MHz, $CDCl_3$ at 25 °C.

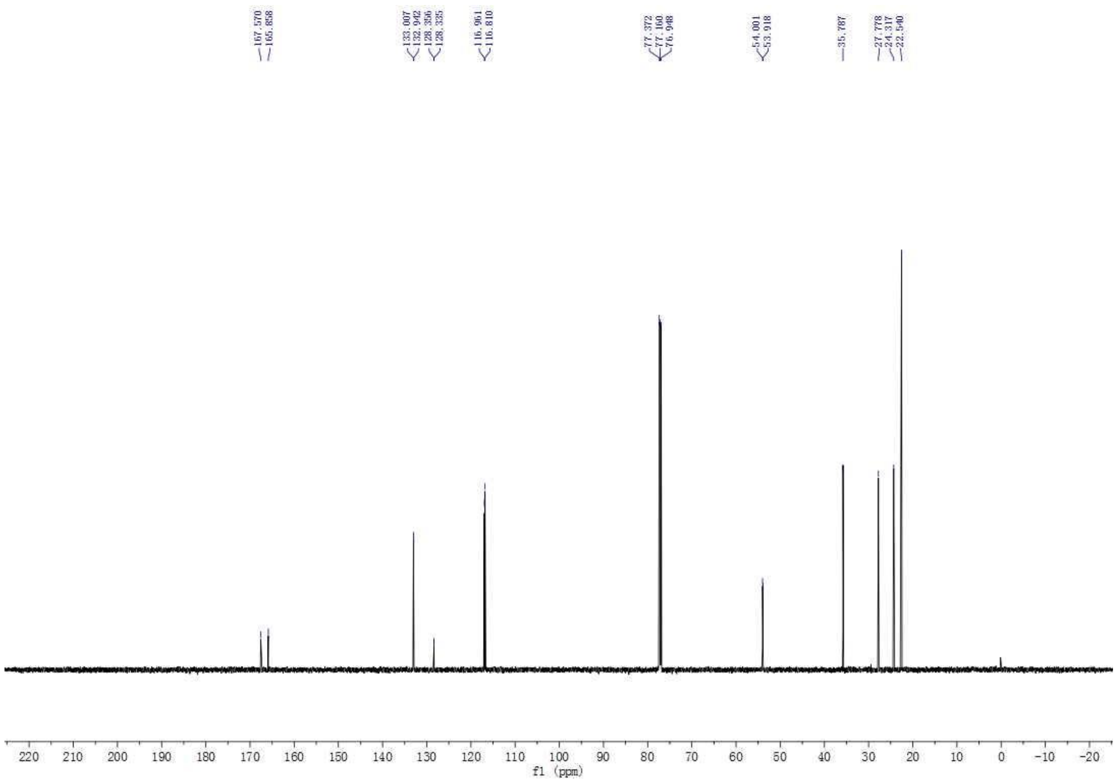

Supplementary Fig. 16. ^{13}C NMR of compound 2e. The sample has been recorded in 150 MHz, $CDCl_3$ at 25 °C.

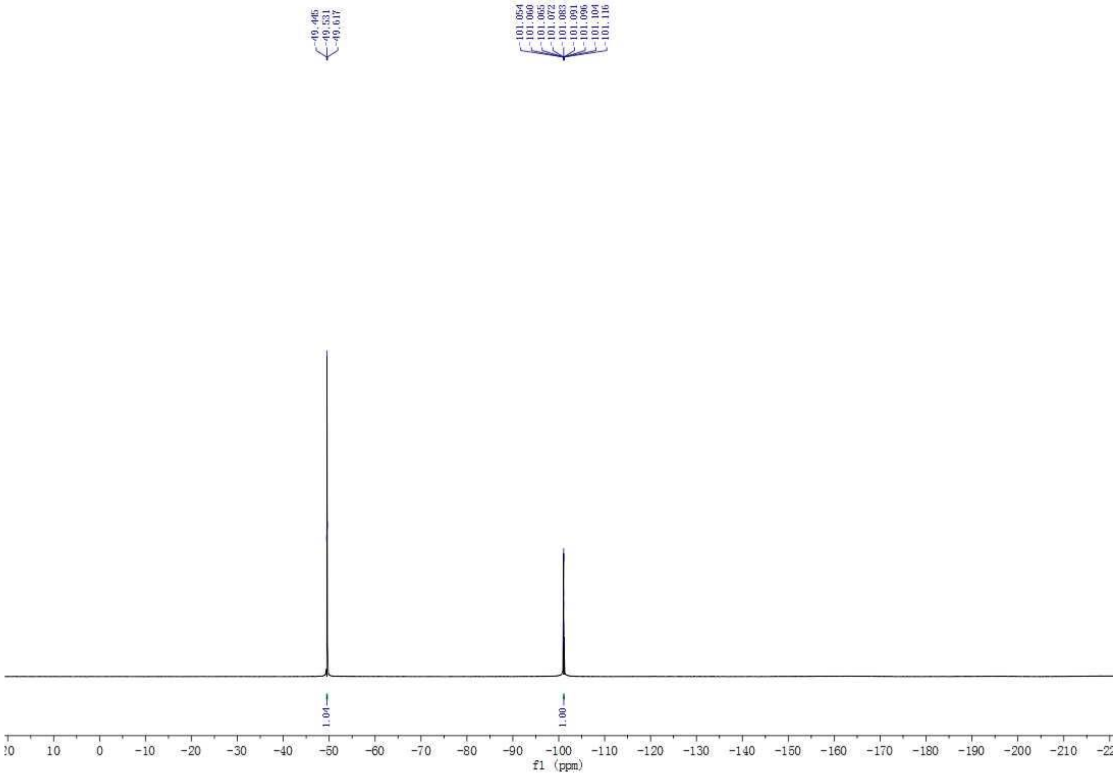

Supplementary Fig. 17. ^{19}F NMR of compound 2e. The sample has been recorded in 470 MHz, $CDCl_3$ at 25 °C.

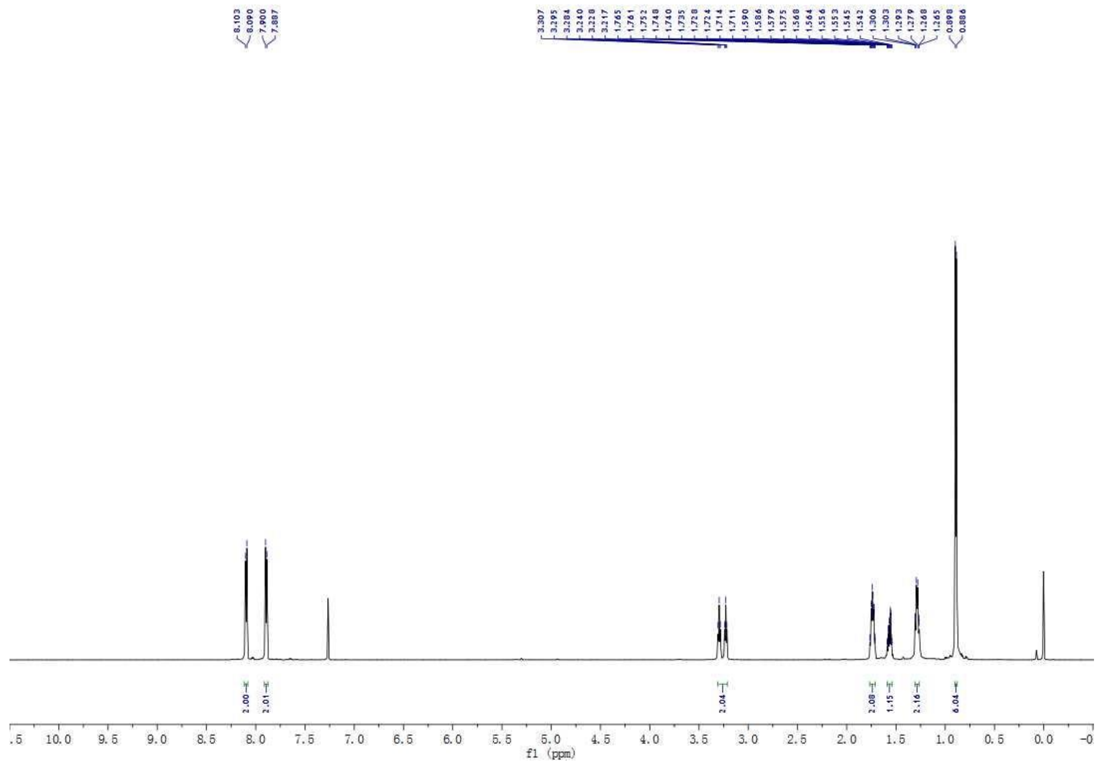

Supplementary Fig. 18. ^{1}H NMR of compound 2f. The sample has been recorded in 600 MHz, $CDCl_3$ at 25 °C.

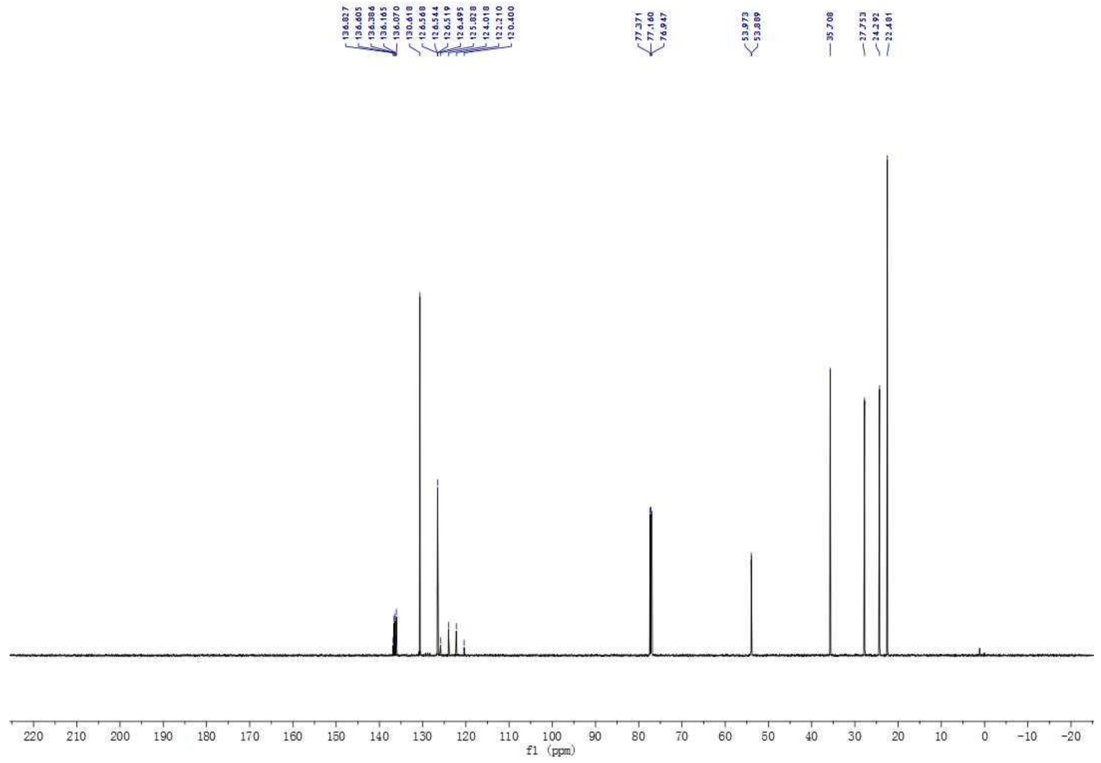

Supplementary Fig. 19. ^{13}C NMR of compound 2f. The sample has been recorded in 150 MHz, $CDCl_3$ at 25 °C.

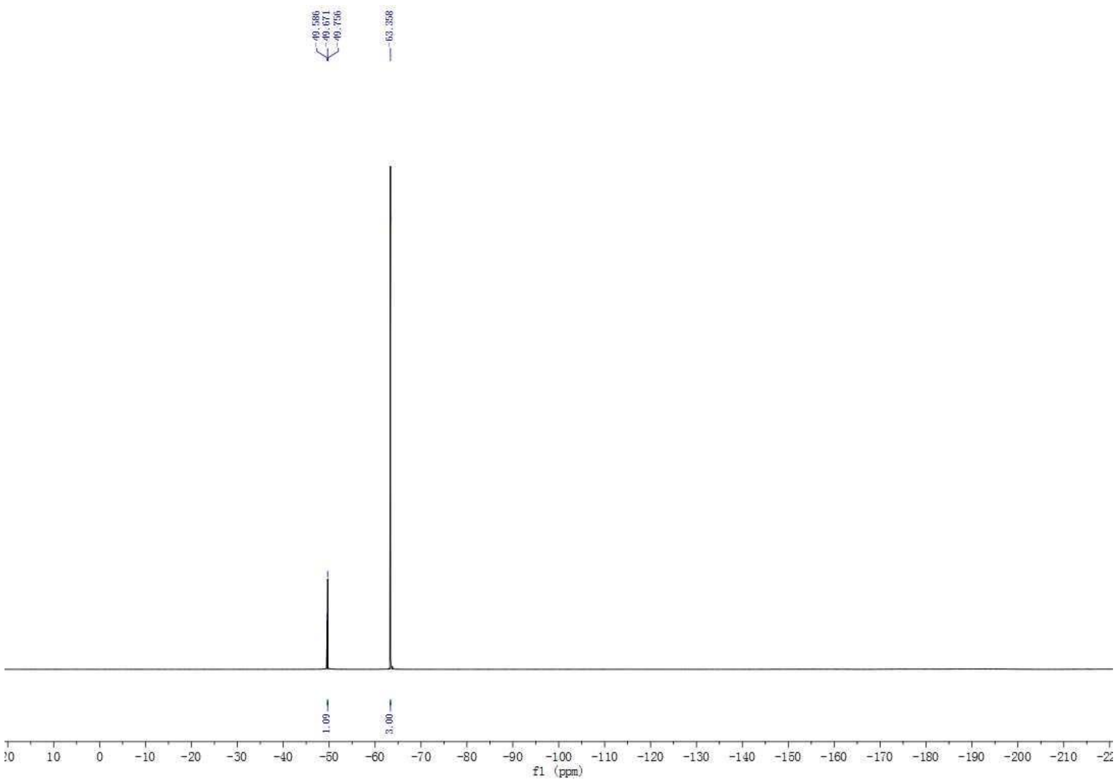

Supplementary Fig. 20. ^{19}F NMR of compound 2f. The sample has been recorded in 470 MHz, $CDCl_3$ at 25 °C.

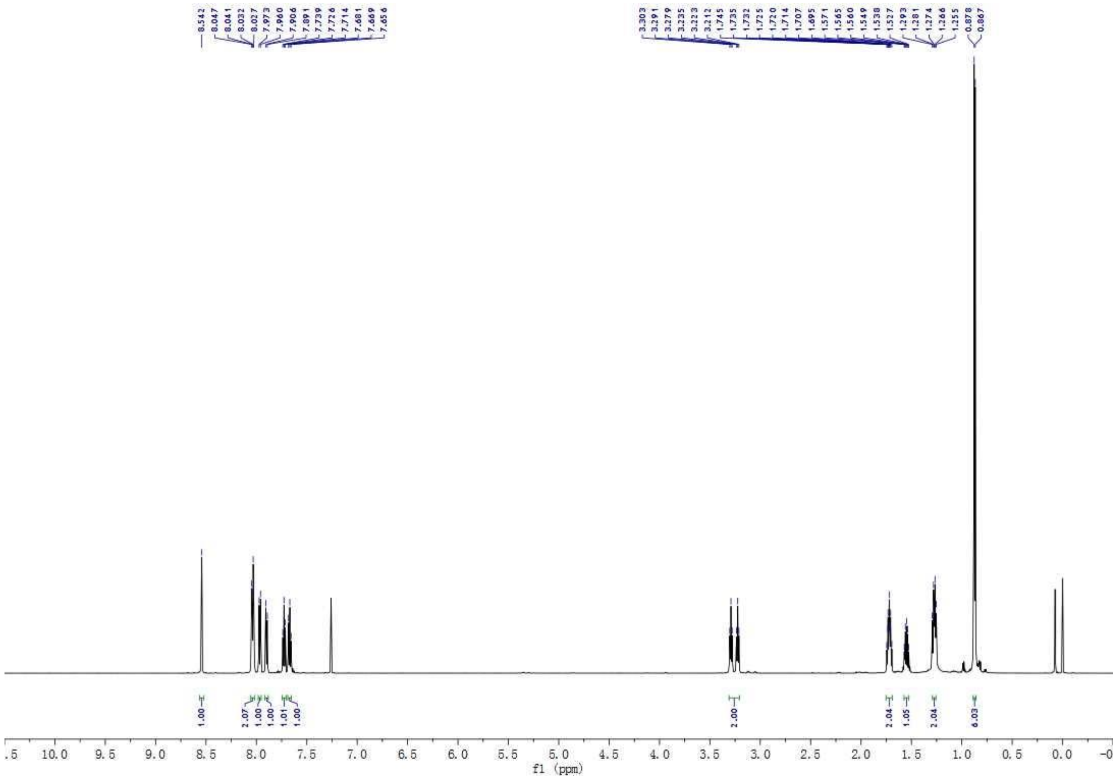

Supplementary Fig. 21. ^{1}H NMR of compound 2g. The sample has been recorded in 600 MHz, $CDCl_3$ at 25 °C.

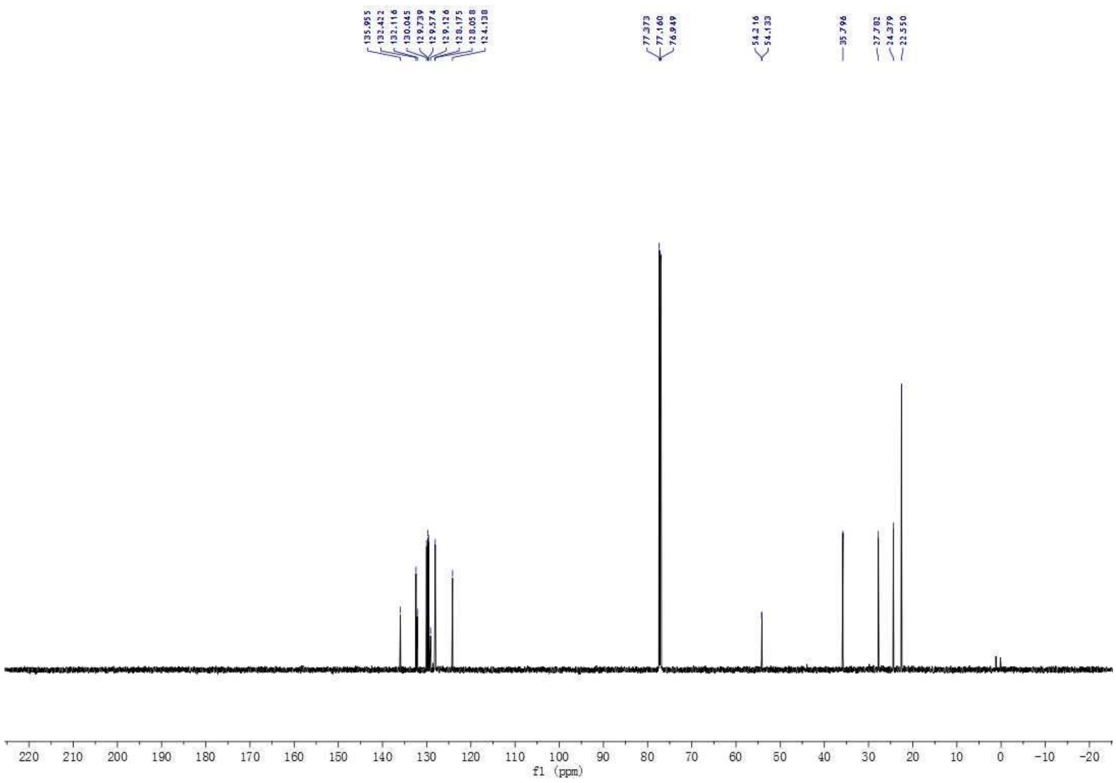

Supplementary Fig. 22. ^{13}C NMR of compound 2g. The sample has been recorded in 150 MHz, $CDCl_3$ at 25 °C.

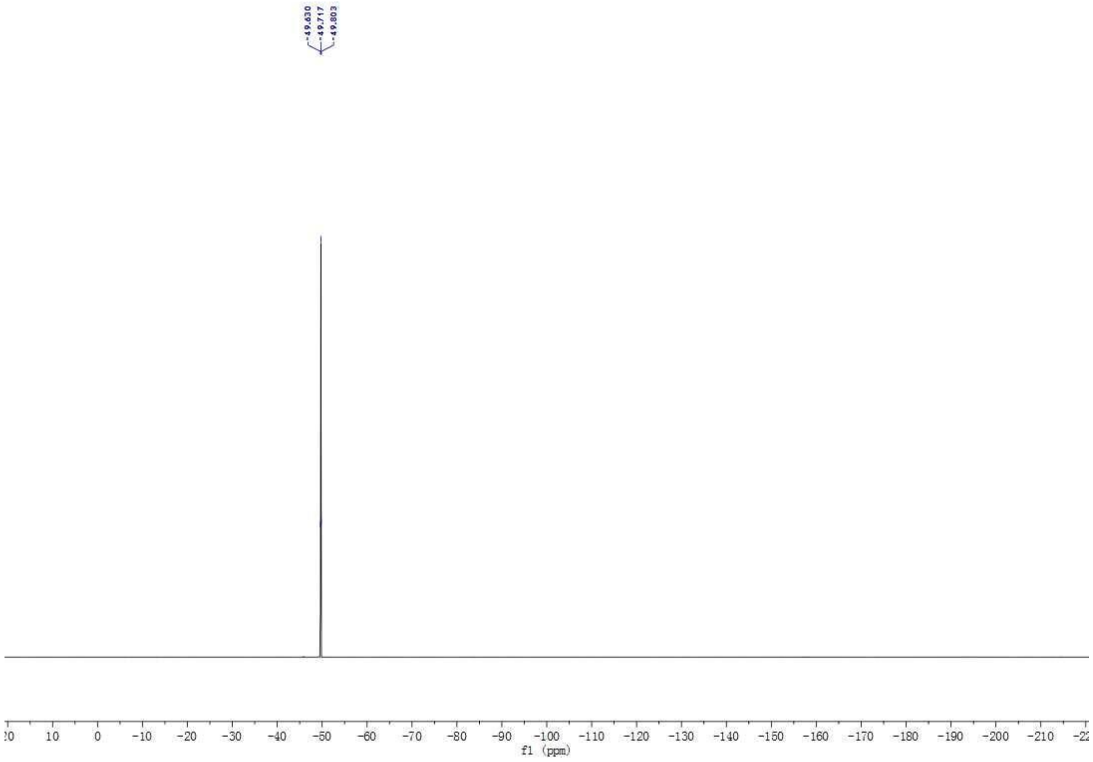

Supplementary Fig. 23. ^{19}F NMR of compound 2g. The sample has been recorded in 470 MHz, $CDCl_3$ at 25 °C.

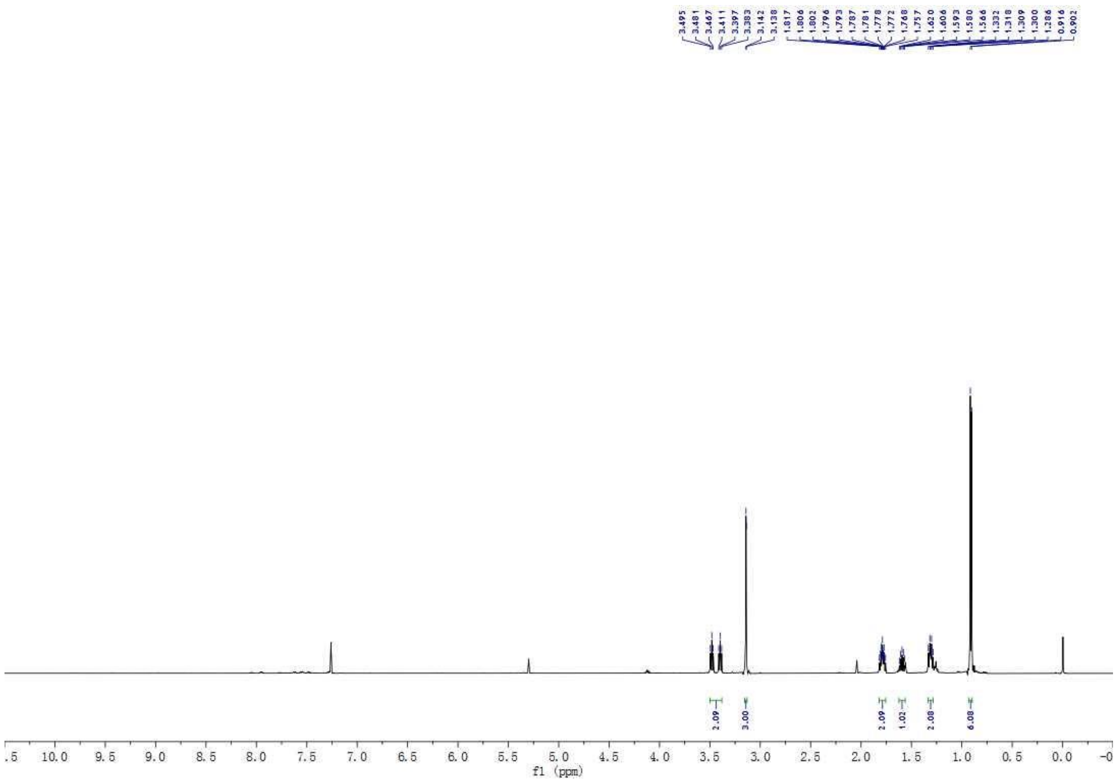

Supplementary Fig. 24. ^{1}H NMR of compound 2h. The sample has been recorded in 500 MHz, $CDCl_3$ at 25 °C.

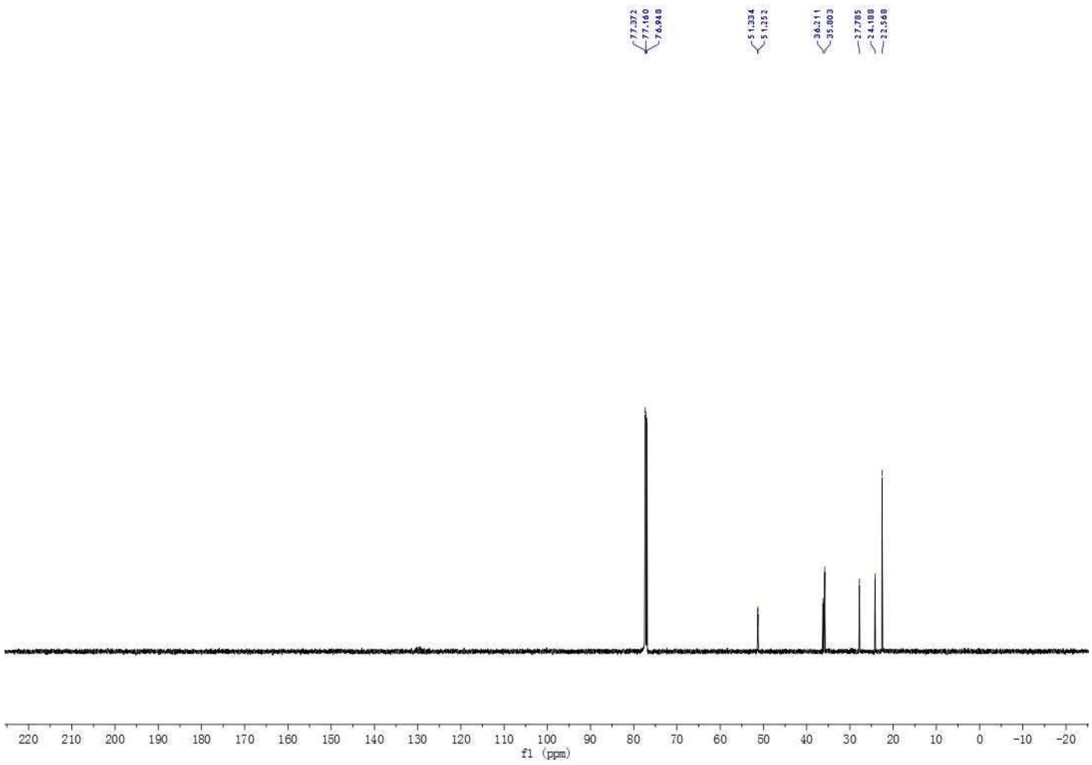

Supplementary Fig. 25. ^{13}C NMR of compound 2h. The sample has been recorded in 150 MHz, $CDCl_3$ at 25 °C

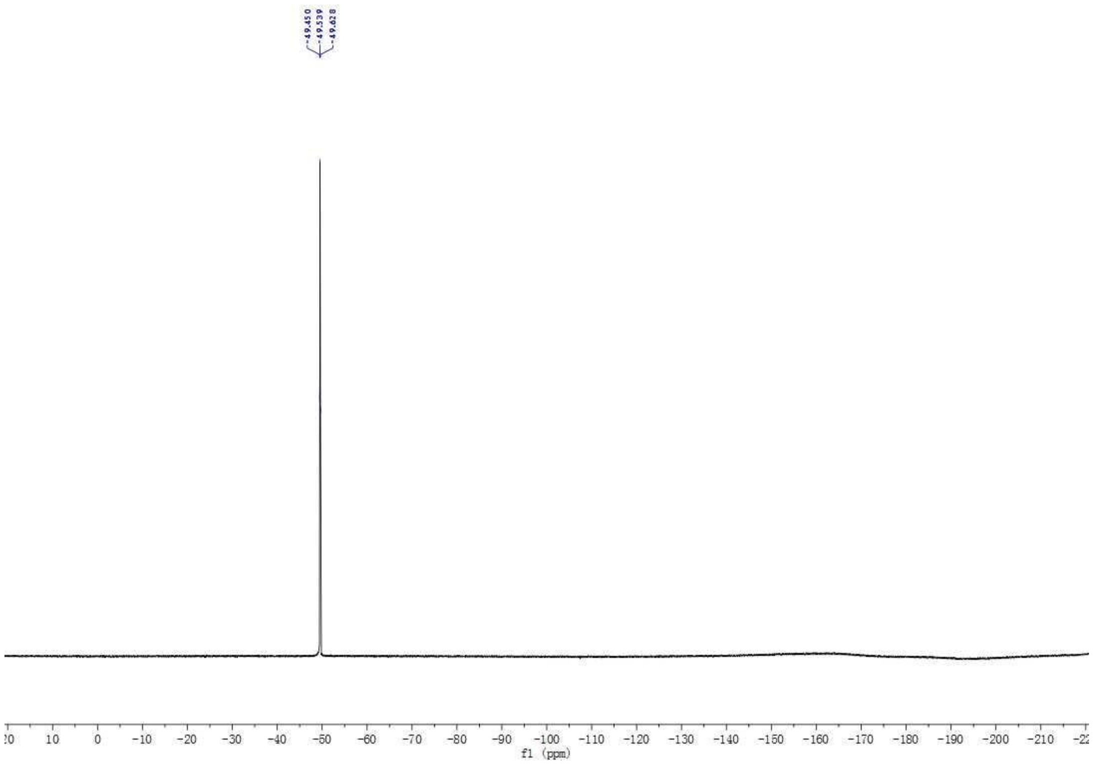

Supplementary Fig. 26. ^{19}F NMR of compound 2h. The sample has been recorded in 470 MHz, $CDCl_3$ at 25 °C

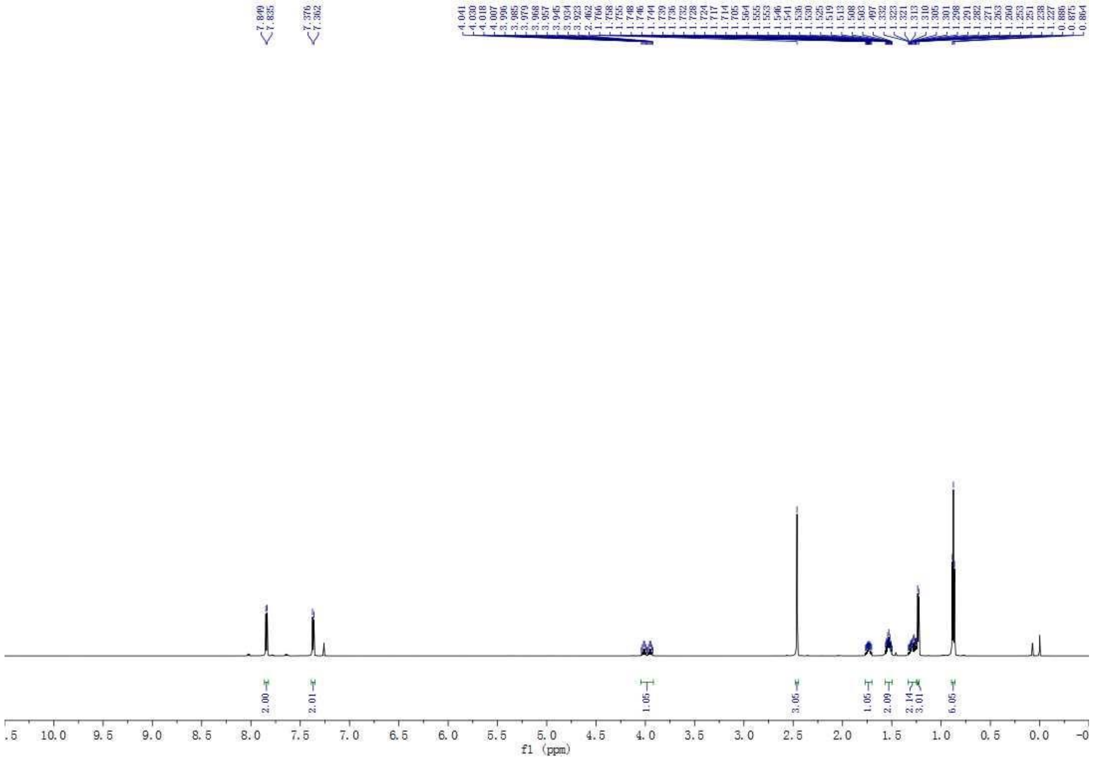

Supplementary Fig. 27. ^{1}H NMR of compound 2i. The sample has been recorded in 600 MHz, $CDCl_3$ at 25 °C

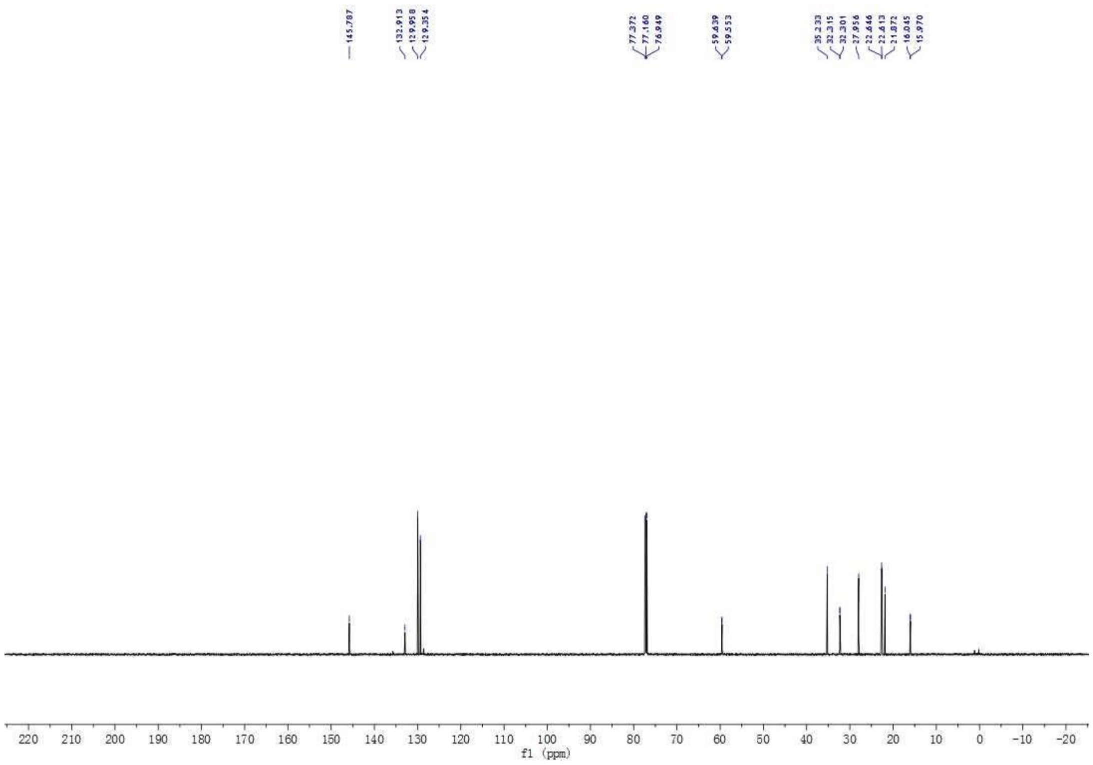

Supplementary Fig. 28. ^{13}C NMR of compound 2i. The sample has been recorded in 150 MHz, $CDCl_3$ at 25 °C

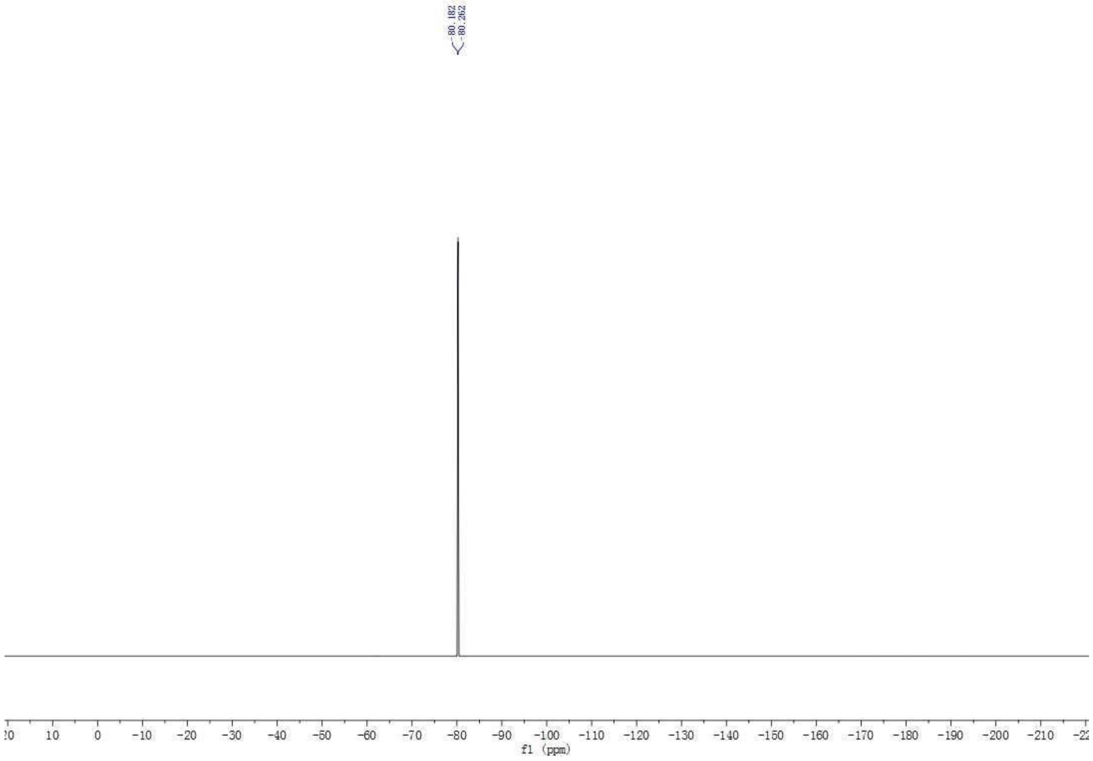

Supplementary Fig. 29. ^{19}F NMR of compound 2i. The sample has been recorded in 470 MHz, $CDCl_3$ at 25 °C

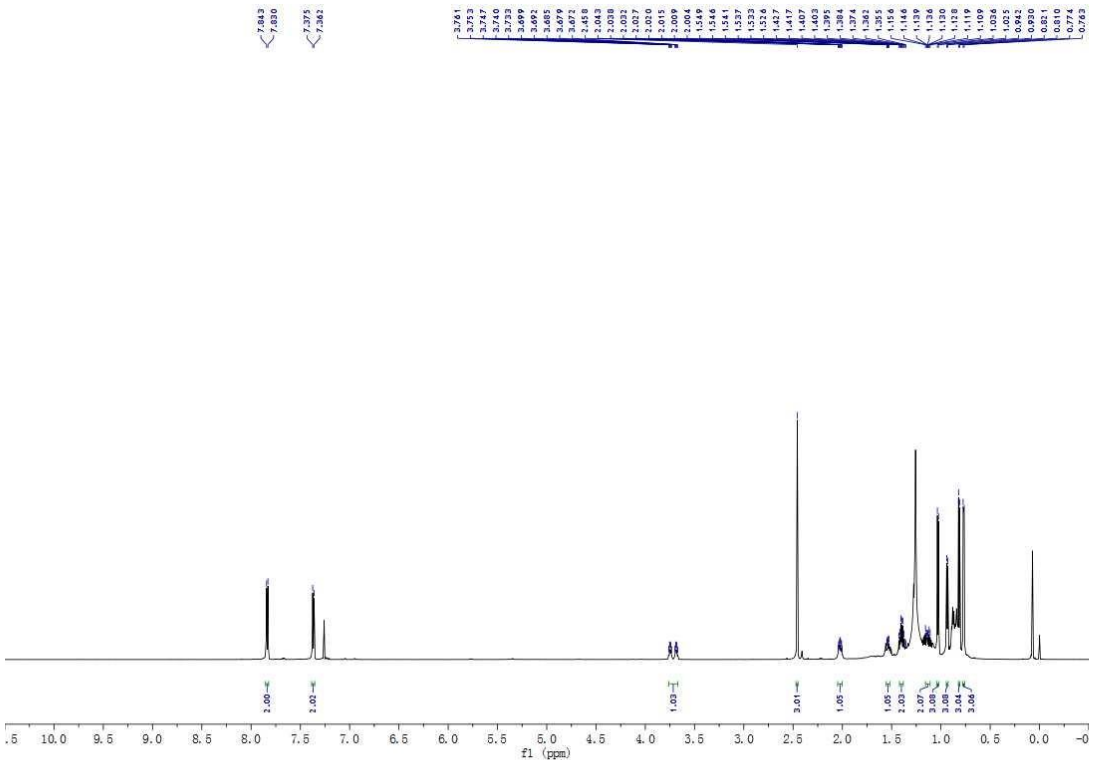

Supplementary Fig. 30. ^{1}H NMR of compound 2j. The sample has been recorded in 600 MHz, $CDCl_3$ at 25 °C

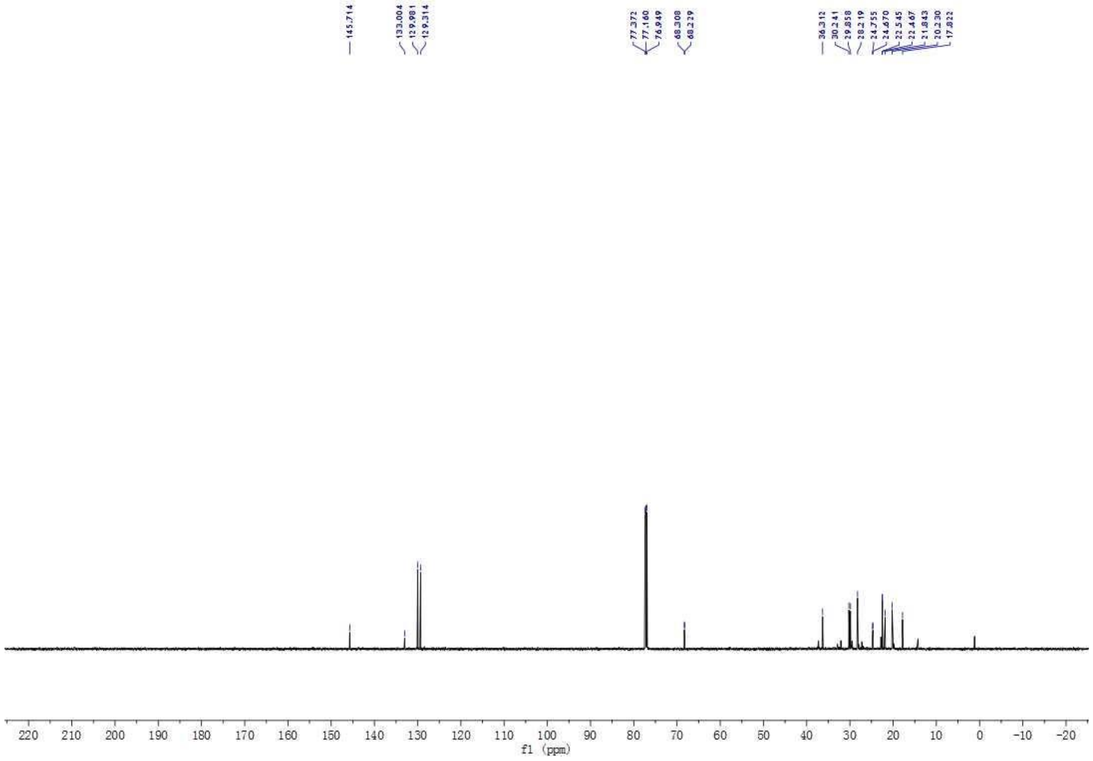

Supplementary Fig. 31. ^{13}C NMR of compound 2j. The sample has been recorded in 150 MHz, $CDCl_3$ at 25 °C

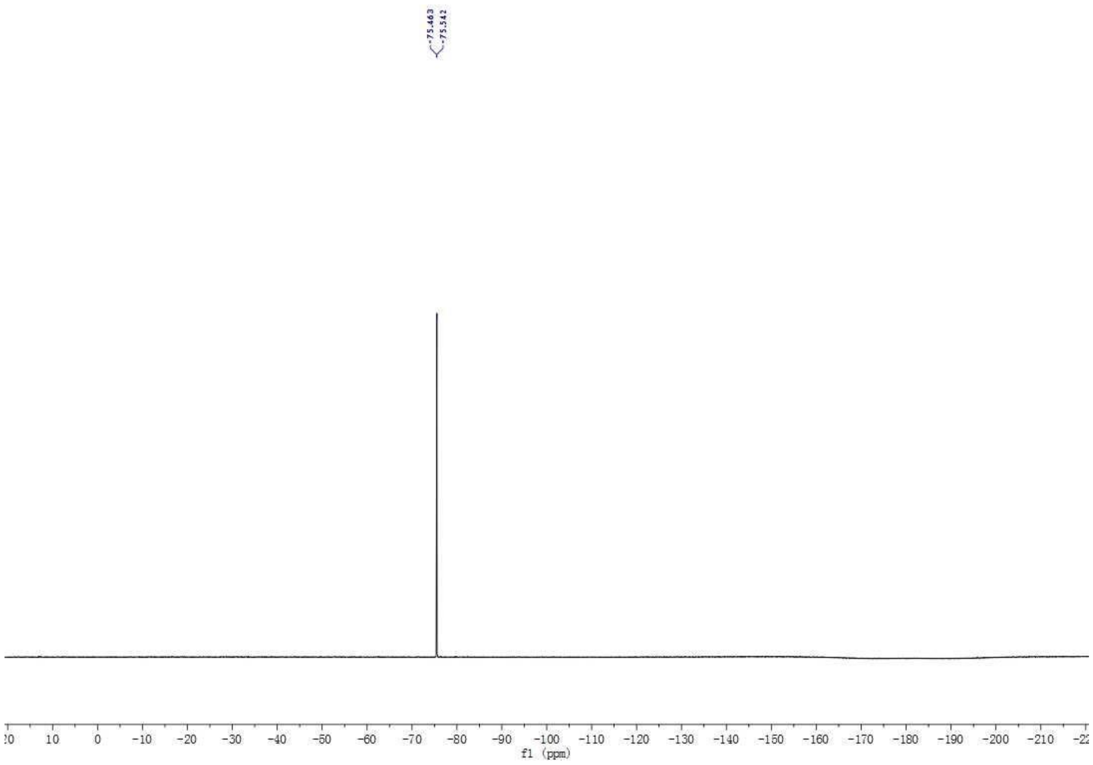

Supplementary Fig. 32. ^{19}F NMR of compound 2j. The sample has been recorded in 470 MHz, $CDCl_3$ at 25 °C

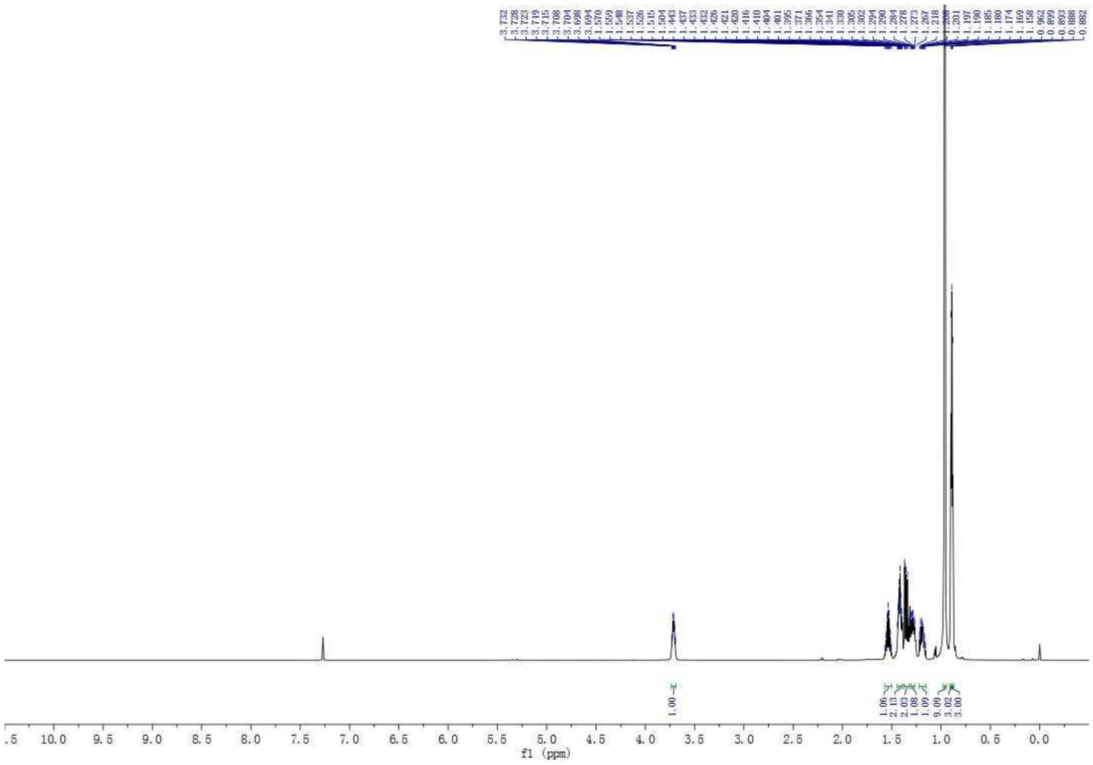

Supplementary Fig. 33. ^{1}H NMR of compound S2. The sample has been recorded in 600 MHz, $CDCl_3$ at 25 °C

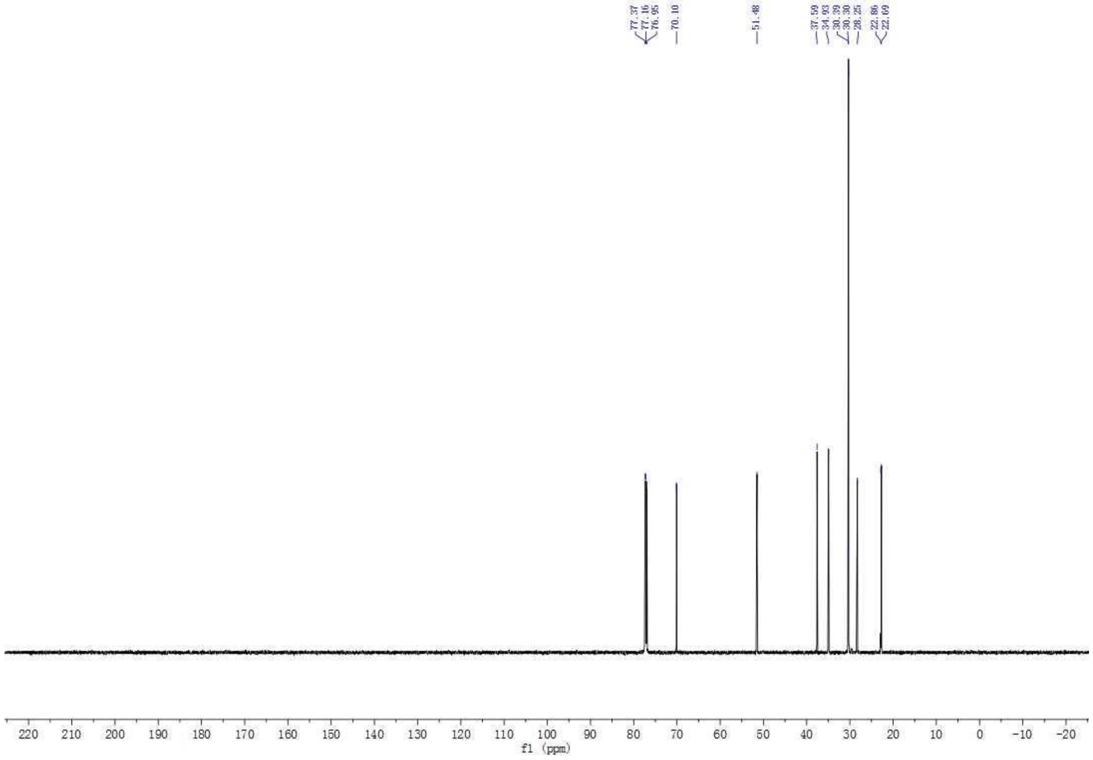

Supplementary Fig. 34. ^{13}C NMR of compound S2. The sample has been recorded in 150 MHz, $CDCl_3$ at 25 °C

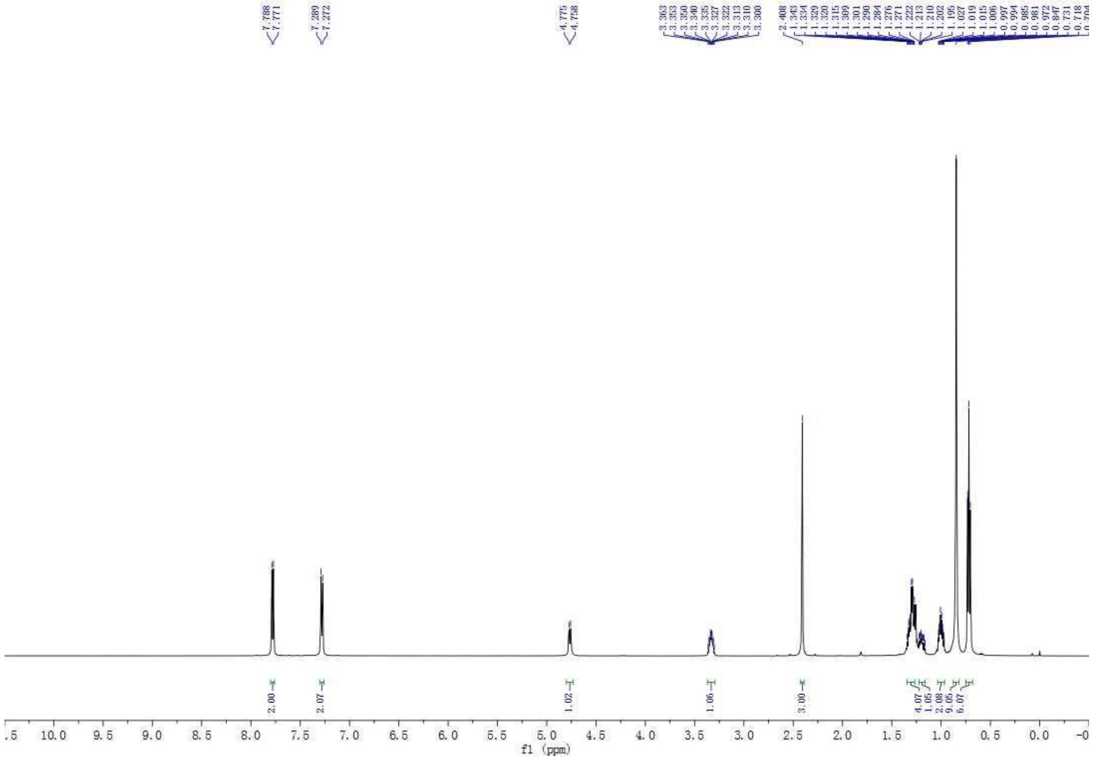

Supplementary Fig. 35. ^{1}H NMR of compound S4. The sample has been recorded in 500 MHz, $CDCl_3$ at 25 °C

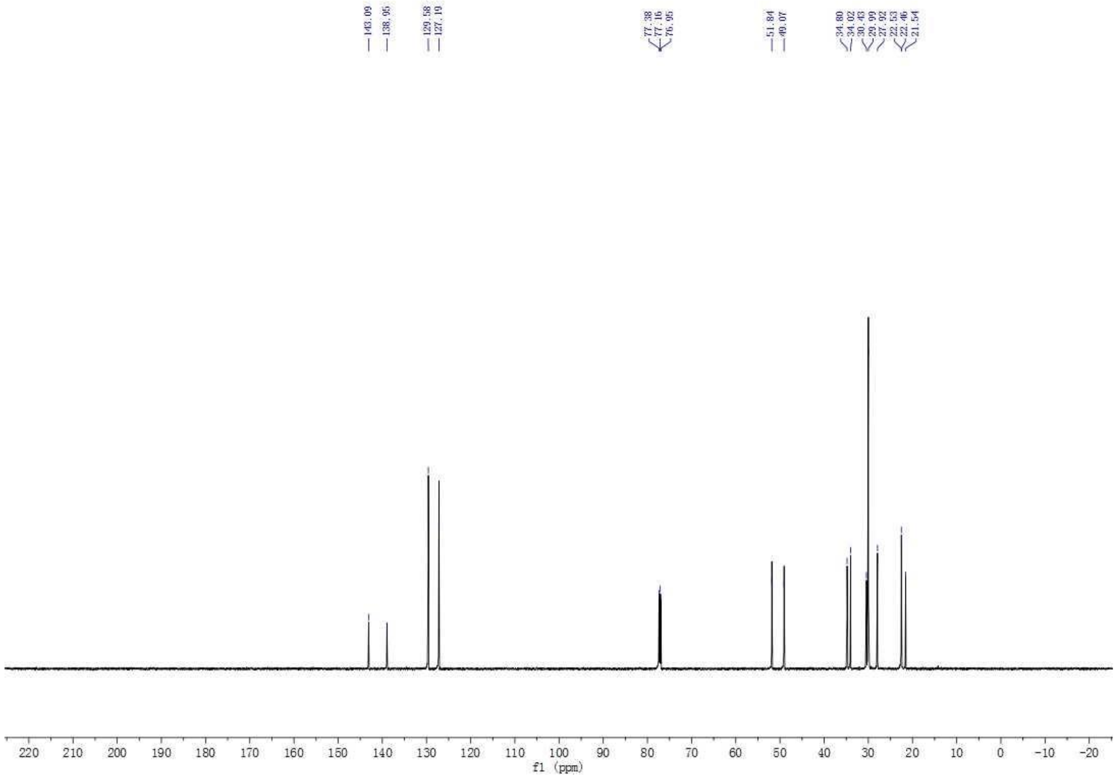

Supplementary Fig. 36. ^{13}C NMR of compound S4. The sample has been recorded in 150 MHz, $CDCl_3$ at 25 °C

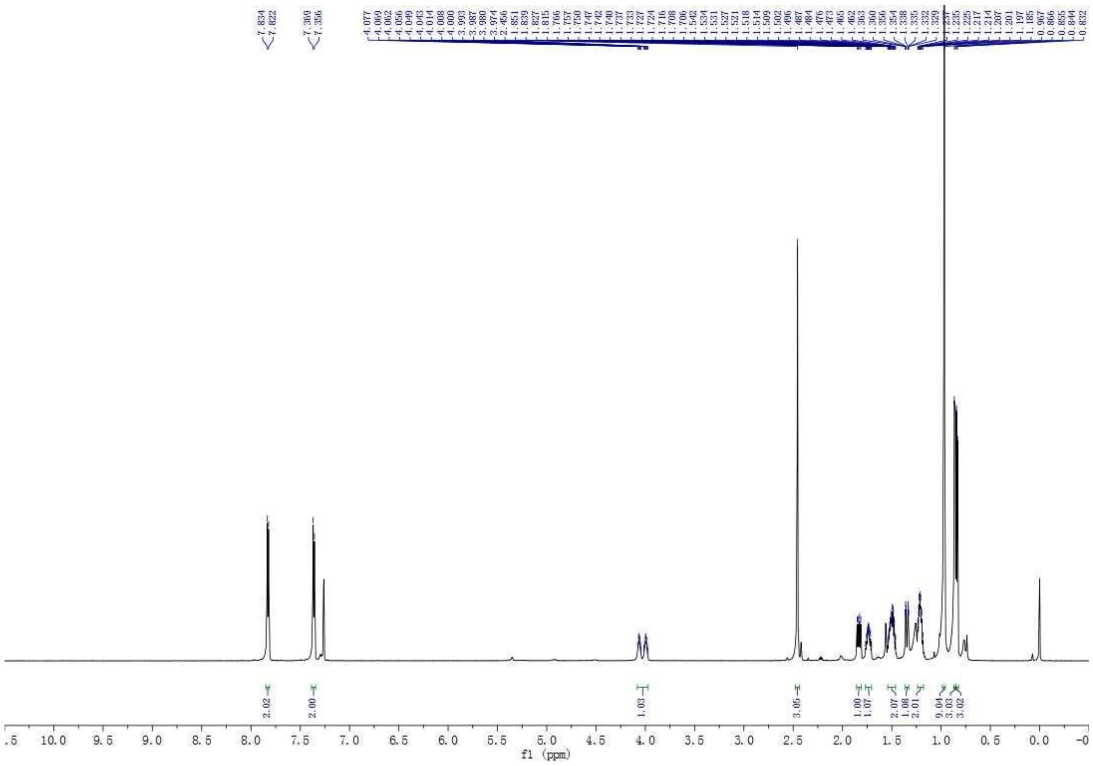

Supplementary Fig. 37. ^{1}H NMR of compound 2k. The sample has been recorded in 600 MHz, $CDCl_3$ at 25 °C

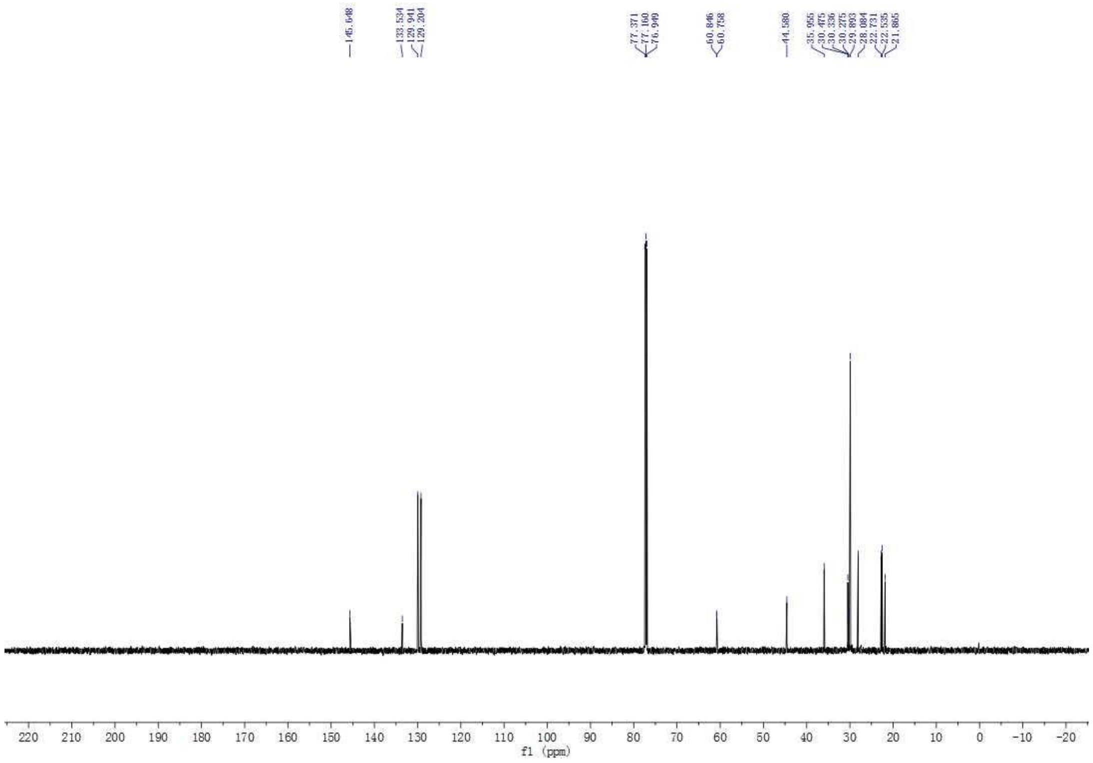

Supplementary Fig. 38. ^{13}C NMR of compound 2k. The sample has been recorded in 150 MHz, $CDCl_3$ at 25 °C

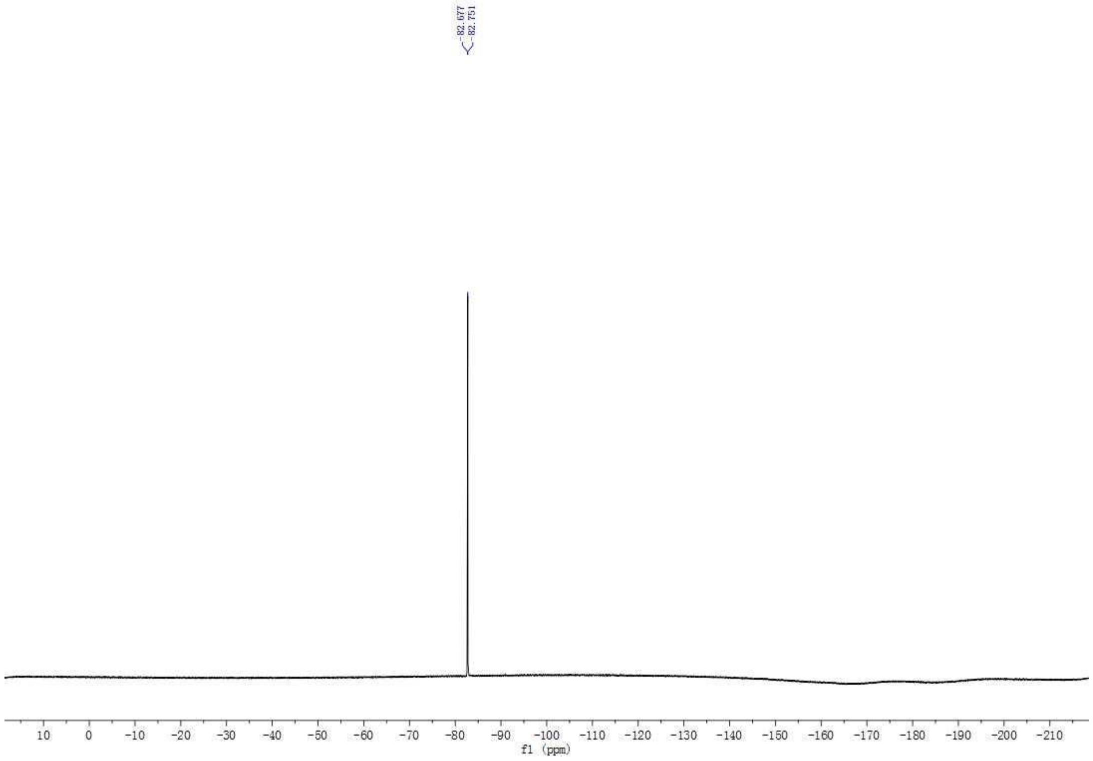

Supplementary Fig. 39. ^{19}F NMR of compound 2k. The sample has been recorded in 565 MHz, $CDCl_3$ at 25 °C

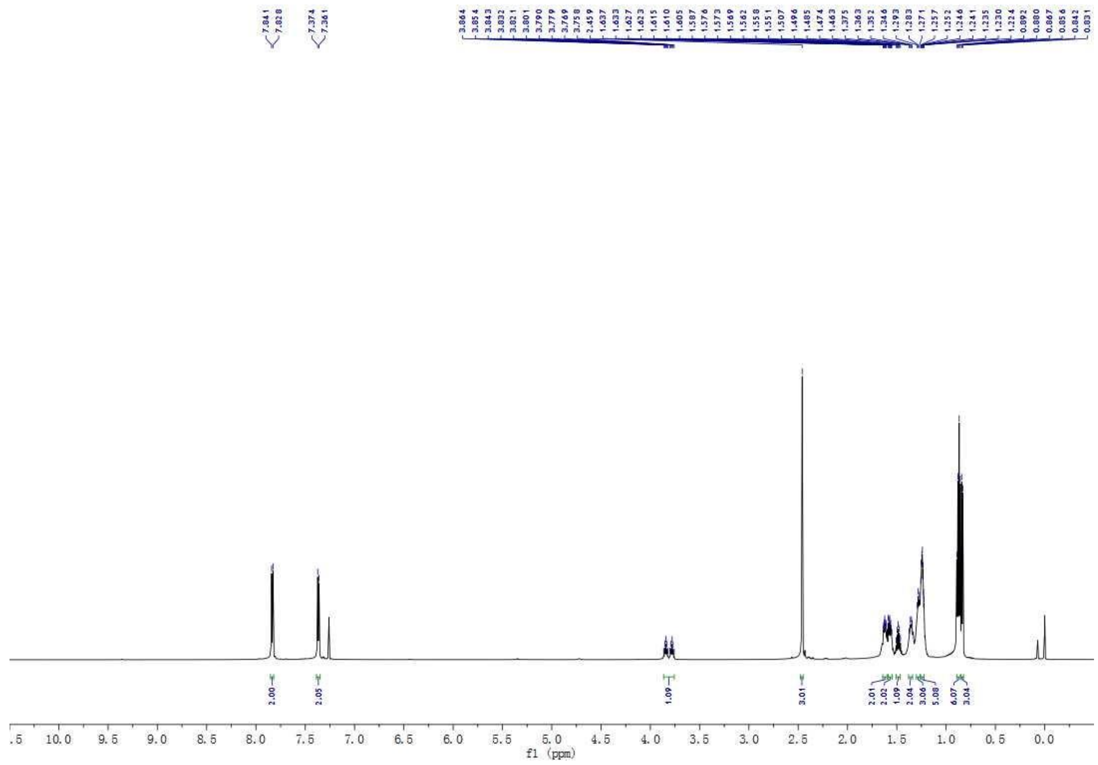

Supplementary Fig. 40. ^{1}H NMR of compound 2l. The sample has been recorded in 600 MHz, $CDCl_3$ at 25 °C

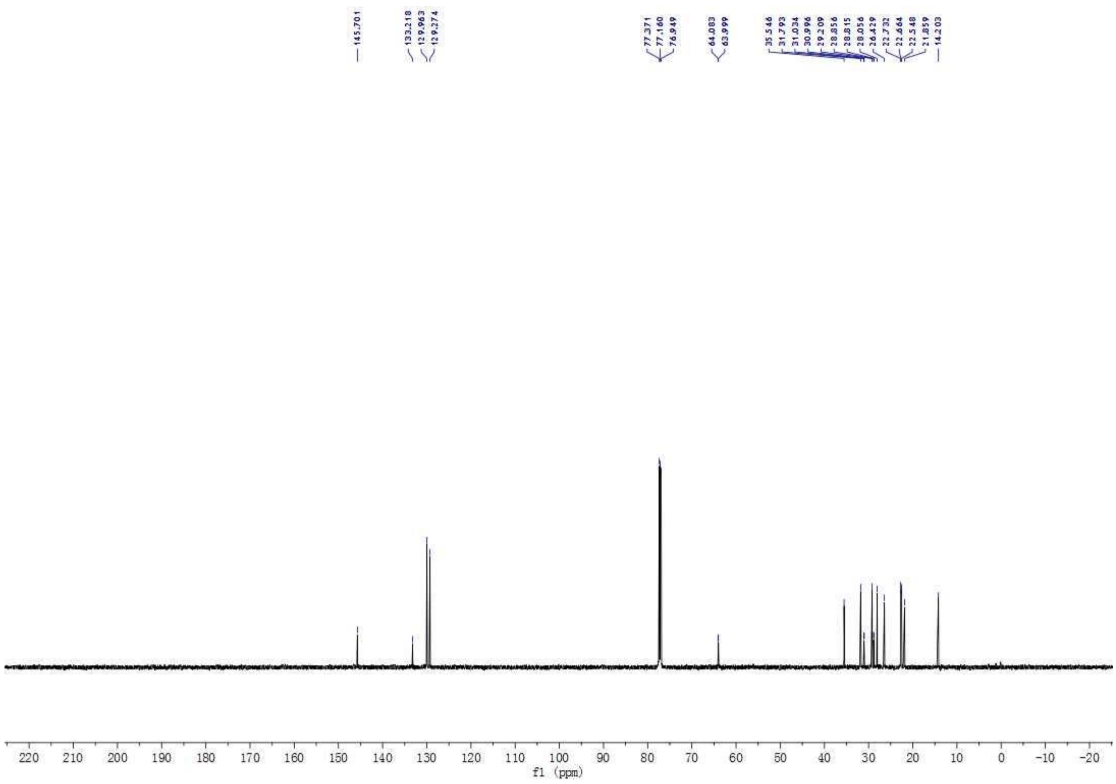

Supplementary Fig. 41. ^{13}C NMR of compound 2l. The sample has been recorded in 150 MHz, $CDCl_3$ at 25 °C

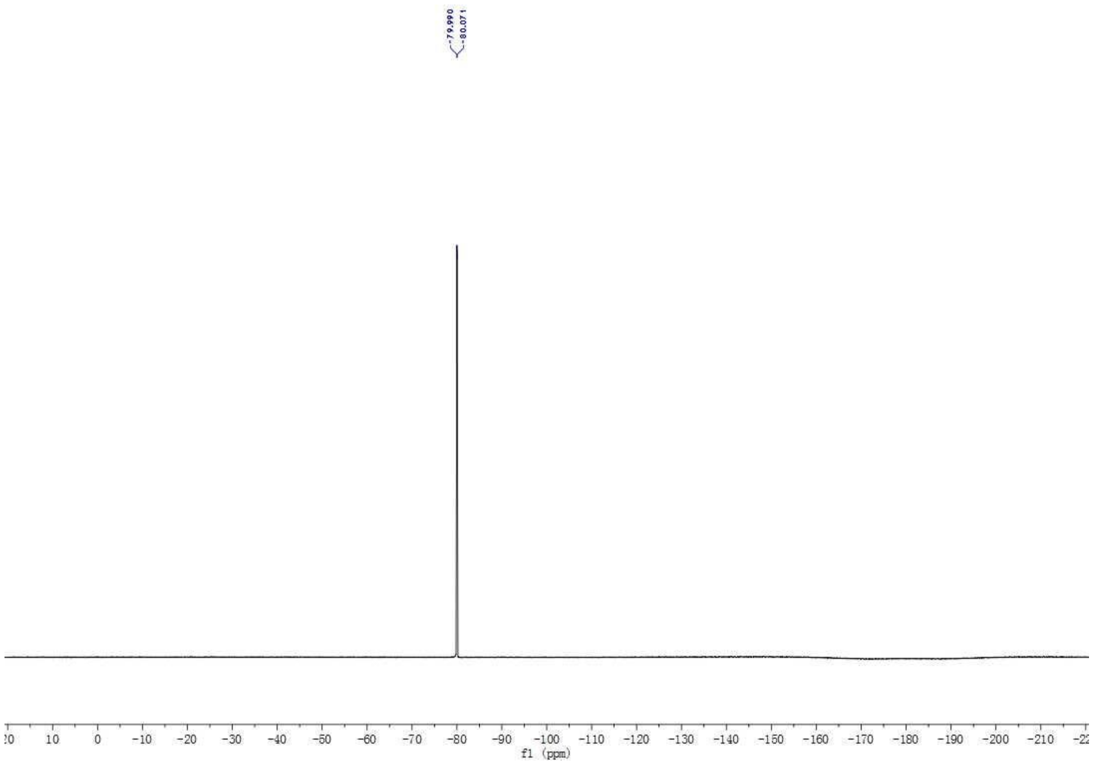

Supplementary Fig. 42. ^{19}F NMR of compound 2l. The sample has been recorded in 470 MHz, $CDCl_3$ at 25 °C

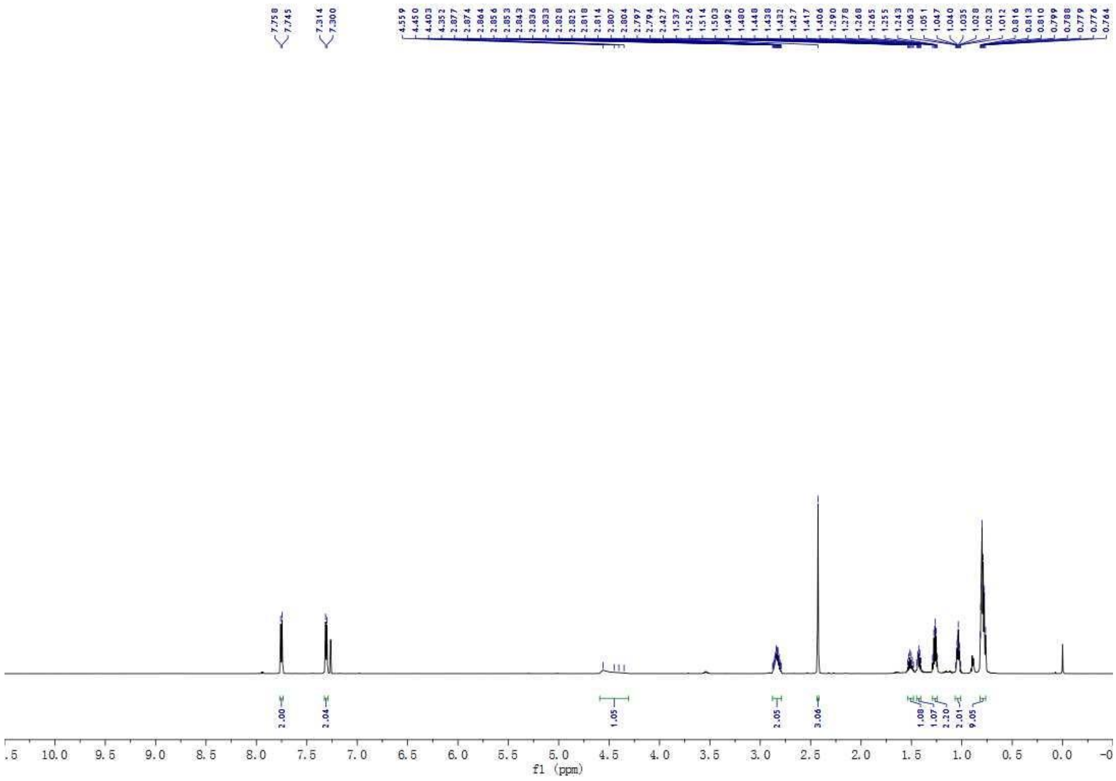

Supplementary Fig. 43. ^{1}H NMR of compound S7. The sample has been recorded in 600 MHz, $CDCl_3$ at 25 °C

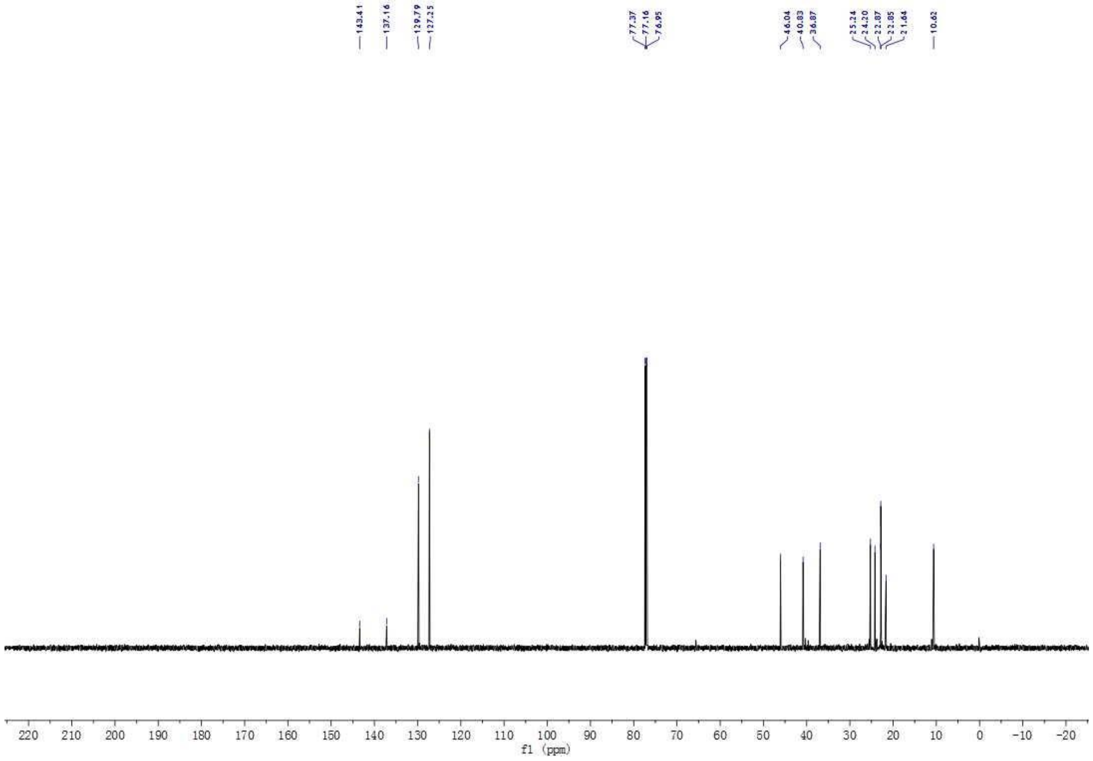

Supplementary Fig. 44. ^{13}C NMR of compound S7. The sample has been recorded in 150 MHz, $CDCl_3$ at 25 °C

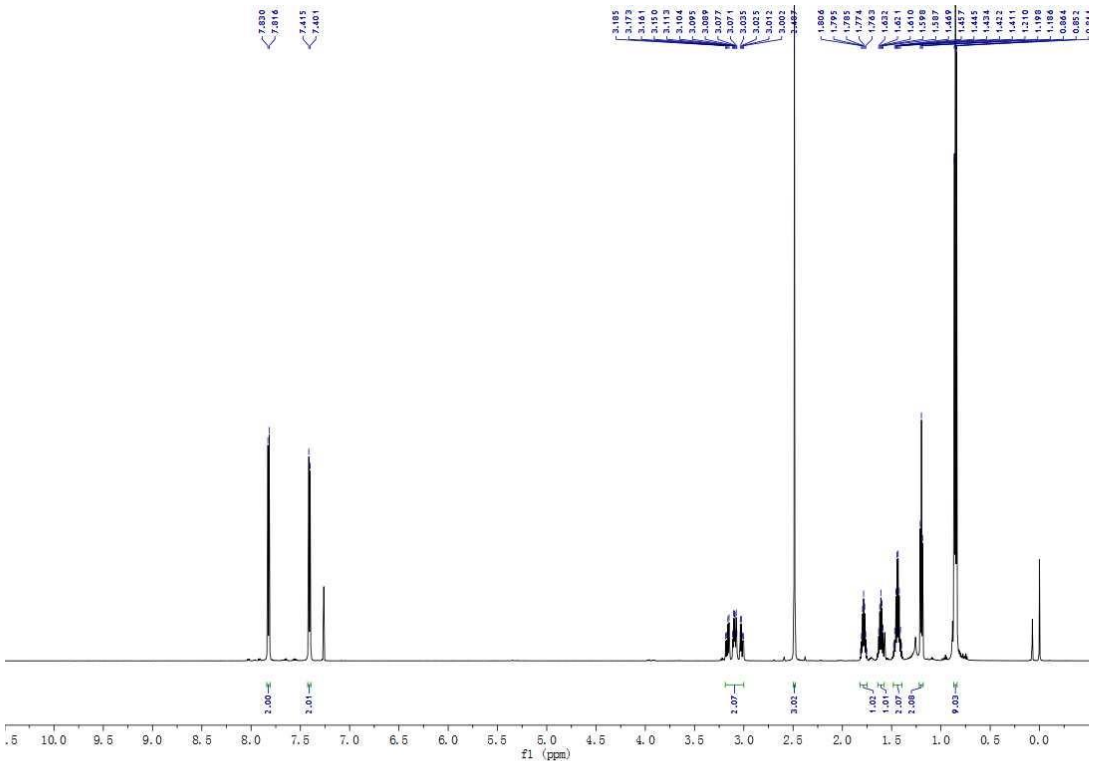

Supplementary Fig. 45. ^{1}H NMR of compound 2m. The sample has been recorded in 600 MHz, $CDCl_3$ at 25 °C

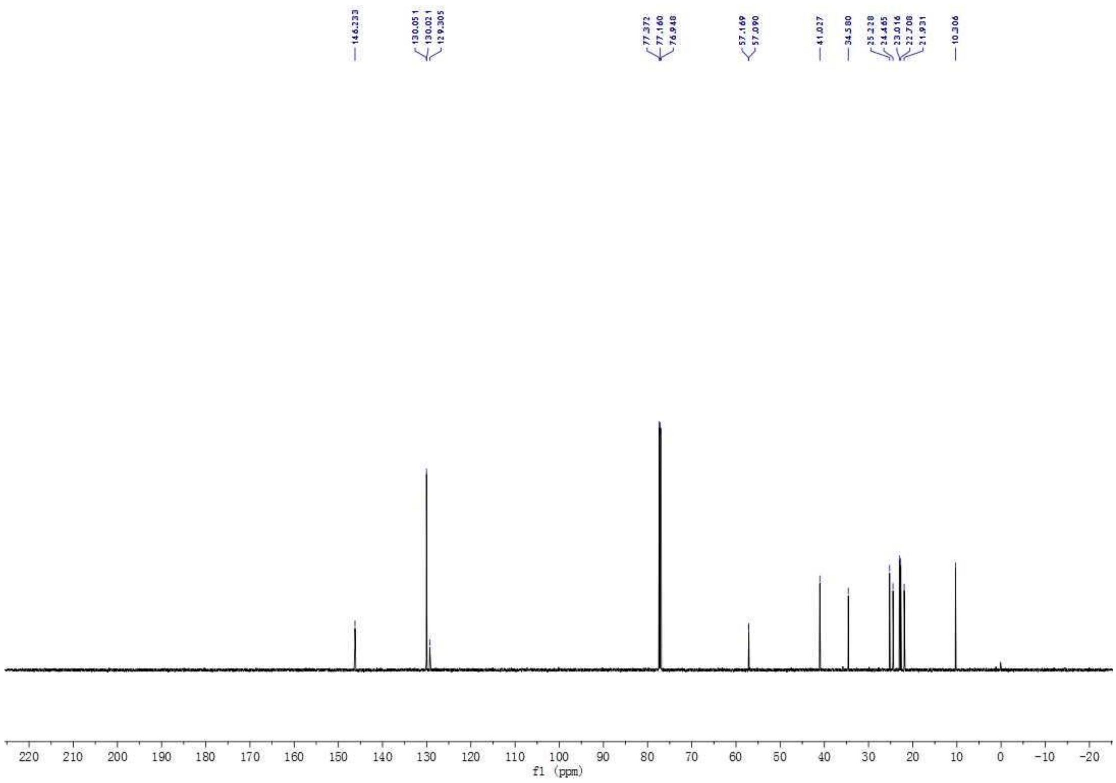

Supplementary Fig. 46. ^{13}C NMR of compound 2m. The sample has been recorded in 150 MHz, $CDCl_3$ at 25 °C

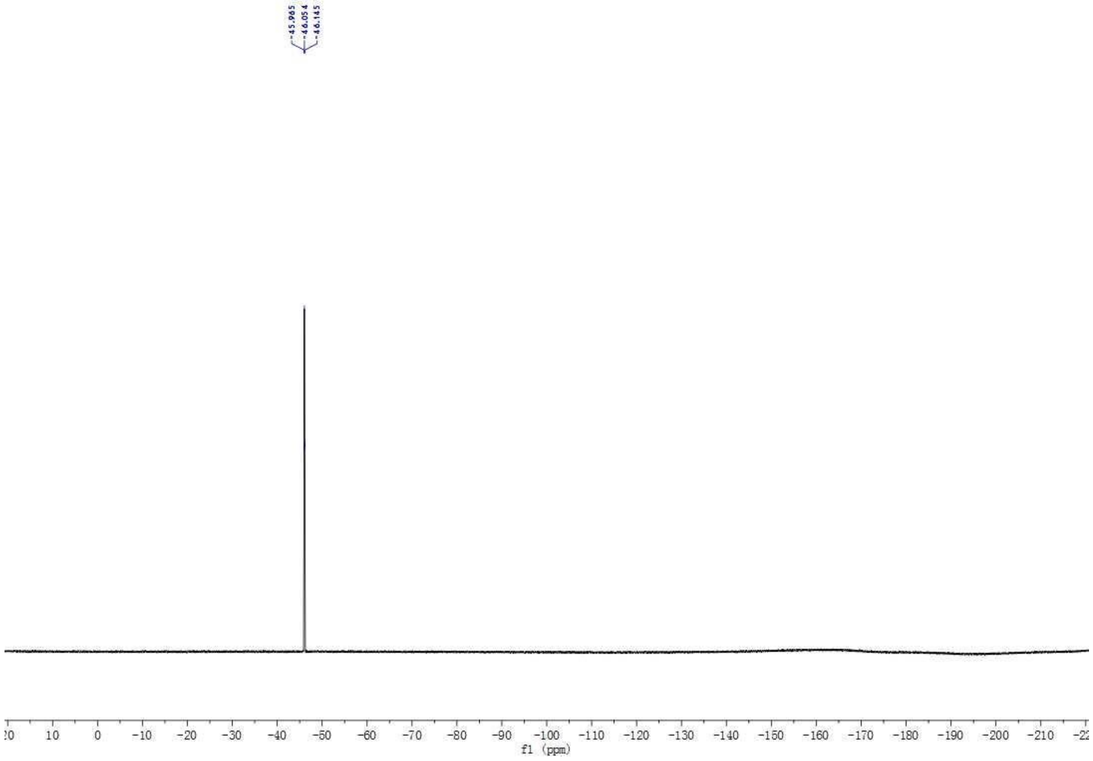

Supplementary Fig. 47. ^{19}F NMR of compound 2m. The sample has been recorded in 470 MHz, $CDCl_3$ at 25 °C

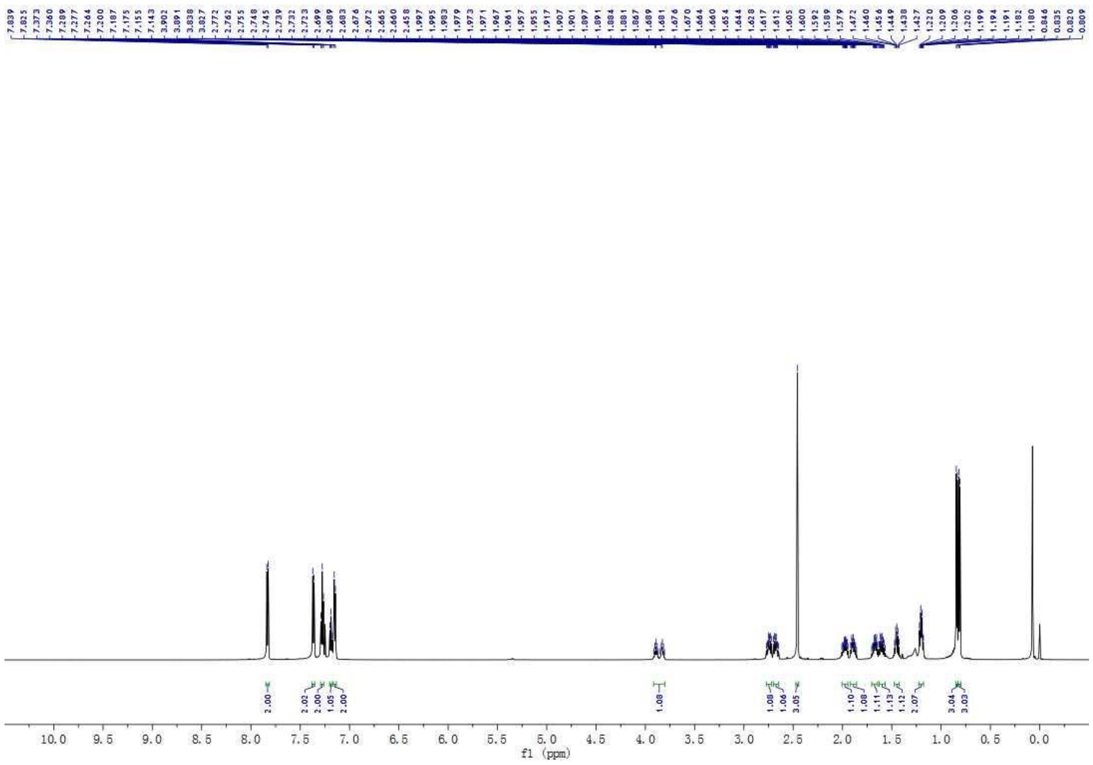

Supplementary Fig. 48. ¹H NMR of compound 2n. The sample has been recorded in 600 MHz, $CDCl_3$ at 25 °C

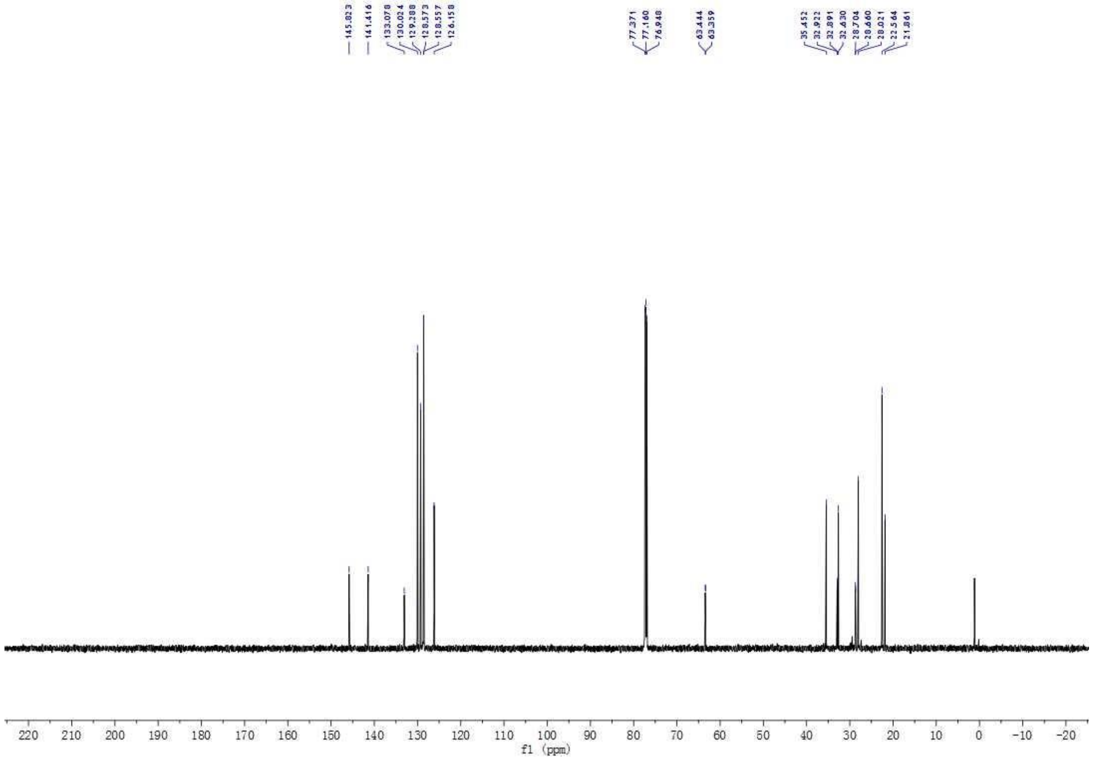

Supplementary Fig. 49. ¹³C NMR of compound 2n. The sample has been recorded in 150 MHz, $CDCl_3$ at 25 °C

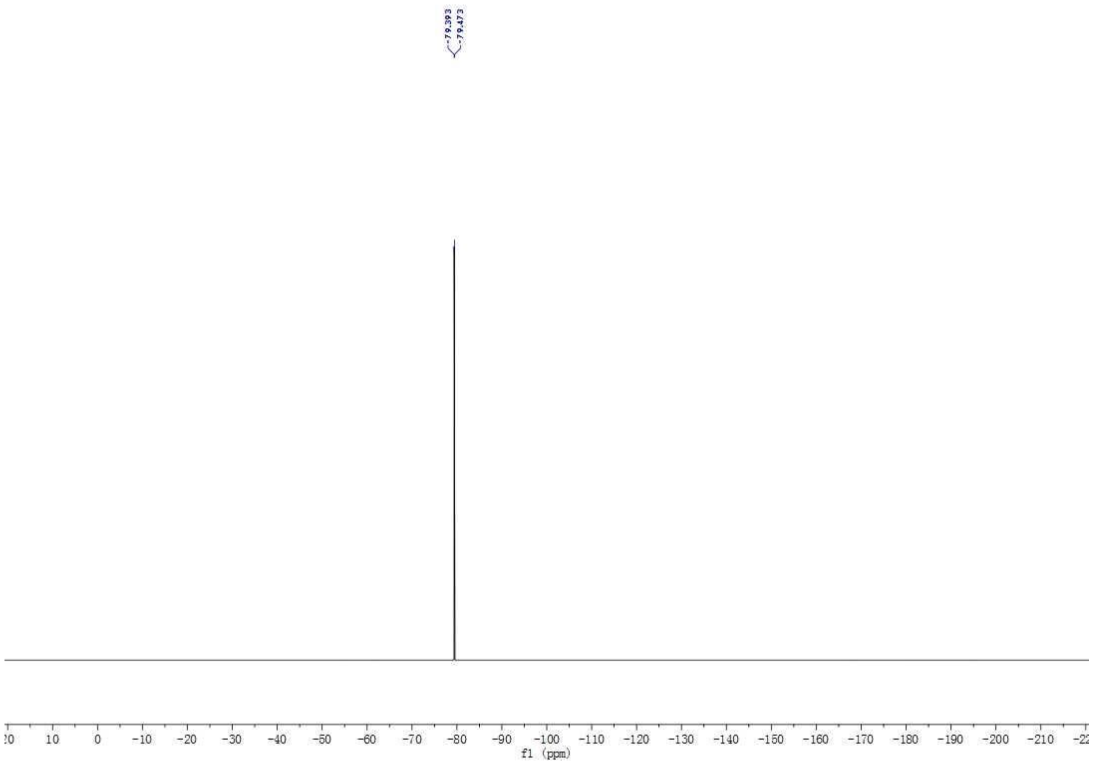

Supplementary Fig. 50. ^{19}F NMR of compound 2n. The sample has been recorded in 470 MHz, $CDCl_3$ at 25 °C

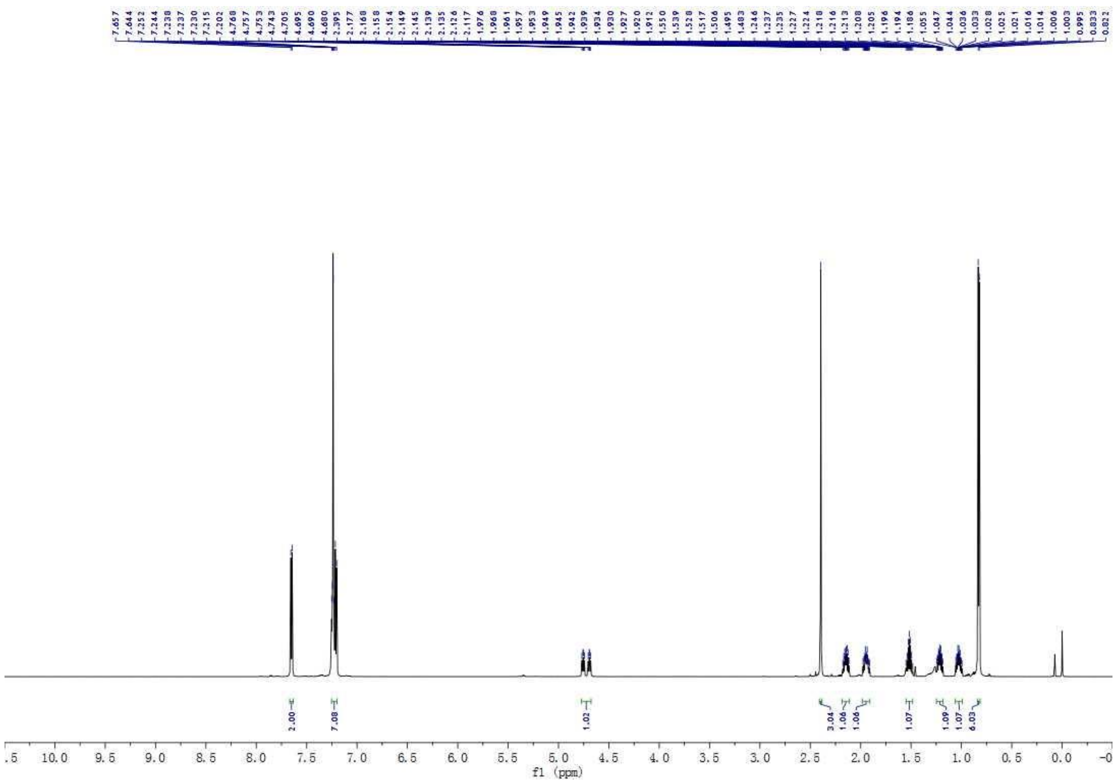

Supplementary Fig. 51. ^{1}H NMR of compound 2o. The sample has been recorded in 600 MHz, $CDCl_3$ at 25 °C

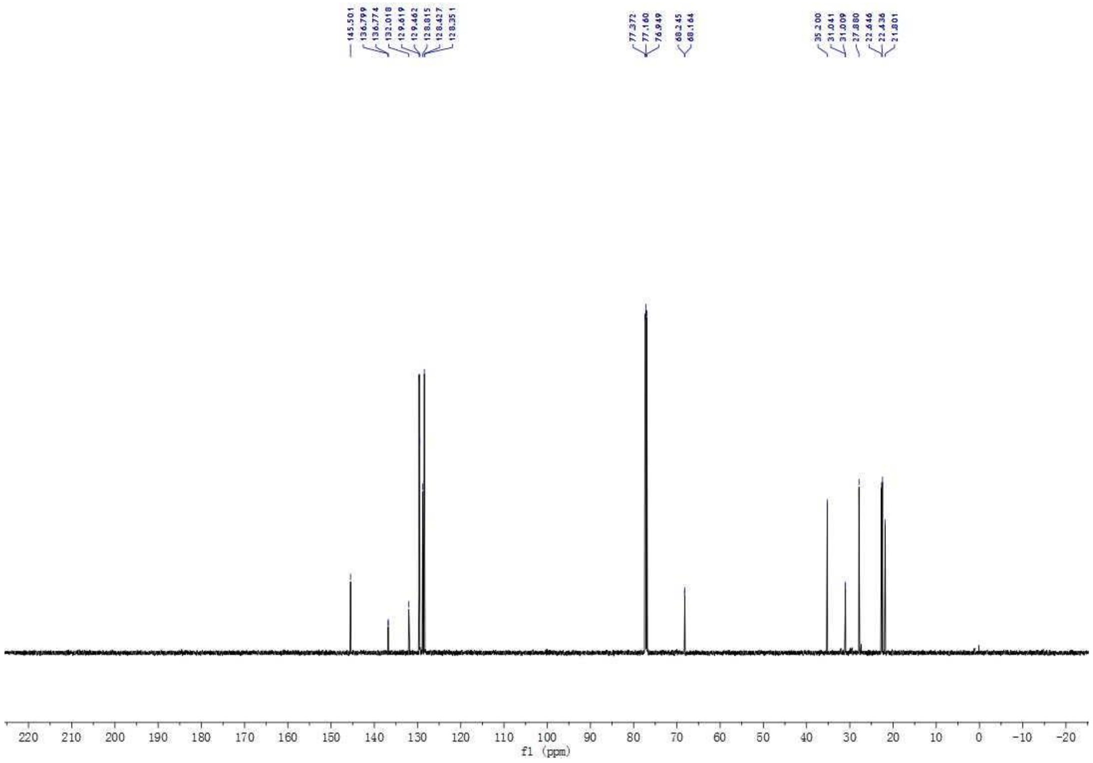

Supplementary Fig. 52. ^{13}C NMR of compound 2o. The sample has been recorded in 150 MHz, $CDCl_3$ at 25 °C

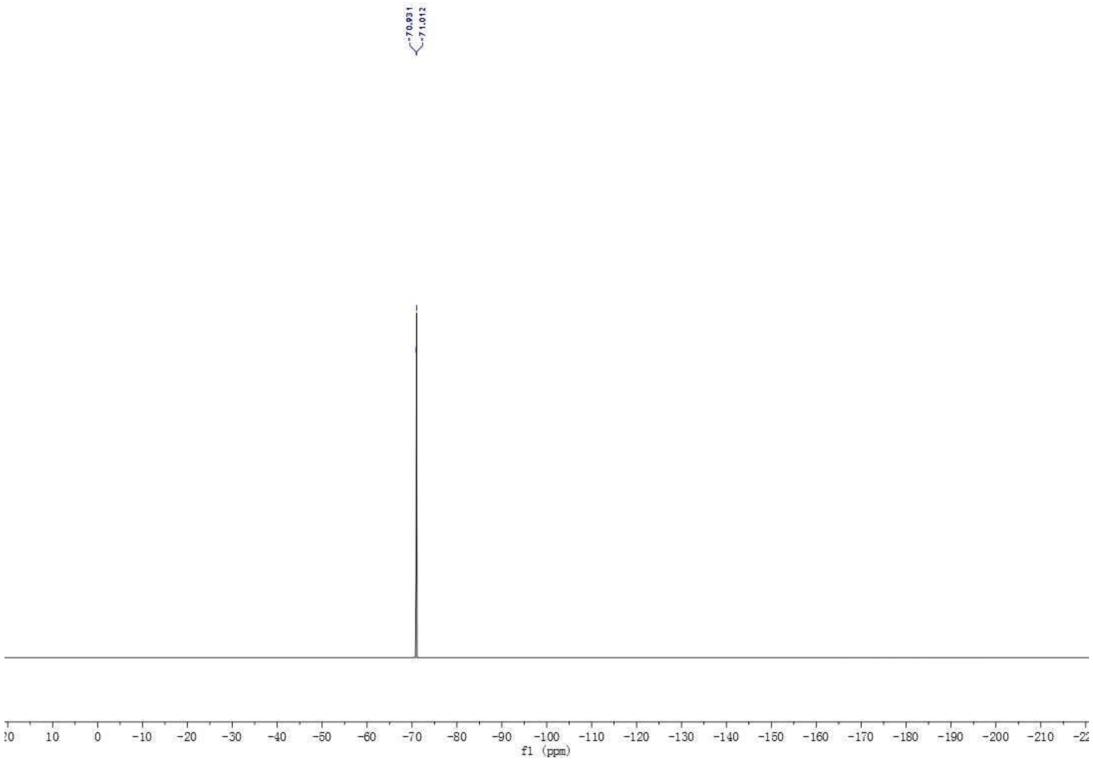

Supplementary Fig. 53. ^{19}F NMR of compound 2o. The sample has been recorded in 470 MHz, $CDCl_3$ at 25 °C

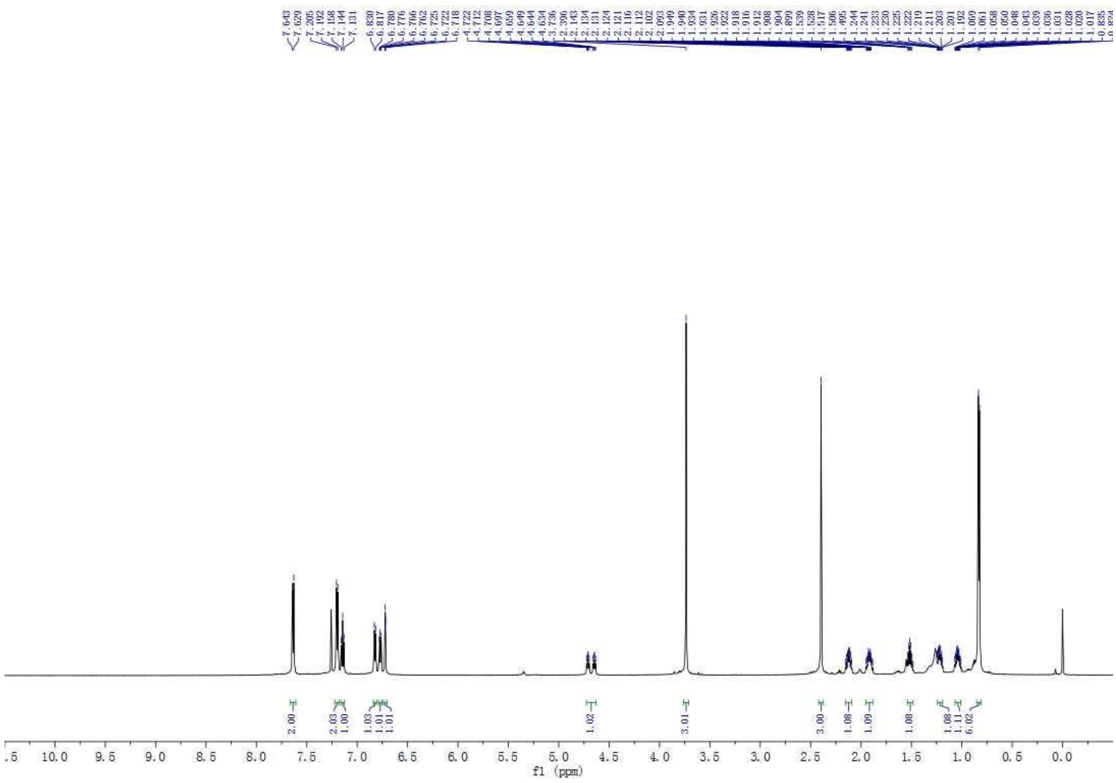

Supplementary Fig. 54. ^{1}H NMR of compound 2p. The sample has been recorded in 600 MHz, $CDCl_3$ at 25 °C

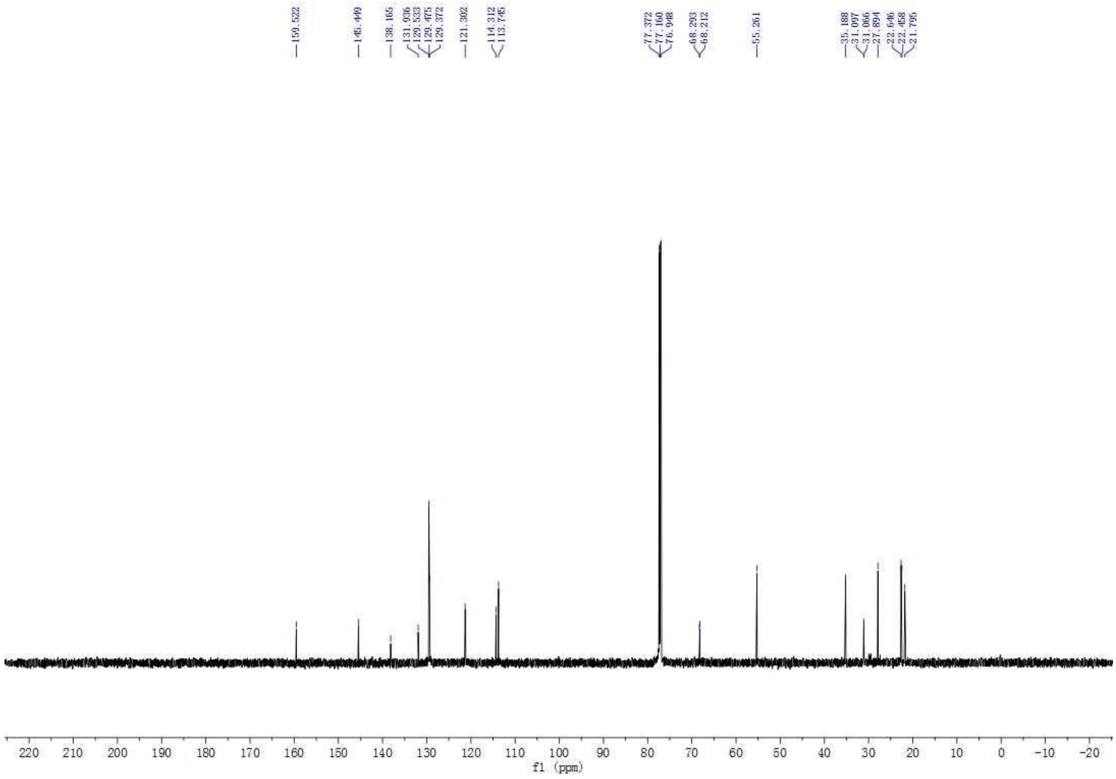

Supplementary Fig. 55. ^{13}C NMR of compound 2p. The sample has been recorded in 150 MHz, $CDCl_3$ at 25 °C

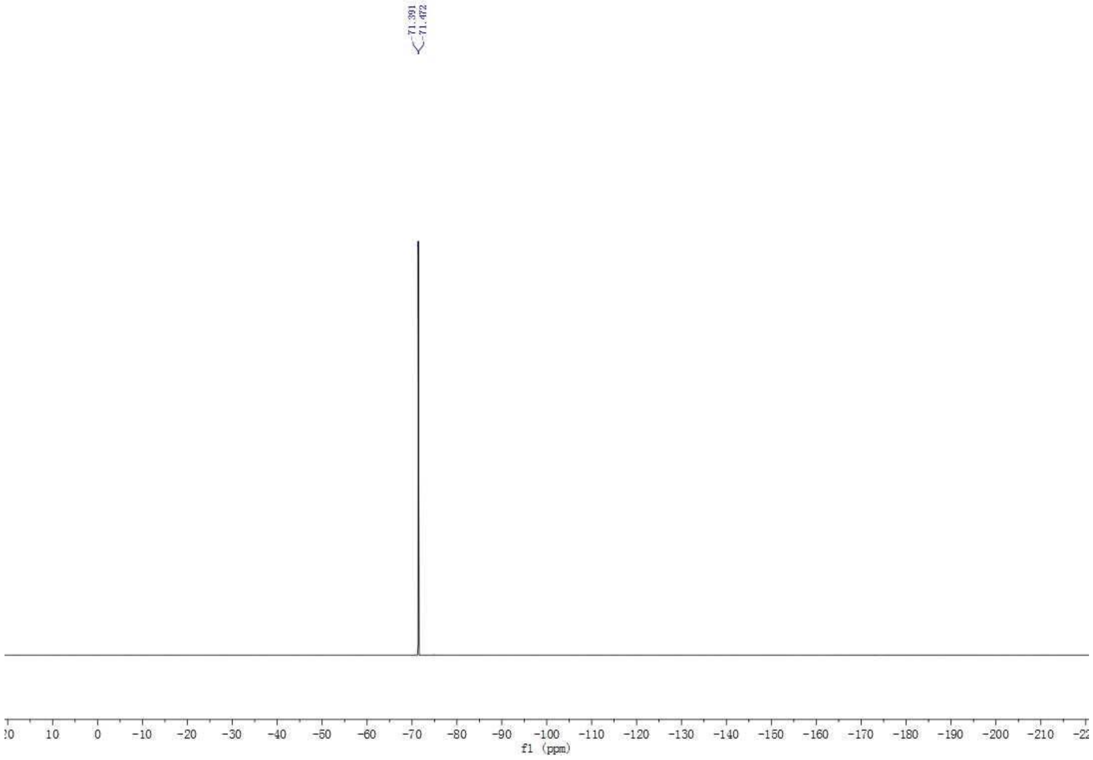

Supplementary Fig. 56. ^{19}F NMR of compound 2p. The sample has been recorded in 470 MHz, $CDCl_3$ at 25 °C

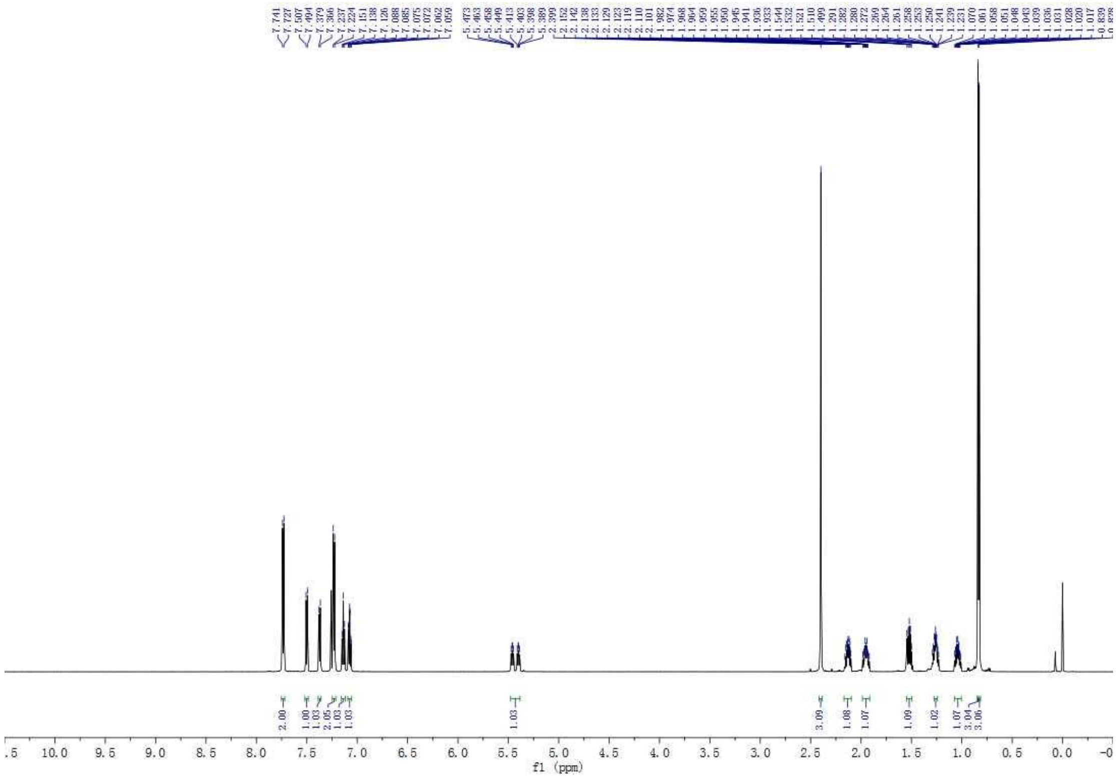

Supplementary Fig. 57. ^{1}H NMR of compound 2q. The sample has been recorded in 600 MHz, $CDCl_3$ at 25 °C

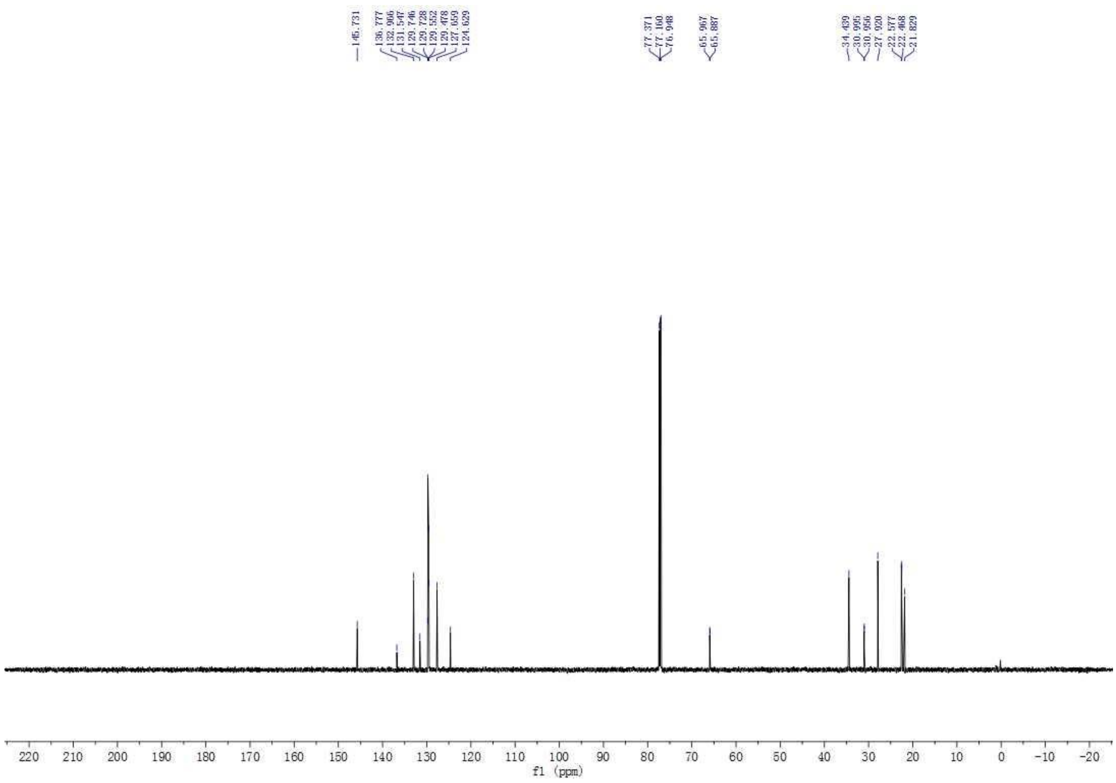

Supplementary Fig. 58. ^{13}C NMR of compound 2q. The sample has been recorded in 150 MHz, $CDCl_3$ at 25 °C

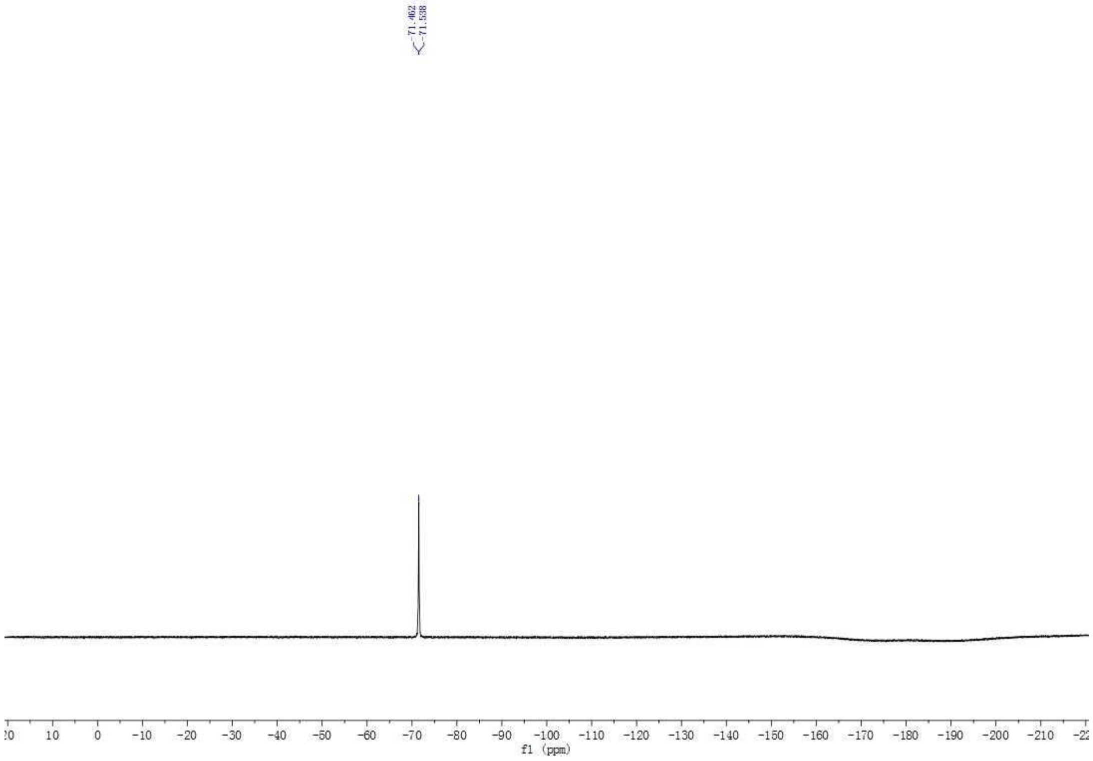

Supplementary Fig. 59. ^{19}F NMR of compound 2q. The sample has been recorded in 470 MHz, $CDCl_3$ at 25 °C

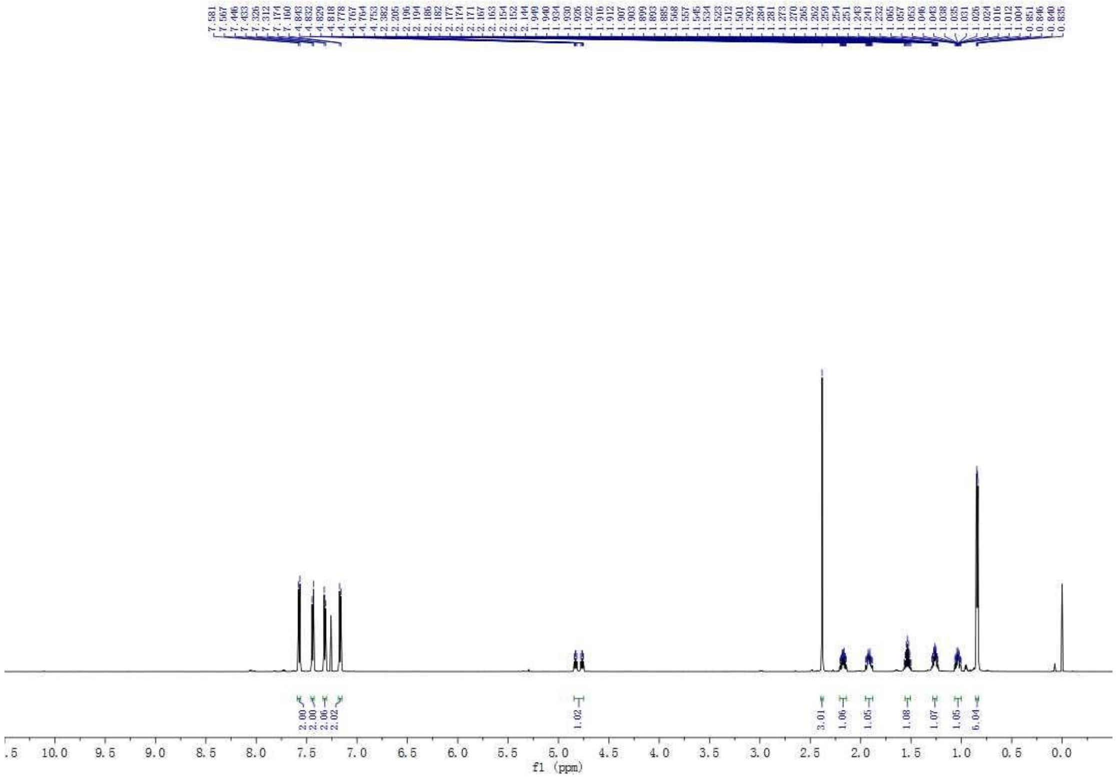

Supplementary Fig. 60. ^{1}H NMR of compound 2r. The sample has been recorded in 600 MHz, $CDCl_3$ at 25 °C

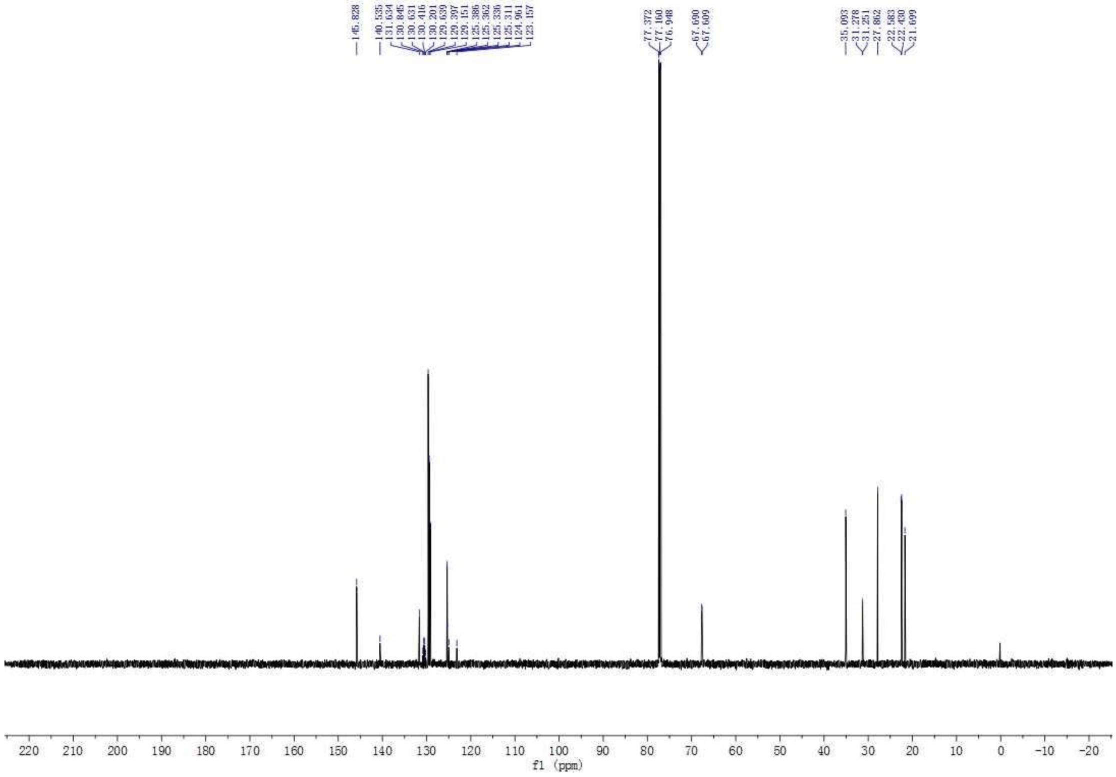

Supplementary Fig. 61. ^{13}C NMR of compound 2r. The sample has been recorded in 150 MHz, $CDCl_3$ at 25 °C

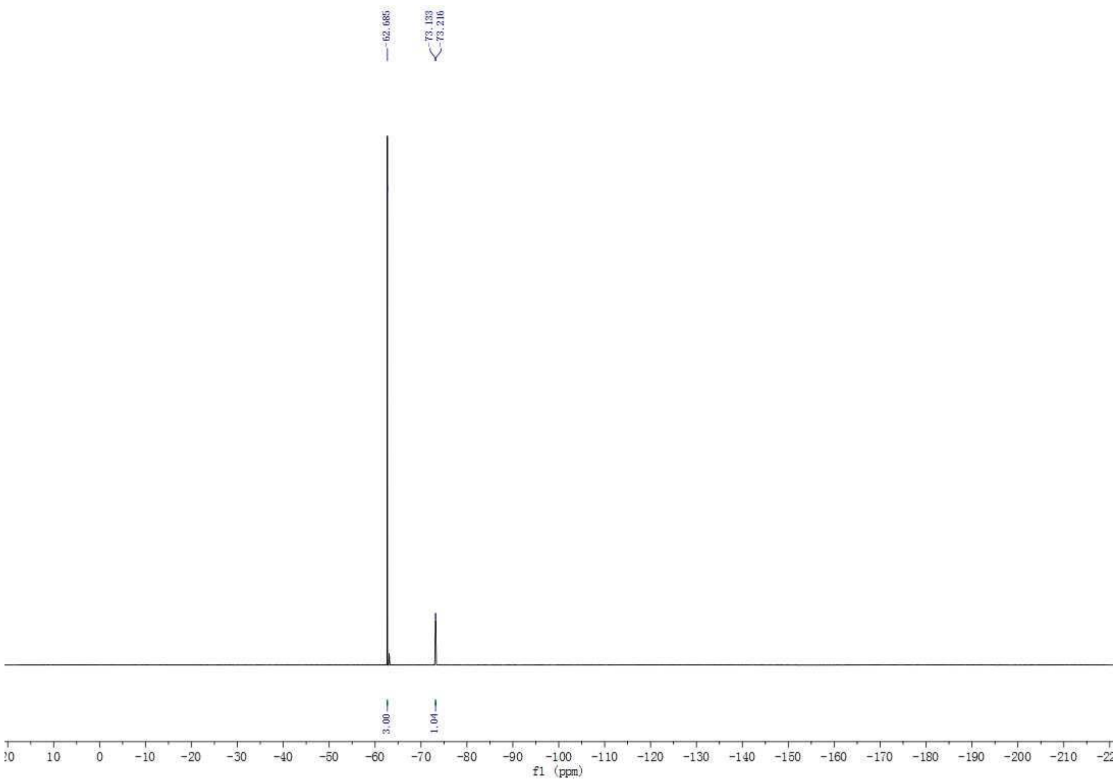

Supplementary Fig. 62. ^{19}F NMR of compound 2r. The sample has been recorded in 470 MHz, $CDCl_3$ at 25 °C

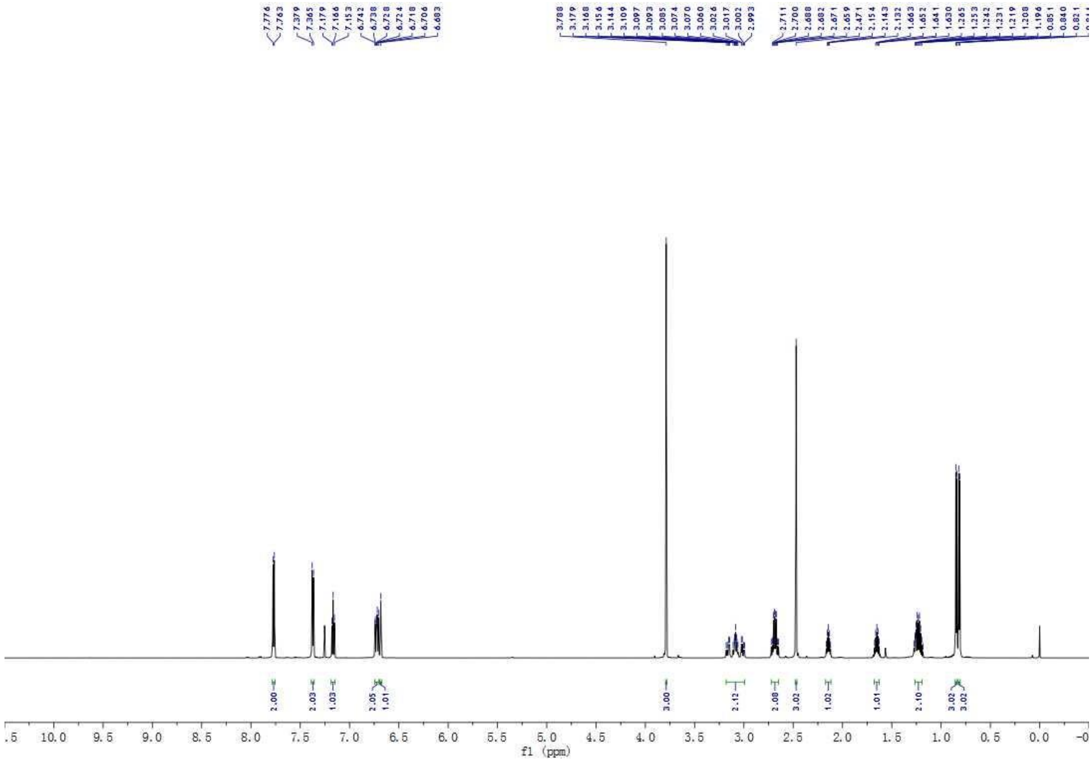

Supplementary Fig. 63. ^{1}H NMR of compound 2s. The sample has been recorded in 600 MHz, $CDCl_3$ at 25 °C

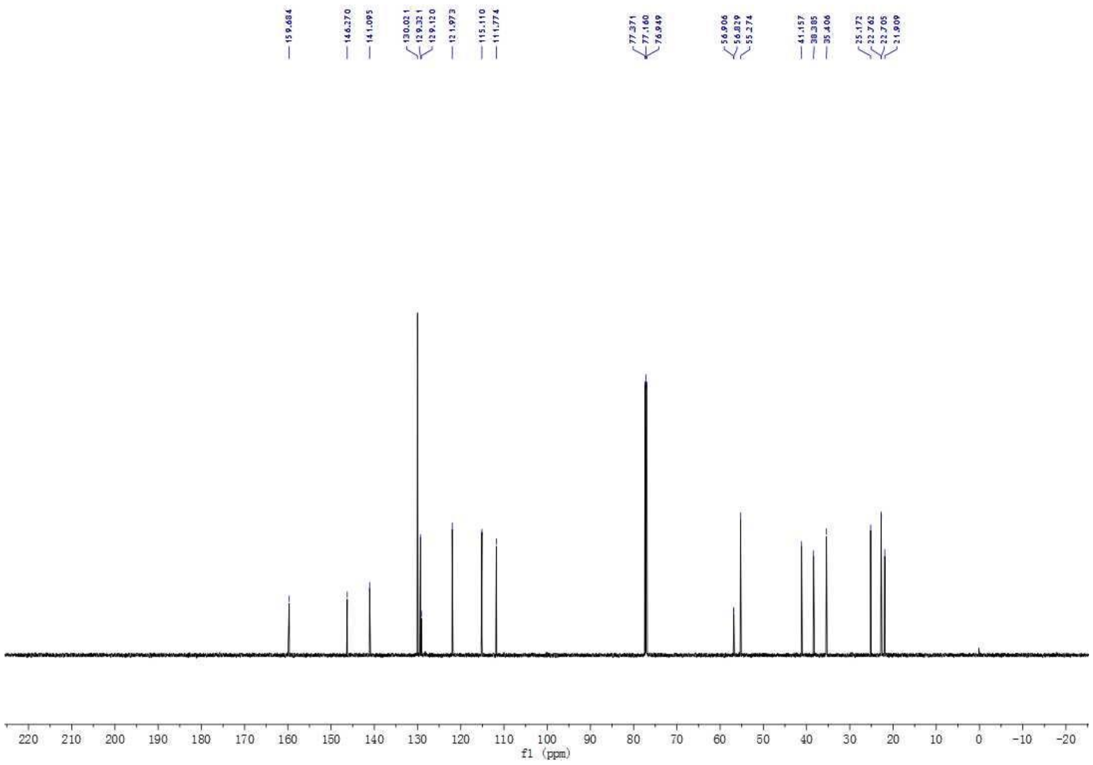

Supplementary Fig. 64. ^{13}C NMR of compound 2s. The sample has been recorded in 150 MHz, $CDCl_3$ at 25 °C

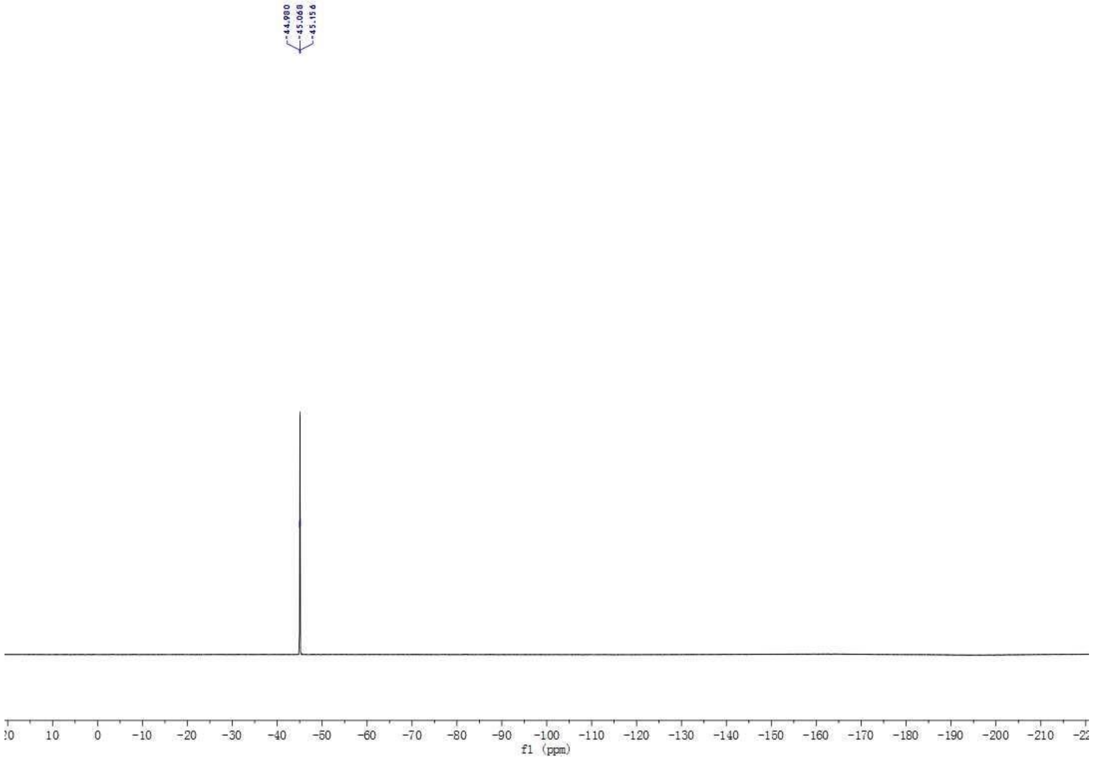

Supplementary Fig. 65. ^{19}F NMR of compound 2s. The sample has been recorded in 470 MHz, $CDCl_3$ at 25 °C

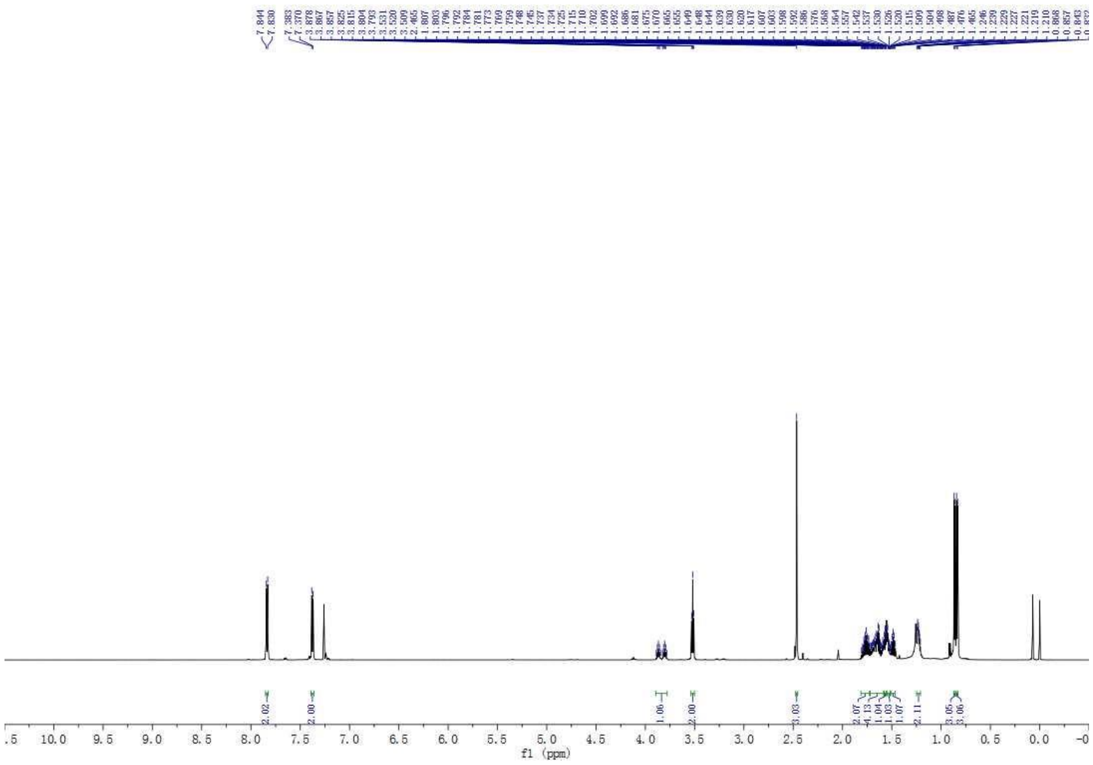

Supplementary Fig. 66. ^{1}H NMR of compound 2t. The sample has been recorded in 600 MHz, $CDCl_3$ at 25 °C

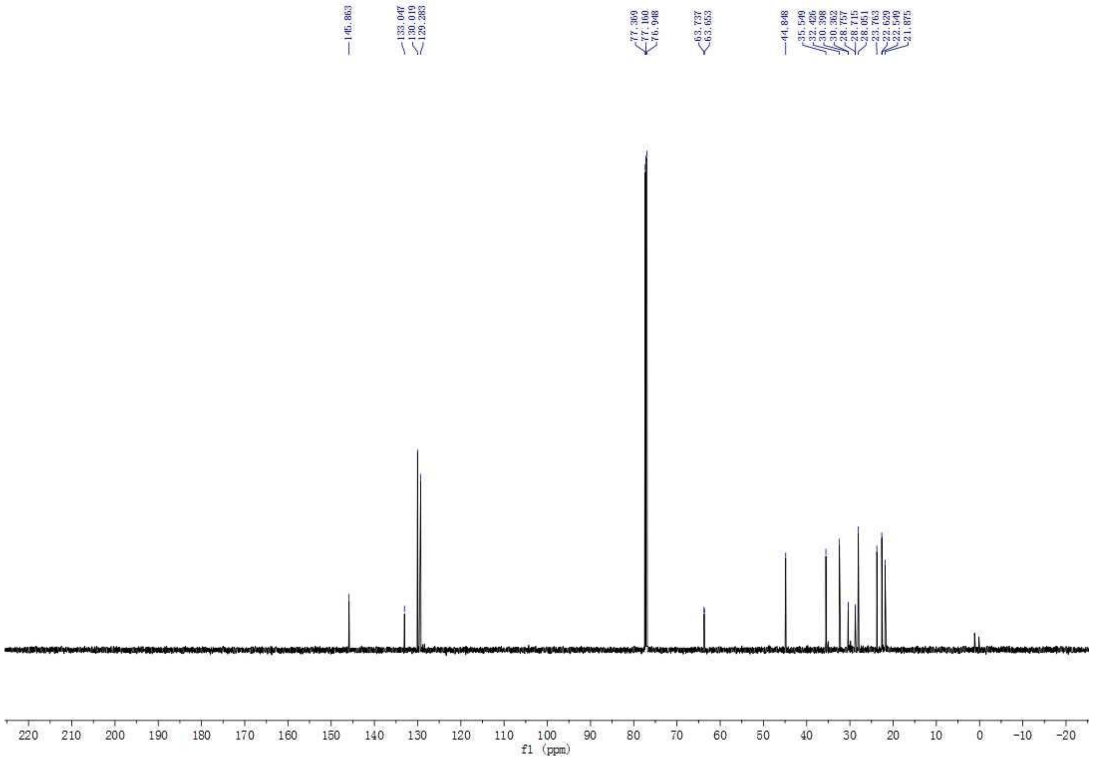

Supplementary Fig. 67. ^{13}C NMR of compound 2t. The sample has been recorded in 150 MHz, $CDCl_3$ at 25 °C

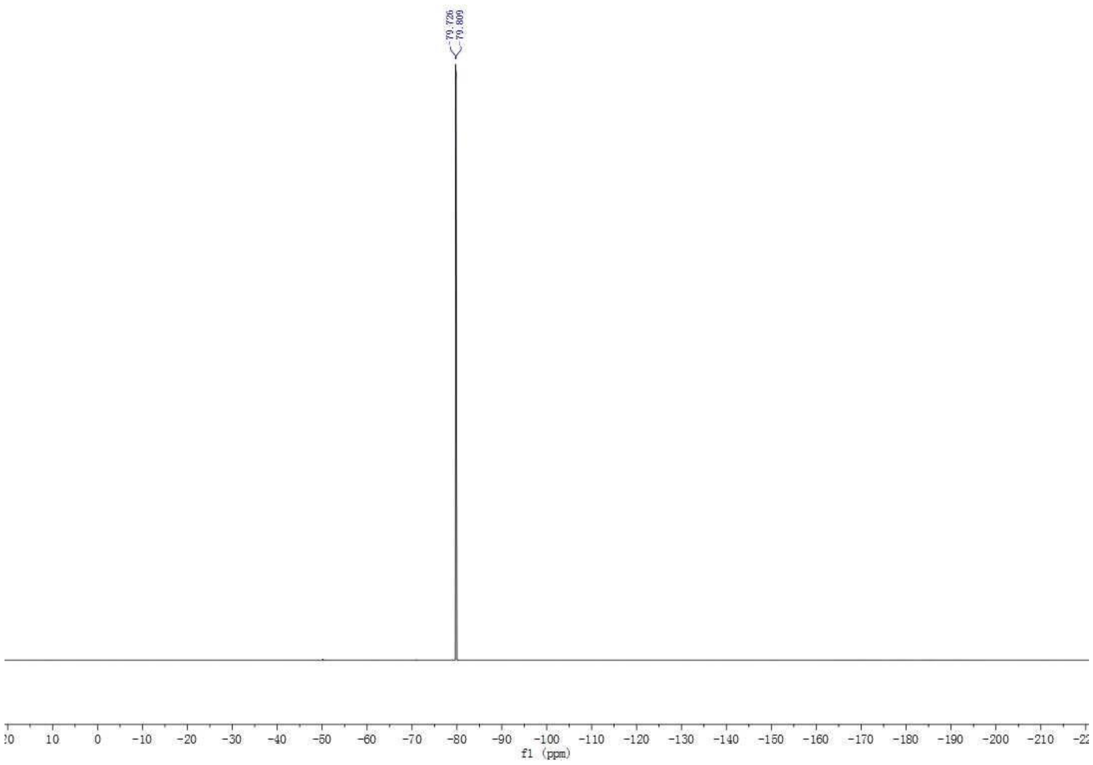

Supplementary Fig. 68. ^{19}F NMR of compound 2t. The sample has been recorded in 470 MHz, $CDCl_3$ at 25 °C

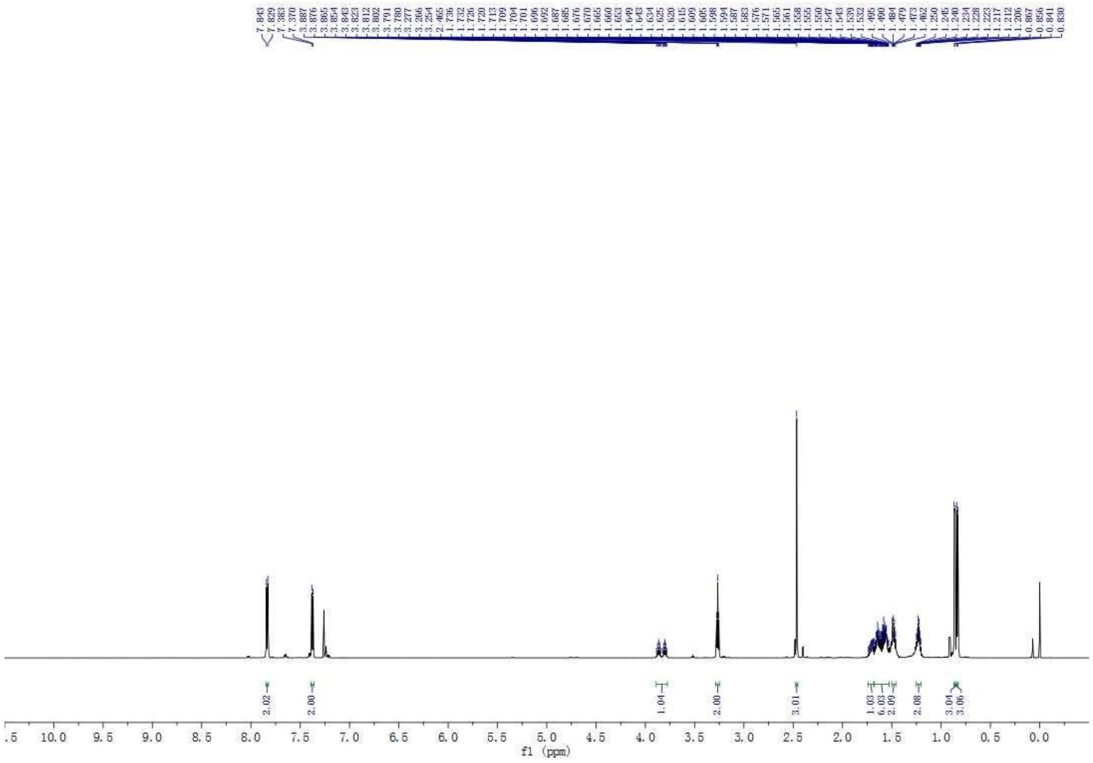

Supplementary Fig. 69. ^{1}H NMR of compound 2u. The sample has been recorded in 600 MHz, $CDCl_3$ at 25 °C

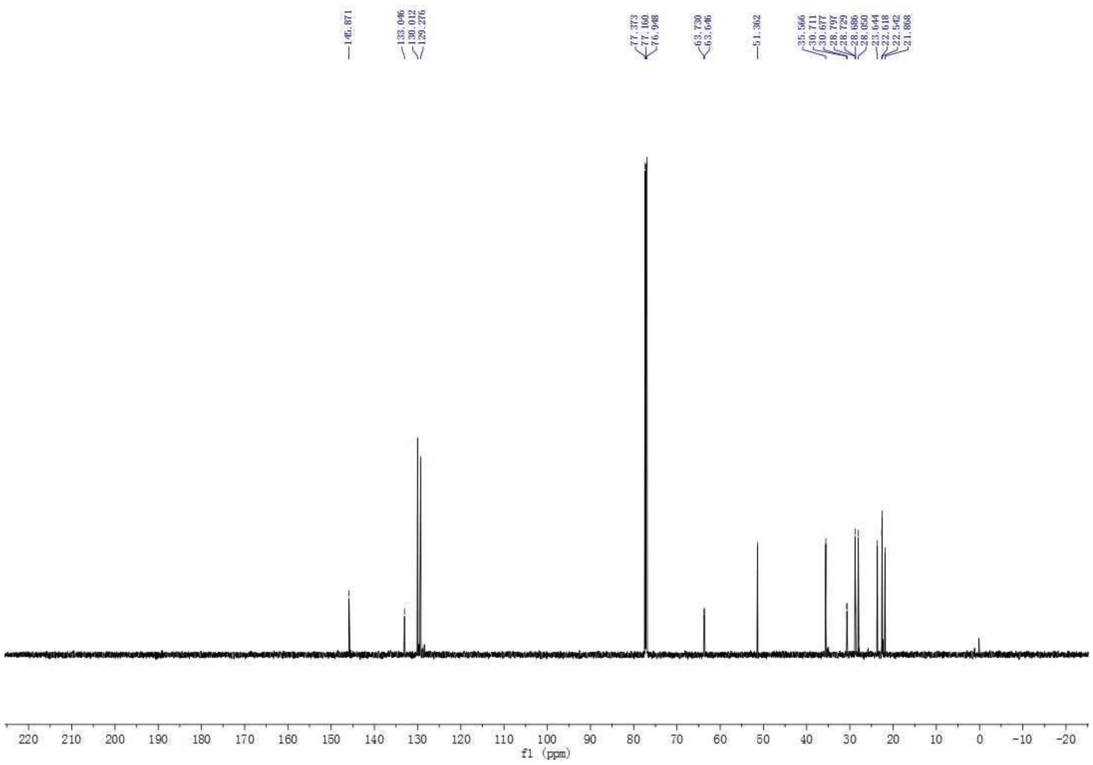

Supplementary Fig. 70. ^{13}C NMR of compound 2u. The sample has been recorded in 150 MHz, $CDCl_3$ at 25 °C

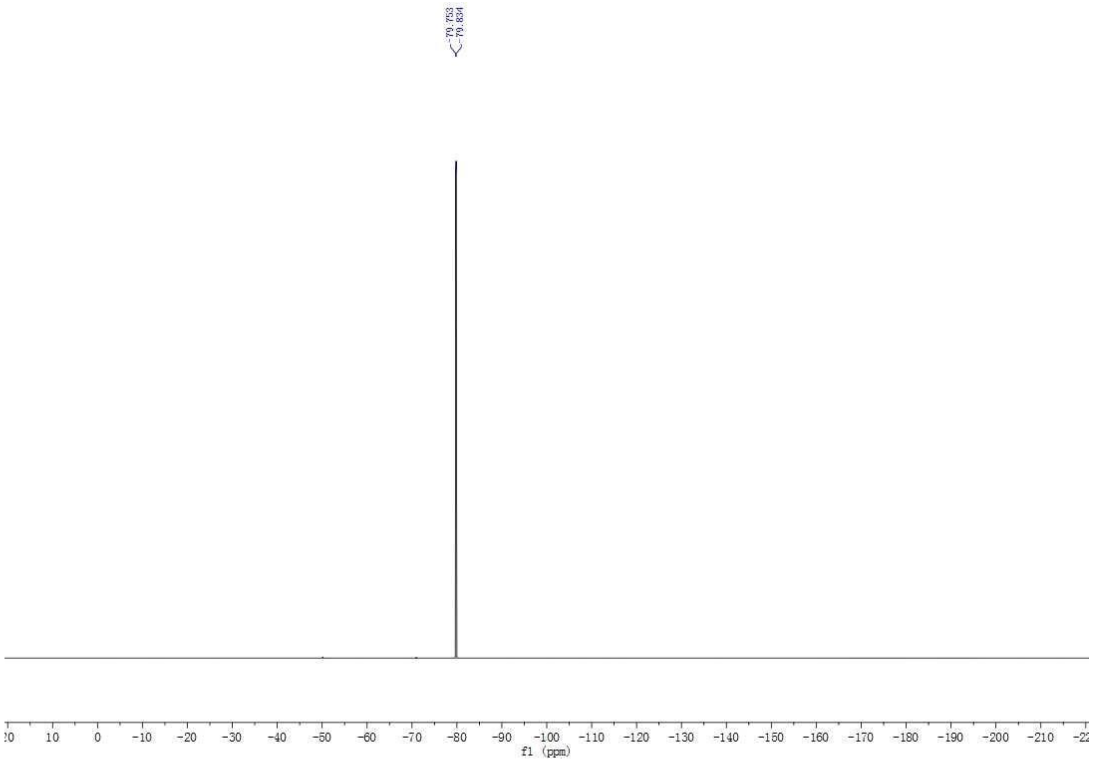

Supplementary Fig. 71. ^{19}F NMR of compound 2u. The sample has been recorded in 470 MHz, $CDCl_3$ at 25 °C

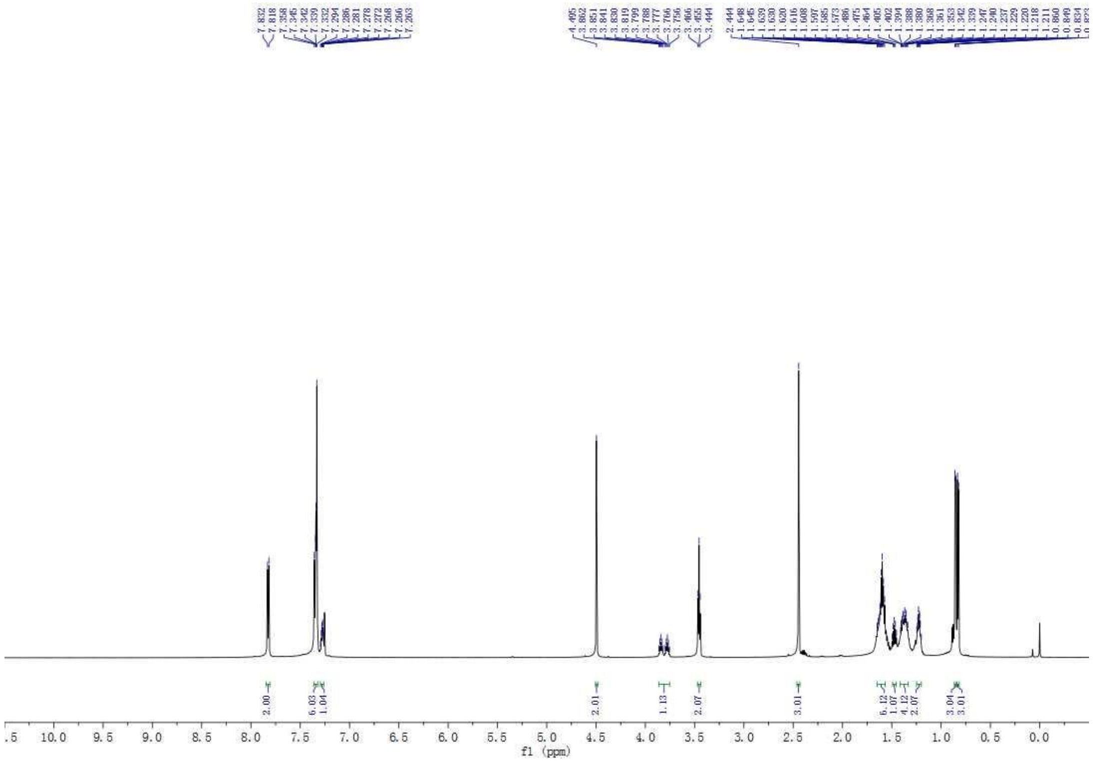

Supplementary Fig. 72. ^{1}H NMR of compound 2v. The sample has been recorded in 600 MHz, $CDCl_3$ at 25 °C

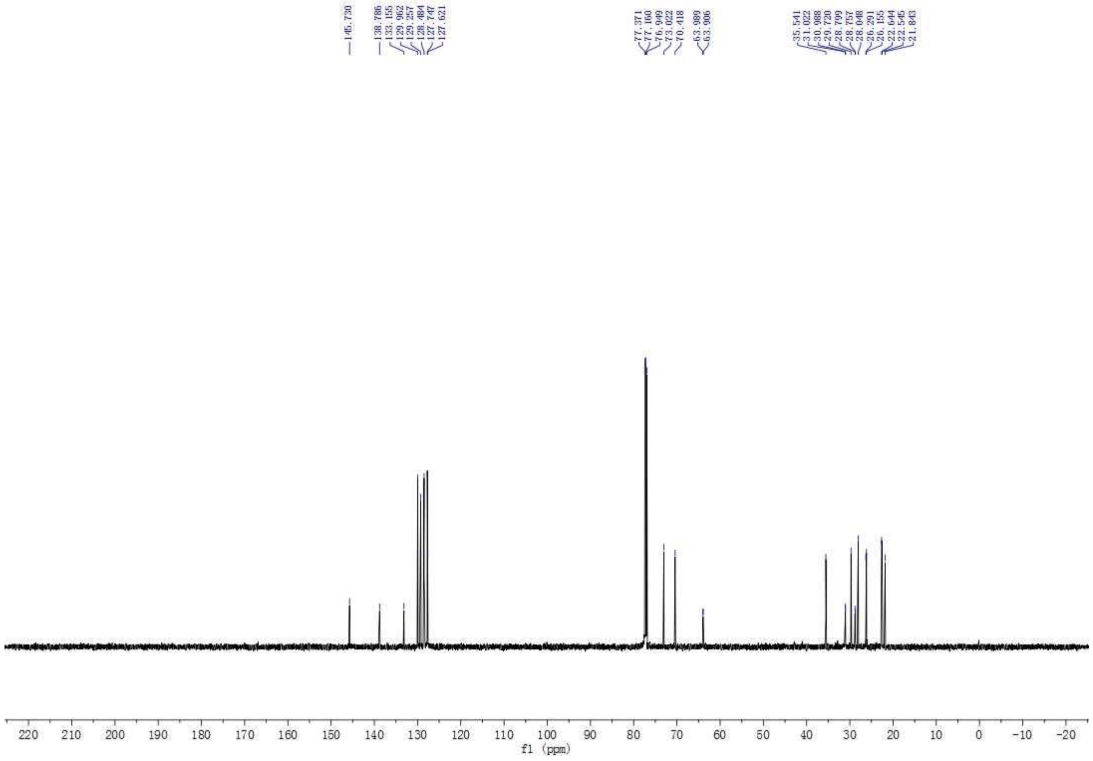

Supplementary Fig. 73. ^{13}C NMR of compound 2v. The sample has been recorded in 150 MHz, $CDCl_3$ at 25 °C

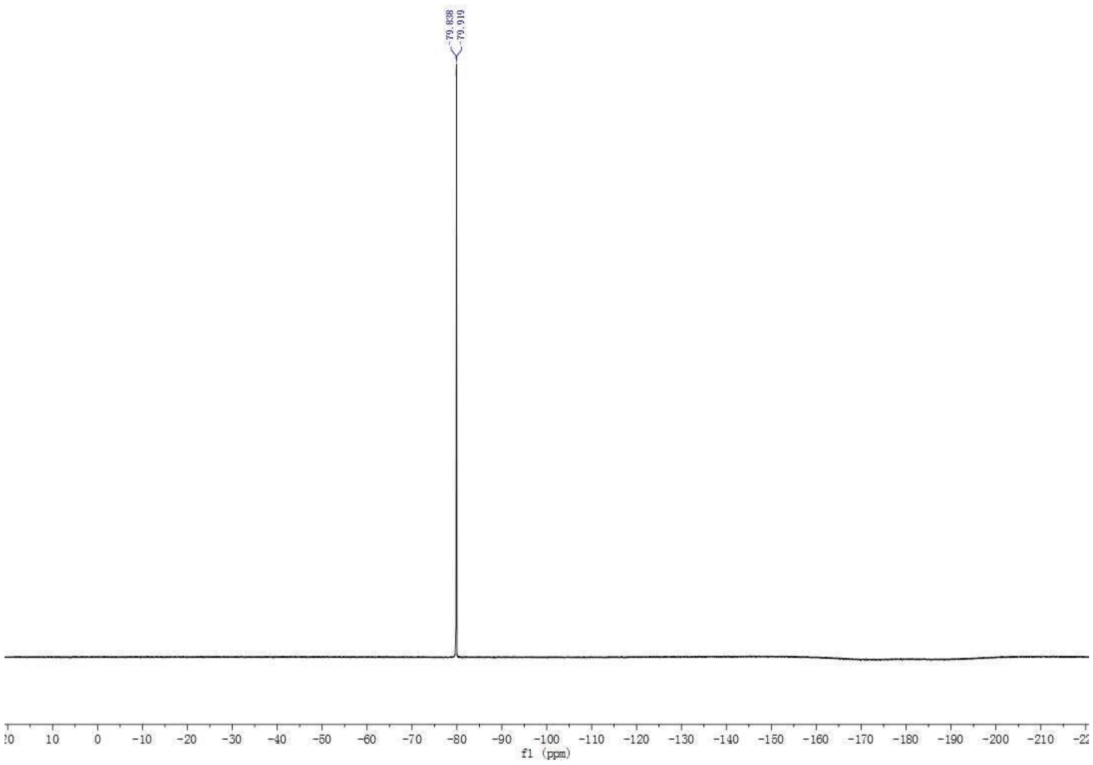

Supplementary Fig. 74. ^{19}F NMR of compound 2v. The sample has been recorded in 470 MHz, $CDCl_3$ at 25 °C

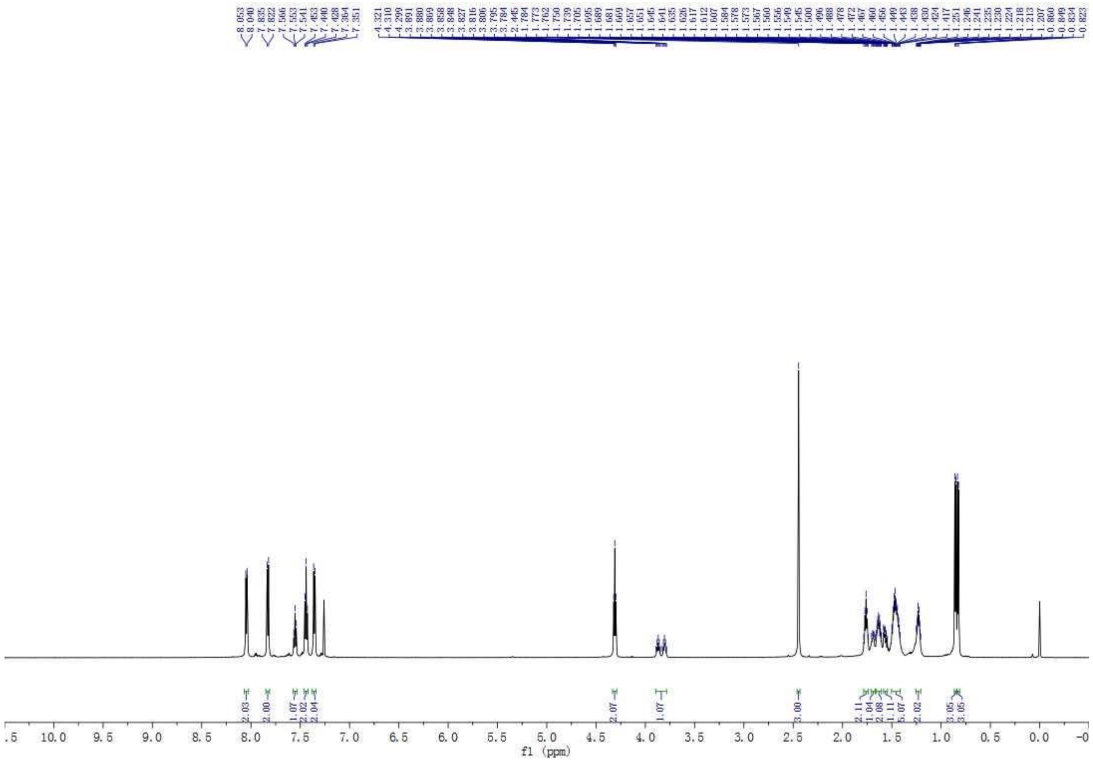

Supplementary Fig. 75. ^{1}H NMR of compound 2w. The sample has been recorded in 600 MHz, $CDCl_3$ at 25 °C

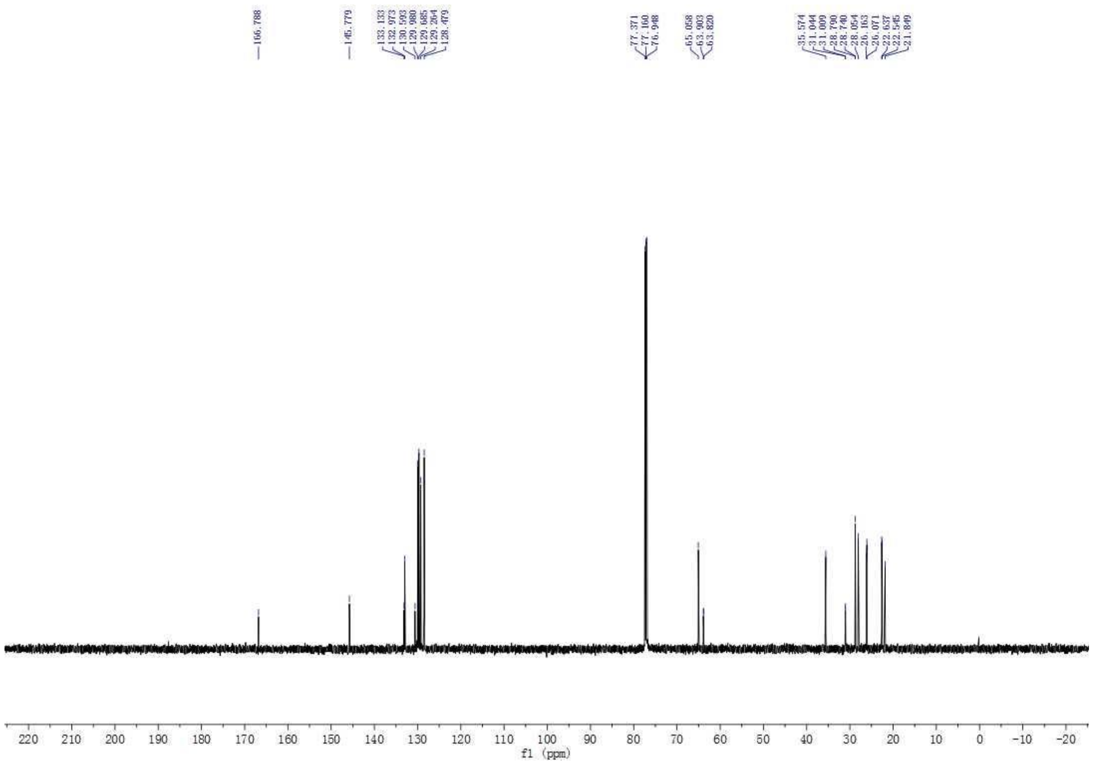

Supplementary Fig. 76. ^{13}C NMR of compound 2w. The sample has been recorded in 150 MHz, $CDCl_3$ at 25 °C

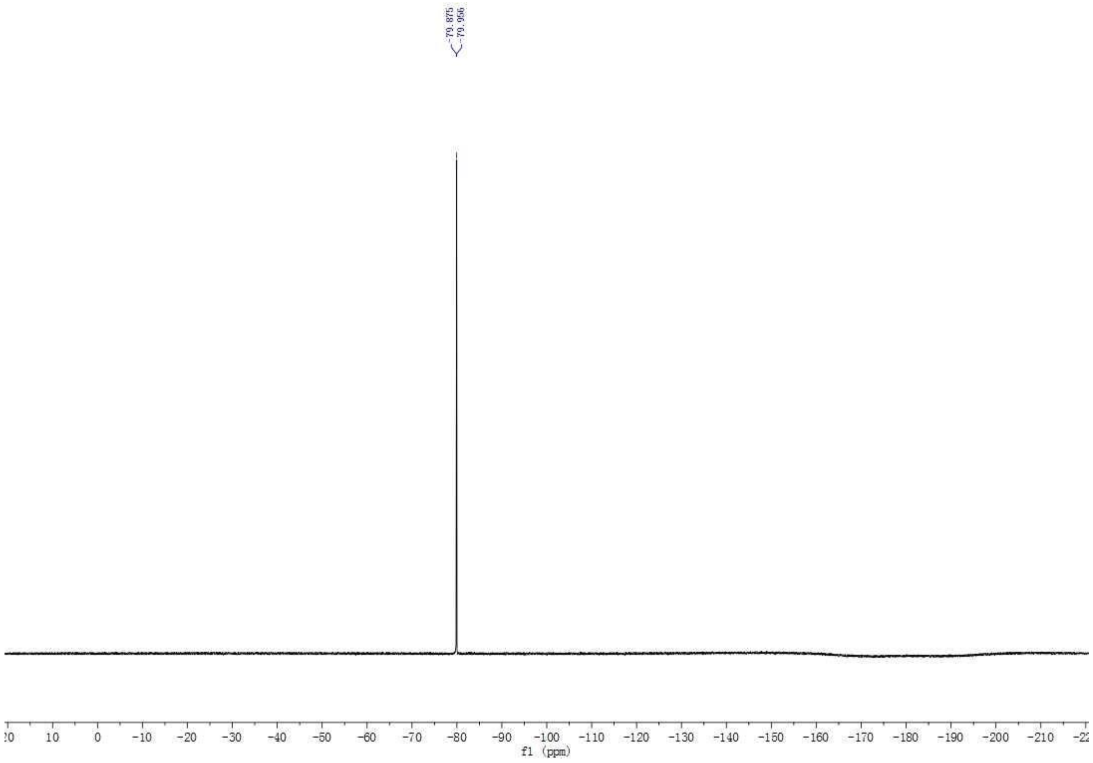

Supplementary Fig. 77. ^{19}F NMR of compound 2w. The sample has been recorded in 470 MHz, $CDCl_3$ at 25 °C

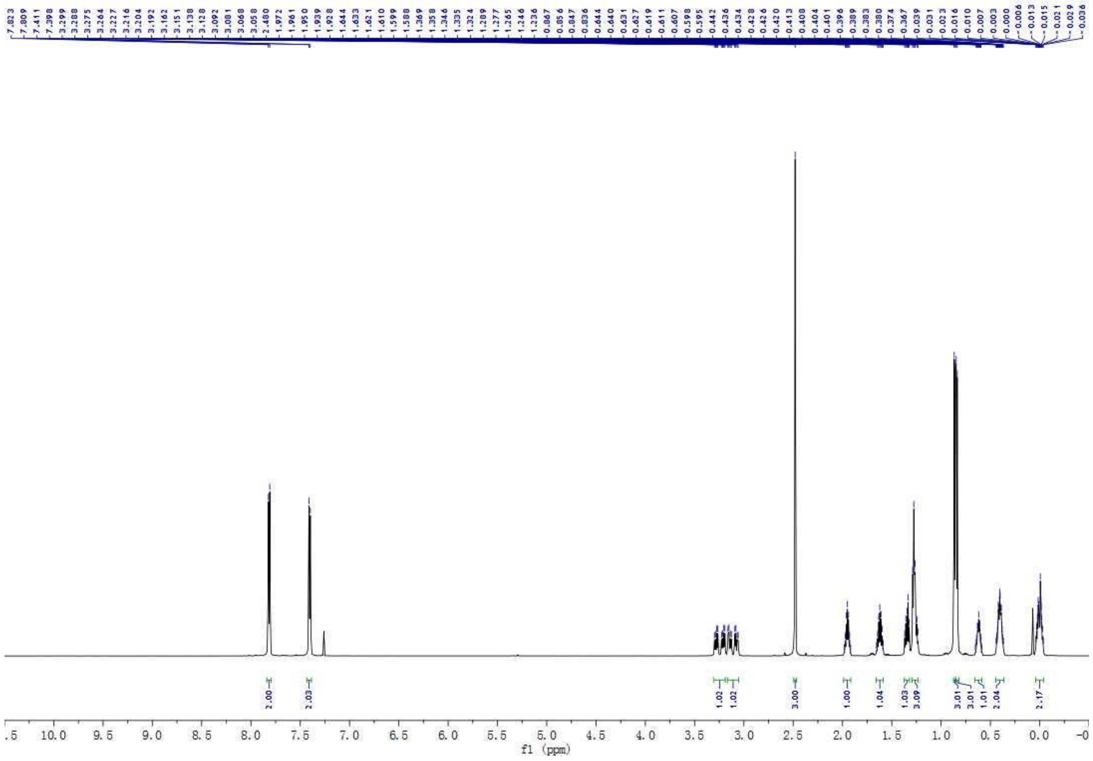

Supplementary Fig. 78. ^{1}H NMR of compound 2y. The sample has been recorded in 600 MHz, $CDCl_3$ at 25 °C

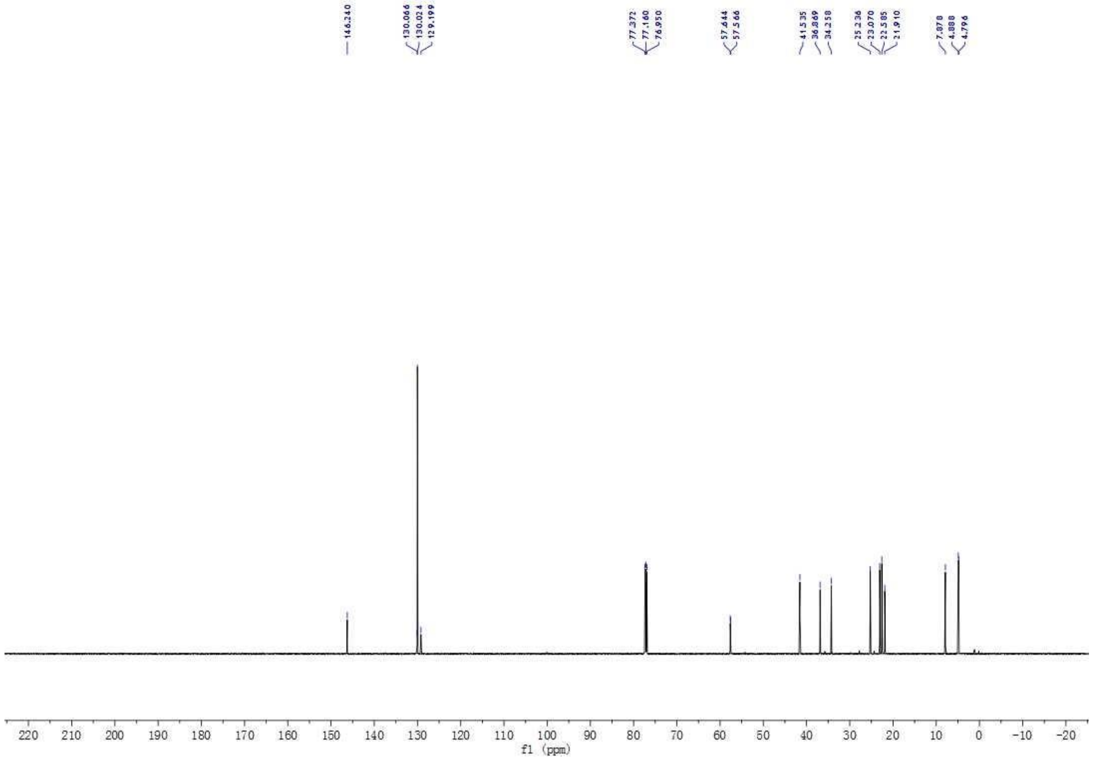

Supplementary Fig. 79. ^{13}C NMR of compound 2y. The sample has been recorded in 150 MHz, $CDCl_3$ at 25 °C

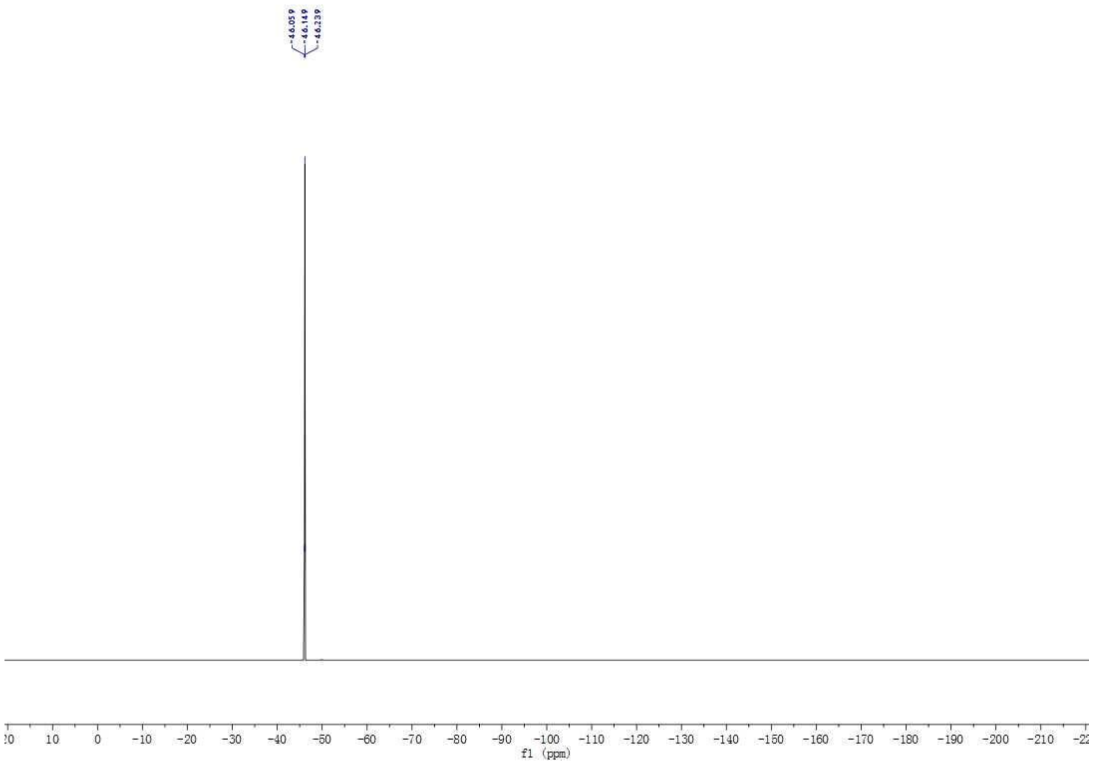

Supplementary Fig. 80. ^{19}F NMR of compound 2y. The sample has been recorded in 470 MHz, $CDCl_3$ at 25 °C

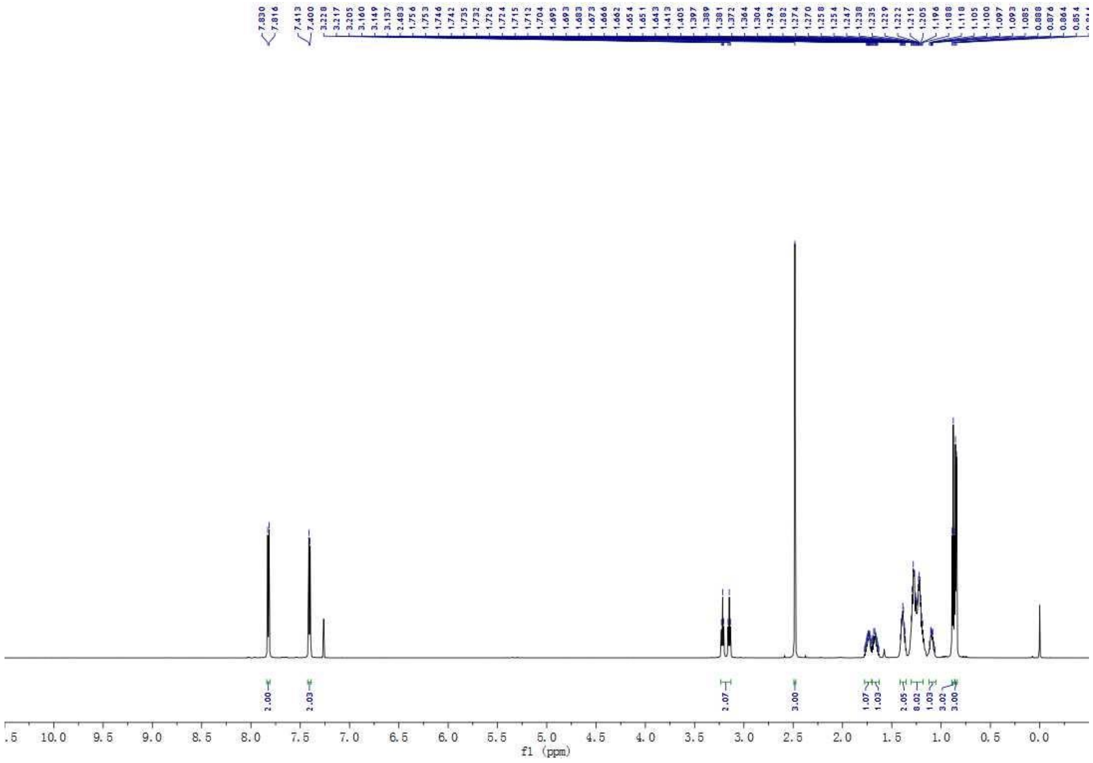

Supplementary Fig. 81. ^{1}H NMR of compound 2z. The sample has been recorded in 600 MHz, $CDCl_3$ at 25 °C

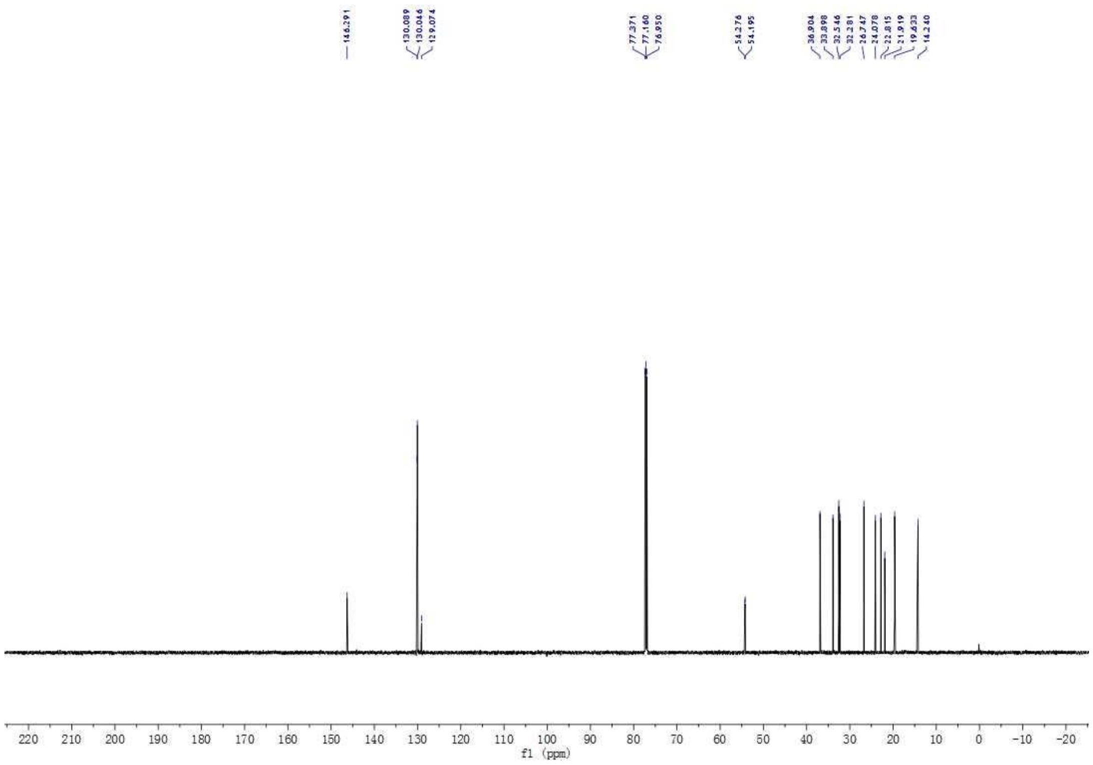

Supplementary Fig. 82. ^{13}C NMR of compound 2z. The sample has been recorded in 150 MHz, $CDCl_3$ at 25 °C

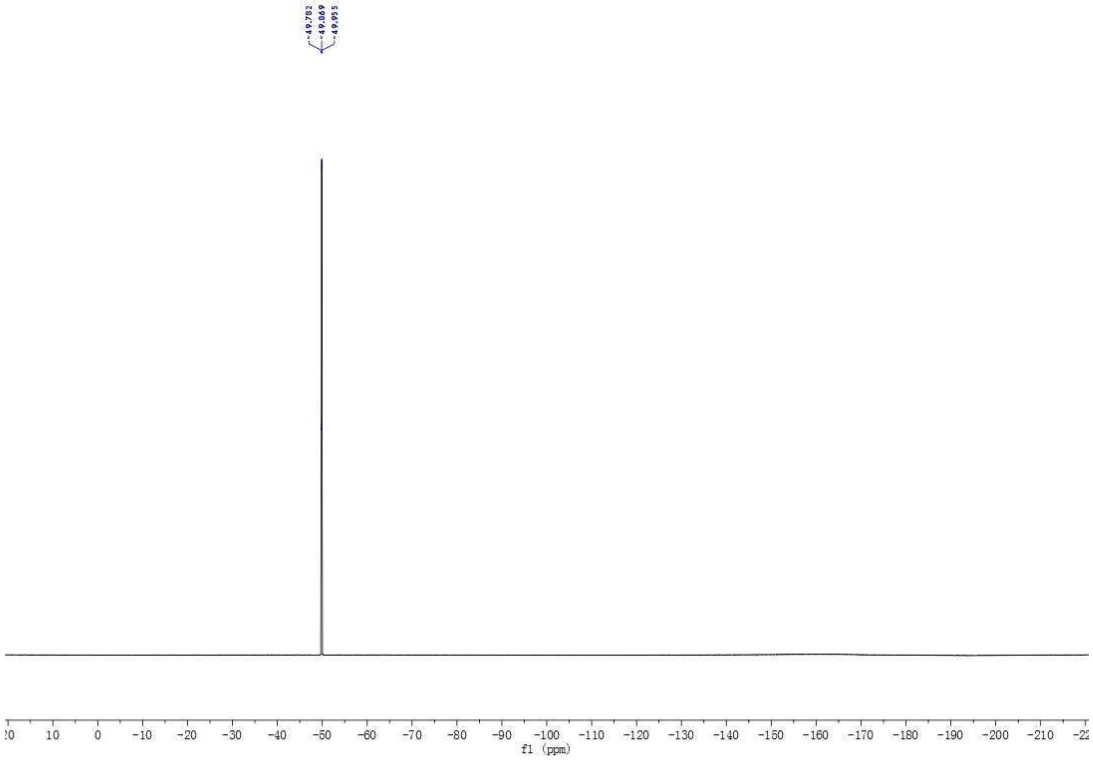

Supplementary Fig. 83. ^{19}F NMR of compound 2z. The sample has been recorded in 470 MHz, $CDCl_3$ at 25 °C

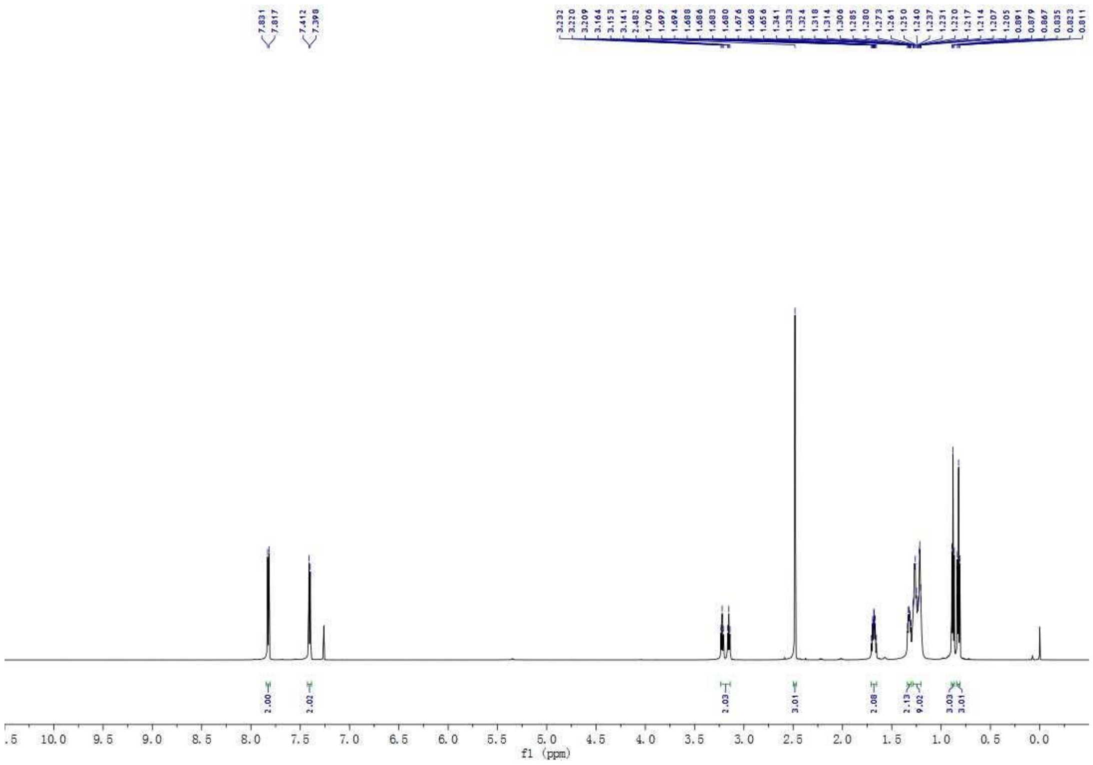

Supplementary Fig. 84. ^{1}H NMR of compound 2aa. The sample has been recorded in 600 MHz, $CDCl_3$ at 25 °C

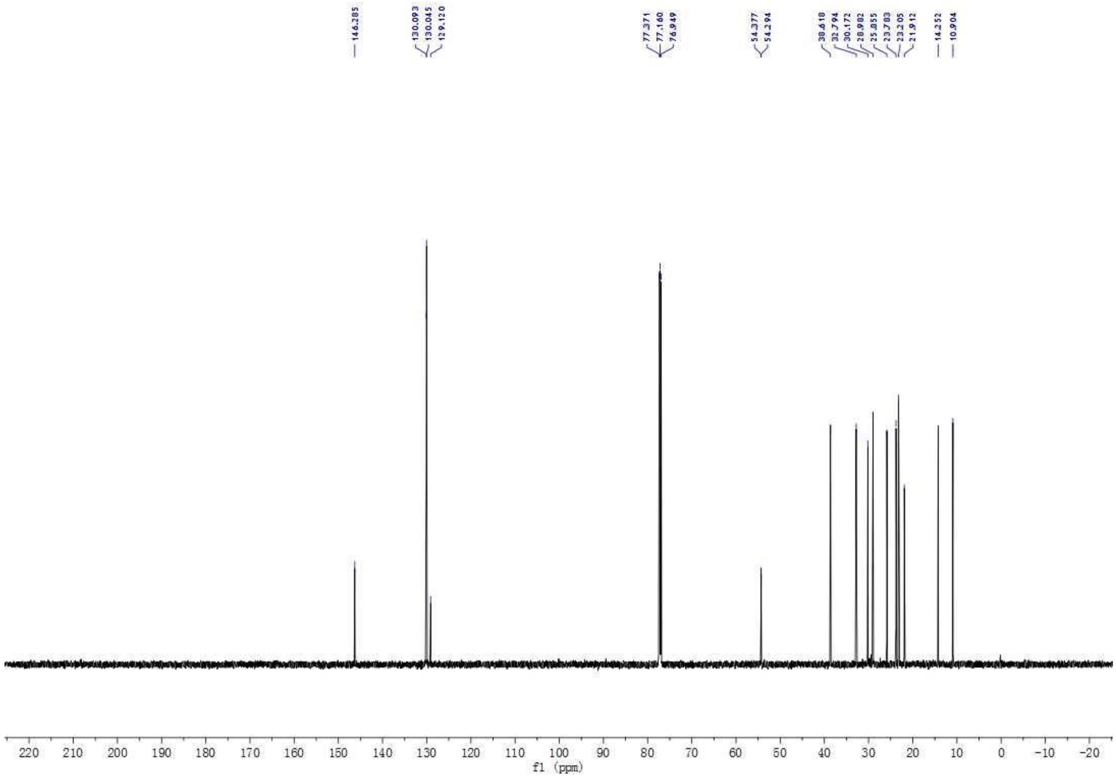

Supplementary Fig. 85. ^{13}C NMR of compound 2aa. The sample has been recorded in 150 MHz, $CDCl_3$ at 25 °C

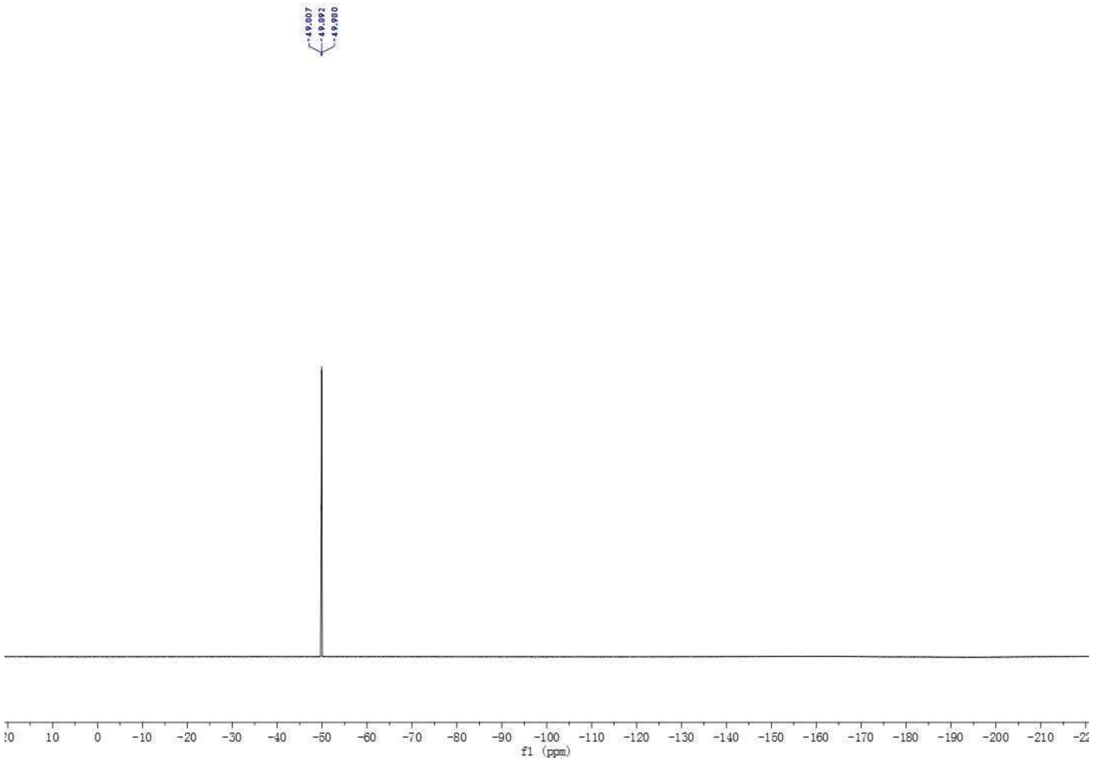

Supplementary Fig. 86. ^{19}F NMR of compound 2aa. The sample has been recorded in 470 MHz, $CDCl_3$ at 25 °C

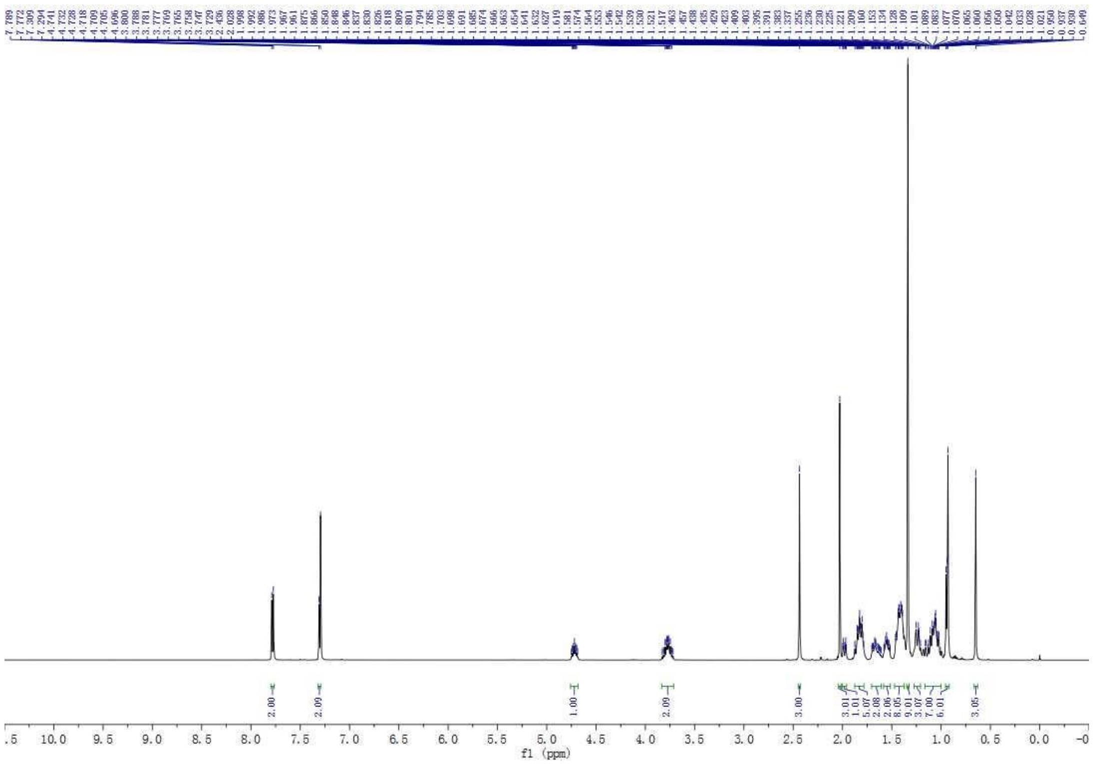

Supplementary Fig. 87. ^{1}H NMR of compound S11. The sample has been recorded in 500 MHz, $CDCl_3$ at 25 °C

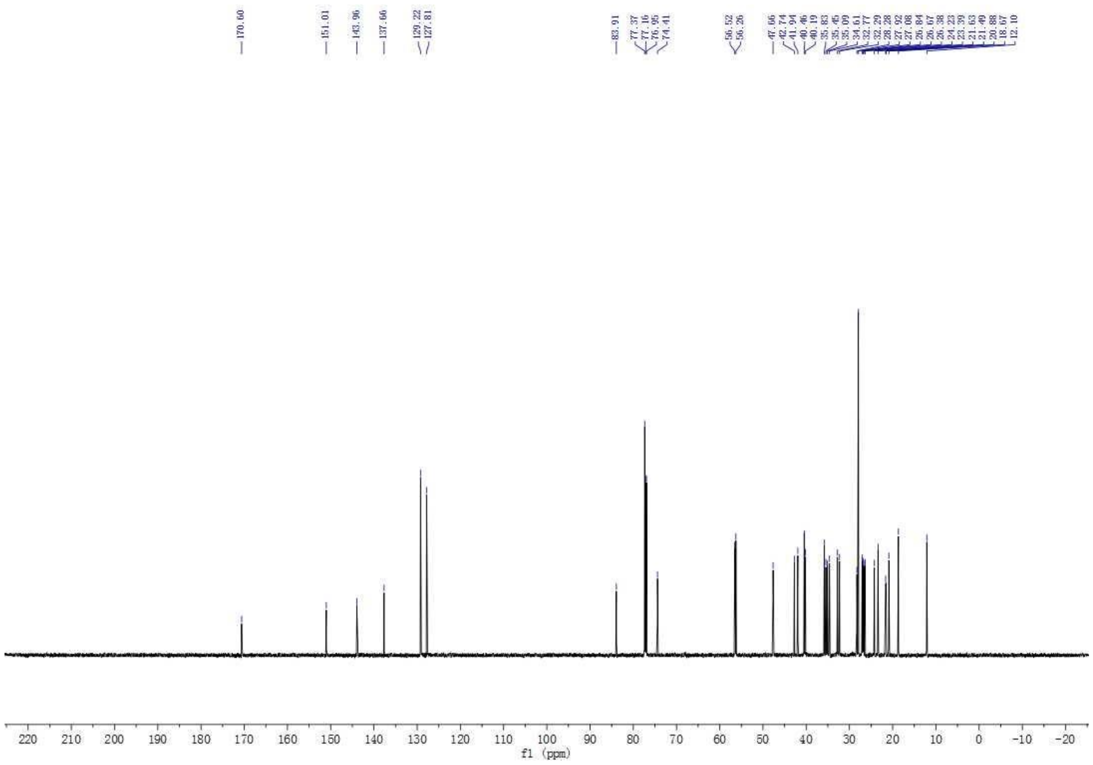

Supplementary Fig. 88. ^{13}C NMR of compound S11. The sample has been recorded in 150 MHz, $CDCl_3$ at 25 °C

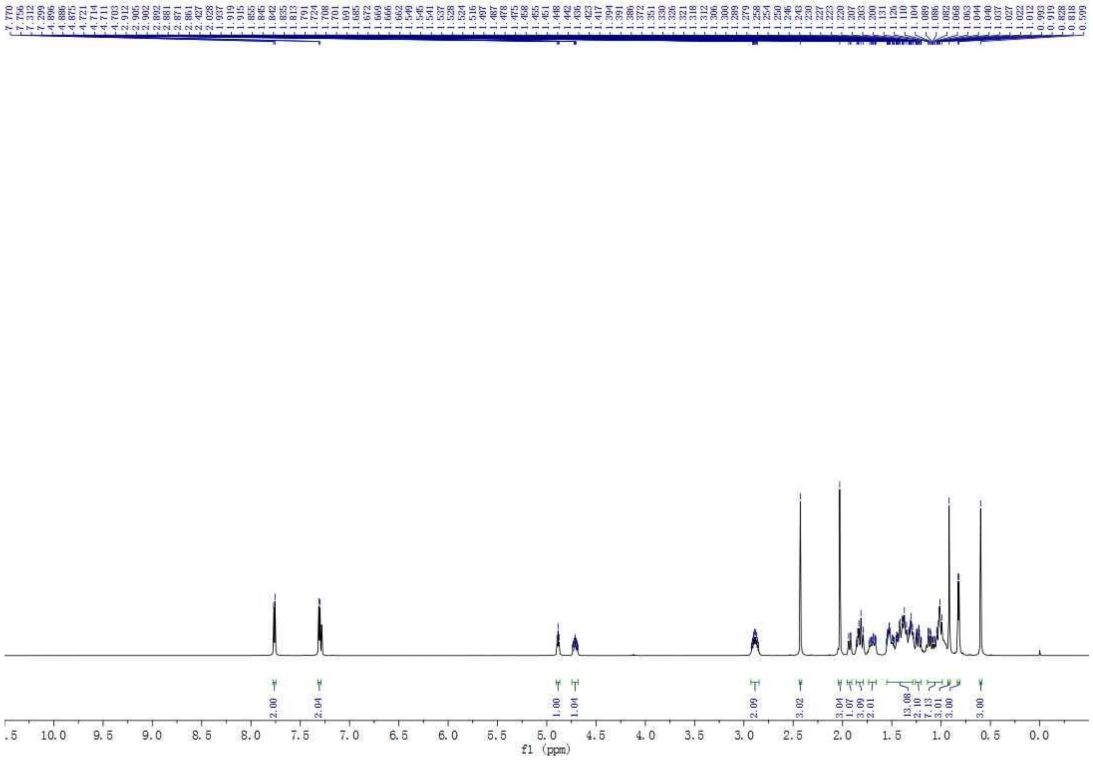

Supplementary Fig. 89. ^{1}H NMR of compound S12. The sample has been recorded in 600 MHz, $CDCl_3$ at 25 °C

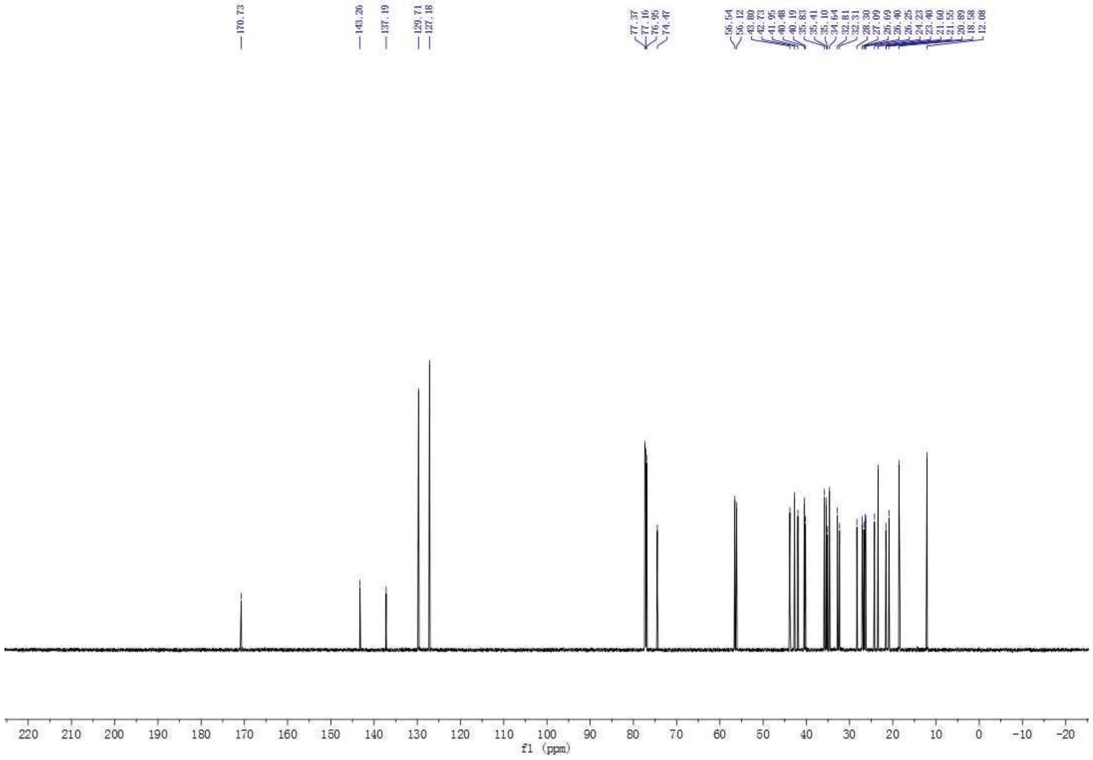

Supplementary Fig. 90. ^{13}C NMR of compound S12. The sample has been recorded in 150 MHz, $CDCl_3$ at 25 °C

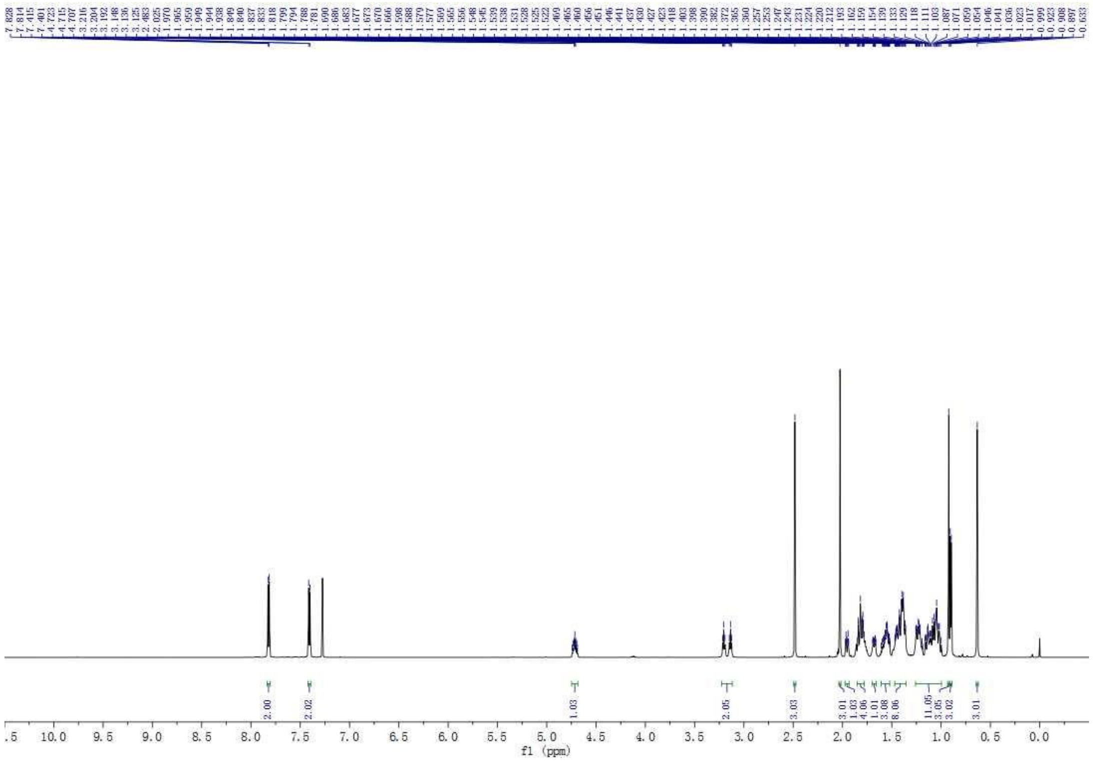

Supplementary Fig. 91. ^{1}H NMR of compound 2ac. The sample has been recorded in 600 MHz, $CDCl_3$ at 25 °C

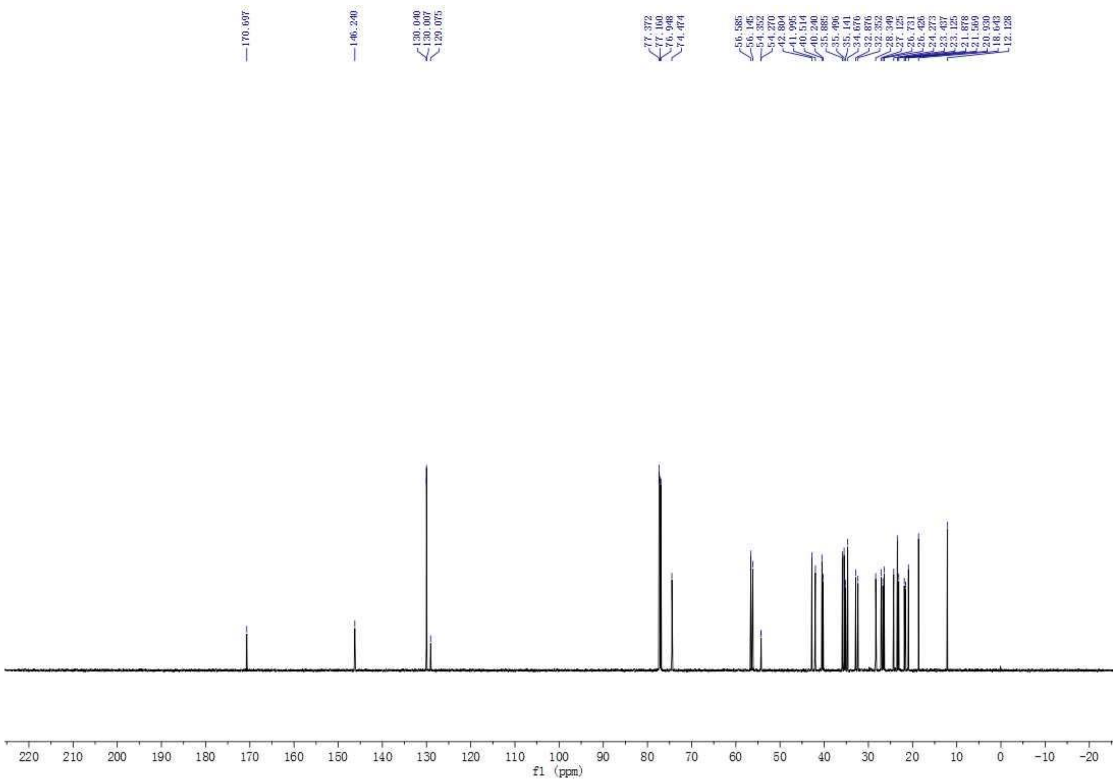

Supplementary Fig. 92. ^{13}C NMR of compound 2ac. The sample has been recorded in 150 MHz, $CDCl_3$ at 25 °C

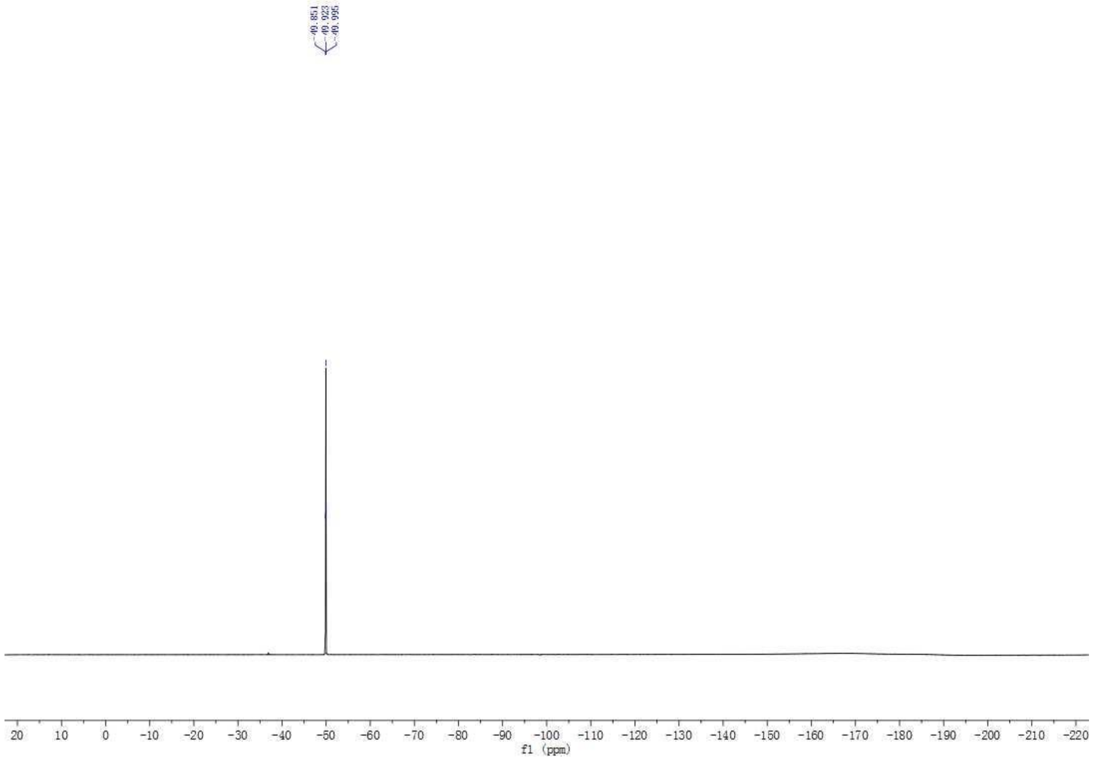

Supplementary Fig. 93. ^{19}F NMR of compound 2ac. The sample has been recorded in 565 MHz, $CDCl_3$ at 25 °C

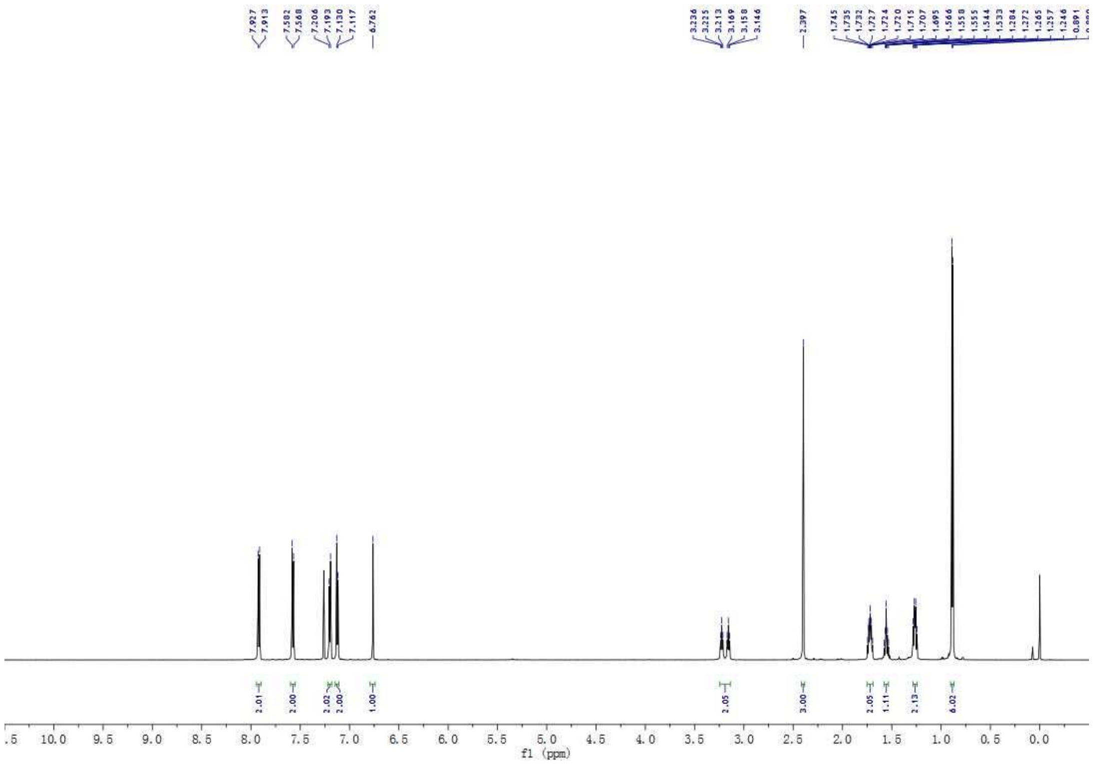

Supplementary Fig. 94. ^{1}H NMR of compound 2ad. The sample has been recorded in 600 MHz, $CDCl_3$ at 25 °C

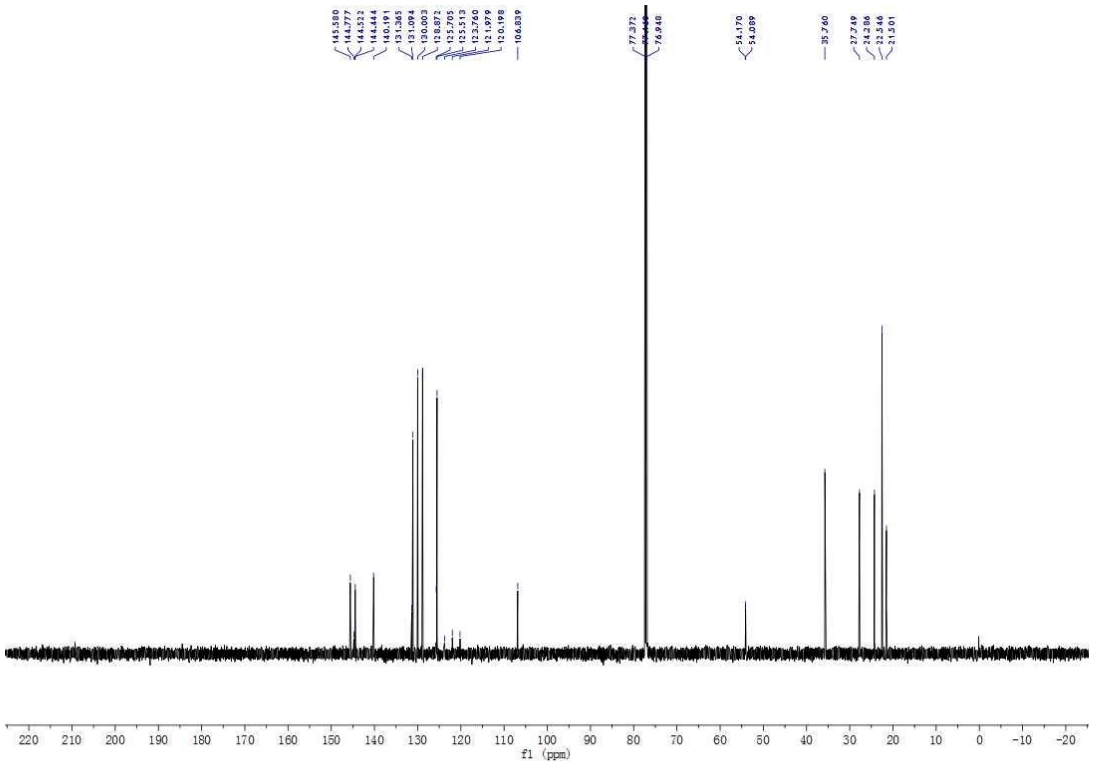

Supplementary Fig. 95. ^{13}C NMR of compound 2ad. The sample has been recorded in 150 MHz, $CDCl_3$ at 25 °C

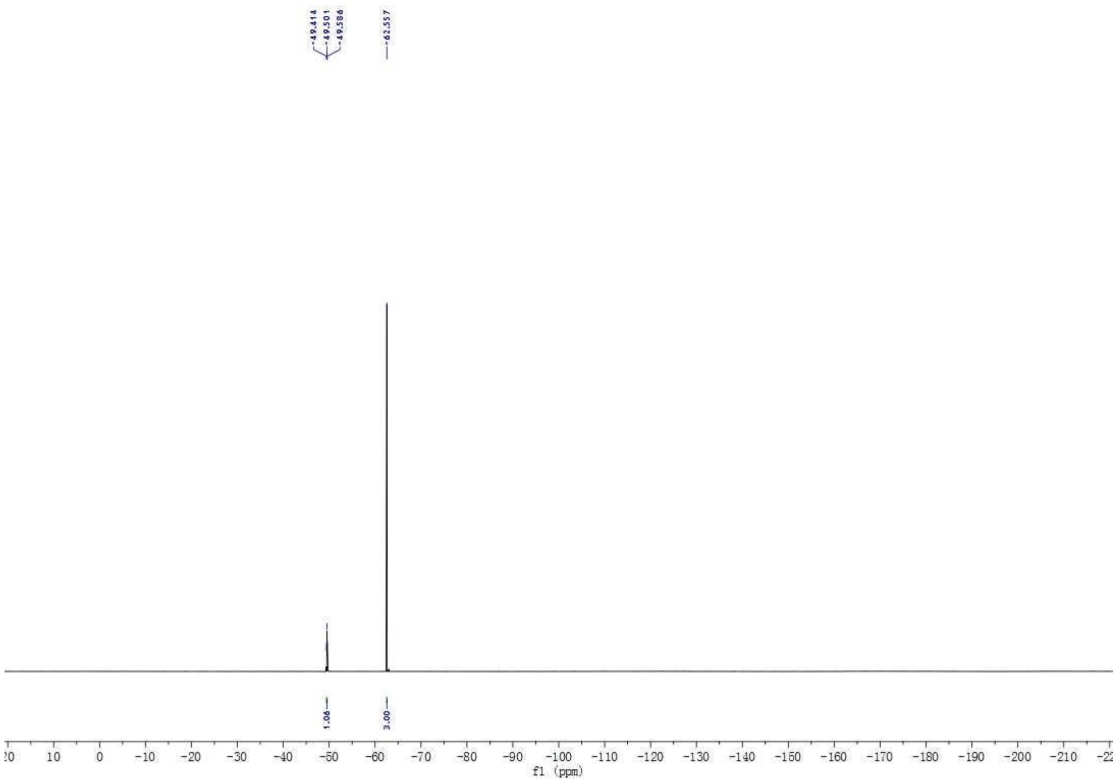

Supplementary Fig. 96. ^{19}F NMR of compound 2ad. The sample has been recorded in 470 MHz, $CDCl_3$ at 25 °C

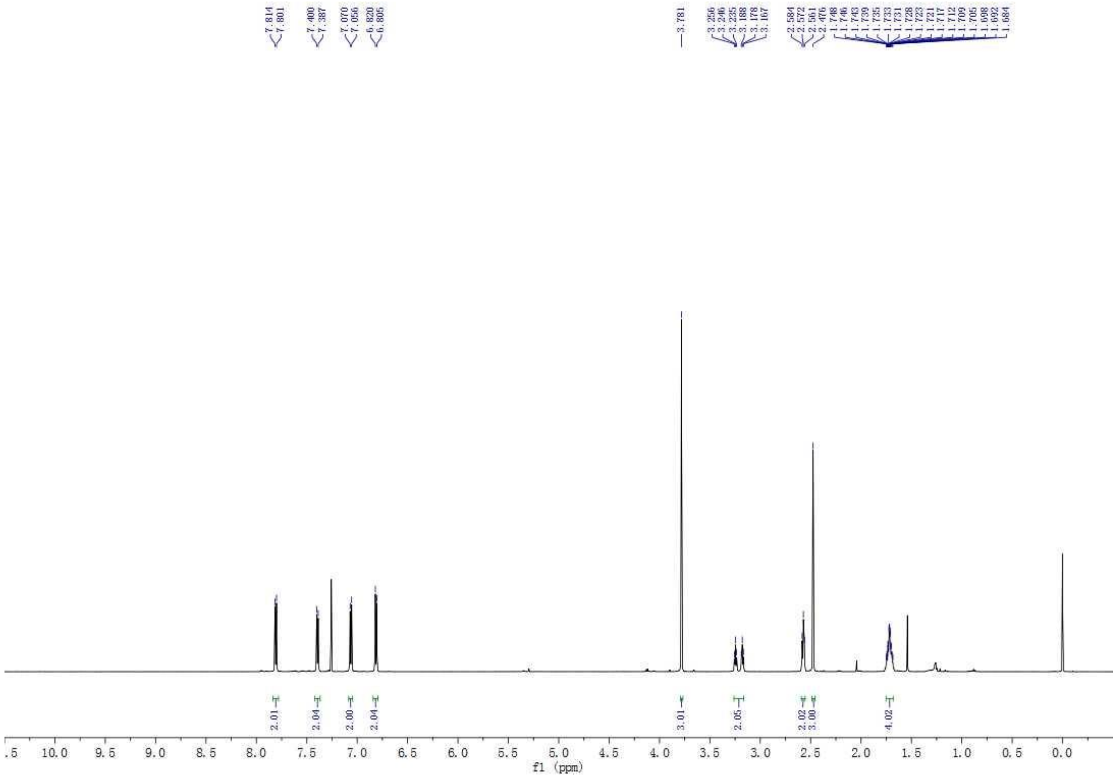

Supplementary Fig. 97. ^{1}H NMR of compound 2an. The sample has been recorded in 600 MHz, $CDCl_3$ at 25 °C

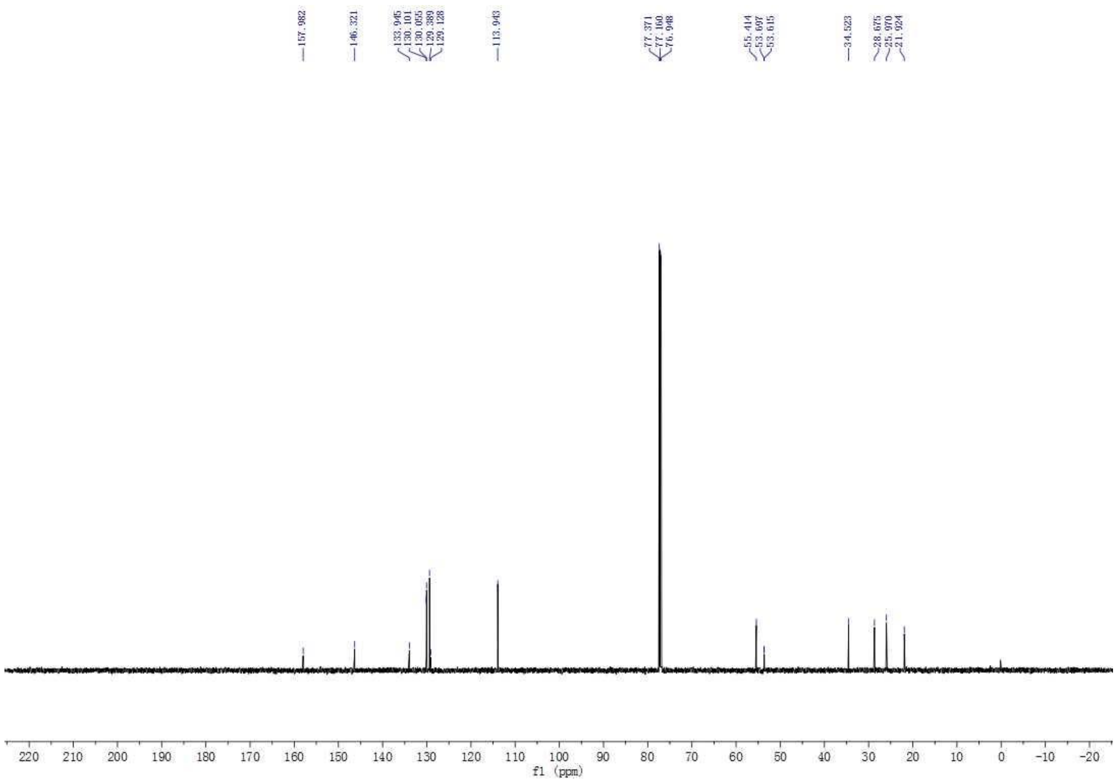

Supplementary Fig. 98. ^{13}C NMR of compound 2an. The sample has been recorded in 150 MHz, $CDCl_3$ at 25 °C

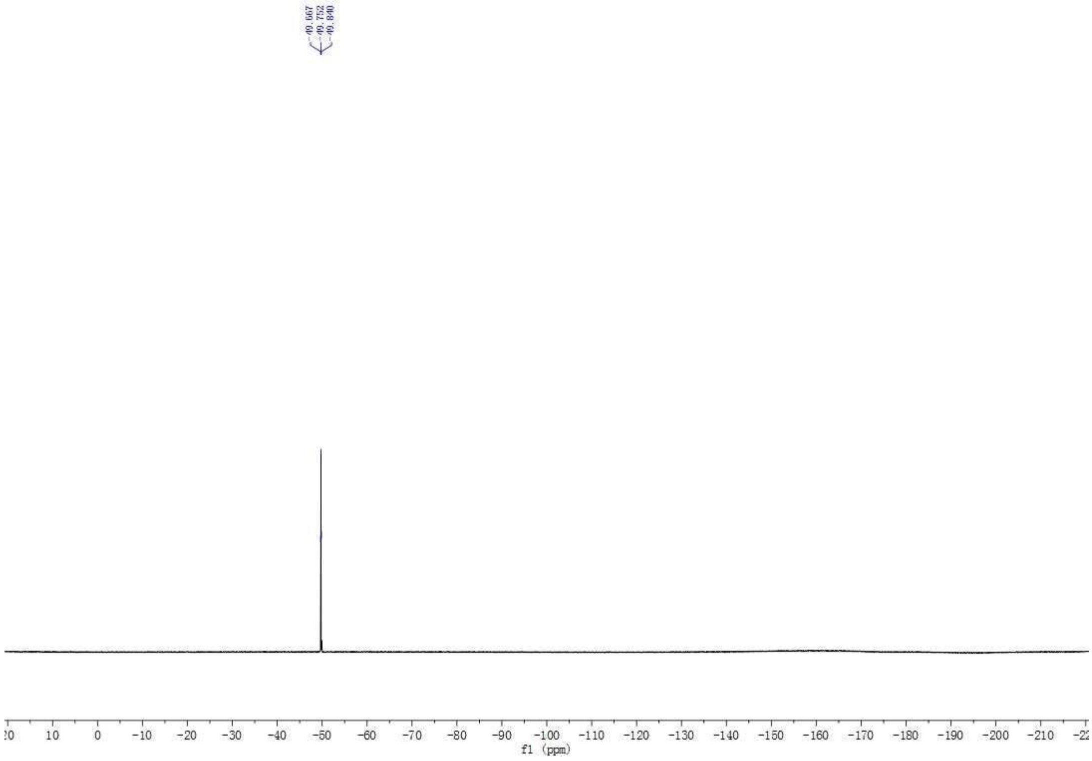

Supplementary Fig. 99. ^{19}F NMR of compound 2an. The sample has been recorded in 470 MHz, $CDCl_3$ at 25 °C

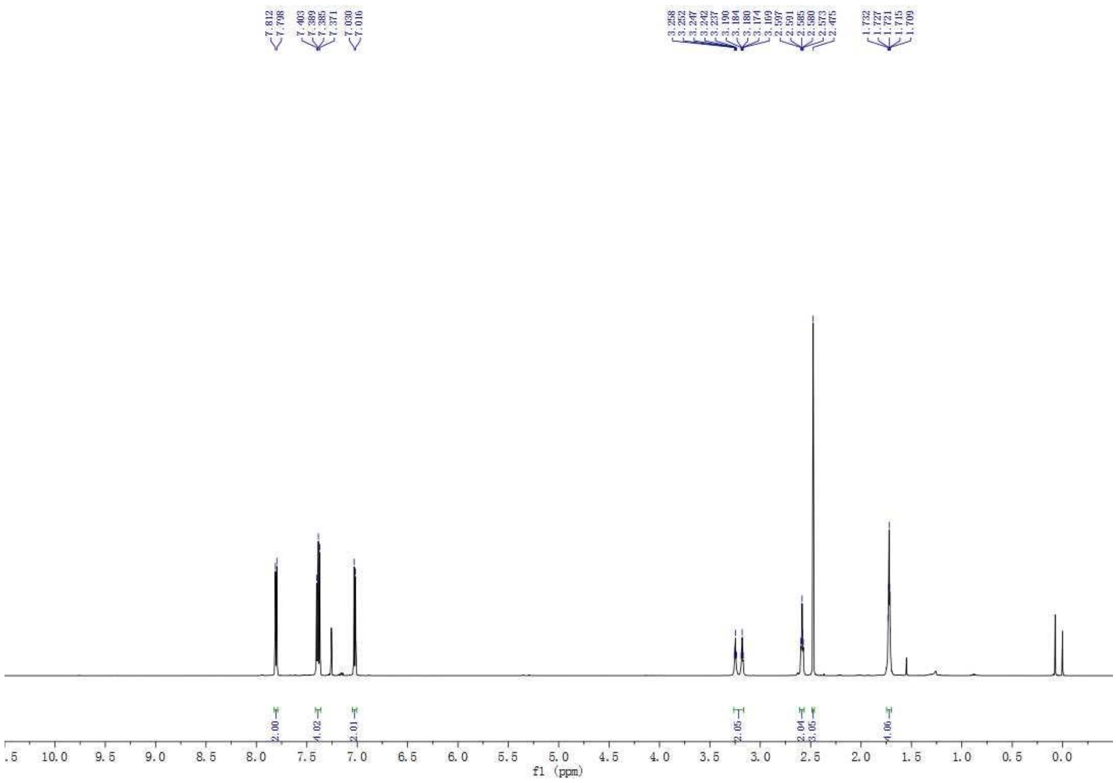

Supplementary Fig. 100. ¹H NMR of compound 2ao. The sample has been recorded in 600 MHz, $CDCl_3$ at 25 °C

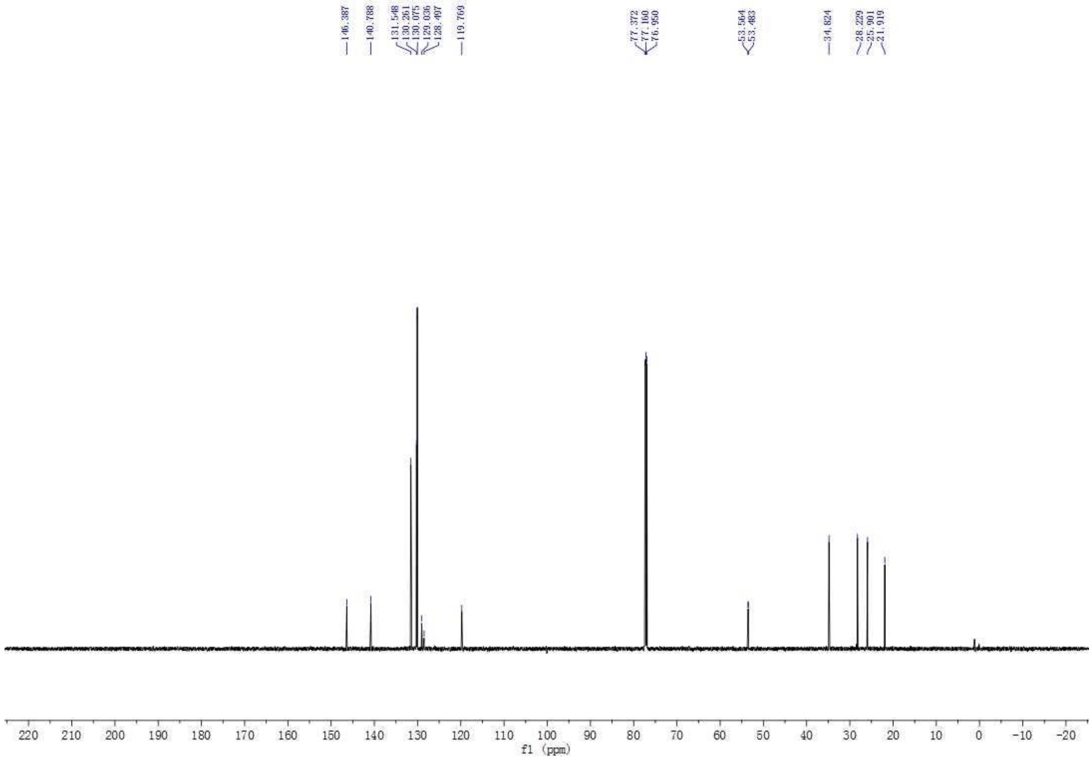

Supplementary Fig. 101. ¹³C NMR of compound 2ao. The sample has been recorded in 150 MHz, $CDCl_3$ at 25 °C

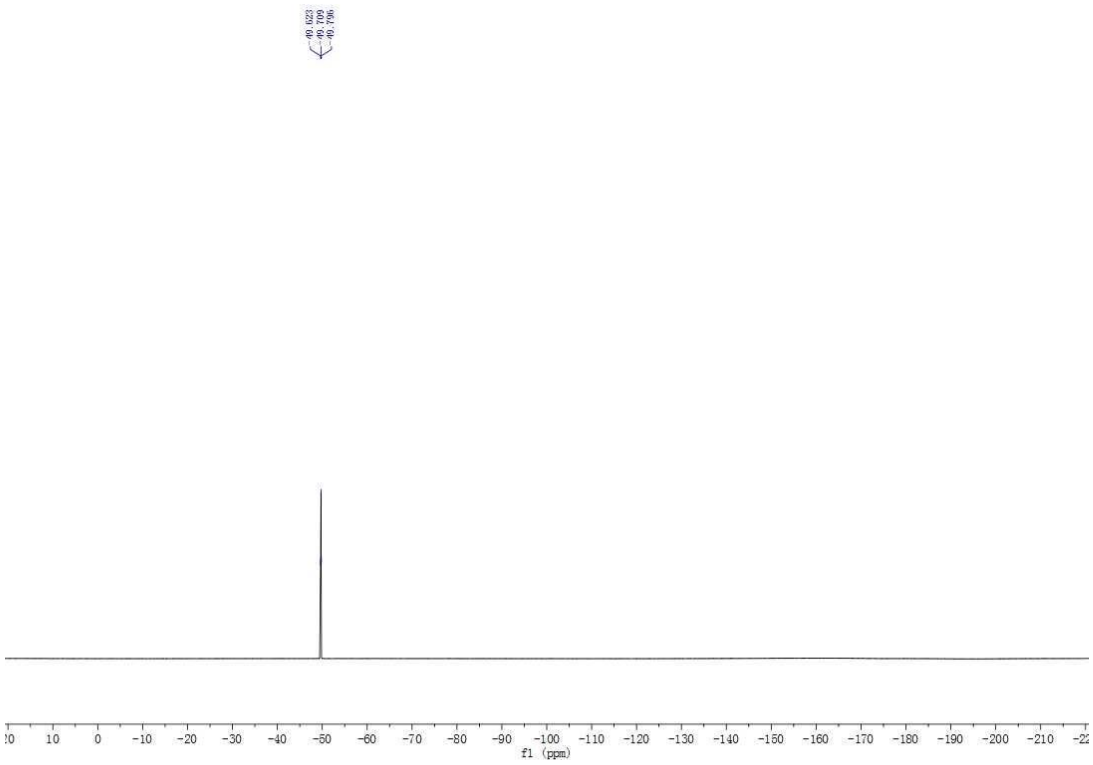

Supplementary Fig. 102. ^{19}F NMR of compound 2ao. The sample has been recorded in 470 MHz, $CDCl_3$ at 25 °C

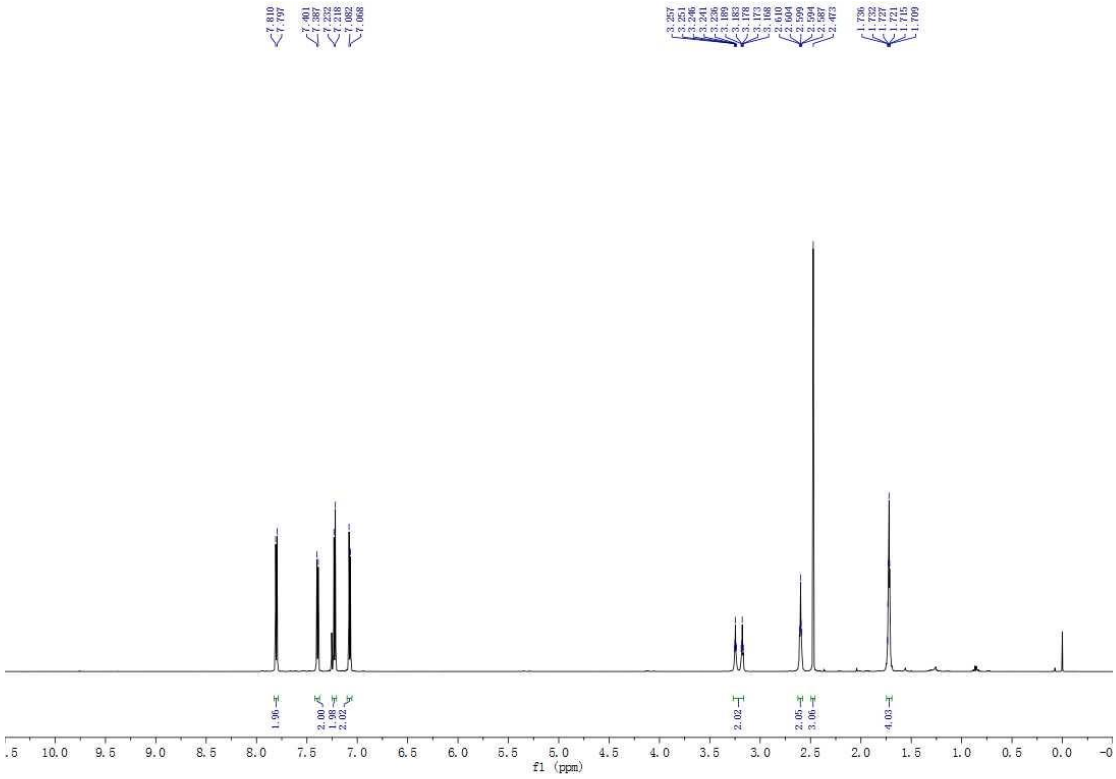

Supplementary Fig. 103. ^{1}H NMR of compound 2ap. The sample has been recorded in 600 MHz, $CDCl_3$ at 25 °C

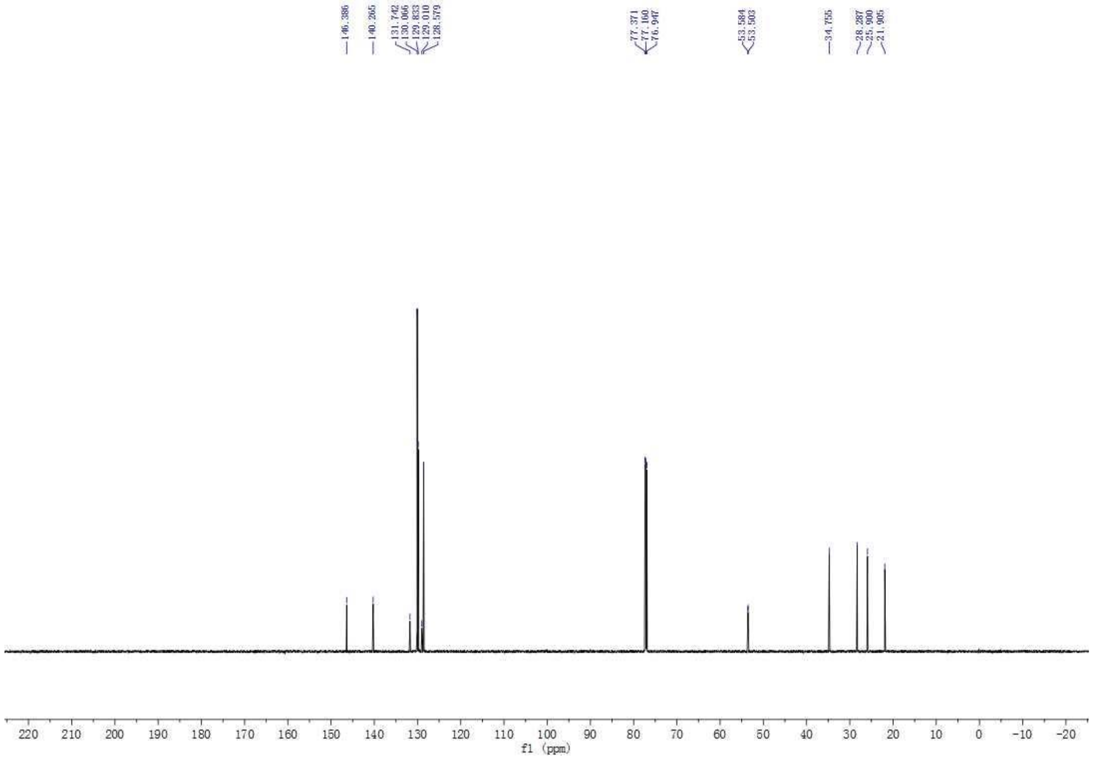

Supplementary Fig. 104. ^{13}C NMR of compound 2ap. The sample has been recorded in 150 MHz, $CDCl_3$ at 25 °C

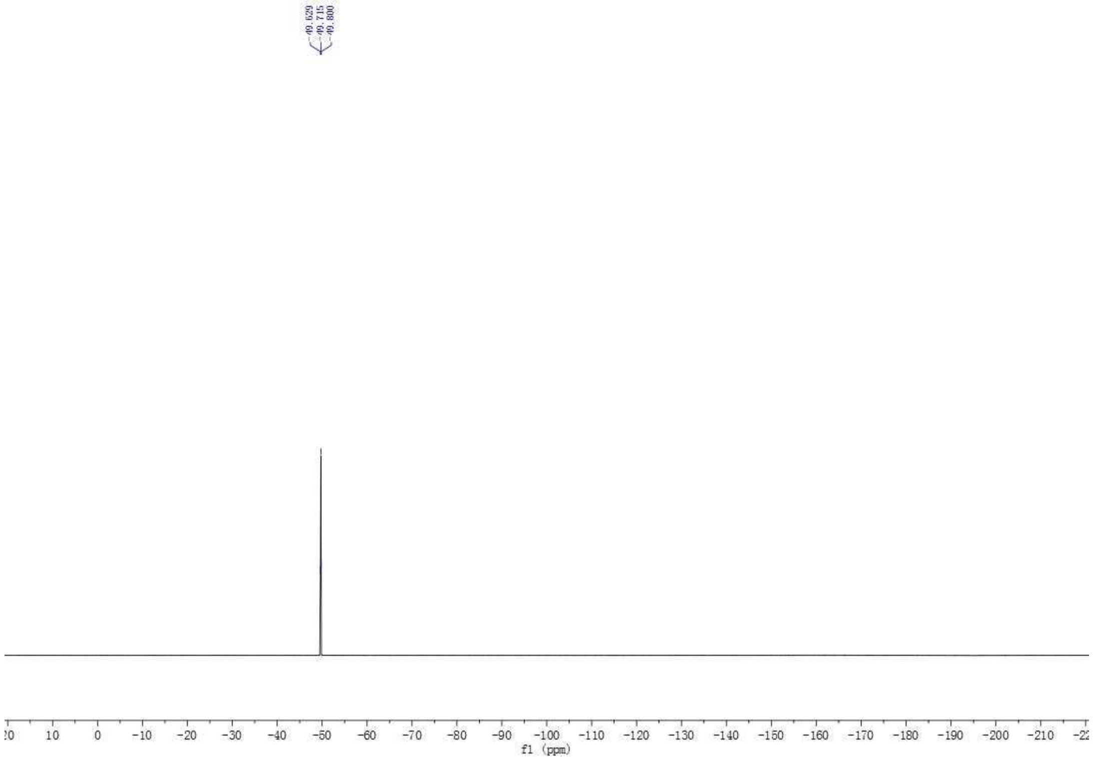

Supplementary Fig. 105. ^{19}F NMR of compound 2ap. The sample has been recorded in 470 MHz, $CDCl_3$ at 25 °C

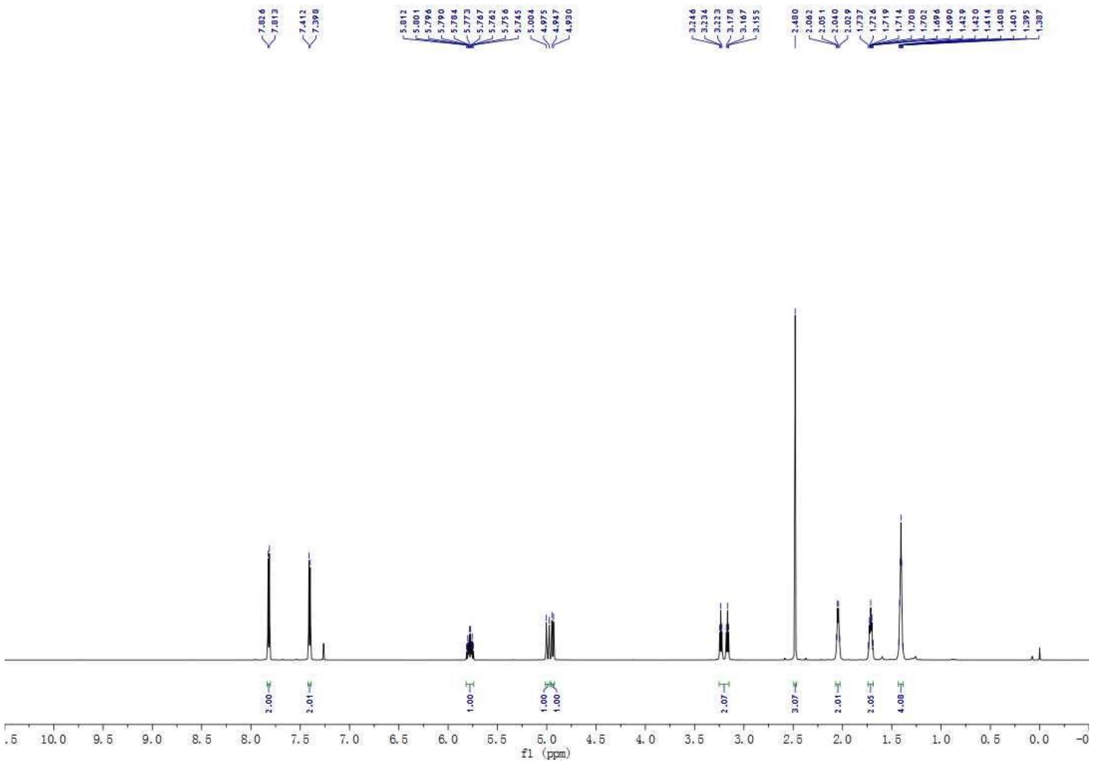

Supplementary Fig. 106. ^{1}H NMR of compound 2as. The sample has been recorded in 600 MHz, $CDCl_3$ at 25 °C

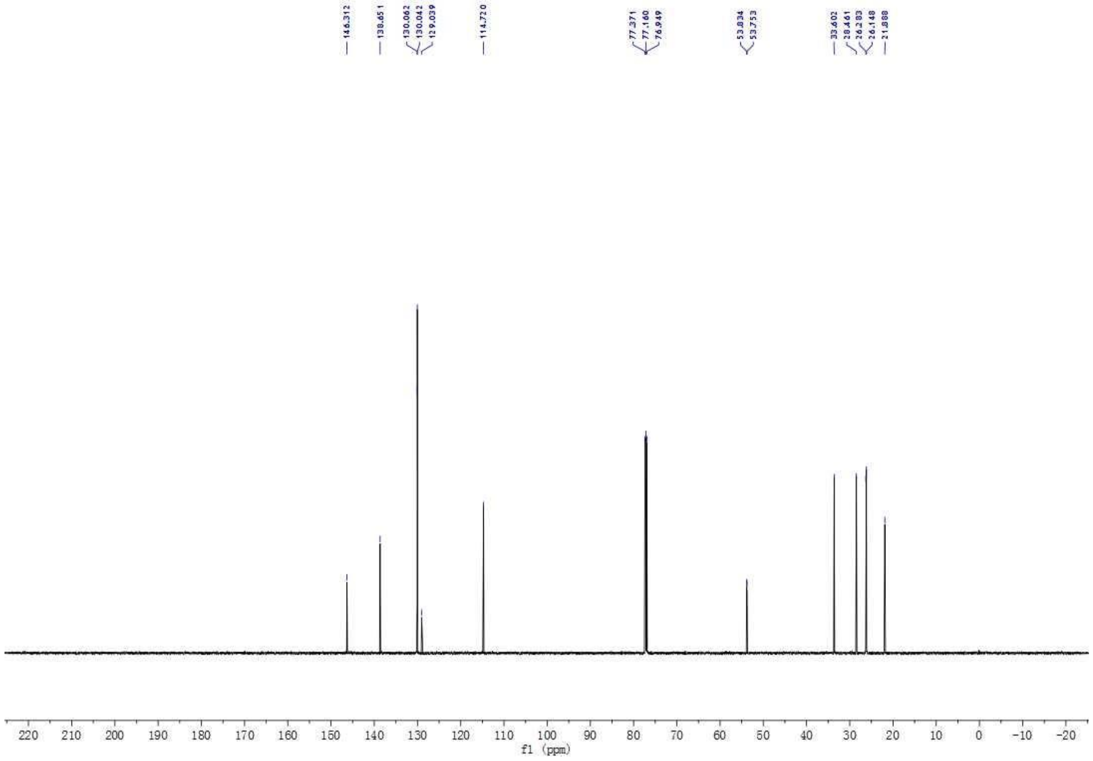

Supplementary Fig. 107. ^{13}C NMR of compound 2as. The sample has been recorded in 150 MHz, $CDCl_3$ at 25 °C

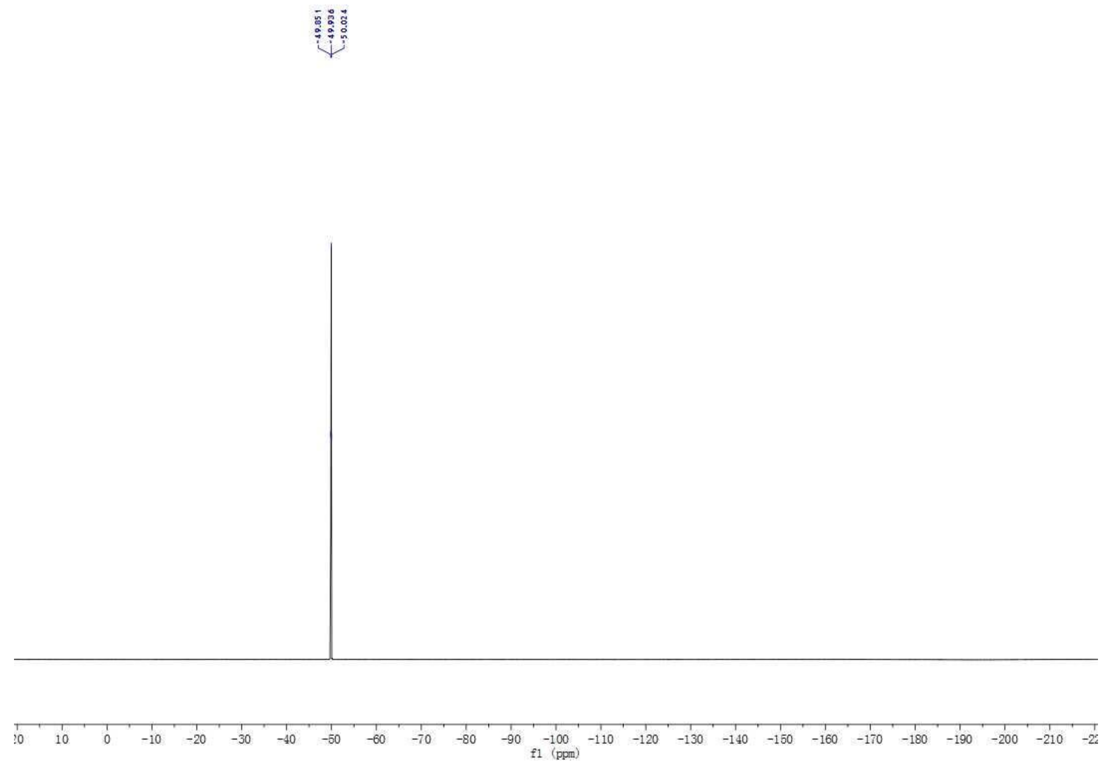

Supplementary Fig. 108. ^{19}F NMR of compound 2as. The sample has been recorded in 470 MHz, $CDCl_3$ at 25 °C

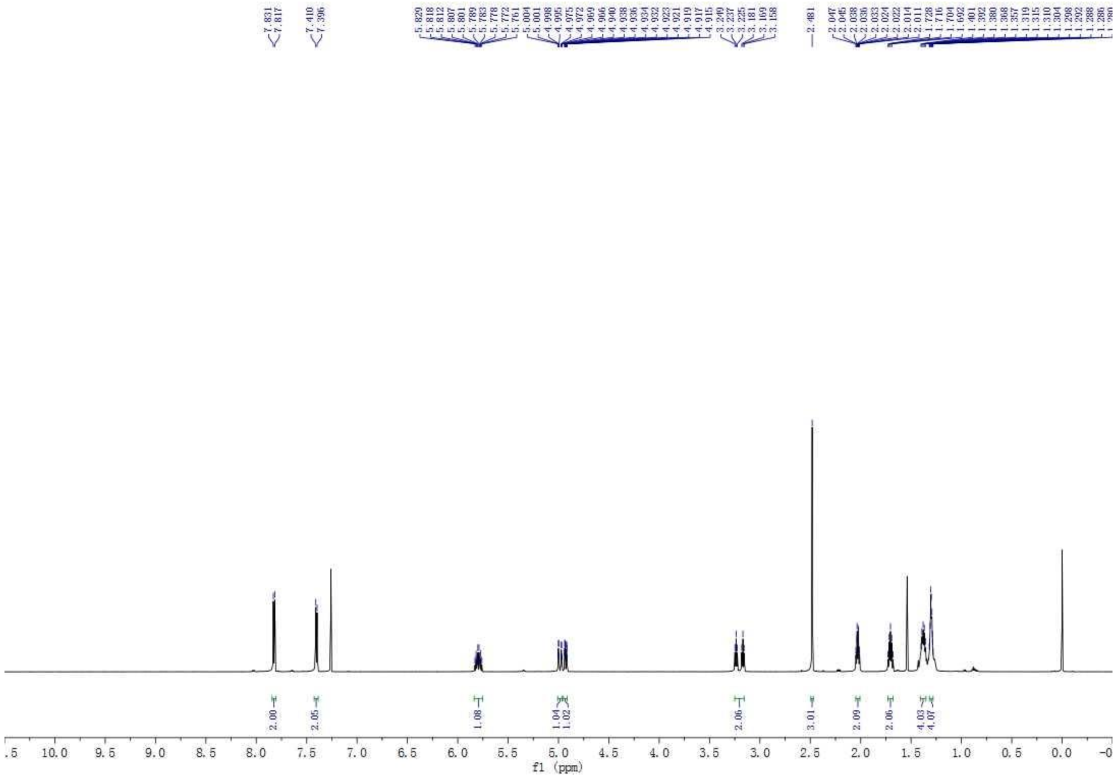

Supplementary Fig. 109. ^{1}H NMR of compound 13. The sample has been recorded in 600 MHz, $CDCl_3$ at 25 °C

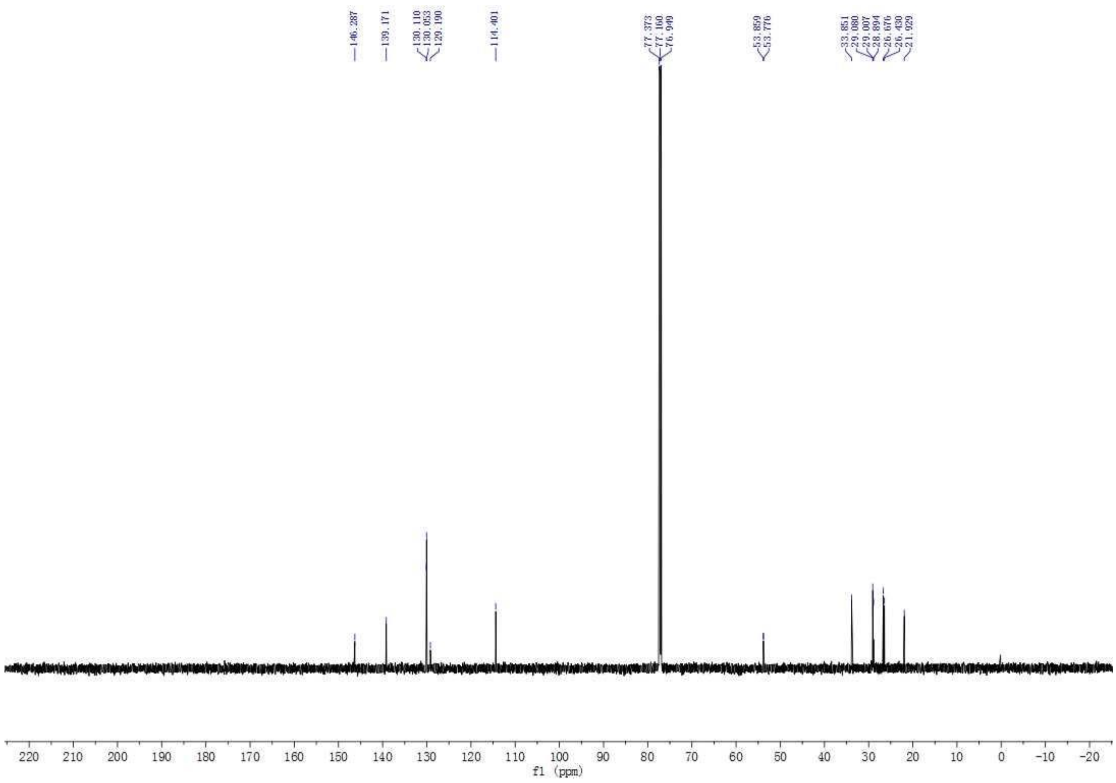

Supplementary Fig. 110. ^{13}C NMR of compound 13. The sample has been recorded in 150 MHz, $CDCl_3$ at 25 °C

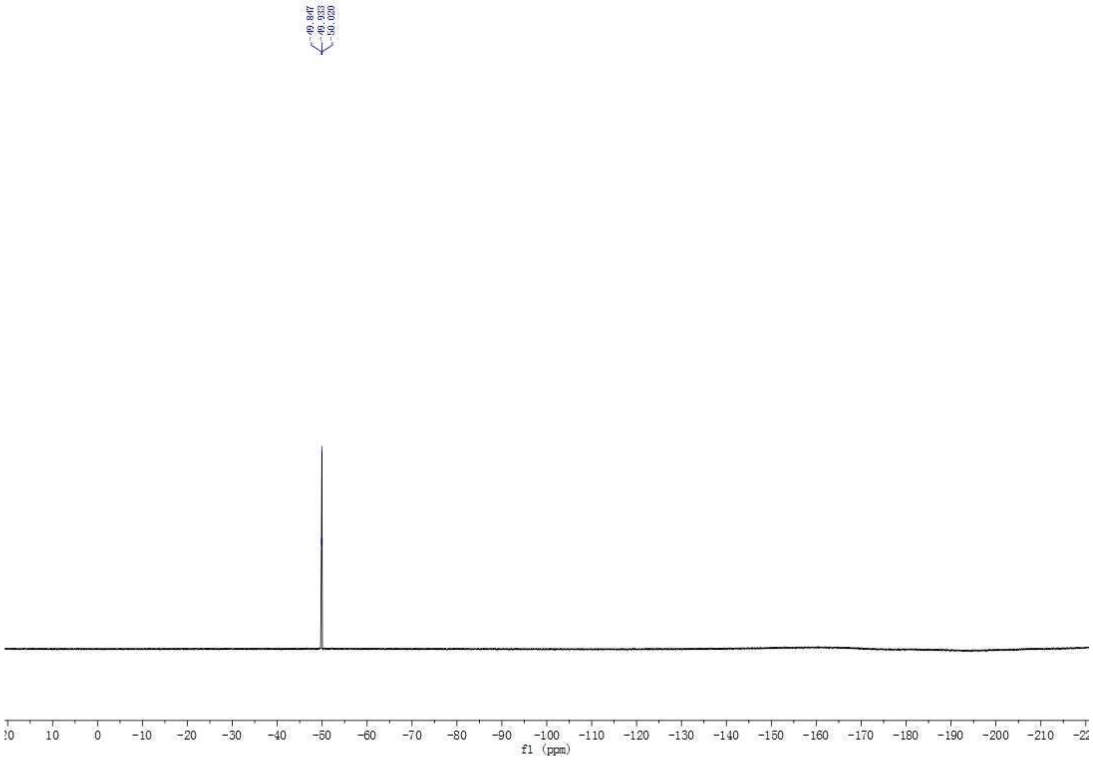

Supplementary Fig. 111. ^{19}F NMR of compound 13. The sample has been recorded in 470 MHz, $CDCl_3$ at 25 °C

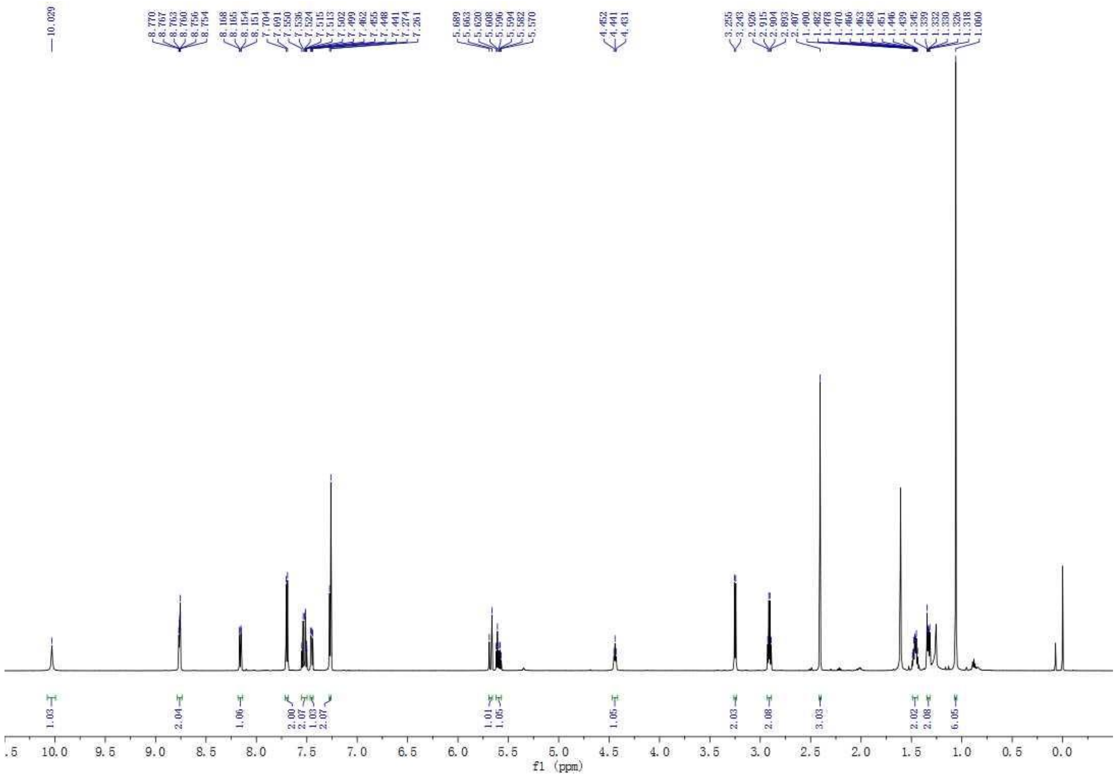

Supplementary Fig. 112. ^{1}H NMR of compound 3a. The sample has been recorded in 600 MHz, $CDCl_3$ at 25 °C

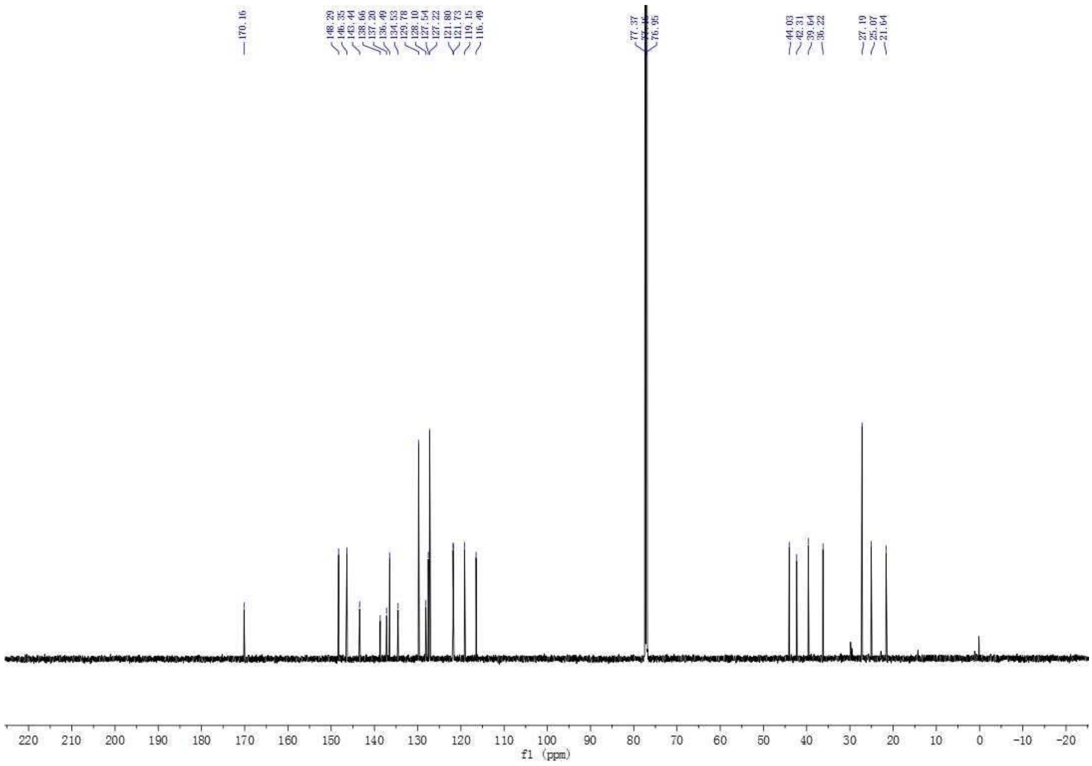

Supplementary Fig. 113. ^{13}C NMR of compound 3a. The sample has been recorded in 150 MHz, $CDCl_3$ at 25 °C

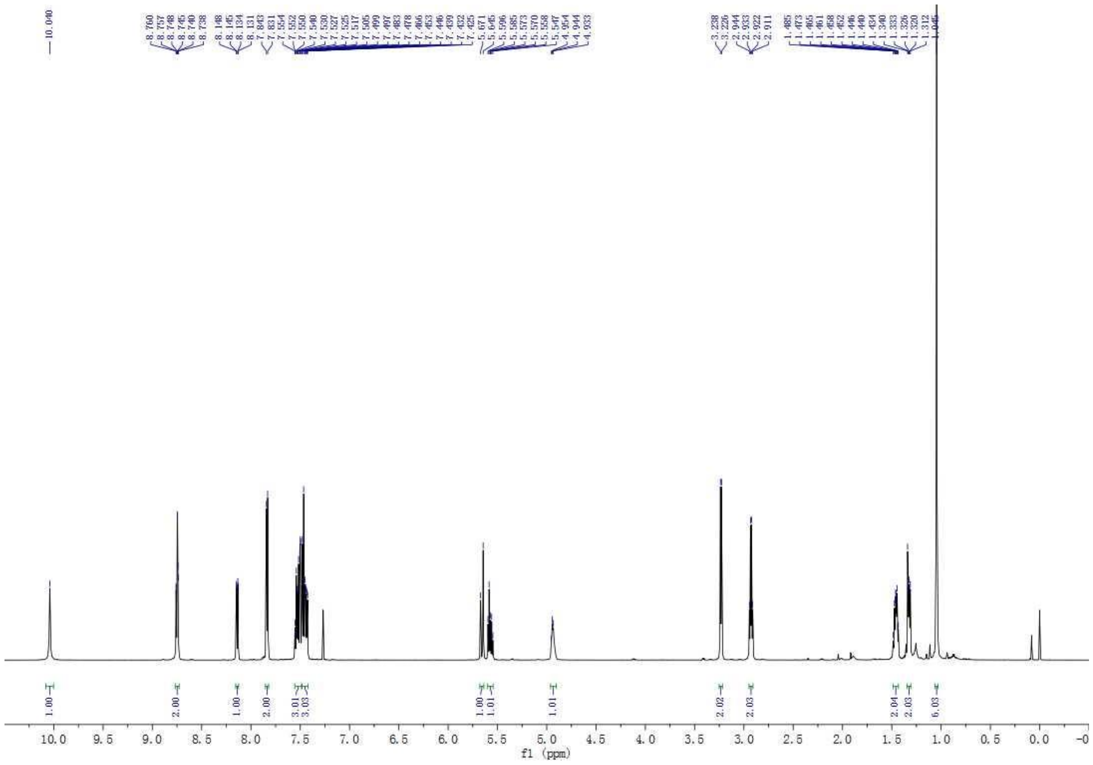

Supplementary Fig. 114. ^{1}H NMR of compound 3b. The sample has been recorded in 600 MHz, $CDCl_3$ at 25 °C

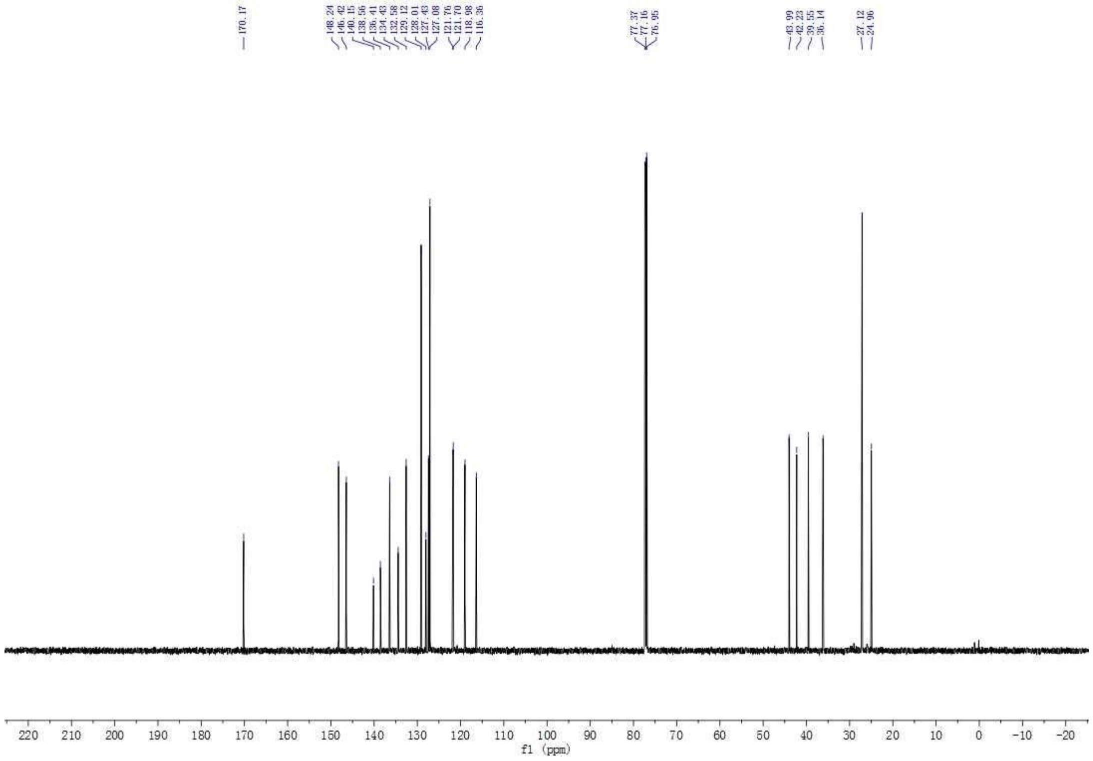

Supplementary Fig. 115. ^{13}C NMR of compound 3b. The sample has been recorded in 150 MHz, $CDCl_3$ at 25 °C

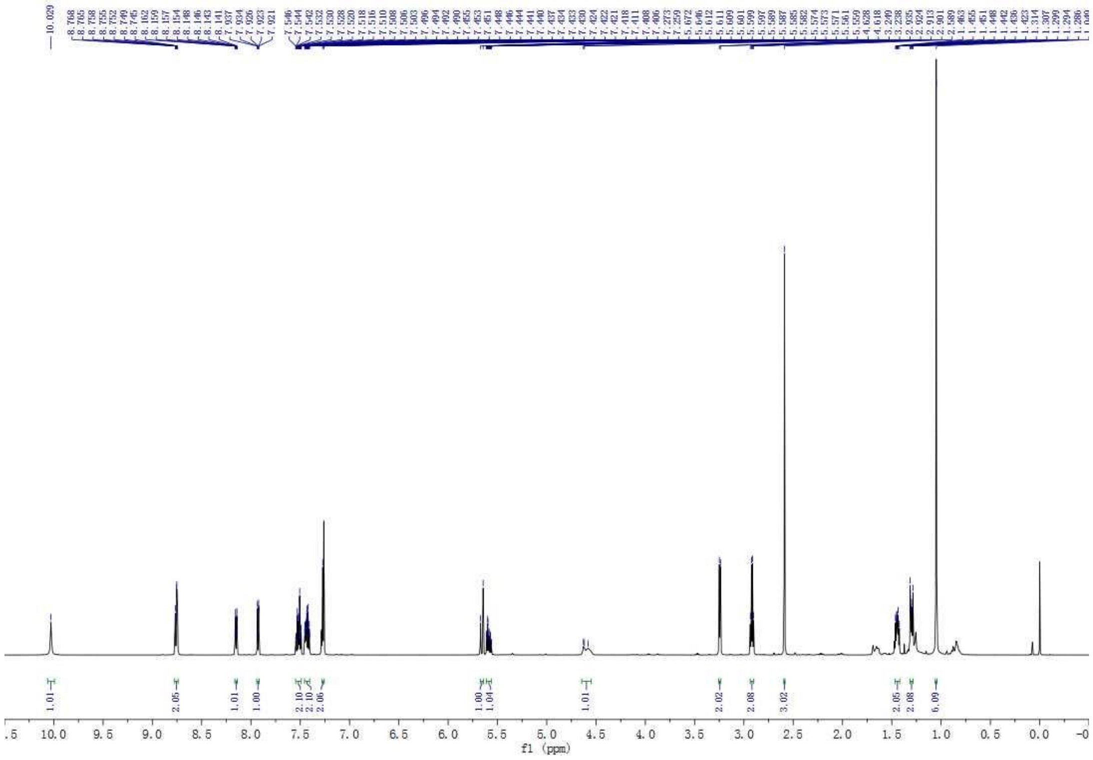

Supplementary Fig. 116. ^{1}H NMR of compound 3c. The sample has been recorded in 600 MHz, $CDCl_3$ at 25 °C

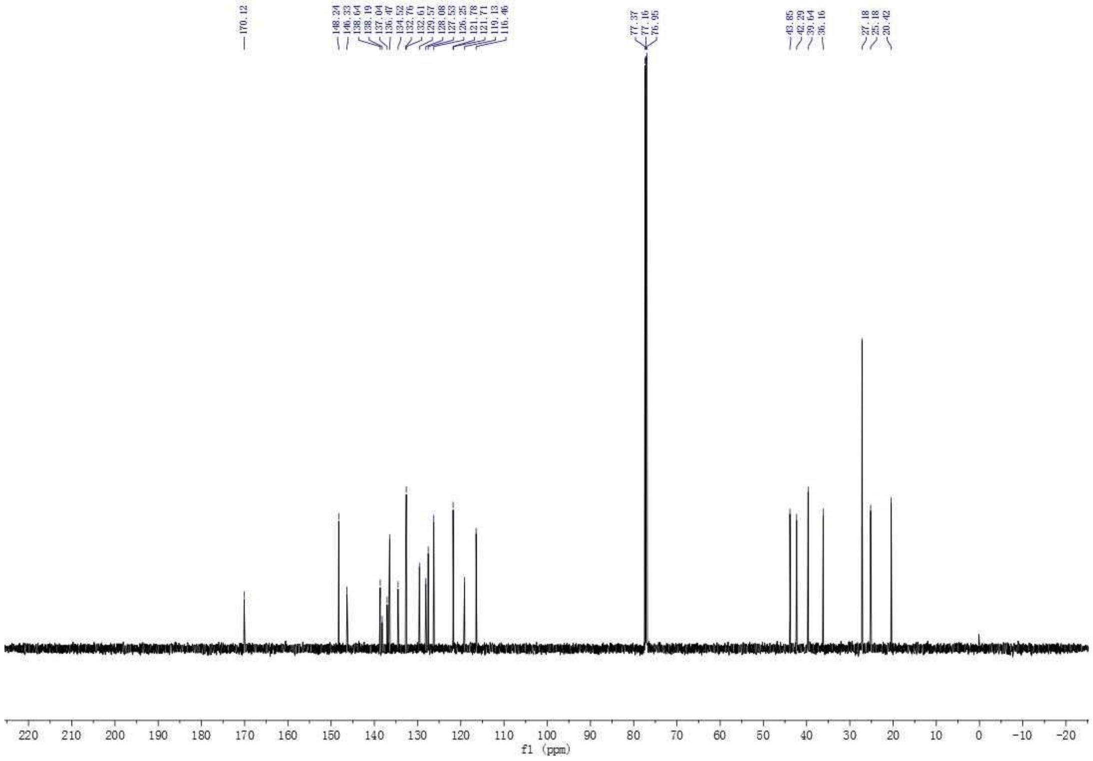

Supplementary Fig. 117. ^{13}C NMR of compound 3c. The sample has been recorded in 150 MHz, $CDCl_3$ at 25 °C

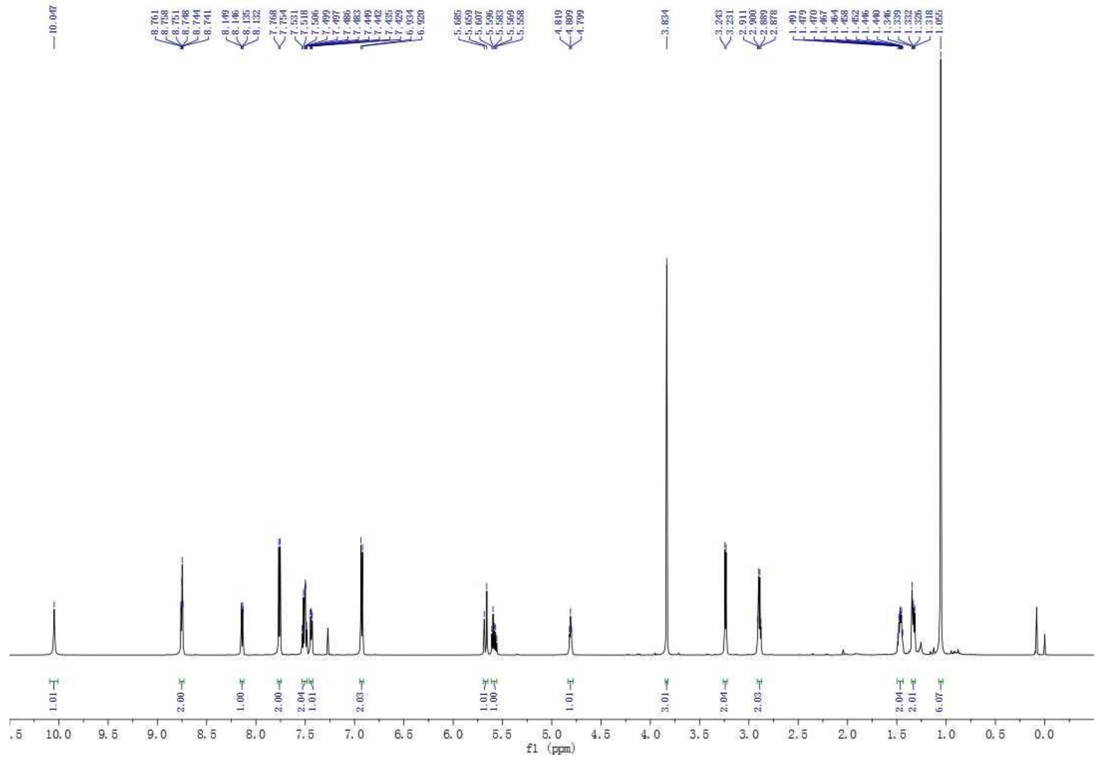

Supplementary Fig. 118. ^{1}H NMR of compound 3d. The sample has been recorded in 600 MHz, $CDCl_3$ at 25 °C

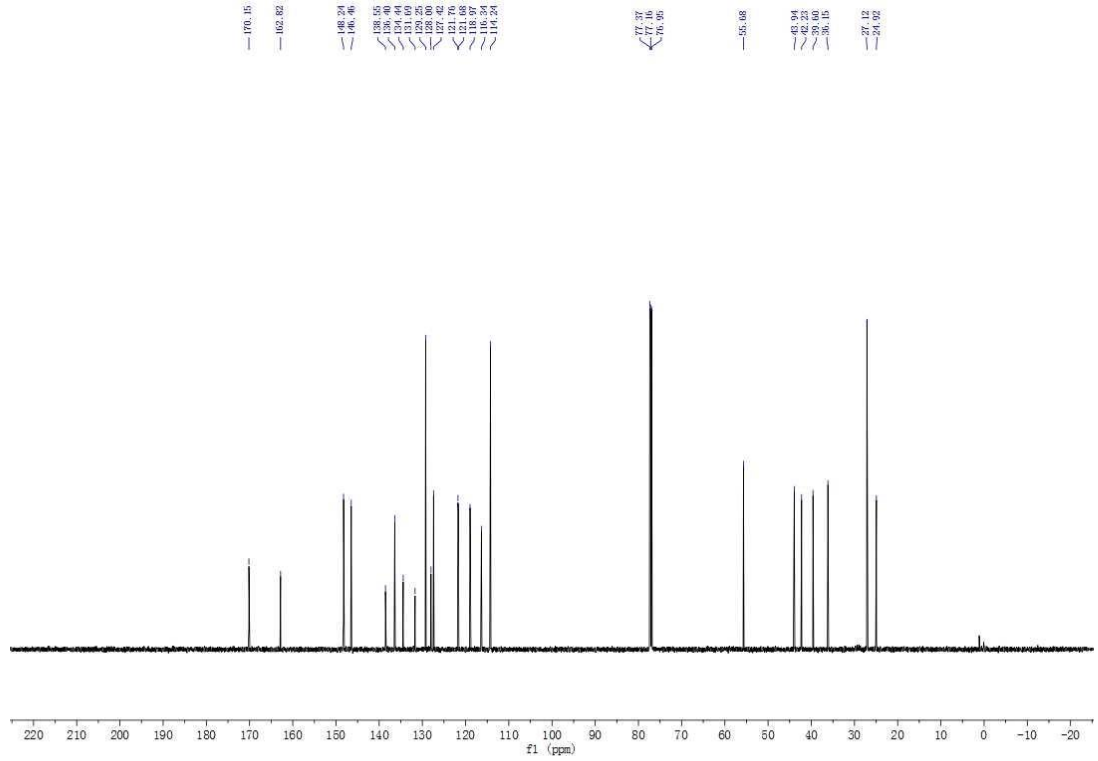

Supplementary Fig. 119. ^{13}C NMR of compound 3d. The sample has been recorded in 150 MHz, $CDCl_3$ at 25 °C

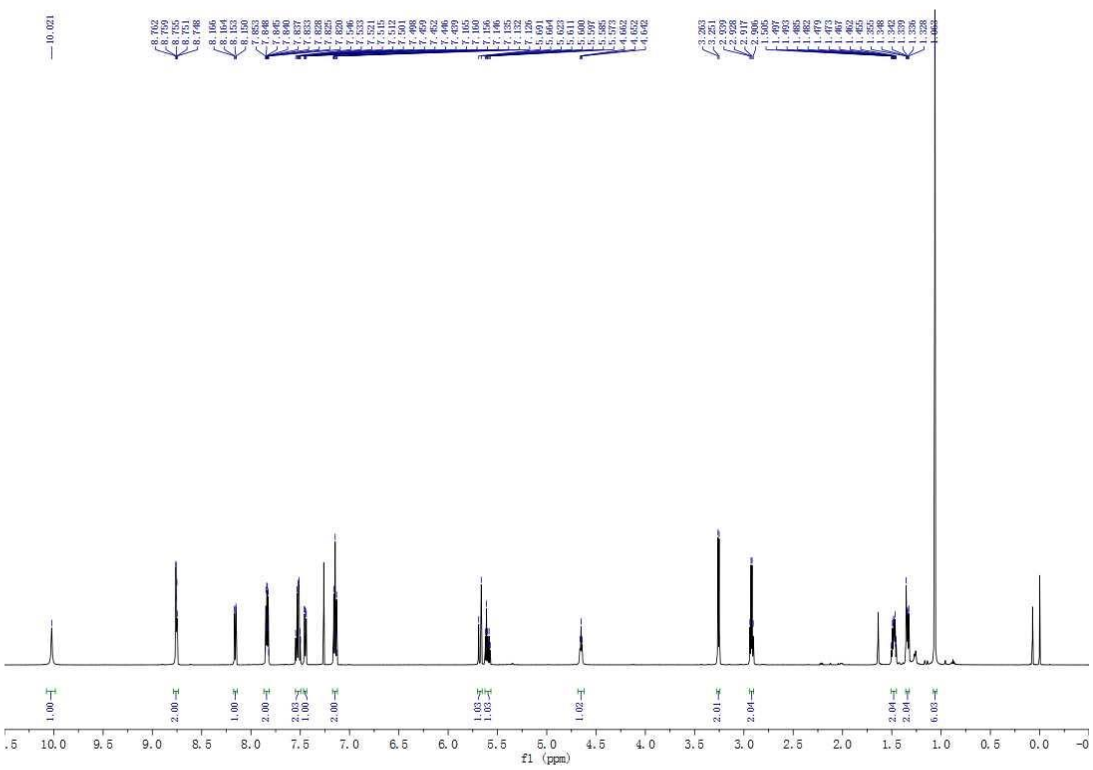

Supplementary Fig. 120. ^{1}H NMR of compound 3e. The sample has been recorded in 600 MHz, $CDCl_3$ at 25 °C

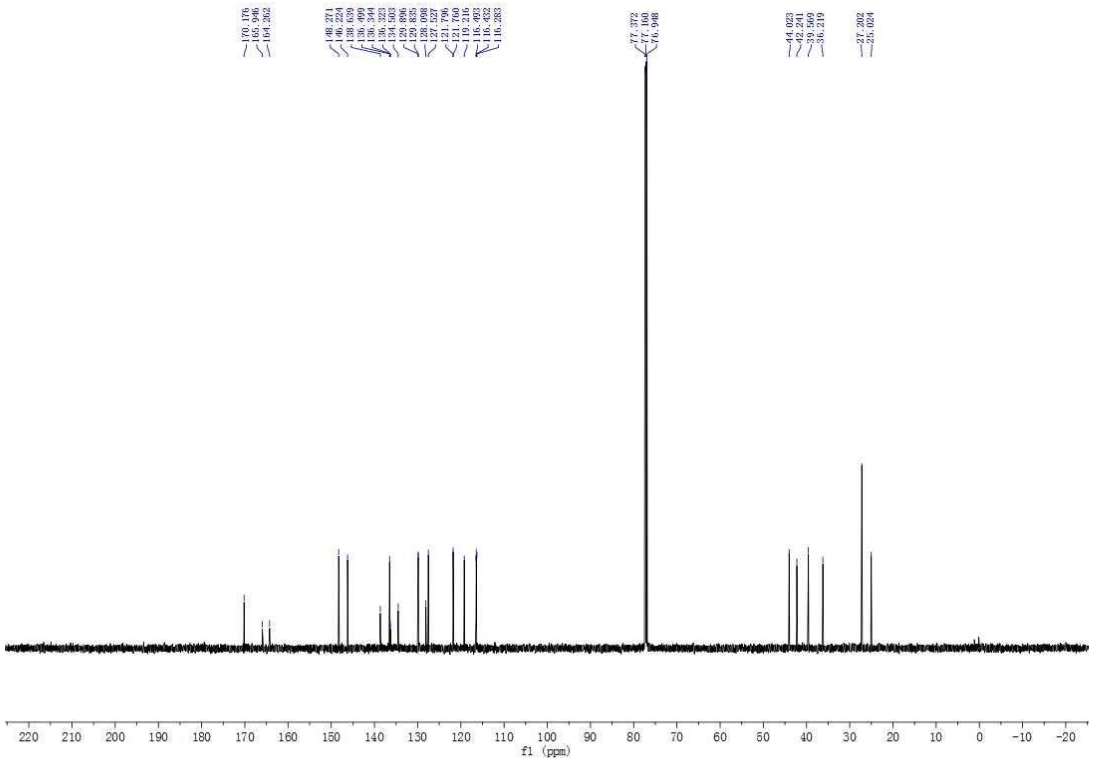

Supplementary Fig. 121. ^{13}C NMR of compound 3e. The sample has been recorded in 150 MHz, $CDCl_3$ at 25 °C

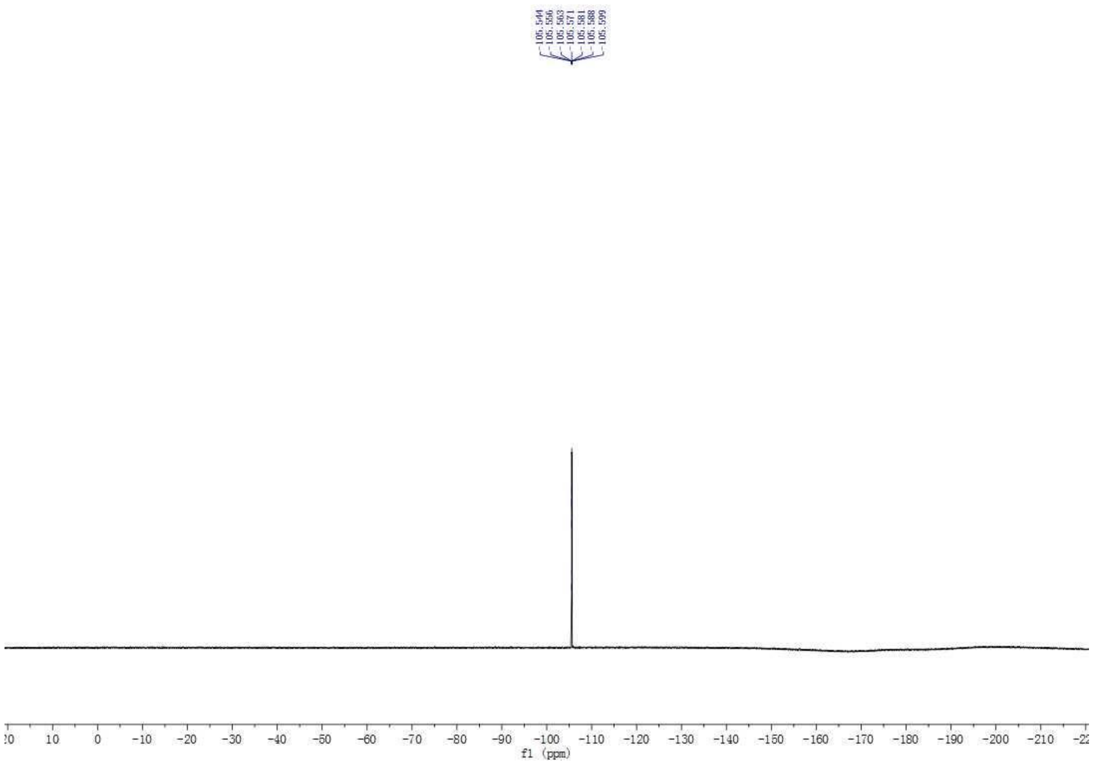

Supplementary Fig. 122. ^{19}F NMR of compound 3e. The sample has been recorded in 470 MHz, $CDCl_3$ at 25 °C

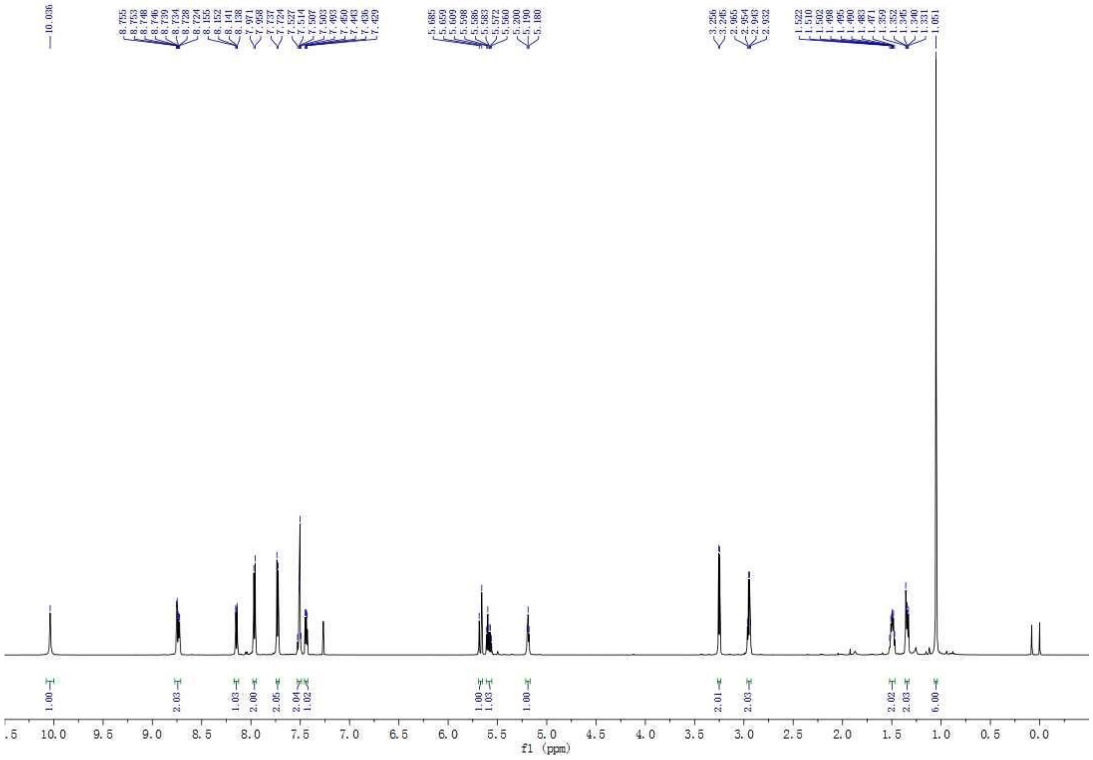

Supplementary Fig. 123. ^{1}H NMR of compound 3f. The sample has been recorded in 600 MHz, $CDCl_3$ at 25 °C

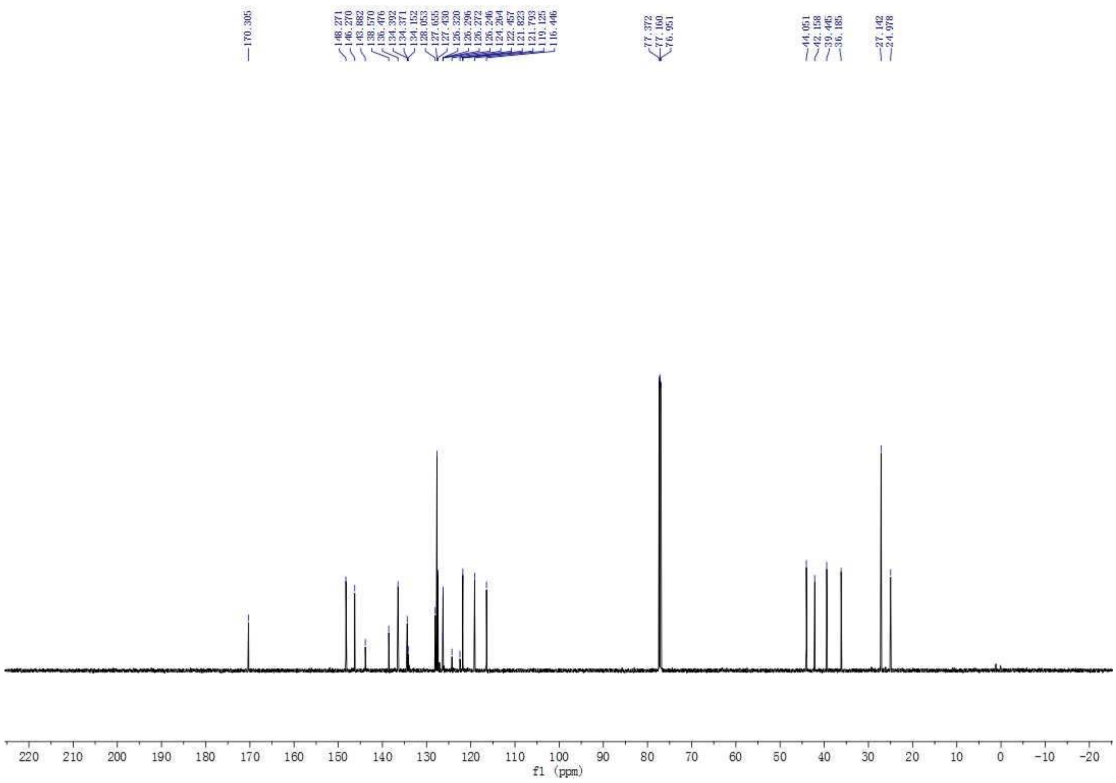

Supplementary Fig. 124. ^{13}C NMR of compound 3f. The sample has been recorded in 150 MHz, $CDCl_3$ at 25 °C

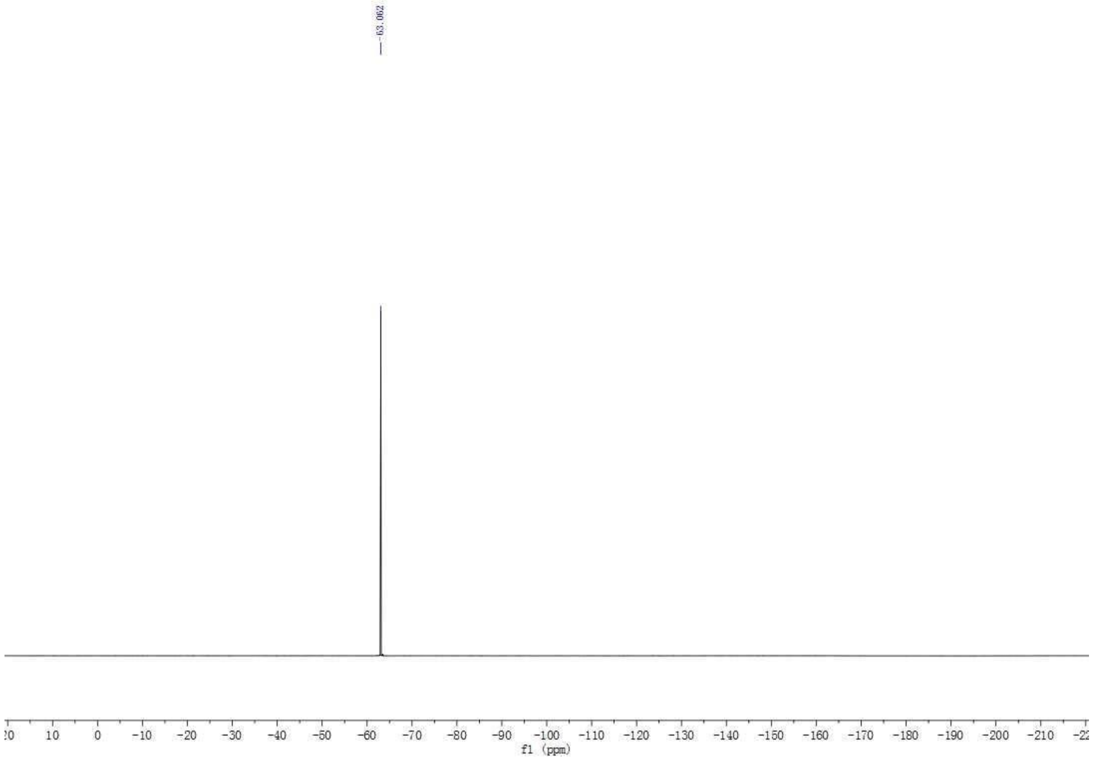

Supplementary Fig. 125. ^{19}F NMR of compound 3f. The sample has been recorded in 470 MHz, $CDCl_3$ at 25 °C

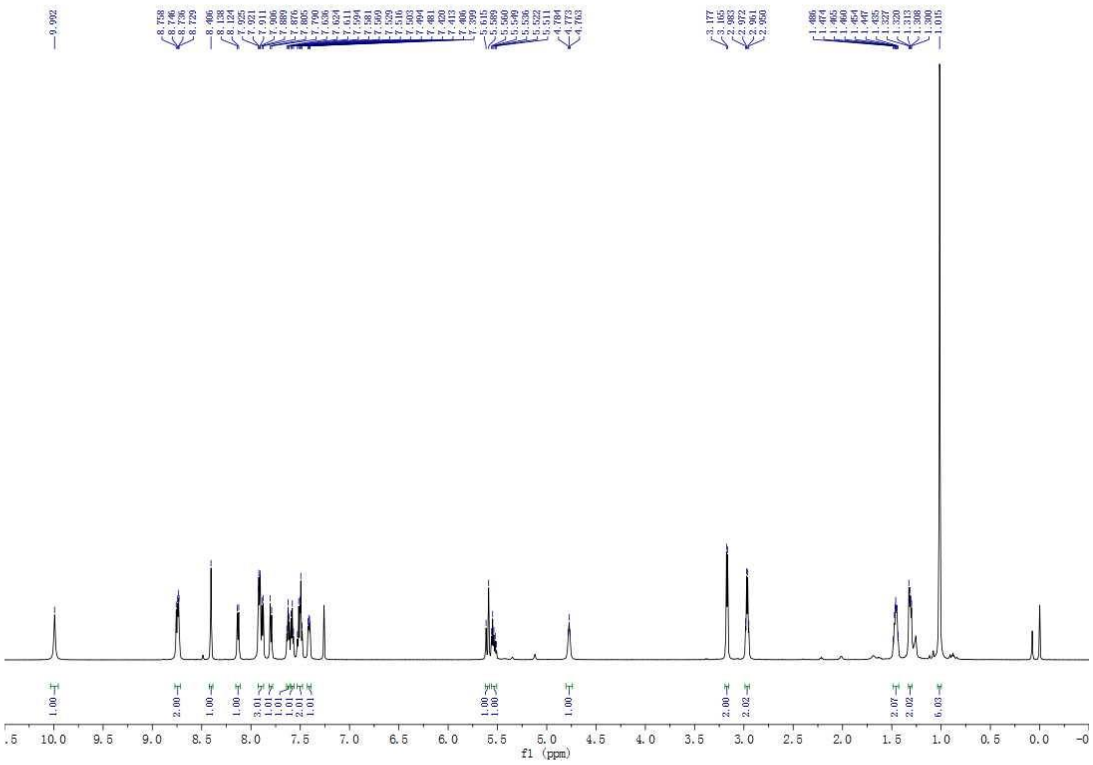

Supplementary Fig. 126. ^{1}H NMR of compound 3g. The sample has been recorded in 600 MHz, $CDCl_3$ at 25 °C

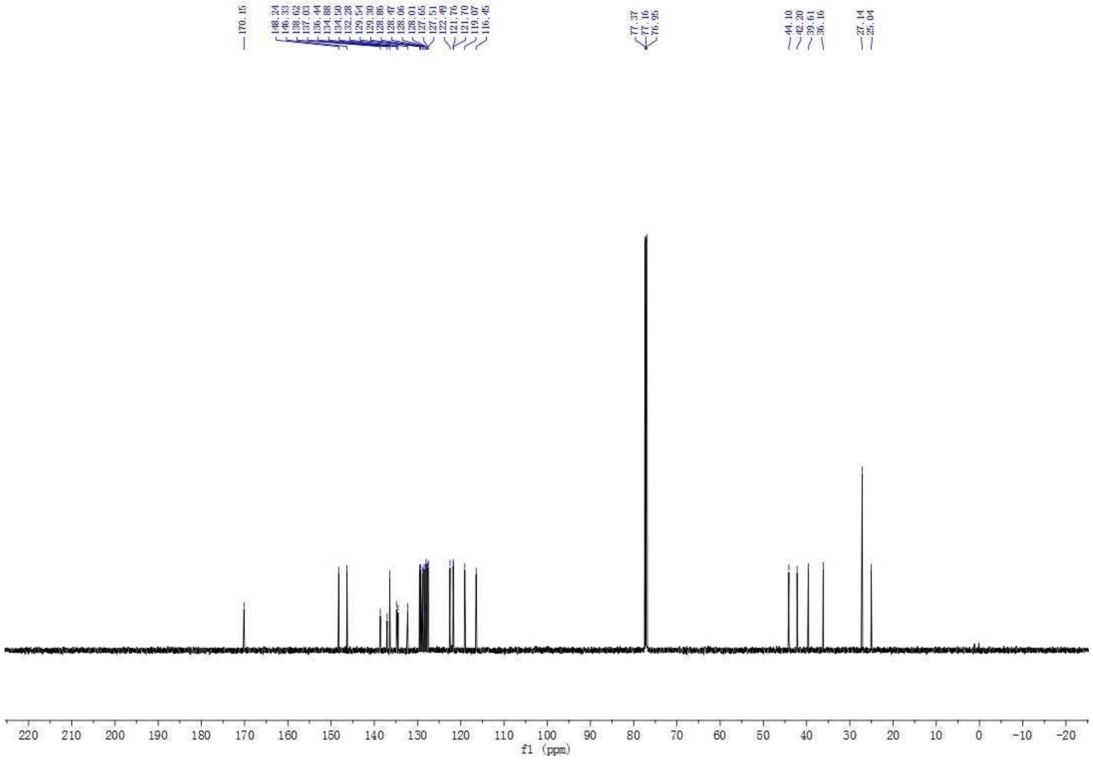

Supplementary Fig. 127. ^{13}C NMR of compound 3g. The sample has been recorded in 150 MHz, $CDCl_3$ at 25 °C

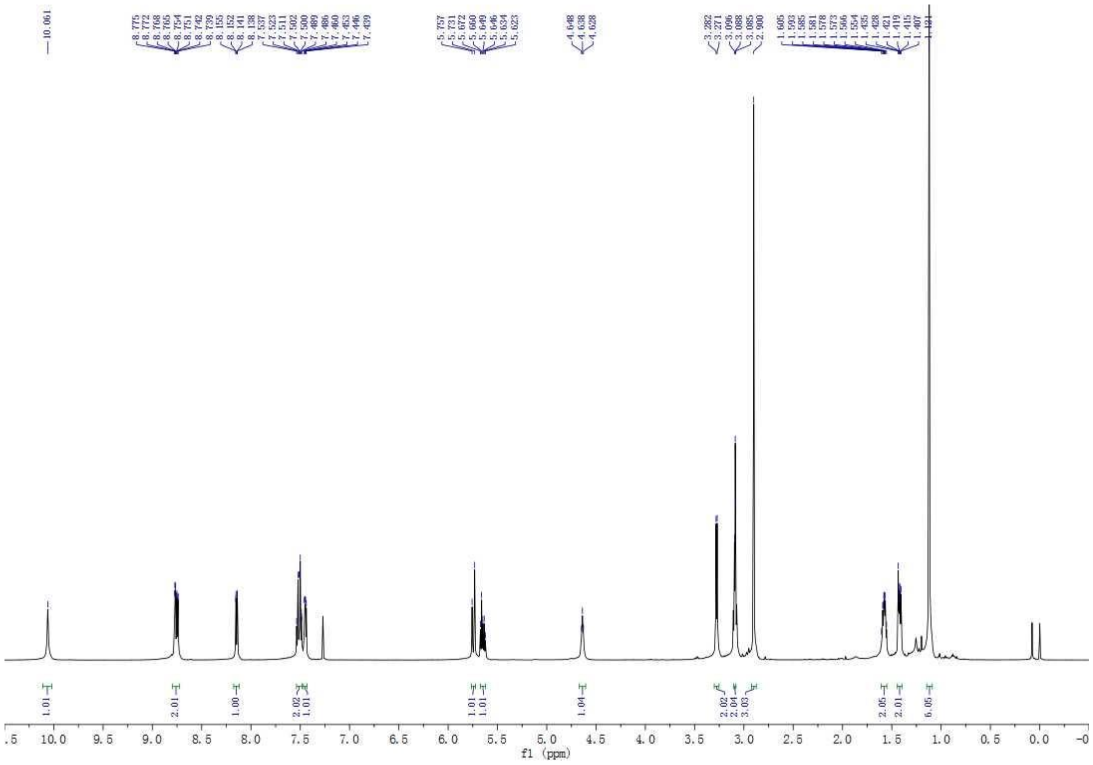

Supplementary Fig. 128. ^{1}H NMR of compound 3h. The sample has been recorded in 600 MHz, $CDCl_3$ at 25 °C

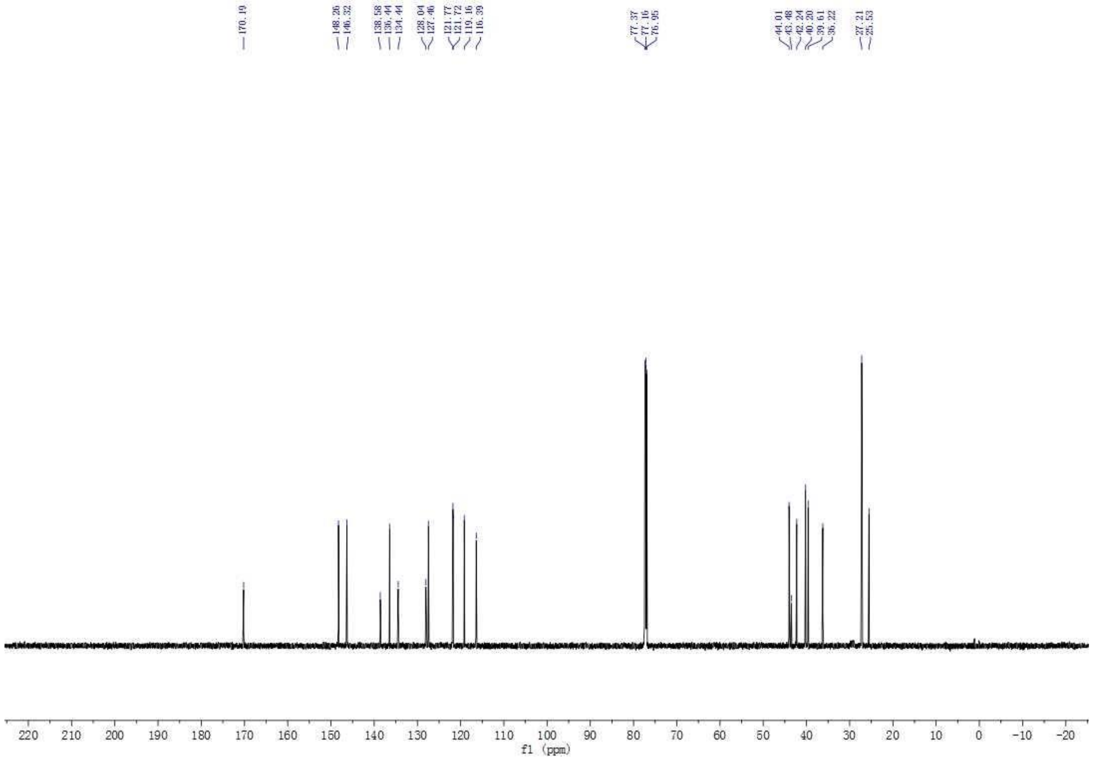

Supplementary Fig. 129. ^{13}C NMR of compound 3h. The sample has been recorded in 150 MHz, $CDCl_3$ at 25 °C

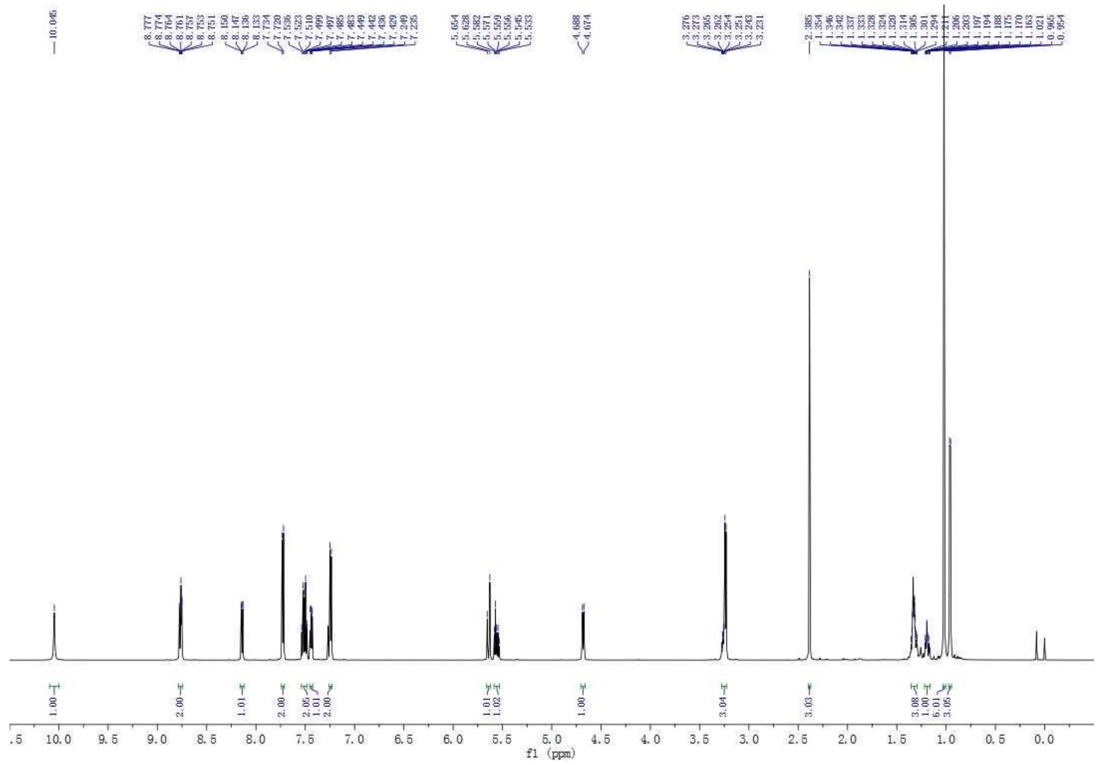

Supplementary Fig. 130. ^{1}H NMR of compound 3i. The sample has been recorded in 600 MHz, $CDCl_3$ at 25 °C

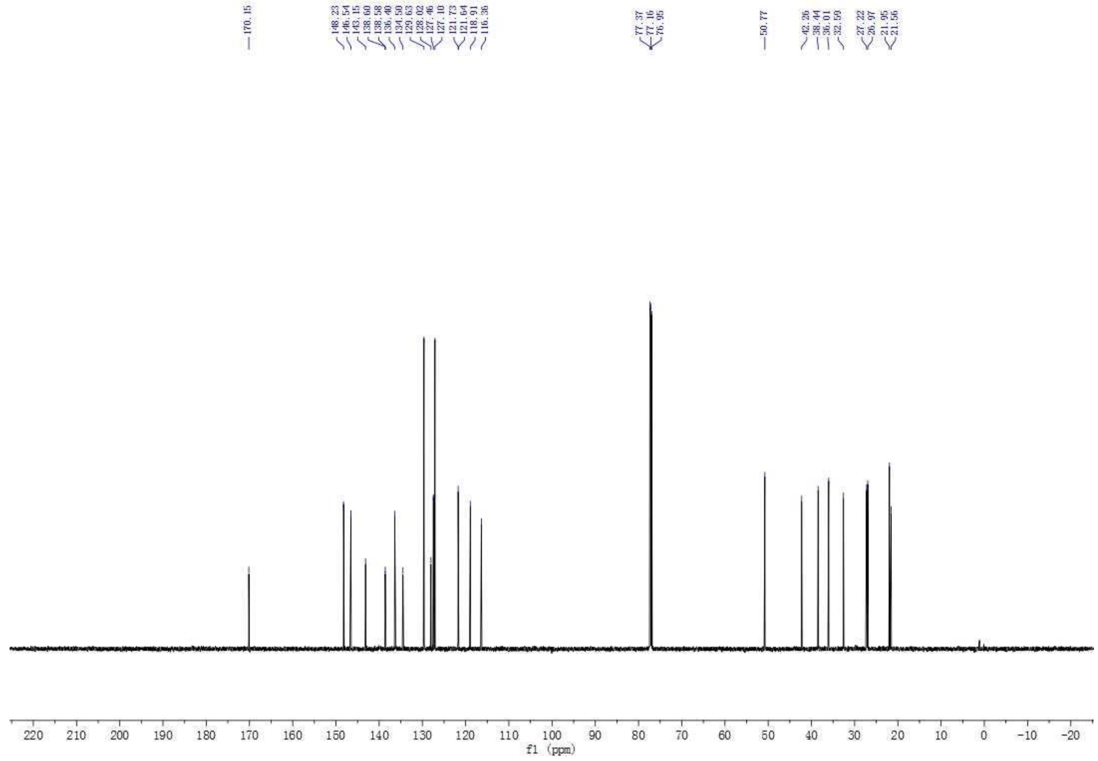

Supplementary Fig. 131. ^{13}C NMR of compound 3i. The sample has been recorded in 150 MHz, $CDCl_3$ at 25 °C

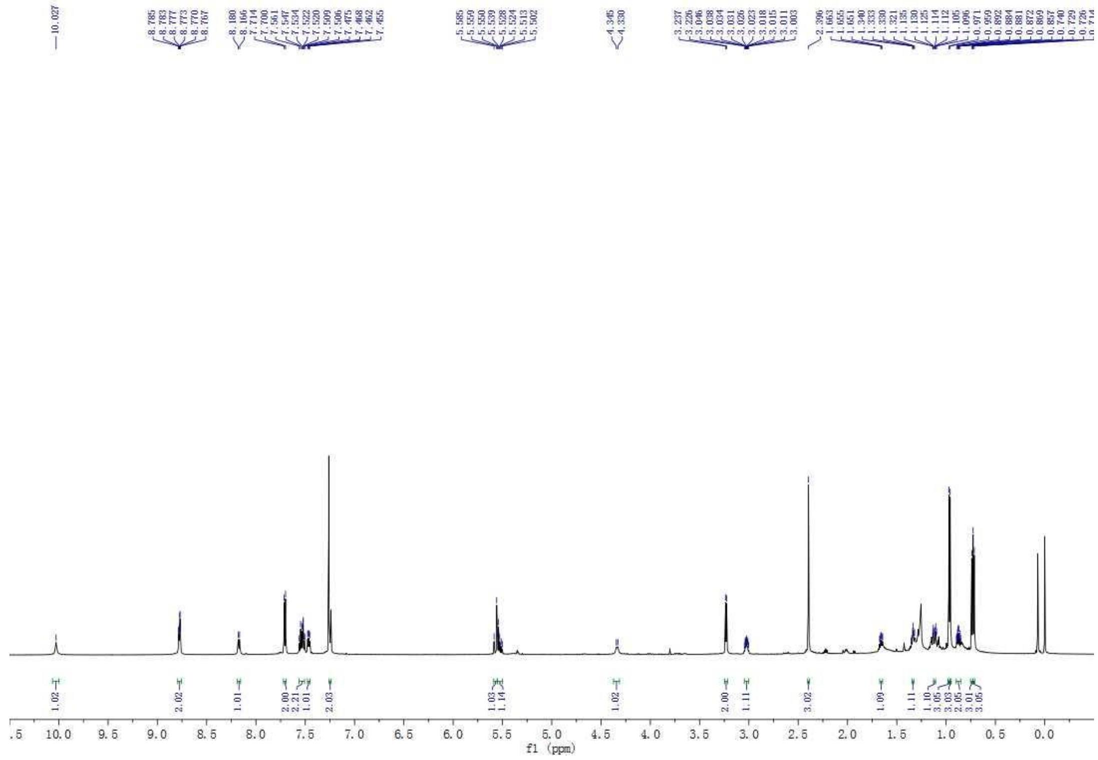

Supplementary Fig. 132. ^{1}H NMR of compound 3j. The sample has been recorded in 600 MHz, $CDCl_3$ at 25 °C

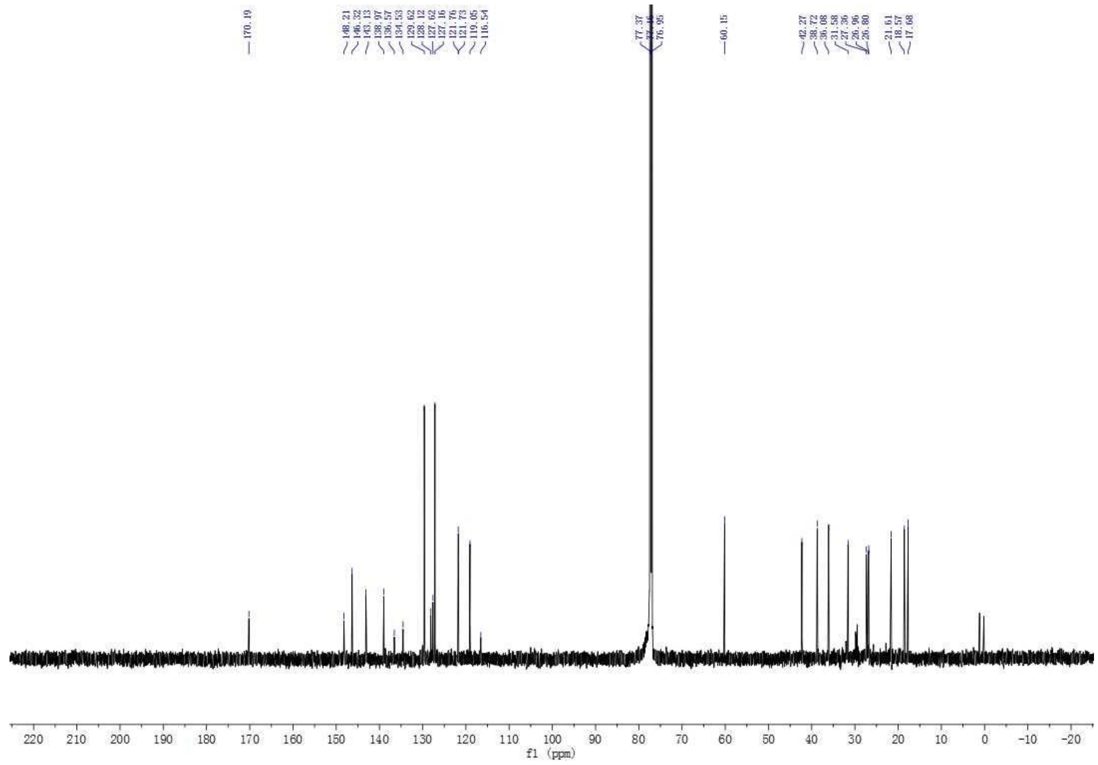

Supplementary Fig. 133. ^{13}C NMR of compound 3j. The sample has been recorded in 150 MHz, $CDCl_3$ at 25 °C

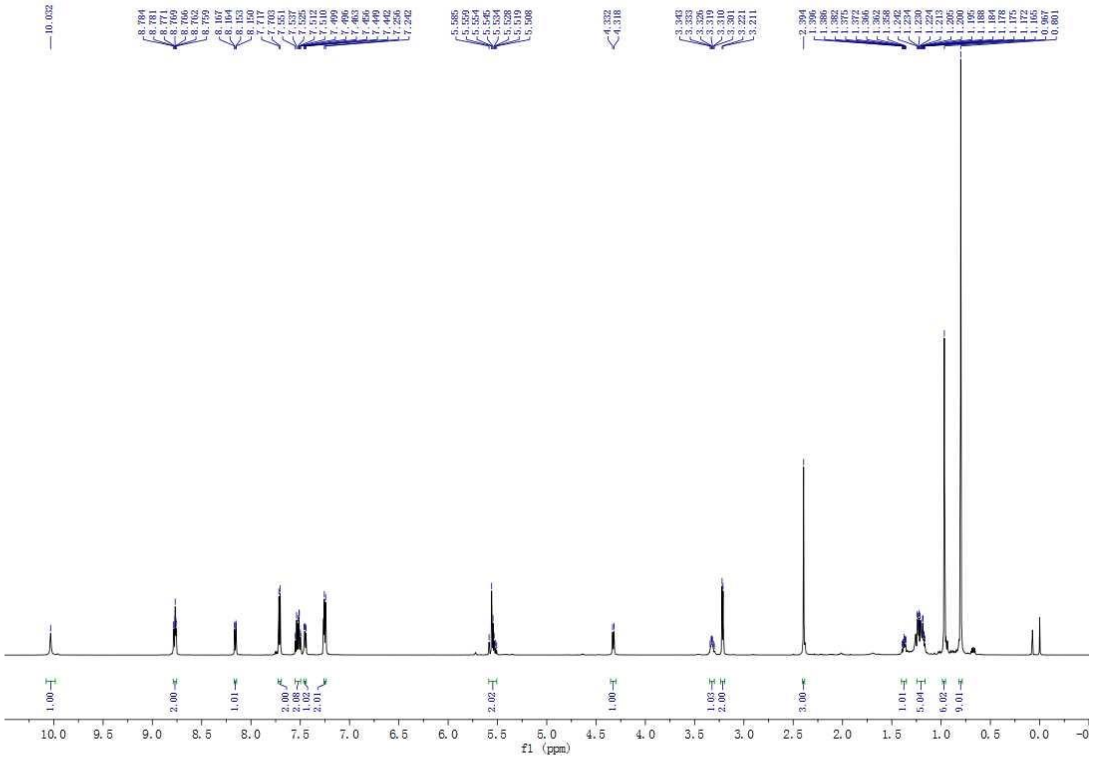

Supplementary Fig. 134. ¹H NMR of compound 3k. The sample has been recorded in 600 MHz, $CDCl_3$ at 25 °C

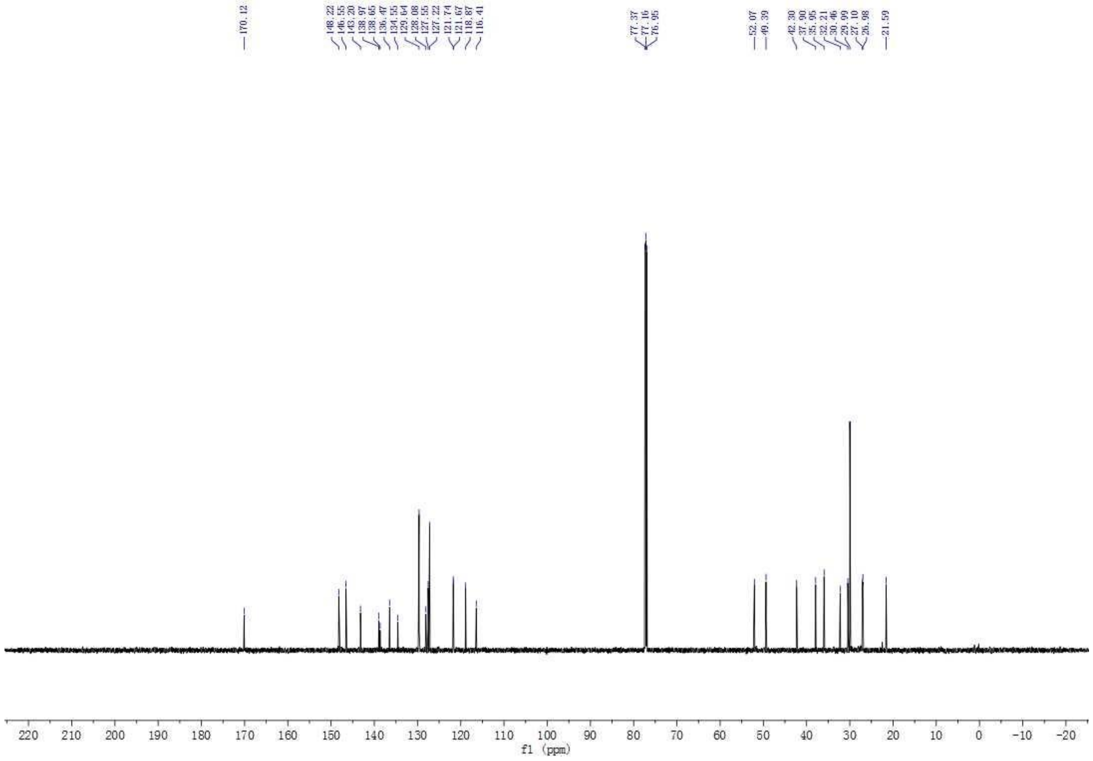

Supplementary Fig. 135. ¹³C NMR of compound 3k. The sample has been recorded in 150 MHz, $CDCl_3$ at 25 °C

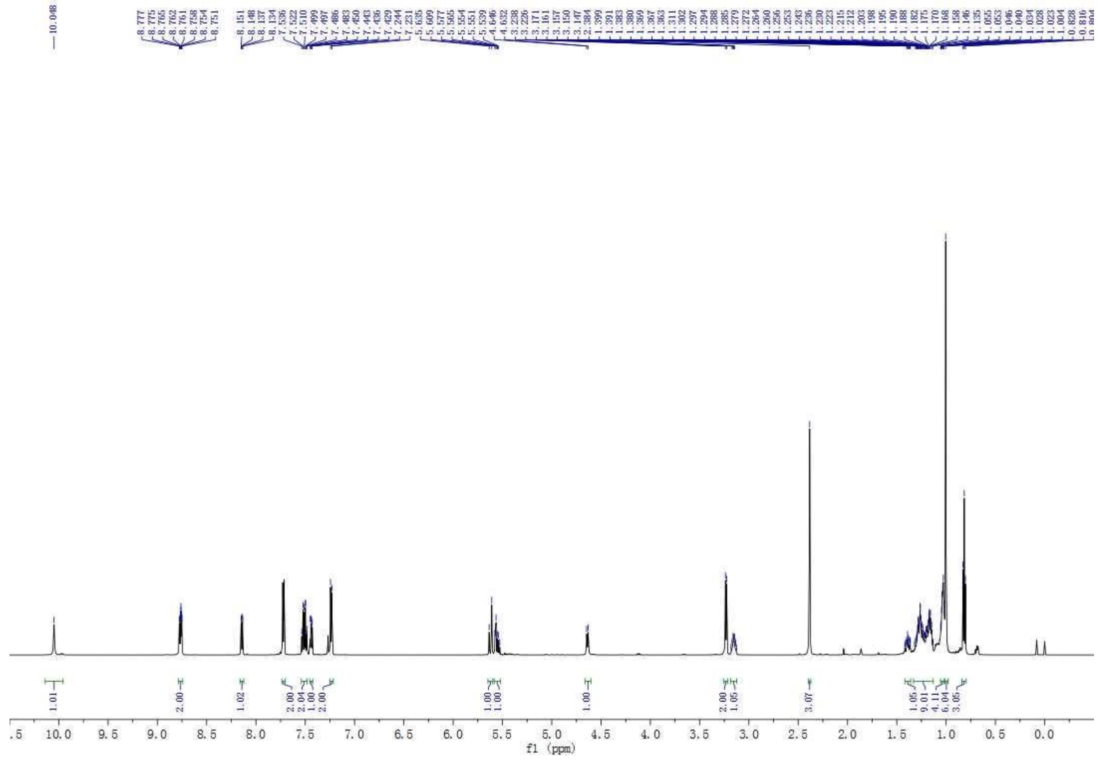

Supplementary Fig. 136. ^{1}H NMR of compound 3l. The sample has been recorded in 600 MHz, $CDCl_3$ at 25 °C

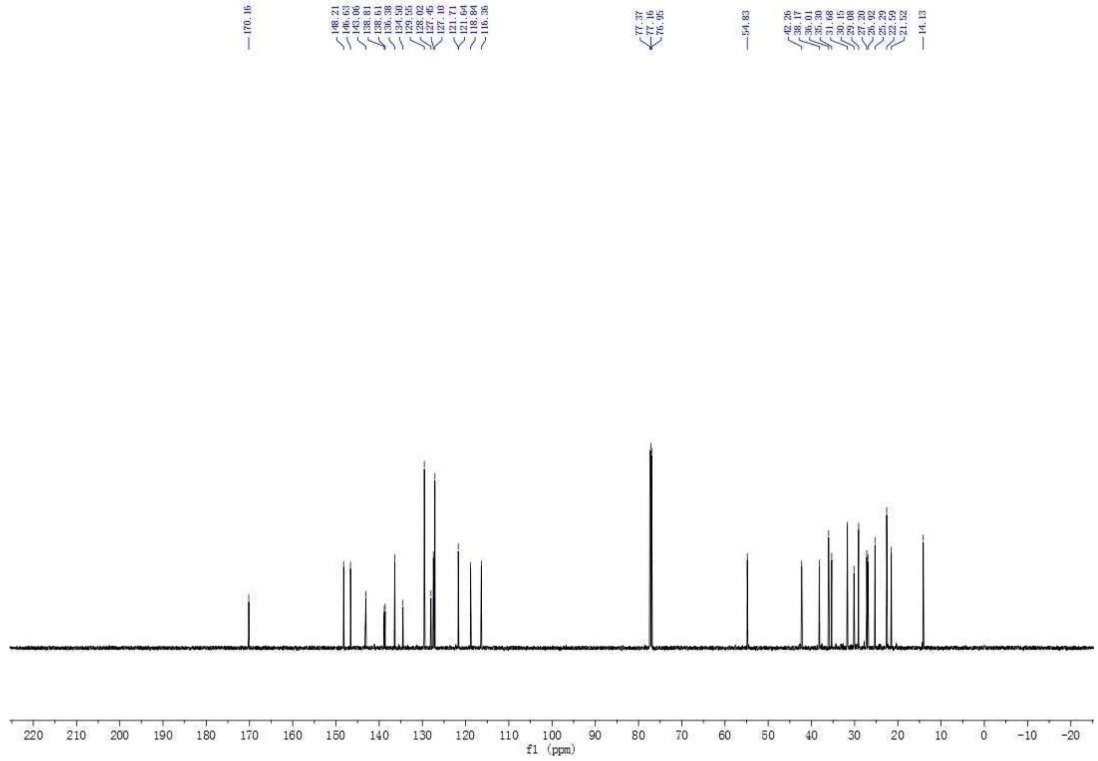

Supplementary Fig. 137. ^{13}C NMR of compound 3l. The sample has been recorded in 150 MHz, $CDCl_3$ at 25 °C

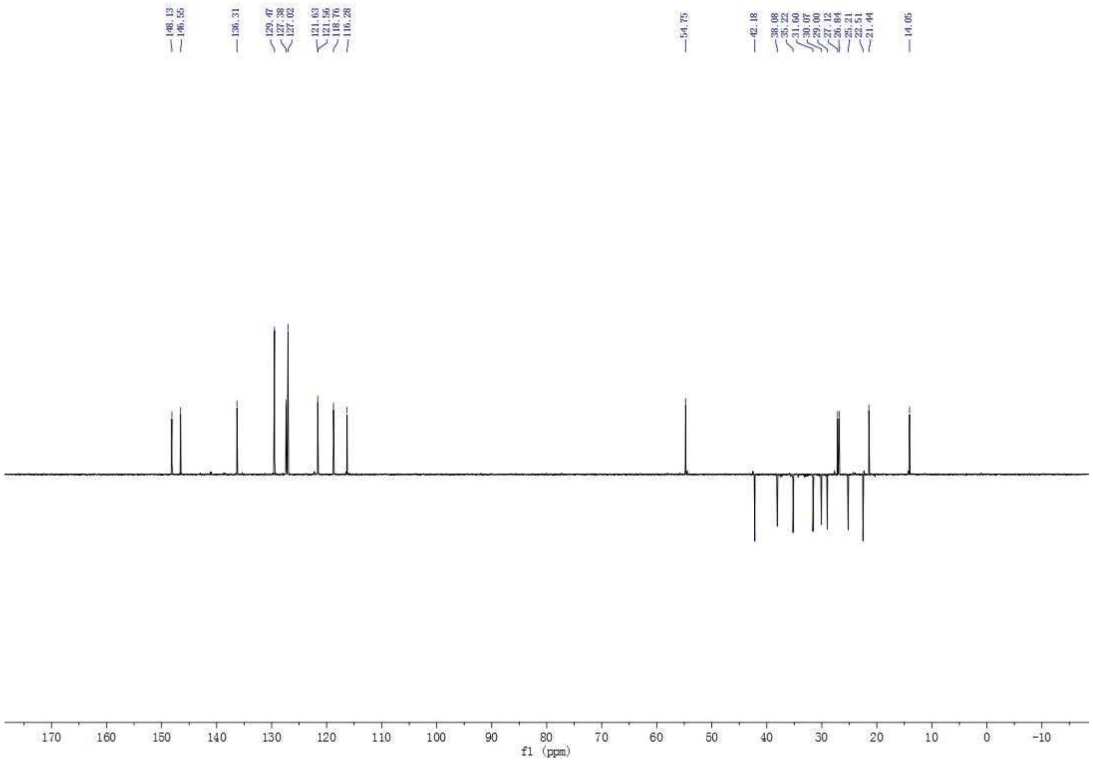

Supplementary Fig. 138. Dept 135° of compound 3l. The sample has been recorded in 150 MHz, $CDCl_3$ at 25 °C

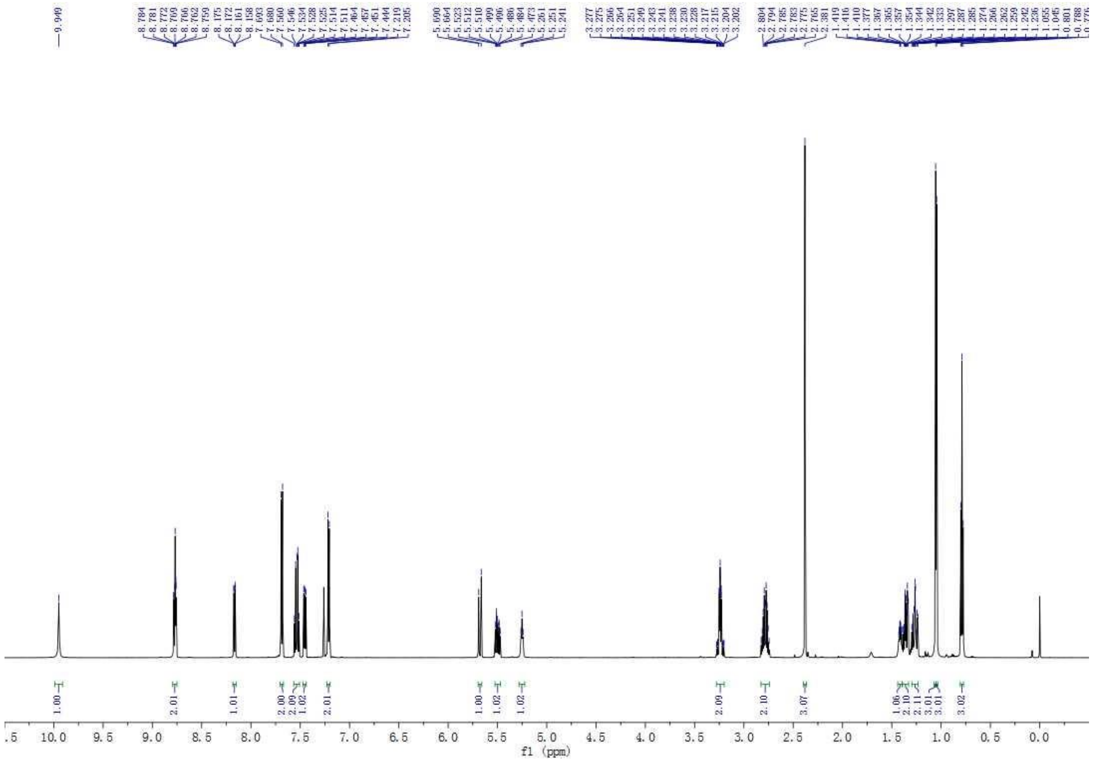

Supplementary Fig. 139. ^{1}H NMR of compound 3m. The sample has been recorded in 600 MHz, $CDCl_3$ at 25 °C

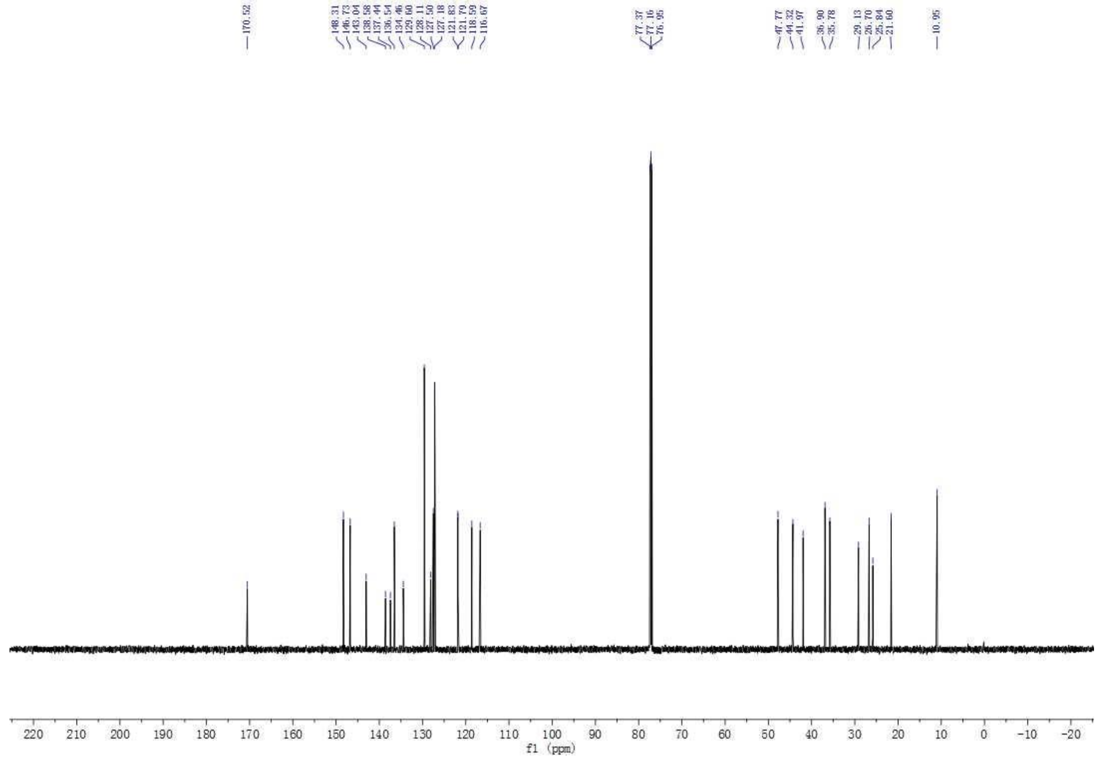

Supplementary Fig. 140. ^{13}C NMR of compound 3m. The sample has been recorded in 150 MHz, $CDCl_3$ at 25 °C

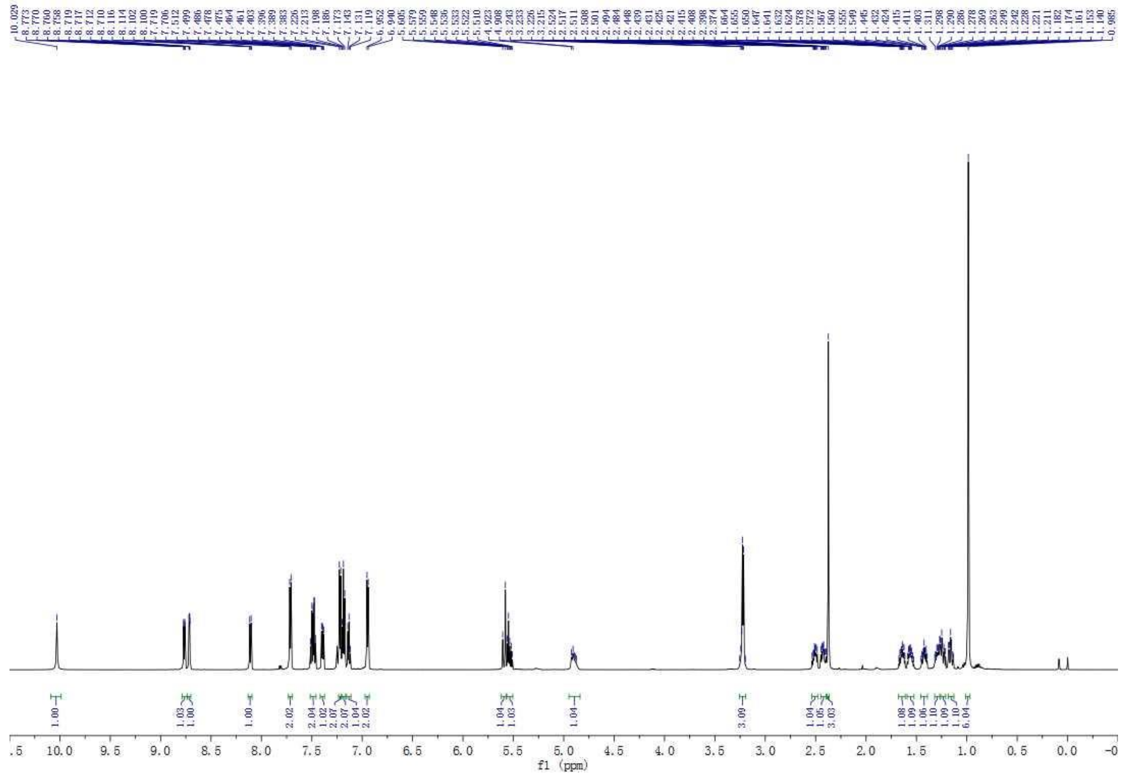

Supplementary Fig. 141. ^{1}H NMR of compound 3n. The sample has been recorded in 600 MHz, $CDCl_3$ at 25 °C

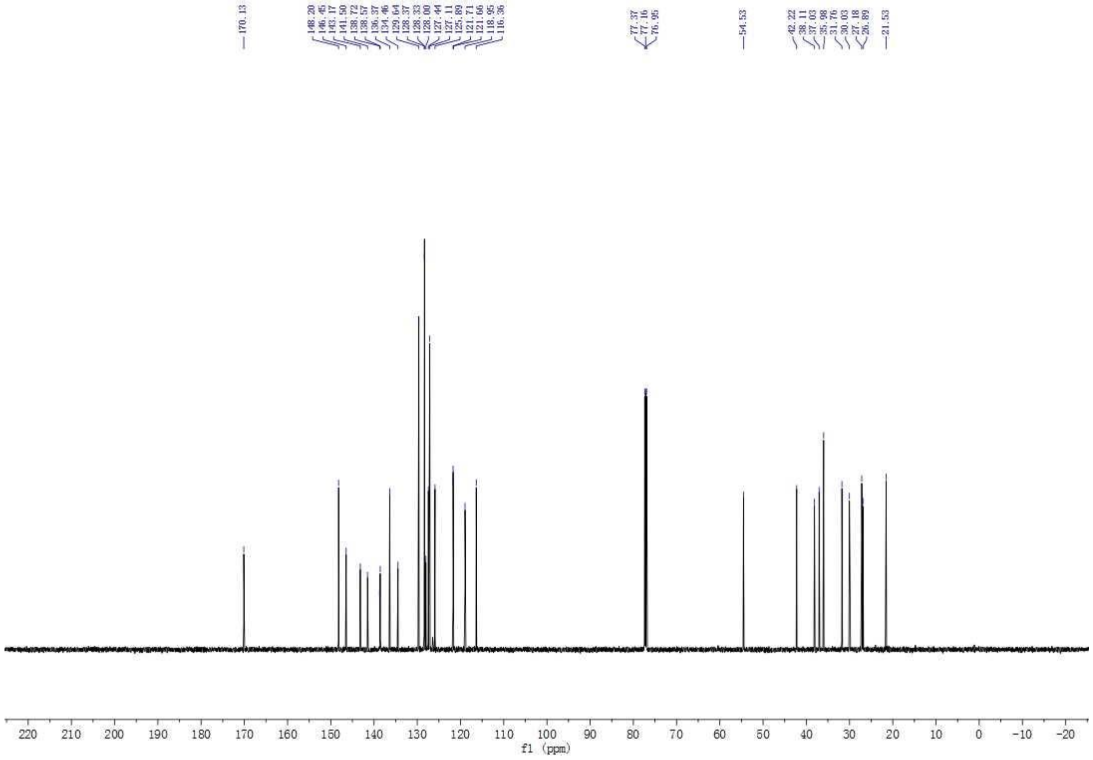

Supplementary Fig. 142. ^{13}C NMR of compound 3n. The sample has been recorded in 150 MHz, $CDCl_3$ at 25 °C

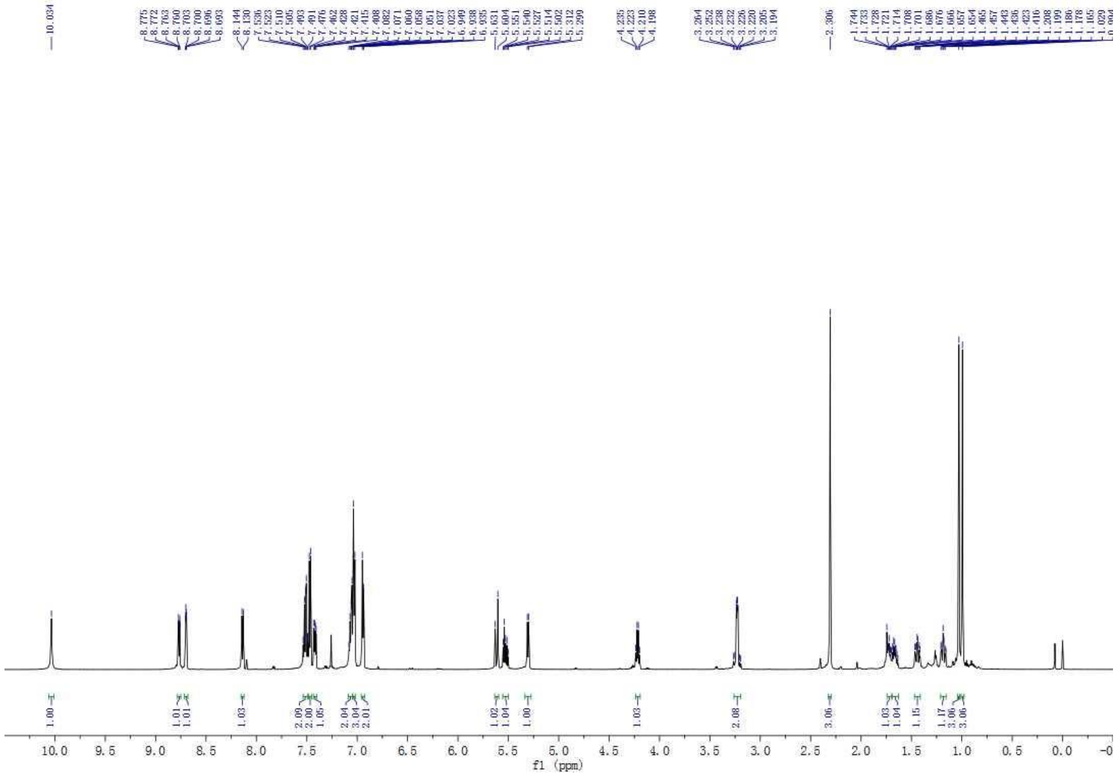

Supplementary Fig. 143. ^{1}H NMR of compound 3o. The sample has been recorded in 600 MHz, $CDCl_3$ at 25 °C

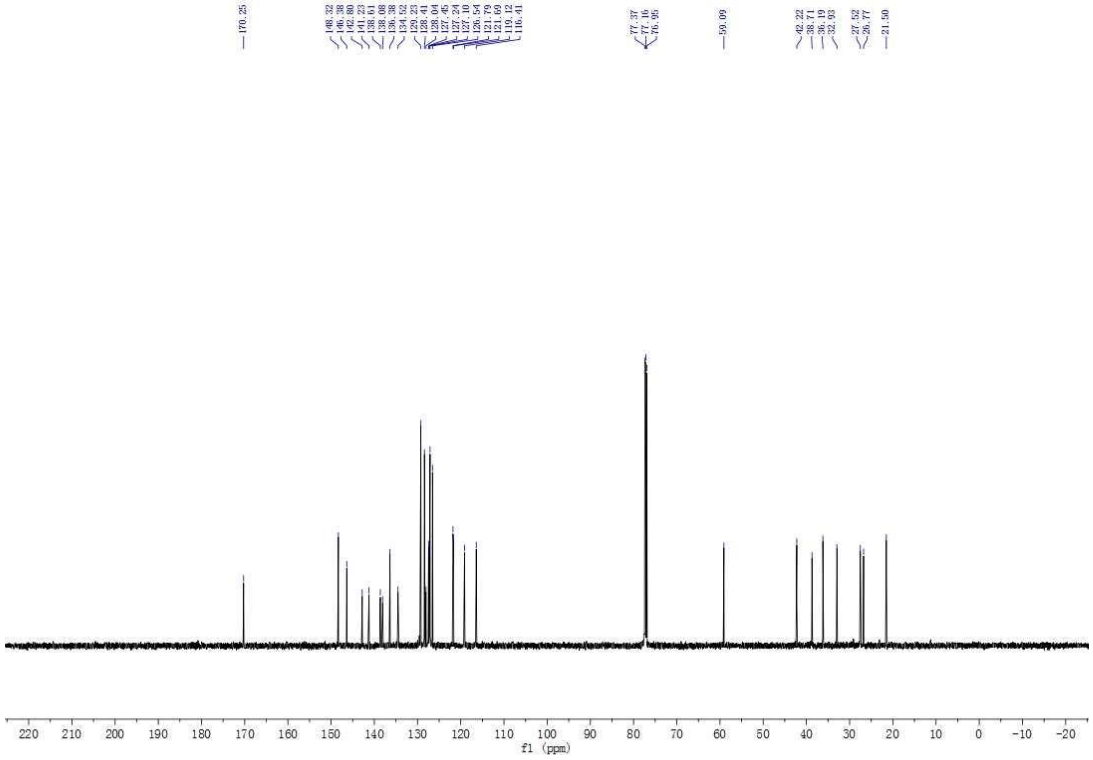

Supplementary Fig. 144. ^{13}C NMR of compound 3o. The sample has been recorded in 150 MHz, $CDCl_3$ at 25 °C

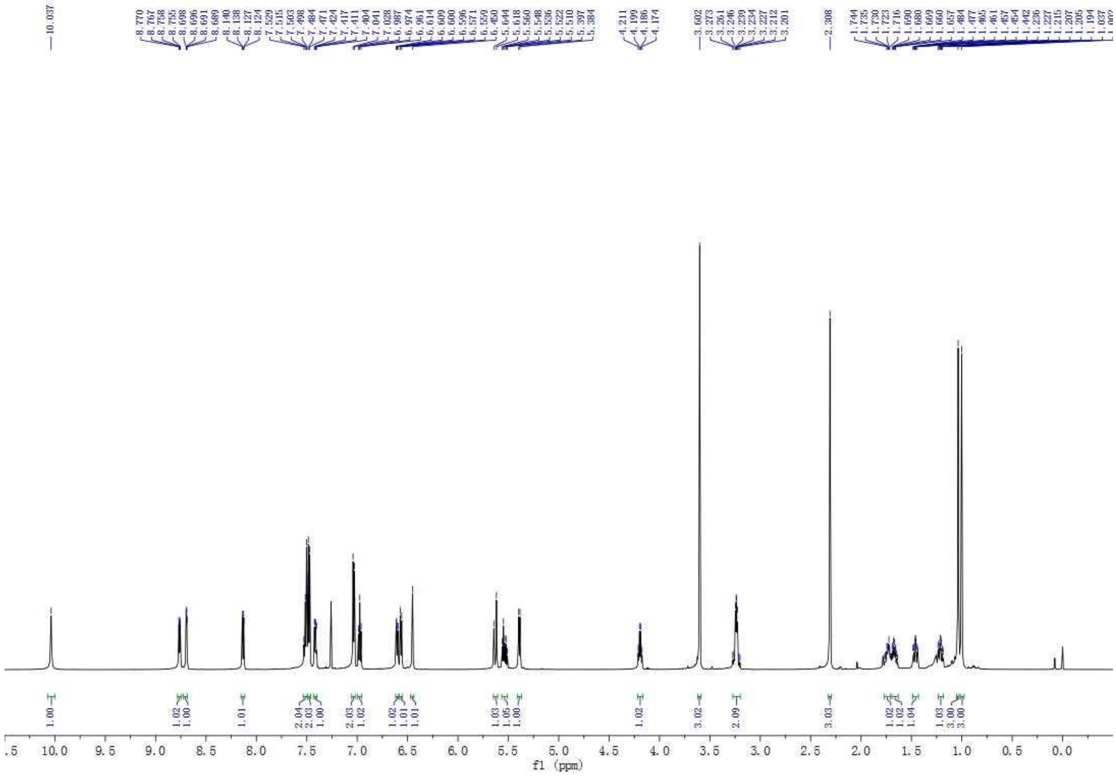

Supplementary Fig. 145. ^{1}H NMR of compound 3p. The sample has been recorded in 600 MHz, $CDCl_3$ at 25 °C

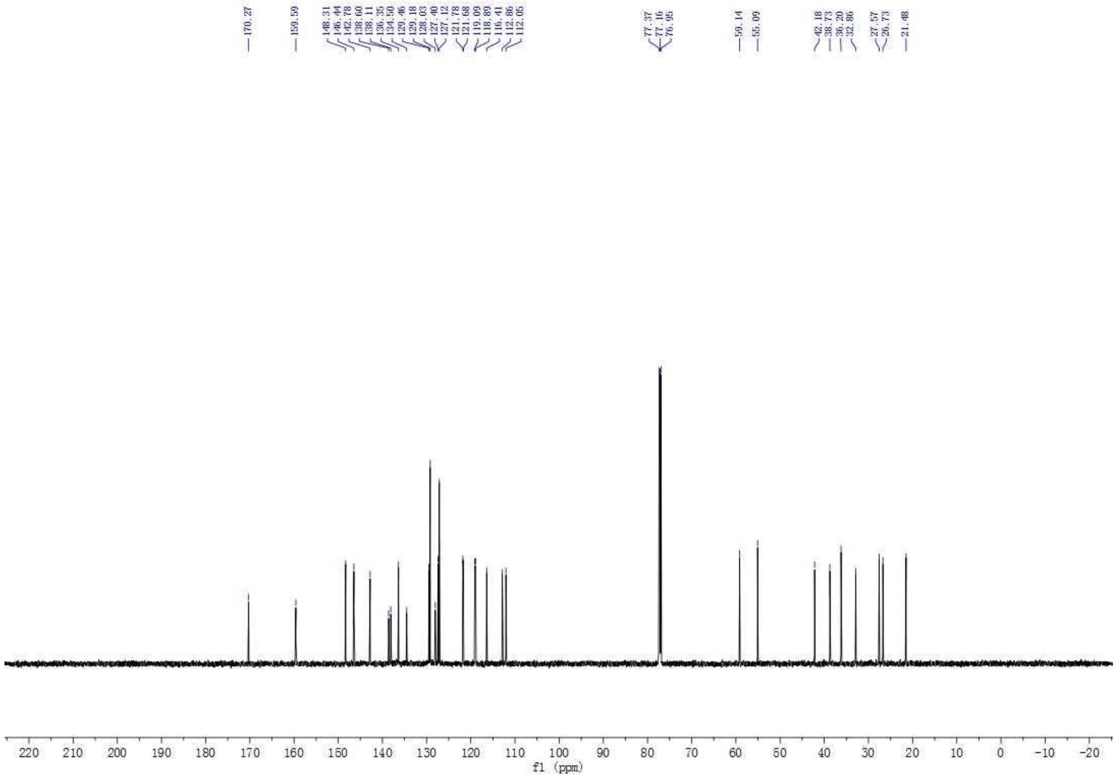

Supplementary Fig. 146. ^{13}C NMR of compound 3p. The sample has been recorded in 150 MHz, $CDCl_3$ at 25 °C

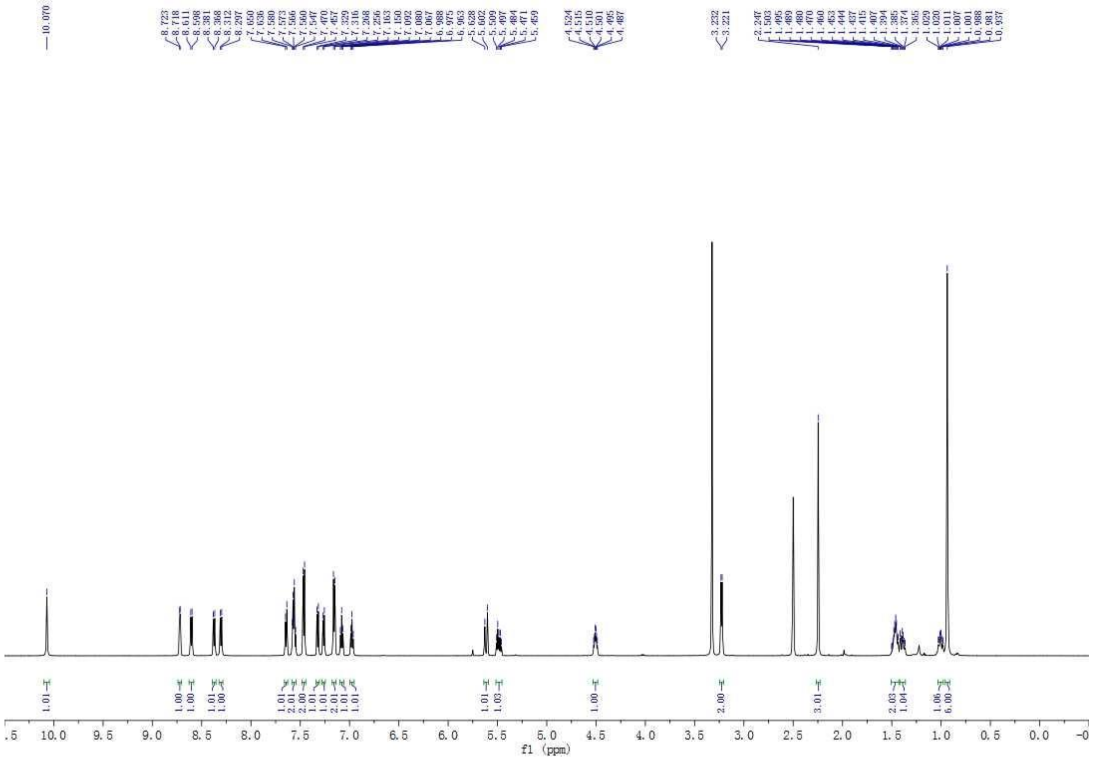

Supplementary Fig. 147. ^{1}H NMR of compound 3q. The sample has been recorded in 600 MHz, DMSO at 25 °C

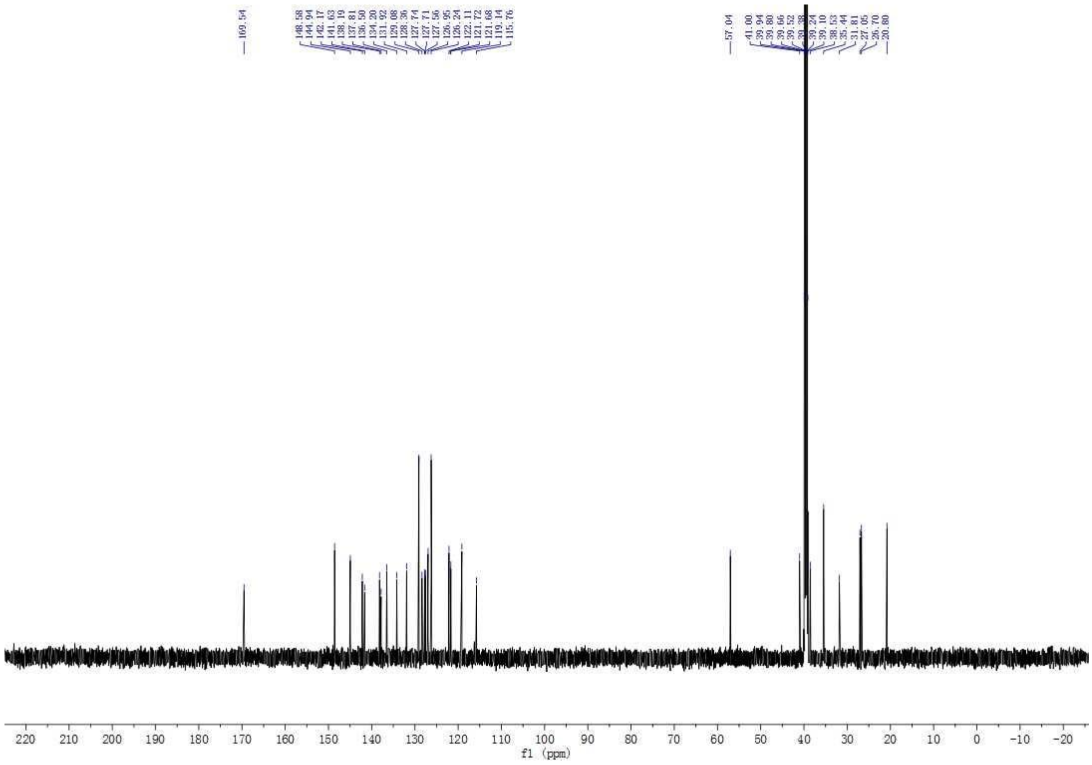

Supplementary Fig. 148. ^{13}C NMR of compound 3q. The sample has been recorded in 150 MHz, DMSO at 25 °C

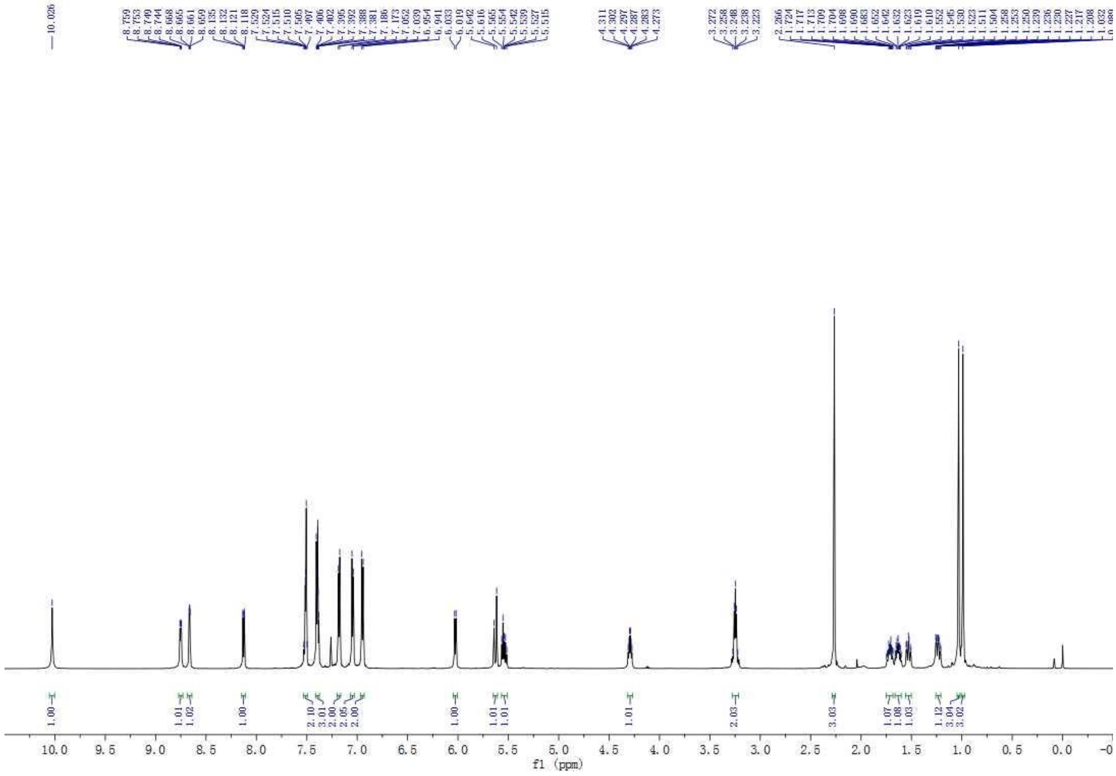

Supplementary Fig. 149. ^{1}H NMR of compound 3r. The sample has been recorded in 600 MHz, $CDCl_3$ at 25 °C

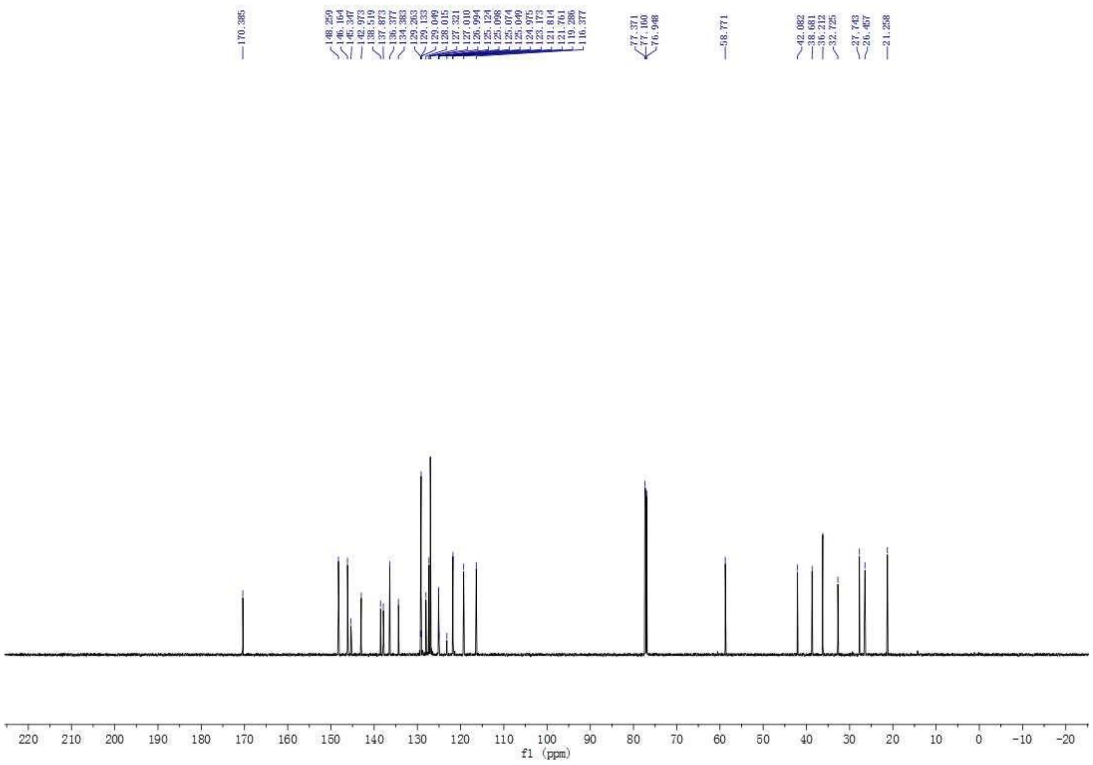

Supplementary Fig. 150. ^{1}H NMR of compound 3r. The sample has been recorded in 150 MHz, $CDCl_3$ at 25 °C

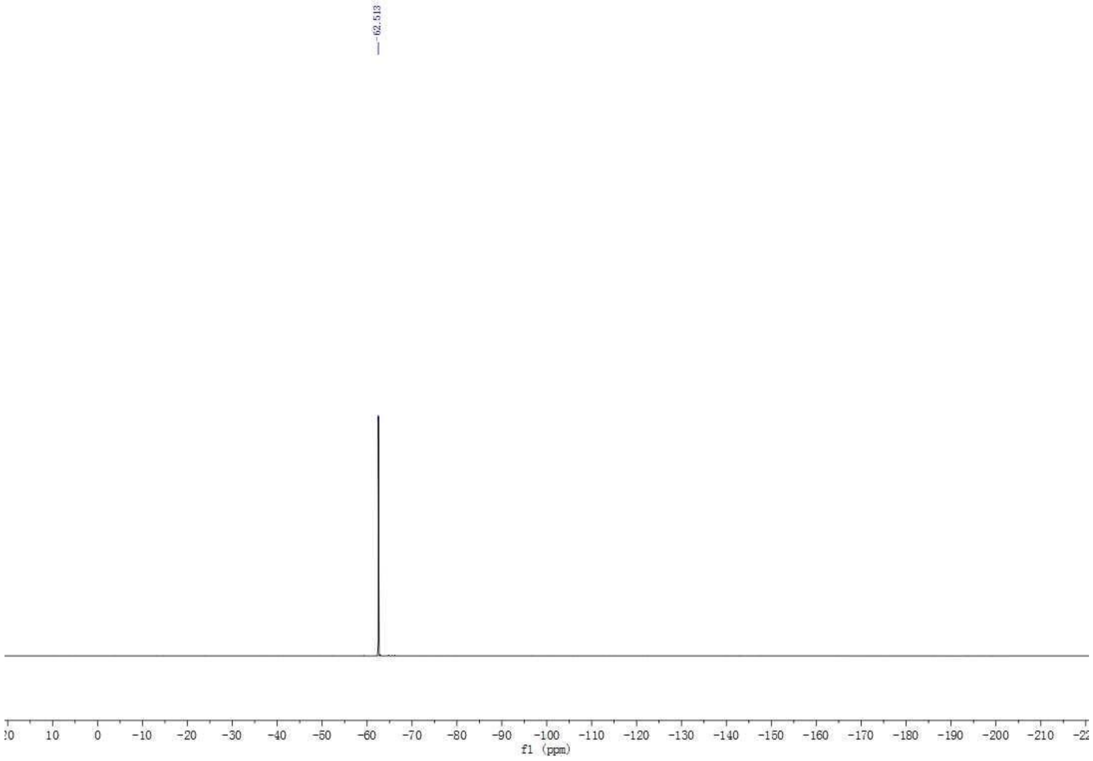

Supplementary Fig. 151. ^{19}F NMR of compound 3r. The sample has been recorded in 470 MHz, $CDCl_3$ at 25 °C

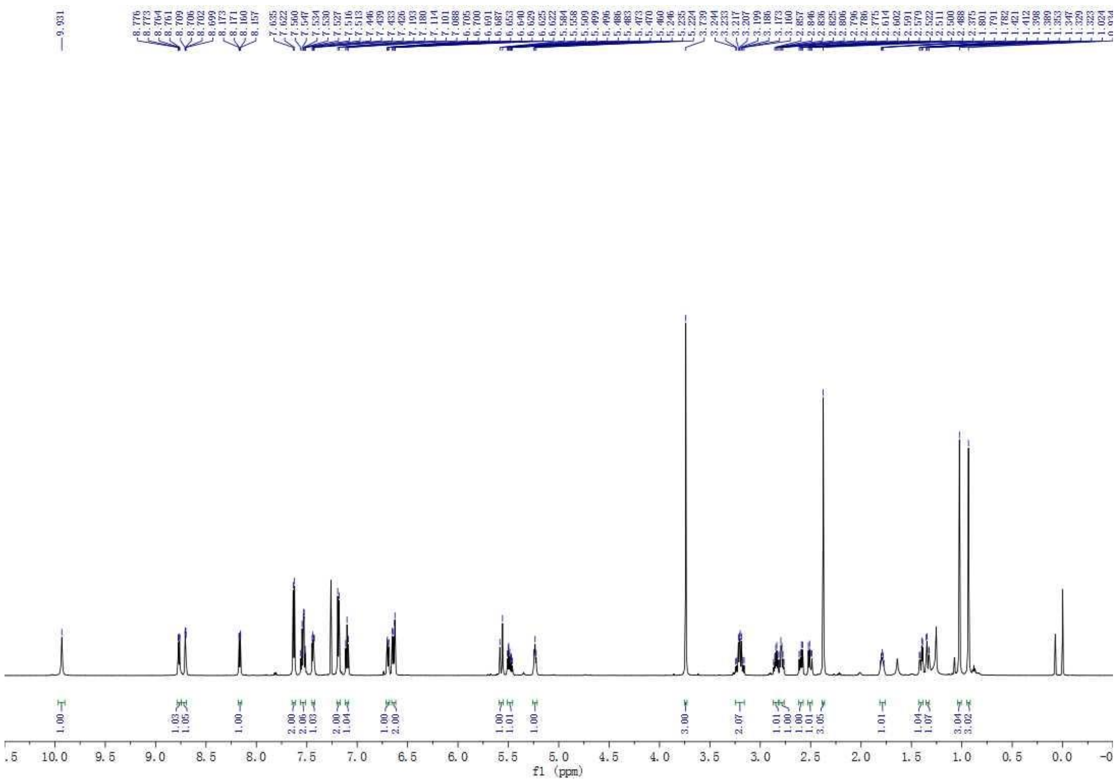

Supplementary Fig. 152. ^{1}H NMR of compound 3s. The sample has been recorded in 600 MHz, $CDCl_3$ at 25 °C

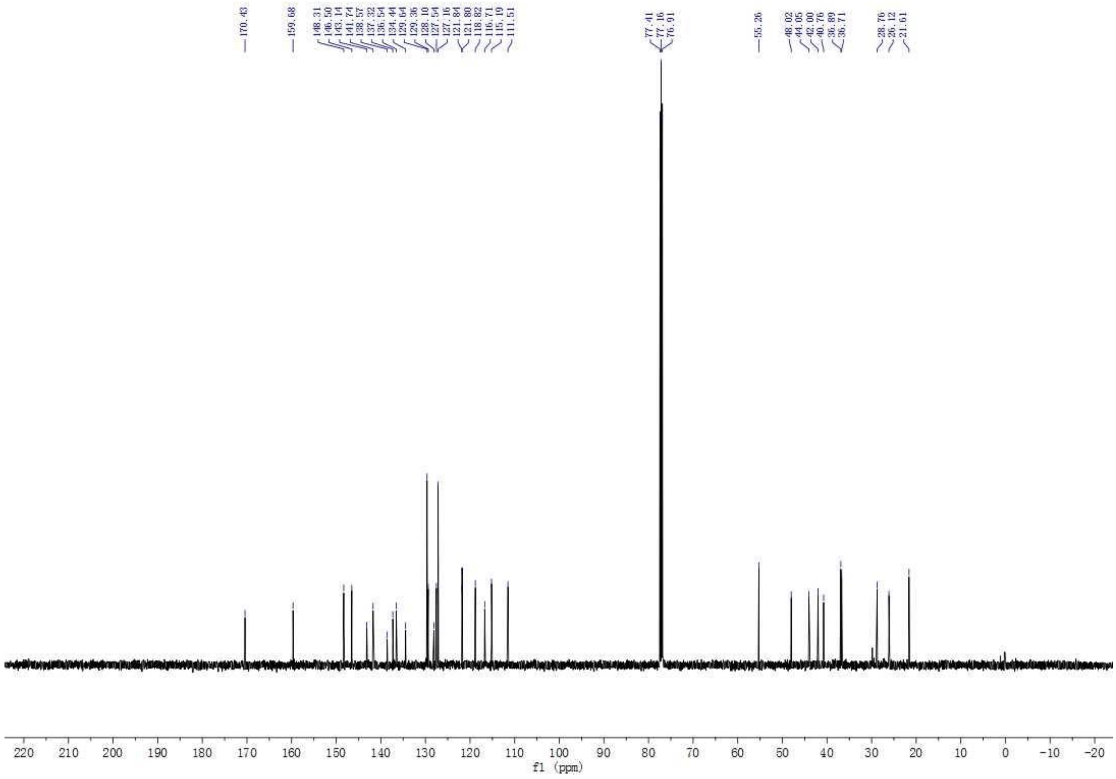

Supplementary Fig. 153. ^{13}C NMR of compound 3s. The sample has been recorded in 150 MHz, $CDCl_3$ at 25 °C

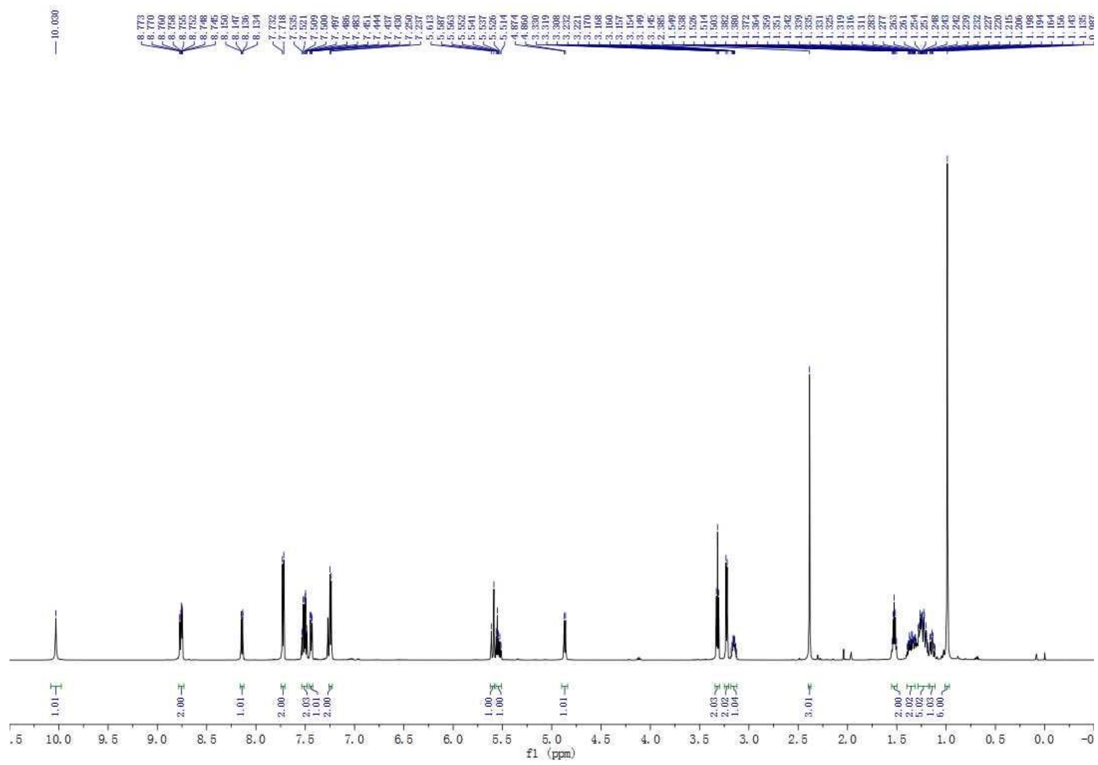

Supplementary Fig. 154. ^{1}H NMR of compound 3t. The sample has been recorded in 600 MHz, $CDCl_3$ at 25 °C

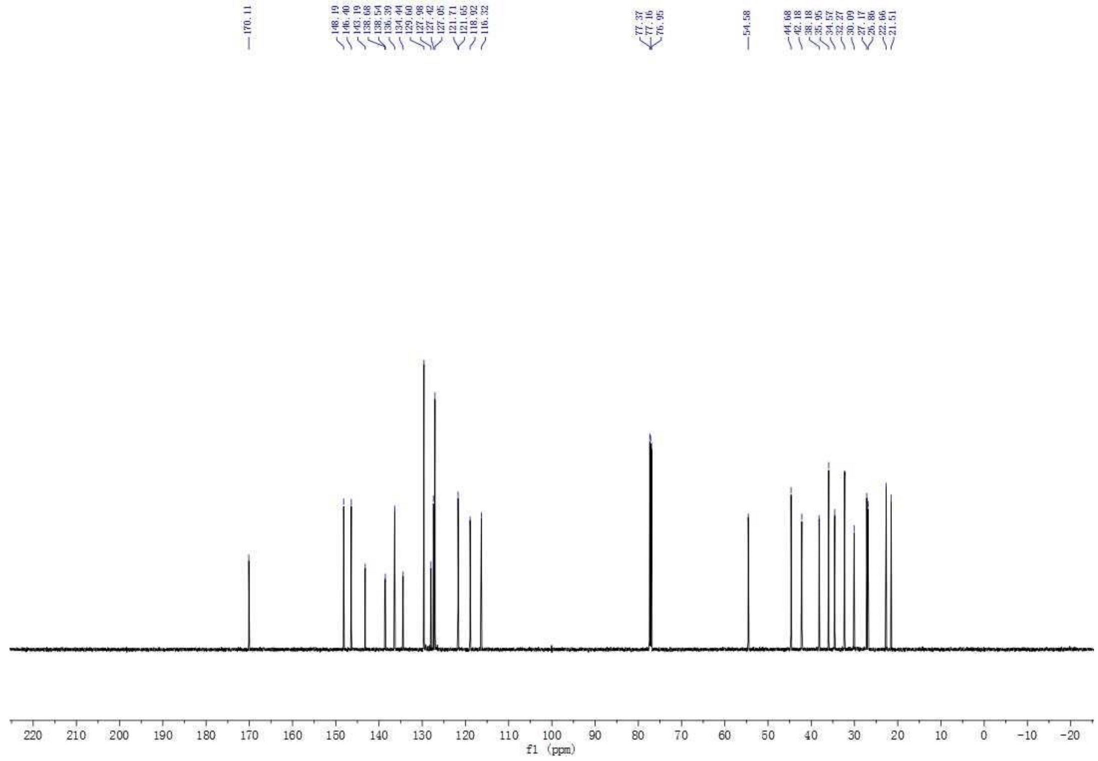

Supplementary Fig. 155. ^{13}C NMR of compound 3t. The sample has been recorded in 150 MHz, $CDCl_3$ at 25 °C

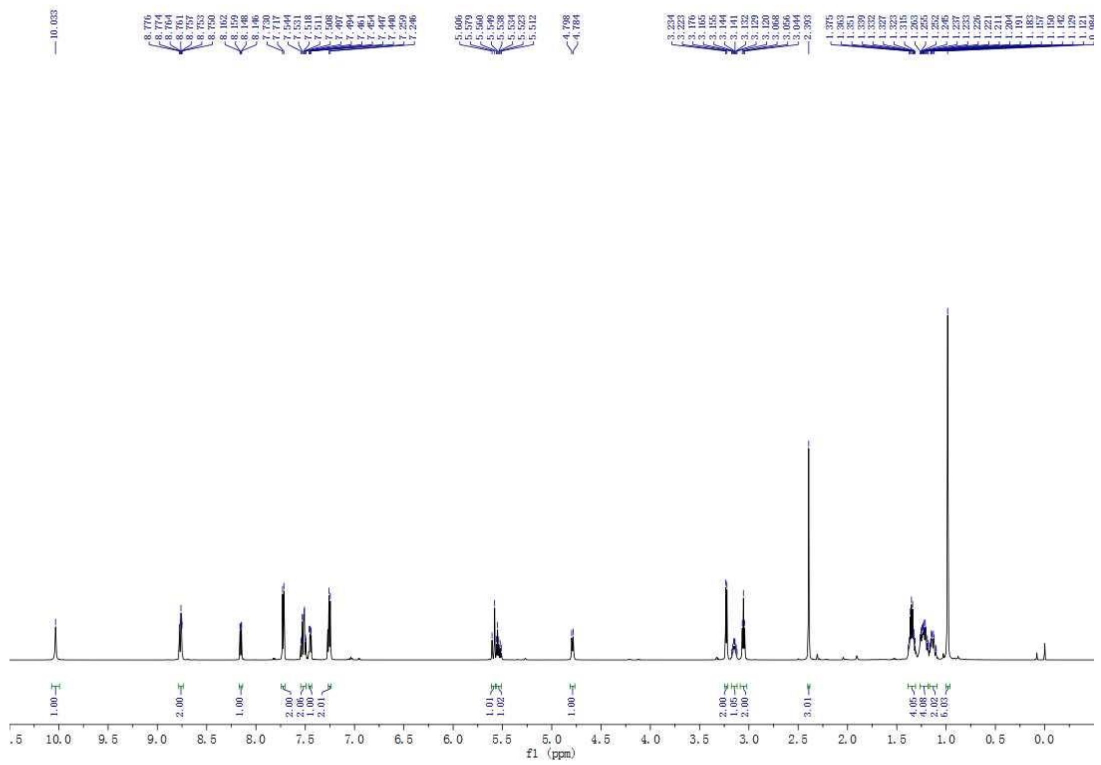

Supplementary Fig. 156. ^{1}H NMR of compound 3u. The sample has been recorded in 600 MHz, $CDCl_3$ at 25 °C

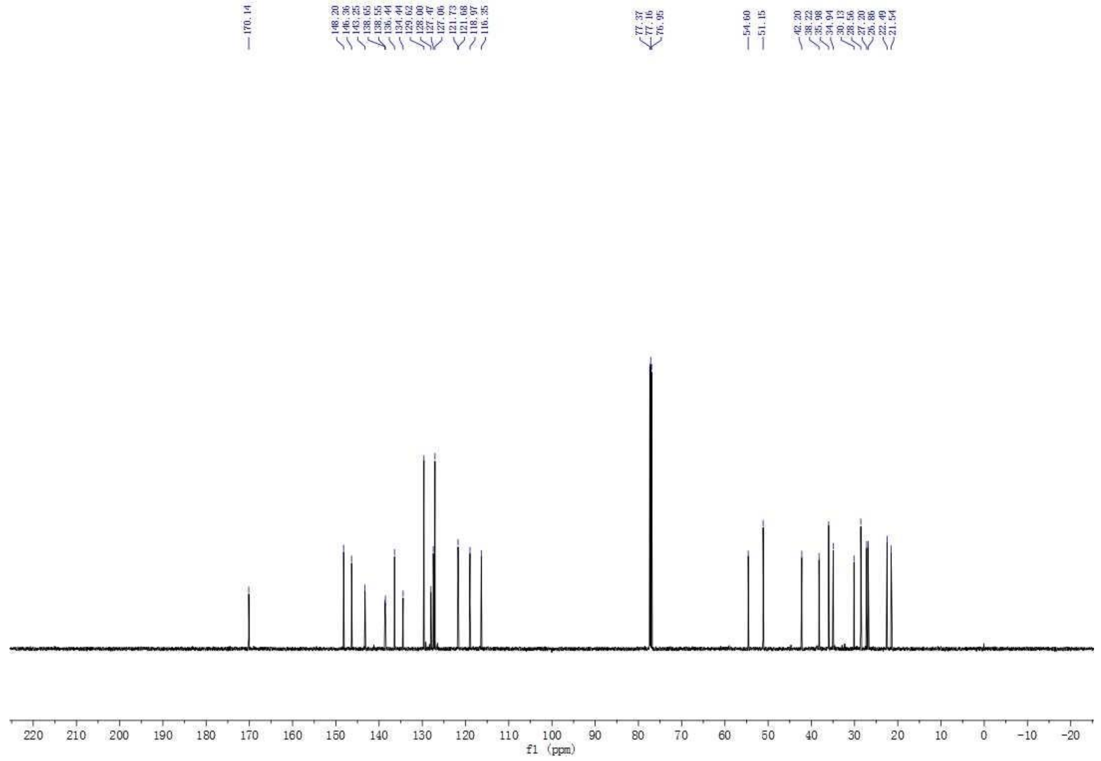

Supplementary Fig. 157. ^{13}C NMR of compound 3u. The sample has been recorded in 150 MHz, $CDCl_3$ at 25 °C

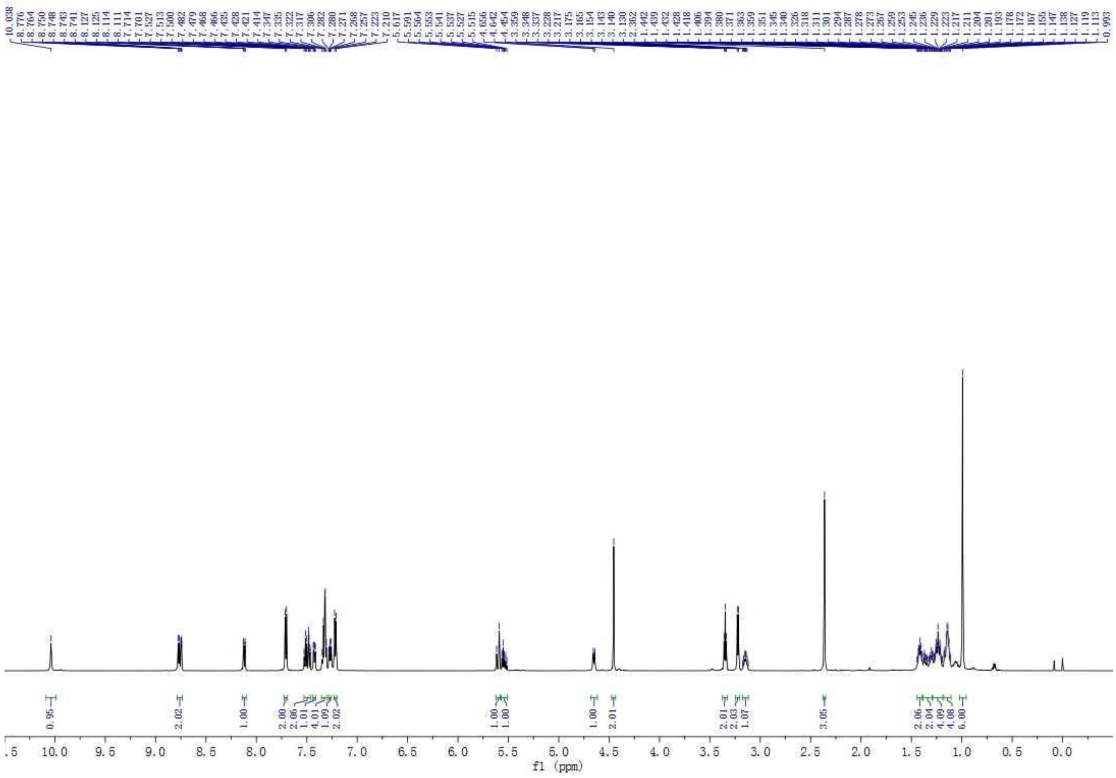

Supplementary Fig. 158. ^{1}H NMR of compound 3v. The sample has been recorded in 600 MHz, $CDCl_3$ at 25 °C

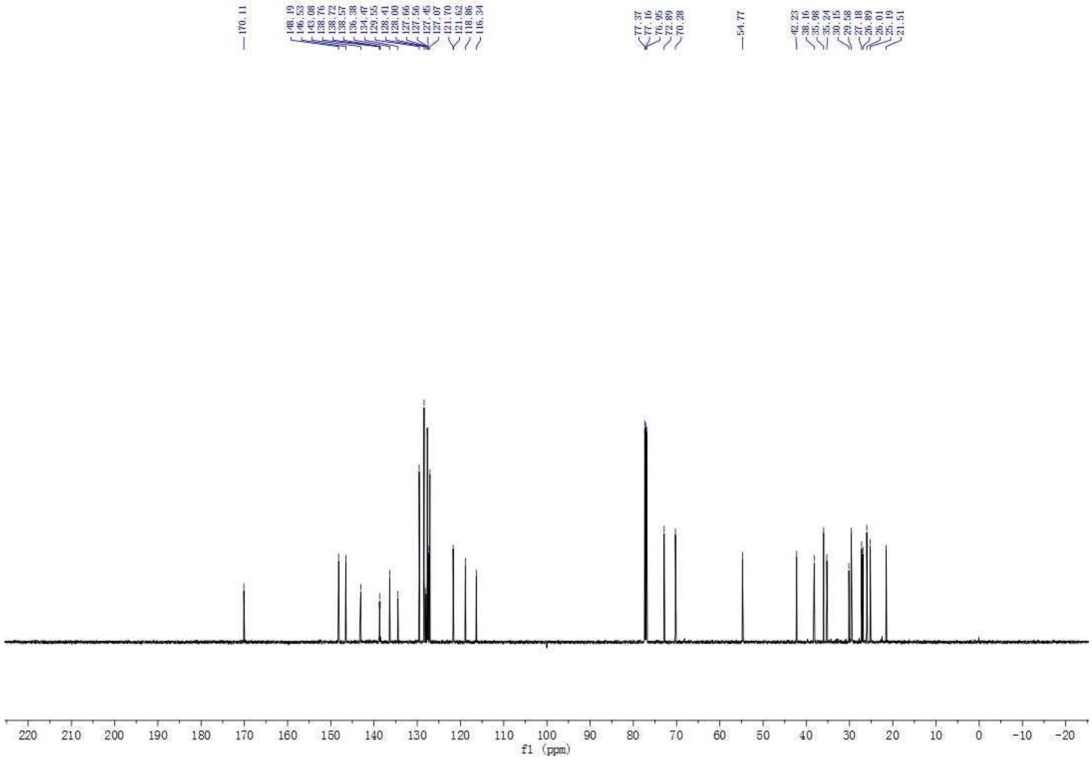

Supplementary Fig. 159. ^{13}C NMR of compound 3v. The sample has been recorded in 150 MHz, $CDCl_3$ at 25 °C

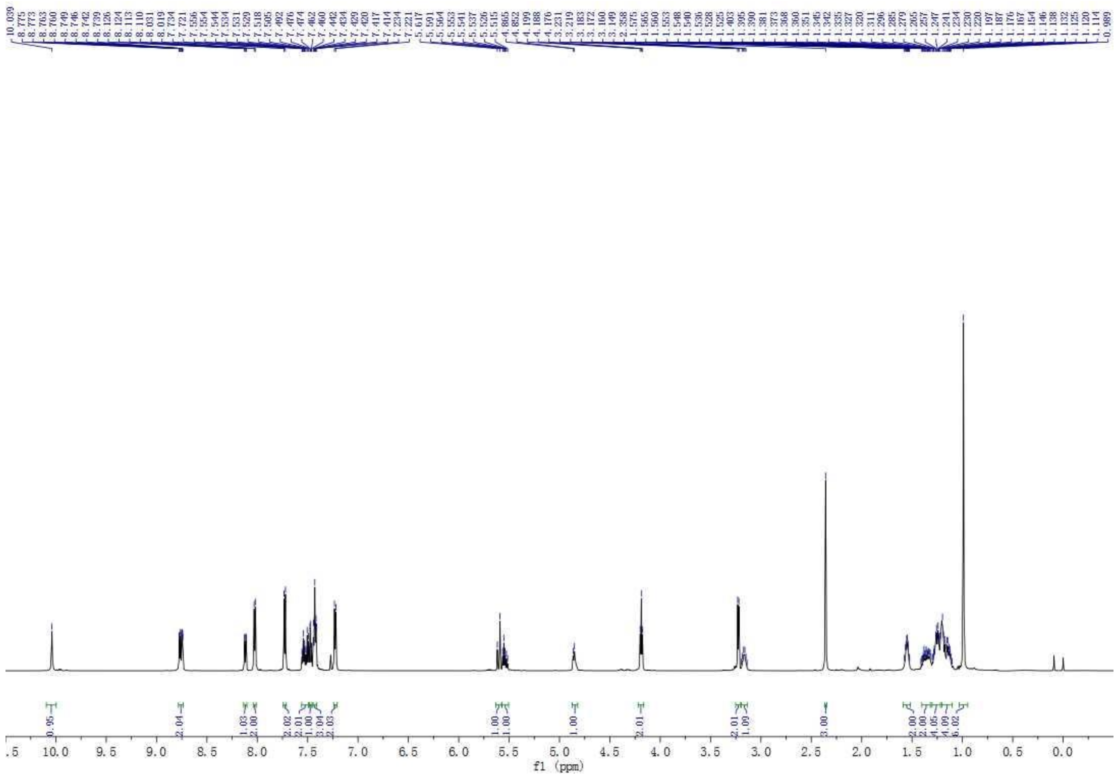

Supplementary Fig. 160. ^{1}H NMR of compound 3w. The sample has been recorded in 600 MHz, $CDCl_3$ at 25 °C

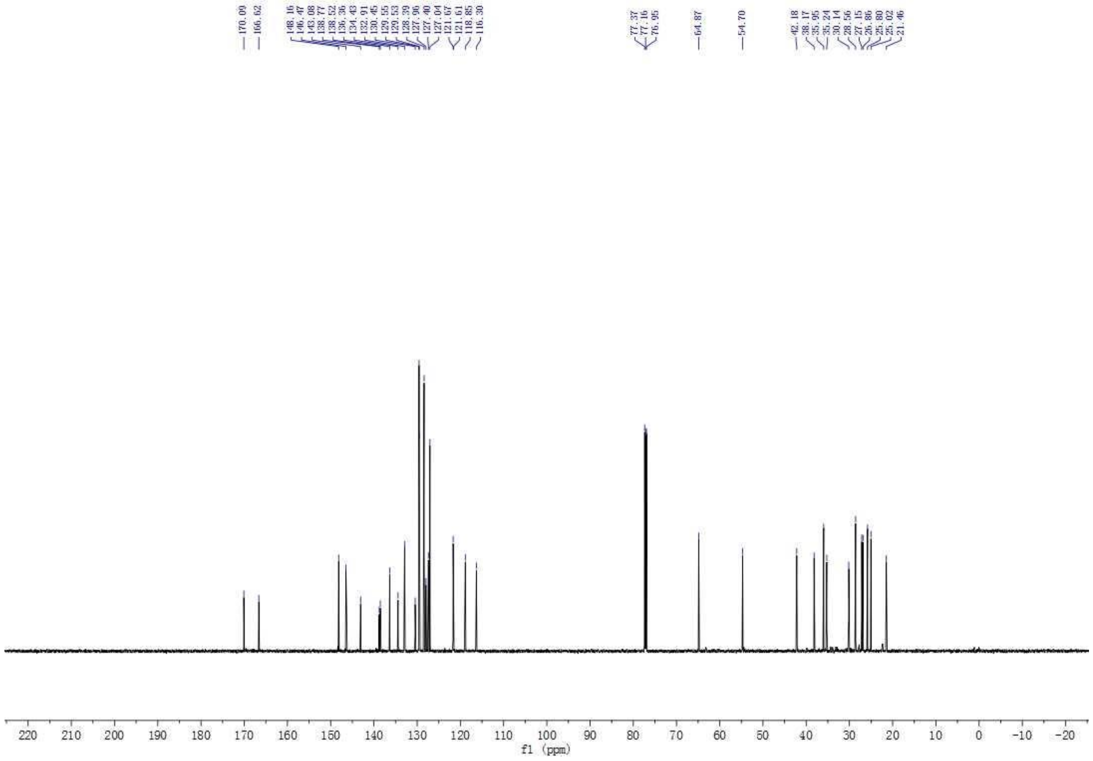

Supplementary Fig. 161. ^{13}C NMR of compound 3w. The sample has been recorded in 150 MHz, $CDCl_3$ at 25 °C

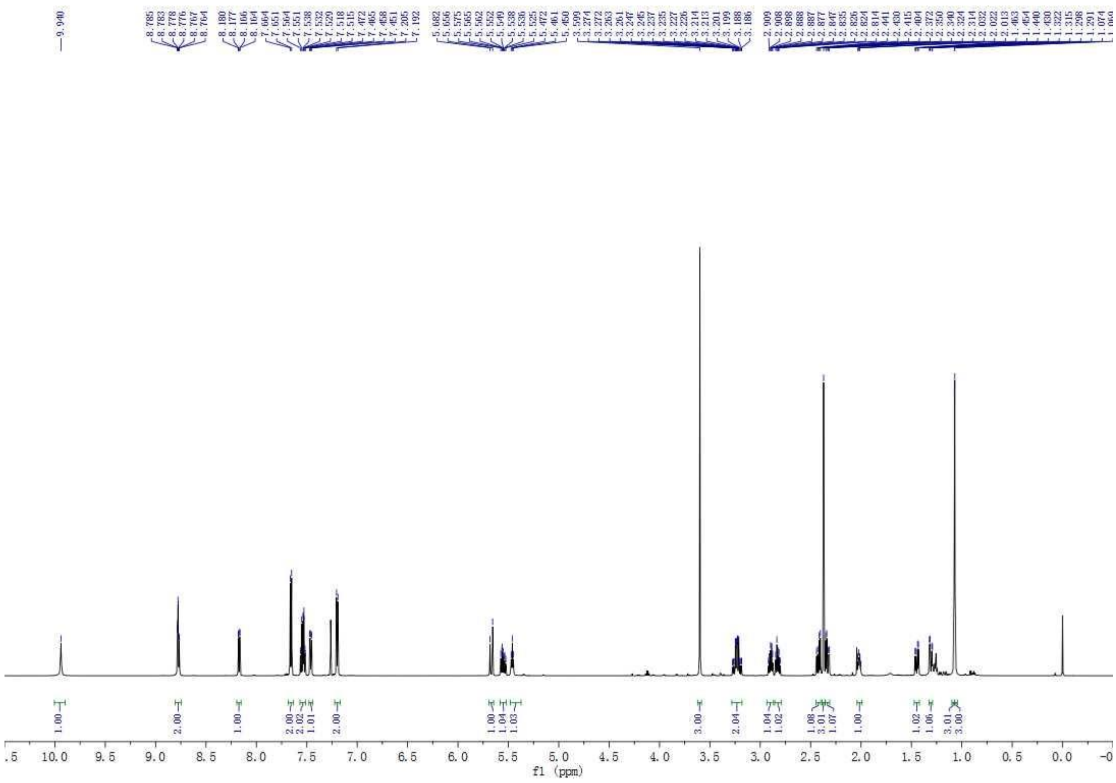

Supplementary Fig. 162. ^{1}H NMR of compound 3x. The sample has been recorded in 600 MHz, $CDCl_3$ at 25 °C

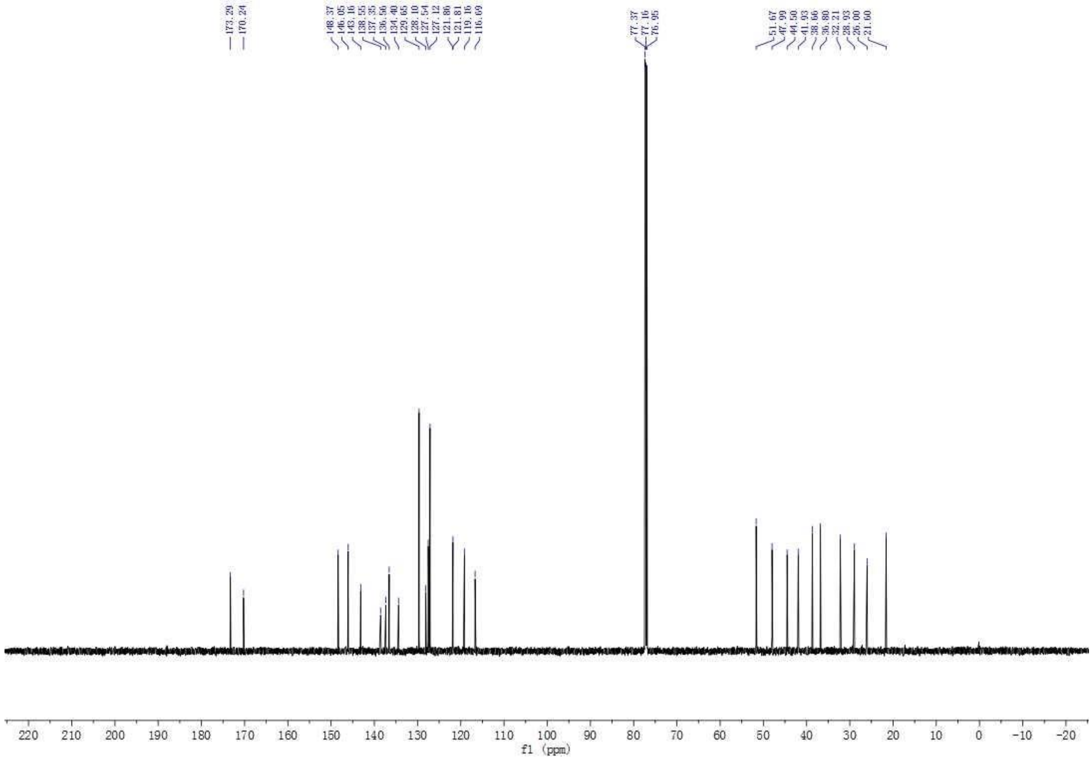

Supplementary Fig. 163. ^{13}C NMR of compound 3x. The sample has been recorded in 150 MHz, $CDCl_3$ at 25 °C

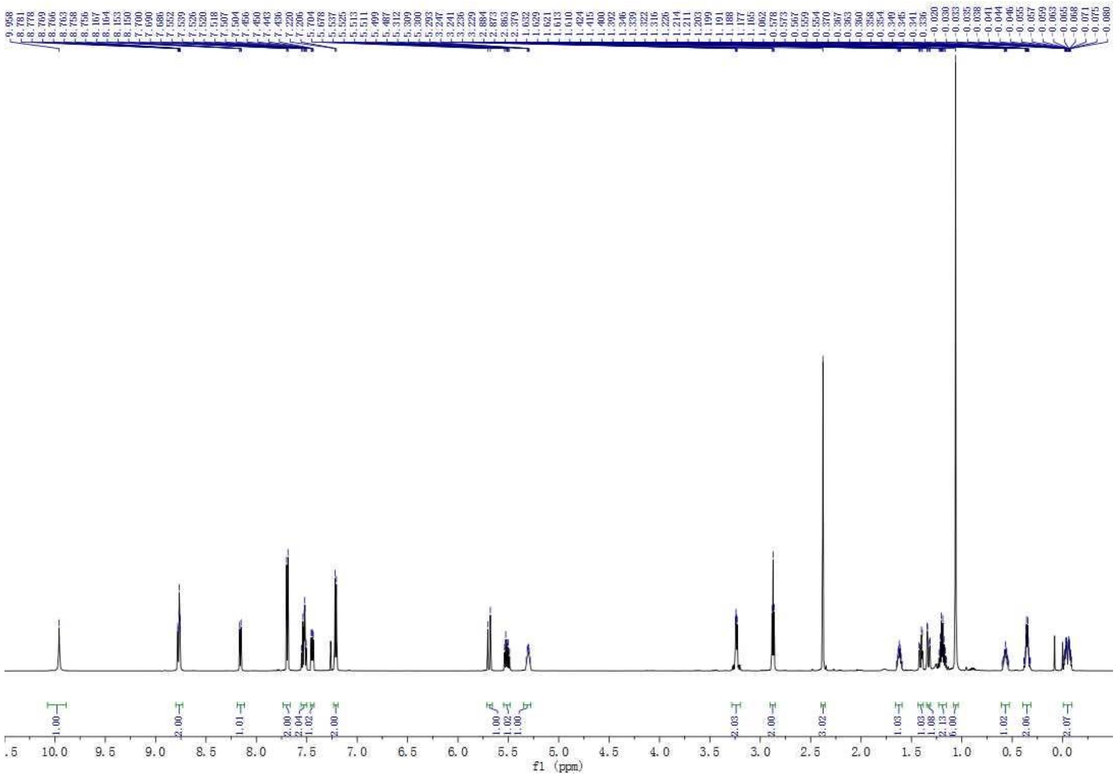

Supplementary Fig. 164. ^{1}H NMR of compound 3y. The sample has been recorded in 600 MHz, $CDCl_3$ at 25 °C

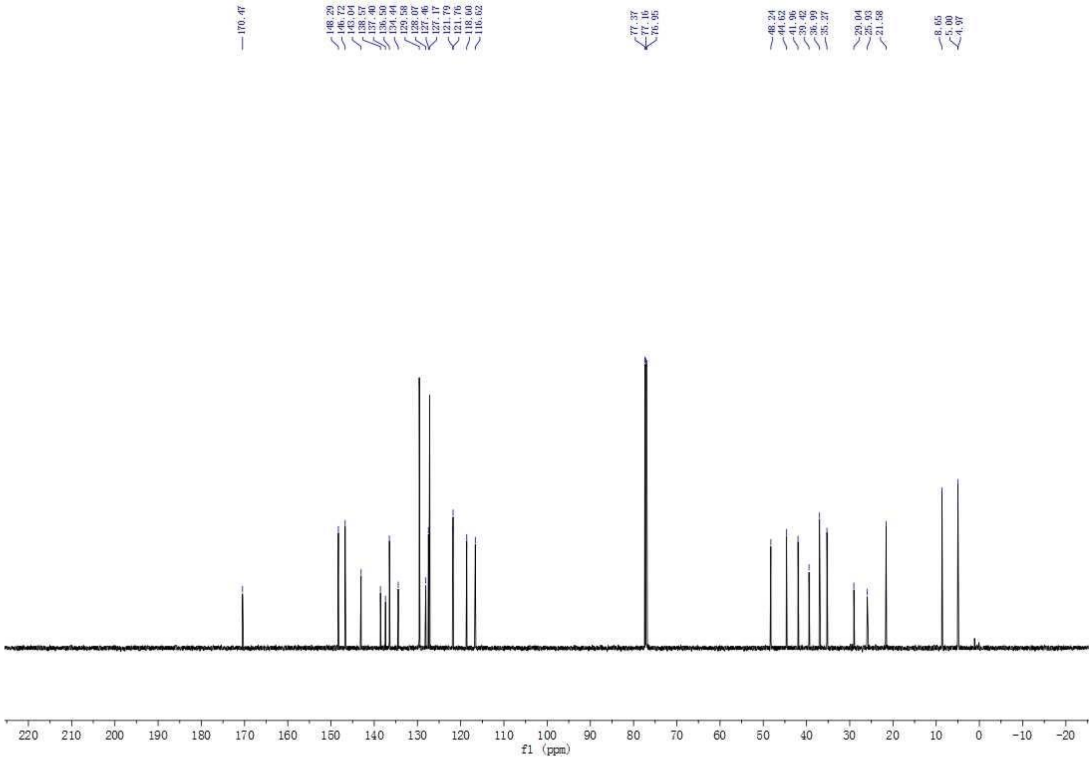

Supplementary Fig. 165. ^{13}C NMR of compound 3y. The sample has been recorded in 150 MHz, $CDCl_3$ at 25 °C

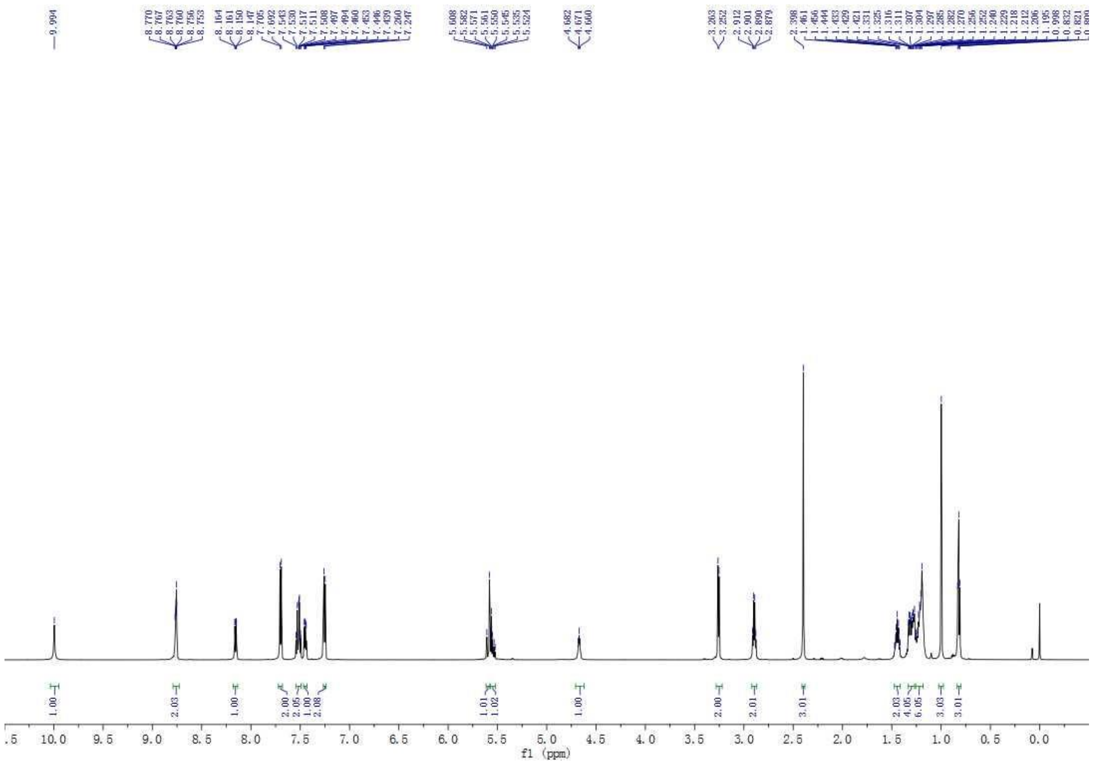

Supplementary Fig. 166. ^{1}H NMR of compound 3z. The sample has been recorded in 600 MHz, $CDCl_3$ at 25 °C

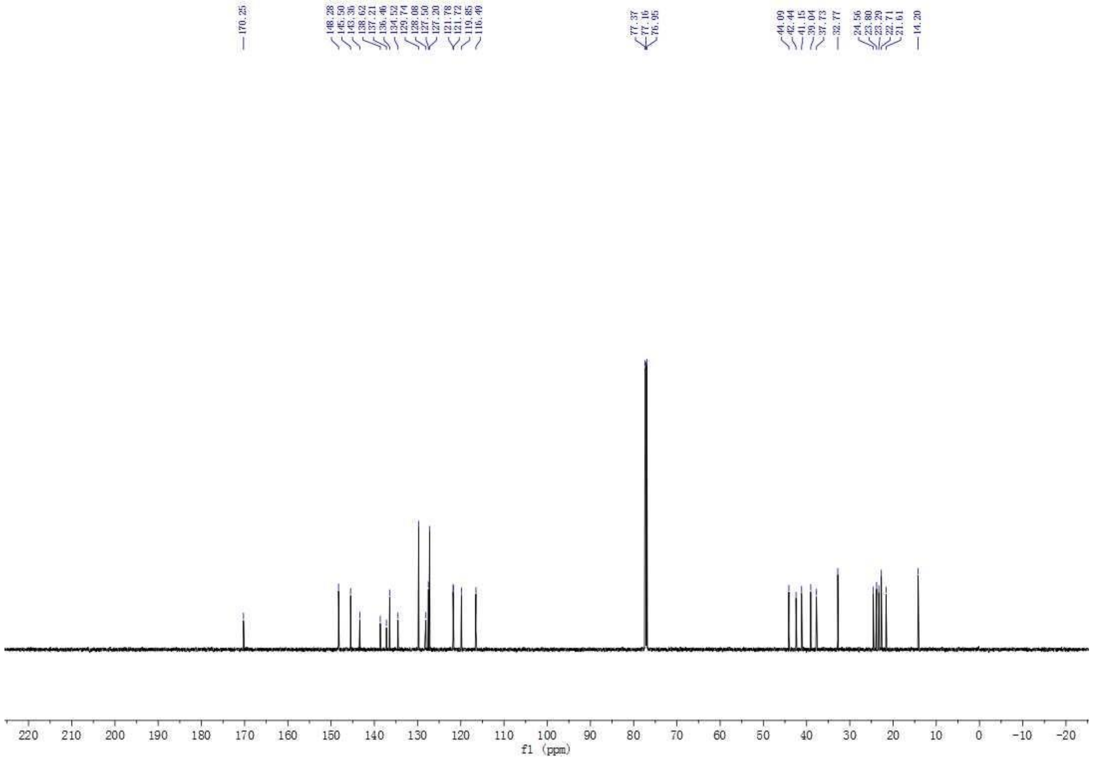

Supplementary Fig. 167. ^{13}C NMR of compound 3z. The sample has been recorded in 150 MHz, $CDCl_3$ at 25 °C

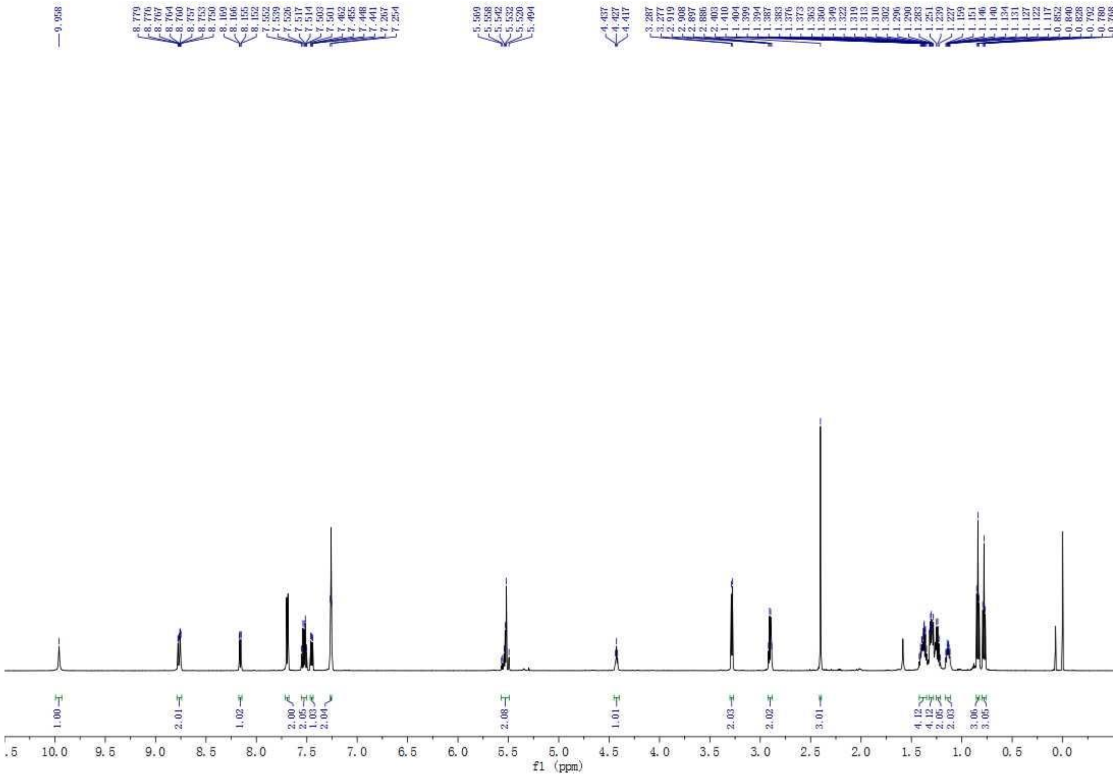

Supplementary Fig. 168. ^{1}H NMR of compound 3aa. The sample has been recorded in 600 MHz, $CDCl_3$ at 25 °C

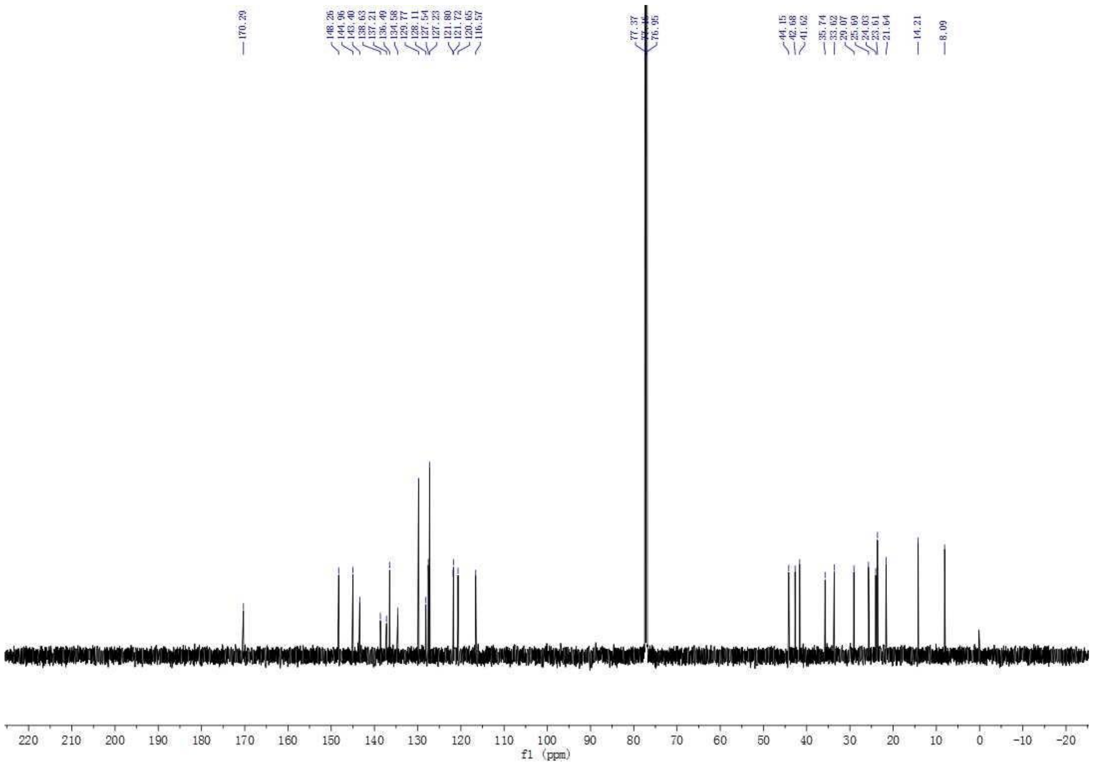

Supplementary Fig. 169. ^{13}C NMR of compound 3aa. The sample has been recorded in 150 MHz, $CDCl_3$ at 25 °C

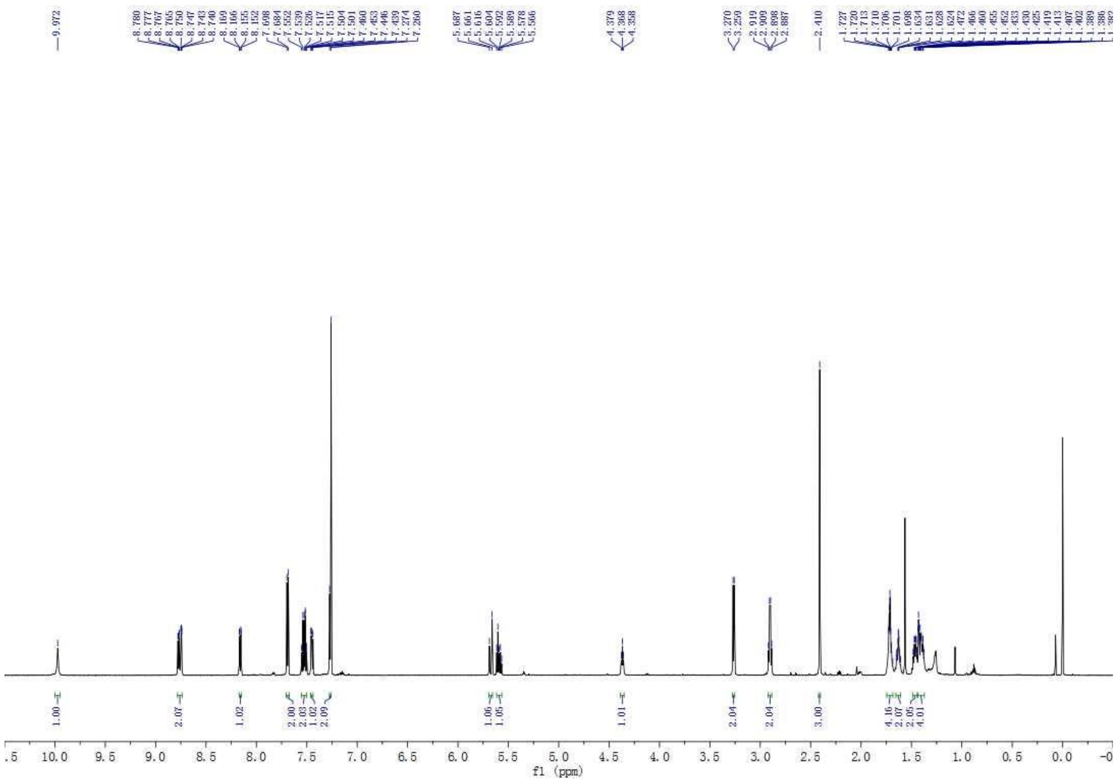

Supplementary Fig. 170. ¹H NMR of compound 3ab. The sample has been recorded in 600 MHz, $CDCl_3$ at 25 °C

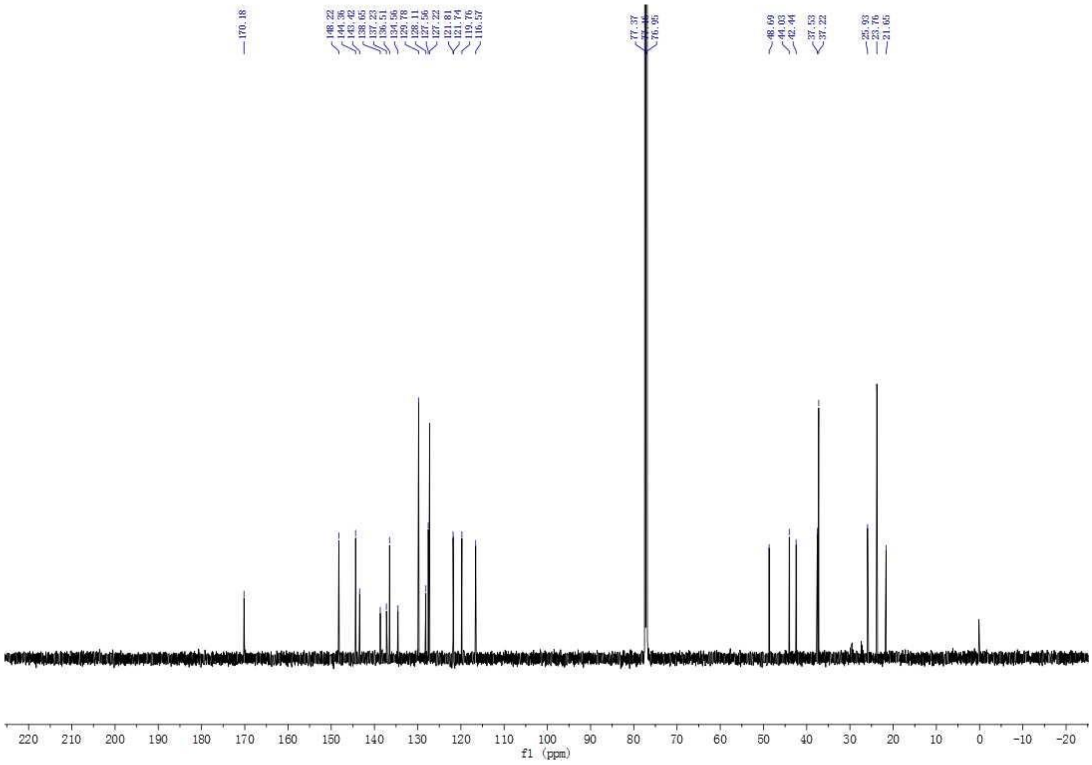

Supplementary Fig. 171. ¹³C NMR of compound 3ab. The sample has been recorded in 150 MHz, $CDCl_3$ at 25 °C

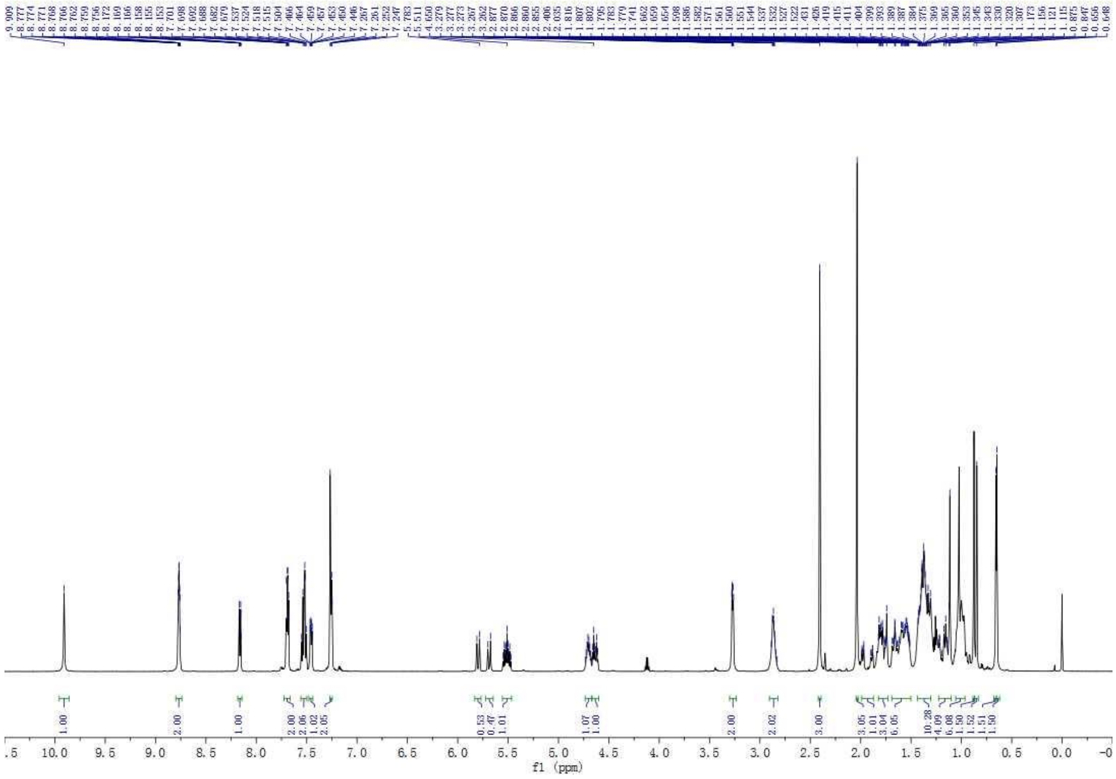

Supplementary Fig. 172. ^{1}H NMR of compound 3ac. The sample has been recorded in 600 MHz, $CDCl_3$ at 25 °C

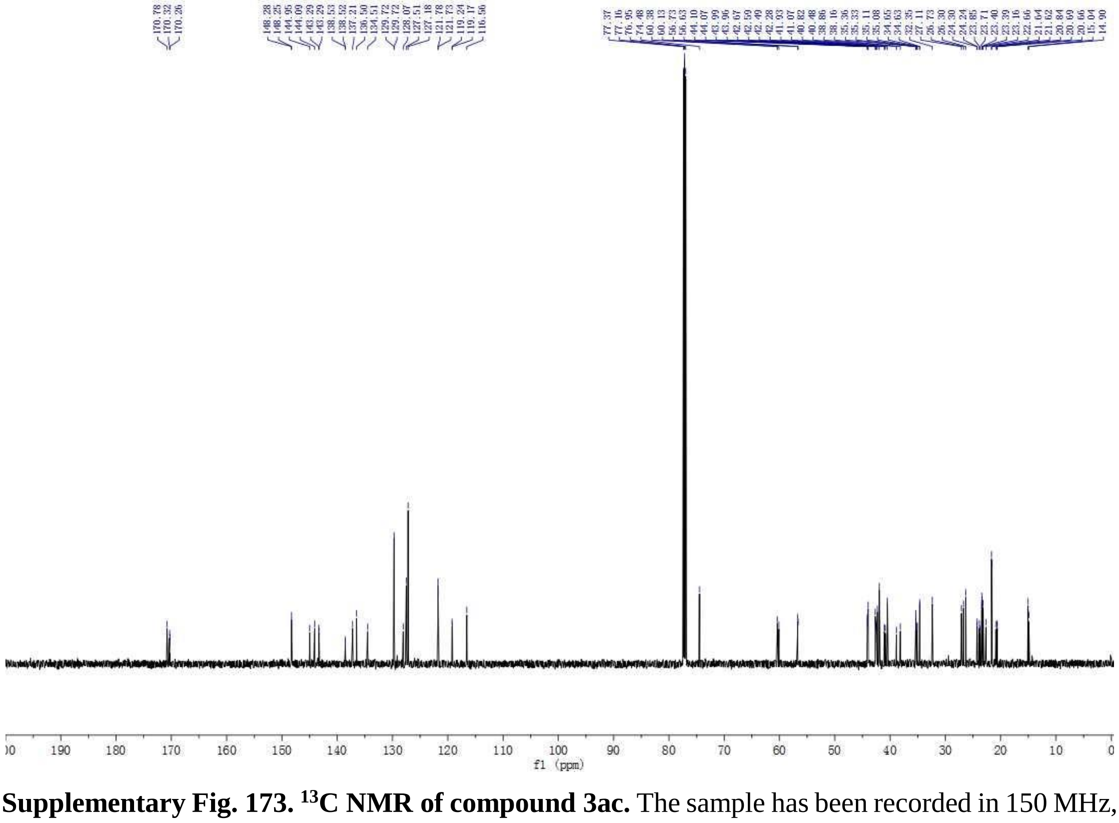

Supplementary Fig. 173. ^{13}C NMR of compound 3ac. The sample has been recorded in 150 MHz, $CDCl_3$ at 25 °C

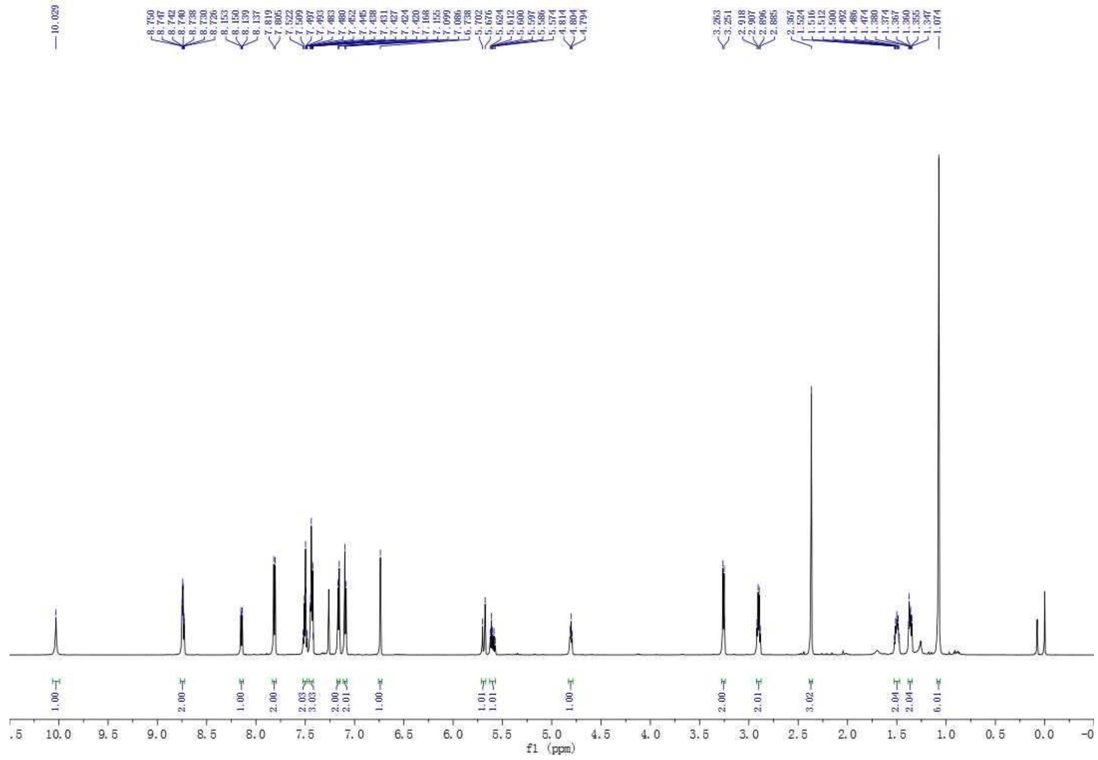

Supplementary Fig. 174. ^{1}H NMR of compound 3ad. The sample has been recorded in 600 MHz, $CDCl_3$ at 25 °C

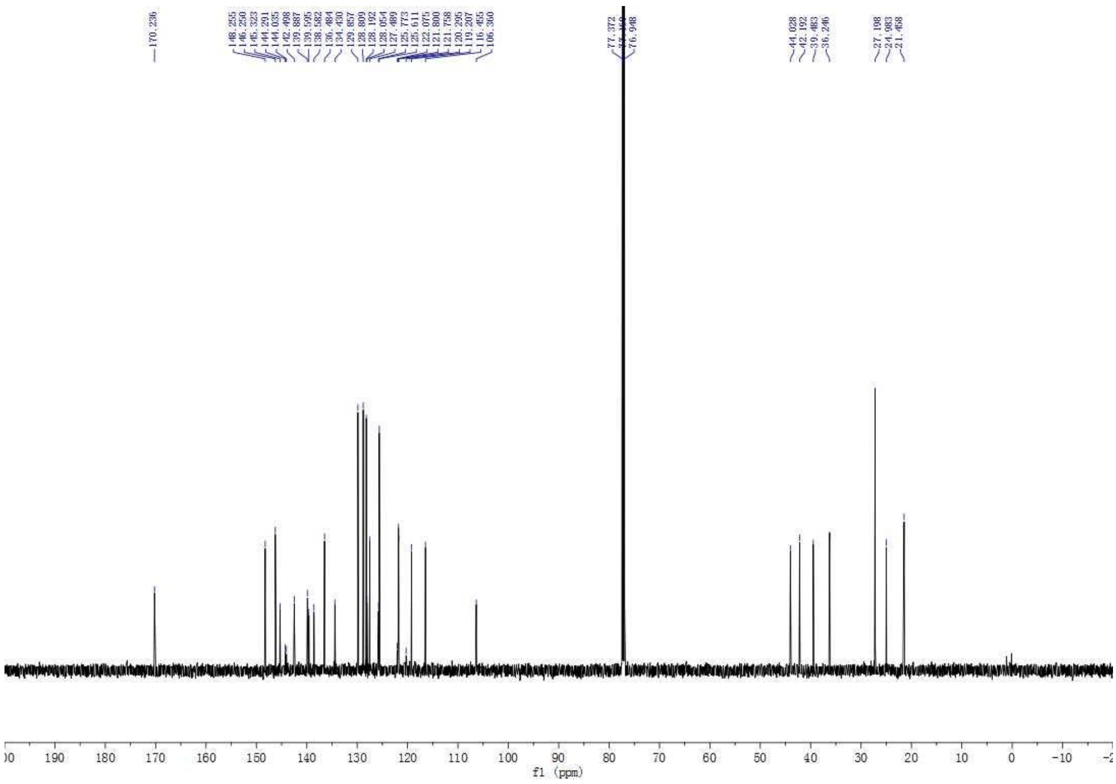

Supplementary Fig. 175. ^{13}C NMR of compound 3ad. The sample has been recorded in 150 MHz, $CDCl_3$ at 25 °C

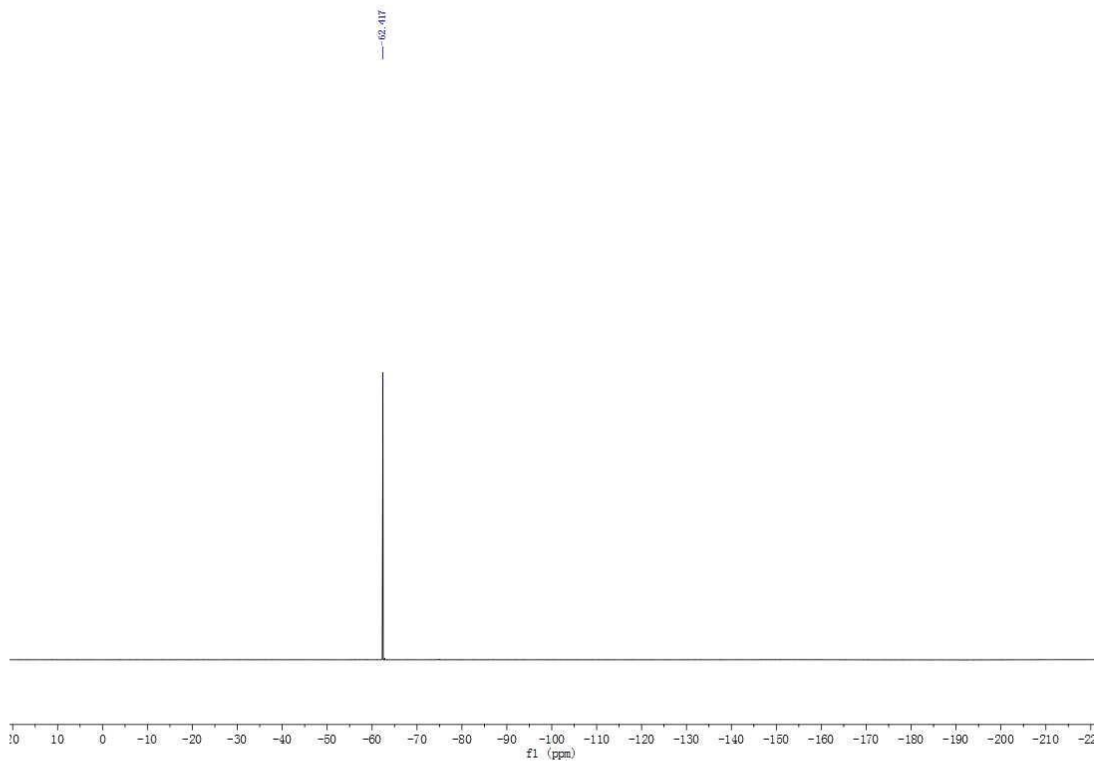

Supplementary Fig. 176. ^{19}F NMR of compound 3ad. The sample has been recorded in 470 MHz, $CDCl_3$ at 25 °C

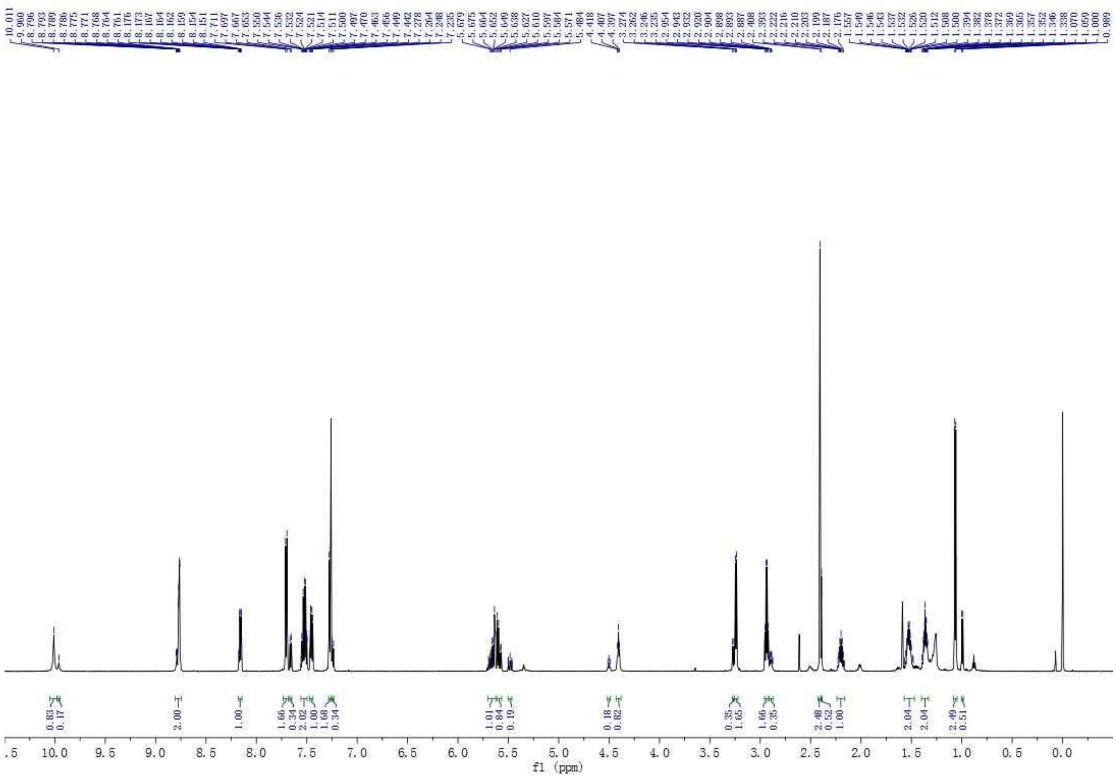

Supplementary Fig. 177. ^{1}H NMR of compound 3ae. The sample has been recorded in 600 MHz, $CDCl_3$ at 25 °C

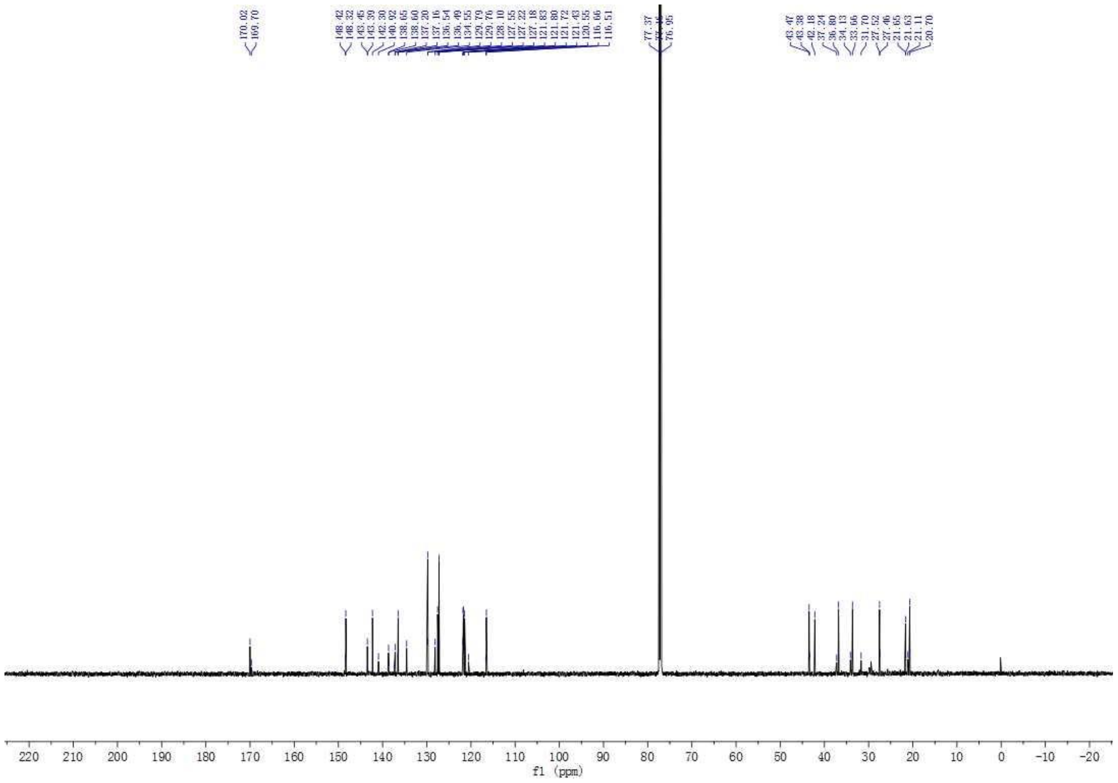

Supplementary Fig. 178. ^{13}C NMR of compound 3ae. The sample has been recorded in 150 MHz, $CDCl_3$ at 25 °C

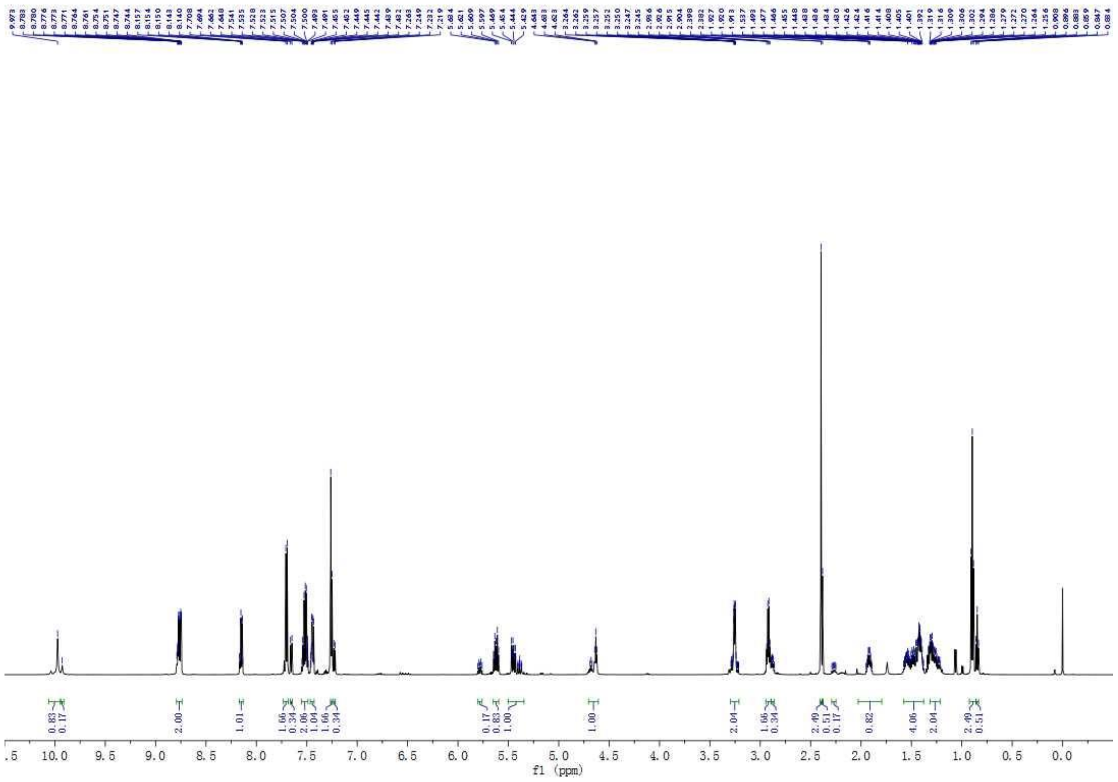

Supplementary Fig. 179. ^{1}H NMR of compound 3af. The sample has been recorded in 600 MHz, $CDCl_3$ at 25 °C

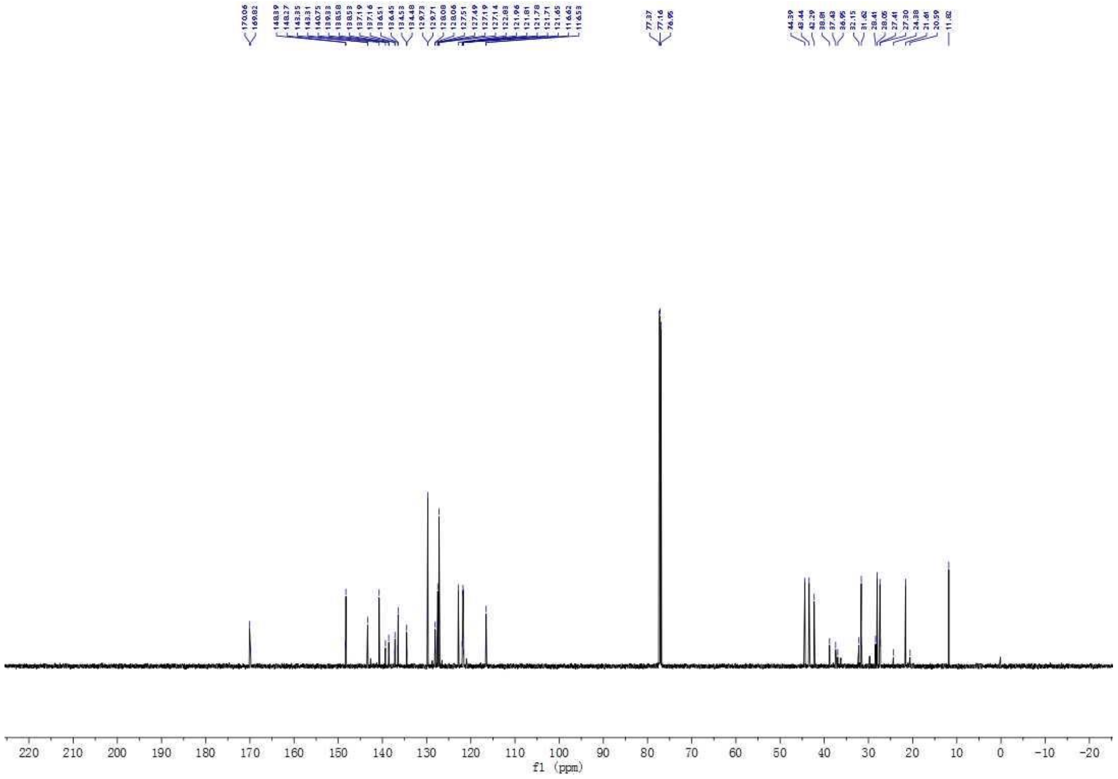

Supplementary Fig. 180. ^{13}C NMR of compound 3af. The sample has been recorded in 150 MHz, $CDCl_3$ at 25 °C

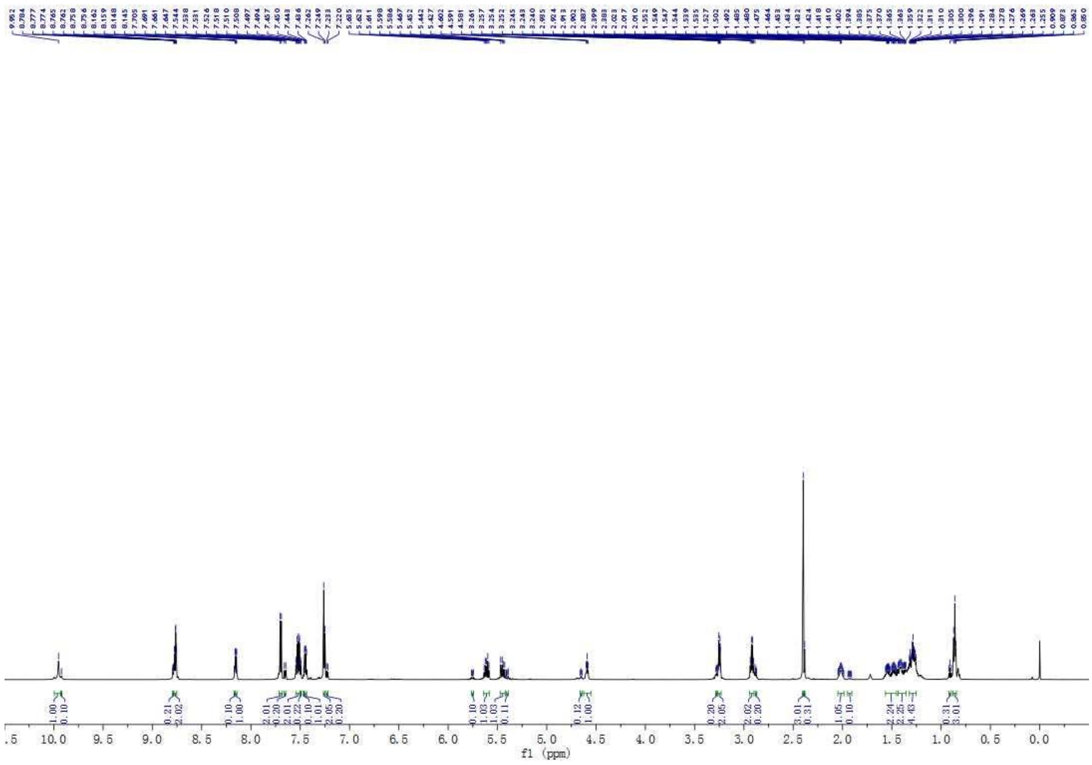

Supplementary Fig. 181. ^{1}H NMR of compound 3ag. The sample has been recorded in 600 MHz, $CDCl_3$ at 25 °C

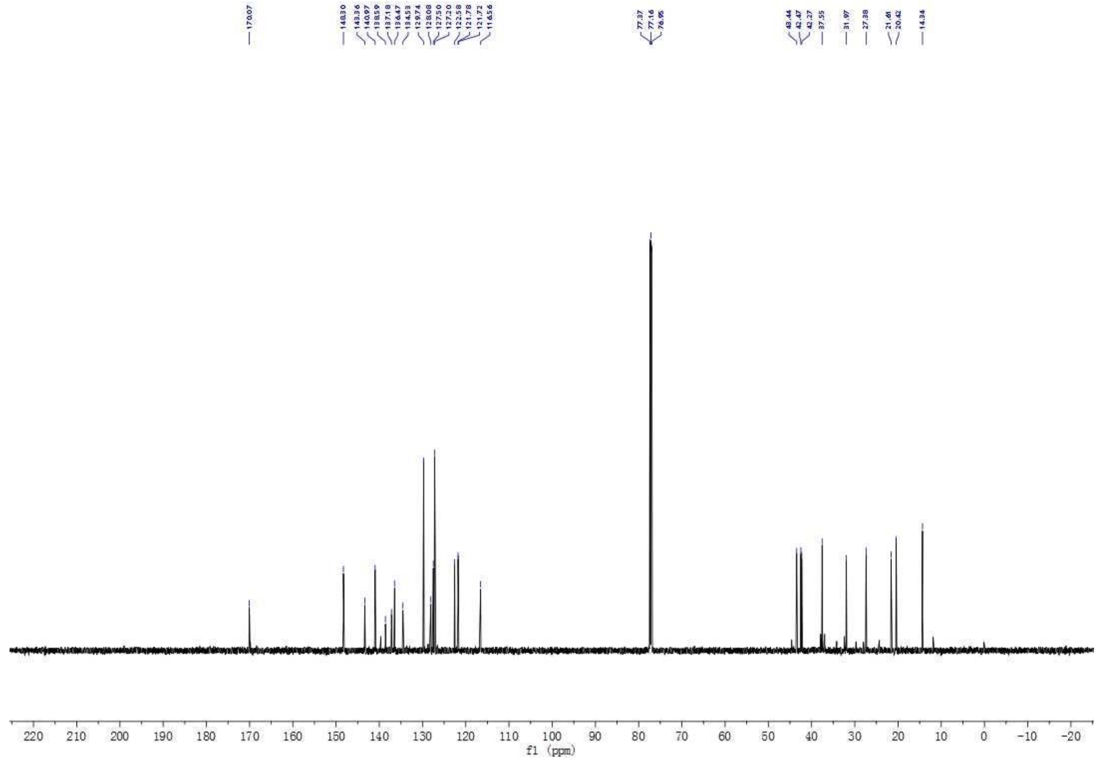

Supplementary Fig. 182. ^{13}C NMR of compound 3ag. The sample has been recorded in 150 MHz, $CDCl_3$ at 25 °C

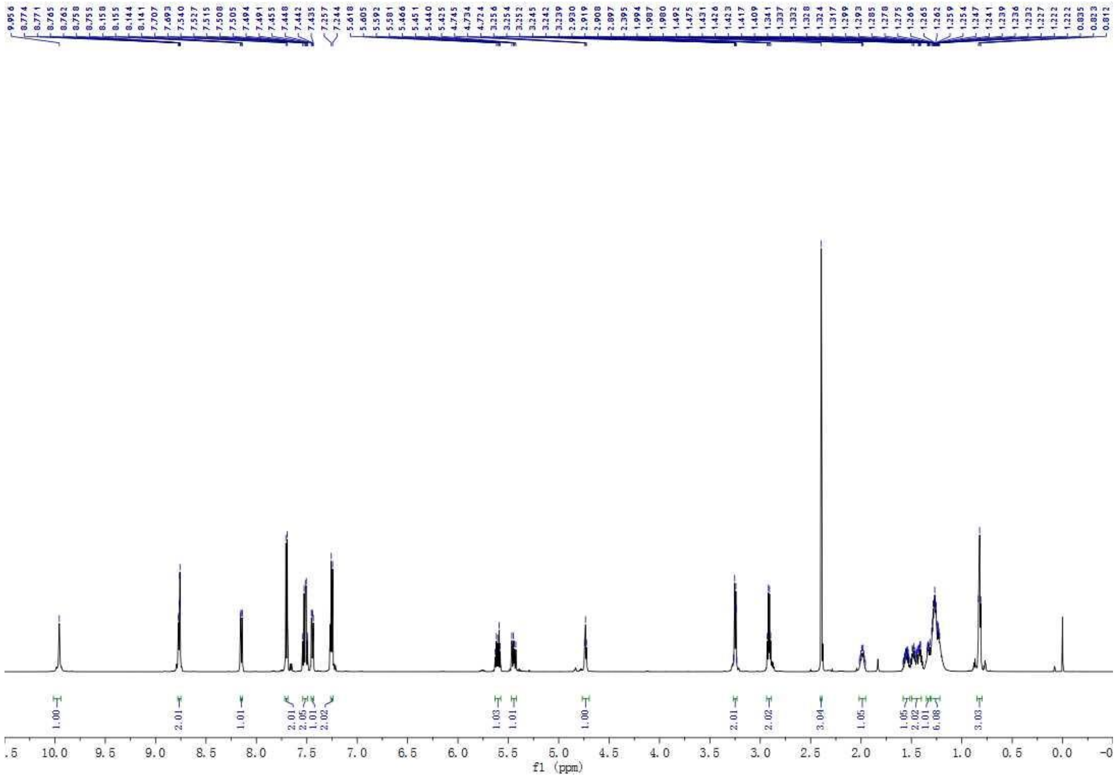

Supplementary Fig. 183. ^{1}H NMR of compound 3ah. The sample has been recorded in 600 MHz, $CDCl_3$ at 25 °C

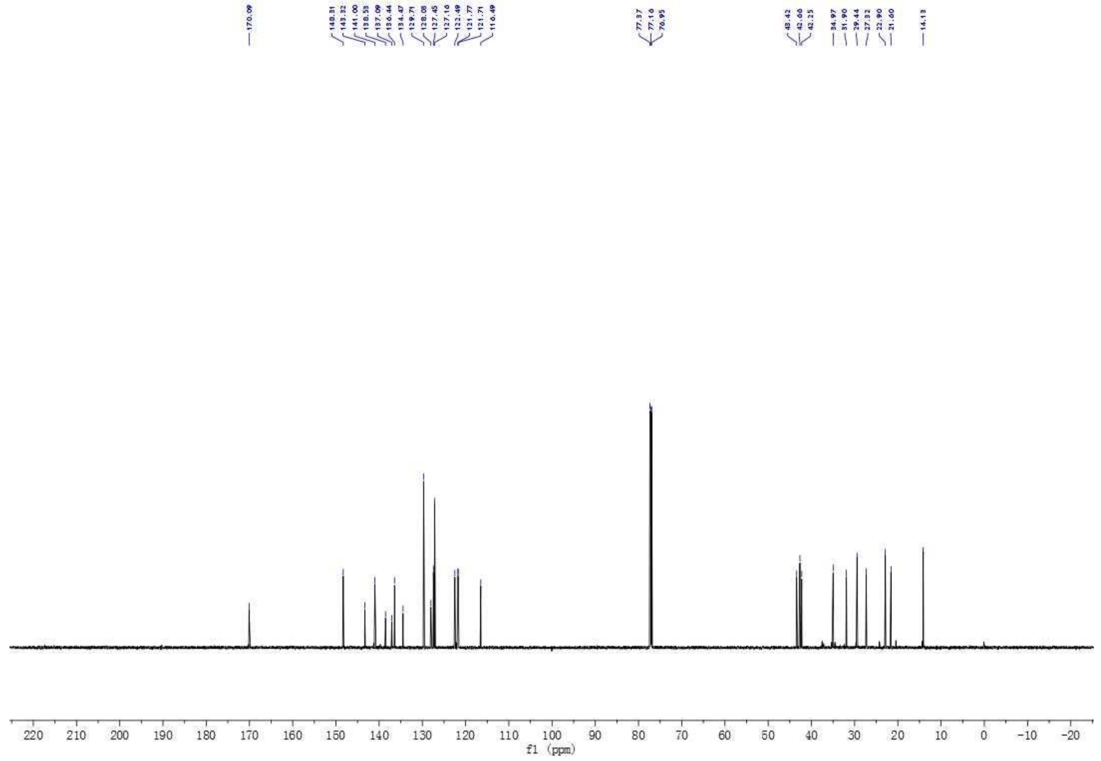

Supplementary Fig. 184. ^{13}C NMR of compound 3ah. The sample has been recorded in 150 MHz, $CDCl_3$ at 25 °C

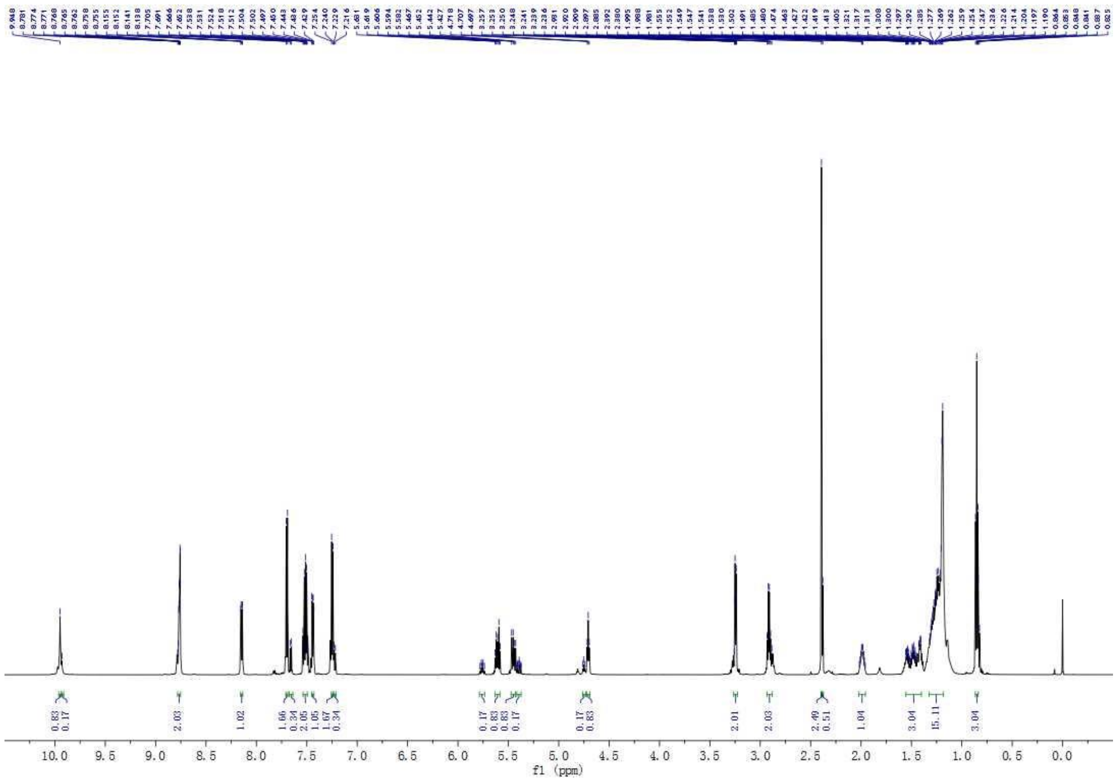

Supplementary Fig. 185. ^{1}H NMR of compound 3ai. The sample has been recorded in 600 MHz, $CDCl_3$ at 25 °C

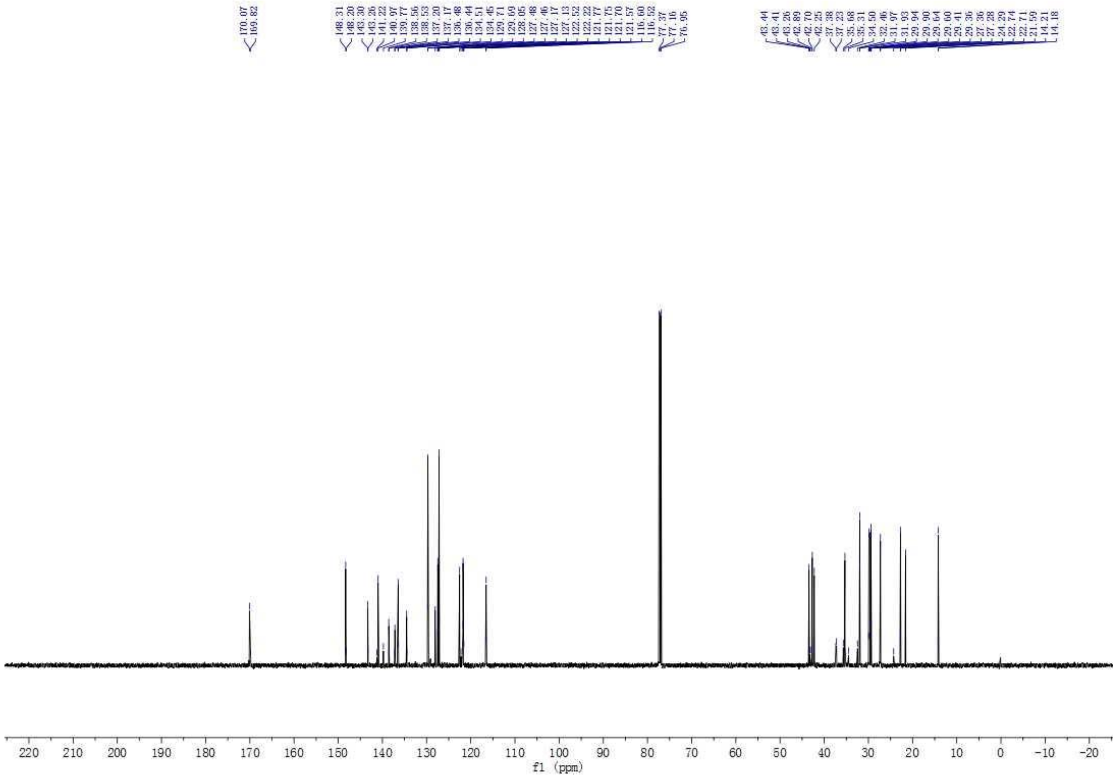

Supplementary Fig. 186. ^{13}C NMR of compound 3ai. The sample has been recorded in 150 MHz, $CDCl_3$ at 25 °C

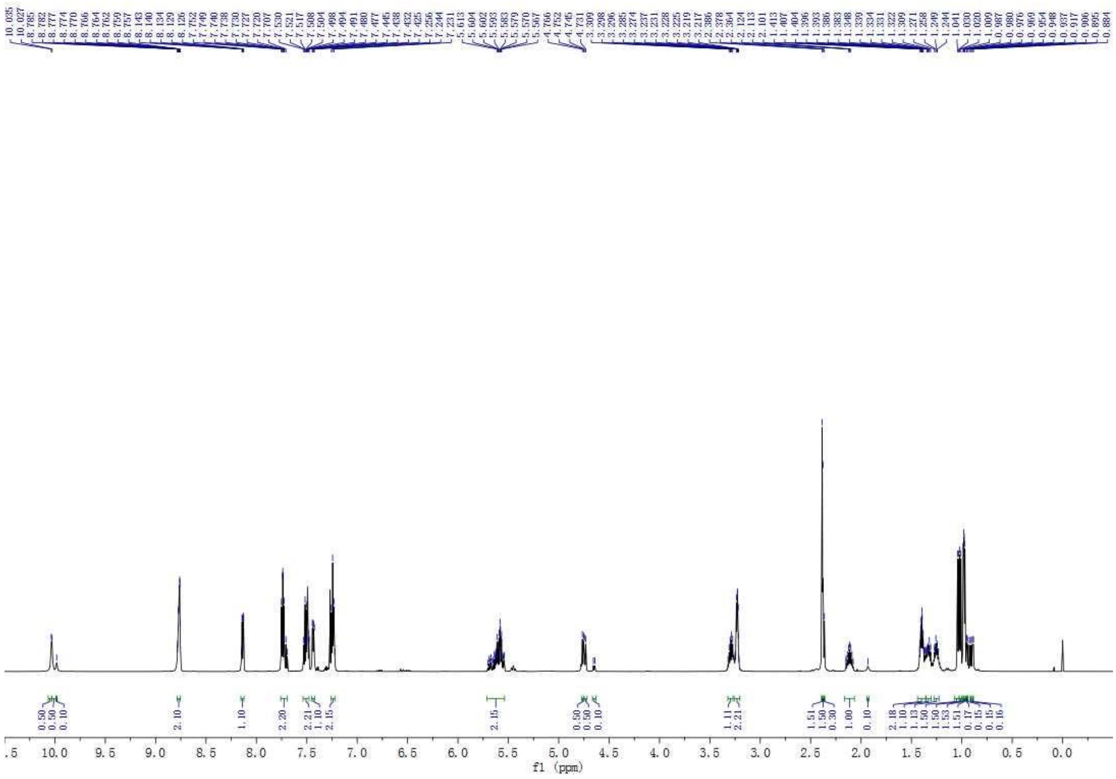

Supplementary Fig. 187. ^{1}H NMR of compound 3aj. The sample has been recorded in 600 MHz, $CDCl_3$ at 25 °C

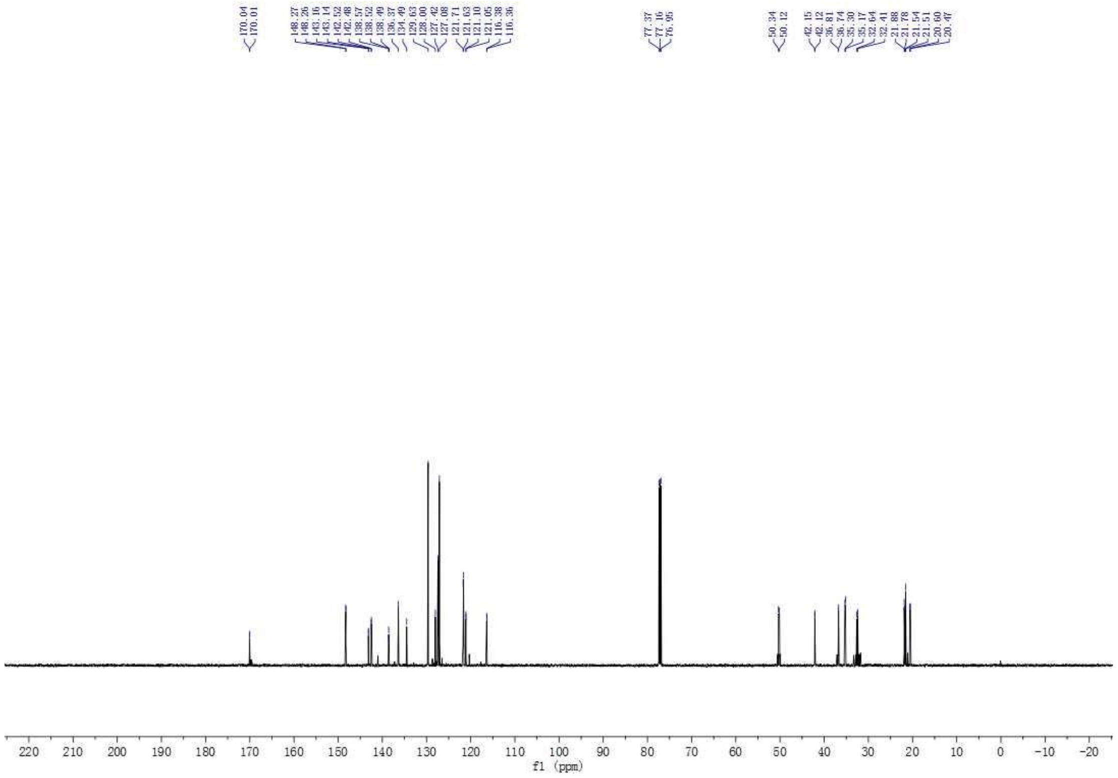

Supplementary Fig. 188. ^{13}C NMR of compound 3aj. The sample has been recorded in 150 MHz, $CDCl_3$ at 25 °C

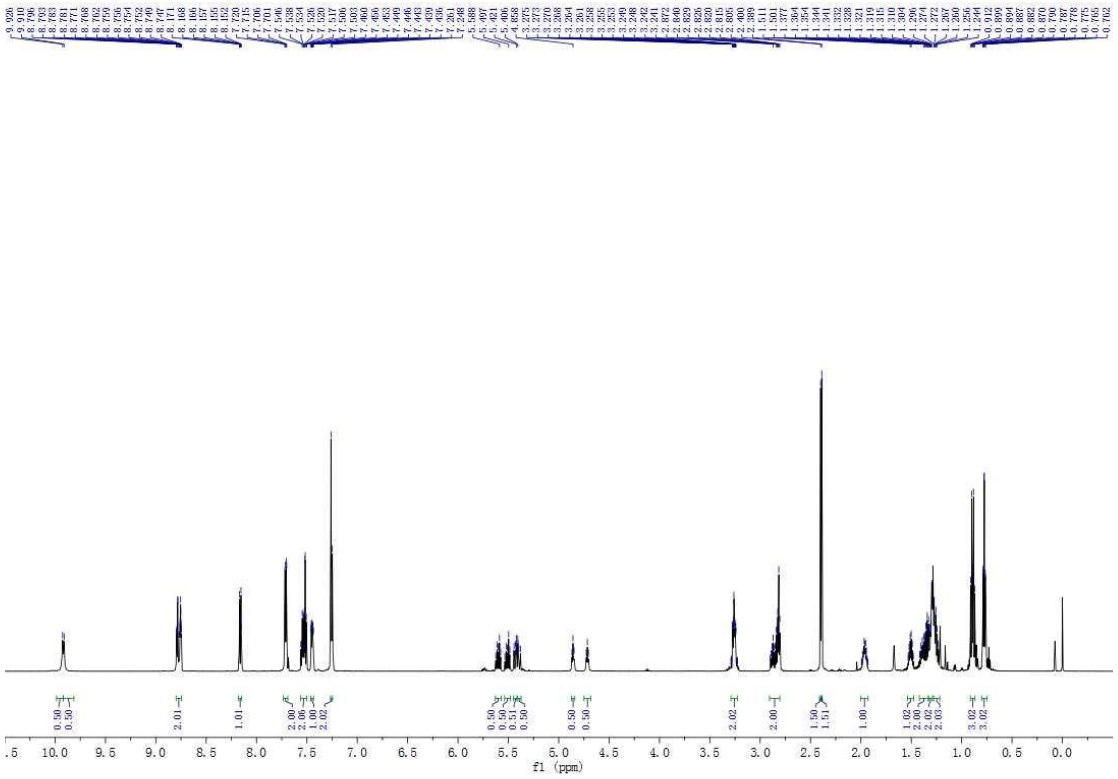

Supplementary Fig. 189. ^{1}H NMR of compound 3ak. The sample has been recorded in 600 MHz, $CDCl_3$ at 25 °C

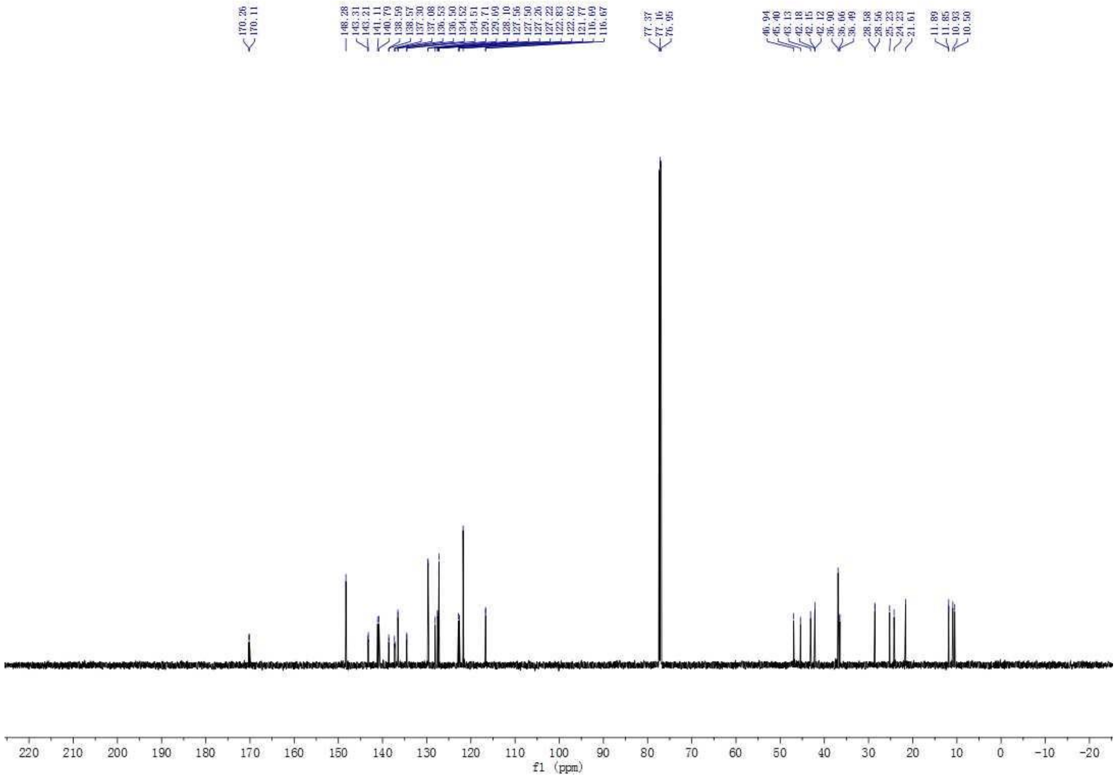

Supplementary Fig. 190. ^{13}C NMR of compound 3ak. The sample has been recorded in150 MHz, $CDCl_3$ at 25 °C

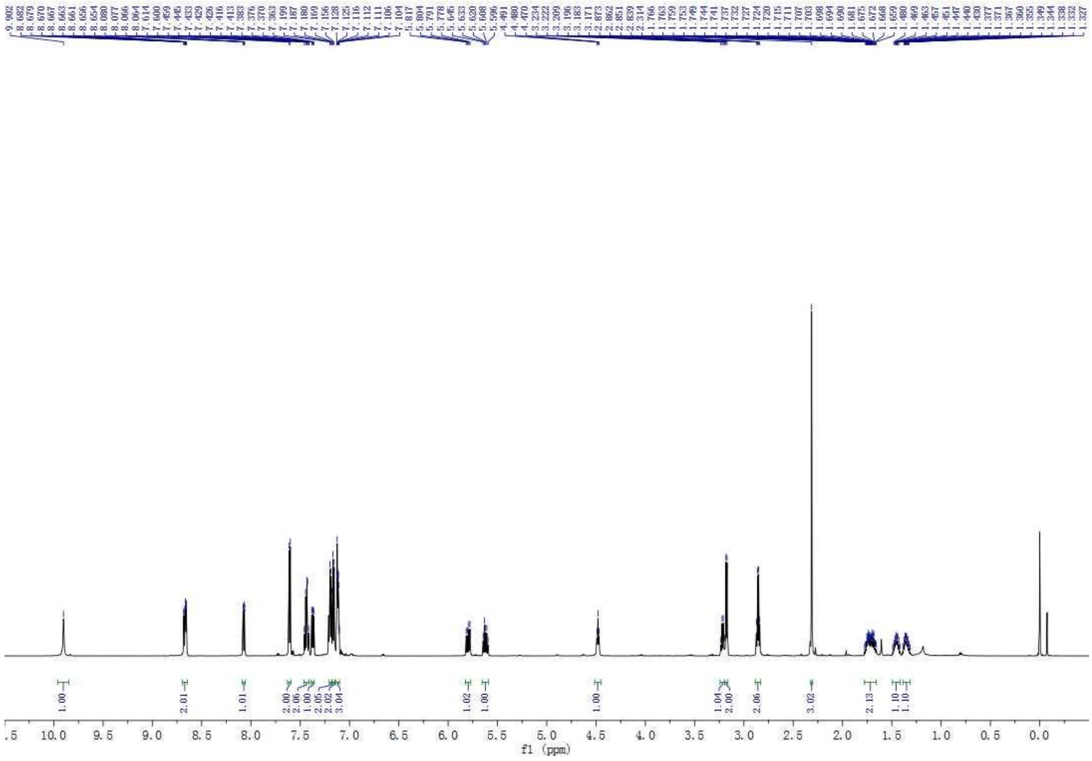

Supplementary Fig. 191. ^{1}H NMR of compound 3al. The sample has been recorded in 600 MHz, $CDCl_3$ at 25 °C

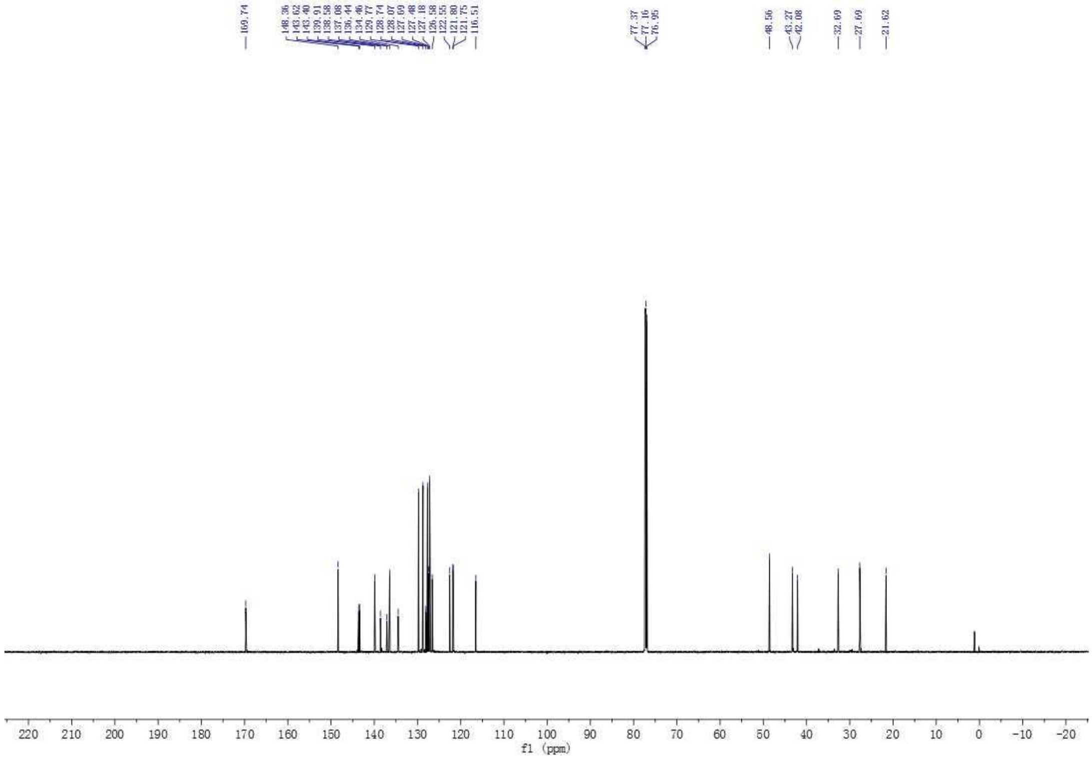

Supplementary Fig. 192. ^{13}C NMR of compound 3al. The sample has been recorded in 150 MHz, $CDCl_3$ at 25 °C

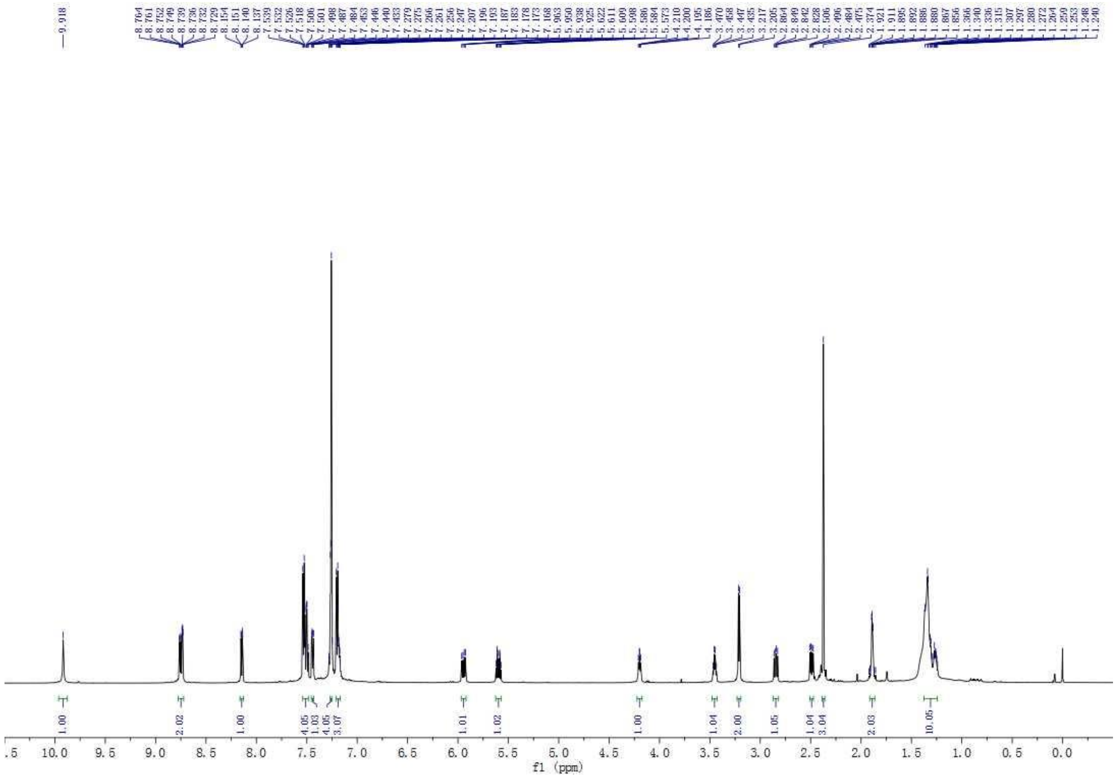

Supplementary Fig. 193. ^{1}H NMR of compound 3am. The sample has been recorded in 600 MHz, $CDCl_3$ at 25 °C

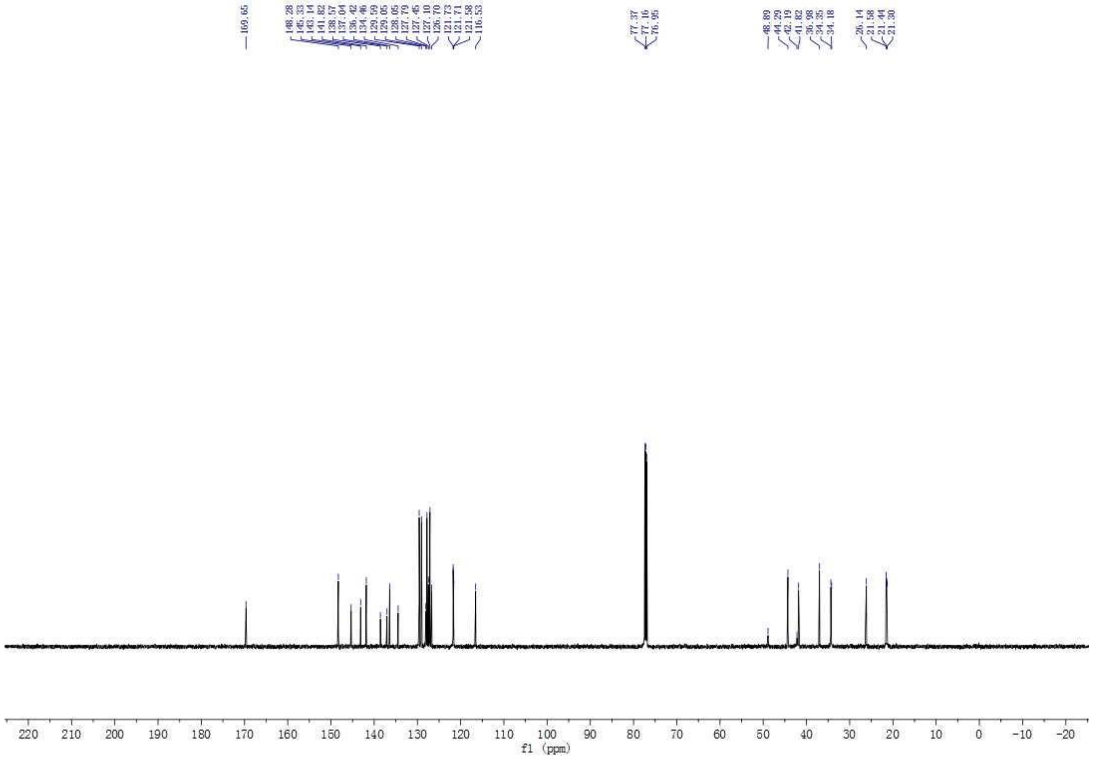

Supplementary Fig. 194. ^{13}C NMR of compound 3am. The sample has been recorded in 150 MHz, $CDCl_3$ at 25 °C

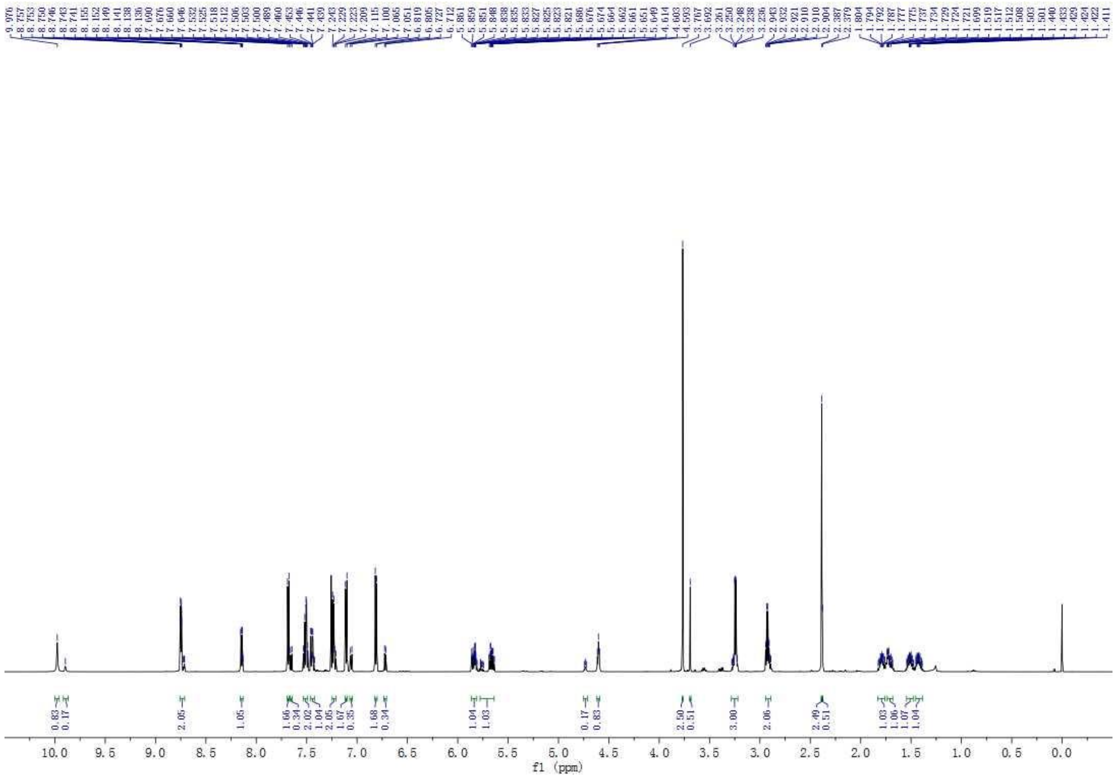

Supplementary Fig. 195. ^{1}H NMR of compound 3an. The sample has been recorded in 600 MHz, $CDCl_3$ at 25 °C

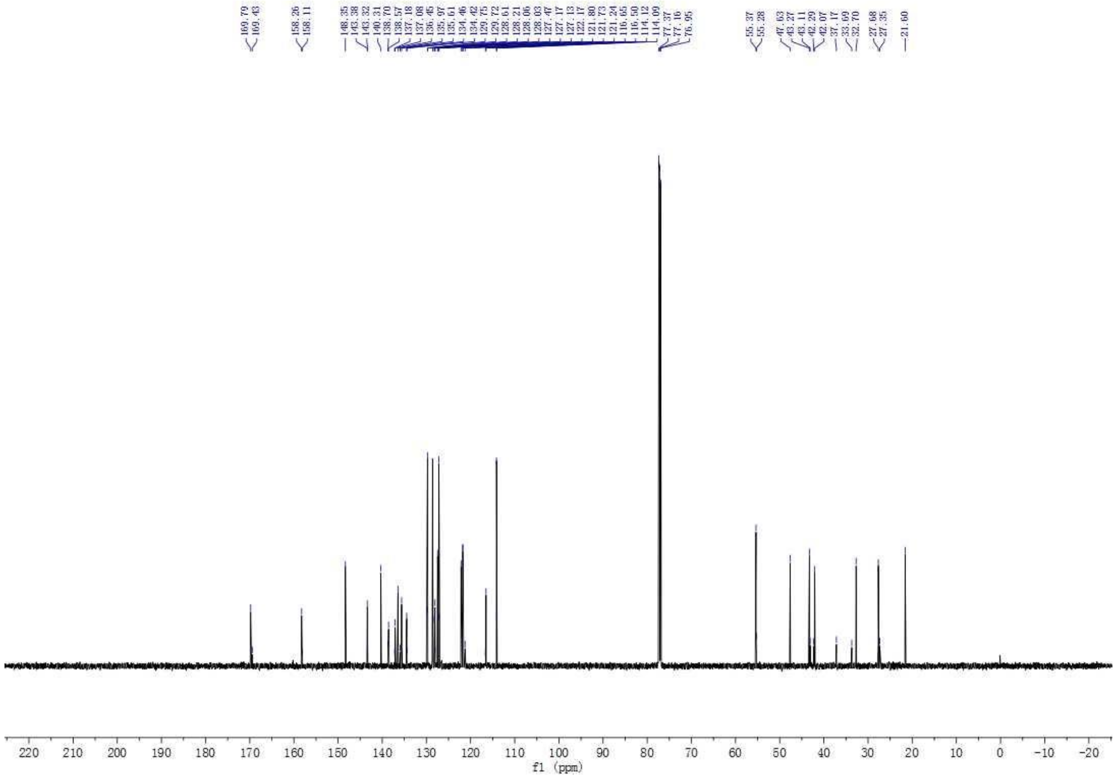

Supplementary Fig. 196. ^{13}C NMR of compound 3an. The sample has been recorded in 150 MHz, $CDCl_3$ at 25 °C

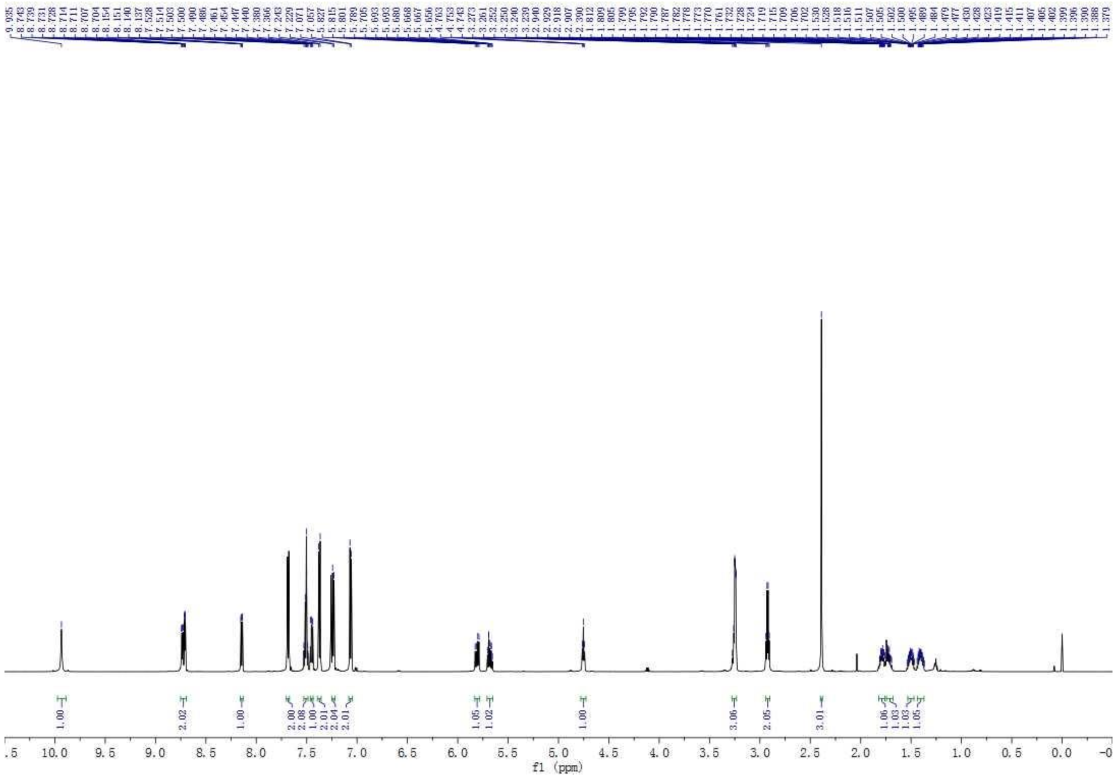

Supplementary Fig. 197. ^{1}H NMR of compound 3ao. The sample has been recorded in 600 MHz, $CDCl_3$ at 25 °C

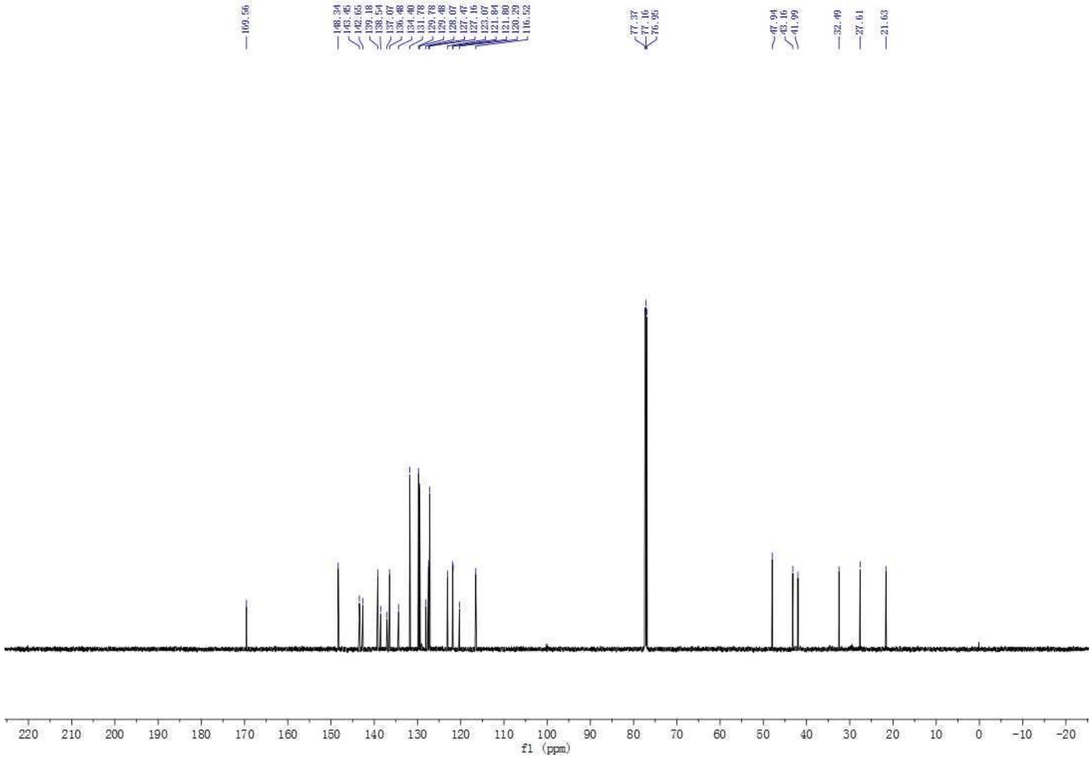

Supplementary Fig. 198. ^{13}C NMR of compound 3ao. The sample has been recorded in 150 MHz, $CDCl_3$ at 25 °C

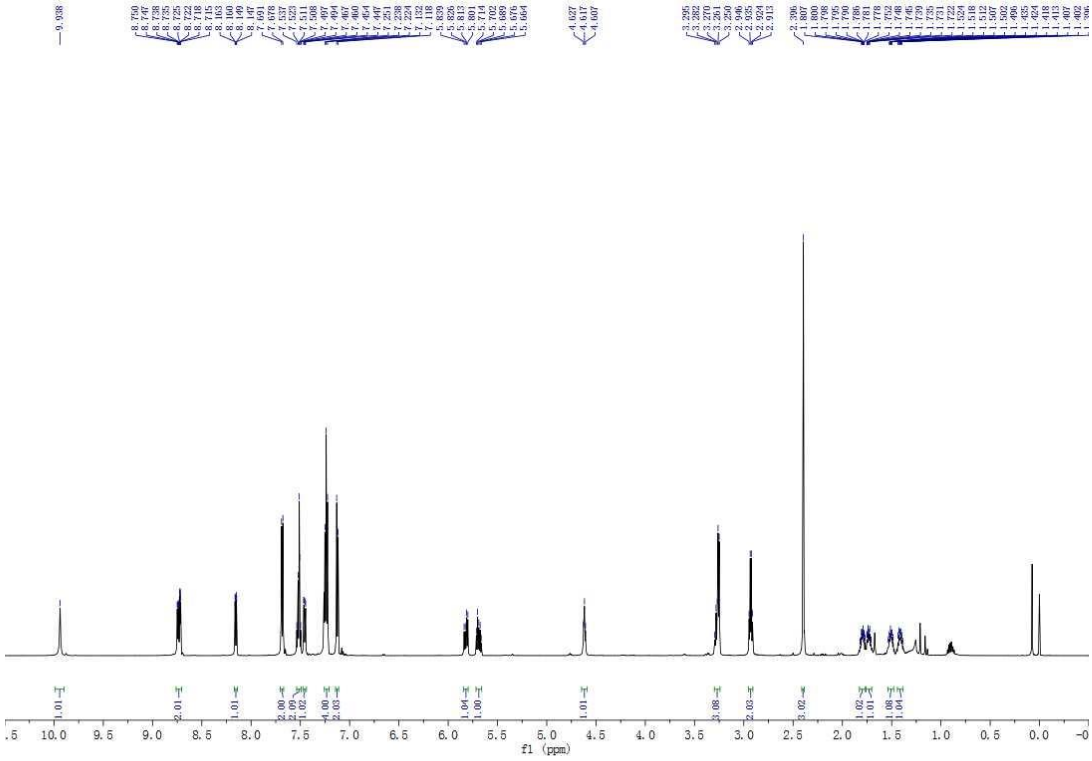

Supplementary Fig. 199. ^{1}H NMR of compound 3ap. The sample has been recorded in 600 MHz, $CDCl_3$ at 25 °C

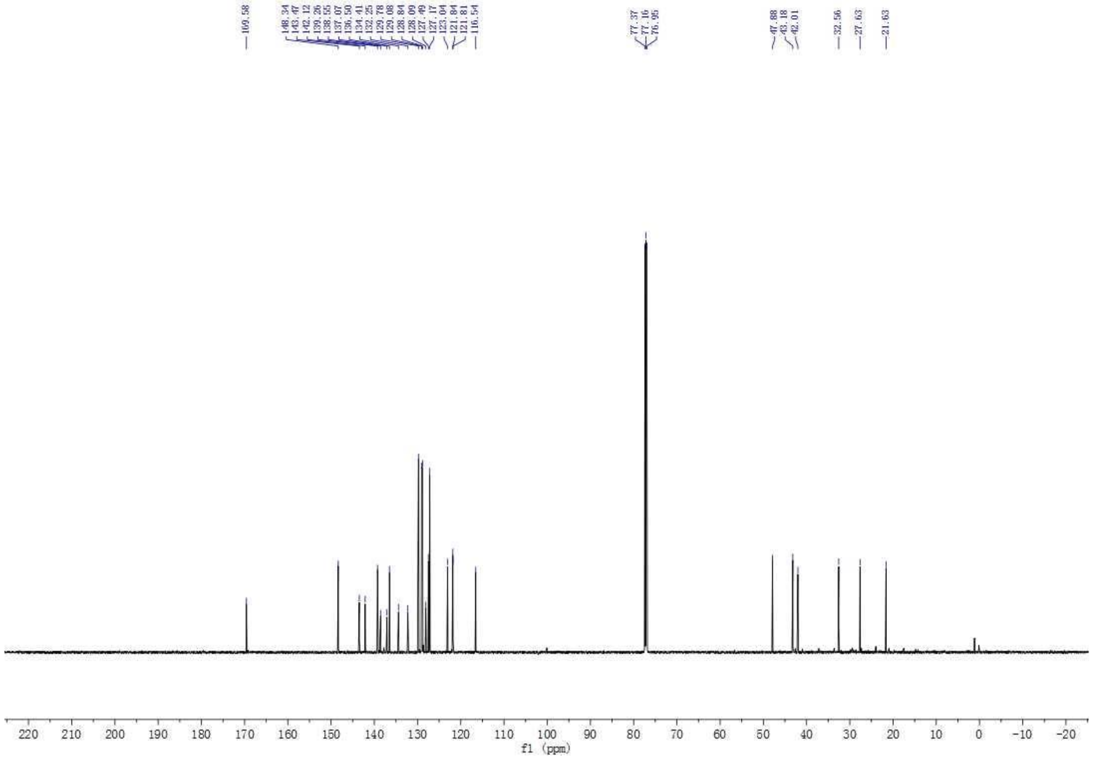

Supplementary Fig. 200. ^{13}C NMR of compound 3ap. The sample has been recorded in 150 MHz, $CDCl_3$ at 25 °C

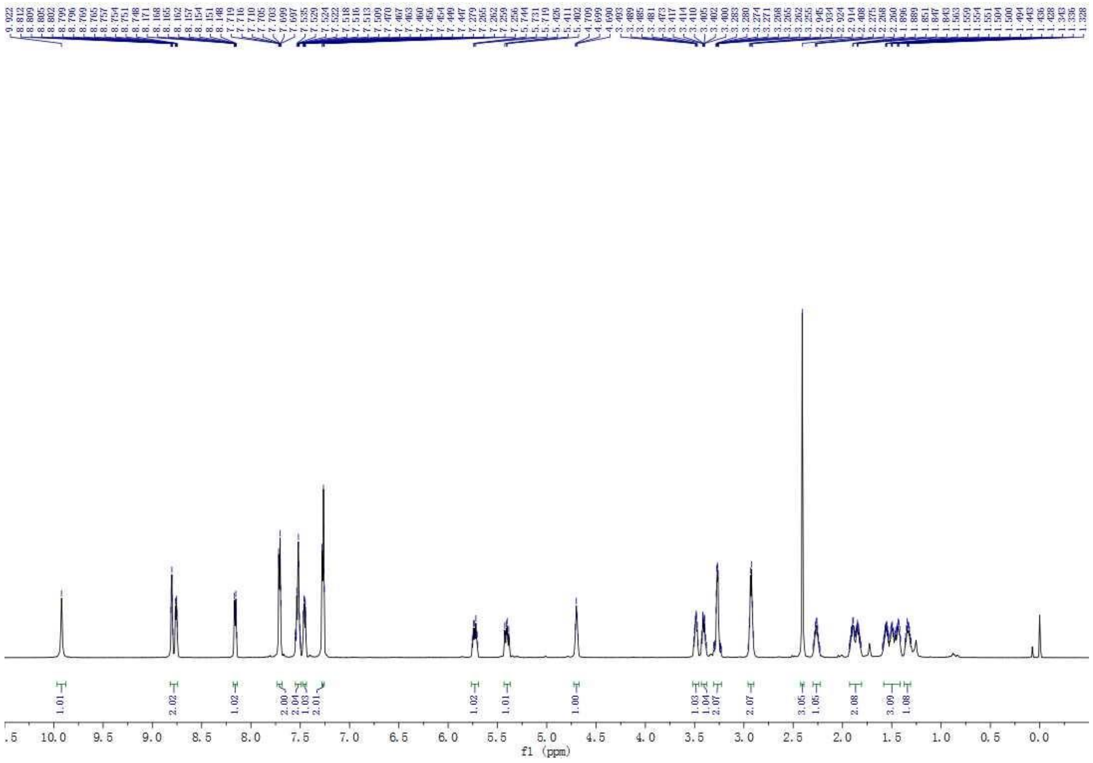

Supplementary Fig. 201. ^{1}H NMR of compound 3aq. The sample has been recorded in 600 MHz, $CDCl_3$ at 25 °C

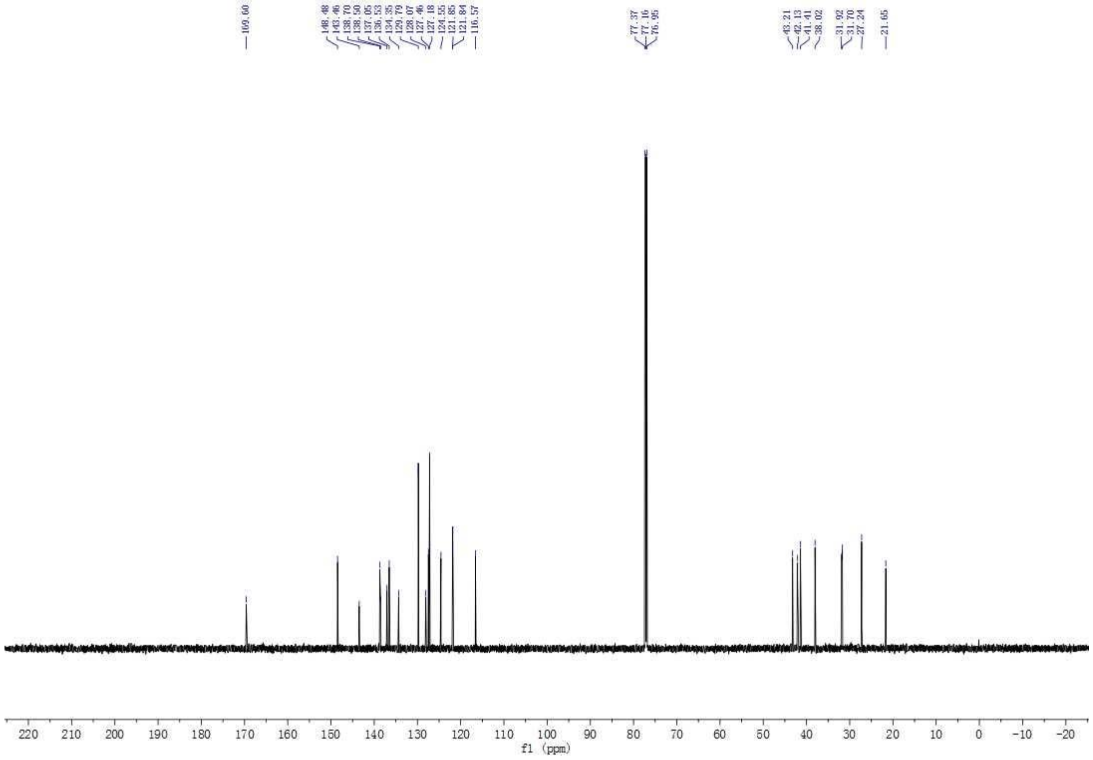

Supplementary Fig. 202. ^{13}C NMR of compound 3aq. The sample has been recorded in 150 MHz, $CDCl_3$ at 25 °C

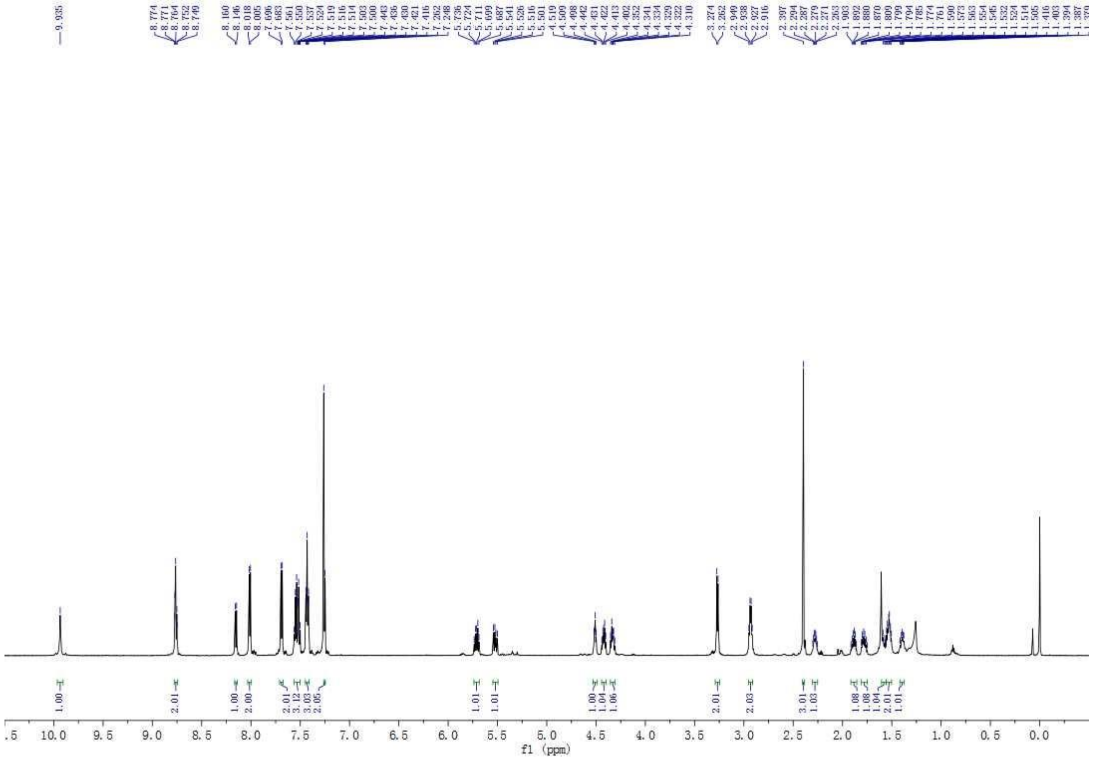

Supplementary Fig. 203. ^{1}H NMR of compound 3ar. The sample has been recorded in 600 MHz, $CDCl_3$ at 25 °C

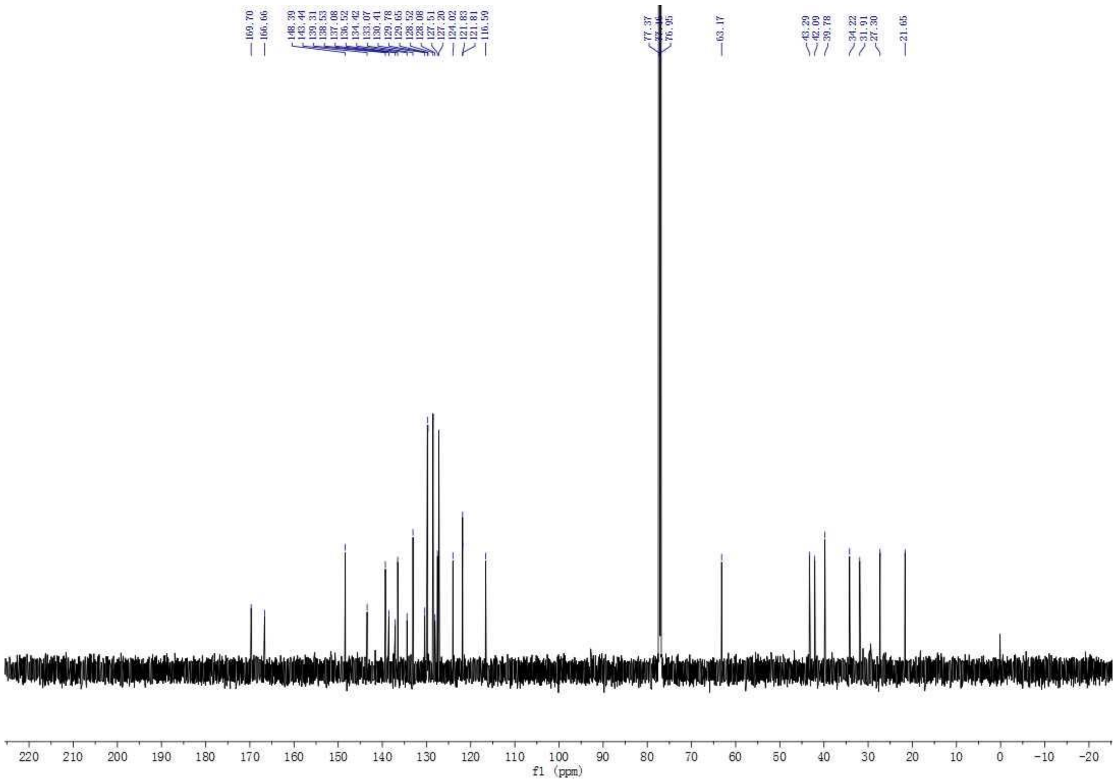

Supplementary Fig. 204. ^{13}C NMR of compound 3ar. The sample has been recorded in 150 MHz, $CDCl_3$ at 25 °C

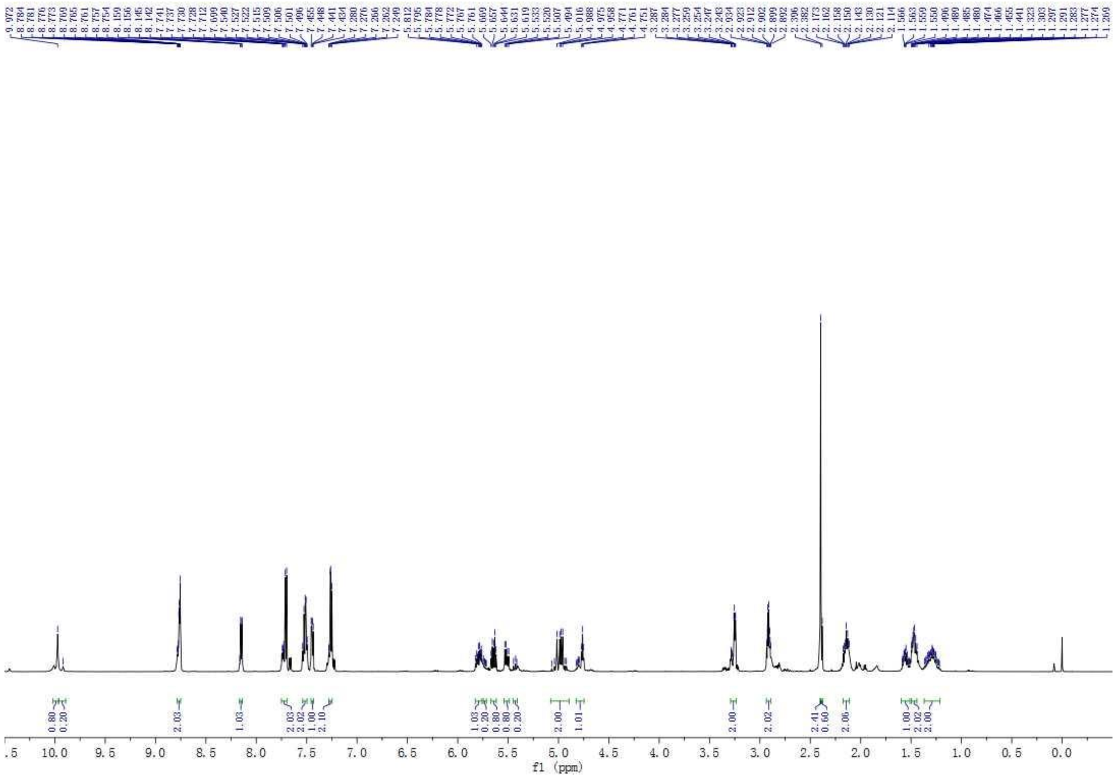

Supplementary Fig. 205. ^{1}H NMR of compound 3as. The sample has been recorded in 600 MHz, $CDCl_3$ at 25 °C

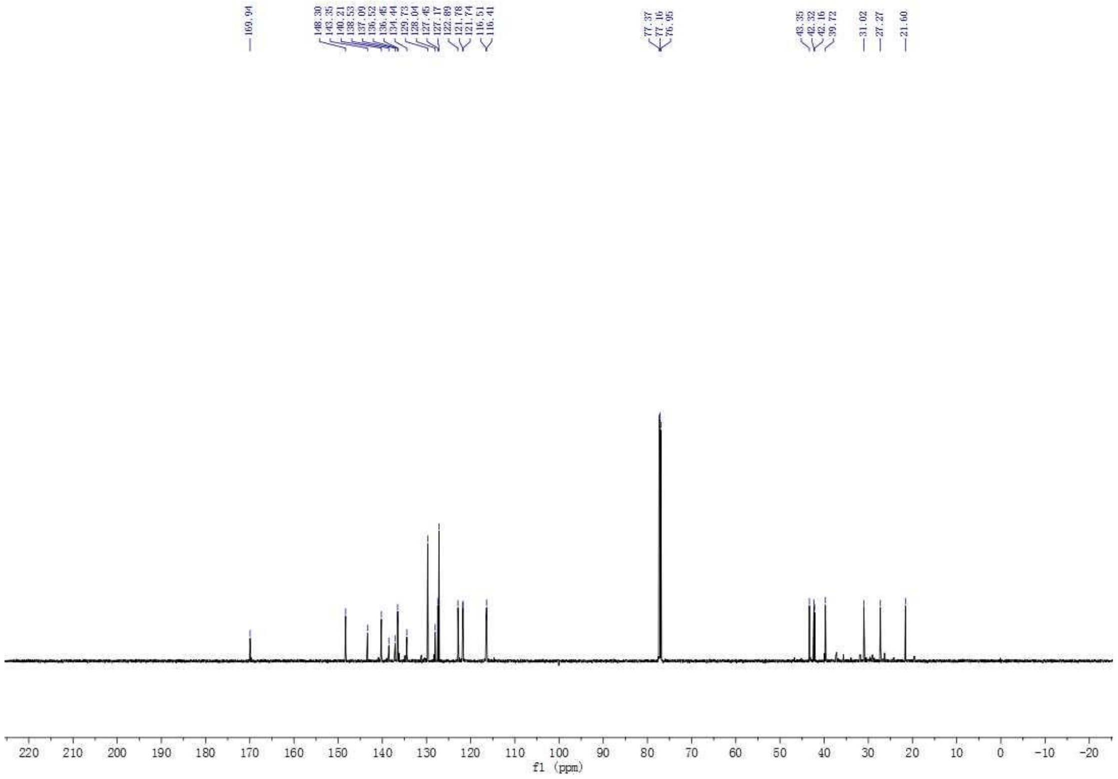

Supplementary Fig. 206. ^{13}C NMR of compound 3as. The sample has been recorded in 150 MHz, $CDCl_3$ at 25 °C

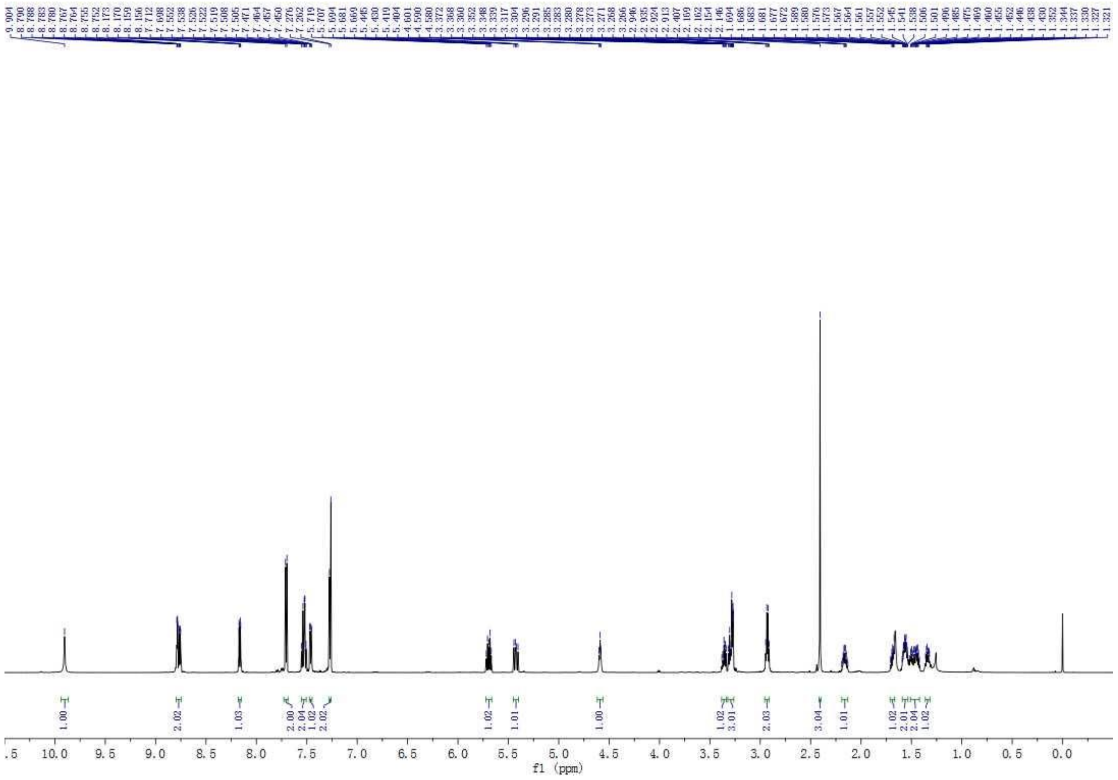

Supplementary Fig. 207. ^{1}H NMR of compound 3at. The sample has been recorded in 600 MHz, $CDCl_3$ at 25 °C

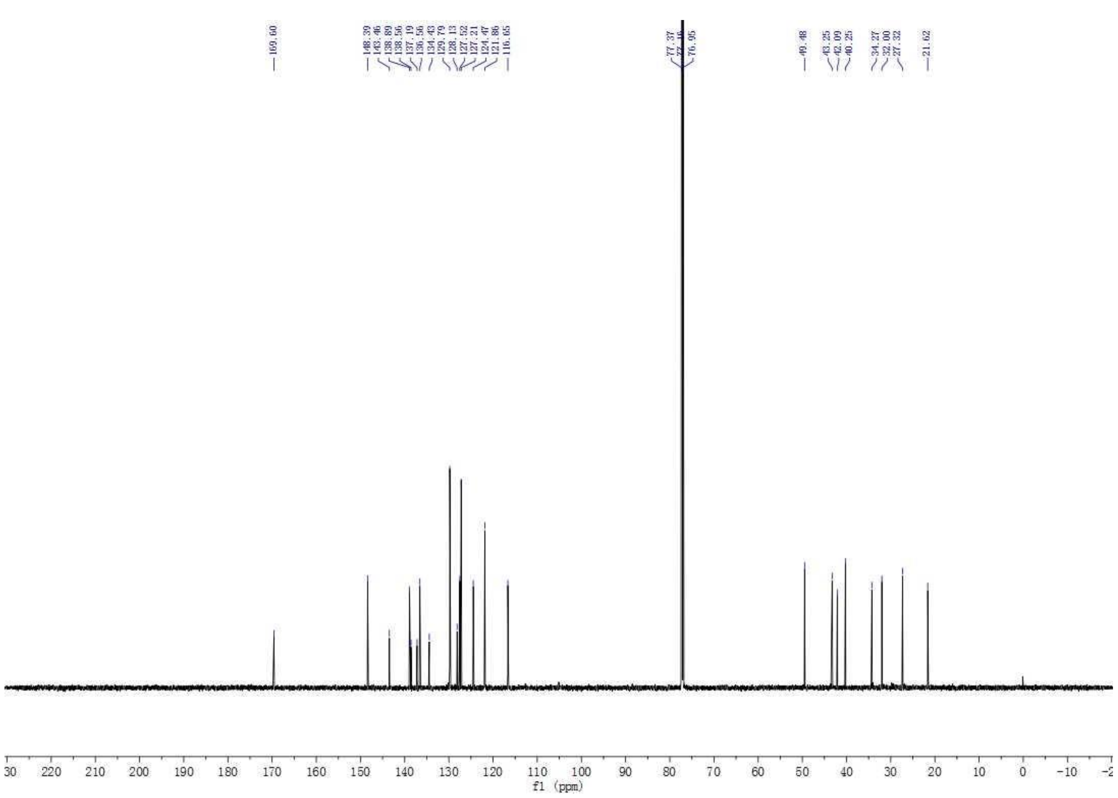

Supplementary Fig. 208. ^{13}C NMR of compound 3at. The sample has been recorded in 150 MHz, $CDCl_3$ at 25 °C

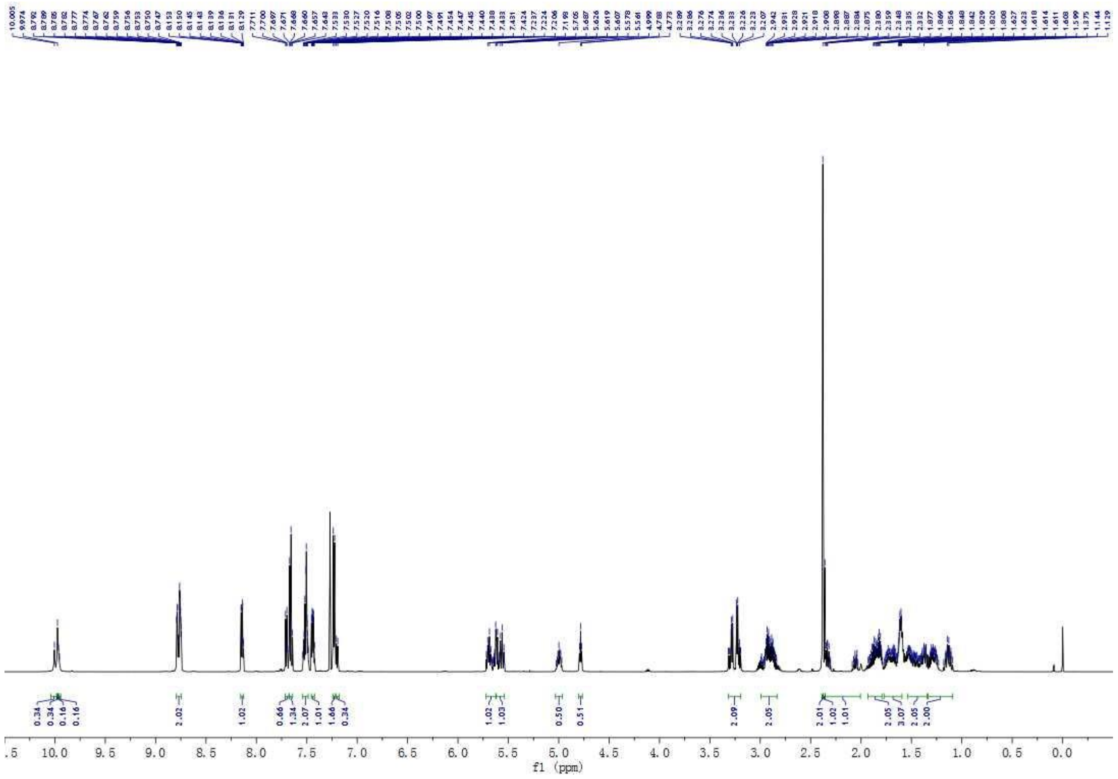

Supplementary Fig. 209. ^{1}H NMR of compound 3au. The sample has been recorded in 600 MHz, $CDCl_3$ at 25 °C

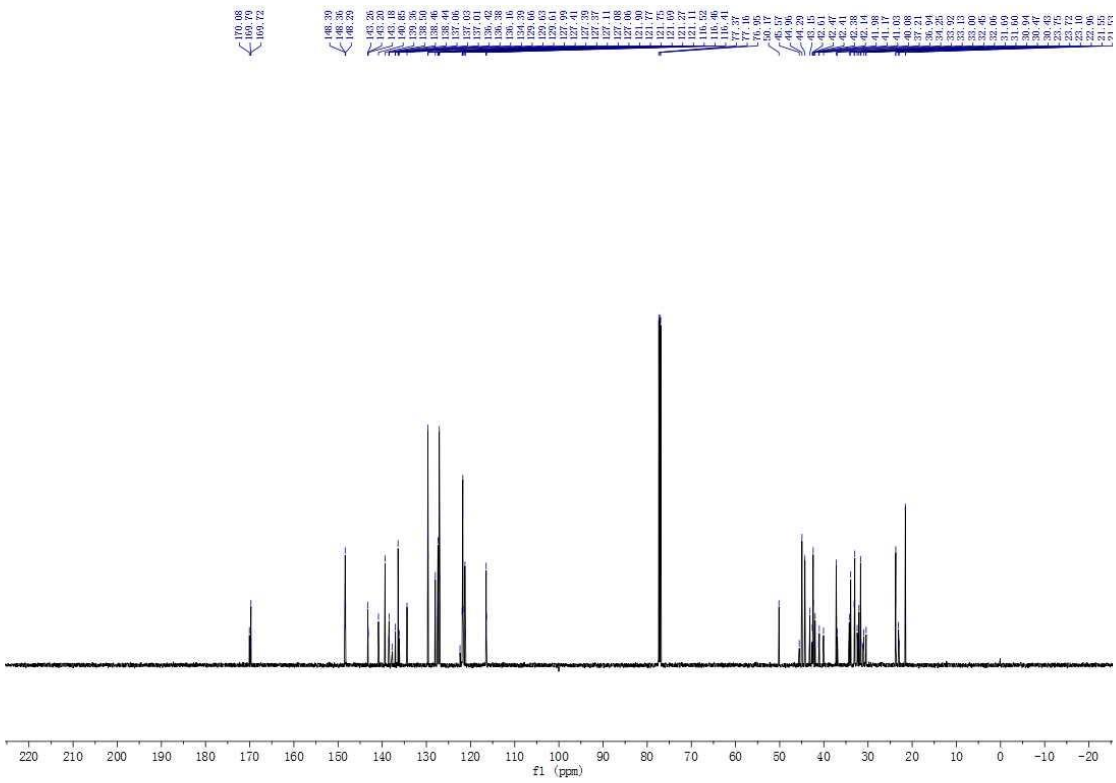

Supplementary Fig. 210. ^{13}C NMR of compound 3au. The sample has been recorded in 150 MHz, $CDCl_3$ at 25 °C

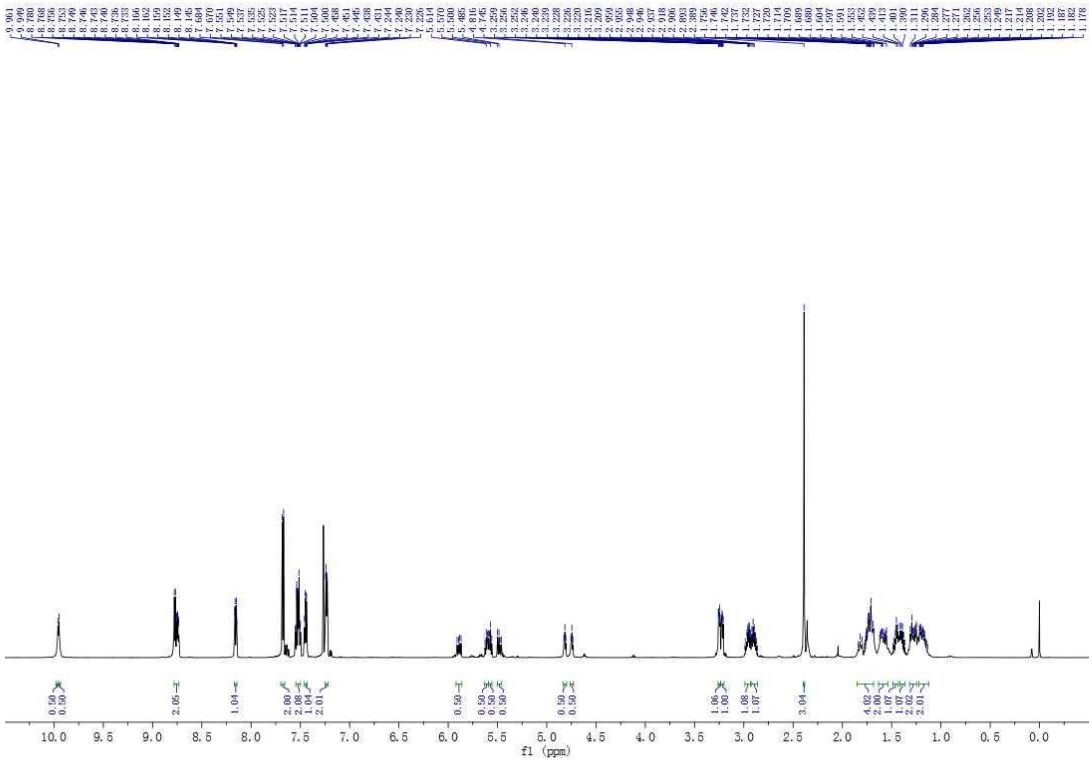

Supplementary Fig. 211. ^{1}H NMR of compound 3av. The sample has been recorded in 600 MHz, $CDCl_3$ at 25 °C

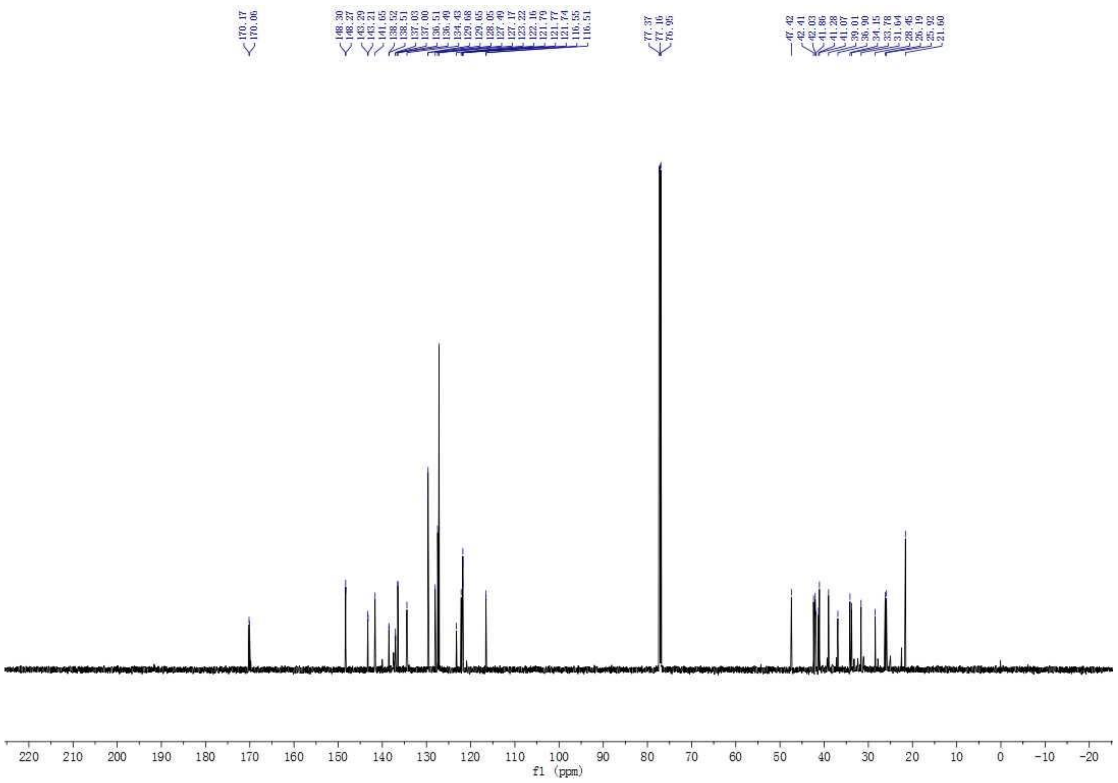

Supplementary Fig. 212. ^{13}C NMR of compound 3av. The sample has been recorded in 150 MHz, $CDCl_3$ at 25 °C

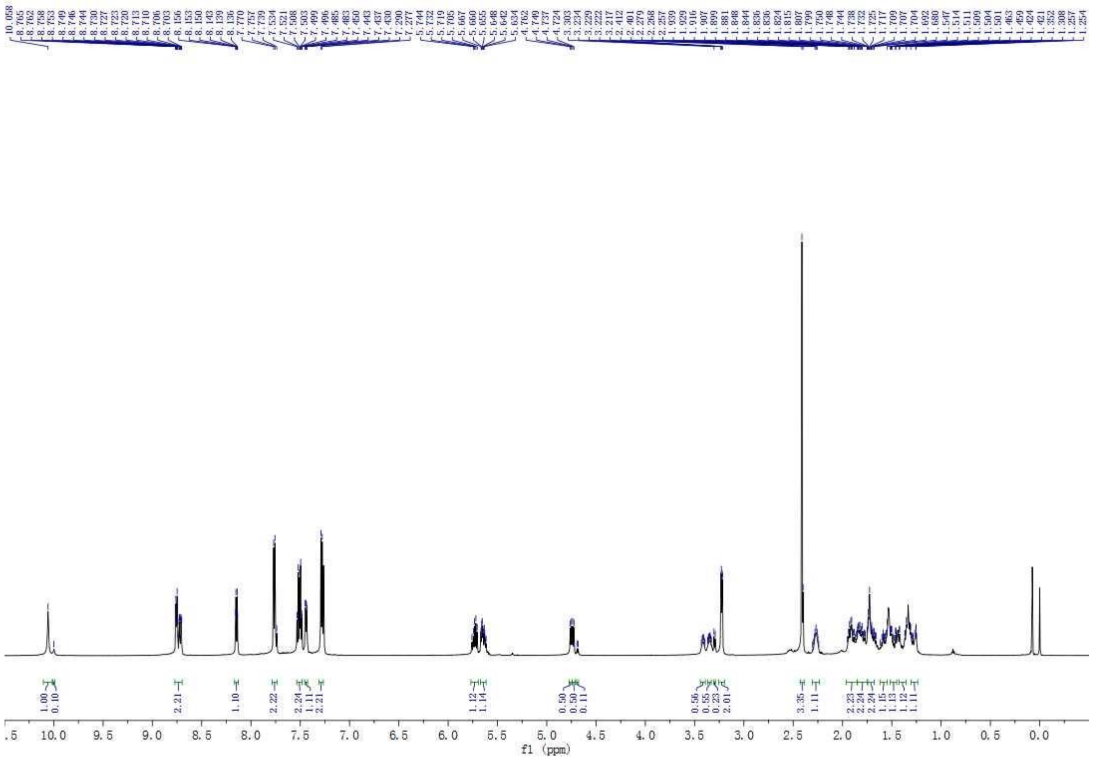

Supplementary Fig. 213. ^{1}H NMR of compound 3aw. The sample has been recorded in 600 MHz, $CDCl_3$ at 25 °C

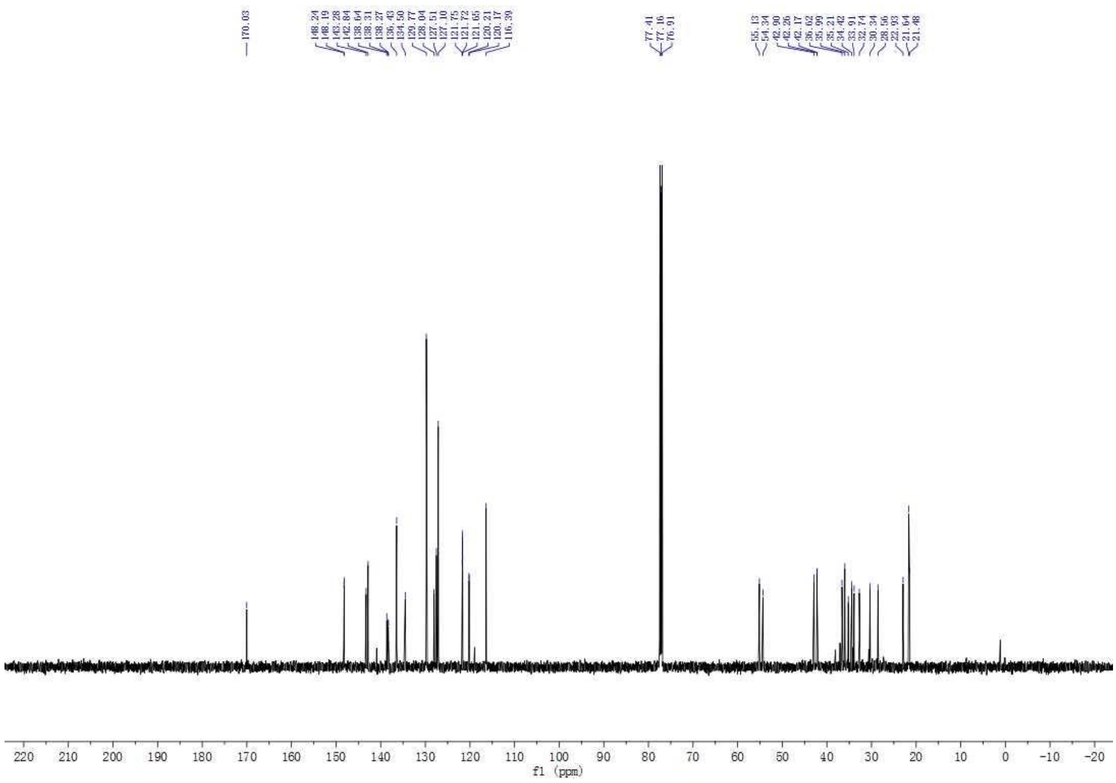

Supplementary Fig. 214. ^{13}C NMR of compound 3aw. The sample has been recorded in 125 MHz, $CDCl_3$ at 25 °C

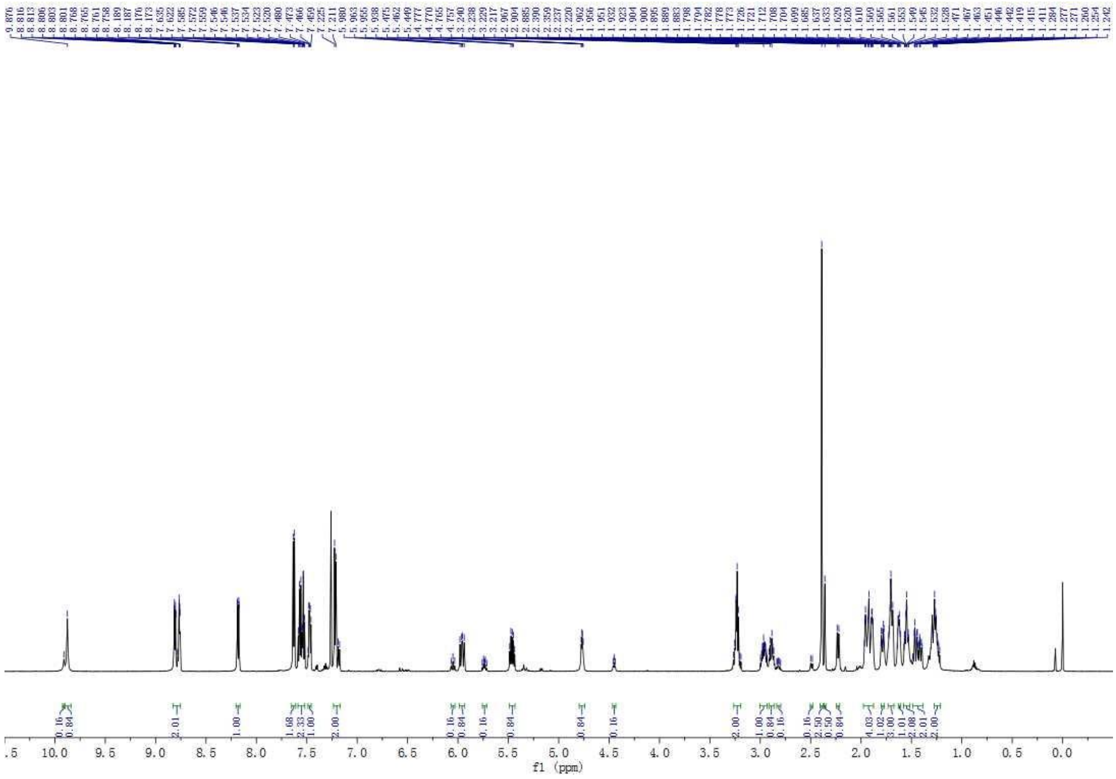

Supplementary Fig. 215. ^{1}H NMR of compound 3ax. The sample has been recorded in 600 MHz, $CDCl_3$ at 25 °C

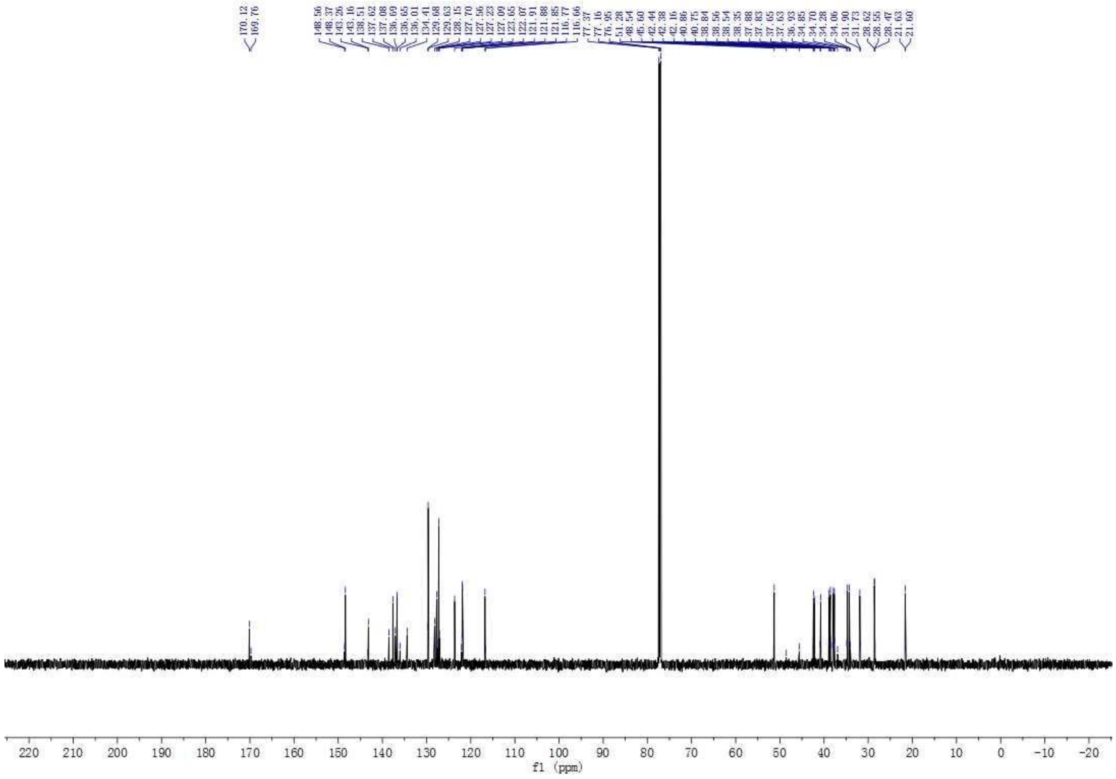

Supplementary Fig. 216. ^{13}C NMR of compound 3ax. The sample has been recorded in 150 MHz, $CDCl_3$ at 25 °C

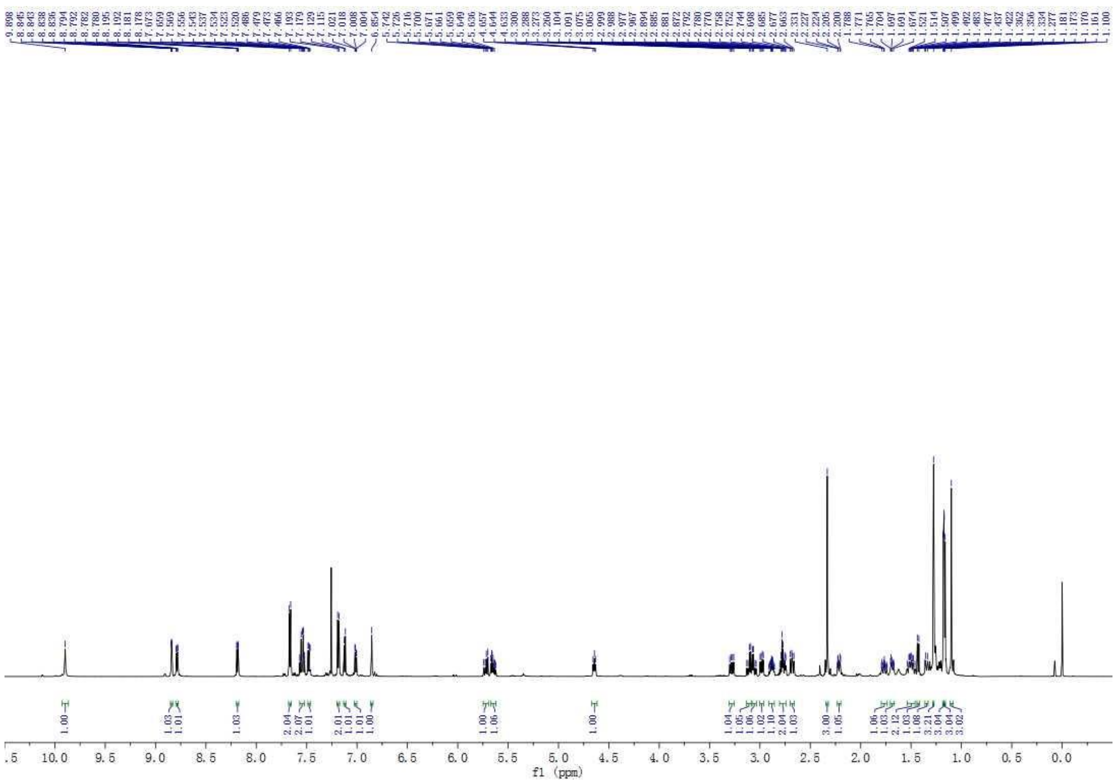

Supplementary Fig. 217. ^{1}H NMR of compound 3ay. The sample has been recorded in 600 MHz, $CDCl_3$ at 25 °C

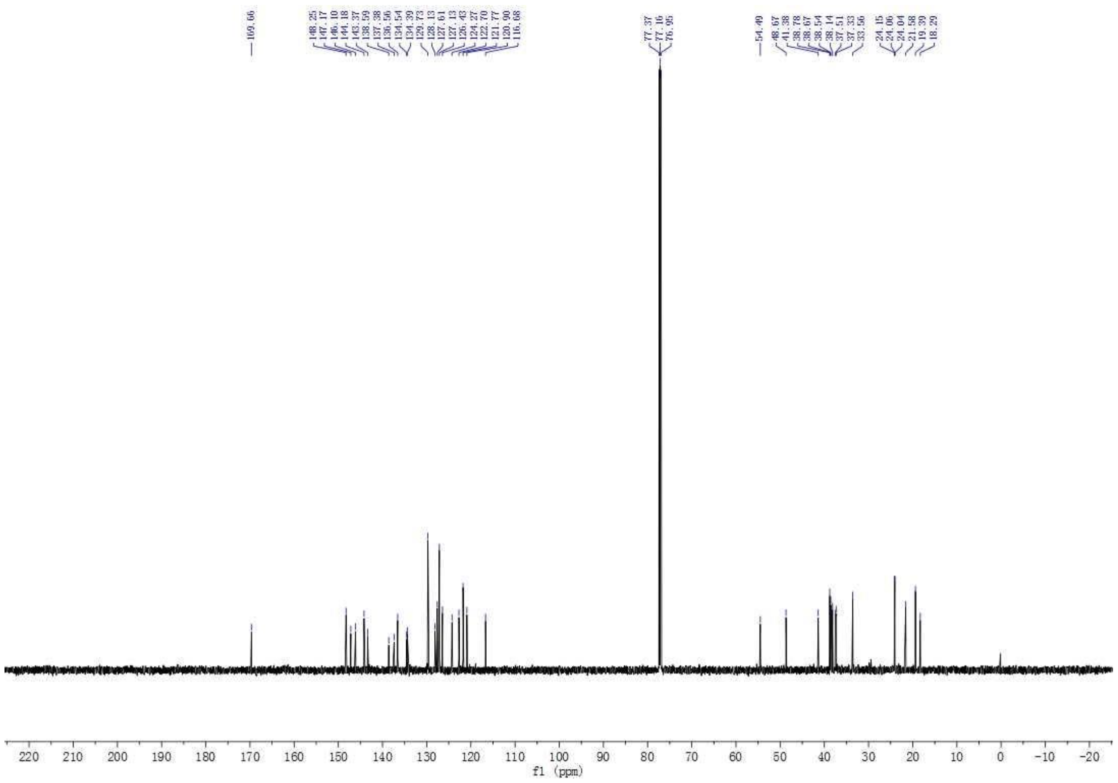

Supplementary Fig. 218. ^{13}C NMR of compound 3ay. The sample has been recorded in 150 MHz, $CDCl_3$ at 25 °C

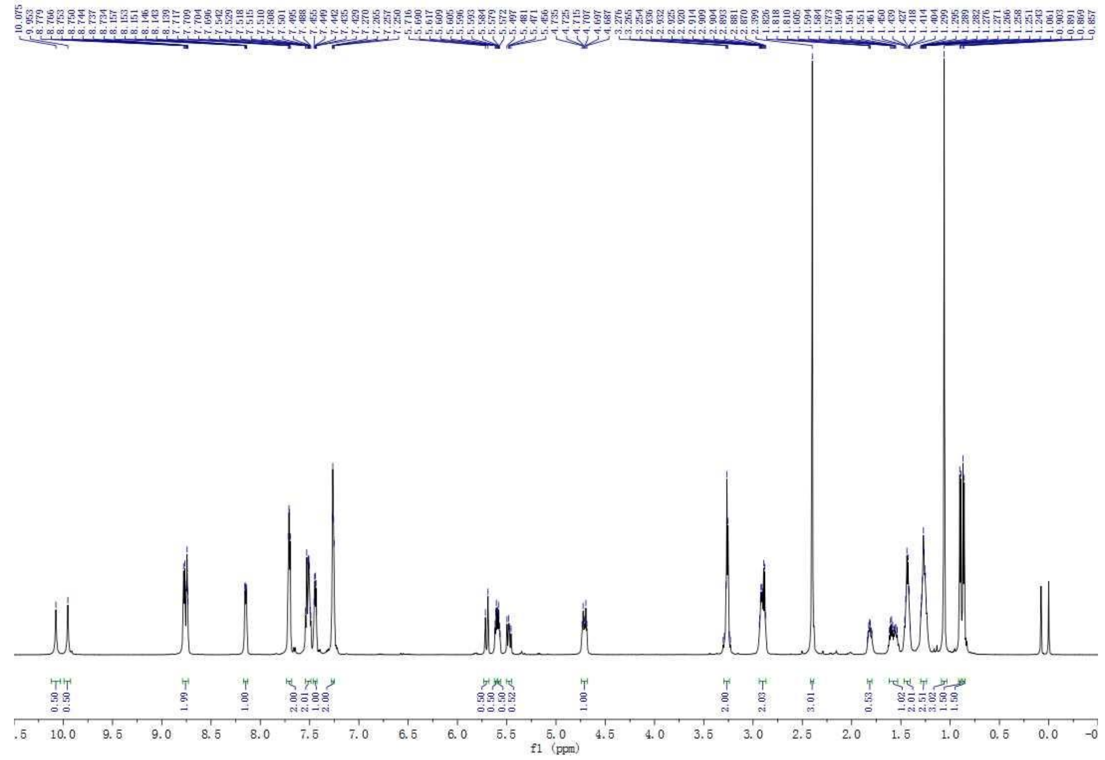

Supplementary Fig. 219. ^{1}H NMR of compound 3az + 3az′ (1:1). The sample has been recorded in 600 MHz, $CDCl_3$ at 25 °C

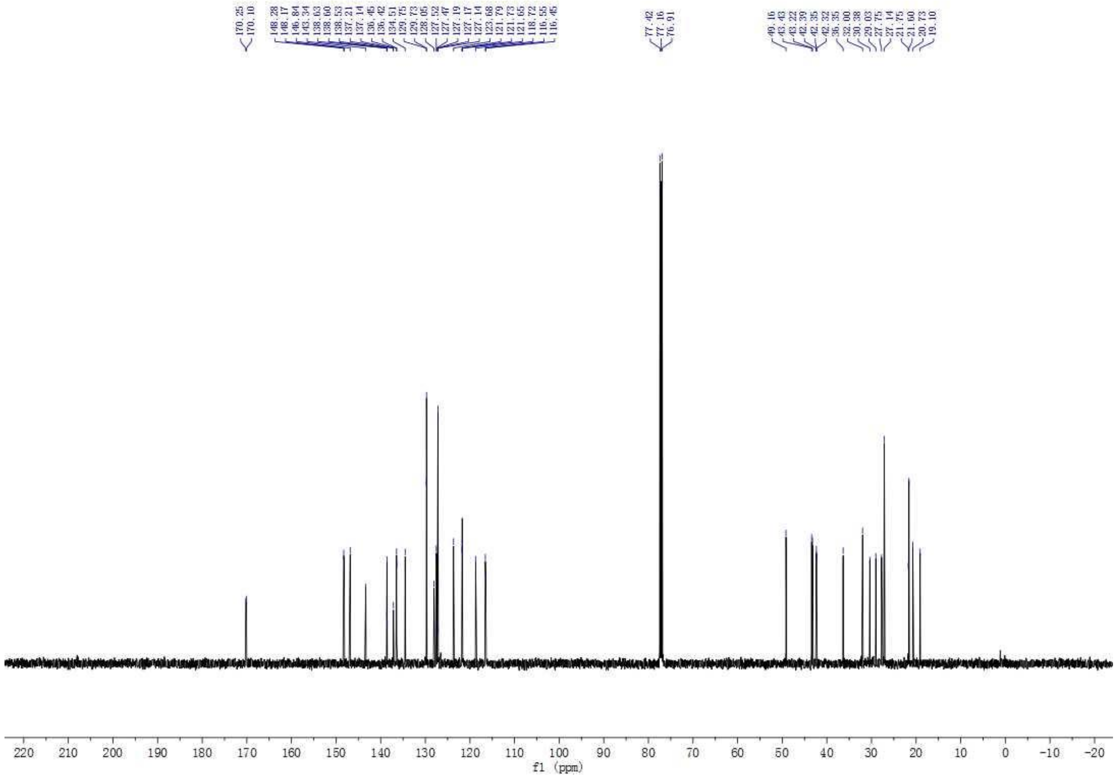

Supplementary Fig. 220. ^{13}C NMR of compound 3az + 3az′ (1:1). The sample has been recorded in 125 MHz, $CDCl_3$ at 25 °C

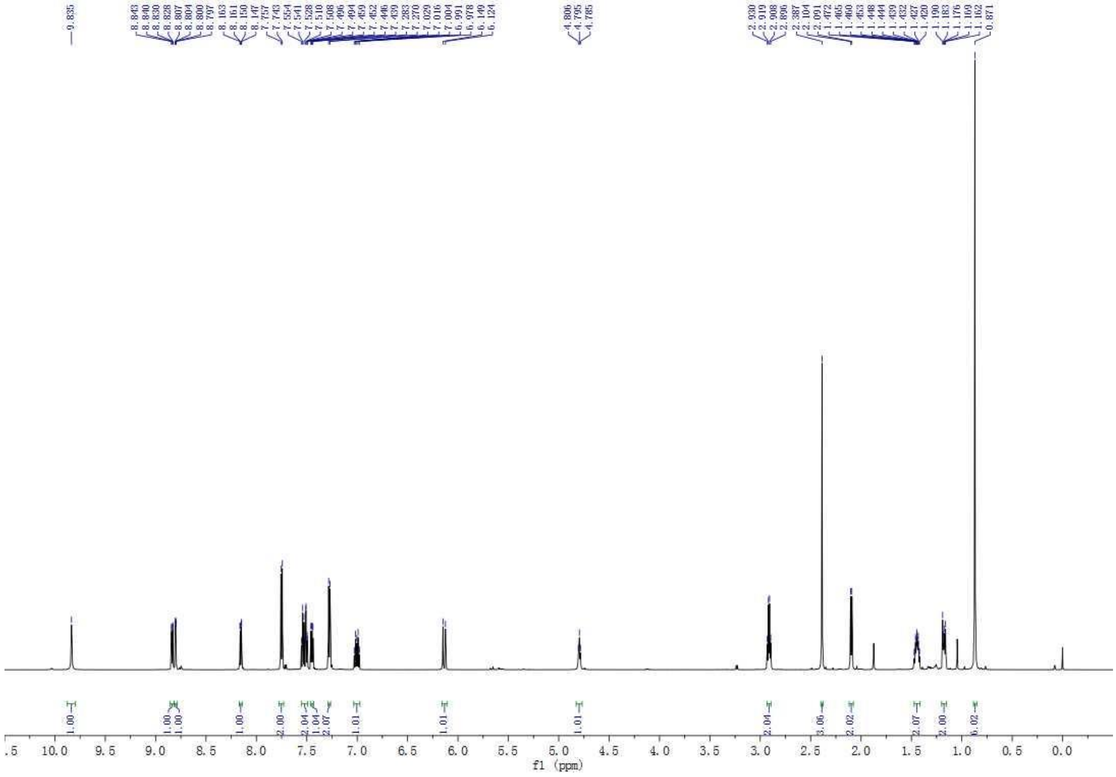

Supplementary Fig. 221. ^{1}H NMR of compound 4a. The sample has been recorded in 600 MHz, $CDCl_3$ at 25 °C

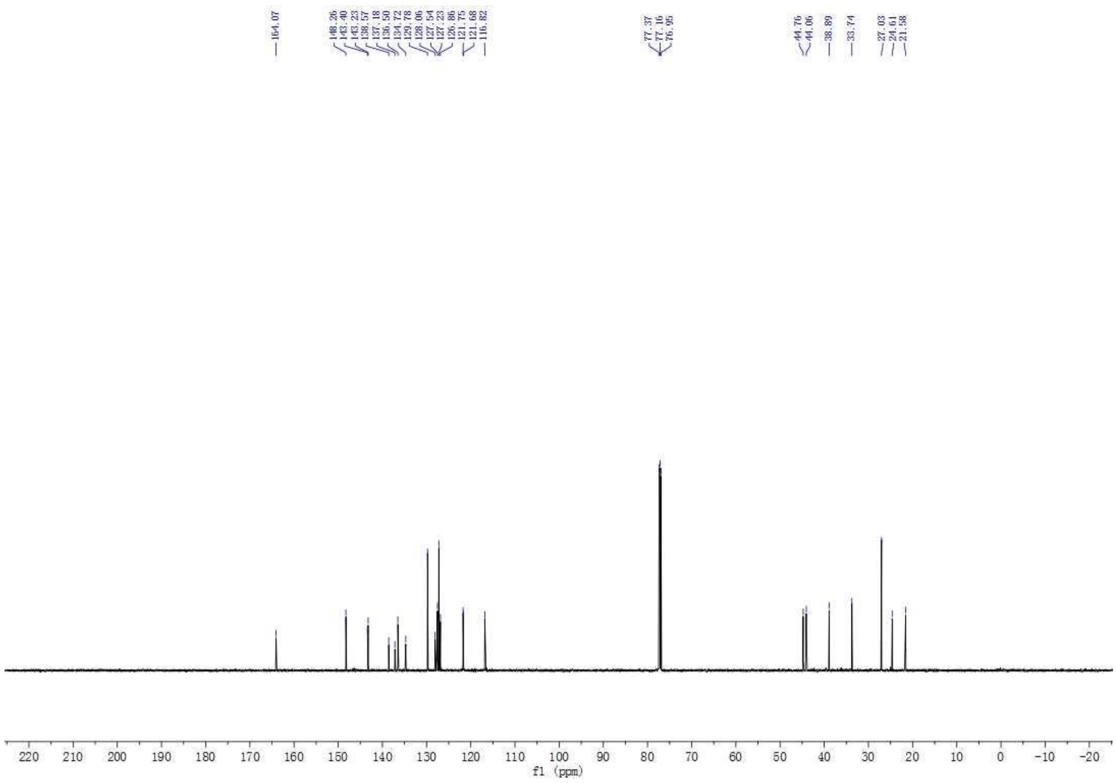

Supplementary Fig. 222. ^{13}C NMR of compound 4a. The sample has been recorded in 150 MHz, $CDCl_3$ at 25 °C

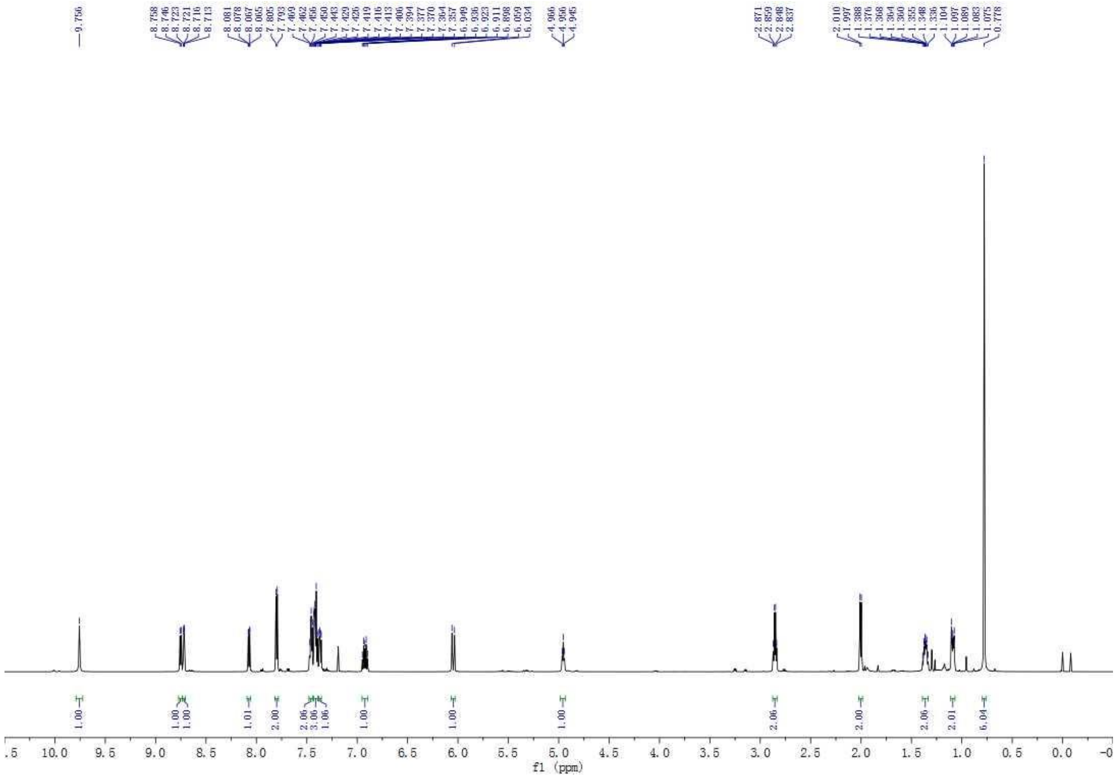

Supplementary Fig. 223. ^{1}H NMR of compound 4b. The sample has been recorded in 600 MHz, $CDCl_3$ at 25 °C

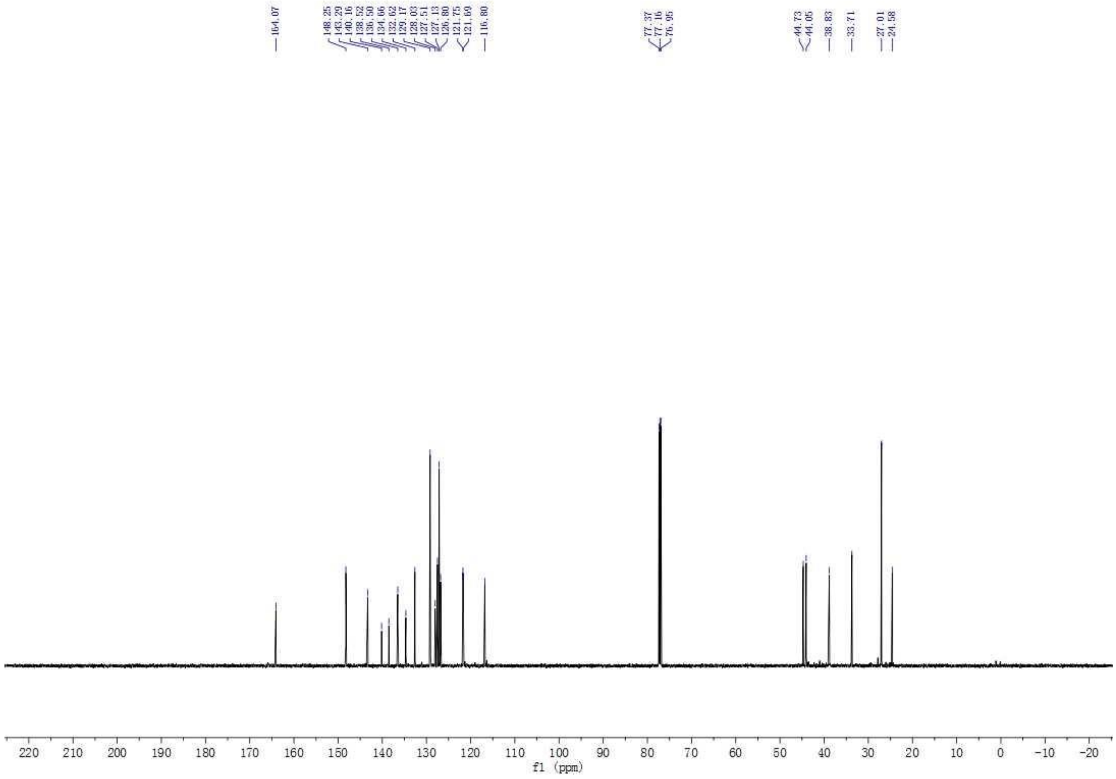

Supplementary Fig. 224. ^{13}C NMR of compound 4b. The sample has been recorded in 150 MHz, $CDCl_3$ at 25 °C

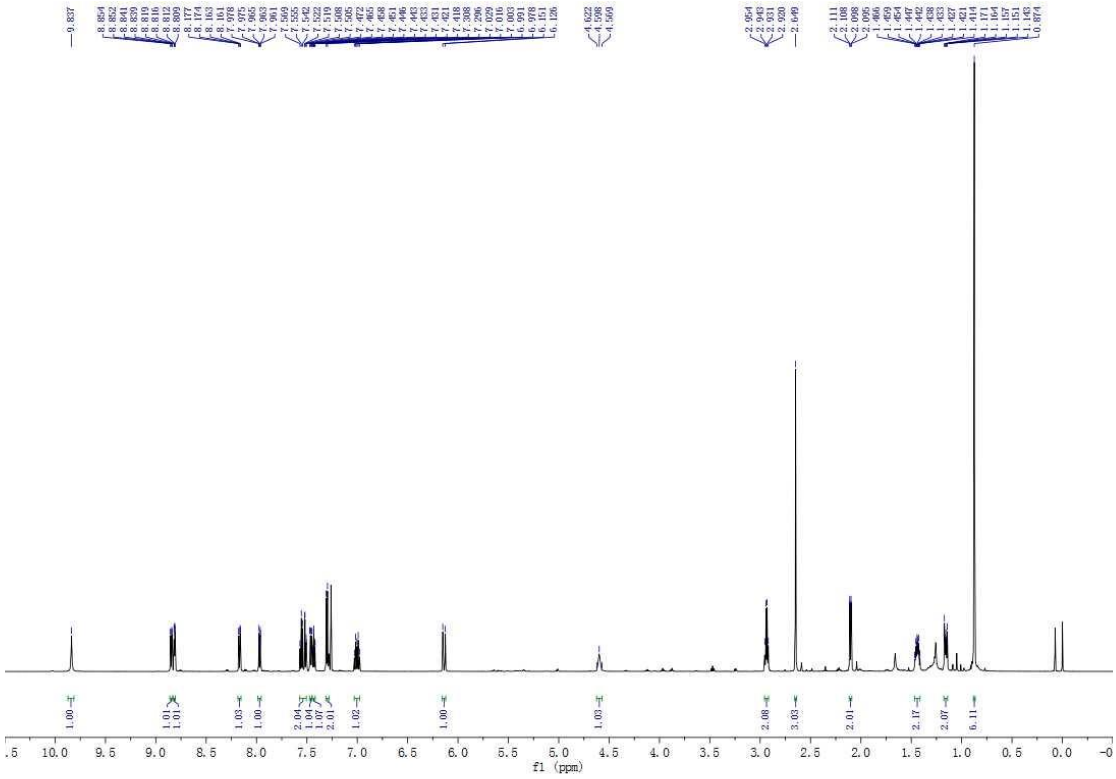

Supplementary Fig. 225. ^{1}H NMR of compound 4c. The sample has been recorded in 600 MHz, $CDCl_3$ at 25 °C

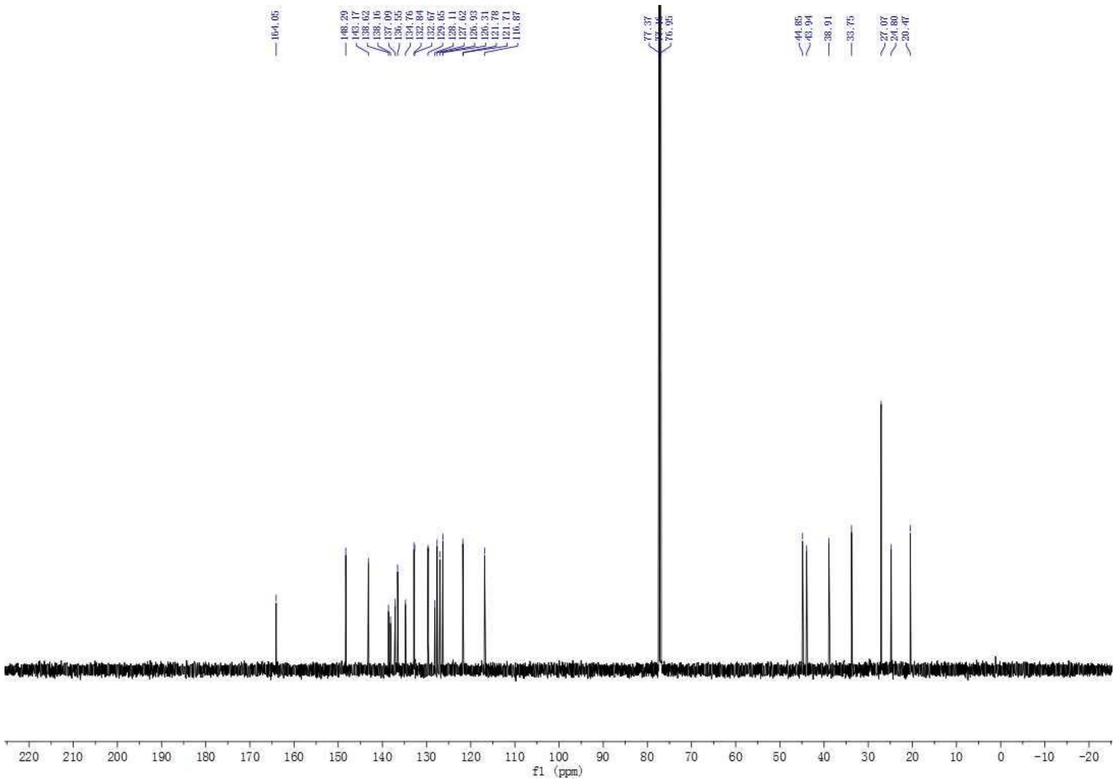

Supplementary Fig. 226. ^{13}C NMR of compound 4c. The sample has been recorded in 150 MHz, $CDCl_3$ at 25 °C

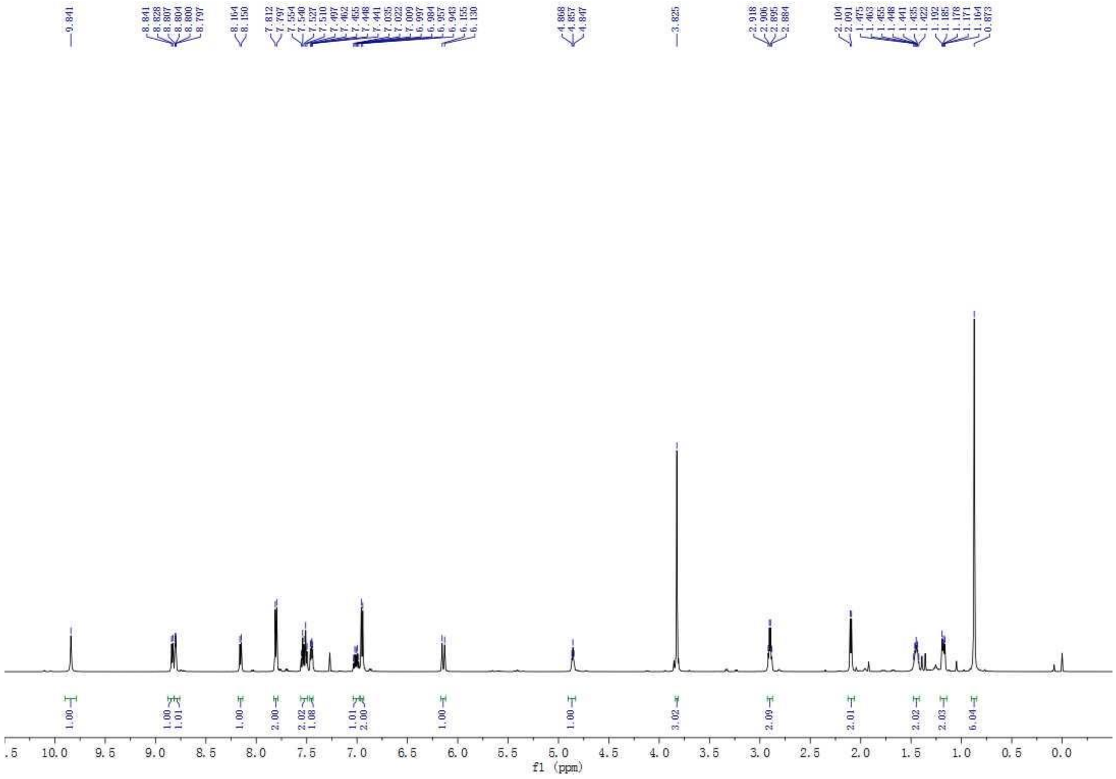

Supplementary Fig. 227. ^{1}H NMR of compound 4d. The sample has been recorded in 600 MHz, $CDCl_3$ at 25 °C

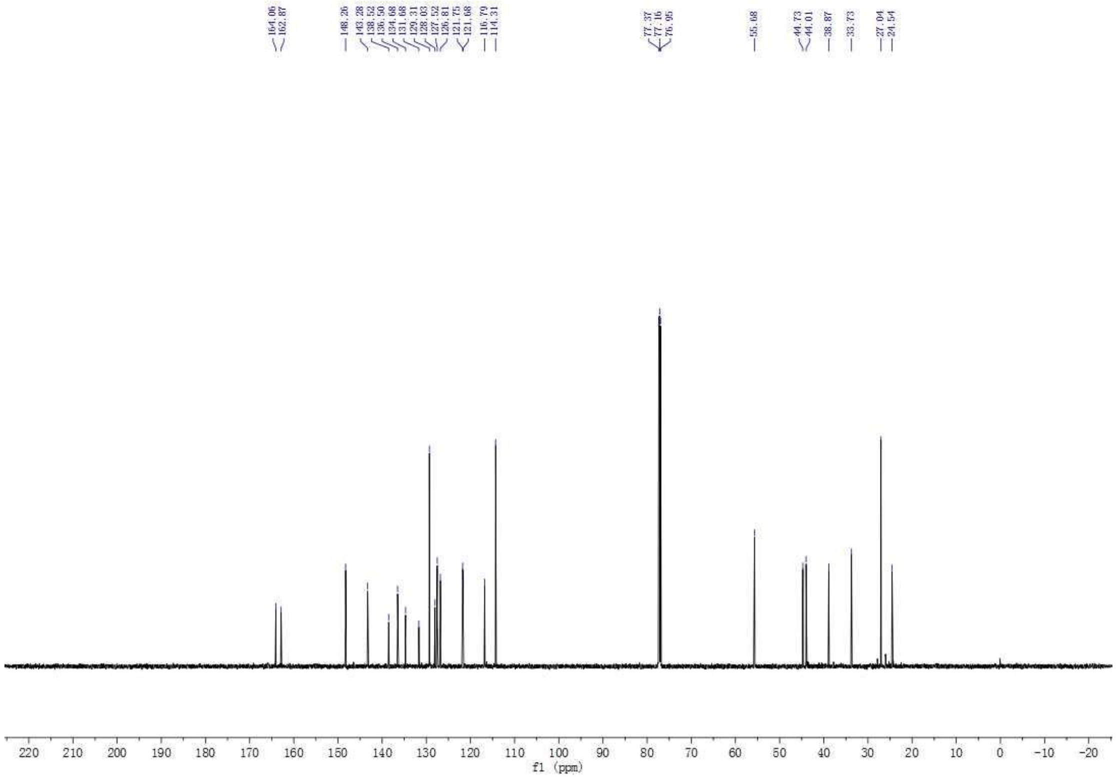

Supplementary Fig. 228. ^{13}C NMR of compound 4d. The sample has been recorded in 150 MHz, $CDCl_3$ at 25 °C

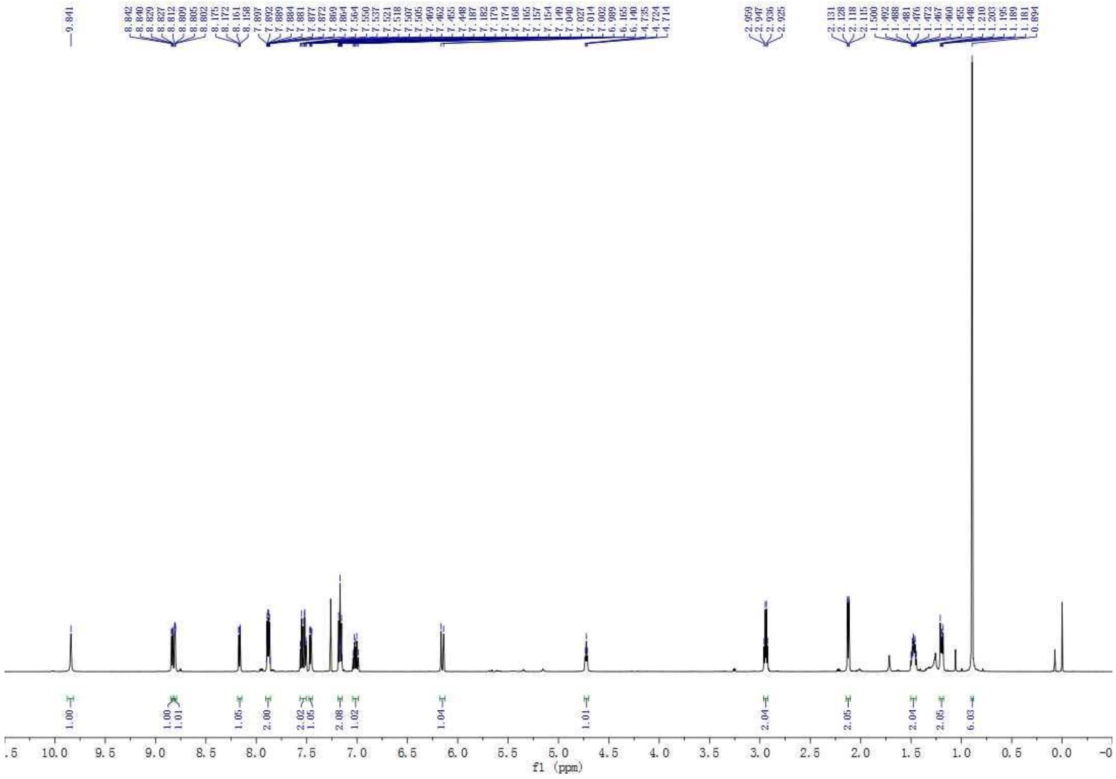

Supplementary Fig. 229. ^{1}H NMR of compound 4e. The sample has been recorded in 600 MHz, $CDCl_3$ at 25 °C

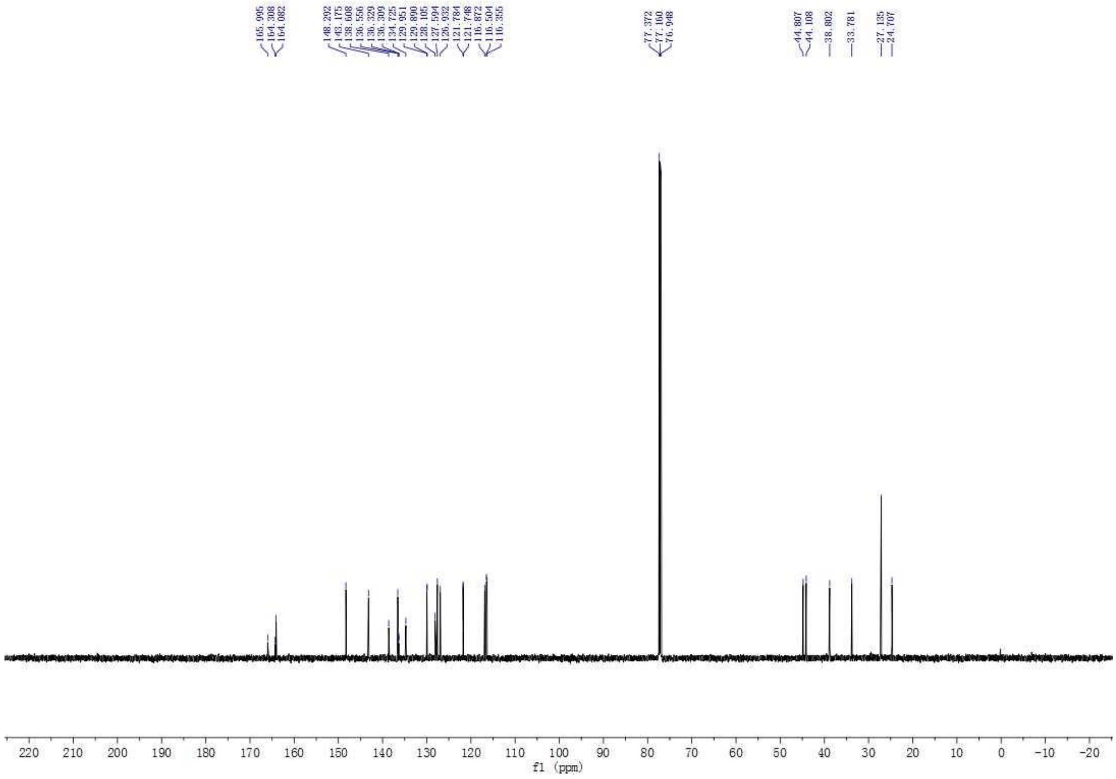

Supplementary Fig. 230. ^{13}C NMR of compound 4e. The sample has been recorded in 150 MHz, $CDCl_3$ at 25 °C

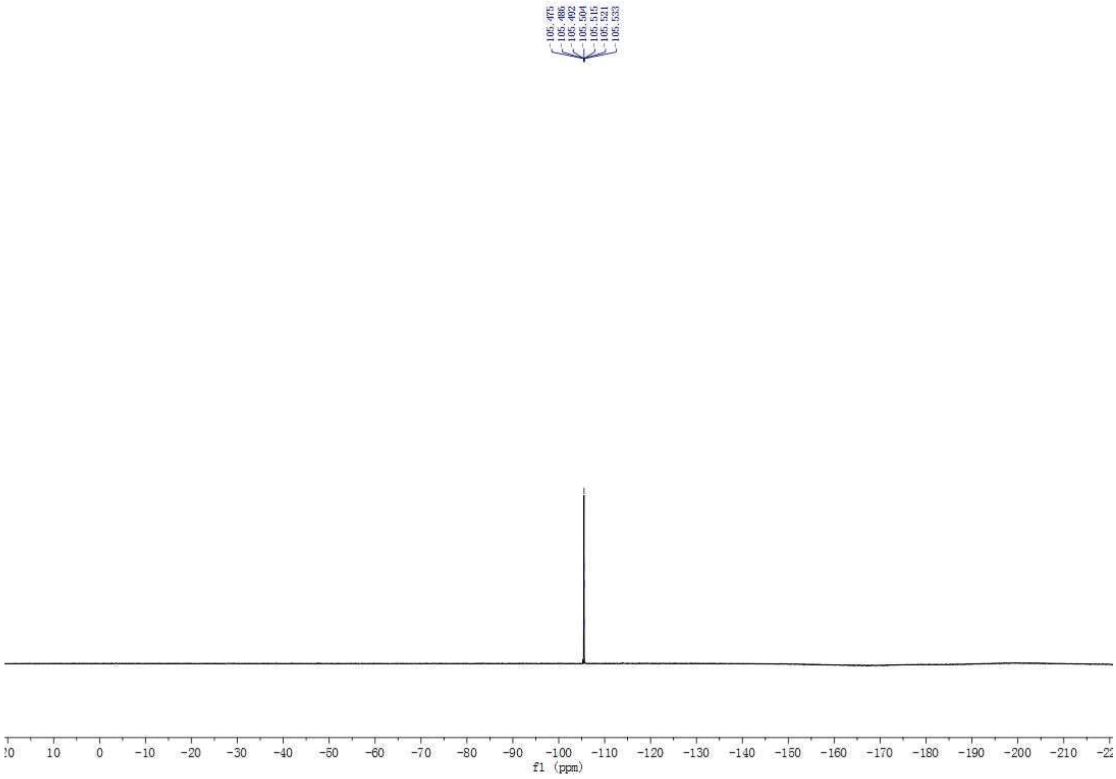

Supplementary Fig. 231. ^{19}F NMR of compound 4e. The sample has been recorded in 470 MHz, $CDCl_3$ at 25 °C

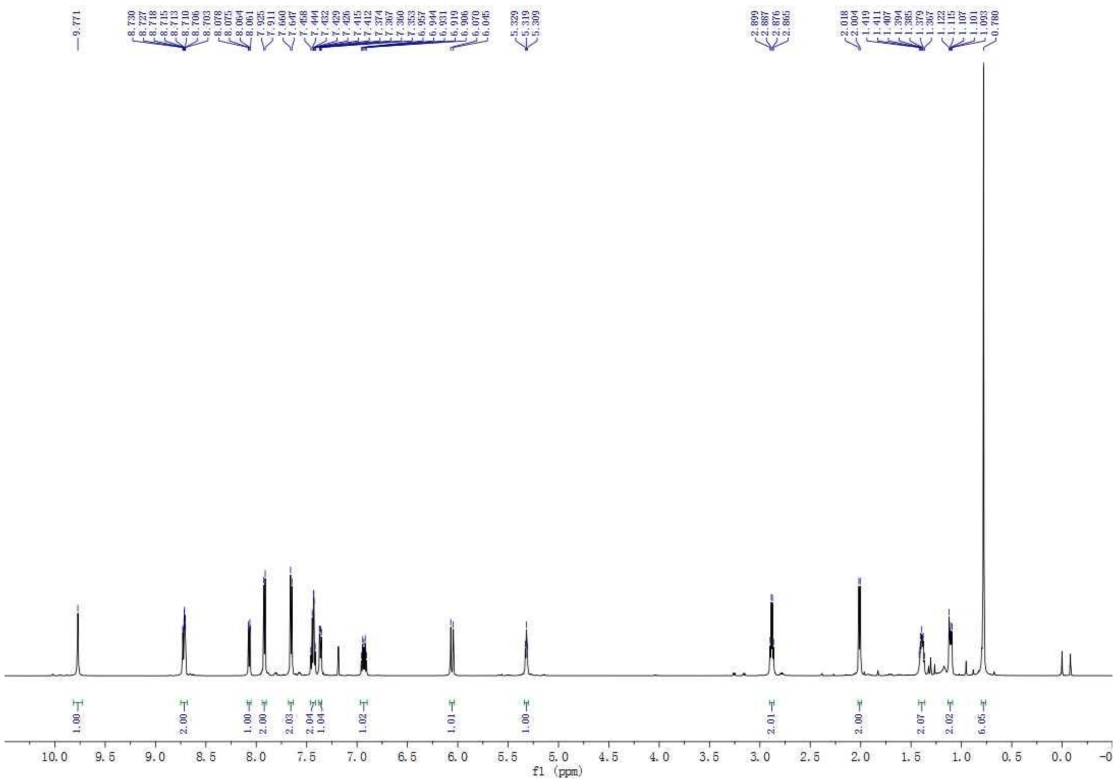

Supplementary Fig. 232. ^{1}H NMR of compound 4f. The sample has been recorded in 600 MHz, $CDCl_3$ at 25 °C

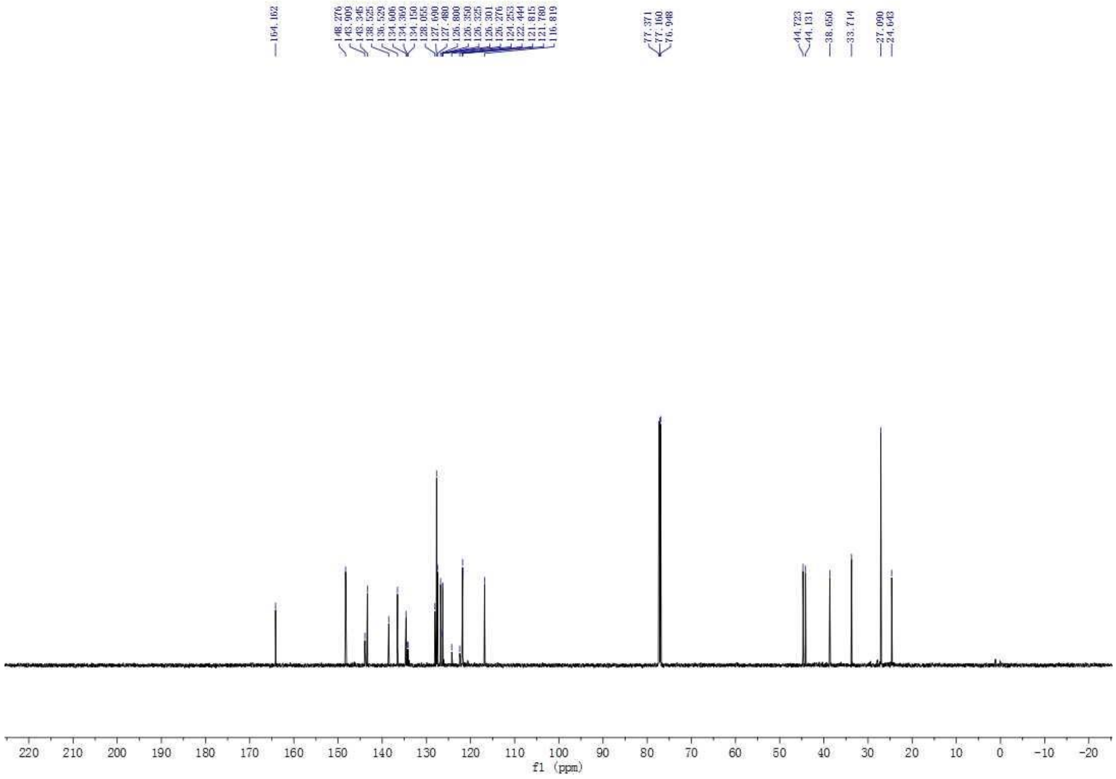

Supplementary Fig. 233. ^{13}C NMR of compound 4f. The sample has been recorded in 150 MHz, $CDCl_3$ at 25 °C

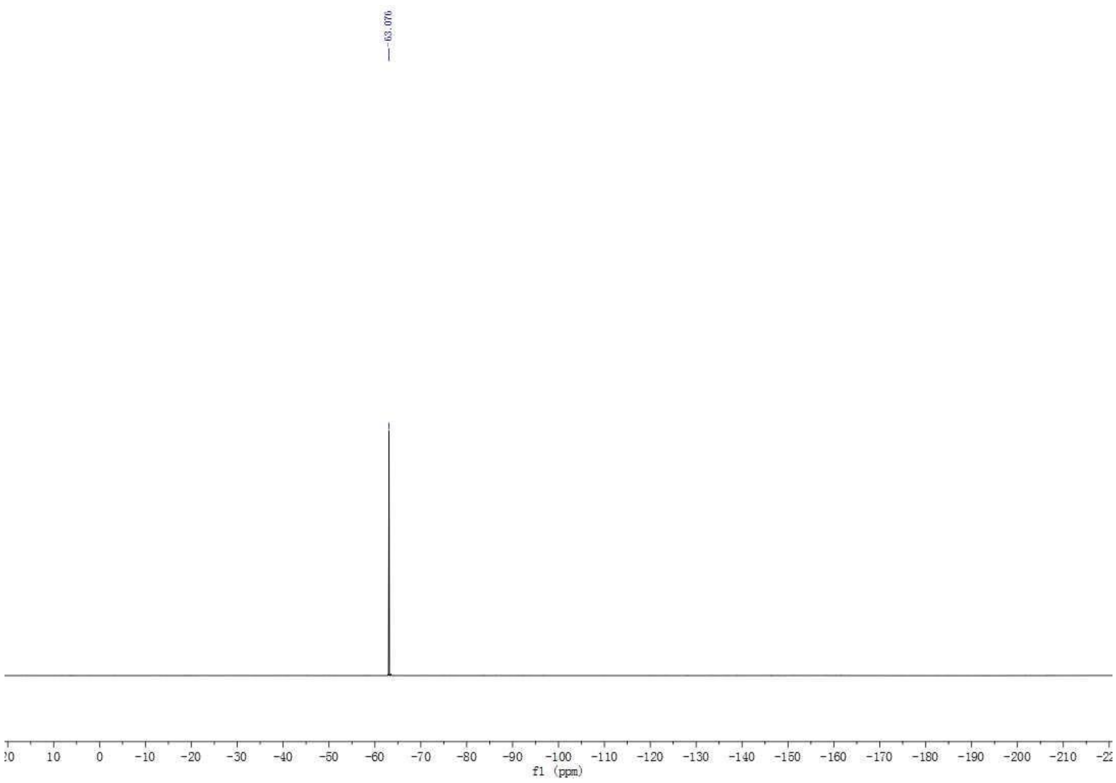

Supplementary Fig. 234. ^{19}F NMR of compound 4f. The sample has been recorded in 470 MHz, $CDCl_3$ at 25 °C

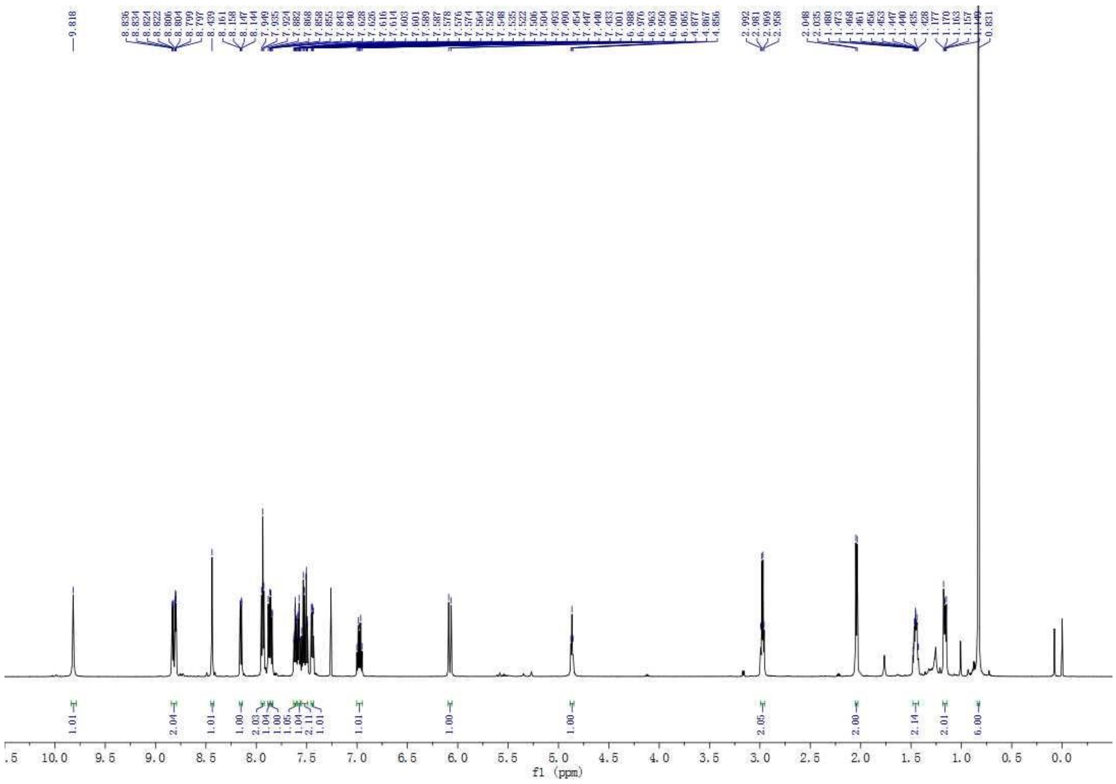

Supplementary Fig. 235. ^{1}H NMR of compound 4g. The sample has been recorded in 600 MHz, $CDCl_3$ at 25 °C

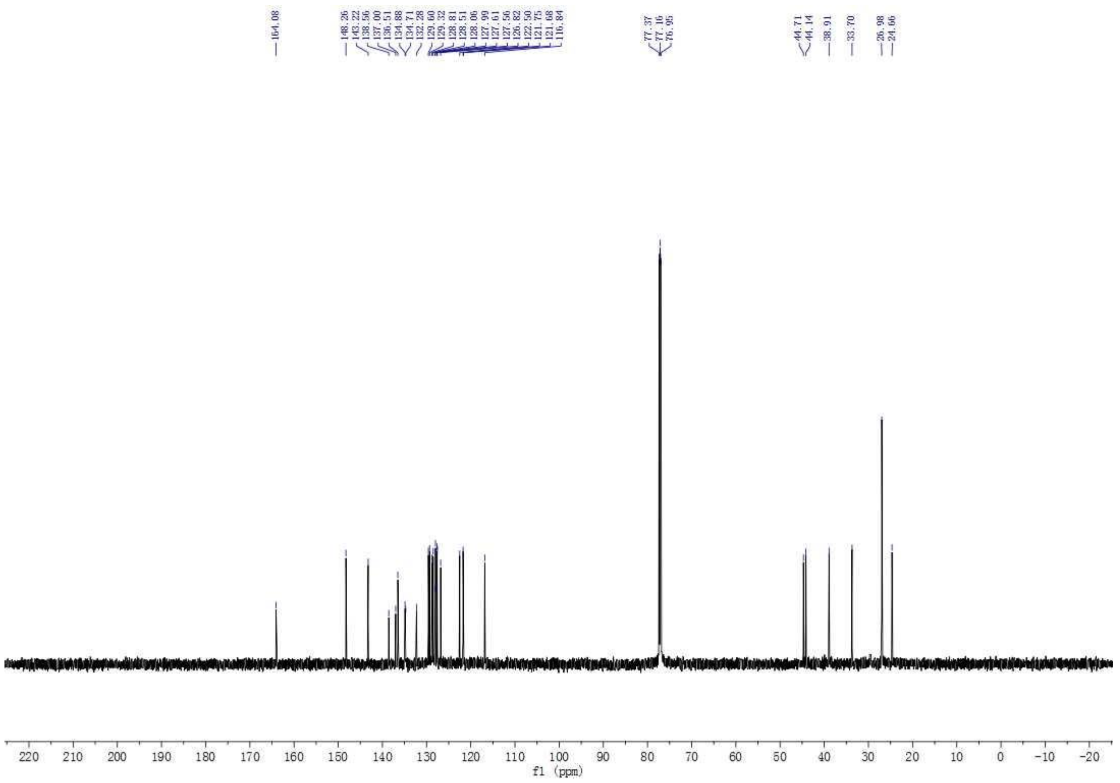

Supplementary Fig. 236. ^{13}C NMR of compound 4g. The sample has been recorded in 150 MHz, $CDCl_3$ at 25 °C

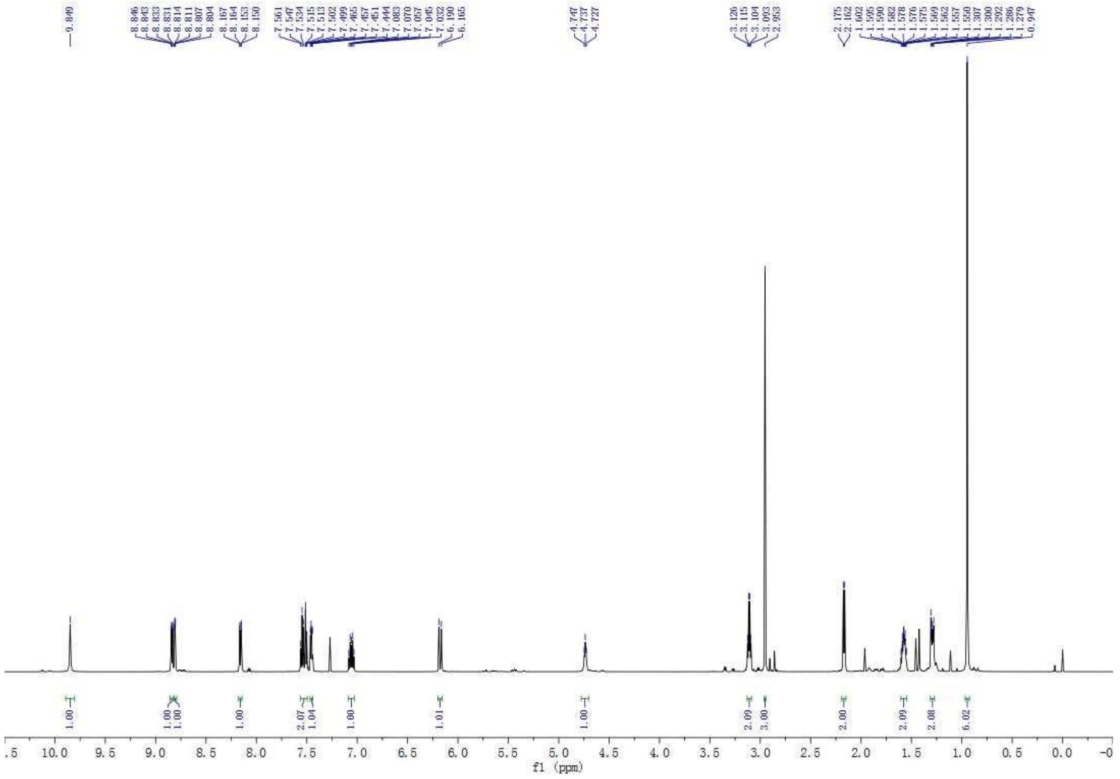

Supplementary Fig. 237. ^{1}H NMR of compound 4h. The sample has been recorded in 600 MHz, $CDCl_3$ at 25 °C

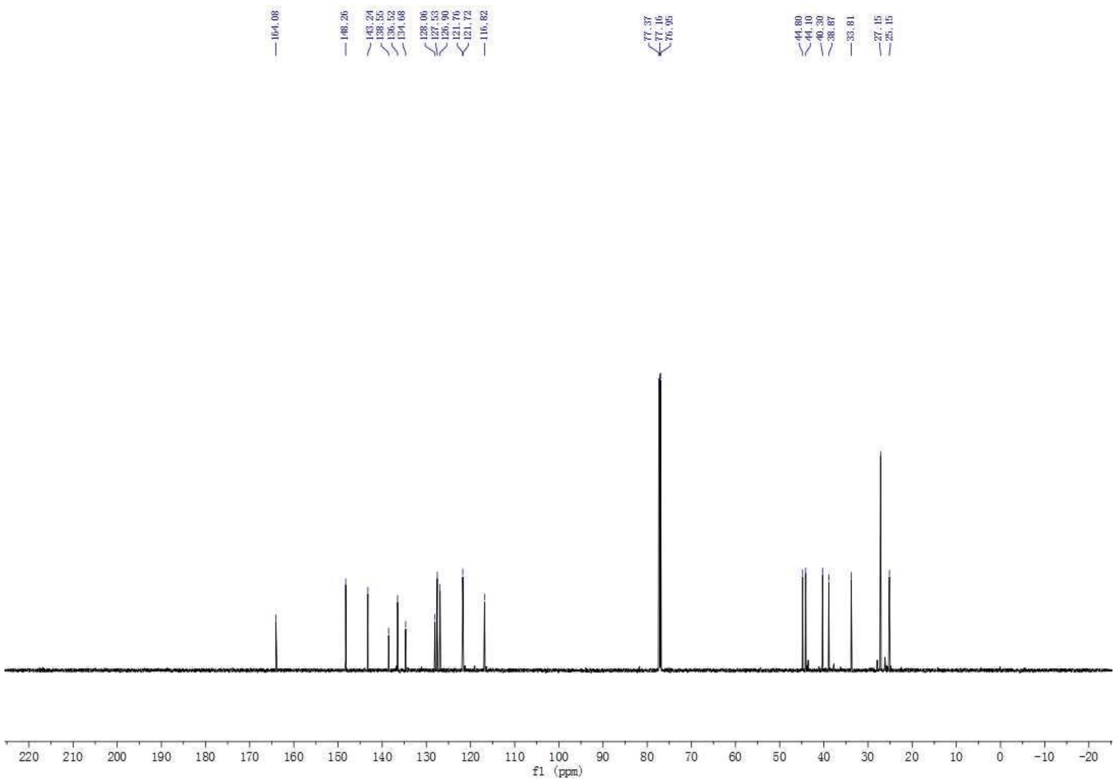

Supplementary Fig. 238. ^{13}C NMR of compound 4h. The sample has been recorded in 150 MHz, $CDCl_3$ at 25 °C

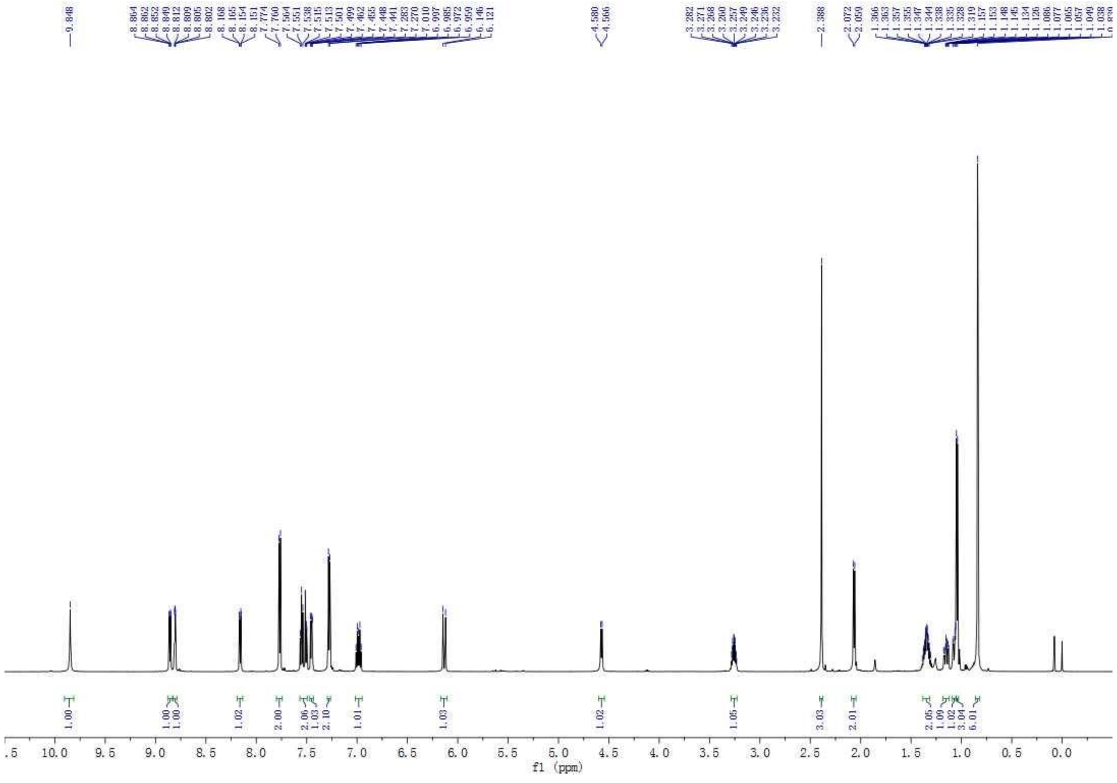

Supplementary Fig. 239. ^{1}H NMR of compound 4i. The sample has been recorded in 600 MHz, $CDCl_3$ at 25 °C

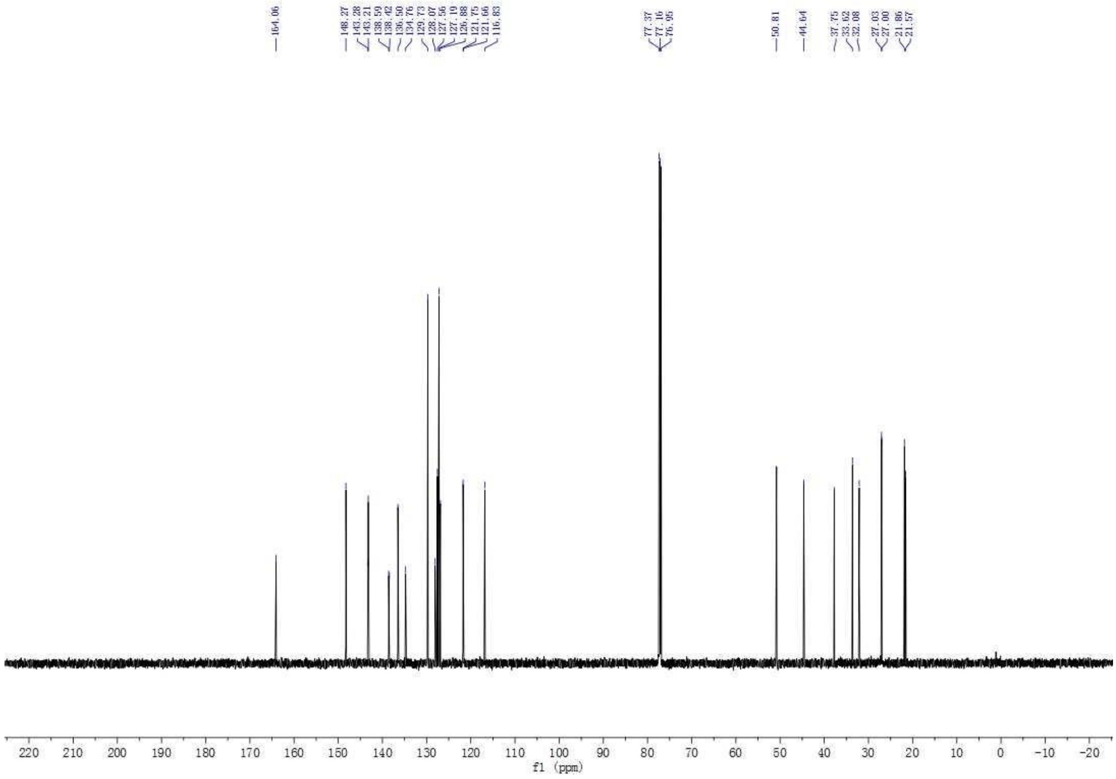

Supplementary Fig. 240. ^{13}C NMR of compound 4i. The sample has been recorded in 150 MHz, $CDCl_3$ at 25 °C

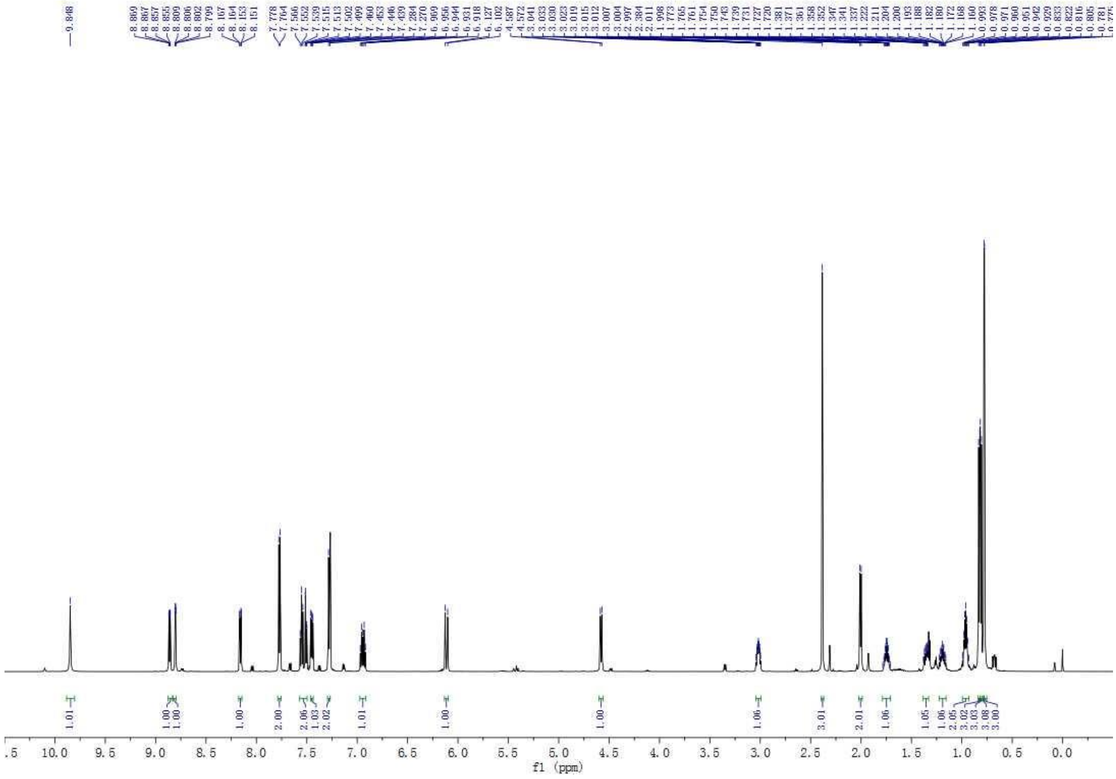

Supplementary Fig. 241. ^{1}H NMR of compound 4j. The sample has been recorded in 600 MHz, $CDCl_3$ at 25 °C

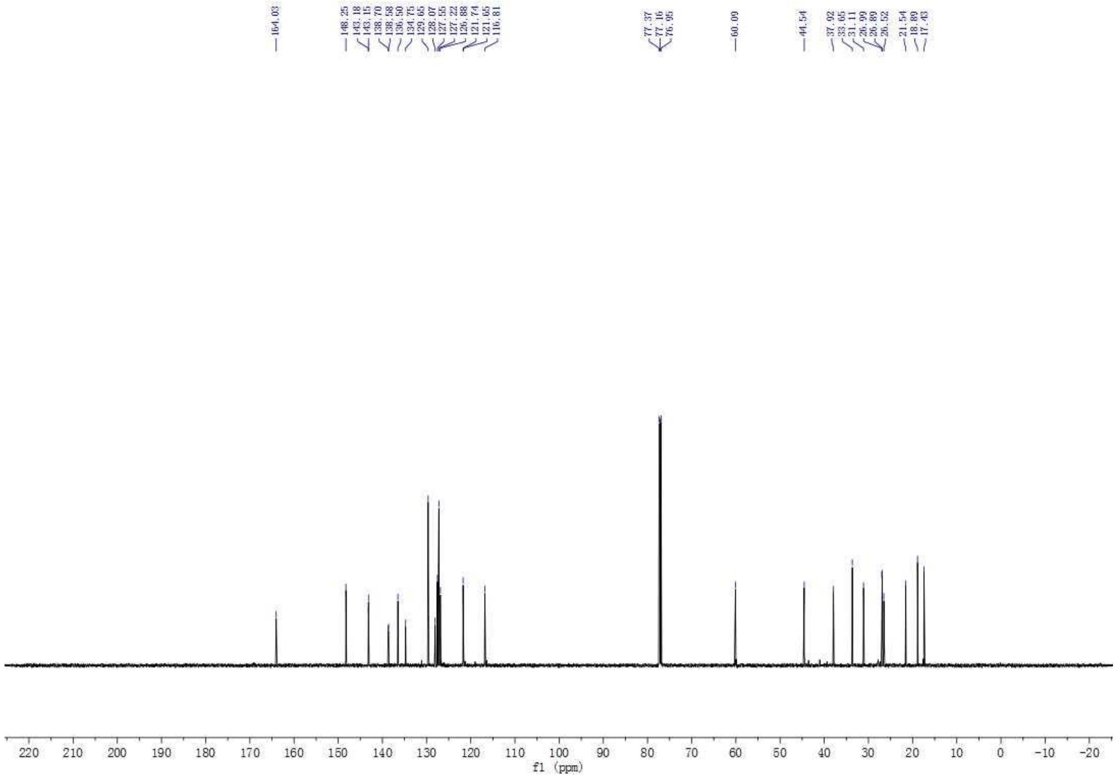

Supplementary Fig. 242. ^{13}C NMR of compound 4j. The sample has been recorded in 150 MHz, $CDCl_3$ at 25 °C

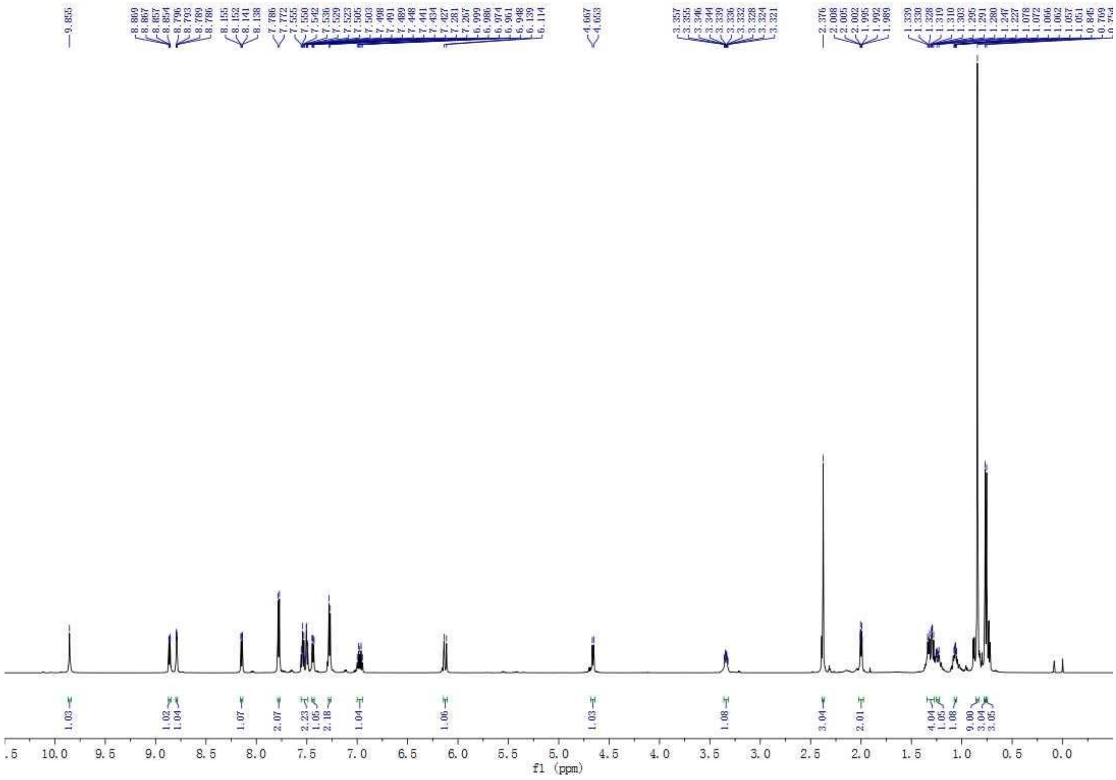

Supplementary Fig. 243. ^{1}H NMR of compound 4k. The sample has been recorded in 600 MHz, $CDCl_3$ at 25 °C

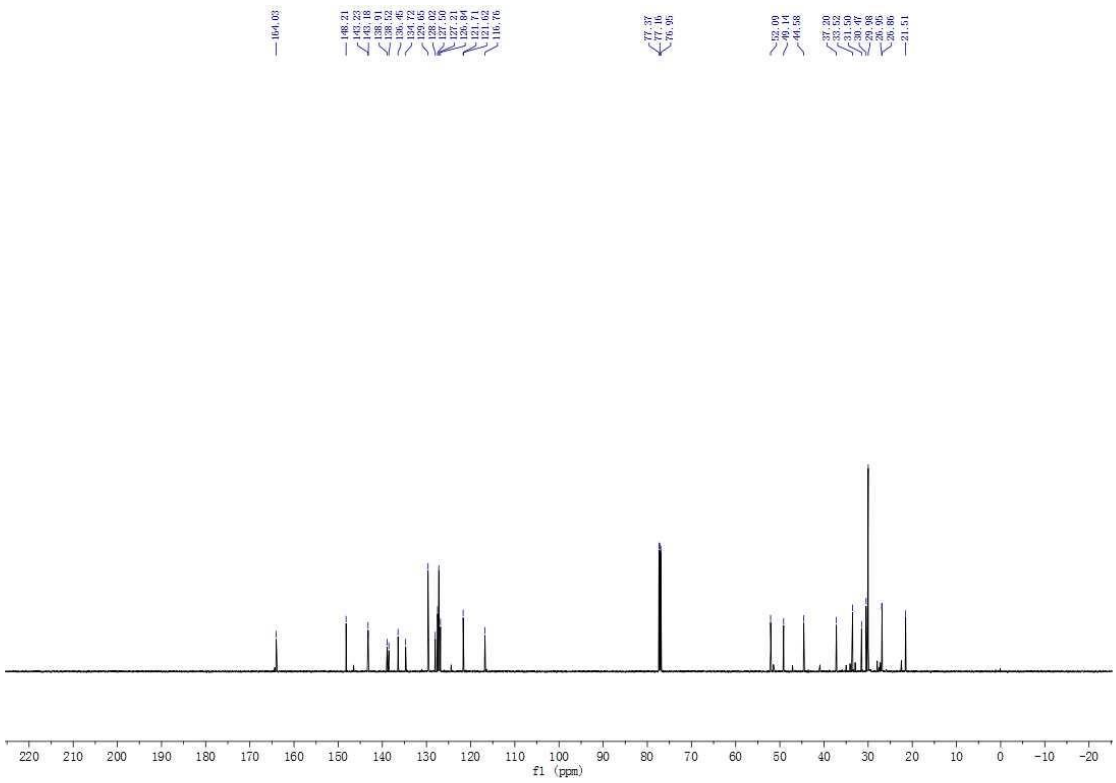

Supplementary Fig. 244. ^{13}C NMR of compound 4k. The sample has been recorded in 150 MHz, $CDCl_3$ at 25 °C

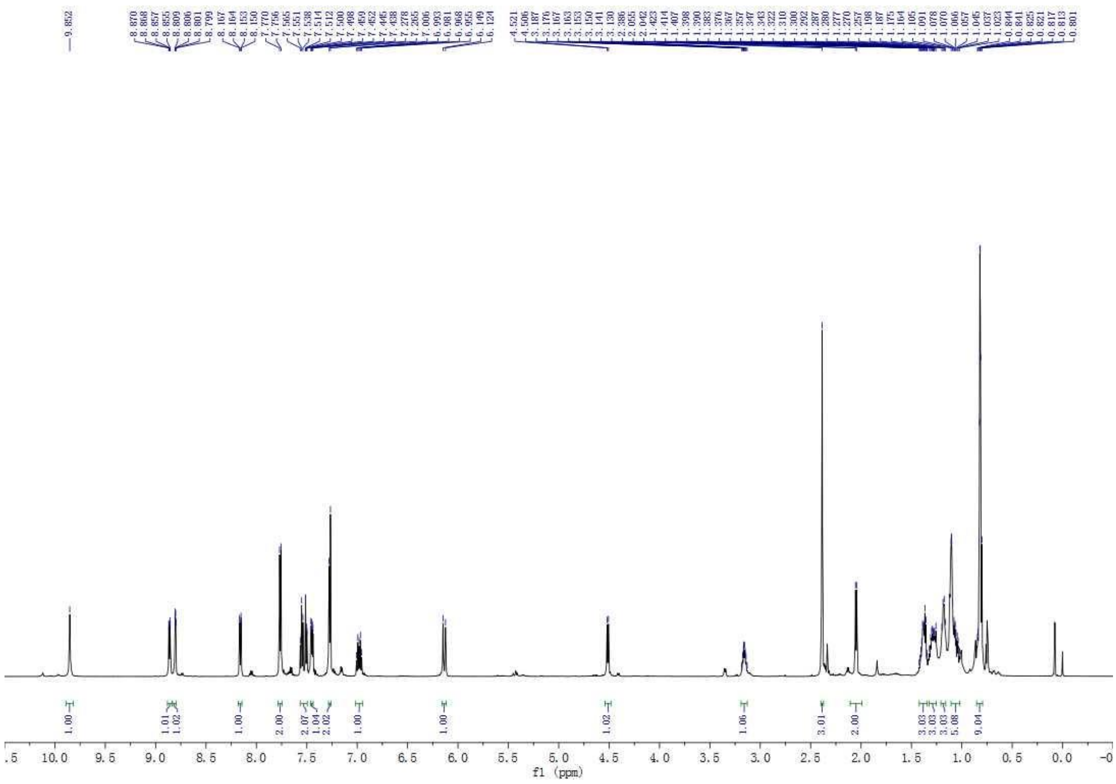

Supplementary Fig. 245. ^{1}H NMR of compound 4l. The sample has been recorded in 600 MHz, $CDCl_3$ at 25 °C

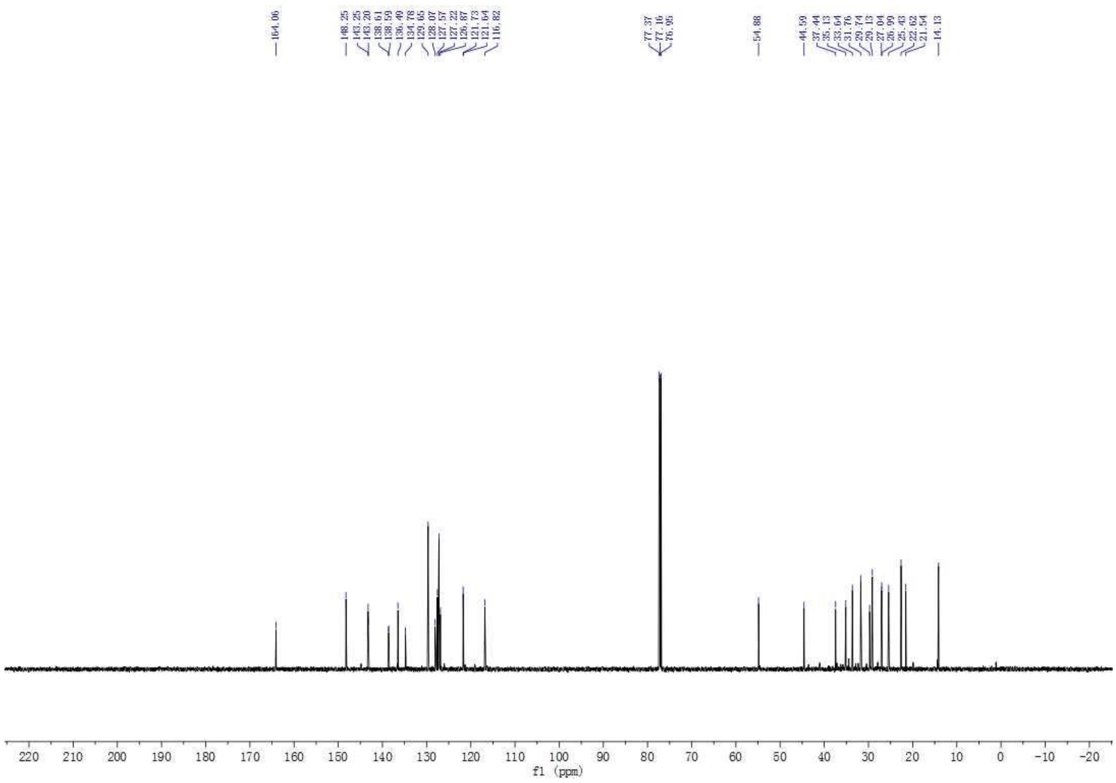

Supplementary Fig. 246. ^{13}C NMR of compound 4l. The sample has been recorded in 150 MHz, $CDCl_3$ at 25 °C

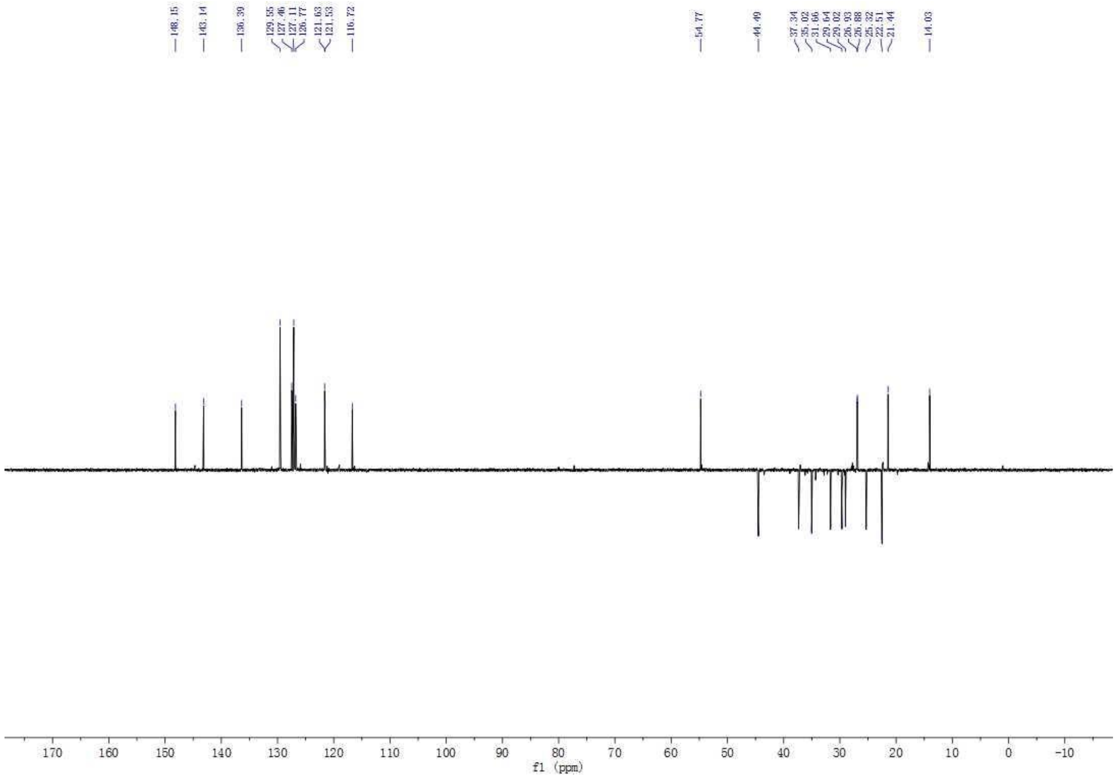

Supplementary Fig. 247. Dept 135° of compound 4l. The sample has been recorded in 150 MHz, $CDCl_3$ at 25 °C

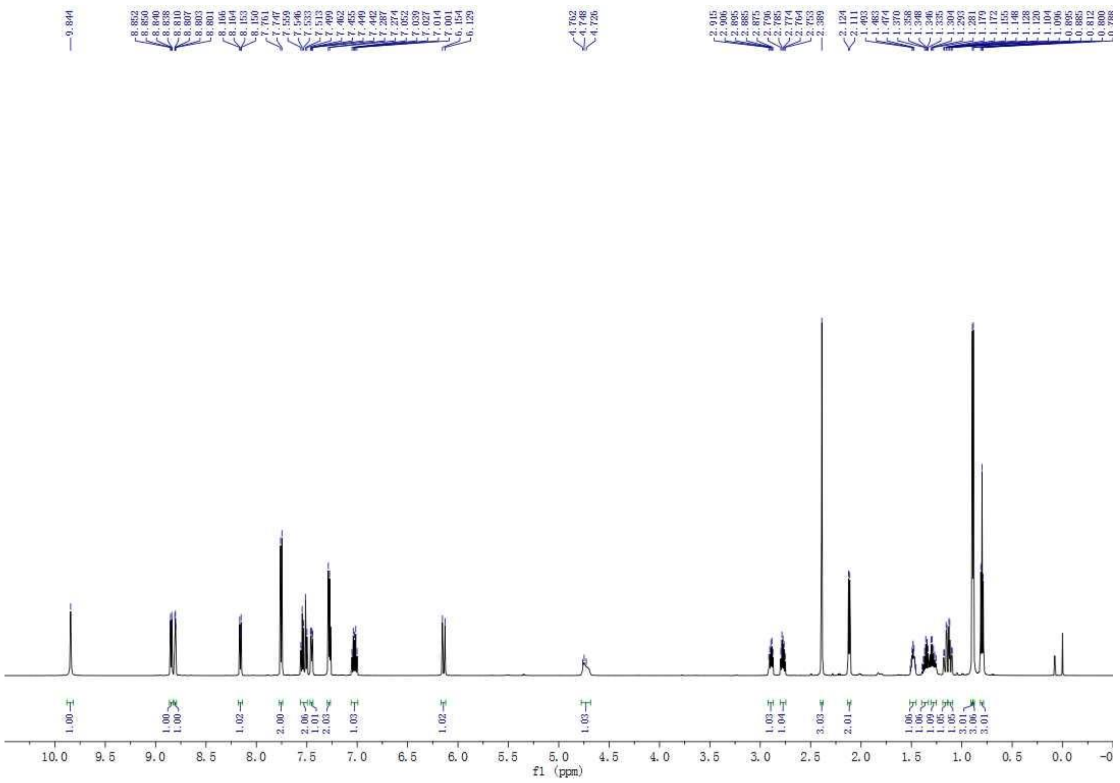

Supplementary Fig. 248. ^{1}H NMR of compound 4m. The sample has been recorded in 600 MHz, $CDCl_3$ at 25 °C

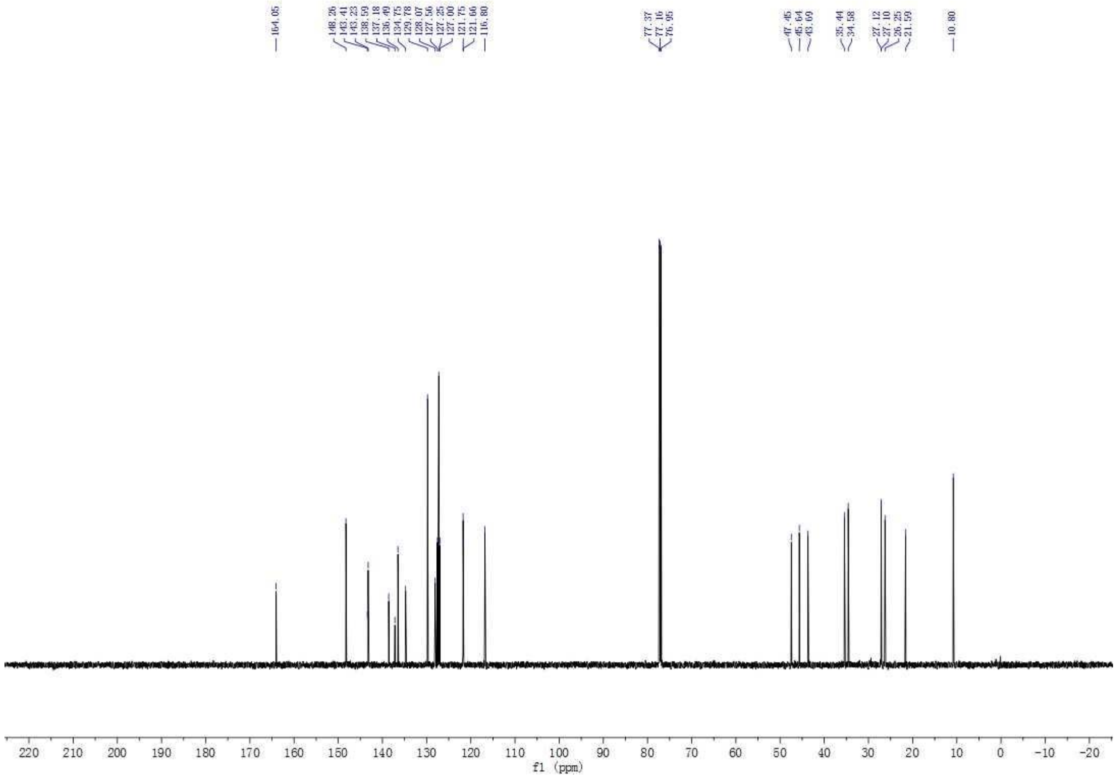

Supplementary Fig. 249. ^{13}C NMR of compound 4m. The sample has been recorded in 150 MHz, $CDCl_3$ at 25 °C

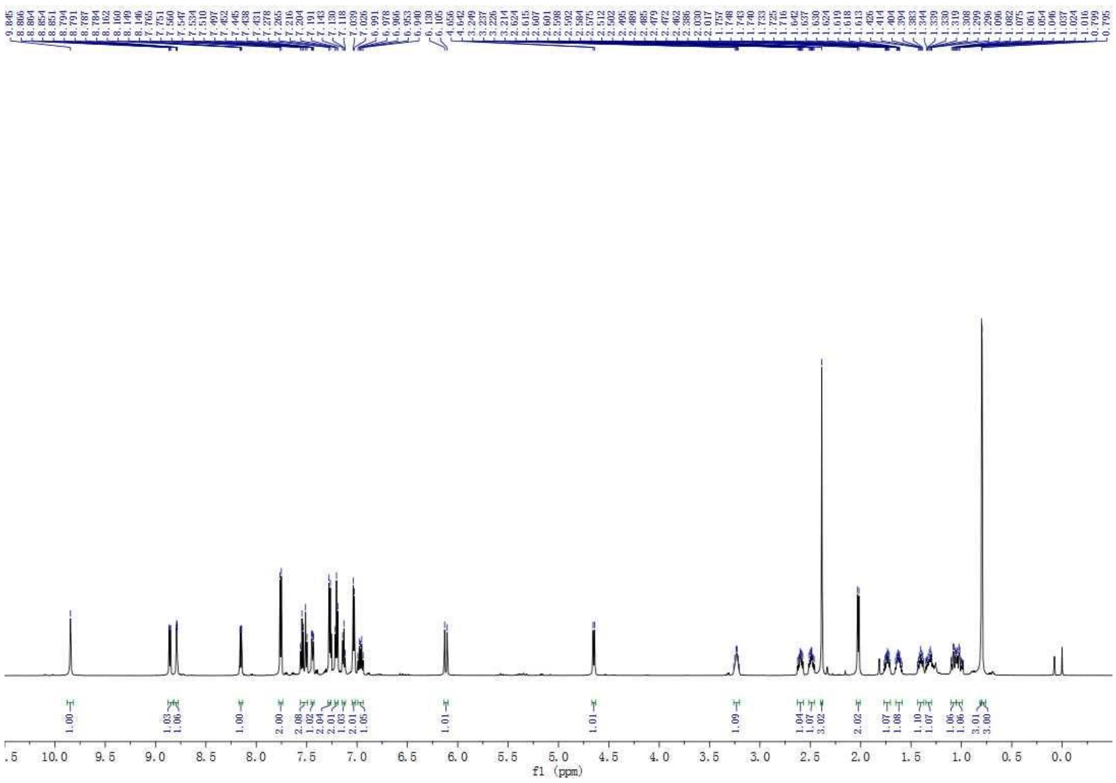

Supplementary Fig. 250. ^{1}H NMR of compound 4n. The sample has been recorded in 600 MHz, $CDCl_3$ at 25 °C

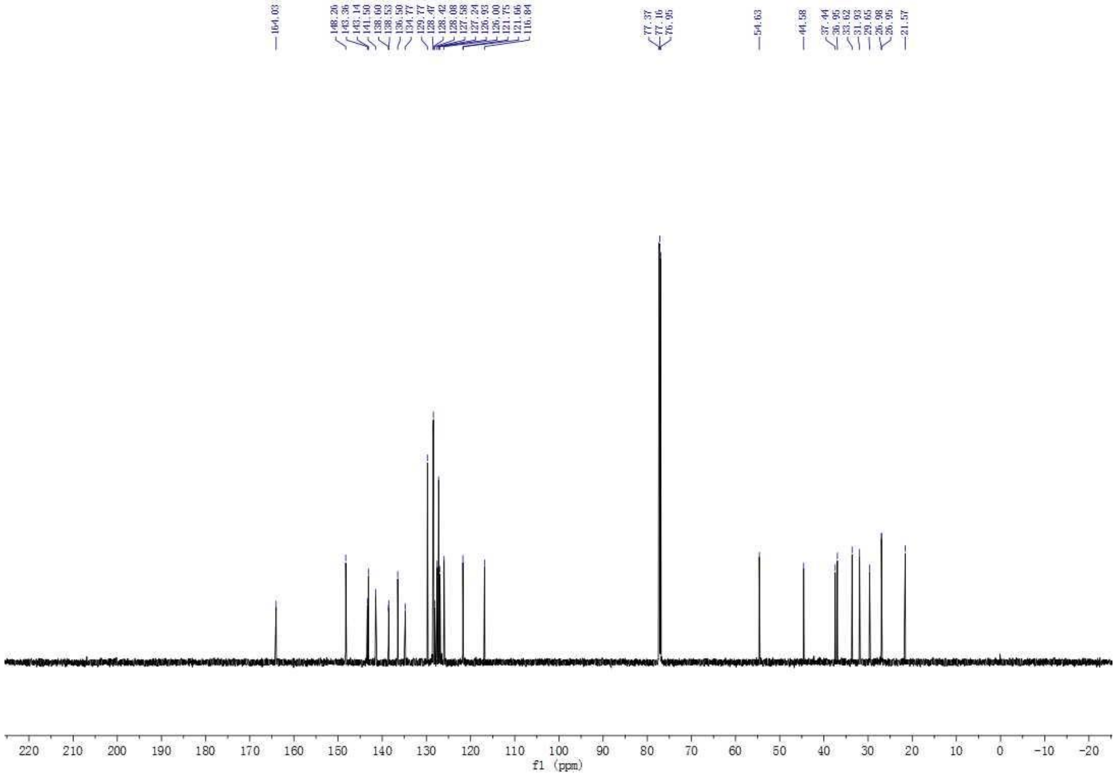

Supplementary Fig. 251. ^{13}C NMR of compound 4n. The sample has been recorded in 150 MHz, $CDCl_3$ at 25 °C

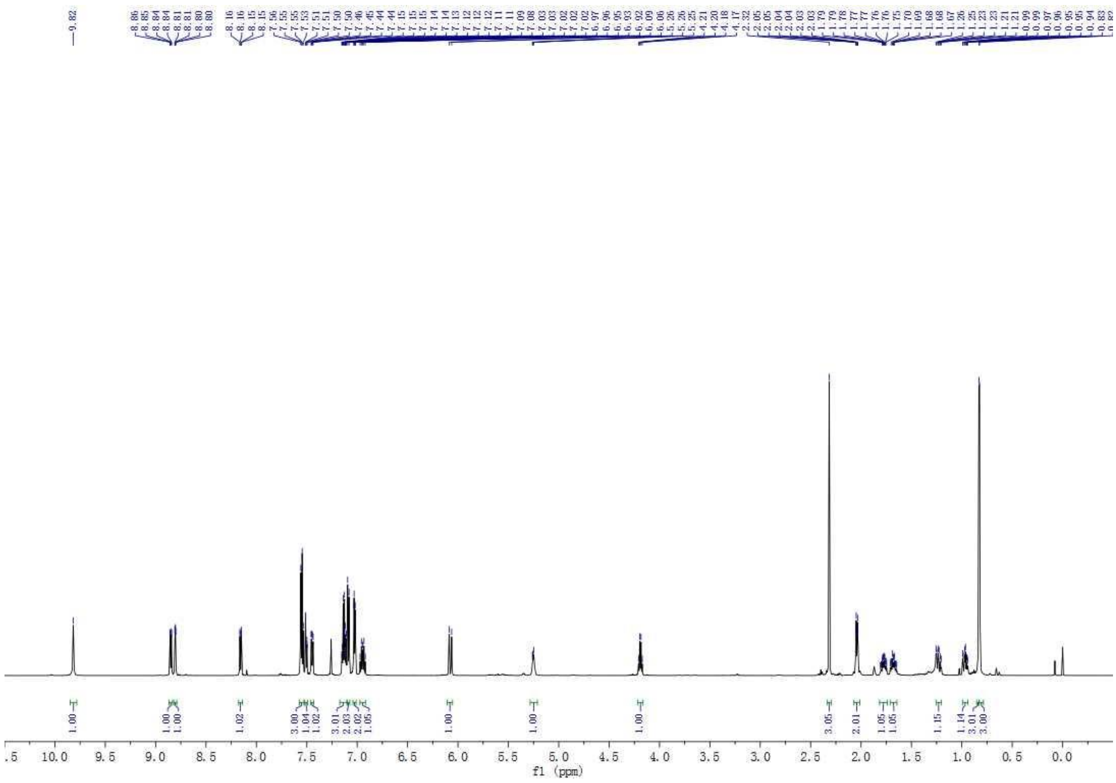

Supplementary Fig. 252. ¹H NMR of compound 4o. The sample has been recorded in 600 MHz, $CDCl_3$ at 25 °C

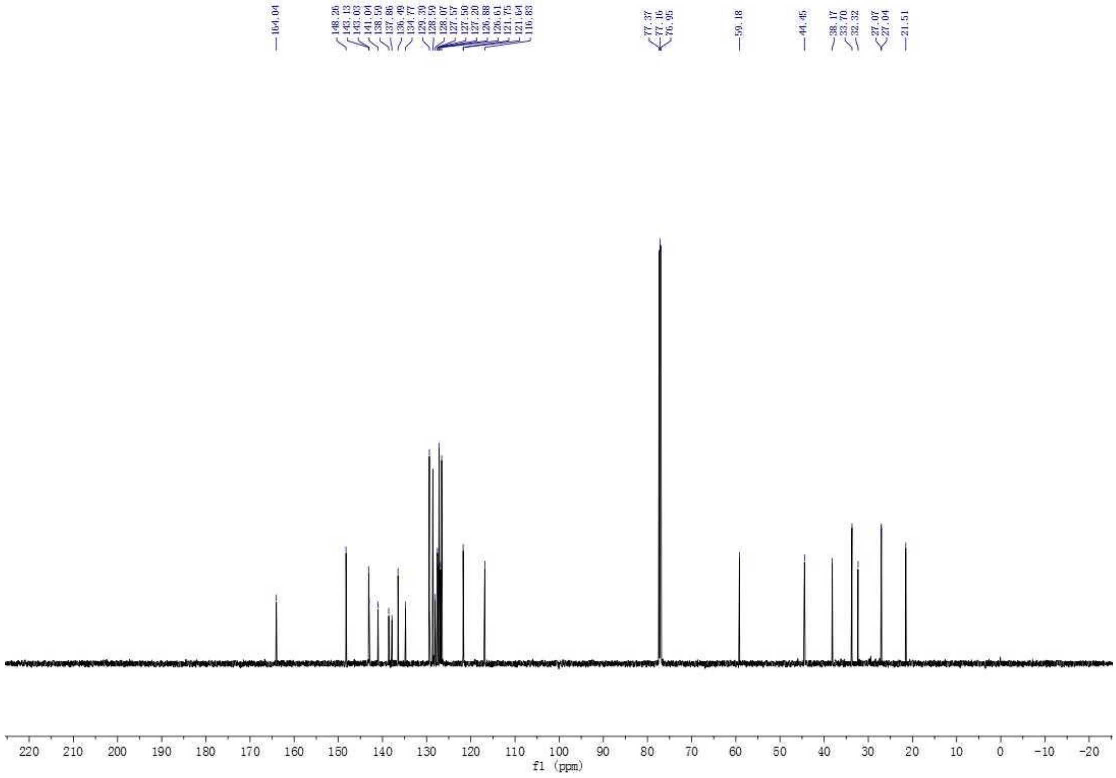

Supplementary Fig. 253. ¹³C NMR of compound 4o. The sample has been recorded in 150 MHz, $CDCl_3$ at 25 °C

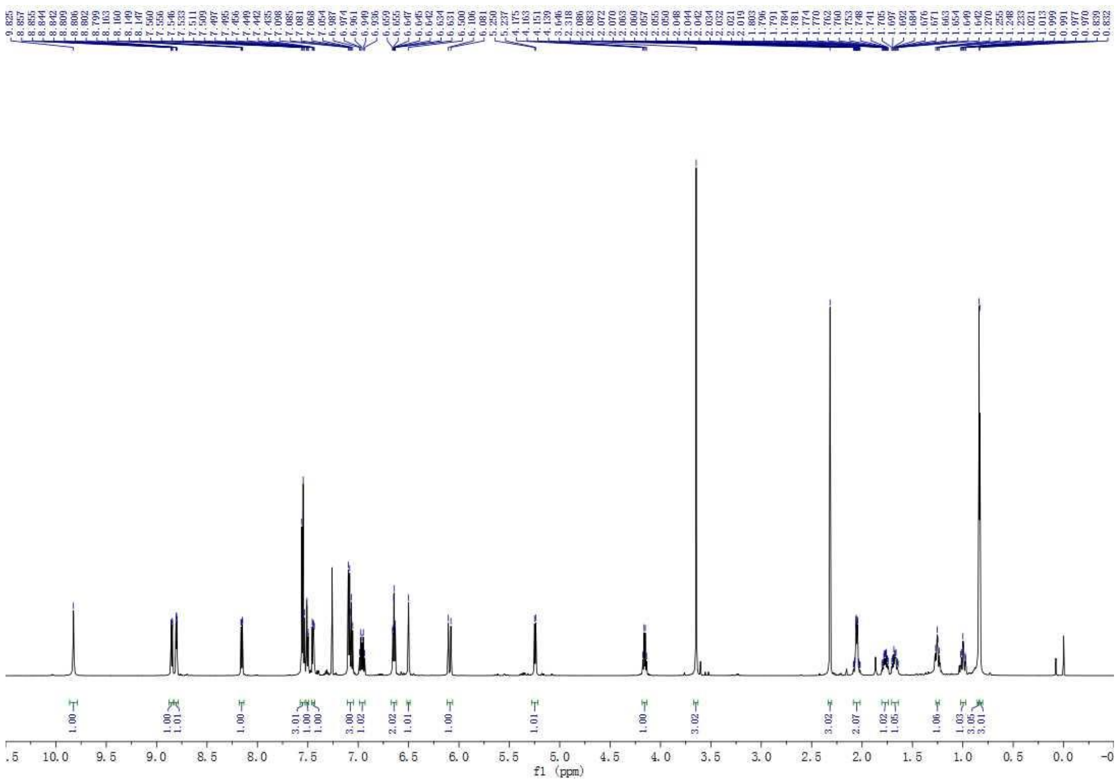

Supplementary Fig. 254. ^{1}H NMR of compound 4p. The sample has been recorded in 600 MHz, $CDCl_3$ at 25 °C

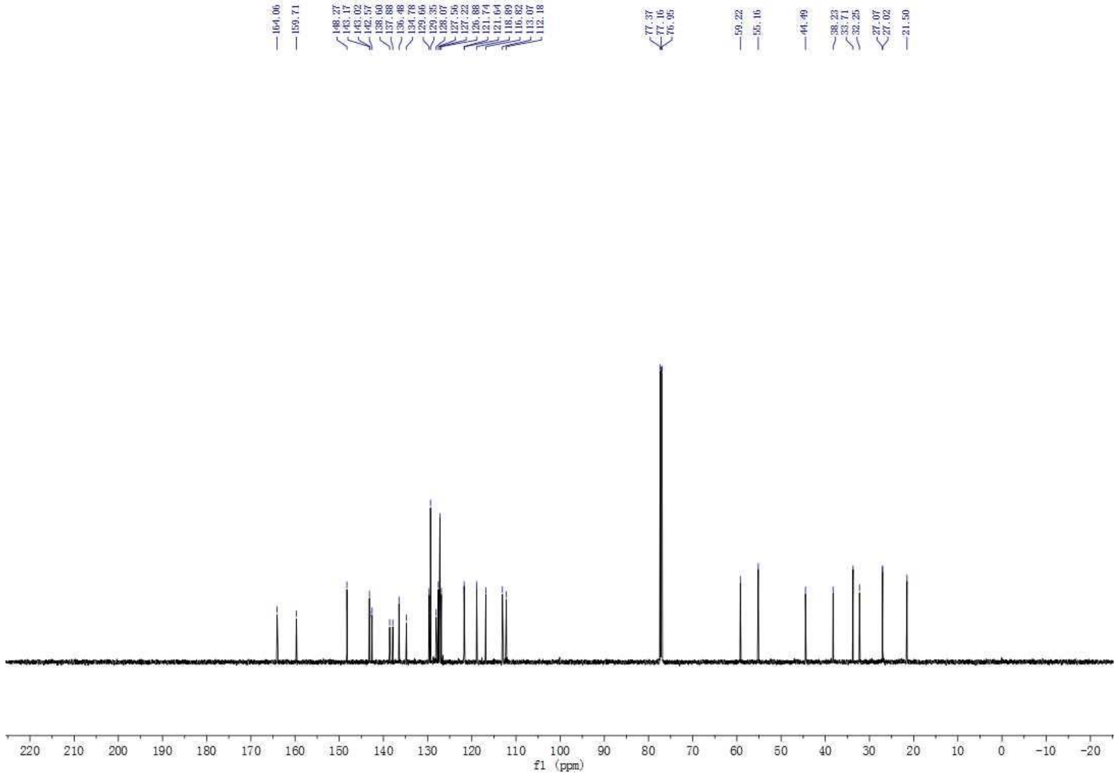

Supplementary Fig. 255. ^{13}C NMR of compound 4p. The sample has been recorded in 150 MHz, $CDCl_3$ at 25 °C

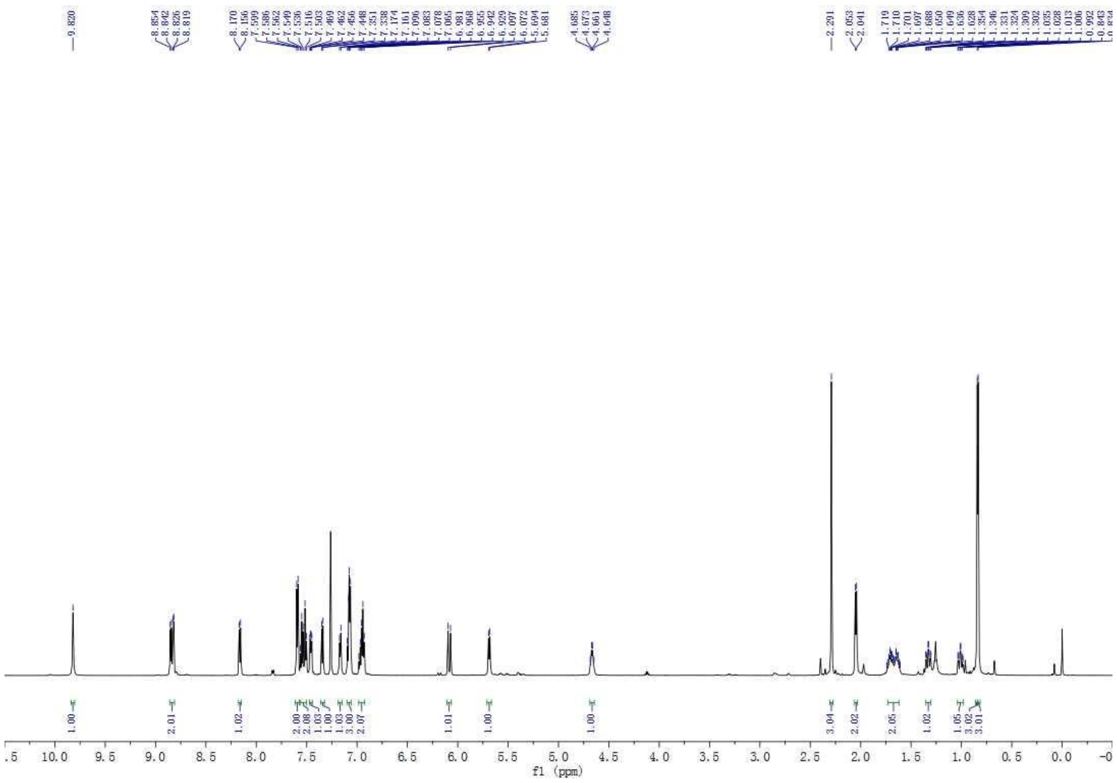

Supplementary Fig. 256. ¹H NMR of compound 4q. The sample has been recorded in 600 MHz, $CDCl_3$ at 25 °C

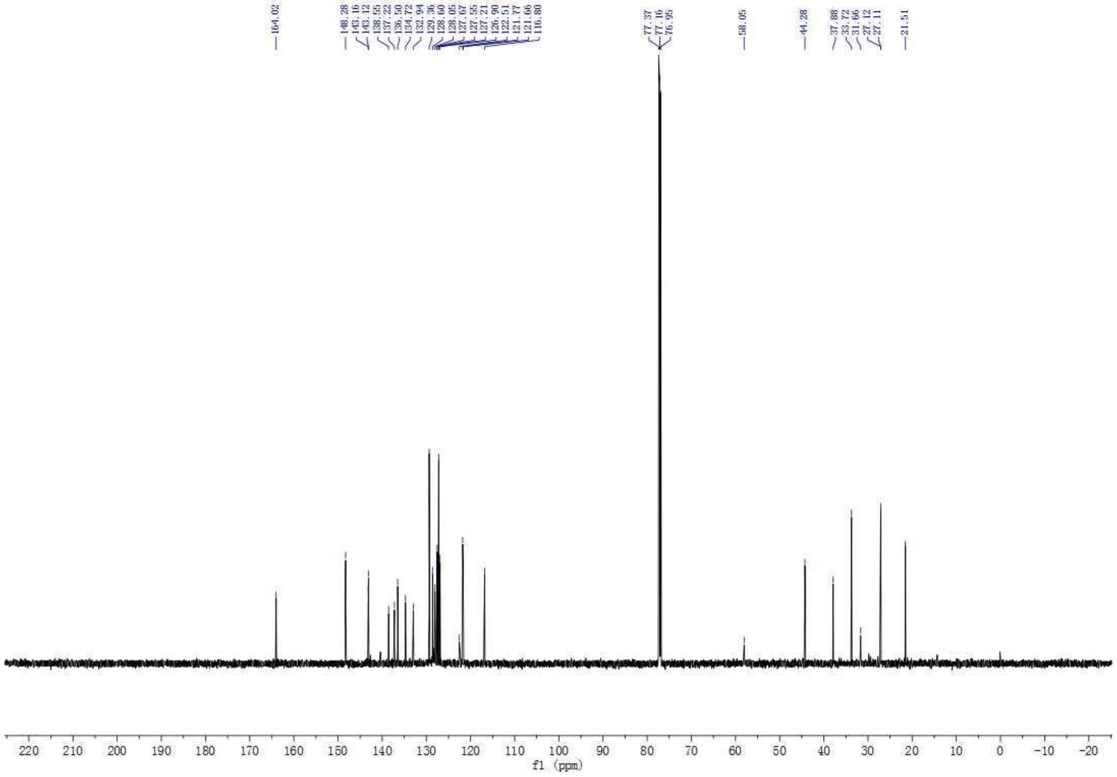

Supplementary Fig. 257. ¹³C NMR of compound 4q. The sample has been recorded in 150 MHz, $CDCl_3$ at 25 °C

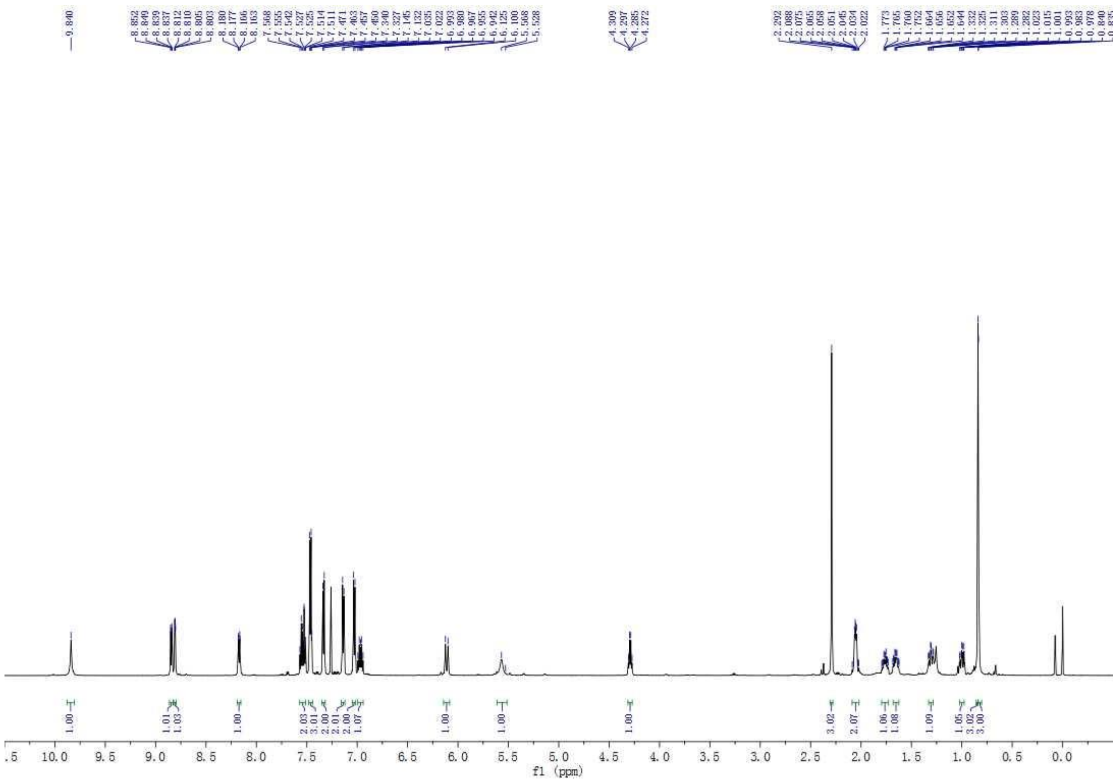

Supplementary Fig. 258. ¹H NMR of compound 4r. The sample has been recorded in 600 MHz, $CDCl_3$ at 25 °C

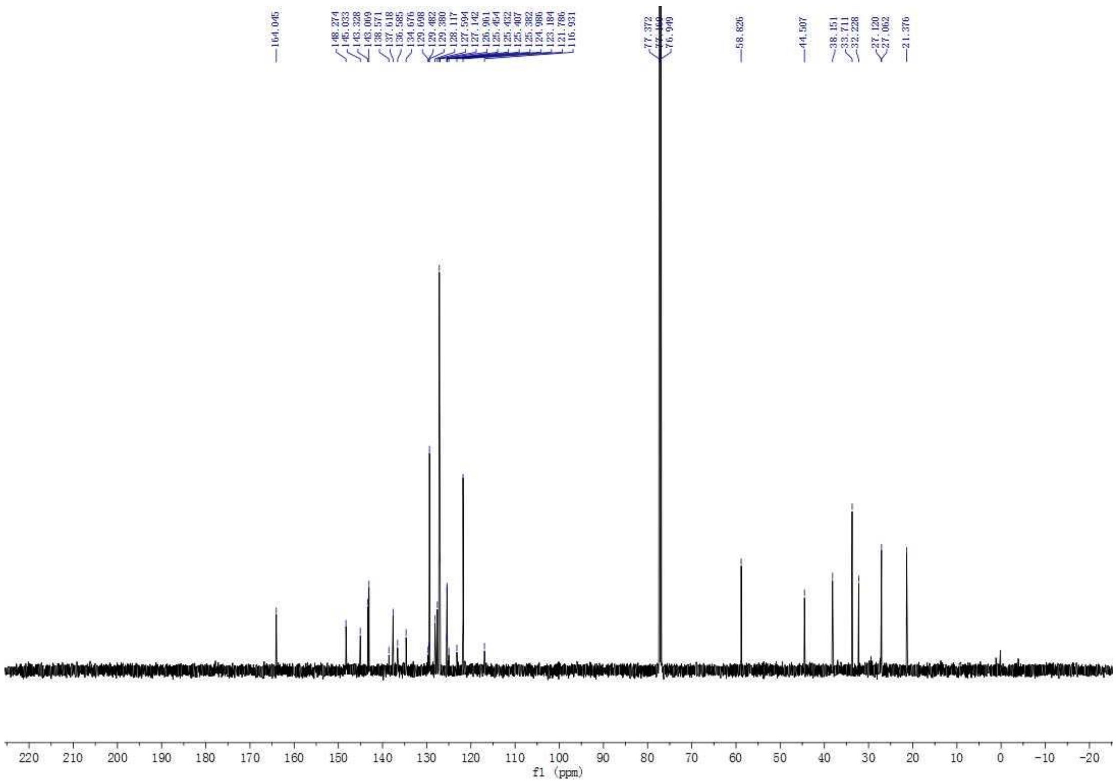

Supplementary Fig. 259. ¹³C NMR of compound 4r. The sample has been recorded in 150 MHz, $CDCl_3$ at 25 °C

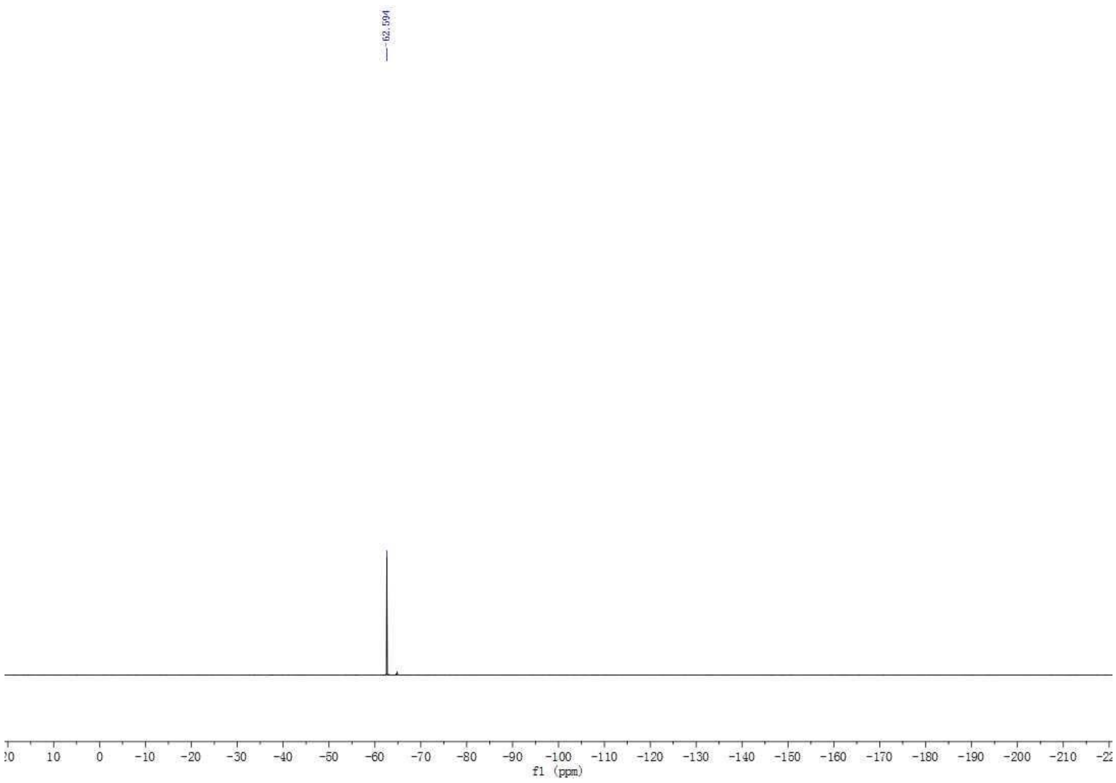

Supplementary Fig. 260. ^{19}F NMR of compound 4r. The sample has been recorded in 470 MHz, $CDCl_3$ at 25 °C

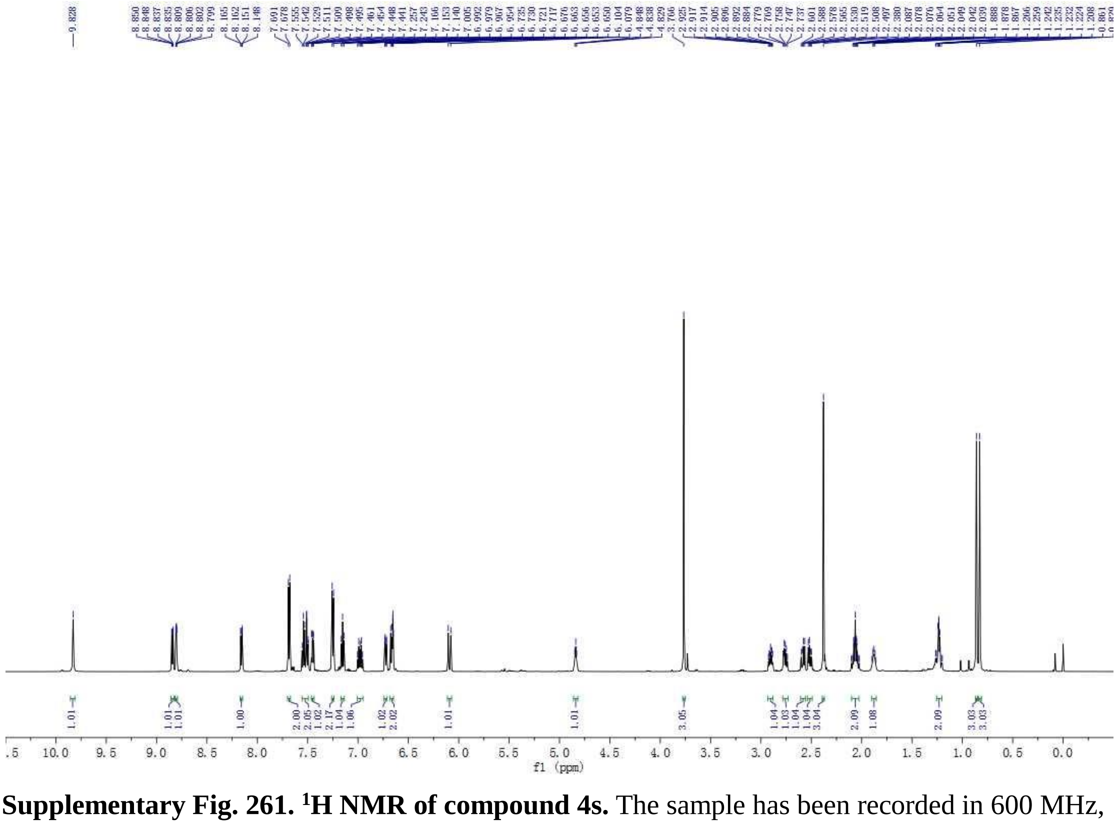

Supplementary Fig. 261. ^{1}H NMR of compound 4s. The sample has been recorded in 600 MHz, $CDCl_3$ at 25 °C

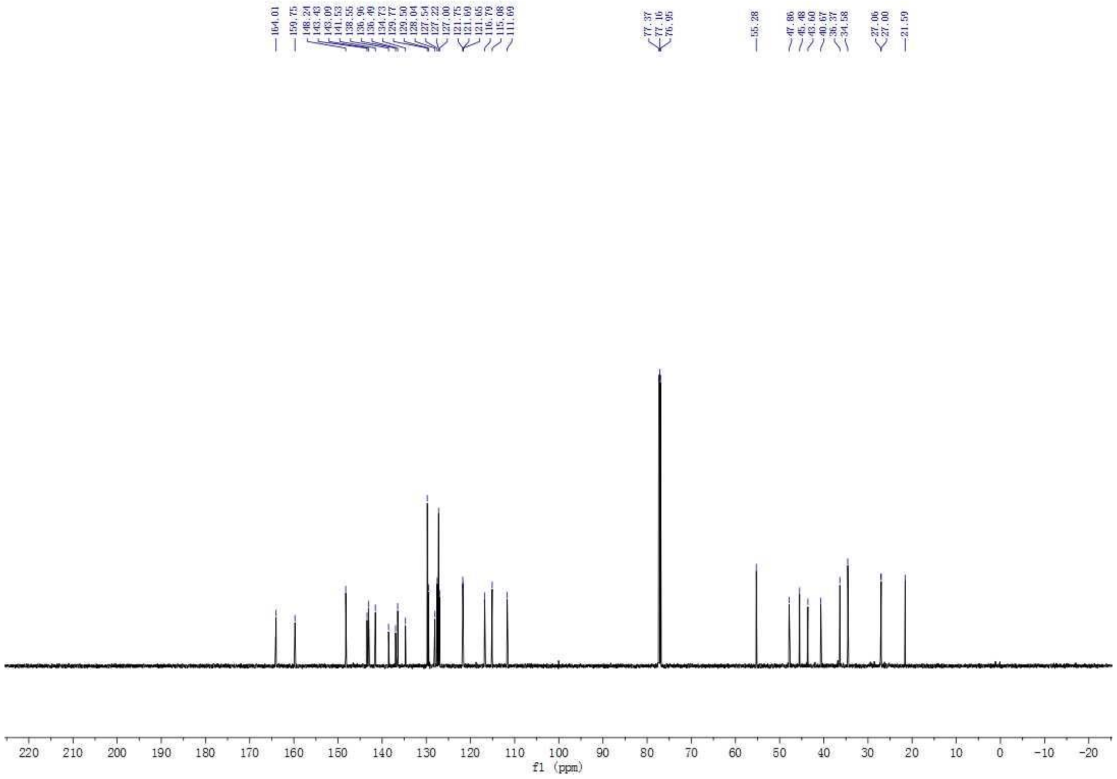

Supplementary Fig. 262. ^{13}C NMR of compound 4s. The sample has been recorded in 150 MHz, $CDCl_3$ at 25 °C

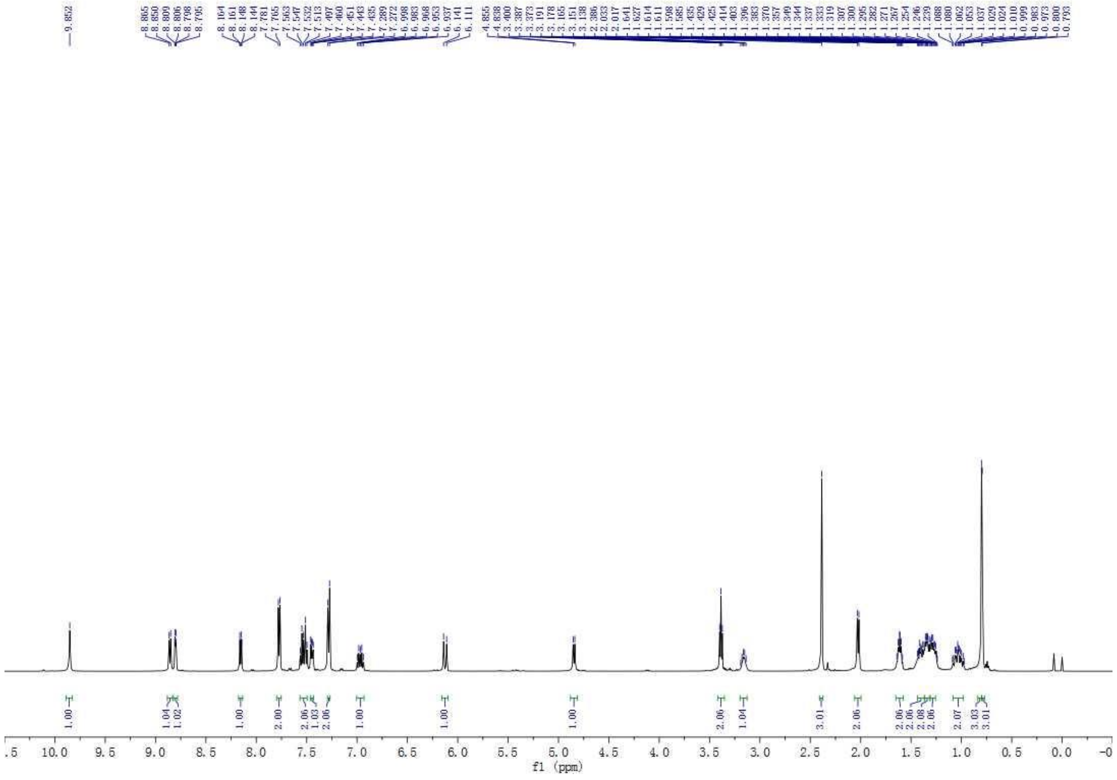

Supplementary Fig. 263. ^{1}H NMR of compound 4t. The sample has been recorded in 500 MHz, $CDCl_3$ at 25 °C

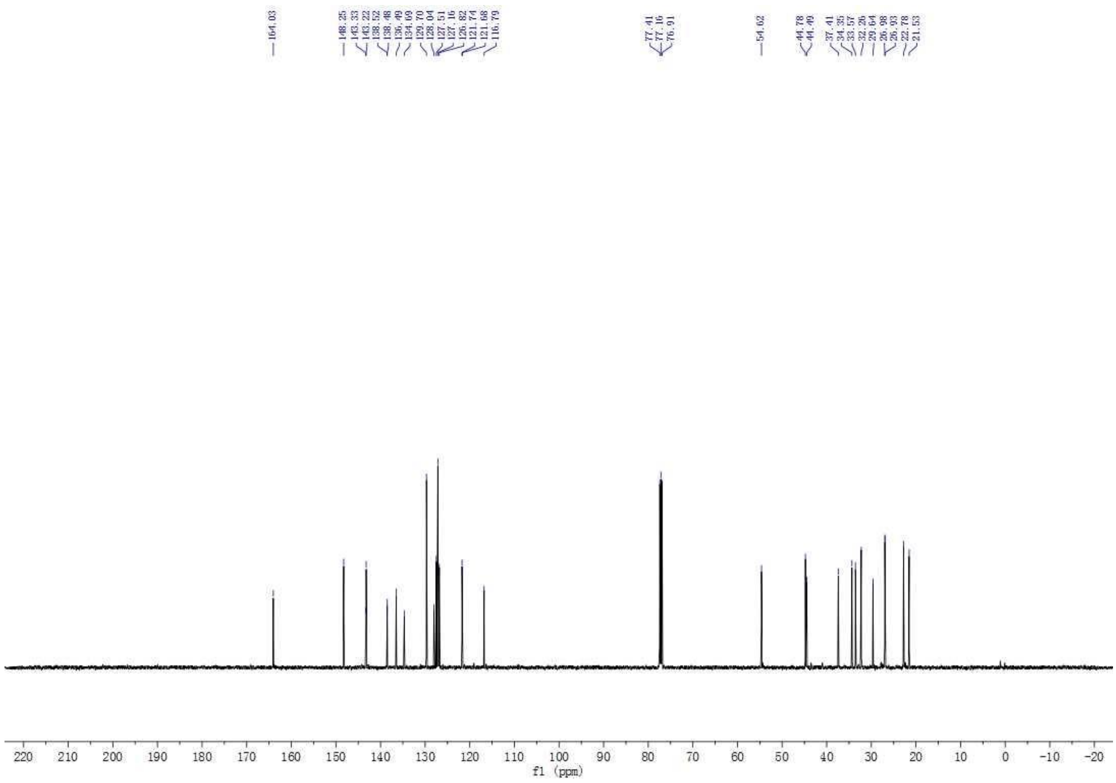

Supplementary Fig. 264. ^{13}C NMR of compound 4t. The sample has been recorded in 125 MHz, $CDCl_3$ at 25 °C

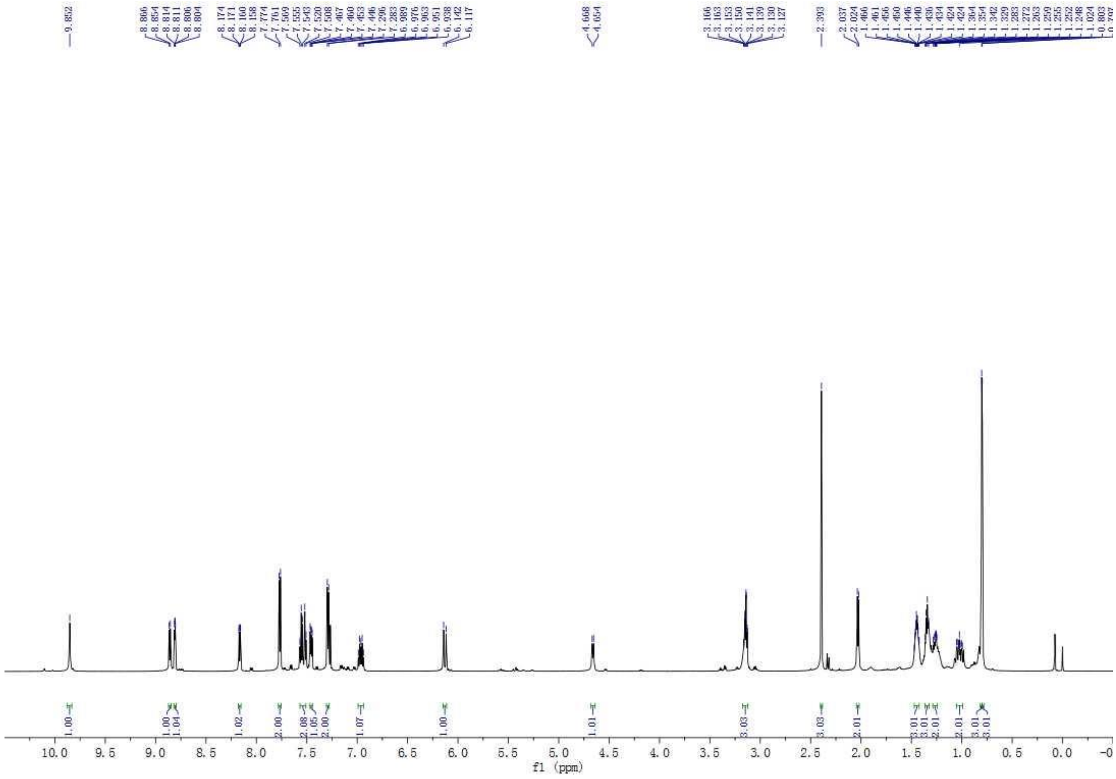

Supplementary Fig. 265. ^{1}H NMR of compound 4u. The sample has been recorded in 600 MHz, $CDCl_3$ at 25 °C

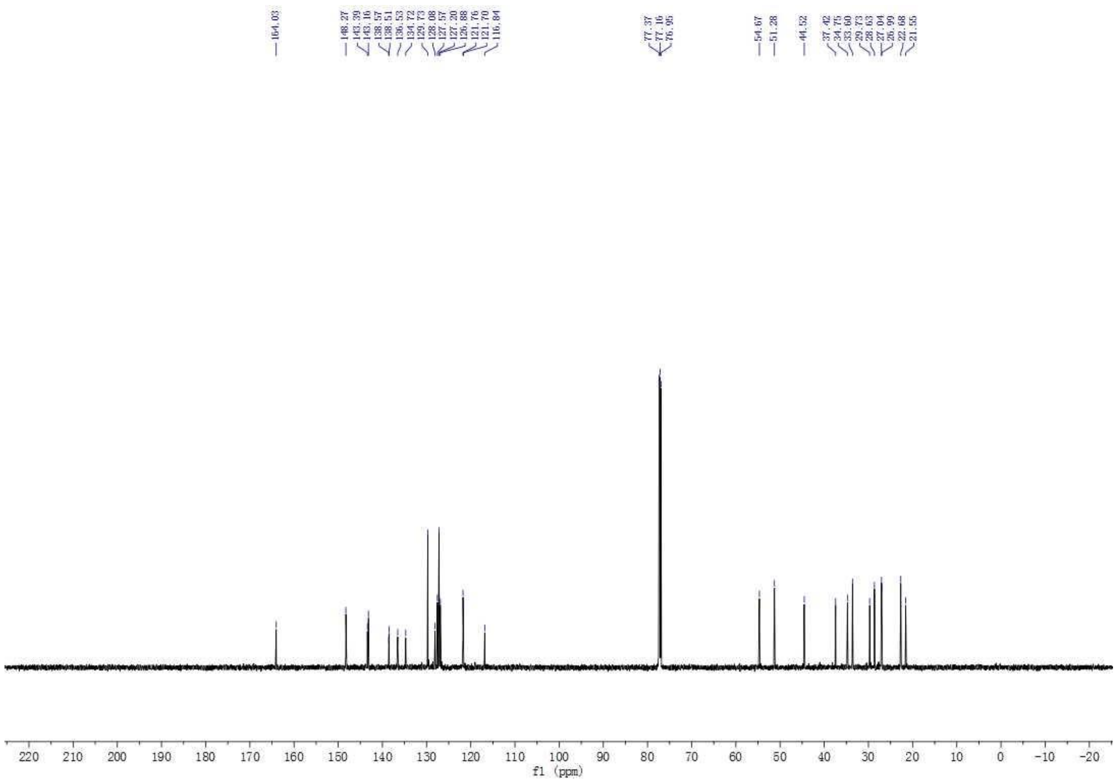

Supplementary Fig. 266. ^{13}C NMR of compound 4u. The sample has been recorded in 150 MHz, $CDCl_3$ at 25 °C

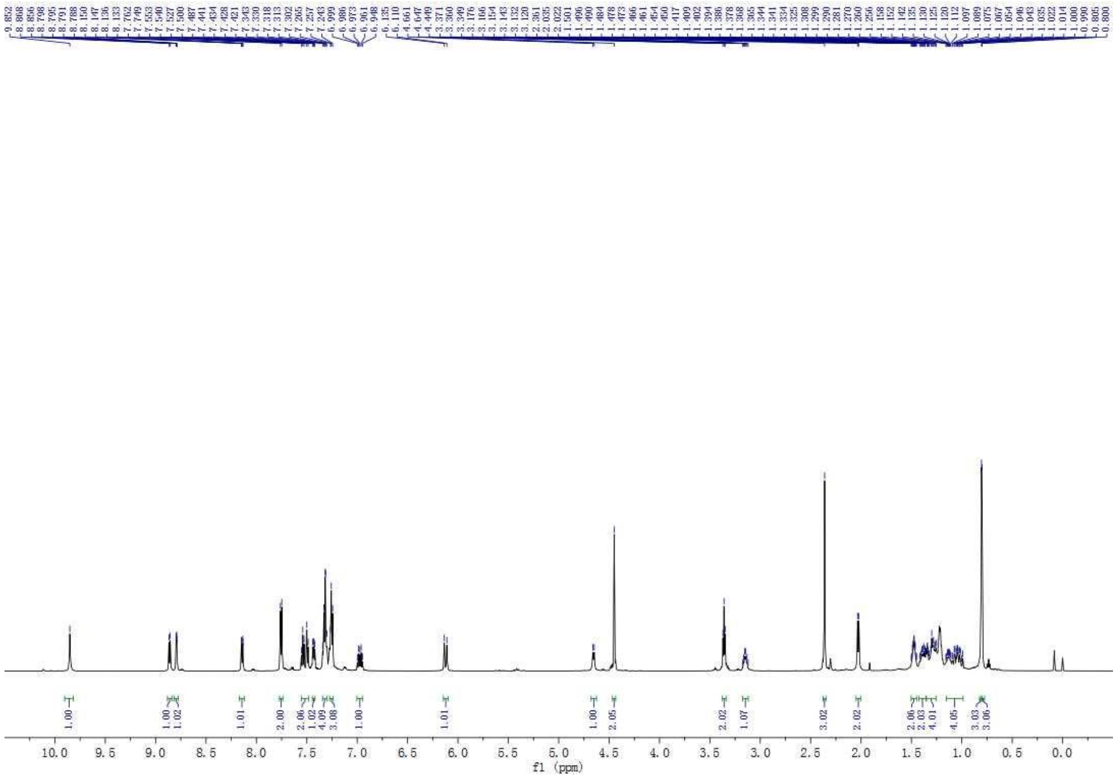

Supplementary Fig. 267. ^{1}H NMR of compound 4v. The sample has been recorded in 600 MHz, $CDCl_3$ at 25 °C

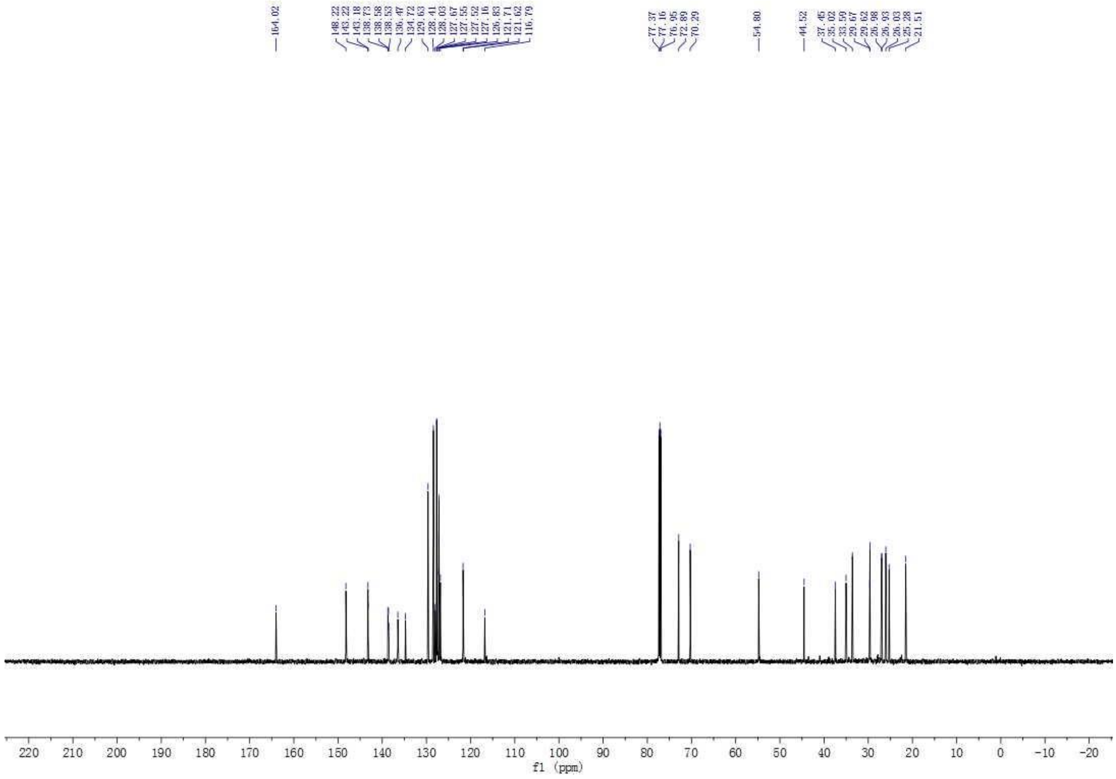

Supplementary Fig. 268. ^{13}C NMR of compound 4v. The sample has been recorded in 150 MHz, $CDCl_3$ at 25 °C

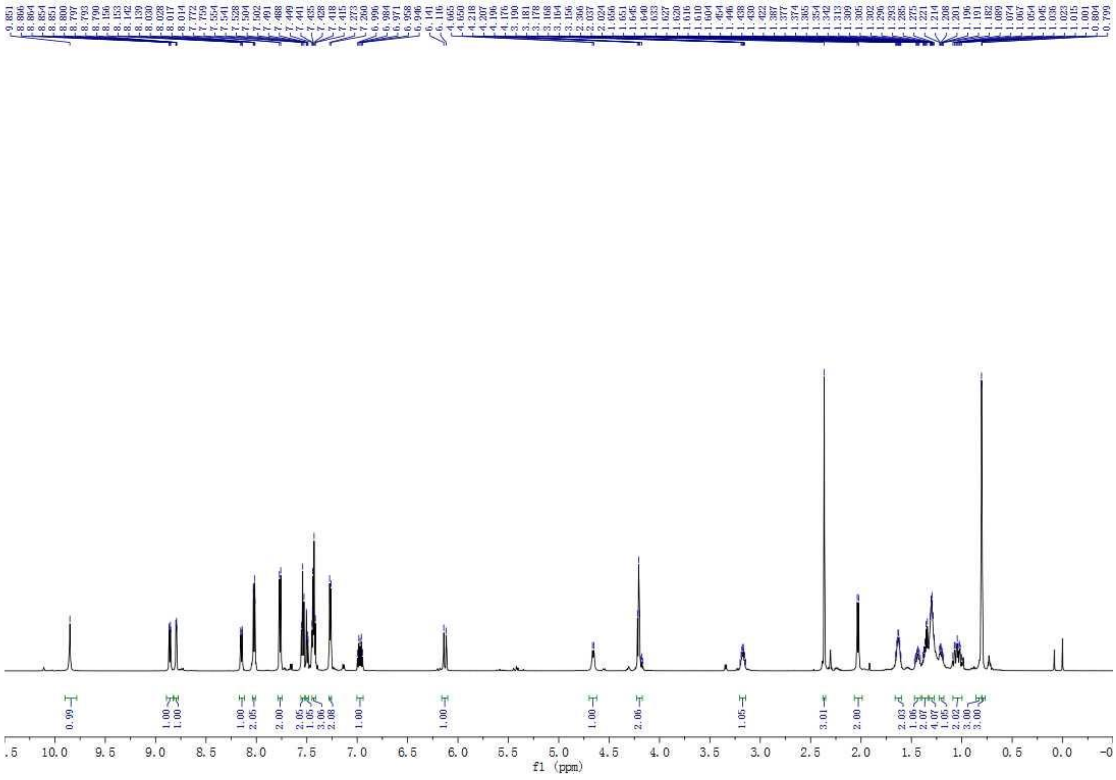

Supplementary Fig. 269. ^{1}H NMR of compound 4w. The sample has been recorded in 600 MHz, $CDCl_3$ at 25 °C

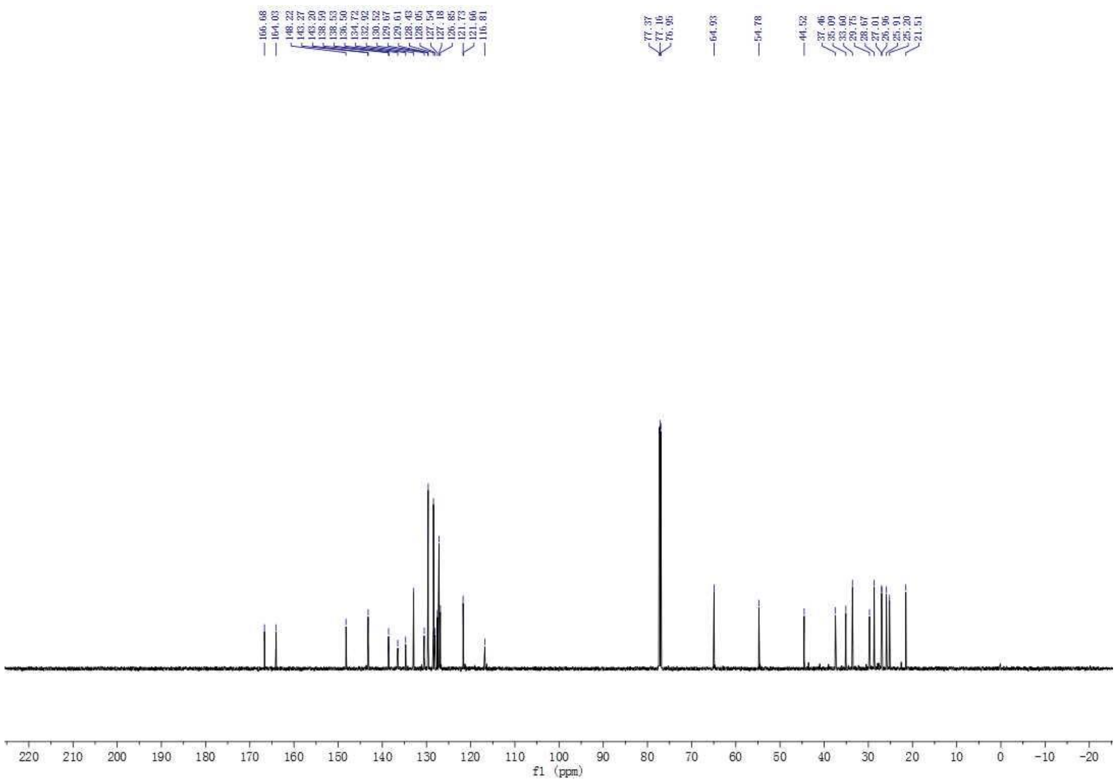

Supplementary Fig. 270. ^{13}C NMR of compound 4w. The sample has been recorded in 150 MHz, $CDCl_3$ at 25 °C

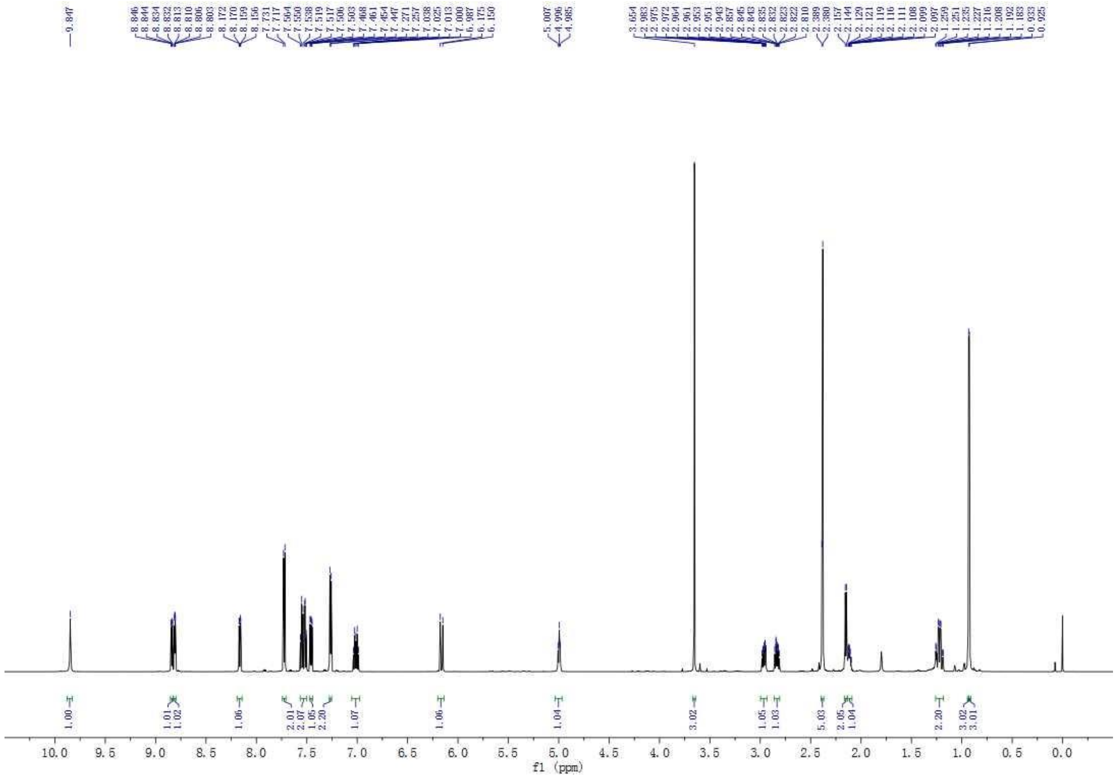

Supplementary Fig. 271. ^{1}H NMR of compound 4x. The sample has been recorded in 600 MHz, $CDCl_3$ at 25 °C

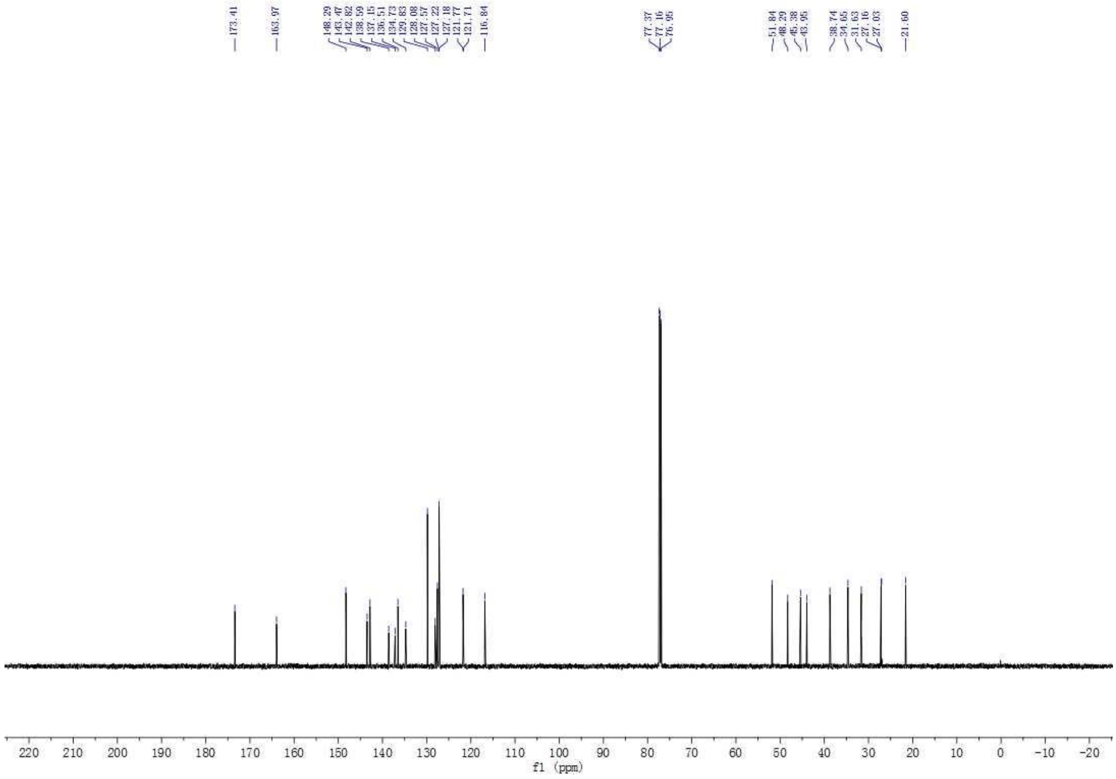

Supplementary Fig. 272. ^{13}C NMR of compound 4x. The sample has been recorded in 150 MHz, $CDCl_3$ at 25 °C

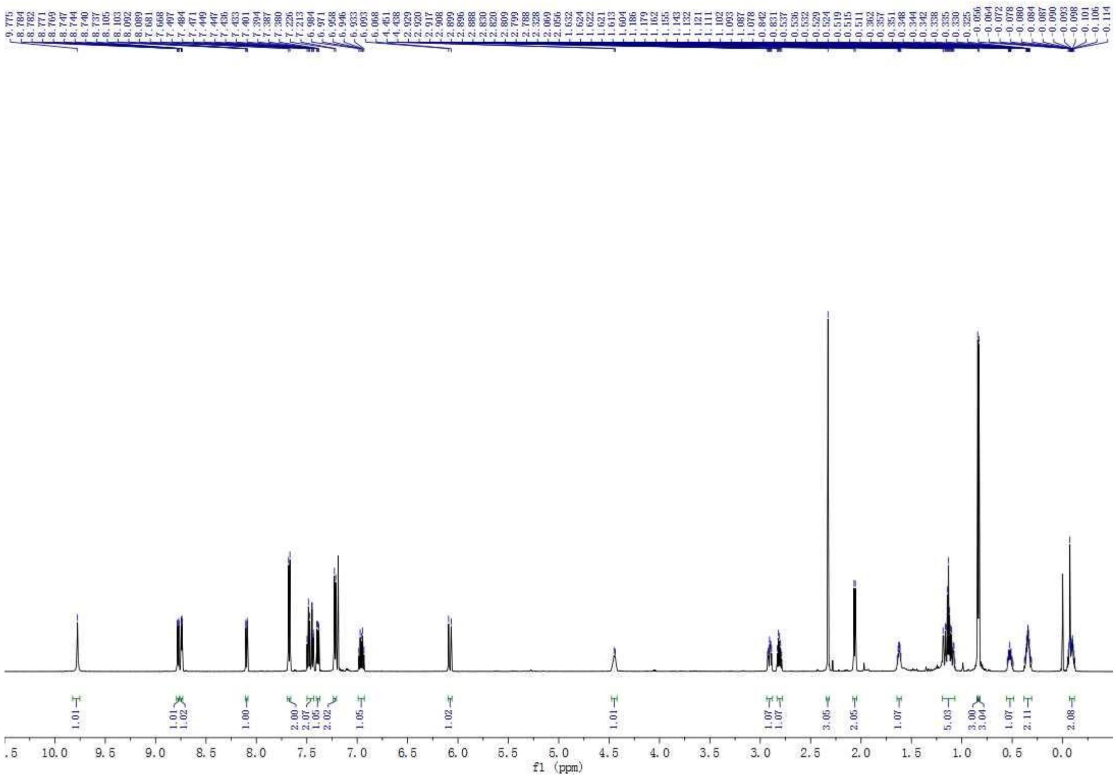

Supplementary Fig. 273. ^{1}H NMR of compound 4y. The sample has been recorded in 600 MHz, $CDCl_3$ at 25 °C

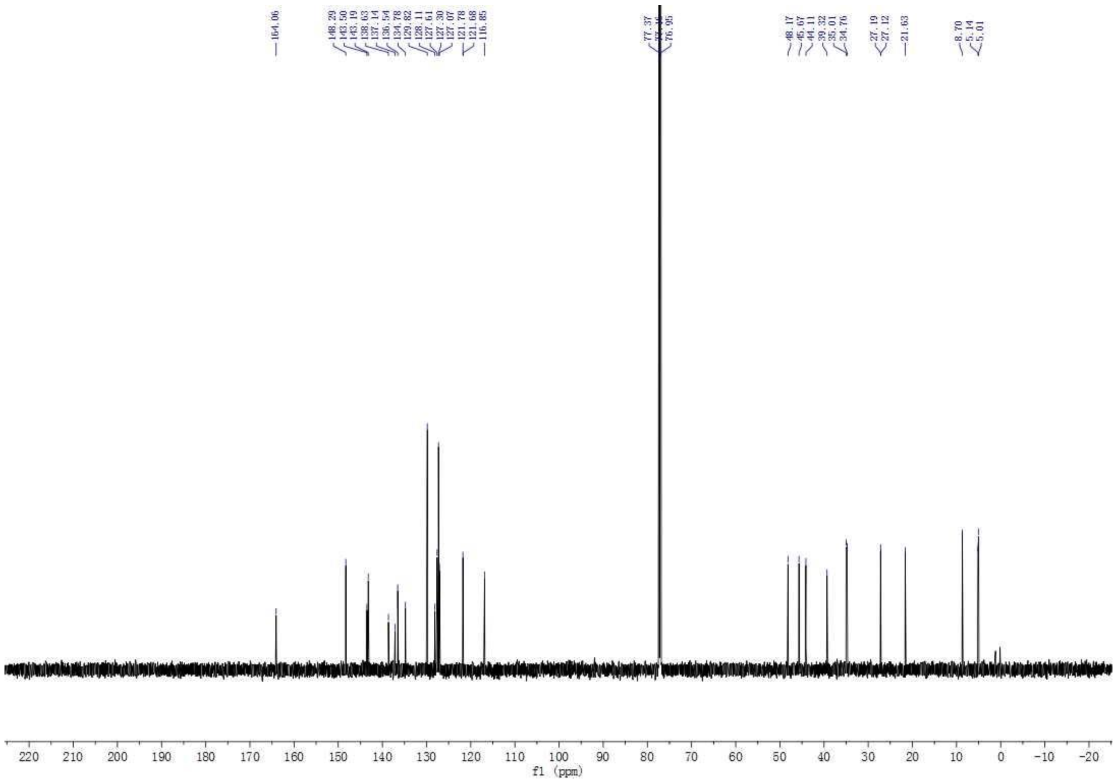

Supplementary Fig. 274. ^{13}C NMR of compound 4y. The sample has been recorded in 150 MHz, $CDCl_3$ at 25 °C

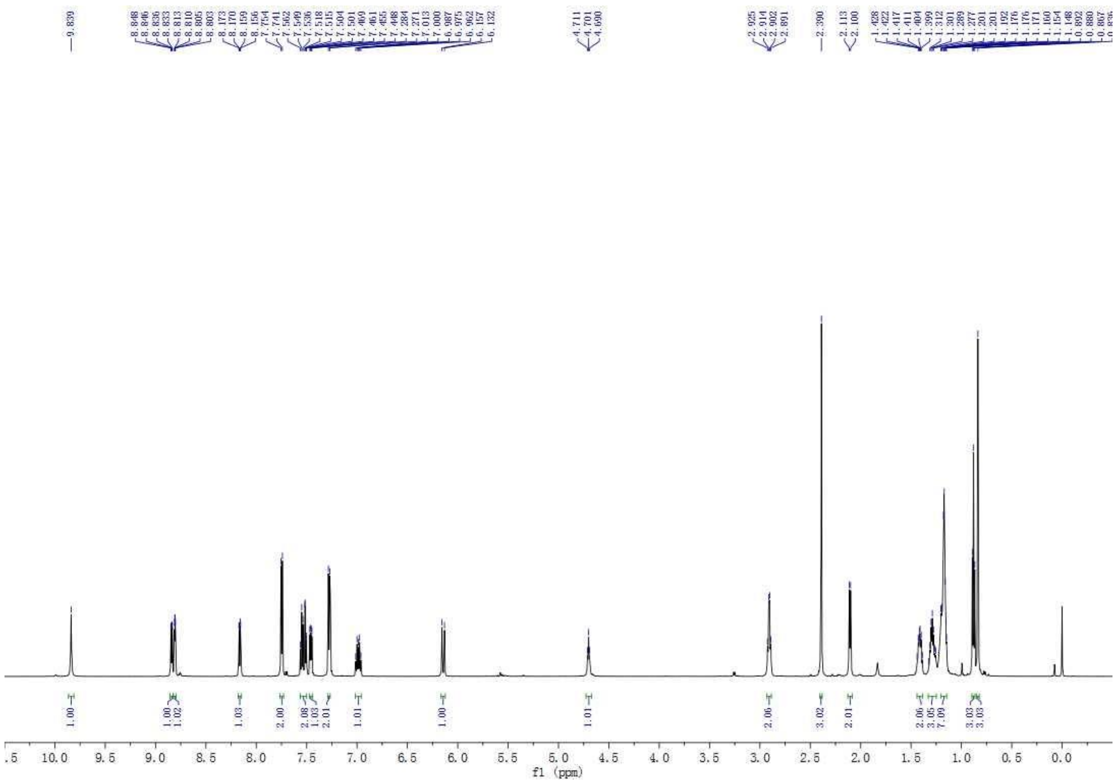

Supplementary Fig. 275. ^{1}H NMR of compound 4z. The sample has been recorded in 600 MHz, $CDCl_3$ at 25 °C

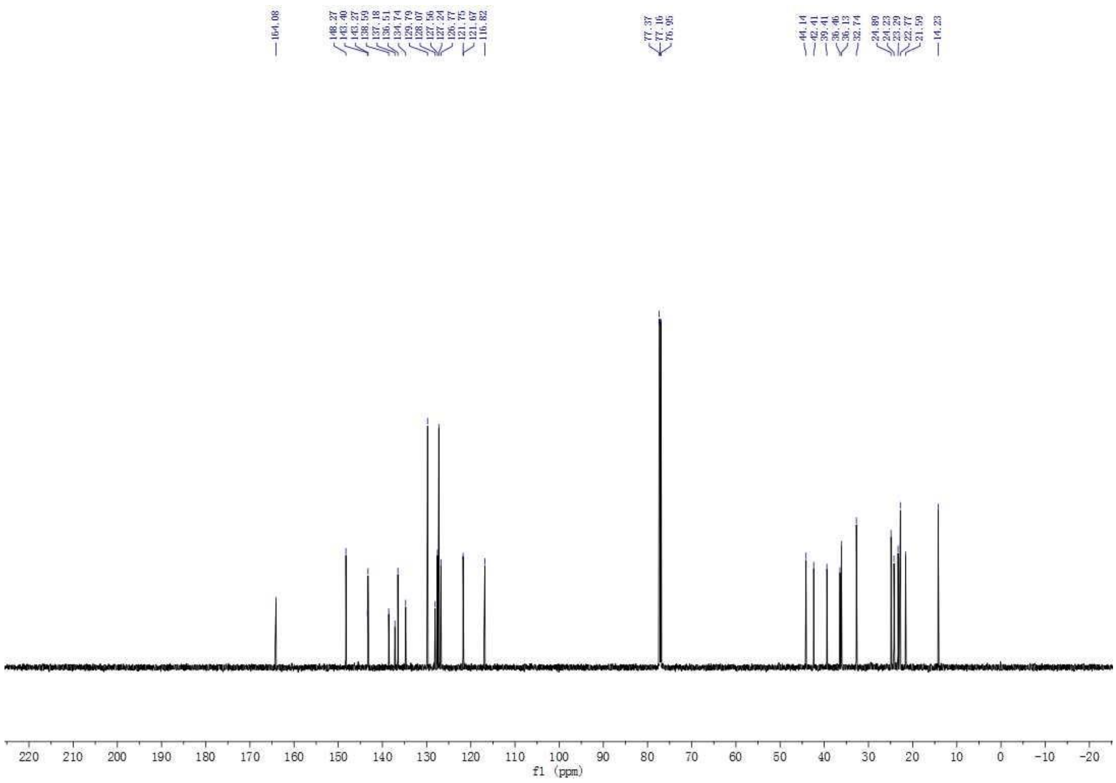

Supplementary Fig. 276. ^{13}C NMR of compound 4z. The sample has been recorded in 150 MHz, $CDCl_3$ at 25 °C

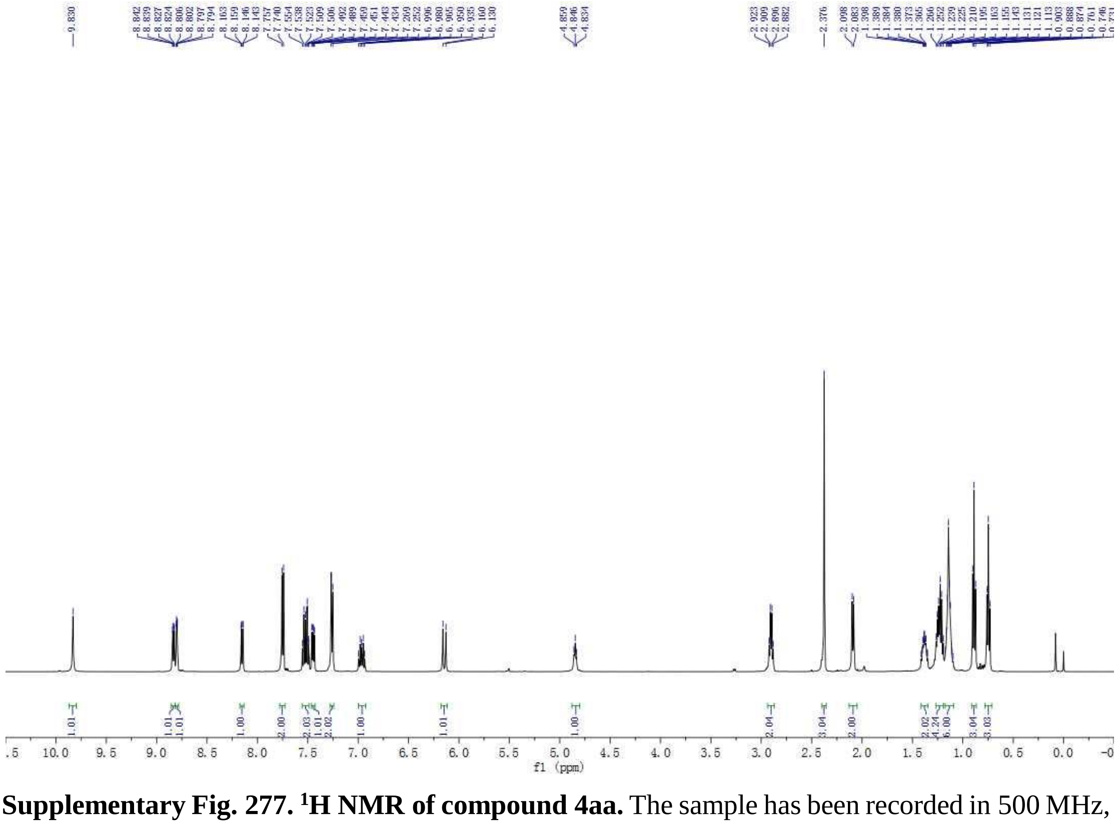

Supplementary Fig. 277. ^{1}H NMR of compound 4aa. The sample has been recorded in 500 MHz, $CDCl_3$ at 25 °C

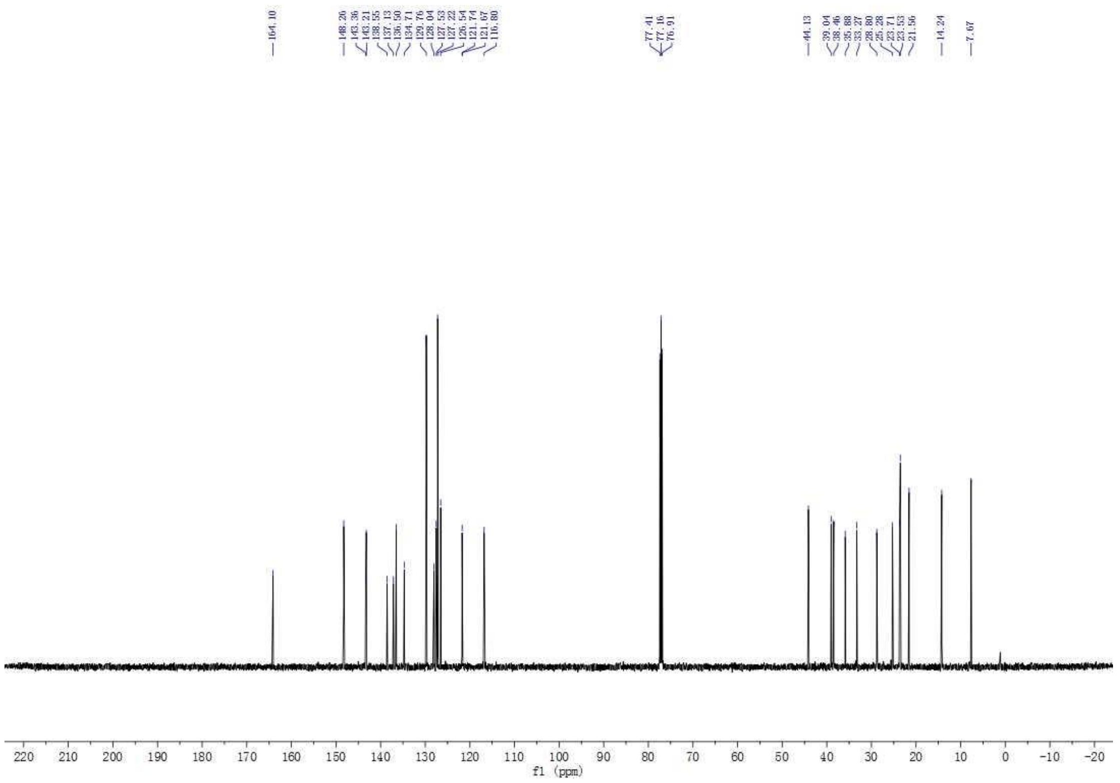

Supplementary Fig. 278. ^{13}C NMR of compound 4aa. The sample has been recorded in 125 MHz, $CDCl_3$ at 25 °C

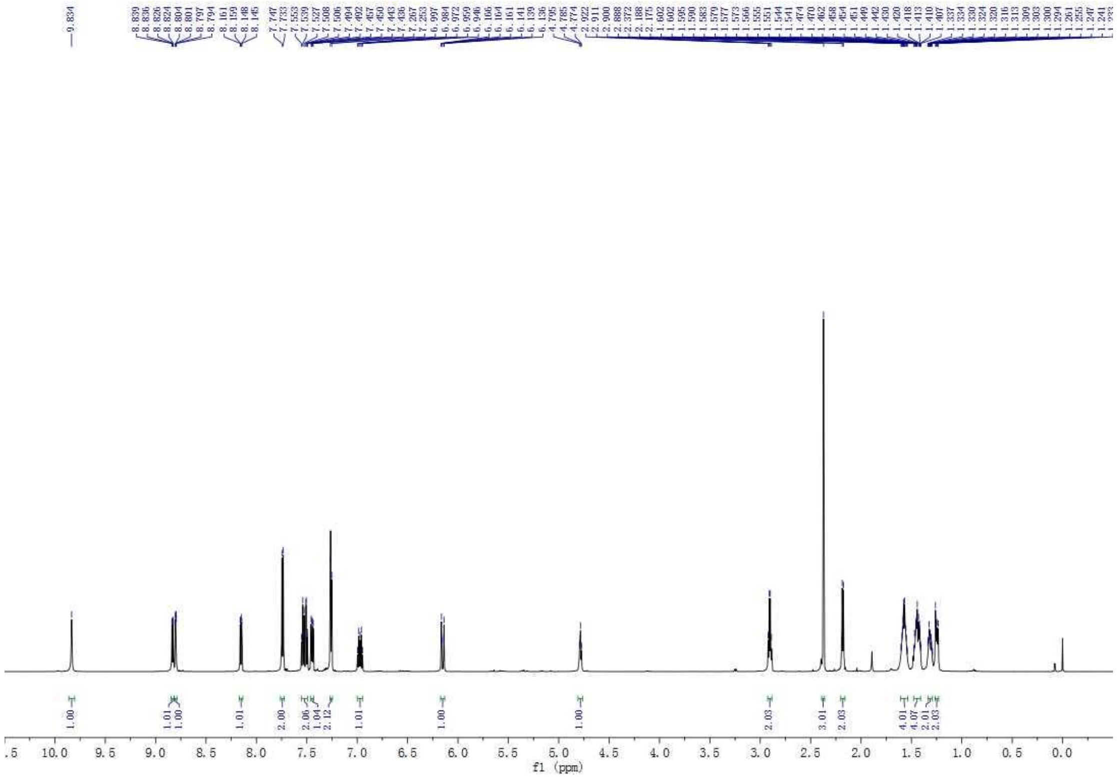

Supplementary Fig. 279. ^{1}H NMR of compound 4ab. The sample has been recorded in 600 MHz, $CDCl_3$ at 25 °C

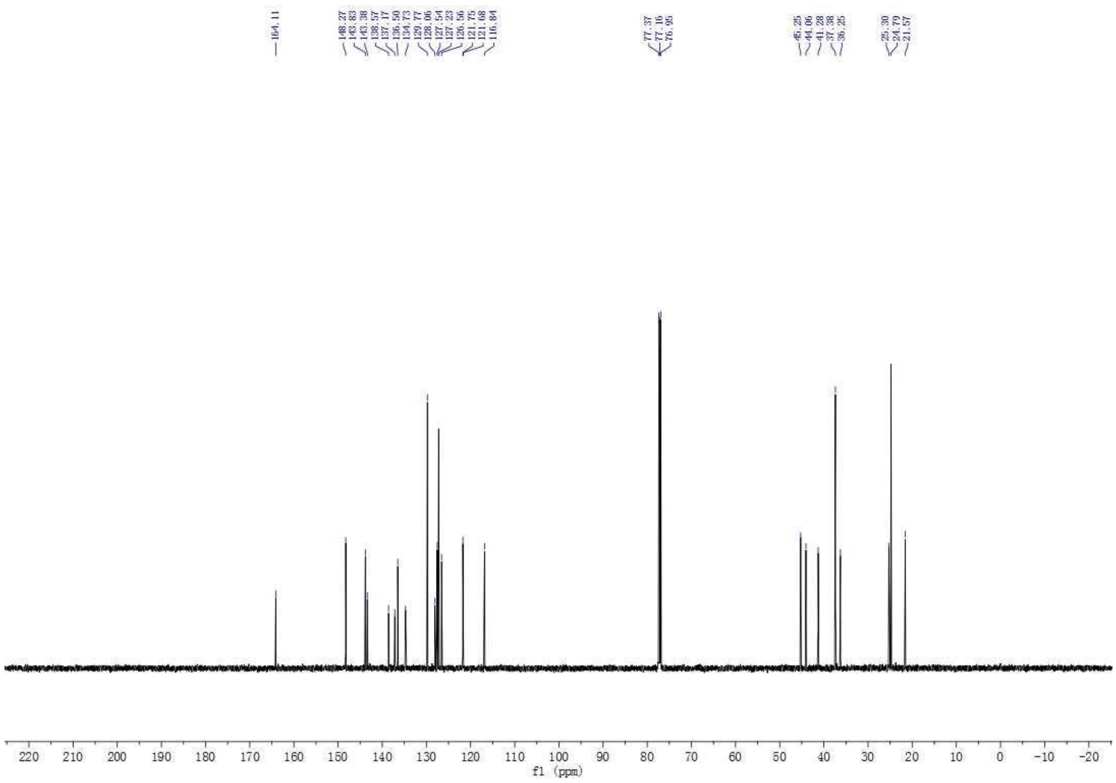

Supplementary Fig. 280. ^{13}C NMR of compound 4ab. The sample has been recorded in 150 MHz, $CDCl_3$ at 25 °C

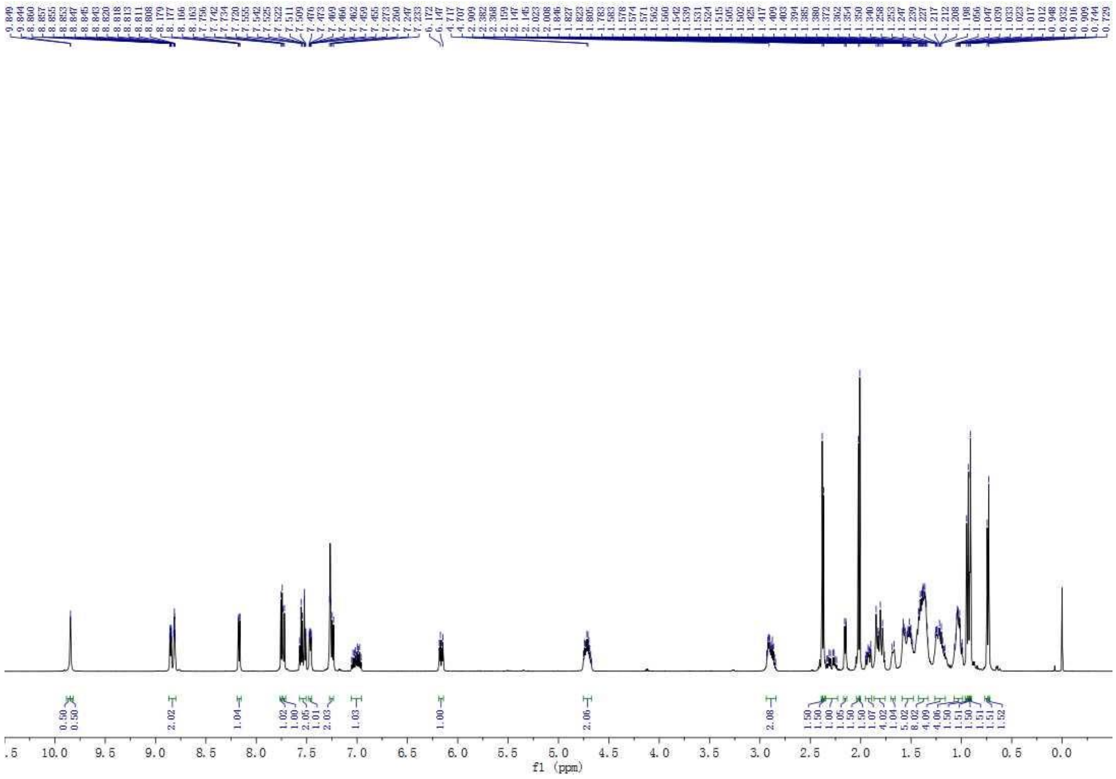

Supplementary Fig. 281. ^{1}H NMR of compound 4ac. The sample has been recorded in 600 MHz, $CDCl_3$ at 25 °C

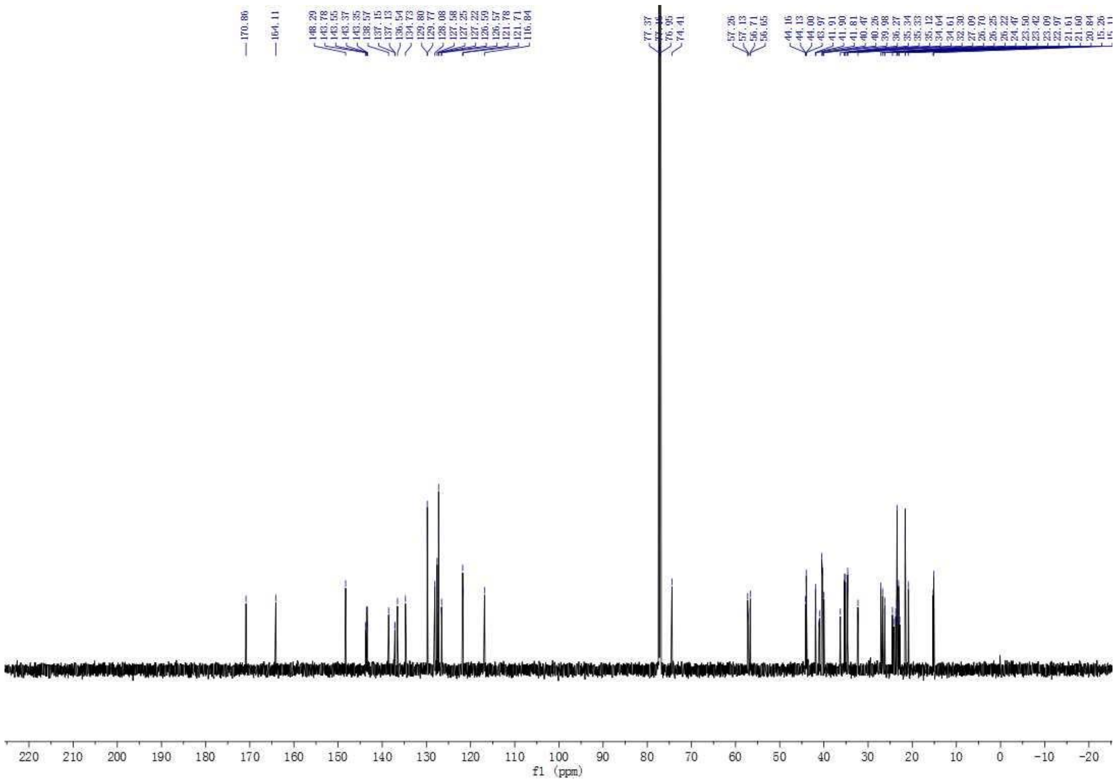

Supplementary Fig. 282. ^{13}C NMR of compound 4ac. The sample has been recorded in 150 MHz, $CDCl_3$ at 25 °C

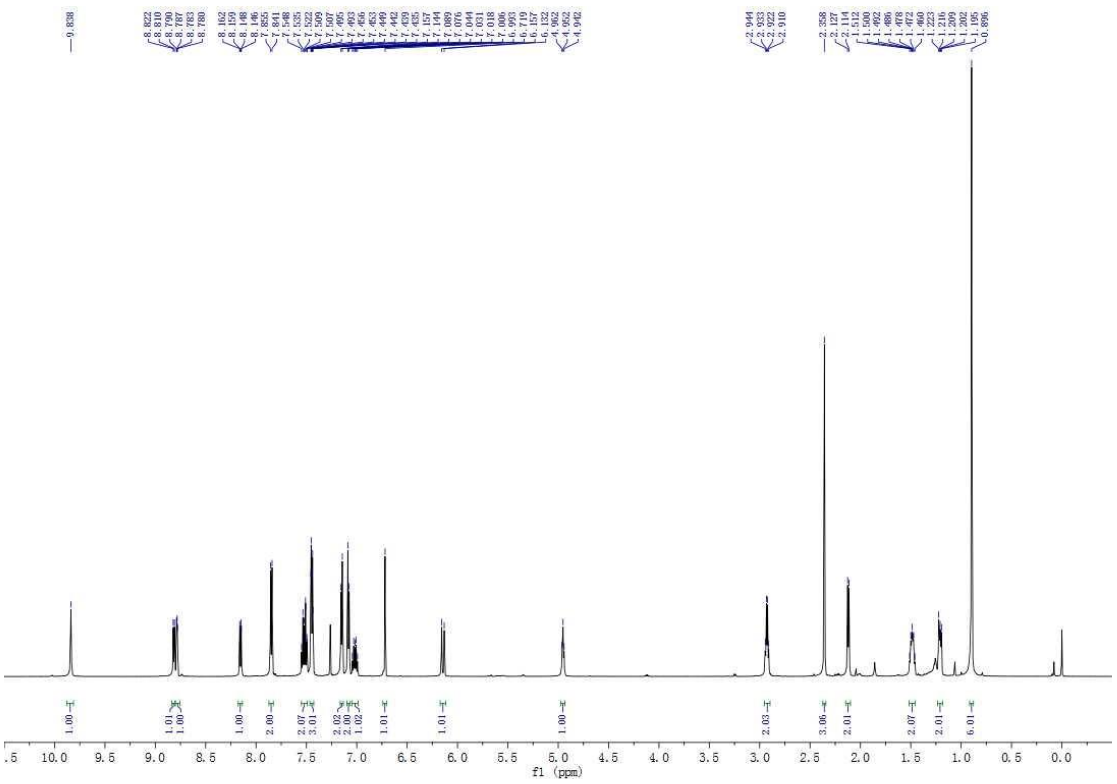

Supplementary Fig. 283. ^{1}H NMR of compound 4ad. The sample has been recorded in 600 MHz, $CDCl_3$ at 25 °C

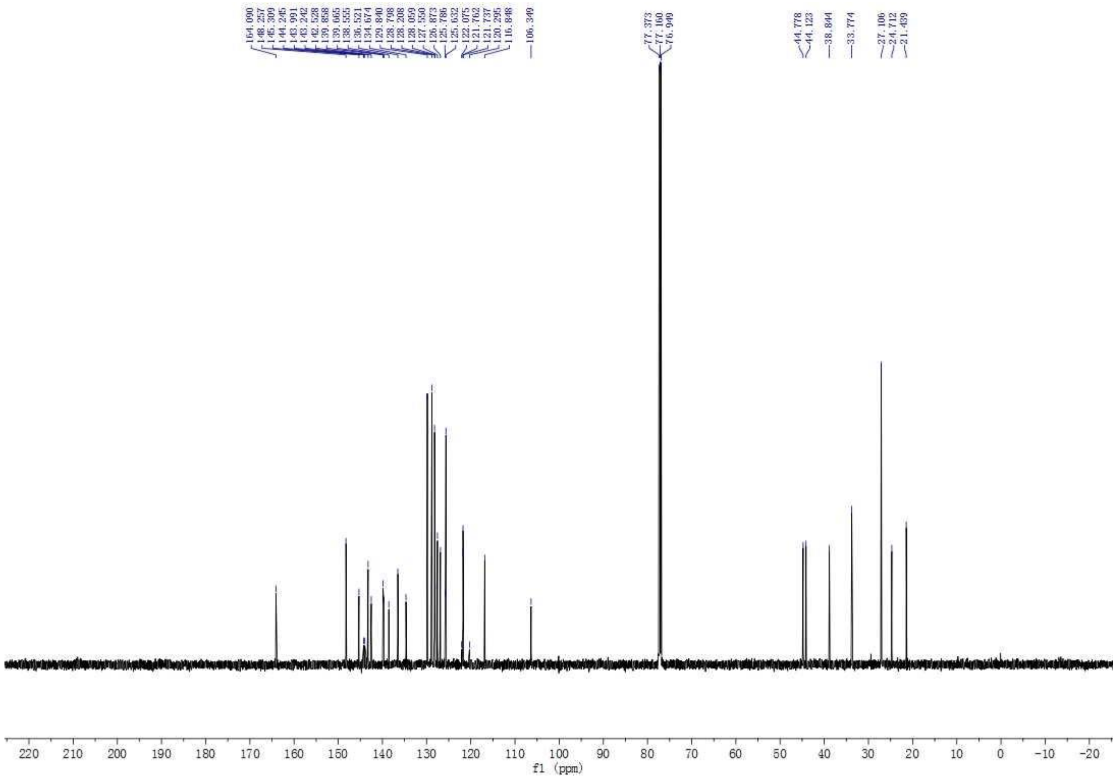

Supplementary Fig. 284. **^{13}C NMR of compound 4ad.** The sample has been recorded in 150 MHz, $CDCl_3$ at 25 °C

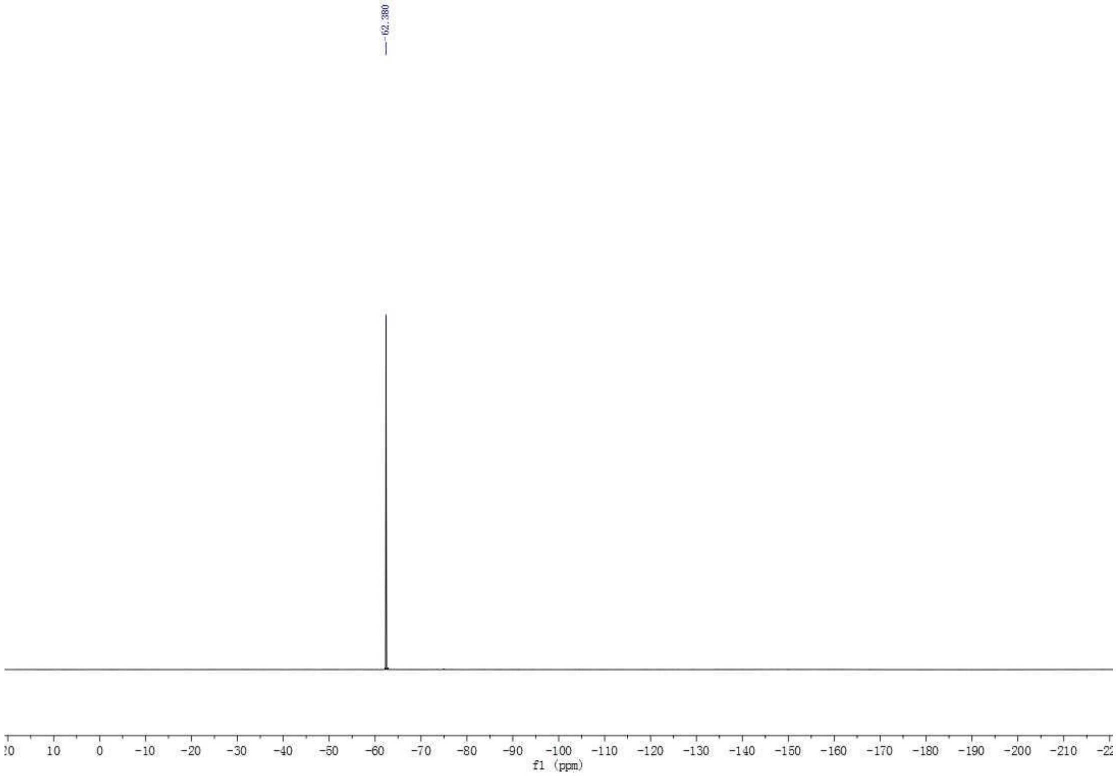

Supplementary Fig. 285. **^{19}F NMR of compound 4ad.** The sample has been recorded in 470 MHz, $CDCl_3$ at 25 °C

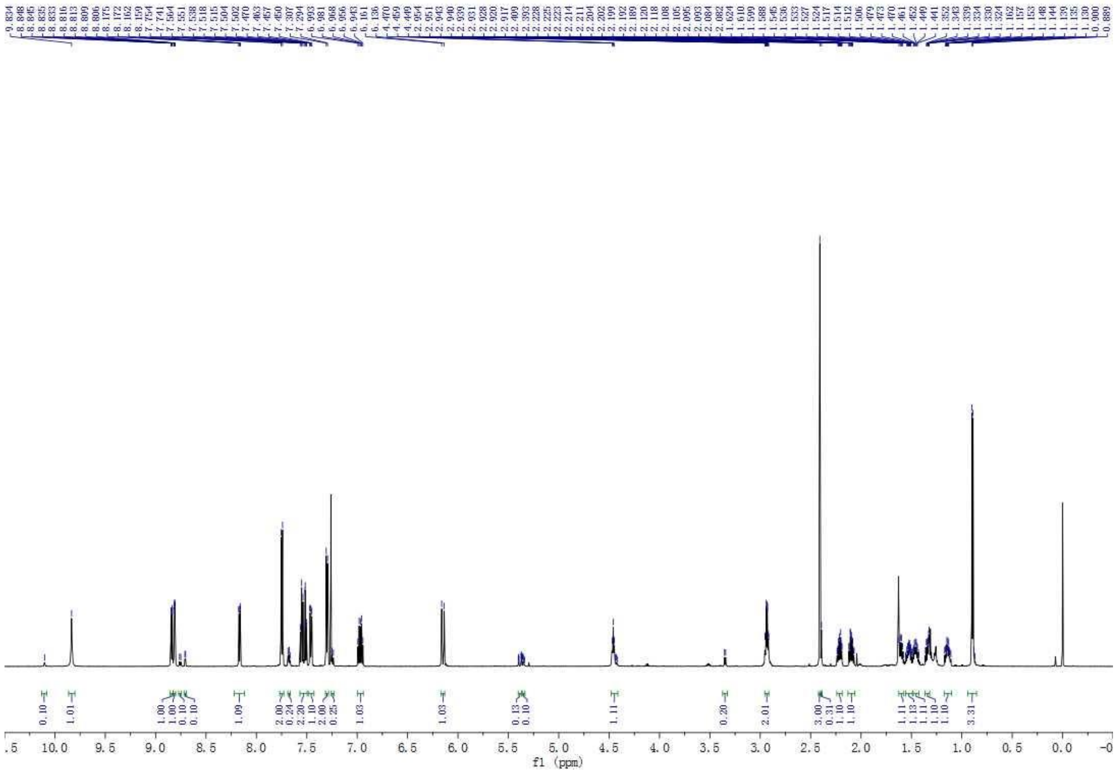

Supplementary Fig. 286. ^{1}H NMR of compound 4ae. The sample has been recorded in 600 MHz, $CDCl_3$ at 25 °C

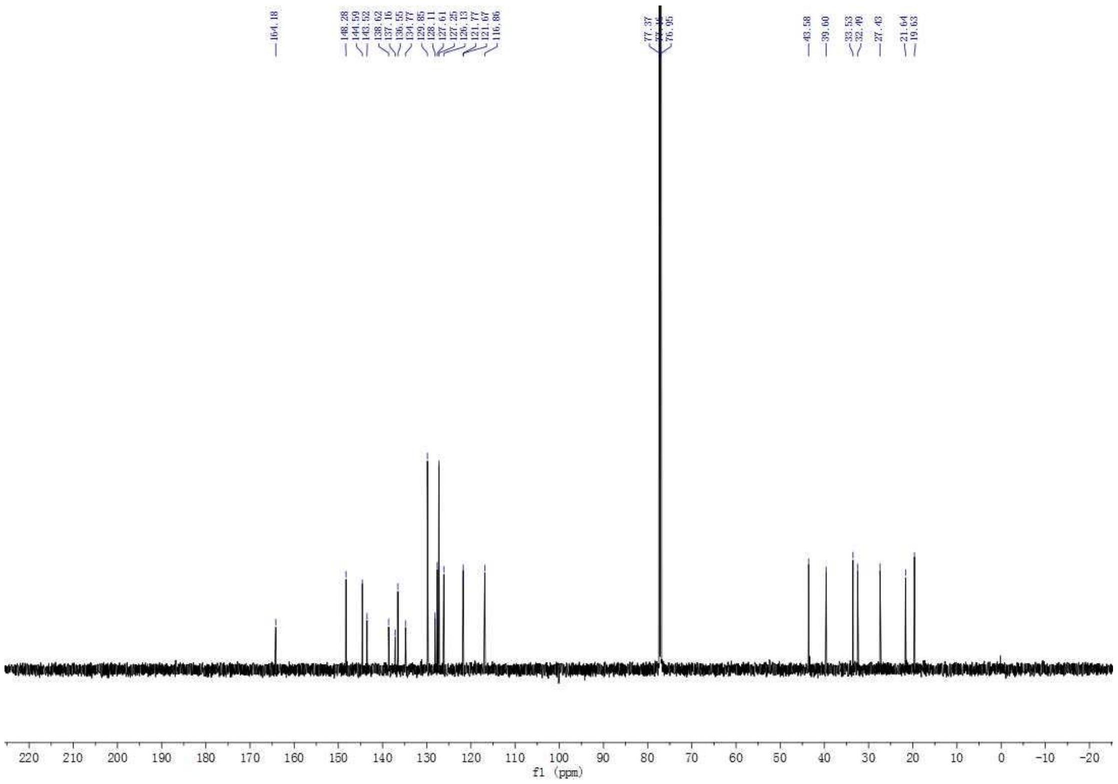

Supplementary Fig. 287. ^{13}C NMR of compound 4ae. The sample has been recorded in 150 MHz, $CDCl_3$ at 25 °C

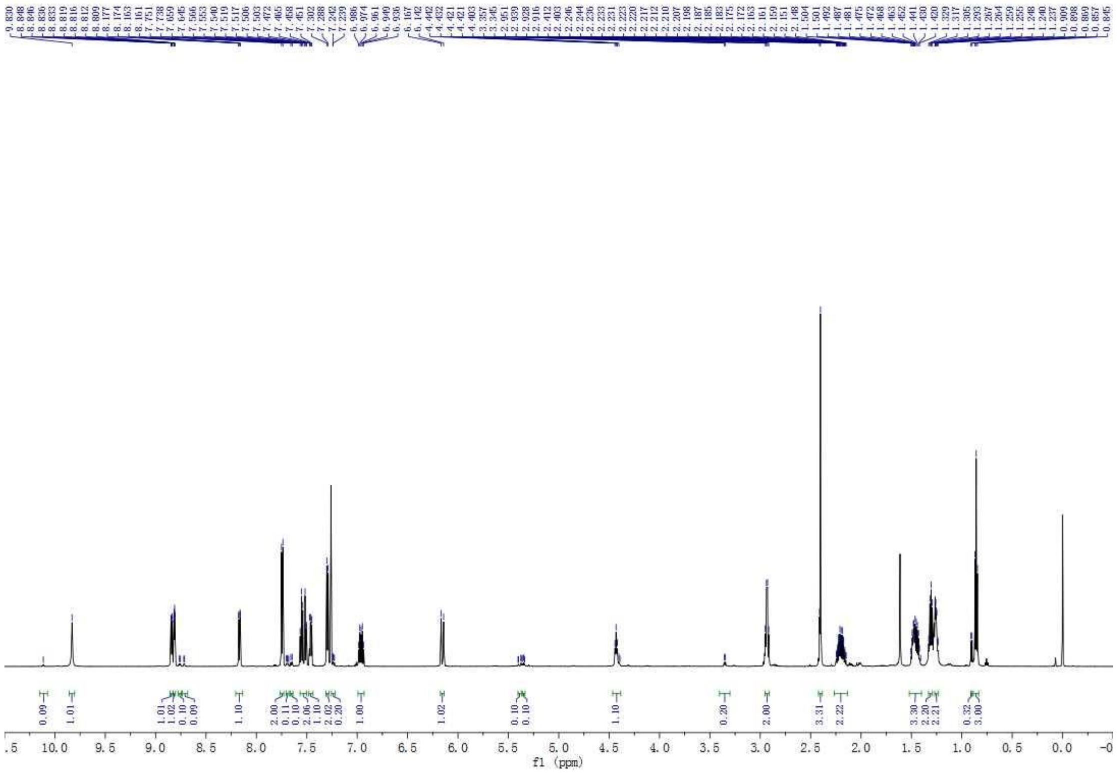

Supplementary Fig. 288. ^{1}H NMR of compound 4af. The sample has been recorded in 600 MHz, $CDCl_3$ at 25 °C

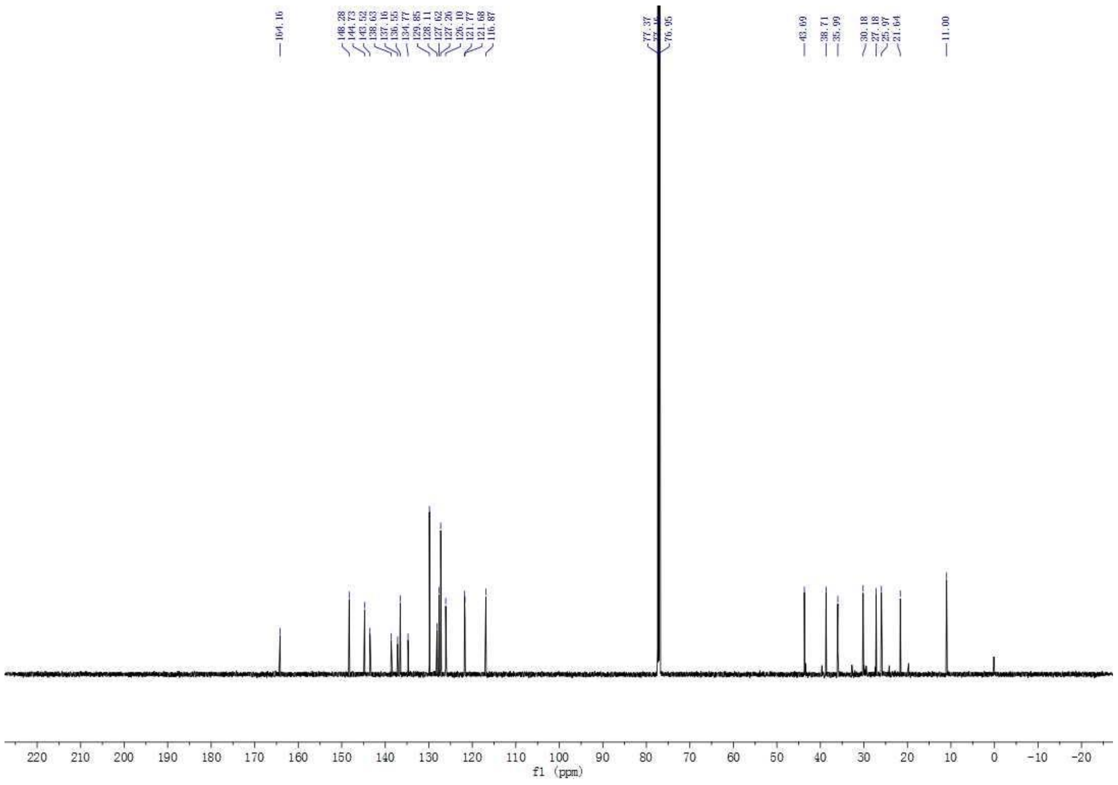

Supplementary Fig. 289. ^{13}C NMR of compound 4af. The sample has been recorded in 150 MHz, $CDCl_3$ at 25 °C

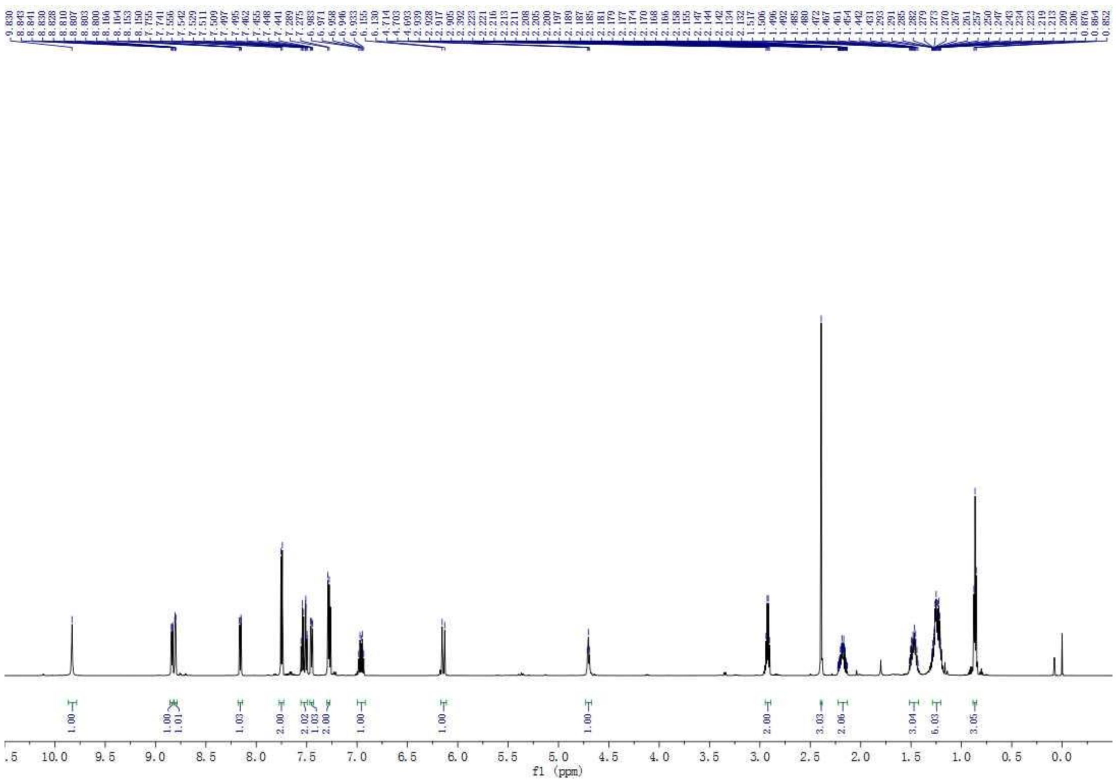

Supplementary Fig. 290. ^{1}H NMR of compound 4ag. The sample has been recorded in 600 MHz, $CDCl_3$ at 25 °C

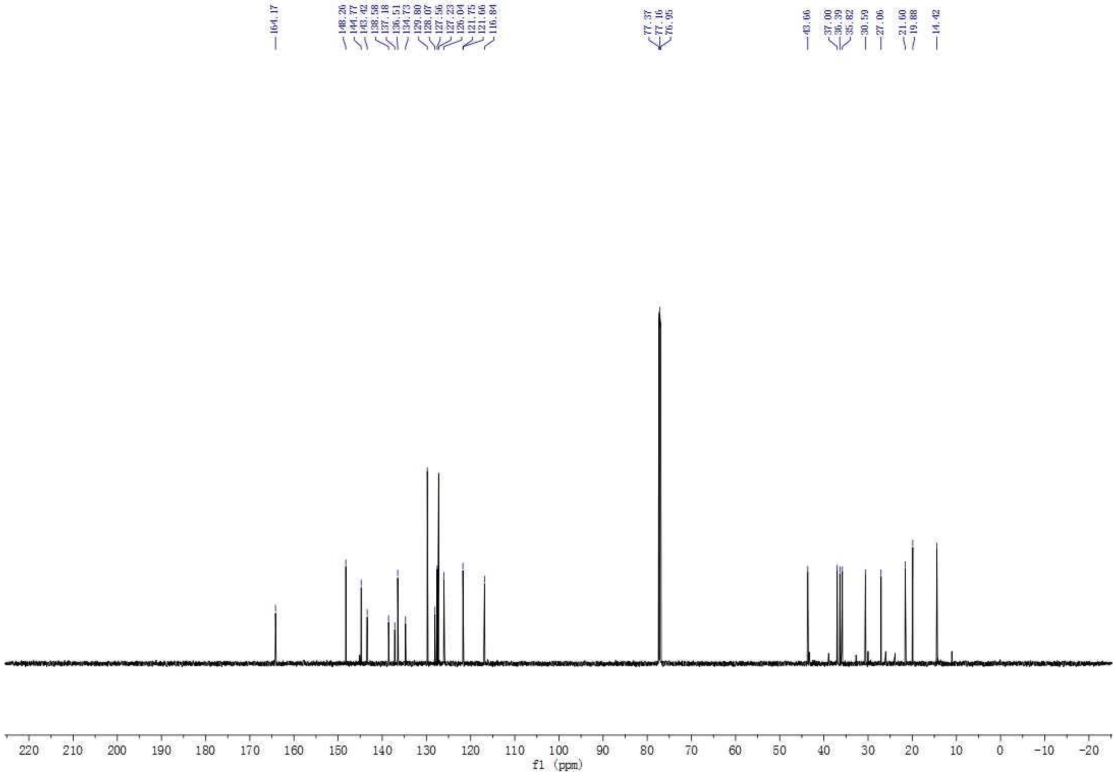

Supplementary Fig. 291. ^{13}C NMR of compound 4ag. The sample has been recorded in 150 MHz, $CDCl_3$ at 25 °C

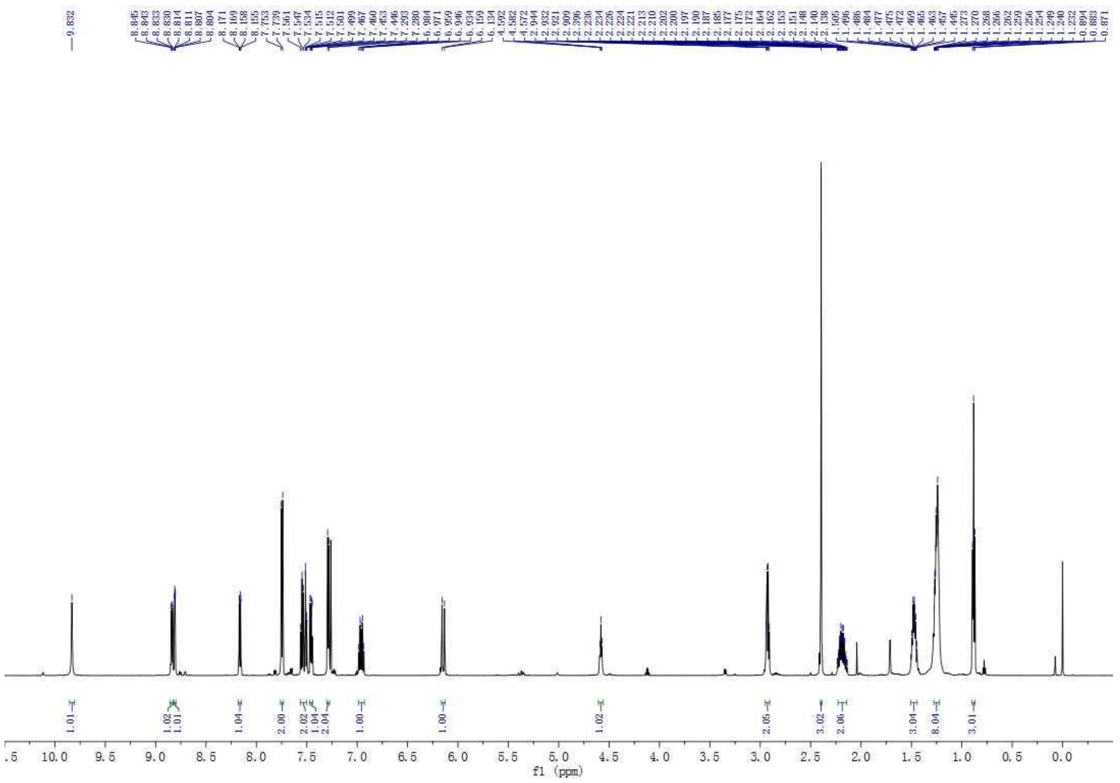

Supplementary Fig. 292. ^{1}H NMR of compound 4ah. The sample has been recorded in 600 MHz, $CDCl_3$ at 25 °C

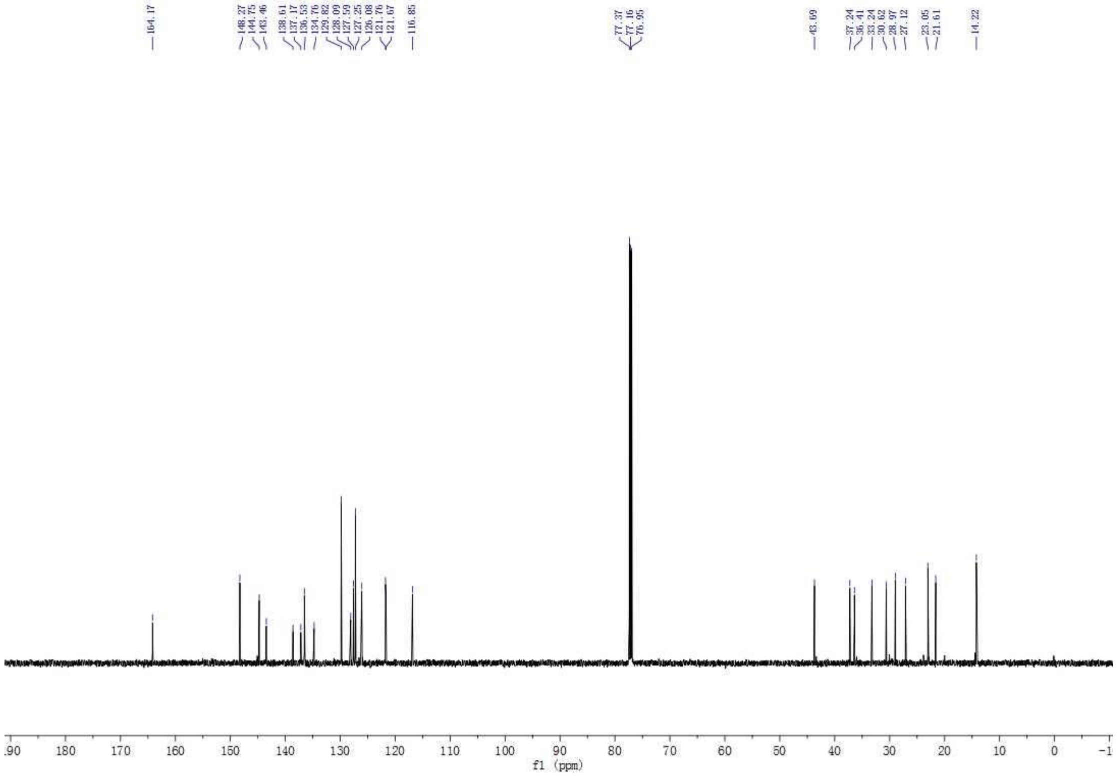

Supplementary Fig. 293. ^{13}C NMR of compound 4ah. The sample has been recorded in 150 MHz, $CDCl_3$ at 25 °C

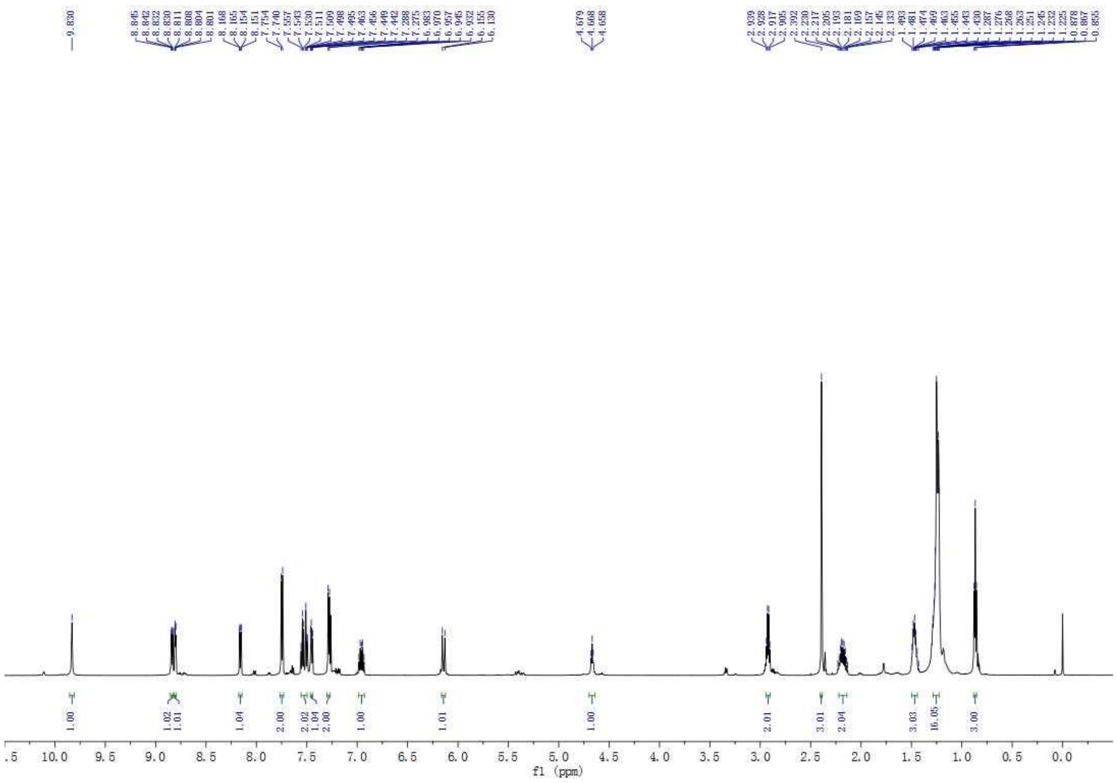

Supplementary Fig. 294. ^{1}H NMR of compound 4ai. The sample has been recorded in 600 MHz, $CDCl_3$ at 25 °C

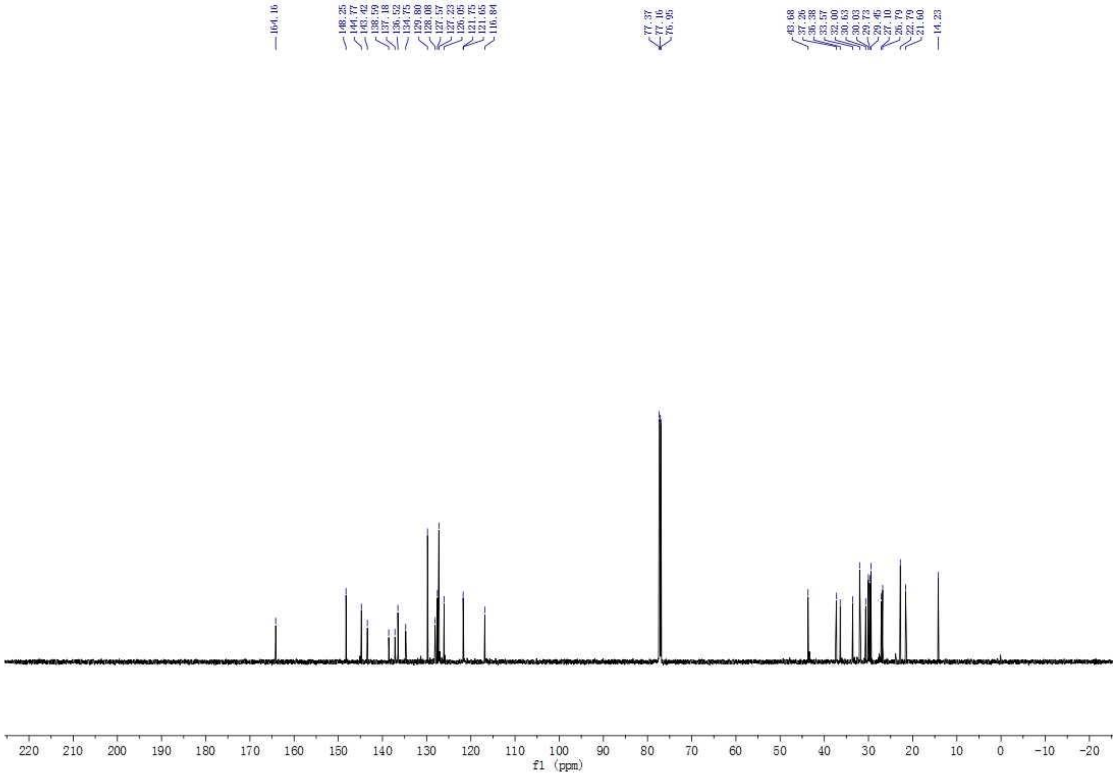

Supplementary Fig. 295. ^{13}C NMR of compound 4ai. The sample has been recorded in 150 MHz, $CDCl_3$ at 25 °C

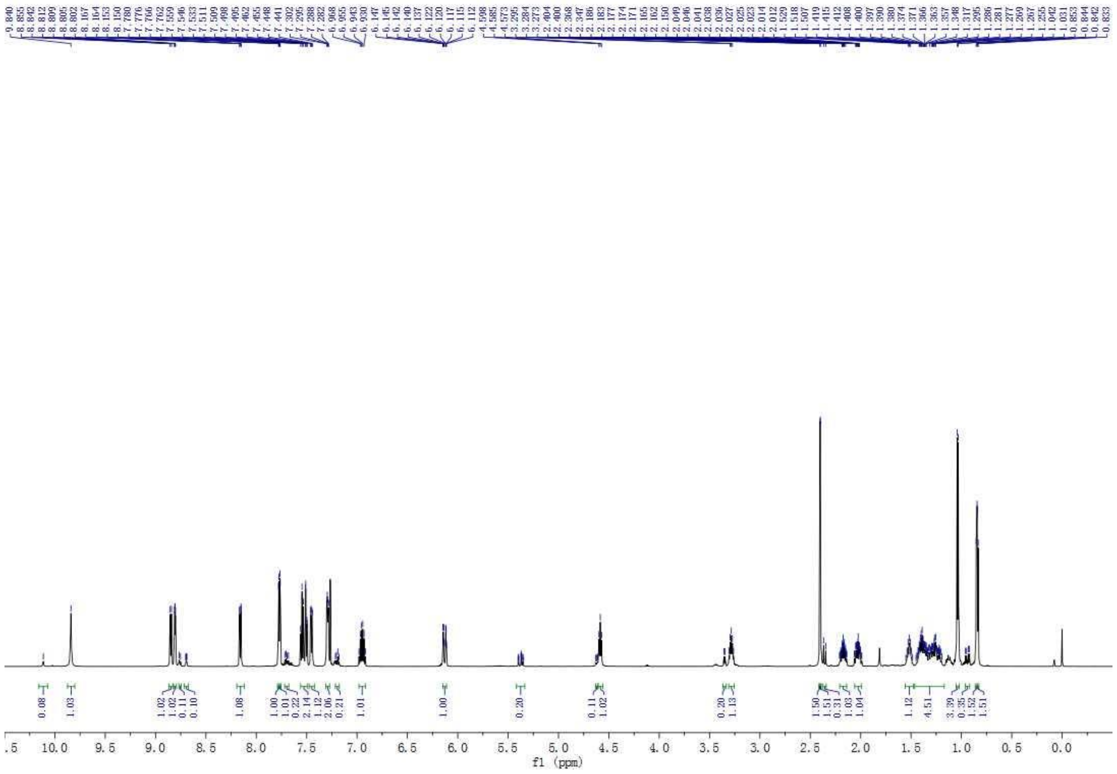

Supplementary Fig. 296. ^{1}H NMR of compound 4aj. The sample has been recorded in 600 MHz, $CDCl_3$ at 25 °C

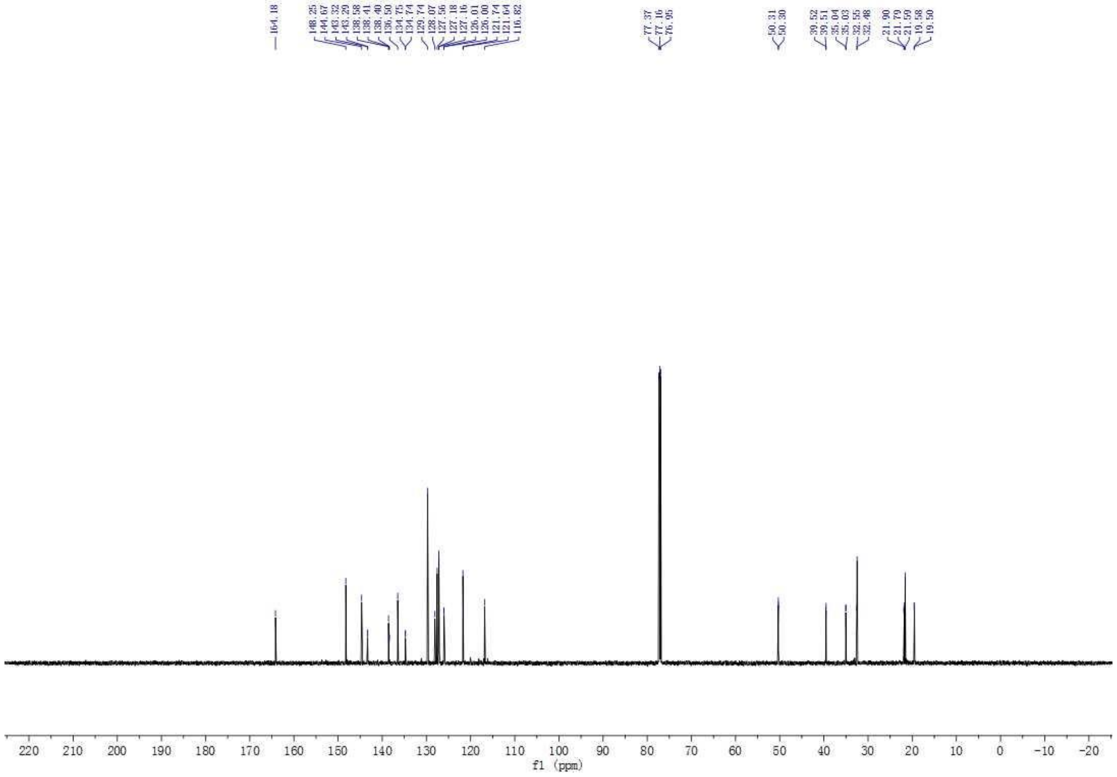

Supplementary Fig. 297. ^{13}C NMR of compound 4aj. The sample has been recorded in 150 MHz, $CDCl_3$ at 25 °C

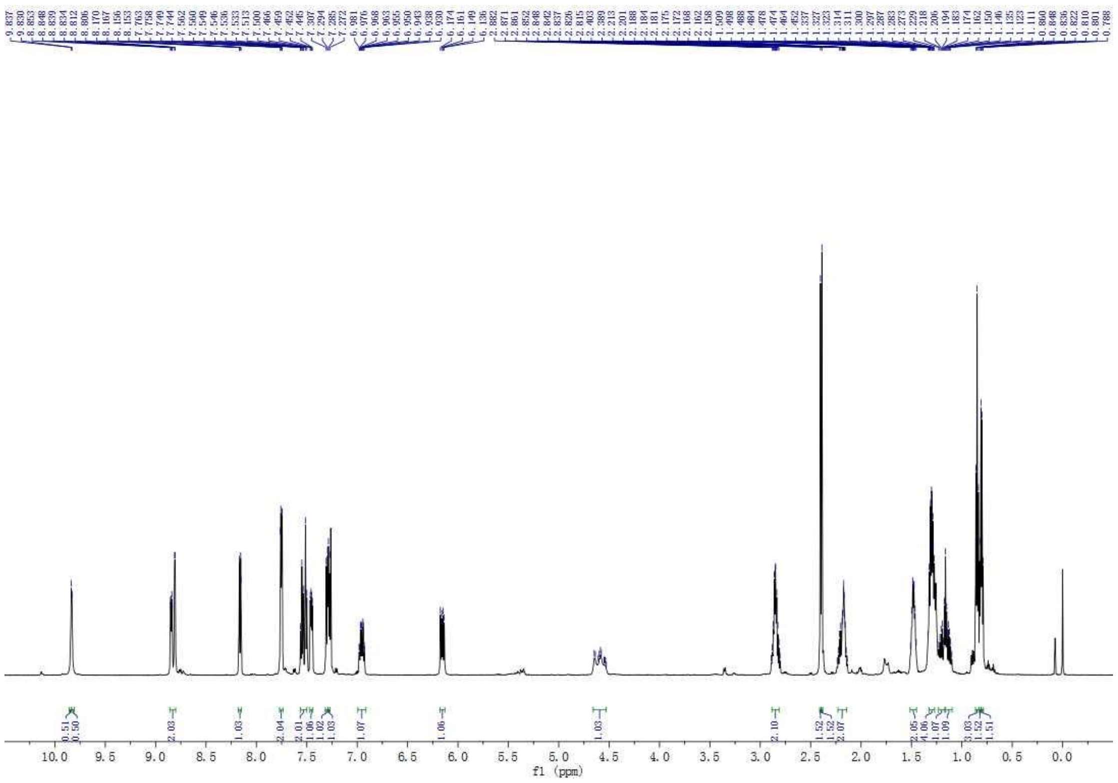

Supplementary Fig. 298. ^{1}H NMR of compound 4ak. The sample has been recorded in 600 MHz, $CDCl_3$ at 25 °C

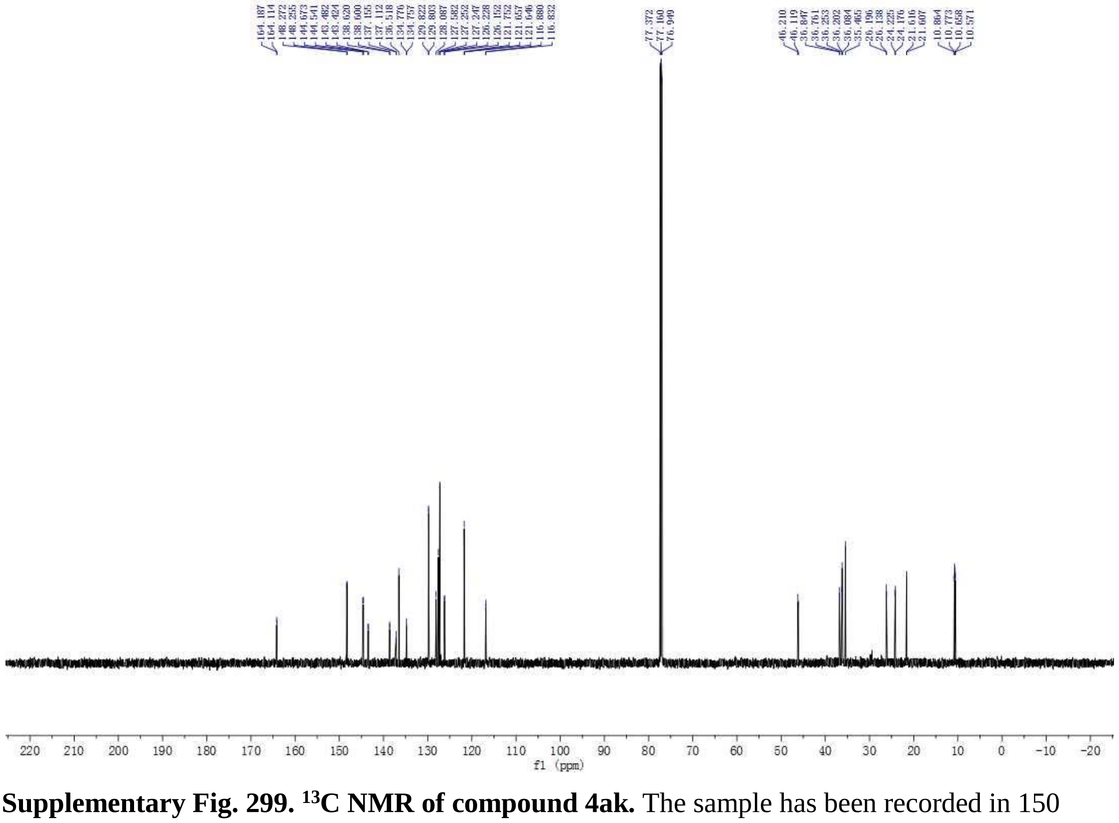

Supplementary Fig. 299. ^{13}C NMR of compound 4ak. The sample has been recorded in 150 MHz, $CDCl_3$ at 25 °C

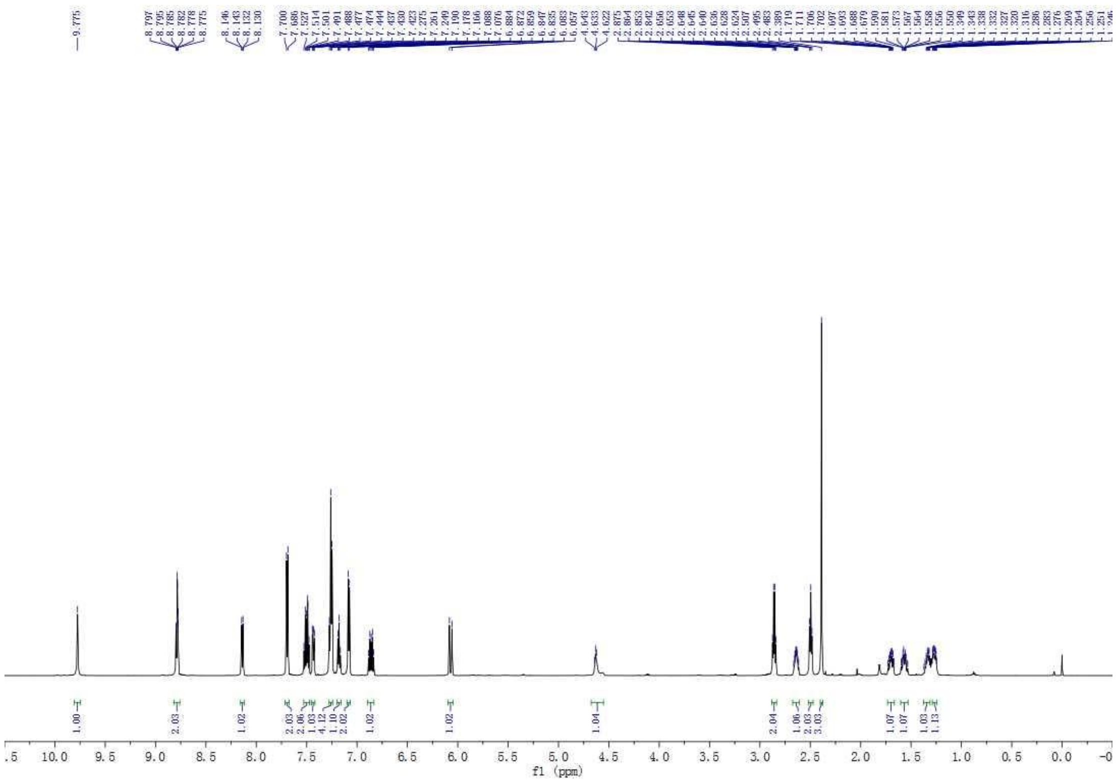

Supplementary Fig. 300. ^{1}H NMR of compound 4al. The sample has been recorded in 600 MHz, $CDCl_3$ at 25 °C

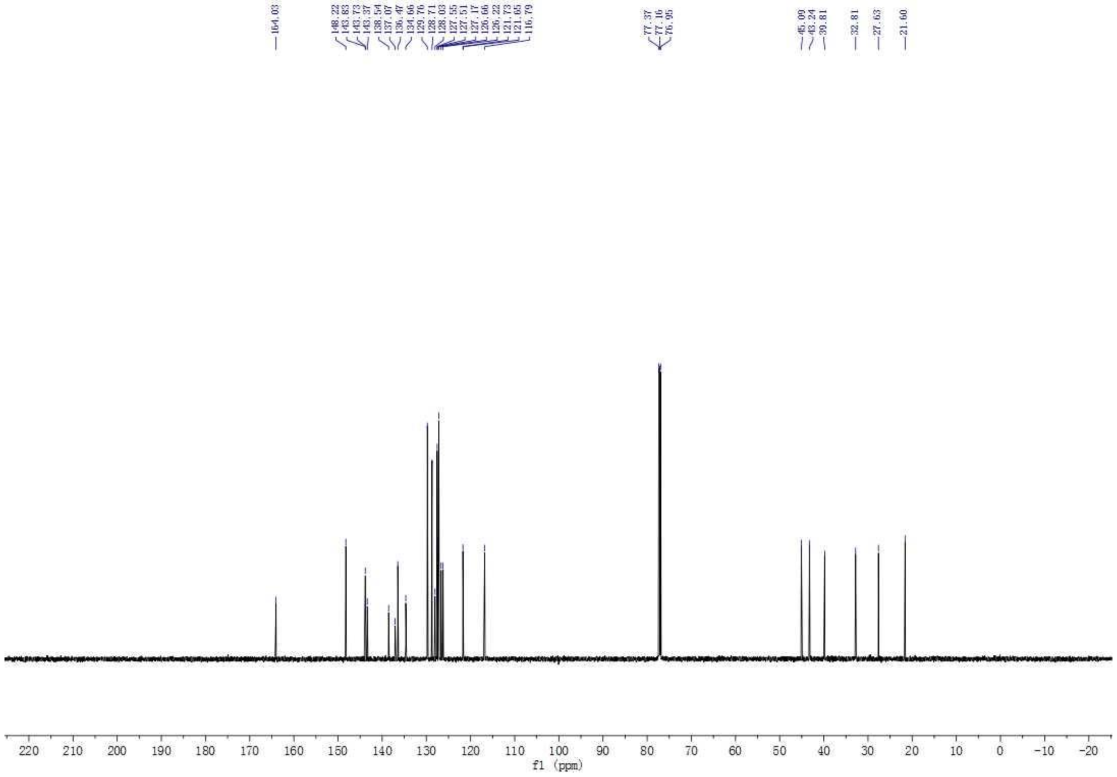

Supplementary Fig. 301. ^{13}C NMR of compound 4al. The sample has been recorded in 150 MHz, $CDCl_3$ at 25 °C

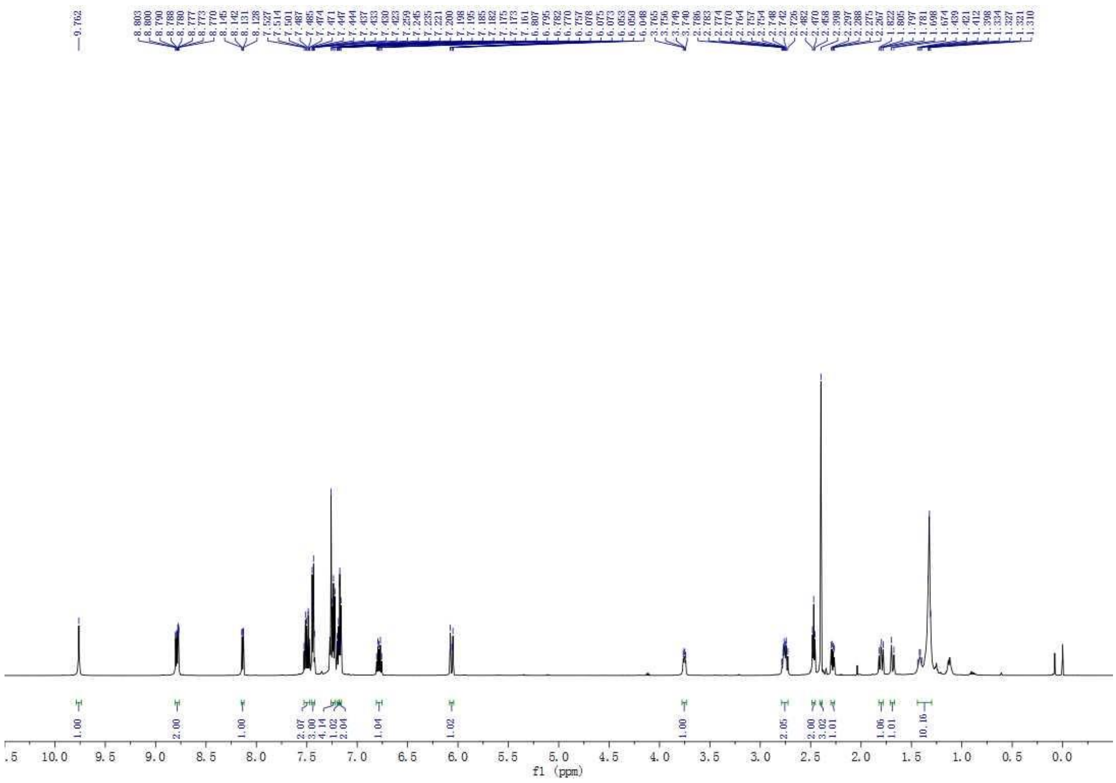

Supplementary Fig. 302. ^{1}H NMR of compound 4am. The sample has been recorded in 600 MHz, $CDCl_3$ at 25 °C

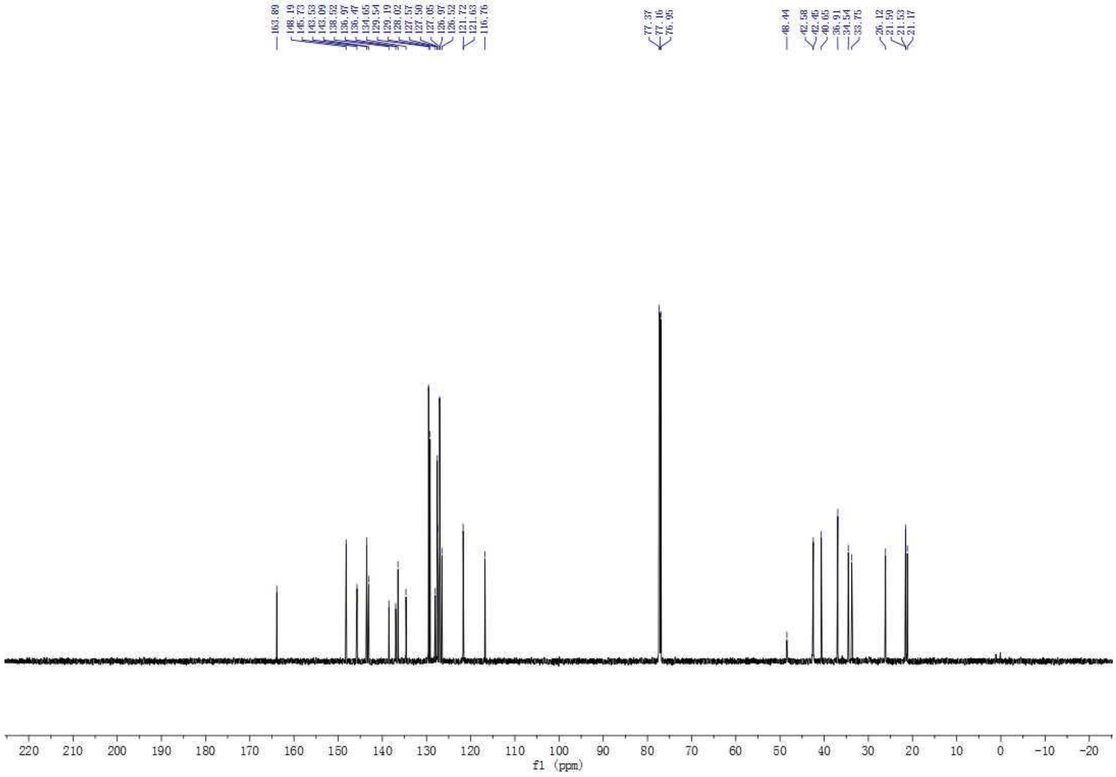

Supplementary Fig. 303. ^{13}C NMR of compound 4am. The sample has been recorded in 150 MHz, $CDCl_3$ at 25 °C

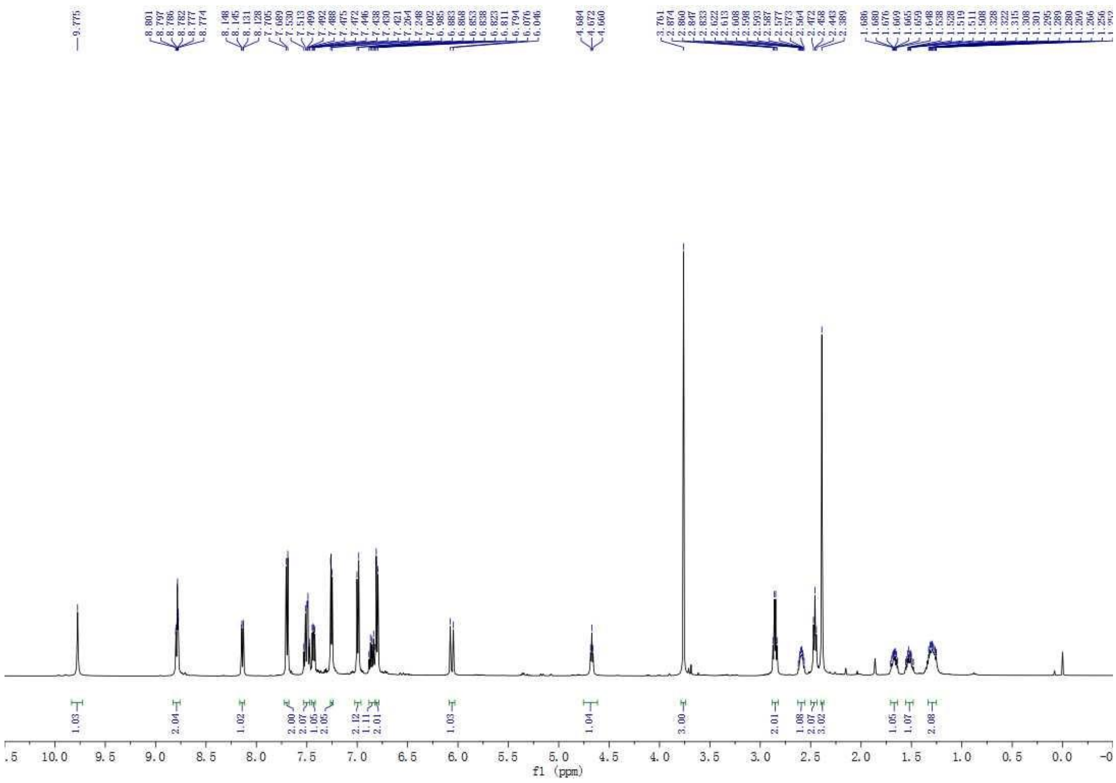

Supplementary Fig. 304. ^{1}H NMR of compound 4an. The sample has been recorded in 500 MHz, $CDCl_3$ at 25 °C

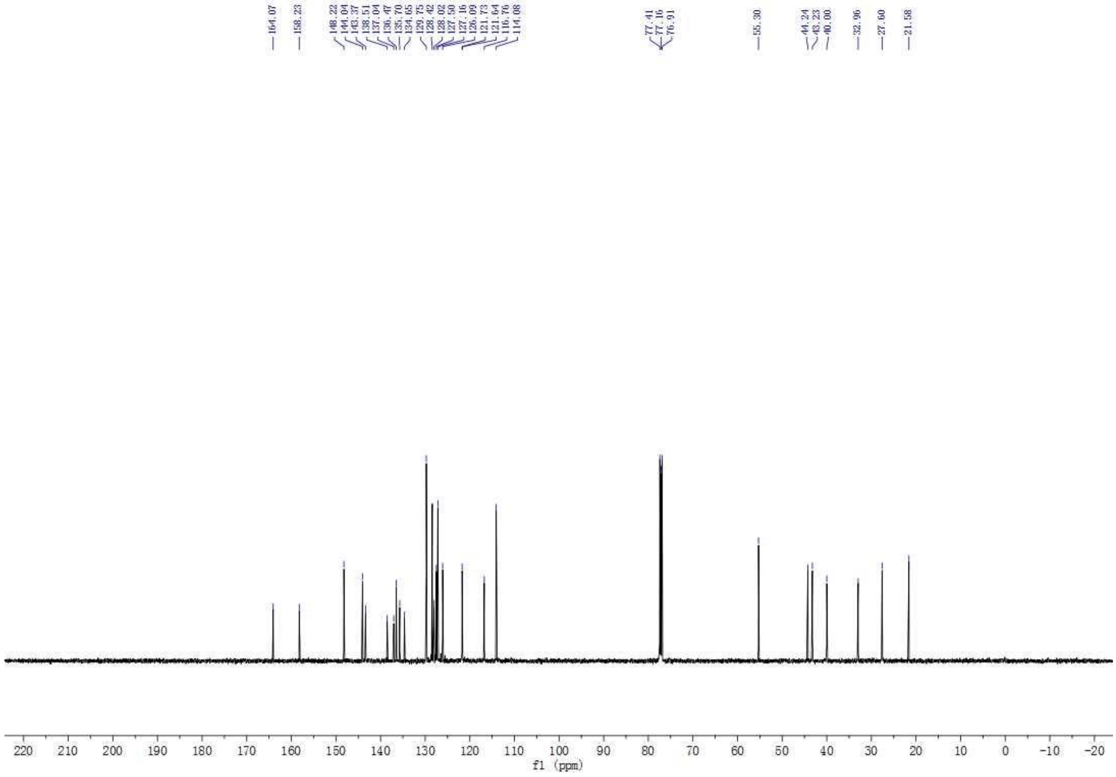

Supplementary Fig. 305. ^{13}C NMR of compound 4an. The sample has been recorded in 125 MHz, $CDCl_3$ at 25 °C

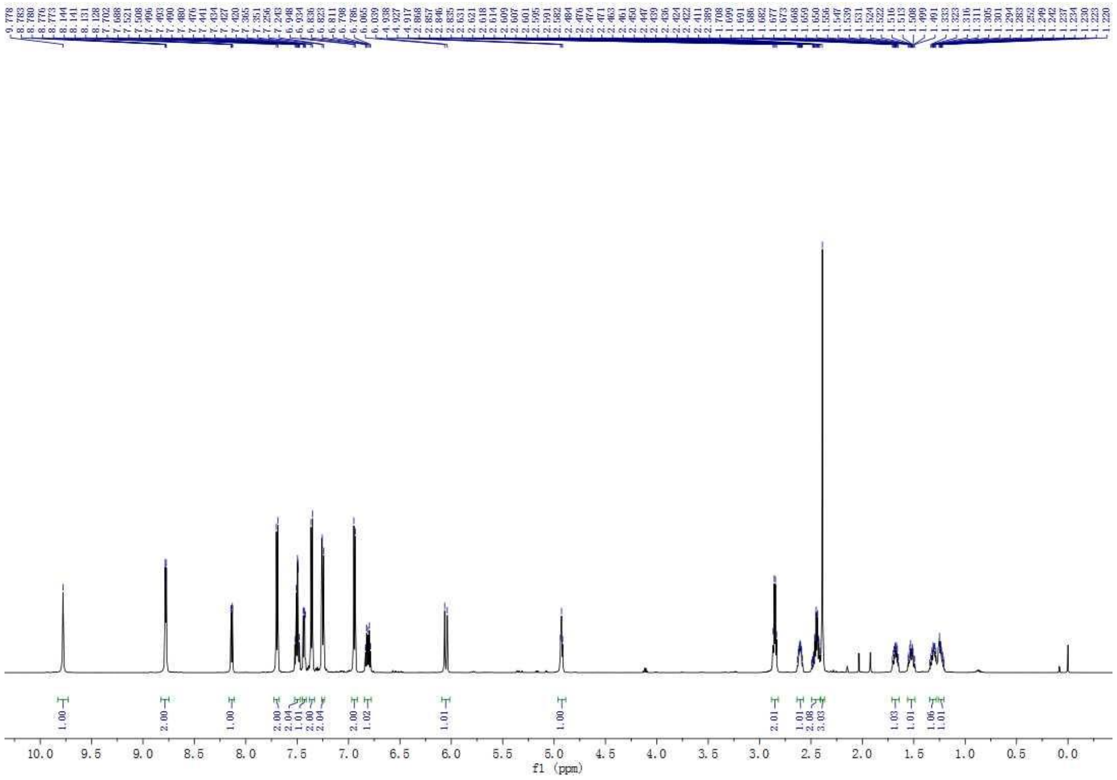

Supplementary Fig. 306. ^{1}H NMR of compound 4ao. The sample has been recorded in 600 MHz, $CDCl_3$ at 25 °C

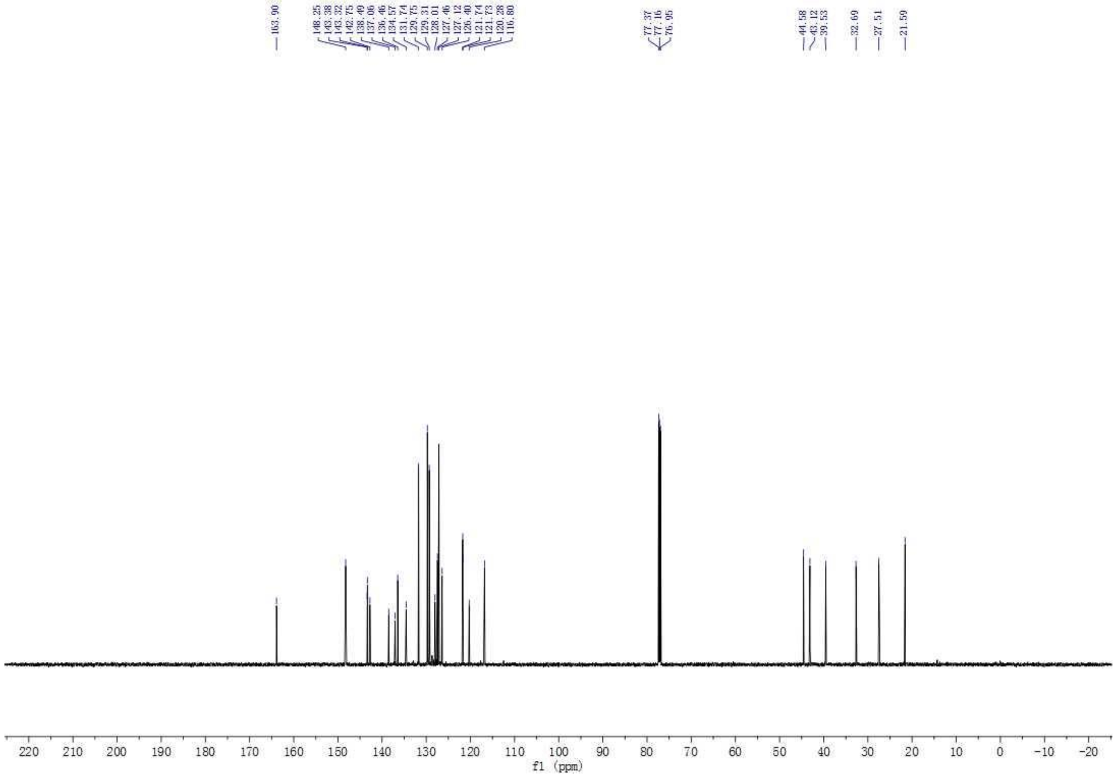

Supplementary Fig. 307. ^{13}C NMR of compound 4ao. The sample has been recorded in 150 MHz, $CDCl_3$ at 25 °C

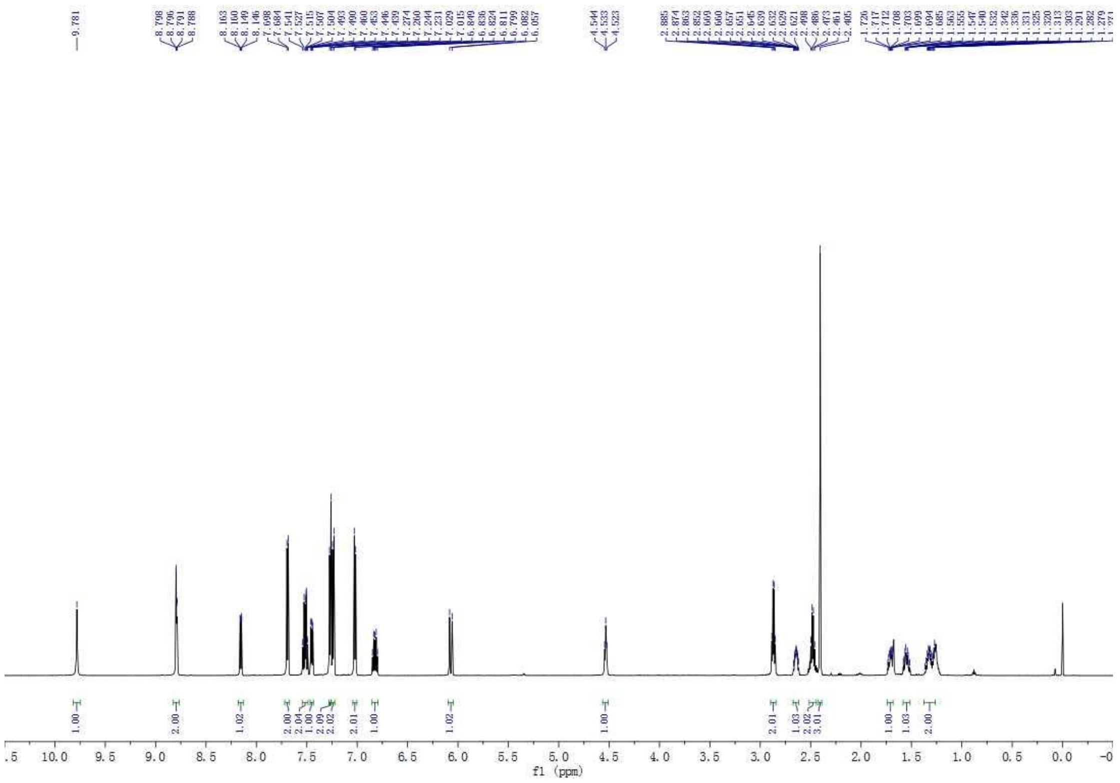

Supplementary Fig. 308. ^{1}H NMR of compound 4ap. The sample has been recorded in 600 MHz, $CDCl_3$ at 25 °C

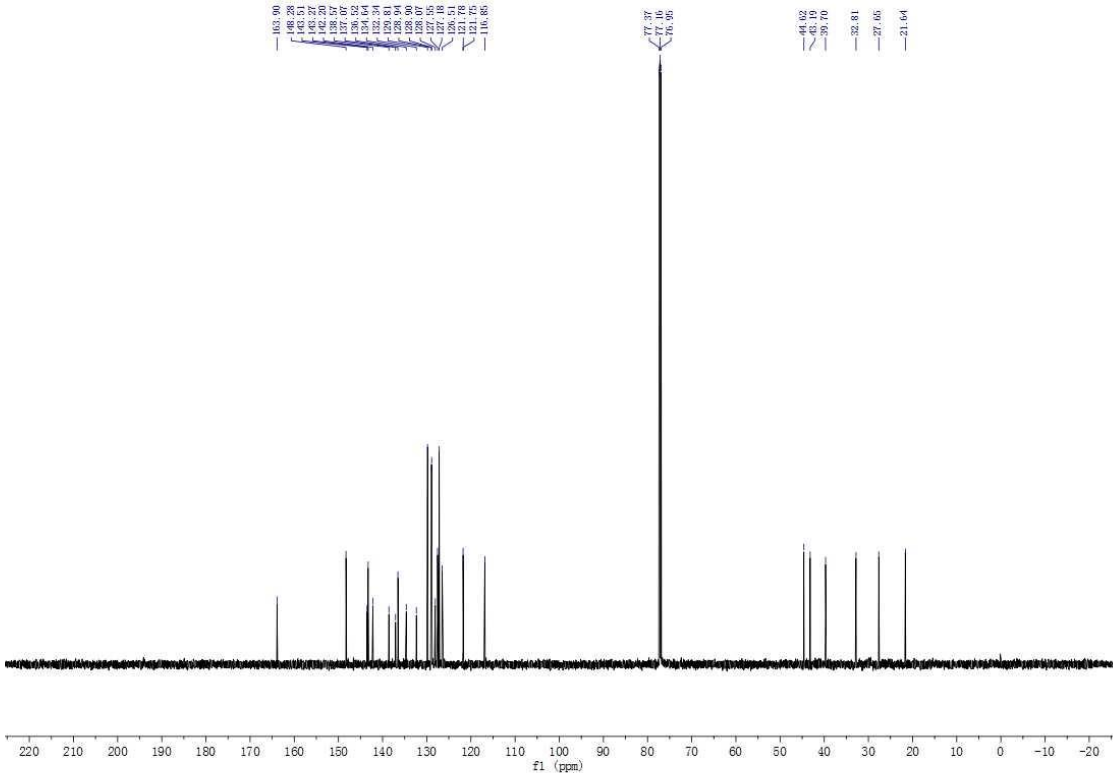

Supplementary Fig. 309. ^{13}C NMR of compound 4ap. The sample has been recorded in 150 MHz, $CDCl_3$ at 25 °C

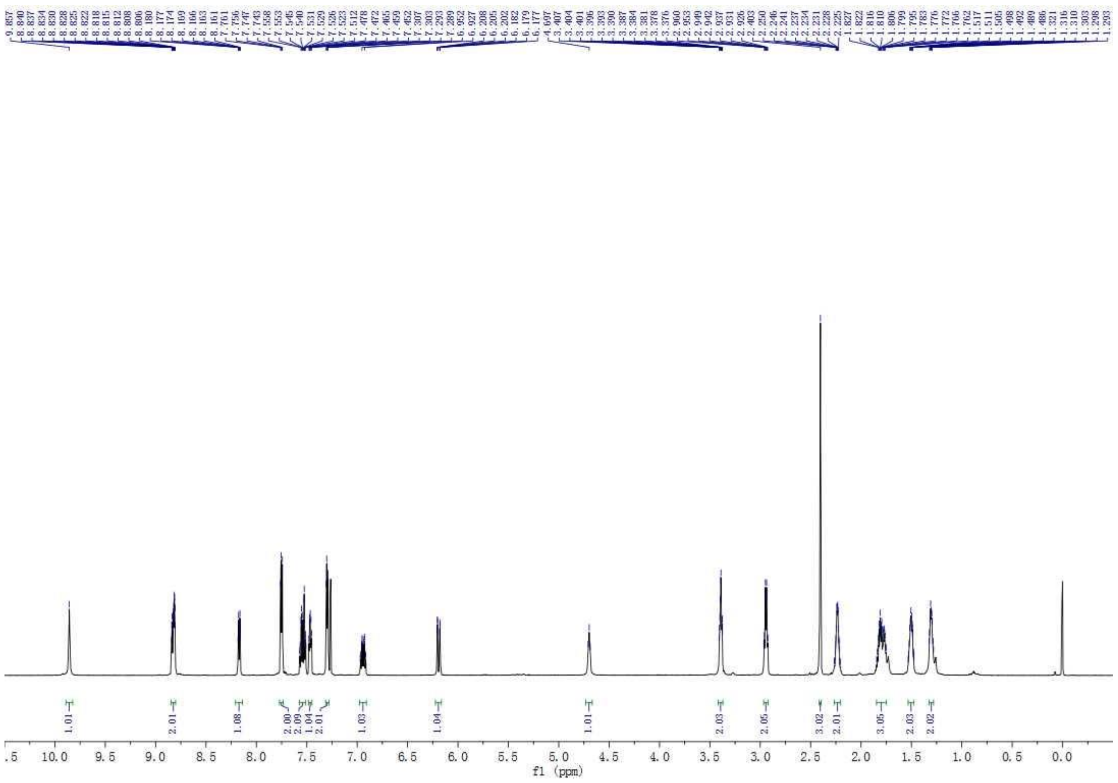

Supplementary Fig. 310. ¹H NMR of compound 4aq. The sample has been recorded in 600 MHz, $CDCl_3$ at 25 °C

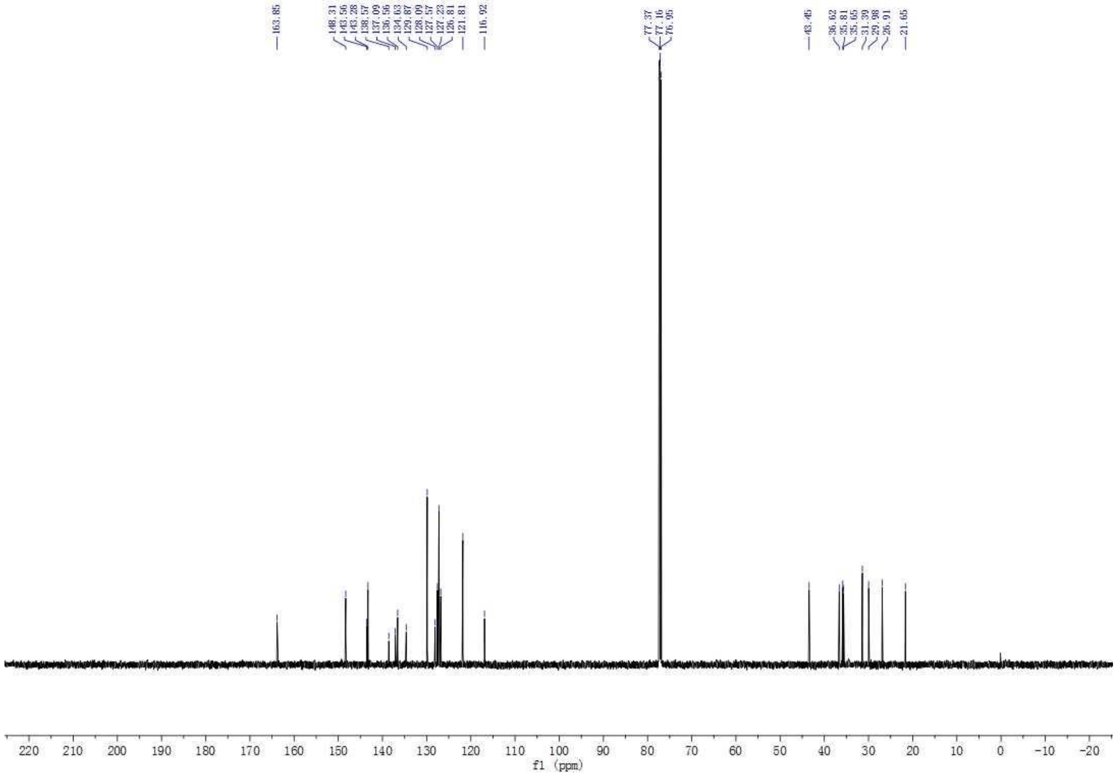

Supplementary Fig. 311. ¹³C NMR of compound 4aq. The sample has been recorded in 150 MHz, $CDCl_3$ at 25 °C

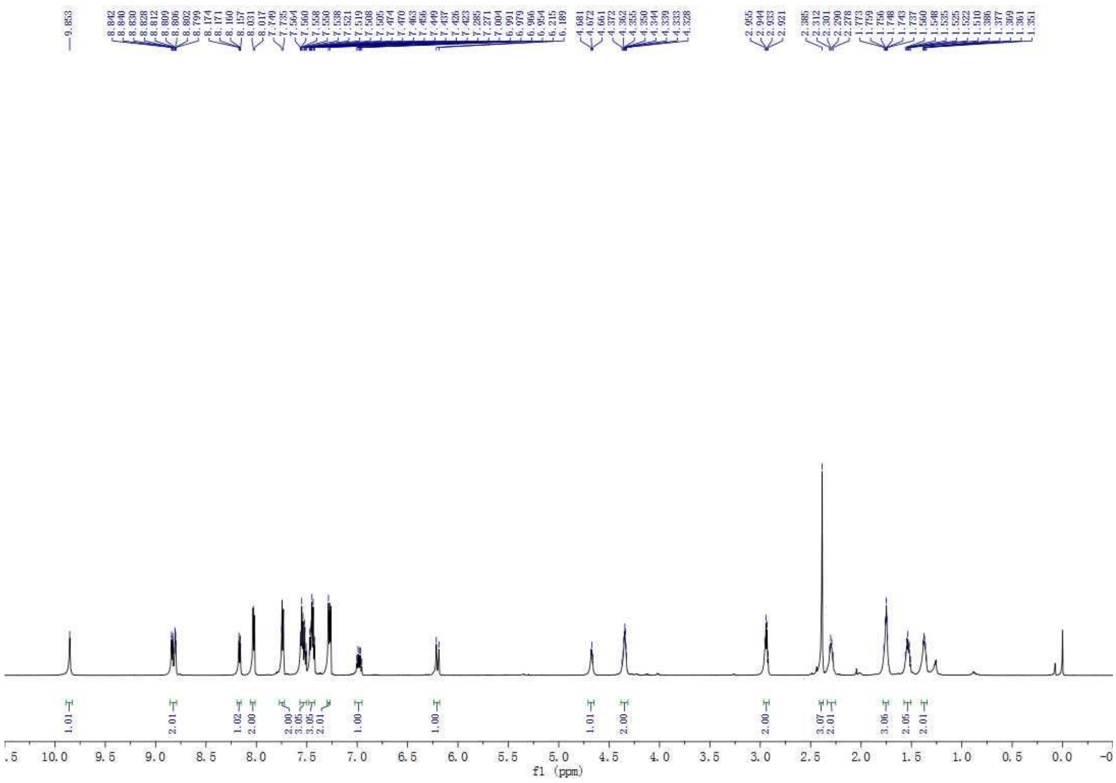

Supplementary Fig. 312. ^{1}H NMR of compound 4ar. The sample has been recorded in 600 MHz, $CDCl_3$ at 25 °C

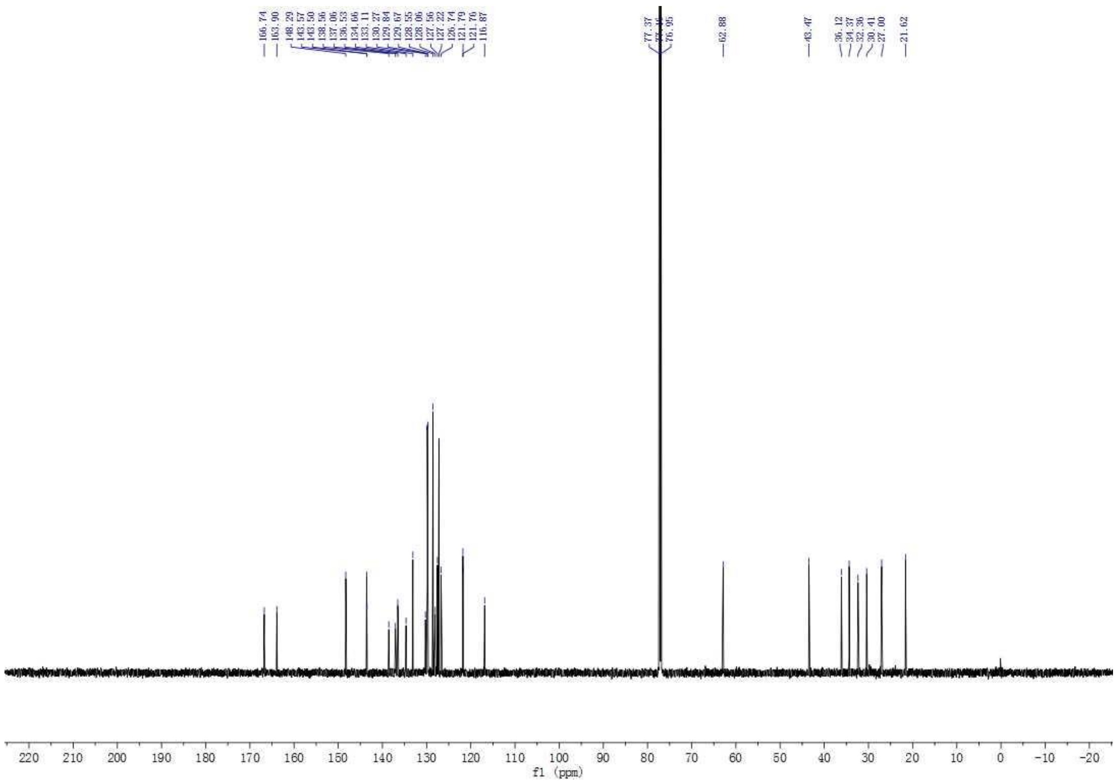

Supplementary Fig. 313. ^{13}C NMR of compound 4ar. The sample has been recorded in 150 MHz, $CDCl_3$ at 25 °C

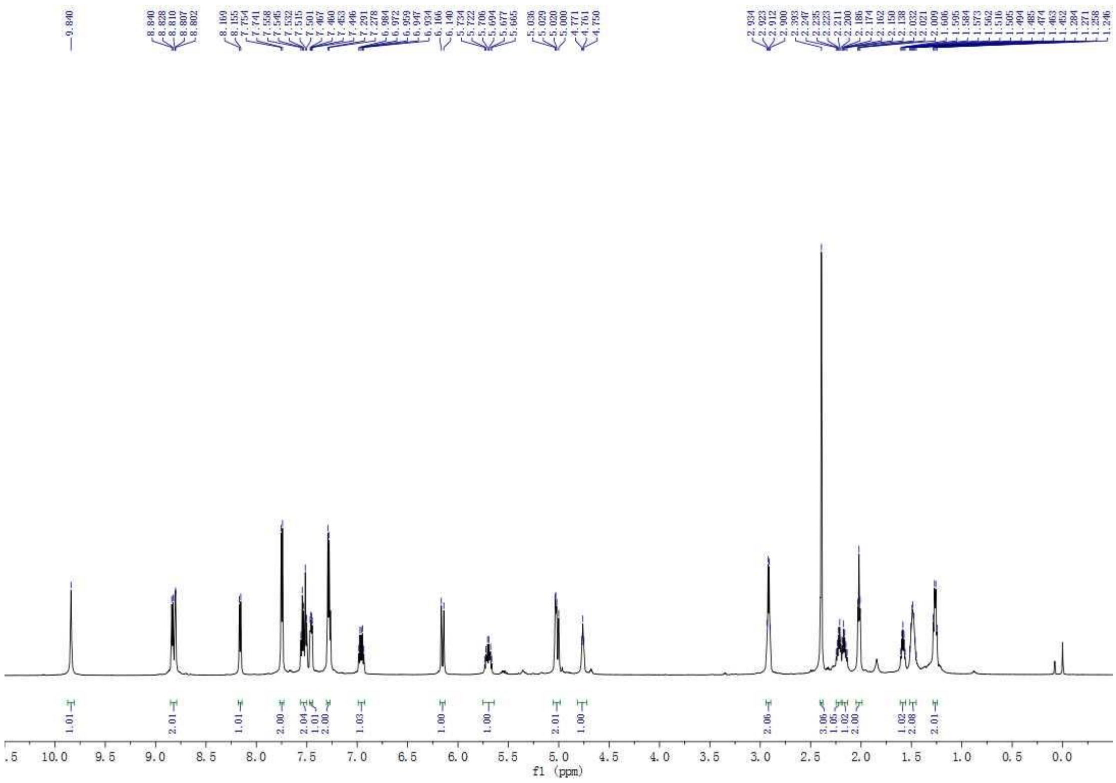

Supplementary Fig. 314. ^{1}H NMR of compound 4as. The sample has been recorded in 600 MHz, $CDCl_3$ at 25 °C

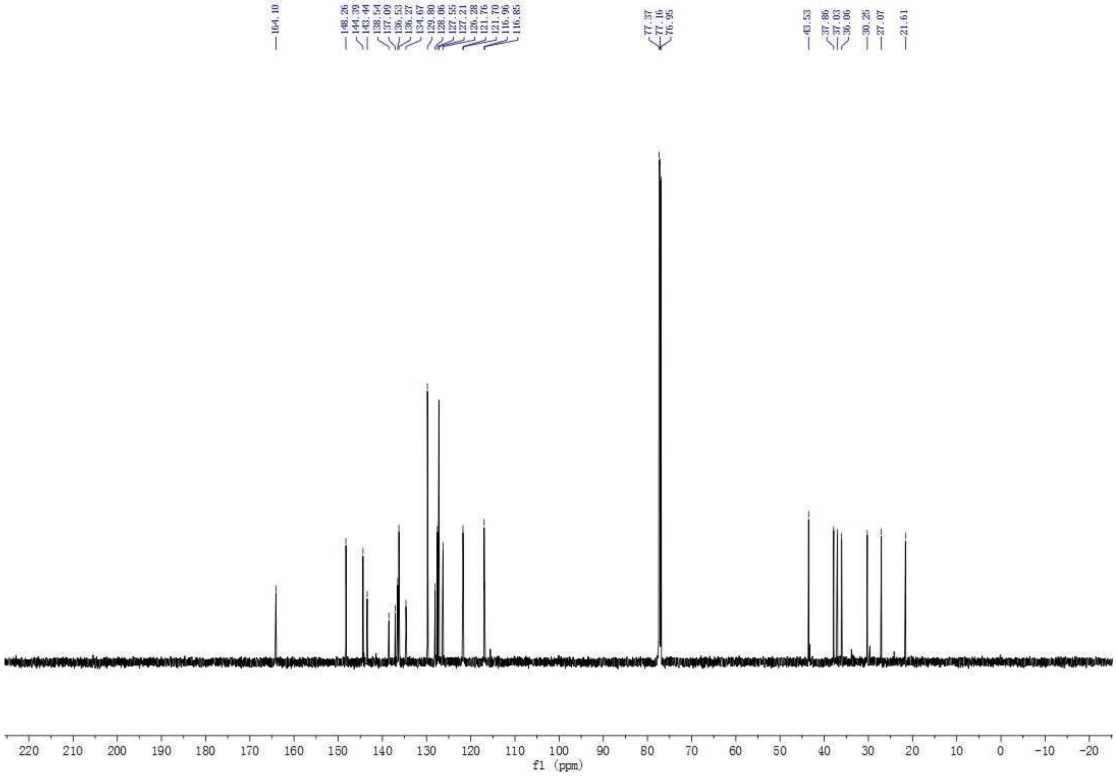

Supplementary Fig. 315. ^{13}C NMR of compound 4as. The sample has been recorded in 150 MHz, $CDCl_3$ at 25 °C

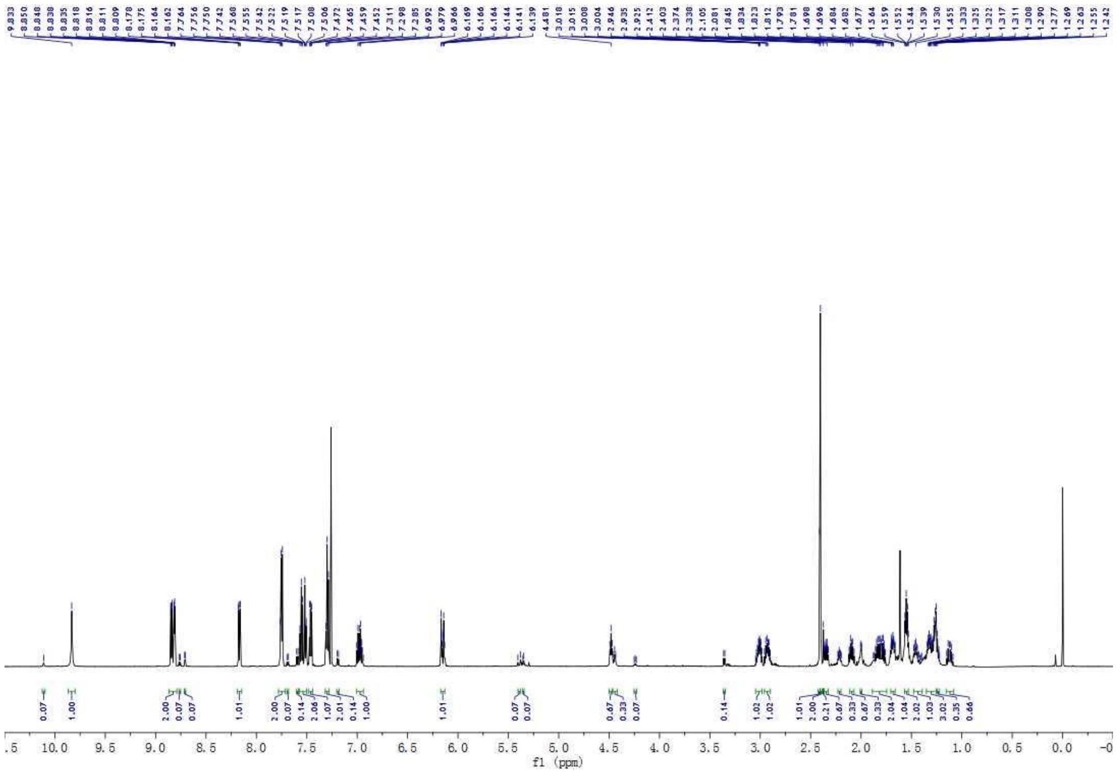

Supplementary Fig. 316. ^{1}H NMR of compound 4au. The sample has been recorded in 600 MHz, $CDCl_3$ at 25 °C

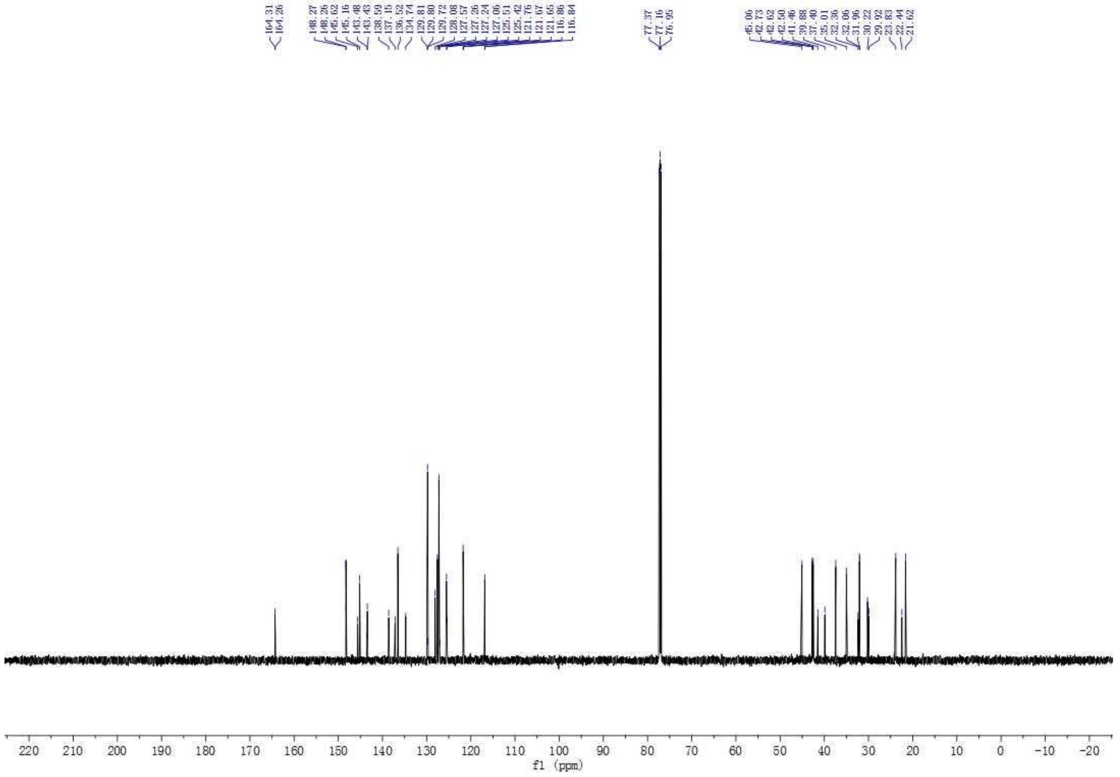

Supplementary Fig. 317. ^{13}C NMR of compound 4au. The sample has been recorded in 150 MHz, $CDCl_3$ at 25 °C

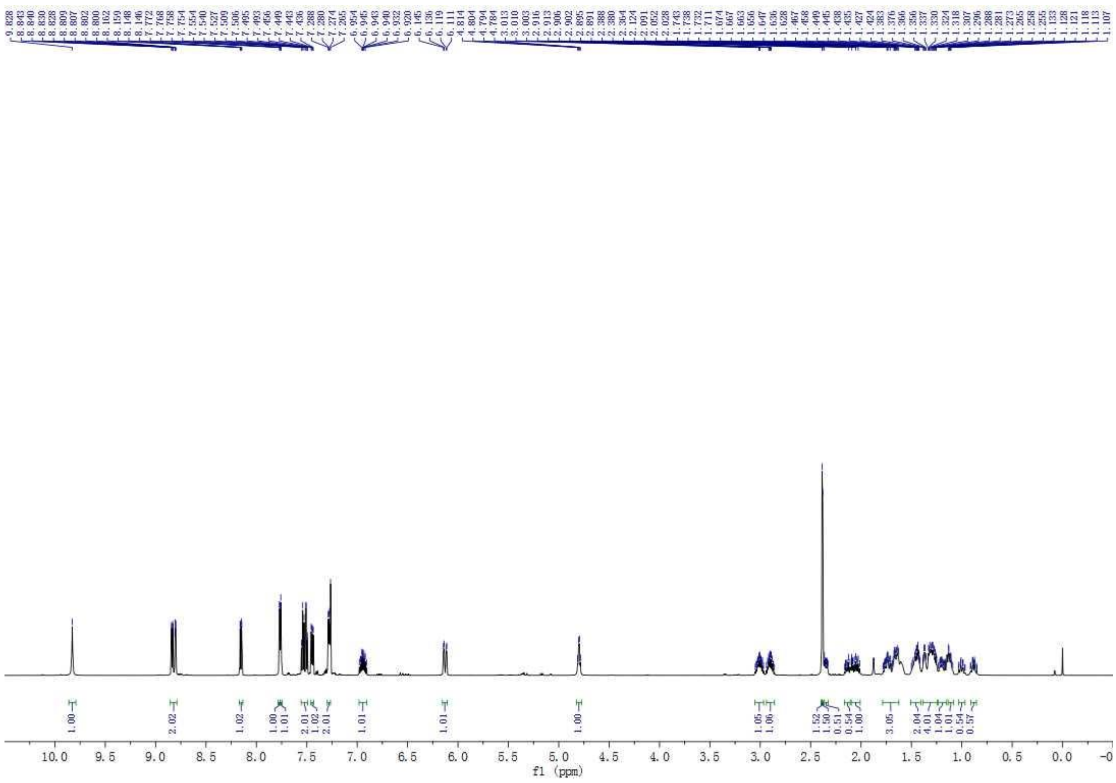

Supplementary Fig. 318. ^{1}H NMR of compound 4av. The sample has been recorded in 600 MHz, $CDCl_3$ at 25 °C

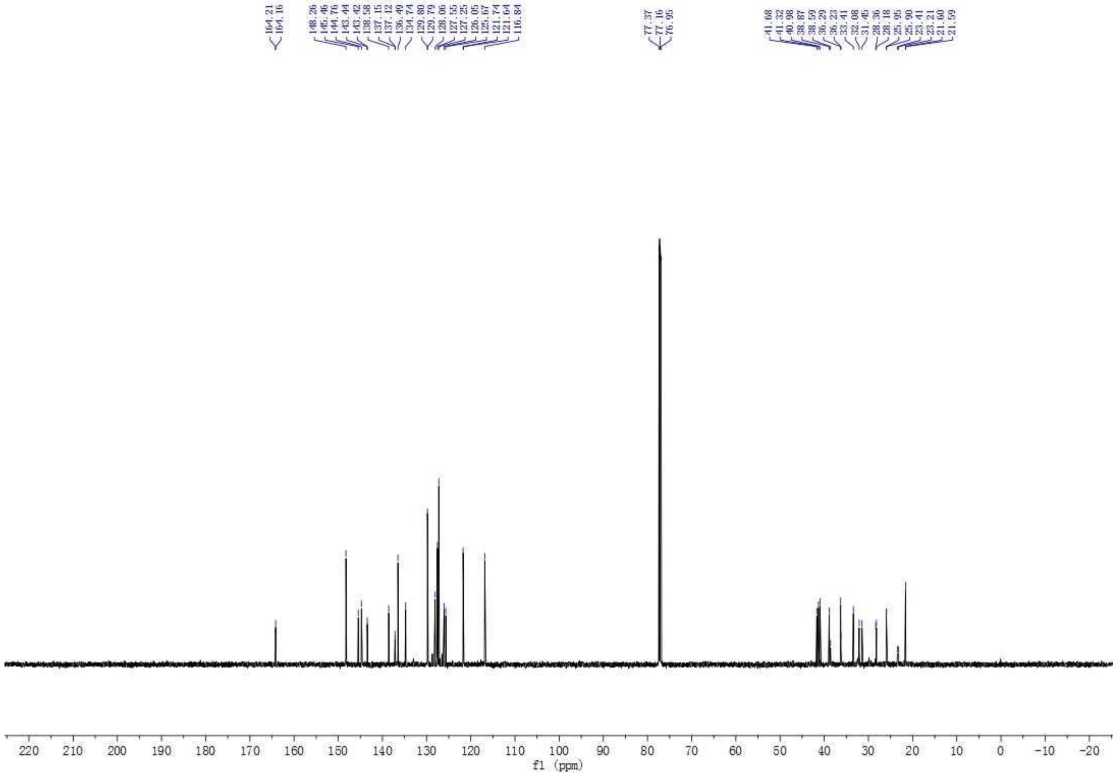

Supplementary Fig. 319. ^{13}C NMR of compound 4av. The sample has been recorded in 150 MHz, $CDCl_3$ at 25 °C

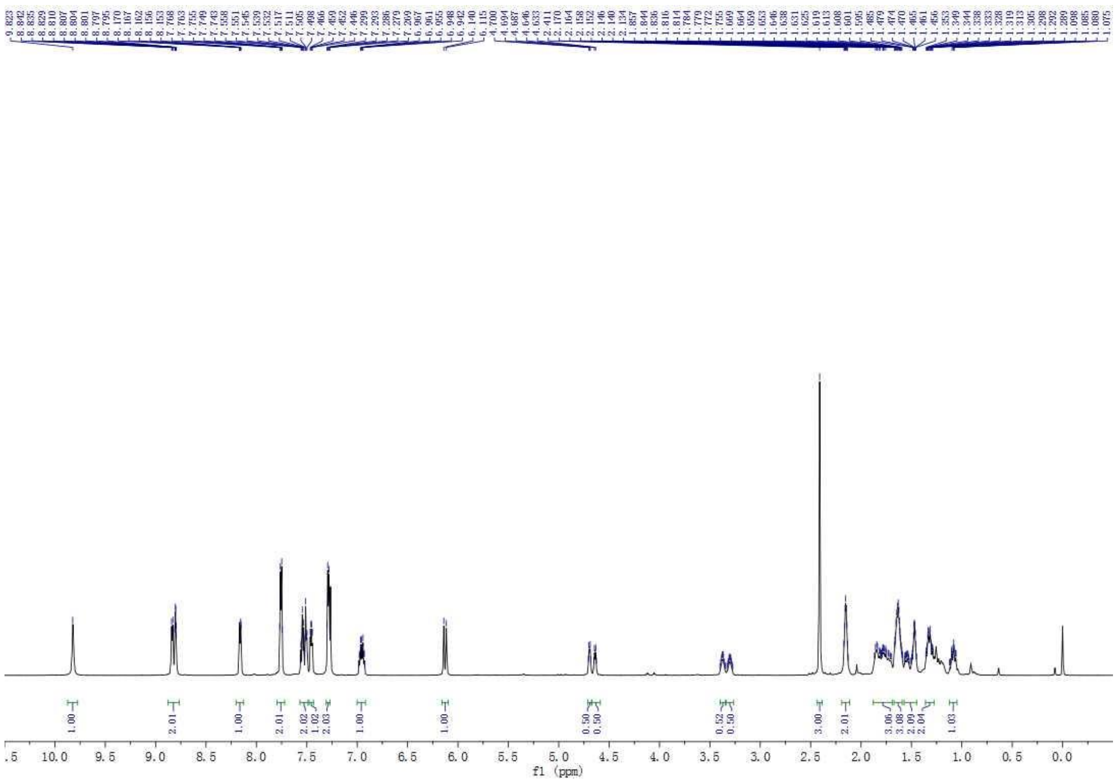

Supplementary Fig. 320. ^{1}H NMR of compound 4aw. The sample has been recorded in 600 MHz, $CDCl_3$ at 25 °C

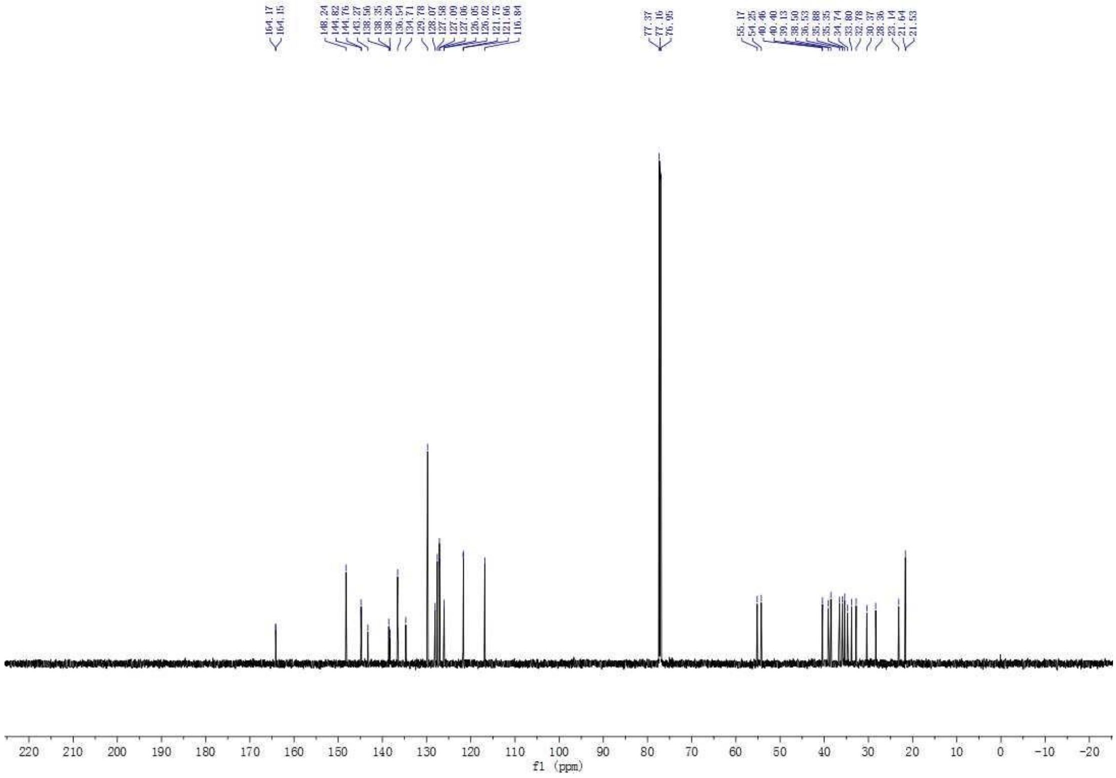

Supplementary Fig. 321. ^{13}C NMR of compound 4aw. The sample has been recorded in 150 MHz, $CDCl_3$ at 25 °C

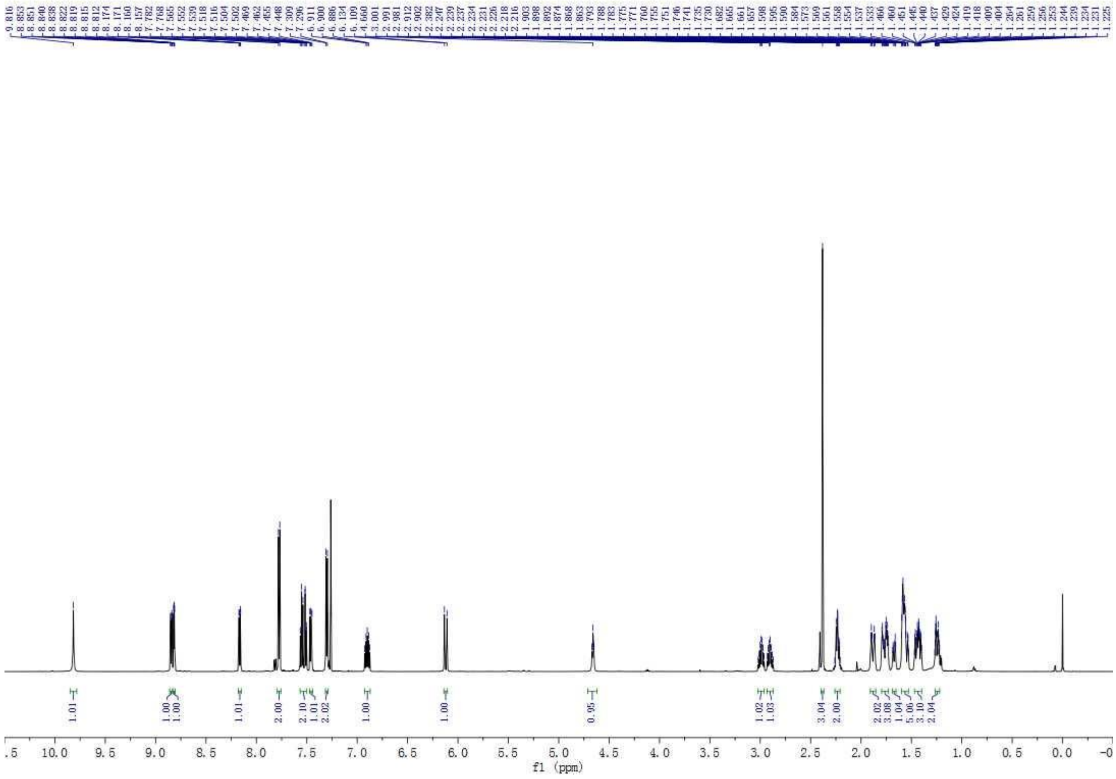

Supplementary Fig. 322. ^{1}H NMR of compound 4ax. The sample has been recorded in 600 MHz, $CDCl_3$ at 25 °C

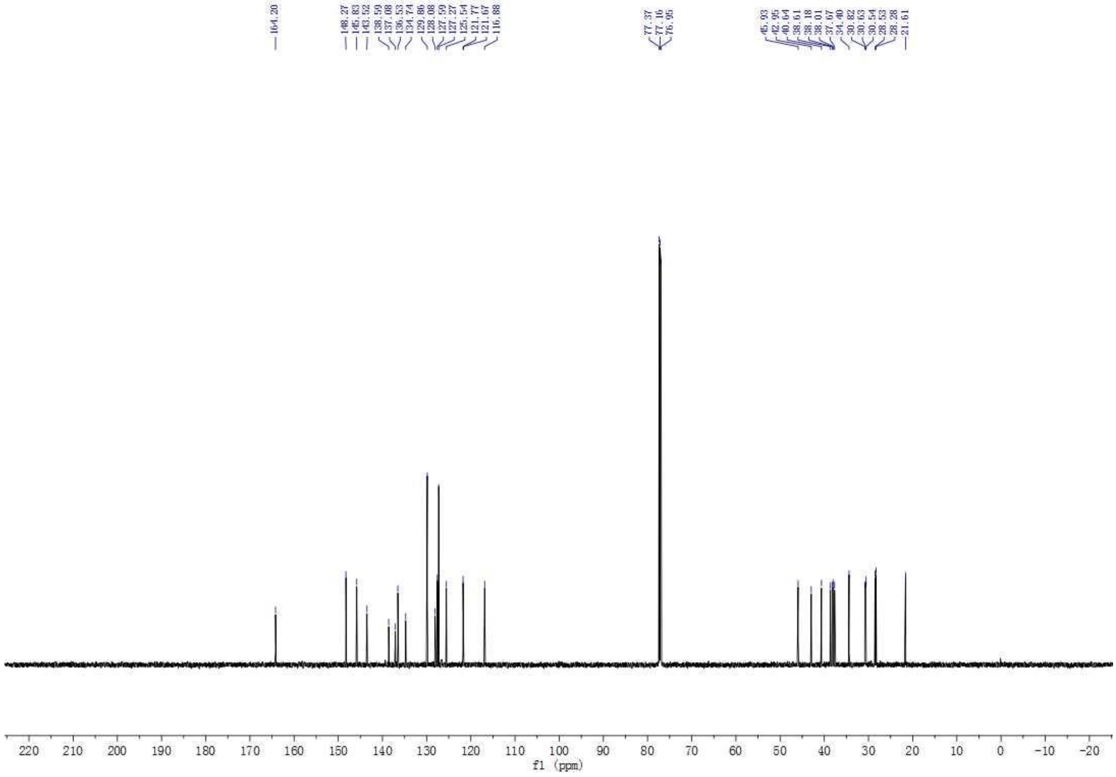

Supplementary Fig. 323. ^{13}C NMR of compound 4ax. The sample has been recorded in 150 MHz, $CDCl_3$ at 25 °C

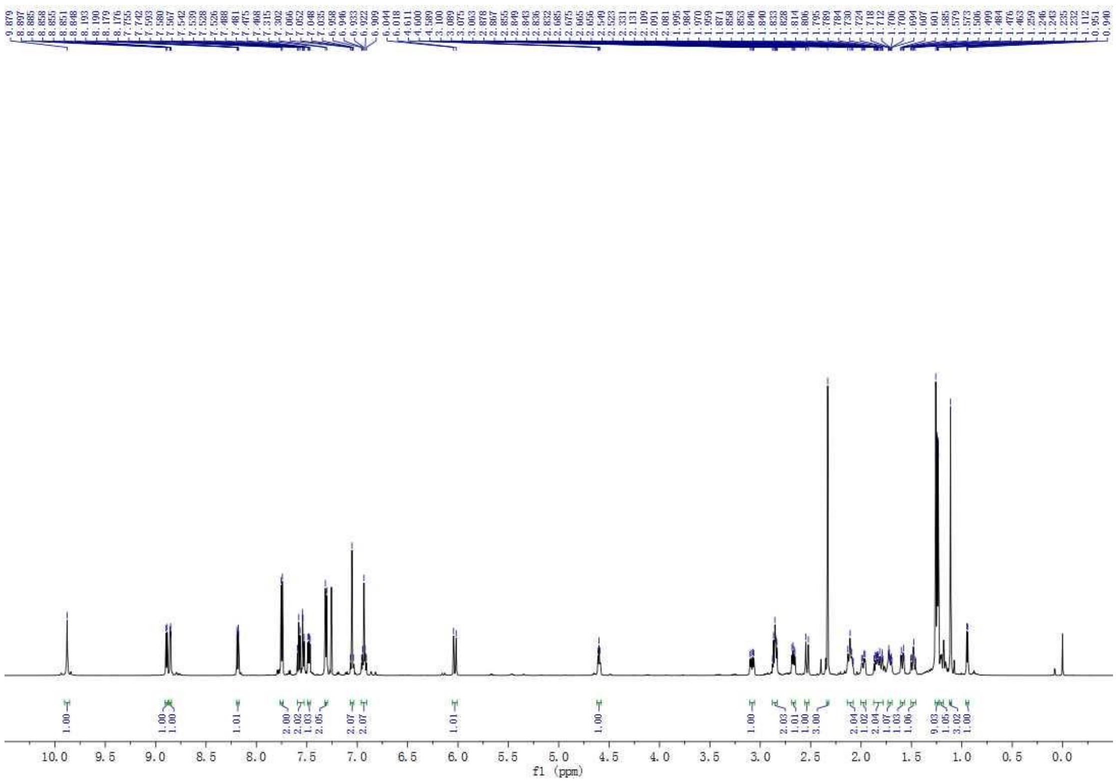

Supplementary Fig. 324. ^{1}H NMR of compound 4ay. The sample has been recorded in 600 MHz, $CDCl_3$ at 25 °C

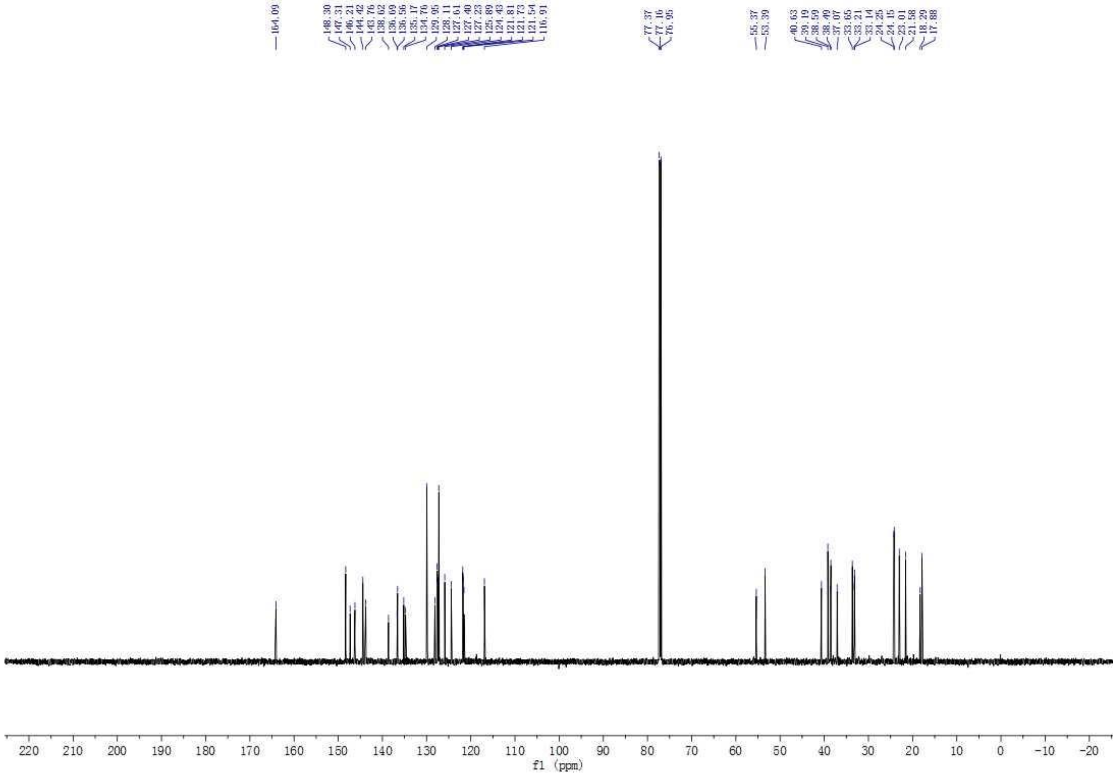

Supplementary Fig. 325. ^{13}C NMR of compound 4ay. The sample has been recorded in 150 MHz, $CDCl_3$ at 25 °C

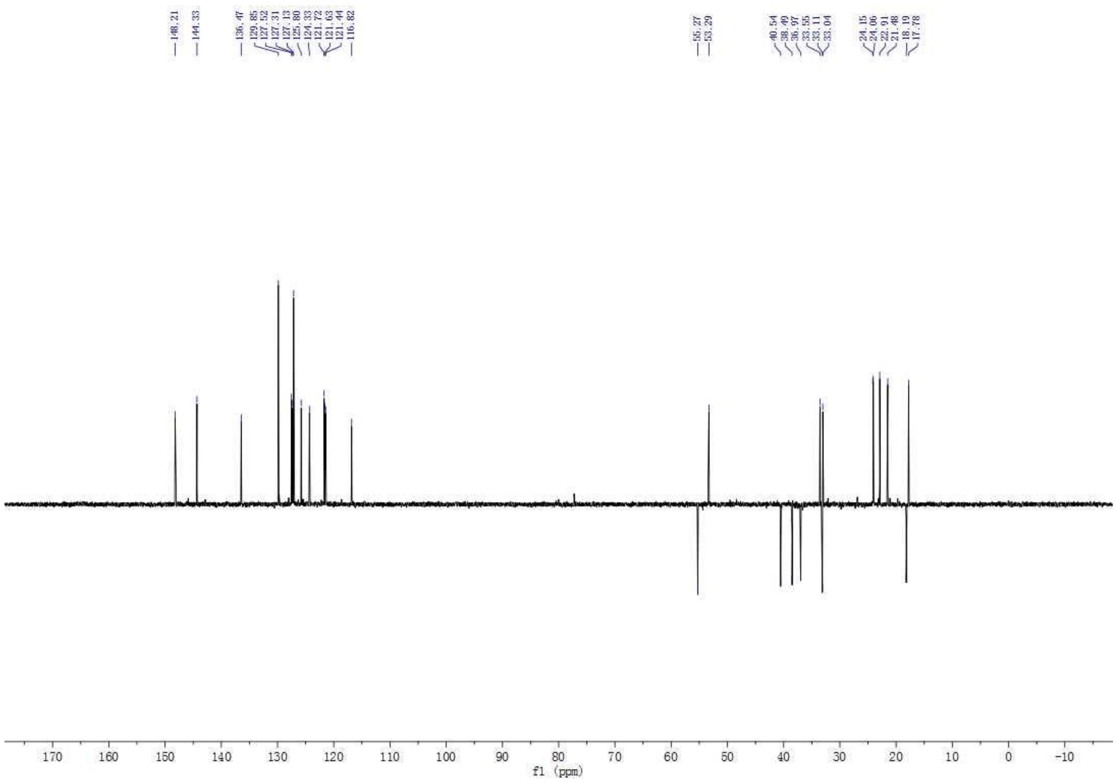

Supplementary Fig. 326. Dept 135° of compound 4ay. The sample has been recorded in 150 MHz, $CDCl_3$ at 25 °C

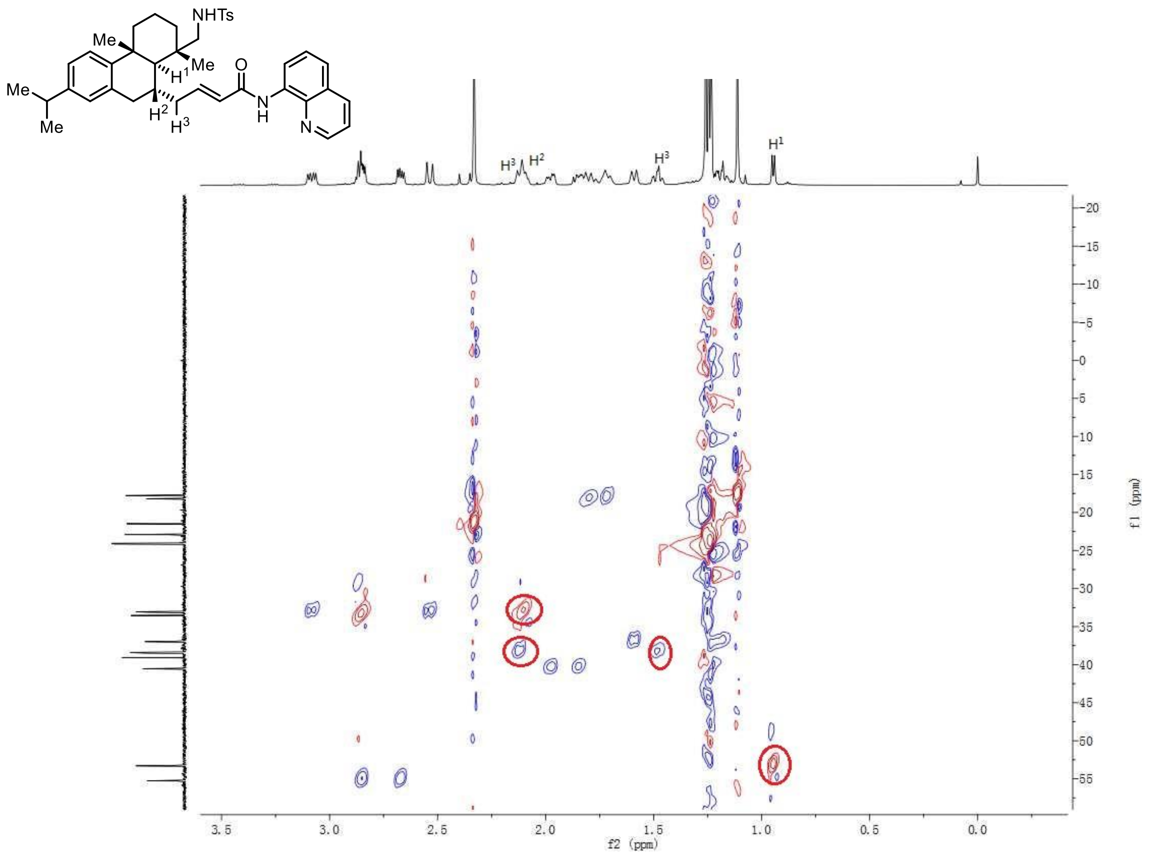

Supplementary Fig. 327. HSQC of compound 4ay. The sample has been recorded in 600 MHz, $CDCl_3$ at 25 °C

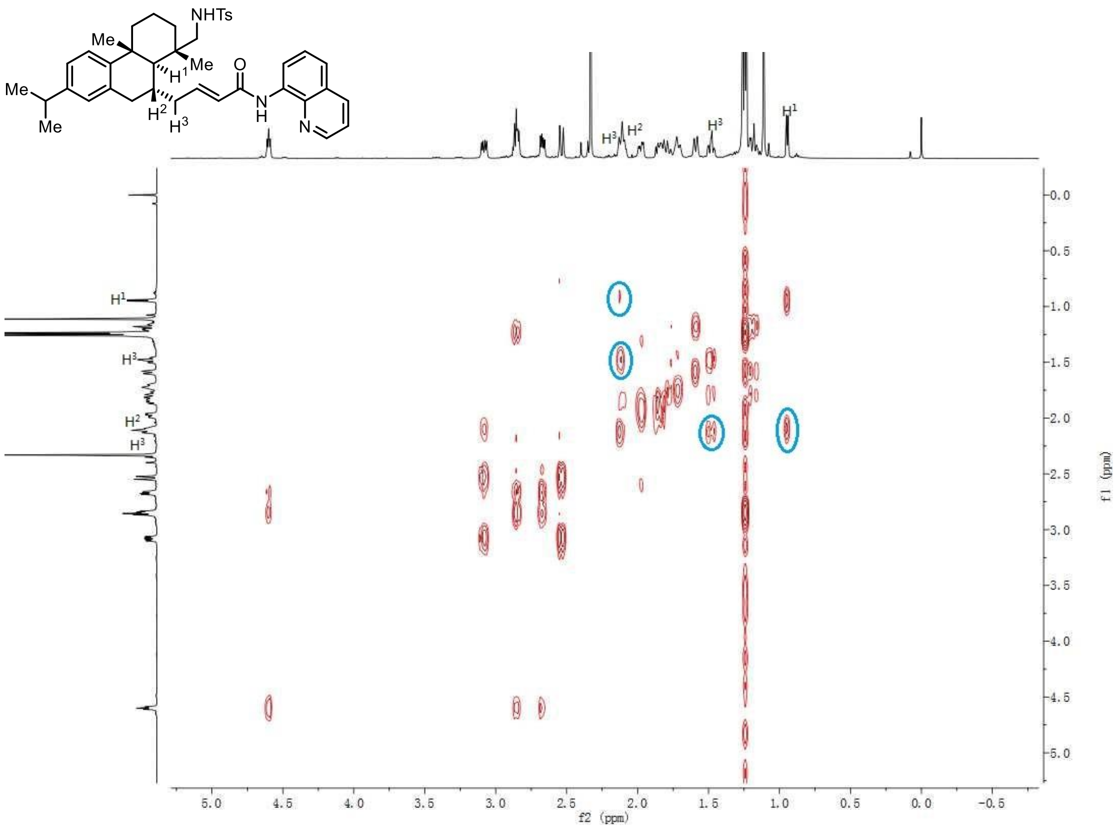

Supplementary Fig. 328. Cosy of compound 4ay. The sample has been recorded in 600 MHz, $CDCl_3$ at 25 °C

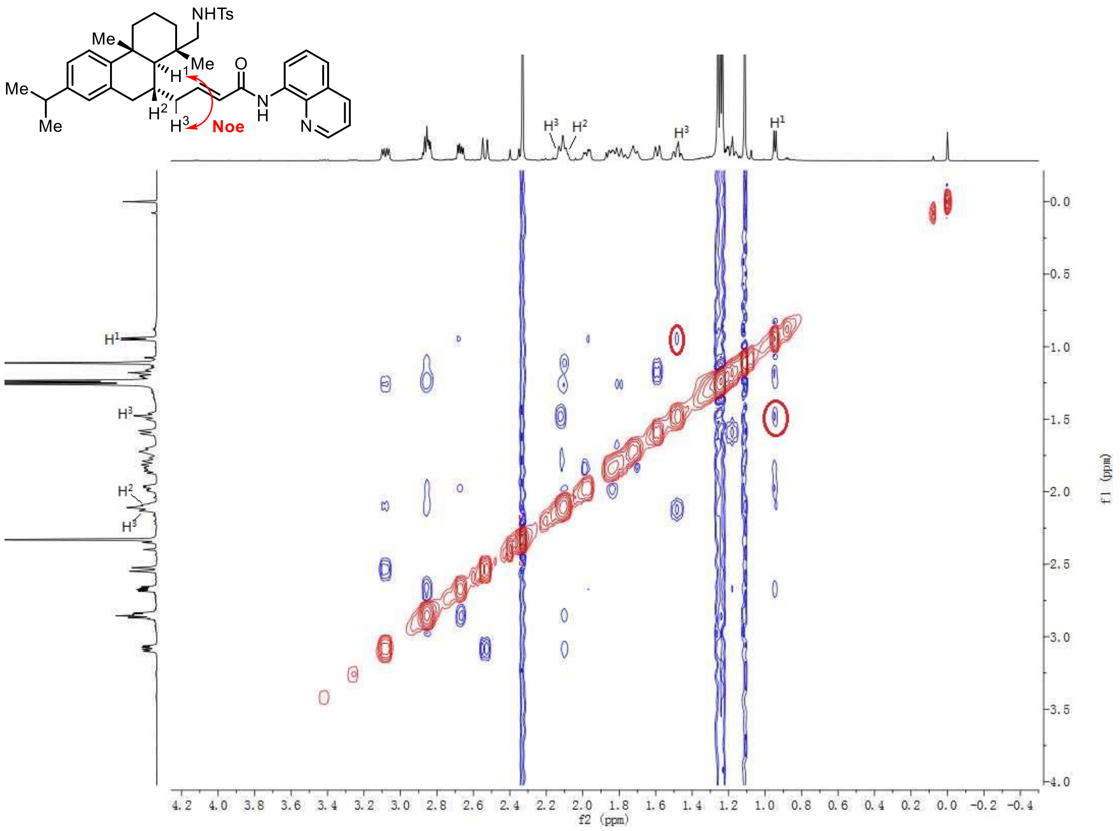

Supplementary Fig. 329. Noesy of compound 4ay. The sample has been recorded in 600 MHz, $CDCl_3$ at 25 °C

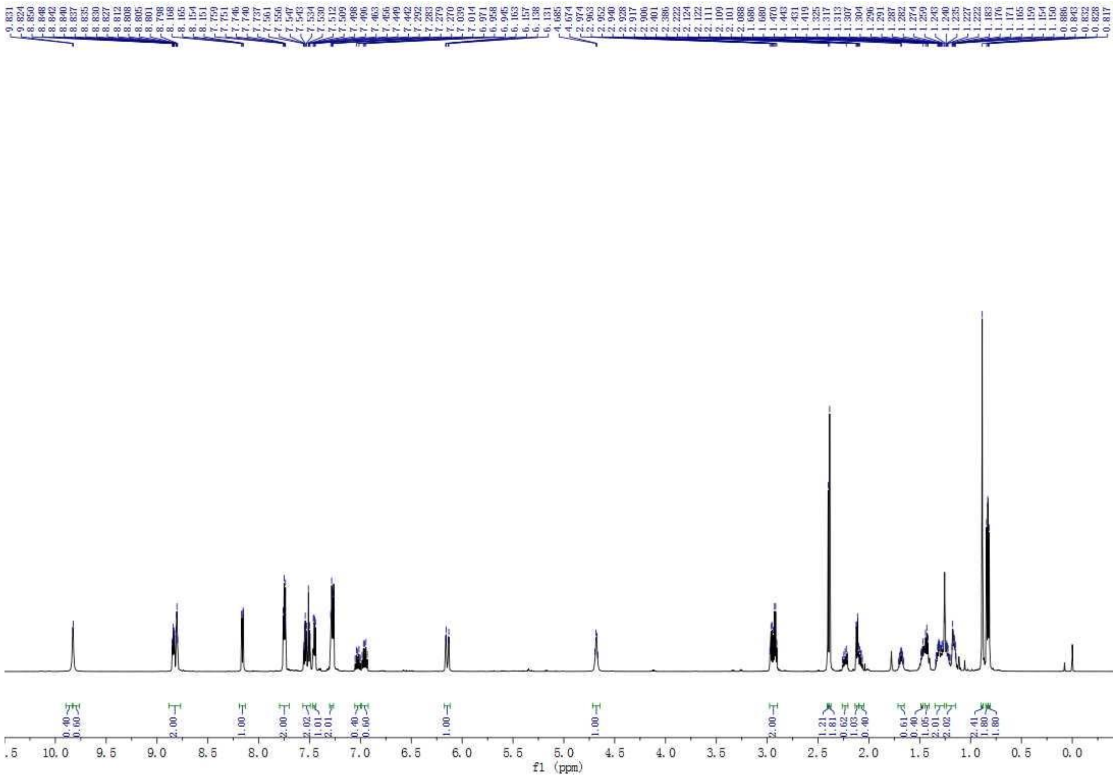

Supplementary Fig. 330. ^{1}H NMR of compound 4az + 4az′ (3:2). The sample has been recorded in 600 MHz, $CDCl_3$ at 25 °C

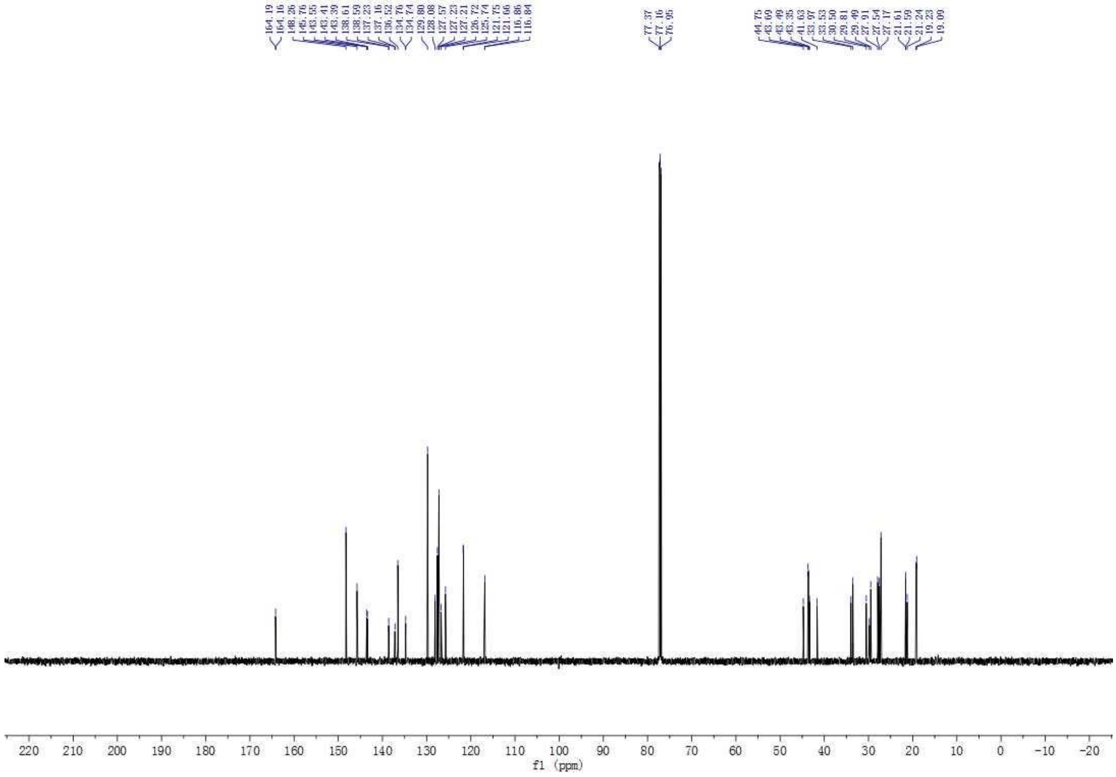

Supplementary Fig. 331. ^{13}C NMR of compound 4az + 4az′ (3:2). The sample has been recorded in 150 MHz, $CDCl_3$ at 25 °C

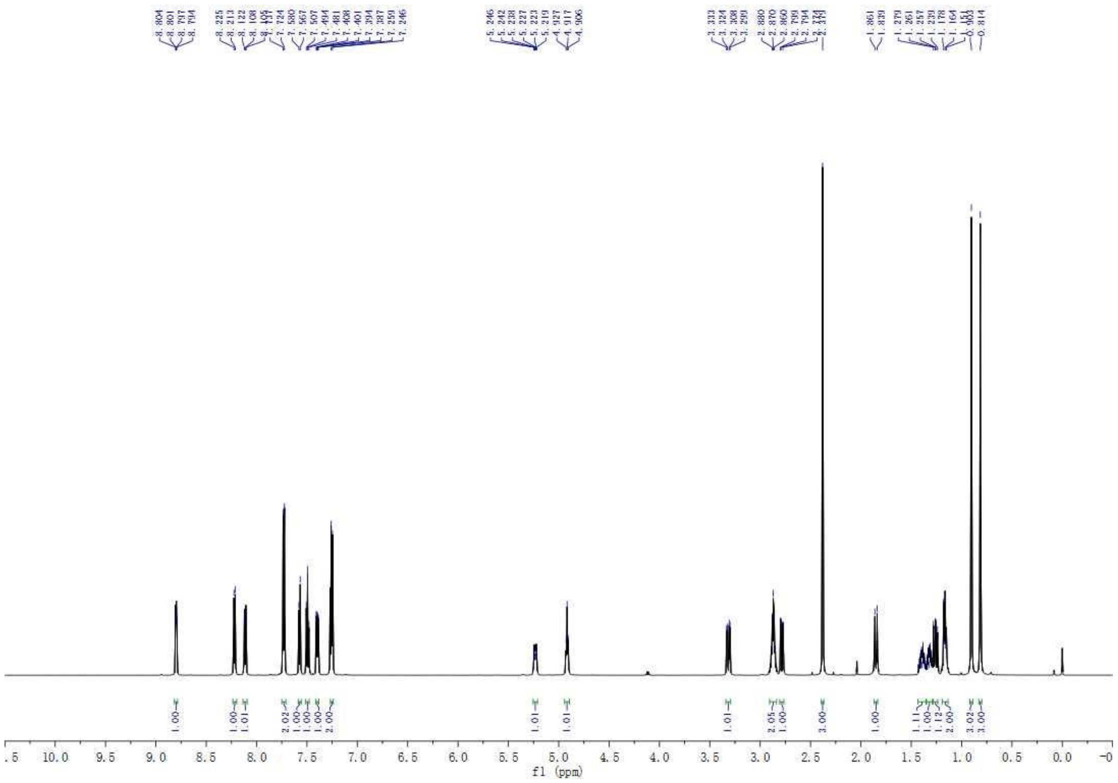

Supplementary Fig. 332. ^{1}H NMR of compound 5a. The sample has been recorded in 600 MHz, $CDCl_3$ at 25 °C

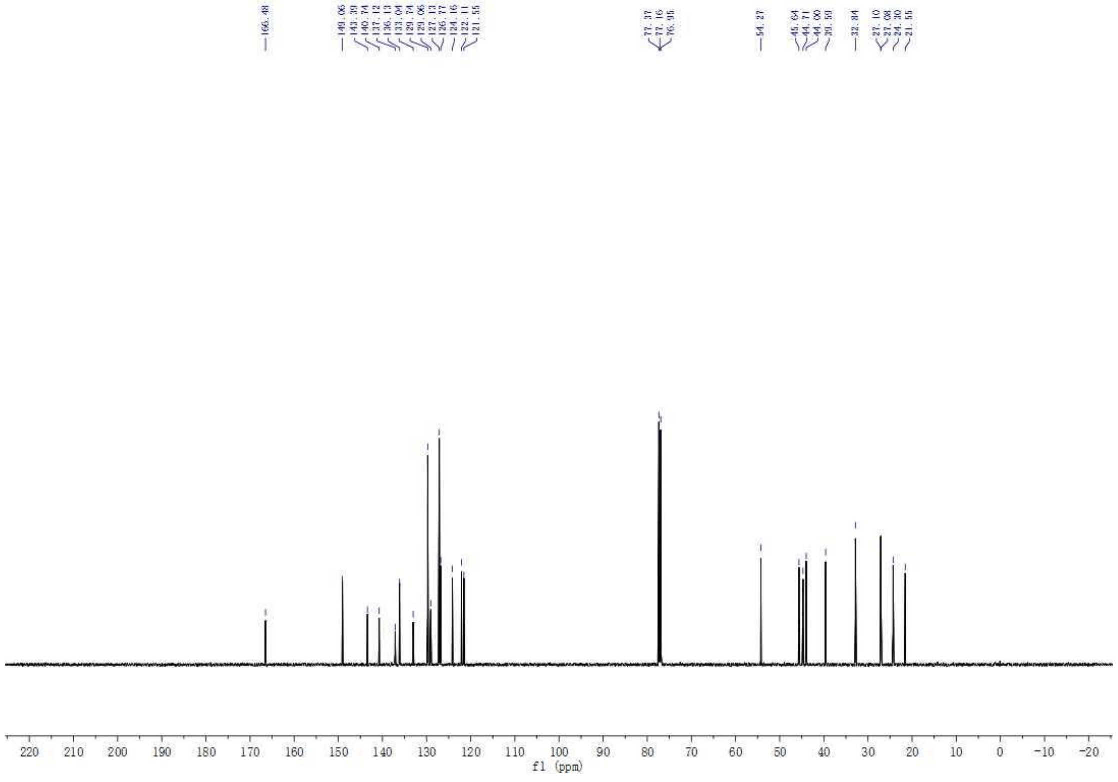

Supplementary Fig. 333. ^{13}C NMR of compound 5a. The sample has been recorded in 150 MHz, $CDCl_3$ at 25 °C

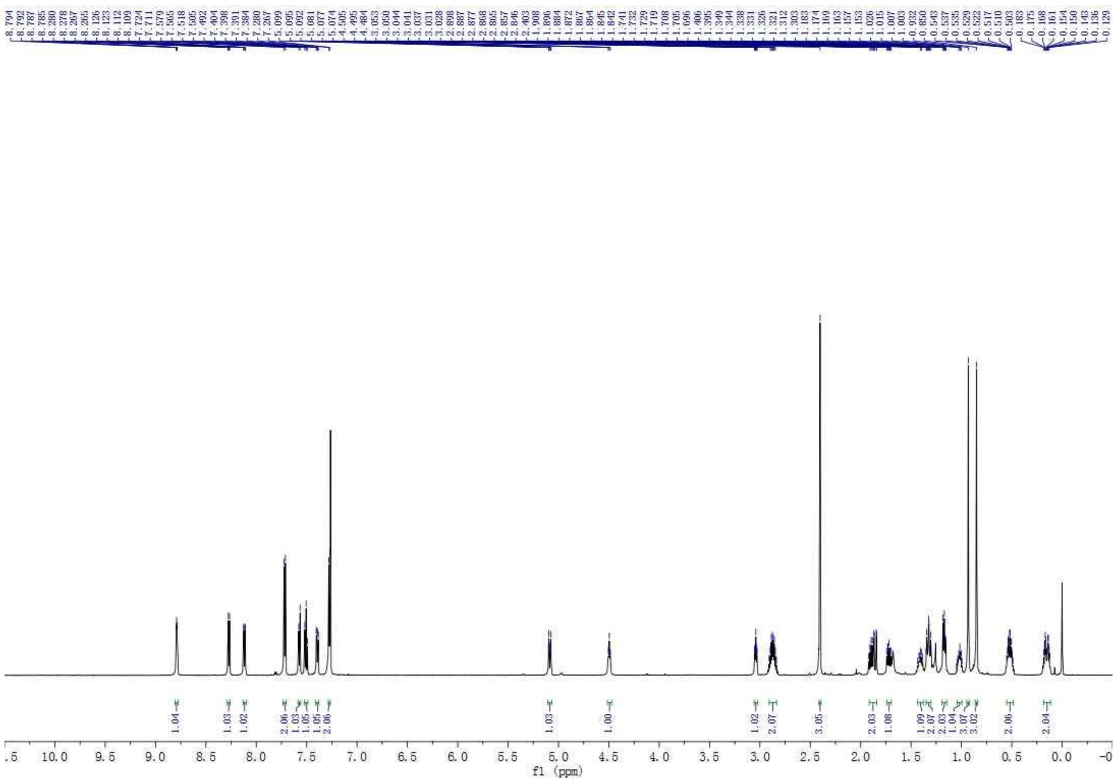

Supplementary Fig. 334. ^{1}H NMR of compound 5b. The sample has been recorded in 600 MHz, $CDCl_3$ at 25 °C

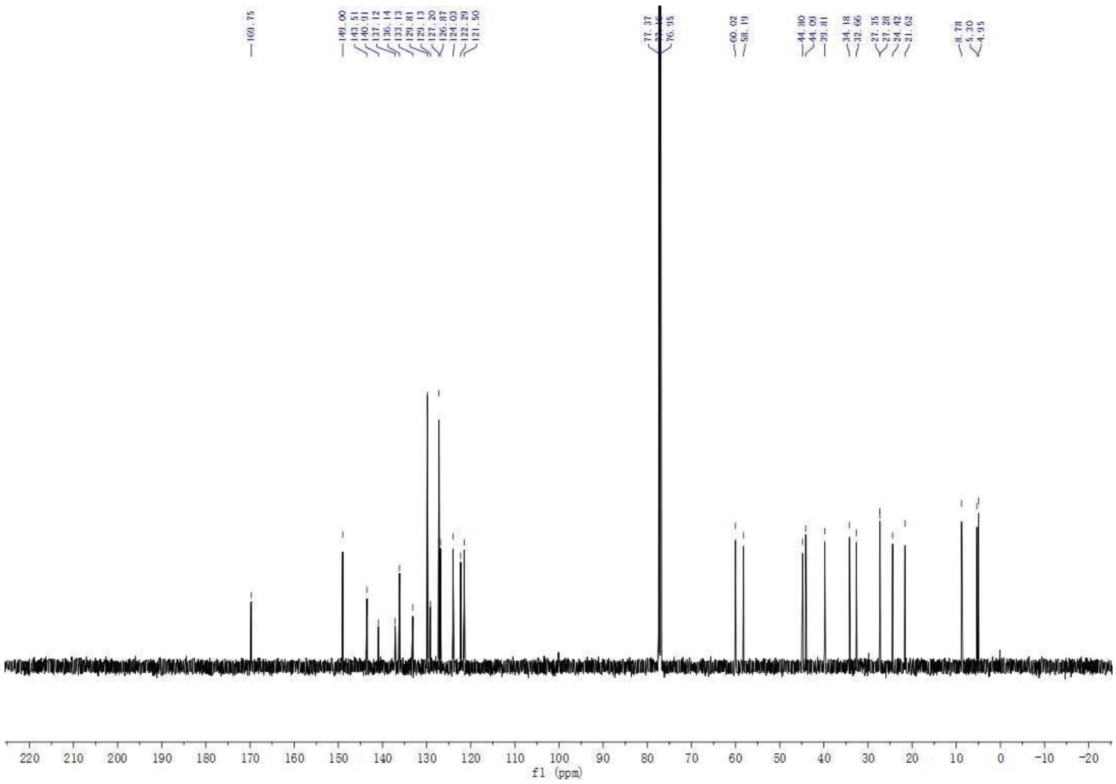

Supplementary Fig. 335. ^{13}C NMR of compound 5b. The sample has been recorded in 150 MHz, $CDCl_3$ at 25 °C

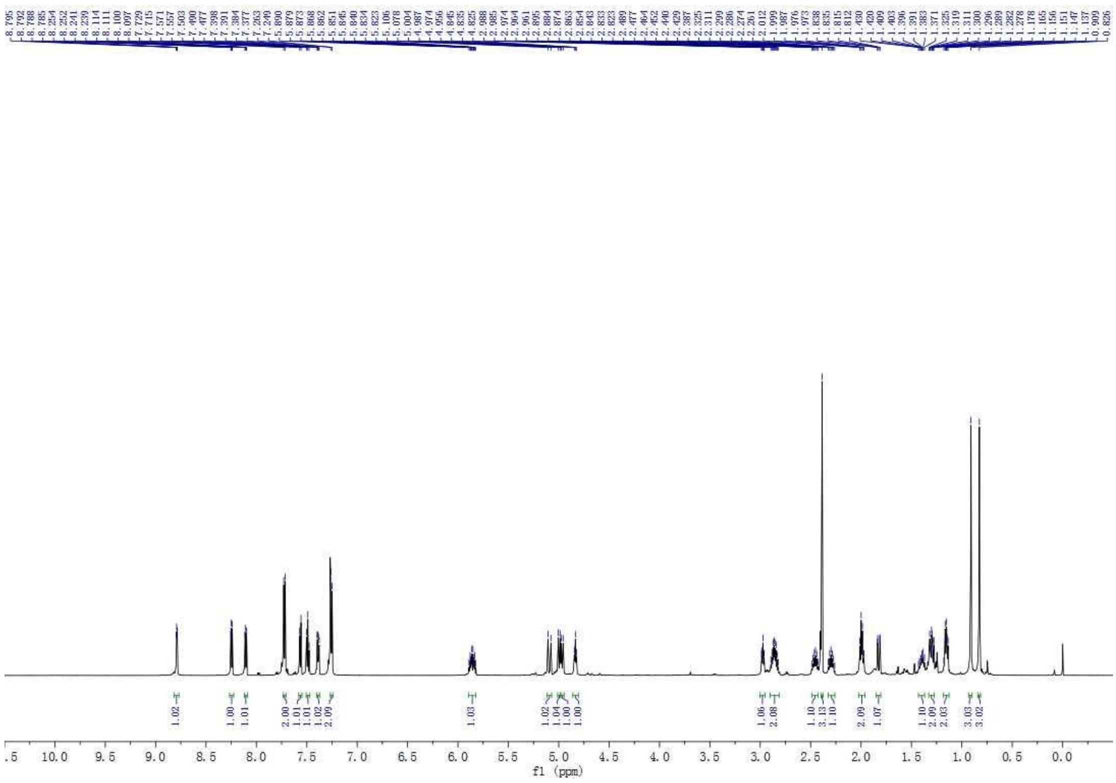

Supplementary Fig. 336. ^{1}H NMR of compound 5c. The sample has been recorded in 600 MHz, $CDCl_3$ at 25 °C

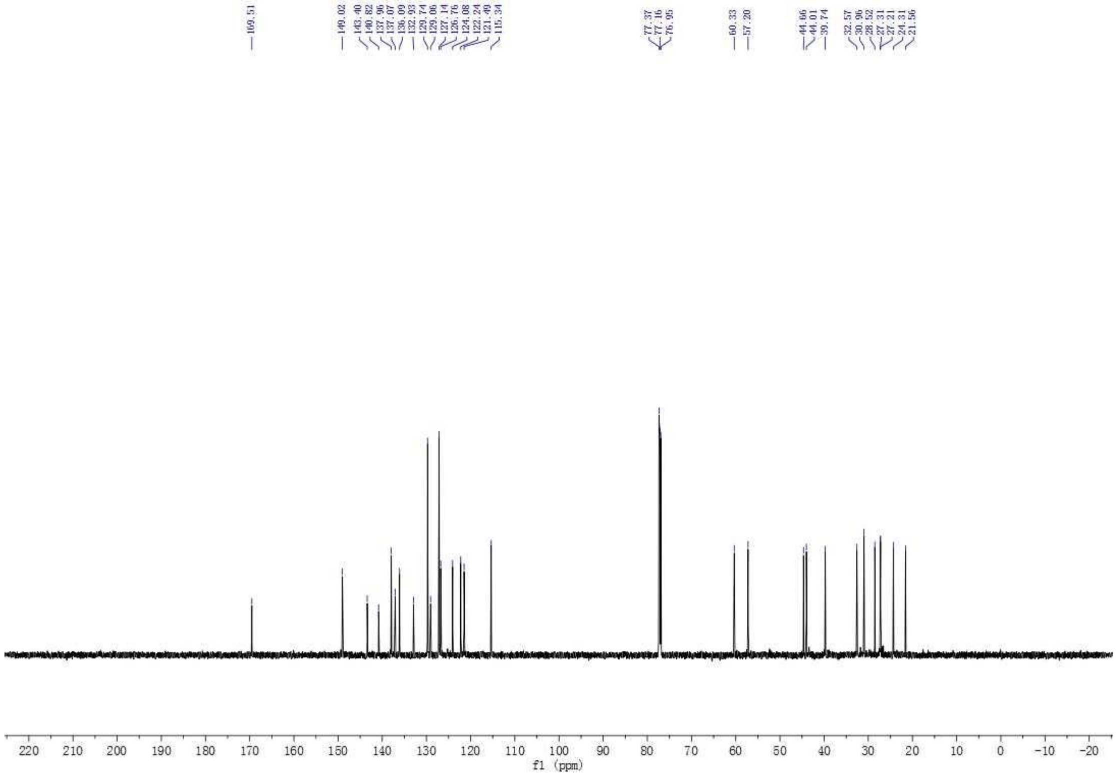

Supplementary Fig. 337. ^{13}C NMR of compound 5c. The sample has been recorded in 150 MHz, $CDCl_3$ at 25 °C

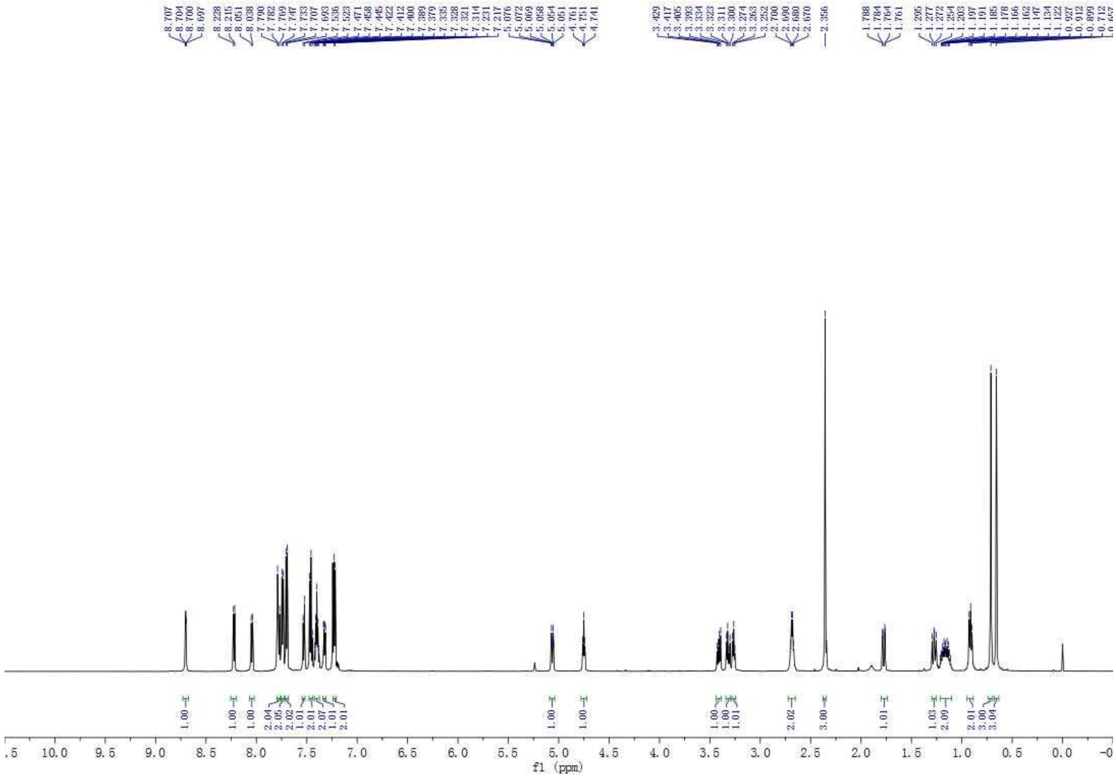

Supplementary Fig. 338. ^{1}H NMR of compound 5d. The sample has been recorded in 600 MHz, $CDCl_3$ at 25 °C

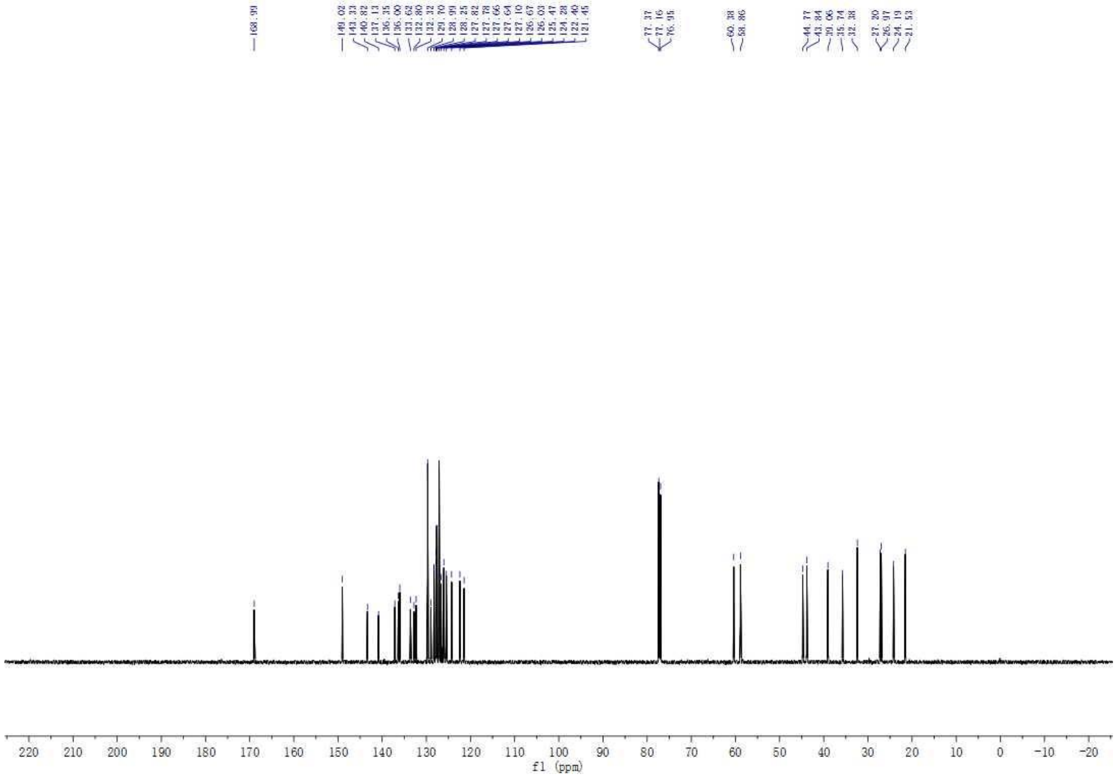

Supplementary Fig. 339. ^{13}C NMR of compound 5d. The sample has been recorded in 150 MHz, $CDCl_3$ at 25 °C

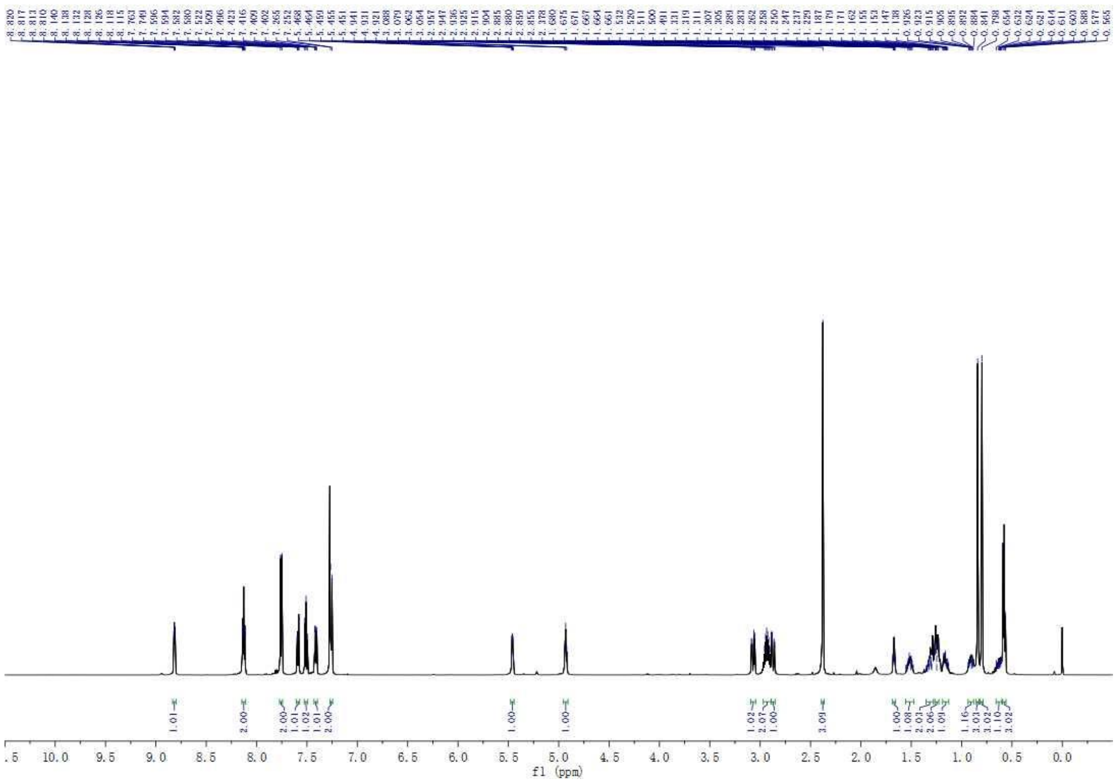

Supplementary Fig. 340. ^{1}H NMR of compound 5e. The sample has been recorded in 600 MHz, $CDCl_3$ at 25 °C

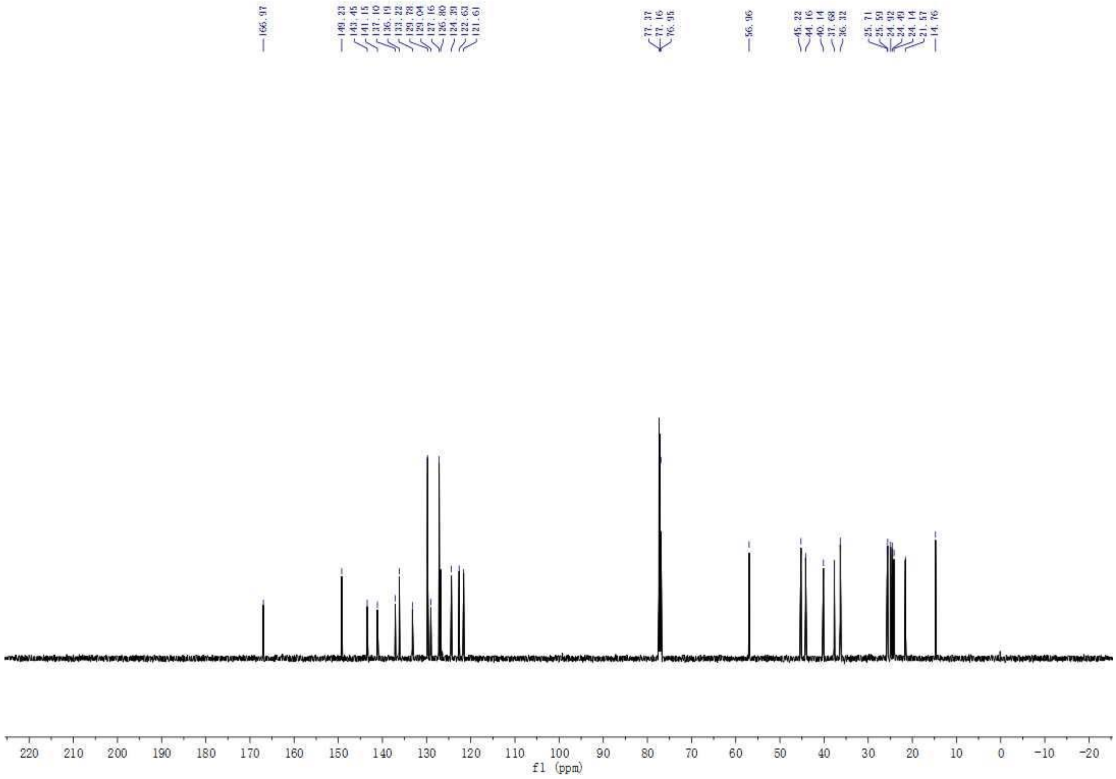

Supplementary Fig. 341. ^{13}C NMR of compound 5e. The sample has been recorded in 150 MHz, $CDCl_3$ at 25 °C

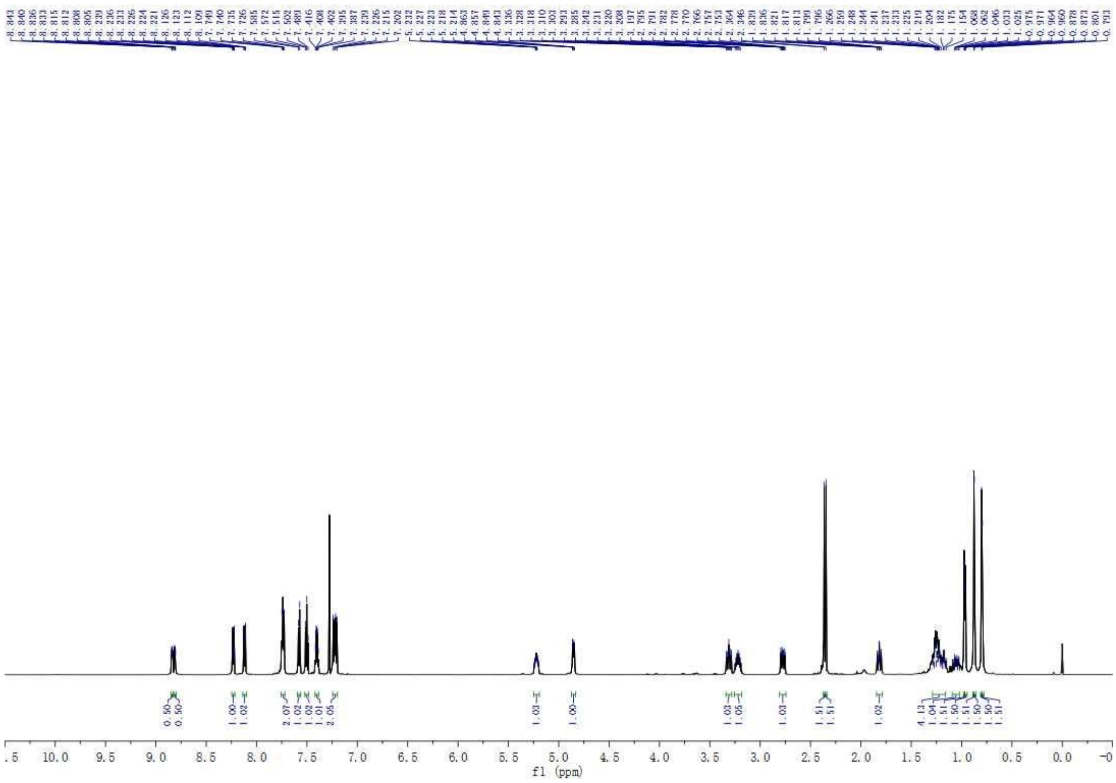

Supplementary Fig. 342. ^{1}H NMR of compound 5f. The sample has been recorded in 600 MHz, $CDCl_3$ at 25 °C

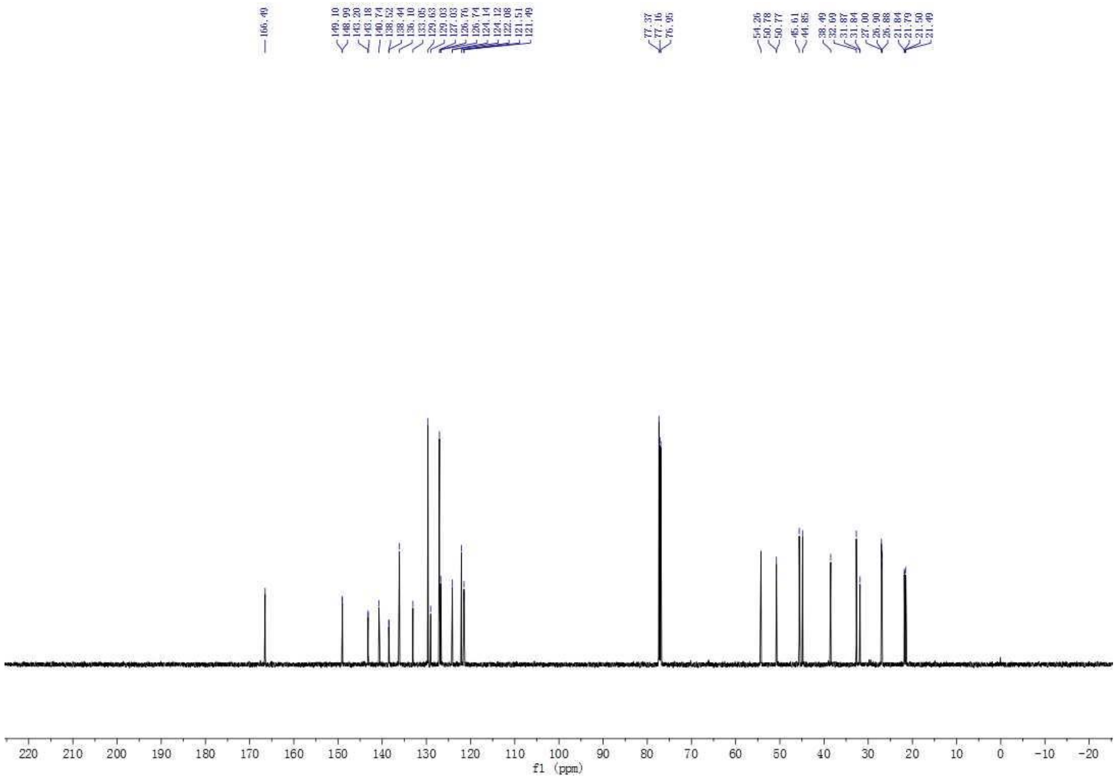

Supplementary Fig. 343. ^{13}C NMR of compound 5f. The sample has been recorded in 150 MHz, $CDCl_3$ at 25 °C

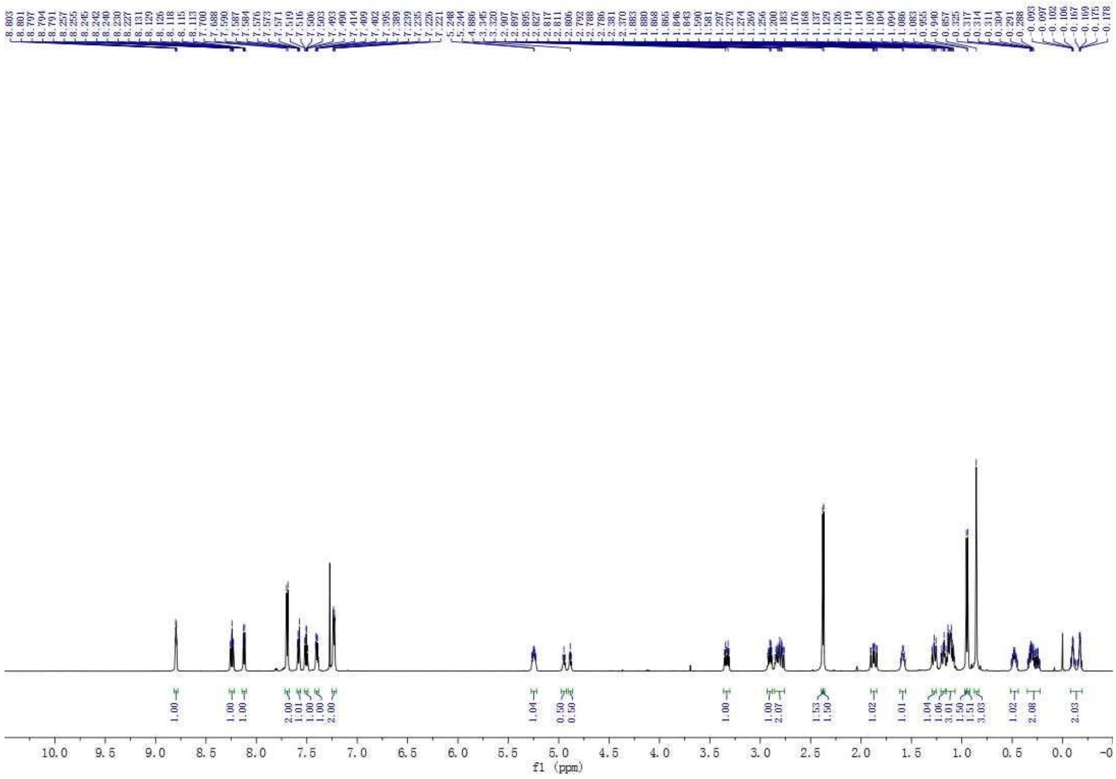

Supplementary Fig. 344. ^{1}H NMR of compound 5g. The sample has been recorded in 600 MHz, $CDCl_3$ at 25 °C

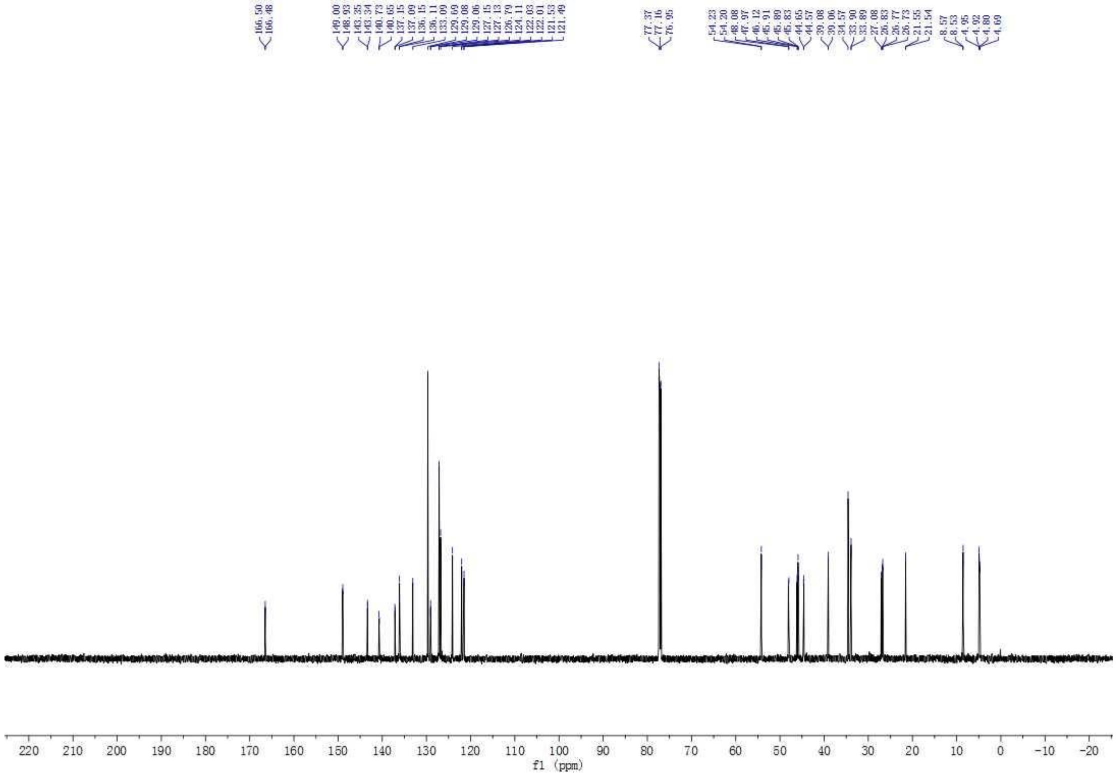

Supplementary Fig. 345. ^{13}C NMR of compound 5g. The sample has been recorded in 150 MHz, $CDCl_3$ at 25 °C

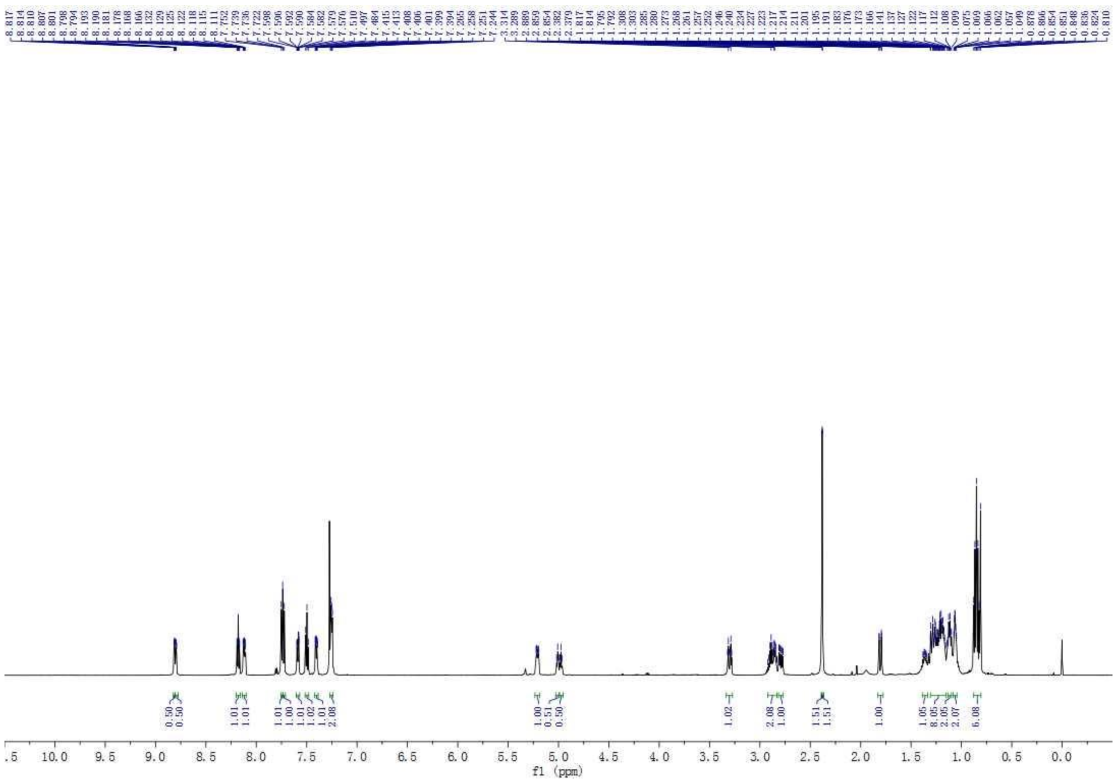

Supplementary Fig. 346. ^{1}H NMR of compound 5h. The sample has been recorded in 600 MHz, $CDCl_3$ at 25 °C

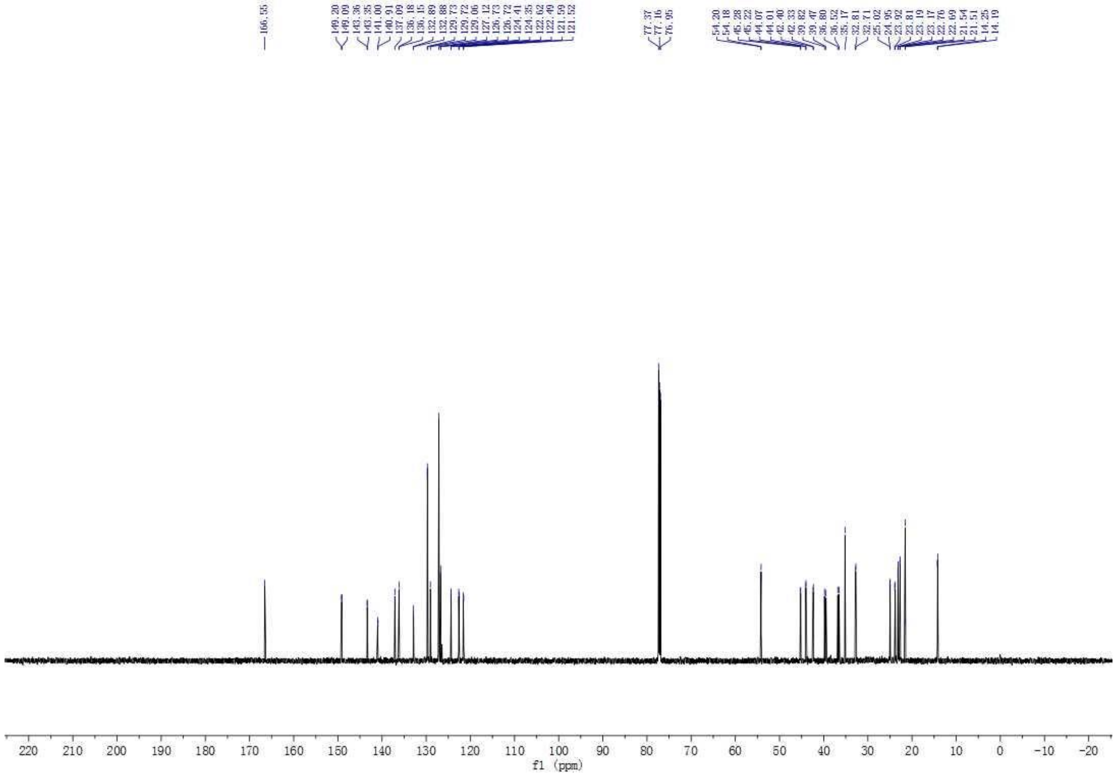

Supplementary Fig. 347. ^{13}C NMR of compound 5h. The sample has been recorded in 150 MHz, $CDCl_3$ at 25 °C

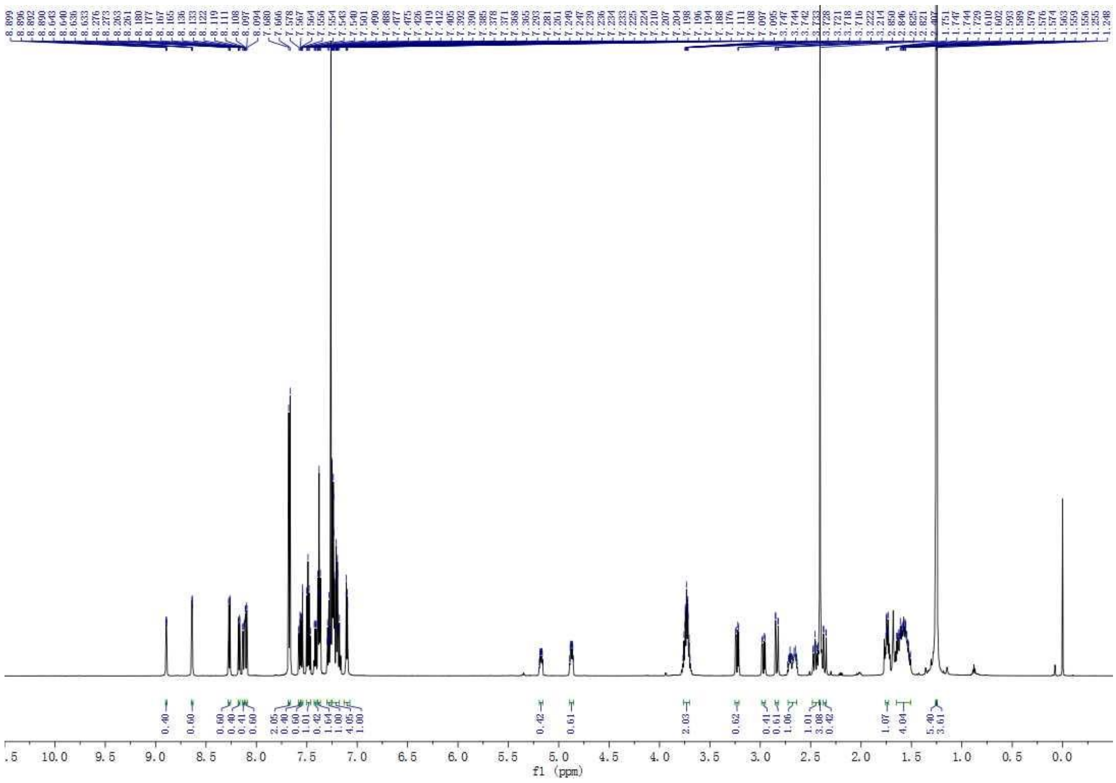

Supplementary Fig. 348. ^{1}H NMR of compound 5i. The sample has been recorded in 600 MHz, $CDCl_3$ at 25 °C

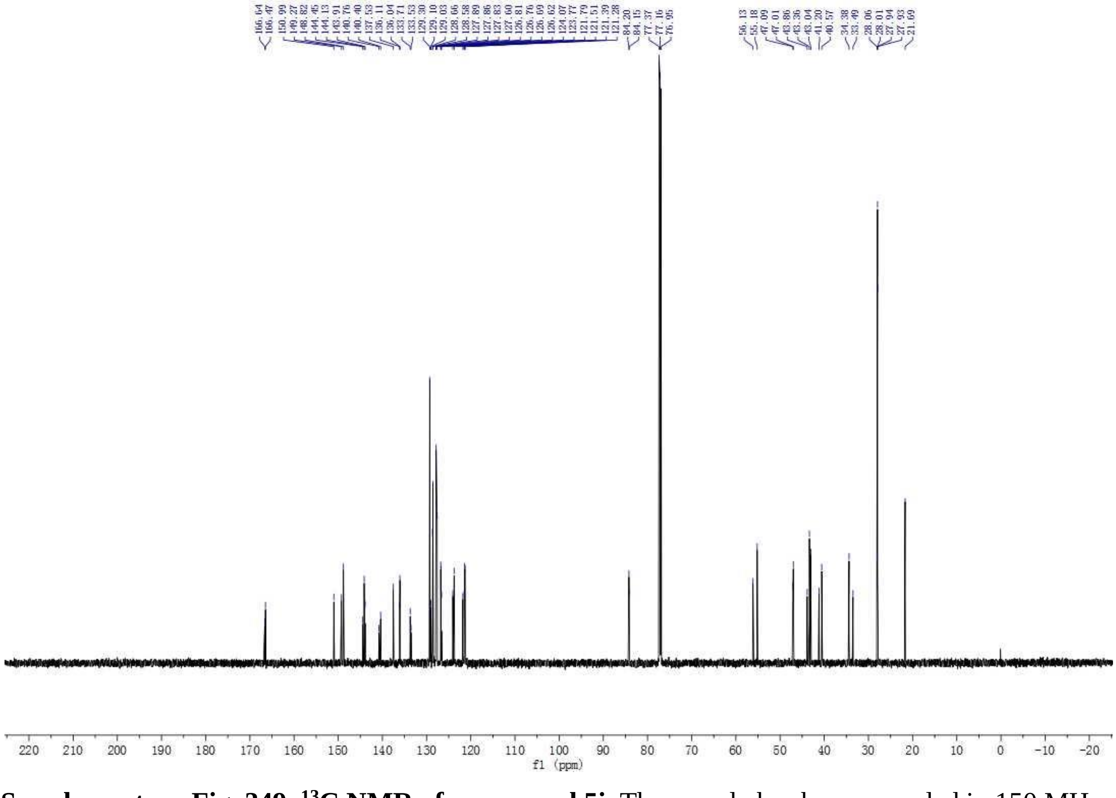

Supplementary Fig. 349. ^{13}C NMR of compound 5i. The sample has been recorded in 150 MHz, $CDCl_3$ at 25 °C

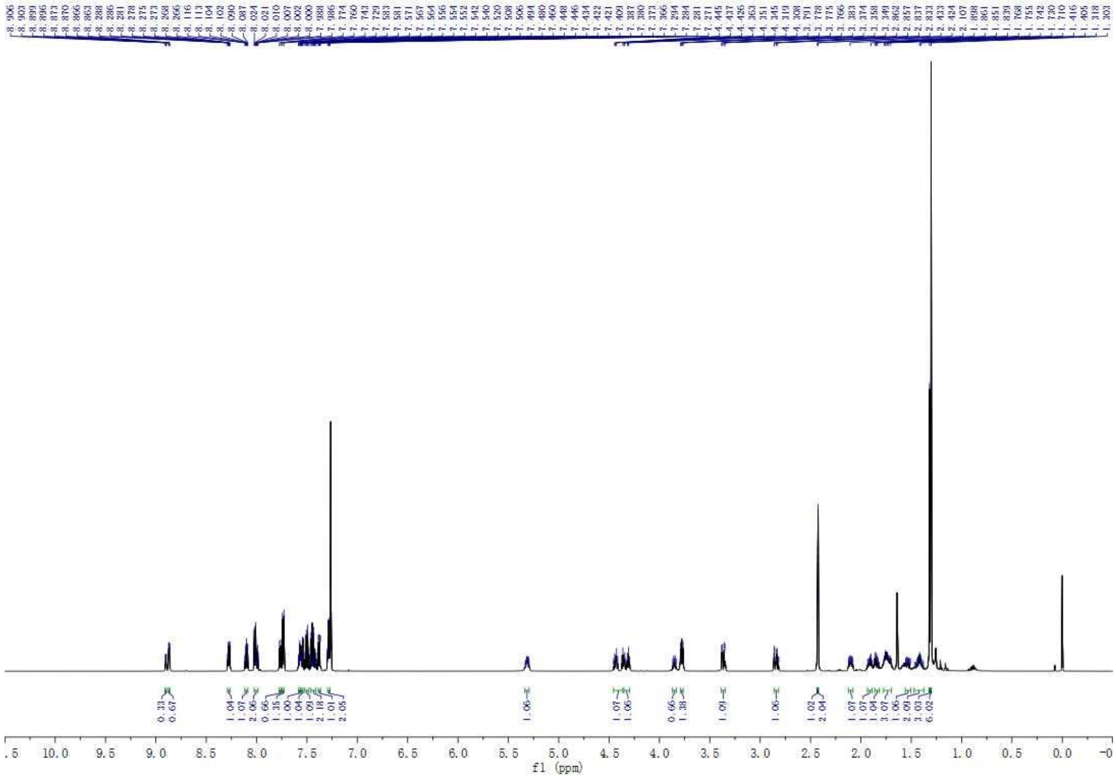

Supplementary Fig. 350. ^{1}H NMR of compound 5j. The sample has been recorded in 600 MHz, $CDCl_3$ at 25 °C

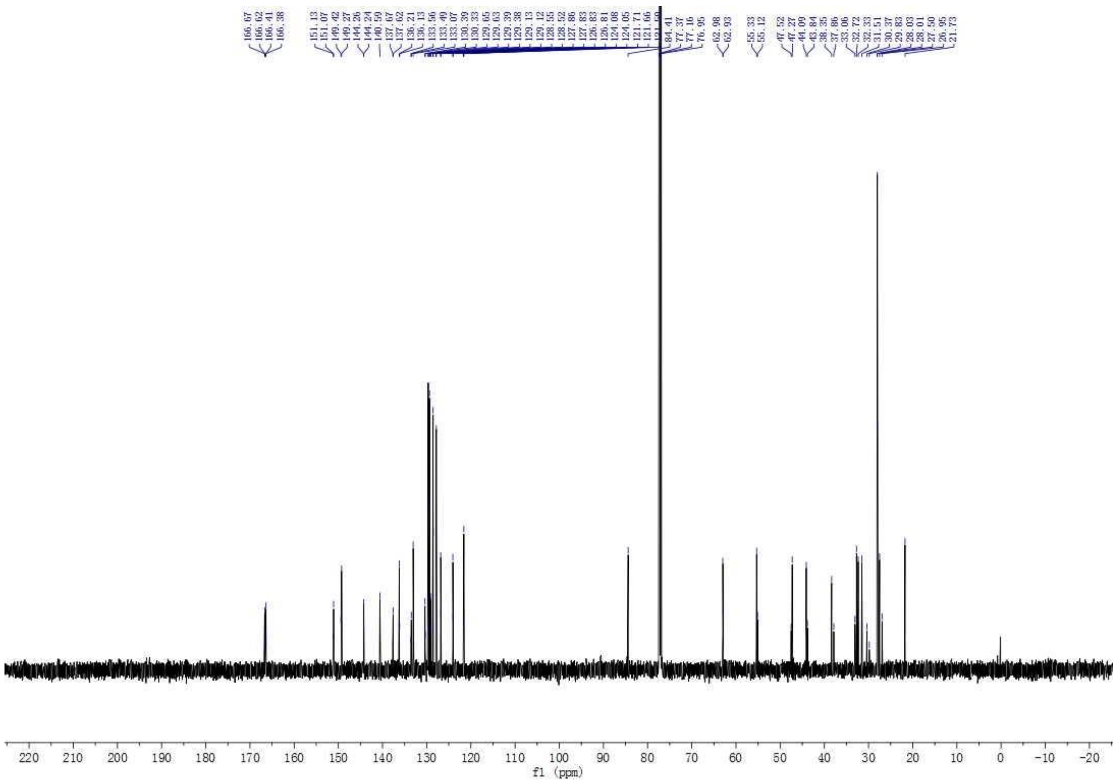

Supplementary Fig. 351. ^{13}C NMR of compound 5j. The sample has been recorded in 150 MHz, $CDCl_3$ at 25 °C

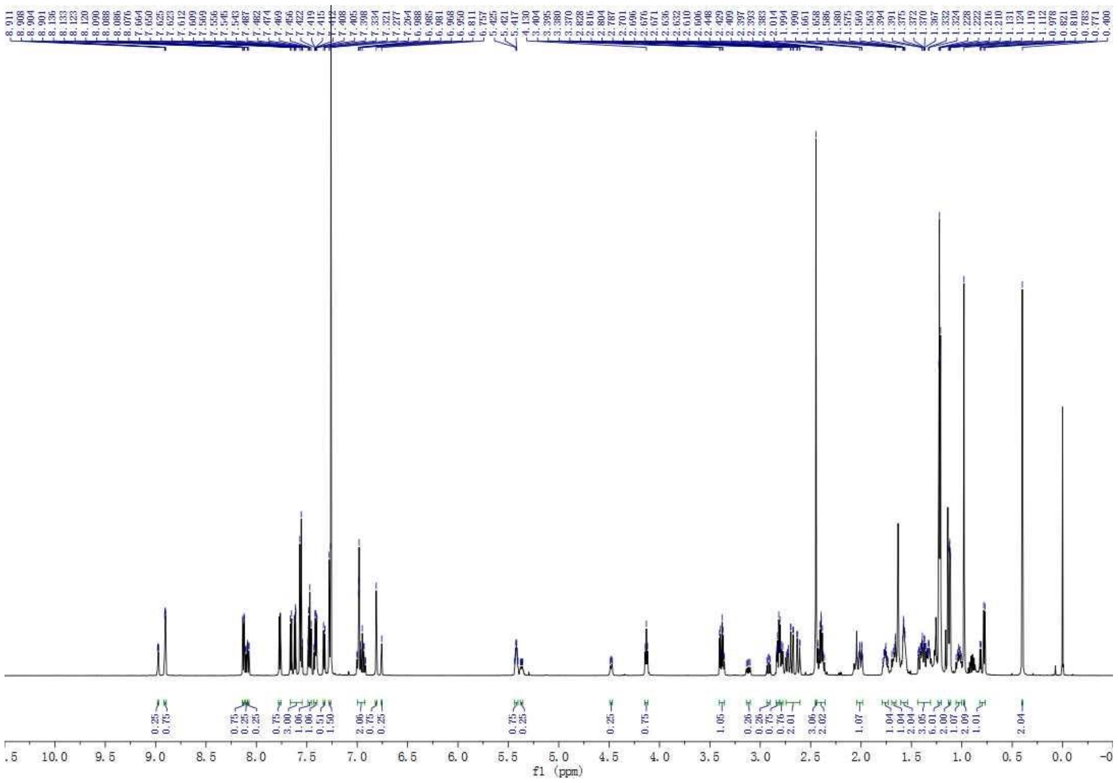

Supplementary Fig. 352. ^{1}H NMR of compound 5k. The sample has been recorded in 600 MHz, $CDCl_3$ at 25 °C

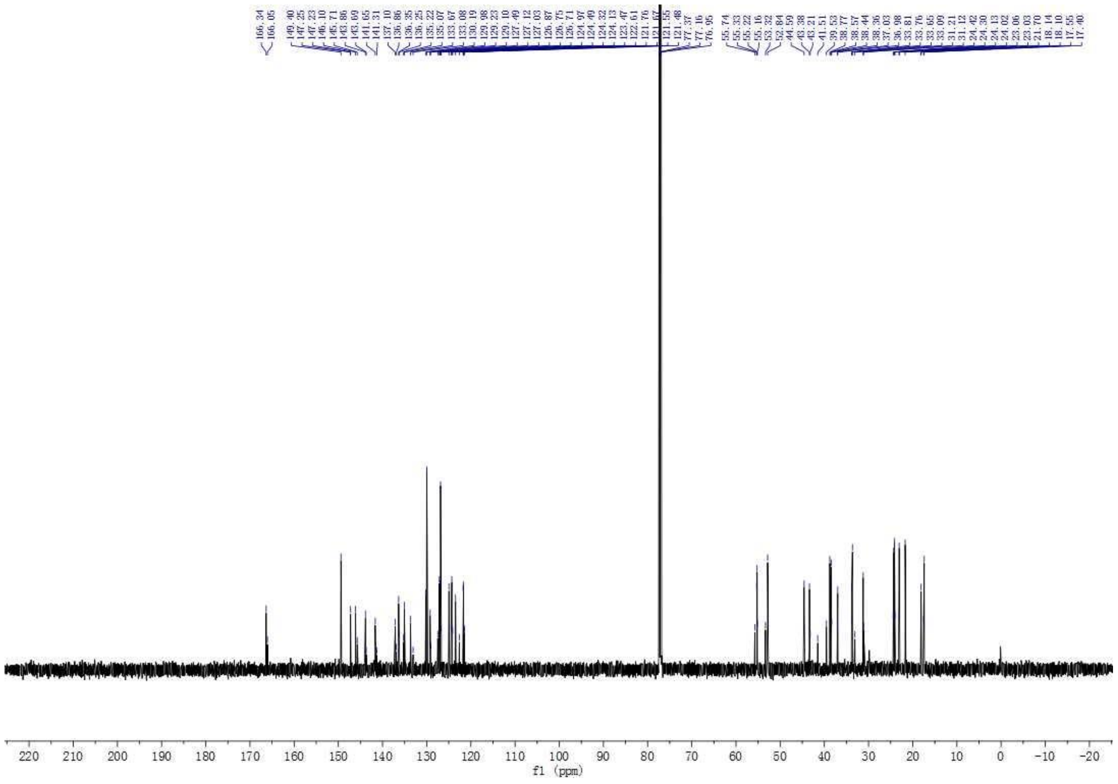

Supplementary Fig. 353. ^{13}C NMR of compound 5k. The sample has been recorded in 150 MHz, $CDCl_3$ at 25 °C

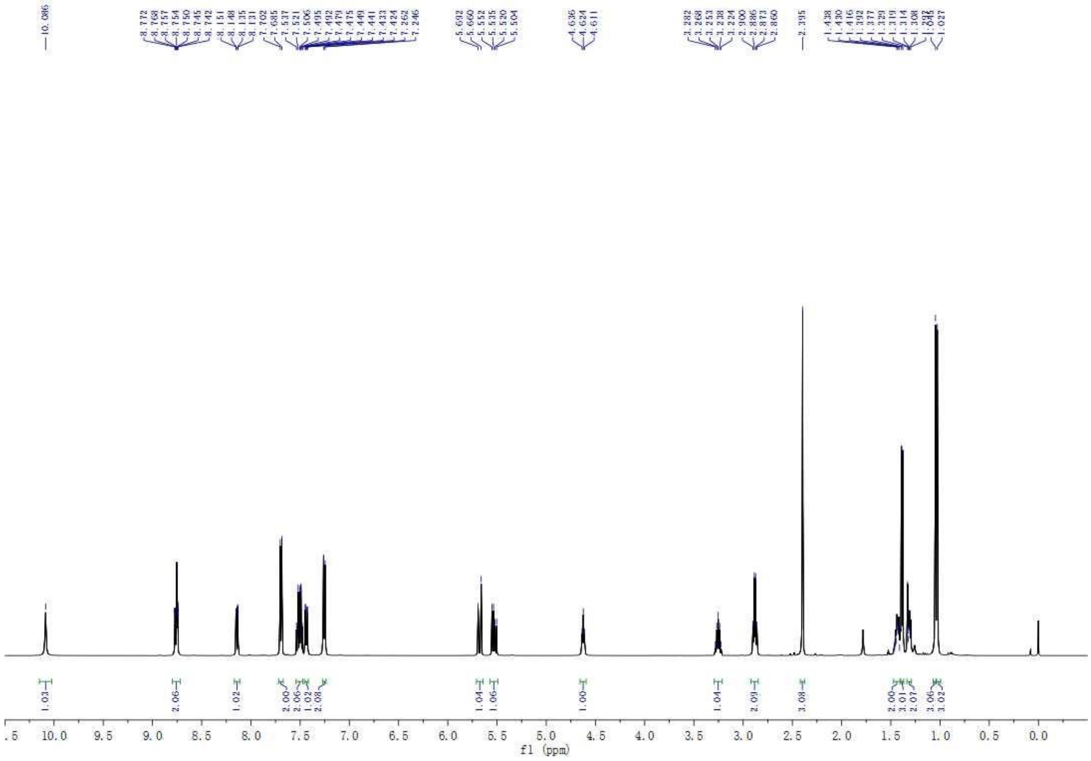

Supplementary Fig. 354. ^{1}H NMR of compound 6a. The sample has been recorded in 500 MHz, $CDCl_3$ at 25 °C

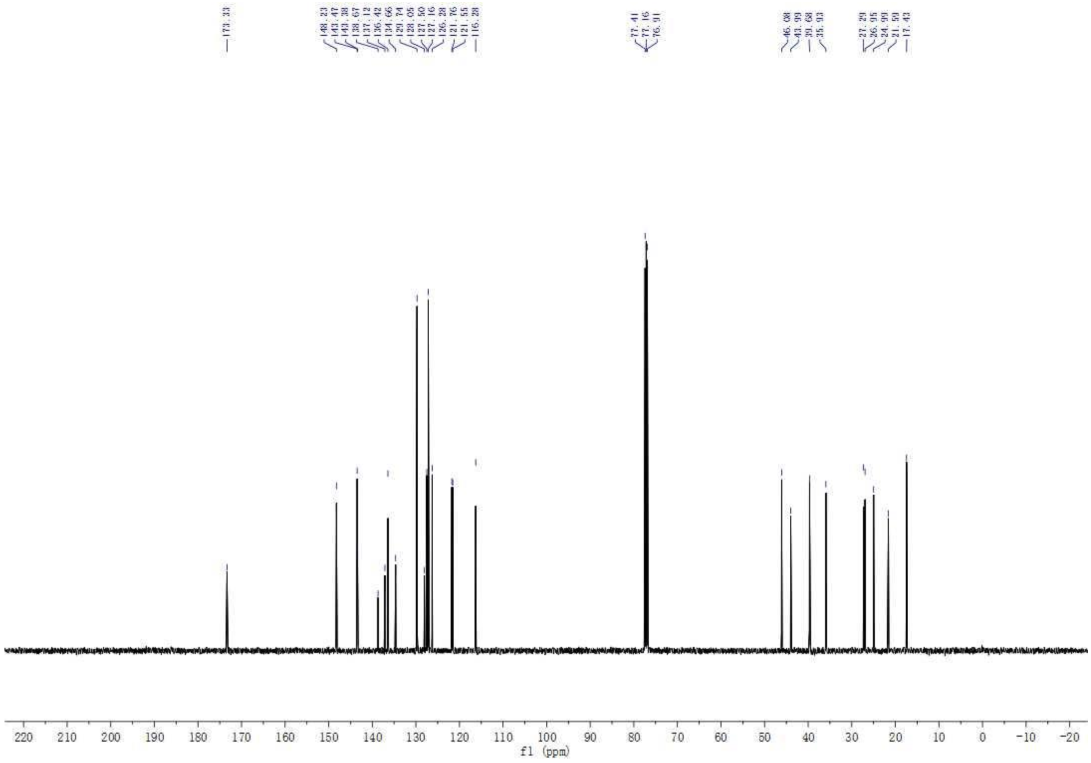

Supplementary Fig. 355. ^{13}C NMR of compound 6a. The sample has been recorded in 125 MHz, $CDCl_3$ at 25 °C

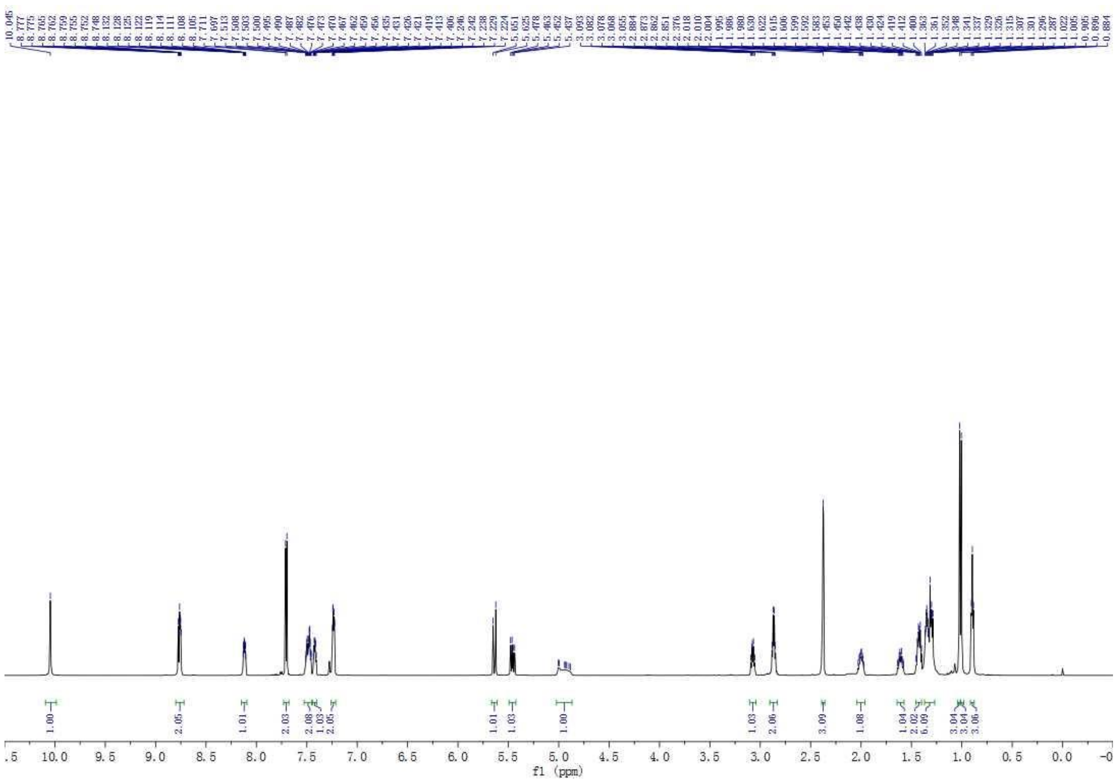

Supplementary Fig. 356. ^{1}H NMR of compound 6b. The sample has been recorded in 600 MHz, $CDCl_3$ at 25 °C

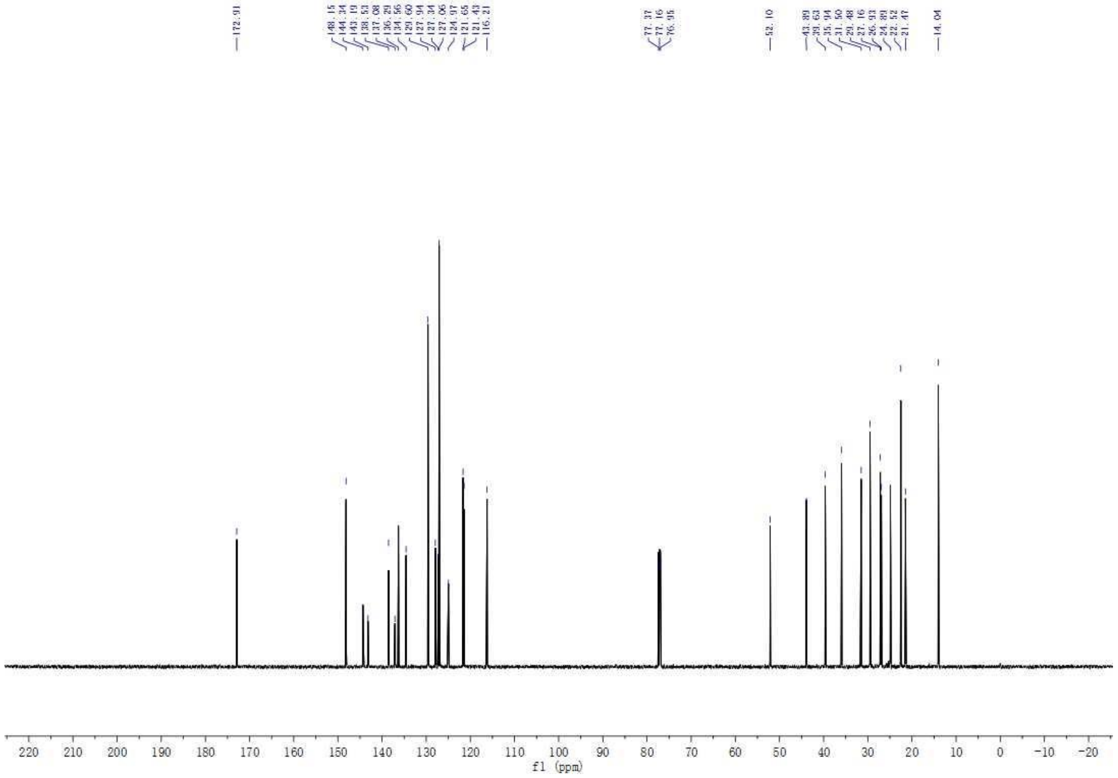

Supplementary Fig. 357. ^{13}C NMR of compound 6b. The sample has been recorded in 150 MHz, $CDCl_3$ at 25 °C

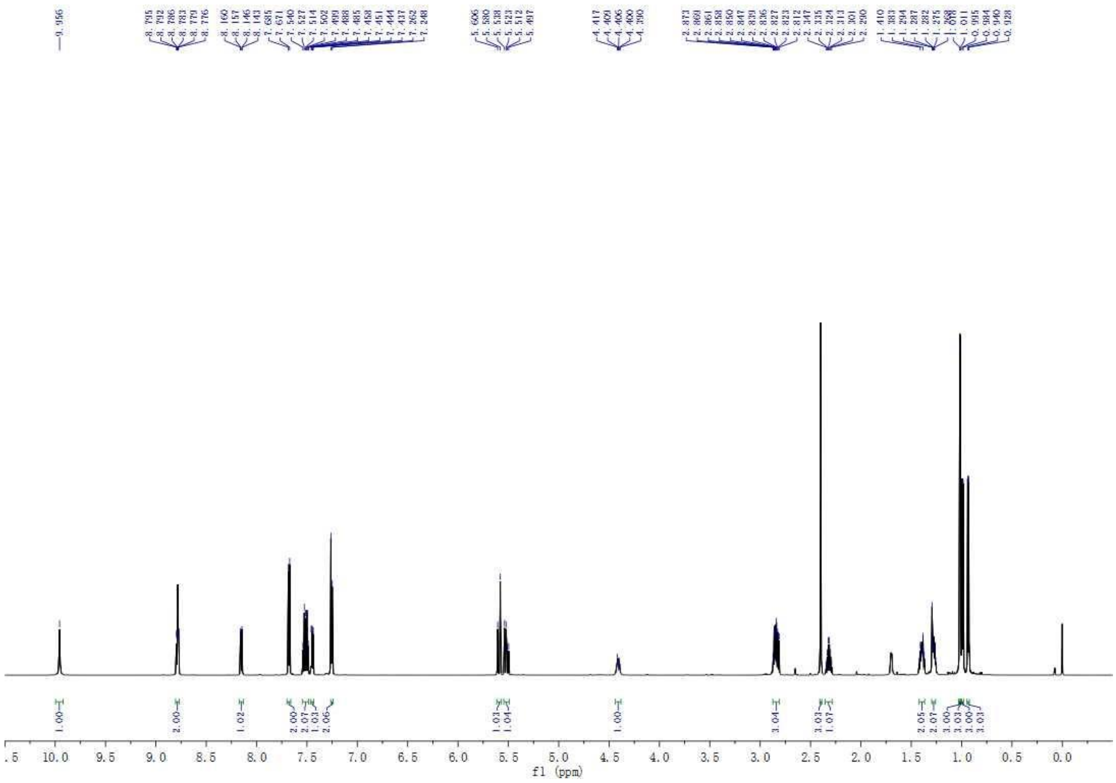

Supplementary Fig. 358. ¹H NMR of compound 6c. The sample has been recorded in 600 MHz, $CDCl_3$ at 25 °C

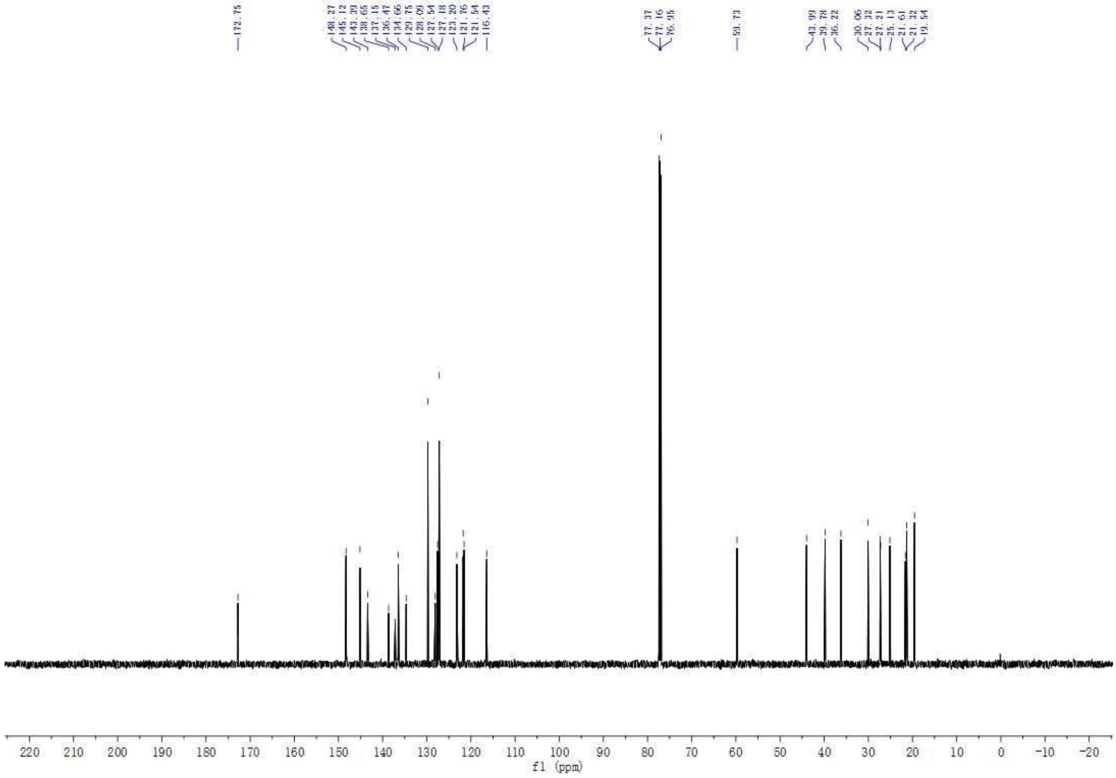

Supplementary Fig. 359. ¹³C NMR of compound 6c. The sample has been recorded in 150 MHz, $CDCl_3$ at 25 °C

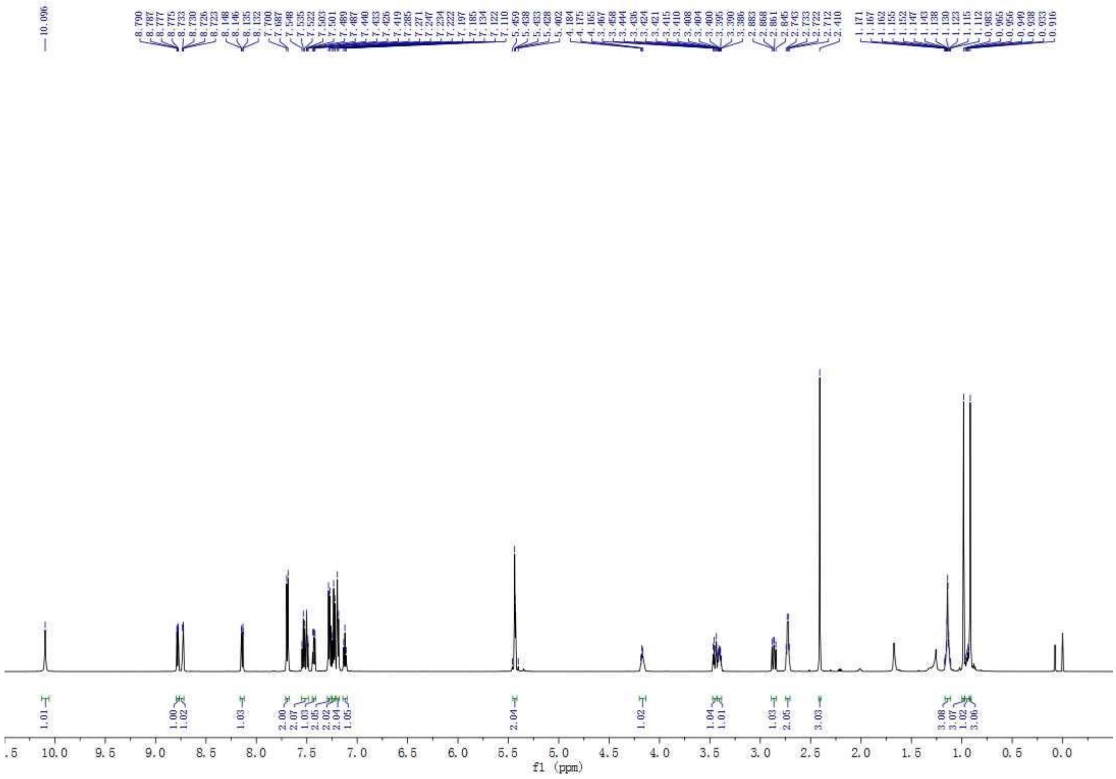

Supplementary Fig. 360. ^{1}H NMR of compound 6d. The sample has been recorded in 600 MHz, $CDCl_3$ at 25 °C

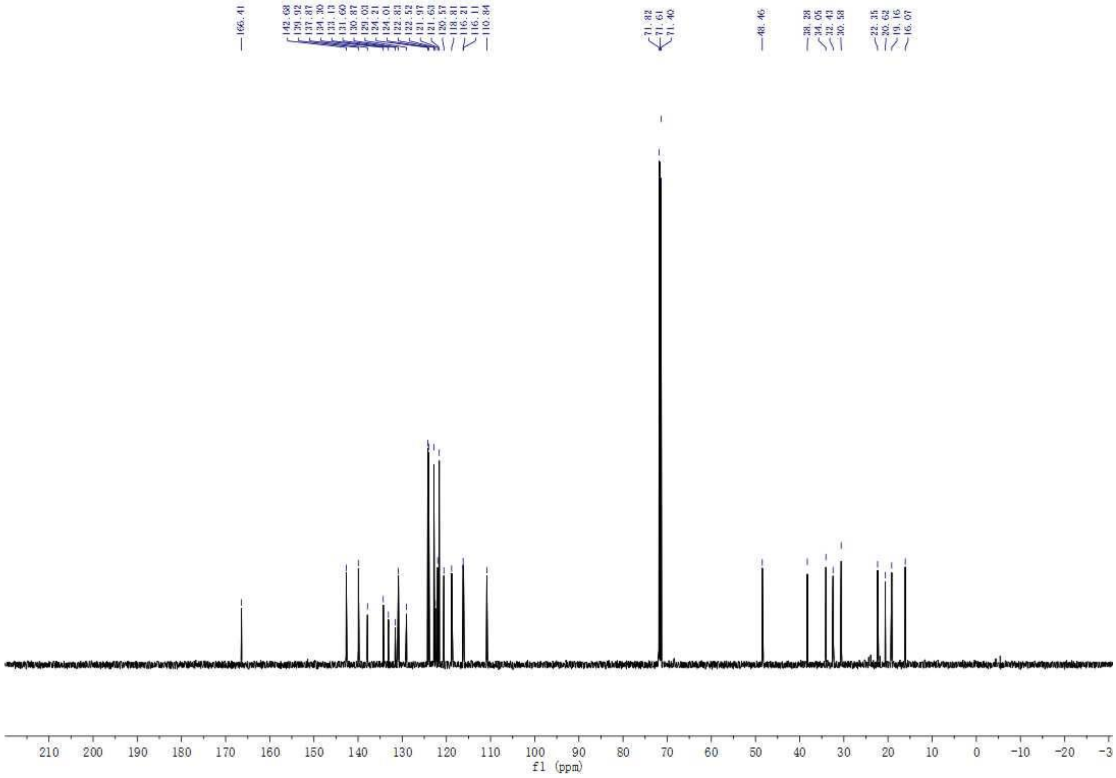

Supplementary Fig. 361. ^{13}C NMR of compound 6d. The sample has been recorded in 150 MHz, $CDCl_3$ at 25 °C

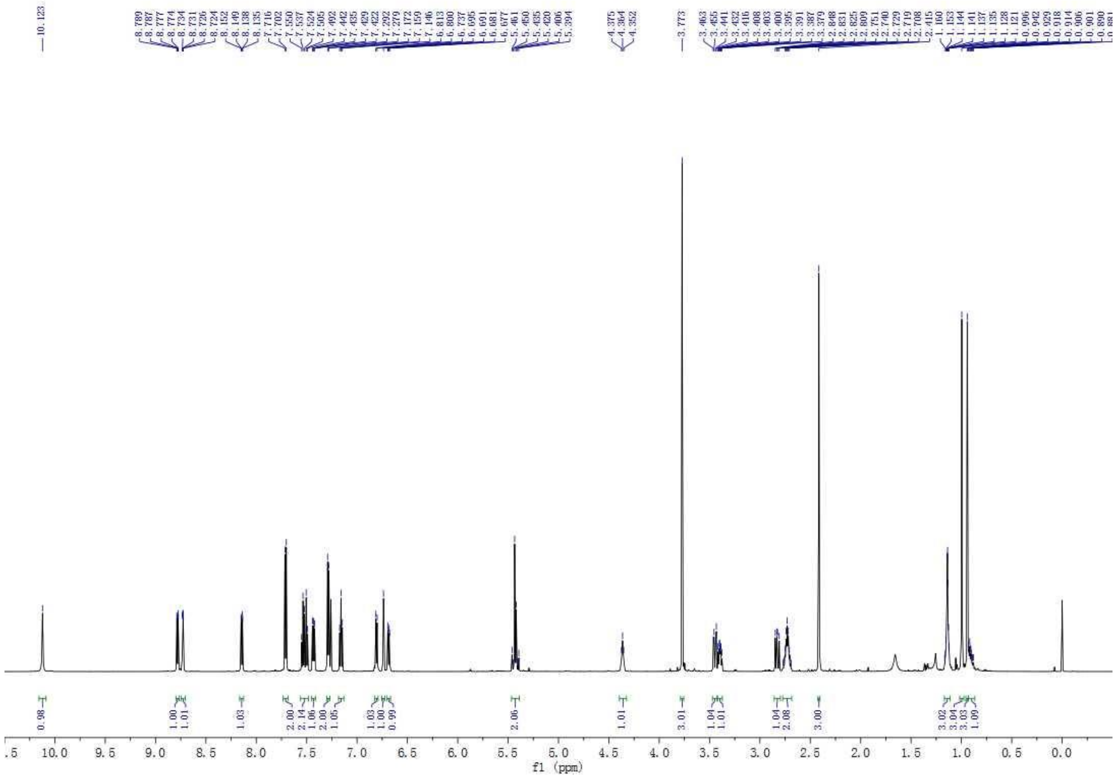

Supplementary Fig. 362. ^{1}H NMR of compound 6e. The sample has been recorded in 600 MHz, $CDCl_3$ at 25 °C

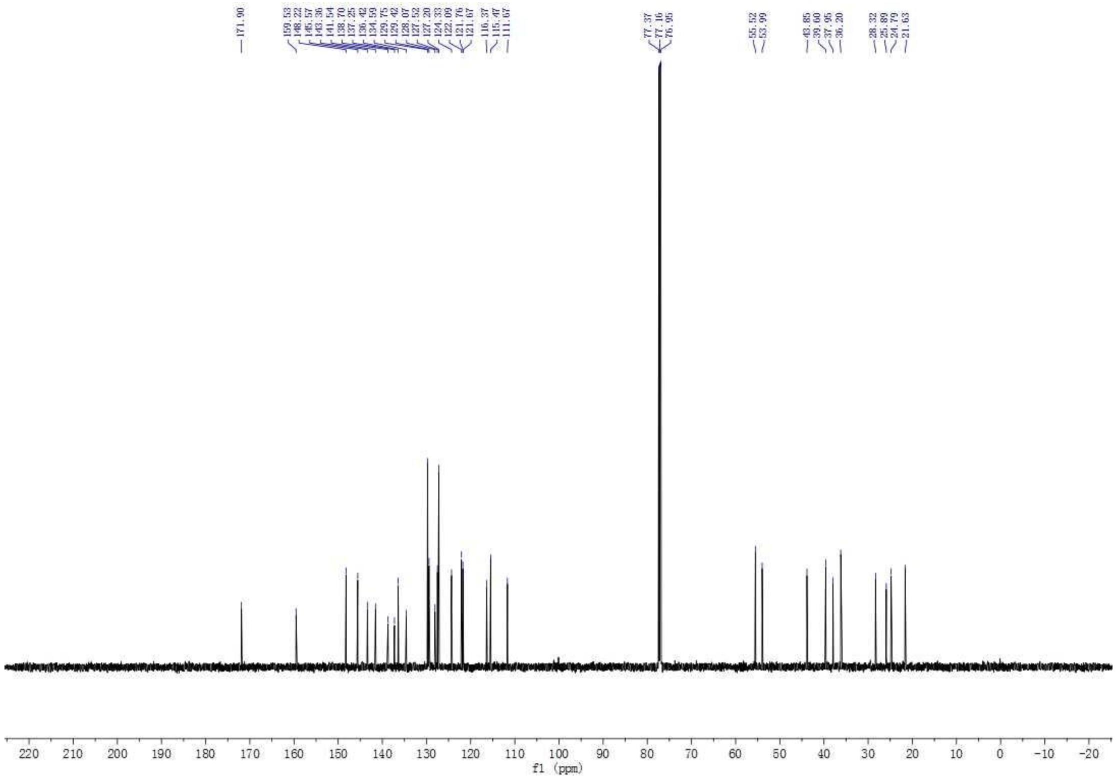

Supplementary Fig. 363. ^{13}C NMR of compound 6e. The sample has been recorded in 150 MHz, $CDCl_3$ at 25 °C

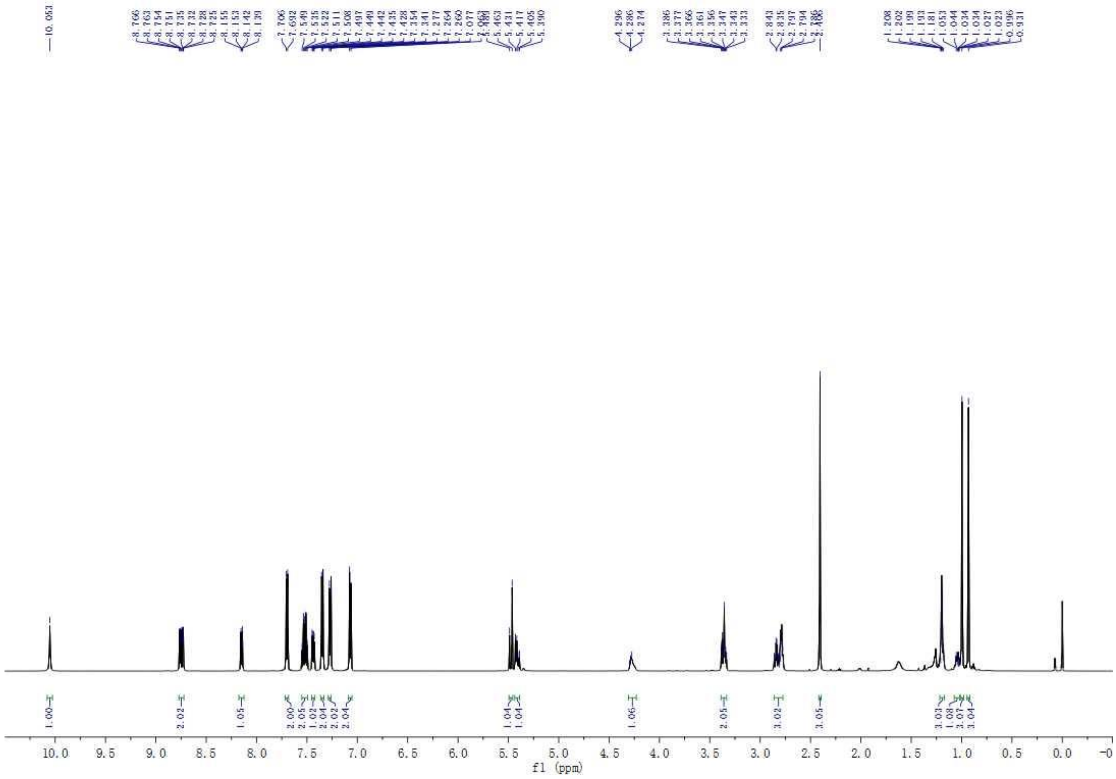

Supplementary Fig. 364. ^{1}H NMR of compound 6f. The sample has been recorded in 600 MHz, $CDCl_3$ at 25 °C

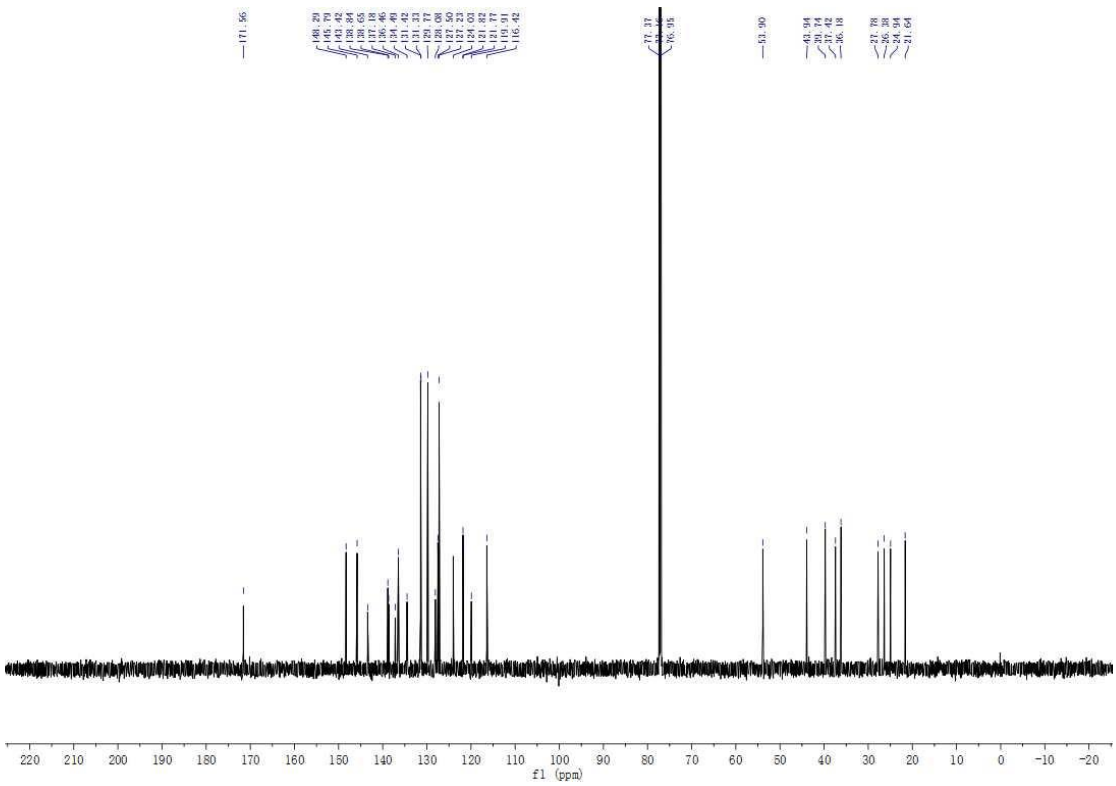

Supplementary Fig. 365. ^{13}C NMR of compound 6f. The sample has been recorded in 150 MHz, $CDCl_3$ at 25 °C

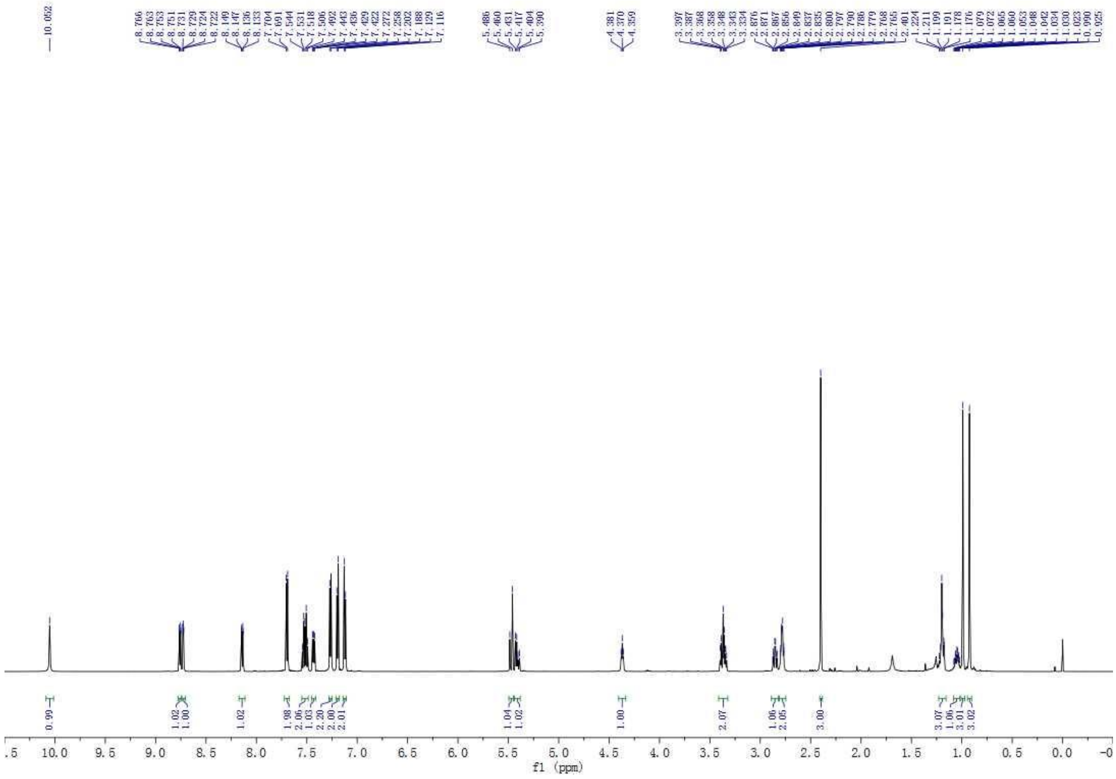

Supplementary Fig. 366. ^{1}H NMR of compound 6g. The sample has been recorded in 600 MHz, $CDCl_3$ at 25 °C

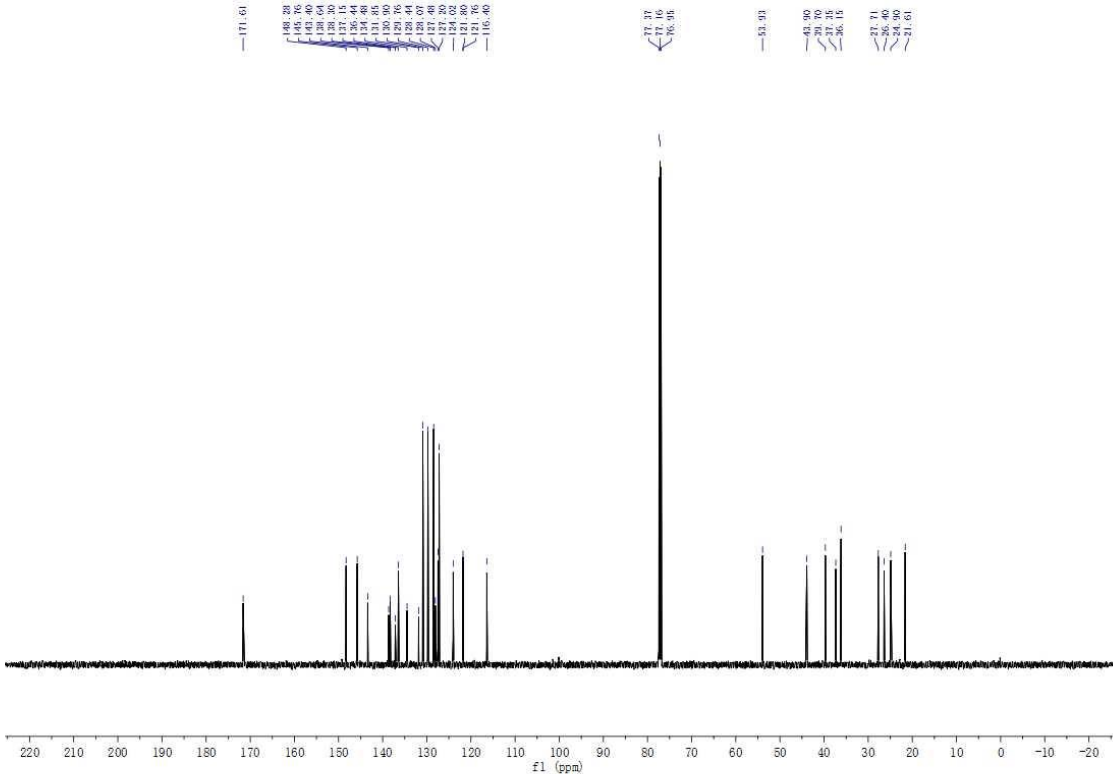

Supplementary Fig. 367. ^{13}C NMR of compound 6g. The sample has been recorded in 150 MHz, $CDCl_3$ at 25 °C

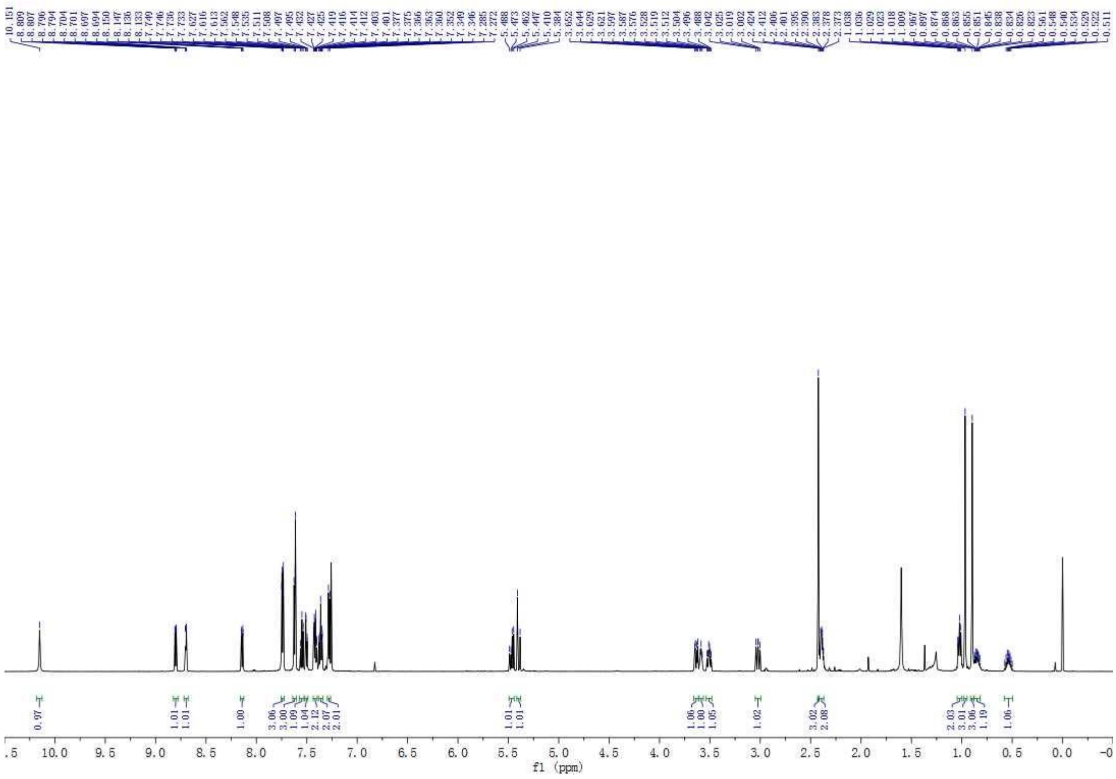

Supplementary Fig. 368. ^{1}H NMR of compound 6h. The sample has been recorded in 600 MHz, $CDCl_3$ at 25 °C

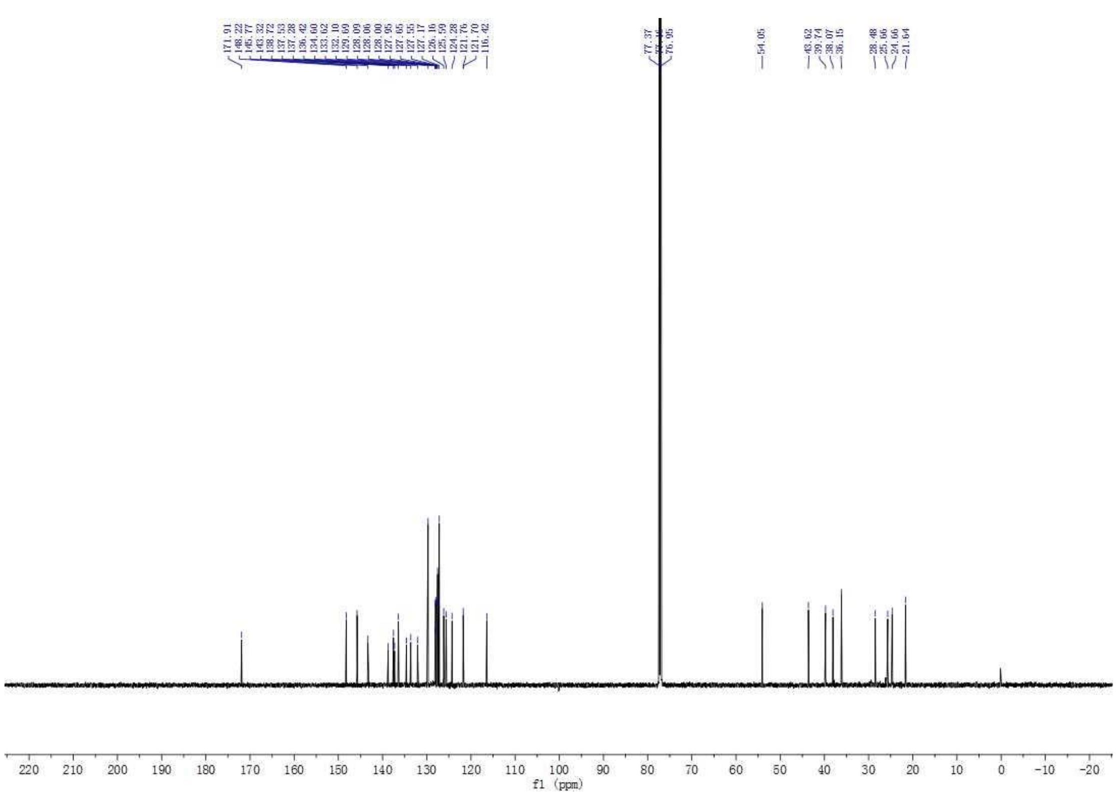

Supplementary Fig. 369. ^{13}C NMR of compound 6h. The sample has been recorded in 150 MHz, $CDCl_3$ at 25 °C

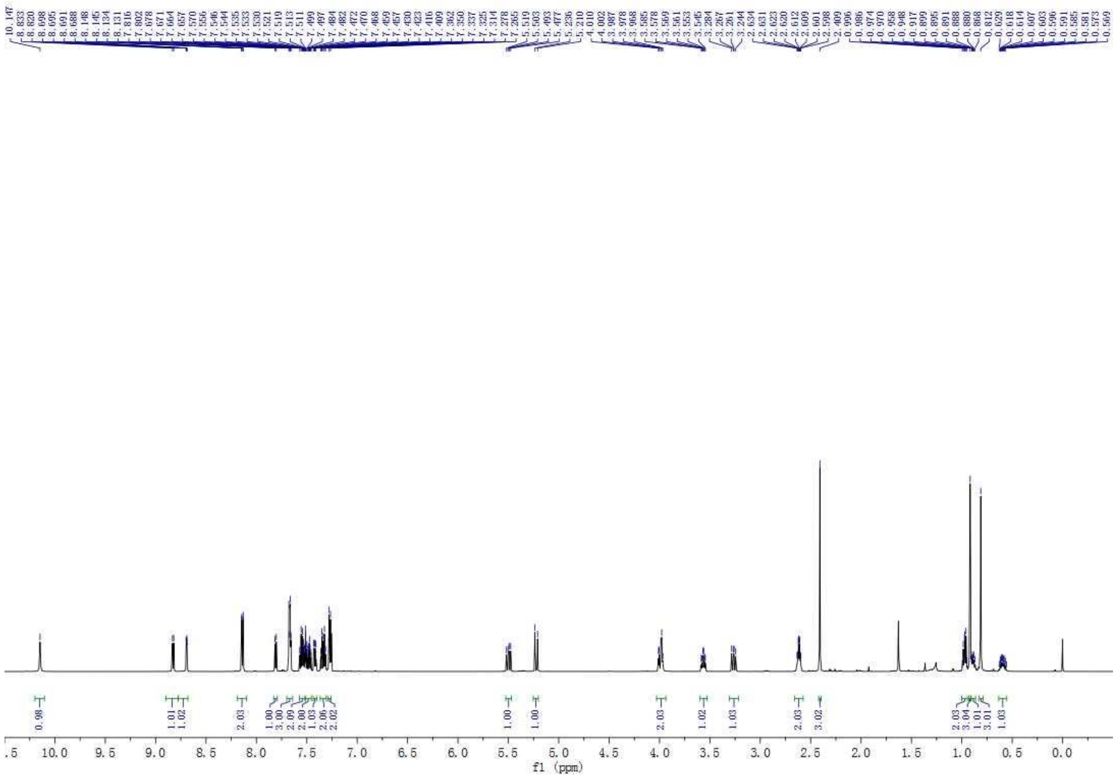

Supplementary Fig. 370. ^{1}H NMR of compound 6i. The sample has been recorded in 600 MHz, $CDCl_3$ at 25 °C

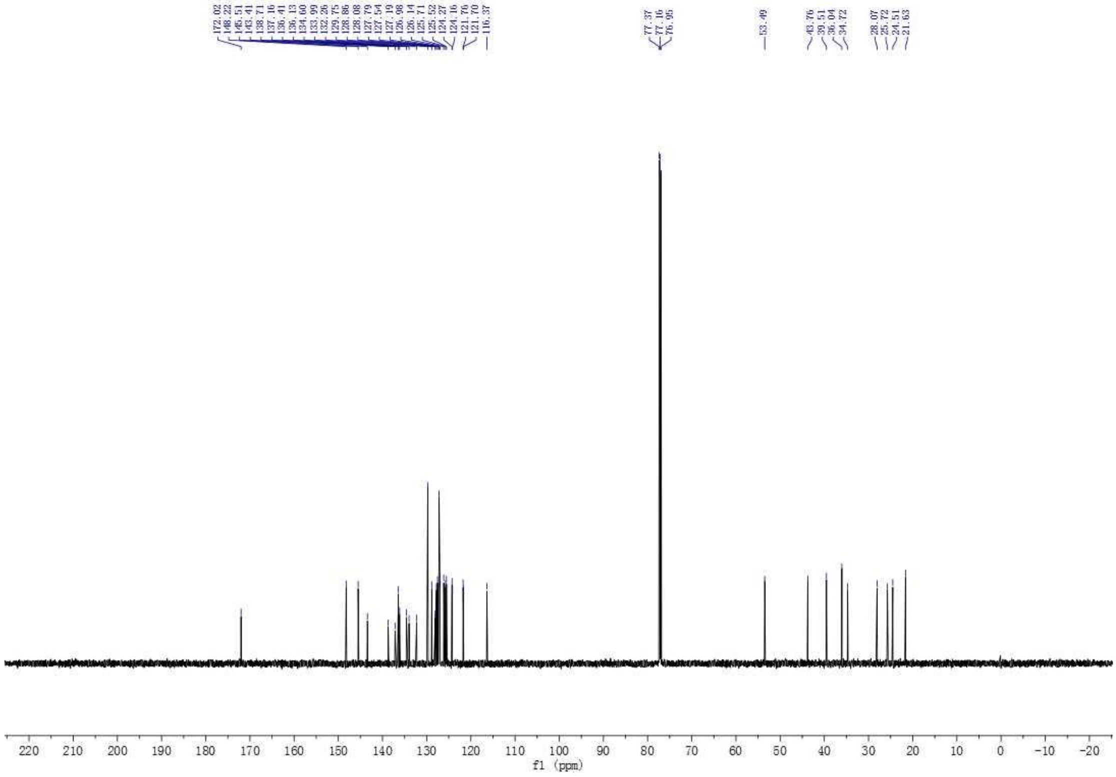

Supplementary Fig. 371. ^{13}C NMR of compound 6i. The sample has been recorded in 150 MHz, $CDCl_3$ at 25 °C

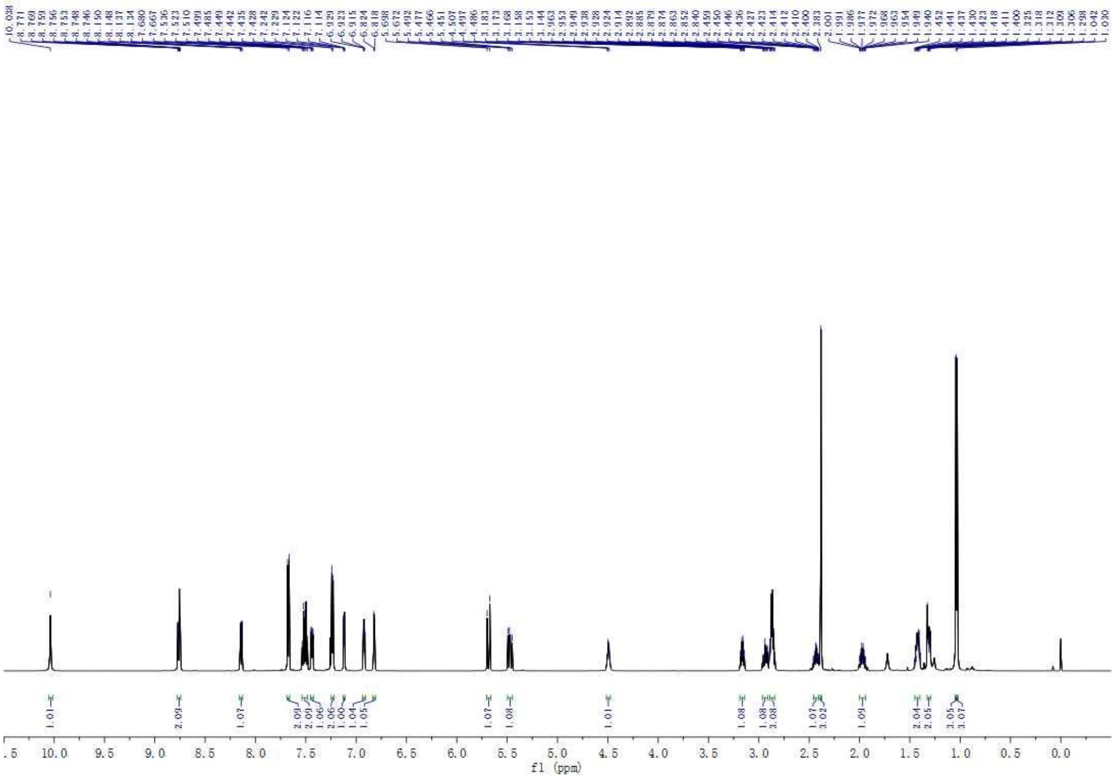

Supplementary Fig. 372. ^{1}H NMR of compound 6j. The sample has been recorded in 600 MHz, $CDCl_3$ at 25 °C

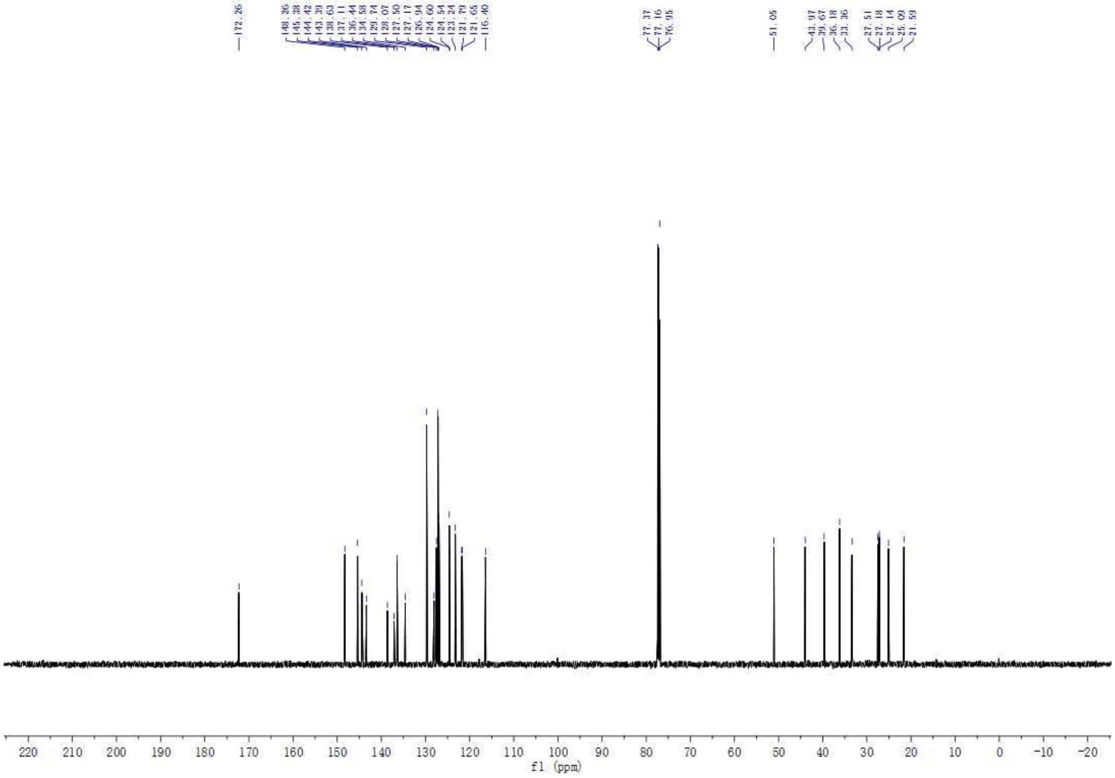

Supplementary Fig. 373. ^{13}C NMR of compound 6j. The sample has been recorded in 150 MHz, $CDCl_3$ at 25 °C

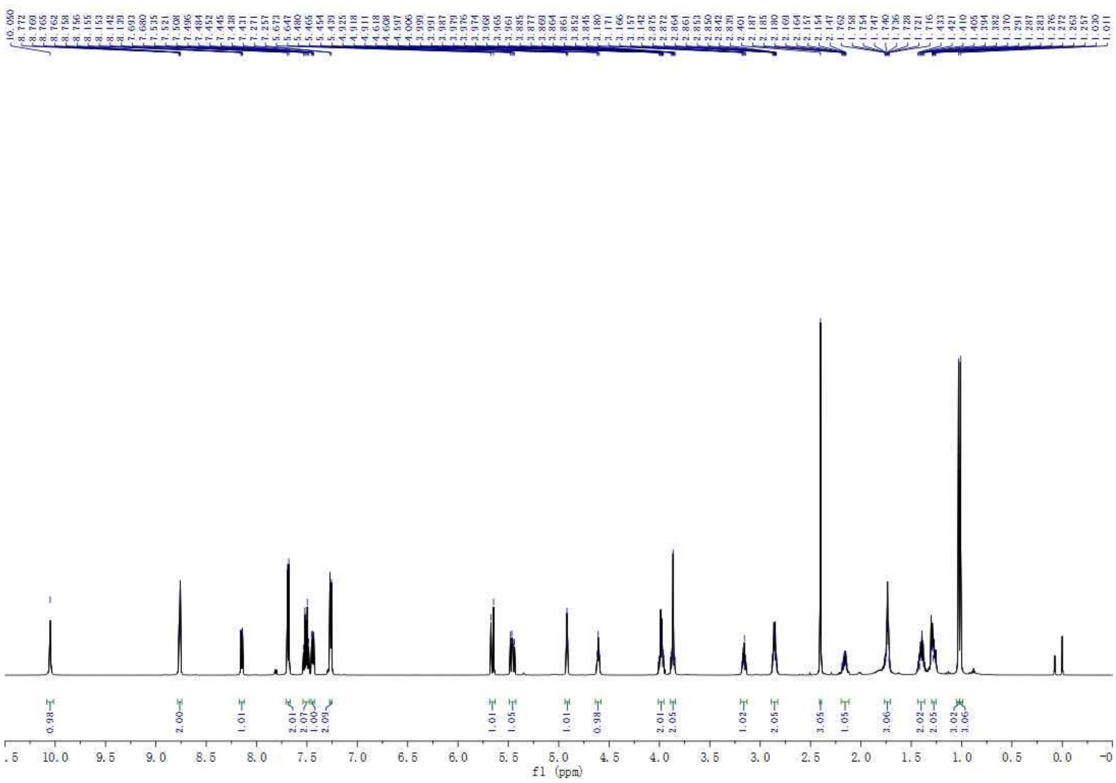

Supplementary Fig. 374. ^{1}H NMR of compound 6k. The sample has been recorded in 600 MHz, $CDCl_3$ at 25 °C

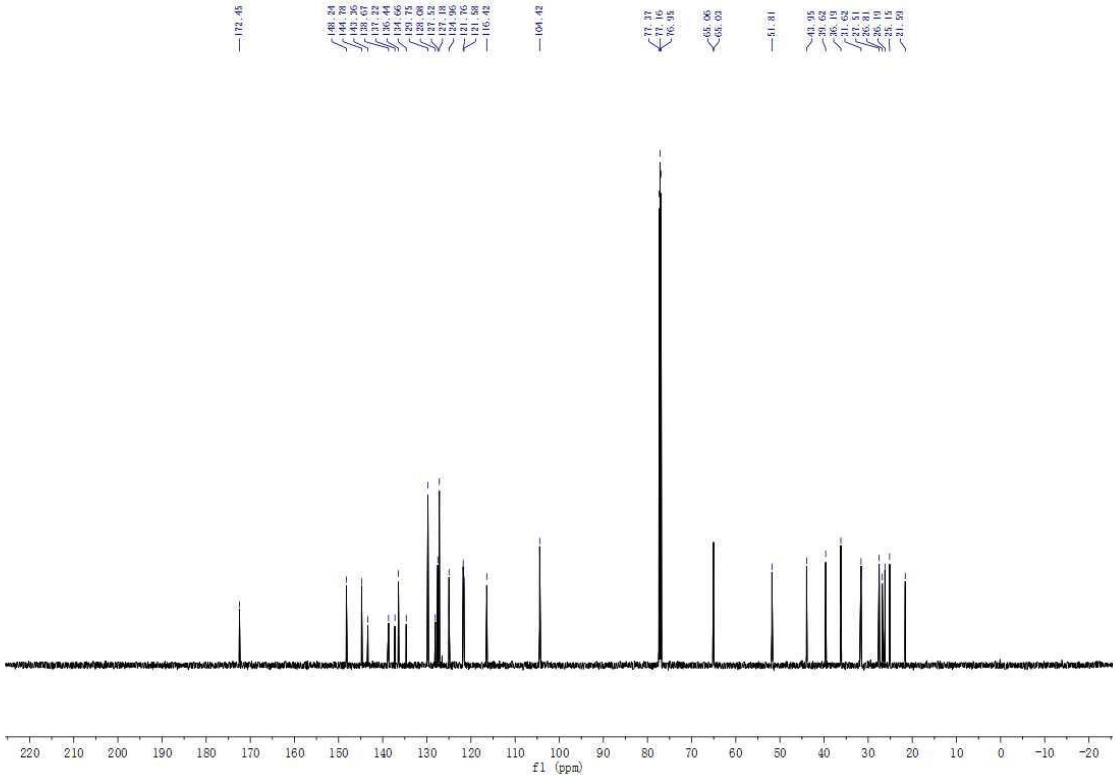

Supplementary Fig. 375. ^{13}C NMR of compound 6k. The sample has been recorded in 150 MHz, $CDCl_3$ at 25 °C

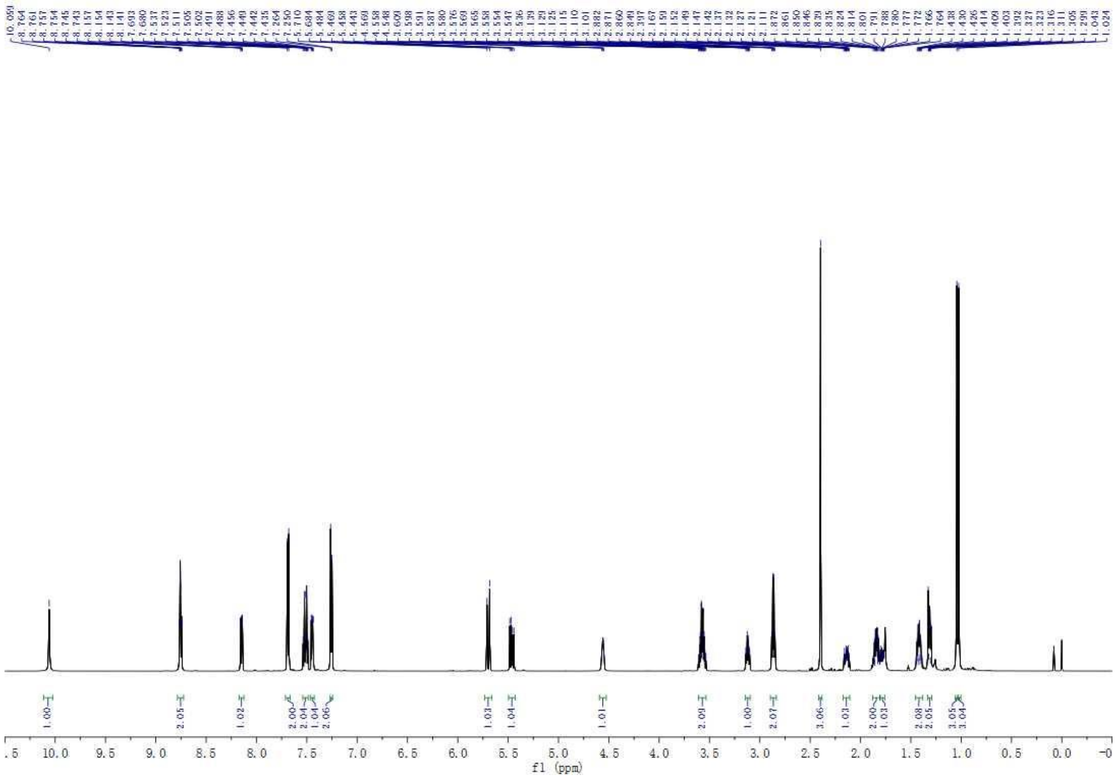

Supplementary Fig. 376. ^{1}H NMR of compound 6l. The sample has been recorded in 600 MHz, $CDCl_3$ at 25 °C

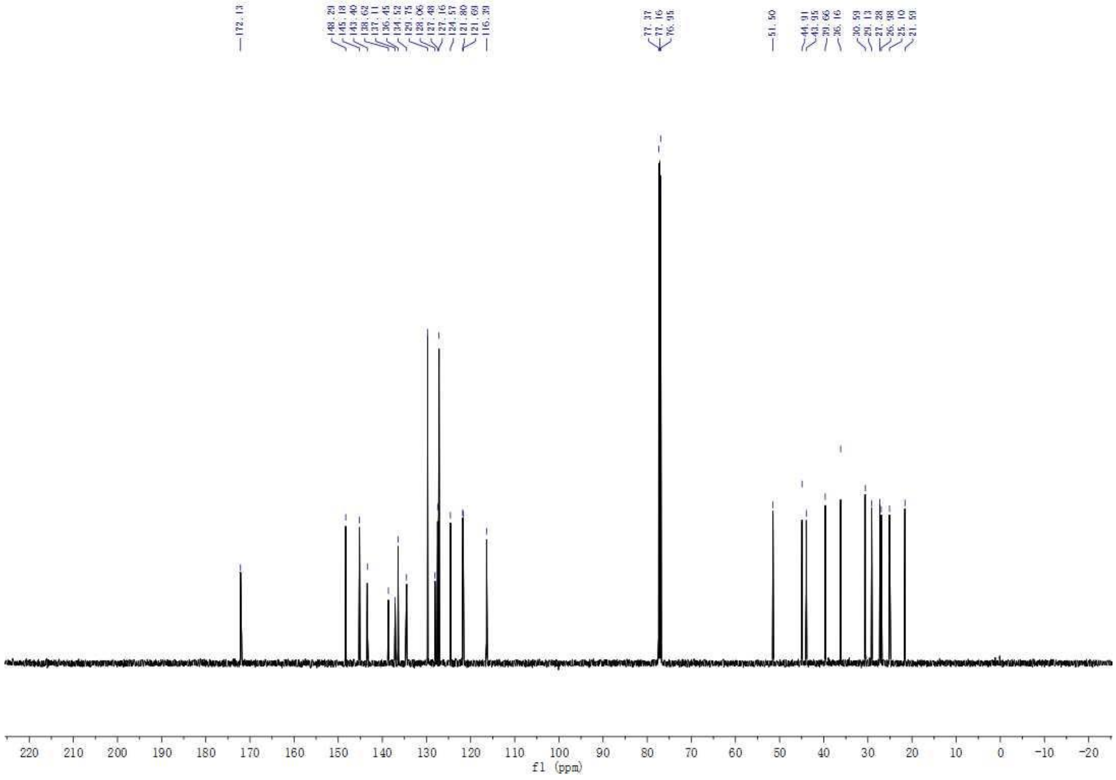

Supplementary Fig. 377. ^{13}C NMR of compound 6l. The sample has been recorded in 150 MHz, $CDCl_3$ at 25 °C

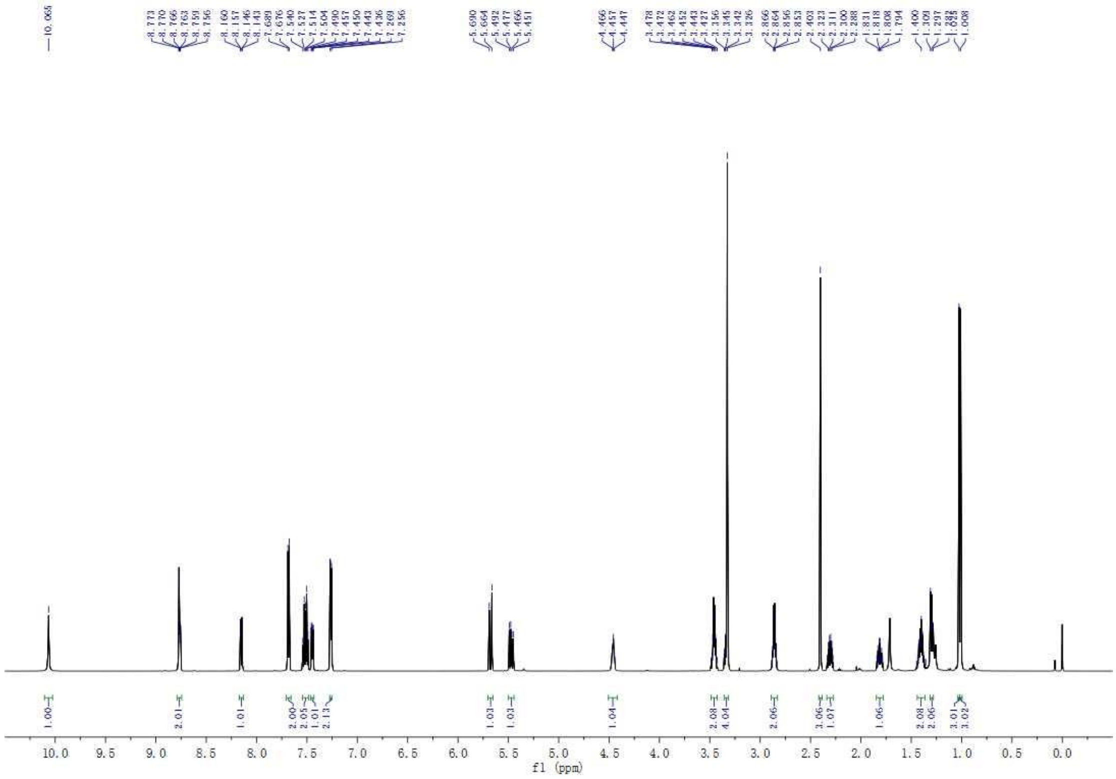

Supplementary Fig. 378. ^{1}H NMR of compound 6m. The sample has been recorded in 600 MHz, $CDCl_3$ at 25 °C

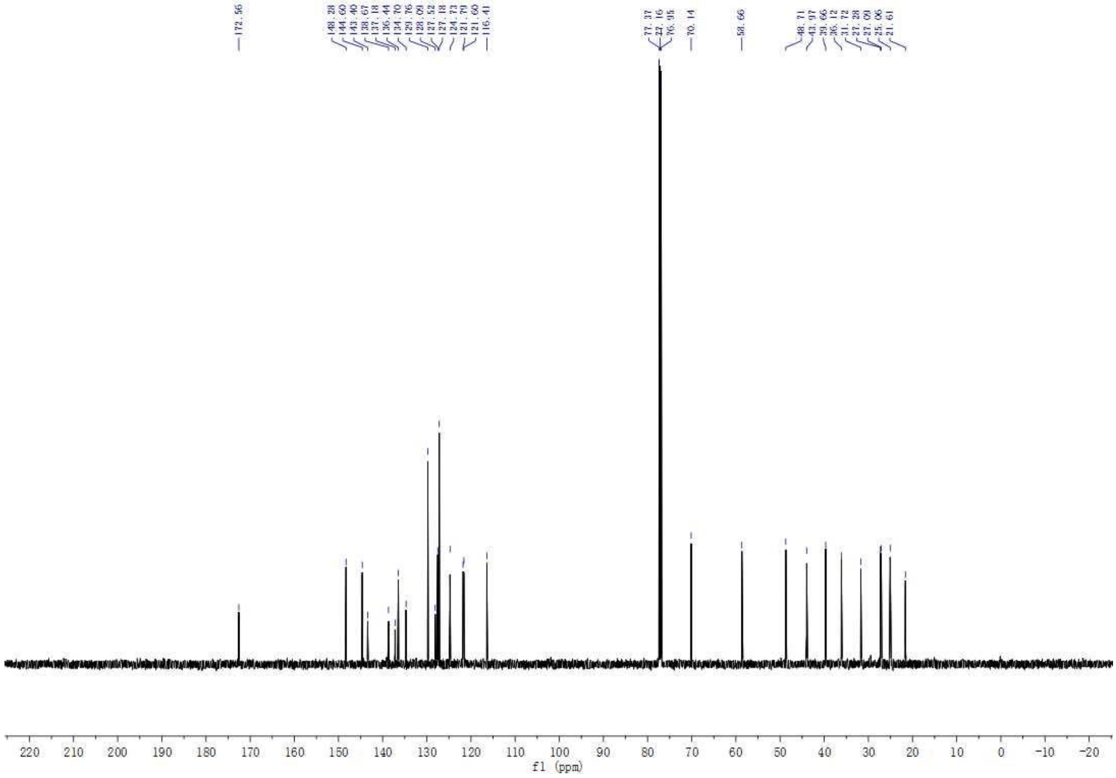

Supplementary Fig. 379. ^{13}C NMR of compound 6m. The sample has been recorded in 150 MHz, $CDCl_3$ at 25 °C

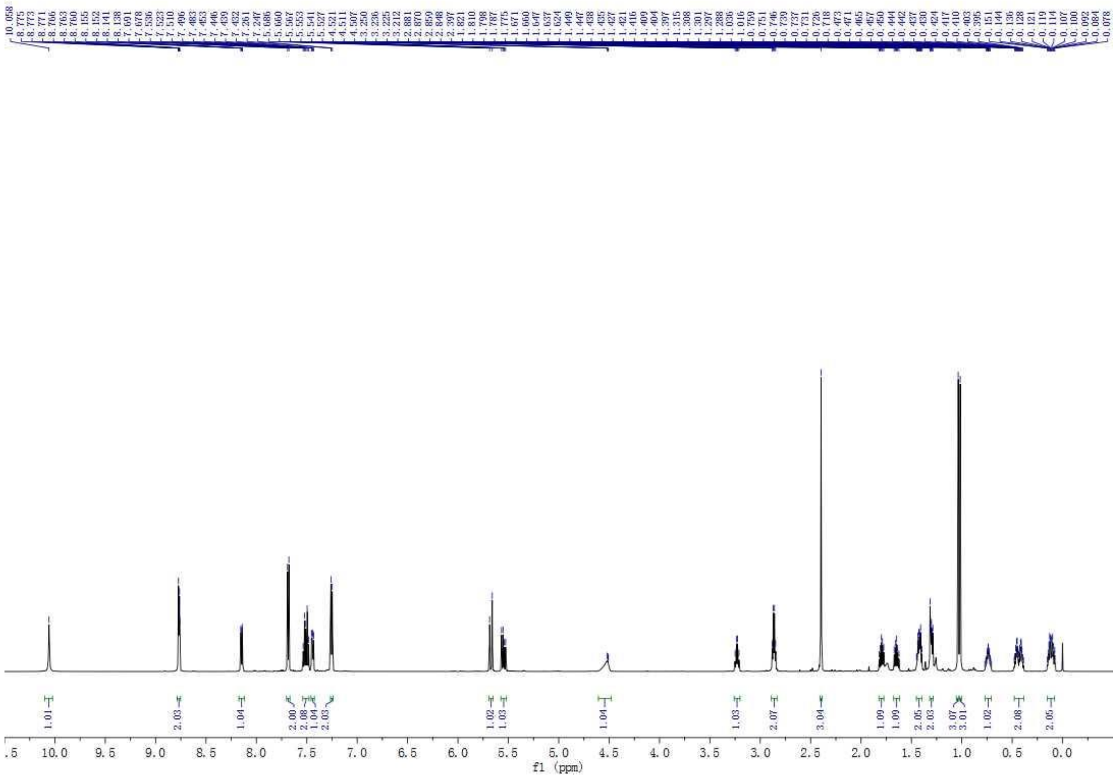

Supplementary Fig. 380. ^{1}H NMR of compound 6n. The sample has been recorded in 600 MHz, $CDCl_3$ at 25 °C

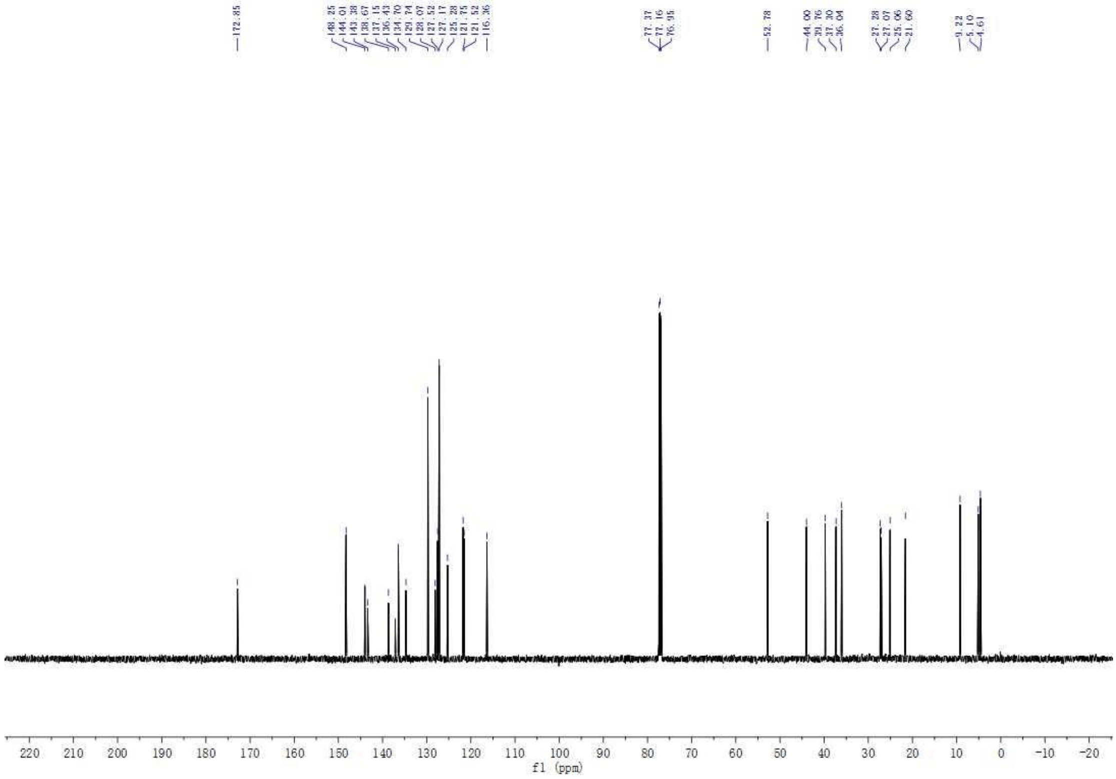

Supplementary Fig. 381. ^{13}C NMR of compound 6n. The sample has been recorded in 150 MHz, $CDCl_3$ at 25 °C

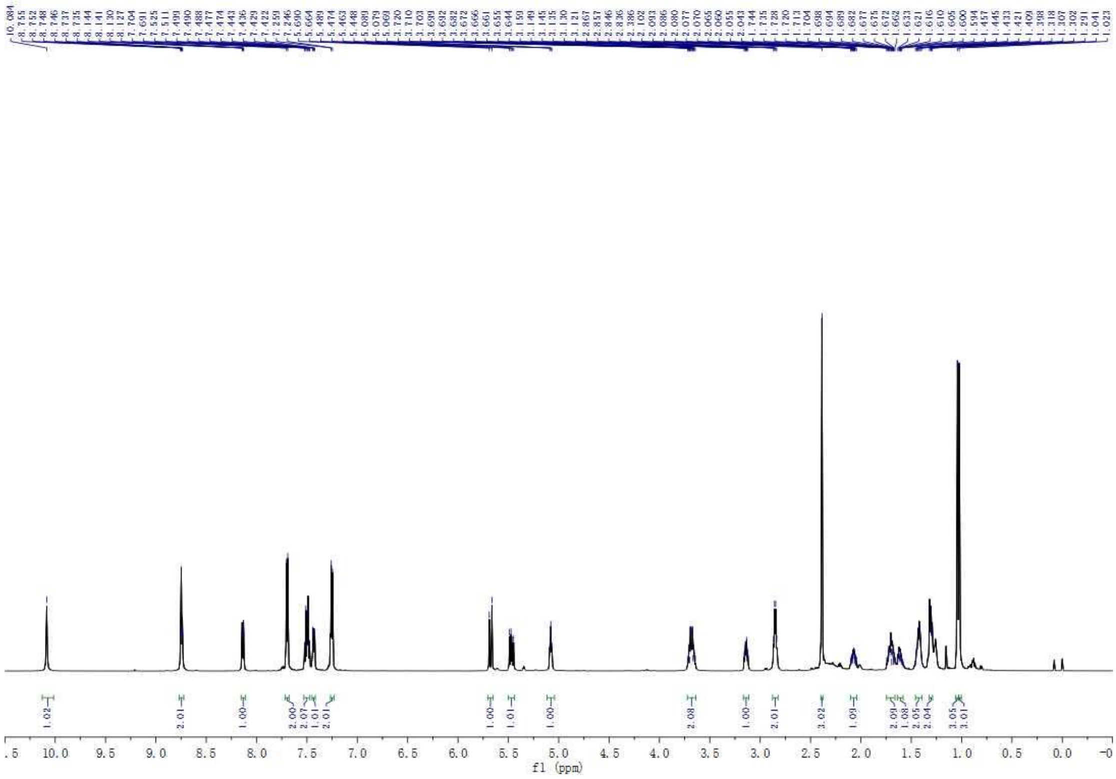

Supplementary Fig. 382. ^{1}H NMR of compound 6o. The sample has been recorded in 600 MHz, $CDCl_3$ at 25 °C

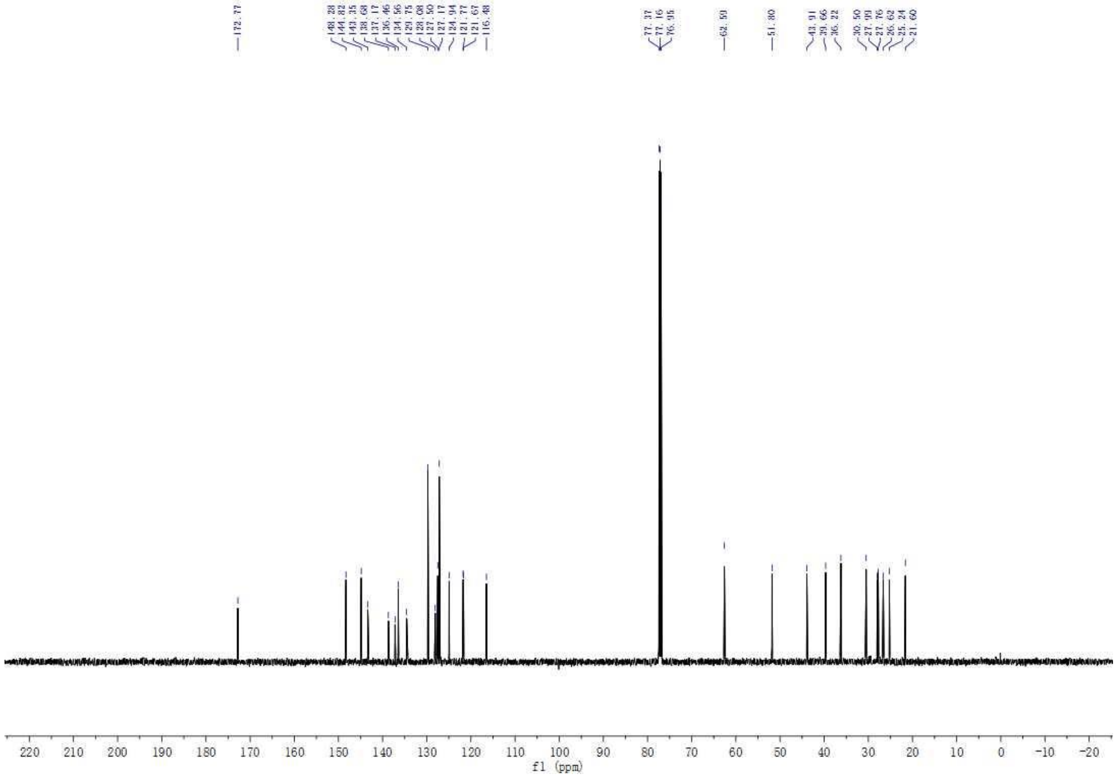

Supplementary Fig. 383. ^{13}C NMR of compound 6o. The sample has been recorded in 150 MHz, $CDCl_3$ at 25 °C

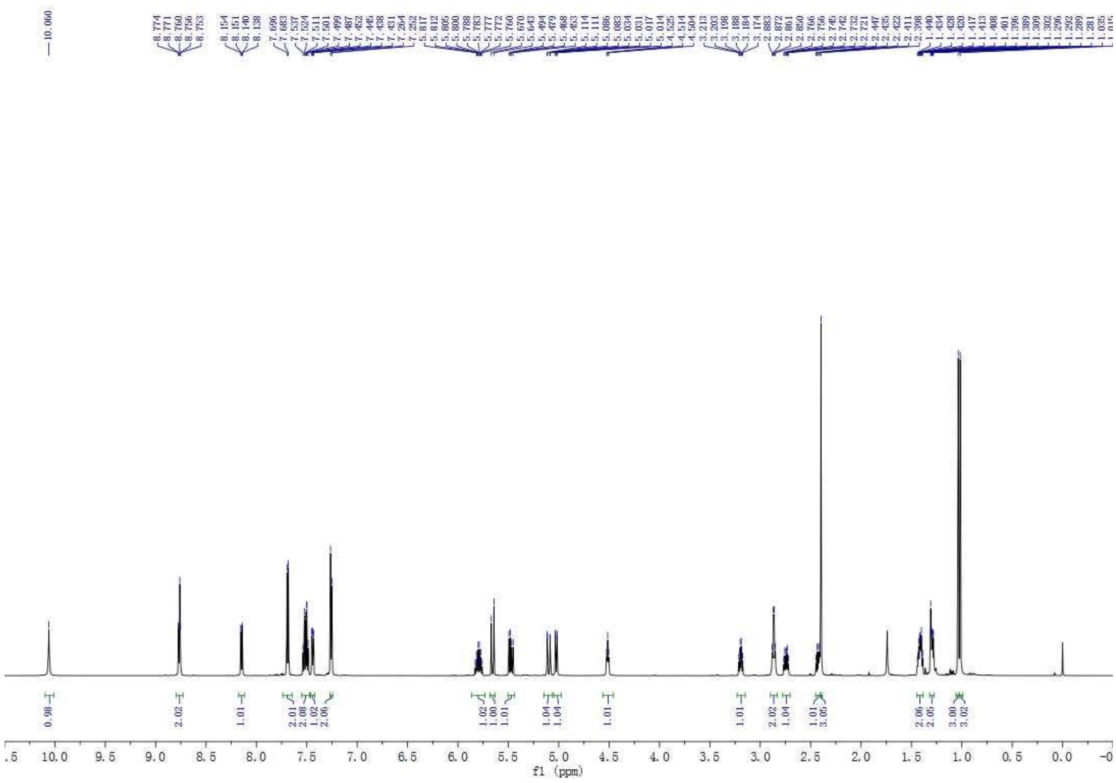

Supplementary Fig. 384. ^{1}H NMR of compound 6p. The sample has been recorded in 600 MHz, $CDCl_3$ at 25 °C

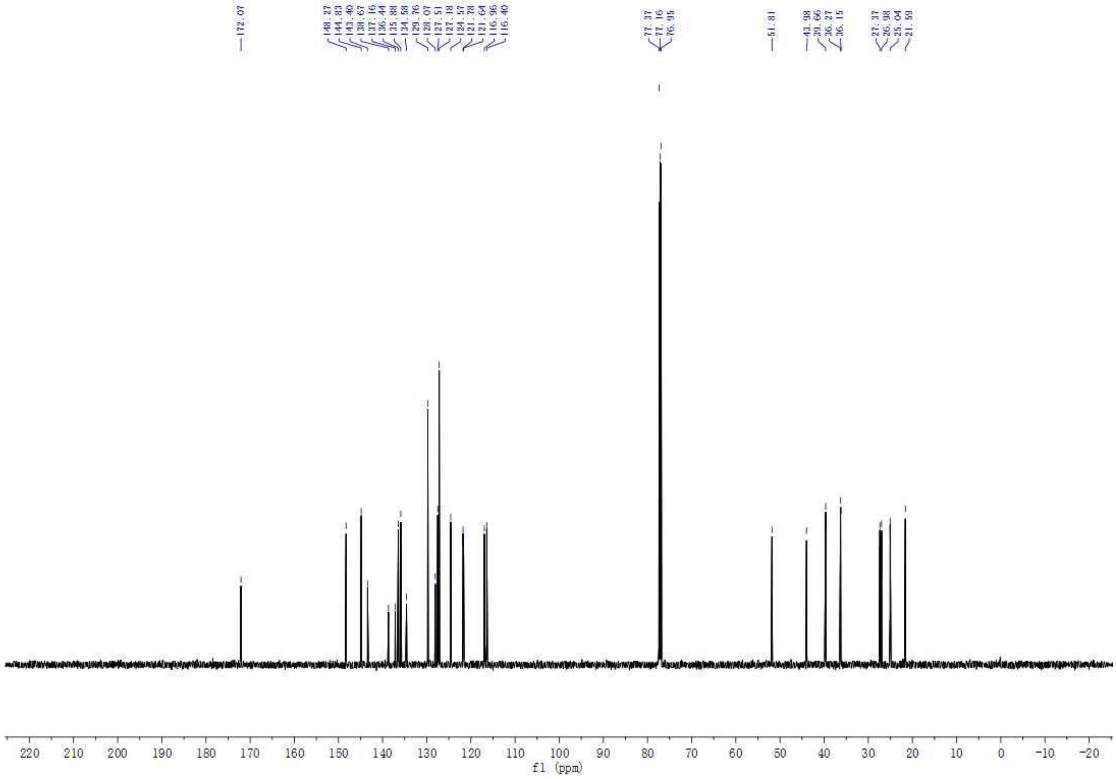

Supplementary Fig. 385. ^{13}C NMR of compound 6p. The sample has been recorded in 150 MHz, $CDCl_3$ at 25 °C

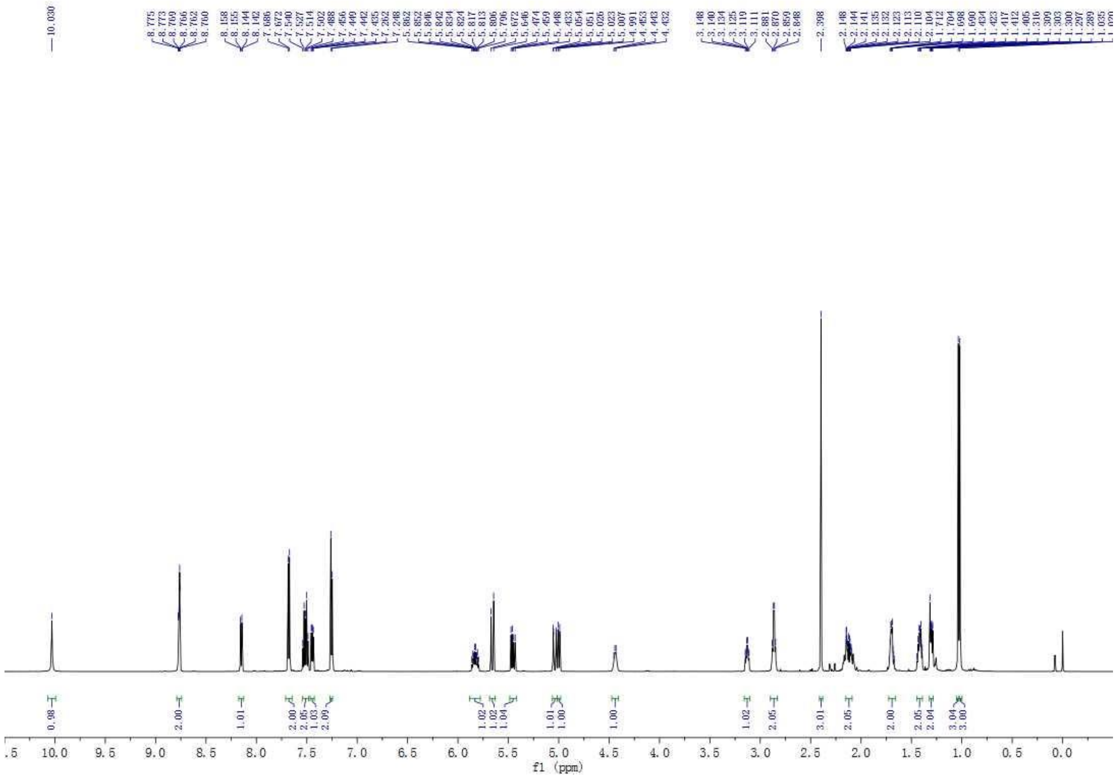

Supplementary Fig. 386. ^{1}H NMR of compound 6q. The sample has been recorded in 600 MHz, $CDCl_3$ at 25 °C

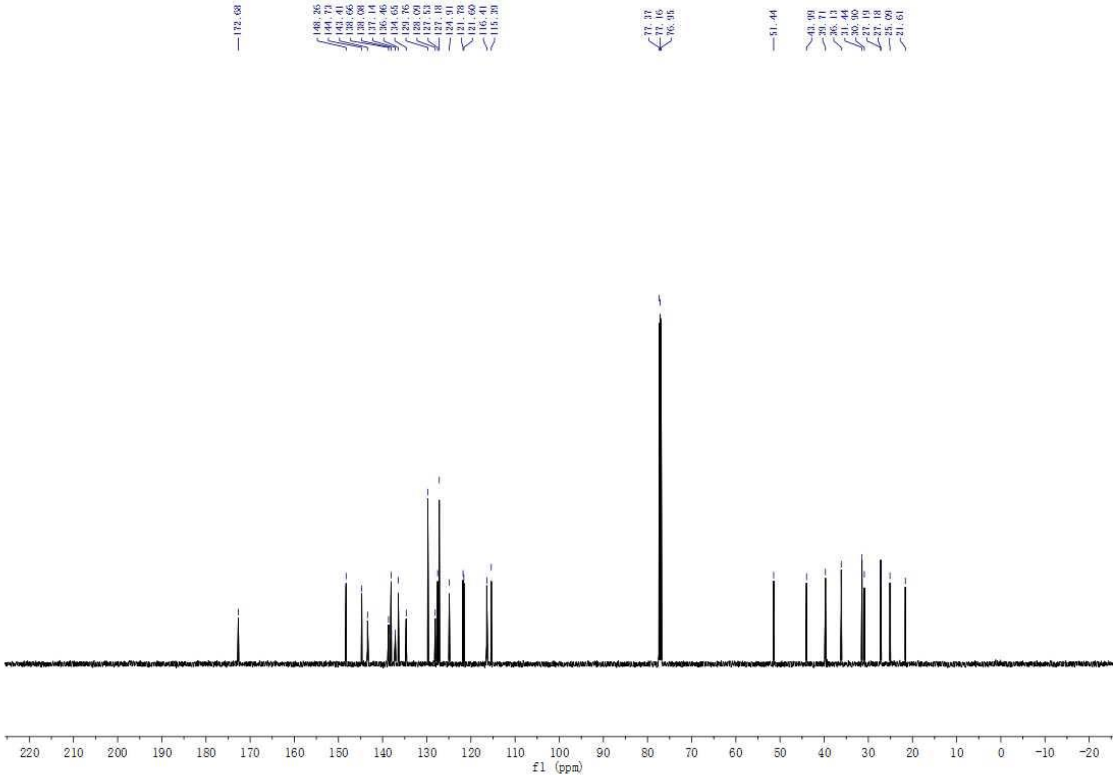

Supplementary Fig. 387. ^{13}C NMR of compound 6q. The sample has been recorded in 150 MHz, $CDCl_3$ at 25 °C

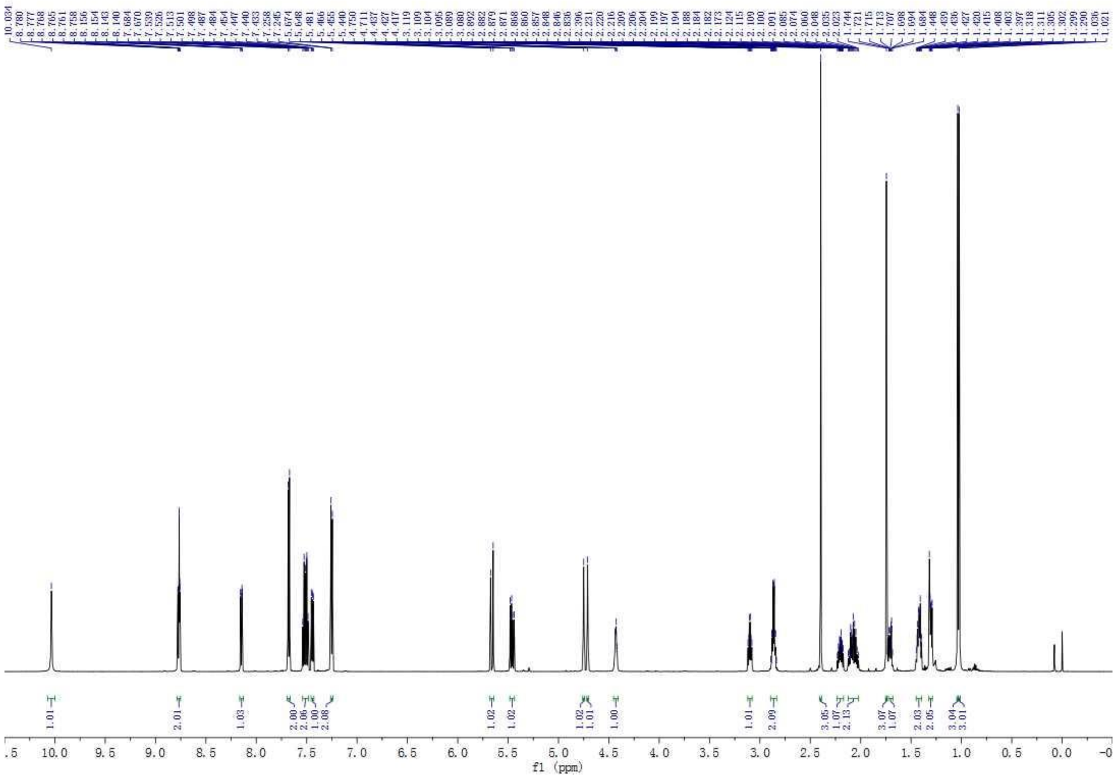

Supplementary Fig. 388. ^{1}H NMR of compound 6r. The sample has been recorded in 600 MHz, $CDCl_3$ at 25 °C

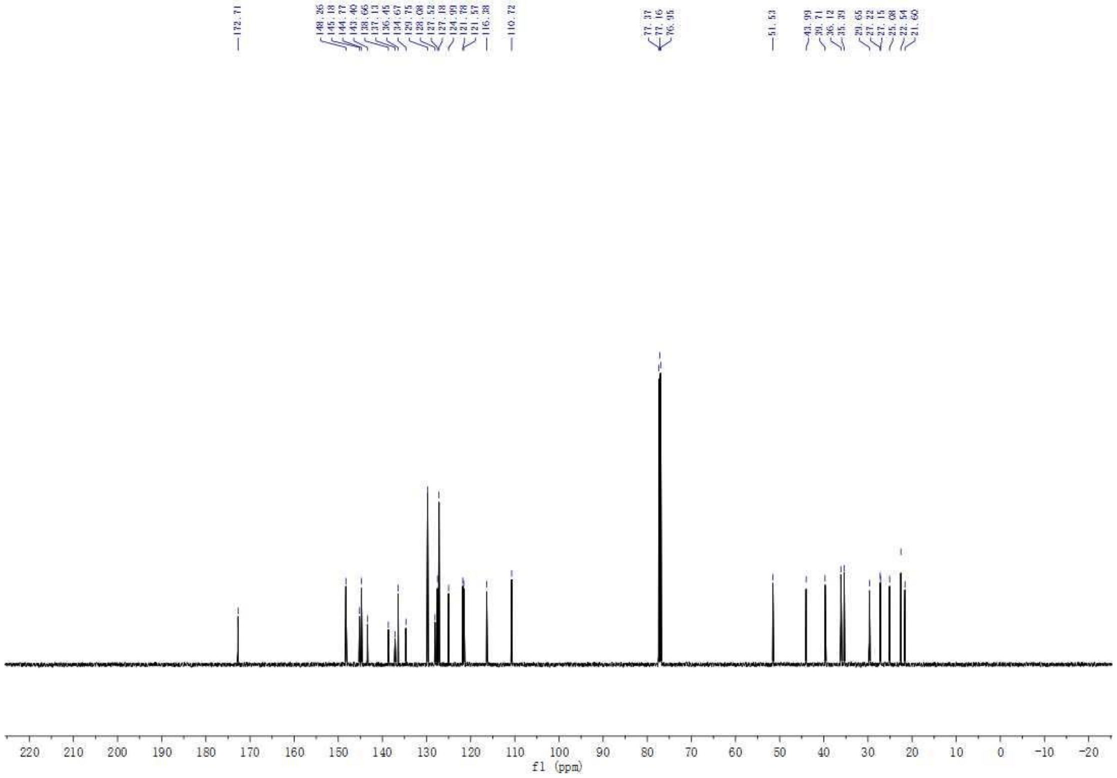

Supplementary Fig. 389. ^{13}C NMR of compound 6r. The sample has been recorded in 150 MHz, $CDCl_3$ at 25 °C

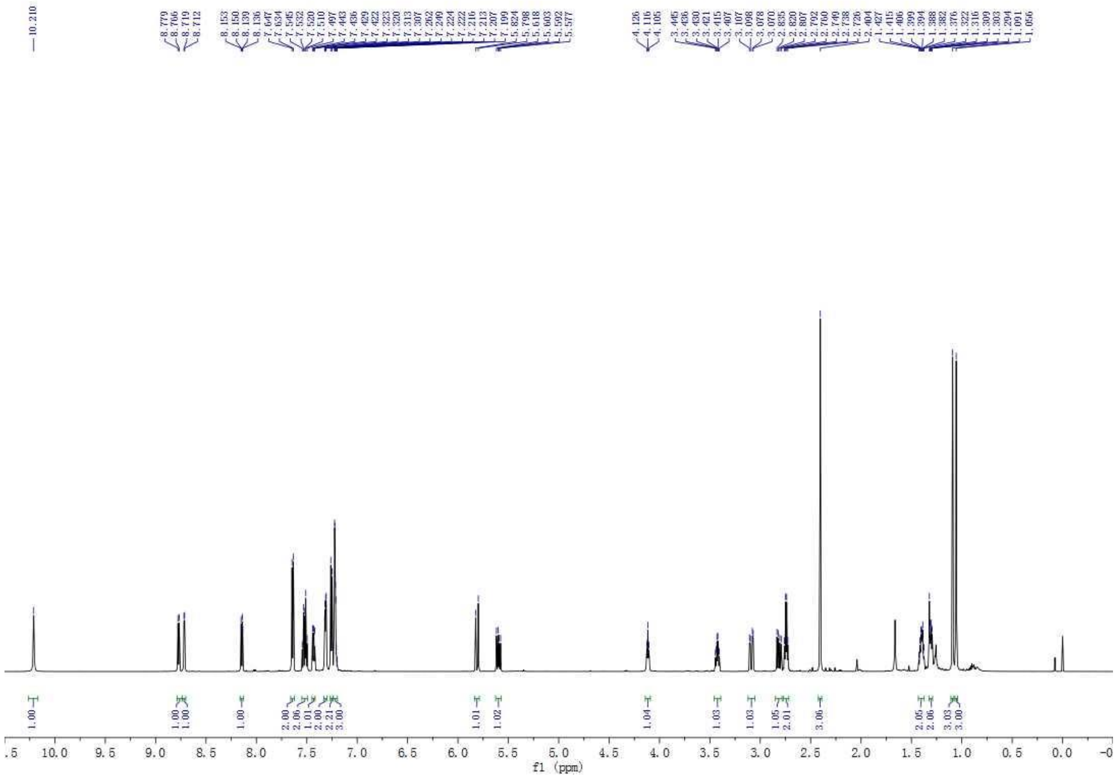

Supplementary Fig. 390. ^{1}H NMR of compound 6s. The sample has been recorded in 600 MHz, $CDCl_3$ at 25 °C

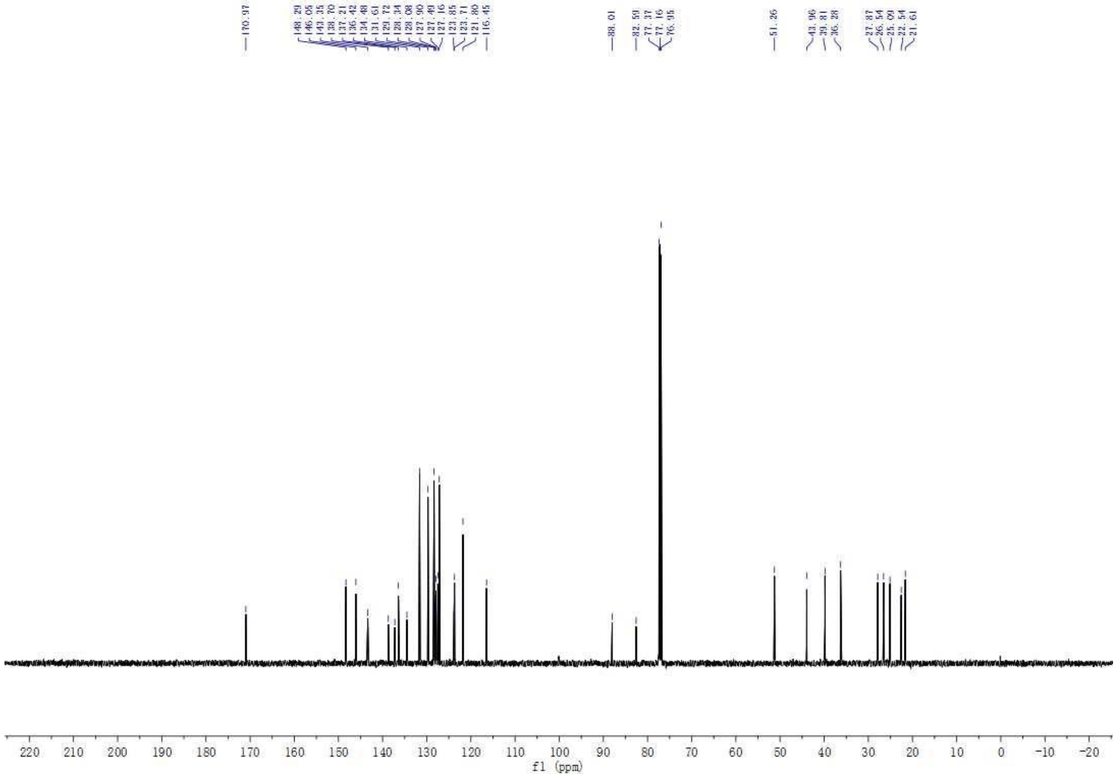

Supplementary Fig. 391. ^{13}C NMR of compound 6s. The sample has been recorded in 150 MHz, $CDCl_3$ at 25 °C

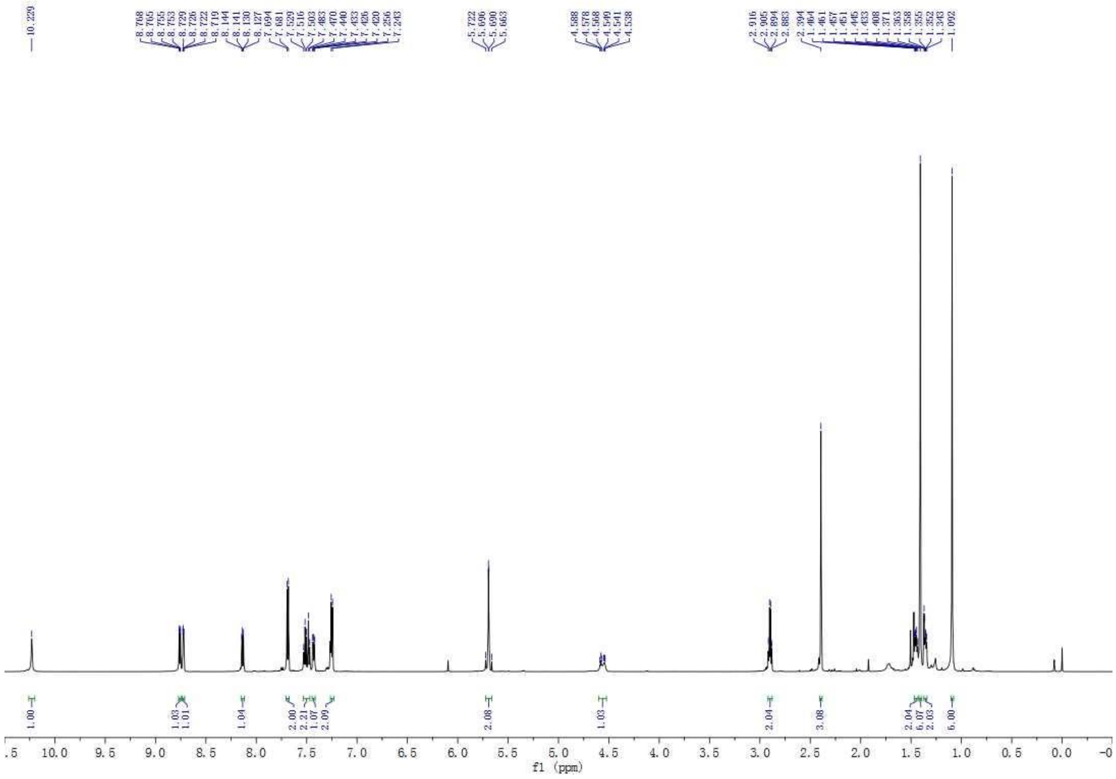

Supplementary Fig. 392. ^{1}H NMR of compound 6t. The sample has been recorded in 600 MHz, $CDCl_3$ at 25 °C

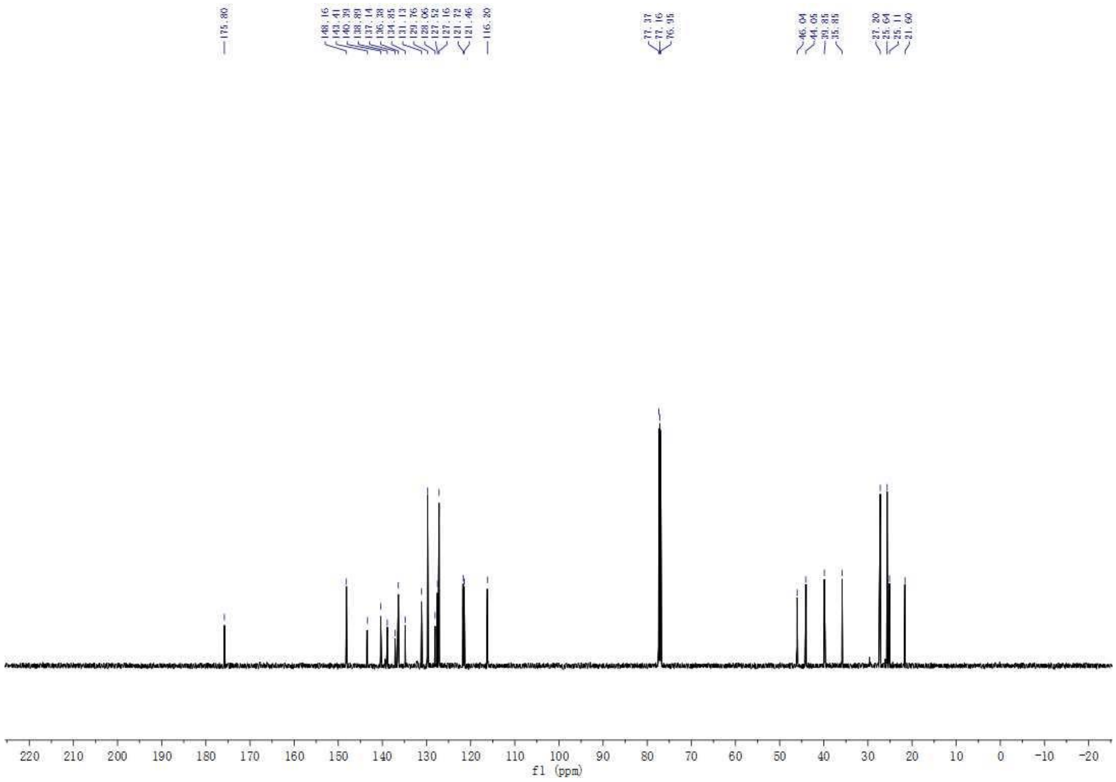

Supplementary Fig. 393. ^{13}C NMR of compound 6t. The sample has been recorded in 150 MHz, $CDCl_3$ at 25 °C

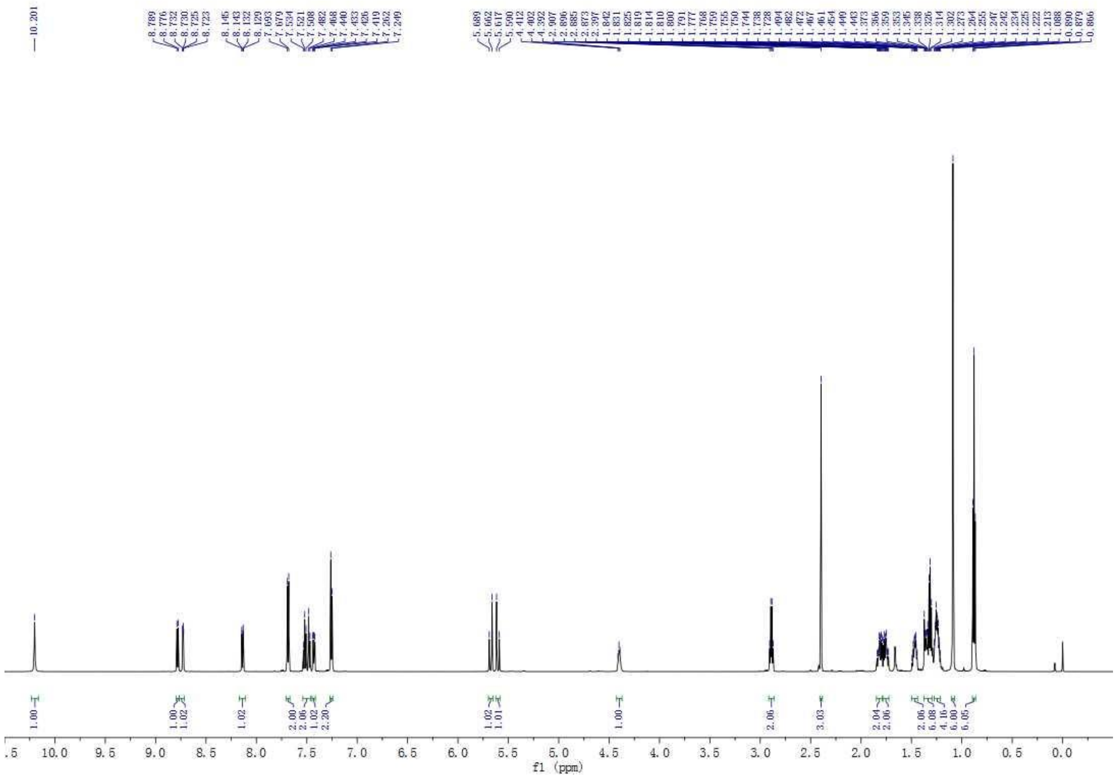

Supplementary Fig. 394. ^{1}H NMR of compound 6u. The sample has been recorded in 600 MHz, $CDCl_3$ at 25 °C

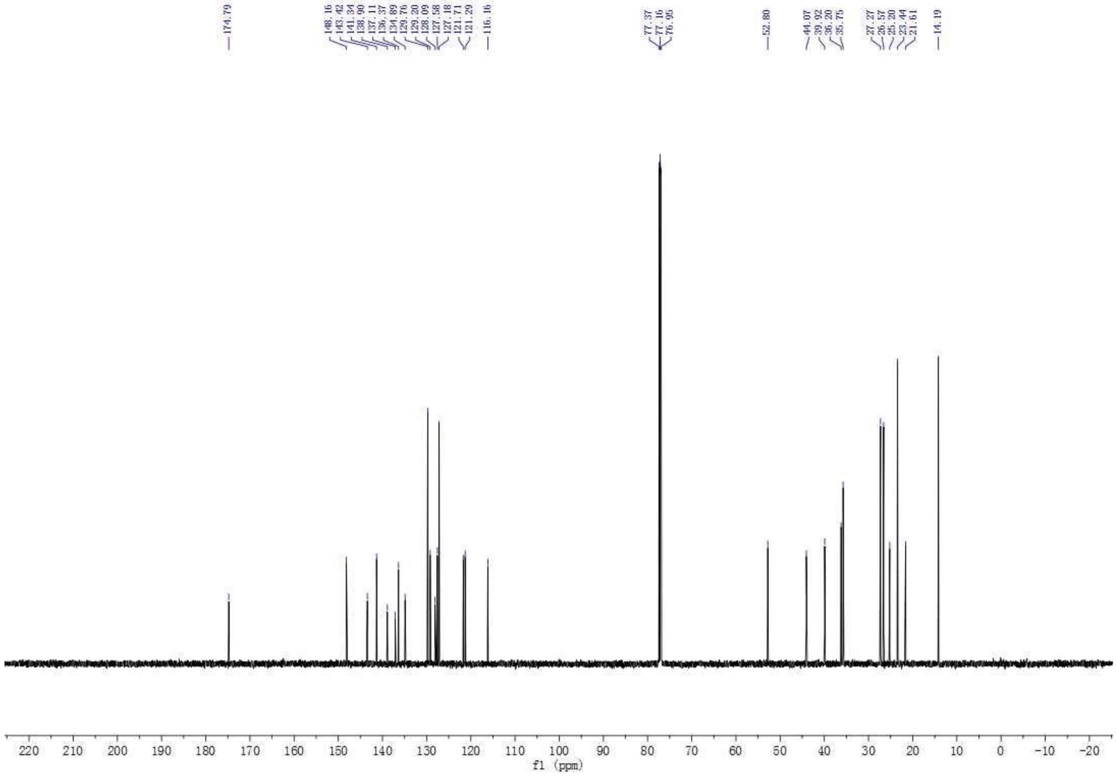

Supplementary Fig. 395. ^{13}C NMR of compound 6u. The sample has been recorded in 150 MHz, $CDCl_3$ at 25 °C

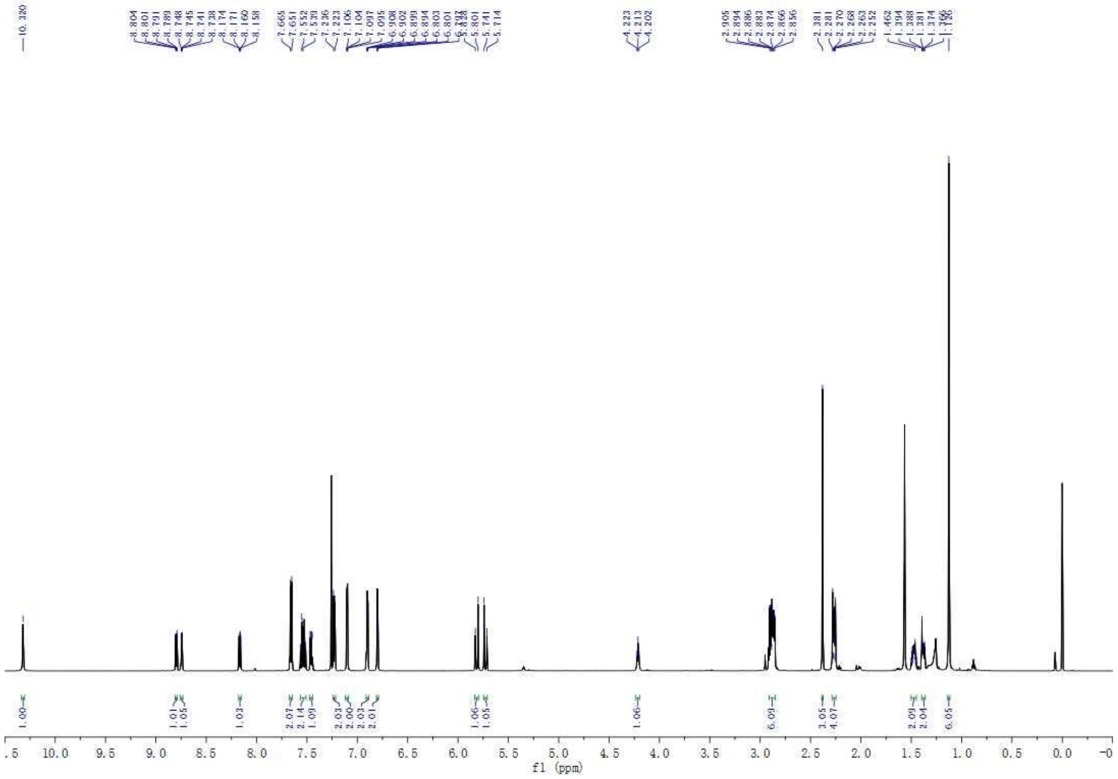

Supplementary Fig. 396. ^{1}H NMR of compound 6v. The sample has been recorded in 600 MHz, $CDCl_3$ at 25 °C

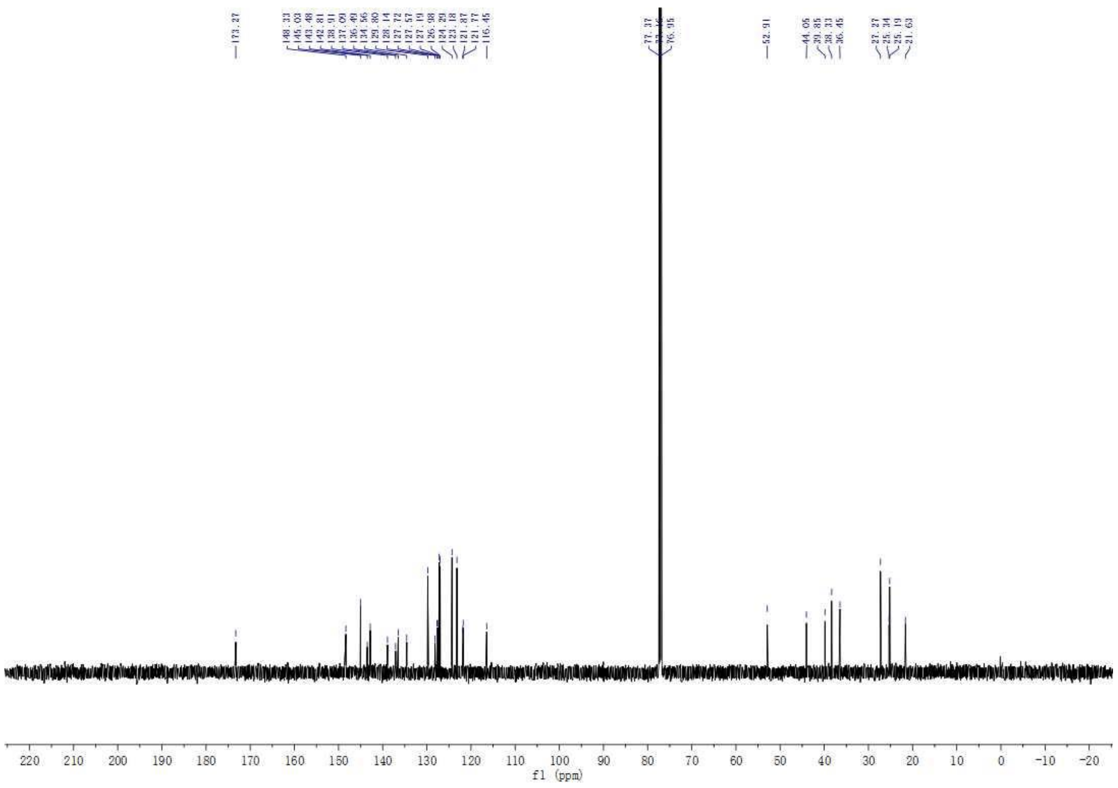

Supplementary Fig. 397. ^{13}C NMR of compound 6v. The sample has been recorded in 150 MHz, $CDCl_3$ at 25 °C

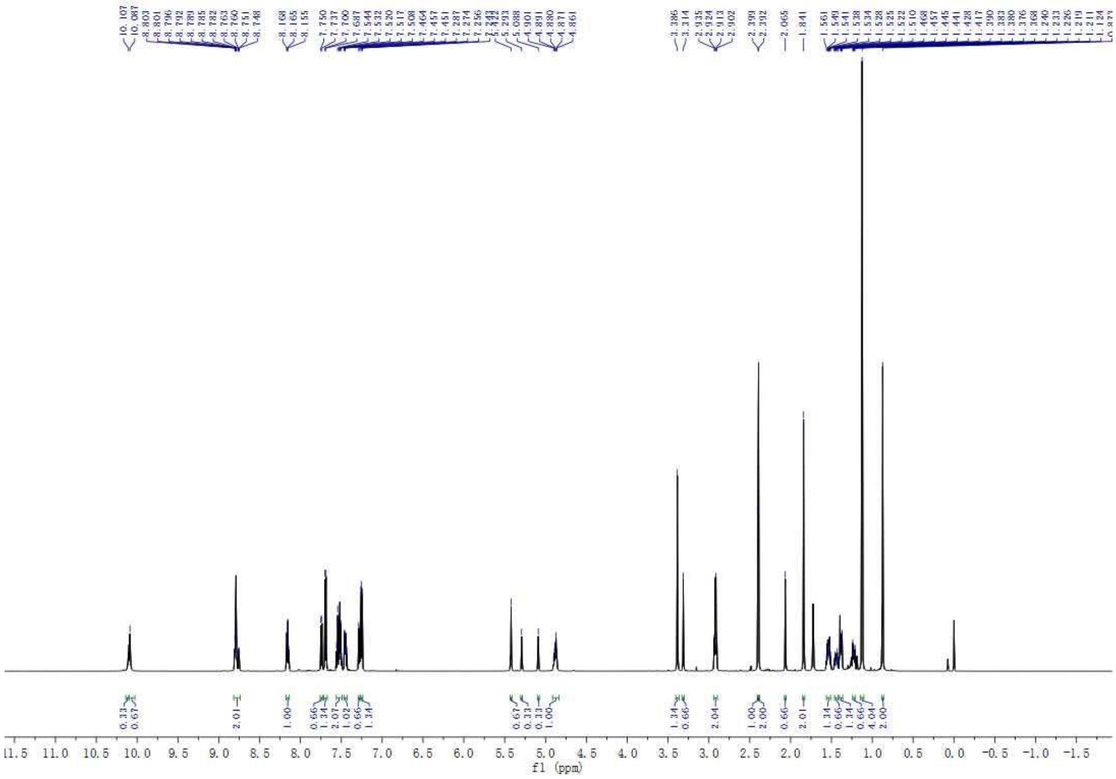

Supplementary Fig. 398. ^{1}H NMR of compound 6w + 6w′ (2:1). The sample has been recorded in 600 MHz, $CDCl_3$ at 25 °C

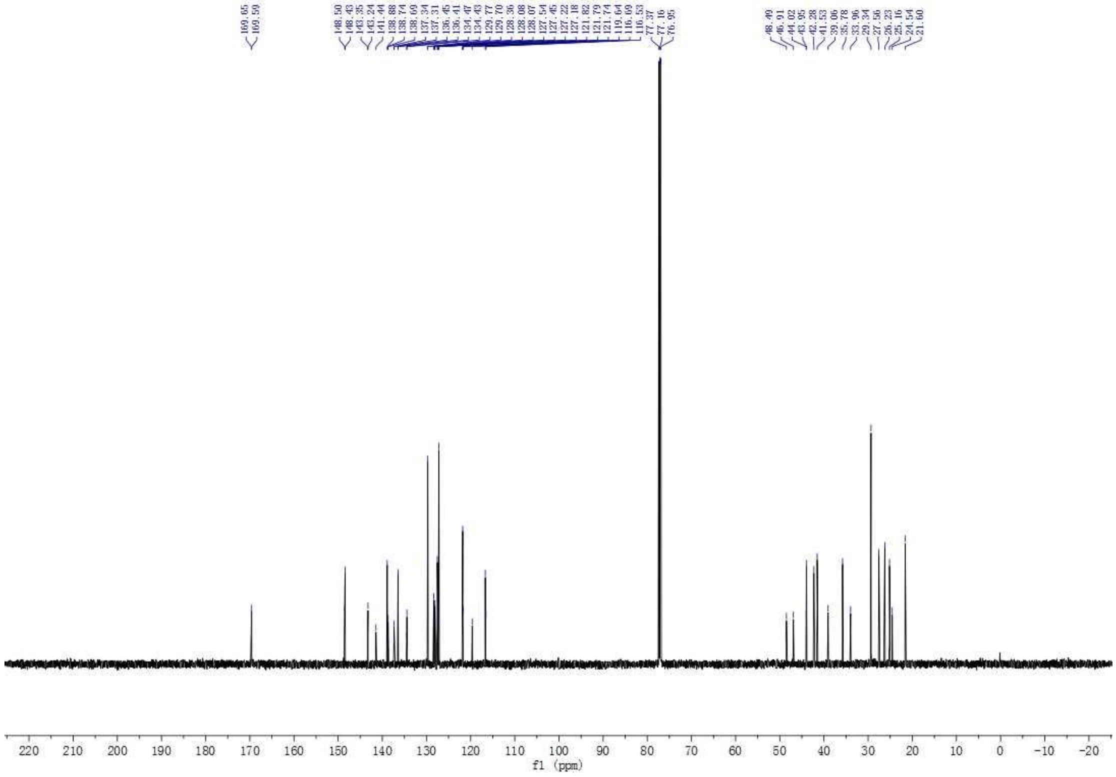

Supplementary Fig. 399. ^{13}C NMR of compound 6w + 6w′ (2:1). The sample has been recorded in 150 MHz, $CDCl_3$ at 25 °C

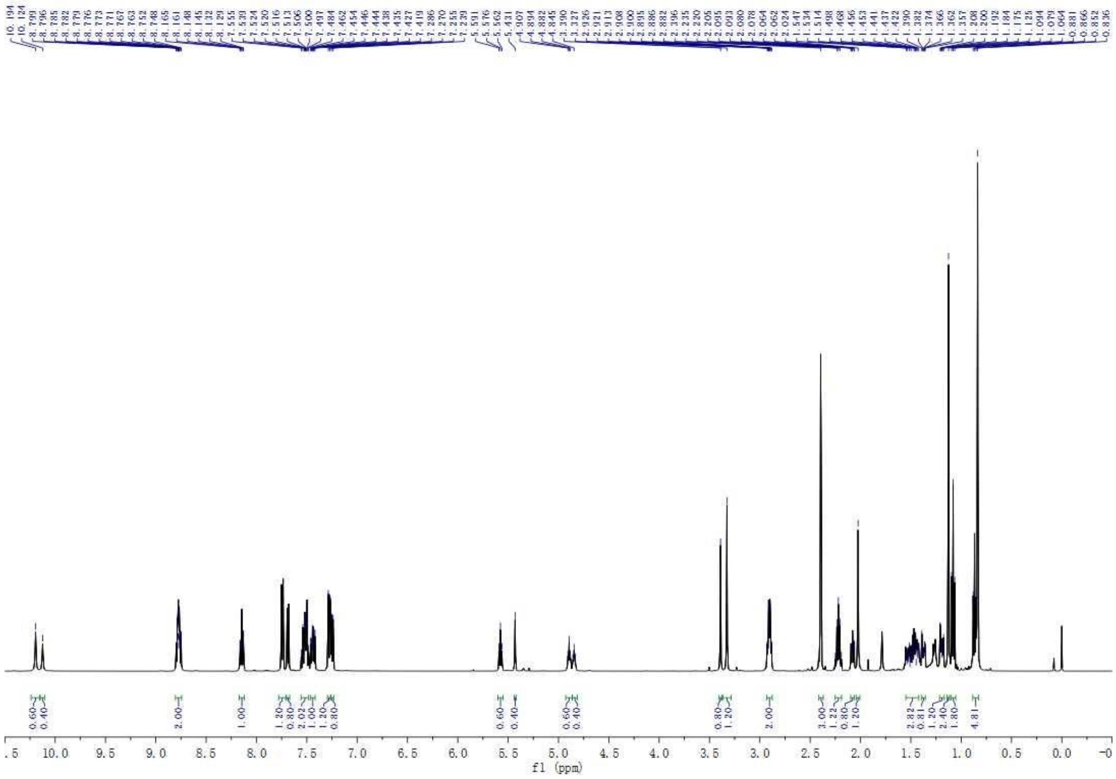

Supplementary Fig. 400. ^{1}H NMR of compound 6x + 6x′ (2:3). The sample has been recorded in 500 MHz, $CDCl_3$ at 25 °C

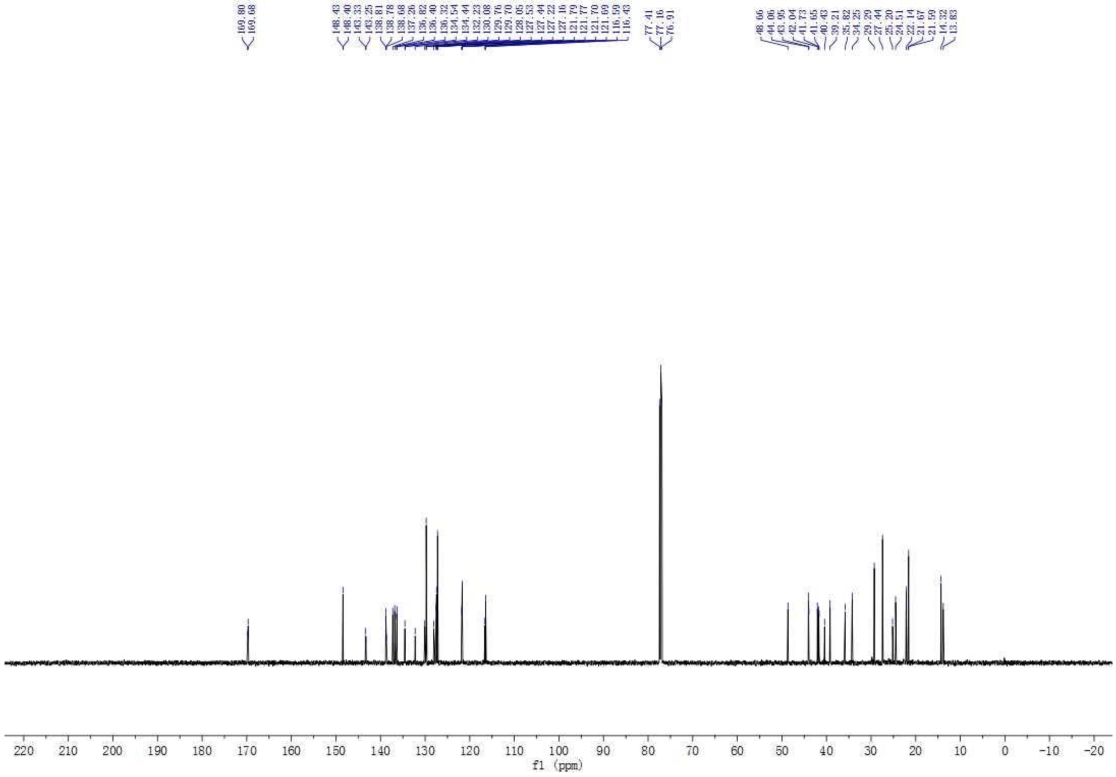

Supplementary Fig. 401. ^{13}C NMR of compound 6x + 6x′ (2:3). The sample has been recorded in 125 MHz, $CDCl_3$ at 25 °C

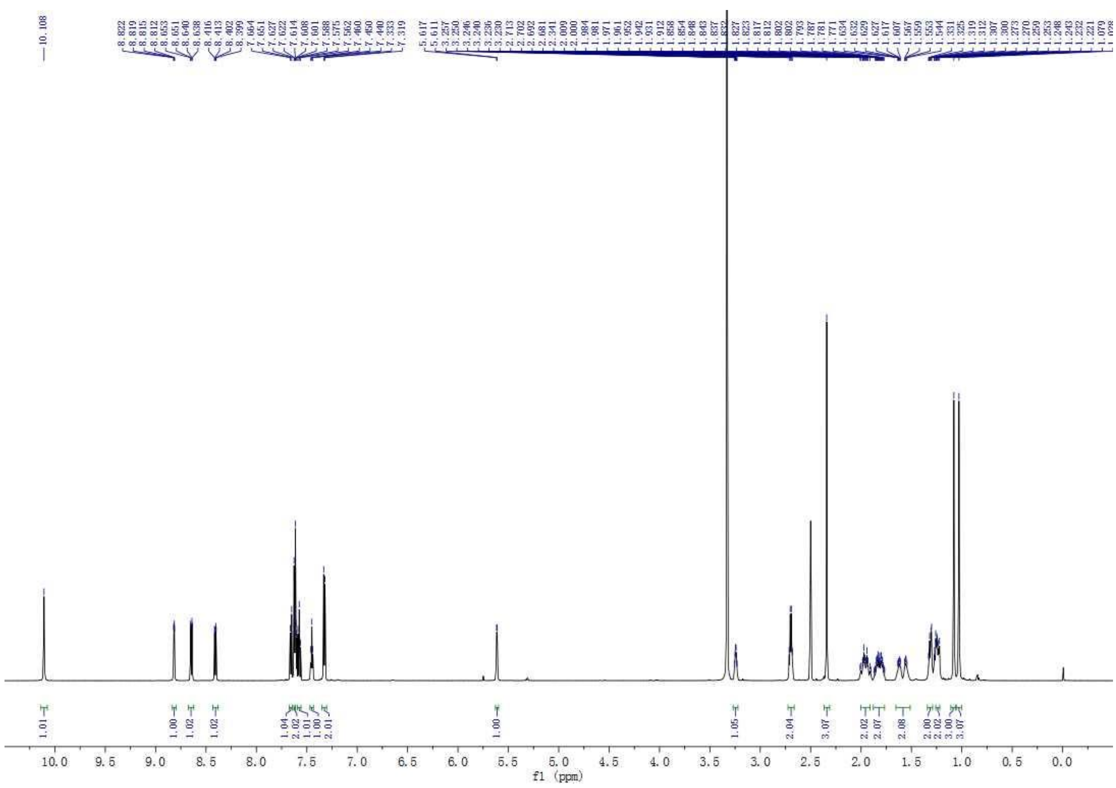

Supplementary Fig. 402. ¹H NMR of compound 6y. The sample has been recorded in 600 MHz, DMSO-D6 at 25 °C

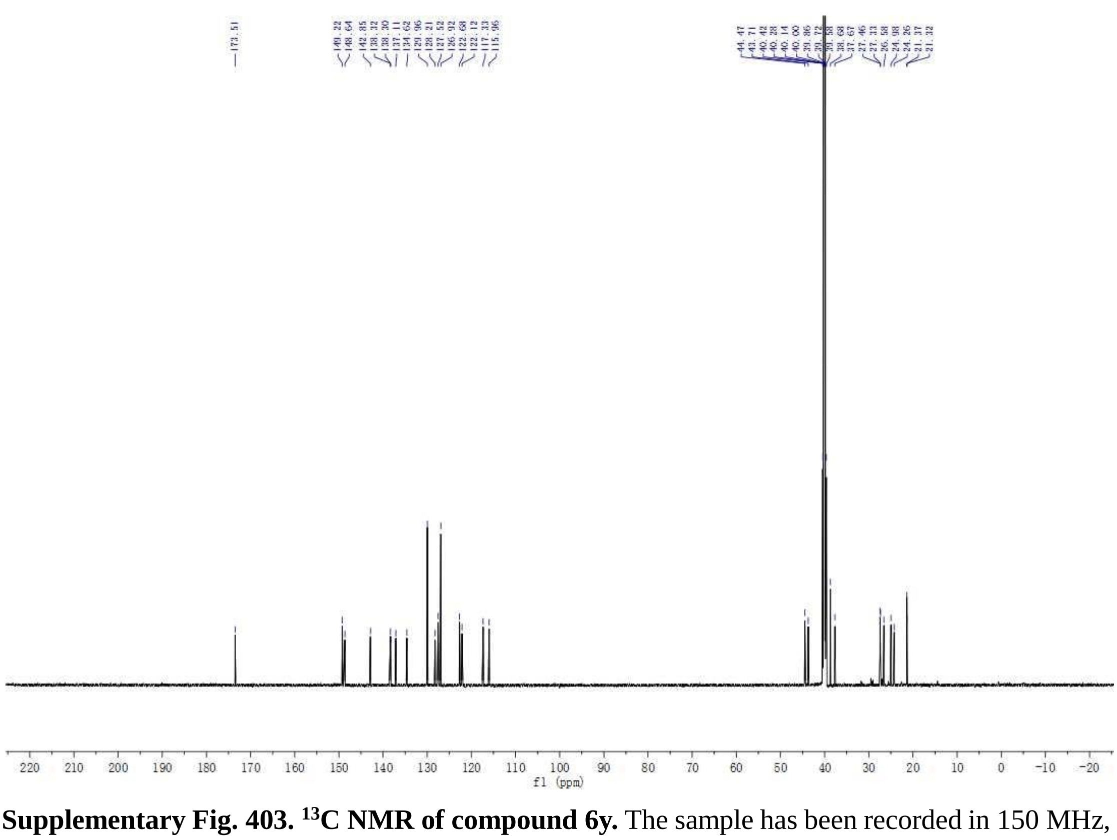

Supplementary Fig. 403. ¹³C NMR of compound 6y. The sample has been recorded in 150 MHz, DMSO-D6 at 25 °C

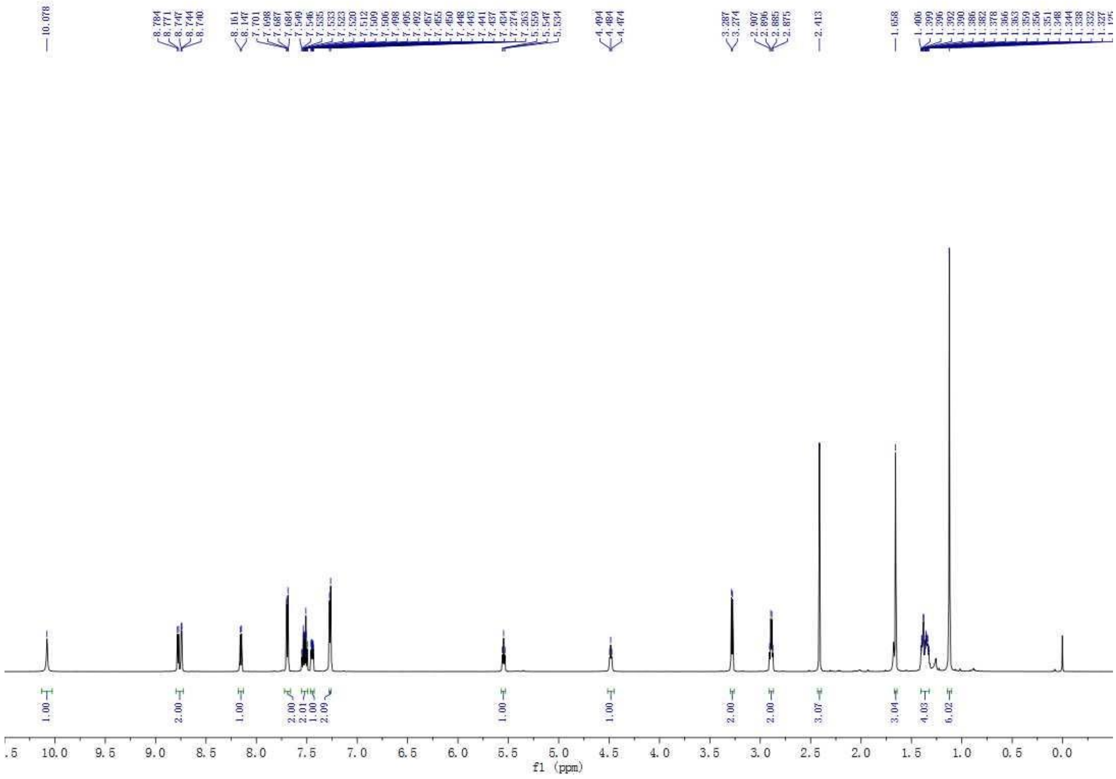

Supplementary Fig. 404. ^{1}H NMR of compound 6z. The sample has been recorded in 600 MHz, $CDCl_3$ at 25 °C

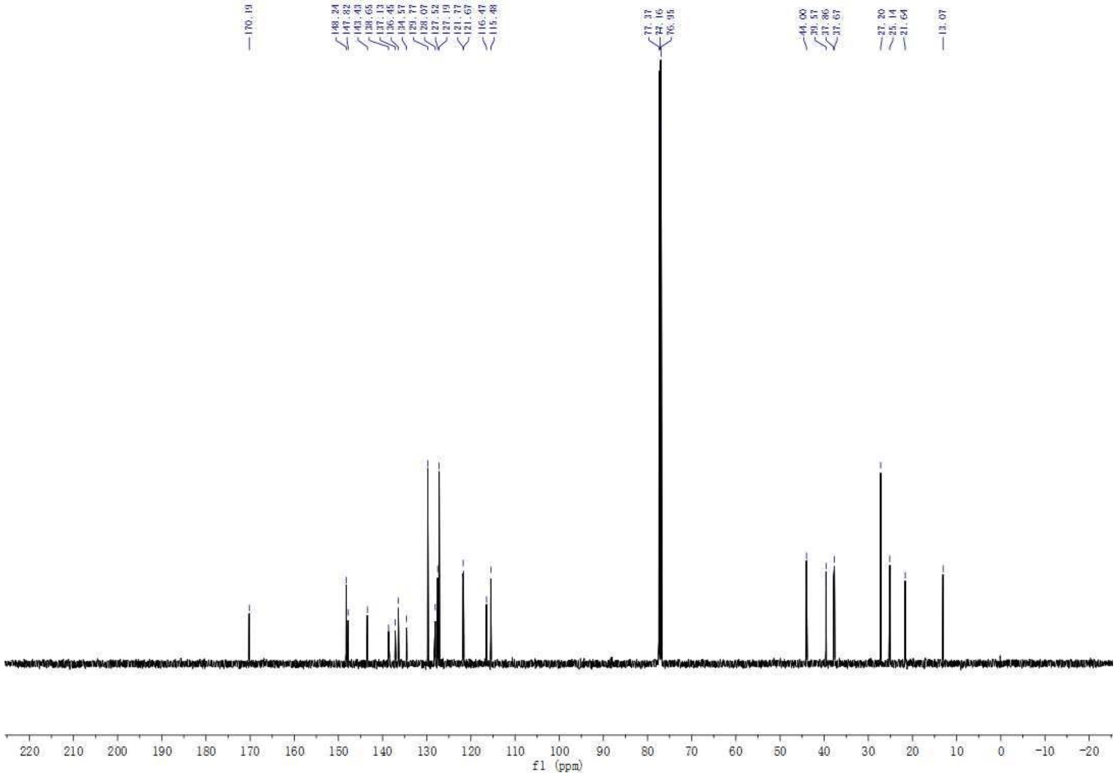

Supplementary Fig. 405. ^{13}C NMR of compound 6z. The sample has been recorded in 150 MHz, $CDCl_3$ at 25 °C

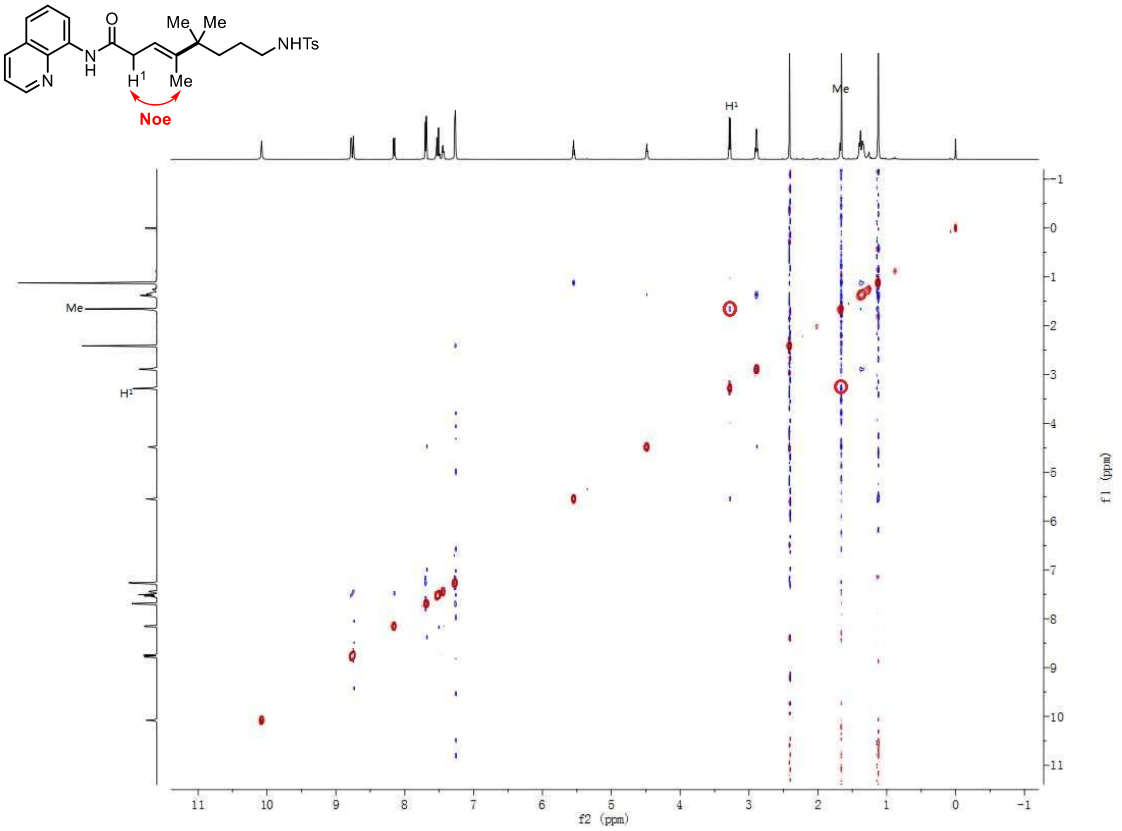

Supplementary Fig. 406. Noesy of compound 6z. The sample has been recorded in 600 MHz, $CDCl_3$ at 25 °C

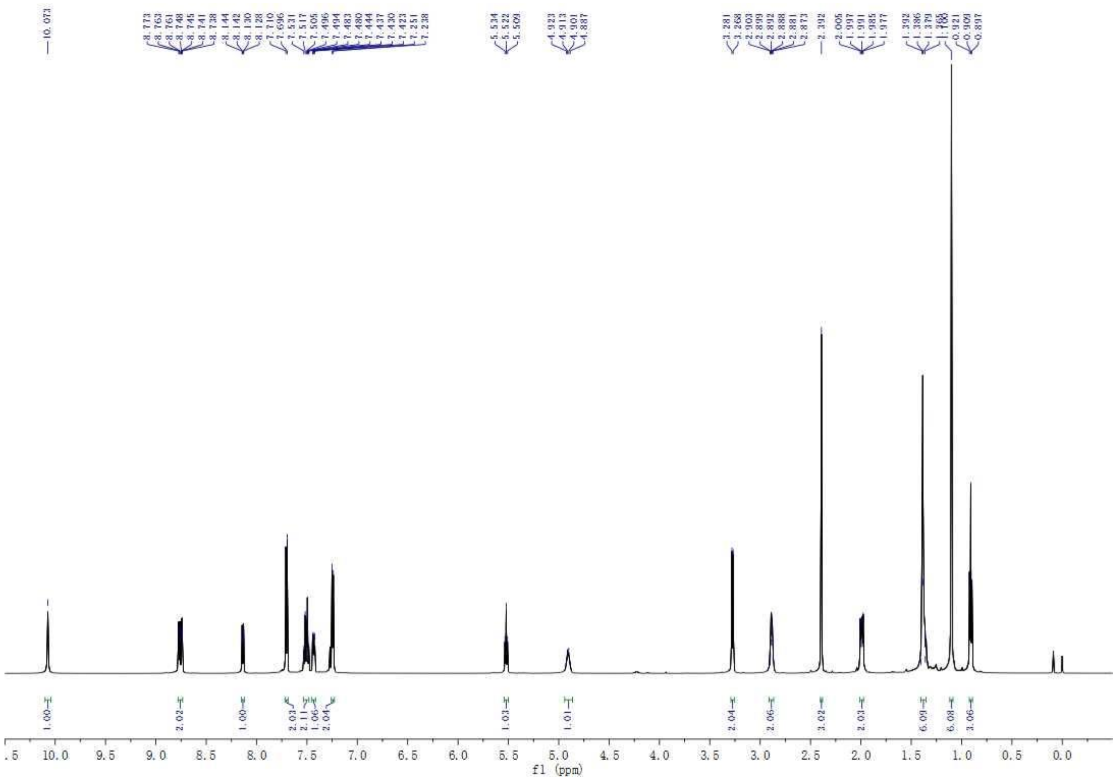

Supplementary Fig. 407. ^{1}H NMR of compound 6aa. The sample has been recorded in 600 MHz, $CDCl_3$ at 25 °C

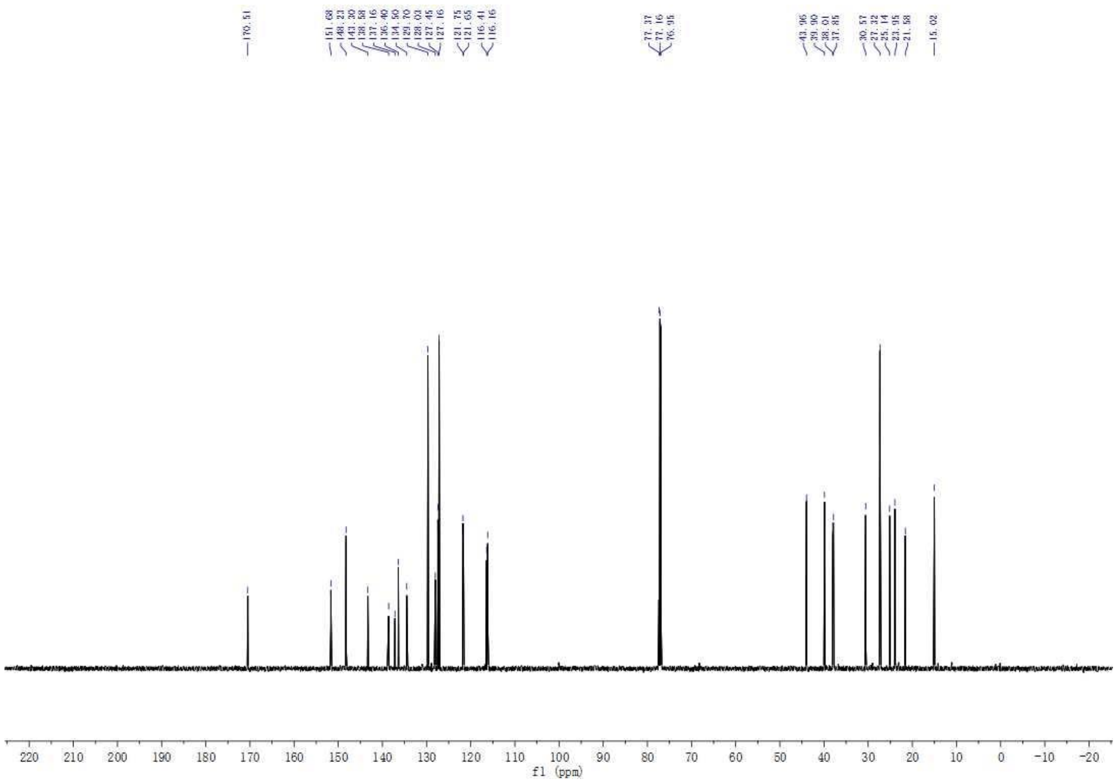

Supplementary Fig. 408. ^{13}C NMR of compound 6aa. The sample has been recorded in 150 MHz, $CDCl_3$ at 25 °C

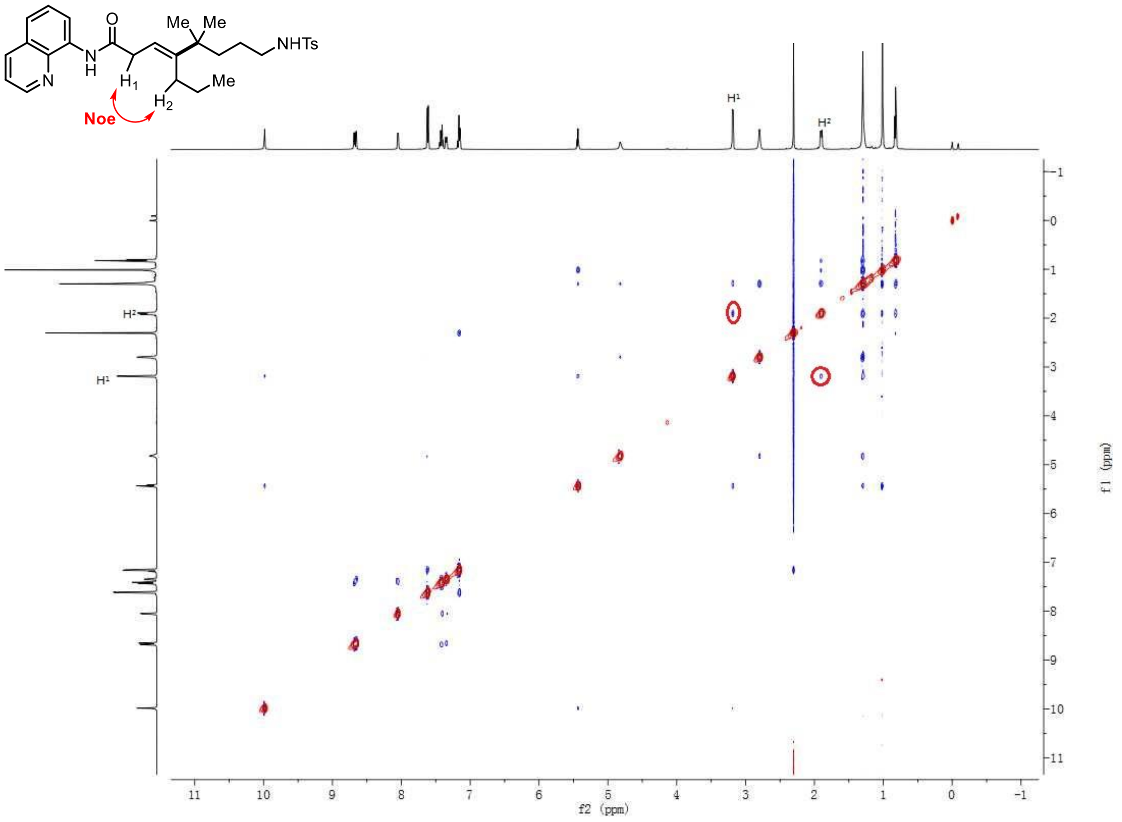

Supplementary Fig. 409. Noesy of compound 6aa. The sample has been recorded in 600 MHz, $CDCl_3$ at 25 °C

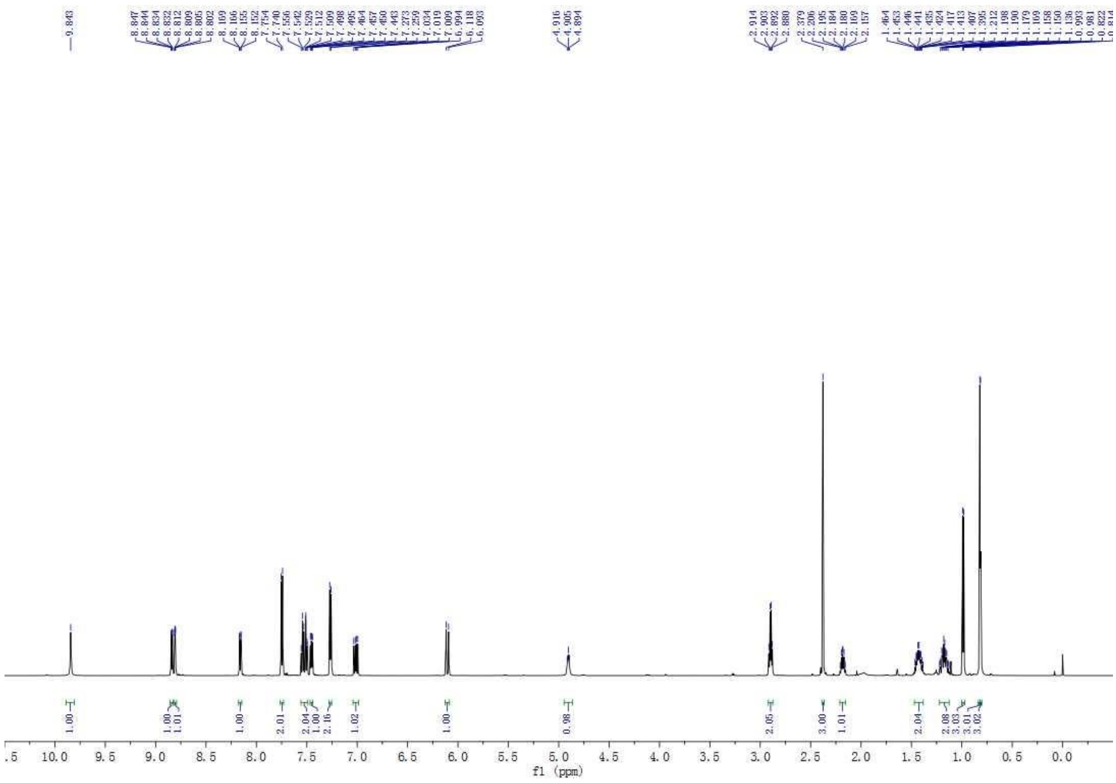

Supplementary Fig. 410. ¹H NMR of compound 7a. The sample has been recorded in 600 MHz, $CDCl_3$ at 25 °C

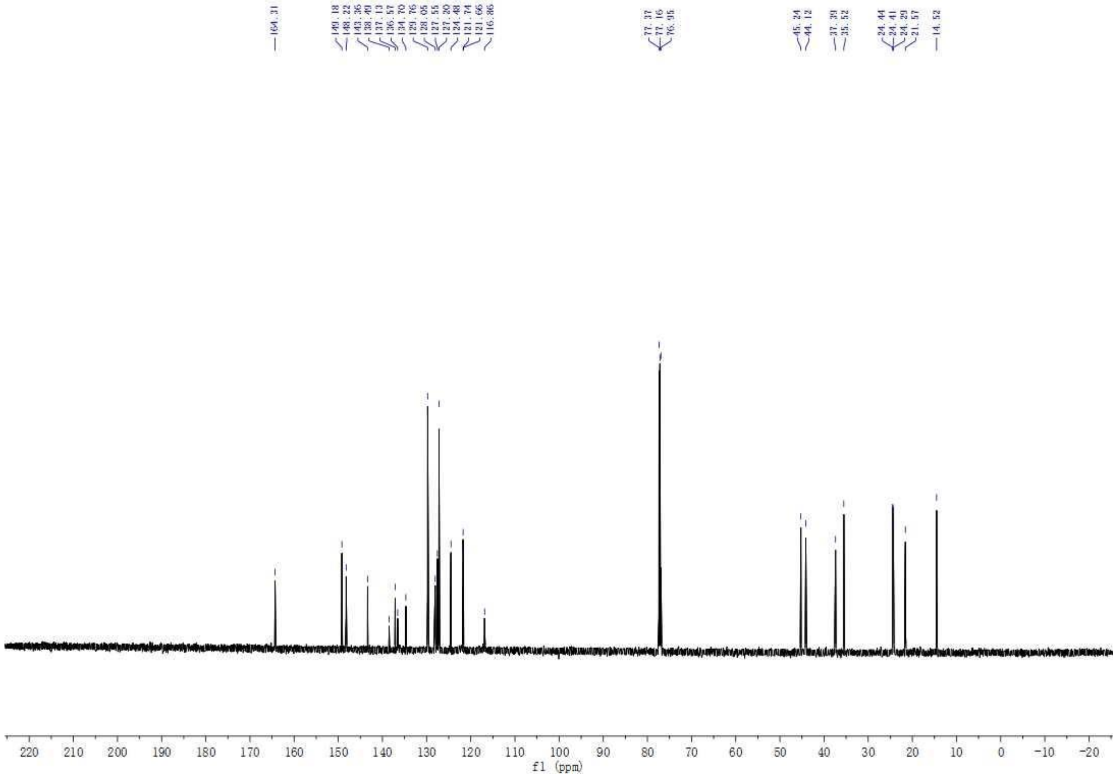

Supplementary Fig. 411. ¹³C NMR of compound 7a. The sample has been recorded in 150 MHz, $CDCl_3$ at 25 °C

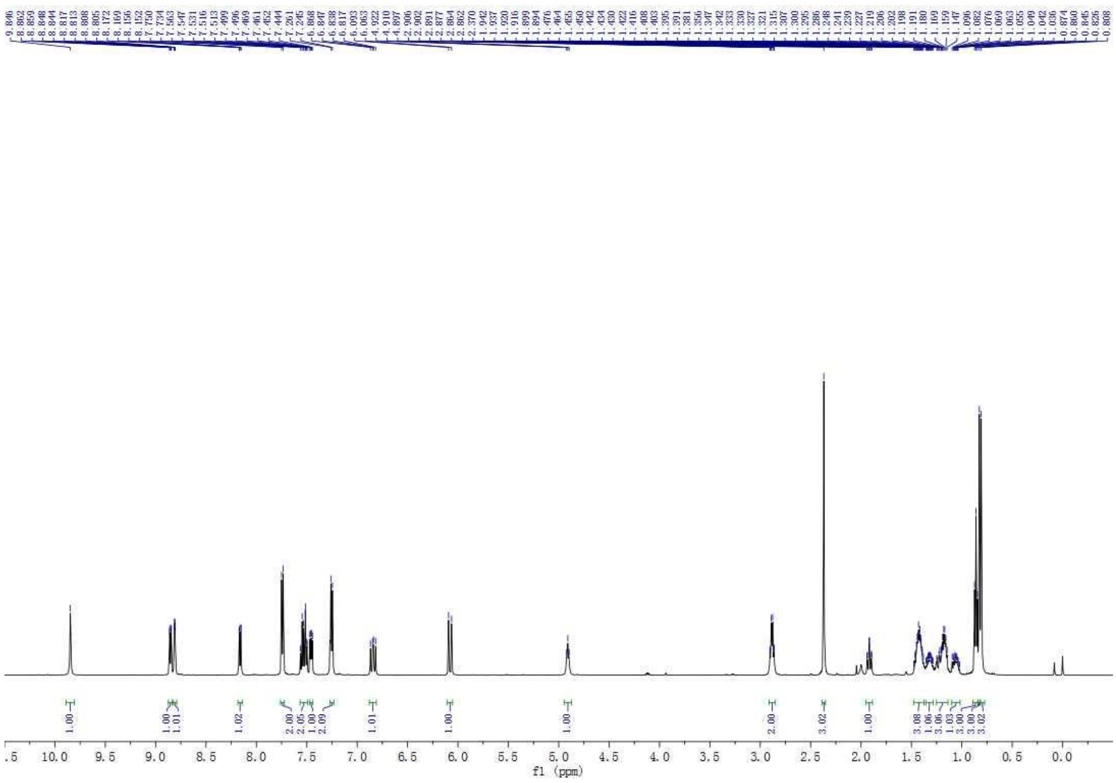

Supplementary Fig. 412. ^{1}H NMR of compound 7b. The sample has been recorded in 500 MHz, $CDCl_3$ at 25 °C

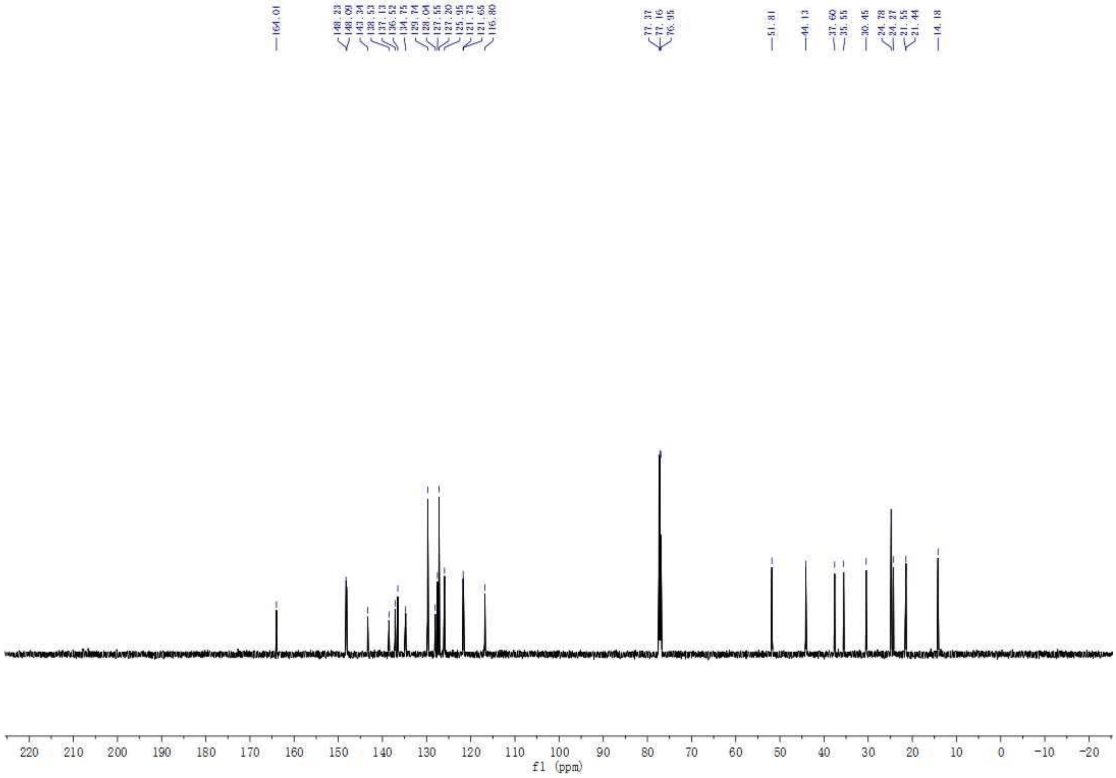

Supplementary Fig. 413. ^{13}C NMR of compound 7b. The sample has been recorded in 150 MHz, $CDCl_3$ at 25 °C

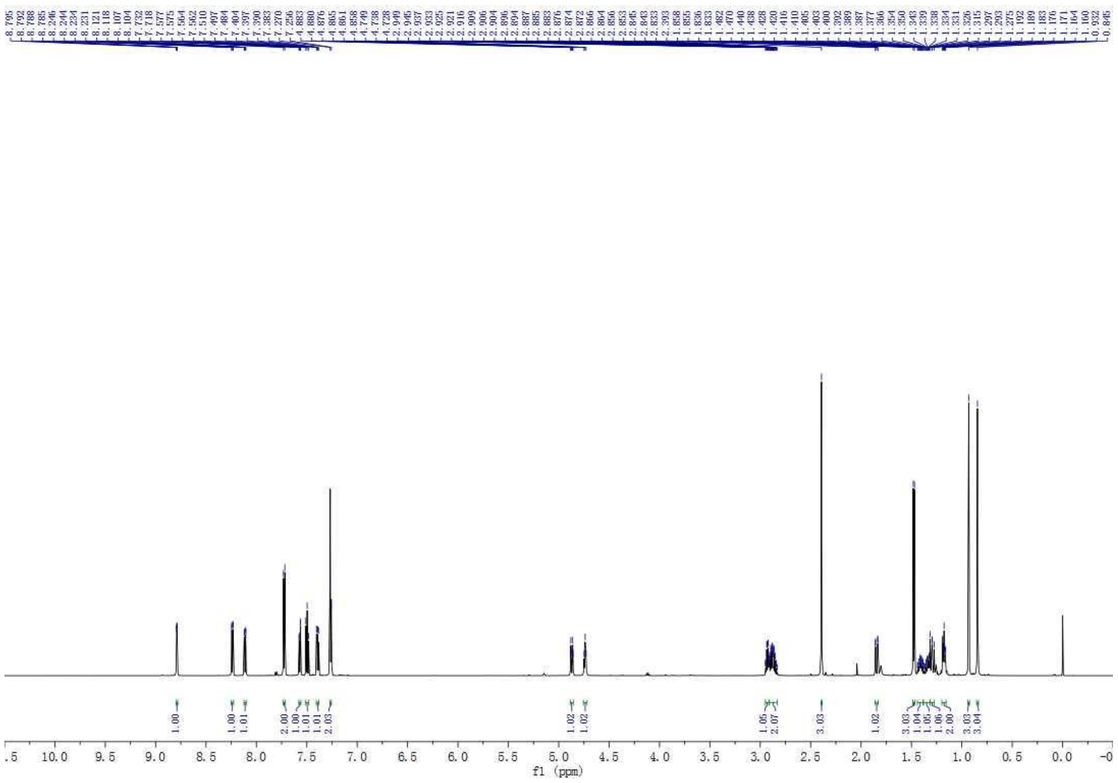

Supplementary Fig. 414. ^{1}H NMR of compound 8. The sample has been recorded in 600 MHz, $CDCl_3$ at 25 °C

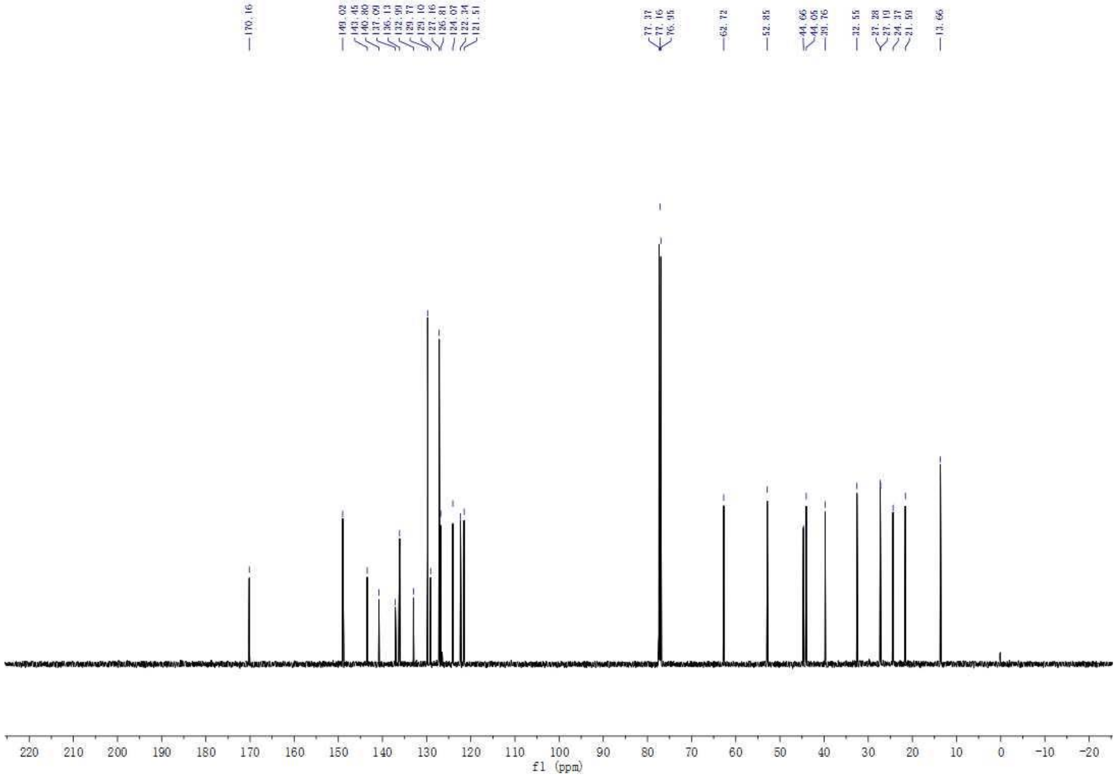

Supplementary Fig. 415. ^{13}C NMR of compound 8. The sample has been recorded in 150 MHz, $CDCl_3$ at 25 °C

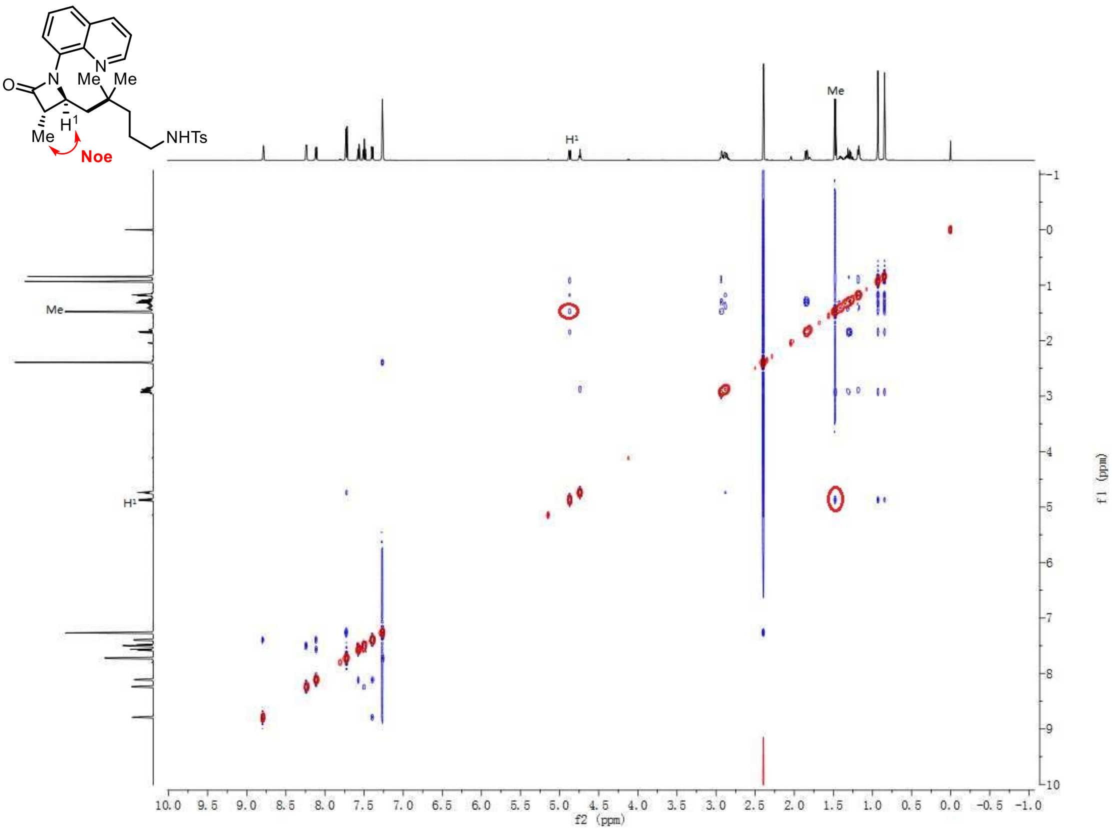

Supplementary Fig. 416. Noesy of compound 8. The sample has been recorded in 600 MHz, $CDCl_3$ at 25 °C

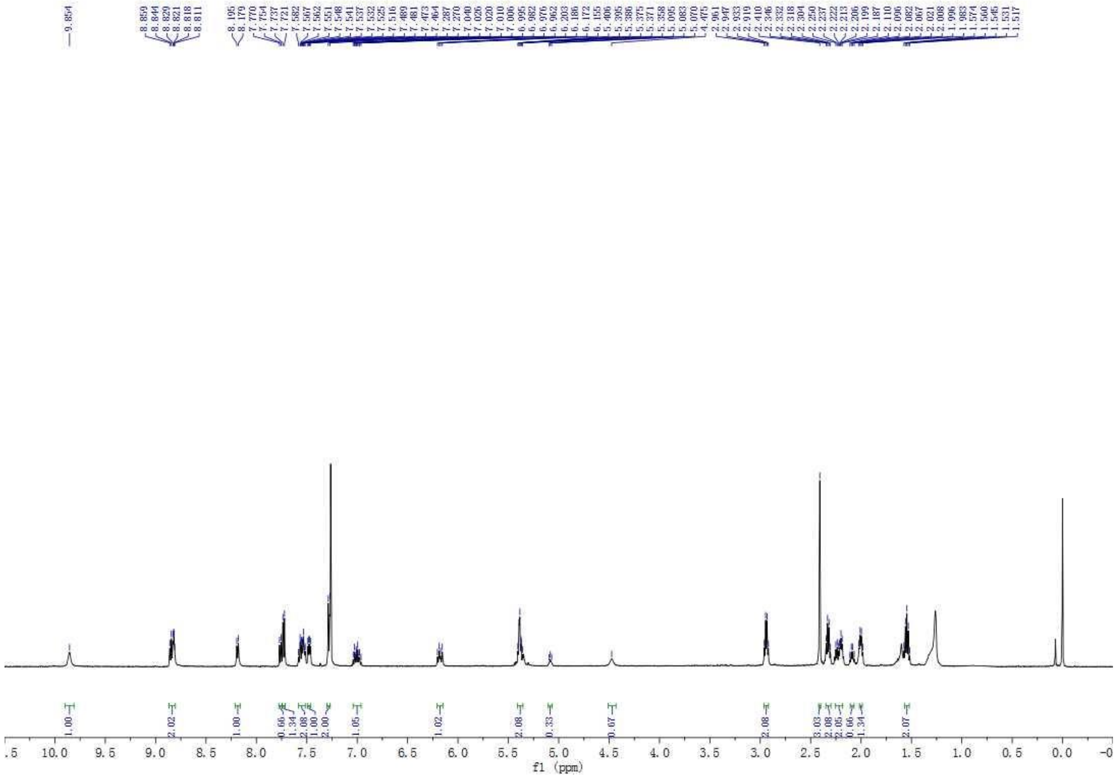

Supplementary Fig. 417. ^{1}H NMR of compound 12. The sample has been recorded in 500 MHz, $CDCl_3$ at 25 °C

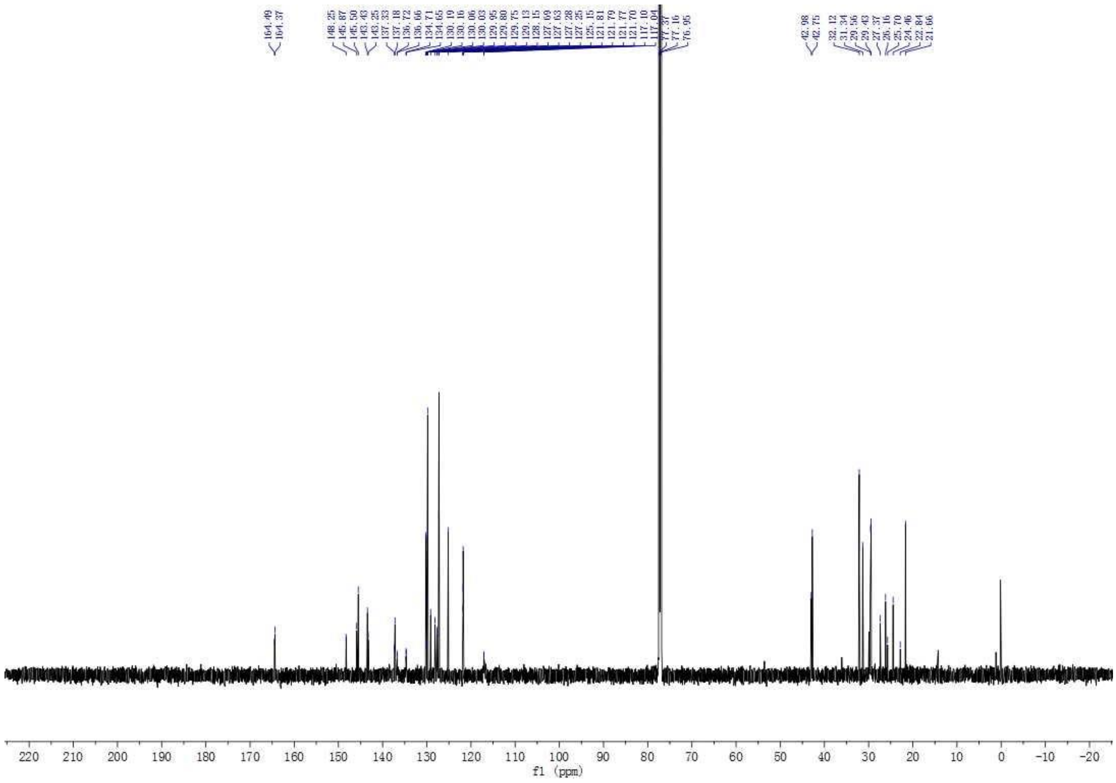

Supplementary Fig. 418. ^{13}C NMR of compound 12. The sample has been recorded in 150 MHz, $CDCl_3$ at 25 °C

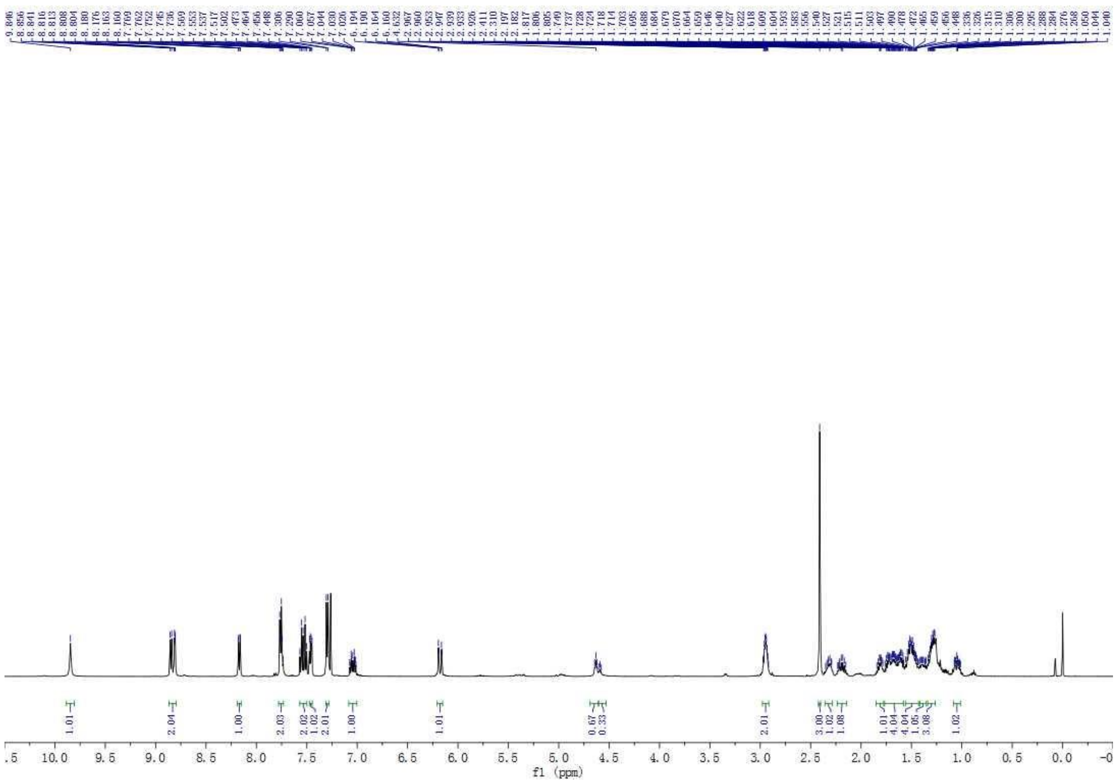

Supplementary Fig. 419. ^{1}H NMR of compound 14. The sample has been recorded in 500 MHz, $CDCl_3$ at 25 °C

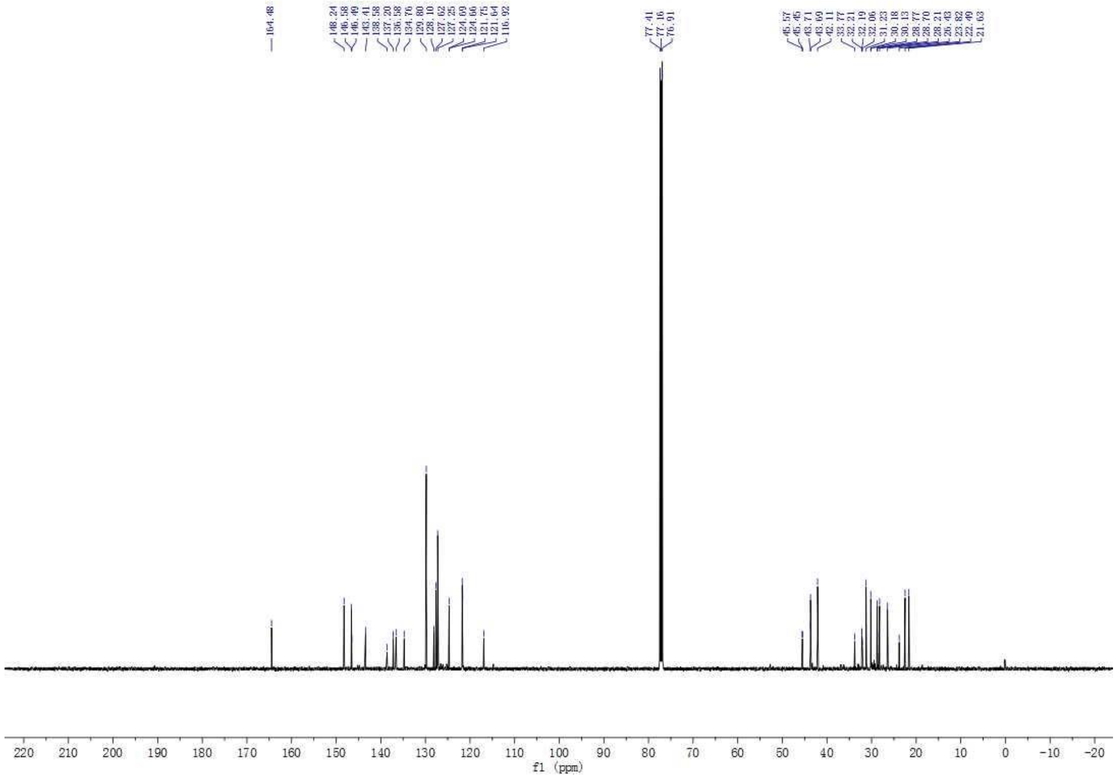

Supplementary Fig. 420. ^{13}C NMR of compound 14. The sample has been recorded in 125 MHz, $CDCl_3$ at 25 °C

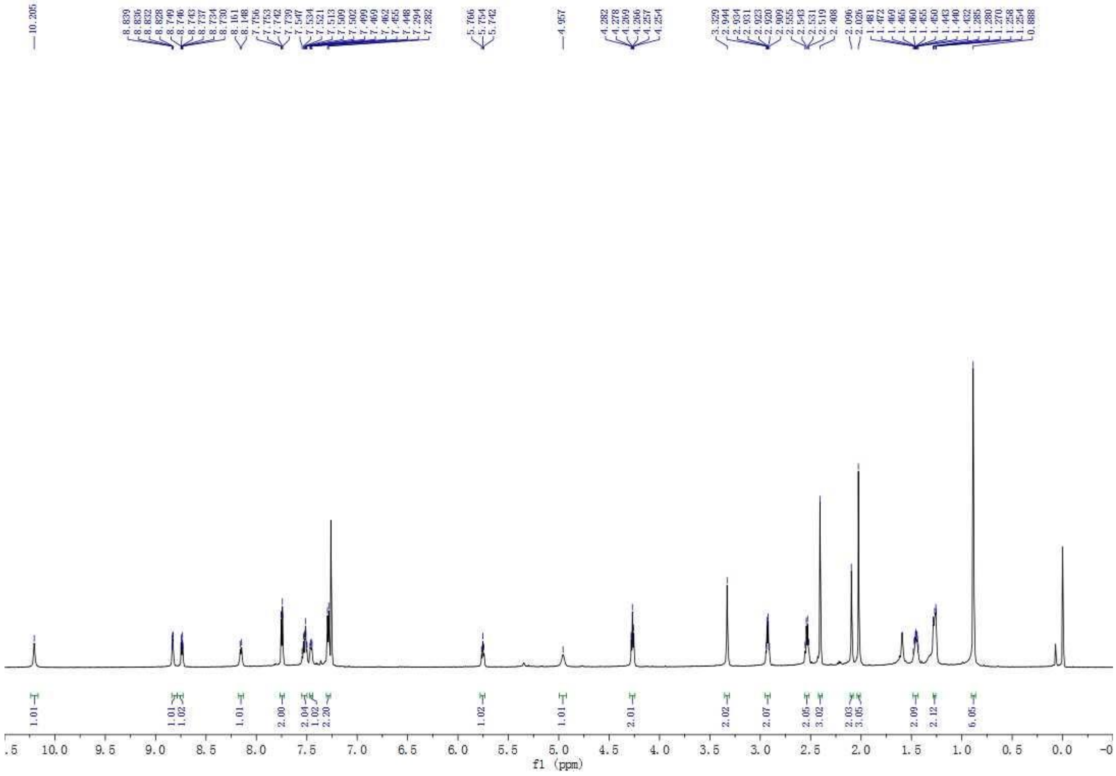

Supplementary Fig. 421. ^{1}H NMR of compound 17. The sample has been recorded in 600 MHz, $CDCl_3$ at 25 °C

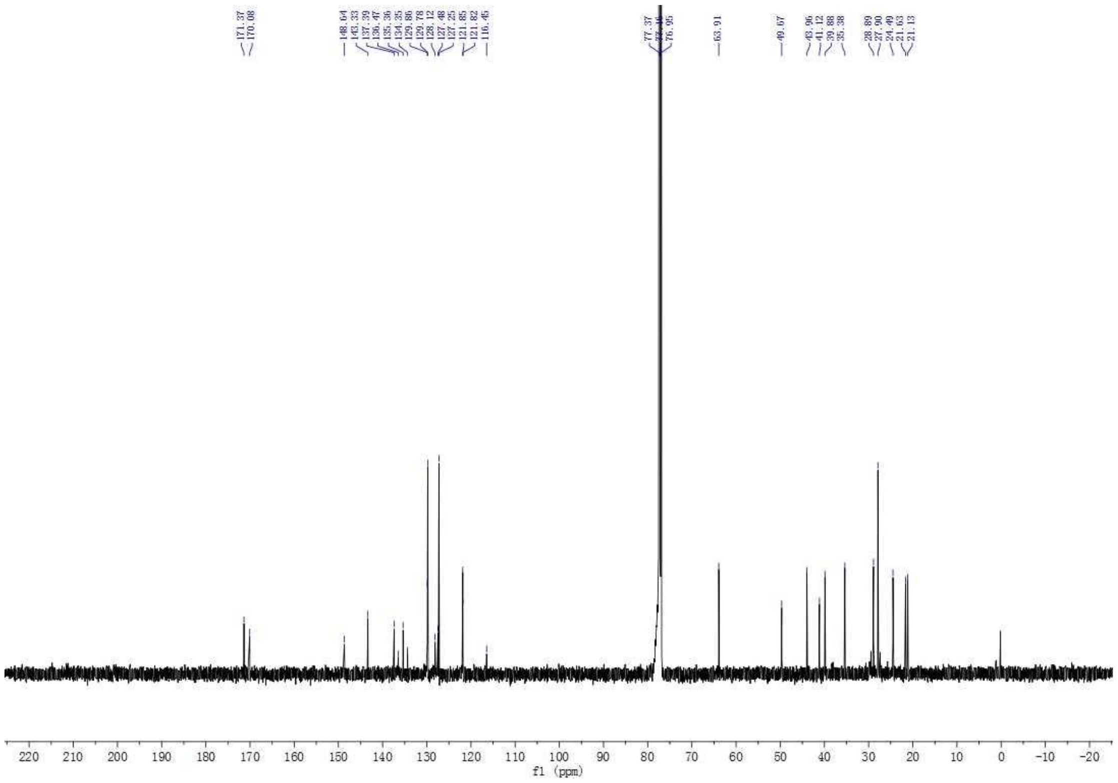

Supplementary Fig. 422. ^{13}C NMR of compound 17. The sample has been recorded in 150 MHz, $CDCl_3$ at 25 °C

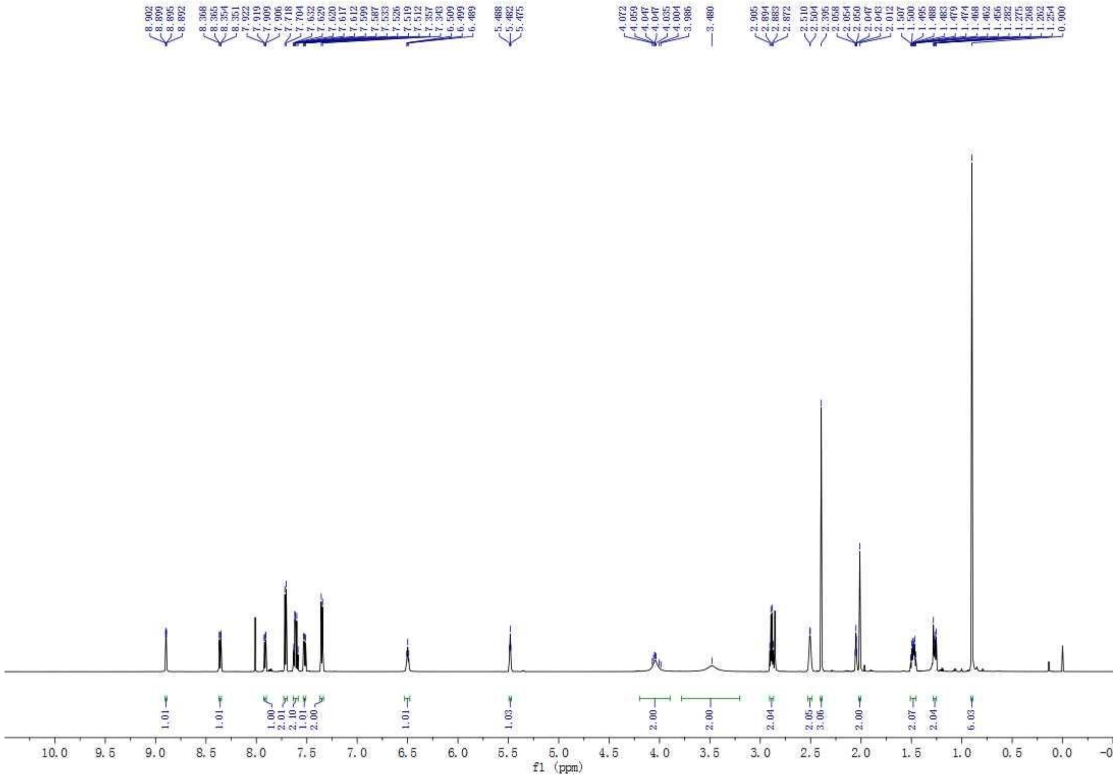

Supplementary Fig. 423. ^{1}H NMR of compound 18. The sample has been recorded in 600 MHz, Acetone-$CDCl_3$ at 25 °C

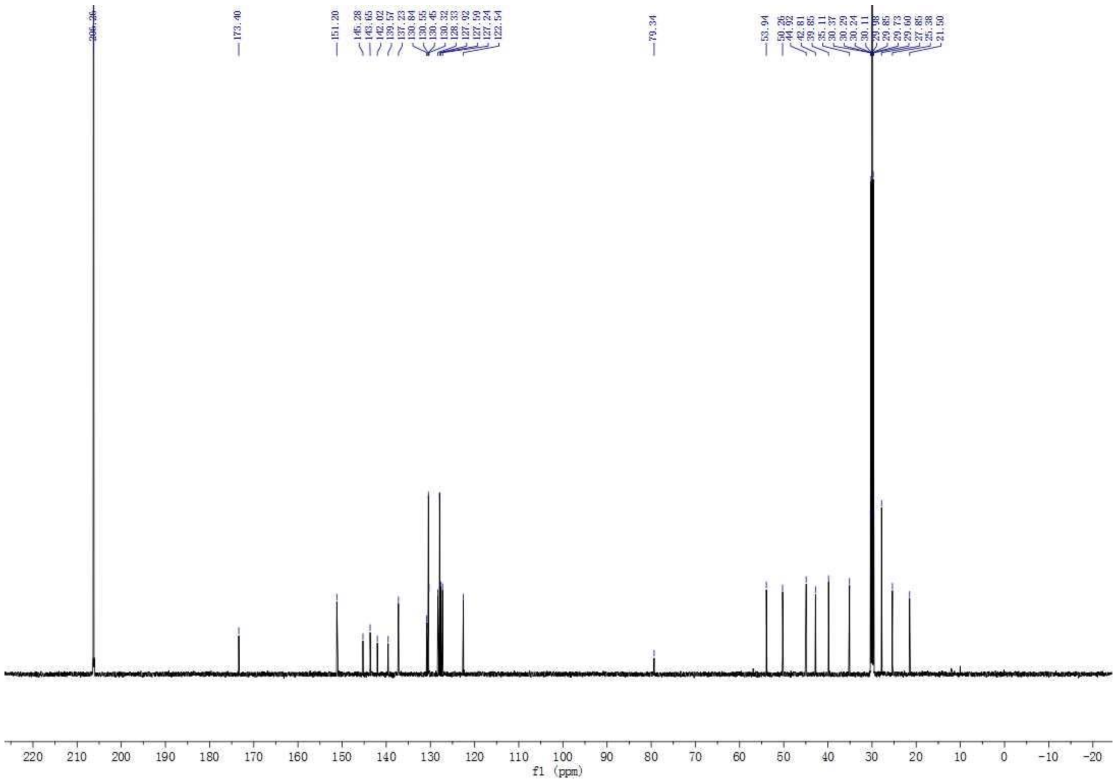

Supplementary Fig. 424. ^{13}C NMR of compound 18. The sample has been recorded in 150 MHz, Acetone-$CDCl_3$ at 25 °C

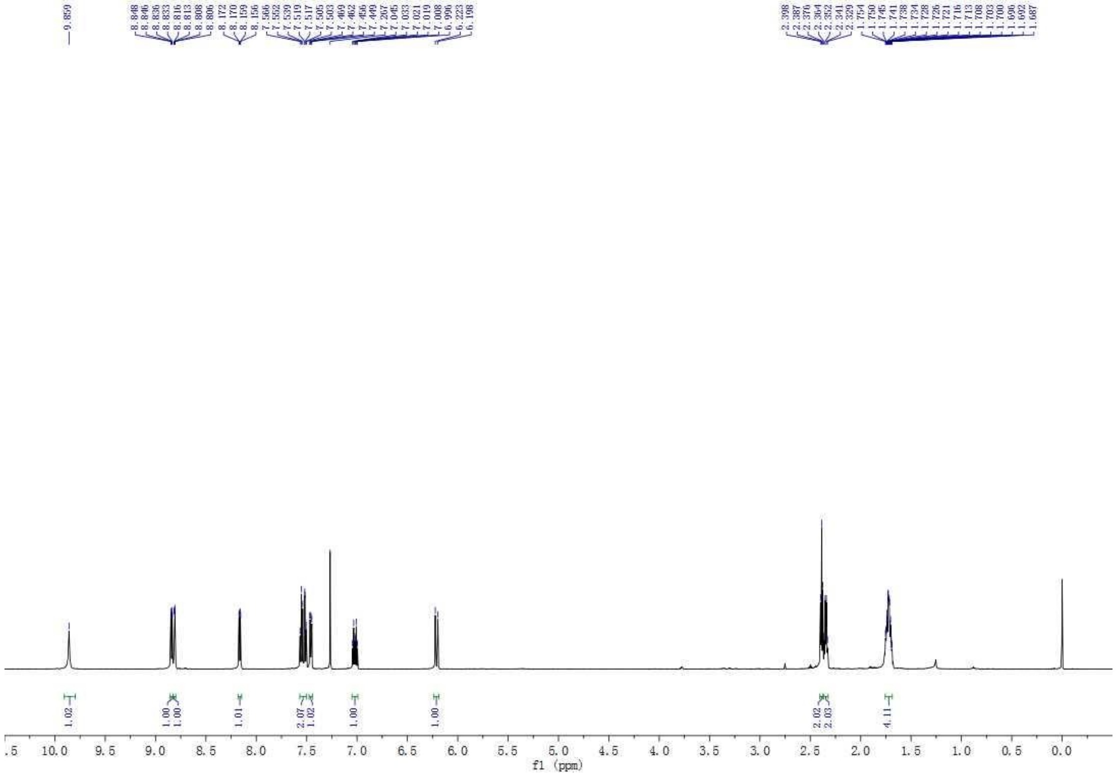

Supplementary Fig. 425. ^{1}H NMR of compound 19. The sample has been recorded in 600 MHz, $CDCl_3$ at 25 °C

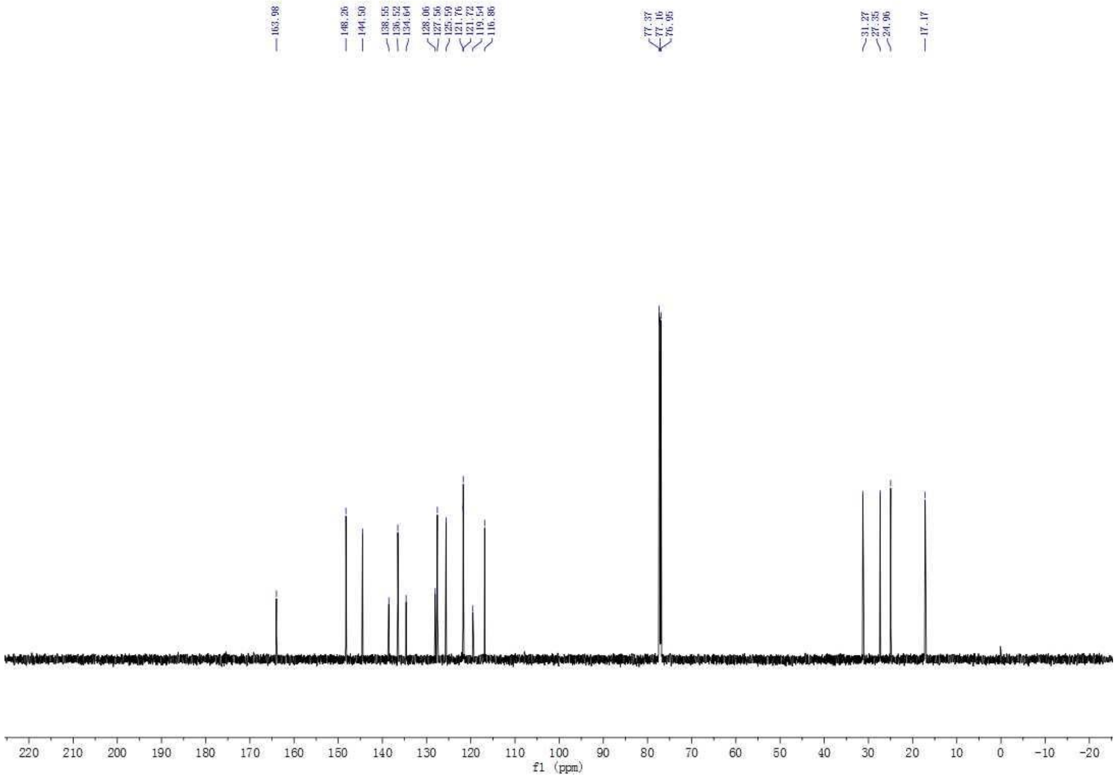

Supplementary Fig. 426. ^{13}C NMR of compound 19. The sample has been recorded in 150 MHz, $CDCl_3$ at 25 °C

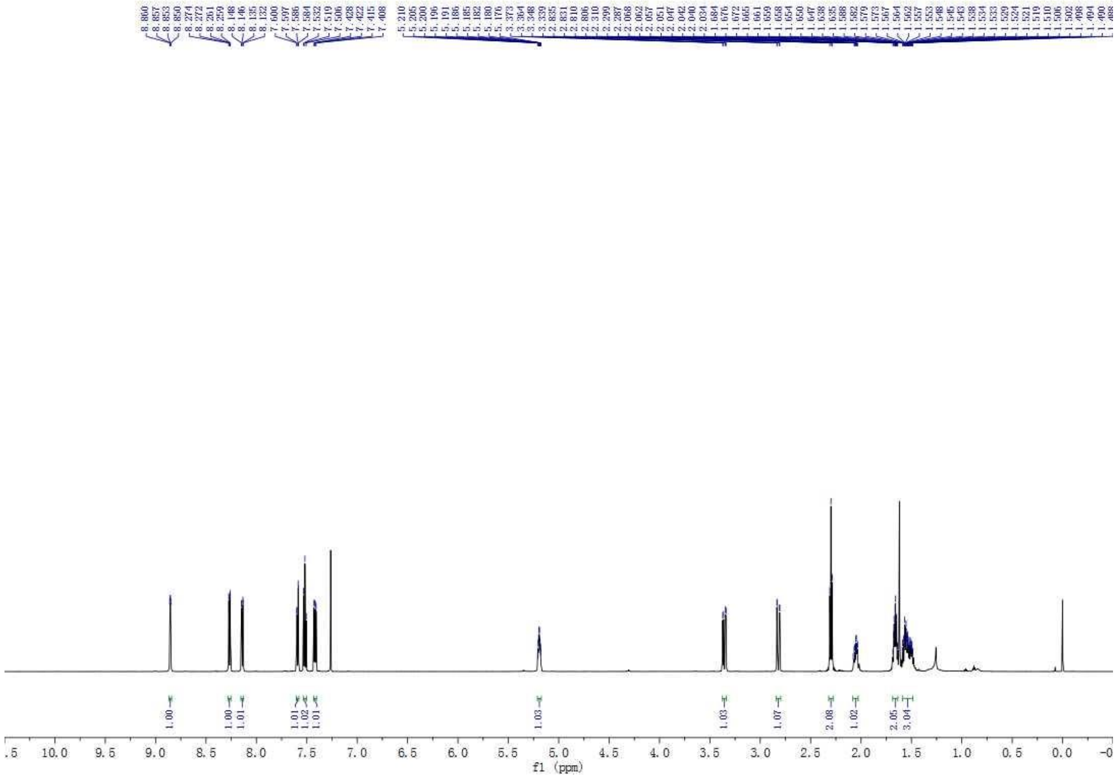

Supplementary Fig. 427. ^{1}H NMR of compound 20. The sample has been recorded in 600 MHz, $CDCl_3$ at 25 °C

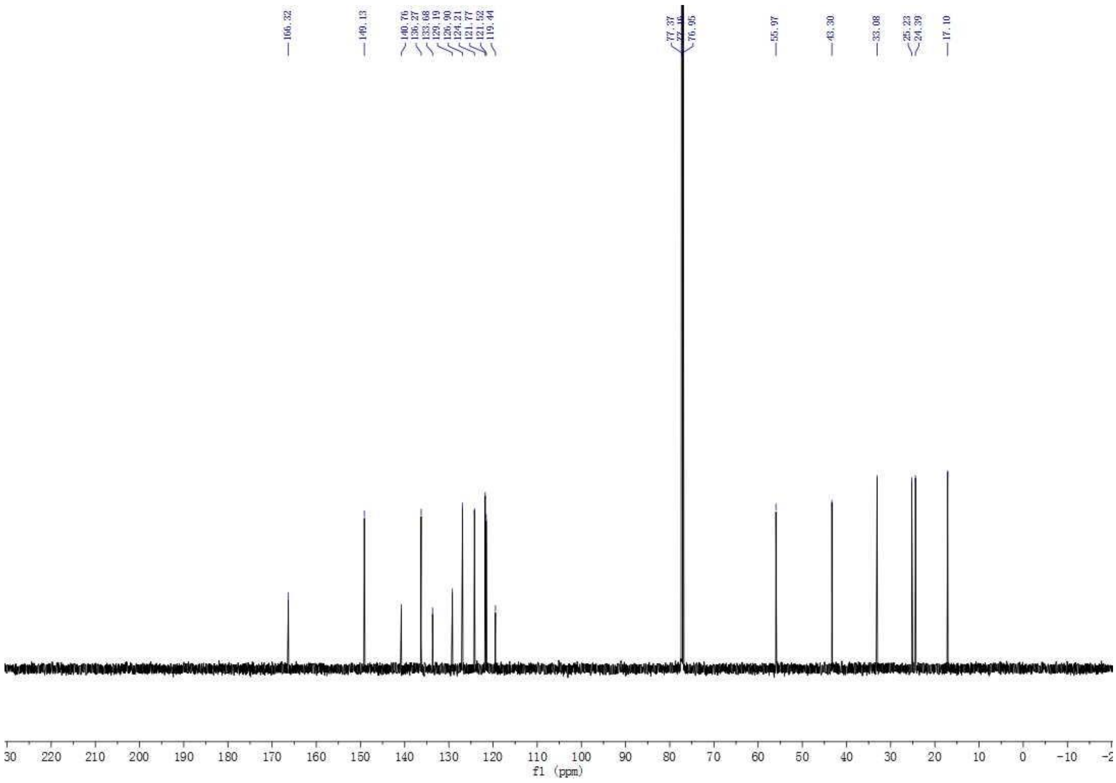

Supplementary Fig. 428. ^{13}C NMR of compound 20. The sample has been recorded in 150 MHz, $CDCl_3$ at 25 °C

Part 4. Supplementary references

[1] Y. Li, Y. Liang, J. Dong, Y. Deng, C. Zhao, Z. Su, W. Guan, X. Bi, Q. Liu, J. Fu, *J. Am. Chem. Soc.* **2019**, *141*, 18475.

[2] T. Yang, Y. Jiang, Y. Luo, J. J. H. Lim, Y. Lan, M. J. Koh, *J. Am. Chem. Soc.* **2020**, *142*, 21410.

[3] C. Tang, R. Zhang, B. Zhu, J. Fu, Y. Deng, L. Tian, W. Guan, X. Bi, *J. Am. Chem. Soc.* **2018**, *140*, 16929.

[4] Y. Deng, C. Zhao, Y. Zhou, H. Wang, X. Li, G.-J. Cheng, J. Fu, *Org. Lett.* **2020**, *22*, 3524.

[5] Z. Bai, S. Zheng, Z. Bai, F. Song, H. Wang, Q. Peng**,** G. Chen, G. He, *ACS Catal.* **2019**, *9*, 6502.

[6] H. Zhang**,** Y. Zhou, P. Tian, C. Jiang, *Org. Lett.* **2019**, *21*, 1921.

[7] A. Modak, E. N. Pinter, S. P. Cook, *J. Am. Chem. Soc.* **2019**, *141*, 18405.

[8] Y. Qin, Y. Han, Y. Tang, J. Wei, M. Yang, *Chem. Sci.* **2020**, *11*, 1276.

[9] Z. Zhang, L. M. Statemana, D. A. Nagib, *Chem. Sci.* **2019**, *10*, 1207.

[10] D. Bafaluy, J. M. Muñoz-Molina, I. Funes-Ardoiz, S. Herold, A. J. D. Aguirre, H. Zhang, F. Maseras, T. R. Belderrain, P. J. Pérez, K. Muñiz, *Angew. Chem. Int. Ed.* **2019**, *58*, 8912.

[11] S. Shi, X. Yang, M. Tang, J. Hu, T.-P. Loh, *Org. Lett.* **2021**, *23*, 4018.

[12] H. Zhang, F. Yu, C. Li, P. Tian, Y. Zhou, Z.-Y. Cao, *Org. Lett.* **2021**, *23*, 4721.

[13] B. Xu, U. K. Tambar, *ACS Catal.* **2019**, *9*, 4627.

[14] Y. Zhu, J. Shi, W. Yu, *Org. Lett.* **2020**, *22*, 8899.

[15] Q.-Q. Min, J.-W. Yang, M.-J. Pang, G.-Z. Ao, F. Liu, *Org. Chem. Front.* **2021**, *8*, 249.

[16] K. Antien, A. Lacambra, F. P. Cossío, S. Massip, D. Deffieux, L. Pouységu, P. A. Peixoto, S. Quideau, *Chem. Eur. J.* **2019**, *25*, 11574.

[17] O. G. Mountanea, D. Limnios, M. G. Kokotou, A. Bourboula, G. Kokotos, *Eur. J. Org. Chem.* **2019**, *10*, 2010.

[18] J. E. Wilson, A. D. Casarez, D. W. C. MacMillan, *J. Am. Chem. Soc.* **2009**, *131*, 11332.

[19] T. Cochet, V. Bellosta, D. Roche, J.-Y. Ortholand, A. Greinerand, J. Cossy, *Chem. Commun.* **2012**, *48*, 10745.

[20] Z. Bai, H. Zhang, H. Wang, H. Yu, G. Chen, G. He, *J. Am. Chem. Soc.* **2021**, *143*, 1195.
