# Supplementary material for: Divergent regioselective Heck-type reaction of unactivated alkenes and N-fluoro-sulfonamides
Source: Nat Commun. 2022 Oct 22;13:6297. doi: 10.1038/s41467-022-33996-1 (PMC9588056; doi:10.1038/s41467-022-33996-1)
Supplement: Supplementary file 3 — Description of Additional Supplementary Files [file 41467_2022_33996_MOESM3_ESM.docx]

**Description of Additional Supplementary Files**

**File Name: Supplementary Data 1
Description:** Cartesian coordinates for the calculated structures.
