# Supplementary material for: Divergent regioselective Heck-type reaction of unactivated alkenes and N-fluoro-sulfonamides
Source: Nat Commun. 2022 Oct 22;13:6297. doi: 10.1038/s41467-022-33996-1 (PMC9588056; doi:10.1038/s41467-022-33996-1)
Supplement: Supplementary file 4 — Supplementary Data 1 [file 41467_2022_33996_MOESM4_ESM.pdf]

Cartesian Coordinates of Optimized Structures

1′

O	-4.09296700	-3.11557800	-2.33609900
N	-4.18687400	-1.30698700	-0.89758600
C	-2.44585500	-1.35476900	-2.49691700
C	-1.84566200	-0.34501700	-1.54751300
C	-3.65240100	-2.05198100	-1.90398900
C	-0.83932900	-0.86268900	-0.55144200
H	-1.70238600	-2.11232500	-2.78770100
H	-1.57620600	0.60626300	-2.01617300
C	-5.32241000	1.52732200	1.58227500
C	-5.79195000	-0.55869400	0.67574900
C	-6.97840900	-0.67996800	1.43720500
C	-7.30509600	0.39342200	2.29693400
C	-6.48491900	1.49257100	2.37295600
H	-4.63651000	2.37504600	1.61729000
C	-5.37114200	-1.58926300	-0.21927400
C	-7.75935000	-1.85150000	1.30510900
H	-8.21300600	0.33127500	2.89656500
H	-6.71488500	2.32764500	3.02946000
C	-7.34528500	-2.83851600	0.44507700
C	-6.16235600	-2.72292500	-0.31642100
H	-8.67240600	-1.95232600	1.89017800
H	-7.93836500	-3.74571900	0.33901000
H	-5.86885600	-3.52443100	-0.98518800
N	-4.99660300	0.54412000	0.76807900
H	-2.78309000	-0.86947800	-3.42492100
Cu	-3.33450100	0.30213500	-0.42076000
H	-1.00385500	-0.41822600	0.44318100
H	-0.98404300	-1.95037000	-0.43414200
C	0.64030900	-0.57597400	-0.92080200
C	1.48909200	-1.32469100	0.12107200
H	1.16932800	-0.99829800	1.12647700
H	1.24620600	-2.40023500	0.05732200
C	0.94979500	-1.08586500	-2.32611200

H	0.78713900	-2.17176800	-2.40339100
H	0.32096300	-0.59469500	-3.08263700
H	1.99340700	-0.88347100	-2.60297500
C	0.90763200	0.92556100	-0.84189500
H	1.93888800	1.16059600	-1.13868500
H	0.24918700	1.49520900	-1.51248600
H	0.75487900	1.30320700	0.18010400
C	2.99799900	-1.15734400	0.01174000
H	3.36396400	-1.49292100	-0.97079900
H	3.27852400	-0.09882400	0.11404000
C	3.71270700	-1.95946500	1.08268100
H	3.46323100	-3.02405800	0.98937700
H	3.38822300	-1.64676000	2.08646900
N	5.18059200	-1.87967900	0.94035600
S	6.00705400	-0.72389200	1.79944900
O	5.08101000	0.39265900	1.99380300
O	6.65230400	-1.30419700	2.98084200
C	7.28112500	-0.23393400	0.67298100
C	8.61549200	-0.36200900	1.03556700
C	6.91277100	0.32235200	-0.55277200
C	9.59727800	0.06987200	0.14925100
H	8.88415700	-0.79372500	1.99718600
C	7.90369200	0.74716300	-1.42070900
H	5.86135800	0.41439300	-0.82405900
C	9.25922600	0.62758700	-1.08378200
H	10.64744900	-0.02760000	0.42304600
H	7.62983300	1.18234600	-2.38198300
C	10.31141400	1.09125300	-2.04121400
H	10.26520400	0.52581900	-2.98220000
H	10.17032300	2.14911400	-2.30133200
H	11.31871400	0.97418800	-1.62528800
H	5.65702600	-2.77821600	1.02231400
C	-1.91887600	4.15574600	-0.31384100
H	-0.84344600	3.92325000	-0.21797900
C	-2.09452400	5.28657000	-1.31545500

H	-1.59181400	6.20271000	-0.97649800
H	-1.68681900	5.02605500	-2.30244600
H	-3.16130100	5.52634000	-1.44752900
C	-2.45585300	4.55526300	1.05164900
H	-3.53504500	4.77174300	0.99045900
H	-2.31133300	3.76145500	1.79559700
H	-1.96003200	5.46136800	1.42734400
C	-2.60379400	2.89149200	-0.83165200
H	-3.69166900	3.11765000	-0.94195500
H	-2.22898900	2.69695400	-1.86323400
O	-2.37584800	1.81206300	0.01074900

TS1

O	-5.86765400	-2.83224300	-0.30493800
N	-4.70198400	-0.81776200	-0.29321200
C	-4.82626600	-2.16372500	-0.27411100
C	-4.18030600	3.09176200	0.54336400
C	-5.59966000	1.42379300	-0.21306400
C	-6.64616800	2.36178200	-0.42929100
C	-6.38569300	3.71586400	-0.11695800
C	-5.15924400	4.08789200	0.37074200
H	-3.18961200	3.34987100	0.92236300
C	-5.79416900	0.02689400	-0.50022500
C	-7.88817100	1.92648100	-0.94257700
H	-7.17557400	4.45093000	-0.27268000
H	-4.93291800	5.12223000	0.61810600
C	-8.05756000	0.59581200	-1.22950600
C	-7.03172000	-0.34471800	-1.01301300
H	-8.68386300	2.65342300	-1.10186200
H	-9.00588000	0.24266500	-1.63295300
H	-7.21296200	-1.38678600	-1.24811700
N	-4.38992200	1.82221100	0.26458100
Cu	-3.08649900	0.04736300	0.36333600
N	5.14340800	-1.49369300	0.29427800
S	5.51962300	-0.09247300	1.11061500

O	4.31291600	0.73715700	1.07854800
O	6.11586400	-0.36343300	2.42318300
C	6.76171400	0.60715000	0.06773900
C	8.04855700	0.79609600	0.55533600
C	6.40871100	0.99366400	-1.22610700
C	9.00099600	1.37759200	-0.27482300
H	8.30181800	0.49326600	1.56905300
C	7.37102300	1.57058000	-2.03656300
H	5.39353500	0.83908300	-1.59036800
C	8.67930200	1.77076100	-1.57432600
H	10.01433900	1.52980600	0.09542600
H	7.11116400	1.87646000	-3.05005300
C	9.70088800	2.39573600	-2.47095500
H	9.85316700	1.79195500	-3.37611500
H	9.37591600	3.39020300	-2.80595700
H	10.66911800	2.50532800	-1.96910900
H	5.93137200	-2.14089800	0.35577300
C	-3.50638100	-2.91043600	-0.16021000
H	-3.36680100	-3.25555300	0.87665000
H	-3.59199300	-3.83293900	-0.76095600
C	-1.02924700	-2.45900200	-0.14437800
H	-0.95780500	-3.40043700	0.42634700
H	-1.11008400	-1.72240000	0.90326600
C	0.24721100	-2.07801200	-0.90282400
C	0.19776700	-0.62953500	-1.38478600
H	0.21928500	0.08524800	-0.54955500
H	1.05436600	-0.40893900	-2.03525500
H	-0.70559600	-0.42507600	-1.97639100
C	0.37376200	-3.00924800	-2.11085000
H	0.36559300	-4.06439100	-1.80086700
H	-0.46218800	-2.85731900	-2.80946300
H	1.30572100	-2.82560300	-2.66377300
C	1.42406600	-2.29566600	0.06528200
H	1.49220700	-3.37616200	0.28564400
H	1.18389100	-1.80860600	1.02440800

C	2.78173200	-1.78742800	-0.39227500
H	2.75427000	-0.69628500	-0.52944200
H	3.07641400	-2.22201200	-1.36011000
C	3.84504700	-2.11367300	0.63907500
H	4.01691400	-3.19560400	0.69250000
H	3.51220400	-1.80355700	1.64294700
C	-2.30251400	-2.19767400	-0.63651200
H	-2.43023400	-1.52148800	-1.48737400
C	-0.74929200	0.16879600	1.95178800
O	-0.84603900	-1.09133000	2.11409100
O	-1.52977600	0.84481200	1.22930400
C	0.39163800	0.86386700	2.63102400
H	0.22888600	1.94409400	2.68874400
H	0.55093700	0.45043300	3.63294600
H	1.30977900	0.67766300	2.05265600

2′

O	-5.90115800	-2.64036300	0.14784400
N	-4.60847000	-0.75748900	-0.25708300
C	-4.81138300	-2.08070100	-0.06118800
C	-3.95187400	3.17460100	0.43519500
C	-5.43467400	1.51796500	-0.22414800
C	-6.45653300	2.47908100	-0.46175200
C	-6.15023200	3.83633200	-0.21004800
C	-4.90437000	4.19158100	0.23937100
H	-2.94539600	3.41880000	0.78086500
C	-5.67533600	0.11572800	-0.45143800
C	-7.71992200	2.06366800	-0.93690800
H	-6.92079500	4.58797000	-0.38295500
H	-4.64245700	5.22820100	0.43727000
C	-7.93383000	0.72769300	-1.16911700
C	-6.93395600	-0.23336600	-0.93067700
H	-8.49577100	2.80827700	-1.11172100
H	-8.89872000	0.38926100	-1.54552500
H	-7.15069500	-1.27774600	-1.12519500

N	-4.20458300	1.90049700	0.21633600
Cu	-2.95220800	0.13001700	0.31486300
N	4.95238900	-1.41795300	0.48097000
S	5.27721100	0.06361100	1.16287800
O	4.05596700	0.85851900	1.01438500
O	5.83880700	-0.06205400	2.51216600
C	6.53613800	0.68566000	0.09137600
C	7.80271600	0.95396500	0.59461000
C	6.21495800	0.93411900	-1.24398600
C	8.76748600	1.47443900	-0.26145400
H	8.03078600	0.75945000	1.64037800
C	7.18895400	1.45261400	-2.07960800
H	5.21522900	0.71894700	-1.62021700
C	8.47760400	1.72981600	-1.60215100
H	9.76517200	1.68801400	0.12084000
H	6.95394900	1.65088800	-3.12541400
C	9.51329200	2.28687500	-2.52691100
H	9.72517000	1.58775500	-3.34763900
H	9.16802000	3.22134100	-2.98957100
H	10.45504900	2.49215600	-2.00512100
H	5.74645000	-2.04236000	0.63179200
C	-3.57559600	-2.97295500	-0.06512100
H	-3.36459400	-3.25696600	0.97945900
H	-3.90834000	-3.90480600	-0.55031400
C	-1.09762000	-2.82011400	-0.33101900
H	-1.00490100	-3.45035500	0.56407700
H	-1.84621200	-1.00989600	2.28497700
C	0.21649000	-2.49783200	-1.00983700
C	0.14005400	-1.20824400	-1.82156100
H	-0.06014700	-0.33809300	-1.17800200
H	1.08151900	-1.02599200	-2.35714500
H	-0.65208400	-1.25814300	-2.58103000
C	0.55753800	-3.66510500	-1.94462000
H	0.57541000	-4.61929000	-1.39781000
H	-0.19418000	-3.75038000	-2.74221500

H	1.53907900	-3.53073700	-2.42212900
C	1.28224400	-2.39005200	0.09858700
H	1.39527900	-3.38895500	0.55721900
H	0.89479600	-1.74308600	0.90454900
C	2.65065000	-1.86912700	-0.31224500
H	2.59258800	-0.80475000	-0.58758300
H	3.03591600	-2.40161700	-1.19574200
C	3.64736000	-2.02582600	0.82041900
H	3.83589500	-3.08725300	1.02320700
H	3.24371400	-1.60124900	1.75360100
C	-2.32751400	-2.47940200	-0.72841400
H	-2.46429900	-1.88767500	-1.63795400
C	-0.48198500	0.21263100	1.81738300
O	-0.94464900	-0.75929200	2.58383400
O	-1.12468500	0.69117800	0.88388600
C	0.87364000	0.69522500	2.18324600
H	0.76930300	1.64387300	2.72670800
H	1.40205700	-0.01722200	2.82286300
H	1.44646900	0.89847500	1.27201100

TS2

O	-2.99370900	1.28620200	-0.10670700
N	-3.10068200	-1.02241300	-0.43611900
C	-2.94779200	0.10203700	0.26023400
C	-4.68897700	-4.28092700	-2.17075300
C	-3.74383600	-2.17954500	-2.43937300
C	-3.83016700	-2.28280100	-3.85439900
C	-4.37939000	-3.46843000	-4.39335700
C	-4.80965000	-4.46807300	-3.55906700
H	-5.02628100	-5.05358300	-1.47925700
C	-3.19985400	-0.99995500	-1.82146600
C	-3.37700100	-1.21854300	-4.66575600
H	-4.45418900	-3.56817900	-5.47631000
H	-5.23752400	-5.38994400	-3.94507900
C	-2.85683300	-0.09822900	-4.06710900

C	-2.76833700	0.01645800	-2.66668400
H	-3.45086700	-1.30699400	-5.74903400
H	-2.50082600	0.72930000	-4.67977800
H	-2.35137000	0.91992000	-2.23394500
N	-4.17831800	-3.18908700	-1.63229500
Cu	-4.00489900	-2.73920800	0.37564800
N	4.88001500	-0.78121400	5.60104900
S	5.54033500	0.72672400	5.85798800
O	4.85630500	1.63572500	4.93660500
O	5.54630400	1.09105400	7.27916900
C	7.20863500	0.46999500	5.33864600
C	8.24156400	0.55580700	6.26375500
C	7.45787900	0.21162900	3.98991000
C	9.54908600	0.37258800	5.82695500
H	8.02665300	0.76443900	7.30959600
C	8.76635400	0.03237500	3.57535400
H	6.63535300	0.15079400	3.27848400
C	9.82958500	0.10964400	4.48553800
H	10.36819800	0.43747700	6.54250500
H	8.97734200	-0.16985500	2.52521100
C	11.23451700	-0.09141200	4.01249400
H	11.37211900	-1.10354200	3.60752900
H	11.48295300	0.60872500	3.20350000
H	11.95958400	0.05070300	4.82197300
H	5.33110500	-1.45151700	6.22682300
C	-2.68568100	-0.07904500	1.77205000
H	-2.54874800	0.88163700	2.28552100
H	-3.77911100	-0.46922900	2.17241200
C	-0.98588200	-1.04805000	3.38027000
H	-1.17499700	-1.94242000	3.99753400
H	-1.20176300	-0.15008000	3.97599500
C	0.53305400	-1.06561700	3.00232600
C	0.83970200	0.13287100	2.10863900
H	0.59311200	1.07995300	2.61162000
H	1.90255300	0.16097100	1.83493700

H	0.26712000	0.09229000	1.16928900
C	0.88214200	-2.36551400	2.28316700
H	0.62479900	-3.24366700	2.89482200
H	0.36381700	-2.45689100	1.31861200
H	1.95724500	-2.41247200	2.06553800
C	1.27953400	-0.96716900	4.34203400
H	1.00141300	-1.84302600	4.95476700
H	0.90655700	-0.08170200	4.88455300
C	2.79663000	-0.87917000	4.26157300
H	3.10663400	0.02664100	3.72109100
H	3.21924400	-1.73720100	3.71770800
C	3.40204700	-0.85021600	5.65238300
H	3.15203200	-1.76883700	6.19752900
H	2.98430000	-0.01830900	6.24201400
C	-1.84579300	-1.09998800	2.19715700
H	-1.82447400	-2.00803400	1.58163100
C	-6.16133900	1.11932300	1.52103500
H	-5.13328100	1.52110600	1.53210700
C	-6.83779500	1.58883400	0.24269800
H	-6.96710700	2.67934400	0.23846400
H	-6.24662800	1.31605500	-0.64399100
H	-7.83586000	1.13585000	0.13684000
C	-6.89748800	1.61082600	2.75780900
H	-7.94042400	1.25754000	2.74948500
H	-6.42816800	1.24723900	3.68139400
H	-6.91791700	2.70831700	2.79973700
C	-6.06168200	-0.39470400	1.50825600
H	-7.06395800	-0.83510700	1.65727000
H	-5.68914200	-0.75080300	0.52951600
O	-5.19660100	-0.85463300	2.54394700
C	-2.80294800	-4.70737300	2.56263500
H	-2.23128300	-4.20991900	1.75277800
C	-1.85180900	-5.06307900	3.69534900
H	-1.04400400	-5.72429300	3.35221800
H	-1.38701700	-4.16986100	4.14101500

H	-2.38850900	-5.58877000	4.50049100
C	-3.45113300	-5.95809400	1.99066200
H	-4.05683700	-6.46143800	2.76123700
H	-4.11359800	-5.72237200	1.14702700
H	-2.69752500	-6.67635500	1.63807200
C	-3.85449900	-3.71273100	3.04616000
H	-4.38630000	-4.18476500	3.90296100
H	-3.31600000	-2.83532100	3.48486900
O	-4.76561700	-3.30910900	2.07771800
H	-5.09776300	-1.88507200	2.41694800

3′

O	-2.88943800	2.58632300	-3.14270200
N	-3.35470300	0.30505600	-3.08585600
C	-2.86170600	1.46611000	-2.59710300
C	-5.59833700	-2.92795700	-3.95864000
C	-4.57920600	-0.98155900	-4.70905100
C	-5.03181500	-1.21851700	-6.03654400
C	-5.80432300	-2.37898800	-6.27008700
C	-6.09259000	-3.23491500	-5.23838200
H	-5.80263700	-3.58957900	-3.11615000
C	-3.79162100	0.18625400	-4.39565300
C	-4.70236500	-0.31071100	-7.06758300
H	-6.16122800	-2.57635300	-7.28114700
H	-6.68326300	-4.13500200	-5.39034100
C	-3.94109600	0.79023600	-6.76368000
C	-3.49331800	1.04172000	-5.45362300
H	-5.05637600	-0.50256500	-8.07989800
H	-3.67149700	1.49684400	-7.54810000
H	-2.90021000	1.92778500	-5.25870400
N	-4.87657400	-1.85352000	-3.70337100
Cu	-3.98335300	-1.19705800	-1.91549100
N	4.40102700	0.01217100	3.98769400
S	4.98290100	1.42818500	4.64629500
O	4.57388100	2.50292800	3.74033000

O	4.61680700	1.56991100	6.06024800
C	6.72626800	1.16588600	4.54298400
C	7.47270500	1.02480500	5.70634900
C	7.32551700	1.12600700	3.28322200
C	8.84624700	0.83441800	5.60208500
H	6.98725300	1.06451400	6.67908100
C	8.69433000	0.93553000	3.20113500
H	6.72460200	1.24100700	2.38221700
C	9.47379200	0.78722900	4.35660800
H	9.44275100	0.72206600	6.50687900
H	9.17717500	0.90130900	2.22448700
C	10.95162700	0.58561600	4.24058400
H	11.42186500	0.45537700	5.22204300
H	11.18305300	-0.29832300	3.63084600
H	11.42831100	1.44345400	3.74663000
H	4.70354000	-0.76894500	4.57367400
C	-2.22565000	1.39860500	-1.24838600
H	-2.23254400	2.34884400	-0.70508600
H	-3.96041800	0.25111700	0.13955900
C	-0.87332600	0.35111800	0.58572000
H	-1.21316500	-0.53640200	1.15045300
H	-1.18736300	1.23707500	1.16242400
C	0.67214300	0.31444700	0.54435700
C	1.19360500	1.61057700	-0.06866500
H	0.92473800	2.47996200	0.55105000
H	2.28727800	1.59669600	-0.17308500
H	0.77257500	1.77135200	-1.07119600
C	1.14492500	-0.87767300	-0.28416100
H	0.72348700	-1.82075500	0.09696400
H	0.84835000	-0.77739500	-1.33761700
H	2.23958100	-0.96912900	-0.26960800
C	1.13760500	0.17542800	2.00274600
H	0.75369700	-0.78351000	2.39548400
H	0.64807500	0.96513700	2.59988700
C	2.63699300	0.24325100	2.25359900

H	3.03560300	1.22797800	1.96986800
H	3.17642200	-0.50420300	1.65210600
C	2.94452400	-0.00992500	3.71699100
H	2.59081900	-1.00522300	4.01381000
H	2.41010300	0.70988900	4.35834300
C	-1.55755100	0.35520000	-0.74045600
H	-1.51786900	-0.57132200	-1.32309900
O	-4.06498000	-2.06762700	-0.10115300
C	-4.19850300	-1.44728600	0.95319800
O	-4.18442500	-0.12737800	1.02201400
C	-4.37409000	-2.10244600	2.27357000
H	-3.49960100	-1.88574800	2.90029100
H	-4.48269700	-3.18223800	2.15228500
H	-5.25091100	-1.68776100	2.78382100

TS3

O	-4.82115800	-1.03469500	-3.29609900
N	-3.99598700	-0.61101500	-1.15231900
C	-3.14816300	0.70711200	-2.77122000
C	-2.40948300	0.59813000	-1.46753600
C	-4.12984800	-0.40804900	-2.51501200
C	-1.29681400	-0.37804100	-1.27962700
H	-2.52188700	0.49382200	-3.64784400
H	-2.25938300	1.53767900	-0.92523800
C	-4.66288300	-0.65323200	2.82519700
C	-4.39342500	-1.86643900	0.87666400
C	-4.54929000	-3.09771700	1.56341400
C	-4.77548000	-3.03828800	2.95783100
C	-4.83199500	-1.82239700	3.59050000
H	-4.70282400	0.32950900	3.29603900
C	-4.15564100	-1.85578200	-0.53075900
C	-4.45547800	-4.31078700	0.84455900
H	-4.89869000	-3.96802000	3.51277400
H	-5.00287700	-1.74558000	4.66119600
C	-4.20086300	-4.28424800	-0.50535800

C	-4.03612300	-3.06686200	-1.19199000
H	-4.57400600	-5.25257800	1.37880300
H	-4.11471100	-5.21534400	-1.06232400
H	-3.83396300	-3.07818400	-2.26035000
N	-4.45203500	-0.67201500	1.52399500
H	-3.63597200	1.68042400	-2.91253800
Cu	-3.97906600	0.87413100	0.13362100
H	-1.43662800	-0.90280400	-0.31871100
H	-1.34149300	-1.14570200	-2.07132500
C	0.10362800	0.27965700	-1.24814700
C	1.11517800	-0.87798800	-1.25702400
H	0.84328800	-1.58188400	-0.45071000
H	0.99170800	-1.43543200	-2.20252100
C	0.29461000	1.17082000	-2.47394900
H	0.17746600	0.59832900	-3.40655200
H	-0.43312600	1.99691300	-2.49030500
H	1.29273600	1.62906700	-2.48465000
C	0.25282800	1.10646200	0.02877800
H	1.21511500	1.63548200	0.04947900
H	-0.52965000	1.87533300	0.12245500
H	0.20038900	0.46603300	0.92229100
C	2.58008900	-0.50052000	-1.09390000
H	2.89195600	0.22926400	-1.85678200
H	2.75307500	-0.03129800	-0.11434100
C	3.46804300	-1.72472800	-1.21243000
H	3.36715400	-2.17586300	-2.20740600
H	3.16416500	-2.49661400	-0.48836500
N	4.89633900	-1.37540200	-1.05721800
S	5.58360000	-1.64056900	0.43370700
O	4.58787700	-1.22668500	1.42356000
O	6.13462000	-2.99497600	0.55002100
C	6.92966500	-0.49561100	0.42216200
C	8.23663800	-0.95932200	0.49815700
C	6.64351600	0.86960300	0.37267300
C	9.27606200	-0.03512000	0.51820700

H	8.43886300	-2.02733200	0.54159300
C	7.69150900	1.77335100	0.39464400
H	5.61175200	1.21522000	0.31361600
C	9.02143100	1.33567000	0.46716700
H	10.30560900	-0.38686600	0.57658900
H	7.48375900	2.84276500	0.35607600
C	10.13633600	2.33319100	0.48598700
H	10.14417600	2.93265700	-0.43476900
H	10.02158000	3.03824800	1.32042500
H	11.11416400	1.84756200	0.58238900
H	5.49605900	-1.76930100	-1.78372100
C	-2.76428800	4.77317800	0.86286400
H	-1.94302100	4.30674200	1.43729600
C	-2.18277700	5.83590900	-0.05575700
H	-1.69800900	6.64248100	0.51168800
H	-1.43437100	5.41526500	-0.74348300
H	-2.97485100	6.29602100	-0.66775800
C	-3.75639500	5.38330800	1.83984200
H	-4.59323600	5.84845000	1.29360600
H	-4.17720200	4.62394800	2.51089800
H	-3.29133300	6.16358400	2.45900200
C	-3.41797500	3.65882700	0.04388000
H	-4.23425300	4.12912500	-0.56050000
H	-2.65823600	3.33675300	-0.71791100
O	-3.87657300	2.62009700	0.82345200

4′

O	-5.14099400	-0.98685100	-3.16951500
N	-3.74938300	-0.79798200	-1.27747200
C	-3.03964500	0.40196700	-2.83185700
C	-2.50320000	0.01280500	-1.43637400
C	-4.18672900	-0.55179400	-2.55559700
C	-1.23974300	-0.81578500	-1.35194100
H	-2.40815400	0.10854100	-3.67787300
H	-2.49094400	0.85198800	-0.72521000

C	-4.82136900	-0.03661700	2.70108800
C	-4.43588300	-1.54181500	0.97270000
C	-4.68722700	-2.63586000	1.84906400
C	-4.98730000	-2.35911800	3.20046400
C	-5.04197400	-1.06061600	3.63317300
H	-4.88982500	1.00597100	3.00883000
C	-4.08897500	-1.82758100	-0.37859100
C	-4.64677200	-3.96217500	1.36083600
H	-5.17367800	-3.19068300	3.87911800
H	-5.26580800	-0.80645100	4.66543200
C	-4.35585000	-4.20654700	0.04409900
C	-4.05912900	-3.13482800	-0.81761500
H	-4.85306300	-4.77643000	2.05423700
H	-4.32695000	-5.22472800	-0.33742600
H	-3.78628400	-3.32757100	-1.85441400
N	-4.53590900	-0.25118600	1.42180600
H	-3.35048300	1.44785200	-2.94150500
Cu	-4.51457400	1.37192600	0.35504200
H	-1.31279100	-1.46895000	-0.46488600
H	-1.20078400	-1.48685100	-2.22890800
C	0.07326800	-0.01844700	-1.24116300
C	1.21486400	-1.03892400	-1.38785800
H	1.02732600	-1.87035000	-0.68525900
H	1.15650800	-1.47800000	-2.39988600
C	0.15595200	1.03376800	-2.34559200
H	0.11061100	0.57134700	-3.34331600
H	-0.66596700	1.76236700	-2.27567100
H	1.09243500	1.60551300	-2.28410700
C	0.14877900	0.65884100	0.12796800
H	1.02632000	1.31597800	0.20421900
H	-0.73483600	1.28350500	0.32681900
H	0.21605800	-0.08760200	0.93421000
C	2.62665700	-0.52259400	-1.15322400
H	2.85306400	0.33292700	-1.80806600
H	2.74445500	-0.17021400	-0.11809300

C	3.64893200	-1.61173500	-1.41574800
H	3.61210500	-1.92808800	-2.46558400
H	3.42456300	-2.50788200	-0.81550800
N	5.02810600	-1.13451800	-1.16812300
S	5.71070400	-1.58644800	0.28098100
O	4.70921400	-1.30375000	1.31100000
O	6.26019800	-2.94601000	0.23278800
C	7.05274500	-0.44547200	0.41047900
C	8.36052400	-0.91321200	0.43245200
C	6.76351100	0.91504800	0.52721200
C	9.39786200	0.00347300	0.56697300
H	8.56440500	-1.97833900	0.34596300
C	7.80991300	1.81125100	0.65967100
H	5.73125100	1.26280800	0.50969200
C	9.14036600	1.37008100	0.68143100
H	10.42802800	-0.35079500	0.58497100
H	7.60025700	2.87714500	0.75007700
C	10.25334200	2.35955500	0.82425200
H	10.25370800	3.07516800	-0.00943600
H	10.14249300	2.94779700	1.74531800
H	11.23261600	1.86807500	0.84990100
H	5.67397100	-1.35738500	-1.92781300
C	-2.47128100	3.78908500	0.53896200
H	-2.29463200	2.77680400	0.95698900
C	-1.12894300	4.41331600	0.18959800
H	-0.48062600	4.50217900	1.07265000
H	-0.58595500	3.82254100	-0.56424800
H	-1.26615600	5.42602200	-0.22135200
C	-3.20047900	4.61695500	1.58522800
H	-3.42545700	5.62075400	1.18982400
H	-4.15271700	4.15408200	1.87886000
H	-2.59544000	4.74637200	2.49391300
C	-3.31928200	3.60524200	-0.72221800
H	-3.47232300	4.62192900	-1.15476600
H	-2.68033600	3.06905300	-1.46735900

O	-4.54378000	2.98500100	-0.53838700

5′

O	-3.51620900	1.50274600	0.59898800
N	-2.91383000	-0.66984600	0.18105100
C	-2.72976800	0.54113000	0.72891800
C	-2.75951600	-4.46220300	-0.87680400
C	-3.97029100	-2.47604000	-0.93515600
C	-5.02339100	-3.07895800	-1.66235900
C	-4.88286400	-4.44844800	-1.98056400
C	-3.76148000	-5.14053100	-1.59190200
H	-1.84738800	-4.96543000	-0.55354200
C	-4.02070500	-1.10038500	-0.56448500
C	-6.13729700	-2.28908000	-2.02861000
H	-5.67703500	-4.94338000	-2.53883900
H	-3.63461000	-6.19356500	-1.82732500
C	-6.17057100	-0.96443900	-1.67253200
C	-5.12376600	-0.35883300	-0.94434000
H	-6.95097300	-2.74432100	-2.59091800
H	-7.02406300	-0.35015300	-1.95257600
H	-5.18725300	0.68956700	-0.67623300
N	-2.86657200	-3.18599400	-0.56616300
Cu	-1.57758800	-1.96779800	0.39121200
N	5.57673500	2.46967100	-2.41828600
S	6.80914600	1.33776400	-2.51846500
O	6.33487200	0.10729000	-3.14529900
O	7.93389900	2.07202100	-3.08911000
C	7.16293900	0.97064300	-0.81765800
C	7.11337300	-0.34476400	-0.37626300
C	7.52448500	2.00740600	0.04100300
C	7.41943500	-0.62246900	0.95203700
H	6.82995300	-1.13656600	-1.06675800
C	7.82574500	1.71246200	1.36016300
H	7.55899500	3.03264100	-0.32409000
C	7.77673800	0.39572300	1.83572500

H	7.38020700	-1.65135400	1.30879500
H	8.10563600	2.51496300	2.04303600
C	8.10189600	0.10281600	3.26776400
H	8.05191200	-0.97045300	3.48456300
H	9.11093600	0.45178400	3.52571600
H	7.40629800	0.61502400	3.94659100
H	5.77741700	3.18797200	-3.11339500
C	-1.48829700	0.57383400	1.57986600
H	-1.03435100	1.57658500	1.56956500
H	-1.79415700	0.36507000	2.61511400
C	0.31150900	-0.05985400	-0.13356400
H	-0.21129300	0.75210100	-0.66571200
H	0.40009600	-0.90353200	-0.83850300
C	1.76702600	0.38691200	0.19331300
C	2.58858900	-0.82454500	0.62840600
H	2.67940200	-1.56140900	-0.18335900
H	3.60166300	-0.52620000	0.93141000
H	2.14206600	-1.34241100	1.48867400
C	1.76410500	1.45141200	1.28827000
H	1.14913100	2.31785300	1.00263200
H	1.37715100	1.05646000	2.23918000
H	2.78208400	1.80833500	1.49299600
C	2.31420700	0.97447500	-1.11932500
H	1.70906200	1.86276200	-1.37455000
H	2.14468900	0.24318300	-1.92924700
C	3.78590700	1.35958300	-1.11606000
H	4.40314200	0.46558400	-0.94485800
H	4.01687300	2.06398900	-0.30265300
C	4.18673800	1.99813800	-2.43362500
H	3.57184700	2.89025500	-2.61603300
H	4.00873200	1.29932500	-3.26725700
C	-0.49342800	-0.43675400	1.07764100
H	0.04645400	-0.98153400	1.85745900
C	-0.62277800	4.46808200	-1.89978800
H	-1.04251400	5.30450600	-2.46523600

H	0.38600600	4.71180200	-1.55064800
H	-0.54340100	3.60316500	-2.57201700
C	-1.50200000	4.09916500	-0.74437800
O	-2.72619600	4.42698800	-0.75162700
O	-1.02139700	3.43150500	0.21998400
Zn	-3.00378100	3.43742700	1.04998600
O	-4.50262200	4.28599300	2.19667200
C	-3.84329300	3.98146800	3.23909400
O	-2.72469500	3.39684500	3.14096700
C	-4.38973700	4.34750000	4.58413000
H	-4.21437000	5.41679300	4.75872800
H	-5.47206400	4.18562900	4.61271500
H	-3.89770300	3.77977500	5.37843000
C	-0.16569200	-3.74416200	1.71949600
O	-0.94717200	-3.54809900	2.63510400
C	0.91806800	-4.83124200	1.83029900
F	0.91909000	-5.41535200	3.02018200
F	0.70555800	-5.77502600	0.90384200
F	2.13138200	-4.31504500	1.61761100
O	-0.11157700	-3.15064700	0.58225900

TS4

O	-4.22601300	1.55229800	-1.88981100
N	-2.96834800	-0.33664000	-1.48242900
C	-3.12330700	0.94396700	-1.77169400
C	-2.37974600	-4.31304100	-1.70463700
C	-3.80453000	-2.57589600	-1.13291900
C	-4.79751200	-3.48665400	-0.68137900
C	-4.51203900	-4.86630200	-0.78777800
C	-3.31142700	-5.28651100	-1.30145100
H	-1.40658900	-4.60166300	-2.10436300
C	-4.03406100	-1.16290900	-1.06905800
C	-6.00602100	-2.99317500	-0.14226500
H	-5.26056900	-5.58376700	-0.45176300
H	-3.06851700	-6.34201700	-1.39301200

C	-6.20196900	-1.63910200	-0.06269500
C	-5.23264900	-0.72888700	-0.52800200
H	-6.75896700	-3.69774100	0.20850700
H	-7.11683900	-1.23716800	0.36812700
H	-5.44882400	0.33110700	-0.45579400
N	-2.61626400	-3.02054300	-1.62282500
Cu	-1.38429400	-1.33276400	-2.05302200
N	6.89013600	0.93593500	-1.65740600
S	7.48564800	-0.62320000	-1.82970400
O	6.32954200	-1.50832800	-1.75618800
O	8.39262000	-0.70776700	-2.97185200
C	8.45871600	-0.79137100	-0.35612500
C	9.82036900	-1.04180500	-0.45657000
C	7.82840800	-0.71223600	0.88495800
C	10.56277500	-1.20811200	0.70807000
H	10.28752600	-1.10471500	-1.43691000
C	8.58252200	-0.88024100	2.03341000
H	6.75916500	-0.51253500	0.94280400
C	9.95911700	-1.13204000	1.96265900
H	11.63281200	-1.40309300	0.64067900
H	8.10253000	-0.81756400	3.01026200
C	10.75186800	-1.32467600	3.21816000
H	10.46396900	-2.25440500	3.72843100
H	11.82776800	-1.37693600	3.01531800
H	10.58027900	-0.50622000	3.92982300
H	7.61057700	1.59556900	-1.94853900
C	-1.85674500	1.74319100	-2.00220900
H	-1.69153400	1.88498800	-3.08271400
H	-2.01760400	2.76832600	-1.61028800
C	0.64170800	1.47811000	-1.78213300
H	0.71909300	2.32473900	-2.48522600
H	0.63307500	0.60060500	-2.70973200
C	1.88142100	1.23654200	-0.91300600
C	1.84616700	-0.15629700	-0.28567000
H	1.95958800	-0.95252600	-1.03505900

H	2.66079800	-0.27441100	0.43930000
H	0.91391400	-0.34315200	0.26562200
C	1.89497100	2.29654300	0.19070000
H	1.87985600	3.31220200	-0.23014800
H	1.01883200	2.19240100	0.84731800
H	2.79009800	2.20571300	0.82020600
C	3.10757300	1.39228300	-1.83124100
H	3.12874500	2.43358000	-2.20039000
H	2.96133000	0.75940300	-2.72028000
C	4.45635700	1.04691300	-1.22004600
H	4.46843900	0.00110100	-0.88188200
H	4.68068200	1.67596700	-0.34443400
C	5.56646600	1.22125300	-2.24038800
H	5.60259700	2.26191600	-2.58945100
H	5.36699600	0.59730600	-3.12624500
C	-0.64643000	1.21353100	-1.34527700
H	-0.79863400	0.63132800	-0.43079600
C	-3.24197700	6.55221200	-2.22735500
H	-3.69738500	7.36851100	-1.65209100
H	-2.16117600	6.57668800	-2.06464000
H	-3.47683200	6.72461900	-3.28229800
C	-3.82890500	5.25764600	-1.76467600
O	-4.97143300	4.90244500	-2.19135600
O	-3.20681200	4.53128900	-0.92956800
Zn	-4.88009200	3.22993000	-1.01768900
O	-5.98591400	2.71179500	0.44491300
C	-5.53954900	2.14731700	1.53862600
O	-6.25551700	1.59721100	2.35520300
C	-4.03161000	2.21249200	1.73796000
H	-3.51590700	1.63037200	0.95598300
H	-3.67079800	3.24847900	1.67454500
H	-3.75016900	1.79369100	2.70871100
C	1.03840700	-1.41202500	-3.52375900
O	1.00346800	-0.18685000	-3.80537300
O	0.23473800	-2.07182700	-2.83728100

C	2.28094200	-2.15045800	-4.05414700
F	2.50653800	-1.84246200	-5.32760600
F	3.34169600	-1.76057300	-3.33581900
F	2.16956700	-3.46713300	-3.95177200

6′

O	-4.42127100	1.44007000	-0.66715500
N	-3.14650900	-0.37581300	-0.06554700
C	-3.30600000	0.89327800	-0.40428200
C	-2.50558700	-4.37168800	-0.17594700
C	-3.98330300	-2.62853200	0.24302300
C	-5.00335900	-3.54354100	0.62569900
C	-4.70238200	-4.92208800	0.56657500
C	-3.46128800	-5.34432200	0.16278700
H	-1.50076800	-4.66399800	-0.48451600
C	-4.23038100	-1.21446800	0.26012000
C	-6.25851300	-3.05869100	1.05274200
H	-5.47379300	-5.63804600	0.85004500
H	-3.20577700	-6.39945900	0.10997800
C	-6.47355300	-1.70597000	1.09157000
C	-5.47850000	-0.79359400	0.69220900
H	-7.03026500	-3.76777500	1.34922300
H	-7.42555100	-1.30648900	1.43586700
H	-5.71505500	0.26352000	0.72517300
N	-2.75488100	-3.07808200	-0.13769600
Cu	-1.53457300	-1.43066300	-0.52188000
N	6.59328000	0.86853600	-0.46263200
S	7.13675900	-0.70003600	-0.69238100
O	5.95309000	-1.55007200	-0.64114000
O	8.03305500	-0.76939500	-1.84381700
C	8.11325600	-0.95998800	0.76539200
C	9.46649400	-1.24423000	0.64581400
C	7.49129800	-0.91736500	2.01258500
C	10.20916600	-1.48328900	1.79775500
H	9.92725400	-1.27812000	-0.33895300

C	8.24521100	-1.15860000	3.14781700
H	6.42828400	-0.69082300	2.08522400
C	9.61389400	-1.44581100	3.05792100
H	11.27264000	-1.70632600	1.71512000
H	7.77166300	-1.12714200	4.12933400
C	10.40721900	-1.70923800	4.30015500
H	10.03120300	-2.59506100	4.83019000
H	11.46704100	-1.87804300	4.07741400
H	10.34011100	-0.86756000	5.00267000
H	7.31748900	1.51808700	-0.76535300
C	-2.08566100	1.78748000	-0.50608800
H	-1.91598700	2.00738100	-1.57362900
H	-2.38958600	2.75940600	-0.08006700
C	0.39500500	1.78229800	-0.10980300
H	0.50088800	2.51630700	-0.92153500
H	0.05188900	0.33652600	-1.79183100
C	1.67517700	1.48262800	0.64231200
C	1.60652300	0.14850200	1.37945300
H	1.52229900	-0.70035700	0.68218700
H	2.50782100	-0.01015600	1.98455500
H	0.75409000	0.10709200	2.07035200
C	1.87109800	2.61533400	1.65875900
H	1.89311700	3.59680600	1.16413200
H	1.04842600	2.62703600	2.38665500
H	2.81057200	2.49644400	2.21585500
C	2.82847100	1.48553400	-0.38181400
H	2.89056800	2.49773800	-0.82036300
H	2.56771400	0.81645100	-1.21732200
C	4.19955900	1.07486400	0.13291200
H	4.18555600	0.02725400	0.46720000
H	4.50888100	1.68634700	0.99480200
C	5.24777100	1.20983200	-0.95664000
H	5.30254800	2.25079400	-1.30223100
H	4.96907400	0.59982500	-1.83025300
C	-0.83257700	1.32289000	0.17047100

H	-0.97349100	0.64371700	1.01561900
C	-3.93361800	6.55007000	-1.23794300
H	-4.52798200	7.32929100	-0.74347200
H	-2.88212100	6.71233900	-0.98503800
H	-4.09053500	6.64726600	-2.31639000
C	-4.40140800	5.21473300	-0.75298000
O	-5.39924300	4.66004500	-1.31008200
O	-3.82476100	4.65609900	0.22919800
Zn	-5.24608000	3.11138400	0.03202300
O	-6.48452300	2.60108300	1.39638100
C	-6.13526400	2.14757800	2.57279100
O	-6.91175900	1.62809700	3.35476500
C	-4.66045700	2.29730200	2.91864300
H	-4.04446200	1.67293200	2.25028600
H	-4.33018600	3.33876900	2.79883900
H	-4.46825000	1.97807800	3.94740600
C	0.84456800	-1.33499900	-2.15365200
O	0.64255400	-0.08638000	-2.47215600
O	0.21165400	-1.99473700	-1.34887900
C	2.07556200	-1.91647000	-2.86146900
F	2.12337600	-1.52783400	-4.12639000
F	3.15472500	-1.46262200	-2.22685500
F	2.06060000	-3.23399500	-2.80698600

TS5

O	-4.43086200	1.33761300	-0.69269400
N	-3.68850500	-0.75375500	-1.32345100
C	-3.50382700	0.52029400	-1.00919100
C	-3.57581700	-4.55375700	-2.51904200
C	-4.86192400	-2.79777400	-1.72135000
C	-6.03367700	-3.59751700	-1.72860800
C	-5.90493300	-4.93694200	-2.16070700
C	-4.68325900	-5.42123100	-2.55340800
H	-2.58700900	-4.89631100	-2.82992100
C	-4.91379200	-1.43169800	-1.28274800

C	-7.25992500	-3.03917500	-1.30250000
H	-6.79061900	-5.57227000	-2.17264600
H	-4.55754500	-6.44787600	-2.88731200
C	-7.29566300	-1.73378600	-0.88577000
C	-6.13745700	-0.93040800	-0.87073600
H	-8.15630600	-3.65818500	-1.30730900
H	-8.23470700	-1.29477600	-0.55340300
H	-6.20734200	0.09706300	-0.53535200
N	-3.66428100	-3.30289900	-2.12117100
Cu	-2.16758300	-1.80081200	-1.92621200
N	6.40300500	1.54516600	-1.54234600
S	7.47292700	0.27614800	-1.77893300
O	6.66748400	-0.93872900	-1.77687800
O	8.36441500	0.55257300	-2.90321700
C	8.44069800	0.35328200	-0.29438700
C	9.80927700	0.56833900	-0.37796200
C	7.81417700	0.15352400	0.93520000
C	10.56074700	0.59166100	0.79259300
H	10.27504500	0.71382400	-1.35015800
C	8.57745400	0.17985100	2.08961000
H	6.73869000	-0.01392700	0.97941000
C	9.96053600	0.39860300	2.03605900
H	11.63585200	0.76125600	0.73824800
H	8.09990300	0.02694000	3.05775400
C	10.76539900	0.41746700	3.29867100
H	10.38576700	1.17029500	4.00272000
H	10.71656600	-0.55144400	3.81427900
H	11.82100200	0.63988200	3.10443100
H	6.87300400	2.41774900	-1.78009900
C	-2.10002200	1.00740100	-1.09481000
H	-1.99334800	1.81440000	-1.83527600
H	-1.91969900	1.61215700	0.09653800
C	0.25198000	0.33385400	-1.74722000
H	0.17246300	1.28889800	-2.29056700
H	0.39391900	-0.45246000	-2.51013300

C	1.51910000	0.34708900	-0.85440200
C	1.81741100	-1.05562000	-0.33327300
H	2.08055300	-1.73506700	-1.15804300
H	2.66274200	-1.04441600	0.36739400
H	0.96607200	-1.49359200	0.20484100
C	1.31097200	1.31110800	0.30965800
H	1.05584500	2.32108300	-0.04796900
H	0.49701200	0.98143400	0.96987700
H	2.21317700	1.39213800	0.93034300
C	2.65818900	0.83352500	-1.76708900
H	2.40480300	1.84793700	-2.12466600
H	2.68069800	0.19093100	-2.66471100
C	4.05185500	0.86195300	-1.15658300
H	4.36990400	-0.14927800	-0.86682300
H	4.07804800	1.48354500	-0.24823000
C	5.06131600	1.42180600	-2.14242500
H	4.76859900	2.43665600	-2.44424500
H	5.08126100	0.81391800	-3.06027100
C	-1.04133300	0.06370100	-1.07132200
H	-1.05426000	-0.69700200	-0.28101800
C	-4.98893300	5.82445800	-3.28900000
H	-4.27293600	5.79312800	-4.11383100
H	-6.00306700	5.63833500	-3.65809300
H	-4.98475900	6.82912100	-2.84812600
C	-4.64083400	4.82914900	-2.23091500
O	-5.47912600	4.58580700	-1.30381900
O	-3.51760600	4.24148700	-2.23944800
Zn	-4.13777400	3.27336200	-0.50378500
O	-3.23134800	3.73389400	1.17665200
C	-2.34518600	3.05929200	1.77718600
O	-1.76739400	2.03171600	1.31935800
C	-1.95036000	3.52543200	3.14424300
H	-1.58144100	4.55589600	3.08022000
H	-2.83581000	3.54183600	3.79030800
H	-1.18293400	2.88260300	3.58106900

C	-0.13482900	-3.39274200	-1.67774900
O	-0.55419500	-3.51685900	-0.53324200
C	0.98561300	-4.32535500	-2.16981300
F	1.63028300	-4.91028300	-1.16668100
F	0.44571900	-5.28901800	-2.93293000
F	1.88780600	-3.67995300	-2.91408200
O	-0.54708600	-2.60009500	-2.59030600

7′

O	-3.26536100	2.22649100	-0.61704700
N	-3.79235900	-0.01362100	-0.32067500
C	-3.00124400	0.95717900	-0.69470400
C	-5.59936100	-3.28785100	1.12759400
C	-5.68756000	-0.99752100	0.77076200
C	-6.96220800	-0.91768200	1.39344800
C	-7.52367600	-2.11084200	1.89993600
C	-6.84661700	-3.29607600	1.77627900
H	-5.03579300	-4.21112600	0.99513300
C	-5.05112700	0.18481900	0.25591200
C	-7.62227600	0.32801900	1.48295000
H	-8.49872400	-2.06733900	2.38516000
H	-7.25282600	-4.22952600	2.15704900
C	-7.02119900	1.44359200	0.96244700
C	-5.75056300	1.37960400	0.35776300
H	-8.60016200	0.37777000	1.96047100
H	-7.52356000	2.40816500	1.01464800
H	-5.31090600	2.28525900	-0.04011800
N	-5.04630500	-2.19122200	0.64749300
Cu	-3.29771000	-2.08442000	-0.40488900
N	6.68303300	-1.26834100	-1.44346400
S	7.31463100	-1.47983200	0.09352600
O	6.69794200	-2.61751000	0.76919600
O	8.76073000	-1.43133500	-0.09637700
C	6.78817900	-0.00765800	0.93813200
C	6.00989800	-0.11404800	2.08284600

C	7.16431400	1.23552600	0.43411900
C	5.58729900	1.04679500	2.72187500
H	5.72701400	-1.09697400	2.45486300
C	6.73482400	2.38169400	1.08301600
H	7.77301300	1.29789200	-0.46649500
C	5.93993100	2.30529500	2.23356300
H	4.97215000	0.97297500	3.61850300
H	7.01638500	3.36053100	0.69413800
C	5.49473000	3.55816300	2.92297700
H	6.34500700	4.07859800	3.38535900
H	5.03769600	4.26365700	2.21635900
H	4.76549200	3.34912200	3.71459700
H	7.37228600	-1.63479300	-2.09982900
C	-1.67143400	0.63204700	-1.26283600
H	-1.36908300	1.26252300	-2.10873100
H	-0.97844600	1.20146800	0.88639200
C	0.45123100	-0.66160900	-1.51269700
H	0.52438000	-0.10247500	-2.45952200
H	0.40191600	-1.72935100	-1.78537300
C	1.74673700	-0.43167800	-0.70311600
C	1.63214200	-1.04191700	0.69227900
H	1.33708500	-2.09964800	0.65413200
H	2.58572000	-0.97194800	1.23473300
H	0.88410100	-0.52093200	1.30713900
C	2.00858400	1.06941200	-0.59752100
H	2.20127100	1.51752400	-1.58421600
H	1.14192100	1.59706300	-0.16831700
H	2.86999700	1.28860800	0.04982900
C	2.86746300	-1.12844900	-1.49631600
H	2.77638300	-0.84669300	-2.56026700
H	2.68593800	-2.21739900	-1.45287300
C	4.28241700	-0.82517500	-1.03180600
H	4.35513900	-0.96235400	0.05639100
H	4.54100100	0.22551600	-1.23395000
C	5.30808600	-1.71303100	-1.71267300

H	5.18746200	-1.65454900	-2.80332800
H	5.15848400	-2.76471100	-1.41989200
C	-0.83713300	-0.33264000	-0.84278600
H	-1.12770200	-0.96251800	0.00787300
C	-0.62942300	6.18608400	-3.29368000
H	0.13737800	5.78320000	-3.95927700
H	-1.48331500	6.54571800	-3.87876000
H	-0.22792500	7.04897000	-2.74917400
C	-1.09326500	5.16048000	-2.31415500
O	-2.03835500	5.44124000	-1.50985000
O	-0.55202200	4.01214300	-2.27029400
Zn	-1.86243500	3.55478000	-0.75038400
O	-0.98986400	3.55541600	1.10223800
C	-0.83656300	2.73879300	2.01479600
O	-0.87583900	1.43861100	1.83883400
C	-0.57398800	3.13809500	3.41878600
H	0.41075300	2.76338700	3.72313700
H	-0.60897900	4.22412100	3.51994200
H	-1.31301600	2.67027600	4.07931600
C	-1.08184200	-3.57646100	-0.36212000
O	-0.96508400	-3.19376300	0.80159500
C	-0.00290600	-4.52436600	-0.92784300
F	1.05584500	-4.62843900	-0.12407600
F	-0.50097200	-5.75319200	-1.10678800
F	0.44961900	-4.10078300	-2.11967900
O	-1.98423200	-3.30447100	-1.20831000

TS6

O	-3.94030200	-1.40354900	-0.20028600
N	-2.80394600	0.42897400	0.64097100
C	-2.07839400	-0.30480400	-1.39141000
C	-1.09448000	0.53836700	-0.64346900
C	-3.07972800	-0.50639600	-0.29293200
C	-0.07988900	-0.05115100	0.24995500
H	-1.65444300	-1.27677200	-1.69581700

H	-0.88468700	1.52542600	-1.07644000
C	-3.30816300	3.77602400	2.83812600
C	-3.26946600	1.46481800	2.77924600
C	-3.53578300	1.37410100	4.16901300
C	-3.70120100	2.58694300	4.87569000
C	-3.59159200	3.78528500	4.21684900
H	-3.20245200	4.70785400	2.28226200
C	-3.09534400	0.27888200	2.00728900
C	-3.60228000	0.10174000	4.78013700
H	-3.91004800	2.55114000	5.94457800
H	-3.71201100	4.73228300	4.73598400
C	-3.39345500	-1.02908800	4.02940800
C	-3.12875900	-0.94479800	2.65012400
H	-3.80426000	0.03783300	5.84845500
H	-3.42561500	-2.00984800	4.49872800
H	-2.95981400	-1.85502200	2.07960700
N	-3.15348900	2.66309100	2.14998600
H	-2.49748100	0.20354900	-2.26977900
Cu	-2.56663000	2.33295700	0.15685200
H	-0.01530500	0.54798200	1.17388200
H	-0.35481000	-1.08303800	0.51680200
C	1.33640800	-0.04550900	-0.40603700
C	2.20659800	-0.92118700	0.51062700
H	2.09480400	-0.55793800	1.54781500
H	1.79231900	-1.94338400	0.49374200
C	1.25886800	-0.64472500	-1.81007400
H	0.82123400	-1.65291400	-1.78167500
H	0.65442400	-0.01598300	-2.48340800
H	2.25537400	-0.71369700	-2.26581200
C	1.87027800	1.38318200	-0.46789300
H	2.84417900	1.41580600	-0.97389300
H	1.20485900	2.05248300	-1.03208700
H	2.00435500	1.80442400	0.53985500
C	3.69085200	-0.96784100	0.17711600
H	3.85786200	-1.27094500	-0.86793000

H	4.14849200	0.02375100	0.30396700
C	4.41533300	-1.95093500	1.07807400
H	4.00934400	-2.96114100	0.93616400
H	4.25636100	-1.69191600	2.13611800
N	5.85069100	-2.03406200	0.75227400
S	6.90788400	-1.06778200	1.61802800
O	6.16199800	0.13402100	1.97334800
O	7.57663800	-1.84043000	2.66262400
C	8.12217000	-0.66892200	0.38729300
C	9.45596700	-0.97838100	0.61428100
C	7.72252900	-0.00304700	-0.77082500
C	10.40308300	-0.62091100	-0.33997700
H	9.74362000	-1.49354300	1.52809800
C	8.67818700	0.34484700	-1.70967200
H	6.67048000	0.22850700	-0.93243800
C	10.03149800	0.04171000	-1.50900900
H	11.45267700	-0.86098700	-0.17176300
H	8.37812000	0.86317800	-2.62072100
C	11.04633600	0.42643800	-2.54068000
H	10.83631500	-0.05674400	-3.50475600
H	11.03880100	1.50991200	-2.72133100
H	12.06117900	0.14346400	-2.23811400
H	6.21279100	-2.98583800	0.72632200
C	-7.52236100	-4.56900000	-2.23812200
H	-7.44421700	-5.09792100	-3.19518600
H	-7.88736400	-5.25166200	-1.46714700
H	-8.23958000	-3.75253200	-2.38161000
C	-6.18962300	-4.00062100	-1.87629700
O	-5.62668500	-3.17739000	-2.66732900
O	-5.61481800	-4.32936000	-0.79472200
Zn	-4.06506000	-3.08002200	-1.35120400
O	-2.19585100	-3.47747100	-1.54719000
C	-1.47539600	-3.78015400	-0.50050400
O	-0.37042800	-3.29904700	-0.28274500
C	-2.09451900	-4.78257900	0.45334400

H	-2.94943100	-4.33686700	0.98662700
H	-2.48131900	-5.65031900	-0.09544100
H	-1.36615600	-5.11310500	1.19956100
C	-1.52695800	5.32884600	-2.55436300
C	-1.64652700	3.92719000	-1.92309600
O	-1.12418900	2.97442800	-2.49424200
O	-2.30339600	3.95558300	-0.83442800
F	-2.73833900	5.84168500	-2.79865500
F	-0.89571300	6.16564400	-1.72127500
F	-0.85398300	5.31308200	-3.70010900

8′

O	-4.08714400	-0.68122000	-0.33931600
N	-2.21171100	-0.10520200	0.92819700
C	-1.78007400	0.09357200	-1.10590700
C	-0.95279600	0.28799300	0.19073000
C	-2.91458800	-0.29985500	-0.20301800
C	0.21988300	-0.63136000	0.43160200
H	-1.44740400	-0.74700300	-1.72831400
H	-0.70893600	1.33817800	0.40384500
C	-3.37487200	2.49932400	3.99884300
C	-2.84210900	0.35508200	3.27353400
C	-3.02717100	-0.15601400	4.58874100
C	-3.38737600	0.74649500	5.61160300
C	-3.55510900	2.07549400	5.32276900
H	-3.51421400	3.54631400	3.73570900
C	-2.45843900	-0.55388600	2.24967800
C	-2.85393200	-1.53698600	4.84126500
H	-3.52676000	0.36709500	6.62325400
H	-3.82898400	2.79914300	6.08492000
C	-2.50634400	-2.39138700	3.82860000
C	-2.30103400	-1.89453700	2.52696000
H	-3.00414700	-1.90158400	5.85662000
H	-2.37544800	-3.45364900	4.02099200
H	-2.00265700	-2.56275800	1.71442100

N	-3.03746100	1.68401400	3.00792200
H	-1.96197700	0.98312000	-1.71858700
Cu	-2.91563600	2.45114800	1.25291000
H	0.34057800	-0.76633500	1.52036900
H	-0.02159700	-1.62328700	0.01479400
C	1.56071700	-0.11957700	-0.13232800
C	2.54545000	-1.29544100	-0.00946600
H	2.49236900	-1.68501000	1.02268500
H	2.18141300	-2.11274700	-0.65723000
C	1.40443400	0.29454300	-1.59554900
H	1.02393000	-0.53408300	-2.21134200
H	0.71899900	1.14840500	-1.70736500
H	2.36458600	0.61249300	-2.02383400
C	2.04953000	1.07662900	0.68378300
H	2.98534500	1.48106300	0.27510700
H	1.32531800	1.90367900	0.67829900
H	2.23877300	0.79318800	1.73037600
C	4.00414200	-1.01097700	-0.33686200
H	4.11269700	-0.55461200	-1.33320100
H	4.43222600	-0.30545800	0.38965300
C	4.82146500	-2.28919700	-0.30547400
H	4.44649000	-2.99455300	-1.05899800
H	4.71772200	-2.78675400	0.67099700
N	6.23695400	-2.04626500	-0.64026000
S	7.30812700	-1.79733900	0.62146400
O	6.54206600	-1.16435400	1.68880900
O	8.06923000	-3.01360100	0.89865000
C	8.42833000	-0.61586700	-0.08387700
C	9.78348500	-0.90858500	-0.14889100
C	7.93314600	0.61220000	-0.52036800
C	10.65505200	0.04407300	-0.66699700
H	10.14556200	-1.87266700	0.20149100
C	8.81452400	1.54818600	-1.03318400
H	6.86561200	0.82172100	-0.46597400
C	10.18736900	1.27928700	-1.11342600

H	11.72060400	-0.17722000	-0.72469300
H	8.43918400	2.51066000	-1.38163400
C	11.12171900	2.31033700	-1.66692100
H	10.81991100	2.62165700	-2.67608800
H	11.12655500	3.21570500	-1.04444300
H	12.15127600	1.93829900	-1.72306000
H	6.63212100	-2.73517000	-1.27831900
C	-8.05120200	-1.43925400	-3.69929800
H	-7.95612100	-1.72839800	-4.75217300
H	-8.82148600	-2.04368700	-3.21467600
H	-8.34579900	-0.38323200	-3.68293100
C	-6.73053800	-1.59149500	-3.01898500
O	-5.71471300	-0.98478100	-3.48939600
O	-6.61078000	-2.31006900	-1.98164300
Zn	-4.60773000	-1.79099400	-1.97862600
O	-3.03767000	-2.89837600	-1.98089200
C	-2.69893600	-3.46654000	-0.85828200
O	-1.58615300	-3.35966000	-0.35124100
C	-3.78726100	-4.25933200	-0.16142400
H	-4.44392800	-3.57045700	0.39405900
H	-4.41293600	-4.80245500	-0.87986800
H	-3.35697600	-4.96083900	0.56064400
C	-1.44555400	4.10420000	-2.18075700
C	-1.60476300	3.65356100	-0.71664900
O	-0.61989700	3.68295300	0.01062500
O	-2.77960000	3.22010600	-0.46529000
F	-1.27075800	3.01132800	-2.94915900
F	-2.51514700	4.75008800	-2.64128500
F	-0.38706300	4.88805900	-2.35296400

TS7

O	-3.17596000	2.00985100	-1.57394300
N	-2.60717200	-0.22881800	-1.36265800
C	-2.35077000	1.04787500	-1.50354200
C	-3.13159700	-3.78640000	0.45693300

C	-4.07596700	-2.01229000	-0.71155600
C	-5.35555600	-2.62894000	-0.67488000
C	-5.46616000	-3.87657100	-0.02349100
C	-4.36157700	-4.45778600	0.54480200
H	-2.23195100	-4.22060900	0.89070100
C	-3.90631300	-0.73668700	-1.34374700
C	-6.45988100	-1.98679700	-1.27821200
H	-6.44053300	-4.36275900	0.01826000
H	-4.41757200	-5.41639900	1.05344000
C	-6.28115500	-0.77552000	-1.89515500
C	-5.01891100	-0.15264800	-1.92945200
H	-7.43656500	-2.46710600	-1.24118100
H	-7.12340500	-0.27289100	-2.36675900
H	-4.91557600	0.80781500	-2.42350700
N	-2.99175400	-2.61654700	-0.14207600
Cu	-1.30514300	-1.58595200	-0.30149700
N	5.49429700	2.99841500	-6.96937400
S	5.19329300	4.40245500	-7.81392300
O	5.93404200	4.26514800	-9.05913900
O	3.75197900	4.60678200	-7.79318100
C	5.93162100	5.72315600	-6.87812500
C	7.30421000	5.94451500	-6.98608100
C	5.14790500	6.49283000	-6.02563100
C	7.88755400	6.94644400	-6.22745900
H	7.90132300	5.34823700	-7.67452000
C	5.75049900	7.49394700	-5.27170900
H	4.07505400	6.31882900	-5.97107200
C	7.12190800	7.73472900	-5.35976700
H	8.95857000	7.13298500	-6.31054100
H	5.14203100	8.10583600	-4.60617400
C	7.77037300	8.82441400	-4.56352100
H	8.66303700	8.46021400	-4.03815100
H	8.09821800	9.64707900	-5.21415200
H	7.08570100	9.24502100	-3.81806000
H	6.47050600	2.72866500	-7.06879900

C	-0.88309600	1.40615400	-1.58239000
H	-0.65276600	2.48567700	-1.55958800
H	-0.40337700	1.09762600	-0.52192600
C	1.35000400	1.05889200	-2.67881300
H	2.01993300	0.21144700	-2.45809200
H	1.63893700	1.92113500	-2.05865800
C	1.56155600	1.42431700	-4.19298400
C	0.58461000	2.53082500	-4.57982800
H	0.66548900	3.39662600	-3.90602400
H	0.77296300	2.88475000	-5.60096900
H	-0.45806300	2.18099700	-4.55299400
C	1.34594900	0.19025900	-5.06220400
H	2.05994800	-0.60838300	-4.81304300
H	0.33044500	-0.21582900	-4.95993600
H	1.47679200	0.43825800	-6.12303200
C	3.01536400	1.91673600	-4.28328600
H	3.67080800	1.17121400	-3.80030700
H	3.10314800	2.83699800	-3.67984300
C	3.53680100	2.18710800	-5.68757900
H	2.86631200	2.85407000	-6.24643500
H	3.61015600	1.25479500	-6.26576000
C	4.91729400	2.82092900	-5.63452500
H	5.60802200	2.17359000	-5.07737300
H	4.88214500	3.77812000	-5.08375000
C	-0.01876700	0.65163000	-2.37862900
H	-0.37075800	-0.31728300	-2.75192000
C	0.28248700	4.33900500	1.08356500
H	0.11436800	4.63310100	2.12394100
H	0.77059500	3.35384900	1.09801400
H	0.94208300	5.05422600	0.58428000
C	-1.01796400	4.20527700	0.35999500
O	-1.08605700	4.49512500	-0.88154100
O	-2.03374600	3.75107000	0.96007200
Zn	-3.08202500	3.87541000	-0.84691800
O	-4.63271500	4.91679800	-1.17500700

C	-5.79760700	4.62566400	-0.64966500
O	-6.82721500	5.21093500	-0.93010100
C	-5.78646900	3.49124900	0.36395600
H	-5.40215700	2.56328700	-0.08755100
H	-5.13489800	3.74218400	1.21393900
H	-6.79380100	3.29886600	0.74513400
C	1.02438800	-0.24513600	0.59159400
O	0.44520600	0.84424600	0.76964100
C	2.49227500	-0.33661100	1.04844400
F	2.80000500	0.59359900	1.94218000
F	2.77392900	-1.52742200	1.56718600
F	3.28223100	-0.15753000	-0.02319500
O	0.59946300	-1.27230600	0.00731500

9′

O	4.27425100	-3.49887000	1.90342500
N	4.20951800	-1.51148000	0.73204300
C	2.45151500	-1.94886600	2.26613400
C	1.82267300	-0.83173700	1.48237500
C	3.73665800	-2.44420700	1.62187800
C	0.78998400	-1.19216500	0.45065100
H	1.76276500	-2.80000000	2.36484700
H	1.61049000	0.07245100	2.06190400
C	5.01945000	1.63787200	-1.48906100
C	5.72689900	-0.45521700	-0.75681000
C	6.92731000	-0.36910300	-1.50091700
C	7.12934900	0.80783000	-2.25569800
C	6.18710400	1.80765100	-2.25258800
H	4.23860400	2.39877000	-1.45957400
C	5.42237900	-1.59495000	0.04484100
C	7.83934700	-1.44722700	-1.44786200
H	8.04473900	0.90822700	-2.83852000
H	6.32657600	2.72001200	-2.82599200
C	7.53487900	-2.54237200	-0.67837800
C	6.34048800	-2.63181900	0.06717600

H	8.76583700	-1.39329300	-2.01730000
H	8.23119500	-3.37811000	-0.63197200
H	6.13484500	-3.51034400	0.66788300
N	4.80742500	0.55024000	-0.77622100
H	2.70965100	-1.62344900	3.28375500
Cu	3.24611900	0.03881300	0.38405400
H	0.91329000	-0.59497800	-0.46640900
H	0.93047900	-2.24703900	0.16127800
C	-0.68022900	-0.96818300	0.90733900
C	-1.55399000	-1.54808400	-0.21839800
H	-1.23838500	-1.09015900	-1.17206900
H	-1.32942200	-2.62618600	-0.30920600
C	-0.94705300	-1.69633200	2.22165900
H	-0.77374800	-2.77910400	2.12644900
H	-0.30555300	-1.32064400	3.03203400
H	-1.98470700	-1.55416500	2.55186400
C	-0.94710000	0.52591000	1.07181600
H	-1.97029200	0.70564300	1.42756100
H	-0.27476600	0.98842500	1.80808400
H	-0.81828000	1.06415100	0.12256200
C	-3.05804700	-1.36673800	-0.07374300
H	-3.42462100	-1.79043800	0.87421700
H	-3.31928700	-0.29918700	-0.07559500
C	-3.79779900	-2.04160600	-1.21427400
H	-3.58509500	-3.11916300	-1.21739000
H	-3.45124800	-1.64829200	-2.18214500
N	-5.25873900	-1.91368500	-1.06863800
S	-6.01281700	-0.63032800	-1.83826200
O	-5.03266300	0.44813500	-1.88699300
O	-6.66008500	-1.08037800	-3.06828800
C	-7.29232700	-0.21667800	-0.68108400
C	-8.61464900	-0.19212500	-1.10294000
C	-6.94485000	0.13048100	0.62330800
C	-9.60431200	0.17900000	-0.19904800
H	-8.86056900	-0.46491700	-2.12677100

C	-7.94294000	0.49634200	1.51004100
H	-5.90266400	0.10011500	0.93846000
C	-9.28570900	0.52860800	1.11258600
H	-10.64552600	0.19740900	-0.52006500
H	-7.68455900	0.76400300	2.53481700
C	-10.34649900	0.94649900	2.08273400
H	-10.22197700	0.44851700	3.05322100
H	-10.30295000	2.02835500	2.27258600
H	-11.35175200	0.71777200	1.70984700
H	-5.76873100	-2.77004500	-1.27907000
C	1.62803200	3.78356900	0.40701400
C	2.34187200	2.48732900	0.83165800
O	3.01792200	2.46157300	1.84727700
O	2.14073900	1.54432200	-0.01274900
F	2.13934500	4.21645300	-0.75468000
F	0.32116000	3.57879100	0.22454700
F	1.77170900	4.75136300	1.30249100

TS8

O	-4.14418700	1.80010800	-1.41562500
N	-3.02496900	-0.24328900	-1.41459700
C	-3.13356500	1.10796800	-1.50200300
C	-2.44185500	-4.22461200	-1.61526600
C	-3.89799700	-2.47820100	-1.15032700
C	-4.93684300	-3.38712400	-0.80845100
C	-4.64889100	-4.76744700	-0.89441000
C	-3.40900500	-5.19435100	-1.29670200
H	-1.44258500	-4.51782000	-1.94119400
C	-4.11341400	-1.05585500	-1.09395000
C	-6.19679100	-2.89405600	-0.40575800
H	-5.43136300	-5.48175800	-0.63707500
H	-3.16613400	-6.25141500	-1.37192800
C	-6.39148100	-1.53775300	-0.35653700
C	-5.37395400	-0.62585400	-0.69573300
H	-6.98836700	-3.59645800	-0.14726400

H	-7.35822800	-1.13960700	-0.05094700
H	-5.57208500	0.43806500	-0.65728500
N	-2.67730900	-2.93188700	-1.54162000
Cu	-1.46540900	-1.22084000	-1.99662100
N	6.94027500	0.80265000	-1.75638700
S	7.56081500	-0.75422900	-1.81748000
O	6.42248500	-1.65364600	-1.67414900
O	8.46584700	-0.90285500	-2.95451300
C	8.54306600	-0.80004700	-0.34085300
C	9.90375700	-1.05801900	-0.42939800
C	7.92121400	-0.62102800	0.89407800
C	10.65405000	-1.13006400	0.73982100
H	10.36489500	-1.20020700	-1.40423000
C	8.68299100	-0.69565700	2.04706000
H	6.85201500	-0.41838900	0.94297000
C	10.05931300	-0.95135200	1.98774300
H	11.72346700	-1.33108900	0.68080200
H	8.20935300	-0.55533700	3.01906900
C	10.86158000	-1.02904100	3.24931000
H	10.79713400	-0.09308500	3.82083000
H	10.49190000	-1.82782600	3.90664300
H	11.92053000	-1.22588100	3.04610800
H	7.63972900	1.44497400	-2.12630700
C	-1.80373900	1.81284500	-1.76040500
H	-1.68367300	2.00544300	-2.83882200
H	-1.86897800	2.81291500	-1.29808300
C	0.68435000	1.39183100	-1.69854500
H	0.76243300	2.28048600	-2.34781400
H	0.64023500	0.56753500	-2.70143600
C	1.94721100	1.07985400	-0.88454400
C	1.89351000	-0.32979800	-0.29818500
H	1.96398400	-1.10666100	-1.07225100
H	2.72468800	-0.49187100	0.39939500
H	0.97173100	-0.50768800	0.27305200
C	2.03124400	2.10059900	0.25324300

H	2.04813300	3.12969900	-0.13428300
H	1.16611500	2.01050300	0.92633400
H	2.93528100	1.95409100	0.85958800
C	3.15003500	1.23171800	-1.83323200
H	3.16682100	2.27358600	-2.20124300
H	2.97940200	0.60202200	-2.71986700
C	4.51367900	0.88365100	-1.25653300
H	4.52585600	-0.15309700	-0.89103100
H	4.77136500	1.53388300	-0.40593200
C	5.59340400	1.02276800	-2.31419500
H	5.59722100	2.04422200	-2.71828500
H	5.38599400	0.34867300	-3.16025700
C	-0.59573200	1.15894200	-1.21559400
H	-0.73268700	0.54019200	-0.32293800
C	0.91580900	-1.40547400	-3.53707900
O	0.94309400	-0.17192100	-3.79546800
O	0.10426000	-2.02853600	-2.83047000
C	2.09004100	-2.20094800	-4.13387100
F	2.27670900	-1.88517300	-5.41169000
F	3.20253900	-1.88214400	-3.45887200
F	1.91065700	-3.51075600	-4.04471400

10′

O	-5.69950300	3.09327000	0.15678600
N	-4.67875200	1.01027000	0.31289100
C	-4.71168900	2.35872200	0.13208600
C	-4.34271400	-3.02268900	0.49028900
C	-5.68551400	-1.14434800	0.76673100
C	-6.78339000	-1.95444400	1.17419400
C	-6.58631400	-3.35226100	1.21656200
C	-5.37379400	-3.89537200	0.87639500
H	-3.36043500	-3.41029500	0.21440400
C	-5.81161500	0.29241400	0.68920700
C	-8.01449300	-1.35536900	1.51520900
H	-7.41772700	-3.98715800	1.52409100

H	-5.19980400	-4.96826500	0.90160500
C	-8.12423100	0.00940900	1.44261600
C	-7.05196500	0.82350000	1.03492500
H	-8.84857500	-1.98442200	1.82410400
H	-9.06648400	0.49183600	1.70038800
H	-7.18588300	1.89608200	0.97678300
N	-4.49360600	-1.71526100	0.44009200
Cu	-3.20102600	-0.16197300	-0.16623200
N	5.18827600	1.38490400	-0.79908200
S	5.59589100	-0.22369700	-1.02770900
O	4.36231400	-0.98362900	-0.86297400
O	6.39142600	-0.38642200	-2.24222700
C	6.66429500	-0.52018300	0.35685300
C	7.95207300	-0.99141000	0.14141600
C	6.17235900	-0.32849700	1.64684600
C	8.76133000	-1.26661800	1.23839600
H	8.31081800	-1.13996600	-0.87465900
C	6.99179000	-0.60727700	2.72720300
H	5.15895100	0.04267900	1.79546200
C	8.29744600	-1.07863800	2.53991800
H	9.77383000	-1.63715400	1.07983600
H	6.61858000	-0.46108300	3.74103200
C	9.17390700	-1.35585400	3.72151400
H	9.51314400	-0.42129700	4.19019400
H	8.63822300	-1.92260600	4.49403500
H	10.06686100	-1.92613900	3.44027200
H	5.92910400	1.97180200	-1.17928200
C	-3.36949300	3.03147000	-0.15783600
H	-3.27130500	3.12425300	-1.25305900
H	-3.48774400	4.06326500	0.20310000
C	-0.88401800	2.73105600	0.03103100
H	-0.77211600	3.42424000	-0.81519600
H	-1.48213400	1.29077200	-1.57064200
C	0.42031500	2.31689800	0.68457200
C	0.30831400	0.97038700	1.39483200

H	0.15921900	0.14168600	0.68566100
H	1.22169300	0.75165800	1.96282700
H	-0.52085200	0.95212800	2.11434600
C	0.77302200	3.40037300	1.71206400
H	0.83491700	4.39193300	1.24109700
H	0.00478100	3.45146200	2.49584900
H	1.73630700	3.19674400	2.20039500
C	1.49371100	2.26220900	-0.42016800
H	1.58865900	3.27447100	-0.85278600
H	1.12540000	1.62231500	-1.23850800
C	2.86998100	1.75998200	-0.01287300
H	2.81138400	0.71895600	0.33676400
H	3.29052600	2.35686700	0.81155500
C	3.83260100	1.81371000	-1.18514900
H	3.92966600	2.84571200	-1.54827500
H	3.44377400	1.21854000	-2.02620900
C	-2.12781400	2.43464700	0.43546100
H	-2.27051400	1.80325700	1.31742800
C	-0.90471700	-0.47832500	-1.86193300
O	-0.96568700	0.76748300	-2.24309500
O	-1.58431900	-1.01260000	-1.00451100
C	0.21449600	-1.23938600	-2.58353700
F	0.17848200	-1.00077200	-3.88674400
F	1.37916200	-0.81421400	-2.09765500
F	0.10398900	-2.53665200	-2.37322600

TS9

O	-2.04785000	1.53346200	-0.19086000
N	-2.84151200	-0.50994400	-1.01478500
C	-2.47059300	0.38233000	-0.08617500
C	-4.51379800	-3.15422900	-3.53841500
C	-3.26663200	-1.21301200	-3.26941700
C	-3.13022500	-1.06612200	-4.67702800
C	-3.73431800	-2.04263100	-5.49872800
C	-4.42564000	-3.08637100	-4.93888000

H	-5.05630100	-3.96690800	-3.05563900
C	-2.67957100	-0.25467000	-2.36998400
C	-2.41206000	0.03059600	-5.20181600
H	-3.64088800	-1.94792200	-6.58076800
H	-4.90053300	-3.85019500	-5.54940500
C	-1.85939800	0.93974000	-4.33617100
C	-1.98783100	0.80768600	-2.94111400
H	-2.31401800	0.13577900	-6.28161000
H	-1.30515700	1.79227700	-4.72654700
H	-1.54656100	1.55149700	-2.28755500
N	-3.96049100	-2.26075000	-2.74084300
Cu	-3.99595600	-2.16247100	-0.69676500
N	2.75458100	-2.63256700	7.54112300
S	3.38933100	-1.28023300	8.30415400
O	2.87944700	-0.12714900	7.57123700
O	3.19515300	-1.36151600	9.74970000
C	5.12132800	-1.47569000	7.97971300
C	6.01668300	-1.52564400	9.03894800
C	5.56250500	-1.52198800	6.65786800
C	7.37672000	-1.63222500	8.76700800
H	5.64854200	-1.48095200	10.06152900
C	6.91905000	-1.62898900	6.40595800
H	4.84371600	-1.48250400	5.84032300
C	7.84574600	-1.68529400	7.45530300
H	8.08801500	-1.67305900	9.59158100
H	7.27736600	-1.66899100	5.37703900
C	9.30804900	-1.80230900	7.15713300
H	9.91103200	-1.80488800	8.07235900
H	9.52906200	-2.72735900	6.60715400
H	9.65344800	-0.97009500	6.52896900
H	2.86420800	-3.43803600	8.15595500
C	-2.55398300	-0.13378000	1.35231100
H	-2.61167800	0.65743800	2.11164500
H	-3.64241500	-0.70020800	1.50941600
C	-1.58871400	-1.66027600	3.11547400

H	-1.95964200	-2.68632500	3.28111200
H	-2.10951900	-0.98412500	3.80930000
C	-0.06036400	-1.64974900	3.43976100
C	0.48307600	-0.23993900	3.22360900
H	-0.02948700	0.49106600	3.86603000
H	1.55546800	-0.18869300	3.45088400
H	0.36168500	0.08938600	2.18100200
C	0.67135900	-2.64210100	2.54073500
H	0.27242900	-3.66082000	2.65553700
H	0.60013900	-2.36613300	1.47942600
H	1.74153200	-2.67283800	2.78181900
C	0.04518700	-2.06401700	4.91607700
H	-0.42861500	-3.05478700	5.03293700
H	-0.56207200	-1.36371200	5.51508200
C	1.44774700	-2.11454900	5.50375800
H	1.94040900	-1.13545100	5.42522800
H	2.07901200	-2.84143700	4.97044700
C	1.40231300	-2.51117900	6.96864600
H	0.93437700	-3.49879800	7.07713700
H	0.78052200	-1.80409700	7.53954500
C	-1.88203400	-1.28360500	1.72865300
H	-1.63609200	-2.00017800	0.93557100
O	-4.74139100	-3.12929500	0.83773100
C	-5.06945500	-2.49435800	1.86399700
O	-4.91691500	-1.28344500	2.13354200
C	-5.67598500	-3.32673900	3.00813700
F	-4.73411000	-3.49730800	3.95065100
F	-6.08108100	-4.52847600	2.61574300
F	-6.70706800	-2.70407700	3.57141000

11′

O	-1.23454300	3.41807400	-2.93791100
N	-1.96051900	1.27828700	-3.52104000
C	-1.53339100	2.25017200	-2.67455800
C	-3.56519400	-1.71969400	-5.69035900

C	-2.53541000	0.36492800	-5.67027000
C	-2.56055900	0.42418800	-7.09188300
C	-3.12520200	-0.66952500	-7.78335500
C	-3.62832900	-1.74278700	-7.09312300
H	-3.95600900	-2.55214700	-5.10442500
C	-1.97385000	1.44630900	-4.89611200
C	-2.03405200	1.55101800	-7.75944600
H	-3.15290100	-0.64150700	-8.87296500
H	-4.06824800	-2.59570100	-7.60387000
C	-1.50599200	2.57481300	-7.01457400
C	-1.47440900	2.53317000	-5.60912200
H	-2.06011600	1.58684500	-8.84790700
H	-1.09729200	3.45258000	-7.51385800
H	-1.05873000	3.36508200	-5.05349300
N	-3.04360500	-0.71590400	-5.01232900
Cu	-2.88300000	-0.37333700	-2.97048100
N	3.51907000	-1.06979700	5.12695400
S	4.13906400	0.19997100	6.02721800
O	3.60481500	1.41982400	5.43339900
O	3.96267300	-0.05028900	7.45583900
C	5.87268100	0.07773500	5.67176400
C	6.77727700	-0.07635600	6.71276500
C	6.30404700	0.18690600	4.35042700
C	8.13671600	-0.12948400	6.42245700
H	6.41643300	-0.15415800	7.73592900
C	7.66017100	0.13154200	4.07984700
H	5.57839000	0.30471800	3.54653000
C	8.59610000	-0.02689900	5.11034700
H	8.85508100	-0.25204200	7.23271600
H	8.01060200	0.21258900	3.05058200
C	10.05789800	-0.08184100	4.79194400
H	10.66510200	-0.23175200	5.69205400
H	10.28332100	-0.89951400	4.09387700
H	10.39462800	0.84677700	4.31118000
H	3.60808900	-1.92966700	5.66592900

C	-1.38623300	1.84195200	-1.23773100
H	-1.51712300	2.66373300	-0.52618300
H	-3.18548300	0.69741800	-0.55299700
C	-0.63551300	0.32759900	0.62739000
H	-1.12532900	-0.63136100	0.87935600
H	-1.08401600	1.09436400	1.28102900
C	0.86389100	0.19480300	0.98539100
C	1.54378700	1.55065800	0.82226200
H	1.13225700	2.29066200	1.52525200
H	2.62497200	1.48407700	1.00292700
H	1.40831500	1.94453500	-0.19431300
C	1.52847000	-0.83234700	0.07221900
H	1.01428300	-1.80481200	0.12002500
H	1.52747500	-0.50032500	-0.97501100
H	2.57830600	-0.99626600	0.34912300
C	0.91584500	-0.26984700	2.44982500
H	0.41413300	-1.25254700	2.51776300
H	0.30463700	0.42343800	3.05433100
C	2.29351900	-0.37525300	3.08685600
H	2.78724200	0.60675300	3.10657200
H	2.94686000	-1.05534700	2.51857800
C	2.19236700	-0.89266000	4.50991000
H	1.72045700	-1.88455100	4.51624600
H	1.54913000	-0.23386600	5.11340200
C	-0.92356800	0.66004200	-0.80034300
H	-0.72372600	-0.12081300	-1.54295200
O	-3.74964700	-1.43529700	-1.49703400
C	-4.10132200	-0.95857300	-0.43256500
O	-3.85756400	0.23678600	0.02345700
C	-4.88827500	-1.79437600	0.58512800
F	-4.08983400	-2.06355300	1.61589200
F	-5.29034500	-2.92857200	0.04331800
F	-5.94234000	-1.12214200	1.02544100

TS10

O	4.53923000	-1.82813300	3.24615900
N	3.83544400	-1.02929000	1.16721900
C	2.95553700	0.03787300	2.97854000
C	2.19755800	0.17152900	1.69613700
C	3.91451100	-1.05462500	2.55906900
C	1.09268500	-0.76029400	1.33935400
H	2.33229200	-0.29354000	3.81977400
H	2.10481900	1.19996100	1.31943100
C	4.29547700	-0.47835800	-2.79959200
C	4.15744300	-1.97400500	-1.03779100
C	4.30720500	-3.08598500	-1.90566800
C	4.45719500	-2.81341200	-3.28427300
C	4.45208300	-1.51769500	-3.73527700
H	4.28843400	0.56575700	-3.11342700
C	3.99479300	-2.16890100	0.36934700
C	4.28348100	-4.39218300	-1.36924000
H	4.57278200	-3.64697700	-3.97676600
H	4.56461200	-1.28290100	-4.79043600
C	4.10755300	-4.56569300	-0.01848100
C	3.95298900	-3.46887200	0.84876500
H	4.39616700	-5.24394000	-2.03854300
H	4.08287100	-5.56917100	0.40196000
H	3.83694800	-3.64167300	1.91464100
N	4.15128100	-0.70040300	-1.50951000
H	3.46652500	0.96636000	3.26349000
Cu	3.77941900	0.59376600	0.10969500
H	1.23466100	-1.12021900	0.30567700
H	1.12915800	-1.65158700	1.99017800
C	-0.30803700	-0.10196500	1.40838800
C	-1.32126400	-1.24547500	1.23702900
H	-1.03688900	-1.83441400	0.34730600
H	-1.21553500	-1.92922400	2.09875800
C	-0.49593600	0.58601300	2.75910200
H	-0.36894000	-0.12290200	3.59156500
H	0.22210100	1.40828200	2.89851300

H	-1.49732900	1.02719700	2.84850500
C	-0.44806000	0.91611500	0.27799800
H	-1.41045400	1.44149200	0.33421800
H	0.33161700	1.69058400	0.31764300
H	-0.39114600	0.42721700	-0.70620300
C	-2.78164700	-0.84160600	1.09786300
H	-3.09798800	-0.18282600	1.92151500
H	-2.93939700	-0.28634600	0.16236600
C	-3.68190100	-2.06319900	1.09039600
H	-3.59033400	-2.60514800	2.04128700
H	-3.37277800	-2.76284600	0.29852900
N	-5.10205900	-1.69240300	0.96058500
S	-5.76575100	-1.70631200	-0.57822900
O	-4.68958000	-1.32473600	-1.48542200
O	-6.50393900	-2.94568100	-0.81085000
C	-6.94677900	-0.38865400	-0.46758300
C	-8.28743600	-0.64634000	-0.71736500
C	-6.50037600	0.89964500	-0.17621700
C	-9.19652700	0.40515000	-0.66635900
H	-8.60991400	-1.65944500	-0.94720600
C	-7.41897700	1.93379400	-0.12979900
H	-5.44433500	1.08086000	0.02041400
C	-8.77898100	1.70291300	-0.37493900
H	-10.25189500	0.21313000	-0.85836900
H	-7.08381600	2.94545200	0.10001500
C	-9.75220300	2.83937500	-0.32872800
H	-9.68378300	3.38605400	0.62128400
H	-9.55000500	3.56594000	-1.12778200
H	-10.78597600	2.49443800	-0.44624000
H	-5.71843500	-2.20313700	1.59077200
C	3.11153100	4.63794300	-0.67792600
C	3.11965500	3.29391700	0.07800800
O	2.53423000	3.21665600	1.15443100
O	3.75228100	2.39710300	-0.56445600
F	4.35538200	5.03505800	-0.96631500

F	2.45065400	4.51083800	-1.83683100
F	2.52673400	5.60925100	0.01605400

12′

O	-4.83051200	-0.56180500	-2.98739600
N	-3.47093600	-0.78552200	-1.06674600
C	-2.80543400	0.79461100	-2.27257600
C	-2.22056100	0.04831800	-1.05258500
C	-3.91691500	-0.24267300	-2.27058600
C	-0.95245800	-0.74680200	-1.27622200
H	-2.18464500	0.78474100	-3.17493900
H	-2.15231100	0.65437200	-0.13808100
C	-4.83988200	-1.22610500	2.84592300
C	-4.19099600	-2.18412500	0.82868000
C	-4.38718100	-3.48857300	1.36265100
C	-4.80170000	-3.60421200	2.70657200
C	-5.01887500	-2.47699000	3.45430700
H	-5.03030400	-0.31171400	3.40497900
C	-3.73657700	-2.05547800	-0.51503900
C	-4.17888400	-4.62361000	0.54630600
H	-4.94627000	-4.59784100	3.12947600
H	-5.33696500	-2.52803800	4.49163100
C	-3.78596600	-4.47415600	-0.75748900
C	-3.55414100	-3.18808900	-1.27943800
H	-4.34133400	-5.61134700	0.97561000
H	-3.63260700	-5.34445000	-1.39135800
H	-3.21833600	-3.06969000	-2.30850400
N	-4.44733200	-1.07470800	1.58893700
H	-3.15256200	1.81504300	-2.07662800
Cu	-4.36944500	0.70655200	0.87033900
H	-0.97070900	-1.63961000	-0.62774200
H	-0.94757200	-1.12190400	-2.31625300
C	0.35486700	0.01744300	-0.98823800
C	1.49479200	-0.85554700	-1.54056900
H	1.35227600	-1.88722300	-1.17222000

H	1.38220500	-0.90990000	-2.63889900
C	0.34576500	1.38258800	-1.67529700
H	0.21290200	1.28801700	-2.76370000
H	-0.45584800	2.02917500	-1.28796500
H	1.28827300	1.91965700	-1.50312300
C	0.51449900	0.21057100	0.51942700
H	1.41563800	0.79367900	0.75180000
H	-0.33167700	0.75939700	0.95532400
H	0.60069500	-0.75693400	1.03715400
C	2.91523400	-0.42416500	-1.20730100
H	3.09209300	0.62796100	-1.48025100
H	3.09857600	-0.51161700	-0.12694700
C	3.92763000	-1.28614000	-1.93808100
H	3.81016300	-1.16852200	-3.02372900
H	3.75559900	-2.35063600	-1.71655600
N	5.31236100	-0.88821100	-1.62782100
S	6.10821300	-1.71677100	-0.40982800
O	5.08171400	-2.12371800	0.54293400
O	7.01172200	-2.71797200	-0.97269600
C	7.10581900	-0.43585200	0.30419900
C	8.48016000	-0.60733600	0.39370200
C	6.48551200	0.70297800	0.81623600
C	9.24520000	0.38347400	1.00030700
H	8.94048900	-1.50651100	-0.00969400
C	7.26224600	1.67877800	1.41615800
H	5.40609900	0.82254300	0.73233500
C	8.65200000	1.53379900	1.51815900
H	10.32534800	0.25919200	1.07357000
H	6.78949600	2.57571700	1.81700200
C	9.47016600	2.60162700	2.17491500
H	9.32701100	3.57309700	1.68269600
H	9.17985200	2.73359900	3.22623000
H	10.54055100	2.36677200	2.15055400
H	5.91886000	-0.83323800	-2.44428300
C	-2.98664100	4.40845300	-0.17700900

C	-3.16511000	3.05744500	0.54104100
O	-2.24292800	2.64817900	1.23625200
O	-4.26699800	2.48814000	0.24243400
F	-2.55824500	4.17326400	-1.43288200
F	-4.11896700	5.10422400	-0.26455800
F	-2.07951300	5.17802900	0.41612500

5a′

O	-3.42806800	1.54061600	1.03363400
N	-3.26570400	-0.28999100	-0.33561200
C	-2.76958800	0.78565900	0.28474300
C	-3.91302200	-3.65595900	-2.28072700
C	-4.82505400	-1.96199100	-0.97669600
C	-6.11203900	-2.54861200	-1.02292200
C	-6.24390300	-3.75317700	-1.74907500
C	-5.15452700	-4.30846100	-2.37489900
H	-3.02180700	-4.05452000	-2.76690300
C	-4.59101700	-0.74996300	-0.26587400
C	-7.17673600	-1.90883200	-0.34819200
H	-7.22128900	-4.23183900	-1.80254300
H	-5.23383200	-5.23483300	-2.93706700
C	-6.93877000	-0.74061100	0.33050900
C	-5.65668700	-0.15221000	0.38174700
H	-8.16973500	-2.35479100	-0.37624900
H	-7.75341100	-0.24137700	0.85136000
H	-5.51092200	0.77288600	0.92783100
N	-3.76210300	-2.53245600	-1.60916700
Cu	-2.08943700	-1.42703600	-1.26177600
N	6.86220200	0.22294900	-1.52441600
S	7.60743600	-0.27730200	-0.10777400
O	7.19342600	-1.62957700	0.25572600
O	9.01882000	0.03135100	-0.31170800
C	6.92653500	0.83139600	1.10142300
C	7.06898000	2.20469000	0.92162800
C	6.25340100	0.31561300	2.20234800

C	6.50503500	3.06624200	1.84990100
H	7.60360800	2.58956700	0.05470100
C	5.69420000	1.19362700	3.12311900
H	6.15870300	-0.76212200	2.32139800
C	5.80255800	2.57601900	2.95686400
H	6.60555700	4.14350600	1.71716000
H	5.16014600	0.79953100	3.98757900
C	5.15702400	3.51857300	3.92477900
H	5.75159200	4.43077100	4.05862900
H	4.16482000	3.83029300	3.56712200
H	5.01475900	3.05529000	4.90830300
H	7.55385500	0.13835500	-2.26886200
C	-1.33970100	1.05680200	-0.09329000
H	-0.83113600	1.48887100	0.78921300
C	0.61472700	-0.20016000	-1.24238700
H	0.52502800	0.52336600	-2.06554000
H	0.78173600	-1.17969800	-1.70467500
C	1.92679800	0.11102400	-0.43980300
C	2.02389200	-0.81388700	0.77091600
H	1.85217600	-1.86382400	0.48818500
H	3.01479300	-0.75123400	1.24105700
H	1.29355500	-0.54799000	1.54806200
C	1.99206900	1.56892300	0.00879000
H	1.99497600	2.25586900	-0.85187700
H	1.16024800	1.84784200	0.67092700
H	2.91251400	1.75433700	0.58116900
C	3.06642300	-0.19446100	-1.43398500
H	2.83957600	0.29265100	-2.39827300
H	3.06487100	-1.28039800	-1.63206100
C	4.45231900	0.23626700	-0.98064400
H	4.62334500	-0.09039800	0.05467100
H	4.54219400	1.33270700	-0.97947900
C	5.54659500	-0.33464100	-1.86473200
H	5.36569600	-0.05692900	-2.91230600
H	5.54800300	-1.43540400	-1.81125200

C	-0.63197800	-0.23014000	-0.42521100
H	-0.60824000	-0.93686400	0.41628700
C	0.33958600	4.95651100	0.95153700
H	0.36953600	4.93578100	-0.14553400
H	0.28840600	6.01133100	1.24519300
H	1.25114100	4.50401200	1.35239000
C	-0.88677500	4.24104000	1.43097000
O	-2.02905100	4.70107300	1.12044200
O	-0.78632800	3.18564800	2.12337900
Zn	-2.94797300	3.16279000	2.16991300
O	-3.48625400	2.91250500	4.14778300
C	-4.63614900	3.38083000	3.87089400
O	-4.91217500	3.74698300	2.69334900
C	-5.64927500	3.50879600	4.96661900
H	-5.69964900	2.57761400	5.54152600
H	-5.32919900	4.29724100	5.65913900
H	-6.63582900	3.76171600	4.56954300
C	-0.40631600	-3.43830100	-1.41159900
O	-0.63219000	-3.60564300	-0.22513200
C	0.61872600	-4.32541300	-2.13920600
F	1.00800900	-5.35079200	-1.39667200
F	0.12161100	-4.80280300	-3.28242700
F	1.70162600	-3.59133700	-2.43599000
O	-0.88406200	-2.54050700	-2.19726900
C	-1.34615900	2.11362400	-1.19966400
H	-1.79602700	1.71615300	-2.12100400
H	-1.92989200	2.98918600	-0.88724200
H	-0.33255600	2.45906800	-1.43189900

TS4a

O	-3.70625500	1.22788100	-1.13962900
N	-2.14993700	-0.40892100	-0.71222600
C	-2.51941000	0.79197000	-1.12721300
C	-1.06916600	-4.28870000	-0.55256500
C	-2.63611300	-2.66957100	-0.00381800

C	-3.44719000	-3.62743600	0.66274000
C	-2.99815500	-4.96701600	0.67542500
C	-1.81593600	-5.30602700	0.06827800
H	-0.11710900	-4.51069100	-1.03703700
C	-3.04052500	-1.29538800	-0.07084500
C	-4.64841100	-3.21804700	1.28186200
H	-3.60787600	-5.71949400	1.17548500
H	-1.44988700	-6.32941700	0.06386700
C	-5.01147200	-1.89768500	1.23188100
C	-4.22236200	-0.94281000	0.56069500
H	-5.26483600	-3.95860200	1.78973800
H	-5.92970000	-1.56283400	1.71031700
H	-4.56431100	0.08545900	0.53771200
N	-1.46140000	-3.03251700	-0.58561100
Cu	-0.53362500	-1.28646900	-1.38068900
N	7.33959600	1.48448100	-2.31692000
S	7.85859200	-0.09038600	-2.56805800
O	7.27420900	-0.65420500	-3.78120100
O	9.30765900	-0.02200400	-2.41483700
C	7.14669400	-0.95127000	-1.18906800
C	6.24075700	-1.97939300	-1.41717600
C	7.48285900	-0.55694400	0.10444600
C	5.64976400	-2.60974400	-0.32758800
H	5.98780700	-2.26403100	-2.43541300
C	6.88693000	-1.19880500	1.17805100
H	8.19119700	0.25518900	0.26082800
C	5.96195500	-2.23180200	0.97909500
H	4.92745400	-3.40814600	-0.49806200
H	7.13716200	-0.89675400	2.19521600
C	5.33939700	-2.92466900	2.15153300
H	5.07755600	-2.21556700	2.94723700
H	4.43125200	-3.46871800	1.86565600
H	6.03394200	-3.65470800	2.59088500
H	8.07650500	2.10068000	-2.65673900
C	-1.43510300	1.71510200	-1.66709500

H	-1.66085100	2.72197300	-1.25212300
C	1.10964900	1.76490900	-1.75629100
H	1.02837000	2.50381000	-2.56870500
H	1.10871400	0.75339800	-2.55492900
C	2.45546200	1.78051300	-1.01834400
C	2.66000700	0.50408400	-0.20252600
H	2.78123600	-0.38585300	-0.83659800
H	3.56094500	0.58483500	0.41887400
H	1.82705300	0.31043300	0.48761000
C	2.45890700	2.98701500	-0.07580300
H	2.23445900	3.91703100	-0.61713600
H	1.70761000	2.87151400	0.71922800
H	3.43756900	3.10781400	0.40828200
C	3.55690600	1.93867400	-2.08777100
H	3.49985000	2.96539900	-2.49103900
H	3.33581800	1.26894000	-2.93221000
C	4.97318400	1.64284500	-1.62308000
H	5.03487300	0.59566800	-1.29387500
H	5.26370400	2.25741500	-0.75617500
C	5.98268000	1.85706800	-2.73888300
H	6.01939800	2.91749800	-3.02165400
H	5.68611600	1.29662500	-3.64096800
C	-0.08261800	1.42076500	-1.14158300
H	-0.05448400	0.95321400	-0.15276900
C	-2.98474100	5.58361600	-3.60827600
H	-2.86978900	5.08677700	-4.57855800
H	-3.69443500	6.40724600	-3.75326600
H	-2.02633800	5.98925900	-3.27434700
C	-3.54774100	4.62138100	-2.61173800
O	-4.58765400	3.95617700	-2.91490200
O	-3.00056300	4.46803200	-1.47796700
Zn	-4.53733700	3.04864900	-1.07539600
O	-5.71597500	3.15555900	0.41327800
C	-5.30544100	3.12486000	1.65603100
O	-6.05381300	3.06624000	2.61379800

C	-3.79474800	3.17162100	1.84523700
H	-3.31774700	2.30183600	1.36558100
H	-3.37483100	4.07911200	1.38780500
H	-3.53552900	3.16120900	2.90805300
C	1.75444500	-1.28235500	-3.05699400
O	1.49435500	-0.14970300	-3.53865700
O	1.11529200	-1.92558700	-2.20352200
C	3.04588100	-1.91777500	-3.60614300
F	2.90652500	-2.18403500	-4.90190900
F	4.06204900	-1.06029000	-3.46258200
F	3.36646100	-3.04179400	-2.97419800
C	-1.54694300	1.84499600	-3.18722600
H	-0.92868400	2.66520100	-3.57126600
H	-1.24084700	0.91534800	-3.68568600
H	-2.58635600	2.05383800	-3.46691900

6a′

O	-4.17745200	1.09940100	0.09752400
N	-2.62977100	-0.52862600	0.55891200
C	-2.98185700	0.67737700	0.14152600
C	-1.56103600	-4.42405600	0.78005700
C	-3.13676000	-2.78819700	1.26396700
C	-3.96891400	-3.73961200	1.91668600
C	-3.52672800	-5.08022700	1.95623600
C	-2.32868700	-5.43156500	1.38844400
H	-0.59567500	-4.65903100	0.32939600
C	-3.53697500	-1.41155800	1.17320900
C	-5.18639800	-3.32628200	2.49927600
H	-4.15472600	-5.82412200	2.44645900
H	-1.96621600	-6.45605100	1.40643800
C	-5.54555300	-2.00597700	2.42860100
C	-4.73765200	-1.05834400	1.77124600
H	-5.81662700	-4.06319700	2.99523900
H	-6.47585600	-1.66547700	2.87912900
H	-5.07809000	-0.03043400	1.73047400

N	-1.94593500	-3.16520100	0.72006400
Cu	-1.00625800	-1.46110000	-0.06696800
N	6.80129400	1.31491300	-1.16603600
S	7.30771400	-0.26310400	-1.40890800
O	6.72076500	-0.83231500	-2.61903800
O	8.75699400	-0.20746200	-1.25638100
C	6.57969400	-1.10330500	-0.02544700
C	5.67140900	-2.13116500	-0.24824100
C	6.90256300	-0.69232200	1.26571800
C	5.05616000	-2.73462300	0.84237400
H	5.44273300	-2.44111200	-1.26490600
C	6.28338600	-1.30861300	2.34187900
H	7.62001600	0.11236900	1.41905900
C	5.34357000	-2.32807200	2.14699900
H	4.33740100	-3.53796900	0.67791100
H	6.52538200	-0.99414700	3.35704100
C	4.63754100	-2.94433900	3.31467900
H	3.74177400	-2.36398700	3.58060000
H	4.30778700	-3.96700000	3.09568600
H	5.27697100	-2.97425600	4.20537800
H	7.53357100	1.92815200	-1.52048400
C	-1.90844400	1.63711700	-0.35716400
H	-2.21136300	2.62154100	0.04312800
C	0.61394500	1.89953700	-0.29685500
H	0.57448100	2.55134600	-1.17950800
H	0.31420200	0.22019700	-1.74683300
C	1.98745600	1.80231600	0.34059600
C	2.16342200	0.52566200	1.16160900
H	2.20871000	-0.37537200	0.52920400
H	3.09712300	0.56451500	1.73725300
H	1.35415400	0.38855200	1.89063800
C	2.13078100	3.01647100	1.26848400
H	1.93927000	3.95492200	0.72936300
H	1.41411800	2.95704800	2.09910300
H	3.14094900	3.07379600	1.69757400

C	3.03972000	1.87968100	-0.78754200
H	3.00512800	2.89463800	-1.22157700
H	2.74547100	1.19790900	-1.60165500
C	4.46482200	1.53499600	-0.38667600
H	4.50155800	0.49278600	-0.03842100
H	4.81598700	2.15381800	0.45395900
C	5.43397300	1.69449200	-1.54722900
H	5.47458200	2.74436200	-1.86630500
H	5.09654000	1.11116200	-2.41976800
C	-0.52495600	1.36553400	0.16868700
H	-0.49163400	0.78579900	1.09446500
C	-3.58831300	5.49117800	-2.44632400
H	-3.25188100	4.93815800	-3.33198200
H	-4.39478400	6.15706400	-2.77490500
H	-2.76353400	6.08339600	-2.04219500
C	-4.11866900	4.52754100	-1.43220800
O	-5.07888800	3.75899700	-1.75636100
O	-3.61376000	4.46576400	-0.27249200
Zn	-5.00723000	2.91355200	0.11950300
O	-6.24449000	2.99817600	1.56767400
C	-5.87059800	2.98803500	2.82118400
O	-6.64491600	2.92380800	3.75886300
C	-4.36684700	3.06281800	3.05293600
H	-3.86375000	2.19088000	2.60507400
H	-3.94567800	3.96678800	2.58927900
H	-4.13745600	3.07781600	4.12260800
C	1.26676200	-1.40612200	-1.86401500
O	0.92793300	-0.25448000	-2.37098200
O	0.72379300	-1.99217000	-0.94474700
C	2.52871500	-1.95969100	-2.53928600
F	2.35989700	-2.02619500	-3.85043300
F	3.53631200	-1.13043900	-2.27280500
F	2.82287800	-3.15875700	-2.07051400
C	-2.01140200	1.74387400	-1.88051800
H	-1.34882700	2.52333400	-2.27731200

H	-1.75983000	0.78814000	-2.36640300
H	-3.03527000	1.99549600	-2.18490200

TS5a

O	-3.38478700	1.38720100	-0.32072700
N	-3.16829700	-0.61003800	-1.44820500
C	-2.70279700	0.53975600	-0.98604500
C	-3.88933000	-4.31703400	-2.74968200
C	-4.71478300	-2.42586000	-1.69192500
C	-5.98508700	-3.03360100	-1.51299900
C	-6.15097100	-4.35290100	-1.99124600
C	-5.10978400	-5.00141900	-2.60455200
H	-3.03685200	-4.78860300	-3.24136600
C	-4.46598400	-1.09522300	-1.21143500
C	-7.01702100	-2.31521400	-0.86894600
H	-7.11690100	-4.84067000	-1.86054400
H	-5.21085800	-6.01718000	-2.97788700
C	-6.77190700	-1.04220700	-0.42575900
C	-5.51128700	-0.43196600	-0.58851600
H	-7.98918200	-2.78738300	-0.73264700
H	-7.55900700	-0.47746400	0.07073500
H	-5.36177700	0.57441200	-0.21872100
N	-3.70472600	-3.08949300	-2.31416500
Cu	-1.99001100	-1.84463500	-2.41211400
N	7.13006400	-0.17841700	-2.50635400
S	7.83259800	-0.73846600	-1.09135600
O	7.35835100	-2.07857400	-0.75875100
O	9.25847300	-0.48350900	-1.26701600
C	7.18465600	0.37546800	0.13250400
C	7.41371200	1.74286200	-0.00384900
C	6.44982300	-0.12785900	1.19773600
C	6.87809400	2.61079900	0.93406000
H	7.99080500	2.11803500	-0.84756000
C	5.91868700	0.75728900	2.12978100
H	6.28303200	-1.20031300	1.28024800

C	6.12128700	2.13289100	2.01127400
H	7.04351800	3.68378900	0.83399600
H	5.33481700	0.37189100	2.96541400
C	5.55444300	3.08874000	3.01515500
H	6.34932200	3.53838700	3.62642300
H	5.02482300	3.91685000	2.52549400
H	4.85389500	2.59290600	3.69751400
H	7.82811200	-0.27619800	-3.24339000
C	-1.27667300	0.86511700	-1.33351700
H	-0.81588300	1.15158200	-0.11753600
C	0.86722900	-0.17886900	-2.27656700
H	0.85142600	0.64868600	-2.99917400
H	0.90210100	-1.10959100	-2.86641100
C	2.19286900	-0.10847300	-1.45949400
C	2.18509900	-1.14566700	-0.33917800
H	1.93697700	-2.15033100	-0.71415000
H	3.16733200	-1.20415700	0.15000600
H	1.45794600	-0.88783100	0.44378700
C	2.36287200	1.29251400	-0.87600100
H	2.46478700	2.04819000	-1.67008300
H	1.51051900	1.56788000	-0.24058700
H	3.26134500	1.35014400	-0.24477800
C	3.31853000	-0.41283900	-2.46620200
H	3.14228600	0.16953800	-3.38755800
H	3.24487500	-1.47431000	-2.75678600
C	4.72120500	-0.11554200	-1.96117400
H	4.85308100	-0.54432400	-0.95779600
H	4.87609700	0.96916500	-1.85996800
C	5.79588900	-0.66985100	-2.87839900
H	5.62764700	-0.32145000	-3.90684700
H	5.75853200	-1.77102000	-2.89224400
C	-0.39410000	-0.22345900	-1.50041300
H	-0.50674300	-1.06350000	-0.80524900
C	-4.90867000	6.34063700	-0.49021800
H	-5.94506600	6.26397400	-0.15066400

H	-4.43957900	7.18766300	0.02680800
H	-4.86895900	6.54403400	-1.56445200
C	-4.14494200	5.09941000	-0.16203200
O	-4.52107300	4.35241400	0.79390300
O	-3.09973300	4.80639300	-0.82222800
Zn	-2.90407100	3.14638300	0.40417000
O	-1.40535500	3.18731500	1.67906000
C	-0.53364800	2.29065000	1.85993300
O	-0.37518000	1.26855100	1.12951000
C	0.41409200	2.44678100	3.00870600
H	1.38642700	2.77240100	2.61479500
H	0.05356200	3.19033400	3.72361100
H	0.57078000	1.48236800	3.50238300
C	0.04085200	-3.51035700	-2.55471800
O	-0.14714300	-3.65714300	-1.35221000
C	1.21142100	-4.25108000	-3.22822600
F	1.96876100	-4.90125100	-2.35236400
F	0.76327300	-5.13479000	-4.12592200
F	2.00182300	-3.38255400	-3.87957800
O	-0.59766200	-2.77688700	-3.37916900
C	-1.13640800	2.09463400	-2.21372600
H	-1.35070900	1.84843200	-3.26425700
H	-1.84722900	2.88304800	-1.93374400
H	-0.13167600	2.52936700	-2.16001200

7a′

O	-3.34671000	1.63253400	0.18645300
N	-3.54659000	-0.48296300	-0.74075300
C	-2.97410900	0.68023000	-0.63974900
C	-4.22721000	-4.42185100	-0.44162100
C	-4.91891400	-2.27287000	0.07968900
C	-6.04630000	-2.76776100	0.78680100
C	-6.21021400	-4.16921300	0.86152300
C	-5.30249700	-4.99966100	0.25727100
H	-3.49317200	-5.04734400	-0.94945600

C	-4.67984900	-0.86050000	-0.00715100
C	-6.95331000	-1.86052800	1.37913100
H	-7.06619000	-4.57052500	1.40394000
H	-5.40125600	-6.08119600	0.29929600
C	-6.73401000	-0.51303700	1.25949400
C	-5.60698600	-0.01206800	0.57767700
H	-7.81568600	-2.24946200	1.91927600
H	-7.43318400	0.19476500	1.70123200
H	-5.46830100	1.06098900	0.50400500
N	-4.04436400	-3.11924200	-0.52782000
Cu	-2.55627900	-2.16786900	-1.57237500
N	6.88226800	0.96960600	-1.15705200
S	7.48658000	0.27853200	0.24376300
O	7.17932300	-1.14728200	0.30864600
O	8.87063900	0.73618100	0.30963500
C	6.53788000	1.08345500	1.51300100
C	6.55751300	2.47357600	1.60158600
C	5.77798200	0.31790600	2.38716100
C	5.78440000	3.09666900	2.56815600
H	7.16052400	3.05622200	0.90698600
C	5.00798300	0.95945000	3.35153800
H	5.77875700	-0.76655800	2.29567800
C	4.99435700	2.35108100	3.45202600
H	5.78645600	4.18423800	2.64359000
H	4.40637400	0.36632900	4.04039900
C	4.14514200	3.04559200	4.47167000
H	4.72016500	3.79775100	5.02746700
H	3.30763000	3.57500800	3.99527400
H	3.72370000	2.33937100	5.19687700
H	7.66534000	1.06585300	-1.80329900
C	-1.74593700	0.97631900	-1.42603300
H	-2.80979100	0.83217700	1.73025300
C	0.67989600	0.34250200	-1.82694000
H	0.69678000	1.16213200	-2.56192300
H	0.85527700	-0.58070300	-2.40564300

C	1.85860200	0.53382500	-0.84519500
C	1.80003000	-0.49028800	0.28532500
H	1.79026400	-1.52177800	-0.09576900
H	2.65927400	-0.38333800	0.96316200
H	0.89436500	-0.37253100	0.89688500
C	1.79036400	1.93291800	-0.24619500
H	1.93698400	2.71273000	-1.01027000
H	0.81185300	2.11039700	0.22611700
H	2.55052700	2.08220600	0.53520300
C	3.14548000	0.35348300	-1.67042700
H	3.05814800	0.95599500	-2.59222400
H	3.20080300	-0.69931500	-1.99873200
C	4.43014600	0.73715300	-0.95575900
H	4.48184700	0.22255800	0.01430800
H	4.44564400	1.81589800	-0.73801300
C	5.66971600	0.39999200	-1.76391700
H	5.59823200	0.85167200	-2.76300600
H	5.75961100	-0.68962200	-1.90045500
C	-0.66410500	0.21300300	-1.20070900
H	-0.78196900	-0.63158800	-0.51408500
C	-2.12376100	6.98622000	0.38770100
H	-2.92266600	7.41622800	0.99643600
H	-1.15422500	7.33823300	0.76100700
H	-2.21181700	7.32672300	-0.64959000
C	-2.14936900	5.49499300	0.43640000
O	-2.90298000	4.87880200	1.24472200
O	-1.38549000	4.83328300	-0.34399600
Zn	-2.09013500	3.12156000	0.48179800
O	-0.94780800	2.26121300	1.94064600
C	-1.10078100	1.16965600	2.50720000
O	-2.16800100	0.42427800	2.37221000
C	-0.07725500	0.62530100	3.43436200
H	-0.10196000	-0.46786000	3.45320800
H	0.91367600	0.98913500	3.14495900
H	-0.29577800	0.99012200	4.44700300

C	-0.08873900	-3.03612100	-1.90531400
O	-0.09737900	-3.11962600	-0.67743600
C	1.20337400	-3.43802400	-2.64722900
F	2.25340000	-3.52316800	-1.82804900
F	1.05036500	-4.62977700	-3.23736000
F	1.52570100	-2.55728800	-3.60687300
O	-1.01470200	-2.67231000	-2.68929100
C	-1.80112700	2.15027500	-2.37055300
H	-1.96435800	1.80150900	-3.40014400
H	-2.63728200	2.82767600	-2.14707000
H	-0.87830400	2.74402900	-2.37080100

TS6a

O	-3.77294000	-1.58394000	-0.04524800
N	-2.71204100	0.31250700	0.75332300
C	-2.01014200	-0.40423900	-1.30524300
C	-1.05976300	0.52122600	-0.60307800
C	-2.95525600	-0.64792900	-0.16108000
C	0.00957800	0.00873100	0.27544600
H	-1.50088900	-1.36000500	-1.52827200
H	-0.92064100	1.52052000	-1.04769600
C	-3.50578400	3.64711700	2.88429100
C	-3.25021300	1.34966100	2.87448200
C	-3.49540900	1.26419000	4.26836400
C	-3.76867800	2.47141100	4.95083400
C	-3.77759200	3.65988600	4.26544800
H	-3.49368000	4.57231000	2.30782100
C	-2.97306700	0.16906300	2.12569300
C	-3.43715800	0.00403600	4.90554200
H	-3.96499600	2.44009400	6.02222900
H	-3.98218500	4.60258600	4.76586800
C	-3.12897100	-1.11810400	4.17599900
C	-2.88470100	-1.03875300	2.79254800
H	-3.62388900	-0.05601100	5.97686100
H	-3.06469500	-2.08702000	4.66616500

H	-2.63143900	-1.93995100	2.23835200
N	-3.25266800	2.53891300	2.21815600
Cu	-2.65346200	2.21767500	0.22151600
H	0.06722600	0.63299800	1.18268600
H	-0.21117000	-1.02695100	0.57510700
C	1.40966100	0.06042500	-0.41183700
C	2.32275000	-0.79609600	0.48060900
H	2.21819200	-0.44511800	1.52282800
H	1.93820400	-1.82991400	0.46228300
C	1.32148000	-0.53173700	-1.81804100
H	0.92106700	-1.55520400	-1.78927000
H	0.68064200	0.08177000	-2.47255900
H	2.30918600	-0.56135300	-2.29681200
C	1.89700500	1.50519100	-0.47497100
H	2.85083900	1.57420600	-1.01487400
H	1.18854800	2.15967200	-1.00291100
H	2.05491600	1.91656900	0.53345200
C	3.80202700	-0.79911300	0.12305300
H	3.96027200	-1.08303500	-0.92872600
H	4.23777000	0.20132800	0.25705000
C	4.56054000	-1.77895000	0.99960200
H	4.17311700	-2.79526100	0.84945500
H	4.40969400	-1.53921200	2.06365300
N	5.99381500	-1.82544000	0.65503700
S	7.03510300	-0.87589400	1.55931700
O	6.29666400	0.33926900	1.88293600
O	7.65204600	-1.65134600	2.63367700
C	8.29620000	-0.50998000	0.36692800
C	9.61089500	-0.87392800	0.62286800
C	7.95045500	0.18079200	-0.79395500
C	10.59384000	-0.54638700	-0.30565200
H	9.85558000	-1.40688100	1.53906300
C	8.94132400	0.49836900	-1.70670600
H	6.91256000	0.45578500	-0.97745800
C	10.27632600	0.13969500	-1.47709300

H	11.62895600	-0.82893500	-0.11518500
H	8.68393500	1.03605000	-2.61958100
C	11.33000700	0.49074000	-2.48142300
H	12.32623300	0.17200200	-2.15354000
H	11.12820000	0.01630800	-3.45160800
H	11.36462400	1.57402000	-2.65976500
H	6.36997400	-2.77095700	0.60031300
C	-7.35021200	-4.66412100	-2.19389500
H	-7.74086200	-5.33514300	-1.42525300
H	-8.05272100	-3.84033200	-2.36383800
H	-7.25647700	-5.20585200	-3.14260600
C	-6.01701300	-4.11206100	-1.80924000
O	-5.44831800	-3.26573000	-2.57066100
O	-5.44570800	-4.48101000	-0.73840600
Zn	-3.89094500	-3.22122500	-1.24395100
O	-2.02558800	-3.59540300	-1.49554700
C	-1.26345900	-3.82458000	-0.46028600
O	-0.19814400	-3.25078900	-0.26724200
C	-1.78400100	-4.85712700	0.51912900
H	-2.63843200	-4.45241000	1.08484300
H	-2.14654500	-5.74703400	-0.00995400
H	-1.00929200	-5.14193100	1.23704900
C	-1.96541500	5.35142800	-2.45750900
C	-1.81251000	3.97408500	-1.78081500
O	-0.95149000	3.20483700	-2.19719800
O	-2.63896300	3.82807000	-0.82557400
F	-3.21872200	5.53555100	-2.88735600
F	-1.69147600	6.32944200	-1.58415200
F	-1.15350400	5.49941400	-3.49906700
C	-2.67562400	0.15488500	-2.55286500
H	-1.93258200	0.34225300	-3.33676300
H	-3.41470900	-0.55370800	-2.95021300
H	-3.19104200	1.10209500	-2.33991200

8a′

O	-4.27943000	-0.79676900	-0.26561300
N	-2.36703200	-0.08749500	0.88678200
C	-2.07605200	0.07761500	-1.19141500
C	-1.16176400	0.28484700	0.05043600
C	-3.13001700	-0.35205500	-0.20052900
C	-0.00271200	-0.66495400	0.25809900
H	-1.74925400	-0.77856000	-1.80080700
H	-0.87847900	1.33573000	0.21199300
C	-2.02724700	2.20285000	4.38254900
C	-2.32699900	0.14198300	3.34851600
C	-2.33581200	-0.51074400	4.61422400
C	-2.15993600	0.27442000	5.77316000
C	-1.99205700	1.62944700	5.66120900
H	-1.92230300	3.27982900	4.26462700
C	-2.45738200	-0.65599800	2.18006000
C	-2.51861500	-1.91024400	4.68917300
H	-2.15935600	-0.21561100	6.74589900
H	-1.84741500	2.26495100	6.52998100
C	-2.66999400	-2.65097700	3.54658200
C	-2.61963300	-2.02122100	2.29002600
H	-2.52925600	-2.38144300	5.67078800
H	-2.80474400	-3.72886100	3.59974500
H	-2.68617500	-2.60990600	1.37362100
N	-2.19437200	1.50208700	3.26742900
Cu	-2.42397300	2.55897200	1.67894100
H	0.13216400	-0.81552400	1.34332300
H	-0.27772300	-1.65149800	-0.15727200
C	1.34898200	-0.20464300	-0.31767900
C	2.30071000	-1.40861700	-0.20034700
H	2.23092000	-1.80999000	0.82639300
H	1.92492200	-2.20871700	-0.86354000
C	1.19333900	0.21023300	-1.77923100
H	0.75563000	-0.59692500	-2.38594300
H	0.55533700	1.10021200	-1.87673400
H	2.16151800	0.47333100	-2.22597000

C	1.87663300	0.97618200	0.49761500
H	2.81346600	1.36371600	0.07552800
H	1.16973300	1.81745400	0.51026000
H	2.07873600	0.67947400	1.53842000
C	3.76717800	-1.14658900	-0.51092600
H	3.89188200	-0.67250500	-1.49694900
H	4.19687300	-0.46109300	0.23294600
C	4.57025600	-2.43377700	-0.49533600
H	4.19730900	-3.12114700	-1.26645000
H	4.45258100	-2.94827200	0.47081800
N	5.99011700	-2.19681000	-0.81327400
S	7.05161600	-1.94728600	0.45746200
O	6.26930500	-1.34788900	1.53240500
O	7.83279700	-3.15531700	0.71254200
C	8.15364900	-0.73093400	-0.21704100
C	9.50933100	-1.01143000	-0.31599600
C	7.64347200	0.50917600	-0.59906400
C	10.36577400	-0.03377300	-0.81255100
H	9.88411900	-1.98471300	-0.00685600
C	8.50989500	1.47009100	-1.09062600
H	6.57637700	0.71122900	-0.51754900
C	9.88282200	1.21408000	-1.20423000
H	11.43166300	-0.24495300	-0.89595400
H	8.12248400	2.44257300	-1.39531300
C	10.80069500	2.27156000	-1.73482400
H	11.83448200	1.91434000	-1.80666100
H	10.48901200	2.60555500	-2.73375100
H	10.79734700	3.16014100	-1.08866200
H	6.39065800	-2.88589800	-1.44763300
C	-5.33645800	-0.44652400	-5.11554800
H	-6.32434700	-0.70589800	-5.50715400
H	-5.32949700	0.63251100	-4.91075200
H	-4.56114500	-0.65378800	-5.85870400
C	-5.06074400	-1.17545800	-3.83858500
O	-3.86303800	-1.44295200	-3.51036100

O	-6.01840000	-1.48986900	-3.06986000
Zn	-4.61556500	-2.22433100	-1.72380100
O	-5.11814400	-3.98672000	-0.81982300
C	-3.90247900	-4.20656300	-0.51400700
O	-2.99331700	-3.40735300	-0.87531500
C	-3.57591900	-5.42544300	0.29371200
H	-4.18707200	-5.43859400	1.20421600
H	-3.84033400	-6.32372500	-0.27718700
H	-2.51354300	-5.46121100	0.55140400
C	-1.81116000	5.08395500	-1.47833700
C	-1.56870700	4.08423500	-0.33106100
O	-0.43740000	3.65819300	-0.13946100
O	-2.65306800	3.79756200	0.28058200
F	-2.57878900	4.52269500	-2.42288600
F	-2.44293300	6.17726200	-1.04168400
F	-0.67833700	5.47147300	-2.05520200
C	-2.44423700	1.26226000	-2.05903200
H	-1.56579300	1.64957600	-2.59033500
H	-3.17962000	0.95070700	-2.81128500
H	-2.87862200	2.07742600	-1.46650000
